# Global age-sex-specific all-cause mortality and life expectancy estimates for 204 countries and territories and 660 subnational locations, 1950–2023: a demographic analysis for the Global Burden of Disease Study 2023

**DOI:** 10.1016/S0140-6736(25)01330-3

**Published:** 2025-10-18

**Authors:** GBD 2023 Demographics Collaborators, Austin E Schumacher, Peng Zheng, Ryan M Barber, Bhoomadevi A, Mohammad Amin Aalipour, Hasan Aalruz, Hazim S Ababneh, Ukachukwu O Abaraogu, Cristiana Abbafati, Nasir Abbas, Mitra Abbasifard, Faezeh Abbaspour, Abdallah H A Abd Al Magied, Samar Abd ElHafeez, Mohammed Altigani Abdalla, Emad M Abdallah, Nadin M I Abdel Razeq, Reda Abdel-Hameed, Wael M Abdel-Rahman, Sherief Abd-Elsalam, Omar Ahmed Abdelwahab, Parsa Abdi, Arash Abdollahi, Meriem Abdoun, Arman Abdous, Deldar Morad Abdulah, Rizwan Suliankatchi Abdulkader, Auwal Abdullahi, Abdullahi Salahudeen Abdulraheem, Habtamu Abebe Abebe Getahun, Parisa Abedi, Armita Abedi, Asrat Agalu Abejew, Roberto Ariel Abeldaño Zuñiga, Syed Hani Abidi, Alemwork Abie, Olumide Abiodun, Olugbenga Olusola Abiodun, Richard Gyan Aboagye, Shady Abohashem, Ulric Sena Abonie, Nagah M Abourashed, Mohamed Abouzid, Dmitry Abramov, Lucas Guimarães Abreu, Dariush Abtahi, Rana Kamal Abu Farha, Fuad Hamdi A Abuadas, Aminu Kende Abubakar, Bilyaminu Abubakar, Eman Abu-Gharbieh, Sawsan Abuhammad, Ahmad Y Abuhelwa, Hana J Abukhadijah, Salahdein Aburuz, Dina Abushanab, Ahmed Abu-Zaid, Anirudh Balakrishna Acharya, Meshack Achore, Juan Manuel Acuna, Tim Adair, Lisa C Adams, Oladimeji Muritala Adebayo, Tajudeen Adesanmi Adebisi, David Adedia, Kamoru Ademola Adedokun, Oluwatobi E Adegbile, Nurudeen A Adegoke, Olumide Thomas Adeleke, Miracle Ayomikun Adesina, Isaac Ayodeji Adesina, Olatunji O Adetokunboh, Temitayo Esther Adeyeoluwa, Mache Tsadik Adhana, Kishor Adhikari, Ripon Kumar Adhikary, Usha Adiga, Tanin Adl Parvar, Mohd Adnan, Qorinah Estiningtyas Sakilah Adnani, Leticia Akua Adzigbli, David Adzrago, Giuseppina Affinito, Aanuoluwapo Adeyimika Afolabi, Rotimi Felix Afolabi, Saira Afzal, Gizachew Beykaso Agafari, Navidha Aggarwal, Mahdi Aghaalikhani, Sepehr Aghajanian, Seyed Mohammad Kazem Aghamir, Feleke Doyore Agide, Mary Dada Agoi, César Agostinis Sobrinho, Anurag Agrawal, Williams Agyemang-Duah, Bright Opoku Ahinkorah, Rabbiya Ahmad, Danish Ahmad, Faisal Ahmad, Aqeel Ahmad, Muayyad M Ahmad, Khurshid Ahmad, Tauseef Ahmad, Waqas Ahmad, Aram Mahmood Ahmed, Muktar Beshir Ahmed, Ayman Ahmed, Ali Ahmed, Anisuddin Ahmed, Mushood Ahmed, Naveed Ahmed, Oli Ahmed, Meqdad Saleh Ahmed, Akeem Olayiwola Ahmed, Mehrunnisha Sharif Ahmed, Syed Anees Ahmed, Gasha Salih Ahmed, Shabbir Ahmed, Haroon Ahmed, Luai A Ahmed, Gulzhanat Aimagambetova, Janardhana P Aithala, Marjan Ajami, Budi Aji, Hossein Akbarialiabad, Saeid Akbarifard, Oluwasefunmi Akeju, Roland Eghoghosoa Akhigbe, Muhammad Nadeem Akhtar, Karolina Akinosoglou, Yagiz Matthew Akiska, Mohammed Ahmed Akkaif, Wole Akosile, Hammad Akram, Ashley E Akrami, Hanadi Al Hamad, Syed Mahfuz Al Hasan, Mohammad Khaled Al Nawayseh, Omar Al Omari, Mohammad Al Qadire, Zain Al Ta'ani, Yazan Al Thaher, Omar Ali Mohammed Al Zaabi, Mohammad Ahmmad Mahmoud Al Zoubi, Mousa Ali Al-Abbadi, Tariq A Alalwan, Ziyad Al-Aly, Mohammad Khursheed Alam, Khurshid Alam, Mostafa Alam, Manjurul Alam, Rasmieh Mustafa Al-Amer, Abebaw Alamrew, Amani Alansari, Turki M Alanzi, Fahmi Y Al-Ashwal, Mohammed Albashtawy, Khalifah A Aldawsari, Mohammed S Aldossary, Robert W Aldridge, Shereen M Aleidi, Bezawit Abeje Alemayehu, Tekletsadik Tekleslassie Alemayehu, Fentahun Alemnew, Ayman Al-Eyadhy, Ali M Alfalki, Abdelazeem M Algammal, Fadwa Naji Alhalaiqa, Mohammed Khaled Al-Hanawi, Aminu Alhassan Alhassan Ibrahim, Ashraf Alhumaidi, Fahad A Alhumaydhi, Shahid Ali, Mohammed Usman Ali, Kamran Ali, Mohammad Daud Ali, Irfan Ali, Syed Shujait Ali, Waad Ali, Haroon Muhammad Ali, Amjad Ali, Rafat Ali, Maratab Ali, Syed Yusuf Ali, Sameer Afif Ali, Akram Al-Ibraheem, Gianfranco Alicandro, Montaha Al-Iede, Sheikh Mohammad Alif, Hamid Alinejad Rokny, Samah W Al-Jabi, Mohamad Aljofan, Moath Saleh Aljohani, Adel Al-Jumaily, Syed Mohamed Aljunid, Ahmad Alkhatib, Mustafa Alkhawam, Atefeh Allahbakhshian, Mohammed Z Allouh, Wesam Taher Almagharbeh, Wael Almahmeed, Md. Al-Mamun, Sabah Al-Marwani, Joseph Uy Almazan, Hesham M Al-Mekhlafi, Omar Almidani, Amr Almobayed, Khaldoon Aied Alnawafleh, Hasan Yaser Alniss, Margret Beaula Alocious Sukumar, Mohammad R Alosta, Saleh A Alqahtani, Jaber S Alqahtani, Mohammad R Alqudimat, Ahmad Rajeh Al-Qudimat, Ahmad Alrawashdeh, Rami H Al-Rifai, Intima Alrimawi, Sahel Majed Alrousan, Salman Khalifah Al-Sabah, Mohammed A Alsabri, Najim Z Alshahrani, Zaid Altaany, Awais Altaf, Alaa B Al-Tammemi, Jaffar A Al-Tawfiq, Malik A Althobiani, Khalid A Altirkawi, Javier Alvarez-Galvez, Nelson Alvis-Guzman, Mohammad Al-Wardat, Yaser Mohammed Al-Worafi, Hany Aly, Mohammad Sharif Ibrahim Alyahya, Karem H Alzoubi, Md. Akib Al-Zubayer, Uchenna Anderson Amaechi, Joy Amafah, Ekiyor Joseph Amafah, Masoud Aman Mohammadi, Faten Amer, Bardia Amidi, Tarek Tawfik Amin, Amr Amin, Alireza Amindarolzarbi, Saeed Amini, Ehsan Amini-Salehi, Nafiu Aminu, Majid Aminzare, Sohrab Amiri, Mohammad Hosein Amirzade-Iranaq, Joanne O Amlag, Dickson A Amugsi, Ganiyu Adeniyi Amusa, Filippos Anagnostakis, Roshan A Ananda, Nazanin Anaraki, Robert Ancuceanu, Deanna Anderlini, David B Anderson, Tudorel Andrei, Song Peng Ang, Nguyen Hoang Anh, Samuel Egyakwa Ankomah, Kabilan Annadurai, Amir Anoushiravani, Sumbul Ansari, Umair Ansari, Alireza Ansari-Moghaddam, Catherine M Antony, Ernoiz Antriyandarti, Boluwatife Stephen Anuoluwa, Iyadunni Adesola Anuoluwa, Saleha Anwar, Sumadi Lukman Anwar, Razique Anwer, Shahnawaz Anwer, Anayochukwu Edward Anyasodor, Geminn Louis Carace Apostol, Juan Pablo Arab, Hossein Arabi, Jalal Arabloo, Mosab Arafat, Demelash Areda, Abdulfatai Aremu, Jorge Arias de la Torre, Ghazal Arjmand, Benedetta Armocida, Johan ārnlöv, Jesu Arockiaraj, Mahwish Arooj, Anton A Artamonov, Deepavalli Arumuganainar, Nurila Aryntayeva, Mahsa Asadi Anar, Majid Asadi-Samani, Syed Mohammed Basheeruddin Asdaq, Saeed Asgary, Mohammad Asghari-Jafarabadi, Tahira Ashraf, Muhammad Abdul Basit Ashraf, Syed Amir Ashraf, Mitra Ashrafi, Milad Ashrafizadeh, Bernard Kwadwo Yeboah Asiamah-Asare, Muhammad Shahzad Aslam, Saeed Aslani, Yuni Asri, Anil Raj Assariparambil, Dereje Zewdu Assefa, Batyrbek Assembekov, Thomas Astell-Burt, Mirbahador Athari, Maha Moh'd Wahbi Atout, Alok Atreya, Julie Alaere Atta, Zeenah A Atwan, Marcel Ausloos, Abolfazl Avan, Núbia Carelli Pereira Avelar, Sana Javaid Awan, Amlaku Mulat Aweke, Babafela B Awosile, Adedapo Wasiu Awotidebe, Beatriz Paulina Ayala Quintanilla, Fekadu Belay Ayalew, Lemessa Assefa A Ayana, Haleh Ayatollahi, Olatunde O Ayinde, Yusuf Oloruntoyin Ayipo, Berrak Itir Itir Ayli, Seyed Mohammad Ayyoubzadeh, Sina Azadnajafabad, Arian Azadnia, James Mba Azam, Alireza Azarboo, Ali Azargoonjahromi, Gulrez Shah Azhar, Farya Azimi, Sadat Abdulla Aziz, Mohd Yusmaidie Aziz, Amin Azizan, Ahmed Y Azzam, Domenico Azzolino, Shahram Babadoust, Abraham Samuel Babu, Giridhara Rathnaiah Babu, Ashish D Badiye, Hunter Baggen, Elahe Baghizadeh, Sana Baghizadeh, Khlood K Baghlaf, Ahmed Salem BaHammam, Najmeh Bahmanziari, Razieh Bahreini, Yogesh Bahurupi, Ruhai Bai, Atif Amin Baig, Arun Balachandran, Wondu Feyisa Balcha, Maher Balkis, Jose Balmori-de-la-Miyar, Mohammadreza Balooch Hasankhani, Ovidiu Constantin Baltatu, Soham Bandyopadhyay, Palash Chandra Banik, Rajon Banik, Angelo Barbato, Suzanne Lyn Barker-Collo, Hiba Jawdat Barqawi, Amadou Barrow, Sandra Barteit, Zarrin Basharat, Shahid Bashir, Azadeh Bashiri, Guido Basile, Pritish Baskaran, Mohammad-Mahdi Bastan, Abdul-Monim Batiha, Kavita Batra, Bernhard T Baune, Mahdis Bayat, Mohammad Amin Bayat Tork, Mohsen Bayati, Mulat Tirfie Bayih, Thomas Beaney, Neeraj Bedi, Narasimha M Beeraka, Jina Behjati, Babak Behnam, Payam Behzadi, Diana Fernanda Bejarano Ramirez, Bezawit K Bekele, Almaz Nibret Belay, Melesse Belayneh, Asnake Gashaw Belayneh, Gokce Belge Bilgin, Bashir Bello, Muhammad Bashir Bello, Umar Muhammad Bello, Olorunjuwon Omolaja Bello, Luis Belo, Apostolos Beloukas, Riyad Bendardaf, Samiun Nazrin Bente Kamal Tune, Habib Benzian, Maria Bergami, Alemshet Yirga Berhie, Abiye Assefa Berihun, Amiel Nazer C Bermudez, Robert S Bernstein, Ajeet Singh Bhadoria, Akshaya Srikanth Bhagavathula, Jeetendra Bhandari, Charmi Bhanushali, Pankaj Bhardwaj, Nikha Bhardwaj, Ashish Bhargava, Sonu Bhaskar, Arushee Bhatnagar, Shuvarthi Bhattacharjee, Priyadarshini Bhattacharjee, Manpreet S Singh Bhatti, Rajbir Bhatti, Gurjit Kaur Bhatti, Jasvinder Singh Bhatti, Soumitra S Bhuyan, Sibhatu Kassa Biadgilign, Raluca Bievel-Radulescu, Can Bilgin, Naif Kandash Binsaleh, Catherine Bisignano, Raaj Kishore Biswas, Mohammad Shahangir Biswas, Bijit Biswas, Ahmad Naoras Bitar, Molalegne Bitew, Bruno Bizzozero-Peroni, Virginia Bodolica, Mahmut Bodur, Lucimere Bohn, Obasanjo Afolabi Bolarinwa, Archith Boloor, Paria Bolourinejad, Sri Harsha Boppana, Berrak Bora Basara, Hamed Borhany, Arturo Borzutzky, Alejandro Botero Carvajal, Souad Bouaoud, Soufiane Boufous, Rupert R A Bourne, Christopher Boxe, Nicola Luigi Bragazzi, Dejana Braithwaite, Susanne Breitner, Hermann Brenner, Edmond D Brewer, Gabrielle Britton, Julie Brown, Annie J Browne, Raffaele Bugiardini, Linh Phuong Bui, Tsion Samuel Bunare, Richard A Burns, Felix Busch, Reinhard Busse, Yasser Bustanji, Zahid A Butt, Nadeem Shafique Butt, Lucero Cahuana-Hurtado, Tianji Cai, Rose Cairns, Daniela Calina, Luis Alberto Cámera, Luciana Aparecida Campos, Ismael Campos-Nonato, Si Cao, Yuchen Cao, Chao Cao, Angelo Capodici, Giulia Carreras, Austin Carter, Andrea Carugno, Márcia Carvalho, Andre F Carvalho, Ana Paula Carvalho-e-Silva, Joao Mauricio Castaldelli-Maia, Carlos A Castañeda-Orjuela, Giulio Castelpietra, Ferrán Catalá-López, Alberico L Catapano, Maria Sofia Cattaruzza, Luca Cegolon, Francieli Cembranel, Muthia Cenderadewi, Ester Cerin, Pamela Roxana Chacón-Uscamaita, Chiranjib Chakraborty, Sandip Chakraborty, Jeffrey Shi Kai Chan, Joht Singh Chandan, Rama Mohan Chandika, Miyuru Chandradasa, Jung-Chen Chang, Vijay Kumar Chattu, Victoria Chatzimavridou-Grigoriadou, Sirshendu Chaudhuri, Akhilanand Chaurasia, Galmesa Bekana Chemeda, An-Tian Chen, Hui Chen, Haowei Chen, Xiang Chen, Hana Chen, Meng Xuan Chen, Haojin Cheng, Ka Ching Cheung, Nicholas WS Chew, Fatemeh Chichagi, Ju-Huei Chien, Odgerel Chimed-Ochir, William C S Cho, Daniel Youngwhan Cho, Bryan Chong, Hitesh Chopra, Shivani Chopra, Sonali Gajanan Choudhari, Shanjida Chowdhury, Mohiuddin Ahsanul Kabir Chowdhury, Sreshtha Chowdhury, Dinh-Toi Chu, Hongyuan Chu, Isaac Sunday Chukwu, Stephen Chukwudeh, Eric Chung, Sheng-Chia Chung, Erin Chung, Sunghyun Chung, Cain C T Clark, Alyssa Columbus, Haley Comfort, Joao Conde, Nathalie Conrad, Samuele Cortese, Paolo Angelo Cortesi, Claudia Cosma, Michael H Criqui, Natalia Cruz-Martins, Garland T Culbreth, Nour Dababo, Ali Dabbagh, Omid Dadras, Zainab Umar Dahiru, Tukur Dahiru, Xiaochen Dai, Mayank Dalakoti, Koustuv Dalal, Gloria Dalla Costa, Emanuele D´Amico, Rakhi Dandona, Lalit Dandona, Lucio D´Anna, Pojsakorn Danpanichkul, Samuel E Danso, Samuel Demissie Darcho, Latefa Ali Dardas, Chengetai Dare, Jai K Das, Barbara A D´Avanzo, Claudio Alberto Dávila-Cervantes, Dimash Davletov, Kairat Davletov, Fernando Pio De la Hoz, Alejandro de la Torre-Luque, Edward Christopher Dee, Sindhura Deekonda, Louisa Degenhardt, Paria Dehesh, Lee Deitesfeld, Denise Myriam Dekker, Pouria Delbari, Mohammad Delsoz, Dessalegn Demeke, Andreas K Demetriades, Edgar Denova-Gutiérrez, Ismail Dergaa, Kebede Deribe, Hunegnaw Almaw Derseh, Emina Derviševic, Hardik Dineshbhai Desai, Abraham Aregay Desta, Vinoth Gnana Chellaiyan Devanbu, Pradeep Kumar Devarakonda, Devananda Devegowda, Arkadeep Dhali, Kuldeep Dhama, Rajinder K Dhamija, Samath Dhamminda Dharmaratne, Meghnath Dhimal, Bibha Dhungel, Marcello Di Pumpo, Diana Dias da Silva, Luis Antonio Diaz, Daniel Diaz, Diego Diaz-Milanes, Elangovan Dilipan, Lauren K Dillard, Zhendong Ding, Xueting Ding, M Ashworth Dirac, Huyen Do, Thao Huynh Phuong Do, Phidelia Theresa Doegah, Sushil Dohare, Klara Georgieva Dokova, Regina-Mae Villanueva Dominguez, Francesco Dondi, Mario D´Oria, Fariba Dorostkar, Ojas Prakashbhai Doshi, Robert Kokou Dowou, Menayit Tamrat Dresse, Tim Robert Driscoll, Jiang Du, Judy R Dubno, Emeka W Dumbili, Samuel C Dumith, Bruce B Duncan, Jennifer Dunne, Andre Rodrigues Duraes, Senbagam Duraisamy, Oyewole Christopher Durojaiye, Siddhartha Dutta, Angel Belle Cheng Dy, Abdel Rahman E'mar, Osamudiamen Ebohon, Ejemai Eboreime, Mohammad Hossein Ebrahimi, Abdelaziz Ed-Dra, David Edvardsson, Ferry Efendi, Behrad Eftekhari, Foolad Eghbali, Shayan Eghdami, Fatemeh Ehsani, Ashkan Eighaei Sedeh, Terje Andreas Eikemo, Ebrahim Eini, Michael Ekholuenetale, Temitope Cyrus Ekundayo, Rabie Adel El Arab, Maysaa El Sayed Zaki, Mohamed Ahmed Eladl, Reza Elahi, Said El-Ashker, Rana Elbeshbeishy, Faris El-Dahiyat, Marwa Eldegwi, Marwan El-Deyarbi, Noha Mousaad Elemam, Ghada Metwally Tawfik ElGohary, Muhammed Elhadi, Mohamed Elhoumed, Waseem El-Huneidi, Omar Abdelsadek Abdou Elmeligy, Mohamed A Elmonem, Adel B Elmoselhi, Mohamed Hassan Elnaem, Mohammed Elshaer, Ibrahim Elsohaby, Chadi Eltaha, Abdelgawad Salah Abdelgawad Eltahawy, Tadele Emagneneh, Syed Emdadul Haque, Theophilus I Emeto, Victor Oghenekparobo Emojevwe, Stanley Chinedu Eneh, Christopher Imokhuede Esezobor, Babak Eshrati, Sharareh Eskandarieh, Majid Eslami, Rafaela Cavalheiro do Espírito Santo, Kara Estep, Elochukwu Ezenwankwo, Natalia Fabin, Heidar Fadavian, Adeniyi Francis Fagbamigbe, Ayesha Fahim, Razana Faiz, Ildar Ravisovich Fakhradiyev, Aliasghar Fakhri-Demeshghieh, Luca Falzone, Qiping Fan, Mohammad Farahmand, Seyed Nooreddin Faraji, Ali Faramarzi, Mohammad Fareed, Andre Faro, Syed Muhammad Yousaf Farooq, Fatemeh Farshad, Farima Farsi, Md. Omar Faruk, Abidemi Omolara Fasanmi, Folorunso Oludayo Fasina, Modupe Margaret Fasina, Ali Fatehizadeh, Davood Fathi, Zareen Fatima, Timur Fazylov, Valery L Feigin, maryam feili, Alireza Feizkhah, Ginenus Fekadu, Xiaoqi Feng, Talukdar Raian Ferdous, Seyed-Mohammad Fereshtehnejad, Nuno Ferreira, Bikila Regassa Feyisa, Alexander Finnemore, Claudio Fiorilla, Ida Fitriana, Luisa S Flor, Artem Alekseevich Fomenkov, Marco Fonzo, Arianna Fornari, Behzad Foroutan, Daniela Fortuna, Matteo Foschi, Maryam Fotouhi, Kayode Raphael Fowobaje, Richard Charles Franklin, Alberto Freitas, Takeshi Fukumoto, Ami Fukunaga, John E Fuller, Nancy Fullman, Blima Fux, Sridevi G, Peter Andras Gaal, Muktar A Gadanya, Dominic Dormenyo Gadeka, Márió Gajdács, Emmanuela Gakidou, Yaseen Galali, Silvano Gallus, Dhanraj Ganapathy, Balasankar Ganesan, Shivaprakash Gangachannaiah, Xiang Gao, Yijie Gao, Bashiru Garba, Miguel Garcia-Argibay, David Garcia-Azorin, Jacopo Garlasco, Zisis Gatzioufas, Rupesh K Gautam, Prem Gautam, Bamba Gaye, Federica Gazzelloni, Hong-Han Ge, Feven Sahle Gebre, Miglas Welay Gebregergis, Haftay Gebremedhin Gebreslassie, Miesa Gelchu, Stefano Gelibter, Nsikakabasi Samuel George, Ali Gerami Matin, Lemma Getacher, Genanew K Getahun, Kalab Yigermal Gete, Peter W Gething, Delaram J Ghadimi, Keyghobad Ghadiri, Arin Ghamkhar, Ali Ghandili, Mohammad-Reza Ghasemi, Moein Ghasemi, Shakiba Ghasemi Assl, Fariba Ghassemi, Ramy Mohamed Ghazy, Nermin Ghith, Zainab Gholami, Nasim Gholizadeh, Elena Ghotbi, Arun Ghuge, Alessandro Gialluisi, Konstantinos Giannakis, Ruth Margaret Gibson, Syed Abdullah Gilani, Tiffany K Gill, Alem Abera Girmay, Alessandro Girombelli, Laszlo Göbölös, Rajesh Kumar Goel, Anil Kumar Goel, Archit Goel, Kimiya Gohari, Mahaveer Golechha, Ali Golestani, Mohsen Golkar, Nelson G M Gomes, Philimon N Gona, Wenping Gong, Sameer Vali Gopalani, Giuseppe Gorini, Yitayal Ayalew Goshu, Alessandra C Goulart, Ayman Grada, Simon Matthew Matthew Graham, Michal Grivna, Ashna Grover, Habtamu Alganeh Guadie, Bin Guan, Shi-Yang Guan, Giovanni Guarducci, Mohammed Ibrahim Mohialdeen Gubari, Avirup Guha, Stefano Guicciardi, Zhifeng Guo, Xingzhi Guo, Zhaoyu Guo, Cui Guo, Zheng Guo, Rajat Das Gupta, Rajeev Gupta, Sapna Gupta, Lalit Gupta, Himanshu Gupta, Roberth Steven Gutiérrez-Murillo, Jose Guzman-Esquivel, Abrham Tesfaye Tesfaye Habteyes, Awoke Derbie Derbie Habteyohannes, Tesfahun Simon Hadaro, Zahra Hadian, Sarah Hafsia, Faraidoon Haghdoost, Arian Haghtalab, Nguyen Hai Nam, Arvin Haj-Mirzaian, Pritam Halder, Sebastian Haller, Rabih Halwani, Islam M Hamad, Randah R Hamadeh, Nadia M Hamdy, Samer Hamidi, Erin B Hamilton, Ahmad Hammoud, Mohammad Hamza, Didem Han Yekdes, Asif Hanif, Nasrin Hanifi, Graeme J Hankey, Fahad Hanna, Md Nuruzzaman Haque, Ashanul Haque, Obaid I Haque, Harapan Harapan, Hilda L Harb, Arief Hargono, Andy Martahan Andreas Hariandja, Josep Maria Haro, Eka Mishbahatul Marah Has, Ahmed I Hasaballah, Md Kamrul Hasan, Faizul Hasan, Towhid Hasan, Hamidreza Hasani, Ali Hasanpour- Dehkordi, Arezou Hashem Zadeh, Mohammad Hashem Hashempur, Nada Tawfig Hashim, Ammarah Hasnain, Ikrama Hassan, Ibrahim Nagmeldin Hassan, Nageeb Hassan, Yusuf Wada Hassan Wada, Mahgol Sadat Hassan Zadeh Tabatabaei, Rasmus J Havmoeller, Simon I Hay, Khezar Hayat, Jiawei He, Jeffrey J Hebert, Mohammad Heidari, Golnaz Heidari, Mehdi Hemmati, Claire A Henson, Claudiu Herteliu, Hamed Hesami, Sumudu Avanthi Hewage, Majid Heydari, Zahra Heydarifard, Yuta Hiraike, Ramesh Holla, Nobuyuki Horita, Md Belal Hossain, Md Mahbub Hossain, Md Sabbir Hossain, Alamgir Hossain, Mohammad Bellal Hossain, Lubna Hossain, Sorin Hostiuc, Mihaela Hostiuc, Peter J Hotez, Jada Averianna Houser, Amir Human Hoveidaei, Hanno Hoven, Alexander Win Hsu, Chengxi Hu, Guoqing Hu, Yefei Huang, Zhenyao Huang, Junjie Huang, Weijun Huang, Mega Hasanul Huda, Ayesha Humayun, Waqar Husain, Kiavash Hushmandi, Javid Hussain, Nawfal R Hussein, Mohamed Ibrahim Husseiny, Hong-Han Huynh, Bing-Fang Hwang, Luigi Francesco Iannone, Segun Emmanuel Ibitoye, Umar Idris Ibrahim, Ismail A. Atef Ismail Ahmed Ibrahim, Ramzi Ibrahim, Anel Ibrayeva, Fidelia Ida, Pulwasha Maria Iftikhar, Adalia Ikiroma, Kevin S Ikuta, Olayinka Stephen Ilesanmi, Irena M Ilic, Milena D Ilic, Masoud Imani, Mustapha Immurana, Lucius Chidiebere Imoh, Leeberk Raja Inbaraj, Arit Inok, Mujahid Iqbal, Muhammad Iqhrammullah, Mustafa Alhaji Isa, Benni Iskandar, Teresa R Iskander, Dr. Md. Shahinul Islam, Md Rabiul Islam, Md Shariful Islam, Farhad Islami, Nahlah Elkudssiah Ismail, Faisal Ismail, Yerlan Ismoldayev, Gaetano Isola, Masao Iwagami, Ihoghosa Osamuyi Iyamu, Mahalaxmi Iyer, Vinothini J, Jalil Jaafari, Udeme Samuel Jacob, Kathryn H Jacobsen, Ali Jadidi, Mohammadsadegh Jafari, Ali Jafari-Khounigh, Morteza Jafarinia, Vennila Jaganathan, Haitham Jahrami, Ayushi Jain, Ammar Abdulrahman Jairoun, Mihajlo Jakovljevic, Ali Jaliliyan, Mohamed Jalloh, Qazi Mohammad Sajid Jamal, Armaan Jamal, Jazlan Jamaluddin, Melika Jameie, Jerin James, Safayet Jamil, Masoud Jamshidi, Shaghayegh JamshidiRastabi, Esmaeil Jarrahi, Tahereh Javaheri, Syed Sarmad Javaid, Anita Javanmardi, Javad Javidnia, Shubha Jayaram, Ruwan Duminda Jayasinghe, Yovanthi Anurangi Jayasinghe, Achala Upendra Jayatilleke, Felix K Jebasingh, Jayakumar Jeganathan, Seogsong Jeong, Bijay Mukesh Jeswani, Zixiang Ji, Min Jiang, Wenyi Jin, Shuai Jin, Mohammad Jokar, Jost B Jonas, Darwin Phan Jones, Tamas Joo, Abu Jor, Nitin Joseph, Abel Joseph, Charity Ehimwenma Joshua, Katie Joskowitz, Kripa Josten, George Joy, Jacek Jerzy Jozwiak, Malik E Juweid, Vaishali K, Zubair Kabir, Dler H. Hussein Kadir, Ashish Kumar Kakkar, Pradnya Vishal Kakodkar, Khalil Kalavani, Sanjay Kalra, Md Moustafa Kamal, Mehnaz Kamal, Sivesh Kathir Kamarajah, Rajesh Kamath, Saltanat Kamenova, Arun Kamireddy, Ramat T Kamorudeen, Devanish Narasimhasanth Kamtam, Naser Kamyari, Oleksandr Kamyshnyi, Mona Kanaan, Saddam Fuad Kanaan, Jiseung Kang, Samuel Berchi Kankam, Kehinde Kazeem Kanmodi, Rami S Kantar, Neeti Kapoor, Sujita Kumar Kar, Paschalis Karakasis, Reema A Karasneh, Mohammad Amin Karimi, Salah Eddin Karimi, Mohmed Isaqali Karobari, Tomasz M Karpinski, Sadanand Karun, Manoj Kumar Kashyap, Eden Asmare Kassahun, Nigussie Assefa Kassaw, Nicholas J Kassebaum, Molly B Kassel, Adarsh Katamreddy, Kanica Kaushal, Foad Kazemi, Nastaran Kazemi rad, Sina Kazemian, Hafte Kahsay Kebede, Chukwudi Keke, John H Kempen, Jessica A Kerr, Emmanuelle Kesse-Guyot, Reza Khademi, Inn Kynn Khaing, Himanshu Khajuria, Nauman Khalid, Sidra Khalid, Hazim O Khalifa, Anas Husam Khalifeh, Anees Ahmed Khalil, Pantea Khalili, Anita Khalili, Alireza Khalilian, Ghazaleh Khalili-Tanha, Mohamed khalis, Faham Khamesipour, Muhammad Mueed Khan, Zahid Khan, Zahid Khan, Abdul A Khan, Md Abdullah Saeed Khan, Serab Khan, Yusuf Saleem Khan, Ramsha Mushtaq Khan, Ajmal Khan, Mohammad Jobair Khan, Muhammad Hamza Khan, Iman Waheed Khan, Muhammad Umer Khan, Sumaiya Khan, Maseer Khan, Fayaz Khan, Srijana Khanal, Shaghayegh Khanmohammadi, Zenith Khashim, Khaled Khatab, Haitham Khatatbeh, Moawiah Mohammad Khatatbeh, Kavin Khatri, Hamid Reza Khayat Kashani, Khalid A Kheirallah, Sunil Kumar Khokhar, Mohammad Saeid Khonji, Zahra Khorrami, Najmaddin Salih Husen S.H. Khoshnaw, Atulya Aman Khosla, Sepehr Khosravi, Majid Khosravi, Mahmood Khosrowjerdi, Jagdish Khubchandani, Zemene Demelash Kifle, Min Seo Kim, Hye Jun Kim, Jinho Kim, Yun Jin Kim, Ruth W Kimokoti, Yohannes Kinfu, Sanjay Kini B, Mary Kirk, Adnan Kisa, Sezer Kisa, Ladli Kishore, Juniper Boroka Kiss, Mika Kivimäki, Shivakumar KM, Ann Kristin Skrindo Knudsen, Nazarii Kobyliak, Sonali Kochhar, Michail Kokkorakis, Ali-Asghar Kolahi, Diana Gladys Kolieghu Tcheumeni, Farzad Kompani, Aida Kondybayeva, Anastasios Georgios Panagiotis Konstas, Isaac Koomson, Gerbrand Koren, Tapos Kormoker, Oleksii Korzh, Karel Kostev, Archana Koul, Sindhura Lakshmi Koulmane Laxminarayana, James-Paul Kretchy, Irene Akwo Kretchy, Kewal Krishan, Chong-Han Kua, Ananya Kuanar, Barthelemy Kuate Defo, Mohammed Kuddus, Ilari Kuitunen, Shikha Kukreti, Mukhtar Kulimbet, Vishnutheertha Kulkarni, Shweta Kulshreshtha, Sanjay Kirshan Kumar, Manasi Kumar, Dewesh Kumar, Tushar Kumar, Vijay Kumar, Jogender Kumar, Nithin Kumar, G Anil Kumar, Avinash Kumar, Jibin Kunjavara, Setor K Kunutsor, Almagul Kurmanova, Maria Dyah Kurniasari, Pramod Kumar Kushawaha, Asep Kusnali, Christina Yeni Yeni Kustanti, Dian Kusuma, Tezer Kutluk, Assylkhan Kuttybayev, Wai Hang Patrick Kwong, Grace Kwakyewaa Kyei, Evans F Kyei, Frank Kyei-Arthur, Ville Kytö, Hmwe Hmwe Kyu, Pallavi L C, Adriano La Vecchia, Carlo La Vecchia, Alessio Lachi, Muhammad Awwal Ladan, Lucie Laflamme, Chandrakant Lahariya, Daphne Teck Ching Lai, Balzhan Lakanova, Anita Lakhani, Dharmesh Kumar Lal, Ratilal Lalloo, Tea Lallukka, Iván Landires, Berthold Langguth, Ariane Laplante-Lévesque, Dylan Lasher, Kamaluddin Latief, Mahrukh Latif, Colleen L L Lau, Saheed Akinmayowa Lawal, Aliyu Lawan, Trang Diep Thanh Le, Huu-Hoai Le, Nhi Huu Hanh Le, Minh Huu Nhat Le, Thao Thi Thu Le, Caterina Ledda, Wei-Chen Lee, Ivan Lee, Seung Won Lee, Yo Han Lee, Vasileios Leivaditis, Matthew J Lennon, Matilde Leonardi, Elvynna Leong, An Li, Jinbo Li, Hui Li, Yongze Li, Jianan Li, Ming-Chieh Li, Wei Li, Wei Li, Chengfeng Li, Wang-Zhong Li, Zhengrui Li, Zhaolong Adrian Li, Weilong Li, Jiaying Li, Yanxue Lian, Xue-Zhen Liang, Virendra S Ligade, Stephen S Lim, Ro-Ting Lin, Queran Lin, Shuzhi Lin, Jialing Lin, Daniel Lindholm, Yuewei Ling, Xuefeng Liu, Xiaofeng Liu, Haipeng Liu, Xianliang Liu, Zhe Liu, Jue Liu, Gang Liu, Yubo Liu, Erand Llanaj, Michael J Loftus, Valerie Lohner, José Francisco López-Gil, Masoud Lotfizadeh, Surbala Devi Lourembam, Rafael Lozano, Shanjie Luan, Jailos Lubinda, Giancarlo Lucchetti, Susu Luo, Jay B Lusk, Angelina M Lutambi, Miltiadis D Lytras, Ellina Lytvyak, Hawraz Ibrahim M. Amin, Zheng Feei Ma, Kevin Sheng-Kai Ma, Kelsey Lynn Maass, Mahmoud Mabrok, Nikolaos Machairas, Monika Machoy, Firoozeh Madadi, Seyed Ataollah Madinezad, Christian Madsen, Aurea Marilia Madureira-Carvalho, Pasquale Maffia, Azzam A Maghazachi, D.R. Mahadeshwara Prasad, Sasikumar Mahalingam, Preeti Maharjan, Mina Maheri, Nozad Hussein Mahmood, Alireza Mahmoudi, Farhad Mahmoudi, Panagiota Maikanti-Charalampous, Rituparna Maiti, Marek Majdan, Abdelrahman M Makram, Reza Malekzadeh, Hardeep Singh Malhotra, Farihah Malik, Ahmad Azam Malik, Deborah Carvalho Malta, Mustapha Mangdow, Emery Manirambona, Lokesh Manjani, Yosef Manla, Fahmida Mannan, Kamaruddeen Mannethodi, Farheen Mansoor, Marjan Mansourian, Mohammad Ali Mansournia, Lorenzo Giovanni Mantovani, Changkun Mao, Tahir Maqbool, Hamid Reza Marateb, Joemer C Maravilla, Konstantinos Margetis, Mirko Marino, Adilson Marques, Gabriel Martinez, Bernardo Alfonso Martinez-Guerra, Ramon Martinez-Piedra, Daniela Martini, Francisco Rogerl∘ndio Martins-Melo, Miquel Martorell, Roy Rillera Marzo, Sammer Marzouk, Sugeng Mashudi, Soroush Masrouri, Clara N Matei, Yasith Mathangasinghe, Stephanie Mathieson, Alexander G Mathioudakis, Medha Mathur, Neeta Mathur, Fernanda Penido Matozinhos, Rita Mattiello, Khurshid A Mattoo, Richard James Maude, Pallab K Maulik, Erin A May, Mahsa Mayeli, Mohsen Mazidi, John J McGrath, Martin McKee, Steven M McPhail, Michael A McPhail, Enkeleint A Mechili, Rishi P Mediratta, Riffat Mehboob, Ravi Mehrotra, Vini Mehta, Tesfahun Mekene Meto, Berhanu Abebaw Mekonnen, Hadush Negash Meles, Addisu Melese, Satish Melwani, Walter Mendoza, Godfred Antony Menezes, Emiru Ayalew Mengistie, George A Mensah, Sultan Ayoub Ayoub Meo, Michelangelo Mercogliano, Tuomo J Meretoja, Atte Meretoja, Tomislav Mestrovic, Chamila Dinushi Kukulege Mettananda, Sachith Mettananda, Mohamed M M Metwally, Tomasz Miazgowski, Irmina Maria Michalek, Andrea Michelerio, Hiwot Soboksa Mideksa, Ted R Miller, Giuseppe Minervini, GK Mini, Mojgan Mirghafourvand, Seyed Ali Mirshahvalad, Mizan Kiros Mirutse, Maryam Mirzaei, Awoke Misganaw, Archana Mishra, Philip B Mitchell, Sayan Mitra, Chaitanya Mittal, Malihe Moazeni, Shivani Modi, Nouh Saad Mohamed, Mona Gamal Mohamed, Jama Mohamed, Khabab Abbasher Hussien Mohamed Ahmed, Taj Mohammad, Sakineh Mohammad-Alizadeh-Charandabi, Abdollah Mohammadian-Hafshejani, Saeed Mohammadpour, Ibrahim Mohammadzadeh, Shafiu Mohammed, Yahaya Mohammed, Hussen Mohammed, Abdulwase Mohammed, Omer Mohammed, Mustapha Mohammed, Suleiman Mohammed, Ammas Siraj Mohammed, Mohammad Mohseni, Ali H Mokdad, Sabrina Molinaro, Amirabbas Mollaei, Shaher Momani, Lorenzo Monasta, Himel Mondal, Stefania Mondello, Mohammad Ali Moni, Marco Montalti, Yousef Moradi, Maziar Moradi-Lakeh, Paula Moraga, Rafael Silveira Moreira, Shane Douglas Morrison, Mahmoud M Morsy, Reza Mosaddeghi Heris, Jonathan F Mosser, Elias Mossialos, Simin Mouodi, Mariana Mourgova, Asma Mousavi, Seyede Zohre Mousavi, Amin Mousavi Khaneghah, Seyed Mohamad Sadegh Mousavi Kiasary, Amanda Movo, Hagar Lotfy Mowafy, Kimia Mozahheb Yousefi, Matías Mrejen, Rabia Mubarak, Faraz Mughal, Syed Aun Muhammad, Oscar J Mujica, Sumoni Mukherjee, Sukhes Mukherjee, Amartya Mukhopadhyay, George Duke Mukoro, M A Muktadir, Francesk Mulita, Chalie Mulugeta, Mulyadi Mulyadi, Malaisamy Muniyandi, Kavita Munjal, Yanjinlkham Munkhsaikhan, Javier Muñoz Laguna, Michio Murakami, Efren Murillo-Zamora, B.V. Murlimanju, Sani Musa, Ali Mushtaq, Sherzad Ibrahim Mustafa, Mubarak Taiwo Mustapha, Sathish Muthu, Saravanan Muthupandian, Claude Mambo Muvunyi, Muhammad Muzaffar, Woojae Myung, Amin Nabavi, Ahamarshan Jayaraman Nagarajan, Shankar Prasad Nagaraju, Mohsen Naghavi, Ganesh R Naik, Firzan Nainu, Hastyar Hama Rashid Najmuldeen, Noureddin Nakhostin Ansari, Gopal Nambi, Vinay Nangia, Jobert Richie Nansseu, Yvonne Nartey, Bruno Ramos Nascimento, Gustavo G Nascimento, Abdallah Y Naser, Abdulqadir J Nashwan, Hamide Nasiri, Mahmoud Nassar, Zuhair S Natto, Zakira Naureen, Samidi Nirasha Kumari Navaratna, Nawsherwan, Biswa Prakash Nayak, Shalini Ganesh Nayak, Smitha Nayak, Shumaila Naz, G. Takop Nchanji, Amanuel Tebabal Nega, Masoud Negahdary, Wubshet D Negash, Ruxandra Irina Negoi, Ionut Negoi, Jalil Nejati, Nikita A Nekliudov, Samata Nepal, Henok Biresaw Netsere, Charles Richard James Newton, Marie Ng, Georges Nguefack-Tsague, Josephine W Ngunjiri, The Phuong Nguyen, Van Thanh Nguyen, Dang Nguyen, Long Nguyen, Nghia Phu Nguyen, Tu Anh Nguyen, Cuong Tat Nguyen, Ambe Marius Ngwa, Robina Khan Niazi, Luciano Nieddu, Ali Nikoobar, Vikram Niranjan, Abebe Melis Nisro, Jan Rene Nkeck, Chukwudi A Nnaji, Shuhei Nomura, Syed Toukir Ahmed Noor, Sana Noreen, Masoud Noroozi, Jean Jacques Noubiap, Valentine C Nriagu, Chisom Adaobi Nri-Ezedi, Jean Claude Nshimiyimana, Mpiko Ntsekhe, Fred Nugen, Atoma Negera Nugusa, Mengistu H Nunemo, Nurfatimah Nurfatimah, Dieta Nurrika, Sylvester Dodzi Dodzi Nyadanu, Felix Kwasi Nyande, Ogochukwu Janet Nzoputam, Bogdan Oancea, Fabio Massimo Oddi, Ismail A Odetokun, Oluwakemi Ololade Odukoya, Joseph Kojo Oduro, Michael Safo Oduro, Akinyemi O D Ofakunrin, Onome Bright Oghenetega, Oluwafunmilayo Tosin Ogundeko-Olugbami, In-Hwan Oh, Sarah Oh, Edel T O'Hagan, Hassan Okati-Aliabad, Sylvester Reuben Okeke, Deborah Oluwatosin Okeke-Obayemi, Olalekan John Okesanya, Osaretin Christabel Okonji, Oluwaseyi Isaiah Olabisi, Andrew T Olagunju, Oladotun Victor Olalusi, Matthew Idowu Olatubi, Arão Belitardo Oliveira, Gláucia Maria Moraes Oliveira, Abdulhakeem Abayomi Olorukooba, Oluseye Olalekan Oludoye, Jacob Olusegun Olusanya, Bolajoko Olubukunola Olusanya, Goran Latif Omer, Sandersan Onie, Obinna E Onwujekwe, Marcel Opitz, Aksoltan Shyhdurdyevna Oradova, Michal Ordak, Verner N Orish, Raffaele Ornello, Atakan Orscelik, Alberto Ortiz, Esteban Ortiz-Prado, Augustus Osborne, John W Ostrominski, Uchechukwu Levi Osuagwu, Olayinka Osuolale, Godfred Otchere, Elham H Othman, Adrian Otoiu, Oche Joseph Otorkpa, Abdu Oumer, Jerry John Ouner, Amel Ouyahia, Mayowa O Owolabi, Irene Amoakoh Owusu, Kolapo Oyebola, Tope Oyelade, Oyetunde T Oyeyemi, Ilker Ozsahin, Mahesh P A, Alicia Padron-Monedero, Jagadish Rao Padubidri, Dimpal Manilal Paija, Keyvan Pakshir, Tamás Palicz, Raffaele Palladino, Raul Felipe Palma-Alvarez, Tejasri Paluvai, Feng Pan, Sujogya Kumar Panda, Songhomitra Panda-Jonas, Seithikurippu R Pandi-Perumal, Carlo Irwin Able Panelo, Helena Ullyartha Pangaribuan, Georgios D Panos, Leonidas D Panos, Ioannis Pantazopoulos, Anca Pantea Stoian, Giovanni Paolino, Ilias Papadimopoulos, Paraskevi Papadopoulou, Parinaz Paranjkhoo, Shahina Pardhan, Peyvand Parhizkar Roudsari, Romil R Parikh, Chulwoo Park, Eun-Kee Park, Seoyeon Park, Arpit Parmar, Swapnil Parve, Maja Pasovic, Roberto Passera, Jay Patel, Satyananda Patel, Mitesh Patel, Sangram Kishor Patel, Hemal M Patel, Bhumi Hemal Patel, Heta Pavan Patel, Riya Jayesh Patel, Neel Navinkumar Patel, Angel J Paternina-Caicedo, Bharat Smita Umakant Patil, Shankargouda Patil, Ashlesh Patil, Apurba Patra, Venkata Suresh Patthipati, Shubhadarshini Pawar, Shrikant Pawar, Hamidreza Pazoki Toroudi, Spencer A Pease, Amy E Peden, Paolo Pedersini, Jarmila Pekarcikova, Veincent Christian Filipino Pepito, Prince Peprah, Emmanuel K Peprah, João Perdigão, Gavin Pereira, Maria Odete Pereira, Pablo Perez-Lopez, Arokiasamy Perianayagam, Norberto Perico, Simone Perna, Pavlo Petakh, Olumuyiwa James Peter, Fanny Emily Petermann-Rocha, Hoang Nhat Pham, Tung Thanh Pham, Hoang Tran Pham, Nhat Truong Pham, Anil K Philip, Michael R Phillips, Zayar Phyo, David M Pigott, Zahra Zahid Piracha, Edoardo Pirera, Moein Piroozkhkah, Enrico Pisoni, Florian Ploeckl, Evgenii Plotnikov, Dimitri Poddighe, Roman V Polibin, Ramesh Poluru, Ville T Ponkilainen, Ion Popa, Djordje S Popovic, Sajjad Pourasghary, Reza Pourbabaki, Farzad Pourghazi, Naeimeh Pourtaheri, Sergio I Prada, Pranil Man Singh Pradhan, Jalandhar Pradhan, Akila Prashant, Elton Junio Sady Prates, Harsh Priya, Nicola Riccardo Pugliese, Hery Purnobasuki, Shuby Puthussery, Jagadeesh Puvvula, Nameer Hashim Qasim, Zhipeng Qi, Xiang Qi, Jia-Yong Qiu, Zahiruddin Syed Quazi, Basuki Rachmat, Raghu Anekal Radhakrishnan, Hadi Raeisi Shahraki, Alberto Raggi, Pracheth Raghuveer, Hawbash Mohammed-Amin Rahim, Sajjad Rahimi, Vafa Rahimi-Movaghar, Muhammad Aziz Rahman, Mahbubur Rahman, Fryad Majeed Rahman, Md Mijanur Rahman, Mohammad Hifz Ur Rahman, Md. Mosfequr Rahman, Mosiur Rahman, Amir Masoud Rahmani, Saeed Rahmani, Masoud Rahmati, Ghasem Rahmatpour Rokni, Hakim Rahmoune, Ivano Raimondo, Diego Raimondo, Sunil Kumar Raina, Jeffrey Pradeep Raj, Adarsh Raja, Sandesh Raja, Erta Rajabi, Gunaseelan Rajendran, Judah Rajendran, Mohammad Amin Rajizadeh, Mahmoud Mohammed Ramadan, Majed Ramadan, Kadar Ramadhan, Chitra Ramasamy, Shakthi Kumaran Ramasamy, Sheena Ramazanu, Zahra Ramezani, Marzieh Ramezani Farani, Juwel Rana, Chhabi Lal Ranabhat, Nemanja Rancic, Smitha Rani, Kumuda Rao, Mithun Rao, Chythra R Rao, Davide Rasella, Vahid Rashedi, Mohammad-Mahdi Rashidi, Ashkan Rasouli-Saravani, Prateek Rastogi, Azad Rasul, Devarajan Rathish, Abdur Rauf, Santosh Kumar Rauniyar, Ilari Rautalin, Ramin Ravangard, Dhwani Ravi, David Laith Rawaf, Reza Rawassizadeh, Ramu Rawat, Bahman Razi, Christian Razo, Murali Mohan Rama Krishna Reddy, Elrashdy Redwan, Sanika Rege, Wajiha Rehman, Rainer Reile, Giuseppe Remuzzi, Bhageerathy Reshmi, Stefano Restaino, Mina Rezaei, Marzieh Rezaei, Nazila Rezaei, Mohsen Rezaeian, Taeho Gregory Rhee, Antonio Luiz P Ribeiro, Tércia Moreira Ribeiro da Silva, Jennifer Rickard, Hannah Elizabeth Robinson-Oden, Hermano Alexandre Lima Rocha, João Rocha Rocha-Gomes, Alfonso J. Rodriguez-Morales, Leonardo Roever, Ravi Rohilla, Iftitakhur Rohmah, Susanne Röhr, David Rojas-Rueda, Megan L Rolfzen, Debby Syahru Romadlon, Michele Romoli, Luca Ronfani, Moustaq Karim Khan Rony, Jennifer Jacqueline Rosauer, Emily Rosenblad, Amirhossein Roshanshad, Morteza Rostamian, Kunle Rotimi, Himanshu Sekhar Rout, Shiva Rouzbahani, Reza Rouzbahani, Hanieh Rouzbahani, Adrija Roy, Nitai Roy, Priyanka Roy, Parimal Roy, Bedanta Roy, Sharmistha Roy, Simanta Roy, Simanta Roy, Shubhanjali Roy, Parameswari Royapuram Parthasarathy, Enrico Rubagotti, Susan Fred Rumisha, Michele Russo, Godfrey M Rwegerera, Poorvikha S, Chandan S N, Aly M A Saad, Zahra Saadatian, Michela Sabbatucci, Korosh Saber, Maha Mohamed Saber-Ayad, Cameron John Sabet, Siamak Sabour, Perminder S Sachdev, Kabir P Sadarangani, Seyed Kiarash Sadat Rafiei, Basema Ahmad Saddik, Adam Saddler, Bashdar Abuzed Sadee, Tarannom Sadegh, Erfan Sadeghi, Ehsan Sadeghi, Fatemeh Sadeghi-Ghyassi, Umar Saeed, Mohd Saeed, Maryam Saeedi, Mehdi Safari, Mahdi Safdarian, Sher Zaman Safi, Rajesh Sagar, Mastooreh Sagharichi, Amene Saghazadeh, Dominic Sagoe, Indranil Saha, Nondo Saha, Narjes Saheb Sharif-Askari, Fatemeh Saheb Sharif-Askari, Amirhossein Sahebkar, Biniyam Sahiledengle, Gülsüm Sahin Bodur, Pragyan Monalisa Sahoo, Zahra Saif, S Mohammad Sajadi, Md Refat Uz Zaman Sajib, Mirza Rizwan Sajid, Payman Salamati, Luciane B Salaroli, Mohamed A Saleh, Mahdi Salehi, Mohammed Z Y Salem, Marwa Rashad Salem, Dauda Salihu, Sohrab Salimi, Pegah Salimi Pormehr, Malik Sallam, Hossein Samadi Kafil, Saad Samargandy, Yoseph Leonardo Samodra, Abdallah M Samy, Sandeep G Sangle, Elaheh Sanjari, Sathish Sankar, Francesca Sanna, Lucas H C C Santos, Milena M Santric-Milicevic, Haaris Saqib, Sivan Yegnanarayana Iyer Saraswathy, Jacob Owusu Owusu Sarfo, Yaser Sarikhani, Tanmay Sarkar, Hemen Sarma, Mohammad Sarmadi, Sachin C Sarode, Gargi Sachin Sarode, Benn Sartorius, Arash Sarveazad, Michele Sassano, Brijesh Sathian, Mukesh Kumar Sathya Narayanan, Maheswar Satpathy, Jennifer Saulam, Mehrdad Savabi Far, Kimia Savoji, Monika Sawhney, Ganesh Kumar Saya, Abu Sayeed, Christophe Schinckus, Jurgen Carlo Schmidt, Maria Inês Schmidt, Aletta Elisabeth Schutte, Ghil Schwarz, David C Schwebel, Falk Schwendicke, Catherine Schwinger, Mario Šekerija, Siddharthan Selvaraj, Yuliya Semenova, Mohammad H Semreen, Ashenafi Kibret Sendekie, Yigit Can Senol, Subramanian Senthilkumaran, Sadaf G Sepanlou, Edson Serván-Mori, Yashendra Sethi, Seyed Mohammad Seyed Alshohadaei, Allen Seylani, Abubakar Sha'aban, Mahan Shafie, Arezoo Shafieioun, Muhammad Shahab, Shazlin Shaharudin, Muhammad Shahbaz, Syed Ahsan Shahid, Samiah Shahid, Wajeehah Shahid, Endrit Shahini, Farshad Shahkarami, Fatemeh Shahrahmani, Hamid R Shahsavari, Moyad Jamal Shahwan, Masood Ali Shaikh, Nafhat Shaikh, Alireza Shakeri, Ali Shakerimoghaddam, Ali S Shalash, Sunder Sham, Muhammad Aaqib Shamim, Mehran Shams-Beyranvand, Anas Shamsi, Alfiya Shamsutdinova, Dan Shan, Mohd Shanawaz, Abhishek Shankar, Ben David Geller Shapiro, Amin Sharifan, Javad Sharifi Rad, Manoj Sharma, Ravi Kumar Sharma, Bunty Sharma, Ujjawal Sharma, Avimanu Sharma, Bhoopesh Kumar Sharma, Vishal Sharma, Armin Shavandi, Ramzi Shawahna, Maryam Shayan, Ali Sheidaei, Aziz Sheikh, Mahabalesh Shetty, Suraj S Shetty, Lin-Hong Shi, Fang Shi, Belayneh Fentahun Shibesh, Desalegn Shiferaw, Tariku Shimels, Md Monir Hossain Shimul, Min-Jeong Shin, Rahman Shiri, Reza Shirkoohi, Aminu Shittu, Abdul-karim Olayinka Shitu, Ivy Shiue, Velizar Shivarov, Ambreen Shoaib, Shayan Shojaei, Sina Shool, Seyed Afshin Shorofi, Sunil Shrestha, Suleiman Adeiza Adeiza Shuaibu, Kerem Shuval, Nicole R S Sibuyi, Emmanuel Edwar Siddig, Mohammad Sidiq, Luís Manuel Lopes Rodrigues Silva, Diego Augusto Santos Silva, Noah Joseph Bernard Silva de Leonardi, Biagio Simonetti, Amit Singh, Balbir Bagicha Singh, Jasvinder A Singh, Baljinder Singh, Harmanjit Singh, Narinder Pal Singh, Puneetpal Singh, Satwinder Singh, Poornima Suryanath Singh, Akanksha Singh, Harpreet Singh, Surendra Singh, Bhim Pratap Singh, Kalpana Singh, Samer Singh, Abhinav Singh, Mukesh Kumar Sinha, Robert Sinto, Freddy Sitas, Dagne Feleke Siyoum, Natia Skhvitaridze, Valentin Yurievich Skryabin, Anna Aleksandrovna Skryabina, David A Sleet, Aalam Sohal, Md. Salman Sohel, Somaye Sohrabi, Anton Sokhan, Shipra Solanki, Solikhah Solikhah, Sameh S M Soliman, Aayushi Sood, Prashant Sood, Soroush Soraneh, Joan B Soriano, Michele Sorrentino, Fernando Sousa, Ireneous N Soyiri, Michael Spartalis, Chandrashekhar T Sreeramareddy, Bahadar S Srichawla, Shyamkumar Sriram, Devin Bailey Srivastava, Jeffrey D Stanaway, Nicholas Steel, Aleksandar Stevanovic, Sebastian Straube, Peter Stubbs, Omer Subasi, Narayan Subedi, Vetriselvan Subramaniyan, Hasnat Sujon, Thitiporn Sukaew, Surajo Kamilu Sulaiman, Auwal Garba Suleiman, Muhammad Suleman, Desy Sulistiyorini, Mark J M Sullman, Jing Sun, Haitong Zhe Sun, Xiaohui Sun, Mao-ling Sun, Zhuanlan Sun, Suraj Sundaragiri, David Sunkersing, Sumam Sunny, Chandan Kumar Swain, Tasmin L Symons, Lukasz Szarpak, Mindy D Szeto, Sree Sudha T Y, Payam Tabaee Damavandi, Rafael Tabarés-Seisdedos, Seyyed Mohammad Tabatabaei, Fatemeh Sadat Tabatabaei, Seyed Shahaboddin Tabatabaei, Shima Tabatabai, Celine Tabche, Ramin Tabibi, Mohammad Tabish, Takahiro Tabuchi, Santosh Kumar Tadakamadla, Buhari Abdullahi Tafida, Farzad Taghizadeh-Hesary, Zanan Mohammed-Ameen Taha, Yasaman Taheri Abkenar, Shima Tajabadi, Iman M Talaat, Mircea Tampa, Jacques Lukenze Tamuzi, Ker-Kan Tan, Shynar Tanabayeva, Haosu Tang, Guodong Tang, Mohsan Tanveer, Sarvenaz Taridashti, Ingan Ukur Tarigan, Mengistie Kassahun Tariku, Saba Tariq, Anika Tasnim, Seyed Mohammad Tavangar, Mebrahtu G. Tedla, Mohamad-Hani Temsah, Reem Temsah, Masayuki Teramoto, Azimeraw Arega Tesfu, Jay Tewari, Alireza Teymouri, Chandan Kumar Thakur, Rekha Thapar, Ismaeel Tharwat, Samar Tharwat, Hadiza Theyra-Enias, Arun James Thirunavukarasu, Muthu Thiruvengadam, Manuel Sebastian Thomas, Jansje Henny Vera Ticoalu, Mariya Vladimirovna Titova, Yves Joel Tochie Noutakdie, Sojit Tomo, Marcello Tonelli, Roman Topor-Madry, Ali Torkashvand, Mathilde Touvier, Marcos Roberto Tovani-Palone, Khaled Trabelsi, Thang Huu Tran, Quynh Thuy Huong Tran, Tam Quoc Minh Tran, Mai Thi Ngoc Tran, Nguyen Tran Minh Duc, Domenico Trico, Indang Trihandini, Samuel Joseph Tromans, Claudia Truppa, Gary Tse, Evangelia Eirini Tsermpini, Munkhtuya Tumurkhuu, Zhouting Tuo, Biruk Shalmeno Tusa, Sok Cin Tye, Stefanos Tyrovolas, Aniefiok John Udoakang, Himayat Ullah, Saeed Ullah, Atta Ullah, Riaz Ullah, Muhammad Umair, Lawan Umar, Muhammad Umar, Muhammad Umar, Bhaskaran Unnikrishnan, Dinesh Upadhya, Era Upadhyay, Dipan Uppal, Jibrin Sammani Usman, Kelechi Julian Uzor, Hande Uzunçibuk, Pratyusha Vadagam, Asokan Govindaraj Vaithinathan, Pascual R Valdez, Mario Valenti, Zahir Vally, Jef Van den Eynde, Javad Varasteh, Joe Varghese, Priya Vart, Santosh Varughese, Tommi Juhani Vasankari, Sampara Vasishta, Srivatsa Surya Vasudevan, Alireza Vaysi, Ashleigh S Vella, Balachandar Vellingiri, Gowri Venkatraman Subramanian, Narayanaswamy Venketasubramanian, Nicholas Alexander Verghese, Poonam Verma, Megan Verma, Madhur Verma, Massimiliano Veroux, Georgios-Ioannis Verras, Dominique Vervoort, Ramesh Vidavalur, Simone Villa, Jorge Hugo Villafañe, David Villarreal-Zegarra, Francesco S Violante, Sharath Chaitanya Vipparthy, Luciano Magalhães Vitorino, Stein Emil Vollset, Avina Vongpradith, Theo Vos, Elpida Vounzoulaki, Linh Vu, Henok Toga Wada, Yasir Waheed, Megha Walia, Lindsey E Wallace, Agnes Wamuyu Wamai, Jin-Yi Wan, Arvinder Wander, Shu Wang, Ruixuan Wang, Xing Wang, Shaopan Wang, Qingzhi Wang, Jinyu Wang, Fang Wang, Wei Wang, Yanzhong Wang, Denny Wang, Youxin Wang, Wanzhou Wang, Yuan-Pang Wang, Mary Njeri Wanjau, Ahmed Bilal Waqar, Muhammad Waqas, Paul Ward, Stefanie Watson, Kosala Gayan Weerakoon, Fei-Long Wei, Xueying Wei, Robert G Weintraub, Daniel J Weiss, Ronny Westerman, Joanna L Whisnant, Taweewat Wiangkham, Yohanes Cakrapradipta Wibowo, Anggi Lukman Wicaksana, Nuwan Darshana Darshana Wickramasinghe, Dakshitha Praneeth Wickramasinghe, Angga Wilandika, Peter Willeit, Andrew Awuah Wireko, Gemechu Kumera Wirtu, Charles Shey Wiysonge, Abay Tadesse Woday, Marcin W Wojewodzic, Axel Walter Wolf, Tewodros Eshete Wonde, Yen Jun Wong, Daniel Tarekegn Worede, Minichil Chanie Chanie Worku, James Fan Wu, Zenghong Wu, Jinyi Wu, Felicia Wu, Peng Wu, Yihun Miskir Wubie, Qing Xia, Zhijia Xia, Hong Xiao, Lishun Xiao, Guangqin Xiao, Na Xiao, Wanqing Xie, Wanqing Xu, Xiaoyue Xu, Suowen Xu, Wang-Dong Xu, Site Xu, Mingyang Xue, Mukesh Kumar Yadav, Vikas Yadav, Sajad Yaghoubi, Saba Yahoo (Syed), Kazumasa Yamagishi, Xinxin Yang, Haibo Yang, Yuichiro Yano, Haiqiang Yao, Laiang Yao, Amir Yarahmadi, Habib Yaribeygi, Haya Yasin, Mohamed A Yassin, Yuichi Yasufuku, Sanni Yaya, Pengpeng Ye, Meghdad Yeganeh, Ali Cem Yekdes, Mohammad Hossein YektaKooshali, Getaneh Atikilt Yemata, Subah Abderehim Yesuf, Saber Yezli, Siyan Yi, Muluken Yigezu, Dehui Yin, Yazachew Engida Yismaw, Malede Berihun Yismaw, Dong Keon Yon, Naohiro Yonemoto, Mustafa Z Younis, Abdilahi Yousuf, Jian Yu, Yong Yu, Chuanhua Yu, Hui Yuan, Ghazala Yunus, Umar Yunusa, Siddhesh Zadey, Vesna Zadnik, Mubashir Zafar, Manijeh Zaghampour, Mondal Hasan Zahid, Emilia Zainal Abidin, Fathiah Zakham, Giulia Zamagni, Sojib Bin Zaman, Abu Sarwar Zamani, Hussaini Zandam, Alireza Zangeneh, Aurora Zanghì, Iman Zare, Kourosh Zarea, Shirin Zaresharifi, Michael Zastrozhin, Mohammed Zawiah, Mohammed G M Zeariya, Dawit Zemedikun, Abay Mulu Zenebe, Sebastian Zensen, Eyael M Zeru, Tiansong Zhan, Yongle Zhan, Xiaoyi Zhang, Liqun Zhang, Haijun Zhang, Yunquan Zhang, Beijian Zhang, Ning Zhang, Xiu-Hang Zhang, Meixin Zhang, Zhiqiang Zhang, Casper J P Zhang, Jinpeng Zhang, Zhongyi Zhao, Shenglin Zhao, Sheng Zhao, Ming-Hua Zheng, Anthony Zhong, Claire Chenwen Zhong, Juexiao Zhou, Jiayan Zhou, Bin Zhu, Abzal Zhumagaliuly, Hafsa Zia, Ghazal Zoghi, Mohamed Ali Zoromba, Rafat Mohammad Zrieq, Liesl J Zuhlke, Lilik Zuhriyah, Alimuddin Zumla, Sa'ed H Zyoud, Shaher H Zyoud, Ahed H Zyoud, Aleksandr Y Aravkin, Christopher J L Murray

**Affiliations:** A Institute for Health Metrics and Evaluation, University of Washington, Seattle, WA, USA; B Department of Health Metrics Sciences, School of Medicine, University of Washington, Seattle, WA, USA; C Amity Institute of Public Health, Amity University, Uttar Pradesh, India; D Shahid Beheshti University of Medical sciences, Shahid Beheshti University of Medical Sciences, Tehran, Iran; E Department of Nursing, Al Zaytoonah University of Jordan, Amman, Jordan; F Department of Radiation Oncology, Massachusetts General Hospital, Boston, MA, USA; G School of Health & Life Sciences, University of the West of Scotland, Paisley, UK; H Department of Medical Rehabilitation, University of Nigeria Nsukka, Enugu, Nigeria; I Department of Legal and Economic Studies, La Sapienza University, Rome, Italy; J Centre for Regenerative Medicine and Health, Chinese Academy of Sciences, Hong Kong, China; K Department of Neuroscience, City University of Hong Kong, Hong Kong, China; L Department of Internal Medicine, Rafsanjan University of Medical Sciences, Rafsanjan, Iran; M Clinical Research Development Unit, Rafsanjan University of Medical Sciences, Rafsanjan, Iran; N Department of Medicine, University of California San Francisco, San Francisco, CA, USA; O College of Pharmacy, Ajman University, Ajman, United Arab Emirates; P Department of Epidemiology, Alexandria University, Alexandria, Egypt; Q Hull York Medical School, University of Hull, Hull, UK; R Department of Biology, Qassim University, Buraydah, Saudi Arabia; S School of Nursing, The Univeristy of Jordan, Amman, Jordan; T Basic Science Department, University of Ha'il, Hail, Saudi Arabia; U Chemistry Department, Al-Azhar University, Cairo, Egypt; V Department of Medical Laboratory Science, University of Sharjah, Sharjah, United Arab Emirates; W Department of Tropical Medicine and Infectious Diseases, Tanta University, Tanta, Egypt; X Internal Medicine Department, Al-Azhar University, Cairo, Egypt; Y Department of Medicine, Memorial University, St. John's, NL, Canada; Z Minimally Invasive Surgery Research Center, Iran University of Medical Sciences, Tehran, Iran; AA Department of Medicine, University of Setif Algeria, Sétif, Algeria; AB Department of Health, Sétif, Algeria; AC Faculty of Veterinary Medicine, Islamic Azad University, Karaj, Iran; AD Community and Maternity Nursing Unit, University of Duhok, Duhok, Iraq; AE National Institute of Epidemiology, Indian Council of Medical Research, Chennai, India; AF Department of Physiotherapy, Bayero University Kano, Kano, Nigeria; AG Department of Physiotherapy, Federal University Wukari, Wukari, Nigeria; AH Department of Pharmacognosy Faculty of Pharmacy, University of Lagos, Lagos, Nigeria; AI Department of Epidemiology and Biostatistics, University of Gondar, Gondar, Ethiopia; AJ Yale School of Medicine, Iran University of Medical Sciences, New Haven, USA; AK Neuroendocrine Department, Harvard University, Boston, MA, USA; AL Department of Emergency Medicine, Zanjan University of Medical Sciences, Zanjan, Iran; AM School of Pharmacy, Bahir Dar University, Bahir Dar, Ethiopia; AN Postgraduate Department, University of Sierra Sur, Miahuatlan de Porfirio Diaz, Mexico; AO Yhteiskuntadatatieteen Keskus (Centre for Social Data Science), University of Helsinki, Helsinki, Finland; AP Department of Biomedical Sciences, Nazarbayev University School of Medicine, Astana, Kazakhstan; AQ Department of Midwifery, Bahir Dar University, Bahir Dar, Ethiopia; AR Department of Community Medicine, Babcock University, Ilishan-Remo, Nigeria; AS Department of Internal Medicine, Federal Medical Centre, Abuja, Nigeria; AT Department of Family and Community Health, University of Health and Allied Sciences, Ho, Ghana; AU School of Population Health, University of New South Wales, Sydney, NSW, Australia; AV Cardiovascular Research Center, Massachusetts General Hospital, Boston, MA, USA; AW Department of Radiology, Harvard University, Boston, MA, USA; AX Department of Sport, Exercise and Rehabilitation, Northumbria University, Newcastle, UK; AY Basic Science Department, Preparatory Year, University of Ha'il, Ha'il, Saudi Arabia; AZ Zoology Department, Faculty of Science, Benha University, Benha, Egypt; BA Department of Physical Pharmacy and Pharmacokinetics, Poznan University of Medical Sciences, Poznan, Poland; BB Cardiovascular Disease, Loma Linda University Medical Center, Loma Linda, CA, USA; BC Department of Pediatric Dentistry, Federal University of Minas Gerais, Belo Horizonte, Brazil; BD Department of Anesthesiology, Shahid Beheshti University of Medical Sciences, Tehran, Iran; BE Clinical Pharmacy and Therapeutics Department, Applied Science Private University, Amman, Jordan; BF Community Helth Nursing Department, Jouf University, Sakaka, Saudi Arabia; BG Graduate School of Public Health, St. Luke's International University, Tokyo, Japan; BH Division of Population Data Science, National Cancer Center, Tokyo, Japan; BI Department of Pharmacology and Toxicology, Usmanu Danfodiyo University, Sokoto, Sokoto, Nigeria; BJ Nigerian Institute of Medical Research, Lagos, Nigeria; BK Clinical Sciences Department, University of Sharjah, Sharjah, United Arab Emirates; BL Department of Biopharmaceutics and Clinical Pharmacy, University of Jordan, Amman, Jordan; BM Department of Nursing, University of Sharjah, Sharjah, United Arab Emirates; BN Maternal and Child Health Nursing, Jordan University of Science and Technology, irbid, Jordan; BO Department Pharmacy Practice and Pharmacotherapeutics, University of Sharjah, Sharjah, United Arab Emirates; BP Medical Research Center, Hamad Medical Corporation, Doha, Qatar; BQ Department of Pharmacology and Therapeutics, United Arab Emirates University, Al Ain, United Arab Emirates; BR College of Pharmacy, University of Jordan, Amman, Jordan; BS Department of Pharmacy, Hamad Medical Corporation, Doha, Qatar; BT Department of Biochemistry and Molecular Medicine, Alfaisal University, Riyadh, Saudi Arabia; BU College of Graduate Health Sciences, University of Tennessee, Memphis, TN, USA; BV Department of Restorative Dentistry, University of Sharjah, Sharjah, United Arab Emirates; BW Department of Population Health, Hofstra University, Hempstead, NY, USA; BX Department of Clinical Medicine, American University of Antigua, Coolidge, Antigua and Barbuda; BY FIU Robert Stempel College of Public Health & Social Work, Florida International University, Miami, FL, USA; BZ Melbourne School of Population and Global Health, University of Melbourne, Melbourne, VIC, Australia; CA Department of Diagnostic and Interventional Radiology, Technical University of Munich, Munich, Germany; CB Stanford University, Palo Alto, CA, USA; CC Institute of Cardiovascular Diseases, University of Ibadan, Ibadan, Nigeria; CD University College Hospital, Ibadan, Ibadan, Nigeria; CE Department of Microbiology, Ladoke Akintola University, Osogbo, Nigeria; CF NMC Healthcare, Independent Consultant, Sharjah, United Arab Emirates; CG School of Basic and Biomedical Sciences, University of Health and Allied Sciences, Ho, Ghana; CH Department of Immunology, Roswell Park Comprehensive Cancer Center, Buffalo, NY, USA; CI Graduate Program Division, University at Buffalo, Buffalo, NY, USA; CJ Department of Pediatrics, East Tennessee State University, Johnson City, TN, USA; CK Center for Cardiovascular Risk Research, Center for Cardiovascular Risk Research, Johnson City, TN, USA; CL Translational Research Team, The University of Sydney, Sydney, NSW, Australia; CM Melanoma Institute Australia, The University of Sydney, Sydney, NSW, Australia; CN Department of Family Medicine, Bowen University, Iwo, Nigeria; CO Department of Family Medicine, Bowen University Teaching Hospital, Ogbomoso, Nigeria; CP Slum and Rural Health Initiative Research Academy, Slum and Rural Health Initiative, Ibadan, Nigeria; CQ Department of Physiotherapy, University of Ibadan, Ibadan, Nigeria; CR Department of Microbiology, University of Medical Sciences, Ondo, Ondo City, Nigeria; CS Division of Biostatistics and Epidemiology, Stellenbosch University, Cape Town, South Africa; CT Department of Pharmacology and Therapeutics, University of Medical Sciences, Ondo, Ondo, Nigeria; CU Department of Veterinary Medicine, University of Ibadan, Ibadan, Nigeria; CV School of Public Health, Mekelle University, Mekelle, Ethiopia; CW Department of Community Medicine, Tribhuvan University, Bharatpur, Nepal; CX Public Health Section, Himalayan Environment and Public Health Network (HEPHN), Chitwan, Nepal; CY Department of Fisheries and Marine Bioscience, Jashore University of Science and Technology, Jashore, Bangladesh; CZ Research School of Population Health, Australian National University, Canberra, ACT, Australia; DA Apollo Institute Of Medical Sciences & Research Chittoor, Apollo Hospital, Chittoor, India; DB School of Medicine, Tehran University of Medical Sciences, Tehran, Iran; DC Department of Biology, University of Hail, Hail, Saudi Arabia; DD Department of Public Health, Universitas Padjadjaran (Padjadjaran University), Bandung, Indonesia; DE Department of Epidemiology and Biostatistics, University of Health and Allied Sciences, Ho, Ghana; DF National Institute on Minority Health and Health Disparities, National Institutes of Health, Bethesda, MD, USA; DG Department of Public Health and Preventive Medicine, University of Naples “Federico II”, Naples, Italy; DH Technical Services Directorate, MSI Nigeria Reproductive Choices, Abuja, Nigeria; DI Department of Epidemiology and Medical Statistics, University of Ibadan, Ibadan, Nigeria; DJ Department of Community Medicine, King Edward Memorial Hospital, Lahore, Pakistan; DK Department of Public Health, Public Health Institute, Lahore, Pakistan; DL Department of Public Health, Wachemo University, Hossana, Ethiopia; DM MM College of Pharmacy, Maharishi Markandeshwar (Deemed to be University), Ambala, India; DN Department of Orthopedic Surgery and Sports Medicine, Boston Children's Hospital, boston, MA, USA; DO Department of Neurosurgery, Alborz University of Medical Sciences, Karaj, Iran; DP Neuroscience Research Center, Iran University of Medical Sciences, Tehran, Iran; DQ Urology Research Center, Tehran University of Medical Sciences, Tehran, Iran; DR Department of Health Education and Health Promotion, Wachemo University, Hossana, Ethiopia; DS Department of Biosciences and Biotechnology, University of Medical Sciences, Ondo, Ondo City, Nigeria; DT Health Research and Innovation Sciences Center, Klaipeda University, Klaipeda, Lithuania; DU SPRINT Sport Physical Activity and Health Research & Innovation Center, Polytechnic Institute of Guarda, Guarda, Portugal; DV Trivedi School of Biosciences, Ashoka University, Sonipat, India; DW Department of Public Health Sciences, Queen's University, Kingston, ON, Canada; DX School of Public Health, University of Technology Sydney, Sydney, NSW, Australia; DY Department of Clinical Pharmacy, Universiti Sains Malaysia, Penang, Malaysia; DZ Department of Pharmacy Practice, The Islamia University of Bahawalpur, Bahawalpur, Pakistan; EA School of Medicine and Psychology, Australian National University, Canberra, ACT, Australia; EB Health Research Institute, University of Canberra, Canberra, ACT, NSW, Australia; EC Biological Production Unit National Institute of Health Islamabad Pakistan, National Institute of Health, Islamabad, Pakistan; ED World Health Organization, World Health Organisation, Islamabad, Pakistan; EE College of Medicine, Shaqra University, Shaqra, Saudi Arabia; EF School of Nursing, University of Jordan, Amman, Jordan; EG Department of Health Informatics, Qassim University, Buraidha, Saudi Arabia; EH School of Public Health, Zhejiang University, Hangzhou, China; EI College of Medicine, University of Cincinnati, Cincinnati, OH, USA; EJ Institute of Research and Development, Duy Tan University, Da Nang, Viet Nam; EK Centre for Research Impact & Outcome, Chitkara University, Punjab, India; EL College of Medicine and Public Health, Flinders University, Adelaide, SA, Australia; EM Faculty of Public Health, Jimma University, Jimma, Ethiopia; EN Institute of Endemic Diseases, University of Khartoum, Khartoum, Sudan; EO Swiss Tropical and Public Health Institute, University of Basel, Basel, Switzerland; EP Department of Pharmacy Practice, Riphah Institute of Pharmaceutical Sciences, Islamabad, Pakistan; EQ Division of Infectious Diseases and Global Public Health (IDGPH), University of California San Diego, San Diego, CA, USA; ER Maternal and Child Health Division, International Centre for Diarrhoeal Disease Research, Bangladesh, Dhaka, Bangladesh; ES Department of Women's and Children's Health, Uppsala University, Uppsala, Sweden; ET Department of Medicine, Rawalpindi Medical University, Rawalpindi, Pakistan; EU Department of Assistance Medical Sciences, University of Tabuk, Tabuk, Saudi Arabia; EV Department of Psychology, University of Chittagong, Chattogram, Bangladesh; EW Department of Pathology and Microbiology, University of Duhok, Duhok, Iraq; EX Department of Veterinary Microbiology, University of Ilorin, Ilorin, Nigeria; EY College of Nursing, Majmaah University, Al Majmaah, Saudi Arabia; EZ Brody School of Medicine, East Carolina University, Greenville, NC, USA; FA Medical Laboratory Science Department, University of Human Development, Sulaymaniyah, Iraq; FB Department of Biosciences, COMSATS Institute of Information Technology, Islamabad, Pakistan; FC Institute of Public Health, United Arab Emirates University, Al Ain, United Arab Emirates; FD School of Medicine, Nazarbayev University, Astana, Kazakhstan; FE Clinical Academic Department of Women's Health, University Medical Center, NU Medicine, Astana, Kazakhstan; FF Department of Orthopedics, Yenepoya Medical College, Mangalore, India; FG National Nutrition and Food Technology Research Institute, Shahid Beheshti University of Medical Sciences, Tehran, Iran; FH Faculty of Medicine and Public Health, Jenderal Soedirman University, Purwokerto, Indonesia; FI St George and Sutherland Clinical School, University of New South Wales, Sydney, NSW, Australia; FJ Department of Water Engineering, Graduate University of Advanced Technology, Kerman, Iran; FK Oxford Vaccine Group, University of Oxford, Oxford, UK; FL Department of Physiology, Ladoke Akintola University, Ogbomoso, Nigeria; FM University Institute of Diet and Nutritional Sciences, The University of Lahore, Lahore, Pakistan; FN Department of Internal Medicine, University of Patras, Patras, Greece; FO Department of Internal Medicine and Infectious Diseases, University General Hospital of Patras, Patras, Greece; FP Biomedical Engineering Department, University of Michigan, Ann Arbor, MI, USA; FQ School of Medicine, George Washington University, Washington, DC, USA; FR Department of Cardiology, Heart, Vascular, and Thoracic Institute, Fudan University, Shanghai, China; FS Faculty of Health and Behavioural Sciences, The University of Queensland, Brisbane, Queensland (QLD), Australia; FT Department of Infection Prevention & Control, Baylor Scott & White Health, Frisco, TX, USA; FU VentrureBlick; FV Chicago College of Osteopathic Medicine, Midwestern University, Downers Grove, IL, USA; FW Feinberg School of Medicine, Northwestern University, Chicago, IL, USA; FX Department of Geriatric and Long Term Care, Hamad Medical Corporation, Doha, Qatar; FY Rumailah Hospital, Hamad Medical Corporation, Doha, Qatar; FZ Department of Surgery, Washington University in St. Louis, St. Louis, MO, USA; GA The University of Jordan, Jordanian Public Health Society, Amman, Jordan; GB American University in the Emirates, Dubai, United Arab Emirates; GC Fundamentals and Administration Department, Sultan Qaboos University, Muscat, Oman; GD Al Al-Bayt University, Mafraq, Jordan; GE Jordan Medical Association, Amman, Jordan; GF Faculty of Pharmacy, Philadelphia University, Amman, Jordan; GG School of Pharmacy, Cardiff University, Cardiff, UK; GH Department of Adult Health and Critical Care, Sultan Qaboos University, Muscat, Oman; GI School of Public Health, University of Texas, Houston, TX, USA; GJ The University of Jordan School of Medicine, University of Jordan, Amman, Jordan; GK Department of Biology, University of Bahrain, Zallaq, Bahrain; GL Department of Research and Development, Washington University in St. Louis, St. Louis, MO, USA; GM Clinical Epidemiology Center, US Department of Veterans Affairs (VA), St. Louis, MO, USA; GN Preventive Dentistry Department, Jouf University, Sakaka, Saudi Arabia; GO Murdoch Business School, Murdoch University, Perth, WA, Australia; GP Department of Oral and Maxillofacial Surgery, Shahid Beheshti University of Medical Sciences, tehran, Iran; GQ Department of Bioengineering, George Mason University, Fairfax, VA, USA; GR School of Nursing, Yarmouk University, Irbid, Jordan; GS School of Nursing and Midwifery, Western Sydney University, Sydney, NSW, Australia; GT Department of Nursing and Midwifery, Woldia University, Woldia, Ethiopia; GU Department of Surgery, Hamad Medical Corporation, Doha, Qatar; GV Department of Health Information Management and Technology, Imam Abdulrahman Bin Faisal University, Dammam, Saudi Arabia; GW Department of Clinical Pharmacy, Al-Ayen Iraqi University, Thi-Qar, Iraq; GX Department of Clinical Pharmacy and Pharmacy Practice, University of Science and Technology, Sana'a, Yemen; GY Department of Community and Mental Health, Al al-Bayt University, Mafraq, Jordan; GZ Division of Pediatric Cardiology, University of Colorado, Aurora, CO, USA; HA Heart Center, King Faisal Specialist Hospital & Research Center, Riyadh, Saudi Arabia; HB General Directorate of Research and Studies, Ministry of Health, Riyadh, Saudi Arabia; HC Institute of Health Informatics, University College London, London, UK; HD College of pharmacy, University of Sharjah, Sharjah, United Arab Emirates; HE School of Pharmacy, The University of Jordan, Amman, Jordan; HF Pediatric Intensive Care Unit, King Saud University, Riyadh, Saudi Arabia; HG Department of Epidemiology and Biostatistics, University of South Carolina, Columbia SC, SC, USA; HH Department of Bacteriology, Immunology, and Mycology, Suez Canal University, Ismailia, Egypt; HI College of Nursing, Qatar University, Doha, Qatar; HJ Department of Health Services and Hospital Administration, King Abdulaziz University, Jeddah, Saudi Arabia; HK Health Economics Research Group, King Abdulaziz University, Jeddah, Saudi Arabia; HL Faculty of Applied Health Sciences (Physiotherapy), Tishk International University, Erbil, Iraq; HM Faculty of Dentistry, Ibn Al-Nafis University for Medical Sciences, Sana'a, Yemen; HN College of Applied Medical Sciences, Qassim University, Buraydah, Saudi Arabia; HO Centre for Biotechnology and Microbiology, University of Swat, Charbagh, Pakistan; HP Department of Medical Rehabilitation (Physiotherapy), University of Maiduguri, Maiduguri, Nigeria; HQ Department of Rehabilitation Sciences, Hong Kong Polytechnic University, Hong Kong, China; HR College of Dental Medicine, Qatar University, Doha, Qatar; HS Faculty of Health, Plymouth University, Plymouth, UK; HT Department of Pharmacy, Mohammed Al-Mana College for Medical Sciences, Dammam, Saudi Arabia; HU Department of Statistics and Operations Research, Aligarh Muslim University, Aligarh, India; HV Center for Biotechnology and Microbiology, University of Swat, Swat, Pakistan; HW Department of Geography, Sultan Qaboos University, Muscat, Oman; HX Department of Biotechnology, University of Malakand, Chakdara, Pakistan; HY Department of Biotechnology and Genetic Engineering, Hazara University Mansehra, Mansehra, Pakistan; HZ Department of Biosciences, Jamia Millia Islamia, New Delhi, India; IA School of Food and Agricultural Sciences, University of Management and Technology, Lahore, Pakistan; IB Biomedical Engineering Department, Johns Hopkins University, Baltimore, MD, USA; IC Department of Nuclear Medicine, King Hussein Cancer Center, Amman, Jordan; ID Department of Diagnostic Radiology and Nuclear Medicine, The University of Jordan, Amman, Jordan; IE Department of Pathophysiology and Transplantation, Università degli Studi di Milano (University of Milan), Milan, Italy; IF Cystic Fibrosis Center, Fondazione IRCCS Ospedale Maggiore Policlinico (IRCCS “Ca' Granda Maggiore Policlinico” Hospital Foundation), Milan, Italy; IG The School of Medicine, The University of Jordan, Amman, New South Wales (NSW), Jordan; IH Institute of Health and Wellbeing, Federation University Australia, Melbourne, VIC, Australia; II School of Public Health and Preventive Medicine, Monash University, Melbourne, VIC, Australia; IJ The Graduate School of Biomedical Engineering, University of New South Wales, Sydney, NSW, Australia; IK Department of Clinical and Community Pharmacy, An-Najah National University, Nablus, Palestine; IL Department of Biomedical Sciences, Nazarbayev University, Astana, Kazakhstan; IM Family and Community Medicine Department, Qassim University, Al Qassim, Saudi Arabia; IN School of Physics, Mathematics and Computing, University of Western Australia, Perth, WA, Australia; IO Information and Communication Technology Research Pole (Lab-STICC), ENSTA Bretagne, Brest, France; IP Department of Public Health and Community Medicine, International Medical University, Kuala Lumpur, Malaysia; IQ International Centre for Casemix and Clinical Coding, National University of Malaysia, Bandar Tun Razak, Malaysia; IR College of Life Sciences, Birmingham City University, Birmingham, UK; IS Cardiovascular Divivsion, University of Alabama, Birmingham, AL, USA; IT Tabriz University of Medical Sciences, Tabriz University of Medical Sciences, Tabriz, Iran; IU College of Medicine and Health Sciences, United Arab Emirates University, Al Ain, United Arab Emirates; IV Faculty of Medicine, Jordan University of Science and Technology, Irbid, Jordan; IW Faculty of Nursing, University of Tabuk, Tabuk, Saudi Arabia; IX Department of Cardiology, Heart, Vascular, and Thoracic Institute, Cleveland Clinic Abu Dhabi, Abu Dhabi, United Arab Emirates; IY College of Medicine and Health Sciences Academic Programs, Khalifa University, Abu Dhabi, United Arab Emirates; IZ BRAC Institute of Governance and Development (BIGD), BRAC University, Dhaka, Bangladesh; JA Department of Public and Community Health, Frontier University Garowe (FUG), Puntland, Somalia; JB Independent Consultant, Amman, Jordan; JC Department of Medicine, Nazarbayev University, Astana, Kazakhstan; JD Department of Parasitology, University of Malaya, Kuala Lumpur, Malaysia; JE Department of Parasitology, Sana'a University, Sana'a, Yemen; JF Department of Urology, Cleveland Clinic Abu Dhabi, Abu Dhabi, United Arab Emirates; JG Nuffield Department of Surgical Sciences, University of Oxford, Oxford, UK; JH Ophthalmology Department, University of Miami, Miami, FL, USA; JI university of tabuk-nursing faculty, King Saud bin Abdulaziz University for Health Sciences, tabuk, Saudi Arabia; JJ College of Pharmacy, University of Sharjah, Sharjah, United Arab Emirates; JK School of Public Health, Institute of Science and Technology, CHENNAI, India; JL Faculty of Nursing, Zarqa University, Zarqa, Jordan; JM Liver, Digestive, and Lifestyle Health Research Section, King Faisal Specialist Hospital & Research Center, Riyadh, Saudi Arabia; JN Division of Gastroenterology and Hepatology, Weill Cornell Medicine, New York, NY, USA; JO Department of Respiratory Care, Prince Sultan Military College of Health Sciences, Dammam, Saudi Arabia; JP American University of the Middle East, Egaila, Kuwait; JQ Surgical Research Section/ Surgery Department, Hamad Medical Corporation, Doha, Qatar; JR Department of Allied Medical Sciences, Jordan University of Science and Technology, Irbid, Jordan; JS Department of Nursing, Georgetown University, Washington, DC, USA; JT Macro-Fiscal Policy Department, Ministry of Finance, Dubai, United Arab Emirates; JU Department of Surgery, Kuwait University, Kuwait, Kuwait; JV Jaber Al Ahmad Al Sabah Hospital, Ministry of Health, Kuwait, Kuwait; JW Department of Emergency Medicine, Sana'a University, Sanaa, Yemen; JX Pediatric Emergency Medicine Department, Drexel University, Philadelphia, PA, USA; JY Department of Family and Community Medicine, University of Jeddah, Jeddah, Saudi Arabia; JZ Department of Basic Sciences, Yarmouk University, Irbid, Jordan; KA Institute of Molecular Biology and Biotechnology, The University of Lahore, Lahore, Pakistan; KB Faculty of Health Sciences, Equator University of Science and Technology, Uganda, Masaka, Uganda; KC Research, Policy, and Training Directorate, Jordan Center for Disease Control, Amman, Jordan; KD Applied Science Research Center, Applied Science Private University, Amman, Jordan; KE Department of Specialty Internal Medicine, Johns Hopkins Aramco Healthcare, Dhahran, Saudi Arabia; KF Department of Medicine, Indiana University School of Medicine, Indianapolis, IN, USA; KG Department of Respiratory Therapy, King Abdulaziz University, Jeddah, Saudi Arabia; KH Respiratory Therapy Unit, King Abdulaziz University, Jeddah, Saudi Arabia; KI University of Sharjah, Sharjah, United Arab Emirates; KJ Faculty of Health Sciences, University of Cadiz, Cadiz, Spain; KK Research Group in Health Economics, Universidad de Cartagena (University of Cartagena), Cartagena, Colombia; KL Research Group in Hospital Management and Health Policies, Universidad de la Costa (University of the Coast), Barranquilla, Colombia; KM Department of Rehabilitation Sciences, Jordan University of Science and Technology, Irbid, Jordan; KN Department of Medical Sciences, Azal University for Human Development, Sana'a, Yemen; KO Department of Clinical Sciences, University of Science and Technology of Fujairah, Fujairah, United Arab Emirates; KP Department of Pediatrics, Cleveland Clinic, Cleveland, OH, USA; KQ Department of Pharmacy Practice and Pharmacotherapeutics, University of Sharjah, Sharjah, United Arab Emirates; KR Department of Clinical Pharmacy, Jordan University of Science and Technology, Irbid, Jordan; KS Maternal and Child Health Division (MCHD), International Centre for Diarrhoeal Disease Research, Bangladesh, Dhaka, Bangladesh; KT Evaluation Unit, Global Alliance for Vaccines and Immunisations, Geneva, Switzerland; KU Global Health Advocacy Incubator (GHAI), University of Central Nicaragua, Washington, DC, USA; KV London School of Hygiene and Tropical Medicine, University of London, London, UK; KW Food and Beverages Safety Research Center, Urmia University of Medical Sciences, Urmia, Iran; KX Department of Pharmacy, An-Najah National University, Nablus, Palestine; KY Student Research Committee, Lorestan University of Medical Sciences, Khorramabad, Iran; KZ Health Policy Research Center, Shiraz University of Medical Sciences, Shiraz, Iran; LA Public Health and Community Medicine Department, Cairo University, Cairo, Egypt; LB College of Medicine, University of Sharjah, Sharjah, United Arab Emirates; LC Department of Radiology and Radiological Science, University of Maryland, Baltimore, MD, USA; LD Department of Health and Management Sciences, Khomein University of Medical Sciences, Khomein, Iran; LE Gastrointestinal and Liver Diseases Research Center, Guilan University of Medical Sciences, Rasht, Iran; LF Department of Pharmaceutics and Pharmaceutical Technology, Usmanu Danfodiyo University, Sokoto, Sokoto, Nigeria; LG School of Pharmacy, University of Botswana, Gaborone, Botswana; LH Department of Food Safety and Hygiene, Zanjan University of Medical Sciences, Zanjan, Iran; LI Spiritual Health Research Center, Baqiyatallah University of Medical Sciences, Tehran, Iran; LJ Universal Scientific Education and Research Network (USERN), Tehran University of Medical Sciences, Tehran, Iran; LK Department of Health and Wellbeing, African Population and Health Research Center, Nairobi, Kenya; LL Department of Medicine, University of Jos, Jos, Nigeria; LM Department of Internal Medicine, Jos University Teaching Hospital, Jos, Nigeria; LN Center for Biomedical Image Computing & Analytics, University of Pennsylvania, Philadelphia, PA, USA; LO University of Bologna, Bologna, Italy; LP Department of General Medicine, Eastern Health, Box Hill, VIC, Australia; LQ Orthopedic Department, Tehran University of Medical Sciences, Tehran, Iran; LR Faculty of Pharmacy, Carol Davila University of Medicine and Pharmacy, Bucharest, Romania; LS Centre for Sensorimotor Performance, The University of Queensland, Brisbane, QLD, Australia; LT Neurology Department, Royal Brisbane and Women's Hospital, Brisbane, QLD, Australia; LU Faculty of Medicine and Health, University of Sydney, Sydney, NSW, Australia; LV Sydney Musculoskeletal Health, University of Sydney, Sydney, NSW, Australia; LW Department of Statistics and Econometrics, Bucharest University of Economic Studies, Bucharest, Romania; LX Department of Internal Medicine, Rutgers University, Toms River, NJ, USA; LY Sarver Heart Center, University of Arizona, Tucson, AZ, USA; LZ Department of General Medicine, Thai Binh University of Medicine and Pharmacy in Vietnam, Thai Binh City, Viet Nam; MA Department of Management, University of Cape Coast, Cape Coast, Ghana; MB Department of Public Health, The Apollo University, Chittoor, India; MC Digestive Diseases Research Institute, Tehran University of Medical Sciences, Tehran, Iran; MD Department of Physiotherapy, Galgotias University, Greater Noida, India; ME Pharmacy Department, Critical Care, Cleveland Clinic Abu Dhabi?, Abu Dhabi?, United Arab Emirates; MF Department of Epidemiology and Biostatistics, Zahedan University of Medical Sciences, Zahedan, Iran; MG Agribusiness Study Program, Sebelas Maret University, Surakarta, Indonesia; MH Department of Environmental and Occupational Health, University of Medical Sciences, Ondo, Ondo, Nigeria; MI Department of Microbiology, University of Medical Sciences, Ondo, Ondo, Nigeria; MJ Centre for Interdisciplinary Research in Basic Sciences (CIRBSc), Jamia Millia Islamia, New Delhi, India; MK School of Chemical and Life Sciences (SCLS), Jamia Hamdard, New Delhi, India; ML Department of Surgery, Gadjah Mada University, Yogyakarta, Indonesia; MM Department of Pathology, Imam Mohammad Ibn Saud Islamic University, Riyadh, Saudi Arabia; MN Rural Health Research Institute, Charles Sturt University, Orange, NSW, Australia; MO School of Medicine and Public Health, Ateneo De Manila University, Pasig City, Philippines; MP Inter-Agency Committee on Environmental Health, Department of Health Philippines, Manila, Philippines; MQ Division of Gastroenterology, Hepatology, and Nutrition, Virginia Commonwealth University, Richmond, VA, USA; MR Gastroenterology Department, Pontifical Catholic University of Chile, Santiago, Chile; MS Geneva University Hospital, University of Geneva, Geneva, Switzerland; MT Health Management and Economics Research Center, Iran University of Medical Sciences, Tehran, Iran; MU College of Pharmacy, Al Ain University, Abu Dhabi, United Arab Emirates; MV College of Art and Science, Ottawa University, Surprise, AZ, USA; MW School of Life Sciences, Arizona State University, Tempe, AZ, USA; MX Department of Veterinary Pharmacology and Toxicology, University of Ilorin, Ilorin, Nigeria; MY Care in Long Term Conditions Research Division, King's College London, London, UK; MZ CIBER Epidemiology and Public Health (CIBERESP), Madrid, Spain; NA School of Medicine, Shahid Beheshti University of Medical Sciences, Tehran, Iran; NB Department of Cardiovascular, Endocrine-Metabolic Diseases and Aging, Istituto Superiore di Sanità (ISS), Rome, Italy; NC Department of Neurobiology, Care Sciences and Society, Karolinska Institutet, Stockholm, Sweden; ND School of Health and Social Studies, Dalarna University, Falun, Sweden; NE Department of Biotechnology, Sri Ramaswamy Memorial Institute of Science and Technology, Kattankulathur, India; NF University College of Medicine & Dentistry, The University of Lahore, Lahore, Pakistan; NG Institute for Biomedical Problems, Russian Academy of Sciences, Moscow, Russia; NH Department of Periodontics, Saveetha University, Chennai, India; NI Department of Public Health, Kazakh National Medical University, Almaty, Kazakhstan; NJ Department of Clinical Disciplines, Al Farabi Kazakh National University, Almaty, Kazakhstan; NK College of Medicine, University of Arizona, Tucson, AZ, USA; NL Basic Health Sciences Institute, Shahrekord University of Medical Sciences, Shahrekord, Iran; NM Department of Pharmacy Practice, AlMaarefa University, Riyadh, Saudi Arabia; NN Research Institute of Dental Sciences, Shahid Beheshti University of Medical Sciences, Tehran, Iran; NO National Agency for Strategic Research in Medical Education (NASRME), Ministry of Health and Medical Education, Tehran, Iran; NP Cabrini Research, Cabrini Health, Malvern, VIC, Australia; NQ Pioneer Journal of Biostatistics and Medical Research (PJBMR), Pakistan, Pakistan; NR Department of Physiology, Faisalabad Medical University, Faisalabad, Pakistan; NS College of Applied Medical Science, University of Hail, Hail, Saudi Arabia; NT School of Medicine, Zanjan University of Medical Sciences, Zanjan, Iran; NU Department of Radiation Oncology, Shandong University, Shandong, China; NV Deakin Health Economics/School of Health and Social Development, Deakin University, Melbourne, Victoria (VIC), Australia; NW School of Traditional Chinese Medicine, Xiamen University Malaysia, Sepang, Malaysia; NX Faculty of Medicine, Nursing, and Health Sciences, Monash University, Melbourne, Victoria (VIC), Australia; NY Nursing Department, Institute of Technology and Health Science RS dr Soepraoen, Malang, Indonesia; NZ Faculty of Health Science, Institute of Technology and Health Science RS dr Soepraoen, Malang, Indonesia; OA Department of Medical-Surgical Nursing, Manipal Academy of Higher Education, Udupi, India; OB Monash Addiction Research Center, Monash University, Melbourne, Victoria (VIC), Australia; OC Atchabar Scientific Research Institute, Kazakh National Medical University, Almaty, Kazakhstan; OD School of Architecture, Design, and Planning, University of Sydney, Sydney, NSW, Australia; OE Keck School of Medicine, University of Southern California, Los Angeles, CA, USA; OF Faculty of Nursing, Philadelphia University, Amman, Jordan; OG Department of Forensic Medicine, Lumbini Medical College, Palpa, Nepal; OH Management Policy and Community Health, University of Texas, Houston, TX, USA; OI College of Medicine, University of Basrah, Basrah, Iraq; OJ School of Business, University of Leicester, Leicester, UK; OK Robarts Research Institute, The University of Western Ontario, London, ON, Canada; OL Departament of Physiotherapy, Federal University of Santa Catarina, Araranguá, Brazil; OM IMBB, The University of Lahore, lahore, Pakistan; ON School of Veterinary Medicine, Texas Tech University, Amarillo, TX, USA; OO School of Nursing and Public Health, University of KwaZulu-Natal, Durban, South Africa; OP The Judith Lumley Centre, La Trobe University, Melbourne, VIC, Australia; OQ Universidad de San Martin de Porres, Lima, Peru; OR Bloomberg School of Public Health, Johns Hopkins University, Baltimore, MD, USA; OS Department of Public Health, Wollega University, Nekemte, Ethiopia; OT Department of Health Behavior and Society, Jimma University, Jimma, Ethiopia; OU Department of Health Information Management, Iran University of Medical Sciences, Tehran, Iran; OV Psychiatry Department, University of Ibadan, Ibadan, Nigeria; OW Medicinal Chemistry Unit, Kwara State University, Malete, Ilorin, Nigeria; OX Centre for Drug Research, Universiti Sains Malaysia, Pinang, Malaysia; OY Department of Public Health, Hacettepe University, ankara, Turkiye; OZ Department of Nephrology, Etlik City Hospital, Ankara, Turkiye; PA Department of Health Information Management, Tehran University of Medical Sciences, Tehran, Iran; PB Research and Technology Deputy, Kurdistan University of Medical Sciences, Sanandaj, Iran; PC Department of Infectious Disease Epidemiology, London School of Hygiene & Tropical Medicine, London, UK; PD Department of Applied Mathematics, Stellenbosch University, Stellenbosch, South Africa; PE Department of Nursing, Shiraz University of Medical Sciences, Shiraz, Iran; PF Consultant, Washington, DC, USA; PG Department of Psychiatry, University of Social Welfare and Rehabilitation Sciences, tehran, Iran; PH Department of Anesthesia, Cihan University, Sulaymaniyah, Iraq; PI Department of Basic Sciences, University of Sulaimani, Sulaymaniyah, Iraq; PJ Advanced Medical & Dental Institute, Universiti Sains Malaysia, Penang, Malaysia; PK Rheumatology Research Center, Tehran University of Medical Sciences, Tehran, Iran; PL Tehran University of Medical Sciences, Tehran, Iran; PM ASIDE Healthcare, Lewes, DE, USA; PN Faculty of Medicine, October 6 University, 6th of October City, Egypt; PO Geriatric Unit, Fondazione IRCCS Ca' Granda Ospedale Maggiore Policlinico, Milan, Italy; PP Department of Medical Biochemical Analysis, Cihan University, Erbil, Iraq; PQ Department of Physiotherapy, Manipal Academy of Higher Education, Manipal, India; PR Department of Population Medicine, Qatar University, Doha, Qatar; PS Department of Forensic Science, Government Institute of Forensic Science Nagpur, Nagpur, India; PT Rashtrasant Tukadoji Maharaj Nagpur University, Nagpur, India; PU The Malaria Atlas Project, Telethon Kids Institute, Perth, WA, Australia; PV Shahid Rajii Hospital, Shahid Beheshti University of Medical Sciences, Tehran, Iran; PW Dental Material Research Center, Islamic Azad University, tehran, Iran; PX Pediatric Dentistry Department, King Abdulaziz University, Jeddah, Saudi Arabia; PY Department of Medicine, King Saud University, Riyadh, Saudi Arabia; PZ Academic Affairs and Prince Naif Health Research Center, King Khalid University Hospital, Riyadh, Saudi Arabia; QA School of Public Health, Tehran University of Medical Sciences, Tehran, Iran; QB College of Optometry, Pacific University, Forest Grove, OR, USA; QC Department of Community Medicine, All India Institute of Medical Sciences, Nagpur, India; QD Clinical Research Center, Nanjing Children's Hospital, Nanjing, China; QE International Medical School, Management and Science University, Alam, Malaysia; QF Robert N Butler Aging Center, Columbia University Medical Center, New York, NY, USA; QG Population Research Centre, Institute for Social and Economic Change, Bengaluru, India; QH Lerner College of Medicine, Cleveland Clinic, Cleveland, OH, USA; QI Chen Senior Medical Center, Tamarac, FL, USA; QJ Anahuac Business School, Universidad Anahuac Mexico, Mexico City, Mexico; QK Department of Epidemiology and Biostatistics, Kerman University of Medical Sciences, Kerman, Iran; QL College of Medicine, Alfaisal University, Riyadh, Saudi Arabia; QM Center of Innovation, Technology and Education (CITE), Anhembi Morumbi University, São José dos Campos, Brazil; QN Department of Neurosurgery, University of Southampton, Southampton, UK; QO Department of Non-communicable Diseases, Bangladesh University of Health Sciences, Dhaka, Bangladesh; QP Department of Health Policy, Mario Negri Institute for Pharmacological Research, Milano, Italy; QQ Scientific Advisory Board, Institut d'Investigació Biomèdica de Girona Dr. Josep Trueta, Girona, Spain; QR School of Psychology, University of Auckland, Auckland, New Zealand; QS Department of Public and Environmental Health, University of The Gambia, Banjul, The Gambia; QT Department of Epidemiology, University of Florida, Gainesville, FL, USA; QU Heidelberg Institute of Global Health (HIGH), Heidelberg University Hospital, Heidelberg, Germany; QV Alpha Genomics Private Limited, Islamabad, Pakistan; QW University Institute of Food Science and Technology, The University of Lahore, Lahore, Pakistan; QX Health Information Management, Shiraz University of Medical Sciences, Shiraz, Iran; QY Department of General Surgery and Medical-Surgical Specialties, University of Catania, Catania, Italy; QZ Department of Community Medicine, Sri Manakula Vinayagar Medical College and Hospital, Puducherry, India; RA Non-communicable Diseases Research Center, Tehran University of Medical Sciences, Tehran, Iran; RB School of Medicine, Iran University of Medical Sciences, Tehran, Iran; RC Faculty of Nursing, King Khalid University, Mahyil Asir, Saudi Arabia; RD Department of Medical Education, University of Nevada Las Vegas, Las Vegas, NV, USA; RE Department of Psychiatry, University of Münster, Münster, Germany; RF Department of Psychiatry, Melbourne Medical School, Melbourne, VIC, Australia; RG Cancer Research Center, Shahid Beheshti University of Medical Sciences, Tehran, Iran; RH Pastor Institute, Tehran University of Medical Sciences, Tehran, Iran; RI Biological Science Division, University of Chicago, Chicago, IL, USA; RJ Health Human Resources Research Center, Shiraz University of Medical Sciences, Shiraz, Iran; RK Department Nutrition and Dietetics, Bahir Dar University, Bahir Dar, Ethiopia; RL The George Institute for Global Health, Imperial College London, London, UK; RM School of Public Health, Dr. D. Y. Patil University, Mumbai, India; RN Jazan University, Jazan, Saudi Arabia; RO Department of Human Anatomy and Histology, I.M. Sechenov First Moscow State Medical University, Moscow, Russia; RP Avicenna Biotech Research, Germantown, MD, USA; RQ Department of Regulatory Affairs, Amarex Clinical Research, Germantown, MD, USA; RR Department of Microbiology, Islamic Azad University, Shahr-e-Qods, Iran; RS Transplant and Hepatobiliary Surgery Service, Hospital Universitario Fundación Santa Fe de Bogotá (University Hospital Santa Fe Foundation of Bogotá), Bogota, Colombia; RT Subdirectorate of Clinical Studies and Clinical Epidemiology, Hospital Universitorio Fundación Santa Fe de Bogotá, Bogotá, Colombia; RU Milken Institute of Public Health, George Washington University, Washington, DC, USA; RV College of Medicine and Health Science, Bahir Dar University, Bahir Dar, Ethiopia; RW Department of Public Health, Bahir Dar University, Bahir Dar, Ethiopia; RX Department of Public Health, University of South Africa, Pretoria, South Africa; RY Department of Emergency and Critical Care Nursing, Bahir Dar University, Bahir Dar, Ethiopia; RZ Department of Radiology, Mayo Clinic, Rochester, MN, USA; SA Bayero University Kano, Higher National School of Veterinary Medicine, Kano, Nigeria; SB North-West University, Mafikeng, South Africa; SC Infectious Disease Research Department, King Abdullah International Medical Research Center, Riyadh, Saudi Arabia; SD Department of Veterinary Microbiology, Usmanu Danfodiyo University, Sokoto, Sokoto, Nigeria; SE Department of Physiotherapy and Paramedicine, Glasgow Caledonian University, Glasgow, UK; SF Department of Biological Sciences, University of Porto, Porto, Portugal; SG Research Unit on Applied Molecular Biosciences (UCIBIO), University of Porto, Porto, Portugal; SH Department of Biomedical Sciences, University of West Attica, Athens, Greece; SI National AIDS Reference Center of Southern Greece, University of West Attica, Athens, Greece; SJ Center of Excellence of Cancer Research, University of Sharjah, Sharjah, United Arab Emirates; SK BRAC James P Grant School of Public Health, BRAC University, Dhaka, Bangladesh; SL Department of Epidemiology and Health Promotion, New York University, New York, NY, USA; SM Dipartimento di Scienze Mediche e Chirurgiche, University of Bologna, Bologna, Italy; SN Department of Nursing, Bahir Dar University, Bahir Dar, Ethiopia; SO School of Public Health, Johns Hopkins University, Baltimore, MD, USA; SP Department of Epidemiology and Biostatistics, University of the Philippines Manila, Manila, Philippines; SQ Department of Epidemiology, Brown University, Providence, RI, USA; SR Hubert Department of Global Health, Emory University, Atlanta, GA, USA; SS Department of Global Health, George Washington University, Washington, DC, USA; ST Department of Community Medicine and Family Medicine, All India Institute of Medical Sciences, Rishikesh, India; SU Community Health Department, University of South Wales, South Wales, UK; SV Department of Public Health, North Dakota State University, Fargo, ND, USA; SW Department of General Practice and Emergency Medicine, Karnali Academy of Health Sciences, Jumla, Nepal; SX Internal Medicine, Saint Vincent Hospital, Worcester, Worcester, MA, USA; SY Department of Community Medicine and Family Medicine, All India Institute of Medical Sciences, Jodhpur, India; SZ School of Public Health, All India Institute of Medical Sciences, Jodhpur, India; TA Department of Anatomy, All India Institute of Medical Sciences, Jodhpur, India; TB Department of Internal Medicine, Wayne State University, Gross Pointe Woods, MI, USA; TC Global Health Neurology Lab, NSW Brain Clot Bank, Sydney, NSW, Australia; TD Division of Cerebrovascular Medicine and Neurology, National Cerebral and Cardiovascular Center, Suita, Japan; TE Department of Family Medicine, Texas Tech University, el paso, TX, USA; TF School of Sport & Health Sciences, University of Brighton, Brighton, UK; TG Department of Public Health Research, Bengal Rural Welfare Service (BRWS), Kolkata, West Bengal, India; TH Translational and Clinical Research Institute, Newcastle University, Newcastle upon Tyne, UK; TI Department of Botanical and Environmental Sciences, Guru Nanak Dev University, Amritsar, India; TJ Department of Pharmaceutical Sciences, Guru Nanak Dev University, Amritsar, India; TK Department of Medical Lab Technology, Chandigarh University, Punjab, India; TL Laboratory of Translational Medicine and Nanotherapeutics, Central University of Punjab, Bathinda, India; TM Department of Health Administration, Rutgers University, New Brunswick, NJ, USA; TN Independent Consultant, Addis Ababa, Ethiopia; TO Fondazione Banca Degli Occhi Del Veneto, Carol Davila University of Medicine and Pharmacy, Venice, Italy; TP TAF Uludag Winter Training Center, Turkish Ministry of Defence, Bursa, Turkiye; TQ Medical Laboratory Sciences, University of Hail, Hail, Saudi Arabia; TR Charles Perkins Centre, University of Sydney, Sydney, NSW, Australia; TS Clinical Research Centre, Sydney Local Health District, Sydney, NSW, Australia; TT Department of Biochemistry and Biotechnology, University of Science and Technology Chittagong, Chittagong, Bangladesh; TU Department of Community Medicine and Family Medicine, All India Institute of Medical Sciences, Deoghar, India; TV Department of Clinical Pharmacy, Universiti Sultan Zainal Abidin, Besut, Malaysia; TW Health Biotechnology Directorate at Bio and Emerging Technology Institute, Addis Ababa University, Addis Ababa, Ethiopia; TX Department of Neurobiology, Care Sciences and Society, Karolinska Institute, Stockholm, Sweden; TY Department of Physical Education and Health, Universidad de la República, Rivera, Uruguay; TZ School of Business Administration, American University of Sharjah, Sharjah, United Arab Emirates; UA Department of Nutrition and Dietetics, Ankara University, Ankara, Turkiye; UB Faculty of Psychology, Education, and Sport, University Lusofona, Porto, Portugal; UC Research Centre for Physical Activity, Health, and Leisure, University of Porto, Porto, Portugal; UD Department of Demography and Population Studies, University of Witwatersrand, Johannesburg, South Africa; UE Department of Internal Medicine, Manipal Academy of Higher Education, Mangalore, India; UF Ophthalmology Department, Isfahan University of Medical Sciences, Isfahan, Iran; UG Department of Anesthesia and Critical Care Medicine, Johns Hopkins University, Baltimore, MD, USA; UH General Directorate of Health Information Systems, Ministry of Health, Ankara, Turkiye; UI Internal Medicine Department, Shahid Beheshti University of Medical Sciences, Tehran, Iran; UJ Pediatric Infectious Diseases and Immunology, Pontificia Universidad Católica de Chile (Pontifical Catholic University of Chile), Santiago, Chile; UK Facultad de Salud (Faculty of Health), Universidad Santiago de Cali, Cali, Colombia; UL Department of Medicine, University Ferhat Abbas of Setif, Sétif, Algeria; UM Department of Epidemiology and Preventive Medicine, University Hospital Saadna Abdenour, Sétif, Algeria; UN Transport and Road Safety (TARS) Research Centre, University of New South Wales, Sydney, NSW, Australia; UO Vision and Eye Research Institute, Anglia Ruskin University, Cambridge, UK; UP Department of Earth, Environment, and Equity, Howard University, Washington, DC, USA; UQ University of Genoa, Genoa, Italy; UR Cancer Population Sciences Program, University of Florida Health Cancer Center, Gainesville, FL, USA; US Institute for Medical Information Processing, Biometry, and Epidemiology, LMU Munich, Neuherberg, Germany; UT Institute of Epidemiology, Helmholtz Zentrum München (German Research Center for Environmental Health), Neuherberg, Germany; UU Division of Clinical Epidemiology and Aging Research, German Cancer Research Center, Heidelberg, Germany; UV Center for Neuroscience, Institute for Scientific Research and High Technology Services, Panama City, Panama; UW Gorgas Memorial Institute for Health Studies, Panama City, Panama; UX Department of Injury, The George Institute for Global Health, Newtown, NSW, Australia; UY Faculty of Medicine, University of New South Wales, Kensington, NSW, Australia; UZ Malaria Atlas Project, Telethon Kids Institute, Nedlands, WA, Australia; VA Department of Medical and Surgical Sciences, University of Bologna, Bologna, Italy; VB College of Health Sciences, VinUniversity, Hanoi, Viet Nam; VC Research Advancement Consortium in Health, Hanoi, Viet Nam; VD Department of Environmental Health, Bahir Dar University, Bahir Dar, Ethiopia; VE National Centre for Epidemiology and Population Health, Australian National University, Canberra, ACT, Australia; VF School of Medicine and Health, Technical University of Munich, Munich, Germany; VG Department of Radiology, University of Cambridge, Cambridge, UK; VH Department of Health Care Management, Technische Universität Berlin, Berlin, Germany; VI Department of Basic Biomedical Sciences, University of Sharjah, Sharjah, United Arab Emirates; VJ School of Public Health Sciences, University of Waterloo, Waterloo, ON, Canada; VK Al Shifa School of Public Health, Al Shifa Trust Eye Hospital, Rawalpindi, Pakistan; VL Department of Family and Community Medicine, King Abdulaziz University, Jeddah, Saudi Arabia; VM School of Public Health and Administration, Peruvian University Cayetano Heredia, Lima, Peru; VN Department of Sociology, University of Macau, Macau, China; VO Faculty of Medicine and Health, University of Sydney, Sydney, New South Wales (NSW), Australia; VP The Children's Hospital at Westmead, New South Wales Poisons Information Centre, Sydney, New South Wales (NSW), Australia; VQ Department of Clinical Pharmacy, University of Medicine and Pharmacy of Craiova, Craiova, Romania; VR Department of Internal and Geriatric Medicine, Hospital Italiano de Buenos Aires (Italian Hospital of Buenos Aires), Buenos Aires, Argentina; VS Board of Directors, Argentine Society of Medicine, Buenos Aires, Argentina; VT Center of Innovation, Technology and Education (CITE), Anhembi Morumbi University, Sao Jose dos Campos, Brazil; VU Center for Nutrition and Health Research, National Institute of Public Health, Cuernavaca, Mexico; VV Department of Anesthesiology, Third Xiangya Hospital of Central South University, Changsha, China; VW Department of Surgery, Chinese Academy of Medical Sciences, Beijing, China; VX Dana-Farber Cancer Institute, Harvard University, Boston, MA, USA; VY Unit of Hygiene and Public Health, Romagna Local Health Authority, Forlì-Cesena, Italy; VZ Interdisciplinary Research Center for Health Science, Sant'Anna School of Advanced Studies, Pisa, Italy; WA Institute for Cancer Research, Prevention and Clinical Network, Florence, Italy; WB Department of Medicine and Surgery, University of Insubria, Varese, Italy; WC Faculty of Health Sciences, University Fernando Pessoa, Porto, Portugal; WD Associated Laboratory for Green Chemistry (LAQV), University of Porto, Porto, Portugal; WE IMPInstitute for Mental and Physical Health and Clinical Translation (IMPACT), Deakin University, Geelong, VIC, Australia; WF School of Health Science, University of Sydney, Sydney, NSW, Australia; WG Education Center of Australia, Health Science College, Sydney, NSW, Australia; WH Department of Psychiatry, University of Sao Paulo, São Paulo, Brazil; WI Public Health Department, National University of Colombia, Bogota, Colombia; WJ Epidemiology and Public Health Evaluation Group, National University of Colombia, Bogota, Colombia; WK Division of Country Health Policies and Systems (CPS), World Health Organisation, Italy; WL Mental Health Flagship, World Health Organization (WHO), Copenhagen, Denmark; WM Institute of Public Goods and Policies (IPP), Spanish National Research Council, Madrid, Spain; WN Centre for Biomedical Research in Mental Health Network (CIBERSAM), Institute of Health Carlos III, Madrid, Spain; WO Department of Pharmacological and Biomolecular Sciences, University of Milan, Milan, Italy; WP MultiMedica Sesto San Giovanni IRCCS, Sesto San Giovanni, Italy; WQ Department of Public Health and Infectious Diseases, La Sapienza University, Rome, Italy; WR Department of Medical, Surgical, and Health Sciences, University of Trieste, Trieste, Italy; WS Public Health Unit, University Health Agency Giuliano-Isontina (ASUGI), Trieste, Italy; WT Department of Nutrition, Federal University of Santa Catarina, Florianópolis, Brazil; WU College of Public Health, Medical, and Veterinary Sciences, James Cook University, Townsville, QLD, Australia; WV Department of Public Health, University of Mataram, Mataram, Indonesia; WW Mary MacKillop Institute for Health Research, Australian Catholic University, Melbourne, VIC, Australia; WX School of Public Health, University of Hong Kong, Hong Kong, China; WY Posgrado de Medicina, Facultad de Ciencias de la Salud, Universidad Cientifica del Sur, Lima, Peru; WZ Department of Biotechnology, Adamas University, Kolkata, India; XA Institute for Skeletal Aging & Orthopedic Surgery, Hallym University, Chuncheon, South Korea; XB State Disease Investigation Laboratory, Animal Resources Development Department, Agartala, India; XC Cardio-Oncology Research Unit, Cardiovascular Analytics Group, Canterbury, UK; XD School of Nursing and Health Studies, Hong Kong Metropolitan University, Hong Kong, China; XE Department of Applied Health Sciences, University of Birmingham, Birmingham, UK; XF Clinical Nutrition Department, Jazan University, Jazan, Saudi Arabia; XG Department of Psychiatry, University of Kelaniya, Ragama, Sri Lanka; XH University Psychiatry Unit, Colombo North Teaching Hospital, Ragama, Sri Lanka; XI College of Medicine, National Taiwan University, Taipei, Taiwan; XJ Department of Nursing, National Taiwan University Hospital, Taipei, Taiwan; XK Department of Epidemiology and Biostatistics, Semey Medical University (SMU), Semey, Kazakhstan; XL Department of Community Medicine, Datta Meghe Institute of Medical Sciences, Sawangi, India; XM Department of Endocrinology, University of Manchester, Manchester, UK; XN Department of Endocrinology, Christie Hospital NHS Foundation Trust, Manchester, UK; XO Department of Public Health, Indian Institute of Public Health, Hyderabad, India; XP Department of Oral Medicine and Radiology, King George's Medical University, Lucknow, India; XQ EPI, Oromia Health Bureau, Addis Ababa, Ethiopia; XR Peking Union Medical College Hospital, Chinese Academy of Medical Sciences & Peking Union Medical College, Beijing, China; XS Science and Technology Department, Northern Jiangsu People's Hospital, yangzhou, China; XT Clinical Research Center, Zhujiang Hospital of Southern Medical University, Guangzhou, China; XU Department of Computer, Electrical and Mathematical Sciences and Engineering, King Abdullah University of Science and Technology, Thuwal, Saudi Arabia; XV Faculty of Humanities and Health Sciences, Curtin University, Miri, Malaysia; XW School of Dentistry, University of Michigan, Ann Arbor, MI, USA; XX School of Chinese Medicine (Teaching and Research Division), Hong Kong Baptist University, Hong Kong, China; XY Yong Loo Lin School of Medicine, National University of Singapore, Singapore, Singapore; XZ Department of Scientific Research, Tehran University of Medical Sciences, Tehran, Iran; YA Department of Laboratory Medicine, Taichung Tzu-Chi Hospital Buddhist Tzu-Chi Medical Foundation, Tanzih, Taiwan; YB Department of Medical Laboratory Science and Biotechnology, Central Taiwan University of Science and Technology, Taiwan; YC Department of Public Health and Health Policy, Hiroshima University, Hiroshima, Japan; YD Department of Clinical Oncology, Queen Elizabeth Hospital, Hong Kong, China; YE Division of Plastic Surgery, University of Wisconsin, Madison, WI, USA; YF Department of Medicine, National University of Singapore, Singapore, Singapore; YG Centre for Research Impact & Outcome, Chitkara University, Rajpura, India; YH Department of Biosciences, Saveetha University, Chennai, India; YI Department of Community Medicine, Jawaharlal Nehru Medical College, Wardha, India; YJ Southeast University, Southeast University, Dhaka, Bangladesh; YK Department of Public Health, Asian University for Women, Chittagong, Bangladesh; YL Robert Stemple College of Public Health and Social Work, Florida International University, Miami, FL, USA; YM The Interdisciplinary Research Group on Biomedicine and Health, VNU International School (VNUIS), Hanoi, Viet Nam; YN Faculty of Applied Sciences, VNU International School (VNUIS), Hanoi, Viet Nam; YO Department of Pediatrics, Peking University, Beijing, China; YP Department of Paediatric Surgery, Federal Medical Centre, Umuahia, Nigeria; YQ Faculty of Social Sciences, Federal University Oye-Ekiti Nigeria, Oye-Ekiti, Nigeria; YR Department of Urology, The University of Queensland, Brisbane, QLD, Australia; YS Department of AndroUrology, AndroUrology Centre, Brisbane, QLD, Australia; YT Department of Health Informatics, University College London, London, UK; YU Health Data Research UK, London, UK; YV Department of Pediatrics, University of Washington, Seattle, WA, USA; YW Department of Health Behavior, Texas A&M University, College Station, TX, USA; YX Department of Biostatistics, Johns Hopkins University, Baltimore, MD, USA; YY Nova Medical School, Nova University of Lisbon, Lisbon, Portugal; YZ Department of Cardiovascular Sciences, Katholieke Universiteit Leuven, Leuven, Belgium; ZA School of Psychology, University of Southampton, Southampton, UK; ZB Department of Child and Adolescent Psychiatry, New York University, New York, NY, USA; ZC Research Center on Public Health (CESP), University of Milan Bicocca, Monza, Italy; ZD Laboratory of Public Health, Instituto Auxologico Italiano IRCCS (Italian Auxological Institute), Milan, Italy; ZE Department of Health Sciences, University of Florence, Florence, Italy; ZF Department of Family Medicine and Public Health, University of California San Diego, La Jolla, CA, USA; ZG Life and Health Sciences Research Institute (ICVS), University of Minho, Braga, Portugal; ZH Institute for Research and Innovation in Health (i3S), University of Porto, Porto, Portugal; ZI Research Department, Cleveland Clinic Abu Dhabi, Abu Dhabi, United Arab Emirates; ZJ Faculty of Medicine, University of Aleppo, Aleppo, Syria; ZK Department of Anesthsia, Critical Care and Pain Medicine, Shahid Beheshti University of Medical Sciences, Tehran, Iran; ZL Research Center for Child Psychiatry, University of Turku, Turku, Finland; ZM Iranian Research Center for HIV/AIDS (IRCHA), Tehran University of Medical Sciences, Tehran, Iran; ZN Departartment of Allied health sciences, Federal University of Health Science Azare, Katagum-Azare, Nigeria; ZO Department of Community Medicine, Ahmadu Bello University, Zaria, Nigeria; ZP Department of Public Health and Primary Care, University of Cambridge, Cambridge, UK; ZQ Cardiovascular Metabolic Translational Research Program, National University of Singapore, Singapore, Singapore; ZR Institute for Health Sciences, Mid Sweden University, Sundsvall, Sweden; ZS Nutrition Department, Harvard University, Boston, MA, USA; ZT Department of Medical and Surgical Sciences and Advanced Technologies “GF Ingrassia”, University of Catania, Catania, Italy; ZU Public Health Foundation of India, Gurugram, India; ZV Department of Brain Sciences, Imperial College London, London, UK; ZW Department of Internal Medicine, Texas Tech University, Lubbock, TX, USA; ZX Ga East Municipal Hospital, Ghana Health Service, Accra, Ghana; ZY Department of Public Health, Haramaya University, Harar, Ethiopia; ZZ The University of Jordan, The University of Jordan, Amman, Jordan; AAA School of Public Health, University of the Witwatersrand, Johannesburg, South Africa; AAB Division of Women and Child Health, Aga Khan University, Karachi, Pakistan; AAC Health Policy, Mario Negri Institute for Pharmacological Research, Milan, Italy; AAD Department of Population and Development, Latin American Faculty of Social Sciences Mexico, Mexico City, Mexico; AAE Atchabarov Scientific-Research Institute of Fundamental and Applied Medicine, Kazakh National Medical University, Almaty, Kazakhstan; AAF Population Health Research Center, Kazakh National Medical University, Almaty, Kazakhstan; AAG Department of Public Health, National University of Colombia, Bogota, Colombia; AAH Department of Legal Medicine, Psychiatry and Pathology, Universidad Complutense de Madrid (Complutense University of Madrid), Madrid, Spain; AAI Memorial Sloan Kettering Cancer Center, Memorial Sloan Kettering Cancer Center, New York, NY, USA; AAJ Department of Pediatrics, Brookdale University Hospital Medical Center, Brooklyn, NY, USA; AAK National Drug and Alcohol Research Centre, University of New South Wales, Sydney, NSW, Australia; AAL Department of Biostatistics and Epidemiology, Kerman University of Medical Sciences, Kerman, Iran; AAM Department of Implementation Research, Bernhard Nocht Institute for Tropical medicine, Hamburg, Germany; AAN Department of Neurosurgery, Tehran University of Medical Sciences, Tehran, Iran; AAO Ophthalmology Department, University of Tennessee, Memphis, TN, USA; AAP Department of Physiology, Bahir Dar University, Bahir Dar, Ethiopia; AAQ Department of Neurosurgery, University of Edinburgh, Edinburgh, UK; AAR Department of Neurosurgery, National Health Service (NHS) Scotland, Edinburgh, UK; AAS Dirección de Nutrición, Salvador Zubiran National Institute of Medical Sciences and Nutrition, Mexico City, Mexico; AAT Department of Biological Sciences, University of Manouba, Manouba, Tunisia; AAU Department of Social Sciences, University of Jendouba, El Kef, Tunisia; AAV Wellcome Trust Brighton and Sussex Centre for Global Health Research, Brighton and Sussex Medical School, Brighton, UK; AAW School of Public Health, Addis Ababa University, Addis Ababa, Ethiopia; AAX Department of Nutrition and Dietetics, Bahir Dar University, Bahir Dar, Ethiopia; AAY Department of Forensic Medicine, University of Sarajevo, Sarajevo, Bosnia and Herzegovina; AAZ Clinical and Public Health Research, Independent Clinician Scientist and Public Health Researcher, Ahmedabad, India; ABA Department of Statistics, Computer Science, Applications “G. Parenti” (DiSIA), University of Florence and University of Palermo, Florence, Italy; ABB Chettinad Hospital & Research Institute, Chettinad Academy of Research and Education, Chennai, India; ABC Department of Cardiology, Icahn School of Medicine at Mount Sinai, New York, NY, USA; ABD JSS Medical College Department of Biochemistry, Jagadguru Sri Shivarathreeswara Academy of Health Education and Research, Mysuru, India; ABE Sheffield Teaching Hospitals NHS Foundation Trust, Sheffield, UK; ABF Division of Pathology, ICAR-Indian Veterinary Research Institute, Bareilly, India; ABG Neurology Department Institute of Human Behavior and Allied Sciences, University of Delhi, New Delhi, India; ABH Department of Community Medicine, University of Peradeniya, Peradeniya, Sri Lanka; ABI Research Department, Nepal Health Research Council, Kathmandu, Nepal; ABJ Institute of Occupational, Social and Environmental Medicine, Goethe University, Frankfurt am Main, Germany; ABK Population Interventions Unit, University of Melbourne, Melbourne, VIC, Australia; ABL Department of Life Science and Public Health, Università Cattolica del Sacro Cuore (Catholic University of the Sacred Heart), Rome, Italy; ABM Escola Superior de Saúde (Higher School of Health), Instituto Politécnico do Porto (Polytechnic Institute of Porto), Porto, Portugal; ABN UCIBIO Applied Molecular Biosciences Unit, University of Porto, Porto, Portugal; ABO Department of Gastroenterology, Pontifical Catholic University of Chile, Santiago, Chile; ABP University of California San Diego, La Jolla, CA, USA; ABQ Public Health Intelligence Unit, National Institute of Public Health, Cuernavaca, Mexico; ABR Department of Quantitative Methods, Loyola University Andalusia, Sevilla, Spain; ABS Health Research Institute, University of Canberra, Canberra, New South Wales (NSW), Australia; ABT Department of Physiology, Saveetha University, Chennai, India; ABU Department of Otolaryngology - Head and Neck Surgery, Medical University of South Carolina, Charleston, SC, USA; ABV Department of Anesthesiology, Central South University, Changsha, China; ABW Joe C. Wen School of Population & Public Health, University of California Irvine, Irvine, CA, USA; ABX Department of Family Medicine, University of Washington, Seattle, WA, USA; ABY Research Institute for Advanced Nursing (RIAN), Dong Nai Technology University, Dong Nai Province, Viet Nam; ABZ Institute of Health Economics and Technology (iHEAT), Hanoi, Viet Nam; ACA Department of Medicine, Can Tho University of Medicine and Pharmacy, Can Tho, Viet Nam; ACB Institute of Health Research, University of Health and Allied Sciences, Ho, Ghana; ACC Department of Public Health, Jazan University, Jazan, Saudi Arabia; ACD Department of Social Medicine and Health Care Organisation, Medical University of Varna, Varna, Bulgaria; ACE Nuclear Medicine Deparment, ASST Spedali Civili di Brescia and Università degli Studi di Brescia, Brescia, Italy; ACF Cardio-Thoraco-Vascular Department, Azienda Sanitaria Universitaria Giuliano Isontina, Trieste, Italy; ACG Department of Medical Laboratory Sciences, Iran University of Medical Sciences, Tehran, Iran; ACH Independent Consultant, Bridgewater, NJ, USA; ACI Department of Epidemiology, University of Pittsburgh, Pittsburgh, PA, USA; ACJ Department of Psychiatry, University of Pittsburgh Medical Center, Pittsburgh, PA, USA; ACK School of Public Health, University of Sydney, Sydney, NSW, Australia; ACL Department of Pathology, China Medical University, Liaoning, China; ACM Department of Otolaryngology-Head and Neck Surgery, Medical University of South Carolina, Charleston, SC, USA; ACN School of Sociology, University College Dublin, Dublin, Ireland; ACO Postgraduate Program in Health Sciences, Federal University of Rio Grande do Sul, Rio Grande, Brazil; ACP Postgraduate Program in Epidemiology, Federal University of Rio Grande do Sul, Porto Alegre, Brazil; ACQ School of Population Health, Curtin University, Perth, WA, Australia; ACR School of Medicine, Federal University of Bahia, Salvador, Brazil; ACS Department of Internal Medicine, Escola Bahiana de Medicina e Saúde Pública (Bahiana School of Medicine and Public Health), Salvador, Brazil; ACT Faculty of Science and Humanities, SRM Institute of Science and Technology, Kattankulathur, India; ACU Department of Infection and Tropical Medicine, University of Sheffield, Sheffield, UK; ACV Department of Pharmacology, All India Institute of Medical Sciences, Rajkot, India; ACW Ateneo Center for Research and Innovation, Ateneo De Manila University, Pasig City, Philippines; ACX Department and Demography and Health, London School of Hygiene & Tropical Medicine, London, UK; ACY Department of Microbiology and Immunology, Northwestern University, Chicago, IL, USA; ACZ Department of Biological and Chemical Sciences, Michael and Cecilia Ibru University, Delta State, Nigeria; ADA Department of Psychiatry, Dalhousie University, Halifax, NS, Canada; ADB Department of Psychiatry, University of Alberta, Edmonton, AB, Canada; ADC Environmental and Occupational Health Research Center, Shahroud University of Medical Sciences, Shahroud, Iran; ADD Higher School of Technology, Sultan Moulay Slimane University, Beni Mellal, Morocco; ADE School of Nursing and Midwifery, La Trobe University, Melbourne, VIC, Australia; ADF Advanced Nursing Department, Universitas Airlangga (Airlangga University), Surabaya, Indonesia; ADG Gastrointestinal and Liver Disease Research Center, Guilan University of Medical Sciences, Rasht, Iran; ADH Iran University of Medical Sciences, Iran University of Medical Sciences, Tehran, Iran; ADI Semnan University of Medical Sciences and Health, Samara University, Semnan, Iran; ADJ Isenberg School of Management, University of Massachusetts Amherst, Amherst, MA, USA; ADK Massachusetts General Hospital, Boston, MA, USA; ADL Centre for Global Health Inequalities Research (CHAIN), Norwegian University of Science and Technology, Trondheim, Norway; ADM Private Orthodontist, Ahvaz, Iran; ADN Faculty of Science and Health, University of Portsmouth, Hampshire, UK; ADO Almoosa College of Health Sciences, Al Ahsa, Saudi Arabia; ADP Clinical Pathology Department-Faculty of Medicine, Mansoura University, Mansoura, Egypt; ADQ Department of Basic Medical Sciences, University of Sharjah, Sharjah, United Arab Emirates; ADR Department of Anatomy and Embryology, Mansoura University, Mansoura, Egypt; ADS Department of Radiology, Tehran University of Medical Sciences, Tehran, Iran; ADT Deanship of Preparatory Year and Supporting Studies, Imam Abdulrahman Bin Faisal University, Dammam, Saudi Arabia; ADU College of Medicine, RAK Medical and Health Sciences University, Ras Al-Khaimah, United Arab Emirates; ADV Faculty of Medicine, Ain Shams University, Cairo, Egypt; ADW Clinical Pharmacy Program, Al Ain University, Al Ain, United Arab Emirates; ADX AAU Health and Biomedical Research Center, Al Ain University, Abu Dhabi, United Arab Emirates; ADY Pediatrics and Neonatology Department, Kafr Elshiekh University, Kafr Elshiekh, Egypt; ADZ Pharmacology and Therapeutic Department, United Arab Emirates University, Al Ain, United Arab Emirates; AEA Sharjah Institute for Medical Research, University of Sharjah, Sharjah, United Arab Emirates; AEB Department of Internal Medicine, Ain Shams University, Cairo, Egypt; AEC Section of Adult Hematology, King Saud University, Riyadh, Saudi Arabia; AED College of Medicine, Korea University, Seoul, South Korea; AEE Houston Methodist Hospital, Houston, TX, USA; AEF the National Institute of Public health Research, Ministry of Health, Nouakchott, Mauritania; AEG Pediatric Dentistry and Dental Public Health Department, Alexandria University, Alexandria, Egypt; AEH Department of Clinical and Chemical Pathology, Cairo University, Cairo, Egypt; AEI Basic Medical Sciences Department, University of Sharjah, Sharjah, United Arab Emirates; AEJ Research Institute of Medical & Health Sciences, University of Sharjah, Sharjah, United Arab Emirates; AEK School of Pharmacy and Pharmaceutical Sciences, Ulster University, Coleraine, UK; AEL Department of Clinical Pathology, Mansoura University, Mansoura, Egypt; AEM Department of Infectious Diseases and Public Health, City University of Hong Kong, Hong Kong, China; AEN Department of Animal Medicine, Zagazig University, Zagazig, Egypt; AEO Department of Pediatrics, University of Texas, Dallas, TX, USA; AEP Faculty of Veterinary Medicine, Damanhour University, Damanhur, Egypt; AEQ Department of Midwifery, Woldia University, Addis Ababa, Ethiopia; AER Department of Research, UChicago Research Bangladesh, Dhaka, Bangladesh; AES Department of Public Health and Tropical Medicine, James Cook University, Townsville, QLD, Australia; AET Department of Physiology, University of Medical Sciences, Ondo, Ondo, Nigeria; AEU Department of Community Health, Obafemi Awolowo University, Ile-Ife, Nigeria; AEV Ivan Research Institute, University of Nigeria, Enugu, Nigeria; AEW Department of Paediatrics, University of Lagos, Lagos, Nigeria; AEX Department of Paediatrics, Lagos University Teaching Hospital, Lagos, Nigeria; AEY Preventive Medicine and Public Health Research Center, Iran University of Medical Sciences, Tehran, Iran; AEZ Multiple Sclerosis Research Center, Tehran University of Medical Sciences, Tehran, Iran; AFA Department of Bacteriology and Virology, Semnan University of Medical Sciences, Semnan, Iran; AFB Cancer Research Center, Semnan University of Medical Sciences, Semnan, Iran; AFC Health Research and Innovation Science Centre, Klaipeda University, Klaipeda, Lithuania; AFD Drexel Dornsife School of Public Health, Drexel University, Philadelphia, PA, USA; AFE Department of Biomedical Sciences, Humanitas University, Milan, Italy; AFF IRCCS Humanitas Research Hospital, Milan, Italy; AFG Department of Electrical and Computer Engineering, Tarbiat Modares University, Tehran, Iran; AFH Research Centre for Healthcare and Community, Coventry University, Coventry, UK; AFI Department of Oral Biology, Riphah International University, Islamabad, Pakistan; AFJ The Maldives National University, The Maldives National University, Male, Maldives; AFK Director of the Scientific and Technological Park, Kazakh National Medical University, Almaty, Kazakhstan; AFL Department of Medicine, Korea University, Seoul, South Korea; AFM Department of Food Hygiene and Quality Control, University of Tehran, Tehran, Iran; AFN Department of Biomedical and Biotechnological Sciences, University of Catania, Catania, Italy; AFO Epidemiology and Biostatistics Unit, IRCCS Pascale, Naples, Italy; AFP Department of Public Health Sciences, Clemson University, Clemson, SC, USA; AFQ Pediatric Infectious Disease Research Center, Tehran University of Medical Sciences, Tehran, Iran; AFR Department of Pathology, Shiraz University of Medical Sciences, Shiraz, Iran; AFS Student Research Committee, Shiraz University of Medical Sciences, Shiraz, Iran; AFT Department of Otolaryngology, Shiraz University of Medical Sciences, Shiraz, Iran; AFU College of Medicine, AlMaarefa University, Riyadh, Saudi Arabia; AFV Saveetha Medical College and Hospital, Saveetha Institute of Medical and Technical Sciences (SIMATS), Chennai, India; AFW Department of Psychology, Federal University of Sergipe, São Cristóvão, Brazil; AFX Department of Radiography and Imaging Technology, Green International University, Lahore, Pakistan; AFY Dentistry Research Institute, Tehran University of Medical Sciences, Tehran, Iran; AFZ Obesity and Eating Habits Research Center, Tehran University of Medical Sciences, Tehran, Iran; AGA Department of Clinical Psychology, University of Dhaka, Dhaka, Bangladesh; AGB Community-based Inclusive Mental Health Department, Centre for Disability in Development (CDD), Dhaka, Bangladesh; AGC Satcher Health Leadership Institute, Morehouse School of Medicine, Atlanta, GA, USA; AGD School of Medicine, Emory University, Atlanta, GA, USA; AGE Department of Veterinary Tropical Diseases, University of Pretoria, Pretoria, South Africa; AGF Animal Production and Health Division (EMPRES), Food and Agriculture Organization of the United Nations, Rome, Italy; AGG Charité University Berlin, Charité Universitätsmedizin Berlin (Charité University Medical Center Berlin), Berlin, Germany; AGH School of Engineering, Edith Cowan University, Joondalup, WA, Australia; AGI Department of Electrical and Computer Engineering (ECE), Tarbiat Modares University, Tehran, Iran; AGJ University Institute of Radiological Sciences and Medical Imaging Technology, The University of Lahore, Lahore, Pakistan; AGK Laboratory of Experimental Medicine, Kazakh National Medical University, Almaty, Kazakhstan; AGL National Institute for Stroke and Applied Neurosciences, Auckland University of Technology, Auckland, New Zealand; AGM Research Center of Neurology, Moscow, Russia; AGN cardiovascular department, Shiraz University of Medical Sciences, Shiraz, Iran; AGO Department of Social Medicine and Epidemiology, Guilan University of Medical Sciences, Rasht, Iran; AGP Department of Pharmacy, Wollega University, Nekemte, Ethiopia; AGQ National Institute of Environmental Health, Chinese Center for Disease Control and Prevention, Beijing, China; AGR Department of Biomedical Engineering, University of Houston, Houston, TX, USA; AGS Division of Neurology, University of Toronto, Toronto, ON, Canada; AGT Department of Neurobiology, Care Sciences, and Society, Karolinska Institute, Stockholm, Sweden; AGU Department of Social Sciences, University of Nicosia, Nicosia, Cyprus; AGV Institute of Health Sciences, Wollega University, Nekemte, Ethiopia; AGW Jimma University, Jimma, Ethiopia; AGX Medical School, Universidad de Navarra, Pamplona, Spain; AGY Department of Public Health, University of Naples “Federico II”, Naples, Italy; AGZ Department of Pharmacology, Gadjah Mada University, Yogyakarta, Indonesia; AHA Department of Cell Biology and Biotechnology, K.A. Timiryazev Institute of Plant Physiology, Moscow, Russia; AHB Department of Cardiac, Thoracic, Vascular Sciences and Public Health, University of Padova, Italy, Padova, Italy; AHC Department of Neurology, Public Health and Disability, Fondazione IRCCS Istituto Neurologico Carlo Besta, Milano, Italy; AHD Department of Pharmacology, Iranshahr University of Medical Sciences, Iranshahr, Iran; AHE Innovation in Healthcare and Social Services Department, Emilia-Romagna Region, Bologna, Italy; AHF Department of Neuroscience, Multiple Sclerosis Research Center, Ravenna, Italy; AHG Department of Biotechnological and Applied Clinical Sciences, University of L'Aquila, L'Aquila, Italy; AHH Department of Radiology, University of Southern California, Los Angeles, CA, USA; AHI Department of Medicine, Iran University of Medical Sciences, Tehran, Iran; AHJ Clinical Epidemiology Division (KEP), Karolinska Institute, Stockholm, Sweden; AHK College of Medicine, Dentistry and Public Health, James Cook University, Townsville, QLD, Australia; AHL MEDCIDS, Faculty of Medicine of the University of Porto, University of Porto, Porto, Portugal; AHM Center for Health Technology and Services Research (CINTESIS), Porto, Portugal; AHN Department of Dermatology, Kyoto Prefectural University of Medicine, Kyoto, Japan; AHO Department of Pathology, Federal University of Espirito Santo, Vitória, Brazil; AHP Department of Community Medicine and Family Medicine, All India Institute of Medical Sciences, Gorakhpur, India; AHQ Health Services Management Training Centre, Semmelweis University, Budapest, Hungary; AHR Department of Applied Social Sciences, Sapientia Hungarian University of Transylvania, Târgu-Mures, Romania; AHS Department of Community Medicine, Bayero University Kano, Kano, Nigeria; AHT Department of Community Medicine, Aminu Kano Teaching Hospital, Kano, Nigeria; AHU School of Public Health, University of Ghana, Legon, Ghana; AHV Department of Oral Biology and Experimental Dental Research, University of Szeged, Szeged, Hungary; AHW Department of Food Technology, Salahaddin University-Erbil, Erbil, Iraq; AHX Department of Nutrition and Dietetics, Cihan University, Erbil, Iraq; AHY Department of Medical Epidemiology, Mario Negri Institute for Pharmacological Research, Milan, Italy; AHZ Department of Prosthodontics, Saveetha University, Chennai, India; AIA School of American Education, Institute of Health & Management, Institute of Health & Management, Australia, Melbourne, VIC, Australia; AIB Swinburne University of Technology, School of Engineering, Melbourne, VIC, Australia; AIC Department of Pharmacology, Manipal Academy of Higher Education, Manipal, India; AID Department of Biostatistics, Xuzhou Medical University, Xuzhou, China; AIE Key Lab of Environment and Health, Xuzhou Medical University, Xuzhou, China; AIF Joint Surgery and Sports Medicine, Institute of Science Tokyo, Tokyo, Japan; AIG Department of Veterinary Public Health and Preventive Medicine, Usmanu Danfodiyo University, Sokoto, Sokoto, Nigeria; AIH Department of Public Health, SIMAD University Mogadishu. Somalia, Mogadishu, Somalia; AII Centre for Innovation in Mental Health, University of Southampton, Southampton, UK; AIJ School of Medicine, Orebro University, Orebro, Sweden; AIK Department of Medicine, University of Valladolid, Valladolid, Spain; AIL Department of Neurology, Hospital Universitario Rio Hortega, Valladolid, Spain; AIM Infectious Diseases Unit, University of Verona, Verona, Italy; AIN Department of Ophthalmology, University of Basel, Basel, Switzerland; AIO Department of Pharmacology, IES Institute of Pharmacy, Bhopal, India; AIP Professional Services Division, Texas State Board of Pharmacy, Austin, TX, USA; AIQ Institute of Health and Development (ISED), Alliance for Medical Research in Africa (AMedRA), Dakar, Senegal; AIR Alliance for Medical Research in Africa (AMedRA), Dakar, Senegal; AIS Independent Consultant, Rome, Italy; AIT School of Public Health, Shandong First Medical University and Shandong Academy of Medical Sciences, Jinan, China; AIU College of Health Sciences, Addis Ababa University, Addis Ababa, Ethiopia; AIV Department of Midwifery, Adigrat University, Adigrat, Ethiopia; AIW School of Public Health, Adigrat University, Adigrat, Ethiopia; AIX School of Public Health, Bule Hora University, Bule Hora, Ethiopia; AIY Department of Neurosciences, Neurology and Stroke Unit, ASST Grande Ospedale Metropolitano Niguarda, Milan, Italy; AIZ Institute of Public Health, Jagiellonian University Medical College, Krakow, Poland; AJA School of Medicine and Population Health, University of Sheffield, Sheffield, UK; AJB School of Engineering and Applied Science, George Washington University, Washington, DC, USA; AJC Department of Public Health, Debre Berhan University, Debre Berhan, Ethiopia; AJD Department of Public Health, Menelik II Medical and Health Science College, Addis Ababa, Ethiopia; AJE Child Health Analytics Research Program, Telethon Kids Institute, Perth, WA, Australia; AJF Mayo Clinic, rochester, MN, USA; AJG Infectious Disease Research Center, Kermanshah University of Medical Sciences, Kermanshah, Iran; AJH Pediatric Department, Kermanshah University of Medical Sciences, Kermanshah, Iran; AJI Research Committee of qom university of medical sciences, Qom University of Medical Sciences, Qom, Iran; AJJ Department of Medical Genetics, Shahid Beheshti University of Medical Sciences, Tehran, Iran; AJK Center for Comprehensive Genetic Services, Shahid Beheshti University of Medical Sciences, Tehran, Iran; AJL Neurology Department of Imam Khomeini hospital, Tehran University of Medical Sciences, Tehran, Iran; AJM Department of Global Health Sciences, University of California San Francisco, San Francisco, CA, USA; AJN Department of Ophthalmology, Tehran University of Medical Sciences, Tehran, Iran; AJO Tropical Health Department, Alexandria University, Alexandria, Egypt; AJP Family and Community Medicine Department, King Khalid University, Abha, Saudi Arabia; AJQ Research Group for Childhood Cancer, Danish Cancer Research Institute, Copenhagen, Denmark; AJR Department of Physics, University of Zanjan, Zanjan, Iran; AJS Department of Dermatology, Mazandaran University of Medical Sciences, Sari, Iran; AJT Obstetrics and Gynecology Department, Shahid Beheshti University of Medical Sciences, Tehran, Iran; AJU Department of Biology, Government Institute of Science, Nagpur, India; AJV Department of Clinical Research, National Institute For Research In Reproductive and Child Health, Mumbai, India; AJW Department of Epidemiology and Prevention, IRCCS Neuromed, Pozzilli, Italy; AJX GBD Collaborating Unit, Norwegian Institute of Public Health, Bergen, Norway; AJY Department of Medicine, Stanford University, Palo Alto, CA, USA; AJZ Department of Biological Sciences and Chemistry (DBSC), University of Nizwa, Nizwa, Oman; AKA Adelaide Medical School, University of Adelaide, Adelaide, SA, Australia; AKB Department of Nursing, Aksum University, Aksum, Ethiopia; AKC Department of Anesthesiology and Critical Care Medicine, Ospedale SS Annunziata Savigliano, Savigliano, Italy; AKD Department of Cardiac Surgery, Cleveland Clinic Abu Dhabi, Abu Dhabi, United Arab Emirates; AKE Lerner College of Medicine, Case Western Reserve University, Cleveland, OH, USA; AKF Department of Pharmaceutical Sciences and Drug Research, Punjabi University Patiala, Patiala, India; AKG Department of Radiation Oncology, All India Institute of Medical Sciences, Punjab, India; AKH Department of Medicine, All India Institute of Medical Sciences, Bathinda, India; AKI Department of Biostatistics, Tarbiat Modares University, Tehran, Iran; AKJ Quantitative Department, Non-Communicable Diseases Research Center (NCDRC), Tehran, Iran; AKK Department of Health Systems and Policy Research, Indian Institute of Public Health, Gandhinagar, India; AKL Non-Communicable Diseases Research Center (NCDRC), Non-Communicable Diseases Research Center (NCDRC), Tehran, Iran; AKM Department of Oral and Maxillofacial Surgery, Shahid Beheshti University of Medical Sciences, Tehran, Iran; AKN Laboratório de Farmacognosia, REQUIMTE/LAQV, Porto, Portugal; AKO Department of Urban Public Health, University of Massachusetts Boston, Boston, MA, USA; AKP Senior Department of Tuberculosis, The Eighth Medical Center of PLA General Hospital, Beijing, China; AKQ College of Health Sciences, University of Sharjah, Sharjah, United Arab Emirates; AKR Oncological Network, Prevention and Research Institute, Institute for Cancer Research, Prevention and Clinical Network, Florence, Italy; AKS Midwifery Department, Debre Tabor University, Debre Tabor, Ethiopia; AKT Department of Epidemiology, Universidade de São Paulo (University of São Paulo), São Paulo, Brazil; AKU Department of Dermatology, Case Western Reserve University, Libertyville, IL, USA; AKV Nuffield Department of Orthopaedics, Rheumatology, and Musculoskeletal Sciences, University of Oxford, Oxford, UK; AKW Liverpool Orthopaedic and Trauma Service, University of Liverpool, Liverpool, UK; AKX Department of Public Health and Preventive Medicine, Charles University, Prague, Czech Republic; AKY National Institutes of Health, Bethesda, MD, USA; AKZ Department of Health Informatics, Bahir Dar University, Bahir Dar, Ethiopia; ALA Tianjin Medical University General Hospital, Tianjin Medical University, Tianjin, China; ALB Cheeloo College of Medicine, Shandong University, Jinan, China; ALC Department of Epidemiology and Biostatistics, Anhui Medical University, Hefei, China; ALD Health Direction, Local Health Authority of Ferrara, Ferrara, Italy; ALE Department of Clinical Science, University Of Sulaimani, Sulaimani, Iraq; ALF Harrington Heart and Vascular Institute, Case Western Reserve University, Cleveland, OH, USA; ALG Division of Cardiovascular Medicine, Ohio State University, Columbus, OH, USA; ALH Department of the Health Directorate, Local Health Authority of Bologna, Bologna, Italy; ALI Department of Biomedical and Neuromotor Sciences, University of Bologna, Bologna, Italy; ALJ Group Health Department, Nanyang Central Hospital, Nanyang, China; ALK Department of Geriatric Neurology, Shaanxi Provincial People's Hospital, Xi'an, China; ALL Big Data Institute, Nuffield Department of Population Health, University of Oxford, Oxford, UK; ALM Department of Urban Planning and Design, University of Hong Kong, Hong Kong, China; ALN Divison of Epidemology, Vanderbilt University Medical Center, Nashville, TN, USA; ALO Department of Epidemiology and Biostatistics, University of South Carolina, Columbia, SC, USA; ALP Centre for Noncommunicable Diseases and Nutrition, BRAC University, Dhaka, Bangladesh; ALQ Department of Preventive Cardiology & Medicine, Eternal Heart Care Centre & Research Institute, Jaipur, India; ALR Department of Medicine, Mahatma Gandhi University Medical Sciences, Jaipur, India; ALS Department of Toxicology, Shriram Institute for Industrial Research, Delhi, India; ALT Department of Anaesthesia, Maulana Azad Medical College, New Delhi, India; ALU College of Medicine and Public Health, Flinders University, Melbourne/Darwin, Victoria (VIC), Australia; ALV Doctoral Program in Biomedical Gerontology, Pontifical Catholic University of Rio Grande do Sul, Porto Alegre, Brazil; ALW Research Unit in Epidemiology Clinic, Mexican Institute of Social Segurity, Colima, Mexico; ALX College of Health Science, Dilla University, Dilla, Ethiopia; ALY Department of Medical Microbiology, Bahir Dar University, Bahir Dar, Ethiopia; ALZ Department of Midwifery, Arba Minch University, Arba Minch, Ethiopia; AMA The George Institute for Global health, University of New South Wales, Sydney, NSW, Australia; AMB School of Medicine, Urmia University of Medical Sciences, Urmia, Iran; AMC School of Medicine, Hamedan University of Medical Sciences, Hamedan, Iran; AMD Department of Liver Tumor, Cancer Center, Cho Ray Hospital, Ho Chi Minh City, Viet Nam; AME Liver Transplant Unit, Cho Ray Hospital, Ho Chi Minh City, Viet Nam; AMF Department of Radiology, Massachusetts General Hospital, Boston, MA, USA; AMG Obesity Research Center, Shahid Beheshti University of Medical Sciences, Tehran, Iran; AMH Department of Community Medicine, Post Graduate Institute of Medical Education and Research, Chandigarh, India; AMI Centre for Community Medicine, All India Institute of Medical Sciences, New Delhi, India; AMJ Department of Infectious Disease Epidemiology, Robert Koch Institute, Berlin, Germany; AMK Department of Public Health, Charité Institute of Public Health, Berlin, Germany; AML Department of Pharmacy, American University of Madaba, Amman, Jordan; AMM Department of Family and Community Medicine, Arabian Gulf University, Manama, Bahrain; AMN Biochemistry Department, Ain Shams University, Cairo, Egypt; AMO School of Health and Environmental Studies, Hamdan Bin Mohammed Smart University, Dubai, United Arab Emirates; AMP Department of Medical and Technical Information Technology, Bauman Moscow State Technical University, Moscow, Russia; AMQ Guthrie Medical Group, Guthrie Medical Group, Cortland, NY, USA; AMR Edirne Public Health Center, Edirne Provincial Health Directorate, Edirne, Turkiye; AMS Sakarya University, Sakarya, Turkiye; AMT Department of Critical Care and Emergency Nursing, Zanjan University of Medical Sciences, Zanjan, Iran; AMU Centre for Neuromuscular and Neurological Disorders (Perron Institute), The University of Western Australia, Perth, WA, Australia; AMV Stroke Research Centre, Perron Institute for Neurological and Translational Science, Perth, WA, Australia; AMW Department of Health and Education, Torrens University Australia, Melbourne, VIC, Australia; AMX Department of Population Science and Human Resource Development, University of Rajshahi, Rajshahi, Bangladesh; AMY Department of Chemistry, University of Hail, Hail, Saudi Arabia; AMZ Department of Medicine, MedStar Health, Baltimore, MD, USA; ANA Medical Research Unit, Universitas Syiah Kuala (Syiah Kuala University), Banda Aceh, Indonesia; ANB Vital and Health Statistics, Ministry of Health, Beirut, Lebanon; ANC Department for Health, University of Bath, Bath, UK; AND Department of Epidemiology Population Biostatistics and Health Promotion, Universitas Airlangga (Airlangga University), Surabaya, Indonesia; ANE Directorate General of Health Human Resources, Ministry of Health, Jakarta, Indonesia; ANF Research Unit, Parc Sanitari Sant Joan de Deu, Barcelona, Spain; ANG Department of Mental Health, Biomedical Research Networking Center for Mental Health Network (CiberSAM), Madrid, Spain; ANH Department of Advanced Nursing, Universitas Airlangga (Airlangga University), Surabaya, Indonesia; ANI School of Nursing and Midwivery, La Trobe University, Bundoora, VIC, Australia; ANJ Department of Zoology and Entomology, Al-Azhar University, Cairo, Egypt; ANK Department of Health Research Methods, Evidence, and Impact, McMaster University, Hamilton, ON, Canada; ANL Department of Biochemistry and Molecular Biology, Tejgaon College, Dhaka, Bangladesh; ANM Faculty of Nursing, Chulalongkorn University, Bangkok, Thailand; ANN Department of Food Technology and Nutrition Science, Noakhali Science and Technology University, Noakhali, Bangladesh; ANO Department of Ophthalmology, Iran University of Medical Sciences, Tehran, Iran; ANP Department of Medical Surgical, Shahroud University of Medical Sciences, Shahrekord, Iran; ANQ institute of radiology and radiological sciences, Tehran University of Medical Sciences, tehran, Iran; ANR institute of radiology and radiological sciences, Johns Hopkins University, Baltimore, MD, USA; ANS Research Center for Traditional Medicine and History of Medicine, Shiraz University of Medical Sciences, Shiraz, Iran; ANT Department of Periodontics, RAK Medical & Health Sciences University, Ras Al-Khaimah, United Arab Emirates; ANU Department of Oral Rehabilitation, University of Khartoum, Khartoum, Sudan; ANV Department of Biotechnology, Lahore University of Biological and Applied Sciences, Lahore, Pakistan; ANW Community Medicine, Federal University Teaching Hospital, Lafia, Nigeria; ANX Department of Epidemiology and Community Medicine, Federal University of Lafia, Lafia, Nigeria; ANY Department of Medicine, University of Khartoum Faculty of Medicine, Khartoum, Sudan; ANZ Ajman University, Ajman, United Arab Emirates; AOA Health Policy and Financing, Society for Family Health, Abuja, Nigeria; AOB Sina Trauma and Surgery Research Center, Tehran University of Medical Sciences, Tehran, Iran; AOC Skaane University Hospital, Skaane County Council, Malmö, Sweden; AOD Institute of Pharmaceutical Sciences, University of Veterinary and Animal Sciences, Lahore, Pakistan; AOE Department of Pharmacy Administration and Clinical Pharmacy, Xian Jiaotong University, Xian, China; AOF Faculty of Kinesiology, University of New Brunswick, Fredericton, NB, Canada; AOG School of Allied Health, Murdoch University, Murdoch, WA, Australia; AOH Community-Oriented Nursing Midwifery Research Center, Shahrekord University of Medical Sciences, Shahrekord, Iran; AOI Independent Consultant, Santa Clara, CA, USA; AOJ Department of Medicine, MedStar Health, Washington, DC, USA; AOK Department of Medicine, Georgetown University, Washington, DC, USA; AOL Babes-Bolyai University, Cluj-Napoca, Romania; AOM Urology and Nephrology Research Center, Shahid Beheshti University of Medical Sciences, Tehran, Iran; AON Ophthalmic Research Center (ORC), Shahid Beheshti University of Medical Sciences, Tehran, Iran; AOO Australian Centre for Health Service Innovations, Queensland University of Technology, Brisbane, Queensland (QLD), Australia; AOP National Agency for Strategic Research in Medical Sciences Education, Ministry of Health and Medical Education, Tehran, Iran; AOQ Department of Virology, Lorestan University of Medical Sciences, Khorramabad, Iran; AOR Graduate School of Medicine, University of Tokyo, Tokyo, Japan; AOS Kasturba Medical College, Mangalore, Manipal Academy of Higher Education, Manipal, India; AOT Department of Pulmonology, Yokohama City University, Yokohama, Japan; AOU National Human Genome Research Institute (NHGRI), National Institutes of Health, Bethesda, MD, USA; AOV School of Population and Public Health, University of British Columbia, Vancouver, BC, Canada; AOW Centre for Advancing Health Outcomes, Vancouver, BC, Canada; AOX Department of Decision and Information Sciences, University of Houston, Houston, TX, USA; AOY Public Health Research Group, Nature Study Society of Bangladesh, Khulna, Bangladesh; AOZ Department of Statistics, Shahjalal University of Science and Technology, Sylhet, Bangladesh; APA Department of Physics, University of Rajshahi, Rajshahi, Bangladesh; APB Department of Population Sciences, University of Dhaka, Dhaka, Bangladesh; APC Department of Maternal and Child Health, International Centre for Diarrhoeal Disease Research, Bangladesh, Dhaka, Bangladesh; APD Department of Legal Medicine and Bioethics, Carol Davila University of Medicine and Pharmacy, Bucharest, Romania; APE Department of Clinical Legal Medicine, National Institute of Legal Medicine Mina Minovici, Bucharest, Romania; APF Department of Internal Medicine, Carol Davila University of Medicine and Pharmacy, Bucharest, Romania; APG National School of Tropical Medicine, Baylor College of Medicine, Houston, TX, USA; APH Rubin Institute for Advanced Orthopedics, Sinai Hospital of Baltimore, Baltimore, MD, USA; API Institute for Occupational and Maritime Medicine (ZfAM), University Medical Center Hamburg-Eppendorf (UKE), Hamburg, Germany; APJ Department of Applied Mathematics, University of Washington, Seattle, WA, USA; APK Department of Psychological and Cognitive Sciences, Tsinghua University, Beijing, China; APL Department of Epidemiology and Health Statistics, Central South University, Changsha, China; APM School of Public Health, Xuzhou Medical University, Xuzhou, China; APN Faculty of Medicine, The Chinese University of Hong Kong, Hong Kong, China; APO Department of Otorhinolaryngology Head and Neck Surgery, Shanghai Jiao Tong University, Shanghai, China; APP Pediatric Nursing Department, University of Indonesia, Depok, Indonesia; APQ Department of Public Health and Community Medicine, Shaikh Zayed Postgraduate Medical Institute, Lahore, Pakistan; APR Department of Humanities, COMSATS University Islamabad, Islamabad, Pakistan; APS Nephrology and Urology Research Center, Baqiyatallah University of Medical Sciences, Tehran, Iran; APT Department of Biological Sciences and Chemistry, University of Nizwa, Nizwa, Oman; APU Department of Biomolecular Sciences, University of Zakho, Zakho, Iraq; APV Artur Riggs Diabetes & Metabolism Research Institute, Cancer Prevention and Research Institute, Duarte, CA, USA; APW Zagazig University, Zagazig, Egypt; APX International Master Program for Translational Science, Taipei Medical University, Taipei, Taiwan; APY Department of Occupational Safety and Health, China Medical University, Taiwan, Taichung, Taiwan; APZ Department of Occupational Therapy, Asia University, Taiwan, Taichung, Taiwan; AQA Department of Biomedical, Metabolic, and Neural Science, University of Modena and Reggio Emilia, Modena, Italy; AQB Department of Health Promotion and Education, University of Ibadan, Ibadan, Nigeria; AQC Faculty of Pharmacy, Sultan Zainal Abidin University, Malaysia, Terengganu, Malaysia; AQD Fenerbahce University, Istanbul, Turkiye; AQE Department of Cardiovascular Medicine, Mayo Clinic, Phoenix, AZ, USA; AQF Science and Technology Park, Kazakh National Medical University, Almaty, Kazakhstan; AQG Pharmacoepidemiology Department, Sanofi, Cambridge, MA, USA; AQH Health Policy and Management Department, City University of New York, New York, NY, USA; AQI Collaborative Alliance Research and Education (CARE) Programme, Episcope Research Service, Aberdeen, Scotland; AQJ Division of Infectious Diseases, Veterans Affairs Greater Los Angeles, Los Angeles, CA, USA; AQK West Africa RCC, Africa Centre for Disease Control and Prevention, Abuja, Nigeria; AQL Department of Community Medicine, University College Hospital, Ibadan, Ibadan, Nigeria; AQM Faculty of Medicine, University of Belgrade, Belgrade, Serbia; AQN Faculty of Medical Sciences, University of Kragujevac, Kragujevac, Serbia; AQO Department of Biostatistics, Iran University of Medical Sciences, Tehran, Iran; AQP Department of Chemical Pathology, University of Jos, Jos, Nigeria; AQQ Department of Chemical Pathology, Jos University Teaching Hospital, Jos, Nigeria; AQR Department of Health Research, ICMR National Institute for Research in Tuberculosis, Chennai, India; AQS Faculty of Health and Life Sciences, University of Exeter, Exeter, UK; AQT Department of Psychology, Wuhan University, Wuhan, China; AQU Faculty of Public Health, Universitas Muhammadiyah Aceh (Muhammadiyah University of Aceh), Banda Aceh, Indonesia; AQV Department of Microbiology, University of Maiduguri, Maiduguri, Nigeria; AQW Department of Biotechnology, Sharda University, Greater Noida, India; AQX School of Pharmacy, Taipei Medical University, Taipei, Taiwan; AQY Department of Pharmaceutical Technology, Sekolah Tinggi Ilmu Farmasi Riau, Pekanbaru, Indonesia; AQZ Independent Researcher, Cairo, Egypt; ARA 4-Green Research Society, Journal of Biological Sciences and Public Health, Dhaka, Dhaka, Bangladesh; ARB School of Pharmacy, BRAC University, Dhaka, Bangladesh; ARC School of Public Health, The University of Queensland, Brisbane, QLD, Australia; ARD Department of Surveillance and Health Equity Science, American Cancer Society, Atlanta, GA, USA; ARE Department of Clinical Pharmacy & Pharmacy Practice, Asian Institute of Medicine, Science and Technology, Bedong, Malaysia; ARF Malaysian Academy of Pharmacy, Puchong, Malaysia; ARG Clinical Laboratory Department, Tobruk University, Tobruk, Libya; ARH Department of Blood Transmitted Diseases, National Centre for Disease Control (NCDC), Tobruk, Libya; ARI Department of Urology, Kazakh National Medical University, Almaty, Kazakhstan; ARJ Department of Health Services Research, University of Tsukuba, Tsukuba, Japan; ARK Department of Non-Communicable Disease Epidemiology, London School of Hygiene & Tropical Medicine, London, UK; ARL Knowledge Translation Program, Centre for Health Evaluation and Outcome Sciences, Vancouver, BC, Canada; ARM Department of Biotechnology, Karpagam Academy of Higher Education, Coimbatore, Coimbatore, India; ARN Department of Environmental Health Engineering, Guilan University of Medical Sciences, Rasht, Iran; ARO Department of Special Education, University of Ibadan, Ibadan, Nigeria; ARP Department of Health Studies, University of Richmond, Richmond, VA, USA; ARQ Department of Nursing, Arak University of Medical Sciences, Arak, Iran; ARR School of Medicine, Volgograd state medical university, Volgograd, Russia; ARS Department of Statistics and Epidemiology, Tabriz University of Medical Sciences, Tabriz, Iran; ART Shiraz Neuroscience Research Center, Shiraz University of Medical Sciences, Shiraz, Iran; ARU Manipal College of Health Professions, Manipal Academy of Higher Education, Udupi, India; ARV College of Medicine and Health Sciences, Arabian Gulf University, Manama, Bahrain; ARW Government Hospitals, Manama, Bahrain; ARX Department of Oral Pathology and Microbiology, King George's Medical University, Lucknow, India; ARY Department of Health and Safety, Dubai Municipality, Dubai, United Arab Emirates; ARZ UNESCO-TWAS Section of Economic & Social Sciences, Humanities & Arts, The World Academy of Sciences UNESCO-TWAS, Trieste, Italy; ASA Shaanxi University of Technology, Hanzhong, China; ASB Department of Surgery, Iran University of Medical Sciences, Tehran, Iran; ASC Department of Neurosurgery, Medical College of Wisconsin, Milwaukee, WI, USA; ASD Department of Health Informatics, Qassim University, Buraydah, Saudi Arabia; ASE Public Health Sciences, University of Chicago, Chicago, IL, USA; ASF Department of Primary Care Medicine, Universiti Malaya, Kuala Lumpur, Malaysia; ASG Iranian Center of Neurological Research, Tehran University of Medical Sciences, Tehran, Iran; ASH SRM Medical College Hospital and Research Centre, Sri Ramaswamy Memorial Institute of Science and Technology, Chengelpet, India; ASI Department of Public Health, Daffodil International University, Dhaka, Bangladesh; ASJ Department of Public and Community Health, Frontier University Garowe, Puntland, Somalia; ASK Institute for Musculoskeletal Health, University of Sydney, Sydney, New South Wales (NSW), Australia; ASL Shahrekord University of Medical Science, Shahrekord University of Medical Science, Shahrekord, Iran; ASM Department of Stem Cells and Developmental Biology, Royan Institution, Tehran, Iran; ASN Health Informatic Lab, Boston University, Boston, MA, USA; ASO Department of Medicine, University of Mississippi Medical Center, Jackson, MS, USA; ASP Department of Medicine, Jinnah Sindh Medical University, Karachi, Pakistan; ASQ KI Solna, Karolinska Institute, Stockholm, Sweden; ASR Invasive Fungi Research Center, Mazandaran University of Medical Sciences, Sari, Iran; ASS Department of Medical Mycology, Mazandaran University of Medical Sciences, Sari, Iran; AST Department of Biochemistry, Government Medical College, Mysuru, India; ASU Department of Oral Medicine and Periodontology, University of Peradeniya, Peradeniya, Sri Lanka; ASV Department of Oral Medicine and Periodontology, Saveetha University, Chennai, India; ASW Department of Research, University of Puthisastra, Phnom Penh, Cambodia; ASX Faculty of Dental Sciences, University of Peradeniya, Peradeniya, Sri Lanka; ASY Postgraduate Institute of Medicine, University of Colombo, Colombo, Sri Lanka; ASZ Faculty of Graduate Studies, Institute for Violence and Injury Prevention, Colombo, Sri Lanka; ATA Department of Endocrinology, Diabetes and Metabolism, Christian Medical College and Hospital (CMC), Vellore, India; ATB Department of Medicine, University of Melbourne, Melbourne, VIC, Australia; ATC Department of General Medicine, Manipal Academy of Higher Education, Mangalore, India; ATD Department of Internal Medicine, GCS Medical College, Hospital & Research Centre, Ahmedabad, India; ATE Department of Public Health, Tongji University, Shanghai, China; ATF Xuzhou Medical University, Xuzhou Medical University, Xuzhou, China; ATG Department of Orthopedics, Wuhan University, Wuhan, China; ATH Department of Biomedical Sciences, City University of Hong Kong, Hong Kong, China; ATI School of Biology and Engineering (School of Health Medicine Modern Industry), Guizhou Medical University, Guiyang, China; ATJ Faculty of Veterinary Medicine, University of Calgary, Calgary, AB, Canada; ATK Young Researchers and Elite Club, Islamic Azad University, Karaj, Iran; ATL Rothschild Foundation Hospital, Institut Français de Myopie, Paris, France; ATM Singapore Eye Research Institute, Singapore Eye Research Institute, Singapore, Singapore; ATN Hungarian Health Management Association, Budapest, Hungary; ATO Department of Biomedical Engineering, Hong Kong Polytechnic University, Hong Kong, China; ATP Department of Community Medicine, Manipal Academy of Higher Education, Mangalore, India; ATQ Department of Gastroenterology and Hepatology, Stanford University, Stanford, CA, USA; ATR Department of Economics, National Open University, Benin City, Nigeria; ATS Population, Fertility, and Mortality Team, University of Washington, Seattle, WA, USA; ATT Nursing and Midwifery Research Department, Hamad Medical Corporation, Doha, Qatar; ATU Department of Family Medicine and Public Health, University of Opole, Opole, Poland; ATV Department of Radiology and Nuclear Medicine, University of Jordan, Amman, Jordan; ATW Research Department, TobaccoFree Research Institute Ireland, Dublin, Ireland; ATX School of Public Health, University College Cork, Cork, Ireland; ATY Department of Statistics, Salahaddin University, Erbil, Iraq; ATZ Department of Business Administrations, Cihan University, Erbil, Iraq; AUA Department of Pharmacology, Post Graduate Institute of Medical Education and Research, Chandigarh, India; AUB Independent Consultant, Pune, India; AUC Department of Health, Khoy Medical Sciences, Khoy, Iran; AUD Department of Endocrinology, Bharti Hospital Karnal, Karnal, India; AUE University Centre for Research and Development, Chandigarh University, Mohali, India; AUF Canberra Business School, University of Canberra, Hawker, ACT, Australia; AUG College of Pharmacy, Jamia Hamdard, Al Kharj, Saudi Arabia; AUH NIHR Global Health Research Unit on Global Surgery, University of Birmingham, Birmingham, UK; AUI Prasanna School of Public Health, Manipal Academy of Higher Education, Manipal, India; AUJ Care and Public Health Research Institute (CAPHRI), Maastricht University, Maastricht, Netherlands; AUK Department of General Medical Practice No. 2, Kazakh National Medical University, Almaty, Kazakhstan; AUL Russell H. Morgan Department of Radiology and Radiological Science, Johns Hopkins University, Baltimore, MD, USA; AUM Department of Public Health, South Wales University, Treforest, UK; AUN Osun State Hospital Management Board; AUO Cardiothoracic Surgery, Stanford University, Palo Alto, CA, USA; AUP Department of Biostatistics and Epidemiology, Abadan University of Medical Sciences, Abadan, Iran; AUQ Microbiology, Virology and Immunology Department, I. Horbachevsky Ternopil National Medical University, Ternopil, Ukraine; AUR Department of Health Sciences, University of York, York, UK; AUS Department of Rehabilitation Sciences, Qatar University, Doha, Qatar; AUT School of Health and Environmental Science, Korea University, Seoul, South Korea; AUU Department of Anesthesia, Critical Care and Pain Medicine, Massachusetts General Hospital, Boston, MA, USA; AUV T. H. Chan School of Public Health, Harvard University, Boston, MA, USA; AUW Office of the Executive Director, Cephas Health Research Initiative Inc, Ibadan, Nigeria; AUX The Hansjörg Wyss Department of Plastic and Reconstructive Surgery, NYU Langone Health, New York, NY, USA; AUY Cleft Lip and Palate Surgery Division, Global Smile Foundation, Norwood, MA, USA; AUZ Department of Psychiatry, King George's Medical University, Lucknow, India; AVA 2nd Department of Cardiology, Aristotle University of Thessaloniki, Thessaloniki, Greece; AVB Department of Basic Medical Sciences, Yarmouk University, Irbid, Jordan; AVC Department of general medicine, Shahid Beheshti University of Medical Sciences, Tehran, Iran; AVD Social Determinants of Health Research Center, Tabriz University of Medical Sciences, Tabriz, Iran; AVE Saveetha Medical College and Hospital, Saveetha University, Chennai, India; AVF Chair and Department of Medical Microbiology, Poznan University of Medical Sciences, Poznan, Poland; AVG Department of Public Health and Mortality Studies, International Institute for Population Sciences, Mumbai, India; AVH Amity Stem Cell Institute (ASCI), Amity University Haryana, Gurugram, India; AVI Department of Reproductive, Family and Population Health, Addis Ababa University, Addis Ababa, Ethiopia; AVJ Department of Anesthesiology & Pain Medicine, University of Washington, Seattle, WA, USA; AVK Department of Medicine, Jacobi Medical Center, New York, NY, USA; AVL Department of Clinical Research and Epidemiology, Institute of Liver and Biliary Sciences, New Delhi, India; AVM Department of Neurosurgery, Johns Hopkins University, Baltimore, MD, USA; AVN Cardiac Primary Prevention Research Center, Tehran University of Medical Sciences, Tehran, Iran; AVO Department of Cardiac Electrophysiology, Tehran University of Medical Sciences, Tehran, Iran; AVP School of Pharmacy, Jimma University, Jimma, Ethiopia; AVQ Center for Tobacco Research, Ohio State University, Columbus, OH, USA; AVR Department of Ophthalmology, Harvard University, Boston, MA, USA; AVS Eye Unit, MyungSung Medical College, Addis Ababa, Ethiopia; AVT Centre for Adolescent Health, Murdoch Childrens Research Institute, Parkville, VIC, Australia; AVU Department of Psychological Medicine, University of Otago, Christchurch, New Zealand; AVV Department of Human Nutrition, National Research Institute for Agriculture, Food and Environment, Jouy-en-Josas, France; AVW Sorbonne Paris Nord University, Bobigny, France; AVX Faculty of Medicine, Mashhad University of Medical Sciences, Mashhad, Iran; AVY Amity Institute of Forensic Sciences, Amity University, Noida, India; AVZ College of Health Sciences, Abu Dhabi University, Abu Dhabi, United Arab Emirates; AWA Lahore Medical Research Center, Lahore Medical Research Center, Lahore, Pakistan; AWB Department of Veterinary Medicine, United Arab Emirates University, Al Ain, United Arab Emirates; AWC Faculty of Veterinary Medicine, Kafrelsheikh University, Kafrelsheikh, Egypt; AWD Department of Nursing, Zarqa University, Zarqa, Jordan; AWE Department of Obstetrics & Gynecology, Iran University of Medical Sciences, Tehran, Iran; AWF Department of Medicine, Guilan University of Medical Sciences, Rasht, Iran; AWG Department of Biostatistics, Mazandaran University of Medical Sciences, Sari, Iran; AWH Department of Medical Genetics and Molecular Medicine, Mashhad University of Medical Sciences, Mashhad, Iran; AWI Department of Public Health, Mohammed VI Center for Research and Innovation, Rabat, Morocco; AWJ Higher Institute of Nursing Professions and Health Techniques, Rabat, Morocco; AWK Halal Research Center of the Islamic Republic of Iran (IRI), Iran Food and Drug Administration, Tehran, Iran; AWL Center for Atmospheric Particle Studies (CAPS), Carnegie Mellon University, Pittsburgh, PA, USA; AWM Department of Mechanical Engineering (MechE), Carnegie Mellon University, Pittsburgh, PA, USA; AWN Department of Cardiology, University of South Wales, Treforest, UK; AWO Department of Cardiology, University of Buckingham, Buckingham, UK; AWP NITVAR, Indian Council of Medical Research, Pune, India; AWQ Academy of Scientific and Innovative Research (AcSIR), India, Ghaziabad, India; AWR Department of Community Medicine, National Institute of Preventive and Social Medicine, Dhaka, Bangladesh; AWS International Center for Chemical and Biological Sciences, International Center for Chemical and Biological Sciences, Karachi, Pakistan; AWT College of Medicine, University of Hail, Hail, Saudi Arabia; AWU Department of Community and Preventive Medicine, King Edward Medical University, Lahore, Pakistan; AWV Natural and Medical Sciences Research Center, University of Nizwa, Nizwa, Oman; AWW BDStatistics Center for Research, Dhaka, Bangladesh; AWX Karachi Medical and Dental College, Karachi, Pakistan; AWY Internal Medicine Department, Reading Hospital Tower Health, Reading, PA, USA; AWZ Centre for Interdisciplinary Research in Basic Sciences, Jamia Millia Islamia, New Delhi, India; AXA Epidemiology Program, Jazan University, Jazan, Saudi Arabia; AXB Department of Physical Therapy, King Abdulaziz University, Jeddah, Saudi Arabia; AXC Central Department of zoology, Tribhuvan University, Kathmandu, Nepal; AXD Department of Epidemiology, Non-Communicable Diseases Research Center (NCDRC), Tehran, Iran; AXE Physiology and Biomedical engineering, Mayo Clinic, Rochester, MN, USA; AXF College of Health, Wellbeing and Life Sciences, Sheffield Hallam University, Sheffield, UK; AXG College of Arts and Sciences, Ohio University, Zanesville, OH, USA; AXH Faculty of Nursing, Yarmouk University, Irbid, Jordan; AXI Department of Orthopaedics, Postgraduate Medical Institute, Sangrur, India; AXJ Department of Neurosurgery, Shahid Beheshti University of Medical Sciences, Tehran, Iran; AXK Department of Public Health, Jordan University of Science and Technology, Irbid, Jordan; AXL Penn Medicine, University of Pennsylvania, Philadelphia, PA, USA; AXM Bone and Joint Reconstruction Research Center, Iran University of Medical Sciences, Tehran, Iran; AXN Ophthalmic Epidemiology Research Center, Shahid Beheshti University of Medical Sciences, Tehran, Iran; AXO University of Sulaimani College of Medicine, Sulaimani Polytechnic University, Sulaymaniyah, Iraq; AXP Department of Internal Medicine, Corewell Health East William Beaumont University Hospital, Royal Oak, MI, USA; AXQ Department of Medical Oncology, Miami Cancer Institute, Miami, FL, USA; AXR Department of Epidemiology and Biostatistics, Non-Communicable Diseases Research Center (NCDRC), Tehran, Iran; AXS Department of Clinical Research, Icahn School of Medicine at Mount Sinai, New York City, NY, USA; AXT Department of Health Management and Economics, Qom University of Medical Sciences, Qom, Iran; AXU Department of Health Economics, Iran University of Medical Sciences, Tehran, Iran; AXV Research Department, University of Inland Norway, Elverum, Norway; AXW Department of Public Health, New Mexico State University, Las Cruces, NM, USA; AXX Department of Pharmacology, University of Gondar, Gondar, Ethiopia; AXY Cardiovascular Disease Initiative, Broad Institute of MIT and Harvard, Cambridge, MA, USA; AXZ Department of Biomedical Sciences, Seoul National University, Seoul, South Korea; AYA Department of Health Policy and Management, Korea University, Seoul, South Korea; AYB Health and Healing Research, Education, and Service, Inc., Boston, MA, USA; AYC Millennium Prevention, Inc., Westwood, MA, USA; AYD The Pacific Community, Noumea, New Caledonia; AYE College of Medicine, Qatar University, Doha, Qatar; AYF Department of Community Medicine, Manipal Academy of Higher Education, Manipal, India; AYG School of Health Sciences, Kristiania University College, Oslo, Norway; AYH Department of International Health and Sustainable Development, Tulane University, New Orleans, LA, USA; AYI Department of Nursing and Health Promotion, Oslo Metropolitan University, Oslo, Norway; AYJ School of Pharmacy and Emerging Sciences, Baddi University of Emerging Sciences & Technology, Himachal Pradesh, India; AYK Child Health Analytics, The Kids Research Institute Australia, Nedlands, WA, Australia; AYL Department of Brain Sciences, University College London, London, UK; AYM Department of Public Health, University of Helsinki, Helsinki, Finland; AYN Department of Public Health Dentistry, Krishna Vishwa Vidyapeeth (Deemed to be University), Karad, India; AYO Centre for Disease Burden, Norwegian Institute of Public Health, Bergen, Norway; AYP Endocrinology Department, Bogomolets National Medical University, Kyiv, Ukraine; AYQ Scientific Department, Medical Laboratory CSD, Kyiv, Ukraine; AYR Department of Global Health, University of Washington, Seattle, WA, USA; AYS Global Healthcare Consulting, New Delhi, India; AYT Department of Medicine, Harvard University, Boston, MA, USA; AYU Social Determinants of Health Research Center, Shahid Beheshti University of Medical Sciences, Tehran, Iran; AYV Mycobacteriology Unit, Center for Health Promotion and Research, Bamenda, Cameroon; AYW Children's Medical Center, Tehran University of Medical Sciences, Tehran, Iran; AYX Scientific and Educational Center for Neurology and Applied Neuroscience, Kazakh National Medical University, Almaty, Kazakhstan; AYY Department of Ophthalmology, Aristotle University of Thessaloniki, Thessaloniki, Greece; AYZ Centre for the Business and Economics of Health, The University of Queensland, Brisbane, Queensland (QLD), Australia; AZA Copernicus Institute of Sustainable Development, Utrecht University, Utrecht, Netherlands; AZB Department of Science and Environmental Studies, The Education University of Hong Kong, Hong Kong, China; AZC Department of General Practice and Family Medicine, Kharkiv National Medical University, Kharkiv, Ukraine; AZD Department of Epidemiology, IQVIA, Frankfurt am Main, Germany; AZE University Hospital Marburg, Marburg, Germany; AZF Amity institute of Public Health and Hospital administration, Amity University Noida, Noida, India; AZG Kasturba Medical College, Manipal, Manipal Academy of Higher Education, Udupi, India; AZH Department of Public Health, Central University, Accra, Ghana; AZI Central University, Accra, Ghana; AZJ School of Pharmacy, University of Ghana, Legon, Ghana; AZK Department of Anthropology, Panjab University, Chandigarh, India; AZL School of Applied Science, Republic Polytechnic, Singapore, Singapore; AZM centre for biotechnology, Siksha ‘O’ Anusandhan Deemed to be University, Bhubaneswar, India; AZN Department of Demography, University of Montreal, Montreal, QC, Canada; AZO Department of Social and Preventive Medicine, University of Montreal, Montreal, QC, Canada; AZP Department of Biochemistry, University of Hail, Hail, Saudi Arabia; AZQ Department of Pediatrics, Kuopio University Hospital, Kuopio, Finland; AZR Institute of Clinical Medicine, University of Eastern Finland, Kuopio, Finland; AZS College of Medicine, National Cheng Kung University, Tainan, Taiwan; AZT Research and Publication Activity Division, Kazakh National Medical University, Almaty, Kazakhstan; AZU Center of Medicine and Public Health, Asfendiyarov Kazakh National Medical University, Almaty, Kazakhstan; AZV Department of Medicine, Queensland Health, Brisbane, QLD, Australia; AZW Amity Centre for Water Studies and Research, Amity University Rajasthan, Jaipur, India; AZX Gastroenterology Department, Ahalia Hospital, Abu Dhabi, United Arab Emirates; AZY Allied Health Sciences, Bahria University Medical and Dental College, Karachi, Pakistan; AZZ Institute for Excellence in Health Equity, New York University, New York, NY, USA; BAA Department of Psychiatry, University of Nairobi, Nairobi, Kenya; BAB Department of Community Medicine, Rajendra Institute of Medical Sciences, Ranchi, India; BAC Department of Anaesthesiology, Rajendra Institute of Medical Sciences, Ranchi, Ranchi, India; BAD Department of Economics, Manipal University, Jaipur, Jaipur, India; BAE Department of Pediatrics, Post Graduate Institute of Medical Education and Research, Chandigarh, India; BAF Department of Community Medicine, Jawaharlal Institute of Postgraduate Medical Education and Research, Karaikal, India; BAG Corporate Nursing and midwifery Research, Hamad Medical Corporation, Doha, Qatar; BAH Section of Cardiology, University of Manitoba, Winnipeg, MB, Canada; BAI Translational Health Sciences, University of Bristol, Bristol, UK; BAJ Department of Clinical Subjects, Al Farabi Kazakh National University, Almaty, Kazakhstan; BAK Faculty of Medicine and Health Science, Universitas Kristen Satya Wacana (Satya Wacana Christian University), Salatiga, Indonesia; BAL School of Nursing, Taipei Medical University, Taipei, Taiwan; BAM Department of Microbiology, Central University of Punjab, Bathinda, India; BAN National Research and Innovation Agency (BRIN), Jakarta, Indonesia; BAO Institute for Health Sciences, STIKES Bethesda Yakkum Yogyakarta Indonesia, Yogyakarta, Indonesia; BAP Department of Public Health and Epidemiology, Khalifa University of Science and Technology, Abu Dhabi, United Arab Emirates; BAQ Faculty of Public Health, University of Indonesia, Depok, Indonesia; BAR Department of Pediatric Oncology, Medicana Health International, Istanbul, Turkiye; BAS Department of Pediatric Oncology, Hacettepe University, Ankara, Turkiye; BAT Department of Nursing, University of Massachusetts Boston, Boston, MA, USA; BAU Department of Environment and Public Health, University of Environment and Sustainable Development, Somanya, Ghana; BAV Clinical Research Center, Turku University Hospital, Turku, Finland; BAW Heart Center, University of Turku, Turku, Finland; BAX Kasturba Medical College, Manipal, Manipal Academy of Higher Education, Manipal, India; BAY Department of Medicine and Surgery, University of Milano - Bicocca, Milan, Italy; BAZ Pediatric Emergency Department, Fondazione IRCCS Ospedale Maggiore Policlinico, Milan, Italy; BBA Department of Clinical Sciences and Community Health, University of Milan, Milan, Italy; BBB Department of Medicine, UniCamillus University, Rome, Italy; BBC Department of Nursing Science, Bayero University Kano, Kano, Nigeria; BBD Department of Global Public Health, Karolinska Institute, Stockholm, Sweden; BBE Institute for Social and Health Sciences, University of South Africa, Pretoria, South Africa; BBF Division of Evidence Synthesis, Foundation for People-centric Health Systems, New Delhi, India; BBG Division of Lifestyle Medicine, Centre for Health: The Specialty Practice, New Delhi, India; BBH School of Digital Science, Universiti Brunei Darussalam (University of Brunei Darussalam), Bandar Seri Begawan, Brunei; BBI Institute of Applied Data Analytics, Universiti Brunei Darussalam (University of Brunei Darussalam), Bandar Seri Begawan, Brunei; BBJ Department of Research, Kazakh National Medical University, Almaty, Kazakhstan; BBK Department of Chemistry, Dayalbagh Educational Institute, Agra, India; BBL Indian Council of Medical Research, New Delhi, India; BBM School of Dentistry, The University of Queensland, Brisbane, QLD, Australia; BBN Unidad de Genética y Salud Pública, Instituto de Ciencias Médicas, Las Tablas, Panama; BBO Ministry of Health, Hospital Joaquín Pablo Franco Sayas, Las Tablas, Panama; BBP Department of Psychiatry and Psychotherapy, University of Regensburg, Regensburg, Germany; BBQ Department of Behavioural Sciences and Learning, Linköping University, Linköping, Sweden; BBR Centre for Family Welfare, University of Indonesia, Depok, Indonesia; BBS Department of Global Health and Health Security, Taipei Medical University, Taipei, Taiwan; BBT Centre for Clinical Research, Faculty of Health, Medicine and Behavioural Sciences, The University of Queensland, Brisbane, QLD, Australia; BBU Health Systems, Administration and Management, Babcock University, Sagamu, Nigeria; BBV Health Services Management Programme, Plasma University, Mogadishu, Somalia; BBW School of Physical Therapy, The University of Western Ontario, London, Ontario (ON), Canada; BBX University of Medicine and Pharmacy at Ho Chi Minh City, Ho Chi Minh City, Viet Nam; BBY Independent Consultant, Ho Chi Minh City, Viet Nam; BBZ Faculty of Medicine, University of Medicine and Pharmacy at Ho Chi Minh City, Ho Chi Minh City, Viet Nam; BCA Department of Cardiovascular Research, Methodist Hospital, Merrillville, IN, USA; BCB International Ph.D. Program in Medicine, Taipei Medical University, Taipei, Taiwan; BCC Research Center for Artificial Intelligence in Medicine, Taipei Medical University, Taipei, Taiwan; BCD Department of Clinical and Experimental Medicine, University of Catania, Catania, Italy; BCE Department of Family Medicine, University of Texas Medical Branch, Galveston, TX, USA; BCF STEM, University of South Australia, Adelaide, SA, Australia; BCG Department of Precision Medicine, Sungkyunkwan University, Suwon, South Korea; BCH Department of Preventive Medicine, Korea University, Seoul, South Korea; BCI Department of Cardiothoracic and Vascular Surgery, Westpfalz Klinikum, Kaiserslautern, Germany; BCJ Department of Cardiothoracic Surgery, University of Patras, Patras, Greece; BCK Centre for Healthy Brain Ageing, University of New South Wales, Sydney, New South Wales (NSW), Australia; BCL SC Neurologia, Salute Pubblica e Disabilità (Neurology, Public Health, Disability Unit), Fondazione IRCCS Istituto Neurologico Carlo Besta (IRCCS Foundation Carlo Besta Neurological Institute), Milan, Italy; BCM Faculty of Science, Universiti Brunei Darussalam (University of Brunei Darussalam), Bandar Seri Begawan, Brunei; BCN Center for Dentistry and Oral Hygiene, University of Groningen, Groningen, Netherlands; BCO Stomatological Hospital, Southern Medical University, Guangzhou, China; BCP Shanxi Medical University, Taiyuan, China; BCQ Department of Rheumatology and Immunology, The People's Hospital of Baoan Shenzhen, Shenzhen, China; BCR Department of Endocrinology and Metabolism, The First Hospital of China Medical University, Shenyang, China; BCS School of Public Health, Xuzhou medical university, Xuzhou, China; BCT Department of Health Promotion and Health Education, National Taiwan Normal University, Taipei, Taiwan; BCU First Clincal Medicine, Shandong University of Traditional Chinese Medicine, Jinan, China; BCV Nutrition & Health Innovation Research Institute, Edith Cowan University, Perth, WA, Australia; BCW The First Affiliated Hospital of Guangzhou Medical University, Guangzhou Medical University, Guangzhou, China; BCX School of Medicine, Shanghai Jiao Tong University, Shanghai, China; BCY Department of Psychiatry, Washington University in St. Louis, St. Louis, MO, USA; BCZ Population Studies Center, University of Pennsylvania, Philadelphia, PA, USA; BDA School of Nursing, Johns Hopkins University, Baltimore, MD, USA; BDB Discipline of Physiology, National University of Ireland - Galway, Galway, Ireland; BDC First Clinical Medical College, Shandong University of Chinese Medicine, jinan, China; BDD Department of Pharmaceutical Regulatory Affairs and Management, Manipal Academy of Higher Education, Manipal, India; BDE College of Public Health, China Medical University, Taiwan, Taichung, Taiwan; BDF Asbestos Diseases Research Institute, Concord, NSW, Australia; BDG WHO Collaborating Centre for Public Health Education and Training, Imperial College London, London, UK; BDH Department of Food Science and Human Nutrition, Iowa State University, Ames, IA, USA; BDI The Center for Drug Safety and Policy Research, Xi'an Jiaotong University, Xi'an, China; BDJ International Centre for Future Health Systems, University of New South Wales, Sydney, NSW, Australia; BDK Department of Medical Sciences, Uppsala University, Uppsala, Sweden; BDL Department of Medicine, Norrtälje Hospital (Tiohundra), Norrtälje, Sweden; BDM Management Science and Engineering, Stanford University, Stanford, CA, USA; BDN Lerner Research Institute, Cleveland Clinic, Cleveland, OH, USA; BDO Department of Quantitative Health Science, Case Western Reserve University, Cleveland, OH, USA; BDP Department of Radiology and Biomedical Imaging, Yale University, New Haven, CT, USA; BDQ Centre for Intelligent Healthcare, Coventry University, Coventry, UK; BDR School of Nursing and Health Sciences, Hong Kong Metropolitan University, Hong Kong, China; BDS College of Mathematics and Computer, Xinyu University, Xinyu, China; BDT Department of Epidemiology and Biostatistics, Peking University, Beijing, China; BDU School of Life Sciences, University of Technology Sydney, Sydney, NSW, Australia; BDV Xiangya Hospital, Central South University, Changsha, China; BDW Department of Molecular Epidemiology, German Institute of Human Nutrition Potsdam-Rehbrücke, Potsdam, Germany; BDX German Center for Diabetes Research (DZD), München-Neuherberg, Germany; BDY Department of Infectious Diseases, Monash University, Melbourne, VIC, Australia; BDZ Department of Infectious Diseases, Alfred Health, Melbourne, VIC, Australia; BEA Department of Cardiology, University of Cologne, Cologne, Germany; BEB School of Medicine, Universidad Espíritu Santo, Samborondón, Ecuador; BEC Vicerrectoría de Investigación y Postgrado, Universidad de Los Lagos, Osorno, Chile; BED Department of Community Health, Shahrekord University of Medical Sciences, Shahrekord, Iran; BEE Social Determinants of Health Research Center, Shahrekord University of Medical Sciences, Shahrekord, Iran; BEF Ashok & Rita Patel Institute of Physiotherapy, Charotar University of Science and Technology, Anand, India; BEG School of Medicine, National Autonomous University of Mexico, Mexico City, Mexico; BEH Department of Spine Surgery, Qingdao Municipal Hospital Group, Qingdao, China; BEI Geospatial Health and Development Team-Child Health Analytics, Telethon Kids Institute, Perth, WA, Australia; BEJ Scientific Research and Surveillance Systems, Macha Research Trust, Choma, Zambia; BEK School of Medicine, Federal University of Juiz de Fora, Juiz de Fora, Brazil; BEL The Third Department of Hepatic Surgery, Eastern Hepatobiliary Surgery Hospital, Shanghai, China; BEM Moores Cancer Center, University of California San Diego, San Diego, CA, USA; BEN Department of Population Health Sciences, Duke University, Durham, NC, USA; BEO Dodoma Medical Research Centre, National Institute for Medical Research in Tanzania, Dodoma, Tanzania; BEP College of Engineering, Effat University, Jeddah, Saudi Arabia; BEQ Management of Information Systems Department, The American College of Greece, Aghia Paraskevi, Greece; BER Department of Medicine, University of Alberta, Edmonton, AB, Canada; BES Tulane University, New Orleans, LA, USA; BET Department of Chemistry, Salahaddin University-Erbil, Erbil, Iraq; BEU Centre for Public Health and Wellbeing, University of the West of England, Bristol, UK; BEV Perelman School of Medicine, University of Pennsylvania, Philadelphia, PA, USA; BEW Faculty of Veterinary Medicine, Suez Canal University, Ismailia, Egypt; BEX Department of Microbiology and Parasitology, King Salman International University, South of Sinai, Egypt; BEY 2nd Department of Propaedeutic Surgery, University of Athens, Athens, Greece; BEZ Department of Periodontology, Pomeranian Medical University, Szczecin, Poland; BFA Anesthesiology Research Center, Shahid Beheshti University of Medical Sciences, Tehran, Iran; BFB Department of Disease Burden, Norwegian Institute of Public Health, Bergen, Norway; BFC Associate Laboratory i4HB, University Institute of Health Sciences - CESPU, Gandra, Portugal; BFD UCIBIO Research Unit on Applied Molecular Biosciences, University Institute of Health Sciences, Gandra, Portugal; BFE School of Infection & Immunity, University of Glasgow, Glasgow, UK; BFF Department of Pharmacy, University of Naples Federico II, Naples, Italy; BFG Department of Forensic Medicine & Toxicology, Mysore Medical College & Research Institute, Mysooru, India; BFH Department of Health & Family Welfare, Government of Karnataka, Bangalore, India; BFI Department of Emergency Medicine, Sri Lakshmi Narayana Institute of Medical Science, Puducherry, Pondicherry, India; BFJ Department of Public Health, Urmia University of Medical Sciences, Urmia, Iran; BFK Research Center, Cihan University, Sulaymaniyah, Iraq; BFL Neurology Department, University of Miami, Miami, FL, USA; BFM Microbiology Department, Nicosia General Hospital, Nicosia, Cyprus; BFN Department of Pharmacology, All India Institute of Medical Sciences, Bhubaneswar, India; BFO Department of Public Health, Trnava University, Trnava, Slovakia; BFP School of Public Health, Imperial College London, London, UK; BFQ The Orthopaedic Department, October 6 University, 6th of October City, Egypt; BFR Digestive Diseases Research Institute (DDRI), Tehran University of Medical Sciences, Tehran, Iran; BFS Non-communicable Disease Research Center, Shiraz University of Medical Sciences, Shiraz, Iran; BFT Department of Neurology, King George's Medical University, Lucknow, India; BFU University Institute of Public Health, The University of Lahore, Lahore, Pakistan; BFV Rabigh Faculty of Medicine, King Abdulaziz University, Jeddah, Saudi Arabia; BFW Department of Maternal-Child Nursing and Public Health, Federal University of Minas Gerais, Belo Horizonte, Brazil; BFX University of Kansas Medical Center, A.T. Still University, Kansas City, KS, USA; BFY College of Medicine and Health Sciences, University of Rwanda, Kigali, Rwanda; BFZ Internal Medicine Department, MedStar Health, Washington, DC, USA; BGA Internal Medicine Department, Eisenhower Health, Palm Desert, CA, USA; BGB Department of Cardiovascular Science, University of Manchester, Manchester, UK; BGC Population Health Research Institute (PHRI), McMaster University, Hamilton, ON, Canada; BGD Corporate Nursing and Midwifery Research Department, Hamad Medical Corporation, Doha, Qatar; BGE International Center for Chemical and Biological Sciences, University of Karachi, Karachi, Pakistan; BGF Department of Epidemiology and Biostatistics, Isfahan University of Medical Sciences, Isfahan, Iran; BGG Biomedical Engineering Research Center (CREB), Universitat Politècnica de Catalunya (Barcelona Tech - UPC), Barcelona, Spain; BGH Department of Epidemiology and Biostatistics, Tehran University of Medical Sciences, Tehran, Iran; BGI School of Medicine and Surgery, University of Milan Bicocca, Monza, Italy; BGJ Laboratory of Public Health, IRCCS Istituto Auxologico Italiano, Milan, Italy; BGK Department of Urology, Anhui Medical University, Hefei, China; BGL Department of Biomedical Engineering, University of Isfahan, Isfahan, Iran; BGM Automatic Control Department, Universitat Politècnica de Catalunya (Barcelona Tech - UPC), Barcelona, Spain; BGN Far Eastern University, Manila, Philippines; BGO Department of Neurosurgery, Icahn School of Medicine at Mount Sinai, New York, NY, USA; BGP Department of Food, Environmental and Nutritional Sciences, University of Milan, Milano, Italy; BGQ Faculty of Human Kinetics, University of Lisbon, Lisbon, Portugal; BGR Department of Economics, Instituto Tecnologico Autonomo de Mexico, Mexico City, Mexico; BGS Department of Infectious Diseases, Instituto Nacional de Nutrición Salvador Zubirán, Mexico City, Mexico; BGT Department of Non-communicable Diseases and Mental Health, Pan American Health Organization, Washington, DC, USA; BGU Department of Food, Environmental and Nutritional Sciences, University of Milan, Milan, Italy; BGV Campus Fortaleza, Federal Institute of Education, Science and Technology of Ceará, Fortaleza, Brazil; BGW Department of Nutrition and Dietetics, University of Concepción, Concepción, Chile; BGX Centre for Healthy Living, University of Concepción, Concepción, Chile; BGY Faculty of Humanities and Health Sciences, Curtin University, Sarawak, Malaysia; BGZ Jeffrey Cheah School of Medicine and Health Sciences, Monash University, Subang Jaya, Malaysia; BHA Medical Scientist Training Program, Northwestern University, Chicago, IL, USA; BHB Department of Nursing, Muhammadiyah University of Surakarta, Ponorogo, Indonesia; BHC Research Institute for Endocrine Sciences, Shahid Beheshti University of Medical Sciences, Tehran, Iran; BHD Department of Dermatology, Carol Davila University of Medicine and Pharmacy, Bucharest, Romania; BHE Board of Directors, Association of Resident Physicians, Bucharest, Romania; BHF Department of Anatomy and Developmental Biology, Monash University, Clayton, VIC, Australia; BHG Department of Anatomy, Genetics and Biomedical Informatics, University of Colombo, Colombo, Sri Lanka; BHH University of Sydney, University of Sydney, Sydney, NSW, Australia; BHI Division of Immunology, Immunity to Infection and Respiratory Medicine, University of Manchester, Manchester, UK; BHJ North West Lung Centre, Manchester University NHS Foundation Trust, Manchester, UK; BHK Department of Community Medicine, Geetanjali Medical College and Hospital, Udaipur, India; BHL Department of Community Medicine, Apollo Institute of Medical Sciences and Research, Hyderabad, India; BHM Department of Social Medicine, Federal University of Rio Grande do Sul, Porto Alegre, Brazil; BHN Department of Prosthetic dental sciences (Substitutive Dental Sciences), Jazan University, Jazan, Saudi Arabia; BHO Department of Epidemiology, Mahidol-Oxford Tropical Medicine Research Unit, Bangkok, Thailand; BHP Nuffield Department of Medicine, University of Oxford, Oxford, UK; BHQ Research Division, The George Institute for Global Health, New Delhi, India; BHR School of Medicine, University of New South Wales, Sydney, NSW, Australia; BHS Nuffield Department of Population Health, University of Oxford, London, UK; BHT Queensland Brain Institute, The University of Queensland, Brisbane, QLD, Australia; BHU National Centre for Register-based Research, Aarhus University, Aarhus, Denmark; BHV Department of Health Services Research and Policy, London School of Hygiene & Tropical Medicine, London, UK; BHW Australian Centre for Health Services Innovation, Queensland University of Technology, Kelvin Grove, QLD, Australia; BHX Digital Health and Informatics Directorate, Queensland Health, Brisbane, QLD, Australia; BHY Department of Healthcare, University of Vlora, Vlora City, Albania; BHZ Clinic of Social and Family Medicine, University of Crete, Heraklion, Greece; BIA Division of Pediatric Hospital Medicine, Stanford University, Palo Alto, CA, USA; BIB National Heart, Lung and Blood Institute, National Heart, Lung, and Blood Institute, Bethesda, MD, USA; BIC Research and Development Department, Lahore Medical Research Center, Lahore, Pakistan; BID Centre for Health Innovation and Policy, Noida, India; BIE Department of Dental Research Cell, Dr. D. Y. Patil University, Pune, India; BIF Department of Public Health, Arba Minch University, Arba Minch, Ethiopia; BIG School of Public Health, Bahir Dar University, Bahir Dar, Ethiopia; BIH École de Santé Publique, Université libre de Bruxelles (ULB), Brussels, Belgium; BII Department of Medical Laboratory Sciences, Adigrat University, Adigrat, Ethiopia; BIJ Department of Medical Laboratory Science, Bahir Dar University, Bahir Dar, Ethiopia; BIK Department of General Practice, Monash University, Melbourne, VIC, Australia; BIL Dirección General de Investigación, Desarrollo e Innovación (DGIDI), Universidad Científica del Sur (University of the South), Lima, Peru; BIM Department of Medical Microbiology and Immunology, Trinity Medical Sciences University, St. Vincent, Saint Vincent and the Grenadines; BIN Kasturba Medical College, Manipal Academy of Higher Education, Mangalore, India; BIO Department of Adult Health Nursing, Bahir Dar University, Bahir Dar, Ethiopia; BIP Center for Translation Research and Implementation Science, National Institutes of Health, Bethesda, MD, USA; BIQ Department of Medicine, University of Cape Town, Cape Town, South Africa; BIR Department of Physiology, King Saud University, Riyadh, Saudi Arabia; BIS Department of Public Health, University “Federico II” of Naples, Naples, Italy; BIT Comprehensive Cancer Center, Helsinki University Hospital, Helsinki, Finland; BIU University of Helsinki, Helsinki, Finland; BIV General Administration Department, Helsinki University Hospital, Helsinki, Finland; BIW School of Health Sciences, University of Melbourne, Melbourne, VIC, Australia; BIX University Centre Varazdin, University North, Varazdin, Croatia; BIY Department of Pharmacology, University of Kelaniya, Ragama, Sri Lanka; BIZ Clinical Medicine Department, Colombo North Teaching Hospital, Ragama, Sri Lanka; BJA Department of Paediatrics, University of Kelaniya, Ragama, Sri Lanka; BJB University Paediatrics Unit, Colombo North Teaching Hospital, Ragama, Sri Lanka; BJC Department of Pathology, Zagazig University, Zagazig, Egypt; BJD Faculty of Veterinary Medicine, King Salman International University, Ras Sedr, Egypt; BJE Department of Propedeutics of Internal Diseases & Arterial Hypertension, Pomeranian Medical University, Szczecin, Poland; BJF Department of Pathology, Maria Sklodowska-Curie National Research Institute of Oncology, Warsaw, Poland; BJG Dermatology Unit, Fondazione IRCCS Policlinico San Matteo, Pavia, Italy; BJH Department of Oncology, Addis Ababa University, Addis Abeba, Ethiopia; BJI College of Human Medicine, Michigan State University, Flint, MI, USA; BJJ Multidisciplinary Department of Medical-Surgical and Dental Specialties, University of Campania Luigi Vanvitelli, Naples, Italy; BJK Saveetha Dental College and Hospitals, Saveetha University, Chennai, India; BJL Department of Public Health Dentistry, Saveetha Institute of Medical and Technical Sciences (SIMATS), Chennai, India; BJM Global Institute of Public Health, Ananthapuri Hospitals and Research Institute, Trivandrum, India; BJN Faculty of Nursing and Midwifery, Tabriz University of Medical Sciences, Tabriz, Iran; BJO University Health Network, University of Toronto, Toronto, ON, Canada; BJP Department of Radiology, Health Sciences North, Sudbury, ON, Canada; BJQ Bergen Center for Ethics and Priority Setting, University of Bergen, Bergen, Norway; BJR Department of Rehabilitation and Sports Medicine, Kermanshah University of Medical Sciences, Kermanshah, Iran; BJS National Data Management Center for Health, Ethiopian Public Health Institute, Addis Ababa, Ethiopia; BJT Discipline of Psychiatry and Mental Health, University of New South Wales, Sydney, NSW, Australia; BJU Central Clinical School, Faculty of Medicine and Health, University of Sydney, Sydney, New South Wales (NSW), Australia; BJV Department of Forensic Medicine and Toxicology, All India Institute of Medical Sciences, Patna, India; BJW Environment Research Center, Isfahan University of Medical Sciences, Isfahan, Iran; BJX Department of Environmental Health Engineering, Isfahan University of Medical Sciences, Isfahan, Iran; BJY Department of Internal Medicine, Albert Einstein Hospital, Philadelphia, PA, USA; BJZ Molecular Biology Unit, Sirius Training and Research Centre, Khartoum, Sudan; BKA Bio-Statistical and Molecular Biology Department, Sirius Training and Research Centre, Khartoum, Sudan; BKB RAK College of Nursing, RAK Medical and Health Sciences University, Ras Al-Khaimah, United Arab Emirates; BKC Nursing College, Sohag University, Sohag, Egypt; BKD College of Applied and Natural Science, University of Hargeisa, Hargeisa, Somalia; BKE Faculty of Medicine, University of Khartoum, Khartoum, Sudan; BKF Department of Biophysics, All India Institute of Medical Sciences, New Delhi, India; BKG Centre for Interdisciplinary Research in Basic Sciences, Jamia Millia Islamia, New Delhi, Delhi, India; BKH Midwifery Department, Tabriz University of Medical Sciences, Tabriz, Iran; BKI Modeling in Health Research Center, Shahrekord University of Medical Sciences, Shahrekord, Iran; BKJ Health Economics Division, Ministry of Health and Medical Education, Mashhad, Iran; BKK Emam-Reza Hospital, Mashhad University of Medical Sciences, Mashhad, Iran; BKL Skull Base Research Center, Shahid Beheshti University of Medical Sciences, Tehran, Iran; BKM Health Systems and Policy Research Unit, Ahmadu Bello University, Zaria, Nigeria; BKN Heidelberg Institute of Global Health (HIGH), Heidelberg University, Heidelberg, Germany; BKO Medical Microbiology Department, Usmanu Danfodiyo University, Sokoto, Sokoto, Nigeria; BKP Medical Microbiology Department, Usmanu Danfodiyo University Teaching Hospital, Sokoto, Nigeria; BKQ Department of Public Health, Dire Dawa University, Dire Dawa, Ethiopia; BKR University of Gondar, university of Gondar, Gondar, Ethiopia; BKS Department of Medicine, Government Medical College Kozhikode, Kozhikode, India; BKT Biomedical Research Center, QU Health, Qatar University, Doha, Qatar; BKU Department of health sciences azare Bauchi State -Nigeria, National Institute for Research in Tribal Health, Bauchi, Nigeria; BKV School of Pharmacy, Haramaya University, Harar, Ethiopia; BKW Department of Health Services Management, Iran University of Medical Sciences, Iran, Iran; BKX Department of Health Services Management, Isfahan University of Medical Sciences, Isfahan, Iran; BKY Institute of Clinical Physiology, National Research Council, Pisa, Italy; BKZ Department Medical-Surgical Nursing, Golestan University of Medical Sciences, Gorgan, Iran; BLA Department of Mathematics, The University of Jordan, Amman, Jordan; BLB Nonlinear Dynamics Research Center (NDRC), Ajman University, Ajman, United Arab Emirates; BLC Clinical Epidemiology and Public Health Research Unit, Burlo Garofolo Institute for Maternal and Child Health, Trieste, Italy; BLD Department of Physiology, All India Institute of Medical Sciences, Deoghar, India; BLE Department of Biomedical and Dental Sciences and Morphofunctional Imaging, Messina University, Messina, Italy; BLF AI & Cyber Futures Institute, Charles Sturt University, Bathurst, NSW, Australia; BLG The University of Queensland, Brisbane, QLD, Australia; BLH Department of Collective Prevention and Public Health, General Directorate for Personal Care, Health, and Welfare, Bologna, Italy; BLI Department of Epidemiology and Biostatistics, Kurdistan University of Medical Sciences, Sanandaj, Iran; BLJ Gastrointestinal and Liver Diseases Research Center, Iran University of Medical Sciences, Tehran, Iran; BLK Computer, Electrical, and Mathematical Sciences and Engineering Division, King Abdullah University of Science and Technology, Thuwal, Saudi Arabia; BLL Department of Public Health, Oswaldo Cruz Foundation, Recife, Brazil; BLM Department of Public Health, Federal University of Pernambuco, Recife, Brazil; BLN Division of Plastic and Reconstructive Surgery, University of Washington Medical Center, Seattle, WA, USA; BLO Faculty of Medicine, October 6 University, Giza, Egypt; BLP Neurosciences Research Center (NSRC), Tabriz University of Medical Sciences, Tabriz, Iran; BLQ Student Research Committee, Tabriz University of Medical Sciences, Tabriz, Iran; BLR Department of Health Policy, London School of Economics and Political Science, London, UK; BLS Department of Surgery and Cancer, Imperial College London, London, UK; BLT Social Determinants of Health Research Center, Babol University of Medical Sciences, Babol, Iran; BLU Department for Statistics and Econometrics, University of National and World Economy, Sofia, Bulgaria; BLV Tehran Heart Center, Cardiovascular Diseases Research Institute, Tehran University of Medical Sciences, Tehran, Iran; BLW Department of audiology, school of rehabilitation, Shahid Beheshti University of Medical Sciences, Tehran, Iran; BLX Faculty of Biotechnologies (BioTech), ITMO University, Saint Petersburg, Russia; BLY Halal Research Center of IRI, Ministry of Health and Medical Education, Tehran, Iran; BLZ Department of Physical and Environmental Sciences, Texas A&M University, Corpus Christi, TX, USA; BMA Shiraz University of Medical Sciences, Shiraz, Shiraz, Iran; BMB Medical Microbiology and Immunology Department, Cairo University, Cairo, Egypt; BMC Antimicrobial Resistance Research Center, Iran University of Medical Sciences, Tehran, Iran; BMD Hazrat-e Rasool General Hospital, Iran University of Medical Sciences, Tehran, Iran; BME René Rachou Institute, Oswaldo Cruz Foundation, Belo Horizonte, Brazil; BMF Arid Agriculture University Rawalpindi, Pakistan, PMAS Arid Agriculture University Rawalpindi, Pakistan, Rawalpindi, Pakistan; BMG School of Medicine, Keele University, Keele, UK; BMH Division of Psychology and Mental Health, University of Manchester, Manchester, UK; BMI Institute of Molecular Biology and Biotechnology, Bahauddin Zakariya University Multan, Multan, Pakistan; BMJ Department of Evidence and Intelligence for Action in Health, Pan American Health Organization, Washington, DC, USA; BMK Knowledge Management Department, Prahlad Omkarwati Foundation (POF), Mumbai, India; BML Changescape Consulting, Independent Consultant, New Delhi, India; BMM Department of Biochemistry, All India Institute of Medical Sciences, Bhopal, India; BMN Department of Medicine, National University Health System, Singapore, Singapore; BMO Department of Surgery, Ahmadu Bello University Teaching Hospital, Zaria, Nigeria; BMP Department of Mechanical Engineering, North Carolina Agricultural and Technical State University, Greensboro, NC, USA; BMQ Department of Surgery, General University Hospital of Patras, Patras, Greece; BMR Faculty of Medicine, University of Thessaly, Larissa, Greece; BMS College of Health Science, Woldia University, woldia, Ethiopia; BMT Department of Nursing, Sam Ratulangi University, Manado, Indonesia; BMU Department of Health Economics, National Institute for Research in Tuberculosis, Chennai, India; BMV Amity Institute of Pharmacy, Amity University, Noida, India; BMW Department of Community and Global Health, The University of Tokyo, Tokyo, Japan; BMX Epidemiology, Biostatistics and Prevention Institute (EBPI), University of Zürich, Zurich, Switzerland; BMY Center for Infectious Disease Education and Research, The University of Osaka, Suita, Japan; BMZ Clinical Epidemiology Research Unit, Mexican Institute of Social Security, Villa de Alvarez, Mexico; BNA Postgraduate in Medical Sciences, Universidad de Colima, Colima, Mexico; BNB Department of Anatomy, Manipal Academy of Higher Education, Mangalore, India; BNC Department of Paediatrics, Ahmadu Bello University, Zaria, Nigeria; BND Department of Internal Medicine, Cleveland Clinic, Cleveland, OH, USA; BNE Department of Pathology and Microbiology, Duhok University, Duhok, Iraq; BNF Operational Research Center in Healthcare, Near East University, Nicosia, Cyprus; BNG Department of Research Methods, Orthopaedic Research Group, Coimbatore, India; BNH Central Research Laboratory, Meenakshi Medical College Hospital and Research Institute, Chennai, Tamil Nadu, India; BNI University of Tabuk, Tabuk, Saudi Arabia; BNJ Prince Fahad bin Sultan Chair for Biomedical Research, University of Tabuk, Tabuk, Saudi Arabia; BNK Director General, Rwanda Biomedical Centre, Kigali, Rwanda; BNL Department of Technology, The University of Lahore, Lahore, Pakistan; BNM Research Centre for Health Sciences (RCHS), The University of Lahore, Lahore, Pakistan; BNN Department of Psychiatry, Seoul National University, Seoul, South Korea; BNO Department of Neuropsychiatry, Seoul National University Bundang Hospital, Seongnam, South Korea; BNP Department of Ophthalmology, University of Tennessee, Memphis, TN, USA; BNQ Research and Analytics Department, Initiative for Financing Health and Human Development, Chennai, India; BNR Department of Research and Analytics, Bioinsilico Technologies, Chennai, India; BNS Department of Nephrology, Manipal Academy of Higher Education, Manipal, India; BNT Department of Computer Science and IT, Torrens University, Adelaide, SA, Australia; BNU Faculty of Pharmacy, Hasanuddin University, Makassar, Indonesia; BNV College of Health Sciences, Cihan University, Sulaymaniyah, Iraq; BNW University of Sulaimani, Sulaymaniyah, Iraq; BNX Department of Physiotherapy, Tehran University of Medical Sciences, Tehran, Iran; BNY Research Center for War-affected People, Tehran University of Medical Sciences, Tehran, Iran; BNZ Department of Health and Rehabilitation Sciences, Prince Sattam bin Abdulaziz University, Al Kharj, Saudi Arabia; BOA Suraj Eye Institute, Nagpur, India; BOB Department for the Control of Disease, Epidemics, and Pandemics, Ministry of Public Health, Yaoundé, Cameroon; BOC Department of Public Heath, University of Yaoundé I, Yaoundé, Cameroon; BOD Bristol Medical School, Population Health Sciences, University of Bristol, Bristol, UK; BOE Department of Clinical Medicine, Federal University of Minas Gerais, Belo Horizonte, Brazil; BOF Clinical Hospital, Federal University of Minas Gerais, Belo Horizonte, Brazil; BOG National Dental Research Institute Singapore, Duke-NUS Medical School, Singapore, Singapore; BOH Department of Applied Pharmaceutical Sciences and Clinical Pharmacy, Isra University, Amman, Jordan; BOI Nursing & Midwifery Research Department (NMRD), Hamad Medical Corporation, Doha, Qatar; BOJ Division of Endocrinology and Diabetes, University of Vermont, South Burlington, VT, USA; BOK Department of Dental Public Health, King Abdulaziz University, Jeddah, Saudi Arabia; BOL Department of Health Policy and Oral Epidemiology, Harvard University, Boston, MA, USA; BOM Dept of Biological Sciences and Chemistry, University of Nizwa, Nizwa, Oman; BON Department of Community Medicine, University of Peradeniya, Kandy, Sri Lanka; BOO Xiamen Cardiovascular Hospital of Xiamen University, Fujian Branch of National Clinical Research Center for Cardiovascular Diseases, Xiamen, China; BOP Manipal College of Nursing, Manipal Academy of Higher Education, Manipal, India; BOQ Manipal Institute of Management, Manipal Academy of Higher Education, Manipal, India; BOR Department of Biological Sciences, National University of Medical Sciences (NUMS), Rawalpindi, Pakistan; BOS Department of Research, TroDDIVaT Initiative, Buea, Cameroon; BOT Department of Microbiology and Parasitology, University of Buea, Buea, Cameroon; BOU Department of Biomedical Engineering, Texas A&M University, College Station, TX, USA; BOV Center for Remote Health Technologies & Systems, Texas A&M University, College Station, TX, USA; BOW Department of Health Systems and Policy, University of Gondar, Gondar, Ethiopia; BOX Department of Anatomy and Embryology, Carol Davila University of Medicine and Pharmacy, Bucharest, Romania; BOY Department of Cardiology, Cardio-Aid, Bucharest, Romania; BOZ Department of General Surgery, Carol Davila University of Medicine and Pharmacy, Bucharest, Romania; BPA Department of General Surgery, Emergency University Hospital of Bucharest, Bucharest, Romania; BPB Health Promotion Research Center, Zahedan University of Medical Sciences, Zahedan, Iran; BPC Clinical Medicine (General Medicine profile), I.M. Sechenov First Moscow State Medical University, Moscow, Russia; BPD Department of Community Medicine, Lumbini Medical College, Palpa, Nepal; BPE School of Nursing, University of Gondar, Gondar, Ethiopia; BPF College of Medicine and Health Sciences, Bahir Dar University, Bahir Dar, Ethiopia; BPG Department of Psychiatry, University of Oxford, Oxford, UK; BPH Department of Neurosciences, Kenya Medical Research Institute/Wellcome Trust Research Programme, Kilifi, Kenya; BPI Department of Public Health, University of Yaoundé I, Yaoundé, Cameroon; BPJ Department of Biological Sciences, University of Embu, Embu, Kenya; BPK Hitotsubashi Institute for Advanced Study (HIAS), Hitotsubashi University, Tokyo, Japan; BPL Institute for Cancer Control, National Cancer Center, Chuo-ku, Japan; BPM Tuberculosis Group, Oxford University Clinical Research Unit, Vietnam, Ho Chi Minh City, Viet Nam; BPN Department of General Medicine, University of Medicine and Pharmacy at Ho Chi Minh City, Ho Chi Minh City, Viet Nam; BPO Harvard T.H. Chan School of Public Health, Harvard University, Cambridge, MA, USA; BPP Department of Medical Engineering, University of South Florida, Tampa, FL, USA; BPQ Faculty of Public Health, VNU University of Medicine and Pharmacy, Hanoi, Viet Nam; BPR International Institute for Training and Research (INSTAR), VNU University of Medicine and Pharmacy, Hanoi, Viet Nam; BPS Nam Can Tho Health Science Institute, Nam Can Tho University, Can Tho, Viet Nam; BPT Department of Pediatrics, New York Medical College, New York, NY, USA; BPU Institute for Global Health Innovations, Duy Tan University, Hanoi, Viet Nam; BPV Department of Public Health, University of Bamenda, Bamenda, Cameroon; BPW International Islamic University Islamabad, Islamabad, Pakistan; BPX Department of Humanities and Social Science, University for International Studies in Rome, Rome, Italy; BPY School of Medicine, University of Limerick, Limerick, Ireland; BPZ Department of Public Health, UNICAF, Larnaca, Cyprus; BQA Department of Pathology, Hawassa University, Hawassa, Ethiopia; BQB Department of Internal Medicine and Specialties, University of Yaoundé I, Yaounde, Cameroon; BQC Technical Department, University of Cape Town, Cape Town, South Africa; BQD School of Public Health and Family Medicine, University of Cape Town, Cape Town, South Africa; BQE Global Research Institute, Keio University, Tokyo, Japan; BQF University Institute of Diet and Nutritional Sciences, The University of Lahore, LAHORE, Pakistan; BQG School of Biomedical Engineering, Science and Health Systems, Drexel University, Philadelphia, PA, USA; BQH Division of Cardiology, University of California San Francisco, San Francisco, CA, USA; BQI Internal Medicine Department, Maimonides Medical Center, Brooklyn, NY, USA; BQJ Department of Paediatrics, Nnamdi Azikiwe University, Awka, Nigeria; BQK Global Health Department, Euclid University, Banqui, Central African Republic; BQL Division of Cardiology, University of Cape Town, Cape Town, South Africa; BQM The Cardiac Clinic, Groote Schuur Hospital, Cape Town, South Africa; BQN School of Information, University of California Berkeley, Berkeley, CA, USA; BQO Department of Public Health, Mattu University, Mattu, Ethiopia; BQP Midwifery Department, Poltekkes Kemenkes Palu, Palu, Indonesia; BQQ Department of Public Health, Banten School of Health Science, South Tangerang, Indonesia; BQR Ministry of Research, Technology and Higher Education, Higher Education Service Institutions (LL-DIKTI) Region IV, Bandung, Indonesia; BQS Department of Nursing, University of Health and Allied Sciences, Ho, Ghana; BQT Department of Physiology, University of Benin, Edo, Nigeria; BQU Department of Physiology, Benson Idahosa University, Benin City, Nigeria; BQV Department of Applied Economics and Quantitative Analysis, University of Bucharest, Bucharest, Romania; BQW Bioinformatics Department, National Institute of Research and Development for Biological Sciences, Bucharest, Romania; BQX Department of Biomedicine and Prevention, University of Rome “Tor Vergata”, Rome, Italy; BQY Department of Veterinary Public Health and Preventive Medicine, University of Ilorin, Ilorin, Nigeria; BQZ Department of Community Health and Primary Care, University of Lagos, Idi Araba, Nigeria; BRA Department of Family and Preventive Medicine, University of Utah, Salt Lake City, UT, USA; BRB Department of Population and Health, University of Cape Coast, Cape Coast, Ghana; BRC PSSM Data Sciences, Pfizer Research & Development, Pfizer Inc., Groton, CT, USA; BRD Department of Pediatrics, University of Jos, Jos, Nigeria; BRE Department of Pediatrics, Jos University Teaching Hospital, Jos, Nigeria; BRF Department of Physiology, Babcock University, Ilisan-Remo, Nigeria; BRG Technical Unit, Malaria Consortium, London, UK; BRH Department of Preventive Medicine, University of Ulsan, Seoul, South Korea; BRI Institute for Global Engagement & Empowerment, Yonsei University, Seoul, South Korea; BRJ Westmead Applied Research Center, University of Sydney, Sydney, NSW, Australia; BRK Centre for Social Research in Health, University of New South Wales, Sydney, NSW, Australia; BRL University of Sydney, Sydney, NSW, Australia; BRM Counselling and Human Development Studies, University of Ibadan, Ibadan, Nigeria; BRN Faculty of Medicine, University of Thessaly, Volos, Greece; BRO Department of Medical Laboratory Science, Federal Neuropsychiatric Hospital, Abeokuta, Nigeria; BRP School of Pharmacy, University of the Western Cape, Cape Town, South Africa; BRQ College of Health Sciences, Bowen University, Iwo, Nigeria; BRR College of Medicine, University of Ibadan, Ibadan, Nigeria; BRS Department of Psychiatry and Behavioural Neurosciences, McMaster University, Hamilton, ON, Canada; BRT Department of Psychiatry, University of Lagos, Lagos, Nigeria; BRU Department of Neurology, University College Hospital, Ibadan, Ibadan, Nigeria; BRV Department of Medicine, University of Ibadan, Ibadan, Nigeria; BRW Department of Nursing Science, Bowen University Iwo, Iwo, Nigeria; BRX Cumming School of Medicine, University of Calgary, Calgary, AB, Canada; BRY Center for Clinical and Epidemiological Research, University of São Paulo, São Paulo, Brazil; BRZ Associação Brasileira de Cefaleia em Salvas e Enxaqueca (ABRACES), São Paulo, Brazil; BSA Cardiology Department, Federal University of Rio de Janeiro, Rio de Janeiro, Brazil; BSB School of Health and Life Sciences, Teesside University, Middlesbrough, UK; BSC Research Policy & Administration, Centre for Healthy Start Initiative, Lagos, Nigeria; BSD Executive Director, Centre for Healthy Start Initiative, Lagos, Nigeria; BSE Surgery Department, Sulaimani University, Sulaimani, Iraq; BSF ENT Department, Tor Vergata University of Rome, Rome, Italy; BSG Black Dog Institute, University of New South Wales, Sydney, NSW, Australia; BSH Department of Global Health and Social Medicine, Harvard University, Boston, MA, USA; BSI Department of Pharmacology and Therapeutics, University of Nigeria Nsukka, Enugu, Nigeria; BSJ Institute of Diagnostic and Interventional Radiology and Neuroradiology, University Hospital Essen, Essen, Germany; BSK Scientific laboratory “Center for Collective Use”, Kazakh National Medical University, Almaty, Kazakhstan; BSL Department of Pharmacotherapy and Pharmaceutical Care, Medical University of Warsaw, Warsaw, Poland; BSM Department of Microbiology and Immunology, University of Health and Allied Sciences, Ho, Ghana; BSN Sickle Cell Unit, Ho Teaching Hospital, Ho, Ghana; BSO Department of Biotechnological and Applied Clinical Sciences, University of L'Aquila, L'Aquila, Italy; BSP Department of Neurology, ASL Avezzano-Sulmona-L'Aquila, L'Aquila, Italy; BSQ Department of Neurosurgery, University of California San Francisco, San Francisco, CA, USA; BSR Department of Nephrology and Hypertension, IIS-Fundacion Jimenez Diaz, Madrid, Spain; BSS Department of Medicine, Autonomous University of Madrid, Madrid, Spain; BST One Health Global Research Group, Universidad de las Americas (University of the Americas), Quito, Ecuador; BSU Department of Biological Sciences, Njala University, Freetown, Sierra Leone; BSV Cardiovascular Division, Harvard University, Boston, MA, USA; BSW School of Medicine, Western Sydney University, Bathurst, NSW, Australia; BSX Department of Optometry and Vision Science, University of KwaZulu-Natal, KwaZulu-Natal, South Africa; BSY Department of Biological Sciences, Elizade University, Ilara-Mokin, Nigeria; BSZ Department of Preventive and Social Medicine, University of Otago, Dunedin, New Zealand; BTA Faculty of Nursing, Applied Science Private University, Amman, Jordan; BTB School of Public Health, Texila American University, Georgetown, Guyana; BTC School of Public Health, Haramaya University, Harar, Ethiopia; BTD School of Nursing, University of California San Francisco, San Francisco, CA, USA; BTE Faculty of Medicine, University Ferhat Abbas of Setif, Sétif, Algeria; BTF Division of Infectious Diseases, University Hospital of Setif, Sétif, Algeria; BTG Department of Medicine, University College Hospital, Ibadan, Ibadan, Nigeria; BTH West African Center for Cell Biology of Infectious Pathogens, University of Ghana, Legon, Ghana; BTI Department of Biochemistry and Nutrition, Nigerian Institute of Medical Research, Lagos, Nigeria; BTJ Division of Medicine, University College London, London, UK; BTK Department of Biosciences and Biotechnology, University of Medical Sciences, Ondo, Ondo, Nigeria; BTL Operational Research Center in Healthcare, Near East University, Nicosia, Turkiye; BTM Department of Mathematical Sciences, Saveetha School of Engineering, SIMATS, Chennai, India; BTN Department of Respiratory Medicine, Jagadguru Sri Shivarathreeswara University, Mysore, India; BTO National School of Public Health, Institute of Health Carlos III, Madrid, Spain; BTP Department of Forensic Medicine and Toxicology, Manipal Academy of Higher Education, Mangalore, India; BTQ Department of Medical Mycology and Parasitology, Shiraz University of Medical Sciences, Shiraz, Iran; BTR Department of Primary Care and Public Health, Imperial College London, London, UK; BTS Department of Mental Health, Hospital Universitari Vall d'Hebron (CIBERSAM), Barcelona, Spain; BTT Biomedical Network Research Centre on Mental Health (CIBERSAM), Barcelona, Spain; BTU Primary Health Center, Directorate of Public Health and Family Welfare, Eluru district, India; BTV Menzies Institute for Medical Research, University of Tasmania, Hobart, TAS, Australia; BTW Centre for Biotechnology, Siksha ‘O’ Anusandhan (Deemed to be University), Bhubaneswar, India; BTX Department of Ophthalmology, Heidelberg University, Heidelberg, Germany; BTY Centre for Research and Development, Chandigarh University, Punjab, India; BTZ Division of Research and Development, Lovely Professional University, Phagwara, India; BUA Department of Clinical Epidemiology, University of the Philippines Manila, Manila, Philippines; BUB Ministry of Health, Jakarta, Indonesia; BUC Division of Ophthalmology & Visual Sciences, University of Nottingham, Nottingham, UK; BUD First Department of Ophthalmology, Aristotle University of Thessaloniki, Thessaloniki, Greece; BUE Department of Neurology, University of Bern, Biel/Biene, Switzerland; BUF Department of Neurology, University of Cyprus, Nicosia, Cyprus; BUG Department of Emergency Medicine, University of Thessaly, Larissa, Greece; BUH Department of Emergency Medicine, University of Bern, Bern, Switzerland; BUI Department of Diabetes, Nutrition and Metabolic Diseases, Carol Davila University of Medicine and Pharmacy, Bucharest, Romania; BUJ Unit of Dermatology, IRCCS Ospedale San Raffaele, Milano, Italy; BUK Department of Medicine and Surgery, University of Bologna, Bologna, Italy; BUL Medical University of Vienna, Vienna, Austria; BUM Department of Science and Mathematics, Deree-The American College of Greece, Athens, Greece; BUN Department of Biophysics, University of Athens, Athens, Greece; BUO Ottawa Hospital Research Institute, Ottawa, ON, Canada; BUP Digestive Diseases Research Center, Tehran University of Medical Sciences, Tehran, Iran; BUQ Cardiac Research Center, Tehran University of Medical Sciences, Tehran, Iran; BUR Division of Health Policy and Management, University of Minnesota, Minneapolis, MN, USA; BUS Department of Sociology, Anthropology, and Public Health, University of Maryland, Baltimore County, Baltimore, MD, USA; BUT Department of Medical Humanities and Social Medicine, Kosin University, Busan, South Korea; BUU Department of Biomedical Data Science, Stanford University, Stanford, CA, USA; BUV Department of Psychiatry, All India Institute of Medical Sciences, Bhubaneswar, India; BUW Department of Primary Care and General Practice, Kazan State Medical University, Kazan, Russia; BUX Department of Cardiology, Parve Nursing Home, Sindkhed Raja, India; BUY Department of Medical Sciences, University of Torino, Torino, Italy; BUZ Department of Imaging, AOU Città della Salute e della Scienza di Torino (AOU City of Health and Science of Turin), Torino, Italy; BVA Faculty of Medicine and Health, University of Leeds, Leeds, UK; BVB Centre for Biotechnology, Siksha ‘O’ Anusandhan Deemed to be University, Bhubaneswar, India; BVC Research and Development Cell, Parul University, Vadodara, India; BVD Department of Research and Training, Population Council Institute, New Delhi, India; BVE Department of Physiotherapy, Charotar University of Science and Technology, Anand, India; BVF Institute of Physiotherapy, Ashok and Rita Patel Institute of Physiotherapy, Anand, India; BVG Roswell Park Comprehensive Cancer Center, The State University of New York at Buffalo, Buffalo, NY, USA; BVH Department of Cardiovascular Medicine, University of Tennessee, Nashville, TN, USA; BVI School of Medicine, University of Sinu, Cartagena, Colombia; BVJ Mahatma Gandhi Institute of Medical Sciences, Sevagram, Maharashtra University of Health Sciences, Wardha, India; BVK College of Dental Medicine, Roseman University of Health Sciences, South Jordan, UT, USA; BVL Department of Physiology, All India Institute of Medical Sciences, Nagpur, India; BVM Department of Human Anatomy, All India Institute of Medical Sciences, Bathinda, India; BVN Department of Internal Medicine, Advent Health, Palm Coast, FL, USA; BVO Department of Hospital Medicine, Sound Physicians, Palm Coast, FL, USA; BVP Department of Interventional Cardiology, Cedars Sinai Medical Center, Los Angeles, CA, USA; BVQ Department of Genetics, Yale University, New Haven, CT, USA; BVR Physiology Research Center, Iran University of Medical Sciences, Tehran, Iran; BVS Department of Physiology, Iran University of Medical Sciences, Tehran, Iran; BVT School of Population Health, University of New South Wales, Kensington, NSW, Australia; BVU IRCCS Fondazione Don Carlo Gnocchi, Milan, Italy; BVV Department of Clinical and Experimental Sciences, University of Brescia, Brescia, Italy; BVW Center for Research and Innovation, Ateneo De Manila University, Pasig City, Philippines; BVX Australian Institute of Health Innovation, Macquarie University, Sydney, NSW, Australia; BVY School of Global Public Health, New York University, New York, NY, USA; BVZ Research Institute for Medicines, Universidade de Lisboa (University of Lisbon), Lisbon, Portugal; BWA School of Population Health, Curtin University, Bentley, WA, Australia; BWB Centre for Fertility and Health, Norwegian Institute of Public Health, Oslo, Norway; BWC Department of Applied Nursing, Federal University of Minas Gerais, Belo Horizonte, Brazil; BWD Faculty of Medicine, Autonomous University of Madrid, Madrid, Spain; BWE Social and Economic Survey Research Institute (SESRI), Qatar University, Doha, Qatar; BWF Mario Negri Institute for Pharmacological Research, Bergamo, Italy; BWG Department of Biochemistry and Pharmacology, Uzhhorod National University, Uzhhorod, Ukraine; BWH Mathematical and Computer Sciences, University of Medical Sciences, Ondo, Ondo, Nigeria; BWI Facultad de Medicina (Faculty of Medicine), Universidad Diego Portales (Diego Portales University), Santiago, Chile; BWJ School of Cardiovascular and Metabolic Health, University of Glasgow, Glasgow, UK; BWK Department of Internal Medicine, University of Arizona, Tucson, AZ, USA; BWL Department of Cardiovascular Medicine, Mayo Clinic, Rochester, MN, USA; BWM Department of Internal Medicine, Weiss Memorial Hospital, Chicago, IL, USA; BWN Department of Integrative Biotechnology, Sungkyunkwan University, Suwon, South Korea; BWO School of Pharmacy, University of Nizwa, Nizwa, Oman; BWP Shanghai Mental Health Center, Shanghai Jiao Tong University, Shanghai, China; BWQ Departments of Psychiatry and Epidemiology, Columbia University, New York, NY, USA; BWR Department of Epidemiology, Hiroshima University, Hiroshima, Japan; BWS International Center of Medical Sciences Research, International Center of Medical Sciences Research, Islamabad, Pakistan; BWT Riphah International University, Islamabad, Pakistan; BWU Department of Promoting Health, Maternal-Infant, Excellence and Internal and Specialized Medicine (PROMISE) G. D´Alessandro, University of Palermo, Palermo, Italy; BWV Department of Bioinformatics, Tehran University of Medical Sciences, Tehran, Iran; BWW Air and Climate Unit, European Commission, Ispra, Italy; BWX School of Economics and Public Policy, University of Adelaide, Adelaide, SA, Australia; BWY Mental Health Research Institute, Tomsk National Research Medical Center, Tomsk, Russia; BWZ Siberian State Medical University, Tomsk, Russia; BXA College of Health Sciences (CHS), VinUniversity, Hanoi, Viet Nam; BXB Department of Epidemiology and Evidence-Based Medicine, I.M. Sechenov First Moscow State Medical University, Moscow, Russia; BXC Department of Data Management and Analysis, The INCLEN Trust International, New Delhi, India; BXD Department of Ortopedics and Traumatology, University of Tampere, Tampere, Finland; BXE Management Department, Bucharest University of Economic Studies, Bucharest, Romania; BXF Academy of Romanian Scientists, Bucharest, Romania; BXG Department of Internal Medicine, University of Novi Sad, Novi Sad, Serbia; BXH Clinic for Endocrinology, Diabetes and Metabolic Disorders, Clinical Center of Vojvodina, Novi Sad, Serbia; BXI Department of Occupational Health and Safety Engineering, Shiraz University of Medical Sciences, Shiraz, Iran; BXJ Department of Physiology and Biomedical Engineering, Mayo Clinic, Rochester, MN, USA; BXK Non-communicable Diseases Research Center, Bam University of Medical Sciences, Bam, Iran; BXL Centro de Investigaciones Clinicas (Clinical Research Center), Fundación Valle del Lili (Valle del Lili Foundation), Cali, Colombia; BXM Centro PROESA, Universidad ICESI, Cali, Colombia; BXN Department of Community Medicine and Public Health, Tribhuvan University, Kathmandu, Nepal; BXO T.H. Chan School of Public Health, Harvard University, Boston, MA, USA; BXP Department of Humanities and Social Sciences, National Institute of Technology Rourkela, Rourkela, India; BXQ Department of Biochemistry, JSS Academy of Higher Education and Research, Mysuru, India; BXR Centre for Dental Education and Research, All India Institute of Medical Sciences, New Delhi, India; BXS Department of Clinical and Experimental Medicine, University of Pisa, Pisa, Italy; BXT Department of Biology, Universitas Airlangga (Airlangga University), Surabaya, Indonesia; BXU Institute for Health Research, University of Bedfordshire, Luton, UK; BXV Department of Biostatistics, Epidemiology, and Informatics, University of Pennsylvania, Philadelphia, PA, USA; BXW Department of Medical instrumentation Techniques Engineering, Al-Rafidain University College, Baghdad, Iraq; BXX Department of Cybersecurity, Kyiv National University of Construction and Architecture, Kyiv, Ukraine; BXY School of Public Health, (Xuzhou Medical University), Xuzhou, China; BXZ Rory Meyers College of Nursing, New York University, New York, NY, USA; BYA Department of Cardiovascular Medicine, Guangdong Cardiovascular Institute, Guangdong Provincial People's Hospital, Guangzhou, China; BYB Department of Respiratory and Critical Care Medicine, The First Affiliated Hospital, and College of Clinical Medicine of Henan University of Science and Technology, Luoyang, China; BYC Global Consortium for Public Health and Research, Jawaharlal Nehru Medical College, Wardha, India; BYD Research Center for Public Health and Nutrition, National Research and Innovation Agency of Indonesia, Jakarta, Indonesia; BYE Manipal College of Dental Sciences, Manipal Academy of Higher Education, Manipal, India; BYF Oman Dental College, Oman; BYG Department of Epidemiology and Biostatistics, Shiraz University of Medical Sciences, Shiraz, Iran; BYH UO Neurologia, Salute Pubblica e Disabilità (The Neurology, Public Health and Disability Unit), Fondazione IRCCS Istituto Neurologico Carlo Besta (IRCCS Foundation Carlo Besta Neurological Institute), Milan, Italy; BYI Department of Epidemiology, National Institute of Mental Health and Neurosciences, Bengaluru, India; BYJ Department of Environmental Health Engineering, Torbat Heydariyeh University of Medical Sciences, Torbat Heydariyeh, Iran; BYK Health Science Research Centre, Torbat Heydariyeh University of Medical Sciences, Torbat Heydariyeh, Iran; BYL Institute of Health and Wellbeing, Federation University Australia, Berwick, VIC, Australia; BYM Department of Epidemiology, Institute of Epidemiology, Disease Control and Research (IEDCR), Dhaka, Bangladesh; BYN Department of Pathobiology and Population Sciences (PPS), Royal Veterinary College (RVC), London, UK; BYO Faculty of Health Sciences, Qaiwan International University, Sulaymaniyah, Iraq; BYP College of Science, University of Sulaimani, Sulaymaniyah, KRG, Iraq; BYQ Collaboration for Cancer Outcomes Research and Evaluation (CCORE), University of New South Wales, Liverpool, NSW, Australia; BYR School of Medicine and Public Health, University of Sydney, Wollongong, NSW, Australia; BYS College of Medicine and Health Sciences, National University of Science and Technology, Sohar, Oman; BYT Future Technology Research Center, National Yunlin University of Science and Technology, Yunlin, Taiwan; BYU Health Service Research and Quality of Life Center (CEReSS), Aix-Marseille University, Marseille, France; BYV Faculty of Medicine, University of Setif Algeria, Sétif, Algeria; BYW LIRSSEI Research Lab, University of Setif Algeria, Sétif, Algeria; BYX Department of Medical, Surgical and Experimental Sciences, University of Sassari, Sassari, Italy; BYY Gynecology and Breast Care Center, Mater Olbia Hospital, Olbia, Italy; BYZ Division of Gynecology and Human Reproduction Physiopathology, IRCCS Azienda Ospedaliero-Universitaria di Bologna, Bologna, Italy; BZA Dr. Rajendra Prasad Government Medical College, Tanda, Kangra, India; BZB Kasturba Medical College Manipal, Manipal Academy of Higher Education, Manipal, India; BZC Department of Cardiology, Dow University of Health Sciences, Karachi, Pakistan; BZD Department of Medicine, Dow University of Health Sciences, Karachi, Pakistan; BZE Department of Infectious Diseases and Tropical Medicine, Tehran University of Medical Sciences, Tehran, Iran; BZF Emergency Medicine Department, Sri Manakula Vinayagar Medical College and Hospital, Puducherry, India; BZG Department of Cardiovascular Medicine, Cleveland Clinic, Cleveland, OH, USA; BZH Physiology Research Center, Kerman University of Medical Sciences, Kerman, Iran; BZI Department of Clinical Sciences, University of Sharjah, Sharjah, United Arab Emirates; BZJ Department of Cardiology, Mansoura University, Mansoura, Egypt; BZK Department of Population Health, King Saud bin Abdulaziz University for Health Sciences, Jeddah, Saudi Arabia; BZL Department of Midwifery, Ministry of Health of the Republic of Indonesia, Palu, Indonesia; BZM Department of Anatomy, Govt. Siddhartha Medical College, Vijayawada, India; BZN Department of Radiology, Stanford University, Stanford, CA, USA; BZO School of Nursing & Health Sciences, Hong Kong Metropolitan University, Hong Kong, China; BZP Saw Swee Hock School of Public Health, National University of Singapore, Singapore, Singapore; BZQ Biological Science and Bioengineering, Inha University, Incheon, South Korea; BZR South Asian Institute for Social Transformation (SAIST), Dhaka, Bangladesh; BZS Department of Epidemiology, Biostatistics and Occupational Health, McGill University, Montreal, QC, Canada; BZT Department of Research, Eastern Scientific LLC, Richmond, KY, USA; BZU Planetary Health Research Centre (PHRC), Kathmandu, Nepal; BZV Centre for Clinical Pharmacology, University of Defence in Belgrade, Belgrade, Serbia; BZW Centre for Clinical Pharmacology, Medical College of Georgia at Augusta University, Belgrade, Serbia; BZX Department of Forensic Medicine and Toxicology, Jagadguru Sri Shivarathreeswara University, Mysore, India; BZY Department of Oral Medicine and Radiology, Nitte (deemed to be) University, Mangalore, India; BZZ Kasturba Medical College Mangalore, Manipal Academy of Higher Education, Manipal, India; CAA Institute of Collective Health, Federal University of Bahia, Salvador, Brazil; CAB Barcelona Institute for Global Health, Barcelona, Spain; CAC Iranian Research Center on Aging, University of Social Welfare and Rehabilitation Sciences, Tehran, Iran; CAD Department of Immunology, Shahid Beheshti University of Medical Sciences, Tehran, Iran; CAE Department of Geography, Soran University, Soran, Iraq; CAF Department of Family Medicine, Rajarata University of Sri Lanka, Anuradhapura, Sri Lanka; CAG University of Swabi, University of swabi, Swabi, Pakistan; CAH Department of Global Health Policy, University of Tokyo, Tokyo, Japan; CAI Department of Neurosurgery, Helsinki University Hospital, Helsinki, Finland; CAJ The National Institute for Stroke and Applied Neurosciences, Auckland University of Technology, Auckland, New Zealand; CAK Department of Health Services Management, Shiraz University of Medical Sciences, Shiraz, Iran; CAL Department of Psychiatry, St. John's National Academy of Health Sciences, Bangalore, India; CAM Inovus Medical, St Helens, UK; CAN Department of Computer Science, Boston University, Boston, MA, USA; CAO Department of Mathematical Demography & Statistics, International Institute for Population Sciences, Mumbai, India; CAP Department of Hematology, North Khorasan University of Medical Sciences, Bojnurd, Iran; CAQ Departmant of Hematology, Tarbiat Modares University, Tehran, Iran; CAR Department of Internal Medicine, Northwest Health, Porter, Valparaiso, IN, USA; CAS Department of Biological Sciences, King Abdulaziz University, Jeddah, Egypt; CAT Department of Protein Research, Research and Academic Institution, Alexandria, Egypt; CAU Institute for Health, Health Care Policy and Aging Research, Rutgers University, New Brunswick, NJ, USA; CAV The School of Pharmaceutical Sciences, University of Science Malaysia, Penang, Malaysia; CAW Department for Epidemiology and Biostatistics, National Institute for Health Development, Tallinn, Estonia; CAX Department of Health Information Management, Manipal Academy of Higher Education, Manipal, India; CAY Department of Obstetrics and Gynecology, Azienda Sanitaria Universitaria Friuli Centrale, Udine, Italy; CAZ School of Environment, Tehran University, Tehran, Iran; CBA Department of Obstetrics and Gynecology - Infertility Clinic, Isfahan University of Medical Sciences, Isfahan, Iran; CBB Department of Epidemiology and Biostatistics, Rafsanjan University of Medical Sciences, Rafsanjan, Iran; CBC Department of Public Health Sciences, University of Connecticut, Farmington, CT, USA; CBD Department of Psychiatry, Yale University, New Haven, CT, USA; CBE Department of Internal Medicine, Federal University of Minas Gerais, Belo Horizonte, Brazil; CBF Centre of Telehealth, Federal University of Minas Gerais, Belo Horizonte, Brazil; CBG Escola de Enfermagem da UFMG, Federal University of Minas Gerais, Belo Horizonte, Brazil; CBH Department of Surgery, University of Minnesota, Minneapolis, MN, USA; CBI Department of Surgery, University Teaching Hospital of Kigali, Kigali, Rwanda; CBJ Community Health Department, Federal University of Ceará, Fortaleza, Brazil; CBK Faculty of Medicine, University of Porto, Porto, Portugal; CBL Faculty of Medicine, Fundacion Universitaria Autonoma de las Americas, Pereira, Colombia; CBM Faculty of Health Sciences, Universidad Cientifica del Sur, Lima, Peru; CBN Department of Clinical Research, University of Sao Paulo, Ribeirão Preto, Brazil; CBO Gilbert and Rose-Marie Chagoury School of Medicine, Lebanese American University, Beirut, Lebanon; CBP Department of Community Medicine, Government Medical College, Chandigarh, India; CBQ Centre for Healthy Brain Ageing (CHeBA), University of New South Wales, Sydney, NSW, Australia; CBR Department of Environmental and Radiological Health Sciences, Colorado State University, Fort Collins, CO, USA; CBS Department of Anesthesiology, University of Nebraska Medical Center, Omaha, NE, USA; CBT Department of Neurosciences, Maurizio Bufalini Hospital, Cesena, Italy; CBU Centre for Global Epilepsy, University of Oxford, Oxford, UK; CBV Action Research Bangladesh, Dhaka, Bangladesh; CBW Department of Ophthalmology and Visual Sciences, University of Wisconsin-Madison, Madison, WI, USA; CBX School of Medicine, Gonabad University of Medical Sciences, Gonabad, Iran; CBY Department of Pharmacy Services, Alberta Health Services, Edmonton, AB, Canada; CBZ West African Postgraduate College of Pharmacists, Lagos, Nigeria; CCA Department of Analytical and Applied Economics, Utkal University, Bhubaneswar, India; CCB RUSA Centre of Excellence in Public Policy and Governance, Utkal University, Bhubaneswar, India; CCC Department of Ophthalmology, University of Miami, Miami, FL, USA; CCD Family and Prevention Medicine, Isfahan University of Medical Sciences, Isfahan, Iran; CCE Isfahan University of Medical Sciences, Islamic Azad University, Isfahan, Iran; CCF Department of Community Medicine, RVM Medical College and Research Centre, Hyderabad, Hyderabad, India; CCG Achutha Menon Centre for Health Science Studies, Sree Chitra Tirunal Institute for Medical Sciences and Technology, Thiruvananthapuram, India; CCH Department of Biochemistry and Food Analysis, Patuakhali Science and Technology University, Patuakhali, Bangladesh; CCI Department of Labour, Government of West Bengal, Kolkata, India; CCJ Department of Veterinary Microbiology, College of Veterinary Science and Animal Husbandry, Agartala, India; CCK Faculty of Medicine, Quest International University Perak, Ipoh, Malaysia; CCL Department of Epidemiology, Florida International University, Miami, FL, USA; CCM Research Department, Indian Institute of Public Health, Delhi, India; CCN Department of Biochemistry, Saveetha University, Chennai, India; CCO Departamento de Ciencias Básicas Médicas, Universidad ICESI, Cali, Colombia; CCP Colombia; CCQ Department of Health Statistics, National Institute for Medical Research, Dar es Salaam, Tanzania; CCR Department of Cardiology, SS. Annunziata Hospital - ASL2 Abruzzo, Chieti, Italy; CCS Department of Internal Medicine, University of Botswana, Gaborone, Botswana; CCT Department of Medicine, St. John's National Academy of Health Sciences, Bangalore, India; CCU Department of Oral and Maxillofacial Surgery, Jagadguru Sri Shivarathreeswara University, Mysore, India; CCV Cardiovascular Department, Zagazig University, Zagazig, Egypt; CCW Faculty of Medicine, Gonabad University of Medical Sciences, Gonabad, Iran; CCX Infectious Diseases Research Center, Gonabad University of Medical Sciences, Gonabad, Iran; CCY Department Infectious Diseases, National Institute of Health, Rome, Italy; CCZ Department for Health Prevention, Ministry of Health, Rome, Italy; CDA Department of Medical Physics, Isfahan University of Medical Sciences, Isfahan, Iran; CDB Department of Medical Pharmacology, Cairo University, Giza, Egypt; CDC Department of Epidemiology, Shahid Beheshti University of Medical Sciences, Tehran, Iran; CDD School of Psychiatry, University of New South Wales, Sydney, NSW, Australia; CDE Neuropsychiatric Institute, Prince of Wales Hospital, Randwick, NSW, Australia; CDF Escuela de Kinesiología, Diego Portales University, Santiago de Chile, Chile; CDG Universidad Autónoma de Chile, Santiago de Chile, Chile; CDH school of public health, Shahid Beheshti University of Medical Sciences, Tehran, Iran; CDI Department of Public Health and Epidemiology, Khalifa University, Abu Dhabi, United Arab Emirates; CDJ Geospatial Health and Development Team, Telethon Kids Institute, Perth, WA, Australia; CDK Department of Food Technology, Salahaddin University, Erbil, Iraq; CDL Department of Computer, University of Science and Culture, Tehran, Iran; CDM Department of Biostatistics, Shiraz University of Medical Sciences, Shiraz, Iran; CDN Research Center for Environmental Determinants of Health, Kermanshah University of Medical Sciences, Kermanshah, Iran; CDO Iranian Research Center for Evidence-based Medicine, Tabriz University of Medical Sciences, Tabriz, Iran; CDP International Center of Medical Sciences Research, Islamabad, Pakistan; CDQ Department of Nursing and Midwifery, Saveh University of Medical Sciences, Saveh, Iran; CDR Department of Health, Shahid Beheshti University of Medical Sciences, Tehran, Iran; CDS Department of Neurology, Christian-Doppler University Hospital, Salzburg, Austria; CDT Spinal Cord Injury and Tissue Regeneration Center Salzburg (SCI-TReCS), Paracelsus Medical University, Salzburg, Austria; CDU Faculty of Medicine, Bioscience and Nursing, MAHSA University, Selangor, Malaysia; CDV Interdisciplinary Research Centre in Biomedical Materials (IRCBM), COMSATS Institute of Information Technology, Lahore, Pakistan; CDW Department of Psychiatry, All India Institute of Medical Sciences, New Delhi, India; CDX Faculty of Medicine, Shahid Beheshti University of Medical Sciences, Tehran, Iran; CDY Research Center for Immunodeficiencies, Tehran University of Medical Sciences, Tehran, Iran; CDZ Department of Psychosocial Science, University of Bergen, Bergen, Norway; CEA ICMR - National Institute for Research in Bacterial Infections, Indian Council of Medical Research, Kolkata, India; CEB Sharjah Institute of Medical Sciences, University of Sharjah, Sharjah, United Arab Emirates; CEC Center for Global Health Research, Saveetha University, Chennai, India; CED Biotechnology Research Center, Mashhad University of Medical Sciences, Mashhad, Iran; CEE Department of Public Health, Madda Walabu University, Bale Robe, Ethiopia; CEF Research Centre for Public Health, Equity and Human Flourishing, Torrens University Australia, Adelaide, SA, Australia; CEG Faculty of Health Sciences, Çankiri Karatekin University, Çankiri, Turkiye; CEH Department of Psychiatry, Ministry of Health, Manama, Bahrain; CEI College of Pharmacy, Al-Hadba University, Mosul, Iraq; CEJ Department of Health and Kinesiology, University of Illinois, Urbana-Champaign, IL, USA; CEK Department of Statistics, University of Gujrat, Gujrat, Pakistan; CEL Department of Integrated Health Education, Federal University of Espirito Santo, Vitória, Brazil; CEM Faculty of Pharmacy, Mansoura University, Mansoura, Egypt; CEN Department of Endocrinology, Mayo Clinic, Rochester, MN, USA; CEO Student Research Committee, Kashan University of Medical Sciences, Kashan, Iran; CEP Technology Management Department, University College of Applied Sciences, Gaza, Palestine; CEQ School of Economics and Management, University of Kassel, Kassel, Germany; CER Public Health and Community Medicine Department, Cairo University, Giza, Egypt; CES College of Nursing, Jouf University, Jouf, Saudi Arabia; CET Department of Research and Development, Tehran University of Medical Sciences, Tehran, Iran; CEU Department of Pathology, Microbiology and Forensic Medicine, The University of Jordan, Amman, Jordan; CEV Department of Clinical Laboratories and Forensic Medicine, The University of Jordan, Amman, Jordan; CEW Drug Applied Research Center, Tabriz University of Medical Sciences, Tabriz, Iran; CEX Department of Community Medicine, King Abdulaziz University, Jeddah, Saudi Arabia; CEY Institute of Epidemiology and Preventive Medicine, National Taiwan University, Taipei, Taiwan; CEZ Benang Merah Research Center, Benang Merah Research Center (BMRC), Minahasa Utara, Indonesia; CFA Department of Entomology, Ain Shams University, Cairo, Egypt; CFB Medical Ain Shams Research Institute (MASRI), Ain Shams University, Cairo, Egypt; CFC Department of Forensic Biology, Government Institute of Forensic Science Chhatrapati Sambhajinagar, Chhatrapati Sambhajinagar Maharashtra, India; CFD Epidemiology and Biostatistics, Tehran University of Medical Sciences, Tehran, Iran; CFE Department of Microbiology, Saveetha University, Chennai, India; CFF University of São Paulo City, São Paulo, Brazil; CFG School of Public Health and Health Management, University of Belgrade, Belgrade, Serbia; CFH Independent Consultant, Thiruvananthapuram, India; CFI Department of Health, Physical Education and Recreation, University of Cape Coast, Cape Coast, Ghana; CFJ Department of Public Health, Jahrom University of Medical Sciences, Jahrom, Iran; CFK Department of Food Processing Technology, West Bengal State Council of Technical Education, Malda, India; CFL Bodoland Univeisty, Botany Department, Kokrajhar, India; CFM Faculty of Science, Queensland University of Technology, Brisbane, QLD, Australia; CFN Health Sciences Research Center, Torbat Heydariyeh University of Medical Sciences, Torbat Heydariyeh, Razavi Khorasan Province, Iran; CFO Department of Oral Pathology and Microbiology, Dr. D. Y. Patil Vidyapeeth, Pune (Deemed to be University), Pune, India; CFP Faculty of Medicine, The University of Queensland, Brisbane, QLD, Australia; CFQ Colorectal Research Center, Iran University of Medical Sciences, Tehran, Iran; CFR Faculty of Health & Social Sciences, Bournemouth University, Bournemouth, UK; CFS Department of Epidemiology, National Institute for Research in Tuberculosis, Chennai, India; CFT UGC Centre of Advanced Study in Psychology, Utkal University, Bhubaneswar, India; CFU Udyam-Global Association for Sustainable Development, Bhubaneswar, India; CFV Department of Medical Informatics, Kagawa University, Miki-cho, Japan; CFW Food Processing and Nutrition, Karnataka State Akkamahadevi Women's University, Vijayapura, India; CFX Precision Medicine Department, Università degli studi della Campania Luigi Vanvitelli (University of Campania Luigi Vanvitelli), Naples, Italy; CFY School of Computing, Clemson University, Clemson, SC, USA; CFZ Department of Public Health Sciences, University of North Carolina at Charlotte, Charlotte, NC, USA; CGA Department of Preventive and Social Medicine, Jawaharlal Institute of Postgraduate Medical Education and Research, Puducherry, India; CGB Department of Post-Harvest Technology and Marketing, Patuakhali Science and Technology University, Patuakhali, Bangladesh; CGC Faculty of Business and Computing, University of the Fraser Valley, Abbotsford, BC, Canada; CGD Department of Finance, International School of Management, Paris, France; CGE Chief Data Officer Directorate, UK Department of Health and Social Care, London, UK; CGF The George Institute for Global Health, Sydney, NSW, Australia; CGG Stroke Unit and Neurology Unit, ASST Grande Ospedale Metropolitano Niguarda, Milan, Italy; CGH Department of Psychology, University of Alabama, Birmingham, AL, USA; CGI Clinic for Conservative Dentistry and Periodontology, University Hospital of the Ludwig-Maximilians-University Munich, Munich, Germany; CGJ Center for International Health, University of Bergen, Bergen, Norway; CGK Department of Medical Statistics, University of Zagreb, Zagreb, Croatia; CGL Department of Epidemiology and Prevention of Chronic Noncommunicable Diseases, Croatian Institute of Public Health, Zagreb, Croatia; CGM Faculty of Dentistry, University of Puthisastra, Phnom Penh, Cambodia; CGN Dr. D. Y. Patil Dental College & Hospital, Dr. D. Y. Patil Vidyapeeth, Pune (Deemed to be University), Pune, India; CGO Department of Clinical Pharmacy, University of Gondar, Gondar, Ethiopia; CGP School of Pharmacy, Curtin University, Perth, WA, Australia; CGQ Emergency Department, Manian Medical Centre, Erode, India; CGR Center for Health Systems Research, National Institute of Public Health, Cuernavaca, Mexico; CGS Department of Medicine, Swami Vivekanand Subharti University, Meerut, India; CGT National Heart, Lung, and Blood Institute, National Institutes of Health, Rockville, MD, USA; CGU Division of Population Medicine, Cardiff University, Cardiff, UK; CGV Department of Neurology, Tehran University of Medical Sciences, Tehran, Iran; CGW Department of Radiology, Northwestern University, Chicago, IL, USA; CGX Dongguan Key Laboratory of Computer-Aided Drug Design, Guangdong Medical University, Dongguan, China; CGY State Key Laboratories of Chemical Resources Engineering, Beijing University Of Chemical Technology, Beijing, China; CGZ School of Health Sciences, Universiti Sains Malaysia, Kota Bharu, Malaysia; CHA H.E.J. Research Institute of Chemistry, University of Karachi, Karachi, Pakistan; CHB Department of Biotechnology, Quaid-i-Azam University Islamabad, Islamabad, Pakistan; CHC Department of Physics, The University of Lahore, Lahore, Pakistan; CHD Gastroenterology Unit, IRCCS, Castellana Grotte (Bari), Italy; CHE Department of Internal Medicine, Tehran University of Medical Sciences, Tehran, Iran; CHF Department of Chemistry, Institute for Advanced Studies in Basic Sciences (IASBS), Zanjan, Iran; CHG Center for Medical and Bio-Allied Health Sciences Research, Ajman University, Ajman, United Arab Emirates; CHH Independent Consultant, Karachi, Pakistan; CHI Department of Medicine, Liaquat University Of Medical and Health Sciences, Jamshoro, Pakistan; CHJ Noncommunicable Diseases Research Center, Neyshabur University of Medical Sciences, Neyshabur, Iran; CHK Neurology Department, Ain Shams University, Cairo, Egypt; CHL Department of Pathology and Laboratory Medicine, Northwell Health, New York, NY, USA; CHM Department of Pharmacology, All India Institute of Medical Sciences, Jodhpur, India; CHN School of Medicine, Alborz University of Medical Sciences, Karaj, Iran; CHO Centre For Interdisciplinary Research In Basic Sciences (CIRBSc), Jamia Millia Islamia, New Delhi, India; CHP Science Department, Kazakh National Medical University, Almaty, Kazakhstan; CHQ National University of Ireland - Galway, Galway, Ireland; CHR Columbia University, New York, NY, USA; CHS Health Education & Promotion, College of Nursing and Health Sciences, Jazan University, Jazan, Saudi Arabia; CHT Department of Radiation Oncology, All India Institute of Medical Sciences, New Delhi, India; CHU Department for Evidence-based Medicine and Evaluation, University for Continuing Education Krems, Krems, Austria; CHV Universidad Espíritu Santo, Samborondón, Ecuador; CHW Department of Social and Behavioral Health, University of Nevada Las Vegas, Las Vegas, NV, USA; CHX Department of University Institute of Biotechnology, Chandigarh University, Punjab, India; CHY Department of Biotechnology, Graphic Era (Deemed to be University), Dehradun, India; CHZ Department of Human Genetics and Molecular Medicine, Central University of Punjab, Bathinda, India; CIA Amity Institute of Biotechnology, Amity University Rajasthan, Rajasthan, India; CIB Department of Forensic Science, Shree Guru Gobind Singh Tricentenary University, Gurugram, India; CIC Institute of Forensic Science & Criminology, Panjab University, Chandigarh, India; CID Department of Engineering, Free University of Brussels, Brussels, Belgium; CIE Department of Physiology, Pharmacology, and Toxicology, An-Najah National University, Nablus, Palestine; CIF Centre for Medical Informatics, University of Edinburgh, Edinburgh, UK; CIG Division of General Internal Medicine, Harvard University, Boston, MA, USA; CIH K S Hegde Medical Academy, Nitte University, Mangalore, India; CII KS Hegde Medical Academy, Nitte University, Mangalore, India; CIJ Department of Medicine & Therapeutics, The Chinese University of Hong Kong, Hong Kong, China; CIK Department of Epidemiology and Health Statistics, Wenzhou Medical University, Wenzhou, China; CIL HIV/AIDS Prevention and Control, Amahara Regional Sate Health Bureau, Bahir Dar, Ethiopia; CIM Department of Public Health, Dambi Dollo University, Dembi Dollo, Ethiopia; CIN Department of Epidemiology, Jimma University, Jimma, Ethiopia; CIO Department of Pharmacology, Saint Paul's Hospital Millennium Medical College, Addis Ababa, Ethiopia; CIP Korea University, Seoul, South Korea; CIQ Finnish Institute of Occupational Health, Helsinki, Finland; CIR Cancer Research Center, Tehran University of Medical Sciences, Tehran, Iran; CIS Cancer Biology Research Center, Tehran University of Medical Sciences, Tehran, Iran; CIT Department of Medicine, Ladoke Akintola University, Ogbomoso, Nigeria; CIU Oulu Business School, University of Oulu, Oulu, Finland; CIV Martti Ahtisaari Institute, University of Oulu, Oulu, Finland; CIW Department of Experimental Research, Medical University Pleven, Pleven, Bulgaria; CIX Department of Genetics, Sofia University “St. Kliment Ohridiski”, Sofia, Bulgaria; CIY Department of Clinical Practice, Jazan University, Jazan, Saudi Arabia; CIZ Student Scientific Research Center, Tehran University of Medical Sciences, Tehran, Iran; CJA Center for Technology and Innovation in Cardiovascular Informatics, Iran University of Medical Sciences, Tehran, Iran; CJB Department of Medical-Surgical Nursing, Mazandaran University of Medical Sciences, Sari, Iran; CJC Department of Nursing and Health Sciences, Flinders University, Adelaide, SA, Australia; CJD Department of Research and Academics, Kathmandu Cancer Center, Bhaktapur, Nepal; CJE Person-Centered Research, Monash University, Box Hill, VIC, Australia; CJF Clinical Pharmacy and Pharmacy Practice, Usmanu Danfodiyo University, Sokoto, Sokoto, Nigeria; CJG Kenneth H. Cooper Institute, Texas Tech University Health Sciences Center, Dallas, TX, USA; CJH Advanced Materials Division, Mintek, Randburg, South Africa; CJI Department of Biotechnology, University of the Western Cape, Bellville, South Africa; CJJ Unit of Basic Medical Sciences, University of Khartoum, Khartoum, Sudan; CJK Department of Medical Microbiology and Infectious Diseases, Erasmus University, Rotterdam, Netherlands; CJL Galgotias Multidisciplinary Research & Development Cell, Galgotias University, Greater Noida, India; CJM Sport Physical Activity and Health Research & Innovation Center (SPRINT), Polytechnic Institute of Guarda, Guarda, Portugal; CJN RISE Health, University of Beira Interior, Covilhã, Portugal; CJO Department of Physical Education, Federal University of Santa Catarina, Florianópolis, Brazil; CJP Department of Law, Economics, Management and Quantitative Methods, University of Sannio, Benevento, Italy; CJQ WSB University in Gdansk, Gdansk, Poland; CJR Department of Laboratory Medicine, All India Institute of Medical Sciences, New Delhi, India; CJS School of Public Health & Zoonoses, Guru Angad Dev Veterinary & Animal Sciences University, Ludhiana, India; CJT School of Veterinary Science, University of Sydney, Sydney, NSW, Australia; CJU School of Medicine, Baylor College of Medicine, Houston, TX, USA; CJV Department of Medicine Service, US Department of Veterans Affairs (VA), Houston, TX, USA; CJW Department of Biochemistry, Central University of Punjab, Bathinda, India; CJX Department of Pharmacology, Government Medical College and Hospital, Chandigarh, India; CJY Faculty of Medicine and Health Sciences, Shree Guru Gobind Singh Tricentenary University, Gurugram, India; CJZ Department of Human Genetics, Punjabi University Patiala, Patiala, India; CKA Department of Computer Science & Engineering, Central University of Punjab, Bathinda, India; CKB Amity institute of Public Health and Hospital Administration, Amity University, Noida, India; CKC School of Pharmaceutical Sciences (Faculty of Pharmacy), IFTM University, Moradabad, India; CKD Department of Community Medicine, Veer Chandra Singh Garhwali Government Institute of Medical Science and Research, Srinagar Garhwal, India; CKE Institute of National Importance on Food Technology, National Institute of Food Technology Entrepreneurship and Management, Sonipat, India; CKF Research Department, Hamad Medical Corporation, Doha, Qatar; CKG Institute of Medical Sciences, Banaras Hindu University, Varanasi, India; CKH Department of Dentistry, All India Institute of Medical Sciences, Bhopal, India; CKI Department of Internal Medicine, University of Indonesia, Jakarta Pusat, Indonesia; CKJ Department of Internal Medicine, Dr. Cipto Mangunkusumo National Hospital, Jakarta Pusat, Indonesia; CKK Centre for Primary Health Care and Equity (CPHCE), University of New South Wales, Sydney, NSW, Australia; CKL Menzies Centre for Health Policy, University of Sydney, Sydney, NSW, Australia; CKM Department of Anesthesiology, New York Medical College, Passaic, NJ, USA; CKN Global and European Health Education and Study Institute, University of Georgia, Tbilisi, Georgia; CKO NCDC, National Center for Disease Control and Public Health, Tbilisi, Georgia; CKP Books Committee, Royal College of Psychiatrists, London, UK; CKQ Royal college of psychiatrists, London, UK; CKR Department of Infectious Diseases and Epidemiology, Pirogov Russian National Research Medical University, Moscow, Russia; CKS Division of Injury Prevention, The Bizzell Group, Atlanta, GA, USA; CKT Rollins School of Public Health, Emory University, Atlanta, GA, USA; CKU Department of Gastroenterology, Creighton University, Phoenix, AZ, USA; CKV Department of Development Studies, Daffodil International University, Dhaka, Bangladesh; CKW School of Medical Education and Learning Technologies, Shahid Beheshti University of Medical Sciences, Tehran, Iran; CKX Department of Infectious Diseases, Kharkiv National Medical University, Kharkiv, Ukraine; CKY Clinical Science Line, Ludwig Boltzmann Institute of Osteologie, Vienna, Austria; CKZ Department of Biochemistry, American University of Integrative Sciences, Bridgetown, Barbados; CLA Faculty of Public Health, Universitas Ahmad Dahlan, Yogyakarta, Indonesia; CLB Department of Medicinal Chemistry, University of Sharjah, Sharjah, United Arab Emirates; CLC Department of Endocrinology, Case Western Reserve University, Cleveland, OH, USA; CLD Independent Consultant, New Delhi, India; CLE School of Medicine, Babol University of Medical Sciences, Babol, Iran; CLF Hospital Universitario de La Princesa, Universidad Autónoma de Madrid (Autonomous University of Madrid), Madrid, Spain; CLG Centro de Investigación Biomédica en Red Enfermedades Respiratorias (CIBERES), Madrid, Spain; CLH Department of Public Health, Experimental and Forensic Medicine, University of Pavia, Pavia, Italy; CLI School of Primary and Allied Health Care, Monash University, Melbourne, VIC, Australia; CLJ Hull York Medical School, University of Hull, Hull City, UK; CLK 3rd Department of Cardiology, University of Athens, Athens, Greece; CLL Medical and Diagnostic Research Centre, University of Hail, Hail, Saudi Arabia; CLM Department of Neurology, University of Massachusetts Medical School, Worcester, MA, USA; CLN College of Health and Public Service, University of North Texas, Denton, TX, USA; CLO Department of Primary Care and Public Health, University of East Anglia, Norwich, UK; CLP Office for Health Improvement and Disparities, UK Department of Health and Social Care, London, UK; CLQ School of Public Health, University of Alberta, Edmonton, Alberta (AB), Canada; CLR Discipline of Physiotherapy, University of Technology Sydney, Sydney, NSW, Australia; CLS Department of Orthopaedics, Massachusetts General Hospital, Boston, MA, USA; CLT Department of Orthopaedics, Harvard University, Cambridge, MA, USA; CLU Research Department, Nepal Development Society, Kathmandu, Nepal; CLV School of Exercise and Nutrition Sciences, Deakin University, Melbourne, VIC, Australia; CLW Department of Medical Sciences, Sunway University, Subang Jaya, Malaysia; CLX Clinical Research Unit, Projahnmo Research Foundation, Dhaka, Bangladesh; CLY Praboromarajchanok Institute, Ministry of Public Health, Nonthaburi, Thailand; CLZ Department of Physiotherapy, Tishk International University, Erbil, Iraq; CMA Department of Community Medicine, Ahmadu Bello University, Kaduna State, Nigeria; CMB School of Life Sciences, Xiamen University, Xiamen, China; CMC Faculty of Health Science, Universitas Indonesia Maju, Jakarta, Indonesia; CMD Department of Life and Health Sciences, University of Nicosia, Nicosia, Cyprus; CME Institute of Integrated Intelligence and Systems, Griffith University, Brisbane, QLD, Australia; CMF Yusuf Hamied Department of Chemistry, University of Cambridge, Cambridgeshire, UK; CMG School of Clinical Medicine, Tsinghua University, Beijing, China; CMH Department of Neurology, Tsinghua University, Beijing, China; CMI The First Hospital of China Medical University, China Medical University, Shenyang, China; CMJ High-Quality Development Evaluation Research Institute, Nanjing University of Posts and Telecommunications, Nanjing, China; CMK Gandhi Medical College, Kaloji Narayana Rao University of Health Sciences (KNRUHS), Secunderabad, India; CML Department of Population Health Sciences, University College London, London, UK; CMM School of Population Health, Curtin University, Perth, VIC, Australia; CMN Department of Clinical Research and Development, LUXMED Group, Warsaw, Poland; CMO Collegium Medicum, John Paul II Catholic University of Lublin, Lublin, Poland; CMP Northwestern University, Chicago, IL, USA; CMQ Department of Pharmacology, All India Institute of Medical Sciences, Deoghar, India; CMR Department of Neurology, Neurocenter of Southern Switzerland (NSI), Lugano, Switzerland; CMS Department of Medicine, University of Valencia, Valencia, Spain; CMT Carlos III Health Institute, Biomedical Research Networking Center for Mental Health Network (CiberSAM), Madrid, Spain; CMU Department of Medical Informatics, Mashhad University of Medical Sciences, Mashhad, Iran; CMV Applied Biomedical Research Center, Mashhad University of Medical Sciences, Mashhad, Iran; CMW neurology department, Shahid Beheshti University of Medical Sciences, tehran, Iran; CMX Department of Medical Education, Shahid Beheshti University of Medical Sciences, Tehran, Iran; CMY Department of Health, Safety, and Environmental Management, Abadan School of Medical Sciences, Abadan, Iran; CMZ Saveetha Medical College and Hospital, Saveetha Institute of Medical and Technical Sciences, Chennai, India; CNA Division of Epidemiology, Tohoku University, Sendai, Japan; CNB School of Dentistry and Oral Health, Griffith University, Gold Coast, QLD, Australia; CNC Department of Physiotherapy, A.T. Still University, Azare, Nigeria; CND The Five Senses Health Institute, Iran University of Medical Sciences, Tehran, Iran; CNE Duhok Research Centre, University of Duhok, Duhok, Iraq; CNF Living Systems Institute, University of Exeter, Exeter, UK; CNG Department of Pathology, Alexandria University, Alexandria, Egypt; CNH Department of Dermato-Venereology, Dr. Victor Babes Clinical Hospital of Infectious Diseases and Tropical Diseases, Bucharest, Romania; CNI Department of Epidemiology, Stellenbosch University, Cape Town, South Africa; CNJ Department of Medicine, Northlands Medical Group, Omuthiya, Namibia; CNK Department of Surgery, National University of Singapore, Singapore, Singapore; CNL Department of Medicine, Kazakh National Medical University, Almaty, Kazakhstan; CNM State Key Laboratory of Numerical Modeling for Atmospheric Sciences and Geophysical Fluid Dynamics (LASG), Chinese Academy of Sciences, Beijing, China; CNN Department of Optometry, Eye Hospital of Shandong University of Traditional Chinese Medicine, Jinan, China; CNO Department of Computer and Software Engineering, National University of Science and Technology (NUST), Islamabad, Pakistan; CNP Department of Psychology, Montclair State University, Montclair, NJ, USA; CNQ National Research and Innovation Agency, Jakarta, Indonesia; CNR Department of Public Health, Debre Markos University, Debre Markos, Ethiopia; CNS Department of Pharmacology and Therapeutics, The University of Faisalabad, Faisalabad, Pakistan; CNT Department of Metabolism and Systems Science, University of Birmingham, Birmingham, UK; CNU Department of Public Health and Informatics, Bangladesh Medical University, Dhaka, Bangladesh; CNV Department of Pathology, Tehran University of Medical Sciences, Tehran, Iran; CNW Indiana University School of Medicine, University of Missouri, Indianapolis, IN, USA; CNX Research Chair for Evidence-Based Health Care and Knowledge Translation, King Saud University, Riyadh, Saudi Arabia; CNY College of Pharmacy, Alfaisal University, Riyadh, Saudi Arabia; CNZ Department of Preventive Medicine, Northwestern University, Chicago, IL, USA; COA Internal Medicine Department, King George's Medical University, Lucknow, India; COB Department of Abdominal Surgery, Katholieke Universiteit Leuven, Leuven, Belgium; COC Clinical Microbiology, Karnali Academy of Health Sciences (KAHS), Jumla, Nepal; COD Department of Economics, The American University in Cairo, Cairo, Egypt; COE Rheumatology and Immunology Unit, Mansoura University, Mansoura, Egypt; COF Department of Radiology, Kaduna State University, Kaduna, Nigeria; COG International Centre for Eye Health, London School of Hygiene & Tropical Medicine, London, UK; COH Department of Applied Bioscience, Konkuk University, Seoul, South Korea; COI Department of Conservative dentistry and Endodontics, Manipal Academy of Higher Education, Mangalore, India; COJ Faculty of Public Health, Universitas Sam Ratulangi (Sam Ratulangi University), Manado, Indonesia; COK ?.?. Timiryazev Institute of Plant Physiology, Russian Academy of Sciences, Moscow, Russia; COL Douala Laquintinie Hospital, Douala, Cameroon, University of Yaoundé I, Douala, Cameroon; COM Department of Biochemistry, All India Institute of Medical Sciences, Jodhpur, India; CON Department of Medicine, University of Calgary, Calgary, AB, Canada; COO Interdisciplinary Health Data Center, Jagiellonian University Medical College, Kraków, Poland; COP Merilyn and Glick Eye Institute, University of Indiana, Indianapolis, IN, USA; COQ Nutritional Epidemiology Research Team (EREN), National Institute for Health and Medical Research (INSERM), Paris, France; COR Department of Health, Medicine and Human Biology, Sorbonne Paris Nord University, Bobigny, France; COS High Institute of Sport and Physical Education of Sfax, University of Sfax, Sfax, Tunisia; COT Department of Movement Sciences and Sports Training, The University of Jordan, Amman, Jordan; COU Department of Internal Medicine, University of Medicine and Pharmacy at Ho Chi Minh City, Ho Chi Minh City, Viet Nam; COV Department of Business Analytics, University of Massachusetts Dartmouth, Dartmouth, MA, USA; COW Second Department of Internal Medicine, Kansai Medical University, Osaka, Japan; COX John T. Milliken Department of Medicine, Washington University in St. Louis, Saint Louis, MO, USA; COY School of Medicine and Dentistry, Griffith University, GoldCoast, QLD, Australia; COZ Molecular Neuroscience Research Center, Shiga University of Medical Science, Shiga, Japan; CPA ALS Vietnam Research and Advocacy Initiative, ALS Vietnam, Quang Ngai, Viet Nam; CPB Department of Health Sciences, University of Leicester, Leicester, UK; CPC Adult Learning Disability Service, Leicestershire Partnership National Health Service Trust, Leicester, UK; CPD CRIMEDIM Center for Research and Training in Global Health, Humanitarian Aid and Disaster Medicine, University of Eastern Piedmont, Novara, Italy; CPE Department of Primary Care, Geneva University Hospital, Geneva, Switzerland; CPF Department of Cardiology, Tianjin Medical University, Tianjin, China; CPG Kent and Medway Medical School, Kent and Medway Medical School, Canterbury, UK; CPH Department of Internal Medicine, Wake Forest University, Winston-Salem, NC, USA; CPI Department of Urology, The Second Hospital of Tianjin Medical University, Tianjin, China; CPJ Department of Epidemiology and Biostatistics, Haramaya University, Harar, Ethiopia; CPK Joslin Diabetes Center, Harvard University, Boston, MA, USA; CPL Department of Nutrition and Food Studies, George Mason University, Fairfax, VA, USA; CPM School of Nursing, Hong Kong Polytechnic University, Hong Kong, China; CPN Hayatabad Medical Complex, Postgraduate Medical Institute, Peshawar, KPK, Pakistan; CPO Department of Biology and Biochemistry, University of Houston, Houston, TX, USA; CPP Medical Genomics Research Department, King Abdullah International Medical Research Center, Riyadh, Saudi Arabia; CPQ Department of Life Sciences, University of Management and Technology, Lahore, Pakistan; CPR Federal University of Health Sciences Azare, Federal Teaching Hospital, Azare, Nigeria; CPS Federal Teaching Hospital Azare, Federal Medical Centre, Azare, Bauchi-State, Nigeria; CPT Lahore Business School, The University of Lahore, Lahore, Pakistan; CPU Department of Medicine, Khairpur Medical College, Khairpur, Pakistan; CPV Kasturba Medical College, Manipal Academy of Higher Education, Manipal, India; CPW Amity Institute of Biotechnology, Amity University Rajasthan, Jaipur, India; CPX Section of Advanced Heart Failure and Transplant Cardiology, Mayo Clinic, Rochester, MN, USA; CPY Harvard Kennedy School, Harvard University, Cambridge, MA, USA; CPZ Department of Orthodontics, University of Trakya, Edirne, Turkiye; CQA Johnson & Johnson, Duquesne University, Pittsburgh, PA, USA; CQB College of Health and Sport Sciences, University of Bahrain, Zallaq, Bahrain; CQC Sociedad Argentina de Medicina, Buenos Aires, Argentina; CQD Hospital Vélez Sarsfield, Buenos Aires, Argentina; CQE Dermatology Unit, IRCCS Humanitas Research Hospital, Milan, Italy; CQF Department of Psychology, Zayed University, Abu Dhabi, United Arab Emirates; CQG Faculty of Sciences, University of Guilan, Rasht, Iran; CQH College of Public Health and Tropical Medicine, Jazan University, Jazan, Saudi Arabia; CQI Department of Internal Medicine, University of Groningen, Groningen, Netherlands; CQJ Department of Nephrology, Christian Medical College and Hospital (CMC), Vellore, India; CQK UKK Institute, Tampere, Finland; CQL Faculty of Medicine and Health Technology, Tampere University, Tampere, Finland; CQM Department of Biochemistry, Apollo Institute of Medical Sciences and Research Chittoor, Chittoor, India; CQN Department of Otolaryngology Head and Neck Surgery, Louisiana State University Health Sciences Center, Shreveport, LA, USA; CQO Biomedical Engineering Department, University of Texas, Arlington, TX, USA; CQP Centre for Healthy Brain Ageing, University of New South Wales, Sydney, NSW, Australia; CQQ Department of Zoology, Central University of Punjab, Bathinda, India; CQR Department of Human Genetics & Molecular Biology, Bharathiar University, Coimbatore, India; CQS PG & Research Department of Chemistry, Auxilium College (Autonomous), Vellore, India; CQT Raffles Neuroscience Centre, Raffles Hospital, Singapore, Singapore; CQU Department of Community Medicine and Family Medicine, All India Institute of Medical Sciences, Bathinda, India; CQV Department of Surgery, University of Southampton, Southampton, UK; CQW College of Medicine and Veterinary Medicine, University of Edinburgh, Edinburgh, UK; CQX Department of Health Policy and Management, Johns Hopkins University, Baltimore, MD, USA; CQY Department of Pediatrics, Cornell University, Ithaca, NY, USA; CQZ Department of Biomedical Sciences for Health, University of Milan, Milano, Italy; CRA Department of Physiotherapy, Universidad Europea de Madrid (European University of Madrid), Villaviciosa de Odón, Spain; CRB Digital Health Research Center, Instituto Peruano de Orientación Psicológica, Lima, Peru; CRC Department of Biomedical Informatics, University of Utah, Salt Lake City, UT, USA; CRD Occupational Medicine Unit, Sant'Orsola Malpighi Hospital, Bologna, Italy; CRE Cardiac Electrophysiology, St Bernard's Medical Center, Jonesboro, AR, USA; CRF Faculty of Medicine of Itajubá, Brazil, Faculty of Medicine of Itajubá, Brazil, Itajubá, Brazil; CRG Diabetes Research Centre, University of Leicester, Leicester, UK; CRH Department of Biomedical Sciences, Arba Minch University, Arba Minch, Ethiopia; CRI Department of Medical Physiology, Addis Ababa University, Addis Ababa, Ethiopia; CRJ NUST School of Health Sciences, National University of Science and Technology (NUST), Islamabad, Pakistan; CRK Széchenyi István University, Gyor, Hungary; CRL Department of Social Sciences, Chuka University, Kenya, Nairobi, Kenya; CRM Population Studies and Research Institute, UoN, University of Nairobi, Nairobi, Kenya; CRN School of Chinese Medicine, Beijing University of Chinese Medicine, Beijing, China; CRO University of Chicago, Chicago, IL, USA; CRP Department of Paediatrics, All India Institute of Medical Sciences, Bathinda, India; CRQ Department of Neurosurgery, Capital Medical University, Beijing, China; CRR Department of Neurosurgery, Beijing Tiantan Hospital, Beijing, China; CRS Department of Health Services Research, Management and Policy, University of Florida, Gainesville, FL, USA; CRT College of Agriculture, Northwest A&F University, Xianyang City, China; CRU Department of Artificial Intelligence, Xiamen University, Xiamen, China; CRV Department of Rehabilitation, Southeast University, Nanjing, China; CRW School of Life Course and Population Sciences, King's College London, London, UK; CRX school of public health, Peking University, Beijing, China; CRY National Institute of Health Data Science, Peking University, Beijing, China; CRZ Department of Psychiatry, University of São Paulo, São Paulo, Brazil; CSA School of Medicine and Dentistry, Griffith University, Gold Coast, QLD, Australia; CSB School of Nursing Sciences, University of Nairobi, Nairobi, Kenya; CSC Faculty of Sciences, The University of Lahore, Lahore, Pakistan; CSD Key Laboratory of Computer-Aided Drug Design, Guangdong Medical University, Dongguan, China; CSE Centre for Health Policy Research, Adelaide, SA, Australia; CSF Department of Parasitology, Rajarata University of Sri Lanka, Anuradhapura, Sri Lanka; CSG Department of Orthopaedics, General Hospital of Central Theater Command, Wuhan, China; CSH Fourth Military Medical University, Xi'an, China; CSI Department of Geriatrics, The Eighth Affiliated Hospital of Sun Yat-sen University, Shenzhen, China; CSJ Cardiology Department, Royal Children's Hospital, Melbourne, VIC, Australia; CSK Department of Critical Care and Neurosciences, Murdoch Childrens Research Institute, Parkville, VIC, Australia; CSL Competence Center of Mortality-Follow-Up of the German National Cohort, Federal Institute for Population Research, Wiesbaden, Germany; CSM Department of Physical Therapy, Naresuan University, Phitsanulok, Thailand; CSN Department of Experimental Pharmacology, Heidelberg University, Mannheim, Germany; CSO Department of Medical Surgical Nursing, Gadjah Mada University, Yogyakarta, Indonesia; CSP Department of Community Medicine, Rajarata University of Sri Lanka, Anuradhapura, Sri Lanka; CSQ Department of Surgery, University of Colombo, Colombo, Sri Lanka; CSR Department of Nursing, Universitas Aisyiyah Bandung, Bandung, Indonesia; CSS Institute of Clinical Epidemiology, Medical University Innsbruck, Innsbruck, Austria; CST Research Organisation, Inter-Continental Omni-Research in Medicine Collaborative, Berlin, Germany; CSU School of Public Health, University of Technology Sydney, Sydney, New South Wales (NSW), Australia; CSV School of Public Health, Debre Markos University, Debre Markos, Ethiopia; CSW Cochrane South Africa, South African Medical Research Council, Cape Town, South Africa; CSX Department of Global Health, Stellenbosch University, Cape Town, South Africa; CSY Department of Public Health, Samara University, Samara, Ethiopia; CSZ Department of Research, Cancer Registry of Norway, Oslo, Norway; CTA Department of Chemical Toxicology, Norwegian Institute of Public Health, Oslo, Norway; CTB Institute of Health and Care Sciences, University of Gothenburg, Gothenburg, Sweden; CTC Faculty of Health Sciences, Oslo Metropolitan University, Oslo, Norway; CTD Faculty of Health, University of Technology Sydney, Australia, NSW, Australia; CTE School of Pharmacy, Monash University, Subang Jaya, Malaysia; CTF Department of Theory and Empiricism of Healthcare, Universität Kassel, Kassel, Germany; CTG Ethiopia; CTH Department of Pharmacy, University of Gondar, Gondar, Ethiopia; CTI Division of Hematology and Oncology, Medical College of Wisconsin, Milwaukee, WI, USA; CTJ Division of Gastroenterology, Huazhong University of Science and Technology, Wuhan, China; CTK Department of Public Health, Wuhan fourth hospital, Wuhan, China; CTL Department of Food Science and Human Nutrition, Michigan State University, East Lansing, MI, USA; CTM Shenzhen Institute of Advanced Technology, Chinese Academy of Sciences, Shenzhen, China; CTN Department of Emergency Medicine and Critical Care Nursing, Bahir Dar University, Bahir Dar, Ethiopia; CTO Australian Centre for Health Services Innovation, Queensland University of Technology, Brisbane, QLD, Australia; CTP Western Institute of Digital-Intelligent Medicine, Chongqing Medical University, Chongqing, China; CTQ School of Public Health, Zhejiang University, Zhejiang, China; CTR Department of Public Health Science, Fred Hutchinson Cancer Research Center, Seattle, WA, USA; CTS Tongji Medical College, Huazhong University of Science and Technology, Wuhan, China; CTT School of Nursing and Rehabilitation, Shandong University, Jinan, China; CTU Department of Intelligent Medical Engineering, Anhui Medical University, Anhui, China; CTV Department of Surgery, The First Affiliated Hospital of Anhui Medical University, Hefei, Anhui, China; CTW Department of Nutrition, Tufts University, Boston, MA, USA; CTX Department of Social and Behavioral Sciences, Harvard University, Boston, MA, USA; CTY Cardiovascular Program, The George Institute for Global Health, Sydney, NSW, Australia; CTZ Department of Endocrinology, University of Science and Technology of China, Hefei, China; CUA School of Medicine, University of Rochester, Rochester, NY, USA; CUB School of Public Health, Southwest Medical University, luzhou, China; CUC Ruijin Hospital, Shanghai Jiao Tong University, Shanghai, China; CUD School of Medicine, Kunming University of Science and Technology, Kunming, China; CUE Department of Environmental Health and Epidemiology, National Institute for Research in Environmental Health, Bhopal, India; CUF Department of Basic Medical Sciences, Neyshabur University of Medical Sciences, Neyshabur, Iran; CUG Department of Community Medicine, Apollo Institute of Medical Sciences and Research, hyderabad, India; CUH Department of Public Health, Juntendo University, Tokyo, Japan; CUI Department of Public Health Medicine, University of Tsukuba, Tsukuba, Japan; CUJ Department of Public Health Administration, Linyi People's Hospital, Linyi, China; CUK Faculty of Medicine, Juntendo University, Tokyo, Japan; CUL School of Traditional Chinese Medicine, Beijing University of Chinese Medicine, Beijing, China; CUM Pritzker School of Medicine, University of Chicago, Chicago, IL, USA; CUN School of Public Health and Primary Care, The Chinese University of Hong Kong, Hong Kong, China; CUO Department of Medicine, Thomas Jefferson University, Philadelphia, PA, USA; CUP Department of Medicine, Mashhad University of Medical Sciences, Mashhad, Iran; CUQ Research Center of Physiology, Semnan University of Medical Sciences, Semnan, Iran; CUR College of Pharmacy and Health Science, Ajman University, Ajman, United Arab Emirates; CUS Hematology Section, Hamad Medical Corporation, Doha, Qatar; CUT Department of Biostatistics and Data Science, The University of Osaka, Suita, Japan; CUU National Center for Chronic and Noncommunicable Disease Control and Prevention, Chinese Center for Disease Control and Prevention, Beijing, China; CUV The George Institute for Global Health, University of New South Wales, Sydney, NSW, Australia; CUW School of Biotechnology, University of Tehran, Tehran, Iran; CUX Department of Public Health, Trakya University, Edirne, Turkiye; CUY Medical Biotechnology Research Center, Guilan University of Medical Sciences, Rasht, Iran; CUZ Department of Epidemiology and Biostatistics, Debre Tabor University, Debre Tabor, Ethiopia; CVA Department of Family Medicine, St. Paul's Hospital Millennium Medical College, Addis Ababa, Ethiopia; CVB Family Medicine Department, St. Peter's Specialized Hospital, Addis Ababa, Ethiopia; CVC Biostatics, Epidemiology, and Science Computing Department, King Faisal Specialist Hospital & Research Center, Riyadh, Saudi Arabia; CVD KHANA Center for Population Health Research, Phnom Penh, Cambodia; CVE Public Health Department (Public health nutrition unit), Dire Dawa University, Dire Dawa Administration, Ethiopia; CVF Department of Epidemiology, Xuzhou Medical University, Xuzhou, China; CVG Department of Pharmacology, Bahir Dar University, Bahir Dar, Ethiopia; CVH Pharmacy Department, Alkan Health Science, Business and Technology College, Bahir Dar, Ethiopia; CVI Department of Pharmacy, Bahir Dar University, Bahir Dar, Ethiopia; CVJ Department of Pediatrics, Kyung Hee University, Seoul, South Korea; CVK Department of Biostatistics, University of Toyama, Toyama, Japan; CVL Department of Health Policy and Management, Jackson State University, Jackson, MS, USA; CVM School of Business & Economics, Universiti Putra Malaysia (University of Putra Malaysia), Kuala Lumpur, Malaysia; CVN Department of Public Health, Jigjiga University, Jigjiga, Ethiopia; CVO Sichuan Provincial Center for Mental Health, University of Electronic Science and Technology of China, Chengdu, China; CVP Key Laboratory of Psychosomatic Medicine, Chinese Academy of Medical Sciences, Chengdu, China; CVQ School of Public Health, Hubei University of Medicine, Shiyan, China; CVR Department of Epidemiology and Biostatistics, Wuhan University, Wuhan, China; CVS Southeast University Affiliated Xuzhou Central Hospital, Clinical Hospital, Xuzhou, China; CVT Department of Basic Science, University of Hail, Hail, Saudi Arabia; CVU Department of Nursing Science, Bayero University, Kano, Nigeria; CVV Faculty of Nursing, University of Alberta, Edmonton, AB, Nigeria; CVW Association for Socially Applicable Research (ASAR), Pune, India; CVX Department of Emergency Medicine, Global Emergency Medicine Innovation and Implementation (GEMINI) Research Center, Durham, NC, USA; CVY Epidemiology and Cancer Registry Sector, Institute of Oncology Ljubljana, Ljubljana, Slovenia; CVZ Family and Community Medicine Department, University of Hail, Hail, Saudi Arabia; CWA Islamic Azad University, Tehran, Iran; CWB Biology & Emerging Pathogens Institute, University of Florida, Gainesville, FL, USA; CWC Faculty of Medicine and Health Sciences, Universiti Putra Malaysia (Putra University of Malaysia), UPM Serdang, Malaysia; CWD Faculty of Medicine and Health Sciences, Hodeidah University, Hodeidah, Yemen; CWE Department of Health Sciences, James Madison University, Harrisonburg, VA, USA; CWF Department of Computer and Self Development, Prince Sattam bin Abdulaziz University, Al Kharj, Saudi Arabia; CWG The Heller School for Social Policy and Management, Brandeis University, Waltham, MA, USA; CWH Social Development and Health Promotion Research Center, Kermanshah University of Medical Sciences, Kermanshah, Iran; CWI Sant'Elia Hospital, University of Catania, Caltanissetta, Italy; CWJ Research and Development Department, Sina Medical Biochemistry Technologies, Shiraz, Iran; CWK Nursing Care Research Center in Chronic Diseases, Ahvaz Jundishapur University of Medical Sciences, Ahvaz, Iran; CWL Department of Dermatology, Shahid Beheshti University of Medical Sciences, Tehran, Iran; CWM Department of Bioengineering and Therapeutical Sciences, University of California San Francisco, San Francisco, CA, USA; CWN Department of Administration, PGxAI, San Francisco, CA, USA; CWO Department of Clinical Practice, Northern Border University, Rafha, Saudi Arabia; CWP Department of Public Health, University of Hail, Hail, Saudi Arabia; CWQ Cardiovascular Epidemiology Research Centre (CERC), University of Western Australia, Perth, WA, Australia; CWR Cardiology Population Health Laboratory, Victor Chang Cardiac Research Institute (VCCRI), Perth, WA, Australia; CWS Institute of Diagnostic and Interventional Radiology and Neuroradiology, University of Duisburg-Essen, Essen, Germany; CWT Department of Psychiatry, Johns Hopkins University, Baltimore, MD, USA; CWU Department of Surgery, University of Hong Kong, Hong Kong, China; CWV Department of Internal Medicine, Jacobi Medical Center, Bronx, NY, USA; CWW Department of Internal Medicine, Albert Einstein College of Medicine, Bronx, NY, USA; CWX Medical Oncology Department of Gastrointestinal Cancer, Cancer Hospital of Dalian University of Technology, Shenyang, China; CWY School of Biomedical Engineering, Dalian University of Technology, Dalian, China; CWZ School of Public Health, Peking University, Beijing, China; CXA Department of International Health, Johns Hopkins University, Baltimore, MD, USA; CXB School of Public Health, Wuhan University of Science and Technology, Wuhan, China; CXC Hubei Province Key Laboratory of Occupational Hazard Identification and Control, Wuhan University of Science and Technology, Wuhan, China; CXD Department of Cardiology, Zhongshan Hospital, Fudan University, Shanghai, China; CXE Department of Obstetrics and Gynecology, Frist Affiliated Hospital of Anhui Medical University, Hefei, China; CXF Burn Surgery Department, The First Hospital of Jilin University, Changchun, China; CXG Tianjin Medical University General Hospital, Tianjin Centers for Disease Control and Prevention, Tianjin, China; CXH XuZhou Medical University, University of Medicine, XuZhou, China; CXI Department of Health Management, Shengjing Hospital of China Medical University, Shenyang, China; CXJ Office of Chongqing Cancer Prevention and Treatment, Chongqing University Cancer Hospital, Chongqing, China; CXK Fuwai Hospital, Chinese Academy of Medical Sciences, Beijing, China; CXL Department of Hepatology, Wenzhou Medical University, wenzhou, China; CXM Harvard Medical School, Harvard University, Boston, MA, USA; CXN Jockey Club School of Public Health and Primary Care, The Chinese University of Hong Kong, Hong Kong, China; CXO School of Data Science, The Chinese University of Hong Kong, Shenzhen, Shenzhen, China; CXP School of Medicine, Stanford University, Palo Alto, CA, USA; CXQ School of Public Health and Emergency Management, Southern University of Science and Technology, Shenzhen, China; CXR Department of Epidemiology, University of Washington, Seattle, WA, USA; CXS Institute of Public Health and Social Sciences, Khyber Medical University, Peshawar, Pakistan; CXT Endocrinology and Metabolism Research Center, Hormozgan University of Medical Sciences, Bandar Abbas, Iran; CXU College of Nursing, Prince Sattam bin Abdulaziz University, Al-Kharj, Saudi Arabia; CXV Faculty of Nursing, Mansoura University, Mansoura, Egypt; CXW Department of Medical-Surgical Nursing, University of Hail, Hail, Saudi Arabia; CXX Applied Science Research Centre, Applied Science Private University, Amman, Jordan; CXY Department of Paediatrics and Child Health, University of Cape Town, Cape Town, South Africa; CXZ Department of Public Health, Universitas Brawijaya, Malang, Indonesia; CYA Center for Clinical Microbiology, University College London, London, UK; CYB NIHR-Biomedical Research Centre (NIHR-BRC), University College London Hospitals, London, UK; CYC Clinical Research Centre, An-Najah National University Hospital, Nablus, Palestine; CYD Department of Building Engineering and Environment, Palestine Technical University (Kadoorie), Tulkarem, Palestine; CYE Civil Engineering and Sustainable Structures, Palestine Technical University (Kadoorie), Tulkarem, Palestine; CYF Department of Chemistry, An-Najah National University, Nablus, Palestine

## Abstract

**Background:**

Comprehensive, comparable, and timely estimates of demographic metrics—including life expectancy and age-specific mortality—are essential for evaluating, understanding, and addressing trends in population health. The COVID-19 pandemic highlighted the importance of timely and all-cause mortality estimates for being able to respond to changing trends in health outcomes, showing a strong need for demographic analysis tools that can produce all-cause mortality estimates more rapidly with more readily available all-age vital registration (VR) data. The Global Burden of Diseases, Injuries, and Risk Factors Study (GBD) is an ongoing research effort that quantifies human health by estimating a range of epidemiological quantities of interest across time, age, sex, location, cause, and risk. This study—part of the latest GBD release, GBD 2023—aims to provide new and updated estimates of all-cause mortality and life expectancy for 1950 to 2023 using a novel statistical model that accounts for complex correlation structures in demographic data across age and time.

**Methods:**

We used 24 025 data sources from VR, sample registration, surveys, censuses, and other sources to estimate all-cause mortality for males, females, and all sexes combined across 25 age groups in 204 countries and territories as well as 660 subnational units in 20 countries and territories, for the years 1950–2023. For the first time, we used complete birth history data for ages 5–14 years, age-specific sibling history data for ages 15–49 years, and age-specific mortality data from Health and Demographic Surveillance Systems. We developed a single statistical model that incorporates both parametric and non-parametric methods, referred to as OneMod, to produce estimates of all-cause mortality for each age-sex-location group. OneMod includes two main steps: a detailed regression analysis with a generalised linear modelling tool that accounts for age-specific covariate effects such as the Socio-demographic Index (SDI) and a population attributable fraction (PAF) for all risk factors combined; and a non-parametric analysis of residuals using a multivariate kernel regression model that smooths across age and time to adaptably follow trends in the data without overfitting. We calibrated asymptotic uncertainty estimates using Pearson residuals to produce 95% uncertainty intervals (UIs) and corresponding 1000 draws. Life expectancy was calculated from age-specific mortality rates with standard demographic methods. For each measure, 95% UIs were calculated with the 25th and 975th ordered values from a 1000-draw posterior distribution.

**Findings:**

In 2023, 60·1 million (95% UI 59·0–61·1) deaths occurred globally, of which 4·67 million (4·59–4·75) were in children younger than 5 years. Due to considerable population growth and ageing since 1950, the number of annual deaths globally increased by 35·2% (32·2–38·4) over the 1950–2023 study period, during which the global age-standardised all-cause mortality rate declined by 66·6% (65·8–67·3). Trends in age-specific mortality rates between 2011 and 2023 varied by age group and location, with the largest decline in under-5 mortality occurring in east Asia (67·7% decrease); the largest increases in mortality for those aged 5–14 years, 25–29 years, and 30–39 years occurring in high-income North America (11·5%, 31·7%, and 49·9%, respectively); and the largest increases in mortality for those aged 15–19 years and 20–24 years occurring in Eastern Europe (53·9% and 40·1%, respectively). We also identified higher than previously estimated mortality rates in sub-Saharan Africa for all sexes combined aged 5–14 years (87·3% higher in GBD 2023 than GBD 2021 on average across countries and territories over the 1950–2021 period) and for females aged 15–29 years (61·2% higher), as well as lower than previously estimated mortality rates in sub-Saharan Africa for all sexes combined aged 50 years and older (13·2% lower), reflecting advances in our modelling approach. Global life expectancy followed three distinct trends over the study period. First, between 1950 and 2019, there were considerable improvements, from 51·2 (50·6–51·7) years for females and 47·9 (47·4–48·4) years for males in 1950 to 76·3 (76·2–76·4) years for females and 71·4 (71·3–71·5) years for males in 2019. Second, this period was followed by a decrease in life expectancy during the COVID-19 pandemic, to 74·7 (74·6–74·8) years for females and 69·3 (69·2–69·4) years for males in 2021. Finally, the world experienced a period of post-pandemic recovery in 2022 and 2023, wherein life expectancy generally returned to pre-pandemic (2019) levels in 2023 (76·3 [76·0–76·6] years for females and 71·5 [71·2–71·8] years for males). 194 (95·1%) of 204 countries and territories experienced at least partial post-pandemic recovery in age-standardised mortality rates by 2023, with 61·8% (126 of 204) recovering to or falling below pre-pandemic levels. There were several mortality trajectories during and following the pandemic across countries and territories. Long-term mortality trends also varied considerably between age groups and locations, demonstrating the diverse landscape of health outcomes globally.

**Interpretation:**

This analysis identified several key differences in mortality trends from previous estimates, including higher rates of adolescent mortality, higher rates of young adult mortality in females, and lower rates of mortality in older age groups in much of sub-Saharan Africa. The findings also highlight stark differences across countries and territories in the timing and scale of changes in all-cause mortality trends during and following the COVID-19 pandemic (2020–23). Our estimates of evolving trends in mortality and life expectancy across locations, ages, sexes, and SDI levels in recent years as well as over the entire 1950–2023 study period provide crucial information for governments, policy makers, and the public to ensure that health-care systems, economies, and societies are prepared to address the world's health needs, particularly in populations with higher rates of mortality than previously known. The estimates from this study provide a robust framework for GBD and a valuable foundation for policy development, implementation, and evaluation around the world.

**Funding:**

Gates Foundation.

## Introduction

Comprehensive, accurate, and timely estimates of mortality and other demographic indicators across locations, age groups, and sexes are necessary for effective public health planning and intervention. Such estimates provide insights into disparities across populations and time, enabling more targeted resource allocation, policy development, and monitoring of the effectiveness of different health systems and programmes. Timely and regularly updated estimates of mortality are also crucial for understanding and responding to rapidly changing health landscapes, such as during and immediately following pandemics and wars and in the aftermath of natural disasters. Understanding mortality trends over time also helps in assessing the broader impacts of social, economic, and environmental factors on health outcomes now and in the future.

Numerous sources produce estimates of mortality and other demographic indicators, including population and fertility. The UN Population Division of the Department of Economic and Social Affairs (UNPD) estimates and projections of global, regional, and national demographic metrics are updated biannually, most recently in the World Population Prospects 2024 revision.^1^ WHO released its latest all-cause mortality estimates in the World Health Statistics 2024 report^2^ and associated Global Health Estimates. The EU and the Organisation for Economic Co-operation and Development produce mortality estimates less regularly, and generally only for a subset of metrics and locations.^3^, ^4^ Many national statistics offices release demographic estimates for their own populations. The Global Burden of Diseases, Injuries, and Risk Factors Study (GBD) publishes peer-reviewed, regularly updated, comprehensive, and globally comparable estimates and forecasts of population health across a range of indicators, including all-cause mortality and life expectancy, for past and future years, from 1950 to 2100.^5^ The first GBD estimates—which included estimates of all-cause mortality—were published in the 1993 World Bank World Development Report, and mortality estimates have been published in every update since GBD 2010.^6^, ^7^, ^8^, ^9^, ^10^, ^11^, ^12^ Most recently, GBD 2021 produced estimates of all-cause mortality and excess mortality due to the COVID-19 pandemic using a novel, unified approach.^12^ Unlike the estimates of other research enterprises, GBD demographic estimates are informed by GBD estimates of disease and injury burden, and vice versa, making the GBD estimates of all-cause mortality and life expectancy in this study comparable and compatible with the latest GBD estimates of cause-specific mortality, healthy life expectancy, years of life lost, years lived with disability, disability-adjusted life-years, risk-attributable burden, population, and fertility.

Estimates of all-cause mortality have historically been produced with model life table systems to derive age-specific mortality rates in places without reliable vital registration (VR) data. A model life table system defines a range of relationships between levels of mortality at different ages. Coale and Demeny^13^ first developed regional model life tables in the 1960s. The Brass logit system further refined the methodology with a relational system that linked a standard life table to a population with a mathematical model of survivorship across ages.^14^ This methodology has been improved upon in various capacities.^15^, ^16^, ^17^ The UNPD uses a combination of various model life table systems to produce estimates.^18^ GBD 2021 also used a model life table system that relied on more than 10 000 empirically observed life tables.^12^ However, these systems all impose age patterns of mortality from observed locations onto locations with little or no data. The age patterns of mortality in locations with sparse data differ substantively from locations whose mortality profiles influence model life table systems. For example, the age patterns of mortality rates in older ages in populations throughout sub-Saharan Africa have been shown to be relatively lower than those in high-income countries from which age patterns are typically drawn in model life table systems.^19^, ^20^ This suggests the need for new methods to estimate age-specific mortality rates without relying on model life tables.

**Research in context**

**Evidence before this study**

The UN Population Division of the Department of Economic and Social Affairs (UNPD) produces estimates and projections of global, regional, and national demographic metrics that are updated biannually. Its latest findings, published in the World Population Prospects 2024 revision, incorporated estimates of excess mortality due to the COVID-19 pandemic from WHO and the World Mortality Database from 2021 as well as weekly or monthly death registration data for 2022 and 2023 from selected countries. WHO releases all-cause mortality estimates that differ from those of UNPD, most recently with the World Health Statistics 2024 report and associated Global Health Estimates. Some national statistics offices also produce their own demographic indicators. The Organisation for Economic Co-operation and Development and the EU, among others, release mortality estimates less regularly and typically only for selected metrics or locations. The Global Burden of Diseases, Injuries, and Risk Factors Study (GBD) generates regularly updated and globally comparable health metrics, including all-cause mortality and life expectancy, for past years, and, for certain metrics, forecasts up to the year 2100. The current GBD 2023 cycle is directly preceded by GBD 2021, which reported demographic estimates for 204 countries and territories and 811 subnational locations for each year from 1950 to 2021. Although each of these studies represents important efforts to provide insights into all-cause mortality estimates, only GBD demographic estimates are informed by and comparable to estimates of disease, injury, and risk factor burden; they are also the only estimates to comply with the GATHER statement, which identify best practices for reporting global health estimates.

**Added value of this study**

GBD 2023 developed a novel methodology to directly use age-specific demographic data in a single statistical model that accounts for complex correlation structures in demographic data across age and time. This model, OneMod, includes two primary components: a complex functional generalised linear model specification, and residual smoothing with a multivariate kernel regression model. This is a notable methodological improvement from GBD 2021 and UNPD approaches, which use multiple separate models for mortality indicators that are then input to model life table systems to estimate age patterns of mortality. The new model is simpler, more transparent, and facilitates the use of standard techniques for statistical inference and model assessment. Furthermore, our model uses covariates to capture mortality effects due to HIV/AIDS and the COVID-19 pandemic in locations with little to no data, rather than post-hoc incorporation of estimates from separate models. GBD 2023 used a suite of customised and validated data processing and modelling tools, systematically analysing thousands of data sources to produce global, regional, national, and subnational demographic estimates by age and sex for each year from 1950 to 2023. For the first time, GBD 2023 included data from complete birth histories for children and adolescents aged 5–14 years, used age-specific mortality data from sibling histories rather than a summary of the probability of death from those aged 15–60 years, and incorporated age-specific mortality data from 38 Health and Demographic Surveillance System sites. Compared to GBD 2021, GBD 2023 incorporated 3127 additional data sources, which includes 1211 location-years of provisional all-age vital registration data, which had not previously been used and which provide more timely information. All estimates are packaged within freely accessible data-sharing and visualisation tools.

**Implications of all the available evidence**

Our study shows higher than previously estimated adolescent mortality and young adult female mortality in much of sub-Saharan Africa, as well as lower old-age mortality in the same region. It also highlights a diversity of trends in all-cause mortality during the COVID-19 pandemic and recovery periods of 2020–23, with stark differences in the timing and extent of mortality fluctuations across countries. Globally comparable estimates show substantial variation between and within countries and territories, which allows analysis of key patterns that can be compared across regions. Additionally, our analyses of evolving long-term trends in mortality and life expectancy across age groups, sexes, and Socio-demographic Index levels reveal changing dynamics and patterns with implications for the future of health-care systems, economies, and societies. Collectively, the estimates reported here provide a robust framework for GBD and a valuable foundation for policy evaluation, development, and implementation around the world.

The GBD 2023 mortality analysis improves upon GBD 2021 by developing a novel methodology to incorporate age-specific demographic data directly into a single statistical model. This model accounts for complex correlation structures across age and time, leading to more accurate age-specific results across the study period. Using a single model to produce all-cause mortality estimates is a major improvement over previous approaches, including by GBD and UNPD, because it is simpler and more transparent, facilitates statistical model assessment with standard techniques, and does not rely on model life tables. In this iteration of GBD, we aimed to provide comprehensive estimates of age-specific all-cause mortality and life expectancy from 1950 to 2023, across global, regional, national, and subnational locations, using novel methods that will enable more timely and accurate mortality updates in the future. This manuscript was produced as part of the GBD Collaborator Network and in accordance with the GBD Protocol.^21^

## Methods

### Overview

For each new GBD round, the latest data and improved methods are used to update the full time series of demographic estimates from 1950 through to the latest year of analysis; GBD 2023 demographic estimates therefore supersede all previous estimates.

The GBD 2023 demographic analysis introduced a novel tool, referred to as OneMod, for estimating all-cause mortality that directly incorporates age-specific data in a single, stagewise statistical model and accounts for complex correlations over time and across age groups. OneMod is a notable methodological improvement over the mortality methods used by previous GBD iterations, the UNPD, and other groups, all of which use multiple separate models and model life table systems. The new model is simpler, using standard techniques for statistical model assessment, and allows for greater transparency. It also allows more flexible specification of inputs in order to incorporate data that were not usable in previous GBD models as well as more direct accounting of mortality due to HIV/AIDS and the COVID-19 pandemic by use of covariates, rather than incorporating separate models for these causes of death in a post-hoc manner. In contrast to previous methods that were reliant on model life table systems that estimate age patterns of mortality in data-sparse locations based on historical mortality data from a limited set of populations, OneMod models age-specific mortality rates directly from the available data such that patterns of mortality are based on data-driven correlations across age. The tool has several components that are unified within a single overarching model, including parametric methods that use covariates, intercepts, trends, and splines, as well as non-parametric fitting that uses kernel methods, which start from the parametric fit and create more detailed predictions by leveraging correlation across age, time, and location in the residuals corresponding to most detailed data. Correlations are encoded into the overall likelihood using kernels, giving rise to a useful smoothing penalty in prediction space that balances the detailed data. The overall likelihood connects all stages, with priors and offsets to transfer information from one stage to the next. Binomial likelihood is used throughout the model to estimate all-cause mortality.

OneMod analysis for this application includes two main components: regression analysis with a generalised linear modelling tool (referred to as SpXMod) that accounts for age-specific covariate effects, and non-parametric modelling of residuals with the Kronecker-factored multivariate kernel regression (KReg) model, which smooths across age and time. A summary of the methods used to produce all-cause mortality estimates is given below; a more detailed description of all GBD 2023 demographics methods—including for all-cause mortality as well as for fertility and population size—is available in appendix 1 (sections 2–6). An analytical flowchart of all-cause mortality estimation is also presented in appendix 1 (figure S1). We compared our estimates of mortality rates and life expectancy to those of UNICEF^22^ and the UNPD's latest World Population Prospects revision.^23^

### All-cause mortality data sources and processing

The GBD 2023 analysis used a range of data types for mortality estimation that were identified from a systematic search of available data from government websites, statistical annuals, demographic compendia, large-scale surveys, and collaborator input; citations and metadata for these input data are available online via the GBD 2023 Sources Tool in the Global Health Data Exchange (GHDx). All-cause mortality was estimated with 24 025 data sources, which included 28 652 location-years of VR data (3726 new location-years across all age groups compared to GBD 2021),^12^ 1644 location-years of sample VR data, and 5208 other sources (355 new surveys, 15 new censuses, and 2213 other new sources; appendix 1 tables S3–S5).

Methods to calculate age-specific mortality rates from each data source were similar to GBD 2021, with some notable exceptions (details provided in appendix 1 section 2.2). First, age-specific mortality rates for age groups younger than 15 years were calculated from complete birth histories rather than aggregated under-5 mortality rates. Data for ages 5–9 years and 10–14 years had previously only been obtained from VR data. Complete birth history data from these ages have been shown to be reliable.^24^ We also assessed the usability of complete birth history data from age groups 15–19 and 20–24 years, but these ages showed downward bias compared to VR and sibling survival history data as well as sparse availability (appendix 1 section 2.2.2). Second, we used a new age-sex splitting algorithm to calculate age-specific mortality rates from summary birth histories (appendix 1 sections 2.2.6 and 2.4). Third, we calculated age-specific mortality rates for ages 15–19 years to 45–49 years from sibling survival histories instead of the probability of death between ages 15 and 60 years (the 45q15 indicator). We assessed the usability of sibling survival history data from age groups 50–54 years and 55–59 years, but these age groups showed downward bias compared to VR data as well as sparse availability due to most surveys only seeking respondents aged 15–49 years (appendix 1 section 2.2.4). Additionally, we incorporated data on age-specific mortality rates from 38 International Network for the Demographic Evaluation of Populations and Their Health (INDEPTH) Health and Demographic Surveillance System (HDSS) sites across 15 countries, mostly in sub-Saharan Africa, totaling 532 location-years of data. Finally, we used a new age-sex splitting algorithm to split mortality rates from aggregated age groups in VR data into age-specific mortality for more granular age groups (appendix 1 section 2.2.6). This includes 8754 location-years of VR data with all-age death counts.

To account for non-sampling variation—which the available sample size does not account for, and which can be substantial depending on the data source and its completeness—we made several weight adjustments as described in appendix 1 (section 2.3.5.1). To ensure data were consistent with a binomial likelihood having parameters less than 1, which is particularly relevant for the neonatal and oldest age groups, we used compactification pre-processing techniques as described in appendix 1 (section 2.3.5.1). Complete methods on data processing are provided in appendix 1 (sections 2.2 and 2.3).

### All-cause mortality estimation

We developed the OneMod mortality pipeline to produce estimates of all-cause mortality across location, age, sex, and time using a single model with both parametric and non-parametric components linked within an overarching statistical framework. We used five covariates that are known to be predictive of all-cause mortality: Socio-demographic Index (SDI), HIV mortality rate, age-standardised COVID-19 mortality rate, a binary covariate indicating whether each location is an island, and an all-risk-factor summary covariate calculated as the total population attributable fraction (PAF) for all risk factors combined for age-standardised all-cause mortality, excluding deaths due to HIV/AIDS and deaths due to fatal discontinuities (ie, events that are stochastic in nature, and therefore do not have a predictable time trend). SDI is a composite indicator of a location's level of social and economic development, measured by lag-distributed income per capita, average years of schooling, and the total fertility rate in women aged younger than 25 years (appendix 1 sections 5 and 6.3–6.4). The first four covariates were available for all locations from 1950 to 2023, with HIV mortality rates being zero for the years before 1981 and COVID-19 mortality rates being zero for the years before 2020, while the fifth covariate was only available from 1990 onwards. Complete methods and covariate details, including an explanation of the covariate selection process, are provided in appendix 1 (section 2.3.5.2).

We then used the new SpXMod stage-wise model fit with a regression model with correlated, dimension-specific intercepts and covariate coefficients. We fit models for datasets from 1950 onwards and 1990 onwards using available covariate sets, with the all-risk covariate only available for datasets from 1990 onwards. In stage 1 of SpXMod, we fit a global age-specific covariate model, along with intercepts by age and location. In stage 2, we used the results from the global covariate model as a constant offset for our global time model and then fit super-region-specific and age-specific trends and intercepts. In stage 3, we used the results from the global time model as a constant offset in a national model, to which we added a country-specific and age-specific intercept. For countries with subnational locations, we used stage 4 to fit a subnational model using the results from the national model as a constant offset. A custom splicing step was used to combine estimates for models from 1950 onwards and 1990 onwards; see appendix 1 (sections 2.3.5.3 and 2.3.5.4) for full details and parameter values.

Next, we used the prediction from the combined model as an offset for a non-parametric kernel regression (implemented via the KReg tool), to smooth SpXMod residuals using similarity across different dimensions (eg, age, time, and location). This step captures additional signals in the data that are not explained by the covariates. See appendix 1 (section 2.3.5.5) for additional information and parameter values.

Finally, to mitigate the effects of the prior kernel smoothing matrix (which necessarily decreases the posterior variance), we used a calibration strategy for uncertainty. Once we obtained model-based asymptotic uncertainty for each estimate based on the model distributions, we picked a geographical granularity (either a specific location or region, depending on the data richness of the location) and scaled each set of uncertainties so that residuals corresponding to observations had a variance of 1. This stage scaled uncertainty estimates so that the scaled Pearson residuals for each geographical granularity have unit variance, which corresponds to the idea that prediction intervals should contain 95% of the observations, based on 1000 draws. See appendix 1 (section 2.3.5.6) for additional details. The age-specific mortality rates estimated from OneMod were used to generate life tables by single-year age groups, with further detailed age groups under the age of 1 year. Sex-redistributed and age-redistributed fatal discontinuities by cause were aggregated by age and sex and added to the estimated mortality from the previous step to generate the final all-cause mortality life tables by location, year, sex, and age. We calculated abridged life tables for the standard GBD age groups from these single-year age group life tables, including fatal discontinuities for each location, year, and sex combination. This culminated in estimating life expectancy at birth, using standard demographic methods.^25^ Finally, we calculated the final mortality envelope from these abridged life tables. See appendix 1 (section 2.5) for additional details.

### Expected mortality based on SDI estimation

There is a well established correlation between SDI and mortality, with higher SDI being associated with lower mortality rates. However, mortality is not driven solely by SDI. As such, for each location, we can estimate the mortality rate or life expectancy that would be expected solely on the basis of SDI and compare these estimates to our estimated mortality and life expectancy to identify locations that overperform or underperform on the basis of their level of social and demographic development. To do this, we analysed the relationship between age-specific log mortality rates and SDI using MR-BRT (meta-regression–Bayesian, regularised, trimmed),^26^ a meta-regression programme. MR-BRT defines a linear mixed-effects model with a B-spline specification for the relationship between outcomes of interest and SDI. We used a cubic spline with five knots between 0 and 1, with left-most and right-most spline segments enforced to be linear, and with slopes matching adjacent interior segments. To ensure that the results were not sensitive to the choice of spline knots, we used a model ensemble of more than 50 cubic spline models, as described above. For each model, interior knot placement was randomly generated to be between 0·1 and 0·9, with minimum inter-knot distance of 0·1 and maximum inter-knot distance of 1·0. The final predictions were obtained with the ensemble aggregate over these 50 models. This model was performed separately for each GBD age-sex group. Expected mortality rates for each age-sex group based on SDI were used to estimate expected life expectancy.

### GBD research and reporting practices

This research complies with the GATHER statement;^27^ a completed GATHER checklist is provided in appendix 1 (table S2). The University of Washington Institutional Review Board granted approval for the study (STUDY00009060) through to July 26, 2026. The software used for analyses included Python (version 3.10.4), Stata (version 15.1), and R (versions 4.2 and 4.4). The statistical code used in GBD 2023 is publicly available online. An international network of collaborators helped provide, review, and analyse the input data and estimates; GBD 2023 drew on the expertise of more than 14 000 collaborators from more than 160 countries and territories.

GBD 2023 produced estimates of all-cause mortality by age-sex-location-year for 25 age groups from early neonatal (0–6 days) to 95 years and older; for males, females, and all sexes combined; in 204 countries and territories grouped into 21 regions and seven super-regions; and for every year from 1950 to 2023. GBD regions were designated on the basis of two criteria: epidemiological similarity and geographical closeness of countries and territories. Super-regions were grouped on the basis of cause of death patterns among regions. See appendix 1 (table S1) for a complete list of locations by hierarchy level. GBD 2023 also includes subnational analyses for 660 locations in 20 countries and territories (Brazil, China, Ethiopia, India, Indonesia, Italy, Iran, Japan, Kenya, Mexico, New Zealand, Nigeria, Norway, Pakistan, the Philippines, Poland, Russia, South Africa, the UK, and the USA; due to space constraints, these estimates are available only in appendix 2 and the online tools, or in some cases they are not available publicly due to agreements with country partners) as well as estimates by SDI quintile. All countries and territories were given an SDI value ranging from 0 (lowest income and educational attainment, and highest fertility) to 100 and then most-specific locations (subnational locations in countries for which they are estimated, countries and territories for all others) were grouped into quintiles from low SDI to high SDI (appendix 2 tables S6A–C).

Uncertainty was propagated through each component of the estimation process. As detailed above, for all-cause mortality, OneMod generated 1000 draws for every location, year, and sex combination. Mean estimates and 95% uncertainty intervals (UIs; the 25th and 975th ranked values from the 1000 draws) for mortality and life expectancy were generated with the draw-level estimates.

Count data are presented to three significant figures and age-standardised rates are presented to 1 decimal place.

### Role of the funding source

The funder of this study had no role in study design, data collection, data analysis, data interpretation, the writing of the report, or the decision to submit the manuscript for publication.

## Results

### Overview

This section presents global, regional, and national-level results for key demographic metrics; given space constraints, estimates at the subnational level are presented in appendix 2. All subnational locations are listed in appendix 1 (table S1).

### Reported civil registration and vital statistics completeness

**Figure 1:**
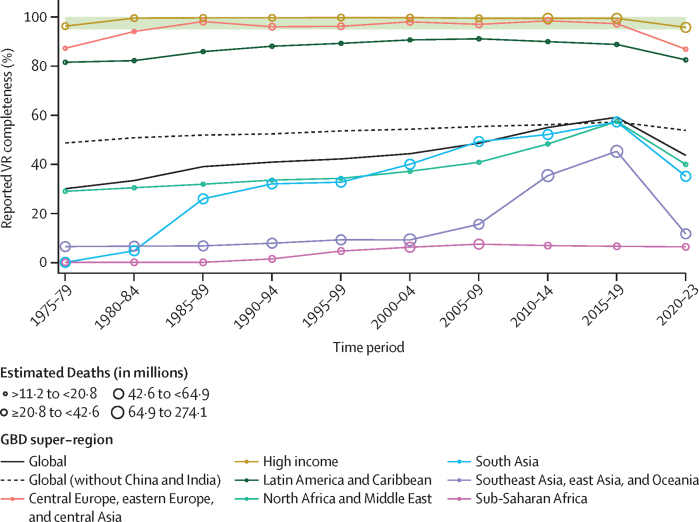
Completeness of reported VR system data by GBD super-region, 1975–2023 Completeness is defined as the total number of deaths registered in all VR systems within a super-region during a 5-year period divided by the total number of estimated deaths within that super-region and period, with 100% completeness indicating that all deaths were registered. The size of the datapoints represents the number of estimated deaths. The solid black line shows global completeness, the dashed black line indicates global completeness excluding China and India, and the other coloured lines indicate GBD super-regions. The green shaded box indicates complete registration (defined as >95%). GBD=Global Burden of Diseases, Injuries, and Risk Factors Study. VR=vital registration.

Between 1975 and 2019, reported global completeness of death registration (ie, the reported proportion of all deaths registered in a VR system) increased considerably, from 30·0% in 1975–79 to 59·8% in 2015–19 (figure 1). Across GBD super-regions, reported VR completeness in 2015–19 varied from 99·6% in the high-income super-region to 6·5% in sub-Saharan Africa. Affected by lags in reporting, reported VR completeness in the 2020–23 period at the global level was lower than in 2015–19, at 45·1%, with the percentage of deaths registered even slightly dropping in the high-income super-region to 95·8%. This relatively small drop in the high-income super-region was partly due to high availability of provisional all-age VR data in this super-region relative to others (incorporated in GBD 2023 for the first time; appendix 2 figures S1A, S1B). Information about the most recent year of available reported VR data and completeness of the VR system for the latest year of reporting for all GBD countries and territories is provided in appendix 2 (figures S1A and S1B).

### Number of deaths

**Figure 2:**
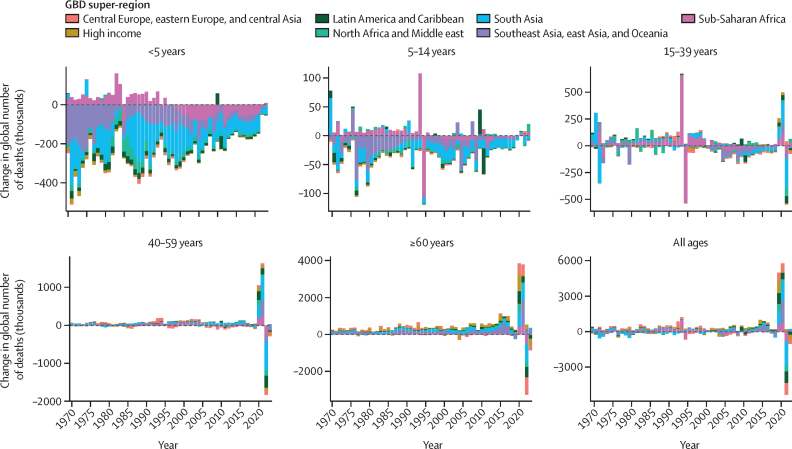
Annual change in number of deaths, for broad age groups and all ages combined, 1970–2023 Annual change is defined as the difference between the number of deaths in the current year and the preceding year. The y-axis scales differ by age group. The large change in the age groups 5–14 years and 15–39 years between 1994 and 1995 was due to deaths during the Rwandan genocide. The large change in the age groups 15–39 years, 40–59 years, and ≥60 years between 2020 and 2022 was due to deaths during the COVID-19 pandemic. GBD=Global Burden of Diseases, Injuries, and Risk Factors Study.

In 2023, 60·1 million (95% UI 59·0–61·1) deaths occurred globally; of these, 4·67 million (4·59–4·75) were in children younger than 5 years (table 1). Due to considerable population growth and ageing since 1950, the number of annual deaths globally, in all ages combined, increased by 35·2% (32·2–38·4) between 1950 and 2023 (appendix 2 table S5A). Under-5 deaths have decreased steadily since 1970, although decreases have slowed since 2010 (figure 2). Initially, most of this decline could be attributed to declines in both the under-5 mortality rate and the under-5 population in southeast Asia, east Asia, and Oceania (especially China) until a tapering off starting around the year 2000. After this, the share of the decline attributed to sub-Saharan Africa began to grow, which then tapered off over the final 5 years of the study period. There were similar declines in deaths in children aged 5–14 years over the study period that have reduced in recent years—although with more fluctuation over time—with the share of declines dominated by south Asia as well as southeast Asia, east Asia, and Oceania. Globally, there were 872 000 (845 000–902 000) deaths in children aged 5–14 years in 2023, compared to 1 739 000 (1 689 000–1 788 000) in 1990, corresponding to a decline of 49·9%. In those aged 15–39 years, deaths fluctuated between increasing and decreasing over the study period—reflecting major stochastic events, such as the Rwandan genocide and its after-effects (1994–95) and the HIV/AIDS epidemic (peaking in sub-Saharan Africa in the late 1990s), with more consistent declines (although at progressively slower rates) between 2005 and 2019. During this recent period, declines were initially dominated by southeast Asia, east Asia, and Oceania, transitioning to south Asia in approximately 2012. After 2019, large increases and subsequent decreases occurred during the COVID-19 pandemic and recovery periods. Those aged 40 years and older had the most extreme increases and subsequent decreases over this period, with especially extreme fluctuations in those aged 60 years and older. The number of deaths in the population aged 60 years and older generally increased, at progressively larger levels, over the entire pre-pandemic period. At the national level, China had the most deaths in 2023 (10·7 million [9·83–11·5]), followed by India (9·85 million [9·20–10·5]) and the USA (3·09 million [3·09–3·09]; table 1). As detailed below, these high counts are largely a reflection of population size; China had the 166th highest age-standardised mortality rate (of 204 countries and territories), India had the 73rd highest rate, and the USA had the 160th highest rate (appendix 2 table S3A).

**Table 1:** Under-5 mortality rate (2023), rate of change in under-5 mortality (2000–23), probability of death between those aged 15–59 years (2023), life expectancy at birth (2023), total number of deaths among children younger than 5 years, and total number of deaths among all ages (2023) globally and for GBD super-regions, regions, and countries and territories

			**Under-5 mortality**	**Under-5 mortality**	**Probability of death aged 15–59 years, 2023**		**Life expectancy at birthin 2023 (years)**			**Total deaths in 2023 (thousands)**	**Total deaths among children <5 years in 2023 (thousands)**
			Mortality rate in 2023 (deaths per 1000)	Annualised rate of change, 2000–23	Female	Male	Female	Male	Both sexes	Both sexes	Both sexes
**Global**			**36·3 (35·7 to 36·9)**	**–2·8% (−2·9 to −2·8)**	**0·11 (0·10 to 0·11)**	**0·16 (0·16 to 0·17)**	**76·3 (76·0 to 76·6)**	**71·5 (71·2 to 71·8)**	**73·8 (73·6 to 74·1)**	**60 100·0 (59 000·0 to 61 100·0)**	**4670·0 (4590·0 to 4750·0)**
**Central Europe, eastern Europe, and central Asia**			**15·0 (14·6 to 15·5)**	**–2·2% (−2·4 to −2·1)**	**0·08 (0·08 to 0·09)**	**0·22 (0·21 to 0·22)**	**78·8 (78·7 to 79·0)**	**69·9 (69·5 to 70·1)**	**74·4 (74·1 to 74·5)**	**4540·0 (4490·0 to 4590·0)**	**70·8 (68·9 to 72·9)**
Central Asia			26·8 (25·8 to 27·7)	−2·2% (−2·3 to −2·0)	0·08 (0·08 to 0·09)	0·17 (0·17 to 0·17)	76·8 (76·6 to 77·0)	70·5 (70·3 to 70·7)	73·7 (73·5 to 73·8)	611·0 (603·0 to 618·0)	55·8 (53·9 to 57·8)
	Armenia		11·8 (10·7 to 13·0)	−3·5% (−4·0 to −3·0)	0·05 (0·04 to 0·06)	0·13 (0·12 to 0·14)	82·6 (82·0 to 83·1)	76·3 (75·5 to 77·1)	79·6 (79·1 to 80·1)	24·7 (23·6 to 25·9)	0·4 (0·4 to 0·4)
	Azerbaijan		50·5 (46·3 to 54·9)	−0·4% (−0·8 to 0·1)	0·07 (0·06 to 0·08)	0·16 (0·15 to 0·17)	75·4 (74·7 to 76·1)	69·9 (69·2 to 70·6)	72·6 (72·1 to 73·1)	69·6 (67·2 to 71·9)	6·1 (5·6 to 6·6)
	Georgia		9·6 (8·6 to 10·7)	−6·4% (−6·9 to −5·9)	0·07 (0·06 to 0·08)	0·22 (0·20 to 0·24)	79·4 (78·9 to 79·9)	70·1 (69·4 to 70·7)	74·7 (74·3 to 75·2)	43·6 (42·0 to 45·3)	0·4 (0·4 to 0·5)
	Kazakhstan		13·7 (12·6 to 14·9)	−3·4% (−3·8 to −3·0)	0·08 (0·08 to 0·09)	0·20 (0·19 to 0·21)	78·7 (78·3 to 79·1)	70·8 (70·3 to 71·2)	74·9 (74·6 to 75·2)	131·0 (128·0 to 134·0)	5·5 (5·1 to 6·0)
	Kyrgyzstan		21·0 (19·4 to 23·0)	−3·2% (−3·6 to −2·8)	0·07 (0·06 to 0·08)	0·15 (0·14 to 0·16)	77·8 (77·2 to 78·5)	72·5 (71·8 to 73·2)	75·2 (74·8 to 75·7)	31·6 (30·3 to 32·8)	3·2 (2·9 to 3·5)
	Mongolia		15·3 (13·8 to 16·9)	−5·2% (−5·7 to −4·8)	0·10 (0·09 to 0·11)	0·27 (0·25 to 0·29)	78·2 (77·4 to 79·0)	68·0 (67·1 to 68·9)	73·1 (72·4 to 73·7)	18·5 (17·7 to 19·4)	1·1 (1·0 to 1·3)
	Tajikistan		25·2 (22·8 to 27·7)	−4·0% (−4·5 to −3·5)	0·10 (0·09 to 0·12)	0·14 (0·12 to 0·16)	73·0 (71·9 to 74·1)	70·6 (69·5 to 71·7)	71·8 (71·0 to 72·5)	50·3 (47·2 to 53·4)	7·0 (6·3 to 7·7)
	Turkmenistan		28·4 (25·9 to 31·4)	−2·5% (−3·0 to −2·0)	0·13 (0·11 to 0·15)	0·22 (0·19 to 0·25)	74·1 (72·8 to 75·3)	68·2 (67·0 to 69·5)	71·1 (70·2 to 72·0)	36·2 (33·9 to 38·5)	3·5 (3·1 to 3·8)
	Uzbekistan		33·3 (31·4 to 35·2)	−0·9% (−1·2 to −0·6)	0·08 (0·08 to 0·09)	0·15 (0·14 to 0·16)	75·0 (74·6 to 75·4)	70·0 (69·6 to 70·3)	72·5 (72·2 to 72·8)	205·0 (201·0 to 209·0)	28·6 (26·9 to 30·2)
Central Europe			4·8 (4·7 to 4·9)	−4·4% (−4·5 to −4·3)	0·06 (0·06 to 0·06)	0·14 (0·14 to 0·14)	81·0 (80·9 to 81·1)	74·4 (74·3 to 74·5)	77·7 (77·7 to 77·8)	1350·0 (1340·0 to 1360·0)	4·7 (4·6 to 4·8)
	Albania		8·7 (7·8 to 9·7)	−4·9% (−5·4 to −4·4)	0·04 (0·04 to 0·05)	0·10 (0·09 to 0·11)	82·1 (81·4 to 82·7)	77·3 (76·6 to 77·9)	79·6 (79·2 to 80·1)	21·0 (19·9 to 22·1)	0·2 (0·2 to 0·2)
	Bosnia and Herzegovina		5·4 (4·9 to 5·8)	−2·7% (−3·1 to −2·3)	0·06 (0·05 to 0·06)	0·11 (0·10 to 0·13)	80·1 (79·4 to 80·8)	75·3 (74·6 to 76·0)	77·7 (77·2 to 78·2)	37·8 (35·8 to 39·9)	0·1 (0·1 to 0·2)
	Bulgaria		6·0 (5·7 to 6·3)	−4·7% (−5·0 to −4·4)	0·08 (0·07 to 0·08)	0·17 (0·17 to 0·18)	79·5 (79·3 to 79·7)	72·5 (72·2 to 72·7)	75·9 (75·7 to 76·1)	102·0 (100·0 to 104·0)	0·3 (0·3 to 0·4)
	Croatia		4·0 (3·9 to 4·2)	−3·3% (−3·5 to −3·2)	0·05 (0·05 to 0·05)	0·12 (0·12 to 0·13)	81·7 (81·4 to 81·9)	75·5 (75·2 to 75·8)	78·6 (78·4 to 78·8)	51·9 (50·8 to 53·0)	0·1 (0·1 to 0·1)
	Czechia		2·5 (2·4 to 2·6)	−3·6% (−3·8 to −3·3)	0·05 (0·05 to 0·05)	0·10 (0·10 to 0·11)	82·8 (82·6 to 82·9)	76·9 (76·8 to 77·1)	79·9 (79·8 to 80·0)	113·0 (112·0 to 114·0)	0·3 (0·3 to 0·3)
	Hungary		4·1 (3·9 to 4·3)	−3·9% (−4·1 to −3·6)	0·07 (0·07 to 0·07)	0·15 (0·14 to 0·15)	80·1 (79·9 to 80·2)	73·4 (73·2 to 73·6)	76·8 (76·7 to 77·0)	128·0 (126·0 to 129·0)	0·4 (0·3 to 0·4)
	Montenegro		4·7 (4·5 to 4·9)	−5·3% (−5·5 to −5·0)	0·06 (0·06 to 0·07)	0·13 (0·12 to 0·14)	79·5 (79·1 to 79·9)	74·7 (74·3 to 75·1)	77·1 (76·8 to 77·3)	7·0 (6·8 to 7·2)	0·0 (0·0 to 0·0)
	North Macedonia		5·7 (5·4 to 6·0)	−4·7% (−5·0 to −4·5)	0·06 (0·06 to 0·07)	0·11 (0·11 to 0·12)	78·9 (78·5 to 79·4)	75·2 (74·6 to 75·7)	77·0 (76·6 to 77·4)	21·0 (20·1 to 22·0)	0·1 (0·1 to 0·1)
	Poland		4·1 (3·9 to 4·3)	−3·7% (−4·0 to −3·5)	0·05 (0·05 to 0·06)	0·14 (0·14 to 0·14)	82·2 (82·1 to 82·4)	75·0 (74·8 to 75·1)	78·6 (78·6 to 78·7)	410·0 (407·0 to 412·0)	1·3 (1·2 to 1·3)
	Romania		6·8 (6·4 to 7·2)	−5·1% (−5·4 to −4·9)	0·07 (0·07 to 0·07)	0·18 (0·17 to 0·18)	80·1 (80·0 to 80·3)	72·5 (72·3 to 72·7)	76·3 (76·1 to 76·4)	246·0 (244·0 to 249·0)	1·1 (1·1 to 1·2)
	Serbia		5·4 (4·8 to 6·0)	−3·4% (−3·9 to −2·9)	0·07 (0·06 to 0·08)	0·13 (0·11 to 0·15)	78·2 (77·4 to 78·9)	73·9 (73·0 to 74·7)	76·0 (75·4 to 76·6)	120·0 (112·0 to 128·0)	0·3 (0·3 to 0·4)
	Slovakia		5·5 (5·3 to 5·8)	−2·5% (−2·7 to −2·3)	0·06 (0·06 to 0·06)	0·13 (0·13 to 0·14)	81·4 (81·2 to 81·7)	74·8 (74·6 to 75·0)	78·2 (78·0 to 78·3)	54·1 (53·3 to 55·0)	0·3 (0·3 to 0·3)
	Slovenia		2·3 (2·2 to 2·4)	−3·9% (−4·1 to −3·7)	0·04 (0·04 to 0·04)	0·08 (0·08 to 0·09)	84·4 (84·1 to 84·6)	79·0 (78·7 to 79·2)	81·6 (81·5 to 81·8)	21·6 (21·1 to 22·1)	0·0 (0·0 to 0·0)
Eastern Europe			6·3 (6·0 to 6·5)	−4·6% (−4·7 to −4·4)	0·10 (0·09 to 0·10)	0·28 (0·27 to 0·29)	78·9 (78·6 to 79·1)	67·8 (67·0 to 68·3)	73·3 (72·8 to 73·7)	2570·0 (2530·0 to 2620·0)	10·3 (9·9 to 10·8)
	Belarus		4·6 (4·3 to 4·9)	−5·6% (−5·9 to −5·3)	0·09 (0·08 to 0·09)	0·23 (0·22 to 0·25)	79·2 (78·6 to 79·7)	69·5 (68·9 to 70·2)	74·5 (74·0 to 74·9)	123·0 (117·0 to 129·0)	0·4 (0·3 to 0·4)
	Estonia		2·8 (2·6 to 2·9)	−5·8% (−6·0 to −5·6)	0·05 (0·05 to 0·06)	0·15 (0·14 to 0·16)	82·9 (82·6 to 83·2)	74·2 (73·8 to 74·6)	78·7 (78·5 to 79·0)	16·3 (15·9 to 16·7)	0·0 (0·0 to 0·0)
	Latvia		3·8 (3·7 to 4·1)	−5·6% (−5·8 to −5·3)	0·08 (0·07 to 0·08)	0·22 (0·21 to 0·23)	80·6 (80·3 to 80·9)	70·7 (70·3 to 71·1)	75·8 (75·5 to 76·1)	27·9 (27·3 to 28·5)	0·1 (0·1 to 0·1)
	Lithuania		3·4 (3·3 to 3·6)	−5·1% (−5·3 to −4·8)	0·07 (0·07 to 0·08)	0·20 (0·19 to 0·21)	81·2 (80·9 to 81·5)	71·9 (71·5 to 72·2)	76·6 (76·4 to 76·9)	38·7 (37·9 to 39·4)	0·1 (0·1 to 0·1)
	Moldova		11·9 (10·7 to 13·2)	−3·6% (−4·1 to −3·1)	0·07 (0·06 to 0·08)	0·19 (0·18 to 0·21)	80·3 (79·6 to 81·0)	71·6 (70·9 to 72·3)	76·0 (75·5 to 76·6)	38·3 (36·6 to 40·0)	0·3 (0·3 to 0·3)
	Russia		6·0 (5·7 to 6·3)	−4·9% (−5·2 to −4·7)	0·10 (0·09 to 0·10)	0·28 (0·27 to 0·30)	78·8 (78·6 to 78·9)	67·6 (66·7 to 68·3)	73·2 (72·6 to 73·6)	1800·0 (1780·0 to 1820·0)	7·6 (7·2 to 8·0)
	Ukraine		8·7 (8·1 to 9·4)	−2·7% (−3·1 to −2·3)	0·10 (0·09 to 0·12)	0·29 (0·27 to 0·30)	78·5 (77·5 to 79·5)	66·6 (65·9 to 67·2)	72·5 (71·9 to 73·2)	534·0 (501·0 to 569·0)	1·9 (1·8 to 2·1)
**High income**			**5·0 (5·0 to 5·0)**	**–1·8% (−1·9 to −1·8)**	**0·06 (0·06 to 0·06)**	**0·10 (0·10 to 0·10)**	**83·7 (83·7 to 83·7)**	**78·5 (78·5 to 78·6)**	**81·1 (81·1 to 81·2)**	**10 700·0 (10 600·0 to 10 700·0)**	**51·0 (50·7 to 51·4)**
Australasia			3·8 (3·6 to 3·9)	−2·4% (−2·5 to −2·2)	0·04 (0·04 to 0·04)	0·07 (0·07 to 0·07)	85·4 (85·4 to 85·5)	81·6 (81·5 to 81·7)	83·5 (83·5 to 83·6)	221·0 (220·0 to 222·0)	1·3 (1·3 to 1·4)
	Australia		3·6 (3·5 to 3·8)	−2·4% (−2·6 to −2·2)	0·04 (0·04 to 0·04)	0·07 (0·07 to 0·07)	85·8 (85·8 to 85·9)	81·9 (81·8 to 82·0)	83·9 (83·8 to 83·9)	182·0 (181·0 to 183·0)	1·1 (1·0 to 1·1)
	New Zealand		4·4 (4·2 to 4·6)	−2·3% (−2·5 to −2·0)	0·05 (0·05 to 0·06)	0·08 (0·08 to 0·09)	83·4 (83·2 to 83·6)	79·9 (79·7 to 80·1)	81·6 (81·5 to 81·8)	38·8 (38·2 to 39·4)	0·3 (0·2 to 0·3)
High-income Asia Pacific			2·4 (2·3 to 2·5)	−3·9% (−4·1 to −3·7)	0·04 (0·04 to 0·04)	0·07 (0·07 to 0·07)	87·1 (87·0 to 87·1)	81·0 (81·0 to 81·1)	84·1 (84·0 to 84·1)	2000·0 (1990·0 to 2010·0)	2·6 (2·5 to 2·7)
	Brunei		9·0 (8·4 to 9·6)	−1·1% (−1·4 to −0·7)	0·08 (0·08 to 0·09)	0·13 (0·12 to 0·14)	79·4 (78·8 to 80·0)	76·9 (76·2 to 77·5)	78·1 (77·6 to 78·5)	2·0 (1·9 to 2·1)	0·1 (0·0 to 0·1)
	Japan		2·3 (2·2 to 2·4)	−3·0% (−3·1 to −2·9)	0·04 (0·04 to 0·04)	0·07 (0·07 to 0·07)	87·2 (87·2 to 87·2)	81·0 (81·0 to 81·1)	84·1 (84·1 to 84·1)	1620·0 (1610·0 to 1620·0)	1·8 (1·8 to 1·9)
	Singapore		1·9 (1·9 to 2·0)	−2·7% (−2·9 to −2·4)	0·03 (0·03 to 0·03)	0·05 (0·05 to 0·05)	87·5 (87·3 to 87·8)	83·1 (82·9 to 83·4)	85·4 (85·2 to 85·5)	26·9 (26·5 to 27·4)	0·1 (0·1 to 0·1)
	South Korea		2·6 (2·3 to 2·9)	−5·1% (−5·6 to −4·5)	0·03 (0·03 to 0·04)	0·07 (0·07 to 0·07)	86·1 (85·9 to 86·2)	80·6 (80·4 to 80·8)	83·4 (83·3 to 83·6)	353·0 (348·0 to 359·0)	0·7 (0·6 to 0·7)
High-income North America			6·5 (6·4 to 6·5)	−1·0% (−1·0 to −0·9)	0·08 (0·08 to 0·08)	0·14 (0·14 to 0·14)	81·4 (81·4 to 81·5)	76·3 (76·3 to 76·3)	78·8 (78·8 to 78·9)	3410·0 (3410·0 to 3420·0)	26·0 (25·8 to 26·2)
	Canada		4·9 (4·8 to 5·1)	−0·9% (−1·1 to −0·7)	0·05 (0·05 to 0·06)	0·09 (0·09 to 0·10)	84·1 (83·8 to 84·4)	79·6 (79·4 to 79·9)	81·9 (81·7 to 82·1)	321·0 (315·0 to 328·0)	1·8 (1·7 to 1·8)
	Greenland		12·9 (12·1 to 13·7)	−2·8% (−3·1 to −2·5)	0·11 (0·10 to 0·12)	0·16 (0·15 to 0·17)	73·4 (72·8 to 74·1)	70·0 (69·3 to 70·7)	71·6 (71·1 to 72·0)	0·5 (0·5 to 0·5)	0·0 (0·0 to 0·0)
	USA		6·6 (6·6 to 6·7)	−1·0% (−1·0 to −0·9)	0·08 (0·08 to 0·08)	0·15 (0·15 to 0·15)	81·1 (81·1 to 81·1)	75·9 (75·9 to 75·9)	78·5 (78·5 to 78·5)	3090·0 (3090·0 to 3090·0)	24·2 (24·0 to 24·4)
Southern Latin America			8·8 (8·5 to 9·1)	−3·0% (−3·1 to −2·8)	0·07 (0·07 to 0·07)	0·12 (0·12 to 0·13)	81·1 (80·8 to 81·4)	75·6 (75·3 to 75·8)	78·4 (78·2 to 78·6)	530·0 (520·0 to 540·0)	6·4 (6·1 to 6·6)
	Argentina		9·4 (9·1 to 9·8)	−3·2% (−3·4 to −3·0)	0·07 (0·07 to 0·08)	0·13 (0·12 to 0·14)	80·3 (79·9 to 80·7)	74·7 (74·3 to 75·0)	77·6 (77·3 to 77·8)	373·0 (363·0 to 384·0)	4·8 (4·6 to 5·0)
	Chile		7·4 (7·1 to 7·8)	−1·9% (−2·1 to −1·6)	0·05 (0·05 to 0·06)	0·10 (0·10 to 0·11)	83·3 (83·1 to 83·4)	78·2 (78·0 to 78·4)	80·8 (80·7 to 80·9)	121·0 (120·0 to 123·0)	1·3 (1·3 to 1·4)
	Uruguay		6·5 (6·1 to 6·8)	−4·2% (−4·5 to −3·9)	0·08 (0·07 to 0·08)	0·15 (0·15 to 0·16)	80·6 (80·3 to 80·8)	74·1 (73·8 to 74·4)	77·4 (77·2 to 77·6)	34·8 (34·2 to 35·4)	0·2 (0·2 to 0·2)
Western Europe			3·6 (3·6 to 3·7)	−2·0% (−2·0 to −1·9)	0·04 (0·04 to 0·04)	0·08 (0·08 to 0·08)	84·4 (84·3 to 84·4)	79·7 (79·6 to 79·7)	82·0 (82·0 to 82·1)	4500·0 (4480·0 to 4530·0)	14·8 (14·6 to 14·9)
	Andorra		4·4 (4·1 to 4·7)	−2·3% (−2·7 to −2·0)	0·05 (0·05 to 0·05)	0·08 (0·08 to 0·09)	86·2 (85·6 to 86·8)	81·8 (81·2 to 82·4)	83·9 (83·4 to 84·3)	0·7 (0·6 to 0·7)	0·0 (0·0 to 0·0)
	Austria		3·0 (2·9 to 3·1)	−2·7% (−2·9 to −2·5)	0·04 (0·04 to 0·04)	0·08 (0·07 to 0·08)	84·2 (84·1 to 84·4)	79·5 (79·4 to 79·7)	81·9 (81·8 to 82·0)	88·5 (87·7 to 89·3)	0·3 (0·2 to 0·3)
	Belgium		2·7 (2·6 to 2·8)	−3·4% (−3·6 to −3·2)	0·05 (0·04 to 0·05)	0·08 (0·07 to 0·08)	84·2 (84·1 to 84·3)	80·0 (79·9 to 80·1)	82·1 (82·0 to 82·2)	112·0 (111·0 to 113·0)	0·3 (0·3 to 0·3)
	Cyprus		3·7 (3·2 to 4·2)	−2·8% (−3·4 to −2·1)	0·04 (0·03 to 0·04)	0·08 (0·07 to 0·09)	82·6 (81·8 to 83·4)	78·6 (77·7 to 79·5)	80·5 (79·9 to 81·1)	10·1 (9·4 to 10·9)	0·1 (0·0 to 0·1)
	Denmark		3·7 (3·5 to 3·9)	−1·8% (−2·0 to −1·5)	0·04 (0·04 to 0·04)	0·07 (0·07 to 0·07)	83·6 (83·4 to 83·7)	79·5 (79·4 to 79·7)	81·5 (81·4 to 81·7)	58·6 (57·9 to 59·3)	0·2 (0·2 to 0·2)
	Finland		2·4 (2·3 to 2·5)	−2·6% (−2·8 to −2·4)	0·04 (0·04 to 0·05)	0·09 (0·08 to 0·09)	84·3 (84·1 to 84·4)	79·2 (79·0 to 79·3)	81·7 (81·6 to 81·8)	61·2 (60·5 to 62·0)	0·1 (0·1 to 0·1)
	France		4·1 (4·0 to 4·2)	−1·2% (−1·3 to −1·0)	0·04 (0·04 to 0·05)	0·09 (0·09 to 0·09)	85·5 (85·4 to 85·5)	79·7 (79·6 to 79·8)	82·6 (82·6 to 82·7)	634·0 (632·0 to 636·0)	2·9 (2·8 to 2·9)
	Germany		3·6 (3·5 to 3·8)	−1·7% (−1·9 to −1·5)	0·05 (0·05 to 0·05)	0·09 (0·09 to 0·09)	83·4 (83·1 to 83·6)	78·5 (78·2 to 78·8)	80·9 (80·7 to 81·1)	1020·0 (998·0 to 1050·0)	2·8 (2·7 to 2·9)
	Greece		4·1 (3·9 to 4·3)	−1·9% (−2·1 to −1·7)	0·05 (0·04 to 0·05)	0·10 (0·10 to 0·11)	83·8 (83·6 to 83·9)	78·3 (78·1 to 78·4)	81·0 (80·8 to 81·1)	132·0 (131·0 to 133·0)	0·3 (0·3 to 0·3)
	Iceland		3·2 (3·1 to 3·4)	−1·4% (−1·7 to −1·1)	0·04 (0·04 to 0·05)	0·07 (0·07 to 0·08)	84·3 (83·8 to 84·6)	80·8 (80·4 to 81·2)	82·5 (82·2 to 82·8)	2·6 (2·5 to 2·7)	0·0 (0·0 to 0·0)
	Ireland		3·3 (3·1 to 3·4)	−3·2% (−3·4 to −2·9)	0·04 (0·04 to 0·04)	0·06 (0·06 to 0·07)	84·3 (84·1 to 84·5)	80·8 (80·6 to 81·0)	82·5 (82·4 to 82·7)	35·6 (35·0 to 36·1)	0·2 (0·2 to 0·2)
	Israel		3·2 (3·1 to 3·4)	−3·2% (−3·4 to −3·0)	0·04 (0·03 to 0·04)	0·07 (0·07 to 0·07)	85·3 (85·2 to 85·5)	81·2 (81·0 to 81·3)	83·3 (83·1 to 83·4)	50·8 (50·1 to 51·5)	0·6 (0·6 to 0·6)
	Italy		2·9 (2·8 to 3·0)	−2·8% (−3·0 to −2·7)	0·04 (0·04 to 0·04)	0·07 (0·07 to 0·07)	85·1 (85·1 to 85·2)	80·7 (80·7 to 80·8)	83·0 (82·9 to 83·0)	672·0 (670·0 to 675·0)	1·2 (1·1 to 1·2)
	Luxembourg		2·8 (2·7 to 3·0)	−2·5% (−2·8 to −2·2)	0·03 (0·03 to 0·04)	0·06 (0·06 to 0·07)	85·1 (84·7 to 85·5)	81·2 (80·8 to 81·7)	83·2 (82·9 to 83·5)	4·3 (4·1 to 4·4)	0·0 (0·0 to 0·0)
	Malta		4·2 (4·0 to 4·5)	−2·8% (−3·1 to −2·5)	0·03 (0·03 to 0·04)	0·06 (0·06 to 0·07)	84·7 (84·3 to 85·1)	81·4 (81·0 to 81·8)	83·0 (82·7 to 83·3)	4·1 (3·9 to 4·2)	0·0 (0·0 to 0·0)
	Monaco		3·7 (3·3 to 4·2)	−2·5% (−3·1 to −1·8)	0·05 (0·04 to 0·06)	0·10 (0·08 to 0·11)	83·1 (82·1 to 84·1)	77·9 (76·8 to 79·0)	80·4 (79·6 to 81·2)	0·5 (0·5 to 0·6)	0·0 (0·0 to 0·0)
	Netherlands		3·8 (3·6 to 3·9)	−2·1% (−2·4 to −1·9)	0·04 (0·04 to 0·04)	0·06 (0·06 to 0·06)	83·6 (83·5 to 83·7)	80·4 (80·3 to 80·5)	82·0 (81·9 to 82·1)	170·0 (168·0 to 171·0)	0·7 (0·6 to 0·7)
	Norway		2·3 (2·2 to 2·4)	−3·1% (−3·3 to −2·9)	0·04 (0·04 to 0·04)	0·06 (0·06 to 0·06)	84·6 (84·5 to 84·8)	81·4 (81·2 to 81·5)	83·0 (82·9 to 83·1)	43·5 (43·0 to 44·0)	0·1 (0·1 to 0·1)
	Portugal		3·3 (3·1 to 3·4)	−3·3% (−3·5 to −3·1)	0·04 (0·04 to 0·04)	0·10 (0·10 to 0·10)	84·8 (84·7 to 85·0)	79·0 (78·8 to 79·1)	82·0 (81·9 to 82·1)	120·0 (118·0 to 121·0)	0·3 (0·3 to 0·3)
	San Marino		4·1 (3·6 to 4·7)	−2·1% (−2·8 to −1·4)	0·04 (0·03 to 0·04)	0·05 (0·04 to 0·05)	87·2 (86·3 to 88·1)	83·2 (82·1 to 84·2)	85·1 (84·4 to 85·8)	0·3 (0·3 to 0·3)	0·0 (0·0 to 0·0)
	Spain		3·4 (3·3 to 3·5)	−2·0% (−2·1 to −1·8)	0·04 (0·04 to 0·04)	0·08 (0·07 to 0·08)	85·9 (85·8 to 85·9)	80·4 (80·3 to 80·5)	83·2 (83·1 to 83·2)	444·0 (442·0 to 446·0)	1·1 (1·1 to 1·2)
	Sweden		2·6 (2·5 to 2·7)	−2·0% (−2·2 to −1·8)	0·04 (0·03 to 0·04)	0·06 (0·05 to 0·06)	85·0 (84·9 to 85·1)	81·6 (81·5 to 81·7)	83·3 (83·2 to 83·4)	94·4 (93·6 to 95·3)	0·3 (0·3 to 0·3)
	Switzerland		3·5 (3·3 to 3·6)	−2·3% (−2·5 to −2·0)	0·03 (0·03 to 0·03)	0·05 (0·05 to 0·05)	86·2 (86·1 to 86·3)	82·6 (82·4 to 82·7)	84·4 (84·3 to 84·5)	71·5 (70·8 to 72·3)	0·3 (0·3 to 0·3)
	UK		4·4 (4·3 to 4·6)	−1·7% (−1·8 to −1·5)	0·06 (0·06 to 0·06)	0·09 (0·09 to 0·09)	82·9 (82·9 to 83·0)	79·0 (79·0 to 79·1)	81·0 (80·9 to 81·0)	668·0 (666·0 to 670·0)	3·1 (3·0 to 3·2)
		England	4·5 (4·3 to 4·6)	−1·6% (−1·8 to −1·5)	0·05 (0·05 to 0·06)	0·09 (0·09 to 0·09)	83·2 (83·1 to 83·2)	79·3 (79·2 to 79·4)	81·2 (81·2 to 81·3)	550·0 (547·0 to 552·0)	2·7 (2·6 to 2·8)
		Northern Ireland	4·7 (4·2 to 5·3)	−1·6% (−2·1 to −1·0)	0·06 (0·05 to 0·06)	0·10 (0·10 to 0·11)	82·5 (82·2 to 82·8)	78·8 (78·4 to 79·1)	80·6 (80·4 to 80·9)	17·5 (17·1 to 17·9)	0·1 (0·1 to 0·1)
		Scotland	4·2 (3·8 to 4·6)	−1·9% (−2·4 to −1·4)	0·07 (0·07 to 0·08)	0·12 (0·11 to 0·13)	81·1 (80·9 to 81·4)	77·1 (76·8 to 77·4)	79·1 (78·9 to 79·3)	63·8 (62·8 to 64·8)	0·2 (0·2 to 0·2)
		Wales	4·2 (3·5 to 4·9)	−1·7% (−2·5 to −0·9)	0·07 (0·06 to 0·08)	0·11 (0·10 to 0·12)	82·0 (81·4 to 82·6)	77·9 (77·3 to 78·6)	80·0 (79·5 to 80·4)	36·6 (35·0 to 38·1)	0·1 (0·1 to 0·1)
**Latin America and Caribbean**			**18·3 (18·0 to 18·6)**	**–2·6% (−2·7 to −2·5)**	**0·09 (0·09 to 0·09)**	**0·17 (0·17 to 0·17)**	**79·0 (78·9 to 79·1)**	**73·0 (72·9 to 73·1)**	**76·0 (76·0 to 76·1)**	**3880·0 (3860·0 to 3900·0)**	**160·0 (158·0 to 163·0)**
Andean Latin America			23·5 (22·7 to 24·4)	−2·4% (−2·6 to −2·2)	0·09 (0·09 to 0·10)	0·15 (0·14 to 0·15)	78·4 (78·1 to 78·6)	73·8 (73·5 to 74·1)	76·1 (75·9 to 76·3)	390·0 (384·0 to 395·0)	27·0 (26·0 to 28·0)
	Bolivia		28·8 (27·3 to 30·4)	−4·0% (−4·3 to −3·8)	0·13 (0·13 to 0·14)	0·19 (0·17 to 0·20)	74·5 (73·9 to 75·2)	69·3 (68·7 to 70·0)	71·9 (71·4 to 72·3)	77·2 (74·3 to 80·0)	6·7 (6·4 to 7·1)
	Ecuador		28·3 (27·1 to 29·5)	−0·1% (−0·3 to 0·2)	0·08 (0·07 to 0·08)	0·16 (0·16 to 0·17)	79·9 (79·6 to 80·1)	73·6 (73·3 to 73·9)	76·7 (76·5 to 76·9)	102·0 (100·0 to 103·0)	8·4 (8·1 to 8·8)
	Peru		19·2 (17·9 to 20·5)	−2·6% (−3·0 to −2·3)	0·09 (0·08 to 0·09)	0·13 (0·12 to 0·14)	79·0 (78·6 to 79·3)	75·3 (74·9 to 75·7)	77·1 (76·8 to 77·4)	212·0 (207·0 to 216·0)	11·8 (11·0 to 12·7)
Caribbean			35·9 (33·8 to 38·1)	−1·5% (−1·8 to −1·2)	0·15 (0·14 to 0·15)	0·21 (0·20 to 0·22)	73·8 (73·3 to 74·4)	69·0 (68·4 to 69·6)	71·4 (71·0 to 71·8)	429·0 (418·0 to 441·0)	28·5 (26·8 to 30·3)
	Antigua and Barbuda		8·4 (7·7 to 9·3)	−2·5% (−2·9 to −2·0)	0·10 (0·09 to 0·11)	0·13 (0·12 to 0·15)	79·7 (79·2 to 80·3)	75·8 (74·6 to 76·9)	77·8 (77·1 to 78·4)	0·6 (0·6 to 0·7)	0·0 (0·0 to 0·0)
	The Bahamas		11·3 (10·7 to 11·9)	−2·1% (−2·4 to −1·8)	0·14 (0·13 to 0·15)	0·23 (0·22 to 0·24)	75·8 (75·2 to 76·4)	69·8 (69·2 to 70·5)	72·8 (72·4 to 73·3)	3·1 (3·0 to 3·3)	0·0 (0·0 to 0·0)
	Barbados		9·8 (9·1 to 10·4)	−2·1% (−2·4 to −1·8)	0·10 (0·09 to 0·10)	0·14 (0·13 to 0·16)	79·0 (78·4 to 79·5)	74·3 (73·7 to 75·0)	76·7 (76·2 to 77·1)	3·3 (3·1 to 3·4)	0·0 (0·0 to 0·0)
	Belize		13·5 (12·6 to 14·4)	−3·7% (−4·0 to −3·3)	0·11 (0·10 to 0·12)	0·19 (0·18 to 0·21)	78·0 (77·3 to 78·7)	72·6 (71·8 to 73·3)	75·2 (74·7 to 75·8)	2·0 (1·9 to 2·1)	0·1 (0·1 to 0·1)
	Bermuda		5·0 (4·7 to 5·3)	−2·8% (−3·1 to −2·6)	0·05 (0·05 to 0·06)	0·12 (0·11 to 0·12)	83·4 (82·9 to 83·8)	78·0 (77·5 to 78·5)	80·7 (80·4 to 81·1)	0·6 (0·6 to 0·6)	0·0 (0·0 to 0·0)
	Cuba		5·7 (5·1 to 6·2)	−1·8% (−2·3 to −1·3)	0·07 (0·06 to 0·08)	0·12 (0·11 to 0·14)	80·6 (79·4 to 81·7)	75·8 (74·7 to 77·0)	78·1 (77·3 to 78·9)	116·0 (107·0 to 125·0)	0·5 (0·5 to 0·6)
	Dominica		9·3 (8·7 to 9·8)	−2·8% (−3·1 to −2·5)	0·10 (0·09 to 0·11)	0·16 (0·15 to 0·17)	78·7 (78·2 to 79·3)	73·8 (73·2 to 74·4)	76·1 (75·7 to 76·6)	0·7 (0·6 to 0·7)	0·0 (0·0 to 0·0)
	Dominican Republic		23·6 (21·7 to 25·6)	−2·1% (−2·5 to −1·6)	0·11 (0·10 to 0·12)	0·20 (0·19 to 0·21)	76·4 (75·8 to 76·9)	69·9 (69·3 to 70·6)	73·0 (72·6 to 73·5)	78·3 (75·6 to 80·9)	4·9 (4·5 to 5·3)
	Grenada		15·3 (13·3 to 17·4)	−1·1% (−1·7 to −0·4)	0·11 (0·10 to 0·13)	0·17 (0·15 to 0·19)	77·2 (76·0 to 78·3)	73·4 (72·1 to 74·7)	75·2 (74·3 to 76·0)	1·0 (0·9 to 1·1)	0·0 (0·0 to 0·0)
	Guyana		28·6 (25·8 to 31·6)	−1·2% (−1·7 to −0·7)	0·17 (0·15 to 0·20)	0·29 (0·26 to 0·32)	72·5 (71·3 to 73·8)	65·8 (64·3 to 67·0)	69·0 (68·0 to 69·9)	6·8 (6·4 to 7·3)	0·4 (0·4 to 0·5)
	Haiti		57·9 (53·5 to 62·5)	−2·6% (−2·9 to −2·2)	0·28 (0·26 to 0·31)	0·33 (0·30 to 0·36)	62·2 (60·9 to 63·3)	59·9 (58·7 to 61·2)	61·0 (60·2 to 61·9)	126·0 (120·0 to 133·0)	20·4 (18·8 to 22·0)
	Jamaica		15·9 (14·0 to 17·7)	−2·5% (−3·1 to −1·9)	0·11 (0·10 to 0·13)	0·16 (0·14 to 0·18)	78·9 (77·6 to 80·1)	73·5 (72·2 to 74·8)	76·1 (75·2 to 77·1)	20·2 (18·9 to 21·7)	0·6 (0·5 to 0·7)
	Puerto Rico		8·2 (7·8 to 8·6)	−1·5% (−1·7 to −1·2)	0·06 (0·06 to 0·06)	0·15 (0·15 to 0·16)	84·5 (84·2 to 84·8)	77·3 (77·0 to 77·7)	81·0 (80·7 to 81·2)	34·2 (33·6 to 34·9)	0·1 (0·1 to 0·1)
	Saint Kitts and Nevis		12·0 (11·3 to 12·7)	−2·6% (−2·9 to −2·4)	0·11 (0·10 to 0·12)	0·18 (0·17 to 0·19)	77·5 (76·9 to 78·0)	72·2 (71·6 to 72·8)	74·8 (74·4 to 75·2)	0·4 (0·4 to 0·4)	0·0 (0·0 to 0·0)
	Saint Lucia		10·6 (9·4 to 12·0)	−2·4% (−3·0 to −1·8)	0·09 (0·08 to 0·10)	0·17 (0·15 to 0·19)	80·2 (79·0 to 81·3)	74·8 (73·5 to 76·0)	77·4 (76·5 to 78·3)	1·3 (1·2 to 1·4)	0·0 (0·0 to 0·0)
	Saint Vincent and the Grenadines		11·3 (10·2 to 12·6)	−3·2% (−3·7 to −2·7)	0·12 (0·10 to 0·14)	0·19 (0·17 to 0·22)	77·9 (76·8 to 79·1)	72·7 (71·5 to 74·0)	75·1 (74·3 to 76·0)	1·0 (1·0 to 1·1)	0·0 (0·0 to 0·0)
	Suriname		29·0 (27·8 to 30·2)	−1·1% (−1·3 to −0·9)	0·12 (0·12 to 0·13)	0·19 (0·18 to 0·20)	75·9 (75·4 to 76·3)	70·4 (69·9 to 70·9)	73·1 (72·8 to 73·5)	4·5 (4·4 to 4·6)	0·3 (0·3 to 0·3)
	Trinidad and Tobago		12·0 (11·4 to 12·7)	−3·9% (−4·1 to −3·6)	0·12 (0·12 to 0·13)	0·20 (0·19 to 0·22)	76·2 (75·8 to 76·7)	70·3 (69·8 to 70·8)	73·2 (72·8 to 73·5)	13·9 (13·4 to 14·3)	0·2 (0·2 to 0·2)
	Virgin Islands		6·8 (6·1 to 7·7)	−2·4% (−3·0 to −1·8)	0·09 (0·08 to 0·11)	0·23 (0·21 to 0·26)	81·6 (80·5 to 82·7)	71·7 (70·3 to 73·1)	76·4 (75·6 to 77·3)	1·0 (0·9 to 1·1)	0·0 (0·0 to 0·0)
Central Latin America			16·9 (16·5 to 17·2)	−2·3% (−2·4 to −2·2)	0·09 (0·09 to 0·09)	0·17 (0·17 to 0·17)	79·3 (79·2 to 79·5)	73·5 (73·3 to 73·6)	76·4 (76·3 to 76·5)	1550·0 (1530·0 to 1560·0)	63·2 (62·0 to 64·6)
	Colombia		11·8 (11·3 to 12·4)	−3·9% (−4·1 to −3·7)	0·06 (0·06 to 0·06)	0·12 (0·12 to 0·13)	83·2 (83·0 to 83·4)	77·0 (76·8 to 77·2)	80·1 (80·0 to 80·3)	267·0 (264·0 to 270·0)	8·6 (8·2 to 9·0)
	Costa Rica		8·1 (7·7 to 8·5)	−2·2% (−2·5 to −2·0)	0·06 (0·06 to 0·07)	0·13 (0·12 to 0·13)	82·2 (81·9 to 82·4)	76·9 (76·6 to 77·2)	79·4 (79·2 to 79·7)	29·2 (28·6 to 29·8)	0·4 (0·4 to 0·4)
	El Salvador		14·8 (13·8 to 15·9)	−2·9% (−3·3 to −2·6)	0·11 (0·10 to 0·12)	0·26 (0·23 to 0·28)	76·8 (76·1 to 77·5)	69·0 (68·0 to 70·0)	73·1 (72·5 to 73·7)	47·1 (44·9 to 49·3)	1·3 (1·2 to 1·3)
	Guatemala		23·5 (21·5 to 25·9)	−3·3% (−3·7 to −2·8)	0·13 (0·11 to 0·15)	0·21 (0·19 to 0·23)	75·5 (74·4 to 76·5)	70·8 (69·6 to 72·0)	73·2 (72·4 to 74·0)	88·5 (83·4 to 93·6)	8·0 (7·3 to 8·8)
	Honduras		10·3 (9·8 to 10·8)	−4·7% (−5·0 to −4·5)	0·09 (0·08 to 0·10)	0·15 (0·14 to 0·16)	77·6 (77·1 to 78·1)	74·4 (73·9 to 75·0)	76·1 (75·7 to 76·5)	47·5 (45·8 to 49·3)	2·5 (2·4 to 2·6)
	Mexico		16·7 (16·3 to 17·1)	−2·1% (−2·2 to −1·9)	0·09 (0·09 to 0·09)	0·18 (0·18 to 0·18)	79·2 (79·1 to 79·4)	73·4 (73·3 to 73·5)	76·3 (76·2 to 76·4)	797·0 (791·0 to 802·0)	30·1 (29·3 to 30·9)
	Nicaragua		13·7 (12·5 to 15·2)	−4·0% (−4·5 to −3·5)	0·09 (0·08 to 0·10)	0·16 (0·14 to 0·18)	79·2 (78·1 to 80·2)	74·1 (72·9 to 75·1)	76·7 (75·9 to 77·5)	29·4 (27·6 to 31·5)	1·7 (1·6 to 1·9)
	Panama		14·4 (13·7 to 15·2)	−2·0% (−2·2 to −1·7)	0·07 (0·06 to 0·07)	0·12 (0·11 to 0·13)	81·9 (81·4 to 82·3)	76·6 (76·0 to 77·1)	79·2 (78·8 to 79·5)	22·6 (21·9 to 23·4)	1·0 (0·9 to 1·0)
	Venezuela		31·2 (29·4 to 33·0)	1·4% (1·1 to 1·6)	0·11 (0·10 to 0·12)	0·21 (0·19 to 0·22)	75·3 (74·7 to 75·9)	68·2 (67·5 to 68·9)	71·8 (71·3 to 72·2)	218·0 (209·0 to 226·0)	9·7 (9·1 to 10·2)
Tropical Latin America			13·6 (13·0 to 14·1)	−3·8% (−4·0 to −3·7)	0·09 (0·08 to 0·09)	0·17 (0·17 to 0·18)	80·1 (80·0 to 80·2)	73·4 (73·2 to 73·6)	76·8 (76·7 to 76·9)	1510·0 (1500·0 to 1520·0)	41·7 (40·0 to 43·5)
	Brazil		13·4 (12·8 to 14·0)	−3·9% (−4·2 to −3·7)	0·08 (0·08 to 0·09)	0·17 (0·17 to 0·18)	80·2 (80·0 to 80·3)	73·5 (73·3 to 73·7)	76·9 (76·8 to 77·0)	1470·0 (1460·0 to 1480·0)	39·8 (38·1 to 41·5)
	Paraguay		18·0 (16·9 to 19·1)	−1·3% (−1·6 to −1·0)	0·11 (0·10 to 0·12)	0·19 (0·18 to 0·21)	76·2 (75·6 to 76·9)	70·0 (69·3 to 70·8)	73·0 (72·5 to 73·5)	42·9 (41·2 to 44·7)	1·9 (1·8 to 2·1)
**North Africa and Middle East**			**23·6 (22·7 to 24·5)**	**–3·4% (−3·5 to −3·2)**	**0·09 (0·09 to 0·10)**	**0·13 (0·13 to 0·14)**	**75·4 (75·0 to 75·7)**	**72·1 (71·8 to 72·5)**	**73·7 (73·4 to 74·0)**	**3420·0 (3340·0 to 3500·0)**	**280·0 (269·0 to 290·0)**
	Afghanistan		52·5 (47·7 to 57·6)	−3·1% (−3·6 to −2·7)	0·14 (0·12 to 0·16)	0·16 (0·14 to 0·18)	69·0 (67·7 to 70·3)	68·1 (67·0 to 69·3)	68·5 (67·6 to 69·3)	178·0 (168·0 to 188·0)	70·6 (64·1 to 77·6)
	Algeria		18·3 (17·5 to 19·1)	−3·0% (−3·3 to −2·8)	0·09 (0·08 to 0·09)	0·12 (0·11 to 0·13)	77·6 (77·1 to 78·2)	74·5 (74·1 to 75·0)	76·0 (75·7 to 76·3)	232·0 (225·0 to 239·0)	16·0 (15·3 to 16·7)
	Bahrain		6·5 (5·9 to 7·3)	−3·1% (−3·6 to −2·5)	0·06 (0·05 to 0·07)	0·09 (0·07 to 0·10)	79·1 (78·0 to 80·2)	75·9 (74·9 to 77·0)	77·2 (76·5 to 78·0)	4·7 (4·3 to 5·1)	0·1 (0·1 to 0·1)
	Egypt		19·8 (17·9 to 22·0)	−3·5% (−4·0 to −3·0)	0·12 (0·10 to 0·14)	0·17 (0·15 to 0·19)	71·1 (69·9 to 72·2)	69·1 (68·0 to 70·2)	70·0 (69·2 to 70·8)	681·0 (628·0 to 738·0)	51·3 (46·2 to 57·2)
	Iran		11·8 (11·1 to 12·4)	−4·1% (−4·4 to −3·9)	0·05 (0·05 to 0·06)	0·10 (0·09 to 0·11)	80·6 (80·1 to 81·1)	77·2 (76·7 to 77·7)	78·8 (78·5 to 79·2)	365·0 (352·0 to 379·0)	12·0 (11·3 to 12·7)
	Iraq		21·8 (21·1 to 22·5)	−3·4% (−3·6 to −3·2)	0·14 (0·12 to 0·15)	0·21 (0·19 to 0·22)	74·2 (73·5 to 74·8)	68·3 (67·7 to 69·0)	71·1 (70·6 to 71·5)	224·0 (216·0 to 232·0)	20·2 (19·5 to 20·9)
	Jordan		13·1 (12·3 to 13·8)	−4·2% (−4·5 to −3·9)	0·06 (0·05 to 0·06)	0·09 (0·08 to 0·09)	80·4 (79·9 to 80·9)	76·9 (76·4 to 77·4)	78·5 (78·1 to 78·8)	40·9 (39·7 to 42·3)	3·0 (2·8 to 3·1)
	Kuwait		8·3 (7·8 to 8·7)	−2·4% (−2·7 to −2·1)	0·04 (0·04 to 0·04)	0·06 (0·06 to 0·07)	81·7 (81·2 to 82·3)	80·9 (80·4 to 81·4)	81·1 (80·7 to 81·5)	9·2 (8·9 to 9·4)	0·4 (0·4 to 0·4)
	Lebanon		20·2 (18·0 to 22·6)	−0·8% (−1·3 to −0·2)	0·07 (0·06 to 0·08)	0·12 (0·11 to 0·14)	80·9 (79·8 to 82·2)	76·8 (75·6 to 78·1)	78·9 (78·1 to 79·7)	30·4 (28·2 to 32·7)	1·6 (1·5 to 1·8)
	Libya		30·3 (28·8 to 31·9)	0·5% (0·2 to 0·7)	0·16 (0·15 to 0·17)	0·16 (0·15 to 0·17)	70·0 (69·2 to 70·7)	70·3 (69·7 to 70·9)	70·1 (69·5 to 70·7)	49·5 (47·7 to 51·6)	3·5 (3·3 to 3·7)
	Morocco		17·6 (16·1 to 19·1)	−4·3% (−4·7 to −3·9)	0·14 (0·12 to 0·16)	0·13 (0·12 to 0·15)	72·8 (71·8 to 73·8)	73·3 (72·5 to 74·2)	73·0 (72·3 to 73·7)	280·0 (262·0 to 300·0)	8·9 (8·2 to 9·7)
	Oman		8·1 (7·6 to 8·8)	−2·7% (−3·1 to −2·4)	0·05 (0·04 to 0·05)	0·09 (0·08 to 0·10)	79·5 (78·6 to 80·3)	75·2 (74·4 to 75·9)	77·0 (76·4 to 77·5)	12·3 (11·7 to 13·0)	0·7 (0·6 to 0·7)
	Palestine		25·4 (24·0 to 27·0)	0·1% (−0·2 to 0·4)	0·15 (0·14 to 0·16)	0·31 (0·29 to 0·33)	72·0 (71·2 to 72·7)	60·7 (59·4 to 62·0)	65·9 (64·9 to 66·9)	38·1 (36·0 to 40·4)	3·3 (3·2 to 3·6)
	Qatar		5·4 (4·8 to 6·2)	−3·9% (−4·4 to −3·3)	0·03 (0·03 to 0·04)	0·05 (0·04 to 0·05)	82·4 (81·3 to 83·5)	80·6 (79·6 to 81·6)	81·4 (80·6 to 82·1)	3·2 (3·0 to 3·5)	0·2 (0·1 to 0·2)
	Saudi Arabia		10·9 (10·0 to 11·9)	−3·4% (−3·9 to −3·0)	0·08 (0·07 to 0·09)	0·11 (0·10 to 0·12)	75·6 (74·6 to 76·5)	73·8 (73·0 to 74·6)	74·5 (73·9 to 75·1)	98·7 (93·0 to 104·0)	4·7 (4·3 to 5·2)
	Sudan		26·6 (24·0 to 29·4)	−5·8% (−6·3 to −5·3)	0·16 (0·14 to 0·18)	0·20 (0·18 to 0·22)	70·5 (69·3 to 71·7)	67·3 (66·2 to 68·5)	68·9 (68·0 to 69·7)	242·0 (226·0 to 259·0)	22·7 (20·4 to 25·1)
	Syria		10·3 (9·8 to 10·9)	−3·0% (−3·2 to −2·8)	0·08 (0·07 to 0·08)	0·12 (0·11 to 0·13)	77·1 (76·6 to 77·6)	73·9 (73·4 to 74·4)	75·4 (75·0 to 75·8)	93·9 (90·2 to 97·5)	2·7 (2·5 to 2·8)
	Tunisia		29·4 (28·1 to 30·7)	−1·6% (−1·8 to −1·4)	0·07 (0·06 to 0·07)	0·12 (0·11 to 0·12)	77·9 (77·5 to 78·4)	73·4 (72·9 to 73·8)	75·6 (75·3 to 75·9)	77·7 (75·5 to 79·9)	4·5 (4·3 to 4·7)
	Türkiye		17·9 (16·5 to 19·4)	−3·7% (−4·1 to −3·3)	0·07 (0·06 to 0·08)	0·11 (0·10 to 0·12)	78·3 (77·2 to 79·4)	73·9 (72·9 to 74·9)	76·1 (75·3 to 76·8)	595·0 (552·0 to 643·0)	18·3 (16·9 to 19·8)
	United Arab Emirates		5·2 (4·7 to 5·6)	−3·3% (−3·7 to −2·8)	0·03 (0·03 to 0·04)	0·04 (0·04 to 0·05)	79·6 (78·9 to 80·4)	80·2 (79·5 to 80·9)	80·1 (79·6 to 80·6)	14·2 (13·3 to 15·2)	0·4 (0·4 to 0·4)
	Yemen		32·8 (29·8 to 36·4)	−3·7% (−4·1 to −3·2)	0·09 (0·07 to 0·10)	0·16 (0·14 to 0·18)	74·2 (72·9 to 75·3)	69·4 (68·4 to 70·6)	71·7 (70·8 to 72·5)	148·0 (140·0 to 158·0)	34·2 (30·9 to 37·9)
**South Asia**			**35·9 (33·4 to 38·3)**	**–3·6% (−3·9 to −3·3)**	**0·14 (0·12 to 0·16)**	**0·18 (0·16 to 0·20)**	**72·4 (71·5 to 73·3)**	**70·1 (69·1 to 71·0)**	**71·2 (70·5 to 71·9)**	**12 500·0 (11 800·0 to 13 200·0)**	**1130·0 (1060·0 to 1210·0)**
	Bangladesh		31·1 (27·8 to 34·9)	−4·2% (−4·7 to −3·7)	0·15 (0·13 to 0·17)	0·16 (0·14 to 0·19)	71·2 (70·1 to 72·2)	71·0 (69·9 to 72·2)	71·1 (70·2 to 71·8)	1090·0 (1020·0 to 1160·0)	103·0 (91·7 to 116·0)
	Bhutan		28·1 (25·6 to 31·1)	−4·5% (−5·0 to −4·0)	0·16 (0·14 to 0·18)	0·16 (0·14 to 0·19)	72·4 (71·2 to 73·6)	72·9 (71·7 to 74·1)	72·6 (71·8 to 73·5)	5·0 (4·7 to 5·3)	0·3 (0·3 to 0·3)
	India		32·1 (28·9 to 35·6)	−3·9% (−4·4 to −3·5)	0·13 (0·11 to 0·15)	0·18 (0·16 to 0·20)	73·0 (71·8 to 74·1)	70·2 (69·1 to 71·3)	71·6 (70·8 to 72·4)	9850·0 (9200·0 to 10 500·0)	692·0 (623·0 to 768·0)
	Nepal		29·0 (26·0 to 32·0)	−4·3% (−4·8 to −3·8)	0·11 (0·09 to 0·12)	0·17 (0·15 to 0·19)	75·1 (74·0 to 76·3)	71·9 (70·7 to 73·1)	73·5 (72·7 to 74·4)	180·0 (169·0 to 192·0)	16·7 (15·0 to 18·5)
	Pakistan		52·5 (47·8 to 57·2)	−2·6% (−3·1 to −2·2)	0·18 (0·15 to 0·20)	0·18 (0·16 to 0·21)	70·1 (68·8 to 71·3)	70·0 (68·7 to 71·2)	70·0 (69·1 to 70·9)	1370·0 (1300·0 to 1450·0)	322·0 (292·0 to 352·0)
**Southeast Asia, east Asia, and Oceania**			**15·2 (14·6 to 15·9)**	**–4·2% (−4·5 to −4·0)**	**0·07 (0·07 to 0·08)**	**0·13 (0·12 to 0·14)**	**80·2 (79·6 to 81·0)**	**74·8 (74·1 to 75·6)**	**77·4 (76·9 to 78·0)**	**16 100·0 (15 300·0 to 17 000·0)**	**329·0 (315·0 to 344·0)**
East Asia			4·9 (4·5 to 5·5)	−8·7% (−9·1 to −8·2)	0·04 (0·04 to 0·05)	0·10 (0·08 to 0·11)	82·6 (81·7 to 83·6)	77·5 (76·5 to 78·6)	80·0 (79·2 to 80·7)	11 100·0 (10 300·0 to 11 900·0)	53·4 (48·6 to 59·3)
	China		4·7 (4·2 to 5·2)	−8·9% (−9·4 to −8·4)	0·04 (0·04 to 0·05)	0·10 (0·08 to 0·11)	82·8 (81·8 to 83·7)	77·6 (76·6 to 78·7)	80·1 (79·3 to 80·8)	10 700·0 (9830·0 to 11 500·0)	48·9 (44·1 to 54·7)
	North Korea		13·5 (12·2 to 15·0)	−5·5% (−7·4 to −4·1)	0·10 (0·08 to 0·11)	0·18 (0·15 to 0·20)	76·0 (74·9 to 77·1)	71·3 (70·2 to 72·5)	73·7 (73·0 to 74·6)	230·0 (211·0 to 248·0)	3·9 (3·5 to 4·3)
	Taiwan*		4·5 (4·3 to 4·7)	−2·4% (−2·7 to −2·2)	0·05 (0·05 to 0·05)	0·13 (0·12 to 0·13)	83·9 (83·7 to 84·0)	77·3 (77·2 to 77·5)	80·5 (80·4 to 80·6)	205·0 (203·0 to 208·0)	0·6 (0·6 to 0·7)
Oceania			32·6 (29·8 to 35·5)	−1·8% (−2·2 to −1·4)	0·22 (0·20 to 0·24)	0·25 (0·23 to 0·27)	67·8 (66·9 to 68·6)	65·6 (64·7 to 66·6)	66·6 (66·0 to 67·3)	99·0 (94·7 to 103·0)	14·5 (13·3 to 15·8)
	American Samoa		11·3 (10·2 to 12·5)	−2·0% (−2·5 to −1·5)	0·16 (0·14 to 0·18)	0·24 (0·21 to 0·27)	74·4 (73·2 to 75·5)	68·0 (66·8 to 69·2)	70·9 (70·0 to 71·8)	0·4 (0·4 to 0·4)	0·0 (0·0 to 0·0)
	Cook Islands		8·3 (7·8 to 8·8)	−3·0% (−3·3 to −2·8)	0·12 (0·11 to 0·13)	0·23 (0·21 to 0·24)	76·5 (75·9 to 77·1)	69·7 (69·0 to 70·4)	72·9 (72·5 to 73·4)	0·1 (0·1 to 0·2)	0·0 (0·0 to 0·0)
	Federated States of Micronesia		12·8 (12·1 to 13·5)	−3·5% (−3·8 to −3·2)	0·19 (0·18 to 0·20)	0·31 (0·29 to 0·33)	70·9 (70·3 to 71·5)	63·7 (63·0 to 64·2)	67·0 (66·5 to 67·4)	0·9 (0·8 to 0·9)	0·0 (0·0 to 0·0)
	Fiji		22·3 (21·2 to 23·5)	−0·1% (−0·4 to 0·1)	0·21 (0·19 to 0·22)	0·25 (0·24 to 0·27)	69·5 (68·9 to 70·0)	66·0 (65·4 to 66·5)	67·7 (67·3 to 68·1)	8·4 (8·1 to 8·7)	0·4 (0·4 to 0·4)
	Guam		15·3 (14·5 to 16·3)	0·8% (0·5 to 1·1)	0·12 (0·11 to 0·12)	0·22 (0·21 to 0·24)	79·5 (78·9 to 80·0)	71·9 (71·3 to 72·5)	75·5 (75·0 to 75·9)	1·2 (1·2 to 1·2)	0·0 (0·0 to 0·0)
	Kiribati		37·3 (35·5 to 39·2)	−2·3% (−2·6 to −2·1)	0·16 (0·15 to 0·17)	0·24 (0·23 to 0·26)	74·5 (73·7 to 75·2)	67·6 (66·8 to 68·3)	71·2 (70·7 to 71·7)	0·7 (0·7 to 0·7)	0·1 (0·1 to 0·1)
	Marshall Islands		17·3 (16·4 to 18·3)	−3·2% (−3·5 to −3·0)	0·31 (0·29 to 0·33)	0·33 (0·31 to 0·36)	65·2 (64·5 to 65·9)	63·1 (62·4 to 63·7)	64·1 (63·6 to 64·6)	0·4 (0·3 to 0·4)	0·0 (0·0 to 0·0)
	Nauru		25·8 (24·5 to 27·1)	−2·5% (−2·8 to −2·3)	0·36 (0·34 to 0·38)	0·48 (0·46 to 0·51)	61·1 (60·4 to 61·7)	57·2 (56·5 to 57·9)	59·0 (58·5 to 59·6)	0·1 (0·1 to 0·1)	0·0 (0·0 to 0·0)
	Niue		10·9 (10·3 to 11·5)	−2·3% (−2·6 to −2·1)	0·16 (0·15 to 0·17)	0·24 (0·22 to 0·25)	74·8 (74·2 to 75·4)	69·5 (68·8 to 70·2)	72·3 (71·8 to 72·8)	0·0 (0·0 to 0·0)	0·0 (0·0 to 0·0)
	Northern Mariana Islands		12·4 (11·7 to 13·1)	−1·0% (−1·3 to −0·7)	0·14 (0·13 to 0·15)	0·21 (0·20 to 0·23)	72·8 (72·3 to 73·4)	69·0 (68·5 to 69·7)	70·8 (70·4 to 71·2)	0·4 (0·4 to 0·4)	0·0 (0·0 to 0·0)
	Palau		22·6 (21·4 to 23·9)	−1·1% (−1·4 to −0·8)	0·18 (0·17 to 0·20)	0·21 (0·20 to 0·23)	70·2 (69·6 to 70·8)	67·7 (67·2 to 68·4)	68·9 (68·4 to 69·3)	0·2 (0·2 to 0·2)	0·0 (0·0 to 0·0)
	Papua New Guinea		35·9 (32·6 to 39·4)	−2·1% (−2·6 to −1·6)	0·22 (0·20 to 0·25)	0·23 (0·21 to 0·27)	67·0 (65·8 to 68·2)	65·8 (64·6 to 67·1)	66·3 (65·5 to 67·2)	70·9 (66·8 to 74·9)	12·8 (11·6 to 14·1)
	Samoa		15·8 (14·9 to 16·6)	−2·2% (−2·4 to −1·9)	0·15 (0·14 to 0·17)	0·22 (0·20 to 0·23)	74·5 (73·9 to 75·1)	70·9 (70·2 to 71·6)	72·6 (72·1 to 73·1)	1·4 (1·3 to 1·4)	0·1 (0·1 to 0·1)
	Solomon Islands		9·2 (8·6 to 9·7)	−2·8% (−3·0 to −2·5)	0·29 (0·27 to 0·30)	0·38 (0·36 to 0·41)	66·2 (65·5 to 66·8)	61·9 (61·3 to 62·6)	63·7 (63·3 to 64·2)	7·0 (6·8 to 7·3)	0·2 (0·2 to 0·2)
	Tokelau		10·4 (9·9 to 11·0)	−3·1% (−3·4 to −2·9)	0·11 (0·10 to 0·12)	0·15 (0·14 to 0·16)	76·1 (75·5 to 76·7)	74·7 (74·1 to 75·4)	75·4 (74·9 to 75·8)	0·0 (0·0 to 0·0)	0·0 (0·0 to 0·0)
	Tonga		10·9 (10·3 to 11·5)	−2·0% (−2·2 to −1·7)	0·17 (0·15 to 0·18)	0·20 (0·18 to 0·21)	72·9 (72·3 to 73·6)	70·4 (69·7 to 71·0)	71·7 (71·3 to 72·2)	0·7 (0·7 to 0·8)	0·0 (0·0 to 0·0)
	Tuvalu		22·6 (21·5 to 23·9)	−4·1% (−4·4 to −3·8)	0·25 (0·23 to 0·27)	0·39 (0·37 to 0·41)	66·8 (66·2 to 67·4)	60·4 (59·7 to 61·1)	63·3 (62·8 to 63·7)	0·1 (0·1 to 0·1)	0·0 (0·0 to 0·0)
	Vanuatu		15·5 (14·6 to 16·3)	−2·4% (−2·7 to −2·1)	0·18 (0·17 to 0·19)	0·32 (0·30 to 0·34)	73·3 (72·7 to 74·0)	67·4 (66·6 to 68·2)	70·3 (69·7 to 70·8)	1·9 (1·8 to 2·0)	0·1 (0·1 to 0·1)
Southeast Asia			24·2 (23·0 to 25·5)	−2·8% (−3·0 to −2·5)	0·13 (0·12 to 0·14)	0·21 (0·20 to 0·22)	76·2 (75·7 to 76·7)	70·1 (69·6 to 70·7)	73·1 (72·7 to 73·5)	4940·0 (4810·0 to 5090·0)	261·0 (249·0 to 276·0)
	Cambodia		22·0 (19·8 to 24·2)	−6·1% (−6·6 to −5·6)	0·14 (0·12 to 0·16)	0·20 (0·18 to 0·22)	75·2 (74·1 to 76·3)	70·8 (69·5 to 72·0)	73·1 (72·2 to 73·9)	102·0 (95·9 to 109·0)	7·8 (7·1 to 8·7)
	Indonesia		26·5 (23·9 to 29·4)	−3·1% (−3·6 to −2·6)	0·15 (0·14 to 0·17)	0·20 (0·18 to 0·23)	75·2 (74·0 to 76·4)	70·0 (68·7 to 71·3)	72·5 (71·5 to 73·4)	1880·0 (1750·0 to 2010·0)	116·0 (105·0 to 129·0)
	Laos		46·4 (41·4 to 51·6)	−4·3% (−4·8 to −3·8)	0·21 (0·19 to 0·24)	0·25 (0·22 to 0·28)	67·2 (66·0 to 68·4)	65·2 (63·9 to 66·5)	66·1 (65·2 to 67·0)	60·3 (56·7 to 63·9)	7·8 (6·9 to 8·7)
	Malaysia		7·3 (6·7 to 7·9)	−1·2% (−1·6 to −0·7)	0·09 (0·08 to 0·10)	0·15 (0·15 to 0·16)	78·5 (78·1 to 78·9)	74·6 (74·2 to 75·1)	76·4 (76·1 to 76·7)	192·0 (186·0 to 197·0)	3·2 (3·0 to 3·5)
	Maldives		14·5 (13·7 to 15·5)	−3·9% (−4·1 to −3·6)	0·05 (0·04 to 0·05)	0·07 (0·06 to 0·07)	77·1 (76·5 to 77·6)	75·2 (74·7 to 75·7)	76·1 (75·7 to 76·4)	1·9 (1·9 to 2·0)	0·1 (0·1 to 0·1)
	Mauritius		14·5 (13·7 to 15·4)	−1·0% (−1·3 to −0·7)	0·11 (0·10 to 0·11)	0·21 (0·19 to 0·22)	77·6 (77·1 to 78·0)	70·3 (69·8 to 70·8)	73·8 (73·5 to 74·2)	12·9 (12·5 to 13·2)	0·2 (0·2 to 0·2)
	Myanmar		42·4 (38·2 to 46·8)	−2·9% (−3·4 to −2·4)	0·18 (0·16 to 0·21)	0·33 (0·30 to 0·37)	69·4 (68·2 to 70·5)	61·6 (60·1 to 63·0)	65·4 (64·4 to 66·4)	568·0 (534·0 to 609·0)	46·7 (42·0 to 51·7)
	Philippines		26·8 (24·9 to 28·9)	−1·1% (−1·4 to −0·7)	0·12 (0·12 to 0·12)	0·21 (0·20 to 0·21)	75·4 (75·1 to 75·6)	68·9 (68·6 to 69·2)	72·1 (71·9 to 72·2)	662·0 (655·0 to 669·0)	53·4 (49·5 to 57·7)
	Seychelles		12·7 (12·1 to 13·3)	−1·1% (−1·3 to −0·9)	0·10 (0·09 to 0·10)	0·18 (0·17 to 0·19)	78·5 (78·1 to 79·0)	72·2 (71·7 to 72·7)	75·1 (74·7 to 75·4)	0·8 (0·8 to 0·8)	0·0 (0·0 to 0·0)
	Sri Lanka		8·5 (8·0 to 9·0)	−2·8% (−3·1 to −2·5)	0·08 (0·07 to 0·08)	0·16 (0·15 to 0·16)	79·5 (79·2 to 79·8)	73·6 (73·3 to 73·9)	76·6 (76·4 to 76·8)	182·0 (178·0 to 185·0)	2·2 (2·1 to 2·4)
	Thailand		7·8 (7·4 to 8·3)	−3·2% (−3·5 to −3·0)	0·09 (0·08 to 0·09)	0·21 (0·21 to 0·21)	81·4 (81·2 to 81·7)	73·7 (73·4 to 73·9)	77·5 (77·3 to 77·7)	592·0 (586·0 to 598·0)	4·0 (3·8 to 4·2)
	Timor-Leste		34·9 (31·4 to 38·1)	−3·8% (−4·3 to −3·4)	0·17 (0·16 to 0·20)	0·17 (0·15 to 0·20)	70·1 (68·9 to 71·3)	69·6 (68·4 to 70·9)	69·9 (69·0 to 70·8)	9·1 (8·5 to 9·7)	1·4 (1·3 to 1·6)
	Viet Nam		11·8 (10·6 to 13·2)	−3·7% (−4·2 to −3·1)	0·10 (0·08 to 0·11)	0·18 (0·16 to 0·21)	80·2 (79·0 to 81·5)	71·9 (70·6 to 73·2)	76·1 (75·2 to 77·1)	677·0 (629·0 to 723·0)	17·7 (15·9 to 19·9)
**Sub-Saharan Africa**			**67·7 (67·1 to 68·3)**	**–3·3% (−3·3 to −3·2)**	**0·24 (0·23 to 0·24)**	**0·28 (0·27 to 0·29)**	**66·1 (65·8 to 66·4)**	**62·2 (62·0 to 62·5)**	**64·2 (64·0 to 64·4)**	**8910·0 (8830·0 to 9000·0)**	**2640·0 (2620·0 to 2670·0)**
Central sub-Saharan Africa			66·3 (64·9 to 67·8)	−3·3% (−3·5 to −3·1)	0·28 (0·26 to 0·30)	0·31 (0·29 to 0·33)	63·5 (62·7 to 64·4)	60·0 (59·3 to 60·8)	61·8 (61·3 to 62·4)	1170·0 (1140·0 to 1200·0)	323·0 (316·0 to 331·0)
	Angola		56·9 (55·1 to 58·7)	−4·1% (−4·4 to −3·9)	0·22 (0·20 to 0·25)	0·32 (0·29 to 0·35)	67·9 (66·6 to 69·1)	60·7 (59·5 to 62·0)	64·3 (63·3 to 65·2)	245·0 (235·0 to 256·0)	71·0 (68·7 to 73·3)
	Central African Republic		108·0 (105·0 to 112·0)	−1·7% (−2·0 to −1·5)	0·39 (0·36 to 0·43)	0·39 (0·35 to 0·42)	55·9 (54·6 to 57·1)	54·3 (53·2 to 55·4)	55·0 (54·2 to 55·9)	77·4 (74·6 to 80·2)	29·7 (28·8 to 30·7)
	Congo (Brazzaville)		49·9 (48·2 to 51·4)	−2·7% (−3·0 to −2·5)	0·29 (0·27 to 0·33)	0·30 (0·27 to 0·33)	64·5 (63·3 to 65·8)	62·9 (61·7 to 64·1)	63·7 (62·8 to 64·6)	43·1 (41·1 to 45·2)	7·7 (7·4 to 7·9)
	DR Congo		67·8 (65·7 to 70·0)	−3·2% (−3·5 to −3·0)	0·29 (0·26 to 0·32)	0·31 (0·28 to 0·34)	62·6 (61·4 to 63·8)	60·0 (58·9 to 61·1)	61·3 (60·5 to 62·2)	780·0 (750·0 to 810·0)	210·0 (204·0 to 218·0)
	Equatorial Guinea		50·9 (49·2 to 52·7)	−3·6% (−3·8 to −3·3)	0·33 (0·30 to 0·36)	0·40 (0·37 to 0·44)	63·9 (62·5 to 65·2)	58·6 (57·2 to 59·8)	61·3 (60·4 to 62·3)	11·6 (11·0 to 12·2)	2·1 (2·0 to 2·2)
	Gabon		35·8 (34·6 to 37·0)	−2·5% (−2·7 to −2·3)	0·26 (0·24 to 0·29)	0·26 (0·23 to 0·29)	67·8 (66·6 to 69·0)	66·4 (65·2 to 67·7)	67·0 (66·2 to 67·9)	14·3 (13·5 to 15·0)	1·7 (1·7 to 1·8)
Eastern sub-Saharan Africa			55·4 (54·8 to 56·1)	−3·8% (−3·8 to −3·7)	0·23 (0·23 to 0·24)	0·28 (0·27 to 0·29)	67·3 (66·9 to 67·7)	63·3 (62·9 to 63·7)	65·2 (64·9 to 65·5)	3060·0 (3010·0 to 3110·0)	792·0 (783·0 to 801·0)
	Burundi		79·3 (76·9 to 82·0)	−3·1% (−3·3 to −2·9)	0·25 (0·22 to 0·27)	0·30 (0·27 to 0·33)	64·2 (63·1 to 65·4)	59·8 (58·6 to 61·0)	61·8 (61·0 to 62·6)	114·0 (110·0 to 118·0)	39·9 (38·6 to 41·3)
	Comoros		45·4 (43·9 to 46·8)	−2·4% (−2·6 to −2·1)	0·10 (0·08 to 0·11)	0·12 (0·11 to 0·13)	78·4 (77·2 to 79·5)	74·3 (73·1 to 75·5)	76·3 (75·5 to 77·1)	4·3 (4·1 to 4·5)	1·0 (1·0 to 1·1)
	Djibouti		47·2 (45·6 to 49·0)	−2·3% (−2·5 to −2·0)	0·22 (0·20 to 0·24)	0·24 (0·22 to 0·27)	69·9 (68·7 to 71·2)	67·2 (66·0 to 68·5)	68·4 (67·4 to 69·3)	8·5 (8·1 to 8·9)	1·7 (1·6 to 1·7)
	Eritrea		57·3 (55·4 to 59·2)	−2·1% (−2·3 to −1·8)	0·28 (0·25 to 0·31)	0·30 (0·28 to 0·33)	64·5 (63·2 to 65·8)	61·3 (60·0 to 62·5)	62·9 (61·9 to 63·8)	58·5 (55·8 to 61·4)	11·9 (11·5 to 12·3)
	Ethiopia		53·4 (51·9 to 55·2)	−4·2% (−4·4 to −4·0)	0·24 (0·22 to 0·27)	0·27 (0·24 to 0·30)	66·7 (65·5 to 67·9)	63·0 (61·8 to 64·1)	64·7 (63·9 to 65·6)	793·0 (758·0 to 828·0)	192·0 (186·0 to 198·0)
	Kenya		34·5 (33·4 to 35·7)	−3·8% (−4·0 to −3·5)	0·20 (0·18 to 0·22)	0·24 (0·21 to 0·26)	71·7 (70·5 to 73·0)	68·2 (66·9 to 69·4)	69·9 (69·0 to 70·8)	280·0 (266·0 to 294·0)	41·2 (39·9 to 42·6)
	Madagascar		59·2 (57·2 to 61·2)	−2·3% (−2·6 to −2·1)	0·26 (0·23 to 0·28)	0·28 (0·26 to 0·31)	63·9 (62·8 to 65·0)	61·2 (60·1 to 62·2)	62·5 (61·7 to 63·3)	231·0 (221·0 to 241·0)	58·5 (56·5 to 60·6)
	Malawi		55·0 (53·2 to 56·7)	−4·5% (−4·8 to −4·3)	0·31 (0·28 to 0·34)	0·36 (0·33 to 0·39)	64·3 (63·0 to 65·6)	59·7 (58·5 to 60·8)	62·0 (61·1 to 62·8)	156·0 (149·0 to 163·0)	33·9 (32·8 to 35·0)
	Mozambique		61·6 (59·8 to 63·4)	−3·9% (−4·1 to −3·7)	0·22 (0·20 to 0·24)	0·30 (0·27 to 0·34)	67·7 (66·6 to 68·9)	62·0 (60·8 to 63·2)	64·9 (64·0 to 65·7)	232·0 (223·0 to 241·0)	73·7 (71·5 to 76·0)
	Rwanda		52·2 (50·5 to 53·9)	−5·0% (−5·2 to −4·7)	0·22 (0·20 to 0·24)	0·30 (0·27 to 0·34)	67·6 (66·5 to 68·7)	61·8 (60·7 to 63·0)	64·8 (64·0 to 65·6)	101·0 (96·7 to 106·0)	19·1 (18·5 to 19·8)
	Somalia		82·6 (79·9 to 85·2)	−3·2% (−3·4 to −3·0)	0·20 (0·18 to 0·22)	0·25 (0·23 to 0·27)	67·0 (65·9 to 68·1)	62·6 (61·6 to 63·7)	64·8 (64·1 to 65·6)	174·0 (169·0 to 180·0)	75·6 (73·0 to 78·1)
	South Sudan		105·0 (102·0 to 108·0)	−0·9% (−1·1 to −0·7)	0·32 (0·29 to 0·35)	0·38 (0·35 to 0·42)	59·0 (57·8 to 60·2)	54·1 (52·9 to 55·4)	56·4 (55·5 to 57·2)	104·0 (100·0 to 109·0)	32·4 (31·3 to 33·5)
	Uganda		53·9 (52·1 to 55·5)	−4·1% (−4·3 to −3·9)	0·23 (0·21 to 0·26)	0·29 (0·26 to 0·32)	68·1 (66·9 to 69·3)	63·1 (61·9 to 64·3)	65·6 (64·8 to 66·5)	290·0 (278·0 to 301·0)	86·1 (83·3 to 88·8)
	Tanzania		43·7 (42·3 to 45·1)	−4·4% (−4·7 to −4·2)	0·18 (0·16 to 0·20)	0·22 (0·20 to 0·25)	71·1 (70·0 to 72·2)	67·8 (66·6 to 68·9)	69·4 (68·6 to 70·3)	363·0 (347·0 to 379·0)	90·0 (87·1 to 92·8)
	Zambia		50·9 (49·3 to 52·5)	−4·3% (−4·5 to −4·0)	0·33 (0·30 to 0·37)	0·37 (0·34 to 0·40)	62·6 (61·3 to 63·8)	60·4 (59·1 to 61·6)	61·5 (60·6 to 62·3)	147·0 (141·0 to 154·0)	34·1 (33·0 to 35·2)
Southern sub-Saharan Africa			40·8 (40·1 to 41·4)	−2·5% (−2·6 to −2·3)	0·24 (0·24 to 0·25)	0·34 (0·33 to 0·35)	69·0 (68·7 to 69·4)	62·9 (62·6 to 63·2)	66·1 (65·8 to 66·3)	760·0 (750·0 to 770·0)	70·2 (69·0 to 71·3)
	Botswana		24·5 (23·8 to 25·3)	−3·1% (−3·3 to −3·0)	0·20 (0·19 to 0·21)	0·26 (0·24 to 0·27)	72·7 (72·0 to 73·3)	67·4 (66·7 to 67·9)	70·1 (69·6 to 70·5)	17·1 (16·6 to 17·6)	1·2 (1·2 to 1·3)
	Eswatini		49·4 (47·8 to 51·1)	−2·0% (−2·2 to −1·7)	0·38 (0·35 to 0·42)	0·42 (0·39 to 0·46)	62·4 (61·0 to 63·9)	58·8 (57·4 to 60·2)	60·8 (59·7 to 61·8)	11·8 (11·2 to 12·4)	1·4 (1·3 to 1·4)
	Lesotho		62·5 (60·8 to 64·4)	−2·4% (−2·6 to −2·1)	0·39 (0·36 to 0·43)	0·48 (0·44 to 0·53)	60·4 (59·1 to 61·8)	55·3 (53·9 to 56·7)	57·9 (56·9 to 58·8)	22·8 (21·6 to 24·0)	2·6 (2·6 to 2·7)
	Namibia		38·3 (37·2 to 39·4)	−2·6% (−2·7 to −2·4)	0·25 (0·24 to 0·26)	0·33 (0·31 to 0·35)	69·1 (68·5 to 69·8)	63·4 (62·6 to 64·1)	66·4 (65·9 to 66·9)	21·5 (21·0 to 22·1)	2·6 (2·5 to 2·6)
	South Africa		34·9 (34·1 to 35·6)	−2·8% (−2·9 to −2·6)	0·23 (0·23 to 0·24)	0·34 (0·34 to 0·35)	69·7 (69·4 to 70·1)	63·3 (63·0 to 63·5)	66·5 (66·3 to 66·8)	572·0 (564·0 to 580·0)	37·3 (36·5 to 38·1)
	Zimbabwe		54·1 (52·3 to 55·8)	−2·0% (−2·2 to −1·7)	0·26 (0·23 to 0·28)	0·30 (0·27 to 0·33)	68·4 (67·1 to 69·7)	63·8 (62·5 to 65·1)	66·3 (65·3 to 67·2)	115·0 (110·0 to 121·0)	25·1 (24·2 to 25·9)
Western sub-Saharan Africa			80·4 (79·2 to 81·6)	−3·1% (−3·3 to −3·0)	0·22 (0·21 to 0·24)	0·25 (0·24 to 0·27)	65·5 (65·0 to 66·0)	62·3 (61·8 to 62·8)	63·9 (63·5 to 64·3)	3920·0 (3850·0 to 3990·0)	1460·0 (1430·0 to 1480·0)
	Benin		66·6 (64·3 to 68·7)	−2·8% (−3·1 to −2·6)	0·18 (0·17 to 0·20)	0·26 (0·24 to 0·29)	69·2 (68·1 to 70·3)	63·4 (62·3 to 64·6)	66·2 (65·3 to 67·0)	95·7 (92·3 to 99·5)	33·5 (32·4 to 34·7)
	Burkina Faso		56·0 (54·2 to 57·9)	−4·9% (−5·1 to −4·7)	0·16 (0·14 to 0·18)	0·22 (0·20 to 0·24)	70·1 (69·1 to 71·2)	65·4 (64·3 to 66·5)	67·7 (67·0 to 68·5)	158·0 (152·0 to 164·0)	54·4 (52·6 to 56·2)
	Cabo Verde		25·5 (24·6 to 26·4)	−3·0% (−3·3 to −2·7)	0·09 (0·08 to 0·10)	0·17 (0·16 to 0·20)	78·5 (77·6 to 79·5)	72·8 (71·8 to 73·9)	75·7 (74·9 to 76·4)	2·9 (2·7 to 3·0)	0·2 (0·2 to 0·2)
	Cameroon		68·2 (65·9 to 70·3)	−2·9% (−3·2 to −2·7)	0·28 (0·25 to 0·31)	0·28 (0·25 to 0·31)	62·9 (61·7 to 64·0)	61·1 (59·9 to 62·2)	62·0 (61·2 to 62·8)	262·0 (252·0 to 273·0)	74·4 (71·8 to 76·8)
	Chad		104·0 (101·0 to 107·0)	−2·3% (−2·5 to −2·0)	0·29 (0·26 to 0·32)	0·31 (0·28 to 0·35)	59·7 (58·6 to 60·8)	56·7 (55·6 to 57·8)	58·0 (57·3 to 58·8)	199·0 (193·0 to 205·0)	94·2 (91·4 to 97·1)
	Côte d'Ivoire		69·8 (67·6 to 71·8)	−2·7% (−2·9 to −2·5)	0·25 (0·22 to 0·27)	0·26 (0·24 to 0·29)	66·2 (65·0 to 67·3)	62·6 (61·4 to 63·7)	64·3 (63·4 to 65·1)	237·0 (228·0 to 247·0)	76·8 (74·3 to 79·1)
	The Gambia		54·5 (52·9 to 56·2)	−2·5% (−2·7 to −2·2)	0·21 (0·19 to 0·23)	0·23 (0·20 to 0·25)	68·6 (67·4 to 69·7)	66·1 (64·9 to 67·2)	67·3 (66·5 to 68·0)	16·0 (15·4 to 16·7)	4·2 (4·1 to 4·4)
	Ghana		43·6 (42·2 to 45·0)	−3·5% (−3·7 to −3·2)	0·25 (0·22 to 0·27)	0·30 (0·27 to 0·34)	68·0 (66·9 to 69·2)	62·9 (61·7 to 64·0)	65·4 (64·6 to 66·3)	240·0 (228·0 to 252·0)	39·1 (37·8 to 40·4)
	Guinea		80·6 (78·2 to 83·1)	−3·0% (−3·2 to −2·7)	0·26 (0·24 to 0·29)	0·27 (0·24 to 0·30)	63·4 (62·3 to 64·6)	61·8 (60·6 to 62·9)	62·5 (61·7 to 63·3)	120·0 (115·0 to 124·0)	40·9 (39·6 to 42·3)
	Guinea-Bissau		72·9 (70·7 to 75·1)	−3·3% (−3·5 to −3·0)	0·30 (0·27 to 0·33)	0·30 (0·27 to 0·34)	61·9 (60·7 to 63·0)	60·5 (59·4 to 61·6)	61·2 (60·4 to 62·0)	17·5 (16·8 to 18·1)	5·2 (5·1 to 5·4)
	Liberia		83·7 (81·3 to 86·3)	−3·5% (−3·7 to −3·3)	0·28 (0·26 to 0·31)	0·28 (0·26 to 0·31)	62·7 (61·5 to 63·9)	60·4 (59·3 to 61·5)	61·5 (60·7 to 62·3)	48·3 (46·4 to 50·2)	14·4 (13·9 to 14·8)
	Mali		86·9 (84·2 to 89·7)	−3·4% (−3·6 to −3·1)	0·24 (0·22 to 0·27)	0·25 (0·23 to 0·28)	64·9 (63·8 to 66·1)	62·4 (61·2 to 63·5)	63·6 (62·8 to 64·3)	198·0 (192·0 to 205·0)	88·8 (86·0 to 91·7)
	Mauritania		41·5 (40·1 to 42·9)	−2·7% (−2·9 to −2·4)	0·20 (0·18 to 0·23)	0·20 (0·18 to 0·22)	68·8 (67·6 to 69·8)	67·5 (66·5 to 68·6)	68·1 (67·4 to 68·8)	28·1 (26·8 to 29·4)	5·7 (5·5 to 5·9)
	Niger		117·0 (113·0 to 121·0)	−2·9% (−3·2 to −2·7)	0·23 (0·21 to 0·25)	0·24 (0·21 to 0·26)	60·6 (59·4 to 61·6)	58·2 (57·1 to 59·3)	59·2 (58·4 to 60·0)	272·0 (264·0 to 282·0)	143·0 (138·0 to 148·0)
	Nigeria		85·6 (83·2 to 88·2)	−3·2% (−3·5 to −3·0)	0·21 (0·19 to 0·23)	0·24 (0·21 to 0·26)	65·9 (64·9 to 67·1)	63·1 (61·9 to 64·1)	64·5 (63·7 to 65·3)	1780·0 (1720·0 to 1850·0)	718·0 (697·0 to 740·0)
	São Tomé and Príncipe		34·9 (33·8 to 36·1)	−3·6% (−3·9 to −3·4)	0·17 (0·15 to 0·19)	0·20 (0·17 to 0·22)	72·1 (71·0 to 73·2)	68·6 (67·4 to 69·7)	70·3 (69·5 to 71·2)	1·2 (1·2 to 1·3)	0·2 (0·1 to 0·2)
	Senegal		43·0 (41·6 to 44·4)	−4·3% (−4·6 to −4·1)	0·17 (0·15 to 0·19)	0·20 (0·17 to 0·22)	71·7 (70·7 to 72·9)	68·5 (67·4 to 69·7)	70·1 (69·2 to 70·8)	102·0 (98·3 to 107·0)	24·3 (23·5 to 25·1)
	Sierra Leone		103·0 (99·5 to 106·0)	−3·1% (−3·3 to −2·9)	0·28 (0·25 to 0·31)	0·32 (0·29 to 0·35)	60·8 (59·6 to 61·9)	57·3 (56·1 to 58·5)	58·9 (58·0 to 59·7)	78·4 (75·4 to 81·5)	25·4 (24·6 to 26·3)
	Togo		61·8 (59·9 to 63·9)	−2·9% (−3·1 to −2·6)	0·24 (0·22 to 0·26)	0·28 (0·25 to 0·30)	66·2 (65·1 to 67·4)	62·8 (61·6 to 63·9)	64·5 (63·7 to 65·4)	62·8 (60·0 to 65·6)	14·7 (14·2 to 15·2)

Data in parentheses are 95% uncertainty intervals. GBD=Global Burden of Diseases, Injuries, and Risk Factors Study.

* UN convention recognises Taiwan as a province of China.

**Figure 3:**
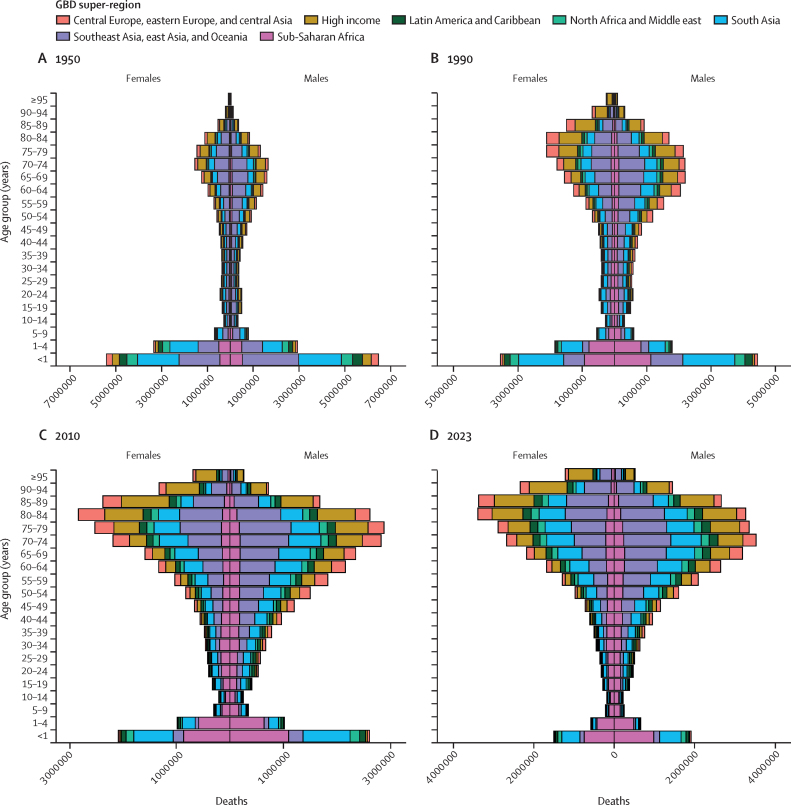
Age-specific deaths by sex and GBD super-region, in 1950 (A), 1990 (B), 2010 (C), and 2023 (D) The number of female deaths (left side) can be compared to male deaths (right side) by age group for four distinct years. The x-axis scales differ by year. Different colours show GBD super-regions. GBD=Global Burden of Diseases, Injuries, and Risk Factors Study.

The distribution of deaths due to all causes combined by age group, sex, and GBD super-region varied substantially over the study period (figure 3). In 1950, deaths in children aged younger than 1 year dominated the global deaths age structure—with 88·9% more deaths than the next-highest age group in the figure—with the largest number of deaths in children younger than 1 year occurring in males in southeast Asia, east Asia, and Oceania (figure 3A). Children aged 1–4 years had the second-largest number of deaths (particularly females in south Asia), followed by those aged 70–74 years. By 1990, the age structure of deaths had begun shifting toward older ages, although the largest number of deaths still occurred in children aged younger than 1 year, with the most deaths now occurring in males in south Asia, followed by those aged 75–79 years (with the most deaths in this age group occurring in males in southeast Asia, east Asia, and Oceania; figure 3B). In 2010, the shift in deaths to older ages continued, with the largest increase from 1990 and the most deaths in 2010 occurring in those aged 80–84 years, followed by, in descending order, those aged 75–79 years, 70–74 years, and those younger than 1 year (figure 3C). By 2010, deaths in children younger than 1 year were highest in sub-Saharan Africa. In 2023, deaths had further shifted to older ages, with the largest number of deaths still occurring in those aged 80–84 years but with nearly as many female deaths occurring in those aged 85–89 years (figure 3D). In 2023, there were approximately half as many deaths in children younger than 1 year as in those aged 80–84 years and just 54·7% as many deaths as in those aged 75–79 years, with the largest proportion of deaths in children younger than 1 year occurring in males in sub-Saharan Africa. Over the entire study period, the absolute number of deaths in children younger than 1 year declined more than in any other age group, although there were still more deaths in those younger than 1 year in 2023 than in any of the 5-year age groups younger than 60 years.

### Age-standardised mortality rates

In 2023, the global age-standardised all-cause mortality rate was 701·5 (95% UI 689·2–713·1) deaths per 100 000, a 66·6% (65·8–67·3) decline from 1950, when the rate was 2098·3 (2067·1–2129·8) deaths per 100 000 (appendix 2 table S3A). Global age-standardised mortality rates in 2023 were significantly lower for females (595·9 [582·0–610·5] per 100 000) than for males (822·1 [801·9–841·5] per 100 000; appendix 2 tables S3B and S3C). At the GBD super-region level, age-standardised mortality rates in 2023 were highest in sub-Saharan Africa (1132·7 [1119·7–1147·6] per 100 000) and lowest in the high-income super-region (443·1 [442·0–444·3]; appendix 2 table S3A). Between 1950 and 2023, the largest decline in age-standardised all-cause mortality rate occurred in southeast Asia, east Asia, and Oceania (78·1% [76·7–79·5] decrease), while the smallest decline occurred in central Europe, eastern Europe, and central Asia (47·0% [46·1–47·8] decrease; appendix 2 table S3A).

Among the GBD countries and territories with populations greater than 1 million, the Central African Republic (1712·9 [95% UI 1630·3–1801·3] per 100 000), South Sudan (1611·1 [1528·8–1696·8] per 100 000), and Chad (1578·6 [1502·5–1662·1] per 100 000) had the highest age-standardised all-cause mortality rates in 2023 (appendix 2 table S3A). The lowest age-standardised all-cause mortality rates occurred in Singapore (301·3 deaths [296·3–306·6] per 100 000), Switzerland (335·1 [331·6–338·4] per 100 000), and Japan (340·8 [340·1–341·5] per 100 000; appendix 2 table S3A). Over the entire 1950–2023 study period, age-standardised mortality rates declined in all 204 countries and territories.

### Age-specific mortality rates

**Figure 4:**
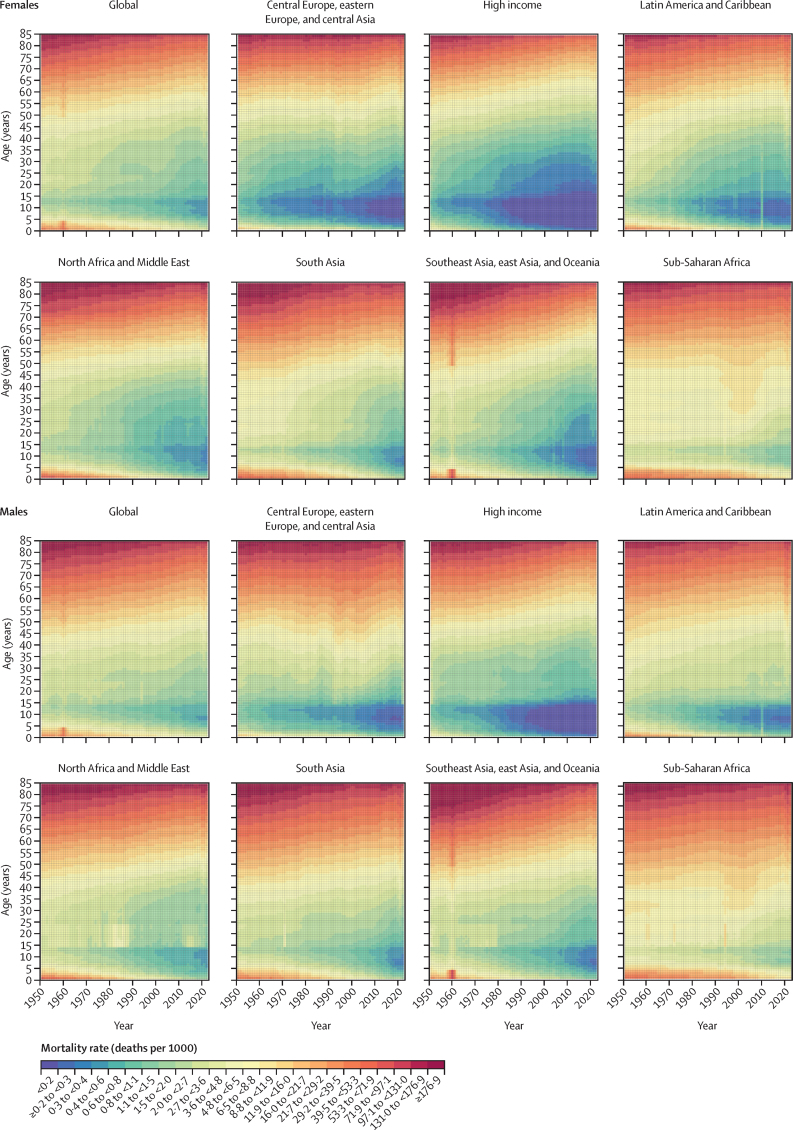
All-cause mortality rates globally and by GBD super-region across the lifespan in females and males, 1950–2023 Mortality rates are expressed as the number of deaths per 1000 population. GBD=Global Burden of Diseases, Injuries, and Risk Factors Study.

For the global populations younger than 15 years and those aged 40 years and older, all-cause mortality rates across the human lifespan broadly declined between 1950 and 2019, and between 2021 and 2023 (figure 4). This pattern was generally consistent across super-regions, with short-term anomalies for events such as war, natural disaster, and famine.^28^ One notable exception to broad trends was increased mortality in sub-Saharan Africa during the 1990s and 2000s due to the HIV/AIDS epidemic in the absence of antiretroviral therapy. Variations in mortality levels and trends across super-regions and over time were observed in those aged 15–39 years, an age group particularly susceptible to mortality shocks.

**Figure 5:**
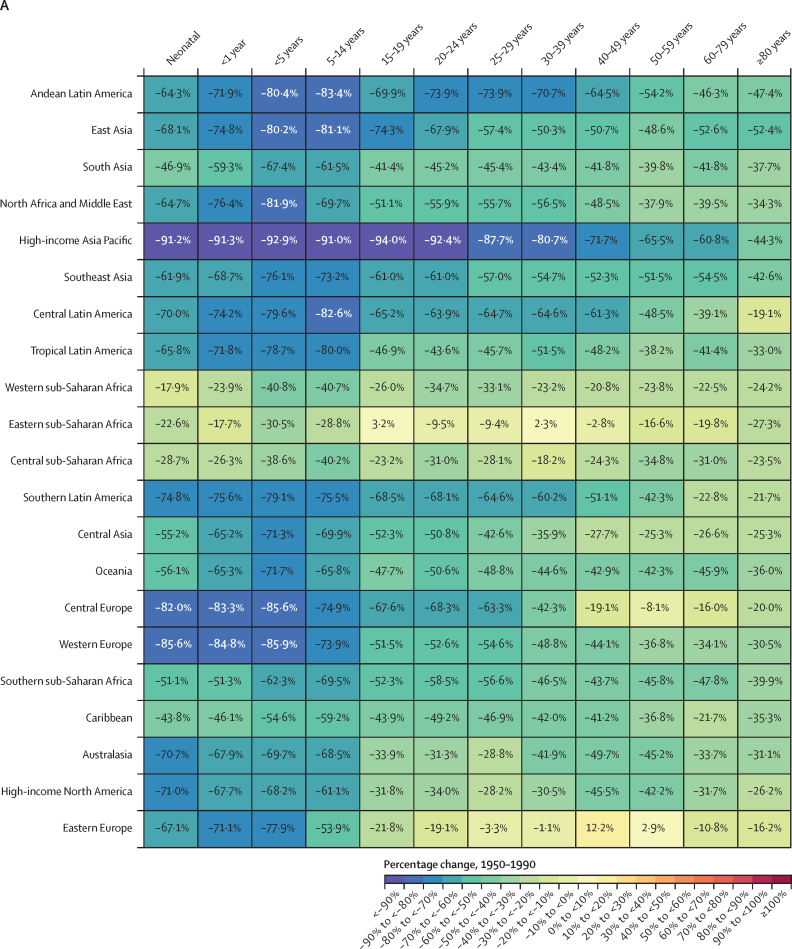

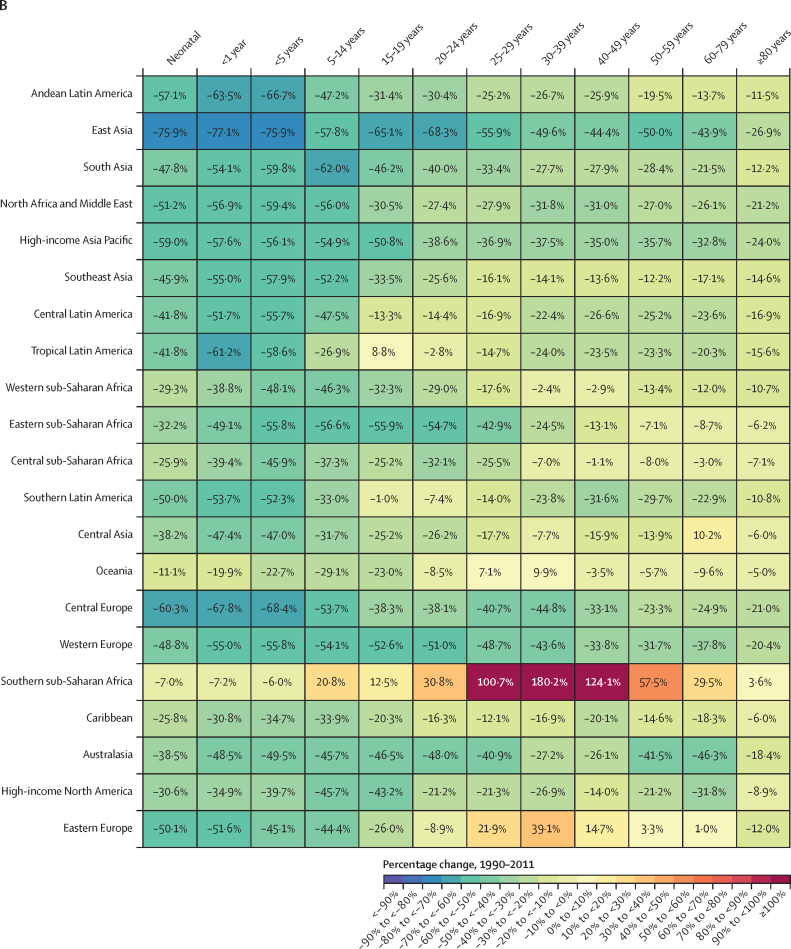

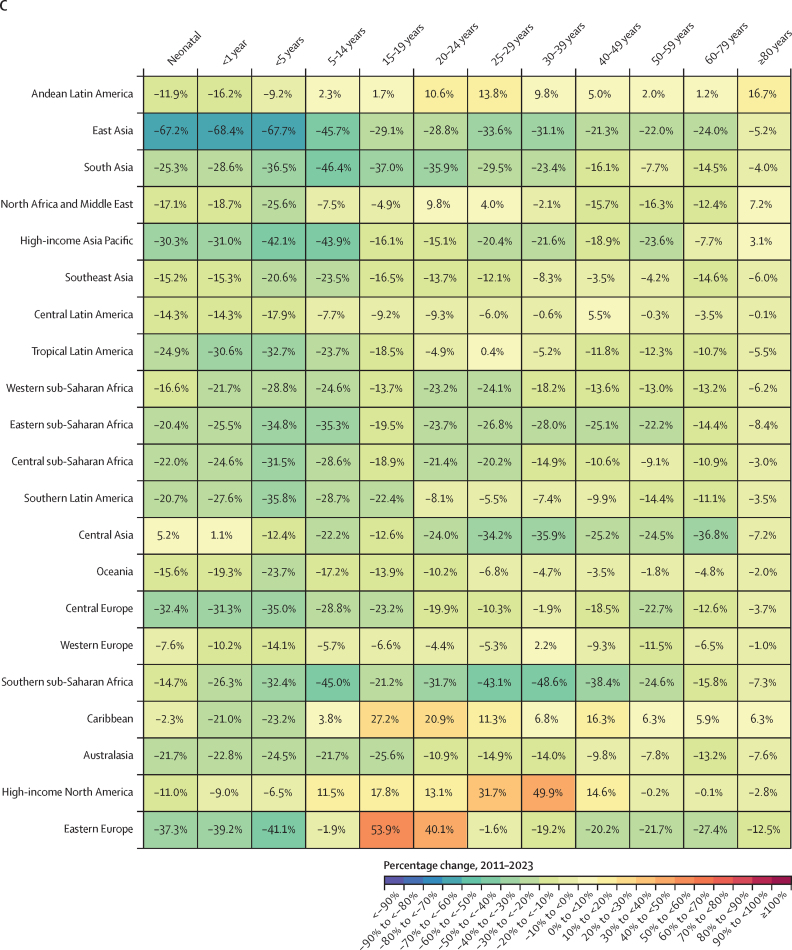
Percentage change in age-specific mortality rates by GBD region, 1950–1990 (A), 1990–2011 (B), and 2011–2023 (C) Percentage change for a year range is calculated as the difference in the estimates between the second year and the first year, divided by the estimate in the first year. The boxes range from blue (indicating a decrease in mortality rate between the 2 years), to yellow (indicating no or minimal change between the 2 years), to red (indicating an increase in mortality rate between the 2 years). Darker colours represent a more substantial change. GBD regions are listed in descending order by greatest increase in life expectancy from 1950 to 2023. GBD=Global Burden of Diseases, Injuries, and Risk Factors Study.

**Figure 6:**
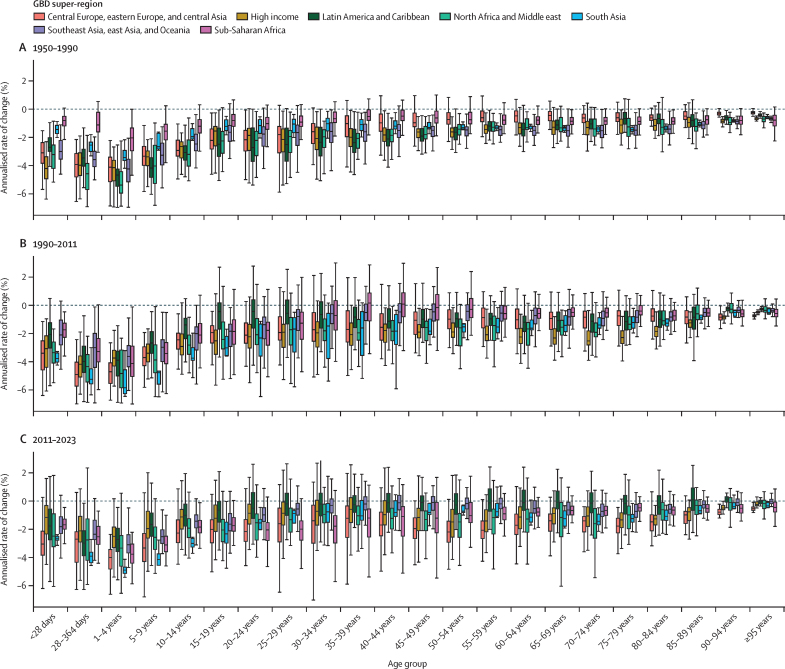
Distribution of ARC in age-specific mortality rates across countries and territories by GBD super-region, 1950–1990 (A), 1990–2011 (B), and 2011–2023 (C) Percentage change for a year range is calculated as the difference in the estimates between the second year and the first year, divided by the estimate in the first year. The boxes represent the middle 50% of the distribution (25th and 75th percentiles), the horizontal line in the boxes indicates the median, and the whiskers show the middle 95% of the distribution (2·5th and 97·5th percentiles). GBD=Global Burden of Diseases, Injuries, and Risk Factors Study.

Between 1950 and 1990, age-specific mortality rates broadly decreased at the regional level (figure 5A). The largest relative decreases (measured by percentage change) were generally observed in the under-5 age groups (as well as in those aged 5–14 years in some regions), the extent of which was less steep in central, eastern, and western sub-Saharan Africa. Increases in mortality rates were observed in eastern Europe in those aged 40–59 years as well as a very small increase in eastern sub-Saharan Africa in those aged 15–19 years. These trends masked substantial variation across countries and territories. Between 1950 and 1990, countries in the sub-Saharan Africa super-region generally had smaller declines in age-specific mortality than other countries, with the median country or territory in sub-Saharan Africa experiencing a smaller annualised rate of decline than the least-improved country or territory in any other super-region for infants aged 28–364 days and children aged 1–4 years (figure 6A). The largest variation in annualised rate of change (ARC) between countries in a single super-region among children and adolescents aged 5–9 years and 10–14 years occurred in north Africa and the Middle East, whereas in those aged 15–19 years and 20–24 years the largest ARC variation was in Latin America and the Caribbean (figure 6A). In adults aged 35–59 years, nearly all the countries and territories with positive ARCs (ie, increasing mortality) were in central Europe, eastern Europe, and central Asia or in sub-Saharan Africa (figure 6A).

Age-specific mortality also generally decreased between 1990 and 2011 across regions, but with several key exceptions (figure 5B, figure 6B). Under-5 mortality rates decreased most sharply in east Asia (75·9% decline), with large declines across all regions except for southern sub-Saharan Africa (6·0% decline). Notable increases in age-specific mortality occurred in eastern Europe in those aged 25–49 years (with smaller increases in those aged 50–79 years) and in southern sub-Saharan Africa in those aged 5 years and older. Between 2011 and 2023, under-5 mortality rates again decreased most sharply in east Asia (67·7% decline) and conversely decreased the least in high-income North America (6·5% decline; figure 5C). High-income North America, Andean Latin America, and the Caribbean saw increases in mortality rates for all age groups between 5–14 years and 40–49 years (figure 5C). High-income North America had the largest increases in mortality rates for those aged 5–14, 25–29 years, and 30–39 years (increases of 11·5%, 31·7%, and 49·9%, respectively; figure 5C). Eastern Europe saw the largest increases in mortality rates for those aged 15–19 years and 20–24 years (53·9% and 40·1%, respectively), while north Africa and the Middle East saw small increases in those aged 20–24 years and 25–29 years (9·8% and 4·0%, respectively; figure 5C). Regions in sub-Saharan Africa saw declines in neonatal mortality during this period that ranged from 14·7% in southern sub-Saharan Africa to 22·0% in central sub-Saharan Africa (figure 5C). At the national level in 2011–23, countries in south Asia had the largest annual declines in age-specific mortality on average in every age group from birth up to age 19 years (figure 6C). In each 5-year age group between 30 years and 74 years, every super-region had at least one country or territory with a positive ARC (ie, an increase) in age-specific mortality, with the exception of South Asia in age groups 45–49 years, 50–54 years, 65–69 years, and 70–74 years. In age groups older than 75 years, countries in central Europe, eastern Europe, and central Asia had the largest annual decreases in mortality on average.

The sub-Saharan Africa super-region saw the largest changes in estimated age-specific mortality rates compared to previous estimates in GBD 2021.^12^ On average across countries and territories over the 1950–2021 time period, mortality rates for all sexes aged 5–14 years were 87·3% higher than estimated in GBD 2021, while mortality rates for females aged 15–29 years were 61·2% higher than previously estimated. Conversely, mortality rates for all sexes aged 50 years and older were 13·2% lower than GBD 2021 on average across countries and territories over the 1950–2021 time period.

### Life expectancy

**Figure 7:**
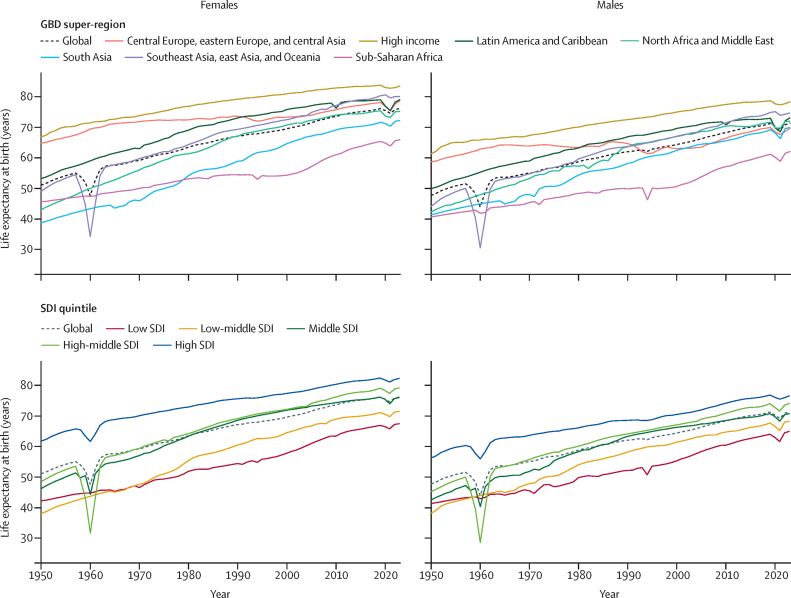
Life expectancy at birth across GBD super-regions and SDI quintiles in females and males, 1950–2023 The different colours represent GBD super-regions in the top row and SDI quintiles in the bottom row. The decline in life expectancy in 1960 for the southeast Asia, east Asia, and Oceania super-region (purple line) was due to famine. GBD=Global Burden of Diseases, Injuries, and Risk Factors Study. SDI=Socio-demographic Index.

Female global life expectancy at birth increased from 51·2 (95% UI 50·6–51·7) years in 1950 to 76·3 (76·0–76·6) years in 2023, an increase of 25·1 (24·5–25·7) years, while male life expectancy increased by 23·6 (23·0–24·2) years over the same period, from 47·9 (47·4–48·4) years to 71·5 (71·2–71·8) years (figure 7; tables 2, 3). Global trends mask substantial heterogeneity between GBD super-regions. In 2023, life expectancy varied from 83·7 (83·7–83·7) years for females and 78·5 (78·5–78·6) years for males in the high-income super-region to 66·1 (65·8–66·4) years for females and 62·2 (62·0–62·5) years for males in sub-Saharan Africa (figure 7; tables 2, 3). Super-regional life expectancy progressed inconsistently over the study period, with stagnation in central Europe, eastern Europe, and central Asia between 1970 and 2000, and in sub-Saharan Africa between 1980 and 2000; these super-regions saw no substantial improvement in life expectancy over these periods. By contrast, the largest absolute increase in female life expectancy occurred in south Asia, nearly doubling between 1950 and 2023, from 38·8 (37·3–40·3) years to 72·4 (71·5–73·3) years, an increase of 33·6 (31·7–35·4) years. For males, the largest absolute increase occurred in southeast Asia, east Asia, and Oceania between 1950 and 2023 (from 44·2 [43·1–45·2] years to 74·8 [74·1–75·6] years, an improvement of 30·6 [29·3–32·1] years). The smallest improvements occurred in males in central Europe, eastern Europe, and central Asia (an increase of 11·0 [10·6–11·4] years, from 58·8 [58·6–59·0] years to 69·9 [69·5–70·1] years). Among countries and territories with populations greater than 1 million, female life expectancy at birth varied in 2023 from 87·5 (87·3–87·8) years in Singapore to 55·9 (54·6–57·1) years in the Central African Republic, as did male life expectancy, from 83·1 (82·9–83·4) years in Singapore to 54·1 (52·9–55·4) years in South Sudan (tables 2, 3).

### Mortality and life expectancy during the COVID-19 pandemic and recovery periods

The number of deaths for all ages and sexes combined in 2023 represents a 8·9% (95% UI 7·2–10·6) decrease from the COVID-19 pandemic era high of 65·9 million (65·6–66·3) deaths in 2021, but an 8·9% (6·8–11·0) increase from 2019 (55·2 million [54·8–55·5]; appendix 2 table S5A). Measured by percentage change in number of deaths, the largest post-pandemic recovery occurred in Tunisia, Bolivia, and Peru, where the number of deaths declined by 37·4% (35·7–39·1), 36·8% (35·1–38·5), and 36·0% (34·6–37·4), respectively, between 2021 and 2023. In total, 162 countries and territories experienced a decrease in deaths over this pandemic recovery period. The number of deaths increased in the remaining 42 countries and territories, with the largest increases occurring in Palestine (101·9% [91·1–113·9]), the United Arab Emirates (14·2% [6·7–22·1]), and Japan (12·1% [11·9–12·3]).

Globally, age-standardised mortality rates increased by 14·0% (95% UI 13·1 to 14·9) from 2019 to 2021, and subsequently declined by 12·4% (10·9 to 13·9) from 2021 to 2023 (appendix 2 table S3A). Super-regional rankings remained unchanged between 2019 and 2023. Between 2019 and 2023, the largest declines occurred in central Europe, eastern Europe, and central Asia (3·4% [2·1 to 4·4] decrease) and sub-Saharan Africa (3·2% [1·7 to 4·5] decrease), while little to no increase occurred in the high-income super-region (2·4% [2·2 to 2·7]); southeast Asia, east Asia, and Oceania (1·8% [–3·8 to 7·5]); and Latin America and the Caribbean (0·7% [0·1 to 1·4]). In the pandemic recovery period (2021–23), age-standardised mortality rates improved for 194 (95·1%) of 204 countries, with the largest declines occurring in Tunisia (40·3% [38·7 to 41·9] improvement), Malawi (39·6% [35·3 to 43·9] improvement), and Namibia (38·7 [37·5-39·9]). Among the remaining ten countries and territories, the largest increases in age-standardised mortality rates between 2021 and 2023 were in Palestine (25·1% [20·3 to 30·5]), Japan (4·7% [4·5 to 5·0]), and New Zealand (4·3% [2·8 to 5·8]). 194 (95·1%) of 204 countries and territories saw at least partial recovery from the pandemic by 2023, with nearly two-thirds (125 [61·3%] of 204) recovering to pre-pandemic (2019) levels (appendix 2 table S3A).

At the global level, the ARC in life expectancy was 0·5% (95% UI 0·5 to 0·5) between 2000 and 2019, and –1·3% (–1·4 to –1·2) between 2019 and 2021 (appendix 2 table S4A), reflecting a decline in overall life expectancy from 76·3 (76·2 to 76·4) years for females and 71·4 (71·3 to 71·5) years for males in 2019 to 74·7 (74·6 to 74·8) years for females and 69·3 (69·2 to 69·4) years for males in 2021 (appendix 2 tables S4B, S4C). Between 2021 and 2023, the ARC was 1·3% (1·1 to 1·5), rising to 76·3 (76·0 to 76·6) years for females and 71·5 (71·2 to 71·8) years for males in 2023 (appendix 2 tables S4B, S4C). These estimates demonstrate a decline in life expectancy in the 2020–21 pandemic period followed by a recovery to 2019 levels by 2023 (figure 7). That said, the impact of the COVID-19 pandemic on life expectancy varied across the world. The ARC during the pandemic years (ie, rates of change from 2019 to 2020 and 2021) varied from –2·7% (–2·8 to –2·7) in Latin America and the Caribbean to just –0·6% (–0·8 to –0·4) in southeast Asia, east Asia, and Oceania (appendix 2 table S4A). Likewise, the largest post-pandemic life expectancy rebound occurred in Latin America and the Caribbean (ARC 2·7% [2·6 to 2·7] in 2021–23), while the smallest rebound occurred in southeast Asia, east Asia, and Oceania (0·4% [0·0 to 0·8] in 2021–23).

At the national level, 157 (77·0%) of 204 countries and territories experienced life expectancy declines over the 2020–21 pandemic period compared to 2019 (appendix 2 table S4A). The largest absolute declines occurred in Malawi (a decrease of 5·6 [95% UI 5·1–6·1] years), Namibia (a decrease of 5·6 [5·5–5·7] years), and Paraguay (a decrease of 5·1 [5·0–5·3] years). Between 2021 and 2023, all but 13 (6·4%) countries and territories saw an increase in life expectancy (appendix 2 table S4A). The largest decrease between 2021 and 2023 occurred in Palestine (a decline of 8·6 [7·7–9·7] years), while the largest increases occurred in Namibia (an increase of 7·7 [7·4–8·1] years), Bolivia (an increase of 7·2 [6·9–7·6] years), and Zimbabwe (an increase of 6·8 [5·7–7·9] years). Similar to rebounds in age-standardised mortality rates, 2023 life expectancy exceeded 2019 life expectancy in nearly two-thirds (130 [63·7%] of 204) of countries and territories, demonstrating that life expectancy has largely returned to and is now exceeding pre-pandemic levels. The largest absolute increases in life expectancy between 2019 and 2023 in countries and territories with populations greater than 1 million occurred in Yemen (an increase of 2·9 [1·9–3·9] years) and Armenia (an increase of 2·3 [1·7–2·8] years).

**Figure 8:**
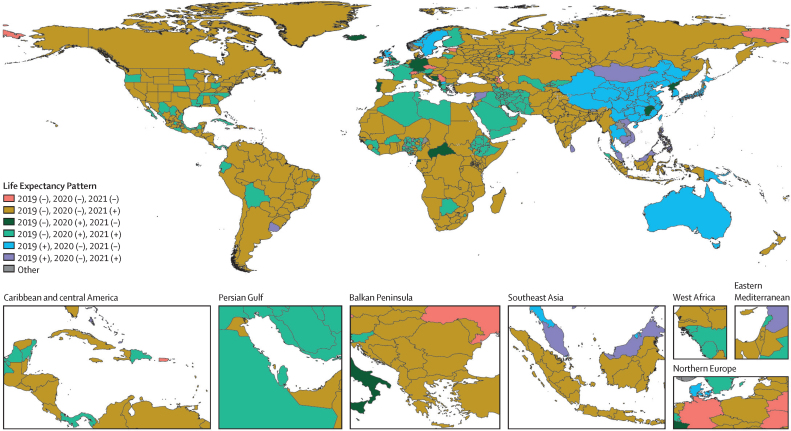
Patterns of location-specific changes in life expectancy during and following the COVID-19 pandemic (2019–22) The life expectancy pattern is based on whether life expectancy increased (+) or decreased (−) between two adjacent years. The + or − after 2019 compares 2019 to 2020, the + or − after 2020 compares 2020 to 2021, and the + or − after 2021 compares 2021 to 2022. For example, the – after 2019 indicates that life expectancy was lower in 2020 compared to 2019. The Other category corresponds to the remaining 2 life expectancy patterns not otherwise listed.

Several major trends emerged when evaluating countries based on yearly increases or decreases in mean life expectancy during the 2020–23 pandemic and recovery periods (figure 8). The majority of countries and territories (126 [61·8%] of 204) had decreases in 2019–20 and 2020–21, followed by an increase in 2021–22. 118 of these countries and territories had a further increase in 2022–23. 33 countries and territories had decreases from 2019 to 2020 followed by increases in 2020–21 and 2021–22, with 29 of these having a further increase in 2022–23. These were the most common patterns, reflecting differences in pandemic severity in differing years across the 2020–2023 period. Additionally, 13 countries and territories had increased life expectancy in 2019–20, decreased life expectancy in 2020–21, and increased life expectancy in 2021–22, while 11 had increased life expectancy in 2019–20, decreased life expectancy in 2020–21, and decreased life expectancy again in 2021–22. Seven countries and territories had decreased life expectancy in all years from 2019 to 2022: Belarus, Canada, Finland, Germany, Portugal, Puerto Rico, and Ukraine. Finally, 11 countries had decreased life expectancy in 2019–20, increased life expectancy in 2020–21, and decreased life expectancy again in 2021–22: Andorra, Chad, Eritrea, France, Iceland, Ireland, Italy, Malta, North Korea, San Marino, and Spain. These varying trajectories were observed within and across super-regions. Countries in sub-Saharan Africa generally had decreases in life expectancy in 2019–20 and 2020–21, followed by an increase in 2021–22, or had a decrease in 2019–20 followed by increases in 2020–21 and 2021–22. Countries that had increased life expectancy from 2019 to 2020 before decreasing in 2021 and 2022 were largely found in the high-income super-region and in southeast Asia, east Asia, and Oceania.

### Estimated life expectancy versus expected life expectancy based on SDI

**Figure 9:**
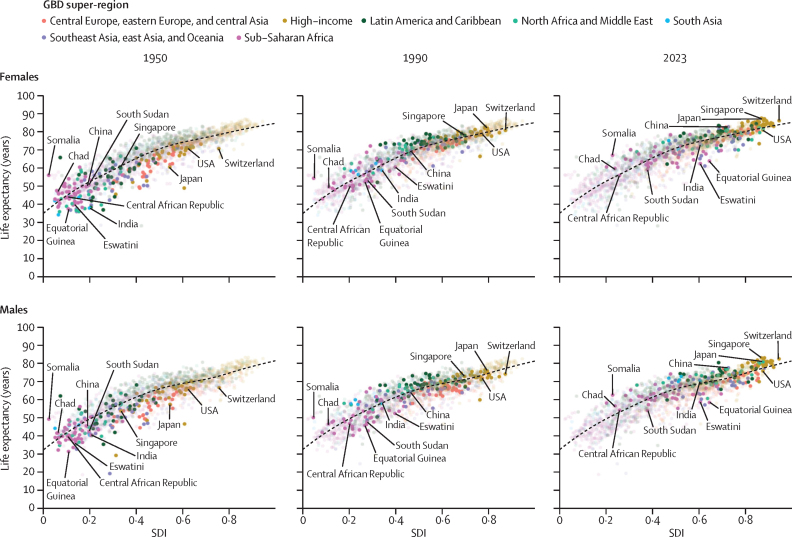
National life expectancy at birth versus SDI, and expected life expectancy based on SDI, in females and males, in 1950, 1990, and 2023 Life expectancy at birth is shown for 204 countries and territories coloured by GBD super-region. Transparent points in all plots show every fifth year between 1950, 2015, and 2023 in the first two columns. The black line represents the expected life expectancy at birth based on SDI, and the shaded area corresponds to 95% uncertainty intervals. The labelled countries are those mentioned in the Results section for having the highest or lowest value of a mortality indicator. GBD=Global Burden of Diseases, Injuries, and Risk Factors Study. SDI=Socio-demographic Index.

Over the 1950–2023 study period, life expectancy at birth was broadly positively associated with SDI level (figure 7, table 4). Likewise, between 1950 and 2023, both life expectancy and SDI increased in all 204 countries and territories. However, there was considerable variation in the relationship between national-level life expectancy (as estimated), and the life expectancy that would be expected solely on the basis of the SDI level for that country (figure 9). In other words, some countries and territories performed better or worse on life expectancy compared to what would be expected based on that location's level of social and economic development. Overall, in 2023, 123 (60·3%) of 204 countries and territories overperformed (ie, estimated life expectancy was higher than expected based on SDI), while the remaining 81 had lower estimated life expectancy than expected based on SDI.

**Table 4:** Life expectancy (estimated, expected based on SDI, and its difference), globally and by SDI quintile, for 1950, 1990, 2000, 2010, and 2023

	**1950**			**1990**			**2000**			**2010**			**2023**		
	Estimated life expectancy	Expected life expectancy	Difference	Estimated life expectancy	Expected life expectancy	Difference	Estimated life expectancy	Expected life expectancy	Difference	Estimated life expectancy	Expected life expectancy	Difference	Estimated life expectancy	Expected life expectancy	Difference
Global	49·5 (49·1 to 49·8)	61·6 (61·6 to 61·7)	−12·2	64·6 (64·5 to 64·8)	69·5 (69·5 to 69·5)	−4·9	67·0 (66·9 to 67·1)	71·0 (70·9 to 71·0)	−4·0	71·0 (70·9 to 71·1)	72·2 (72·2 to 72·3)	−1·2	73·8 (73·6 to 74·1)	73·7 (73·7 to 73·7)	0·1
Low SDI	41·8 (41·4 to 42·3)	46·6 (46·5 to 46·7)	−4·8	53·3 (53·1 to 53·5)	53·8 (53·8 to 53·9)	−0·5	56·6 (56·4 to 56·7)	56·7 (56·6 to 56·7)	−0·1	61·9 (61·8 to 62·1)	60·7 (60·7 to 60·8)	1·2	66·3 (66·0 to 66·6)	66·5 (66·5 to 66·6)	−0·2
Low-middle SDI	38·1 (37·3 to 39·0)	50·3 (50·2 to 50·3)	−12·1	59·4 (59·0 to 59·7)	60·4 (60·4 to 60·5)	−1·0	63·0 (62·8 to 63·2)	63·7 (63·6 to 63·7)	−0·7	66·8 (66·6 to 67·0)	67·0 (67·0 to 67·1)	−0·2	70·0 (69·5 to 70·5)	71·0 (70·9 to 71·0)	−1·0
Middle SDI	44·3 (43·8 to 44·8)	53·2 (53·1 to 53·3)	−8·9	65·9 (65·7 to 66·1)	66·5 (66·5 to 66·6)	−0·6	69·1 (68·9 to 69·2)	69·1 (69·1 to 69·1)	−0·0	71·4 (71·2 to 71·6)	71·0 (70·9 to 71·0)	0·4	73·4 (73·1 to 73·7)	73·2 (73·2 to 73·3)	0·2
High-middle SDI	46·9 (46·2 to 47·6)	54·8 (54·7 to 54·9)	−7·9	66·5 (66·3 to 66·9)	68·1 (68·1 to 68·2)	−1·6	69·6 (69·4 to 69·8)	70·4 (70·3 to 70·4)	−0·8	73·5 (73·3 to 73·7)	72·2 (72·2 to 72·3)	1·3	76·6 (76·2 to 77·0)	74·3 (74·3 to 74·4)	2·2
High SDI	59·1 (58·7 to 59·4)	67·9 (67·9 to 67·9)	−8·8	72·2 (72·0 to 72·3)	73·5 (73·4 to 73·5)	−1·3	74·0 (73·9 to 74·1)	74·8 (74·7 to 74·8)	−0·8	77·3 (77·2 to 77·4)	76·1 (76·1 to 76·2)	1·2	79·5 (79·3 to 79·7)	77·9 (77·8 to 77·9)	1·7

Life expectancies and differences between estimated and expected life expectancies are given in years. A positive difference indicates that the estimated life expectancy is better than would be expected based on the basis of SDI, while a negative difference indicates worse than expected life expectancy. SDI=Socio-demographic Index.

For females in 2023, the super-regions with the largest proportion of countries and territories with a higher than expected life expectancy were the high-income super-region (32 of 36 countries and territories above expected), Latin America and the Caribbean (24 of 33), and central Europe, eastern Europe, and central Asia (18 of 29; table 2). Meanwhile, the largest proportion of countries and territories with lower than expected life expectancy were found in southeast Asia, east Asia, and Oceania (24 of 34 countries and territories below expected), sub-Saharan Africa (29 of 46), and south Asia (three of five). At the national level, the largest positive difference between estimated and expected life expectancy in females in 2023 was in Somalia (12·5 years; ie, it had the largest absolute overperformance in estimated life expectancy compared to SDI-expected life expectancy). Among countries and territories with populations greater than 1 million, the largest negative difference was in Eswatini (–12·4 years; table 2).

**Table 2:** Female life expectancy (estimated, expected based on SDI, and their difference) for 1950, 1990, 2000, 2010, and 2023, and SDI in 2023, globally and for GBD super-regions, regions, and countries and territories

			**1950**			**1990**			**2000**			**2010**			**2023**			**SDI**
			Estimated life expectancy	Expected life expectancy	Difference	Estimated life expectancy	Expected life expectancy	Difference	Estimated life expectancy	Expected life expectancy	Difference	Estimated life expectancy	Expected life expectancy	Difference	Estimated life expectancy	Expected life expectancy	Difference	
**Global**			**51·2 (50·6 to 51·7)**	**63·7 (63·6 to 63·7)**	**–12·5**	**67·3 (67·1 to 67·5)**	**72·2 (72·2 to 72·3)**	**–4·9**	**69·7 (69·6 to 69·8)**	**73·8 (73·8 to 73·9)**	**–4·1**	**73·7 (73·5 to 73·8)**	**75·2 (75·1 to 75·3)**	**–1·5**	**76·3 (76·0 to 76·6)**	**76·8 (76·8 to 76·8)**	**–0·5**	**0·68**
**Central Europe, eastern Europe, and central Asia**			**64·9 (64·7 to 65·2)**	**71·8 (71·7 to 71·8)**	**–6·9**	**73·8 (73·7 to 73·8)**	**75·8 (75·7 to 75·8)**	**–2·0**	**73·5 (73·4 to 73·5)**	**76·9 (76·9 to 77·0)**	**–3·5**	**75·9 (75·9 to 76·0)**	**78·3 (78·2 to 78·3)**	**–2·4**	**78·8 (78·7 to 79·0)**	**79·4 (79·4 to 79·5)**	**–0·6**	**0·78**
Central Asia			58·5 (58·0 to 59·1)	67·6 (67·6 to 67·7)	−9·1	71·1 (71·0 to 71·3)	73·3 (73·3 to 73·4)	−2·2	71·2 (71·0 to 71·4)	74·1 (74·1 to 74·2)	−2·9	73·7 (73·5 to 73·8)	75·6 (75·6 to 75·7)	−2·0	76·8 (76·6 to 77·0)	76·5 (76·5 to 76·6)	0·2	0·67
	Armenia		61·0 (59·4 to 62·4)	68·7 (68·7 to 68·8)	−7·8	73·7 (73·2 to 74·2)	72·8 (72·7 to 72·8)	0·9	75·4 (75·0 to 75·9)	74·1 (74·1 to 74·2)	1·3	77·3 (76·9 to 77·7)	76·2 (76·1 to 76·2)	1·1	82·6 (82·0 to 83·1)	77·7 (77·7 to 77·8)	4·8	0·72
	Azerbaijan		49·0 (47·0 to 50·8)	66·5 (66·4 to 66·6)	−17·5	70·1 (69·6 to 70·6)	74·8 (74·7 to 74·8)	−4·7	71·6 (71·1 to 72·0)	74·1 (74·1 to 74·2)	−2·6	74·1 (73·7 to 74·5)	76·2 (76·1 to 76·2)	−2·0	75·4 (74·7 to 76·1)	77·5 (77·4 to 77·5)	−2·1	0·71
	Georgia		62·6 (61·5 to 63·8)	73·1 (73·1 to 73·2)	−10·5	73·1 (72·7 to 73·5)	76·4 (76·4 to 76·5)	−3·3	73·1 (72·5 to 73·7)	75·6 (75·6 to 75·7)	−2·5	76·4 (75·9 to 76·8)	76·8 (76·8 to 76·8)	−0·4	79·4 (78·9 to 79·9)	78·8 (78·8 to 78·9)	0·6	0·75
	Kazakhstan		64·2 (63·1 to 65·4)	68·7 (68·7 to 68·8)	−4·5	72·2 (71·9 to 72·5)	74·5 (74·4 to 74·5)	−2·3	71·2 (70·9 to 71·5)	76·0 (76·0 to 76·1)	−4·8	73·6 (73·2 to 73·9)	77·1 (77·0 to 77·1)	−3·5	78·7 (78·3 to 79·1)	78·0 (78·0 to 78·1)	0·7	0·73
	Kyrgyzstan		56·8 (55·3 to 58·3)	67·6 (67·6 to 67·7)	−10·9	70·8 (70·3 to 71·3)	72·0 (72·0 to 72·1)	−1·2	71·2 (70·8 to 71·7)	72·8 (72·7 to 72·8)	−1·5	73·5 (73·0 to 73·9)	73·1 (73·1 to 73·2)	0·3	77·8 (77·2 to 78·5)	75·3 (75·3 to 75·4)	2·5	0·63
	Mongolia		48·0 (46·2 to 49·8)	59·0 (59·0 to 59·2)	−11·0	65·1 (64·4 to 65·7)	69·8 (69·7 to 69·8)	−4·7	66·9 (66·3 to 67·5)	72·6 (72·5 to 72·6)	−5·7	71·6 (71·0 to 72·1)	74·5 (74·4 to 74·5)	−2·9	78·2 (77·4 to 79·0)	76·3 (76·2 to 76·3)	1·9	0·66
	Tajikistan		52·7 (51·1 to 54·4)	60·0 (60·0 to 60·1)	−7·3	69·5 (68·9 to 70·0)	70·0 (69·9 to 70·0)	−0·5	70·4 (69·8 to 71·0)	68·7 (68·7 to 68·8)	1·7	73·6 (73·1 to 74·0)	70·9 (70·9 to 71·0)	2·6	73·0 (71·9 to 74·1)	73·0 (72·9 to 73·0)	0·1	0·55
	Turkmenistan		55·4 (53·9 to 56·9)	67·9 (67·8 to 68·0)	−12·5	69·4 (68·9 to 70·0)	73·8 (73·8 to 73·9)	−4·4	70·2 (69·7 to 70·8)	74·0 (73·9 to 74·0)	−3·8	72·9 (72·4 to 73·5)	75·5 (75·4 to 75·5)	−2·5	74·1 (72·8 to 75·3)	76·8 (76·8 to 76·8)	−2·7	0·68
	Uzbekistan		59·2 (57·8 to 60·4)	63·7 (63·6 to 63·7)	−4·5	71·2 (70·9 to 71·6)	71·4 (71·3 to 71·4)	−0·1	71·2 (70·8 to 71·5)	73·3 (73·3 to 73·4)	−2·1	72·8 (72·5 to 73·1)	75·1 (75·0 to 75·1)	−2·3	75·0 (74·6 to 75·4)	75·5 (75·4 to 75·5)	−0·4	0·63
Central Europe			61·7 (61·5 to 61·9)	69·8 (69·7 to 69·8)	−8·1	74·6 (74·6 to 74·7)	75·6 (75·6 to 75·7)	−1·0	76·5 (76·5 to 76·6)	77·2 (77·2 to 77·2)	−0·7	79·0 (79·0 to 79·1)	78·8 (78·8 to 78·9)	0·2	81·0 (80·9 to 81·1)	80·4 (80·4 to 80·5)	0·6	0·81
	Albania		56·3 (54·8 to 57·7)	62·0 (62·0 to 62·1)	−5·7	74·9 (74·4 to 75·5)	72·8 (72·7 to 72·8)	2·2	78·1 (77·6 to 78·5)	73·7 (73·6 to 73·7)	4·4	80·5 (79·9 to 81·0)	75·8 (75·7 to 75·8)	4·7	82·1 (81·4 to 82·7)	77·5 (77·4 to 77·5)	4·6	0·71
	Bosnia and Herzegovina		53·0 (52·4 to 53·7)	59·4 (59·3 to 59·5)	−6·4	75·3 (75·0 to 75·6)	72·0 (72·0 to 72·1)	3·3	77·3 (76·9 to 77·6)	74·1 (74·1 to 74·2)	3·1	78·6 (78·3 to 78·8)	76·3 (76·2 to 76·3)	2·3	80·1 (79·4 to 80·8)	77·7 (77·7 to 77·8)	2·3	0·71
	Bulgaria		61·6 (61·2 to 62·0)	68·5 (68·4 to 68·5)	−6·9	74·7 (74·6 to 74·9)	75·3 (75·3 to 75·4)	−0·6	75·1 (75·0 to 75·3)	76·7 (76·6 to 76·7)	−1·5	77·6 (77·4 to 77·7)	78·1 (78·1 to 78·2)	−0·6	79·5 (79·3 to 79·7)	79·3 (79·2 to 79·3)	0·2	0·77
	Croatia		58·4 (58·0 to 58·6)	69·3 (69·2 to 69·3)	−10·9	76·2 (76·0 to 76·3)	76·3 (76·2 to 76·3)	−0·1	78·0 (77·8 to 78·1)	77·1 (77·0 to 77·1)	0·9	79·8 (79·7 to 80·0)	78·4 (78·4 to 78·5)	1·4	81·7 (81·4 to 81·9)	79·6 (79·5 to 79·6)	2·1	0·78
	Czechia		67·5 (67·3 to 67·7)	73·3 (73·3 to 73·4)	−5·8	75·4 (75·3 to 75·5)	76·7 (76·6 to 76·7)	−1·3	78·4 (78·2 to 78·5)	79·0 (78·9 to 79·1)	−0·6	80·7 (80·6 to 80·8)	80·1 (80·1 to 80·2)	0·5	82·8 (82·6 to 82·9)	81·0 (81·0 to 81·0)	1·8	0·83
	Hungary		65·3 (64·9 to 65·7)	70·7 (70·7 to 70·8)	−5·4	73·8 (73·7 to 73·9)	75·9 (75·8 to 75·9)	−2·1	76·1 (75·9 to 76·2)	77·6 (77·6 to 77·6)	−1·5	78·4 (78·2 to 78·5)	79·0 (78·9 to 79·1)	−0·6	80·1 (79·9 to 80·2)	80·0 (80·0 to 80·1)	0·1	0·79
	Montenegro		63·0 (62·5 to 63·5)	68·5 (68·4 to 68·5)	−5·5	75·9 (75·7 to 76·2)	76·4 (76·4 to 76·5)	−0·5	75·9 (75·7 to 76·1)	76·4 (76·4 to 76·5)	−0·5	77·5 (77·3 to 77·8)	78·1 (78·1 to 78·2)	−0·6	79·5 (79·1 to 79·9)	79·9 (79·8 to 79·9)	−0·3	0·79
	North Macedonia		49·6 (49·0 to 50·2)	66·5 (66·4 to 66·6)	−16·9	73·3 (73·1 to 73·6)	74·8 (74·7 to 74·8)	−1·4	75·0 (74·7 to 75·2)	75·9 (75·8 to 75·9)	−0·9	76·8 (76·6 to 77·0)	77·5 (77·4 to 77·5)	−0·6	78·9 (78·5 to 79·4)	79·0 (78·9 to 79·1)	−0·0	0·76
	Poland		62·5 (62·3 to 62·8)	70·7 (70·7 to 70·8)	−8·2	75·7 (75·6 to 75·8)	75·6 (75·6 to 75·7)	0·1	78·1 (78·0 to 78·2)	77·7 (77·7 to 77·8)	0·3	80·5 (80·4 to 80·6)	79·6 (79·5 to 79·6)	0·9	82·2 (82·1 to 82·4)	81·4 (81·4 to 81·4)	0·8	0·85
	Romania		63·0 (62·3 to 63·6)	66·5 (66·4 to 66·6)	−3·5	73·3 (73·2 to 73·5)	74·9 (74·9 to 75·0)	−1·6	74·7 (74·5 to 74·8)	76·2 (76·1 to 76·2)	−1·5	77·7 (77·5 to 77·8)	77·6 (77·6 to 77·6)	0·1	80·1 (80·0 to 80·3)	79·3 (79·2 to 79·3)	0·9	0·77
	Serbia		55·3 (54·6 to 55·9)	67·4 (67·3 to 67·4)	−12·0	73·0 (72·7 to 73·3)	75·5 (75·4 to 75·5)	−2·5	74·1 (73·8 to 74·3)	76·0 (76·0 to 76·1)	−2·0	76·6 (76·4 to 76·8)	78·0 (78·0 to 78·1)	−1·4	78·2 (77·4 to 78·9)	79·7 (79·7 to 79·8)	−1·6	0·78
	Slovakia		64·2 (63·8 to 64·6)	72·0 (72·0 to 72·1)	−7·8	75·5 (75·3 to 75·6)	76·0 (76·0 to 76·1)	−0·6	77·4 (77·3 to 77·6)	78·1 (78·1 to 78·2)	−0·7	79·3 (79·1 to 79·4)	79·6 (79·5 to 79·6)	−0·3	81·4 (81·2 to 81·7)	80·6 (80·6 to 80·6)	0·9	0·81
	Slovenia		62·2 (61·7 to 62·6)	72·8 (72·7 to 72·8)	−10·6	77·2 (77·1 to 77·4)	77·9 (77·8 to 77·9)	−0·6	79·7 (79·5 to 79·8)	79·3 (79·2 to 79·3)	0·4	82·5 (82·3 to 82·6)	80·6 (80·6 to 80·6)	1·9	84·4 (84·1 to 84·6)	81·4 (81·4 to 81·4)	2·9	0·84
Eastern Europe			68·1 (67·7 to 68·5)	73·1 (73·1 to 73·2)	−5·0	74·5 (74·5 to 74·6)	76·5 (76·5 to 76·6)	−2·0	73·1 (73·0 to 73·1)	77·6 (77·6 to 77·6)	−4·5	75·2 (75·2 to 75·3)	79·1 (79·1 to 79·2)	−3·9	78·9 (78·6 to 79·1)	80·6 (80·6 to 80·6)	−1·7	0·81
	Belarus		70·5 (69·9 to 71·1)	70·0 (69·9 to 70·0)	0·5	75·6 (75·4 to 75·7)	75·5 (75·4 to 75·5)	0·1	74·4 (74·2 to 74·5)	76·7 (76·6 to 76·7)	−2·3	76·5 (76·4 to 76·7)	78·6 (78·5 to 78·6)	−2·0	79·2 (78·6 to 79·7)	80·3 (80·3 to 80·3)	−1·1	0·80
	Estonia		70·6 (70·1 to 71·0)	73·3 (73·3 to 73·4)	−2·7	74·9 (74·7 to 75·1)	76·7 (76·6 to 76·7)	−1·8	76·4 (76·2 to 76·6)	78·4 (78·4 to 78·5)	−2·0	80·3 (80·1 to 80·4)	80·1 (80·1 to 80·2)	0·1	82·9 (82·6 to 83·2)	81·7 (81·7 to 81·7)	1·2	0·85
	Latvia		72·1 (71·7 to 72·6)	73·8 (73·8 to 73·9)	−1·7	74·8 (74·6 to 75·0)	76·9 (76·9 to 77·0)	−2·1	75·7 (75·5 to 75·9)	78·3 (78·2 to 78·3)	−2·6	78·1 (77·9 to 78·3)	80·1 (80·1 to 80·2)	−2·0	80·6 (80·3 to 80·9)	81·6 (81·5 to 81·6)	−0·9	0·85
	Lithuania		67·5 (66·9 to 67·9)	70·0 (69·9 to 70·0)	−2·5	75·8 (75·6 to 75·9)	76·3 (76·2 to 76·3)	−0·5	77·2 (77·0 to 77·4)	77·7 (77·7 to 77·8)	−0·5	78·4 (78·2 to 78·6)	79·7 (79·7 to 79·8)	−1·3	81·2 (80·9 to 81·5)	81·7 (81·7 to 81·7)	−0·5	0·86
	Moldova		57·8 (56·4 to 59·1)	67·4 (67·3 to 67·4)	−9·6	71·5 (71·1 to 71·9)	74·5 (74·4 to 74·5)	−2·9	72·6 (72·2 to 73·0)	75·1 (75·0 to 75·1)	−2·4	74·7 (74·2 to 75·1)	76·3 (76·2 to 76·3)	−1·6	80·3 (79·6 to 81·0)	78·3 (78·2 to 78·3)	2·0	0·73
	Russia		67·8 (67·2 to 68·3)	73·1 (73·1 to 73·2)	−5·3	74·4 (74·3 to 74·4)	76·8 (76·8 to 76·8)	−2·4	72·6 (72·5 to 72·7)	77·9 (77·8 to 77·9)	−5·3	75·0 (75·0 to 75·1)	79·3 (79·2 to 79·3)	−4·2	78·8 (78·6 to 78·9)	80·9 (80·9 to 80·9)	−2·1	0·82
	Ukraine		69·2 (68·2 to 70·2)	73·5 (73·4 to 73·5)	−4·3	75·0 (74·8 to 75·1)	76·2 (76·1 to 76·2)	−1·2	73·8 (73·6 to 73·9)	76·7 (76·6 to 76·7)	−2·9	75·2 (75·1 to 75·4)	78·0 (78·0 to 78·1)	−2·8	78·5 (77·5 to 79·5)	79·1 (79·1 to 79·2)	−0·7	0·76
**High income**			**67·0 (66·9 to 67·1)**	**74·1 (74·1 to 74·2)**	**–7·1**	**79·4 (79·3 to 79·4)**	**79·0 (78·9 to 79·1)**	**0·4**	**81·2 (81·1 to 81·2)**	**80·0 (80·0 to 80·1)**	**1·2**	**83·0 (83·0 to 83·1)**	**80·9 (80·9 to 80·9)**	**2·2**	**83·7 (83·7 to 83·7)**	**82·0 (81·9 to 82·0)**	**1·7**	**0·87**
Australasia			71·9 (71·8 to 72·0)	73·7 (73·6 to 73·7)	−1·8	79·6 (79·6 to 79·7)	78·3 (78·2 to 78·3)	1·4	82·0 (82·0 to 82·1)	79·4 (79·4 to 79·5)	2·6	84·0 (83·9 to 84·0)	80·3 (80·3 to 80·3)	3·7	85·4 (85·4 to 85·5)	81·7 (81·7 to 81·7)	3·7	0·86
	Australia		72·0 (71·8 to 72·2)	73·3 (73·3 to 73·4)	−1·3	79·9 (79·8 to 80·0)	78·1 (78·1 to 78·2)	1·8	82·3 (82·2 to 82·4)	79·3 (79·2 to 79·3)	3·0	84·2 (84·1 to 84·3)	80·3 (80·3 to 80·3)	3·9	85·8 (85·8 to 85·9)	81·7 (81·7 to 81·7)	4·1	0·85
	New Zealand		71·4 (71·1 to 71·7)	74·5 (74·4 to 74·5)	−3·0	78·4 (78·2 to 78·6)	78·8 (78·8 to 78·9)	−0·4	80·9 (80·7 to 81·0)	79·9 (79·8 to 79·9)	1·0	82·8 (82·6 to 83·0)	80·6 (80·6 to 80·6)	2·2	83·4 (83·2 to 83·6)	82·0 (81·9 to 82·0)	1·4	0·87
High-income Asia Pacific			57·3 (56·8 to 57·7)	71·4 (71·3 to 71·4)	−14·1	80·8 (80·7 to 80·9)	79·4 (79·4 to 79·5)	1·4	83·7 (83·6 to 83·8)	80·7 (80·7 to 80·7)	3·0	86·0 (85·9 to 86·0)	81·4 (81·4 to 81·4)	4·5	87·1 (87·0 to 87·1)	82·4 (82·3 to 82·4)	4·7	0·88
	Brunei		52·3 (51·5 to 53·2)	64·6 (64·6 to 64·7)	−12·3	73·8 (73·5 to 74·2)	76·7 (76·6 to 76·7)	−2·8	75·6 (75·3 to 76·0)	78·3 (78·2 to 78·3)	−2·6	78·0 (77·7 to 78·3)	79·9 (79·8 to 79·9)	−1·8	79·4 (78·8 to 80·0)	81·0 (81·0 to 81·0)	−1·6	0·83
	Japan		60·8 (60·7 to 60·9)	72·8 (72·7 to 72·8)	−12·0	82·1 (82·1 to 82·2)	80·0 (80·0 to 80·1)	2·1	84·6 (84·6 to 84·7)	80·9 (80·9 to 80·9)	3·8	86·4 (86·4 to 86·4)	81·4 (81·4 to 81·4)	5·0	87·2 (87·2 to 87·2)	82·2 (82·2 to 82·3)	4·9	0·88
	Singapore		60·8 (60·3 to 61·2)	61·4 (61·3 to 61·4)	−0·6	78·3 (78·1 to 78·5)	77·3 (77·3 to 77·4)	1·0	81·5 (81·3 to 81·8)	79·6 (79·5 to 79·6)	2·0	85·1 (84·9 to 85·3)	81·3 (81·3 to 81·3)	3·8	87·5 (87·3 to 87·8)	82·1 (82·1 to 82·1)	5·4	0·87
	South Korea		45·6 (43·9 to 47·0)	60·0 (60·0 to 60·1)	−14·5	76·0 (75·7 to 76·3)	77·3 (77·3 to 77·4)	−1·4	79·7 (79·4 to 79·9)	79·9 (79·8 to 79·9)	−0·2	84·0 (83·7 to 84·2)	81·4 (81·4 to 81·4)	2·5	86·1 (85·9 to 86·2)	82·6 (82·6 to 82·7)	3·4	0·89
High-income North America			71·3 (71·2 to 71·3)	75·3 (75·3 to 75·4)	−4·0	79·0 (79·0 to 79·0)	79·4 (79·4 to 79·5)	−0·4	79·7 (79·6 to 79·7)	80·3 (80·3 to 80·3)	−0·6	81·3 (81·3 to 81·3)	81·1 (81·1 to 81·2)	0·2	81·4 (81·4 to 81·5)	82·4 (82·3 to 82·4)	−0·9	0·88
	Canada		70·9 (70·7 to 71·0)	75·6 (75·6 to 75·7)	−4·7	80·6 (80·5 to 80·6)	80·1 (80·1 to 80·2)	0·4	81·9 (81·8 to 82·0)	81·1 (81·1 to 81·2)	0·8	83·6 (83·6 to 83·7)	81·8 (81·8 to 81·9)	1·8	84·1 (83·8 to 84·4)	82·5 (82·4 to 82·5)	1·6	0·89
	Greenland		49·0 (48·2 to 49·8)	74·9 (74·9 to 75·0)	−25·9	66·6 (66·2 to 67·0)	79·1 (79·1 to 79·2)	−12·5	67·6 (67·2 to 68·0)	79·1 (79·1 to 79·2)	−11·5	70·6 (70·2 to 71·0)	80·9 (80·9 to 80·9)	−10·2	73·4 (72·8 to 74·1)	81·8 (81·8 to 81·9)	−8·4	0·86
	USA		71·4 (71·3 to 71·4)	75·3 (75·3 to 75·4)	−4·0	78·9 (78·8 to 78·9)	79·4 (79·4 to 79·5)	−0·6	79·4 (79·4 to 79·5)	80·1 (80·1 to 80·2)	−0·7	81·0 (81·0 to 81·1)	81·1 (81·1 to 81·2)	−0·1	81·1 (81·1 to 81·1)	82·2 (82·2 to 82·3)	−1·1	0·88
Southern Latin America			62·6 (62·4 to 62·8)	70·5 (70·4 to 70·5)	−7·9	76·2 (76·1 to 76·3)	74·8 (74·7 to 74·8)	1·4	78·5 (78·4 to 78·6)	76·3 (76·2 to 76·3)	2·2	79·6 (79·6 to 79·7)	77·2 (77·2 to 77·2)	2·4	81·1 (80·8 to 81·4)	79·3 (79·2 to 79·3)	1·8	0·77
	Argentina		66·1 (65·9 to 66·3)	71·2 (71·1 to 71·2)	−5·1	75·8 (75·7 to 75·9)	74·9 (74·9 to 75·0)	0·9	77·9 (77·8 to 78·0)	76·3 (76·2 to 76·3)	1·6	78·9 (78·8 to 79·0)	77·1 (77·0 to 77·1)	1·8	80·3 (79·9 to 80·7)	79·1 (79·1 to 79·2)	1·2	0·76
	Chile		53·0 (52·7 to 53·3)	68·2 (68·1 to 68·3)	−15·2	76·9 (76·7 to 77·0)	74·5 (74·4 to 74·5)	2·4	80·1 (79·9 to 80·2)	76·3 (76·2 to 76·3)	3·8	81·6 (81·4 to 81·7)	77·6 (77·6 to 77·6)	4·0	83·3 (83·1 to 83·4)	79·9 (79·8 to 79·9)	3·4	0·79
	Uruguay		68·2 (67·8 to 68·6)	70·5 (70·4 to 70·5)	−2·3	76·8 (76·6 to 77·0)	74·5 (74·4 to 74·5)	2·3	78·5 (78·3 to 78·7)	75·6 (75·6 to 75·7)	2·9	80·0 (79·8 to 80·2)	76·8 (76·8 to 76·8)	3·2	80·6 (80·3 to 80·8)	78·6 (78·5 to 78·6)	2·0	0·75
Western Europe			69·0 (68·9 to 69·0)	73·7 (73·6 to 73·7)	−4·7	79·6 (79·5 to 79·6)	78·8 (78·8 to 78·9)	0·7	81·6 (81·6 to 81·6)	80·0 (80·0 to 80·1)	1·6	83·5 (83·5 to 83·5)	80·9 (80·9 to 80·9)	2·6	84·4 (84·3 to 84·4)	82·0 (81·9 to 82·0)	2·4	0·86
	Andorra		74·3 (73·7 to 74·9)	74·5 (74·4 to 74·5)	−0·1	83·1 (82·7 to 83·4)	79·7 (79·7 to 79·8)	3·4	84·2 (83·9 to 84·5)	80·4 (80·4 to 80·5)	3·8	85·7 (85·3 to 86·0)	81·6 (81·5 to 81·6)	4·1	86·2 (85·6 to 86·8)	82·4 (82·3 to 82·4)	3·8	0·88
	Austria		67·9 (67·7 to 68·1)	74·9 (74·9 to 75·0)	−7·0	79·0 (78·9 to 79·1)	78·7 (78·6 to 78·8)	0·3	81·2 (81·1 to 81·3)	79·9 (79·8 to 79·9)	1·3	83·1 (83·0 to 83·2)	80·7 (80·7 to 80·7)	2·3	84·2 (84·1 to 84·4)	81·7 (81·7 to 81·7)	2·5	0·85
	Belgium		70·8 (70·4 to 71·2)	73·7 (73·6 to 73·7)	−2·9	79·3 (79·2 to 79·4)	78·8 (78·8 to 78·9)	0·4	81·0 (80·9 to 81·1)	80·0 (80·0 to 80·1)	1·0	82·6 (82·5 to 82·7)	81·0 (81·0 to 81·0)	1·6	84·2 (84·1 to 84·3)	82·2 (82·2 to 82·3)	2·0	0·88
	Cyprus		64·9 (63·8 to 66·0)	67·1 (67·0 to 67·2)	−2·2	77·4 (76·7 to 78·1)	76·0 (76·0 to 76·1)	1·4	79·4 (78·7 to 80·1)	78·7 (78·6 to 78·8)	0·7	81·6 (80·9 to 82·3)	80·7 (80·7 to 80·7)	0·9	82·6 (81·8 to 83·4)	81·7 (81·7 to 81·7)	0·9	0·86
	Denmark		71·6 (71·4 to 71·8)	75·1 (75·0 to 75·1)	−3·4	77·8 (77·7 to 78·0)	80·6 (80·6 to 80·6)	−2·7	79·1 (79·0 to 79·3)	81·6 (81·5 to 81·6)	−2·4	81·3 (81·2 to 81·5)	82·4 (82·3 to 82·4)	−1·0	83·6 (83·4 to 83·7)	83·3 (83·2 to 83·3)	0·3	0·92
	Finland		68·3 (68·1 to 68·6)	72·6 (72·5 to 72·6)	−4·3	79·1 (79·0 to 79·3)	79·3 (79·2 to 79·3)	−0·1	81·3 (81·1 to 81·4)	80·4 (80·4 to 80·5)	0·8	83·3 (83·2 to 83·5)	81·4 (81·4 to 81·4)	1·9	84·3 (84·1 to 84·4)	82·6 (82·6 to 82·7)	1·6	0·89
	France		69·8 (69·6 to 69·9)	71·6 (71·5 to 71·6)	−1·8	81·4 (81·4 to 81·5)	78·6 (78·5 to 78·6)	2·9	82·9 (82·9 to 83·0)	79·7 (79·7 to 79·8)	3·2	84·7 (84·6 to 84·7)	80·7 (80·7 to 80·7)	4·0	85·5 (85·4 to 85·5)	81·8 (81·8 to 81·9)	3·7	0·86
	Germany		70·0 (69·7 to 70·3)	75·1 (75·0 to 75·1)	−5·1	78·6 (78·6 to 78·7)	80·0 (80·0 to 80·1)	−1·4	81·1 (81·1 to 81·2)	81·0 (81·0 to 81·0)	0·1	82·8 (82·8 to 82·9)	81·7 (81·7 to 81·7)	1·1	83·4 (83·1 to 83·6)	82·4 (82·3 to 82·4)	1·0	0·88
	Greece		69·9 (69·5 to 70·3)	70·2 (70·2 to 70·3)	−0·3	79·4 (79·3 to 79·5)	76·9 (76·9 to 77·0)	2·5	80·8 (80·7 to 80·9)	78·7 (78·6 to 78·8)	2·1	82·8 (82·7 to 82·9)	80·0 (80·0 to 80·1)	2·8	83·8 (83·6 to 83·9)	81·0 (81·0 to 81·0)	2·8	0·83
	Iceland		73·7 (73·3 to 74·2)	72·8 (72·7 to 72·8)	1·0	80·4 (80·1 to 80·7)	79·1 (79·1 to 79·2)	1·3	81·7 (81·4 to 82·0)	80·3 (80·3 to 80·3)	1·4	83·5 (83·2 to 83·8)	81·4 (81·4 to 81·4)	2·1	84·3 (83·8 to 84·6)	82·6 (82·6 to 82·7)	1·6	0·89
	Ireland		67·1 (66·8 to 67·4)	74·1 (74·1 to 74·2)	−7·0	77·7 (77·6 to 77·9)	78·6 (78·5 to 78·6)	−0·8	79·4 (79·2 to 79·6)	80·3 (80·3 to 80·3)	−0·9	82·7 (82·6 to 82·9)	81·7 (81·7 to 81·7)	1·0	84·3 (84·1 to 84·5)	83·0 (82·9 to 83·1)	1·2	0·91
	Israel		69·0 (68·7 to 69·4)	71·6 (71·5 to 71·6)	−2·6	79·1 (78·9 to 79·2)	78·1 (78·1 to 78·2)	0·9	81·1 (81·0 to 81·3)	79·3 (79·2 to 79·3)	1·8	83·7 (83·6 to 83·9)	80·0 (80·0 to 80·1)	3·7	85·3 (85·2 to 85·5)	81·3 (81·3 to 81·3)	4·1	0·84
	Italy		67·4 (67·3 to 67·6)	70·9 (70·9 to 71·0)	−3·5	80·3 (80·2 to 80·3)	78·0 (78·0 to 78·1)	2·3	82·5 (82·4 to 82·5)	79·3 (79·2 to 79·3)	3·2	84·3 (84·2 to 84·3)	80·1 (80·1 to 80·2)	4·1	85·1 (85·1 to 85·2)	81·1 (81·1 to 81·2)	4·0	0·83
	Luxembourg		68·8 (68·4 to 69·1)	74·5 (74·4 to 74·5)	−5·7	78·6 (78·3 to 78·8)	79·4 (79·4 to 79·5)	−0·8	81·1 (80·8 to 81·3)	80·7 (80·7 to 80·7)	0·4	83·3 (83·0 to 83·5)	81·7 (81·7 to 81·7)	1·6	85·1 (84·7 to 85·5)	82·8 (82·7 to 82·8)	2·4	0·89
	Malta		65·8 (65·3 to 66·3)	65·6 (65·5 to 65·7)	0·2	78·4 (78·1 to 78·7)	76·4 (76·4 to 76·5)	2·0	80·5 (80·2 to 80·8)	78·0 (78·0 to 78·1)	2·5	82·9 (82·7 to 83·2)	79·3 (79·2 to 79·3)	3·7	84·7 (84·3 to 85·1)	81·0 (81·0 to 81·0)	3·7	0·83
	Monaco		71·8 (70·8 to 72·9)	75·9 (75·8 to 75·9)	−4·1	79·7 (79·0 to 80·5)	81·4 (81·4 to 81·4)	−1·7	80·7 (80·0 to 81·4)	82·2 (82·2 to 82·3)	−1·5	81·9 (81·2 to 82·6)	82·9 (82·8 to 82·9)	−1·0	83·1 (82·1 to 84·1)	83·5 (83·4 to 83·6)	−0·4	0·92
	Netherlands		72·5 (72·4 to 72·7)	75·6 (75·6 to 75·7)	−3·1	80·0 (79·9 to 80·1)	80·3 (80·3 to 80·3)	−0·3	80·6 (80·5 to 80·7)	81·4 (81·4 to 81·4)	−0·9	82·6 (82·5 to 82·7)	82·2 (82·2 to 82·3)	0·4	83·6 (83·5 to 83·7)	83·1 (83·1 to 83·2)	0·4	0·91
	Norway		73·6 (73·3 to 73·8)	76·8 (76·8 to 76·8)	−3·2	79·8 (79·7 to 80·0)	80·3 (80·3 to 80·3)	−0·5	81·3 (81·2 to 81·5)	81·6 (81·5 to 81·6)	−0·2	83·2 (83·1 to 83·3)	82·4 (82·3 to 82·4)	0·8	84·6 (84·5 to 84·8)	83·3 (83·2 to 83·3)	1·4	0·92
	Portugal		61·8 (61·4 to 62·0)	66·2 (66·1 to 66·3)	−4·4	77·7 (77·5 to 77·8)	75·1 (75·0 to 75·1)	2·6	80·3 (80·1 to 80·4)	76·9 (76·9 to 77·0)	3·3	83·1 (83·0 to 83·2)	78·3 (78·2 to 78·3)	4·8	84·8 (84·7 to 85·0)	79·7 (79·7 to 79·8)	5·1	0·79
	San Marino		72·6 (71·5 to 73·6)	74·0 (73·9 to 74·0)	−1·4	82·2 (81·5 to 83·0)	79·7 (79·7 to 79·8)	2·5	84·1 (83·4 to 84·8)	81·1 (81·1 to 81·2)	3·0	86·8 (86·1 to 87·4)	81·8 (81·8 to 81·9)	5·0	87·2 (86·3 to 88·1)	82·5 (82·4 to 82·5)	4·7	0·88
	Spain		64·4 (64·3 to 64·6)	68·2 (68·1 to 68·3)	−3·8	80·4 (80·3 to 80·5)	76·5 (76·5 to 76·6)	3·9	82·8 (82·8 to 82·9)	78·1 (78·1 to 78·2)	4·7	85·0 (84·9 to 85·0)	79·3 (79·2 to 79·3)	5·7	85·9 (85·8 to 85·9)	80·4 (80·4 to 80·5)	5·4	0·81
	Sweden		72·5 (72·3 to 72·6)	75·8 (75·7 to 75·8)	−3·3	80·6 (80·5 to 80·7)	80·0 (80·0 to 80·1)	0·6	82·0 (81·9 to 82·1)	81·4 (81·4 to 81·4)	0·6	83·5 (83·4 to 83·6)	82·1 (82·1 to 82·1)	1·4	85·0 (84·9 to 85·1)	83·1 (83·1 to 83·2)	1·8	0·91
	Switzerland		71·1 (70·9 to 71·3)	78·8 (78·8 to 78·9)	−7·8	81·0 (80·9 to 81·2)	82·2 (82·2 to 82·3)	−1·2	82·9 (82·7 to 83·0)	82·8 (82·7 to 82·8)	0·1	84·6 (84·4 to 84·7)	83·4 (83·3 to 83·5)	1·2	86·2 (86·1 to 86·3)	84·0 (83·9 to 84·1)	2·2	0·95
	UK		70·9 (70·9 to 71·0)	75·1 (75·0 to 75·1)	−4·1	78·5 (78·4 to 78·5)	79·1 (79·1 to 79·2)	−0·6	80·3 (80·2 to 80·3)	80·3 (80·3 to 80·3)	−0·0	82·3 (82·3 to 82·4)	81·1 (81·1 to 81·2)	1·2	82·9 (82·9 to 83·0)	82·4 (82·3 to 82·4)	0·5	0·88
		England	71·5 (71·3 to 71·8)	75·5 (75·4 to 75·5)	−3·9	78·7 (78·6 to 78·8)	79·3 (79·2 to 79·3)	−0·6	80·5 (80·4 to 80·5)	80·4 (80·4 to 80·5)	0·0	82·6 (82·5 to 82·6)	81·1 (81·1 to 81·2)	1·4	83·2 (83·1 to 83·2)	82·5 (82·4 to 82·5)	0·7	0·88
		Northern Ireland	68·0 (67·1 to 69·0)	74·5 (74·4 to 74·5)	−6·5	77·6 (77·3 to 77·9)	78·8 (78·8 to 78·9)	−1·2	79·8 (79·6 to 80·1)	80·1 (80·1 to 80·2)	−0·3	81·8 (81·5 to 82·0)	80·9 (80·9 to 80·9)	0·9	82·5 (82·2 to 82·8)	82·0 (81·9 to 82·0)	0·5	0·86
		Scotland	67·5 (66·6 to 68·4)	73·3 (73·3 to 73·4)	−5·8	76·8 (76·6 to 77·0)	78·6 (78·5 to 78·6)	−1·8	78·6 (78·4 to 78·8)	80·0 (80·0 to 80·1)	−1·4	80·6 (80·4 to 80·8)	81·0 (81·0 to 81·0)	−0·4	81·1 (80·9 to 81·4)	82·4 (82·3 to 82·4)	−1·2	0·88
		Wales	69·9 (68·6 to 71·2)	71·6 (71·5 to 71·6)	−1·6	78·6 (78·1 to 79·1)	77·6 (77·6 to 77·6)	1·0	79·9 (79·5 to 80·4)	79·1 (79·1 to 79·2)	0·8	81·9 (81·5 to 82·4)	80·1 (80·1 to 80·2)	1·8	82·0 (81·4 to 82·6)	81·7 (81·7 to 81·7)	0·3	0·85
**Latin America and Caribbean**			**53·3 (52·8 to 53·9)**	**58·0 (57·9 to 58·2)**	**–4·7**	**73·2 (73·1 to 73·3)**	**71·4 (71·3 to 71·4)**	**1·9**	**76·0 (75·9 to 76·1)**	**73·3 (73·3 to 73·4)**	**2·7**	**76·4 (76·2 to 76·6)**	**75·1 (75·0 to 75·1)**	**1·4**	**79·0 (78·9 to 79·1)**	**76·7 (76·6 to 76·7)**	**2·3**	**0·67**
Andean Latin America			43·9 (43·2 to 44·6)	59·4 (59·3 to 59·5)	−15·5	72·6 (72·3 to 72·8)	71·2 (71·1 to 71·2)	1·4	75·4 (75·2 to 75·6)	72·8 (72·7 to 72·8)	2·6	78·4 (78·2 to 78·5)	74·8 (74·7 to 74·8)	3·6	78·4 (78·1 to 78·6)	76·8 (76·8 to 76·8)	1·6	0·68
	Bolivia		36·8 (35·7 to 38·1)	56·4 (56·3 to 56·6)	−19·6	65·6 (65·3 to 66·0)	67·4 (67·3 to 67·4)	−1·7	69·4 (69·0 to 69·7)	70·7 (70·7 to 70·8)	−1·3	71·9 (71·6 to 72·2)	73·1 (73·1 to 73·2)	−1·2	74·5 (73·9 to 75·2)	75·5 (75·4 to 75·5)	−0·9	0·63
	Ecuador		51·6 (50·7 to 52·5)	61·4 (61·3 to 61·4)	−9·8	73·3 (73·0 to 73·5)	72·4 (72·4 to 72·4)	0·9	74·6 (74·4 to 74·8)	73·3 (73·3 to 73·4)	1·3	78·5 (78·3 to 78·7)	74·9 (74·9 to 75·0)	3·6	79·9 (79·6 to 80·1)	77·1 (77·0 to 77·1)	2·8	0·69
	Peru		44·2 (43·1 to 45·2)	59·7 (59·6 to 59·8)	−15·5	74·8 (74·4 to 75·1)	71·6 (71·5 to 71·6)	3·2	78·1 (77·8 to 78·4)	73·1 (73·1 to 73·2)	5·0	80·7 (80·4 to 81·0)	75·1 (75·0 to 75·1)	5·7	79·0 (78·6 to 79·3)	76·9 (76·9 to 77·0)	2·0	0·69
Caribbean			56·8 (56·2 to 57·5)	61·7 (61·7 to 61·7)	−4·9	69·8 (69·5 to 70·1)	72·4 (72·4 to 72·4)	−2·6	72·3 (72·0 to 72·6)	73·7 (73·6 to 73·7)	−1·4	57·0 (55·5 to 58·4)	75·2 (75·1 to 75·3)	−18·2	73·8 (73·3 to 74·4)	76·4 (76·4 to 76·5)	−2·6	0·66
	Antigua and Barbuda		60·9 (60·2 to 61·5)	61·0 (61·0 to 61·1)	−0·2	76·1 (75·8 to 76·5)	75·1 (75·0 to 75·1)	1·1	77·7 (77·4 to 78·1)	76·4 (76·4 to 76·5)	1·3	80·1 (79·8 to 80·4)	77·7 (77·7 to 77·8)	2·4	79·7 (79·2 to 80·3)	78·8 (78·8 to 78·9)	0·9	0·76
	The Bahamas		63·2 (62·5 to 64·0)	70·2 (70·2 to 70·3)	−7·0	74·7 (74·4 to 75·1)	77·3 (77·3 to 77·4)	−2·6	74·7 (74·4 to 75·0)	78·6 (78·5 to 78·6)	−3·9	75·5 (75·2 to 75·8)	79·4 (79·4 to 79·5)	−3·9	75·8 (75·2 to 76·4)	80·6 (80·6 to 80·6)	−4·8	0·81
	Barbados		59·6 (58·9 to 60·2)	65·3 (65·2 to 65·4)	−5·7	76·4 (76·1 to 76·8)	76·3 (76·2 to 76·3)	0·1	77·6 (77·2 to 77·9)	76·9 (76·9 to 77·0)	0·6	79·5 (79·2 to 79·8)	77·9 (77·8 to 77·9)	1·6	79·0 (78·4 to 79·5)	78·8 (78·8 to 78·9)	0·1	0·75
	Belize		60·3 (59·5 to 61·1)	59·0 (59·0 to 59·2)	1·3	75·5 (75·1 to 75·9)	67·6 (67·6 to 67·7)	7·8	73·6 (73·2 to 74·0)	71·4 (71·3 to 71·4)	2·2	76·5 (76·0 to 76·9)	73·7 (73·6 to 73·7)	2·8	78·0 (77·3 to 78·7)	75·5 (75·4 to 75·5)	2·5	0·63
	Bermuda		67·5 (67·0 to 68·0)	67·1 (67·0 to 67·2)	0·4	78·1 (77·8 to 78·4)	77·3 (77·3 to 77·4)	0·8	80·0 (79·7 to 80·3)	78·4 (78·4 to 78·5)	1·5	83·3 (83·1 to 83·6)	79·7 (79·7 to 79·8)	3·6	83·4 (82·9 to 83·8)	80·9 (80·9 to 80·9)	2·5	0·83
	Cuba		71·3 (70·4 to 72·3)	65·6 (65·5 to 65·7)	5·7	77·3 (76·9 to 77·7)	74·1 (74·1 to 74·2)	3·2	79·3 (78·9 to 79·7)	74·3 (74·3 to 74·4)	5·0	80·5 (80·2 to 80·9)	75·8 (75·7 to 75·8)	4·8	80·6 (79·4 to 81·7)	77·2 (77·2 to 77·2)	3·4	0·69
	Dominica		58·3 (57·4 to 59·2)	63·7 (63·6 to 63·7)	−5·3	76·8 (76·5 to 77·2)	73·0 (72·9 to 73·0)	3·9	77·8 (77·4 to 78·1)	76·0 (76·0 to 76·1)	1·7	78·2 (77·9 to 78·5)	77·3 (77·3 to 77·4)	0·8	78·7 (78·2 to 79·3)	78·8 (78·8 to 78·9)	−0·1	0·75
	Dominican Republic		56·5 (55·5 to 57·3)	49·4 (49·3 to 49·5)	7·0	73·4 (73·1 to 73·8)	68·5 (68·4 to 68·5)	5·0	77·3 (76·9 to 77·6)	70·9 (70·9 to 71·0)	6·3	78·2 (77·8 to 78·5)	73·8 (73·8 to 73·9)	4·4	76·4 (75·8 to 76·9)	76·3 (76·2 to 76·3)	0·1	0·66
	Grenada		57·1 (55·8 to 58·3)	51·2 (51·2 to 51·3)	5·9	73·1 (72·4 to 73·7)	69·3 (69·2 to 69·3)	3·8	75·8 (75·2 to 76·4)	73·5 (73·4 to 73·5)	2·3	77·6 (76·9 to 78·2)	75·2 (75·1 to 75·3)	2·4	77·2 (76·0 to 78·3)	76·8 (76·8 to 76·8)	0·4	0·68
	Guyana		55·1 (54·0 to 56·2)	57·7 (57·6 to 57·9)	−2·6	68·2 (67·4 to 68·9)	68·7 (68·7 to 68·8)	−0·6	69·7 (69·0 to 70·4)	72·0 (72·0 to 72·1)	−2·3	71·1 (70·4 to 71·8)	74·1 (74·1 to 74·2)	−3·0	72·5 (71·3 to 73·8)	77·2 (77·2 to 77·2)	−4·7	0·70
	Haiti		38·2 (36·4 to 40·0)	52·9 (52·9 to 53·0)	−14·7	52·2 (51·4 to 53·1)	60·4 (60·3 to 60·4)	−8·1	56·8 (56·1 to 57·6)	64·0 (63·9 to 64·1)	−7·2	27·9 (26·1 to 29·7)	67·1 (67·0 to 67·2)	−39·1	62·2 (60·9 to 63·3)	69·3 (69·2 to 69·3)	−7·1	0·46
	Jamaica		59·8 (58·7 to 60·8)	64·0 (63·9 to 64·1)	−4·2	76·4 (75·7 to 76·9)	72·8 (72·7 to 72·8)	3·6	76·5 (75·9 to 77·1)	74·8 (74·7 to 74·8)	1·7	79·2 (78·6 to 79·8)	76·0 (76·0 to 76·1)	3·2	78·9 (77·6 to 80·1)	77·1 (77·0 to 77·1)	1·8	0·69
	Puerto Rico		62·4 (62·0 to 62·9)	66·5 (66·4 to 66·6)	−4·0	79·2 (79·0 to 79·4)	76·9 (76·9 to 77·0)	2·2	80·5 (80·3 to 80·7)	78·3 (78·2 to 78·3)	2·2	82·7 (82·6 to 83·0)	79·6 (79·5 to 79·6)	3·2	84·5 (84·2 to 84·8)	81·6 (81·5 to 81·6)	2·9	0·85
	Saint Kitts and Nevis		66·1 (65·5 to 66·6)	42·7 (42·4 to 43·0)	23·3	71·5 (71·2 to 71·9)	74·0 (73·9 to 74·0)	−2·4	74·5 (74·2 to 74·8)	75·9 (75·8 to 75·9)	−1·4	76·9 (76·6 to 77·2)	77·6 (77·6 to 77·6)	−0·7	77·5 (76·9 to 78·0)	78·8 (78·8 to 78·9)	−1·4	0·75
	Saint Lucia		55·7 (54·3 to 57·3)	57·7 (57·6 to 57·9)	−2·0	73·2 (72·5 to 73·9)	71·4 (71·3 to 71·4)	1·9	76·6 (75·9 to 77·3)	74·6 (74·6 to 74·7)	2·0	78·8 (78·1 to 79·4)	76·2 (76·1 to 76·2)	2·6	80·2 (79·0 to 81·3)	77·5 (77·4 to 77·5)	2·7	0·70
	Saint Vincent and the Grenadines		59·1 (57·7 to 60·5)	57·1 (57·0 to 57·3)	2·0	74·0 (73·3 to 74·7)	70·2 (70·2 to 70·3)	3·8	74·3 (73·7 to 75·0)	72·6 (72·5 to 72·6)	1·7	76·1 (75·5 to 76·7)	74·3 (74·3 to 74·4)	1·8	77·9 (76·8 to 79·1)	76·3 (76·2 to 76·3)	1·7	0·66
	Suriname		63·7 (63·2 to 64·2)	59·0 (59·0 to 59·2)	4·6	73·5 (73·3 to 73·8)	71·8 (71·7 to 71·8)	1·8	74·0 (73·8 to 74·3)	73·3 (73·3 to 73·4)	0·7	75·4 (75·2 to 75·7)	75·1 (75·0 to 75·1)	0·4	75·9 (75·4 to 76·3)	76·2 (76·1 to 76·2)	−0·3	0·65
	Trinidad and Tobago		60·3 (59·8 to 60·9)	65·3 (65·2 to 65·4)	−5·0	71·4 (71·1 to 71·6)	75·1 (75·0 to 75·1)	−3·7	72·3 (72·0 to 72·6)	76·4 (76·4 to 76·5)	−4·1	76·2 (75·9 to 76·5)	78·1 (78·1 to 78·2)	−1·9	76·2 (75·8 to 76·7)	79·3 (79·2 to 79·3)	−3·0	0·77
	Virgin Islands		65·4 (64·5 to 66·4)	69·8 (69·7 to 69·8)	−4·4	75·0 (74·4 to 75·6)	76·3 (76·2 to 76·3)	−1·3	77·8 (77·2 to 78·3)	77·9 (77·8 to 77·9)	−0·1	80·7 (80·1 to 81·3)	79·4 (79·4 to 79·5)	1·3	81·6 (80·5 to 82·7)	81·0 (81·0 to 81·0)	0·6	0·83
Central Latin America			52·7 (52·3 to 53·0)	58·7 (58·6 to 58·8)	−6·0	73·9 (73·8 to 74·0)	70·7 (70·7 to 70·8)	3·2	76·8 (76·7 to 76·9)	73·0 (72·9 to 73·0)	3·8	78·6 (78·6 to 78·7)	74·6 (74·6 to 74·7)	4·0	79·3 (79·2 to 79·5)	76·4 (76·4 to 76·5)	2·9	0·67
	Colombia		55·2 (54·7 to 55·6)	58·7 (58·6 to 58·8)	−3·6	75·4 (75·2 to 75·6)	70·7 (70·7 to 70·8)	4·7	77·7 (77·5 to 77·9)	73·0 (72·9 to 73·0)	4·7	81·1 (81·0 to 81·3)	74·9 (74·9 to 75·0)	6·2	83·2 (83·0 to 83·4)	77·1 (77·0 to 77·1)	6·1	0·69
	Costa Rica		60·2 (59·6 to 60·7)	61·4 (61·3 to 61·4)	−1·2	79·1 (78·8 to 79·3)	72·2 (72·2 to 72·3)	6·9	80·2 (79·9 to 80·4)	74·1 (74·1 to 74·2)	6·0	82·4 (82·2 to 82·6)	75·6 (75·6 to 75·7)	6·7	82·2 (81·9 to 82·4)	77·7 (77·7 to 77·8)	4·4	0·72
	El Salvador		49·0 (48·2 to 49·7)	54·2 (54·1 to 54·4)	−5·2	73·3 (73·0 to 73·6)	65·3 (65·2 to 65·4)	8·0	76·3 (76·0 to 76·6)	69·0 (68·9 to 69·1)	7·3	77·7 (77·4 to 78·0)	72·0 (72·0 to 72·1)	5·7	76·8 (76·1 to 77·5)	74·9 (74·9 to 75·0)	1·9	0·61
	Guatemala		43·9 (42·8 to 44·9)	53·6 (53·5 to 53·7)	−9·7	64·8 (64·3 to 65·3)	60·4 (60·3 to 60·4)	4·4	70·9 (70·4 to 71·3)	65·0 (64·9 to 65·0)	5·9	74·2 (73·8 to 74·6)	69·8 (69·7 to 69·8)	4·4	75·5 (74·4 to 76·5)	73·1 (73·1 to 73·2)	2·4	0·55
	Honduras		41·9 (41·2 to 42·7)	53·3 (53·2 to 53·4)	−11·3	73·0 (72·7 to 73·3)	61·4 (61·3 to 61·4)	11·6	74·1 (73·8 to 74·4)	64·6 (64·6 to 64·7)	9·5	75·7 (75·4 to 76·0)	68·7 (68·7 to 68·8)	6·9	77·6 (77·1 to 78·1)	71·8 (71·7 to 71·8)	5·8	0·52
	Mexico		51·9 (51·3 to 52·5)	59·0 (59·0 to 59·2)	−7·1	74·0 (73·8 to 74·1)	71·6 (71·5 to 71·6)	2·4	76·9 (76·8 to 77·0)	73·7 (73·6 to 73·7)	3·2	78·1 (78·0 to 78·2)	75·1 (75·0 to 75·1)	3·1	79·2 (79·1 to 79·4)	77·2 (77·2 to 77·2)	2·0	0·69
	Nicaragua		51·4 (49·8 to 53·1)	54·5 (54·4 to 54·7)	−3·1	73·9 (73·3 to 74·5)	62·7 (62·6 to 62·7)	11·2	77·7 (77·1 to 78·3)	67·1 (67·0 to 67·2)	10·6	77·7 (77·2 to 78·3)	69·8 (69·7 to 69·8)	8·0	79·2 (78·1 to 80·2)	72·4 (72·4 to 72·4)	6·8	0·54
	Panama		65·4 (64·9 to 65·9)	62·7 (62·6 to 62·7)	2·7	78·6 (78·3 to 78·9)	73·0 (72·9 to 73·0)	5·7	80·1 (79·9 to 80·4)	74·3 (74·3 to 74·4)	5·8	81·2 (81·0 to 81·5)	75·3 (75·3 to 75·4)	5·9	81·9 (81·4 to 82·3)	77·7 (77·7 to 77·8)	4·1	0·72
	Venezuela		60·1 (59·7 to 60·6)	61·4 (61·3 to 61·4)	−1·2	74·6 (74·5 to 74·8)	72·0 (72·0 to 72·1)	2·6	77·4 (77·2 to 77·5)	73·7 (73·6 to 73·7)	3·7	78·8 (78·6 to 78·9)	74·9 (74·9 to 75·0)	3·9	75·3 (74·7 to 75·9)	74·0 (73·9 to 74·0)	1·3	0·58
Tropical Latin America			55·7 (54·2 to 57·0)	55·8 (55·7 to 56·0)	−0·1	73·6 (73·4 to 73·7)	71·4 (71·3 to 71·4)	2·2	76·2 (76·0 to 76·4)	73·5 (73·4 to 73·5)	2·7	78·5 (78·3 to 78·6)	75·5 (75·4 to 75·5)	3·0	80·1 (80·0 to 80·2)	76·9 (76·9 to 77·0)	3·2	0·68
	Brazil		55·4 (53·9 to 56·8)	55·5 (55·4 to 55·7)	−0·0	73·5 (73·3 to 73·6)	71·6 (71·5 to 71·6)	1·9	76·2 (76·0 to 76·3)	73·7 (73·6 to 73·7)	2·5	78·5 (78·4 to 78·7)	75·5 (75·4 to 75·5)	3·0	80·2 (80·0 to 80·3)	76·9 (76·9 to 77·0)	3·3	0·68
	Paraguay		65·4 (64·8 to 66·1)	58·4 (58·3 to 58·5)	7·1	76·7 (76·4 to 77·0)	69·8 (69·7 to 69·8)	6·9	77·1 (76·8 to 77·4)	72·4 (72·4 to 72·4)	4·7	76·8 (76·5 to 77·0)	74·5 (74·4 to 74·5)	2·3	76·2 (75·6 to 76·9)	76·7 (76·6 to 76·7)	−0·4	0·67
**North Africa and Middle East**			**43·1 (42·5 to 43·8)**	**52·9 (52·9 to 53·0)**	**–9·8**	**67·2 (67·0 to 67·3)**	**68·2 (68·1 to 68·3)**	**–1·0**	**70·9 (70·8 to 71·1)**	**72·0 (72·0 to 72·1)**	**–1·1**	**74·1 (74·0 to 74·3)**	**74·6 (74·6 to 74·7)**	**–0·5**	**75·4 (75·0 to 75·7)**	**76·9 (76·9 to 77·0)**	**–1·6**	**0·68**
	Afghanistan		50·1 (48·1 to 52·0)	46·3 (46·0 to 46·4)	3·9	59·8 (58·8 to 60·7)	51·2 (51·2 to 51·3)	8·5	62·1 (61·2 to 63·0)	51·2 (51·2 to 51·3)	10·8	66·3 (65·6 to 67·1)	55·8 (55·7 to 56·0)	10·5	69·0 (67·7 to 70·3)	61·4 (61·3 to 61·4)	7·6	0·33
	Algeria		36·5 (35·2 to 37·8)	48·7 (48·6 to 48·8)	−12·1	69·9 (69·6 to 70·2)	68·5 (68·4 to 68·5)	1·4	73·6 (73·3 to 73·9)	71·8 (71·7 to 71·8)	1·8	75·7 (75·4 to 76·0)	74·5 (74·4 to 74·5)	1·3	77·6 (77·1 to 78·2)	76·3 (76·2 to 76·3)	1·3	0·66
	Bahrain		49·0 (47·1 to 50·6)	54·5 (54·4 to 54·7)	−5·6	71·1 (70·3 to 71·7)	74·1 (74·1 to 74·2)	−3·1	72·3 (71·6 to 73·0)	75·9 (75·8 to 75·9)	−3·6	75·8 (75·1 to 76·4)	77·6 (77·6 to 77·6)	−1·8	79·1 (78·0 to 80·2)	79·7 (79·7 to 79·8)	−0·6	0·78
	Egypt		39·6 (37·2 to 42·1)	55·8 (55·7 to 56·0)	−16·2	64·7 (64·4 to 64·9)	67·6 (67·6 to 67·7)	−3·0	68·7 (68·4 to 68·9)	71·8 (71·7 to 71·8)	−3·1	69·9 (69·7 to 70·1)	73·7 (73·6 to 73·7)	−3·8	71·1 (69·9 to 72·2)	76·7 (76·6 to 76·7)	−5·6	0·68
	Iran		37·6 (36·1 to 39·1)	51·2 (51·2 to 51·3)	−13·7	67·8 (67·4 to 68·2)	68·7 (68·7 to 68·8)	−0·9	74·6 (74·2 to 74·9)	73·7 (73·6 to 73·7)	0·9	78·3 (78·0 to 78·6)	76·0 (76·0 to 76·1)	2·3	80·6 (80·1 to 81·1)	77·9 (77·8 to 77·9)	2·7	0·72
	Iraq		59·1 (58·3 to 60·1)	49·8 (49·7 to 49·9)	9·3	70·3 (69·9 to 70·6)	67·4 (67·3 to 67·4)	2·9	70·9 (70·5 to 71·2)	69·8 (69·7 to 69·8)	1·1	71·8 (71·5 to 72·2)	72·2 (72·2 to 72·3)	−0·4	74·2 (73·5 to 74·8)	76·2 (76·1 to 76·2)	−2·0	0·65
	Jordan		47·5 (46·4 to 48·6)	52·9 (52·9 to 53·0)	−5·4	74·1 (73·8 to 74·5)	73·0 (72·9 to 73·0)	1·2	74·0 (73·6 to 74·3)	74·5 (74·4 to 74·5)	−0·5	76·6 (76·4 to 76·9)	76·2 (76·1 to 76·2)	0·5	80·4 (79·9 to 80·9)	77·9 (77·8 to 77·9)	2·5	0·72
	Kuwait		66·9 (66·2 to 67·5)	61·4 (61·3 to 61·4)	5·5	74·1 (73·8 to 74·4)	76·9 (76·9 to 77·0)	−2·8	76·5 (76·1 to 76·8)	78·4 (78·4 to 78·5)	−2·0	78·8 (78·4 to 79·1)	80·3 (80·3 to 80·3)	−1·5	81·7 (81·2 to 82·3)	82·2 (82·2 to 82·3)	−0·5	0·88
	Lebanon		58·0 (56·1 to 59·8)	61·4 (61·3 to 61·4)	−3·3	77·2 (76·5 to 78·0)	73·5 (73·4 to 73·5)	3·7	80·6 (79·9 to 81·3)	74·8 (74·7 to 74·8)	5·9	82·0 (81·5 to 82·6)	76·3 (76·2 to 76·3)	5·8	80·9 (79·8 to 82·2)	78·0 (78·0 to 78·1)	2·9	0·72
	Libya		35·9 (34·5 to 37·2)	49·4 (49·3 to 49·5)	−13·5	73·1 (72·7 to 73·5)	74·0 (73·9 to 74·0)	−0·9	75·0 (74·7 to 75·3)	76·8 (76·8 to 76·8)	−1·8	75·4 (75·1 to 75·7)	78·6 (78·5 to 78·6)	−3·1	70·0 (69·2 to 70·7)	79·3 (79·2 to 79·3)	−9·3	0·77
	Morocco		38·8 (36·9 to 40·8)	45·0 (44·7 to 45·2)	−6·2	65·1 (64·5 to 65·7)	63·7 (63·6 to 63·7)	1·5	68·3 (67·7 to 68·9)	67·4 (67·3 to 67·4)	0·9	70·7 (70·2 to 71·3)	71·2 (71·1 to 71·2)	−0·4	72·8 (71·8 to 73·8)	74·9 (74·9 to 75·0)	−2·1	0·61
	Oman		43·6 (41·8 to 45·4)	47·9 (47·7 to 48·0)	−4·3	72·7 (72·0 to 73·3)	68·2 (68·1 to 68·3)	4·5	76·8 (76·1 to 77·3)	75·2 (75·1 to 75·3)	1·6	77·7 (77·2 to 78·3)	77·7 (77·7 to 77·8)	−0·0	79·5 (78·6 to 80·3)	79·6 (79·5 to 79·6)	−0·1	0·78
	Palestine		54·0 (52·7 to 55·0)	49·1 (48·9 to 49·1)	4·9	73·4 (73·0 to 73·7)	66·8 (66·7 to 66·9)	6·6	74·9 (74·5 to 75·2)	69·8 (69·7 to 69·8)	5·1	76·2 (76·0 to 76·5)	72·2 (72·2 to 72·3)	4·0	72·0 (71·2 to 72·7)	75·8 (75·7 to 75·8)	−3·7	0·64
	Qatar		48·7 (46·7 to 50·6)	57·1 (57·0 to 57·3)	−8·4	72·4 (71·7 to 73·1)	75·5 (75·4 to 75·5)	−3·1	73·8 (73·1 to 74·6)	78·0 (78·0 to 78·1)	−4·2	77·8 (77·0 to 78·5)	80·0 (80·0 to 80·1)	−2·2	82·4 (81·3 to 83·5)	81·8 (81·8 to 81·9)	0·5	0·86
	Saudi Arabia		35·8 (33·5 to 38·1)	42·3 (41·9 to 42·6)	−6·5	71·6 (71·0 to 72·2)	68·2 (68·1 to 68·3)	3·4	75·6 (75·1 to 76·1)	75·9 (75·8 to 75·9)	−0·3	74·5 (74·1 to 74·9)	78·7 (78·6 to 78·8)	−4·2	75·6 (74·6 to 76·5)	81·0 (81·0 to 81·0)	−5·4	0·83
	Sudan		40·3 (38·1 to 42·4)	47·1 (46·9 to 47·2)	−6·8	57·4 (56·4 to 58·3)	59·4 (59·3 to 59·5)	−2·0	61·8 (60·9 to 62·5)	63·4 (63·3 to 63·4)	−1·6	67·2 (66·4 to 68·0)	69·3 (69·2 to 69·3)	−2·1	70·5 (69·3 to 71·7)	73·8 (73·8 to 73·9)	−3·3	0·57
	Syria		58·1 (57·1 to 59·0)	50·2 (50·1 to 50·2)	7·9	71·2 (70·9 to 71·6)	66·8 (66·7 to 66·9)	4·5	73·0 (72·8 to 73·2)	69·0 (68·9 to 69·1)	4·0	76·0 (75·8 to 76·2)	72·2 (72·2 to 72·3)	3·8	77·1 (76·6 to 77·6)	74·3 (74·3 to 74·4)	2·8	0·59
	Tunisia		44·6 (43·4 to 45·6)	49·8 (49·7 to 49·9)	−5·2	71·4 (71·1 to 71·8)	69·5 (69·4 to 69·6)	1·9	74·1 (73·8 to 74·5)	73·1 (73·1 to 73·2)	1·0	76·7 (76·4 to 77·0)	75·3 (75·3 to 75·4)	1·4	77·9 (77·5 to 78·4)	76·9 (76·9 to 77·0)	1·0	0·68
	Türkiye		51·3 (49·1 to 53·4)	56·7 (56·6 to 56·9)	−5·4	71·2 (70·8 to 71·6)	70·0 (69·9 to 70·0)	1·2	75·3 (75·0 to 75·7)	73·1 (73·1 to 73·2)	2·2	80·0 (79·7 to 80·3)	75·3 (75·3 to 75·4)	4·7	78·3 (77·2 to 79·4)	78·1 (78·1 to 78·2)	0·2	0·73
	United Arab Emirates		45·4 (43·8 to 47·0)	52·3 (52·2 to 52·3)	−6·9	71·9 (71·3 to 72·4)	75·9 (75·8 to 75·9)	−4·0	74·5 (74·0 to 75·1)	79·1 (79·1 to 79·2)	−4·6	77·3 (76·8 to 77·8)	81·1 (81·1 to 81·2)	−3·8	79·6 (78·9 to 80·4)	81·8 (81·8 to 81·9)	−2·2	0·86
	Yemen		43·0 (40·7 to 45·3)	43·2 (42·8 to 43·5)	−0·2	62·6 (61·7 to 63·4)	53·3 (53·2 to 53·4)	9·3	67·8 (67·1 to 68·6)	59·0 (59·0 to 59·2)	8·8	71·8 (71·0 to 72·5)	65·6 (65·5 to 65·7)	6·2	74·2 (72·9 to 75·3)	68·2 (68·1 to 68·3)	6·0	0·45
**South Asia**			**38·8 (37·3 to 40·3)**	**51·9 (51·9 to 52·0)**	**–13·1**	**59·0 (58·4 to 59·6)**	**61·0 (61·0 to 61·1)**	**–2·0**	**64·8 (64·4 to 65·2)**	**65·0 (64·9 to 65·0)**	**–0·1**	**69·3 (68·9 to 69·6)**	**69·0 (68·9 to 69·1)**	**0·3**	**72·4 (71·5 to 73·3)**	**74·1 (74·1 to 74·2)**	**–1·8**	**0·59**
	Bangladesh		42·0 (40·1 to 43·7)	40·4 (39·9 to 40·7)	1·6	56·6 (55·8 to 57·4)	55·2 (55·0 to 55·3)	1·4	63·8 (63·1 to 64·4)	59·7 (59·6 to 59·8)	4·1	69·3 (68·7 to 69·9)	64·3 (64·3 to 64·4)	5·0	71·2 (70·1 to 72·2)	71·6 (71·5 to 71·6)	−0·4	0·52
	Bhutan		34·1 (31·9 to 36·2)	41·8 (41·4 to 42·1)	−7·7	58·7 (57·8 to 59·6)	54·5 (54·4 to 54·7)	4·2	63·0 (62·2 to 63·7)	59·7 (59·6 to 59·8)	3·3	69·5 (68·8 to 70·2)	66·5 (66·4 to 66·6)	3·0	72·4 (71·2 to 73·6)	72·0 (72·0 to 72·1)	0·4	0·52
	India		37·8 (35·9 to 39·7)	52·6 (52·6 to 52·7)	−14·8	58·9 (58·2 to 59·6)	61·7 (61·7 to 61·7)	−2·8	64·9 (64·4 to 65·4)	65·9 (65·8 to 66·0)	−1·0	69·6 (69·1 to 70·0)	69·8 (69·7 to 69·8)	−0·2	73·0 (71·8 to 74·1)	74·8 (74·7 to 74·8)	−1·8	0·61
	Nepal		44·7 (43·0 to 46·5)	45·8 (45·6 to 46·0)	−1·1	58·6 (57·7 to 59·4)	53·3 (53·2 to 53·4)	5·3	66·7 (66·0 to 67·3)	57·7 (57·6 to 57·9)	8·9	72·8 (72·1 to 73·4)	63·4 (63·3 to 63·4)	9·4	75·1 (74·0 to 76·3)	69·5 (69·4 to 69·6)	5·6	0·47
	Pakistan		43·7 (41·9 to 45·4)	49·4 (49·3 to 49·5)	−5·7	62·6 (61·7 to 63·4)	60·0 (60·0 to 60·1)	2·6	65·1 (64·3 to 65·8)	63·7 (63·6 to 63·7)	1·4	67·3 (66·6 to 68·1)	67·4 (67·3 to 67·4)	−0·0	70·1 (68·8 to 71·3)	71·6 (71·5 to 71·6)	−1·5	0·51
**Southeast Asia, east Asia, and Oceania**			**49·1 (48·1 to 50·1)**	**53·3 (53·2 to 53·4)**	**–4·1**	**69·5 (69·0 to 70·0)**	**69·8 (69·7 to 69·8)**	**–0·3**	**72·7 (72·3 to 73·0)**	**73·1 (73·1 to 73·2)**	**–0·5**	**77·3 (77·0 to 77·6)**	**75·5 (75·4 to 75·5)**	**1·8**	**80·2 (79·6 to 81·0)**	**77·5 (77·4 to 77·5)**	**2·8**	**0·71**
	East Asia		50·0 (48·6 to 51·3)	52·3 (52·2 to 52·3)	−2·3	70·0 (69·4 to 70·6)	69·8 (69·7 to 69·8)	0·2	73·2 (72·7 to 73·6)	73·3 (73·3 to 73·4)	−0·1	78·8 (78·4 to 79·3)	75·9 (75·8 to 75·9)	2·9	82·6 (81·7 to 83·6)	78·1 (78·1 to 78·2)	4·5	0·73
	China		50·1 (48·6 to 51·4)	51·9 (51·9 to 52·0)	−1·8	69·8 (69·2 to 70·4)	69·3 (69·2 to 69·3)	0·5	73·1 (72·6 to 73·6)	73·0 (72·9 to 73·0)	0·1	78·9 (78·4 to 79·3)	75·8 (75·7 to 75·8)	3·1	82·8 (81·8 to 83·7)	78·1 (78·1 to 78·2)	4·6	0·73
	North Korea		42·3 (40·7 to 43·8)	58·0 (57·9 to 58·2)	−15·7	74·7 (74·0 to 75·3)	70·9 (70·9 to 71·0)	3·7	71·9 (69·7 to 73·4)	70·9 (70·9 to 71·0)	1·0	75·3 (74·7 to 76·0)	72·6 (72·5 to 72·6)	2·7	76·0 (74·9 to 77·1)	73·8 (73·8 to 73·9)	2·2	0·58
	Taiwan*		59·4 (58·7 to 60·0)	58·4 (58·3 to 58·5)	1·0	77·0 (76·8 to 77·1)	76·8 (76·8 to 76·8)	0·2	79·7 (79·6 to 79·9)	79·0 (78·9 to 79·1)	0·8	82·6 (82·5 to 82·7)	81·0 (81·0 to 81·0)	1·6	83·9 (83·7 to 84·0)	82·4 (82·3 to 82·4)	1·5	0·88
Oceania			49·6 (48·5 to 50·5)	53·9 (53·8 to 54·0)	−4·4	65·9 (65·3 to 66·3)	65·6 (65·5 to 65·7)	0·3	66·3 (65·8 to 66·9)	67·6 (67·6 to 67·7)	−1·3	66·9 (66·4 to 67·4)	68·7 (68·7 to 68·8)	−1·9	67·8 (66·9 to 68·6)	69·8 (69·7 to 69·8)	−2·0	0·48
	American Samoa		64·2 (63·2 to 65·3)	69·8 (69·7 to 69·8)	−5·5	73·2 (72·6 to 73·8)	75·1 (75·0 to 75·1)	−1·8	72·4 (71·7 to 73·0)	75·9 (75·8 to 75·9)	−3·5	72·6 (71·9 to 73·2)	76·7 (76·6 to 76·7)	−4·1	74·4 (73·2 to 75·5)	78·6 (78·5 to 78·6)	−4·2	0·74
	Cook Islands		53·8 (53·1 to 54·5)	64·6 (64·6 to 64·7)	−10·9	71·3 (71·0 to 71·6)	74·0 (73·9 to 74·0)	−2·7	73·9 (73·5 to 74·2)	76·4 (76·4 to 76·5)	−2·5	75·5 (75·2 to 75·9)	78·0 (78·0 to 78·1)	−2·5	76·5 (75·9 to 77·1)	79·4 (79·4 to 79·5)	−2·9	0·78
	Federated States of Micronesia		49·0 (48·3 to 49·8)	54·2 (54·1 to 54·4)	−5·2	68·0 (67·7 to 68·3)	69·3 (69·2 to 69·3)	−1·2	68·3 (68·0 to 68·6)	71·6 (71·5 to 71·6)	−3·3	70·1 (69·8 to 70·5)	73·0 (72·9 to 73·0)	−2·9	70·9 (70·3 to 71·5)	74·5 (74·4 to 74·5)	−3·5	0·60
	Fiji		60·2 (59·6 to 60·8)	59·0 (59·0 to 59·2)	1·1	70·6 (70·3 to 70·9)	72·6 (72·5 to 72·6)	−2·0	69·7 (69·4 to 70·0)	74·6 (74·6 to 74·7)	−4·9	69·2 (68·9 to 69·5)	75·6 (75·6 to 75·7)	−6·4	69·5 (68·9 to 70·0)	76·9 (76·9 to 77·0)	−7·5	0·69
	Guam		71·5 (71·1 to 72·0)	73·8 (73·8 to 73·9)	−2·3	77·2 (76·9 to 77·6)	76·8 (76·8 to 76·8)	0·4	78·6 (78·2 to 78·9)	78·0 (78·0 to 78·1)	0·6	80·4 (80·1 to 80·7)	78·6 (78·5 to 78·6)	1·8	79·5 (78·9 to 80·0)	80·4 (80·4 to 80·5)	−1·0	0·81
	Kiribati		51·6 (50·6 to 52·5)	58·4 (58·3 to 58·5)	−6·8	69·3 (68·9 to 69·7)	67·4 (67·3 to 67·4)	1·9	70·8 (70·4 to 71·2)	69·0 (68·9 to 69·1)	1·8	72·6 (72·2 to 72·9)	70·5 (70·4 to 70·5)	2·1	74·5 (73·7 to 75·2)	72·8 (72·7 to 72·8)	1·7	0·54
	Marshall Islands		48·6 (47·8 to 49·4)	54·9 (54·7 to 55·0)	−6·2	65·2 (64·9 to 65·5)	67·9 (67·8 to 68·0)	−2·7	64·7 (64·4 to 65·1)	70·0 (69·9 to 70·0)	−5·3	65·0 (64·6 to 65·3)	71·8 (71·7 to 71·8)	−6·8	65·2 (64·5 to 65·9)	74·8 (74·7 to 74·8)	−9·6	0·61
	Nauru		50·3 (49·7 to 51·0)	68·5 (68·4 to 68·5)	−18·1	61·3 (61·0 to 61·6)	73·3 (73·3 to 73·4)	−12·0	59·1 (58·7 to 59·4)	72·2 (72·2 to 72·3)	−13·1	59·4 (59·0 to 59·7)	72·4 (72·4 to 72·4)	−13·0	61·1 (60·4 to 61·7)	75·3 (75·3 to 75·4)	−14·2	0·63
	Niue		55·7 (55·0 to 56·3)	62·4 (62·3 to 62·4)	−6·7	73·2 (72·9 to 73·5)	74·6 (74·6 to 74·7)	−1·4	73·7 (73·4 to 74·0)	75·9 (75·8 to 75·9)	−2·2	74·6 (74·3 to 74·9)	77·2 (77·2 to 77·2)	−2·6	74·8 (74·2 to 75·4)	78·7 (78·6 to 78·8)	−3·9	0·75
	Northern Mariana Islands		55·4 (54·8 to 56·0)	68·2 (68·1 to 68·3)	−12·8	71·7 (71·4 to 71·9)	77·7 (77·7 to 77·8)	−6·1	71·8 (71·5 to 72·1)	78·7 (78·6 to 78·8)	−6·9	72·3 (72·0 to 72·6)	79·0 (78·9 to 79·1)	−6·7	72·8 (72·3 to 73·4)	79·9 (79·8 to 79·9)	−7·0	0·79
	Palau		50·5 (49·7 to 51·2)	67·6 (67·6 to 67·7)	−17·2	69·0 (68·7 to 69·3)	76·9 (76·9 to 77·0)	−7·9	69·4 (69·2 to 69·8)	77·9 (77·8 to 77·9)	−8·4	70·0 (69·7 to 70·3)	78·3 (78·2 to 78·3)	−8·3	70·2 (69·6 to 70·8)	78·8 (78·8 to 78·9)	−8·7	0·76
	Papua New Guinea		45·2 (43·7 to 46·6)	48·3 (48·1 to 48·4)	−3·1	63·7 (63·0 to 64·4)	60·7 (60·7 to 60·8)	3·0	64·7 (63·9 to 65·4)	63·7 (63·6 to 63·7)	1·0	65·5 (64·8 to 66·3)	65·6 (65·5 to 65·7)	−0·1	67·0 (65·8 to 68·2)	67·4 (67·3 to 67·4)	−0·4	0·43
	Samoa		62·0 (61·3 to 62·8)	59·7 (59·6 to 59·8)	2·3	74·6 (74·3 to 74·9)	70·7 (70·7 to 70·8)	3·9	74·6 (74·3 to 75·0)	72·0 (72·0 to 72·1)	2·6	75·1 (74·8 to 75·4)	73·5 (73·4 to 73·5)	1·6	74·5 (73·9 to 75·1)	74·8 (74·7 to 74·8)	−0·2	0·60
	Solomon Islands		53·6 (52·9 to 54·2)	51·2 (51·2 to 51·3)	2·3	65·5 (65·2 to 65·9)	60·4 (60·3 to 60·4)	5·2	66·9 (66·5 to 67·2)	64·0 (63·9 to 64·1)	2·9	67·8 (67·5 to 68·1)	65·9 (65·8 to 66·0)	1·9	66·2 (65·5 to 66·8)	69·0 (68·9 to 69·1)	−2·8	0·46
	Tokelau		53·4 (52·6 to 54·1)	57·7 (57·6 to 57·9)	−4·3	73·7 (73·4 to 74·0)	73·1 (73·1 to 73·2)	0·6	74·2 (73·9 to 74·5)	74·8 (74·7 to 74·8)	−0·5	75·2 (74·9 to 75·5)	76·4 (76·4 to 76·5)	−1·2	76·1 (75·5 to 76·7)	78·7 (78·6 to 78·8)	−2·6	0·75
	Tonga		59·6 (58·9 to 60·3)	58·4 (58·3 to 58·5)	1·2	73·5 (73·2 to 73·8)	71·4 (71·3 to 71·4)	2·2	73·9 (73·5 to 74·2)	73·1 (73·1 to 73·2)	0·7	73·6 (73·2 to 73·9)	74·0 (73·9 to 74·0)	−0·4	72·9 (72·3 to 73·6)	75·6 (75·6 to 75·7)	−2·7	0·64
	Tuvalu		47·4 (46·5 to 48·3)	58·7 (58·6 to 58·8)	−11·3	63·1 (62·8 to 63·5)	68·2 (68·1 to 68·3)	−5·1	62·1 (61·7 to 62·6)	71·4 (71·3 to 71·4)	−9·2	66·1 (65·8 to 66·5)	73·1 (73·1 to 73·2)	−7·0	66·8 (66·2 to 67·4)	74·9 (74·9 to 75·0)	−8·1	0·61
	Vanuatu		58·4 (57·7 to 59·0)	53·3 (53·2 to 53·4)	5·1	71·5 (71·2 to 71·9)	63·0 (63·0 to 63·1)	8·5	72·2 (71·8 to 72·5)	65·3 (65·2 to 65·4)	6·9	73·2 (72·9 to 73·6)	67·4 (67·3 to 67·4)	5·9	73·3 (72·7 to 74·0)	69·8 (69·7 to 69·8)	3·6	0·48
Southeast Asia			47·3 (46·3 to 48·2)	54·5 (54·4 to 54·7)	−7·3	69·2 (68·8 to 69·5)	69·5 (69·4 to 69·6)	−0·3	72·2 (71·9 to 72·5)	72·6 (72·5 to 72·6)	−0·4	74·5 (74·2 to 74·8)	74·3 (74·3 to 74·4)	0·2	76·2 (75·7 to 76·7)	76·3 (76·2 to 76·3)	−0·1	0·66
	Cambodia		47·2 (45·5 to 48·8)	52·3 (52·2 to 52·3)	−5·1	61·3 (60·4 to 62·1)	59·0 (59·0 to 59·2)	2·2	65·2 (64·4 to 66·0)	62·0 (62·0 to 62·1)	3·2	72·8 (72·1 to 73·5)	67·1 (67·0 to 67·2)	5·7	75·2 (74·1 to 76·3)	70·9 (70·9 to 71·0)	4·3	0·50
	Indonesia		43·1 (41·4 to 44·9)	52·9 (52·9 to 53·0)	−9·8	67·9 (67·0 to 68·8)	69·0 (68·9 to 69·1)	−1·1	72·0 (71·3 to 72·8)	72·4 (72·4 to 72·4)	−0·4	73·4 (72·6 to 74·2)	74·1 (74·1 to 74·2)	−0·7	75·2 (74·0 to 76·4)	76·4 (76·4 to 76·5)	−1·2	0·67
	Laos		35·9 (33·9 to 37·8)	48·7 (48·6 to 48·8)	−12·7	52·7 (51·6 to 53·8)	57·7 (57·6 to 57·9)	−5·0	59·0 (58·2 to 59·9)	61·4 (61·3 to 61·4)	−2·3	65·8 (65·0 to 66·5)	67·4 (67·3 to 67·4)	−1·6	67·2 (66·0 to 68·4)	71·6 (71·5 to 71·6)	−4·4	0·52
	Malaysia		51·6 (50·3 to 52·8)	54·2 (54·1 to 54·4)	−2·7	73·7 (73·3 to 74·1)	73·7 (73·6 to 73·7)	0·0	75·2 (74·9 to 75·5)	76·2 (76·1 to 76·2)	−1·0	77·2 (76·6 to 77·8)	77·6 (77·6 to 77·6)	−0·4	78·5 (78·1 to 78·9)	79·3 (79·2 to 79·3)	−0·8	0·77
	Maldives		39·5 (38·6 to 40·4)	52·9 (52·9 to 53·0)	−13·5	64·9 (64·6 to 65·2)	61·4 (61·3 to 61·4)	3·5	71·5 (71·2 to 71·8)	70·0 (69·9 to 70·0)	1·5	75·6 (75·3 to 75·8)	73·8 (73·8 to 73·9)	1·7	77·1 (76·5 to 77·6)	76·3 (76·2 to 76·3)	0·8	0·66
	Mauritius		54·2 (53·5 to 54·8)	60·4 (60·3 to 60·4)	−6·2	73·9 (73·6 to 74·3)	73·3 (73·3 to 73·4)	0·6	75·5 (75·2 to 75·8)	75·2 (75·1 to 75·3)	0·3	77·5 (77·3 to 77·9)	76·7 (76·6 to 76·7)	0·9	77·6 (77·1 to 78·0)	78·3 (78·2 to 78·3)	−0·7	0·74
	Myanmar		37·4 (35·4 to 39·2)	48·7 (48·6 to 48·8)	−11·3	59·2 (58·3 to 60·0)	60·7 (60·7 to 60·8)	−1·5	63·0 (62·2 to 63·8)	64·0 (63·9 to 64·1)	−1·0	67·5 (66·7 to 68·2)	69·3 (69·2 to 69·3)	−1·8	69·4 (68·2 to 70·5)	72·2 (72·2 to 72·3)	−2·8	0·53
	Philippines		57·0 (56·3 to 57·7)	61·0 (61·0 to 61·1)	−4·0	72·2 (71·9 to 72·6)	71·6 (71·5 to 71·6)	0·7	74·2 (74·0 to 74·4)	73·0 (72·9 to 73·0)	1·2	74·4 (74·2 to 74·6)	73·8 (73·8 to 73·9)	0·6	75·4 (75·1 to 75·6)	76·5 (76·5 to 76·6)	−1·2	0·67
	Seychelles		63·3 (62·8 to 63·9)	65·6 (65·5 to 65·7)	−2·2	75·4 (75·1 to 75·7)	74·5 (74·4 to 74·5)	1·0	76·8 (76·5 to 77·1)	76·5 (76·5 to 76·6)	0·3	77·5 (77·2 to 77·8)	77·3 (77·3 to 77·4)	0·2	78·5 (78·1 to 79·0)	78·8 (78·8 to 78·9)	−0·3	0·75
	Sri Lanka		55·2 (54·8 to 55·6)	63·4 (63·3 to 63·4)	−8·2	74·0 (73·8 to 74·3)	72·8 (72·7 to 72·8)	1·2	76·6 (76·4 to 76·8)	74·6 (74·6 to 74·7)	2·0	77·8 (77·6 to 78·0)	76·2 (76·1 to 76·2)	1·6	79·5 (79·2 to 79·8)	78·1 (78·1 to 78·2)	1·3	0·73
	Thailand		57·1 (56·6 to 57·5)	55·2 (55·0 to 55·3)	1·9	75·3 (75·2 to 75·5)	70·9 (70·9 to 71·0)	4·4	74·8 (74·6 to 75·0)	73·8 (73·8 to 73·9)	1·0	78·4 (78·3 to 78·5)	75·2 (75·1 to 75·3)	3·2	81·4 (81·2 to 81·7)	76·9 (76·9 to 77·0)	4·5	0·69
	Timor-Leste		36·7 (34·8 to 38·8)	46·7 (46·5 to 46·8)	−9·9	58·2 (57·2 to 59·0)	57·4 (57·3 to 57·6)	0·8	64·4 (63·6 to 65·2)	62·7 (62·6 to 62·7)	1·7	69·4 (68·7 to 70·1)	65·9 (65·8 to 66·0)	3·5	70·1 (68·9 to 71·3)	70·2 (70·2 to 70·3)	−0·1	0·48
	Viet Nam		56·1 (54·8 to 57·3)	53·9 (53·8 to 54·0)	2·2	73·5 (72·9 to 74·1)	66·5 (66·4 to 66·6)	7·0	77·2 (76·6 to 77·9)	70·9 (70·9 to 71·0)	6·3	78·6 (77·9 to 79·4)	73·7 (73·6 to 73·7)	5·0	80·2 (79·0 to 81·5)	75·9 (75·8 to 75·9)	4·4	0·64
**Sub-Saharan Africa**			**45·8 (45·2 to 46·3)**	**49·4 (49·3 to 49·5)**	**–3·6**	**54·6 (54·3 to 54·9)**	**58·7 (58·6 to 58·8)**	**–4·1**	**54·5 (54·2 to 54·7)**	**61·0 (61·0 to 61·1)**	**–6·6**	**61·2 (61·0 to 61·4)**	**64·6 (64·6 to 64·7)**	**–3·4**	**66·1 (65·8 to 66·4)**	**69·8 (69·7 to 69·8)**	**–3·6**	**0·47**
Central sub-Saharan Africa			45·7 (44·6 to 46·8)	49·4 (49·3 to 49·5)	−3·7	54·4 (53·7 to 55·0)	59·4 (59·3 to 59·5)	−5·0	54·6 (54·0 to 55·3)	60·7 (60·7 to 60·8)	−6·1	59·5 (59·0 to 60·0)	64·6 (64·6 to 64·7)	−5·1	63·5 (62·7 to 64·4)	70·0 (69·9 to 70·0)	−6·5	0·48
	Angola		45·5 (43·7 to 47·3)	48·3 (48·1 to 48·4)	−2·8	54·0 (53·0 to 55·0)	57·4 (57·3 to 57·6)	−3·4	58·3 (57·4 to 59·2)	60·0 (60·0 to 60·1)	−1·7	64·1 (63·4 to 64·9)	65·3 (65·2 to 65·4)	−1·1	67·9 (66·6 to 69·1)	70·9 (70·9 to 71·0)	−3·0	0·50
	Central African Republic		44·0 (42·3 to 45·7)	45·4 (45·2 to 45·6)	−1·4	50·2 (49·2 to 51·0)	52·9 (52·9 to 53·0)	−2·8	43·8 (42·8 to 44·8)	54·2 (54·1 to 54·4)	−10·4	50·9 (50·0 to 51·8)	54·9 (54·7 to 55·0)	−4·0	55·9 (54·6 to 57·1)	56·4 (56·3 to 56·6)	−0·6	0·26
	Congo (Brazzaville)		38·2 (36·4 to 39·9)	50·5 (50·5 to 50·6)	−12·3	56·7 (55·8 to 57·6)	66·5 (66·4 to 66·6)	−9·8	55·9 (55·1 to 56·9)	68·7 (68·7 to 68·8)	−12·8	63·0 (62·3 to 63·7)	70·7 (70·7 to 70·8)	−7·7	64·5 (63·3 to 65·8)	74·0 (73·9 to 74·0)	−9·5	0·58
	DR Congo		47·1 (45·3 to 48·7)	49·4 (49·3 to 49·5)	−2·3	54·6 (53·7 to 55·5)	58·4 (58·3 to 58·5)	−3·8	54·5 (53·6 to 55·4)	56·7 (56·6 to 56·9)	−2·3	58·5 (57·7 to 59·2)	58·0 (57·9 to 58·2)	0·4	62·6 (61·4 to 63·8)	65·3 (65·2 to 65·4)	−2·6	0·40
	Equatorial Guinea		37·4 (35·6 to 39·1)	45·8 (45·6 to 46·0)	−8·5	52·5 (51·5 to 53·3)	57·1 (57·0 to 57·3)	−4·6	57·7 (56·9 to 58·6)	66·2 (66·1 to 66·3)	−8·4	61·9 (61·1 to 62·7)	72·8 (72·7 to 72·8)	−10·9	63·9 (62·5 to 65·2)	75·9 (75·8 to 75·9)	−12·0	0·65
	Gabon		41·4 (39·7 to 43·1)	50·2 (50·1 to 50·2)	−8·8	64·4 (63·7 to 65·1)	67·1 (67·0 to 67·2)	−2·7	62·5 (61·7 to 63·3)	71·2 (71·1 to 71·2)	−8·7	65·6 (64·8 to 66·3)	73·1 (73·1 to 73·2)	−7·5	67·8 (66·6 to 69·0)	75·5 (75·4 to 75·5)	−7·7	0·63
Eastern sub-Saharan Africa			47·1 (46·5 to 47·6)	46·3 (46·0 to 46·4)	0·8	52·6 (52·2 to 52·9)	54·9 (54·7 to 55·0)	−2·3	53·3 (53·0 to 53·7)	56·7 (56·6 to 56·9)	−3·4	62·3 (62·0 to 62·6)	61·0 (61·0 to 61·1)	1·3	67·3 (66·9 to 67·7)	67·4 (67·3 to 67·4)	−0·1	0·43
	Burundi		43·6 (41·5 to 45·4)	44·5 (44·2 to 44·8)	−1·0	50·6 (49·6 to 51·6)	53·6 (53·5 to 53·7)	−3·0	49·3 (48·4 to 50·3)	54·2 (54·1 to 54·4)	−4·9	61·7 (60·9 to 62·5)	55·8 (55·7 to 56·0)	5·9	64·2 (63·1 to 65·4)	59·4 (59·3 to 59·5)	4·9	0·31
	Comoros		54·2 (52·8 to 55·6)	45·8 (45·6 to 46·0)	8·4	68·8 (68·1 to 69·6)	57·4 (57·3 to 57·6)	11·4	72·1 (71·4 to 72·8)	62·4 (62·3 to 62·4)	9·8	76·7 (76·0 to 77·4)	66·2 (66·1 to 66·3)	10·5	78·4 (77·2 to 79·5)	70·0 (69·9 to 70·0)	8·4	0·48
	Djibouti		52·8 (51·6 to 54·1)	50·2 (50·1 to 50·2)	2·6	64·0 (63·2 to 64·7)	61·4 (61·3 to 61·4)	2·6	65·1 (64·4 to 65·9)	63·0 (63·0 to 63·1)	2·1	66·7 (66·0 to 67·5)	65·6 (65·5 to 65·7)	1·1	69·9 (68·7 to 71·2)	70·5 (70·4 to 70·5)	−0·5	0·49
	Eritrea		38·4 (36·5 to 40·3)	41·8 (41·4 to 42·1)	−3·4	50·3 (49·3 to 51·2)	53·9 (53·8 to 54·0)	−3·6	58·2 (57·4 to 59·0)	60·4 (60·3 to 60·4)	−2·2	61·8 (61·0 to 62·6)	62·7 (62·6 to 62·7)	−0·9	64·5 (63·2 to 65·8)	66·5 (66·4 to 66·6)	−2·0	0·41
	Ethiopia		45·8 (44·3 to 47·5)	41·8 (41·4 to 42·1)	4·0	48·0 (47·0 to 49·0)	48·3 (48·1 to 48·4)	−0·3	52·0 (51·1 to 52·8)	50·2 (50·1 to 50·2)	1·8	62·4 (61·7 to 63·1)	55·8 (55·7 to 56·0)	6·6	66·7 (65·5 to 67·9)	65·3 (65·2 to 65·4)	1·4	0·39
	Kenya		49·6 (48·1 to 51·1)	45·8 (45·6 to 46·0)	3·8	63·9 (63·2 to 64·7)	61·7 (61·7 to 61·7)	2·2	58·9 (58·0 to 59·8)	64·6 (64·6 to 64·7)	−5·7	66·3 (65·6 to 67·0)	67·6 (67·6 to 67·7)	−1·3	71·7 (70·5 to 73·0)	73·0 (72·9 to 73·0)	−1·2	0·55
	Madagascar		46·0 (44·4 to 47·4)	47·9 (47·7 to 48·0)	−1·9	53·2 (52·4 to 54·1)	57·1 (57·0 to 57·3)	−3·9	58·5 (57·7 to 59·2)	57·4 (57·3 to 57·6)	1·1	62·2 (61·5 to 62·9)	59·0 (59·0 to 59·2)	3·1	63·9 (62·8 to 65·0)	64·3 (64·3 to 64·4)	−0·4	0·38
	Malawi		46·2 (44·2 to 48·1)	43·2 (42·8 to 43·5)	3·0	47·6 (46·6 to 48·6)	53·6 (53·5 to 53·7)	−6·0	45·9 (44·9 to 46·7)	55·5 (55·4 to 55·7)	−9·6	59·8 (59·0 to 60·6)	59·4 (59·3 to 59·5)	0·4	64·3 (63·0 to 65·6)	66·2 (66·1 to 66·3)	−1·9	0·41
	Mozambique		51·6 (49·9 to 53·3)	44·5 (44·2 to 44·8)	7·1	55·3 (54·3 to 56·4)	51·2 (51·2 to 51·3)	4·1	56·5 (55·6 to 57·4)	52·9 (52·9 to 53·0)	3·5	63·2 (62·4 to 64·0)	56·1 (56·0 to 56·3)	7·1	67·7 (66·6 to 68·9)	61·7 (61·7 to 61·7)	6·1	0·34
	Rwanda		39·6 (37·8 to 41·4)	47·5 (47·3 to 47·6)	−7·9	49·7 (48·8 to 50·7)	58·0 (57·9 to 58·2)	−8·3	49·5 (48·5 to 50·4)	58·4 (58·3 to 58·5)	−8·9	65·2 (64·5 to 65·9)	63·0 (63·0 to 63·1)	2·2	67·6 (66·5 to 68·7)	69·0 (68·9 to 69·1)	−1·4	0·46
	Somalia		56·2 (54·7 to 57·7)	37·9 (37·3 to 38·3)	18·4	54·5 (53·5 to 55·5)	40·4 (39·9 to 40·7)	14·1	56·3 (55·3 to 57·2)	40·8 (40·4 to 41·2)	15·4	54·7 (53·7 to 55·6)	46·7 (46·5 to 46·8)	8·0	67·0 (65·9 to 68·1)	54·5 (54·4 to 54·7)	12·5	0·23
	South Sudan		50·8 (49·2 to 52·1)	52·3 (52·2 to 52·3)	−1·5	53·3 (52·4 to 54·3)	57·7 (57·6 to 57·9)	−4·4	56·0 (55·1 to 56·8)	59·0 (59·0 to 59·2)	−3·0	58·6 (57·7 to 59·4)	62·7 (62·6 to 62·7)	−4·1	59·0 (57·8 to 60·2)	64·3 (64·3 to 64·4)	−5·3	0·38
	Tanzania		46·9 (45·2 to 48·6)	45·0 (44·7 to 45·2)	1·9	56·3 (55·4 to 57·2)	56·7 (56·6 to 56·9)	−0·4	54·9 (54·0 to 55·8)	58·7 (58·6 to 58·8)	−3·8	63·4 (62·6 to 64·2)	63·0 (63·0 to 63·1)	0·4	71·1 (70·0 to 72·2)	69·3 (69·2 to 69·3)	1·9	0·47
	Uganda		50·2 (48·7 to 51·7)	44·1 (43·8 to 44·3)	6·1	50·1 (49·1 to 51·0)	51·6 (51·6 to 51·6)	−1·5	51·2 (50·3 to 52·1)	54·5 (54·4 to 54·7)	−3·4	62·3 (61·4 to 63·0)	61·0 (61·0 to 61·1)	1·2	68·1 (66·9 to 69·3)	67·9 (67·8 to 68·0)	0·2	0·44
	Zambia		45·4 (43·8 to 46·9)	47·9 (47·7 to 48·0)	−2·5	50·1 (49·1 to 51·0)	59·7 (59·6 to 59·8)	−9·6	43·8 (42·8 to 44·8)	60·4 (60·3 to 60·4)	−16·6	57·0 (56·3 to 57·8)	64·6 (64·6 to 64·7)	−7·6	62·6 (61·3 to 63·8)	71·2 (71·1 to 71·2)	−8·6	0·51
Southern sub-Saharan Africa			55·1 (54·0 to 56·1)	59·4 (59·3 to 59·5)	−4·3	70·8 (70·2 to 71·4)	71·6 (71·5 to 71·6)	−0·8	58·8 (58·5 to 59·1)	73·5 (73·4 to 73·5)	−14·7	60·4 (60·1 to 60·7)	74·8 (74·7 to 74·8)	−14·4	69·0 (68·7 to 69·4)	76·3 (76·2 to 76·3)	−7·3	0·66
	Botswana		49·0 (48·1 to 49·8)	47·9 (47·7 to 48·0)	1·1	67·8 (67·5 to 68·2)	67·9 (67·8 to 68·0)	−0·1	51·5 (51·0 to 52·0)	72·8 (72·7 to 72·8)	−21·3	68·4 (68·1 to 68·8)	75·3 (75·3 to 75·4)	−6·9	72·7 (72·0 to 73·3)	76·8 (76·8 to 76·8)	−4·1	0·68
	Eswatini		40·0 (38·3 to 41·5)	47·9 (47·7 to 48·0)	−7·9	60·6 (59·8 to 61·4)	65·6 (65·5 to 65·7)	−4·9	53·9 (52·9 to 54·9)	69·8 (69·7 to 69·8)	−15·9	51·4 (50·3 to 52·5)	72·4 (72·4 to 72·4)	−21·0	62·4 (61·0 to 63·9)	74·8 (74·7 to 74·8)	−12·4	0·61
	Lesotho		50·0 (48·8 to 51·2)	50·5 (50·5 to 50·6)	−0·5	62·0 (61·4 to 62·7)	62·0 (62·0 to 62·1)	−0·0	50·4 (49·4 to 51·4)	65·6 (65·5 to 65·7)	−15·1	56·2 (55·3 to 57·1)	68·5 (68·4 to 68·5)	−12·2	60·4 (59·1 to 61·8)	71·4 (71·3 to 71·4)	−11·0	0·51
	Namibia		47·1 (46·3 to 47·9)	52·9 (52·9 to 53·0)	−5·8	64·1 (63·7 to 64·5)	67·6 (67·6 to 67·7)	−3·5	57·9 (57·5 to 58·3)	70·7 (70·7 to 70·8)	−12·8	65·4 (65·0 to 65·7)	72·8 (72·7 to 72·8)	−7·4	69·1 (68·5 to 69·8)	75·2 (75·1 to 75·3)	−6·1	0·62
	South Africa		56·3 (54·8 to 57·7)	61·7 (61·7 to 61·7)	−5·4	72·5 (71·6 to 73·3)	72·8 (72·7 to 72·8)	−0·3	62·3 (61·9 to 62·6)	74·6 (74·6 to 74·7)	−12·4	60·8 (60·4 to 61·2)	75·8 (75·7 to 75·8)	−14·9	69·7 (69·4 to 70·1)	77·2 (77·2 to 77·2)	−7·5	0·70
	Zimbabwe		55·4 (54·1 to 56·7)	51·2 (51·2 to 51·3)	4·2	68·6 (67·9 to 69·4)	66·5 (66·4 to 66·6)	2·1	49·3 (48·3 to 50·3)	68·7 (68·7 to 68·8)	−19·4	58·6 (57·7 to 59·4)	67·4 (67·3 to 67·4)	−8·8	68·4 (67·1 to 69·7)	70·5 (70·4 to 70·5)	−2·1	0·49
Western sub-Saharan Africa			42·9 (41·7 to 44·0)	48·3 (48·1 to 48·4)	−5·3	53·2 (52·7 to 53·7)	57·1 (57·0 to 57·3)	−3·9	54·8 (54·4 to 55·3)	59·7 (59·6 to 59·8)	−4·9	61·2 (60·8 to 61·6)	63·7 (63·6 to 63·7)	−2·5	65·5 (65·0 to 66·0)	69·0 (68·9 to 69·1)	−3·5	0·46
	Benin		47·0 (44·9 to 49·0)	45·8 (45·6 to 46·0)	1·2	57·7 (56·7 to 58·7)	53·9 (53·8 to 54·0)	3·8	61·1 (60·3 to 61·9)	56·1 (56·0 to 56·3)	5·0	65·9 (65·1 to 66·7)	59·0 (59·0 to 59·2)	6·8	69·2 (68·1 to 70·3)	65·9 (65·8 to 66·0)	3·3	0·41
	Burkina Faso		47·6 (45·5 to 49·4)	41·3 (40·9 to 41·6)	6·3	51·8 (50·7 to 52·9)	47·9 (47·7 to 48·0)	4·0	55·8 (54·9 to 56·8)	50·9 (50·9 to 50·9)	4·9	65·5 (64·7 to 66·2)	54·2 (54·1 to 54·4)	11·2	70·1 (69·1 to 71·2)	59·4 (59·3 to 59·5)	10·8	0·31
	Cabo Verde		60·7 (59·3 to 62·0)	49·4 (49·3 to 49·5)	11·3	72·3 (71·8 to 72·9)	58·4 (58·3 to 58·5)	14·0	75·5 (74·9 to 76·0)	64·0 (63·9 to 64·1)	11·5	78·5 (78·0 to 79·1)	69·5 (69·4 to 69·6)	9·0	78·5 (77·6 to 79·5)	73·3 (73·3 to 73·4)	5·2	0·56
	Cameroon		41·6 (39·5 to 43·4)	47·9 (47·7 to 48·0)	−6·3	56·8 (56·0 to 57·6)	59·4 (59·3 to 59·5)	−2·5	52·8 (52·0 to 53·7)	62·4 (62·3 to 62·4)	−9·5	56·5 (55·7 to 57·4)	65·3 (65·2 to 65·4)	−8·7	62·9 (61·7 to 64·0)	70·2 (70·2 to 70·3)	−7·3	0·49
	Chad		47·0 (45·4 to 48·6)	41·8 (41·4 to 42·1)	5·2	49·7 (48·7 to 50·7)	46·3 (46·0 to 46·4)	3·5	51·3 (50·4 to 52·3)	48·3 (48·1 to 48·4)	3·0	56·3 (55·5 to 57·1)	51·6 (51·6 to 51·6)	4·7	59·7 (58·6 to 60·8)	55·2 (55·0 to 55·3)	4·6	0·24
	Côte d'Ivoire		44·3 (42·5 to 46·0)	46·7 (46·5 to 46·8)	−2·3	56·2 (55·4 to 57·1)	58·0 (57·9 to 58·2)	−1·8	52·2 (51·4 to 53·1)	61·4 (61·3 to 61·4)	−9·1	60·9 (60·1 to 61·7)	63·4 (63·3 to 63·4)	−2·5	66·2 (65·0 to 67·3)	68·5 (68·4 to 68·5)	−2·3	0·45
	The Gambia		51·5 (49·8 to 53·1)	47·9 (47·7 to 48·0)	3·6	63·2 (62·4 to 63·9)	55·5 (55·4 to 55·7)	7·7	65·0 (64·2 to 65·7)	59·4 (59·3 to 59·5)	5·6	67·5 (66·7 to 68·1)	62·7 (62·6 to 62·7)	4·8	68·6 (67·4 to 69·7)	67·6 (67·6 to 67·7)	0·9	0·43
	Ghana		48·5 (46·8 to 49·9)	54·9 (54·7 to 55·0)	−6·4	58·4 (57·6 to 59·3)	64·0 (63·9 to 64·1)	−5·6	61·0 (60·3 to 61·7)	67·6 (67·6 to 67·7)	−6·7	65·6 (64·8 to 66·3)	70·2 (70·2 to 70·3)	−4·7	68·0 (66·9 to 69·2)	73·8 (73·8 to 73·9)	−5·8	0·57
	Guinea		39·0 (37·0 to 41·0)	41·8 (41·4 to 42·1)	−2·8	49·3 (48·2 to 50·4)	51·6 (51·6 to 51·6)	−2·3	54·7 (53·8 to 55·6)	54·2 (54·1 to 54·4)	0·5	59·7 (59·0 to 60·5)	56·7 (56·6 to 56·9)	3·0	63·4 (62·3 to 64·6)	62·7 (62·6 to 62·7)	0·8	0·36
	Guinea-Bissau		40·5 (38·6 to 42·4)	42·3 (41·9 to 42·6)	−1·8	52·8 (51·9 to 53·8)	53·3 (53·2 to 53·4)	−0·4	55·6 (54·7 to 56·5)	56·1 (56·0 to 56·3)	−0·5	58·6 (57·8 to 59·4)	58·7 (58·6 to 58·8)	−0·1	61·9 (60·7 to 63·0)	64·0 (63·9 to 64·1)	−2·1	0·37
	Liberia		46·7 (44·9 to 48·4)	49·1 (48·9 to 49·1)	−2·4	51·0 (50·0 to 52·0)	55·5 (55·4 to 55·7)	−4·5	54·0 (53·0 to 54·9)	54·9 (54·7 to 55·0)	−0·9	59·2 (58·4 to 60·0)	58·4 (58·3 to 58·5)	0·8	62·7 (61·5 to 63·9)	64·6 (64·6 to 64·7)	−1·9	0·38
	Mali		42·1 (39·8 to 44·2)	41·8 (41·4 to 42·1)	0·3	48·9 (47·8 to 50·0)	47·5 (47·3 to 47·6)	1·5	54·5 (53·5 to 55·3)	49·8 (49·7 to 49·9)	4·7	62·7 (61·9 to 63·5)	52·9 (52·9 to 53·0)	9·7	64·9 (63·8 to 66·1)	58·0 (57·9 to 58·2)	6·9	0·28
	Mauritania		53·5 (52·2 to 54·8)	52·9 (52·9 to 53·0)	0·6	61·9 (61·1 to 62·6)	61·7 (61·7 to 61·7)	0·2	65·2 (64·5 to 65·9)	65·0 (64·9 to 65·0)	0·3	67·2 (66·6 to 67·9)	67·1 (67·0 to 67·2)	0·2	68·8 (67·6 to 69·8)	71·8 (71·7 to 71·8)	−3·0	0·52
	Niger		42·8 (40·5 to 45·1)	40·4 (39·9 to 40·7)	2·5	43·1 (41·8 to 44·4)	43·6 (43·3 to 43·9)	−0·5	48·9 (47·8 to 50·0)	45·8 (45·6 to 46·0)	3·1	57·0 (56·1 to 57·8)	48·3 (48·1 to 48·4)	8·7	60·6 (59·4 to 61·6)	52·9 (52·9 to 53·0)	7·6	0·20
	Nigeria		40·8 (38·5 to 42·9)	49·1 (48·9 to 49·1)	−8·3	52·8 (51·7 to 53·9)	59·0 (59·0 to 59·2)	−6·2	54·4 (53·5 to 55·3)	61·0 (61·0 to 61·1)	−6·6	61·1 (60·3 to 61·9)	65·9 (65·8 to 66·0)	−4·8	65·9 (64·9 to 67·1)	71·4 (71·3 to 71·4)	−5·5	0·51
	São Tomé and Príncipe		49·8 (47·9 to 51·7)	51·6 (51·6 to 51·6)	−1·8	65·4 (64·7 to 66·2)	60·4 (60·3 to 60·4)	5·1	66·8 (66·1 to 67·5)	61·7 (61·7 to 61·7)	5·1	68·8 (68·2 to 69·5)	65·9 (65·8 to 66·0)	3·0	72·1 (71·0 to 73·2)	72·0 (72·0 to 72·1)	0·1	0·52
	Senegal		49·9 (48·0 to 51·7)	44·5 (44·2 to 44·8)	5·3	60·1 (59·2 to 60·9)	55·2 (55·0 to 55·3)	5·0	64·3 (63·6 to 65·2)	58·4 (58·3 to 58·5)	6·0	69·3 (68·6 to 70·0)	61·0 (61·0 to 61·1)	8·2	71·7 (70·7 to 72·9)	66·5 (66·4 to 66·6)	5·3	0·41
	Sierra Leone		44·2 (42·4 to 45·9)	47·1 (46·9 to 47·2)	−2·9	49·6 (48·6 to 50·6)	53·6 (53·5 to 53·7)	−4·0	50·8 (49·8 to 51·8)	53·9 (53·8 to 54·0)	−3·1	55·7 (54·9 to 56·6)	58·0 (57·9 to 58·2)	−2·3	60·8 (59·6 to 61·9)	64·6 (64·6 to 64·7)	−3·9	0·38
	Togo		43·0 (41·0 to 44·8)	45·8 (45·6 to 46·0)	−2·9	58·2 (57·3 to 59·1)	58·4 (58·3 to 58·5)	−0·1	57·9 (57·1 to 58·7)	61·0 (61·0 to 61·1)	−3·1	61·3 (60·5 to 62·1)	63·4 (63·3 to 63·4)	−2·1	66·2 (65·1 to 67·4)	69·0 (68·9 to 69·1)	−2·8	0·46

Data in parentheses are 95% uncertainty intervals. Life expectancies and differences between estimated and expected life expectancies are given in years. A positive difference indicates that the estimated life expectancy is better than would be expected solely on the basis of SDI, while a negative difference indicates worse than expected life expectancy. SDI=Socio-demographic Index. GBD=Global Burden of Diseases, Injuries, and Risk Factors Study.

* UN convention recognises Taiwan as a province of China.

Estimates for males had some overlap with females, but with notable differences, especially at the super-region level (figure 9; table 3). For males, the largest proportions with better than expected life expectancy were in south Asia (five of five countries and territories), the high-income super-region (33 of 36), and north Africa and the Middle East (15 of 21), while the largest proportions with worse than expected life expectancy were in central Europe, eastern Europe, and central Asia (18 of 29), southeast Asia, east Asia, and Oceania (19 of 34), and sub-Saharan Africa (24 of 46). The country with a population greater than 1 million with the largest positive difference between estimated and expected life expectancy in males in 2023 was Somalia (11·3 years), while the largest negative difference was in Equatorial Guinea (–11·4 years; table 3).

**Table 3:** Male life expectancy (estimated, expected based on SDI, and their difference) for 1950, 1990, 2000, 2010, and 2023, and SDI in 2023, globally and for GBD super-regions, regions, and countries and territories

			**1950**			**1990**			**2000**			**2010**			**2023**			**SDI**
			Estimated life expectancy	Expected life expectancy	Difference	Estimated life expectancy	Expected life expectancy	Difference	Estimated life expectancy	Expected life expectancy	Difference	Estimated life expectancy	Expected life expectancy	Difference	Estimated life expectancy	Expected life expectancy	Difference	
**Global**			**47·9 (47·4 to 48·4)**	**59·8 (59·8 to 59·9)**	**–11·9**	**62·2 (62·0 to 62·4)**	**67·0 (66·9 to 67·0)**	**–4·8**	**64·5 (64·4 to 64·7)**	**68·3 (68·2 to 68·4)**	**–3·8**	**68·4 (68·3 to 68·6)**	**69·4 (69·3 to 69·5)**	**–1·0**	**71·5 (71·2 to 71·8)**	**70·8 (70·7 to 70·8)**	**0·7**	**0·68**
**Central Europe, eastern Europe, and central Asia**			**58·8 (58·6 to 59·0)**	**66·7 (66·6 to 66·7)**	**–7·8**	**64·8 (64·7 to 64·8)**	**69·9 (69·8 to 69·9)**	**–5·1**	**63·2 (63·1 to 63·2)**	**70·9 (70·8 to 70·9)**	**–7·7**	**66·6 (66·5 to 66·6)**	**72·2 (72·2 to 72·3)**	**–5·7**	**69·9 (69·5 to 70·1)**	**73·6 (73·5 to 73·7)**	**–3·8**	**0·78**
Central Asia			52·4 (51·8 to 52·9)	63·2 (63·1 to 63·2)	−10·8	63·5 (63·3 to 63·7)	67·9 (67·8 to 67·9)	−4·4	63·8 (63·6 to 64·0)	68·6 (68·5 to 68·6)	−4·8	66·6 (66·5 to 66·8)	69·7 (69·7 to 69·8)	−3·1	70·5 (70·3 to 70·7)	70·5 (70·5 to 70·6)	0·0	0·67
	Armenia		56·4 (54·8 to 57·9)	64·1 (64·0 to 64·1)	−7·7	67·0 (66·5 to 67·5)	67·5 (67·4 to 67·5)	−0·4	69·7 (69·1 to 70·2)	68·6 (68·5 to 68·6)	1·1	70·7 (70·1 to 71·1)	70·2 (70·1 to 70·2)	0·5	76·3 (75·5 to 77·1)	71·6 (71·6 to 71·7)	4·7	0·72
	Azerbaijan		44·6 (42·8 to 46·4)	62·2 (62·1 to 62·3)	−17·6	62·1 (61·5 to 62·6)	69·1 (69·0 to 69·1)	−7·0	65·3 (64·8 to 65·9)	68·6 (68·5 to 68·6)	−3·2	68·8 (68·4 to 69·2)	70·2 (70·1 to 70·2)	−1·4	69·9 (69·2 to 70·6)	71·4 (71·3 to 71·4)	−1·5	0·71
	Georgia		55·2 (54·0 to 56·5)	67·8 (67·7 to 67·8)	−12·5	65·1 (64·6 to 65·5)	70·4 (70·4 to 70·4)	−5·3	65·7 (65·0 to 66·5)	69·7 (69·7 to 69·8)	−4·0	67·0 (66·5 to 67·4)	70·8 (70·7 to 70·8)	−3·8	70·1 (69·4 to 70·7)	72·9 (72·8 to 73·0)	−2·8	0·75
	Kazakhstan		55·9 (54·7 to 57·1)	64·1 (64·0 to 64·1)	−8·2	62·9 (62·6 to 63·3)	68·8 (68·8 to 68·9)	−5·9	60·7 (60·3 to 61·0)	70·1 (70·0 to 70·1)	−9·4	63·8 (63·5 to 64·2)	71·0 (71·0 to 71·0)	−7·2	70·8 (70·3 to 71·2)	71·9 (71·9 to 72·0)	−1·1	0·73
	Kyrgyzstan		49·5 (48·0 to 50·9)	63·2 (63·1 to 63·2)	−13·7	62·7 (62·1 to 63·2)	66·8 (66·8 to 66·9)	−4·2	62·9 (62·3 to 63·4)	67·5 (67·4 to 67·5)	−4·6	65·4 (64·9 to 65·8)	67·8 (67·7 to 67·8)	−2·4	72·5 (71·8 to 73·2)	69·5 (69·5 to 69·6)	3·0	0·63
	Mongolia		45·2 (43·3 to 47·0)	55·7 (55·7 to 55·8)	−10·5	58·1 (57·4 to 58·7)	64·9 (64·9 to 65·0)	−6·9	61·1 (60·5 to 61·7)	67·3 (67·3 to 67·4)	−6·2	62·7 (62·1 to 63·3)	68·8 (68·8 to 68·9)	−6·1	68·0 (67·1 to 68·9)	70·3 (70·2 to 70·3)	−2·3	0·66
	Tajikistan		47·9 (46·2 to 49·6)	56·6 (56·6 to 56·7)	−8·7	63·6 (62·9 to 64·2)	65·2 (65·1 to 65·2)	−1·6	65·8 (65·2 to 66·4)	64·1 (64·0 to 64·1)	1·7	69·9 (69·4 to 70·4)	65·9 (65·9 to 66·0)	4·0	70·6 (69·5 to 71·7)	67·6 (67·6 to 67·7)	3·0	0·55
	Turkmenistan		49·3 (47·7 to 50·8)	63·4 (63·3 to 63·5)	−14·1	62·5 (61·9 to 63·1)	68·3 (68·2 to 68·4)	−5·8	62·9 (62·3 to 63·5)	68·5 (68·4 to 68·5)	−5·5	66·0 (65·5 to 66·6)	69·6 (69·6 to 69·7)	−3·6	68·2 (67·0 to 69·5)	70·8 (70·7 to 70·8)	−2·5	0·68
	Uzbekistan		54·4 (53·0 to 55·8)	59·8 (59·8 to 59·9)	−5·4	65·2 (64·9 to 65·6)	66·3 (66·3 to 66·3)	−1·1	65·3 (65·0 to 65·6)	67·9 (67·8 to 67·9)	−2·6	67·5 (67·2 to 67·9)	69·3 (69·2 to 69·3)	−1·8	70·0 (69·6 to 70·3)	69·6 (69·6 to 69·7)	0·3	0·63
Central Europe			57·5 (57·3 to 57·7)	64·9 (64·9 to 65·0)	−7·5	67·1 (67·0 to 67·2)	69·7 (69·7 to 69·8)	−2·6	69·2 (69·2 to 69·3)	71·1 (71·1 to 71·2)	−1·9	72·0 (71·9 to 72·0)	72·9 (72·8 to 73·0)	−0·9	74·4 (74·3 to 74·5)	75·0 (74·9 to 75·0)	−0·5	0·81
	Albania		53·8 (52·4 to 55·2)	58·4 (58·3 to 58·5)	−4·6	69·2 (68·6 to 69·7)	67·5 (67·4 to 67·5)	1·7	72·4 (72·0 to 72·9)	68·2 (68·1 to 68·2)	4·3	75·9 (75·4 to 76·4)	69·9 (69·8 to 69·9)	6·0	77·3 (76·6 to 77·9)	71·4 (71·3 to 71·4)	5·9	0·71
	Bosnia and Herzegovina		50·7 (50·0 to 51·3)	56·0 (56·0 to 56·1)	−5·4	69·5 (69·2 to 69·8)	66·8 (66·8 to 66·9)	2·7	72·2 (71·9 to 72·6)	68·6 (68·5 to 68·6)	3·7	73·7 (73·5 to 74·0)	70·3 (70·2 to 70·3)	3·4	75·3 (74·6 to 76·0)	71·6 (71·6 to 71·7)	3·7	0·71
	Bulgaria		58·3 (57·9 to 58·7)	63·9 (63·8 to 63·9)	−5·6	68·0 (67·9 to 68·2)	69·5 (69·5 to 69·6)	−1·5	68·2 (68·1 to 68·4)	70·6 (70·6 to 70·7)	−2·4	70·6 (70·4 to 70·8)	72·1 (72·0 to 72·2)	−1·5	72·5 (72·2 to 72·7)	73·4 (73·3 to 73·6)	−1·0	0·77
	Croatia		54·3 (54·0 to 54·7)	64·5 (64·5 to 64·6)	−10·2	68·8 (68·5 to 68·9)	70·3 (70·2 to 70·3)	−1·5	70·8 (70·6 to 71·0)	71·0 (71·0 to 71·0)	−0·2	73·4 (73·2 to 73·5)	72·4 (72·3 to 72·5)	1·0	75·5 (75·2 to 75·8)	73·8 (73·7 to 73·9)	1·7	0·78
	Czechia		62·7 (62·5 to 63·0)	67·9 (67·8 to 67·9)	−5·2	67·7 (67·6 to 67·9)	70·6 (70·6 to 70·7)	−2·9	71·6 (71·5 to 71·8)	73·1 (73·0 to 73·2)	−1·4	74·5 (74·3 to 74·6)	74·6 (74·5 to 74·7)	−0·1	76·9 (76·8 to 77·1)	75·7 (75·7 to 75·8)	1·2	0·83
	Hungary		60·7 (60·3 to 61·1)	65·7 (65·7 to 65·8)	−5·0	65·2 (65·0 to 65·3)	70·0 (69·9 to 70·0)	−4·8	67·5 (67·3 to 67·6)	71·5 (71·5 to 71·6)	−4·0	70·7 (70·6 to 70·8)	73·1 (73·0 to 73·2)	−2·4	73·4 (73·2 to 73·6)	74·4 (74·3 to 74·5)	−1·0	0·79
	Montenegro		62·0 (61·6 to 62·4)	63·9 (63·8 to 63·9)	−1·8	70·2 (70·0 to 70·5)	70·4 (70·4 to 70·4)	−0·2	70·6 (70·4 to 70·9)	70·4 (70·4 to 70·4)	0·2	72·7 (72·4 to 72·9)	72·1 (72·0 to 72·2)	0·6	74·7 (74·3 to 75·1)	74·2 (74·1 to 74·3)	0·5	0·79
	North Macedonia		50·6 (50·0 to 51·1)	62·2 (62·1 to 62·3)	−11·6	69·0 (68·8 to 69·3)	69·1 (69·0 to 69·1)	−0·0	70·5 (70·3 to 70·8)	70·0 (69·9 to 70·0)	0·6	73·0 (72·8 to 73·3)	71·4 (71·3 to 71·4)	1·7	75·2 (74·6 to 75·7)	73·1 (73·0 to 73·2)	2·1	0·76
	Poland		56·8 (56·6 to 57·0)	65·7 (65·7 to 65·8)	−9·0	66·8 (66·7 to 66·9)	69·7 (69·7 to 69·8)	−2·9	69·7 (69·6 to 69·8)	71·6 (71·6 to 71·7)	−1·9	72·3 (72·2 to 72·4)	73·8 (73·7 to 73·9)	−1·5	75·0 (74·8 to 75·1)	76·3 (76·2 to 76·3)	−1·3	0·85
	Romania		59·5 (58·8 to 60·3)	62·2 (62·1 to 62·3)	−2·7	66·9 (66·8 to 67·1)	69·2 (69·1 to 69·2)	−2·2	67·6 (67·5 to 67·8)	70·2 (70·1 to 70·2)	−2·5	70·2 (70·1 to 70·4)	71·5 (71·5 to 71·6)	−1·3	72·5 (72·3 to 72·7)	73·4 (73·3 to 73·6)	−0·9	0·77
	Serbia		51·3 (50·7 to 52·0)	62·9 (62·9 to 63·0)	−11·6	67·2 (66·9 to 67·5)	69·6 (69·6 to 69·7)	−2·4	68·7 (68·5 to 69·0)	70·1 (70·0 to 70·1)	−1·3	71·7 (71·4 to 71·9)	71·9 (71·9 to 72·0)	−0·3	73·9 (73·0 to 74·7)	74·0 (73·9 to 74·1)	−0·1	0·78
	Slovakia		60·3 (60·0 to 60·7)	66·8 (66·8 to 66·9)	−6·5	66·7 (66·6 to 66·9)	70·1 (70·0 to 70·1)	−3·3	69·2 (69·0 to 69·4)	72·1 (72·0 to 72·2)	−2·9	71·7 (71·5 to 71·9)	73·8 (73·7 to 73·9)	−2·1	74·8 (74·6 to 75·0)	75·2 (75·1 to 75·2)	−0·4	0·81
	Slovenia		57·2 (56·8 to 57·6)	67·5 (67·4 to 67·5)	−10·2	69·1 (68·9 to 69·4)	71·8 (71·7 to 71·9)	−2·6	71·9 (71·7 to 72·1)	73·4 (73·3 to 73·6)	−1·5	76·0 (75·8 to 76·1)	75·2 (75·1 to 75·2)	0·8	79·0 (78·7 to 79·2)	76·3 (76·2 to 76·3)	2·7	0·84
Eastern Europe			60·9 (60·5 to 61·2)	67·8 (67·7 to 67·8)	−6·9	64·5 (64·5 to 64·6)	70·5 (70·5 to 70·6)	−6·0	60·6 (60·6 to 60·7)	71·5 (71·5 to 71·6)	−10·9	64·1 (64·1 to 64·2)	73·2 (73·1 to 73·4)	−9·1	67·8 (67·0 to 68·3)	75·2 (75·1 to 75·2)	−7·4	0·81
	Belarus		64·0 (63·4 to 64·6)	65·2 (65·1 to 65·2)	−1·2	66·0 (65·8 to 66·2)	69·6 (69·6 to 69·7)	−3·6	62·9 (62·7 to 63·1)	70·6 (70·6 to 70·7)	−7·7	64·7 (64·5 to 64·9)	72·5 (72·5 to 72·7)	−7·8	69·5 (68·9 to 70·2)	74·8 (74·7 to 74·8)	−5·3	0·80
	Estonia		62·4 (62·0 to 62·9)	67·9 (67·8 to 67·9)	−5·5	64·8 (64·6 to 65·1)	70·6 (70·6 to 70·7)	−5·8	65·3 (65·0 to 65·5)	72·4 (72·3 to 72·5)	−7·1	70·3 (70·1 to 70·6)	74·6 (74·5 to 74·7)	−4·3	74·2 (73·8 to 74·6)	76·7 (76·6 to 76·7)	−2·5	0·85
	Latvia		64·3 (63·7 to 64·8)	68·3 (68·2 to 68·4)	−4·0	64·7 (64·5 to 65·0)	70·9 (70·8 to 70·9)	−6·2	64·8 (64·6 to 65·1)	72·2 (72·2 to 72·3)	−7·4	68·1 (67·9 to 68·4)	74·6 (74·5 to 74·7)	−6·5	70·7 (70·3 to 71·1)	76·5 (76·4 to 76·5)	−5·7	0·85
	Lithuania		61·3 (60·8 to 61·9)	65·2 (65·1 to 65·2)	−3·8	65·9 (65·6 to 66·1)	70·3 (70·2 to 70·3)	−4·4	66·4 (66·1 to 66·6)	71·6 (71·6 to 71·7)	−5·3	67·2 (67·0 to 67·5)	74·0 (73·9 to 74·1)	−6·8	71·9 (71·5 to 72·2)	76·7 (76·6 to 76·7)	−4·8	0·86
	Moldova		51·7 (50·2 to 53·0)	62·9 (62·9 to 63·0)	−11·3	64·7 (64·2 to 65·2)	68·8 (68·8 to 68·9)	−4·1	65·1 (64·6 to 65·6)	69·3 (69·2 to 69·3)	−4·2	66·3 (65·9 to 66·8)	70·3 (70·2 to 70·3)	−4·0	71·6 (70·9 to 72·3)	72·2 (72·2 to 72·3)	−0·6	0·73
	Russia		60·2 (59·7 to 60·6)	67·8 (67·7 to 67·8)	−7·6	64·0 (63·9 to 64·0)	70·8 (70·7 to 70·8)	−6·8	59·6 (59·5 to 59·6)	71·8 (71·7 to 71·9)	−12·2	63·5 (63·5 to 63·6)	73·4 (73·3 to 73·6)	−9·9	67·6 (66·7 to 68·3)	75·5 (75·5 to 75·6)	−7·9	0·82
	Ukraine		63·0 (62·5 to 63·6)	68·0 (68·0 to 68·1)	−5·0	65·7 (65·6 to 65·8)	70·2 (70·1 to 70·2)	−4·5	62·6 (62·5 to 62·7)	70·6 (70·6 to 70·7)	−8·1	65·4 (65·3 to 65·5)	71·9 (71·9 to 72·0)	−6·6	66·6 (65·9 to 67·2)	73·2 (73·1 to 73·4)	−6·7	0·76
**High income**			**61·5 (61·3 to 61·6)**	**68·6 (68·5 to 68·6)**	**–7·1**	**72·7 (72·7 to 72·7)**	**73·1 (73·0 to 73·2)**	**–0·4**	**75·2 (75·1 to 75·2)**	**74·4 (74·3 to 74·5)**	**0·8**	**77·6 (77·6 to 77·7)**	**75·5 (75·5 to 75·6)**	**2·1**	**78·5 (78·5 to 78·6)**	**77·0 (76·9 to 77·1)**	**1·5**	**0·87**
Australasia			67·0 (66·9 to 67·1)	68·2 (68·1 to 68·2)	−1·2	73·6 (73·5 to 73·7)	72·2 (72·2 to 72·3)	1·3	76·7 (76·7 to 76·8)	73·6 (73·5 to 73·7)	3·1	79·6 (79·5 to 79·7)	74·8 (74·7 to 74·8)	4·8	81·6 (81·5 to 81·7)	76·7 (76·6 to 76·7)	4·9	0·86
	Australia		66·9 (66·7 to 67·0)	67·9 (67·8 to 67·9)	−1·0	73·8 (73·7 to 73·9)	72·1 (72·0 to 72·2)	1·7	76·9 (76·8 to 77·0)	73·4 (73·3 to 73·6)	3·5	79·7 (79·6 to 79·8)	74·8 (74·7 to 74·8)	4·9	81·9 (81·8 to 82·0)	76·7 (76·6 to 76·7)	5·3	0·85
	New Zealand		67·5 (67·2 to 67·8)	68·8 (68·8 to 68·9)	−1·3	72·7 (72·5 to 72·8)	72·9 (72·8 to 73·0)	−0·2	75·9 (75·8 to 76·1)	74·2 (74·1 to 74·3)	1·7	79·0 (78·8 to 79·2)	75·2 (75·1 to 75·2)	3·8	79·9 (79·7 to 80·1)	77·0 (76·9 to 77·1)	2·9	0·87
High-income Asia Pacific			49·6 (49·0 to 50·3)	66·3 (66·3 to 66·3)	−16·7	74·1 (74·0 to 74·2)	73·6 (73·5 to 73·7)	0·5	76·7 (76·6 to 76·7)	75·4 (75·3 to 75·4)	1·3	79·2 (79·1 to 79·2)	76·3 (76·2 to 76·3)	2·9	81·0 (81·0 to 81·1)	77·5 (77·4 to 77·6)	3·5	0·88
	Brunei		53·1 (52·2 to 54·0)	60·6 (60·6 to 60·7)	−7·5	70·2 (69·9 to 70·6)	70·6 (70·6 to 70·7)	−0·4	72·7 (72·4 to 73·1)	72·2 (72·2 to 72·3)	0·5	75·1 (74·8 to 75·4)	74·2 (74·1 to 74·3)	0·9	76·9 (76·2 to 77·5)	75·7 (75·7 to 75·8)	1·1	0·83
	Japan		57·4 (57·3 to 57·5)	67·5 (67·4 to 67·5)	−10·0	76·0 (76·0 to 76·1)	74·4 (74·3 to 74·5)	1·6	77·8 (77·7 to 77·8)	75·5 (75·5 to 75·6)	2·2	79·7 (79·7 to 79·8)	76·3 (76·2 to 76·3)	3·4	81·0 (81·0 to 81·1)	77·4 (77·3 to 77·4)	3·7	0·88
	Singapore		53·8 (53·4 to 54·3)	57·8 (57·8 to 57·9)	−4·0	72·9 (72·7 to 73·1)	71·2 (71·2 to 71·3)	1·7	76·8 (76·6 to 77·0)	73·8 (73·7 to 73·9)	3·0	80·4 (80·2 to 80·6)	76·1 (76·1 to 76·1)	4·3	83·1 (82·9 to 83·4)	77·2 (77·1 to 77·3)	6·0	0·87
	South Korea		29·4 (28·0 to 30·9)	56·6 (56·6 to 56·7)	−27·2	67·5 (67·2 to 67·8)	71·2 (71·2 to 71·3)	−3·7	72·5 (72·3 to 72·8)	74·2 (74·1 to 74·3)	−1·7	77·2 (77·0 to 77·5)	76·3 (76·2 to 76·3)	0·9	80·6 (80·4 to 80·8)	77·9 (77·7 to 78·0)	2·8	0·89
High-income North America			65·9 (65·8 to 65·9)	69·5 (69·5 to 69·6)	−3·6	72·2 (72·2 to 72·2)	73·6 (73·5 to 73·7)	−1·4	74·4 (74·4 to 74·4)	74·8 (74·7 to 74·8)	−0·4	76·6 (76·5 to 76·6)	75·9 (75·9 to 75·9)	0·6	76·3 (76·3 to 76·3)	77·5 (77·4 to 77·6)	−1·2	0·88
	Canada		66·6 (66·5 to 66·8)	69·7 (69·7 to 69·8)	−3·1	74·2 (74·1 to 74·2)	74·6 (74·5 to 74·7)	−0·4	76·6 (76·5 to 76·7)	75·9 (75·9 to 75·9)	0·7	79·3 (79·2 to 79·3)	76·8 (76·8 to 76·9)	2·4	79·6 (79·4 to 79·9)	77·7 (77·6 to 77·8)	1·9	0·89
	Greenland		46·7 (45·9 to 47·5)	69·2 (69·1 to 69·2)	−22·5	60·0 (59·6 to 60·4)	73·2 (73·1 to 73·4)	−13·2	62·6 (62·2 to 63·0)	73·2 (73·1 to 73·4)	−10·6	66·4 (66·0 to 66·7)	75·5 (75·5 to 75·6)	−9·2	70·0 (69·3 to 70·7)	76·8 (76·8 to 76·9)	−6·8	0·86
	USA		65·9 (65·8 to 65·9)	69·5 (69·5 to 69·6)	−3·7	72·0 (72·0 to 72·0)	73·6 (73·5 to 73·7)	−1·6	74·1 (74·1 to 74·2)	74·6 (74·5 to 74·7)	−0·4	76·3 (76·2 to 76·3)	75·9 (75·9 to 75·9)	0·3	75·9 (75·9 to 75·9)	77·4 (77·3 to 77·4)	−1·4	0·88
Southern Latin America			57·9 (57·7 to 58·0)	65·6 (65·5 to 65·6)	−7·7	69·2 (69·2 to 69·3)	69·1 (69·0 to 69·1)	0·2	71·4 (71·3 to 71·4)	70·3 (70·2 to 70·3)	1·1	73·4 (73·3 to 73·4)	71·1 (71·1 to 71·2)	2·3	75·6 (75·3 to 75·8)	73·4 (73·3 to 73·6)	2·1	0·77
	Argentina		60·8 (60·7 to 61·0)	66·1 (66·1 to 66·2)	−5·3	68·9 (68·8 to 69·0)	69·2 (69·1 to 69·2)	−0·3	70·5 (70·4 to 70·6)	70·3 (70·2 to 70·3)	0·2	72·5 (72·4 to 72·6)	71·0 (71·0 to 71·0)	1·5	74·7 (74·3 to 75·0)	73·2 (73·1 to 73·4)	1·4	0·76
	Chile		48·6 (48·3 to 48·9)	63·6 (63·6 to 63·7)	−15·0	70·1 (69·9 to 70·3)	68·8 (68·8 to 68·9)	1·3	74·0 (73·8 to 74·1)	70·3 (70·2 to 70·3)	3·7	75·9 (75·7 to 76·1)	71·5 (71·5 to 71·6)	4·4	78·2 (78·0 to 78·4)	74·2 (74·1 to 74·3)	4·0	0·79
	Uruguay		64·6 (64·2 to 65·0)	65·6 (65·5 to 65·6)	−0·9	69·5 (69·3 to 69·7)	68·8 (68·8 to 68·9)	0·7	70·9 (70·6 to 71·1)	69·7 (69·7 to 69·8)	1·1	72·7 (72·5 to 72·9)	70·8 (70·7 to 70·8)	2·0	74·1 (73·8 to 74·4)	72·5 (72·5 to 72·7)	1·5	0·75
Western Europe			64·5 (64·4 to 64·5)	68·2 (68·1 to 68·2)	−3·7	73·0 (72·9 to 73·0)	72·9 (72·8 to 73·0)	0·1	75·6 (75·6 to 75·6)	74·4 (74·3 to 74·5)	1·2	78·3 (78·3 to 78·4)	75·5 (75·5 to 75·6)	2·8	79·7 (79·6 to 79·7)	77·0 (76·9 to 77·1)	2·7	0·86
	Andorra		67·5 (66·8 to 68·2)	68·8 (68·8 to 68·9)	−1·3	76·5 (76·1 to 76·9)	74·0 (73·9 to 74·1)	2·5	78·1 (77·8 to 78·5)	75·0 (74·9 to 75·0)	3·1	80·3 (79·9 to 80·6)	76·5 (76·4 to 76·5)	3·8	81·8 (81·2 to 82·4)	77·5 (77·4 to 77·6)	4·3	0·88
	Austria		62·8 (62·6 to 63·0)	69·2 (69·1 to 69·2)	−6·4	72·3 (72·2 to 72·4)	72·7 (72·6 to 72·8)	−0·4	75·2 (75·0 to 75·3)	74·2 (74·1 to 74·3)	1·0	77·6 (77·5 to 77·7)	75·4 (75·3 to 75·4)	2·2	79·5 (79·4 to 79·7)	76·7 (76·6 to 76·7)	2·9	0·85
	Belgium		66·2 (65·8 to 66·5)	68·2 (68·1 to 68·2)	−2·0	72·6 (72·5 to 72·8)	72·9 (72·8 to 73·0)	−0·3	74·7 (74·6 to 74·8)	74·4 (74·3 to 74·5)	0·3	77·3 (77·2 to 77·4)	75·7 (75·7 to 75·8)	1·6	80·0 (79·9 to 80·1)	77·4 (77·3 to 77·4)	2·6	0·88
	Cyprus		59·6 (58·4 to 60·7)	62·7 (62·6 to 62·8)	−3·1	72·8 (71·9 to 73·6)	70·1 (70·0 to 70·1)	2·7	74·8 (74·0 to 75·6)	72·7 (72·6 to 72·8)	2·1	77·1 (76·4 to 77·8)	75·4 (75·3 to 75·4)	1·7	78·6 (77·7 to 79·5)	76·7 (76·6 to 76·7)	1·9	0·86
	Denmark		69·3 (69·0 to 69·5)	69·3 (69·2 to 69·3)	−0·0	72·2 (72·1 to 72·4)	75·2 (75·1 to 75·2)	−2·9	74·6 (74·4 to 74·7)	76·5 (76·4 to 76·5)	−1·9	77·2 (77·0 to 77·3)	77·5 (77·4 to 77·6)	−0·4	79·5 (79·4 to 79·7)	78·7 (78·5 to 78·9)	0·8	0·92
	Finland		61·1 (60·8 to 61·3)	67·3 (67·3 to 67·4)	−6·2	71·1 (71·0 to 71·3)	73·4 (73·3 to 73·6)	−2·3	74·3 (74·2 to 74·5)	75·0 (74·9 to 75·0)	−0·7	76·9 (76·7 to 77·0)	76·3 (76·2 to 76·3)	0·6	79·2 (79·0 to 79·3)	77·9 (77·7 to 78·0)	1·3	0·89
	France		64·4 (64·3 to 64·5)	66·5 (66·4 to 66·5)	−2·1	73·1 (73·1 to 73·2)	72·5 (72·5 to 72·7)	0·6	75·4 (75·4 to 75·5)	74·0 (73·9 to 74·1)	1·4	78·2 (78·1 to 78·2)	75·4 (75·3 to 75·4)	2·8	79·7 (79·6 to 79·8)	76·8 (76·8 to 76·9)	2·9	0·86
	Germany		65·3 (65·0 to 65·6)	69·3 (69·2 to 69·3)	−4·0	72·1 (72·1 to 72·2)	74·4 (74·3 to 74·5)	−2·3	75·2 (75·2 to 75·2)	75·7 (75·7 to 75·8)	−0·5	77·8 (77·7 to 77·8)	76·7 (76·6 to 76·7)	1·1	78·5 (78·2 to 78·8)	77·5 (77·4 to 77·6)	1·0	0·88
	Greece		66·6 (66·2 to 67·0)	65·4 (65·3 to 65·4)	1·3	74·7 (74·6 to 74·8)	70·9 (70·8 to 70·9)	3·8	75·9 (75·8 to 76·0)	72·7 (72·6 to 72·8)	3·2	77·8 (77·6 to 77·9)	74·4 (74·3 to 74·5)	3·4	78·3 (78·1 to 78·4)	75·7 (75·7 to 75·8)	2·5	0·83
	Iceland		69·1 (68·6 to 69·6)	67·5 (67·4 to 67·5)	1·6	75·5 (75·1 to 75·8)	73·2 (73·1 to 73·4)	2·2	77·9 (77·5 to 78·2)	74·8 (74·7 to 74·8)	3·1	79·9 (79·6 to 80·2)	76·3 (76·2 to 76·3)	3·6	80·8 (80·4 to 81·2)	77·9 (77·7 to 78·0)	2·9	0·89
	Ireland		64·8 (64·6 to 65·1)	68·6 (68·5 to 68·6)	−3·7	72·3 (72·1 to 72·5)	72·5 (72·5 to 72·7)	−0·3	74·1 (73·9 to 74·3)	74·8 (74·7 to 74·8)	−0·7	78·3 (78·2 to 78·5)	76·7 (76·6 to 76·7)	1·7	80·8 (80·6 to 81·0)	78·4 (78·2 to 78·5)	2·4	0·91
	Israel		67·0 (66·6 to 67·5)	66·5 (66·4 to 66·5)	0·6	75·6 (75·4 to 75·8)	72·1 (72·0 to 72·2)	3·5	76·9 (76·8 to 77·1)	73·4 (73·3 to 73·6)	3·5	80·1 (79·9 to 80·3)	74·4 (74·3 to 74·5)	5·7	81·2 (81·0 to 81·3)	76·1 (76·1 to 76·1)	5·1	0·84
	Italy		64·0 (63·9 to 64·1)	65·9 (65·9 to 66·0)	−1·9	73·7 (73·6 to 73·7)	71·9 (71·9 to 72·0)	1·7	76·5 (76·4 to 76·5)	73·4 (73·3 to 73·6)	3·1	79·2 (79·2 to 79·3)	74·6 (74·5 to 74·7)	4·6	80·7 (80·7 to 80·8)	75·9 (75·9 to 75·9)	4·8	0·83
	Luxembourg		63·9 (63·5 to 64·4)	68·8 (68·8 to 68·9)	−4·9	71·7 (71·4 to 72·0)	73·6 (73·5 to 73·7)	−1·9	75·1 (74·8 to 75·4)	75·4 (75·3 to 75·4)	−0·3	78·4 (78·1 to 78·7)	76·7 (76·6 to 76·7)	1·7	81·2 (80·8 to 81·7)	78·0 (77·9 to 78·2)	3·2	0·89
	Malta		63·4 (62·8 to 63·9)	61·4 (61·4 to 61·5)	1·9	74·1 (73·7 to 74·4)	70·4 (70·4 to 70·4)	3·7	76·0 (75·7 to 76·3)	71·9 (71·9 to 72·0)	4·0	78·7 (78·4 to 79·0)	73·4 (73·3 to 73·6)	5·3	81·4 (81·0 to 81·8)	75·7 (75·7 to 75·8)	5·7	0·83
	Monaco		66·1 (65·0 to 67·2)	70·0 (69·9 to 70·0)	−3·9	72·8 (72·0 to 73·5)	76·3 (76·2 to 76·3)	−3·5	73·5 (72·7 to 74·4)	77·4 (77·3 to 77·4)	−3·9	75·1 (74·3 to 75·8)	78·2 (78·1 to 78·3)	−3·2	77·9 (76·8 to 79·0)	79·0 (78·8 to 79·2)	−1·1	0·92
	Netherlands		70·2 (70·1 to 70·4)	69·7 (69·7 to 69·8)	0·5	73·8 (73·7 to 73·9)	74·8 (74·7 to 74·8)	−1·0	75·5 (75·4 to 75·6)	76·3 (76·2 to 76·3)	−0·8	78·7 (78·6 to 78·8)	77·4 (77·3 to 77·4)	1·3	80·4 (80·3 to 80·5)	78·6 (78·4 to 78·7)	1·9	0·91
	Norway		70·2 (70·0 to 70·4)	70·8 (70·7 to 70·8)	−0·5	73·5 (73·4 to 73·6)	74·8 (74·7 to 74·8)	−1·3	76·0 (75·8 to 76·1)	76·5 (76·4 to 76·5)	−0·5	78·8 (78·6 to 78·9)	77·5 (77·4 to 77·6)	1·2	81·4 (81·2 to 81·5)	78·7 (78·5 to 78·9)	2·6	0·92
	Portugal		56·7 (56·4 to 56·9)	61·9 (61·9 to 62·0)	−5·3	70·7 (70·6 to 70·8)	69·3 (69·2 to 69·3)	1·4	73·4 (73·3 to 73·5)	70·9 (70·8 to 70·9)	2·5	76·9 (76·8 to 77·1)	72·2 (72·2 to 72·3)	4·7	79·0 (78·8 to 79·1)	74·0 (73·9 to 74·1)	5·0	0·79
	San Marino		67·3 (66·1 to 68·5)	68·5 (68·4 to 68·5)	−1·2	74·9 (74·1 to 75·6)	74·0 (73·9 to 74·1)	0·9	77·6 (76·9 to 78·3)	75·9 (75·9 to 75·9)	1·7	81·6 (80·8 to 82·3)	76·8 (76·8 to 76·9)	4·8	83·2 (82·1 to 84·2)	77·7 (77·6 to 77·8)	5·4	0·88
	Spain		59·5 (59·4 to 59·7)	63·6 (63·6 to 63·7)	−4·1	73·2 (73·2 to 73·3)	70·5 (70·5 to 70·6)	2·7	75·9 (75·8 to 76·0)	72·1 (72·0 to 72·2)	3·8	78·9 (78·9 to 79·0)	73·4 (73·3 to 73·6)	5·5	80·4 (80·3 to 80·5)	75·0 (74·9 to 75·0)	5·4	0·81
	Sweden		69·8 (69·7 to 70·0)	69·9 (69·8 to 69·9)	−0·0	74·9 (74·8 to 75·0)	74·4 (74·3 to 74·5)	0·5	77·4 (77·3 to 77·5)	76·3 (76·2 to 76·3)	1·1	79·6 (79·5 to 79·7)	77·2 (77·1 to 77·3)	2·4	81·6 (81·5 to 81·7)	78·6 (78·4 to 78·7)	3·1	0·91
	Switzerland		66·7 (66·5 to 66·9)	72·9 (72·8 to 73·0)	−6·2	74·2 (74·1 to 74·4)	77·4 (77·3 to 77·4)	−3·1	77·2 (77·1 to 77·3)	78·0 (77·9 to 78·2)	−0·8	80·1 (80·0 to 80·2)	78·9 (78·7 to 79·0)	1·2	82·6 (82·4 to 82·7)	79·7 (79·4 to 79·9)	2·9	0·95
	UK		66·2 (66·1 to 66·3)	69·3 (69·2 to 69·3)	−3·1	72·9 (72·8 to 72·9)	73·2 (73·1 to 73·4)	−0·4	75·5 (75·4 to 75·5)	74·8 (74·7 to 74·8)	0·7	78·3 (78·3 to 78·4)	75·9 (75·9 to 75·9)	2·4	79·0 (79·0 to 79·1)	77·5 (77·4 to 77·6)	1·5	0·88
		England	66·6 (66·4 to 66·8)	69·6 (69·6 to 69·7)	−3·1	73·1 (73·0 to 73·2)	73·4 (73·3 to 73·6)	−0·3	75·7 (75·7 to 75·8)	75·0 (74·9 to 75·0)	0·8	78·6 (78·6 to 78·7)	75·9 (75·9 to 75·9)	2·7	79·3 (79·2 to 79·4)	77·7 (77·6 to 77·8)	1·6	0·88
		Northern Ireland	65·5 (64·4 to 66·5)	68·8 (68·8 to 68·9)	−3·4	71·7 (71·4 to 72·0)	72·9 (72·8 to 73·0)	−1·1	75·0 (74·7 to 75·2)	74·6 (74·5 to 74·7)	0·4	77·4 (77·1 to 77·6)	75·5 (75·5 to 75·6)	1·8	78·8 (78·4 to 79·1)	77·0 (76·9 to 77·1)	1·7	0·86
		Scotland	64·4 (63·4 to 65·3)	67·9 (67·8 to 67·9)	−3·6	71·2 (70·9 to 71·4)	72·5 (72·5 to 72·7)	−1·4	73·3 (73·1 to 73·5)	74·4 (74·3 to 74·5)	−1·1	76·3 (76·0 to 76·5)	75·7 (75·7 to 75·8)	0·5	77·1 (76·8 to 77·4)	77·5 (77·4 to 77·6)	−0·4	0·88
		Wales	65·3 (64·0 to 66·8)	66·5 (66·4 to 66·5)	−1·1	72·9 (72·4 to 73·4)	71·5 (71·5 to 71·6)	1·4	75·1 (74·6 to 75·5)	73·2 (73·1 to 73·4)	1·8	77·9 (77·4 to 78·4)	74·6 (74·5 to 74·7)	3·3	77·9 (77·3 to 78·6)	76·7 (76·6 to 76·7)	1·3	0·85
**Latin America and Caribbean**			**50·1 (49·4 to 50·7)**	**54·8 (54·8 to 55·0)**	**–4·8**	**67·1 (67·0 to 67·2)**	**66·3 (66·3 to 66·3)**	**0·8**	**69·8 (69·7 to 69·9)**	**67·9 (67·8 to 67·9)**	**1·9**	**71·0 (70·9 to 71·1)**	**69·3 (69·2 to 69·3)**	**1·7**	**73·0 (72·9 to 73·1)**	**70·6 (70·6 to 70·7)**	**2·4**	**0·67**
Andean Latin America			42·1 (41·5 to 42·7)	56·0 (56·0 to 56·1)	−13·9	68·3 (68·1 to 68·5)	66·1 (66·1 to 66·2)	2·2	70·9 (70·8 to 71·1)	67·5 (67·4 to 67·5)	3·5	74·1 (74·0 to 74·3)	69·1 (69·0 to 69·1)	5·1	73·8 (73·5 to 74·1)	70·8 (70·7 to 70·8)	3·0	0·68
	Bolivia		35·5 (34·5 to 36·7)	53·3 (53·2 to 53·5)	−17·8	60·5 (60·1 to 60·9)	62·9 (62·9 to 63·0)	−2·4	64·4 (64·0 to 64·7)	65·7 (65·7 to 65·8)	−1·4	67·1 (66·7 to 67·4)	67·8 (67·7 to 67·8)	−0·7	69·3 (68·7 to 70·0)	69·6 (69·6 to 69·7)	−0·3	0·63
	Ecuador		50·3 (49·4 to 51·2)	57·8 (57·8 to 57·9)	−7·5	68·7 (68·5 to 69·0)	67·2 (67·1 to 67·2)	1·6	69·1 (68·8 to 69·3)	67·9 (67·8 to 67·9)	1·2	73·2 (72·9 to 73·4)	69·2 (69·1 to 69·2)	4·0	73·6 (73·3 to 73·9)	71·0 (71·0 to 71·0)	2·6	0·69
	Peru		42·0 (41·1 to 42·9)	56·3 (56·3 to 56·4)	−14·3	71·0 (70·6 to 71·3)	66·5 (66·4 to 66·5)	4·5	74·5 (74·2 to 74·8)	67·8 (67·7 to 67·8)	6·7	77·3 (77·0 to 77·6)	69·3 (69·2 to 69·3)	8·0	75·3 (74·9 to 75·7)	70·9 (70·8 to 70·9)	4·4	0·69
Caribbean			55·9 (55·4 to 56·5)	58·1 (58·1 to 58·2)	−2·2	65·6 (65·3 to 65·9)	67·2 (67·1 to 67·2)	−1·5	68·0 (67·7 to 68·2)	68·2 (68·1 to 68·2)	−0·2	59·1 (58·0 to 60·0)	69·4 (69·3 to 69·5)	−10·3	69·0 (68·4 to 69·6)	70·4 (70·4 to 70·4)	−1·4	0·66
	Antigua and Barbuda		57·2 (55·9 to 58·3)	57·5 (57·5 to 57·6)	−0·3	70·5 (69·8 to 71·2)	69·3 (69·2 to 69·3)	1·2	72·4 (71·6 to 73·1)	70·4 (70·4 to 70·4)	2·0	74·9 (74·2 to 75·5)	71·6 (71·6 to 71·7)	3·2	75·8 (74·6 to 76·9)	72·9 (72·8 to 73·0)	2·9	0·76
	The Bahamas		58·2 (57·5 to 59·0)	65·4 (65·3 to 65·4)	−7·1	67·2 (66·8 to 67·5)	71·2 (71·2 to 71·3)	−4·1	67·7 (67·3 to 68·0)	72·5 (72·5 to 72·7)	−4·9	69·3 (68·9 to 69·6)	73·6 (73·5 to 73·7)	−4·3	69·8 (69·2 to 70·5)	75·2 (75·1 to 75·2)	−5·3	0·81
	Barbados		54·7 (54·0 to 55·4)	61·2 (61·1 to 61·2)	−6·5	71·4 (71·0 to 71·8)	70·3 (70·2 to 70·3)	1·1	72·9 (72·5 to 73·3)	70·9 (70·8 to 70·9)	2·0	74·5 (74·1 to 74·8)	71·8 (71·7 to 71·9)	2·7	74·3 (73·7 to 75·0)	72·9 (72·8 to 73·0)	1·4	0·75
	Belize		56·9 (56·0 to 57·7)	55·7 (55·7 to 55·8)	1·1	71·1 (70·6 to 71·5)	63·2 (63·1 to 63·2)	7·9	67·4 (66·9 to 67·9)	66·3 (66·3 to 66·3)	1·1	71·3 (70·8 to 71·7)	68·2 (68·1 to 68·2)	3·1	72·6 (71·8 to 73·3)	69·6 (69·6 to 69·7)	3·0	0·63
	Bermuda		61·8 (61·3 to 62·4)	62·7 (62·6 to 62·8)	−0·8	70·4 (70·1 to 70·8)	71·2 (71·2 to 71·3)	−0·8	73·1 (72·7 to 73·4)	72·4 (72·3 to 72·5)	0·7	77·5 (77·2 to 77·8)	74·0 (73·9 to 74·1)	3·5	78·0 (77·5 to 78·5)	75·5 (75·5 to 75·6)	2·4	0·83
	Cuba		68·1 (67·0 to 69·2)	61·4 (61·4 to 61·5)	6·7	73·5 (73·1 to 73·9)	68·6 (68·5 to 68·6)	4·9	75·2 (74·8 to 75·6)	68·7 (68·6 to 68·7)	6·5	76·5 (76·2 to 76·8)	69·9 (69·8 to 69·9)	6·6	75·8 (74·7 to 77·0)	71·1 (71·1 to 71·2)	4·7	0·69
	Dominica		54·7 (53·8 to 55·6)	59·8 (59·8 to 59·9)	−5·1	71·9 (71·5 to 72·2)	67·6 (67·6 to 67·7)	4·2	72·4 (72·1 to 72·8)	70·1 (70·0 to 70·1)	2·3	73·0 (72·7 to 73·4)	71·2 (71·2 to 71·3)	1·8	73·8 (73·2 to 74·4)	72·9 (72·8 to 73·0)	0·9	0·75
	Dominican Republic		54·9 (54·0 to 55·9)	46·3 (46·3 to 46·4)	8·6	68·4 (68·0 to 68·8)	63·9 (63·8 to 63·9)	4·6	71·0 (70·6 to 71·4)	65·9 (65·9 to 66·0)	5·1	72·1 (71·8 to 72·5)	68·3 (68·2 to 68·4)	3·8	69·9 (69·3 to 70·6)	70·3 (70·2 to 70·3)	−0·4	0·66
	Grenada		53·6 (52·3 to 54·9)	48·1 (48·1 to 48·2)	5·5	68·1 (67·4 to 68·8)	64·5 (64·5 to 64·6)	3·6	70·4 (69·7 to 71·1)	68·0 (68·0 to 68·1)	2·4	72·8 (72·1 to 73·5)	69·4 (69·3 to 69·5)	3·4	73·4 (72·1 to 74·7)	70·8 (70·7 to 70·8)	2·6	0·68
	Guyana		51·6 (50·4 to 52·7)	54·5 (54·5 to 54·7)	−3·0	61·9 (61·1 to 62·6)	64·1 (64·0 to 64·1)	−2·2	63·5 (62·8 to 64·3)	66·8 (66·8 to 66·9)	−3·3	64·7 (64·0 to 65·5)	68·6 (68·5 to 68·6)	−3·8	65·8 (64·3 to 67·0)	71·1 (71·1 to 71·2)	−5·3	0·70
	Haiti		40·5 (38·8 to 42·1)	49·9 (49·8 to 49·9)	−9·4	50·5 (49·6 to 51·4)	56·9 (56·9 to 57·0)	−6·4	55·5 (54·7 to 56·3)	60·1 (60·0 to 60·2)	−4·6	34·8 (33·1 to 36·4)	62·7 (62·6 to 62·8)	−27·9	59·9 (58·7 to 61·2)	64·5 (64·5 to 64·6)	−4·6	0·46
	Jamaica		56·5 (55·4 to 57·5)	60·1 (60·0 to 60·2)	−3·6	73·4 (72·8 to 74·1)	67·5 (67·4 to 67·5)	6·0	72·4 (71·7 to 73·0)	69·1 (69·0 to 69·1)	3·3	75·5 (74·9 to 76·0)	70·1 (70·0 to 70·1)	5·4	73·5 (72·2 to 74·8)	71·0 (71·0 to 71·0)	2·5	0·69
	Puerto Rico		59·6 (59·2 to 60·1)	62·2 (62·1 to 62·3)	−2·6	70·3 (70·0 to 70·5)	70·9 (70·8 to 70·9)	−0·6	72·6 (72·4 to 72·9)	72·2 (72·2 to 72·3)	0·4	75·5 (75·2 to 75·7)	73·8 (73·7 to 73·9)	1·7	77·3 (77·0 to 77·7)	76·5 (76·4 to 76·5)	0·9	0·85
	Saint Kitts and Nevis		62·0 (61·4 to 62·7)	39·8 (39·4 to 40·0)	22·3	66·1 (65·7 to 66·5)	68·5 (68·4 to 68·5)	−2·4	68·4 (68·1 to 68·8)	70·0 (69·9 to 70·0)	−1·5	70·5 (70·2 to 70·9)	71·5 (71·5 to 71·6)	−1·0	72·2 (71·6 to 72·8)	72·9 (72·8 to 73·0)	−0·7	0·75
	Saint Lucia		52·6 (51·0 to 54·1)	54·5 (54·5 to 54·7)	−2·0	68·0 (67·3 to 68·8)	66·3 (66·3 to 66·3)	1·7	71·3 (70·5 to 72·0)	69·0 (68·9 to 69·0)	2·3	72·7 (72·0 to 73·4)	70·2 (70·1 to 70·2)	2·5	74·8 (73·5 to 76·0)	71·4 (71·3 to 71·4)	3·5	0·70
	Saint Vincent and the Grenadines		57·8 (56·4 to 59·2)	53·9 (53·8 to 54·1)	3·8	69·3 (68·6 to 70·0)	65·4 (65·3 to 65·4)	3·9	69·4 (68·7 to 70·2)	67·3 (67·3 to 67·4)	2·1	71·7 (71·0 to 72·3)	68·7 (68·6 to 68·7)	3·0	72·7 (71·5 to 74·0)	70·3 (70·2 to 70·3)	2·4	0·66
	Suriname		59·6 (59·1 to 60·1)	55·7 (55·7 to 55·8)	3·8	68·1 (67·8 to 68·4)	66·7 (66·6 to 66·7)	1·5	68·1 (67·8 to 68·3)	67·9 (67·8 to 67·9)	0·2	69·5 (69·2 to 69·7)	69·3 (69·2 to 69·3)	0·2	70·4 (69·9 to 70·9)	70·2 (70·1 to 70·2)	0·2	0·65
	Trinidad and Tobago		57·1 (56·6 to 57·6)	61·2 (61·1 to 61·2)	−4·1	66·8 (66·5 to 67·1)	69·3 (69·2 to 69·3)	−2·5	67·2 (66·9 to 67·5)	70·4 (70·4 to 70·4)	−3·2	70·3 (70·0 to 70·6)	72·1 (72·0 to 72·2)	−1·7	70·3 (69·8 to 70·8)	73·4 (73·3 to 73·6)	−3·1	0·77
	Virgin Islands		60·3 (59·3 to 61·4)	64·9 (64·9 to 65·0)	−4·6	68·7 (68·0 to 69·4)	70·3 (70·2 to 70·3)	−1·6	69·9 (69·1 to 70·6)	71·8 (71·7 to 71·9)	−1·9	71·3 (70·5 to 72·0)	73·6 (73·5 to 73·7)	−2·3	71·7 (70·3 to 73·1)	75·7 (75·7 to 75·8)	−4·1	0·83
	Central Latin America		49·8 (49·4 to 50·1)	55·4 (55·4 to 55·5)	−5·7	68·0 (67·9 to 68·1)	65·7 (65·7 to 65·8)	2·2	70·9 (70·8 to 71·0)	67·6 (67·6 to 67·7)	3·3	72·7 (72·6 to 72·8)	69·0 (68·9 to 69·0)	3·7	73·5 (73·3 to 73·6)	70·4 (70·4 to 70·4)	3·0	0·67
	Colombia		52·2 (51·8 to 52·6)	55·4 (55·4 to 55·5)	−3·2	68·5 (68·3 to 68·7)	65·7 (65·7 to 65·8)	2·8	70·0 (69·8 to 70·2)	67·6 (67·6 to 67·7)	2·4	74·9 (74·7 to 75·1)	69·2 (69·1 to 69·2)	5·7	77·0 (76·8 to 77·2)	71·0 (71·0 to 71·0)	6·0	0·69
	Costa Rica		58·2 (57·6 to 58·7)	57·8 (57·8 to 57·9)	0·3	74·5 (74·2 to 74·7)	67·0 (66·9 to 67·0)	7·5	75·2 (74·9 to 75·5)	68·6 (68·5 to 68·6)	6·6	77·2 (77·0 to 77·4)	69·7 (69·7 to 69·8)	7·4	76·9 (76·6 to 77·2)	71·6 (71·6 to 71·7)	5·2	0·72
	El Salvador		46·7 (45·8 to 47·6)	51·2 (51·1 to 51·3)	−4·5	63·9 (63·5 to 64·4)	61·2 (61·1 to 61·2)	2·8	67·4 (67·0 to 67·9)	64·3 (64·3 to 64·4)	3·1	69·0 (68·5 to 69·5)	66·8 (66·8 to 66·9)	2·2	69·0 (68·0 to 70·0)	69·2 (69·1 to 69·2)	−0·2	0·61
	Guatemala		44·2 (43·2 to 45·1)	50·5 (50·4 to 50·6)	−6·3	59·7 (59·1 to 60·3)	56·9 (56·9 to 57·0)	2·8	64·6 (64·1 to 65·1)	60·9 (60·8 to 61·0)	3·7	68·4 (68·0 to 68·8)	64·9 (64·9 to 65·0)	3·4	70·8 (69·6 to 72·0)	67·8 (67·7 to 67·8)	3·0	0·55
	Honduras		40·4 (39·6 to 41·2)	50·2 (50·1 to 50·3)	−9·8	68·1 (67·7 to 68·4)	57·8 (57·8 to 57·9)	10·3	70·3 (70·0 to 70·6)	60·6 (60·6 to 60·7)	9·7	72·5 (72·2 to 72·8)	64·1 (64·0 to 64·1)	8·4	74·4 (73·9 to 75·0)	66·7 (66·6 to 66·7)	7·8	0·52
	Mexico		48·4 (47·8 to 48·9)	55·7 (55·7 to 55·8)	−7·4	68·4 (68·2 to 68·5)	66·5 (66·4 to 66·5)	1·9	72·0 (71·9 to 72·2)	68·2 (68·1 to 68·2)	3·9	72·8 (72·7 to 72·9)	69·3 (69·2 to 69·3)	3·5	73·4 (73·3 to 73·5)	71·1 (71·1 to 71·2)	2·3	0·69
	Nicaragua		46·9 (45·2 to 48·3)	51·5 (51·4 to 51·6)	−4·6	68·0 (67·4 to 68·7)	59·0 (58·9 to 59·0)	9·1	71·9 (71·3 to 72·5)	62·7 (62·6 to 62·8)	9·2	72·2 (71·7 to 72·8)	64·9 (64·9 to 65·0)	7·3	74·1 (72·9 to 75·1)	67·2 (67·1 to 67·2)	6·9	0·54
	Panama		62·6 (62·0 to 63·1)	59·0 (58·9 to 59·0)	3·6	73·8 (73·5 to 74·1)	67·6 (67·6 to 67·7)	6·2	75·0 (74·8 to 75·3)	68·7 (68·6 to 68·7)	6·3	75·2 (75·0 to 75·5)	69·5 (69·5 to 69·6)	5·7	76·6 (76·0 to 77·1)	71·6 (71·6 to 71·7)	4·9	0·72
	Venezuela		57·7 (57·2 to 58·2)	57·8 (57·8 to 57·9)	−0·1	68·8 (68·6 to 69·0)	66·8 (66·8 to 66·9)	2·0	69·9 (69·7 to 70·1)	68·2 (68·1 to 68·2)	1·7	70·6 (70·4 to 70·7)	69·2 (69·1 to 69·2)	1·4	68·2 (67·5 to 68·9)	68·5 (68·4 to 68·5)	−0·2	0·58
Tropical Latin America			50·9 (49·3 to 52·4)	52·7 (52·6 to 52·8)	−1·9	66·3 (66·1 to 66·5)	66·3 (66·3 to 66·3)	−0·0	68·9 (68·8 to 69·1)	68·0 (68·0 to 68·1)	0·9	71·3 (71·2 to 71·5)	69·6 (69·6 to 69·7)	1·7	73·4 (73·2 to 73·6)	70·9 (70·8 to 70·9)	2·5	0·68
	Brazil		50·6 (49·1 to 52·2)	52·4 (52·3 to 52·5)	−1·8	66·1 (65·9 to 66·3)	66·5 (66·4 to 66·5)	−0·4	68·8 (68·7 to 69·0)	68·2 (68·1 to 68·2)	0·7	71·3 (71·2 to 71·5)	69·6 (69·6 to 69·7)	1·7	73·5 (73·3 to 73·7)	70·9 (70·8 to 70·9)	2·6	0·68
	Paraguay		62·2 (61·5 to 62·9)	55·1 (55·1 to 55·3)	7·1	73·3 (72·9 to 73·6)	64·9 (64·9 to 65·0)	8·3	72·3 (71·9 to 72·6)	67·2 (67·1 to 67·2)	5·1	71·1 (70·8 to 71·4)	68·8 (68·8 to 68·9)	2·3	70·0 (69·3 to 70·8)	70·6 (70·6 to 70·7)	−0·6	0·67
**North Africa and Middle East**			**42·4 (41·9 to 42·9)**	**49·9 (49·8 to 49·9)**	**–7·4**	**63·8 (63·6 to 63·9)**	**63·6 (63·6 to 63·7)**	**0·1**	**67·3 (67·1 to 67·4)**	**66·8 (66·8 to 66·9)**	**0·4**	**70·5 (70·4 to 70·7)**	**69·0 (68·9 to 69·0)**	**1·6**	**72·1 (71·8 to 72·5)**	**70·9 (70·8 to 70·9)**	**1·3**	**0·68**
	Afghanistan		48·2 (46·5 to 49·9)	43·2 (43·0 to 43·3)	5·0	58·1 (57·2 to 58·9)	48·1 (48·1 to 48·2)	9·9	60·3 (59·5 to 61·1)	48·1 (48·1 to 48·2)	12·2	65·0 (64·2 to 65·6)	52·7 (52·6 to 52·8)	12·2	68·1 (67·0 to 69·3)	57·8 (57·8 to 57·9)	10·3	0·33
	Algeria		35·7 (34·6 to 36·8)	45·6 (45·5 to 45·7)	−9·8	67·0 (66·7 to 67·3)	63·9 (63·8 to 63·9)	3·2	70·8 (70·5 to 71·1)	66·7 (66·6 to 66·7)	4·2	73·4 (73·1 to 73·7)	68·8 (68·8 to 68·9)	4·6	74·5 (74·1 to 75·0)	70·3 (70·2 to 70·3)	4·2	0·66
	Bahrain		48·9 (47·4 to 50·5)	51·5 (51·4 to 51·6)	−2·5	69·0 (68·4 to 69·6)	68·6 (68·5 to 68·6)	0·4	69·5 (68·9 to 70·1)	70·0 (69·9 to 70·0)	−0·5	74·1 (73·6 to 74·6)	71·5 (71·5 to 71·6)	2·6	75·9 (74·9 to 77·0)	74·0 (73·9 to 74·1)	1·9	0·78
	Egypt		39·9 (38·0 to 41·9)	52·7 (52·6 to 52·8)	−12·8	63·0 (62·7 to 63·2)	63·2 (63·1 to 63·2)	−0·2	66·2 (66·0 to 66·4)	66·7 (66·6 to 66·7)	−0·4	67·6 (67·4 to 67·8)	68·2 (68·1 to 68·2)	−0·6	69·1 (68·0 to 70·2)	70·6 (70·6 to 70·7)	−1·5	0·68
	Iran		41·6 (40·5 to 42·6)	48·1 (48·1 to 48·2)	−6·6	65·2 (64·9 to 65·5)	64·1 (64·0 to 64·1)	1·1	71·1 (70·8 to 71·4)	68·2 (68·1 to 68·2)	2·9	74·5 (74·3 to 74·8)	70·1 (70·0 to 70·1)	4·5	77·2 (76·7 to 77·7)	71·8 (71·7 to 71·9)	5·4	0·72
	Iraq		52·1 (51·4 to 53·0)	46·7 (46·6 to 46·8)	5·4	63·6 (63·3 to 63·8)	62·9 (62·9 to 63·0)	0·6	64·6 (64·2 to 64·9)	64·9 (64·9 to 65·0)	−0·4	65·5 (65·1 to 65·8)	67·0 (66·9 to 67·0)	−1·5	68·3 (67·7 to 69·0)	70·2 (70·1 to 70·2)	−1·8	0·65
	Jordan		44·9 (44·0 to 45·8)	49·9 (49·8 to 49·9)	−4·9	68·0 (67·7 to 68·4)	67·6 (67·6 to 67·7)	0·4	69·8 (69·5 to 70·1)	68·8 (68·8 to 68·9)	0·9	73·3 (73·1 to 73·5)	70·2 (70·1 to 70·2)	3·1	76·9 (76·4 to 77·4)	71·8 (71·7 to 71·9)	5·1	0·72
	Kuwait		63·5 (63·0 to 64·1)	57·8 (57·8 to 57·9)	5·7	69·9 (69·5 to 70·4)	70·9 (70·8 to 70·9)	−0·9	74·6 (74·3 to 74·9)	72·4 (72·3 to 72·5)	2·2	77·4 (77·2 to 77·7)	74·8 (74·7 to 74·8)	2·6	80·9 (80·4 to 81·4)	77·4 (77·3 to 77·4)	3·5	0·88
	Lebanon		57·3 (55·9 to 58·7)	57·8 (57·8 to 57·9)	−0·5	72·3 (71·5 to 73·2)	68·0 (68·0 to 68·1)	4·3	78·0 (77·3 to 78·7)	69·1 (69·0 to 69·1)	8·9	78·7 (78·1 to 79·2)	70·3 (70·2 to 70·3)	8·4	76·8 (75·6 to 78·1)	71·9 (71·9 to 72·0)	4·9	0·72
	Libya		36·7 (35·5 to 37·8)	46·3 (46·3 to 46·4)	−9·6	68·4 (68·1 to 68·7)	68·5 (68·4 to 68·5)	−0·0	71·3 (71·0 to 71·6)	70·8 (70·7 to 70·8)	0·5	72·8 (72·6 to 73·1)	72·5 (72·5 to 72·7)	0·3	70·3 (69·7 to 70·9)	73·4 (73·3 to 73·6)	−3·1	0·77
	Morocco		41·1 (39·4 to 42·8)	41·9 (41·7 to 42·1)	−0·8	65·0 (64·4 to 65·5)	59·8 (59·8 to 59·9)	5·1	68·3 (67·8 to 68·9)	62·9 (62·9 to 63·0)	5·4	71·2 (70·7 to 71·8)	66·1 (66·1 to 66·2)	5·1	73·3 (72·5 to 74·2)	69·2 (69·1 to 69·2)	4·1	0·61
	Oman		41·0 (39·6 to 42·4)	44·8 (44·7 to 44·9)	−3·8	66·2 (65·7 to 66·8)	63·6 (63·6 to 63·7)	2·6	70·3 (69·8 to 70·8)	69·4 (69·3 to 69·5)	0·9	71·5 (71·1 to 71·9)	71·6 (71·6 to 71·7)	−0·2	75·2 (74·4 to 75·9)	73·8 (73·7 to 73·9)	1·3	0·78
	Palestine		49·1 (47·5 to 50·1)	46·0 (45·9 to 46·0)	3·1	68·3 (68·0 to 68·7)	62·4 (62·4 to 62·5)	5·9	70·6 (70·3 to 70·9)	64·9 (64·9 to 65·0)	5·6	72·6 (72·3 to 72·8)	67·0 (66·9 to 67·0)	5·6	60·7 (59·4 to 62·0)	69·9 (69·8 to 69·9)	−9·1	0·64
	Qatar		47·9 (46·2 to 49·5)	53·9 (53·8 to 54·1)	−6·1	71·4 (70·8 to 72·0)	69·6 (69·6 to 69·7)	1·7	73·3 (72·7 to 73·9)	71·9 (71·9 to 72·0)	1·4	77·2 (76·7 to 77·8)	74·4 (74·3 to 74·5)	2·8	80·6 (79·6 to 81·6)	76·8 (76·8 to 76·9)	3·8	0·86
	Saudi Arabia		35·0 (33·1 to 36·8)	39·3 (39·0 to 39·6)	−4·3	66·3 (65·8 to 66·9)	63·6 (63·6 to 63·7)	2·7	71·1 (70·7 to 71·6)	70·0 (69·9 to 70·0)	1·2	69·7 (69·4 to 70·1)	72·7 (72·6 to 72·8)	−3·0	73·8 (73·0 to 74·6)	75·7 (75·7 to 75·8)	−1·9	0·83
	Sudan		40·3 (38·5 to 42·0)	44·0 (43·8 to 44·1)	−3·7	54·6 (53·8 to 55·4)	56·0 (56·0 to 56·1)	−1·5	58·7 (58·0 to 59·4)	59·5 (59·5 to 59·6)	−0·8	64·3 (63·5 to 65·0)	64·5 (64·5 to 64·6)	−0·3	67·3 (66·2 to 68·5)	68·3 (68·2 to 68·4)	−1·0	0·57
	Syria		52·7 (51·8 to 53·5)	47·1 (47·0 to 47·1)	5·6	67·3 (67·0 to 67·6)	62·4 (62·4 to 62·5)	4·8	69·9 (69·7 to 70·1)	64·3 (64·3 to 64·4)	5·6	73·2 (73·0 to 73·4)	67·0 (66·9 to 67·0)	6·2	73·9 (73·4 to 74·4)	68·7 (68·6 to 68·7)	5·2	0·59
	Tunisia		43·8 (42·9 to 44·7)	46·7 (46·6 to 46·8)	−2·9	66·8 (66·5 to 67·1)	64·7 (64·7 to 64·8)	2·0	69·1 (68·8 to 69·3)	67·8 (67·7 to 67·8)	1·3	71·2 (71·0 to 71·5)	69·5 (69·5 to 69·6)	1·7	73·4 (72·9 to 73·8)	70·9 (70·8 to 70·9)	2·5	0·68
	Türkiye		46·7 (45·0 to 48·3)	53·6 (53·5 to 53·8)	−7·0	65·2 (64·9 to 65·6)	65·2 (65·1 to 65·2)	0·1	69·2 (68·9 to 69·5)	67·8 (67·7 to 67·8)	1·5	74·6 (74·3 to 74·8)	69·5 (69·5 to 69·6)	5·0	73·9 (72·9 to 74·9)	72·1 (72·0 to 72·2)	1·8	0·73
	United Arab Emirates		44·9 (43·6 to 46·3)	49·2 (49·1 to 49·2)	−4·3	69·5 (69·0 to 70·0)	70·0 (69·9 to 70·0)	−0·4	71·7 (71·3 to 72·2)	73·2 (73·1 to 73·4)	−1·5	76·2 (75·8 to 76·6)	75·9 (75·9 to 75·9)	0·3	80·2 (79·5 to 80·9)	76·8 (76·8 to 76·9)	3·4	0·86
	Yemen		40·5 (38·7 to 42·3)	40·2 (39·9 to 40·5)	0·3	57·3 (56·5 to 58·2)	50·2 (50·1 to 50·3)	7·1	62·6 (61·9 to 63·4)	55·7 (55·7 to 55·8)	6·9	66·9 (66·2 to 67·6)	61·4 (61·4 to 61·5)	5·4	69·4 (68·4 to 70·6)	63·6 (63·6 to 63·7)	5·8	0·45
**South Asia**			**41·5 (40·1 to 42·9)**	**48·8 (48·8 to 48·9)**	**–7·3**	**58·3 (57·7 to 58·9)**	**57·5 (57·5 to 57·6)**	**0·8**	**62·6 (62·2 to 63·0)**	**60·9 (60·8 to 61·0)**	**1·7**	**65·8 (65·4 to 66·2)**	**64·3 (64·3 to 64·4)**	**1·5**	**70·1 (69·1 to 71·0)**	**68·6 (68·5 to 68·6)**	**1·5**	**0·59**
	Bangladesh		44·3 (42·7 to 45·8)	37·5 (37·0 to 37·8)	6·9	57·5 (56·7 to 58·3)	52·1 (52·0 to 52·2)	5·4	63·5 (62·8 to 64·3)	56·3 (56·3 to 56·4)	7·2	67·4 (66·7 to 68·0)	60·4 (60·3 to 60·4)	7·0	71·0 (69·9 to 72·2)	66·5 (66·4 to 66·5)	4·5	0·52
	Bhutan		39·4 (37·5 to 41·4)	38·8 (38·5 to 39·1)	0·6	60·0 (59·3 to 60·8)	51·5 (51·4 to 51·6)	8·6	63·9 (63·1 to 64·7)	56·3 (56·3 to 56·4)	7·6	69·4 (68·6 to 70·1)	62·2 (62·1 to 62·3)	7·2	72·9 (71·7 to 74·1)	66·8 (66·8 to 66·9)	6·0	0·52
	India		40·6 (38·9 to 42·3)	49·5 (49·5 to 49·6)	−8·9	58·1 (57·4 to 58·8)	58·1 (58·1 to 58·2)	0·0	62·5 (62·0 to 63·0)	61·7 (61·6 to 61·8)	0·8	65·8 (65·3 to 66·2)	64·9 (64·9 to 65·0)	0·8	70·2 (69·1 to 71·3)	69·1 (69·0 to 69·1)	1·2	0·61
	Nepal		45·9 (44·4 to 47·4)	42·8 (42·6 to 42·9)	3·1	57·9 (57·1 to 58·8)	50·2 (50·1 to 50·3)	7·7	63·9 (63·2 to 64·6)	54·5 (54·5 to 54·7)	9·4	68·5 (68·0 to 69·1)	59·5 (59·5 to 59·6)	9·0	71·9 (70·7 to 73·1)	64·7 (64·7 to 64·8)	7·2	0·47
	Pakistan		46·1 (44·5 to 47·7)	46·3 (46·3 to 46·4)	−0·2	61·7 (60·9 to 62·4)	56·6 (56·6 to 56·7)	5·1	63·5 (62·7 to 64·4)	59·8 (59·8 to 59·9)	3·7	65·9 (65·1 to 66·6)	62·9 (62·9 to 63·0)	2·9	70·0 (68·7 to 71·2)	66·5 (66·4 to 66·5)	3·5	0·51
**Southeast Asia, east Asia, and Oceania**			**44·2 (43·1 to 45·2)**	**50·2 (50·1 to 50·3)**	**–6·0**	**63·9 (63·4 to 64·4)**	**64·9 (64·9 to 65·0)**	**–1·1**	**67·1 (66·7 to 67·5)**	**67·8 (67·7 to 67·8)**	**–0·6**	**71·7 (71·3 to 72·0)**	**69·6 (69·6 to 69·7)**	**2·0**	**74·8 (74·1 to 75·6)**	**71·4 (71·3 to 71·4)**	**3·5**	**0·71**
East Asia			44·4 (43·0 to 45·8)	49·2 (49·1 to 49·2)	−4·7	64·5 (63·9 to 65·1)	64·9 (64·9 to 65·0)	−0·5	68·0 (67·5 to 68·5)	67·9 (67·8 to 67·9)	0·1	73·5 (73·0 to 73·9)	70·0 (69·9 to 70·0)	3·5	77·5 (76·5 to 78·6)	72·1 (72·0 to 72·2)	5·5	0·73
	China		45·3 (43·8 to 46·8)	48·8 (48·8 to 48·9)	−3·5	64·3 (63·7 to 64·9)	64·5 (64·5 to 64·6)	−0·2	67·9 (67·4 to 68·4)	67·6 (67·6 to 67·7)	0·3	73·5 (73·0 to 73·9)	69·9 (69·8 to 69·9)	3·6	77·6 (76·6 to 78·7)	72·1 (72·0 to 72·2)	5·6	0·73
	North Korea		19·5 (18·3 to 20·6)	54·8 (54·8 to 55·0)	−35·4	67·7 (67·0 to 68·4)	65·9 (65·9 to 66·0)	1·8	65·7 (63·5 to 67·2)	65·9 (65·9 to 66·0)	−0·2	69·7 (69·0 to 70·4)	67·3 (67·3 to 67·4)	2·4	71·3 (70·2 to 72·5)	68·3 (68·2 to 68·4)	3·0	0·58
	Taiwan*		55·3 (54·7 to 55·8)	55·1 (55·1 to 55·3)	0·1	71·9 (71·7 to 72·0)	70·8 (70·7 to 70·8)	1·1	74·1 (73·9 to 74·2)	73·1 (73·0 to 73·2)	1·0	76·4 (76·3 to 76·5)	75·7 (75·7 to 75·8)	0·6	77·3 (77·2 to 77·5)	77·5 (77·4 to 77·6)	−0·2	0·88
Oceania			47·9 (46·9 to 48·8)	50·8 (50·8 to 50·9)	−2·9	61·4 (60·9 to 61·9)	61·4 (61·4 to 61·5)	−0·0	62·8 (62·3 to 63·3)	63·2 (63·1 to 63·2)	−0·3	63·9 (63·4 to 64·4)	64·1 (64·0 to 64·1)	−0·2	65·6 (64·7 to 66·6)	64·9 (64·9 to 65·0)	0·7	0·48
	American Samoa		59·3 (58·3 to 60·3)	64·9 (64·9 to 65·0)	−5·7	66·3 (65·6 to 67·0)	69·3 (69·2 to 69·3)	−3·0	66·5 (65·8 to 67·1)	70·0 (69·9 to 70·0)	−3·5	67·8 (67·1 to 68·5)	70·6 (70·6 to 70·7)	−2·9	68·0 (66·8 to 69·2)	72·5 (72·5 to 72·7)	−4·6	0·74
	Cook Islands		53·4 (52·7 to 54·0)	60·6 (60·6 to 60·7)	−7·3	65·6 (65·2 to 65·9)	68·5 (68·4 to 68·5)	−2·8	68·0 (67·7 to 68·4)	70·4 (70·4 to 70·4)	−2·4	69·2 (68·9 to 69·6)	71·9 (71·9 to 72·0)	−2·7	69·7 (69·0 to 70·4)	73·6 (73·5 to 73·7)	−3·9	0·78
	Federated States of Micronesia		41·5 (40·8 to 42·1)	51·2 (51·1 to 51·3)	−9·7	57·9 (57·6 to 58·2)	64·5 (64·5 to 64·6)	−6·6	59·4 (59·1 to 59·7)	66·5 (66·4 to 66·5)	−7·1	61·8 (61·5 to 62·2)	67·6 (67·6 to 67·7)	−5·8	63·7 (63·0 to 64·2)	68·8 (68·8 to 68·9)	−5·2	0·60
	Fiji		59·5 (58·9 to 60·1)	55·7 (55·7 to 55·8)	3·8	64·7 (64·4 to 65·0)	67·3 (67·3 to 67·4)	−2·6	65·0 (64·6 to 65·3)	69·0 (68·9 to 69·0)	−4·0	65·5 (65·2 to 65·8)	69·7 (69·7 to 69·8)	−4·2	66·0 (65·4 to 66·5)	70·9 (70·8 to 70·9)	−4·9	0·69
	Guam		67·2 (66·7 to 67·8)	68·3 (68·2 to 68·4)	−1·1	71·3 (70·9 to 71·6)	70·8 (70·7 to 70·8)	0·5	72·7 (72·3 to 73·0)	71·9 (71·9 to 72·0)	0·8	73·1 (72·7 to 73·4)	72·5 (72·5 to 72·7)	0·5	71·9 (71·3 to 72·5)	75·0 (74·9 to 75·0)	−3·1	0·81
	Kiribati		46·0 (45·1 to 46·9)	55·1 (55·1 to 55·3)	−9·1	61·0 (60·6 to 61·3)	62·9 (62·9 to 63·0)	−2·0	62·9 (62·5 to 63·3)	64·3 (64·3 to 64·4)	−1·4	65·1 (64·8 to 65·5)	65·6 (65·5 to 65·6)	−0·4	67·6 (66·8 to 68·3)	67·5 (67·4 to 67·5)	0·1	0·54
	Marshall Islands		44·1 (43·3 to 44·8)	51·8 (51·7 to 51·9)	−7·7	58·7 (58·4 to 59·1)	63·4 (63·3 to 63·5)	−4·7	59·8 (59·5 to 60·2)	65·2 (65·1 to 65·2)	−5·3	61·4 (61·1 to 61·7)	66·7 (66·6 to 66·7)	−5·3	63·1 (62·4 to 63·7)	69·1 (69·0 to 69·1)	−6·0	0·61
	Nauru		47·1 (46·3 to 47·8)	63·9 (63·8 to 63·9)	−16·8	55·4 (55·0 to 55·8)	67·9 (67·8 to 67·9)	−12·5	54·7 (54·3 to 55·1)	67·0 (66·9 to 67·0)	−12·3	55·4 (55·0 to 55·8)	67·2 (67·1 to 67·2)	−11·8	57·2 (56·5 to 57·9)	69·5 (69·5 to 69·6)	−12·3	0·63
	Niue		50·3 (49·6 to 51·0)	58·7 (58·6 to 58·7)	−8·4	65·9 (65·6 to 66·2)	69·0 (68·9 to 69·0)	−3·1	67·2 (66·8 to 67·5)	70·0 (69·9 to 70·0)	−2·8	68·7 (68·4 to 69·1)	71·1 (71·1 to 71·2)	−2·4	69·5 (68·8 to 70·2)	72·7 (72·6 to 72·8)	−3·2	0·75
	Northern Mariana Islands		52·4 (51·8 to 53·0)	63·6 (63·6 to 63·7)	−11·3	66·0 (65·7 to 66·3)	71·6 (71·6 to 71·7)	−5·6	66·9 (66·6 to 67·2)	72·7 (72·6 to 72·8)	−5·8	67·9 (67·6 to 68·3)	73·1 (73·0 to 73·2)	−5·1	69·0 (68·5 to 69·7)	74·2 (74·1 to 74·3)	−5·2	0·79
	Palau		46·6 (45·8 to 47·3)	63·2 (63·1 to 63·2)	−16·6	64·0 (63·6 to 64·3)	70·9 (70·8 to 70·9)	−6·9	65·3 (65·0 to 65·6)	71·8 (71·7 to 71·9)	−6·5	66·5 (66·2 to 66·8)	72·2 (72·2 to 72·3)	−5·7	67·7 (67·2 to 68·4)	72·9 (72·8 to 73·0)	−5·1	0·76
	Papua New Guinea		45·0 (43·5 to 46·4)	45·2 (45·1 to 45·3)	−0·2	60·5 (59·8 to 61·2)	57·2 (57·2 to 57·3)	3·3	62·3 (61·6 to 63·0)	59·8 (59·8 to 59·9)	2·5	63·6 (62·9 to 64·3)	61·4 (61·4 to 61·5)	2·1	65·8 (64·6 to 67·1)	62·9 (62·9 to 63·0)	2·9	0·43
	Samoa		57·6 (56·8 to 58·3)	56·3 (56·3 to 56·4)	1·2	68·6 (68·2 to 69·0)	65·7 (65·7 to 65·8)	2·9	69·2 (68·8 to 69·6)	66·8 (66·8 to 66·9)	2·4	70·5 (70·2 to 70·9)	68·0 (68·0 to 68·1)	2·5	70·9 (70·2 to 71·6)	69·1 (69·0 to 69·1)	1·8	0·60
	Solomon Islands		49·9 (49·3 to 50·5)	48·1 (48·1 to 48·2)	1·7	59·1 (58·8 to 59·5)	56·9 (56·9 to 57·0)	2·2	60·8 (60·5 to 61·2)	60·1 (60·0 to 60·2)	0·8	62·0 (61·6 to 62·3)	61·7 (61·6 to 61·8)	0·3	61·9 (61·3 to 62·6)	64·3 (64·3 to 64·4)	−2·4	0·46
	Tokelau		51·3 (50·5 to 52·1)	54·5 (54·5 to 54·7)	−3·2	70·1 (69·8 to 70·4)	67·8 (67·7 to 67·8)	2·3	71·6 (71·3 to 71·9)	69·1 (69·0 to 69·1)	2·5	73·4 (73·0 to 73·7)	70·4 (70·4 to 70·4)	2·9	74·7 (74·1 to 75·4)	72·7 (72·6 to 72·8)	2·0	0·75
	Tonga		56·0 (55·4 to 56·8)	55·1 (55·1 to 55·3)	0·9	68·0 (67·7 to 68·3)	66·3 (66·3 to 66·3)	1·7	68·8 (68·5 to 69·1)	67·8 (67·7 to 67·8)	1·0	69·8 (69·5 to 70·1)	68·5 (68·4 to 68·5)	1·4	70·4 (69·7 to 71·0)	69·7 (69·7 to 69·8)	0·6	0·64
	Tuvalu		44·7 (43·9 to 45·5)	55·4 (55·4 to 55·5)	−10·8	56·2 (55·9 to 56·6)	63·6 (63·6 to 63·7)	−7·4	54·9 (54·4 to 55·3)	66·3 (66·3 to 66·3)	−11·4	60·0 (59·7 to 60·4)	67·8 (67·7 to 67·8)	−7·8	60·4 (59·7 to 61·1)	69·2 (69·1 to 69·2)	−8·8	0·61
	Vanuatu		50·8 (50·1 to 51·5)	50·2 (50·1 to 50·3)	0·6	62·9 (62·5 to 63·3)	59·3 (59·2 to 59·3)	3·6	64·4 (64·0 to 64·8)	61·2 (61·1 to 61·2)	3·2	66·2 (65·8 to 66·6)	62·9 (62·9 to 63·0)	3·3	67·4 (66·6 to 68·2)	64·9 (64·9 to 65·0)	2·5	0·48
Southeast Asia			43·3 (42·4 to 44·2)	51·5 (51·4 to 51·6)	−8·2	62·6 (62·2 to 62·9)	64·7 (64·7 to 64·8)	−2·2	65·4 (65·1 to 65·7)	67·3 (67·3 to 67·4)	−1·9	67·8 (67·4 to 68·1)	68·7 (68·6 to 68·7)	−0·9	70·1 (69·6 to 70·7)	70·3 (70·2 to 70·3)	−0·2	0·66
	Cambodia		41·7 (40·0 to 43·3)	49·2 (49·1 to 49·2)	−7·5	55·2 (54·2 to 56·1)	55·7 (55·7 to 55·8)	−0·6	59·4 (58·5 to 60·3)	58·4 (58·3 to 58·5)	1·0	67·0 (66·3 to 67·8)	62·7 (62·6 to 62·8)	4·4	70·8 (69·5 to 72·0)	65·9 (65·9 to 66·0)	4·8	0·50
	Indonesia		41·1 (39·5 to 42·8)	49·9 (49·8 to 49·9)	−8·8	62·5 (61·6 to 63·3)	64·3 (64·3 to 64·4)	−1·8	66·4 (65·6 to 67·1)	67·2 (67·1 to 67·2)	−0·8	67·3 (66·5 to 68·1)	68·6 (68·5 to 68·6)	−1·3	70·0 (68·7 to 71·3)	70·4 (70·4 to 70·4)	−0·4	0·67
	Laos		32·9 (30·9 to 34·8)	45·6 (45·5 to 45·7)	−12·6	48·9 (47·9 to 50·1)	54·5 (54·5 to 54·7)	−5·6	54·9 (54·0 to 55·8)	57·8 (57·8 to 57·9)	−2·9	62·2 (61·4 to 63·0)	62·9 (62·9 to 63·0)	−0·7	65·2 (63·9 to 66·5)	66·5 (66·4 to 66·5)	−1·3	0·52
	Malaysia		48·0 (46·8 to 49·2)	51·2 (51·1 to 51·3)	−3·1	69·3 (68·9 to 69·7)	68·2 (68·1 to 68·2)	1·1	70·5 (70·2 to 70·8)	70·2 (70·1 to 70·2)	0·3	72·8 (72·1 to 73·5)	71·5 (71·5 to 71·6)	1·3	74·6 (74·2 to 75·1)	73·4 (73·3 to 73·6)	1·2	0·77
	Maldives		45·5 (44·4 to 46·5)	49·9 (49·8 to 49·9)	−4·4	65·0 (64·7 to 65·4)	57·8 (57·8 to 57·9)	7·2	70·7 (70·4 to 71·0)	65·2 (65·1 to 65·2)	5·6	73·9 (73·6 to 74·2)	68·3 (68·2 to 68·4)	5·6	75·2 (74·7 to 75·7)	70·3 (70·2 to 70·3)	4·9	0·66
	Mauritius		50·8 (50·2 to 51·4)	56·9 (56·9 to 57·0)	−6·2	66·1 (65·7 to 66·4)	67·9 (67·8 to 67·9)	−1·8	68·5 (68·2 to 68·9)	69·4 (69·3 to 69·5)	−0·9	70·6 (70·3 to 70·9)	70·6 (70·6 to 70·7)	−0·0	70·3 (69·8 to 70·8)	72·2 (72·2 to 72·3)	−1·9	0·74
	Myanmar		32·9 (31·0 to 34·6)	45·6 (45·5 to 45·7)	−12·6	51·0 (50·1 to 51·9)	57·2 (57·2 to 57·3)	−6·2	55·1 (54·3 to 56·0)	60·1 (60·0 to 60·2)	−5·0	59·5 (58·8 to 60·4)	64·5 (64·5 to 64·6)	−5·0	61·6 (60·1 to 63·0)	67·0 (66·9 to 67·0)	−5·4	0·53
	Philippines		54·9 (54·3 to 55·5)	57·5 (57·5 to 57·6)	−2·7	65·6 (65·4 to 65·9)	66·5 (66·4 to 66·5)	−0·8	67·1 (66·9 to 67·3)	67·6 (67·6 to 67·7)	−0·5	67·4 (67·2 to 67·6)	68·3 (68·2 to 68·4)	−0·9	68·9 (68·6 to 69·2)	70·5 (70·5 to 70·6)	−1·6	0·67
	Seychelles		57·5 (57·0 to 58·0)	61·4 (61·4 to 61·5)	−3·9	66·1 (65·7 to 66·4)	68·8 (68·8 to 68·9)	−2·7	67·7 (67·3 to 68·0)	70·5 (70·5 to 70·6)	−2·9	69·3 (69·0 to 69·7)	71·2 (71·2 to 71·3)	−1·9	72·2 (71·7 to 72·7)	72·9 (72·8 to 73·0)	−0·7	0·75
	Sri Lanka		55·6 (55·1 to 56·1)	59·5 (59·5 to 59·6)	−3·9	65·5 (65·2 to 65·9)	67·5 (67·4 to 67·5)	−1·9	67·5 (67·3 to 67·8)	69·0 (68·9 to 69·0)	−1·4	70·7 (70·4 to 70·9)	70·2 (70·1 to 70·2)	0·5	73·6 (73·3 to 73·9)	72·1 (72·0 to 72·2)	1·5	0·73
	Thailand		52·2 (51·7 to 52·6)	52·1 (52·0 to 52·2)	0·1	68·6 (68·4 to 68·8)	65·9 (65·9 to 66·0)	2·6	67·4 (67·2 to 67·6)	68·3 (68·2 to 68·4)	−1·0	72·0 (71·8 to 72·1)	69·4 (69·3 to 69·5)	2·5	73·7 (73·4 to 73·9)	70·9 (70·8 to 70·9)	2·8	0·69
	Timor-Leste		38·5 (36·4 to 40·5)	43·6 (43·4 to 43·7)	−5·1	56·8 (56·0 to 57·7)	54·2 (54·1 to 54·4)	2·6	62·9 (62·1 to 63·6)	59·0 (58·9 to 59·0)	3·9	67·7 (67·0 to 68·4)	61·7 (61·6 to 61·8)	6·0	69·6 (68·4 to 70·9)	65·4 (65·3 to 65·4)	4·3	0·48
	Viet Nam		46·3 (45·0 to 47·4)	50·8 (50·8 to 50·9)	−4·6	64·9 (64·3 to 65·4)	62·2 (62·1 to 62·3)	2·7	68·0 (67·4 to 68·5)	65·9 (65·9 to 66·0)	2·0	69·7 (69·0 to 70·5)	68·2 (68·1 to 68·2)	1·5	71·9 (70·6 to 73·2)	70·0 (69·9 to 70·0)	2·0	0·64
**Sub-Saharan Africa**			**40·8 (40·3 to 41·3)**	**46·3 (46·3 to 46·4)**	**–5·5**	**50·0 (49·7 to 50·2)**	**55·4 (55·4 to 55·5)**	**–5·5**	**50·8 (50·5 to 51·1)**	**57·5 (57·5 to 57·6)**	**–6·7**	**57·3 (57·1 to 57·5)**	**60·6 (60·6 to 60·7)**	**–3·3**	**62·2 (62·0 to 62·5)**	**64·9 (64·9 to 65·0)**	**–2·7**	**0·47**
Central sub-Saharan Africa			39·6 (38·5 to 40·6)	46·3 (46·3 to 46·4)	−6·7	49·3 (48·7 to 49·9)	56·0 (56·0 to 56·1)	−6·7	50·4 (49·8 to 51·1)	57·2 (57·2 to 57·3)	−6·8	55·8 (55·3 to 56·4)	60·6 (60·6 to 60·7)	−4·8	60·0 (59·3 to 60·8)	65·2 (65·1 to 65·2)	−5·1	0·48
	Angola		37·5 (35·8 to 39·0)	45·2 (45·1 to 45·3)	−7·7	46·4 (45·5 to 47·4)	54·2 (54·1 to 54·4)	−7·8	50·5 (49·6 to 51·3)	56·6 (56·6 to 56·7)	−6·2	56·4 (55·6 to 57·1)	61·2 (61·1 to 61·2)	−4·8	60·7 (59·5 to 62·0)	65·9 (65·9 to 66·0)	−5·2	0·50
	Central African Republic		40·0 (38·4 to 41·4)	42·4 (42·1 to 42·5)	−2·4	46·9 (46·0 to 47·8)	49·9 (49·8 to 49·9)	−3·0	45·0 (44·1 to 45·8)	51·2 (51·1 to 51·3)	−6·1	50·6 (49·7 to 51·4)	51·8 (51·7 to 51·9)	−1·1	54·3 (53·2 to 55·4)	53·3 (53·2 to 53·5)	1·0	0·26
	Congo (Brazzaville)		36·6 (35·0 to 38·2)	47·4 (47·4 to 47·5)	−10·8	53·7 (52·9 to 54·6)	62·2 (62·1 to 62·3)	−8·5	54·4 (53·6 to 55·2)	64·1 (64·0 to 64·1)	−9·7	61·0 (60·3 to 61·6)	65·7 (65·7 to 65·8)	−4·8	62·9 (61·7 to 64·1)	68·5 (68·4 to 68·5)	−5·5	0·58
	DR Congo		40·7 (39·1 to 42·3)	46·3 (46·3 to 46·4)	−5·6	49·9 (49·1 to 50·8)	55·1 (55·1 to 55·3)	−5·2	50·5 (49·6 to 51·4)	53·6 (53·5 to 53·8)	−3·2	55·6 (54·8 to 56·4)	54·8 (54·8 to 55·0)	0·7	60·0 (58·9 to 61·1)	61·2 (61·1 to 61·2)	−1·2	0·40
	Equatorial Guinea		31·6 (30·0 to 33·3)	42·8 (42·6 to 42·9)	−11·2	45·9 (45·1 to 46·8)	53·9 (53·8 to 54·1)	−8·0	51·0 (50·2 to 51·8)	61·9 (61·9 to 62·0)	−11·0	56·8 (56·1 to 57·5)	67·5 (67·4 to 67·5)	−10·7	58·6 (57·2 to 59·8)	70·0 (69·9 to 70·0)	−11·4	0·65
	Gabon		38·5 (36·7 to 40·0)	47·1 (47·0 to 47·1)	−8·6	59·7 (58·8 to 60·4)	62·7 (62·6 to 62·8)	−3·0	60·6 (59·9 to 61·4)	66·1 (66·1 to 66·2)	−5·5	63·9 (63·2 to 64·6)	67·8 (67·7 to 67·8)	−3·9	66·4 (65·2 to 67·7)	69·6 (69·6 to 69·7)	−3·2	0·63
Eastern sub-Saharan Africa			41·3 (40·7 to 41·9)	43·2 (43·0 to 43·3)	−1·9	47·4 (47·0 to 47·8)	51·8 (51·7 to 51·9)	−4·4	49·7 (49·2 to 50·0)	53·6 (53·5 to 53·8)	−4·0	58·3 (58·0 to 58·6)	57·5 (57·5 to 57·6)	0·7	63·3 (62·9 to 63·7)	62·9 (62·9 to 63·0)	0·3	0·43
	Burundi		37·9 (36·1 to 39·6)	41·5 (41·3 to 41·7)	−3·7	45·5 (44·5 to 46·5)	50·5 (50·4 to 50·6)	−5·0	41·7 (40·8 to 42·6)	51·2 (51·1 to 51·3)	−9·4	56·6 (55·8 to 57·4)	52·7 (52·6 to 52·8)	3·9	59·8 (58·6 to 61·0)	56·0 (56·0 to 56·1)	3·7	0·31
	Comoros		48·8 (47·3 to 50·2)	42·8 (42·6 to 42·9)	6·0	64·4 (63·6 to 65·2)	54·2 (54·1 to 54·4)	10·1	68·2 (67·5 to 68·9)	58·7 (58·6 to 58·7)	9·5	72·9 (72·2 to 73·6)	61·9 (61·9 to 62·0)	11·0	74·3 (73·1 to 75·5)	65·2 (65·1 to 65·2)	9·1	0·48
	Djibouti		47·7 (46·4 to 49·0)	47·1 (47·0 to 47·1)	0·6	59·4 (58·7 to 60·3)	57·8 (57·8 to 57·9)	1·6	61·3 (60·5 to 62·0)	59·3 (59·2 to 59·3)	2·0	63·5 (62·7 to 64·3)	61·4 (61·4 to 61·5)	2·1	67·2 (66·0 to 68·5)	65·6 (65·5 to 65·6)	1·7	0·49
	Eritrea		32·4 (30·6 to 34·2)	38·8 (38·5 to 39·1)	−6·5	40·5 (39·0 to 41·9)	50·8 (50·8 to 50·9)	−10·4	53·6 (52·7 to 54·4)	56·9 (56·9 to 57·0)	−3·3	58·3 (57·4 to 59·1)	59·0 (58·9 to 59·0)	−0·7	61·3 (60·0 to 62·5)	62·2 (62·1 to 62·3)	−0·9	0·41
	Ethiopia		39·1 (37·6 to 40·7)	38·8 (38·5 to 39·1)	0·3	41·5 (40·6 to 42·3)	45·2 (45·1 to 45·3)	−3·7	47·8 (46·4 to 48·8)	47·1 (47·0 to 47·1)	0·7	58·9 (58·2 to 59·6)	52·7 (52·6 to 52·8)	6·2	63·0 (61·8 to 64·1)	61·2 (61·1 to 61·2)	1·8	0·39
	Kenya		46·3 (44·9 to 47·7)	42·8 (42·6 to 42·9)	3·5	60·2 (59·4 to 60·9)	58·1 (58·1 to 58·2)	2·1	55·7 (54·9 to 56·6)	60·6 (60·6 to 60·7)	−4·9	62·6 (61·9 to 63·4)	63·2 (63·1 to 63·2)	−0·5	68·2 (66·9 to 69·4)	67·6 (67·6 to 67·7)	0·6	0·55
	Madagascar		41·4 (40·0 to 42·9)	44·8 (44·7 to 44·9)	−3·4	50·5 (49·7 to 51·4)	53·9 (53·8 to 54·1)	−3·4	55·8 (55·1 to 56·6)	54·2 (54·1 to 54·4)	1·6	59·3 (58·6 to 59·9)	55·7 (55·7 to 55·8)	3·5	61·2 (60·1 to 62·2)	60·4 (60·3 to 60·4)	0·8	0·38
	Malawi		39·9 (38·1 to 41·6)	40·2 (39·9 to 40·5)	−0·3	43·9 (42·9 to 44·8)	50·5 (50·4 to 50·6)	−6·7	44·2 (43·3 to 45·0)	52·4 (52·3 to 52·5)	−8·2	55·0 (54·2 to 55·9)	56·0 (56·0 to 56·1)	−1·0	59·7 (58·5 to 60·8)	61·9 (61·9 to 62·0)	−2·3	0·41
	Mozambique		44·7 (43·1 to 46·3)	41·5 (41·3 to 41·7)	3·2	49·5 (48·5 to 50·4)	48·1 (48·1 to 48·2)	1·3	51·7 (50·9 to 52·6)	49·9 (49·8 to 49·9)	1·9	56·9 (56·1 to 57·7)	53·0 (52·9 to 53·2)	3·9	62·0 (60·8 to 63·2)	58·1 (58·1 to 58·2)	3·9	0·34
	Rwanda		33·8 (32·1 to 35·5)	44·4 (44·3 to 44·5)	−10·6	43·0 (42·0 to 43·9)	54·8 (54·8 to 55·0)	−11·9	43·4 (42·5 to 44·3)	55·1 (55·1 to 55·3)	−11·7	59·3 (58·6 to 60·0)	59·3 (59·2 to 59·3)	0·1	61·8 (60·7 to 63·0)	64·3 (64·3 to 64·4)	−2·5	0·46
	Somalia		49·3 (47·9 to 50·8)	35·0 (34·5 to 35·5)	14·3	50·2 (49·2 to 51·2)	37·5 (37·0 to 37·8)	12·8	52·6 (51·7 to 53·5)	37·9 (37·5 to 38·3)	14·7	50·3 (49·4 to 51·2)	43·6 (43·4 to 43·7)	6·7	62·6 (61·6 to 63·7)	51·5 (51·4 to 51·6)	11·2	0·23
	South Sudan		42·8 (41·3 to 44·3)	49·2 (49·1 to 49·2)	−6·4	47·8 (46·8 to 48·7)	54·5 (54·5 to 54·7)	−6·8	50·5 (49·6 to 51·4)	55·7 (55·7 to 55·8)	−5·2	54·0 (53·2 to 54·8)	59·0 (58·9 to 59·0)	−5·0	54·1 (52·9 to 55·4)	60·4 (60·3 to 60·4)	−6·2	0·38
	Uganda		42·7 (41·1 to 44·2)	41·1 (40·8 to 41·3)	1·6	44·3 (43·5 to 45·2)	48·5 (48·5 to 48·5)	−4·2	46·1 (45·2 to 47·0)	51·5 (51·4 to 51·6)	−5·3	56·5 (55·7 to 57·3)	57·5 (57·5 to 57·6)	−1·0	63·1 (61·9 to 64·3)	63·4 (63·3 to 63·5)	−0·3	0·44
	Tanzania		43·8 (42·1 to 45·2)	41·9 (41·7 to 42·1)	1·8	53·8 (52·9 to 54·6)	53·6 (53·5 to 53·8)	0·1	53·8 (52·9 to 54·6)	55·4 (55·4 to 55·5)	−1·7	61·5 (60·7 to 62·3)	59·3 (59·2 to 59·3)	2·2	67·8 (66·6 to 68·9)	64·5 (64·5 to 64·6)	3·3	0·47
	Zambia		42·2 (40·7 to 43·6)	44·8 (44·7 to 44·9)	−2·6	47·8 (46·9 to 48·7)	56·3 (56·3 to 56·4)	−8·5	42·5 (41·7 to 43·4)	56·9 (56·9 to 57·0)	−14·4	53·9 (53·1 to 54·6)	60·6 (60·6 to 60·7)	−6·8	60·4 (59·1 to 61·6)	66·1 (66·1 to 66·2)	−5·8	0·51
Southern sub-Saharan Africa			47·5 (46·4 to 48·6)	56·0 (56·0 to 56·1)	−8·5	62·0 (61·3 to 62·7)	66·5 (66·4 to 66·5)	−4·5	52·6 (52·3 to 52·9)	68·0 (68·0 to 68·1)	−15·4	55·2 (54·9 to 55·4)	69·1 (69·0 to 69·1)	−13·9	62·9 (62·6 to 63·2)	70·3 (70·2 to 70·3)	−7·4	0·66
	Botswana		44·2 (43·4 to 45·0)	44·8 (44·7 to 44·9)	−0·6	61·0 (60·7 to 61·4)	63·4 (63·3 to 63·5)	−2·4	46·6 (46·2 to 47·0)	67·5 (67·4 to 67·5)	−20·9	63·1 (62·8 to 63·4)	69·5 (69·5 to 69·6)	−6·5	67·4 (66·7 to 67·9)	70·8 (70·7 to 70·8)	−3·4	0·68
	Eswatini		36·2 (34·8 to 37·5)	44·8 (44·7 to 44·9)	−8·6	52·5 (51·6 to 53·3)	61·4 (61·4 to 61·5)	−8·9	46·6 (45·7 to 47·5)	64·9 (64·9 to 65·0)	−18·4	45·0 (44·0 to 46·0)	67·2 (67·1 to 67·2)	−22·1	58·8 (57·4 to 60·2)	69·1 (69·0 to 69·1)	−10·3	0·61
	Lesotho		43·1 (41·7 to 44·3)	47·4 (47·4 to 47·5)	−4·4	55·1 (54·4 to 55·9)	58·4 (58·3 to 58·5)	−3·3	45·3 (44·4 to 46·1)	61·4 (61·4 to 61·5)	−16·1	50·1 (49·3 to 51·0)	63·9 (63·8 to 63·9)	−13·7	55·3 (53·9 to 56·7)	66·3 (66·3 to 66·3)	−11·0	0·51
	Namibia		40·6 (39·8 to 41·3)	49·9 (49·8 to 49·9)	−9·3	56·4 (56·0 to 56·8)	63·2 (63·1 to 63·2)	−6·8	52·1 (51·6 to 52·5)	65·7 (65·7 to 65·8)	−13·7	58·9 (58·5 to 59·3)	67·5 (67·4 to 67·5)	−8·6	63·4 (62·6 to 64·1)	69·4 (69·3 to 69·5)	−6·0	0·62
	South Africa		48·1 (46·6 to 49·6)	58·1 (58·1 to 58·2)	−10·0	63·0 (62·1 to 63·9)	67·5 (67·4 to 67·5)	−4·5	55·6 (55·3 to 55·9)	69·0 (68·9 to 69·0)	−13·3	55·4 (55·1 to 55·7)	69·9 (69·8 to 69·9)	−14·5	63·3 (63·0 to 63·5)	71·1 (71·1 to 71·2)	−7·8	0·70
	Zimbabwe		49·1 (47·8 to 50·4)	48·1 (48·1 to 48·2)	1·0	61·4 (60·6 to 62·1)	62·2 (62·1 to 62·3)	−0·8	44·6 (43·7 to 45·5)	64·1 (64·0 to 64·1)	−19·5	54·8 (53·9 to 55·6)	62·9 (62·9 to 63·0)	−8·2	63·8 (62·5 to 65·1)	65·6 (65·5 to 65·6)	−1·8	0·49
Western sub-Saharan Africa			39·5 (38·6 to 40·5)	45·2 (45·1 to 45·3)	−5·7	50·3 (49·8 to 50·7)	53·9 (53·8 to 54·1)	−3·7	52·1 (51·7 to 52·5)	56·3 (56·3 to 56·4)	−4·3	57·8 (57·5 to 58·2)	59·8 (59·8 to 59·9)	−2·0	62·3 (61·8 to 62·8)	64·3 (64·3 to 64·4)	−2·0	0·46
	Benin		40·5 (38·8 to 42·1)	42·8 (42·6 to 42·9)	−2·3	51·3 (50·4 to 52·1)	50·8 (50·8 to 50·9)	0·4	55·2 (54·4 to 56·0)	53·0 (52·9 to 53·2)	2·2	59·4 (58·6 to 60·1)	55·7 (55·7 to 55·8)	3·6	63·4 (62·3 to 64·6)	61·7 (61·6 to 61·8)	1·8	0·41
	Burkina Faso		41·7 (39·8 to 43·5)	38·4 (38·0 to 38·7)	3·3	48·3 (47·3 to 49·3)	44·8 (44·7 to 44·9)	3·5	51·0 (50·0 to 51·9)	47·8 (47·7 to 47·8)	3·2	60·9 (60·1 to 61·6)	51·2 (51·1 to 51·3)	9·7	65·4 (64·3 to 66·5)	56·0 (56·0 to 56·1)	9·3	0·31
	Cabo Verde		56·2 (54·9 to 57·5)	46·3 (46·3 to 46·4)	9·9	66·4 (65·8 to 67·1)	55·1 (55·1 to 55·3)	11·3	68·6 (67·9 to 69·2)	60·1 (60·0 to 60·2)	8·5	71·2 (70·5 to 71·8)	64·7 (64·7 to 64·8)	6·4	72·8 (71·8 to 73·9)	67·9 (67·8 to 67·9)	4·9	0·56
	Cameroon		38·3 (36·5 to 39·9)	44·8 (44·7 to 44·9)	−6·5	53·3 (52·4 to 54·1)	56·0 (56·0 to 56·1)	−2·7	51·0 (50·1 to 51·8)	58·7 (58·6 to 58·7)	−7·7	55·0 (54·2 to 55·8)	61·2 (61·1 to 61·2)	−6·2	61·1 (59·9 to 62·2)	65·4 (65·3 to 65·4)	−4·3	0·49
	Chad		41·8 (40·3 to 43·3)	38·8 (38·5 to 39·1)	2·9	46·7 (45·8 to 47·6)	43·2 (43·0 to 43·3)	3·5	48·3 (47·5 to 49·1)	45·2 (45·1 to 45·3)	3·2	52·9 (52·0 to 53·7)	48·5 (48·5 to 48·5)	4·4	56·7 (55·6 to 57·8)	52·1 (52·0 to 52·2)	4·6	0·24
	Côte d'Ivoire		39·8 (38·1 to 41·3)	43·6 (43·4 to 43·7)	−3·8	53·0 (52·2 to 53·9)	54·8 (54·8 to 55·0)	−1·8	51·8 (51·0 to 52·7)	57·8 (57·8 to 57·9)	−6·0	58·5 (57·7 to 59·3)	59·5 (59·5 to 59·6)	−1·1	62·6 (61·4 to 63·7)	63·9 (63·8 to 63·9)	−1·3	0·45
	The Gambia		47·0 (45·5 to 48·5)	44·8 (44·7 to 44·9)	2·2	59·0 (58·1 to 59·8)	52·4 (52·3 to 52·5)	6·6	61·0 (60·3 to 61·7)	56·0 (56·0 to 56·1)	4·9	64·3 (63·5 to 65·0)	59·0 (58·9 to 59·0)	5·3	66·1 (64·9 to 67·2)	63·2 (63·1 to 63·2)	2·9	0·43
	Ghana		43·5 (42·1 to 44·9)	51·8 (51·7 to 51·9)	−8·3	54·2 (53·4 to 54·9)	60·1 (60·0 to 60·2)	−5·9	56·5 (55·8 to 57·2)	63·2 (63·1 to 63·2)	−6·7	59·8 (59·1 to 60·5)	65·4 (65·3 to 65·4)	−5·5	62·9 (61·7 to 64·0)	68·3 (68·2 to 68·4)	−5·4	0·57
	Guinea		36·7 (34·9 to 38·5)	38·8 (38·5 to 39·1)	−2·1	47·9 (46·9 to 48·9)	48·5 (48·5 to 48·5)	−0·6	52·3 (51·4 to 53·2)	51·2 (51·1 to 51·3)	1·2	57·6 (56·9 to 58·4)	53·6 (53·5 to 53·8)	4·0	61·8 (60·6 to 62·9)	59·0 (58·9 to 59·0)	2·8	0·36
	Guinea-Bissau		36·2 (34·2 to 38·0)	39·3 (39·0 to 39·6)	−3·1	48·8 (47·8 to 49·8)	50·2 (50·1 to 50·3)	−1·4	51·8 (51·0 to 52·7)	53·0 (52·9 to 53·2)	−1·2	56·7 (55·9 to 57·4)	55·4 (55·4 to 55·5)	1·2	60·5 (59·4 to 61·6)	60·1 (60·0 to 60·2)	0·5	0·37
	Liberia		39·0 (37·3 to 40·6)	46·0 (45·9 to 46·0)	−7·0	43·4 (42·4 to 44·4)	52·4 (52·3 to 52·5)	−9·0	49·7 (48·9 to 50·6)	51·8 (51·7 to 51·9)	−2·0	56·8 (56·0 to 57·5)	55·1 (55·1 to 55·3)	1·6	60·4 (59·3 to 61·5)	60·6 (60·6 to 60·7)	−0·2	0·38
	Mali		39·1 (37·0 to 41·0)	38·8 (38·5 to 39·1)	0·3	47·7 (46·6 to 48·8)	44·4 (44·3 to 44·5)	3·3	52·0 (51·1 to 52·9)	46·7 (46·6 to 46·8)	5·3	59·1 (58·3 to 59·9)	49·9 (49·8 to 49·9)	9·3	62·4 (61·2 to 63·5)	54·8 (54·8 to 55·0)	7·6	0·28
	Mauritania		50·4 (49·1 to 51·7)	49·9 (49·8 to 49·9)	0·6	59·5 (58·7 to 60·2)	58·1 (58·1 to 58·2)	1·4	62·4 (61·7 to 63·1)	60·9 (60·8 to 61·0)	1·5	65·0 (64·3 to 65·7)	62·7 (62·6 to 62·8)	2·3	67·5 (66·5 to 68·6)	66·7 (66·6 to 66·7)	0·9	0·52
	Niger		39·3 (37·2 to 41·3)	37·5 (37·0 to 37·8)	1·8	42·1 (40·8 to 43·4)	40·6 (40·4 to 40·9)	1·5	47·7 (46·6 to 48·8)	42·8 (42·6 to 42·9)	4·9	54·4 (53·5 to 55·3)	45·2 (45·1 to 45·3)	9·2	58·2 (57·1 to 59·3)	49·9 (49·8 to 49·9)	8·4	0·20
	Nigeria		38·7 (36·8 to 40·6)	46·0 (45·9 to 46·0)	−7·2	50·3 (49·3 to 51·2)	55·7 (55·7 to 55·8)	−5·5	51·9 (51·0 to 52·6)	57·5 (57·5 to 57·6)	−5·7	57·9 (57·2 to 58·8)	61·7 (61·6 to 61·8)	−3·7	63·1 (61·9 to 64·1)	66·3 (66·3 to 66·3)	−3·2	0·51
	São Tomé and Príncipe		45·6 (44·0 to 47·4)	48·5 (48·5 to 48·5)	−2·9	61·7 (60·9 to 62·5)	56·9 (56·9 to 57·0)	4·8	62·2 (61·5 to 62·9)	58·1 (58·1 to 58·2)	4·1	64·3 (63·6 to 65·0)	61·7 (61·6 to 61·8)	2·6	68·6 (67·4 to 69·7)	66·8 (66·8 to 66·9)	1·8	0·52
	Senegal		44·4 (42·7 to 45·9)	41·5 (41·3 to 41·7)	2·8	55·2 (54·3 to 56·1)	52·1 (52·0 to 52·2)	3·1	59·3 (58·5 to 60·1)	55·1 (55·1 to 55·3)	4·2	65·1 (64·4 to 65·8)	57·5 (57·5 to 57·6)	7·6	68·5 (67·4 to 69·7)	62·2 (62·1 to 62·3)	6·3	0·41
	Sierra Leone		37·9 (36·4 to 39·5)	44·0 (43·8 to 44·1)	−6·1	44·9 (43·9 to 46·0)	50·5 (50·4 to 50·6)	−5·6	46·2 (45·4 to 47·2)	50·8 (50·8 to 50·9)	−4·6	52·1 (51·3 to 53·0)	54·8 (54·8 to 55·0)	−2·7	57·3 (56·1 to 58·5)	60·6 (60·6 to 60·7)	−3·3	0·38
	Togo		38·4 (36·6 to 40·2)	42·8 (42·6 to 42·9)	−4·4	53·4 (52·5 to 54·3)	55·1 (55·1 to 55·3)	−1·7	53·6 (52·8 to 54·5)	57·5 (57·5 to 57·6)	−3·9	57·7 (56·9 to 58·5)	59·5 (59·5 to 59·6)	−1·8	62·8 (61·6 to 63·9)	64·3 (64·3 to 64·4)	−1·5	0·46

Data in parentheses are 95% uncertainty intervals. Life expectancies and differences between estimated and expected life expectancies are given in years. A positive difference indicates that the estimated life expectancy is better than would be expected solely on the basis of SDI, while a negative difference indicates worse than expected life expectancy. SDI=Socio-demographic Index. GBD=Global Burden of Diseases, Injuries, and Risk Factors Study.

* UN convention recognises Taiwan as a province of China.

## Discussion

### Main findings

This novel demographic analysis identified several key trends in all-cause mortality across age, location, and time. First, life expectancy and mortality have largely recovered to pre-COVID-19 pandemic levels, with essentially no difference in life expectancy or age-standardised mortality rates between 2019 and 2023 at the global level and in approximately two-thirds of countries and territories. That said, these broad patterns mask considerable heterogeneity in life expectancy and mortality trajectories during and following the pandemic at the national level. We also found that mortality patterns in recent years (2011–23) varied by age group and location, including widespread declines in the under-5 age group, with the largest declines occurring in east Asia; decreases in those aged 5–19 years across nearly all regions except for eastern Europe, high-income North America, and the Caribbean; and the largest increases in those aged 20–39 years occurring in high-income North America. These and other age-location-specific findings indicate that mortality rates in certain populations are substantially higher or lower than previously estimated by GBD, UNPD, and other major demographic studies. In particular, we identified higher than previously estimated mortality rates in sub-Saharan Africa for adolescents and young adult females, as well as lower than previously estimated rates mortality in older age groups in sub-Saharan Africa, reflecting advances in our modelling approach to incorporate previously unusable and more readily available and reliable data sources.

### Data availability and gaps

While the completeness of death registration has continuously increased globally since 1950, this progress has not been observed in all GBD super-regions. Civil registration and vital statistics are overwhelmingly scarce in sub-Saharan Africa, contributing to wide uncertainty for many estimates in this super-region. While South Africa has the best functioning VR system in the region, many others do not report any deaths from a central VR system. Developing and strengthening VR systems in these nations is crucial to improve data availability and quality to inform more accurate demographic and health estimates.^29^ Although establishing fully complete death registration is the long-term goal, a short-term focus on establishing sample registration systems that are representative at the national level is one way to improve data availability and quality with less investment of money and time, while continuing to scale up national systems.^30^ Development of such systems is currently underway in Sierra Leone,^31^ Mozambique,^32^ and Zambia.^33^ Systems such as these will be able to provide more timely data, which is imperative for tracking emerging health events such as the COVID-19 pandemic if they are able to capture all deaths. Furthermore, releasing VR data as soon as possible, even in a preliminary state, is useful to inform trends in most recent years. Our new methodology allows incorporation of provisional data at large aggregation scales, such as yearly total deaths, which aids timeliness and relevancy of estimates.

Additionally, many countries and territories would benefit from collecting data from other sources, such as nationally representative surveys. For the 2020–23 period, 49 countries and territories had no available data on under-5 mortality, and 67 countries and territories had no available nationally representative data on mortality in those older than 15 years, predominantly in sub-Saharan Africa. However, funding of these collection efforts is in a concerning state, with the most prominent source of survey data in low-income and middle-income countries, the USAID Demographic and Health Surveys (DHS) Program, having had its operations suspended potentially permanently.^34^ These data are crucial to inform health estimates; for 33 countries and territories, DHS has been the source of the majority of their data since 2000, with many of these countries and territories having high mortality and poor health outcomes, such as the Central African Republic and South Sudan. Losing these surveys in the coming years is of major concern to the estimation of demographic indicators and several other health metrics.

### Long-term mortality trends

Many agencies and researchers have used mortality trends since 1950 to understand the global landscape of health trajectories.^35^, ^36^, ^37^, ^38^ One notable example is the UN Sustainable Development Goal (SDG) target 3.2 of an under-5 mortality rate of no more than 25 deaths per 1000 livebirths by 2030.^39^ Progress on this target has varied considerably between countries and territories, with notable declines over the study period, but 69 of 204 countries and territories (as of 2023) are not on track to reach this target based on their trajectory of under-5 mortality rate between 2010 and 2023 (appendix 2 table S2A). To accelerate progress in these locations, reducing global inequities in access to high-quality health care, particularly maternal, neonatal, and child health services; vaccines; safe water, sanitation, and hygiene; and other essential health programmes and policies will be crucial, with greater funding and attention paid to the populations most affected by under-5 mortality.^28^, ^39^ The substantial achievements in reducing under-5 mortality over the past several decades^40^ are being threatened by the suspension of USAID—the US government contributed 22·6% of all development assistance for health in 2023^41^—and cuts to other funding sources, such as UK foreign aid.^42^ To maintain progress or even prevent reversals in under-5 mortality in high-risk populations, it will be imperative to mitigate the effects of these funding cuts, while also expanding other funding streams such as those from non-governmental organisations.

Although not all countries are on track to meet the SDG targets for under-5 mortality rates, the emphasis placed on policies and funding to address neonatal and under-5 mortality around the world has led to much larger improvements in the under-5 mortality rate over the past 25 years than in older children (aged 5–14 years), adolescents (aged 15–19 years), and young adults (aged 20–39 years). In particular, we found that mortality rates in those aged 5–14 years for both males and females and those aged 15–29 years for females in sub-Saharan Africa were higher than previously believed in sub-Saharan Africa, primarily due to the additional data included from complete birth histories. These data indicate higher mortality in those aged 5–14 years, which increased estimates for these ages directly and increased estimates for those aged 15–29 years indirectly through modelled age correlations. We also identified trends of substantively increasing mortality since 2011 in those aged 20–39 years in parts of North and Latin America, particularly the USA, Canada, Mexico, and Brazil. Our findings on mortality in these age groups illustrate that the current narrative on global mortality patterns does not follow the best available findings, and that prevailing approaches and policy priorities for mortality reduction around the world must shift to better address the current reality.

High mortality rates in children and adolescents aged 5–14 years in sub-Saharan Africa are heavily influenced by the particularly high rates of respiratory infections and tuberculosis, other infectious diseases, and unintentional injuries, for which we estimated approximately double the mortality rate in 2021 in this study compared to GBD 2021.^28^, ^43^ Meanwhile, mortality rates in females aged 15–29 years in sub-Saharan Africa are affected most by higher than previously estimated rates of maternal mortality and, to a lesser extent, road injuries and meningitis.^28^, ^43^ By contrast, high mortality rates in the population aged 20–39 years in North and Latin America reflect high and persistent rates of so-called deaths of despair—a category of deaths due mainly to suicide, drug overdoses, and alcoholism driven by economic, social, and psychological factors.^44^ Policy makers in these locations should thus prioritise policies that improve access to care and address the social determinants of health for these age groups in particular.

The different age patterns in mortality identified in this study largely reflect the methodological improvements in as well as access to and use of more, different, and timelier data sources than was previously possible in demographic modelling. In particular, we have used data from complete birth histories for the first time to model adolescent and young adult mortality, while in adult age groups we have stopped using less reliable sibling history data and are now also using HDSS data for older ages. Our novel approach to modelling all-cause mortality—and the capacity of this approach to incorporate more timely all-age VR data—gives us better insight than ever before into historical and recent trends as well as the current state of mortality across ages, sexes, and locations.

### Mortality experiences during the COVID-19 pandemic

Our global analysis of age-standardised mortality rates and life expectancy during and after the COVID-19 pandemic reveals a nuanced view of health outcomes influenced by various factors. The post-pandemic period marked a substantial recovery in age-standardised mortality rates for 192 of 204 countries and territories, with substantial declines in mortality in countries such as Peru, Tunisia, and Namibia, and with complete recovery to 2019 levels in nearly two-thirds of countries and territories. However, the persistence of increased age-standardised mortality rates in some countries, alongside the incomplete recovery of life expectancy to pre-pandemic levels in more than a third of countries globally, highlights the uneven nature of pandemic recovery. Several factors could explain the disparate trajectories in mortality and life expectancy observed across countries and regions. Variations in health-care system resilience, public health responses, socioeconomic conditions, and the prevalence of comorbidities are likely to have played considerable roles in shaping outcomes.^45^, ^46^ The patterns of changes in life expectancy during the pandemic further highlight the differential impacts of COVID-19. The pronounced decline in life expectancy in Latin America and the Caribbean during the pandemic, followed by a significant rebound, could suggest that the initial severe impact of the pandemic was met with effective recovery efforts, or that having a severe pandemic experience led to a smaller vulnerable population in subsequent years (ie, high infection-acquired immunity); meanwhile, the modest rebound in life expectancy in some countries within the high-income super-region (including New Zealand and Japan) might reflect ongoing challenges in pandemic management and recovery strategies or differential virus transmission over time.^47^, ^48^ For several locations, most notably Palestine, decreases in life expectancy in the pandemic recovery period (2021–23) were largely due to the ongoing conflict, rather than a reflection of challenges in pandemic recovery.^28^ The variable patterns of changes in life expectancy across countries highlight the influence of pandemic severity, health-care system capacity, public health interventions, and vaccine rollout effectiveness at different stages of the pandemic. Inconsistent increases in life expectancy underscore the need for targeted health policy interventions and health system strengthening to address these persistent challenges. These findings have major implications for global health policy and planning. The uneven recovery across countries emphasises the necessity for continued international cooperation, investment in health-care infrastructure, and tailored public health strategies to address the unique challenges posed by the pandemic and ensure equitable health outcomes. Moreover, the experiences of nations that have shown substantial recovery or resilience can offer valuable lessons in pandemic preparedness and response for future global health crises. These findings call for a nuanced, multifaceted approach to health policy and international cooperation to address the ongoing challenges of pandemic recovery and strengthen global health systems against future threats.

### “Death” of model life table methods

The estimates presented in this Article are based on a new method that directly models age-specific mortality rates from all available data rather than relying on model life tables, which—despite being invaluable tools for estimating age patterns of mortality in populations where empirical data might be scarce—are associated with considerable challenges that affect their accuracy and applicability. The primary issue stems from the assumption that mortality patterns and the distribution of deaths by age in the models are directly applicable to the target populations. This assumption can lead to notable inaccuracies, as it fails to account for unique health challenges, demographic shifts, and the impact of specific interventions in different locales.^25^ The reliance on historical mortality data from a limited set of populations, notably from middle-income and high-income countries, risks introducing a bias that might not fully represent the mortality patterns of diverse global populations and might fail to adequately account for more recent developments, including the progress against infectious diseases, the rise of non-communicable diseases, and the impacts of global health initiatives.^16^ For example, there is evidence that mortality in older age groups in sub-Saharan Africa might be lower than estimates based on model life tables due to differing population-level characteristics compared to locations with mortality data used in model life tables.^19^, ^20^ Misestimating mortality rates can lead to misallocation of resources, inadequately designed health interventions, and a misunderstanding of the health needs of a population. This is especially crucial in regions undergoing rapid changes in health profiles due to factors such as disease outbreaks, considerable improvements in health care, or socioeconomic development.

Another limitation of model life table systems used in GBD 2021 and by UNPD is their reliance on input parameters, such as child and adult mortality, which are estimated via separate models. Additionally, estimates from these model life table systems account for excess mortality from the HIV/AIDS epidemic and the COVID-19 pandemic via post-hoc incorporation of separately modelled estimates. These methods can be described as multistage modelling approaches that combine disconnected models. Multistage modelling methods have been shown to produce less accurate inference and predictions compared to unified frameworks that simultaneously estimate all outcomes in a single model.^49^ By using a single model to estimate all age-specific mortality rates that includes covariates to account for excess mortality due to the HIV/AIDS epidemic and the COVID-19 pandemic, the method we developed for GBD 2023 is a marked improvement over previous methods. Furthermore, using separate estimates of excess mortality due to the COVID-19 pandemic can lead to further unreliable estimates. As we move further into the pandemic recovery period, using counterfactual mortality estimates as a basis to calculate excess mortality becomes more dangerous, since counterfactual estimates based on the assumption that the pandemic had not occurred are extrapolations of trends before 2019, which might not hold in more recent years. Our model avoids this limitation by directly incorporating information on COVID-19 mortality via covariates.

In response to these challenges, we developed a new method to directly model age-specific mortality rates from all available data without reliance on model life table systems. Our method allows the use of more data than previously used in GBD 2021—namely, data from children and adolescents aged 5–14 years from complete birth histories. Furthermore, age patterns of mortality estimated by OneMod in data-sparse locations are driven by modelled correlations among age groups that borrow strength across space, time, and covariates using all available data. This contrasts with model life table systems that impose age patterns based on historical mortality data from a limited set of populations. Finally, our method is based on published statistical methodology that draws on statistical theory for increased reliability of estimates. This improvement is essential for enhancing the utility of mortality estimates in guiding public health research, policy making, and resource allocation more effectively. Since modelled estimates are the primary (or only) source of information on mortality in locations where data are scarce, improved reliability is paramount for local mortality monitoring. Although our estimates in these locations have substantial uncertainty, providing the best available estimates along with uncertainty intervals based on statistical theory allows decision makers to act on the basis of as much information as possible.

We used simulated data to verify the efficacy of OneMod as a modelling tool, and real data and past model results to validate OneMod results for understanding the pattern of mortality. For simulated data, we used mortality patterns for cardiovascular disease and ischaemic heart disease as true rates of interest and created noisy and down-sampled data. We compared the performance of OneMod to a state-of-the-art multistage ensemble tool (CODEm) for both in-sample and out-of-sample performance (*vs* true rates) and found that OneMod was able to consistently improve in both metrics. That is, OneMod had greater modelling power than previous tools, and was also less prone to over-fitting. Due to the complexity of previous modelling techniques in demographics analysis, it was not possible to do a head-to-head performance comparison on simulated data; instead, we validated results by comparing OneMod estimates to those produced in previous years by our highly specialised modelling pipeline. We found that in locations with many years of complete vital registration data, as well as for child age groups in locations with many reliable complete birth history surveys providing datapoints across many overlapping years, OneMod results and past results corresponded closely, but in locations with sparser data, particularly adult age groups in countries throughout sub-Saharan Africa, the results differed, and those of OneMod were consistent with new data types that we were able to incorporate into the model. We reviewed all results produced by OneMod and, where they differed from past results, found that it was easier to understand and explain the OneMod predictions from a scientific perspective. This includes OneMod estimates better following trends in data sources as well as, when no data are present, being able to better explain estimates based on factors such as covariate effects, regional trends, or age correlations.

### Comparisons between GBD 2023 estimates and other estimates

As detailed above, there have been many improvements in data processing and statistical modelling approaches in GBD 2023 compared to other demographic studies, particularly with respect to the novel tool introduced in this GBD cycle to estimate all-cause mortality by directly incorporating age-specific data in a single statistical model that does not require the use of model life table systems. These differences resulted in some important differences in estimates between sources.

We identified several key changes to age-sex-location-specific mortality estimates in GBD 2023 compared to GBD 2021.^12^ Most notably, our latest mortality rate estimates for both males and females aged 5–14 years and females aged 15–29 years in sub-Saharan Africa were substantially higher than previously estimated (87·3% and 61·2% higher on average across countries and territories, respectively, for GBD 2023 compared to GBD 2021 over the 1950–2021 time period). Conversely, mortality rates in older age groups (aged ≥50 years) in sub-Saharan Africa were 13·2% lower on average across countries and territories for GBD 2023 compared to GBD 2021 over the 1950–2021 time period. However, estimates for locations with high-quality VR data were nearly identical between GBD 2023 and GBD 2021.

The latest UNICEF child mortality report,^22^ published in 2025, estimated a global under-5 mortality rate of 36·7 (90% UI 34·7–41·1) deaths per 1000 livebirths in 2023, compared to our estimate of 36·3 (95% UI 35·7–36·9) deaths per 1000 livebirths in 2023 (appendix 2 table 2A). At the national level, our 2023 under-5 mortality rate estimates had a mean relative difference 8·6% higher than those of UNICEF, ranging from 73·8% lower to 188·2% higher across countries and territories. The UNICEF report likewise estimated 803 000 (90% UI 747 800–895 000) global deaths among children aged 5–14 years in 2023 (a 51·3% decline from 1990), compared to our higher estimate of 872 000 (95% UI 845 000–902 000) deaths, a 49·9% decline from 1990 (appendix 2 table S7). Differences in national-level mortality rates for children and adolescents aged 5–14 years in 2023 between GBD 2023 and UNICEF varied by country, with our estimates an average of 17·6% higher than UNICEF's, ranging from 48·8% lower to 300·9% higher across countries and territories (appendix 2 table S7). These differences were primarily driven by differences in data inclusion and processing.

The UNPD's latest World Population Prospects revision^23^ also estimated global life expectancy of 73·2 years in 2023, a 26·8-year increase from their estimate of 46·4 years in 1950. For comparison, we estimated a global life expectancy of 73·8 (95% UI 73·6–74·1) years in 2023, a 24·4-year increase from our estimate of 49·5 (95% UI 49·1–49·8) years in 1950. At the super-regional level, the largest discrepancy between these two sources was found in sub-Saharan Africa, where life expectancy in 2023 was 62·2 years according to UNPD and 64·2 years according to GBD 2023. National-level differences between the two sources varied in 2023, from 8·0 years lower to 10·1 years higher (mean difference 0·1 years). Over the entire 1950–2023 study period, our life expectancy estimates had a mean difference of being 1·5 years higher than those from UNPD, ranging from 4·3 years lower to 13·1 years higher at the national level. The largest differences were observed in location-years with scarce high-quality VR data or large fatal discontinuities with high uncertainty in magnitude.

For model validation and further comparison to UNPD World Population Prospects estimates, we compared age-specific and sex-specific death counts from our study to those from all location-years of VR data deemed to have complete death registration. We similarly compared these VR data to UNPD estimates.^23^ Our estimates had a mean absolute error (MAE) of 46·8 and root mean squared error (RMSE) of 171, indicating good fit to the data. These were lower (indicating better fit) than the MAE of 262 and RMSE of 904 for UNPD estimates.

### Limitations and future directions

This study has several key limitations. First, as with any modelling study of this scale, the estimates are limited by the availability and scope of data sources, which varies considerably by location. The precision of estimates relies on the accuracy of the data used in the model. As discussed above, the absence of high-quality vital statistics and civil registration systems in many low-income and middle-income countries results in large-scale uncertainty in our estimates for these locations (reflected in wider 95% UIs). Furthermore, delays in reporting limit the availability of recent VR data, which specifically affects the latest years of estimates. Of 118 countries and territories with VR data in 2021, 105 had VR data for 2022 and only 81 had VR data for 2023 at the time of analysis. Prompt reporting of VR data is necessary for tracking emerging health events and responding to changes in demographic and health trends, even if these data are in a provisional or aggregated state, since our latest mortality model can use these preliminary data. Future development of reliable data sources is crucial because estimates improve as the quality of underlying data improves. Subsequent GBD cycles will provide revised estimates after additional data for recent years become available.

Second, sparse data availability restricted analyses of more granular subpopulations, including for most subnational locations as well as for different races and ethnicities within a single country or territory. The considerable heterogeneity in demographic patterns and health outcomes that can exist between certain subpopulations is obscured by the geographical scale at which our estimates are published, limiting the utility of our estimates to inform subpopulation-specific interventions. Expanding our work in this area will require more comprehensive and detailed data, such as by socioeconomic status, race, ethnicity, and smaller administrative levels across more countries and territories.^40^, ^50^, ^51^

Third, our death distribution methods and existing correction strategies, which are used to estimate completeness of death registration from vital statistics systems, might not accurately account for migration between certain countries and territories. This is especially true for countries with high levels of migration, including the United Arab Emirates, Qatar, and Saudi Arabia.^52^, ^53^ As population ageing, climate change, conflict, and other complex factors drive the increased need for migration in the coming years,^54^, ^55^, ^56^ improving our methods to more thoroughly account for migration will become increasingly important.

Fourth, we assumed a binomial distribution for our OneMod model. Although we modelled mortality rates, using a binomial likelihood was found to be more stable than other likelihoods tested such as the Poisson; Poisson is limited in extrapolating over low-data regimes and in locations that take extreme values of covariates. To account for mortality rates above 1, particularly for neonatal age groups, we used re-scaling to pre-process the data for binomial likelihood. Future work will explore additional likelihood parameterisations of OneMod to assess robustness.

Fifth, we used pre-processing and stage-wise analysis to disaggregate all-age and all-sexes-combined datapoints. Stage-wise analysis is typically more stable and robust but gives age-specific and sex-specific data more power to determine the age and sex patterns that are then applied. Future work will explore modelling the aggregation mechanism within OneMod, so that we can ascertain the impact of stagewise analysis compared to an all-at-once analysis that can use both specific and aggregated data.

Finally, we were unable to propagate uncertainty for all covariates used in the analyses or from our age-sex splitting method, due to computational resource limitations. 95% UIs reflect uncertainty from data sampling and model estimation, but not uncertainty from SDI and PAF covariates or from splitting data in aggregated age-sex groups into more granular mortality rates. This limitation is mitigated to some extent by our use of uncertainty calibration, which adjusts 95% UIs to guarantee the expected properties in Pearson residuals at a given level of location granularity, such as country or region (appendix 1 section 2.3.5.6). Future iterations of GBD will investigate incorporation of covariate uncertainty into OneMod, as well as adding additional uncertainty from age-sex splitting, to allow for all sources of uncertainty to be included in modelling.

### Conclusion

The novel methodology presented in this study allows us to produce timelier and more accurate estimates of all-cause mortality and life expectancy across ages, sexes, locations, and years than ever before, which will be crucial for understanding and responding to long-term and emerging health trends across global populations now and in the coming years. Using this approach, we identified several key differences in mortality trends compared to those previously estimated, including higher rates of adolescent mortality, higher rates of young adult mortality in females, and lower rates of mortality in older age groups in much of sub-Saharan Africa. These and other differences indicate that the existing narrative on mortality patterns does not perfectly align with the current reality of health outcomes for certain populations, and that prevailing policy priorities for mortality reduction around the world must shift if they are to best address this reality. In this first GBD report to investigate mortality in the several years following the height of the COVID-19 pandemic, we also found that most of the world has recovered to pre-pandemic levels of mortality and life expectancy, although with considerable differences in trends, both temporally and in magnitude, across locations. The findings from this study will help inform policy development, implementation, and evaluation to ensure that health-care systems, economies, and societies are prepared to address the world's greatest health needs.

#### GBD 2023 Demographics Collaborators

#### Affiliations

#### Contributors

Please see appendix 3 (pp 66–90) for more detailed information about individual author contributions to the research, divided into the following categories: managing the overall research enterprise; writing the first draft of the manuscript; primary responsibility for applying analytical methods to produce estimates; primary responsibility for seeking, cataloguing, extracting, or cleaning data; designing or coding figures and tables; providing data or critical feedback on data sources; developing methods or computational machinery; providing critical feedback on methods or results; drafting the manuscript or revising it critically for important intellectual content; and managing the estimation or publications process. The corresponding and senior authors had full access to all the data in the study and final responsibility for the decision to submit the manuscript for publication.

#### Data sharing

For detailed information on data sources and estimates, please visit the Global Health Data Exchange GBD 2023 website at http://ghdx.healthdata.org/gbd-2023.

For more on **Global Health Estimates** see https://www.who.int/data/global-health-estimates

For the **GBD 2023 Sources Tool** see https://ghdx.healthdata.org/gbd-2023/sources

For more on **INDEPTH** see https://indepth-network.org/

For the **statistical code** see http://ghdx.healthdata.org/gbd-2023/code

## Declaration of interests

D Adzrago reports support for the present manuscript from the National Institute on Minority Health and Health Disparities at the National Institutes of Health. D Adzrago's efforts are supported by the Division of Intramural Research at the National Institute on Minority Health and Health Disparities, National Institutes of Health (ZIA MD000015). Opinions and comments expressed in this Article belong to the author and do not necessarily reflect those of the US Government, Department of Health and Human Services, National Institutes of Health, and National Institute on Minority Health and Health Disparities; support for meetings and/or travel from the National Institute on Minority Health and Health Disparities at the National Institutes of Health, outside the submitted work. S Afzal reports support for the present manuscript from the Institute of Public Health, Lahore, Pakistan; grants or contracts by the Dean Institute of Public Health, Lahore, Pakistan; honoraria for experts, lectures, visiting speakers and educational seminars were provided by the Dean Institute of Public Health, Lahore, Pakistan; support for attending meetings and travel was provided by the Dean Institute of Public Health, Lahore, Pakistan; leadership or fiduciary roles in board, society, committee or advocacy groups, paid or unpaid as a Member of the Pakistan Higher Education Commission Research Committee, Member of the Pakistan Medical and Dental Commission Research and Journals Committee, Member of the Pakistan National Bioethics Committee, Member of the Pakistan Society of Internal Medicine, Member of the Pakistan Association of Medical Editors, Member of the Medical Microbiology and Infectious Diseases Society, Fellow of LEADS (Cohort15), 2010, Fellow of the Faculty of Public Health UK, Fellow of the College of Physicians and Surgeons Pakistan; receipt of equipment, materials, drugs, and services including computer software and equipment from Bergen University Norway for research writing; and other financial or non-financial support from the Dean Public Health Institute of Public Health, Lahore, Pakistan; outside the submitted work. C Agostinis Sobrinho reports grants or contracts from Fundação para a Ciência e a Tecnologia (FCT)- (CEECINST/00093/2021/CP2815/CT0001); outside the submitted work. R Ancuceanu reports royalties or licenses from AbbVie and Merck Romania; payment or honoraria for lectures, presentations, speakers bureaus, manuscript writing, or educational events from AbbVie, Laropharm, Reckitt, and Merck Romania; and support for attending meetings and/or travel from Merck Romania and Reckitt; outside the submitted work. J Arnlov reports payment or honoraria for lecture fees, speakers bureaus, manuscript writing, or educational events from AstraZeneca, Boehringer Ingelheim, and Novartis; and participation on a Data Safety Monitoring Board or Advisory Board with AstraZeneca, Boehringer Ingelheim, and Astella; outside the submitted work. M S Aslam reports grants or contracts from Xiamen University Malaysia Research Fund (XMUMRF; grant number XMUMRF/2025-C15/ITCM/0006), Project title: Therapeutic and Toxicity Evaluation of Selected Medicinal Herbs for NAFLD: Exploring the Inter-Organelle Contact Sites Modulation Theory Role: Co-Investigator Dates: Jan 2025–Dec 2027 (ongoing) Internal XMUMRF research grant administered by Xiamen University Malaysia; funds disbursed to institutional research account only; no salary, honoraria, or personal payments to author, and grant number XMUMRF/2023-C11/ISEM/0041, project title: Children's Rights Education in the Early Years of Divorce: An Exploration of Adolescents’ Perspectives Role: Co-Investigator Dates: Jan 2023 – Dec 2025 (ongoing) Internal XMUMRF research grant administered by Xiamen University Malaysia; funds disbursed to institutional research account only; no salary, honoraria, or personal payments to author; outside the submitted work. R Bai reports support for the present manuscript from the Fundamental Research Funds for the Central Universities (number 3092301101). O C Baltatu reports support for the present manuscript from Alfaisal University, Anima Institute (AI) Research Professor Fellowship, National Council for Scientific and Technological Development Fellowship (CNPq, 304224/2022-7); Leadership or fiduciary roles in board, society, committee, or advocacy groups, paid or unpaid as Managing Partner, VividiWise Analytics and as a Biotech Advisory Board Member, São José dos Campos Technology Park – CITE; outside the submitted work. S Barteit reports grants or contracts from the Carl-Zeiss Foundation research grant and the German research foundation (DFG) research grant; stock or stock options from the CHEERS company; outside the submitted work. A Beloukas reports grants or contracts with Gilead (research grant and sponsorship to the University of West Attica) and GSK (research sponsorship to the University of West Attica); payment or honoraria for lectures, presentations, speakers bureaus, manuscript writing, or educational events from Gilead and GSK (paid to the University of West Attica); support for attending meetings and/or travel from Gilead and GSK (paid to the University of West Attica); receipt of equipment, materials, drugs, services from Cepheid, and provided FOC reagents for a research project; outside the submitted work. S Bhaskar reports grants or contracts from Japan Society for the Promotion of Science (JSPS), Japanese Ministry of Education, Culture, Sports, Science and Technology (MEXT), Grant-in-Aid for Scientific Research (KAKENHI) (Grant ID: 23KF0126), JSPS and the Australian Academy of Science, JSPS International Fellowship (Grant ID: P23712; leadership or fiduciary role in other board, society, committee, or advocacy group, paid or unpaid as District Chair, Diversity, Equity, Inclusion & Belonging of Rotary District 9675 (Sydney, Australia), as Chair, Founding Member and Manager of the Global Health & Migration Hub Community, Global Health Hub Germany (Berlin, Germany), as Editorial Board Member of *PLOS One*, *BMC Neurology*, *Frontiers in Neurology*, *Frontiers in Stroke*, *Frontiers in Public Health*, *Journal of Aging Research*, *Neurology International*, *Diagnostics*, & *BMC Medical Research Methodology*, as a member of the College of Reviewers, Canadian Institutes of Health Research (CIHR), Government of Canada, as the Director of Research of World Headache Society (Bengaluru, India), as Expert Adviser/Reviewer of Cariplo Foundation (Milan, Italy), as Visiting Director of National Cerebral and Cardiovascular Center, Department of Neurology, Division of Cerebrovascular Medicine and Neurology, Suita (Osaka, Japan), as Member, Scientific Review Committee of Cardiff University Biobank (Cardiff, UK), as Chair of Rotary Reconciliation Action Plan, and Healthcare and Medical Adviser at Japan Connect (Osaka, Japan); outside the submitted work. M Carvalho reports other financial or non-financial support from LAQV/REQUIMTE, University of Porto (Porto, Portugal), FCT/MCTES under the scope of the project UIDP/50006/2020 (DOI 10.54499/UIDP/50006/2020); outside the submitted work. N Conrad reports grants or contracts from Wellcome Trust Career Development Award (grant number 318034/Z/24/Z) (personal fellowship for research support paid to their institution) and the Research Foundation Flanders (grant number 12ZU922N; personal fellowship for research support paid to their institution); outside the submitted work. S Cortese reports grants or contracts from the NIHR, European Research Agency; payment or honoraria for lectures, presentations, speakers bureaus, manuscript writing, or educational events from with ACAMH, BAP, Medice; support for attending meetings and/or travel from ACAMH, BAP, Medice; leadership or fiduciary roles in board, society, committee, or advocacy groups, paid or unpaid with Stein Committee and Eunethydid; outside the submitted work. E C Dee reports support for the present manuscript from NIH/NCI and the Prostate Cancer Foundation, funding in part through the NIH/NCI Support Grant P30 CA008748, and funding in part through the Prostate Cancer Foundation Young Investigator Award. A K Demetriades reports leadership, non-fiduciary roles in board, society, committee, or advocacy groups, paid or unpaid with EANS (European Association of Neurosurgical Societies) as a Board member, AO SPINE as a Steering Committee Member of the Knowledge Forum Degenerative, and Board Member of the Global Neuro Foundation; outside the submitted work. X Ding reports grants or contracts from the American Heart Association 2-year predoctoral fellowship (DOI: 10.58275/AHA.25PRE1373497.pc.gr.227106; quarterly payments made to institution); outside the submitted work. A Faro reports support for the present manuscript from the Brazilian National Council for Scientific and Technological Development (CNPq, Brazil) CNPq-funded researcher (PQ). A Fomenkov reports support for the present manuscript from the Ministry of Science and Higher Education of the Russian Federation (themes number 122042600086-7 and number 122042700043-9). R Franklin reports support for attending meetings and/or travel from ACTM Annual Conference 2022–2024; leadership or fiduciary roles in board, society, committee, or advocacy groups, paid or unpaid as President of the Australasian College of Tropical Medicine, President of Kidsafe Australia, Board Member of Royal Life Saving Society Australia, and Board Member of Auschem Training; outside the submitted work. N Fullman reports grants or contracts from the Gates Foundation (since March 2024, for work focused on drivers of non-vaccination in select countries); and other financial or non-financial support from Gates Ventures (June 2020 to June 2025); outside the submitted work. N Ghith reports grants or contracts from salary during employment at the Technical University of Denmark between 2019–2022 covered by a grant from Novo Nordisk Foundation (NNF16OC0021856) paid to the Technical University of Denmark between 2019–2022; support for attending meetings and/or travel from Danish Data Science Institute at the Technical University of Denmark, travel grant in 2023; outside the submitted work. A Guha reports grants or contracts from the American Heart Association and the US Department of Defense; leadership or fiduciary roles in board, society, committee or advocacy groups, paid or unpaid on the Health Equity Task Force Zero Prostate Cancer; outside the submitted work. C Herteliu reports grants or contracts from a project “Analysis of the impact of Covid-19 on the main demographic indicators in Romania and the Republic of Moldova by using econometric modeling” code PN-IV-P8-8.3-ROMD-2023-0208 funded by the Romanian Ministry of Research, Innovation and Digitalization (MCID) through UEFISCDI. C Herteliu is partially supported by a grant from the European Commision Horizon 4PCAN (Personalised Cancer Primary Prevention Research through Citizen Participation and Digitally Enabled Social Innovation). C Herteliu is partially supported by the project “Societal and Economic Resilience within multi-hazards environment in Romania” funded by European Union – NextgenerationEU and Romanian Government, under National Recovery and Resilience Plan for Romania, contract number 760050/ 23.05.2023, cod PNRR-C9-I8-CF 267/ 29.11.2022, through the Romanian Ministry of Research, Innovation and Digitalization, within Component 9, Investment I8. C Herteliu is partially supported by the project “A better understanding of socio-economic systems using quantitative methods from Physics” funded by European Union – NextgenerationEU and Romanian Government, under National Recovery and Resilience Plan for Romania, contract number 760034/ 23.05.2023, cod PNRR-C9-I8-CF 255/ 29.11.2022, through the Romanian Ministry of Research, Innovation and Digitalization, within Component 9, Investment I8; outside the submitted work. A H Hoveidaei reports leadership or fiduciary roles in board, society, committee, or advocacy groups, paid or unpaid as Guest Editor of *Frontiers in Sports and Active living*, as Editorial Board Member of *International Orthopaedics*, *Bone Reports*, *BMC Research Notes*, *PLoS One*, *Frontiers in Rehabilitation Sciences* (unpaid); outside the submitted work. I Ilic reports grants or contracts from the Ministry of Science, Technological Development and Innovation of the Republic of Serbia (number 451-03-137/2025-03/200110); outside the submitted work. M Ilic reports grants or contracts from the Ministry of Science, Technological Development and Innovation of the Republic of Serbia (number 451-03-47/2023-01/200111); outside the submitted work. N A Ismail reports leadership or fiduciary roles in board, society, committee or advocacy groups, unpaid as the Bursar and Council Member, Malaysian Academy of Pharmacy and Committee Member of the Malaysian Pharmacists Society Education Chapter Committee; outside the submitted work. I Iyamu reports grants or contracts from the Canadian Institutes for Health Research (CIHR) Health Systems Impact Fellowship (funding reference number IF8-196153), the Michael Smith Health Research BC Trainee Award (award number - HSIF-2024-04465), the CIHR Canadian HIV Trials Network (CTN+) postdoctoral fellowship; consulting fees from Excellence Community Education Welfare Scheme; support for attending meetings and/or travel from Pacific Public Health Foundation; leadership or fiduciary roles in board, society, committee, or advocacy groups, paid or unpaid as Vice President - Public Health Association of British Columbia; participation on a DSMB or advisory board as Vice President - Public Health Association of British Columbia; outside the submitted work. T Joo reports support for the present manuscript from the National Research, Development and Innovation Office in Hungary (RRF-2.3.1-21-2022-00006), Data-Driven Health Division of National Laboratory for Health Security, Funding of participation in the research project. J Jozwiak reports payment or honoraria for lectures, presentations, speakers bureaus, manuscript writing, educational events or personal fees from Novartis, Adamed, Amgen, Novo Nordisk, and Boehringer Ingelheim; outside the submitted work. M K Kashyap reports grants or contracts from Indian Council of Medical Research (ICMR), New Delhi, India (Grant # NCD/Ad-hoc/112/2021-22); outside the submitted work. N J Kassebaum reports grants or contracts from the Gates Foundation (grant funding for health metrics research); outside the submitted work. J H Kempen reports support for the present manuscript from Sight for Souls and Mass Eye and Ear Global Surgery Program; leadership or fiduciary roles in board, society, committee or advocacy groups, paid or unpaid from Sight for Souls; stock or stock options in Betaliq and Tarsier; outside the submitted work. J Khubchandani reports grants or contracts from Merck Pharma and the National Science Foundation, outside the submitted work. M Kivimaki reports grants or contracts from the Wellcome Trust (221854/Z/20/Z), the UK Medical Research Council (MR/Y014154/1), the National Institute on Aging (National Institutes of Health), USA (R01AG056477, R01AG062553), the Research Council of Finland (350426; PI of research grants to university); outside the submitted work. A G Konstas reports grants or contracts from Thea Pharmaceuticals, Omni Vision, Vianex, Santen, Intermed, Bayer; consulting fees from Thea Pharmaceuticals and Santen; payment or honoraria for lectures, presentations, speakers bureaus, manuscript writing, or educational events from Thea Pharmaceuticals, Vianex, Intermed, and Esteve Pharmaceuticals; support for attending meetings and/or travel from Vianex, Thea Pharmaceuticals, Intermed, and Santen; outside the submitted work. K Krishan reports other financial or non-financial interests from the UGC Centre of Advanced Study, CAS II, awarded to the Department of Anthropology, Panjab University (Chandigarh, India), outside the submitted work. T Lallukka reports support for the present manuscript from the Research Council of Finland (grant number 330527) payments made to institution. M Leonardi reports grants or contracts from the Ministry of Health, European Commission (payments made to institution); support for attending meetings and/or travel from WFNR, EAN, Italian Society of Neurology; participation on a DSMB or advisory board as a Member of the Board of the Centre of Bioethics of Catholic University; outside the submitted work. M-C Li reports grants or contracts from the National Science and Technology Council, Taiwan (NSTC 113-2314-B-003-002) and the “Higher Education Sprout Project” of National Taiwan Normal University; leadership or fiduciary roles in board, society, committee or advocacy groups, paid or unpaid as Technical Editor of the *Journal of the American Heart Association*; outside the submitted work. W-Z Li reports support for the present manuscript from the National Natural Science Foundation of China (82303338) and the Grant of State Key Laboratory of Respiratory Disease (SKLRD-Z-202401). D Lindholm reports stock or stock options with AstraZeneca (during time of employment); other financial or non-financial interests as a former employee of AstraZeneca; outside the submitted work. J Liu reports support for the present manuscript and grants or contracts from the National Natural Science Foundation (72474005) and Beijing Natural Science Foundation (L222027). V Lohner reports support for the present manuscript from Marga and Walter Boll Foundation, Kerpen, Germany. E Lytvyak reports grants or contracts from University of Alberta (Principal Investigator), Advanz Pharma (Co-Principal Investigator), WCB Alberta (Co-Principal Investigator), CPSA (Co-Principal Investigator); payment or honoraria for lectures, presentations, speakers bureaus, manuscript writing or educational events from the Alberta Obesity Society; other financial or non-financial interests from the University of Alberta as an employee and from Alberta Health Services; outside the submitted work. P Maffia reports grant or contract funding from the British Heart Foundation, NextGenerationEU PNRR, Heart Research UK, Italian Ministry of University, BBSRC International Partnerships Funding, and Scottish Founding Council; leadership or fiduciary roles in board, society, committee, or advocacy groups, paid or unpaid as the Vice-President and Chair of the Engagement Committee for the British Pharmacological Society (BPS), Chair of the Immunopharmacology Committee and vice-Chair of the Basic and Translational Section for the International Union of Basic and Clinical Pharmacology (IUPHAR), and Chair of the Translational Research Medical Review Panel for Heart Research UK (HRUK). Additionally, P Maffia serves as a Nucleus Member of the European Society of Cardiology (ESC) Working Group on Atherosclerosis & Vascular Biology and Cell Biology of the Heart and serves on the Executive Committee of the British Atherosclerosis Society (BAS), a part of the Immunotherapy Committee of the International Union of Immunological Societies (IUIS), and is a member of the Translational Clinical Studies (TCS) Grant Panel for the Chief Scientist Office (CSO); outside the submitted work. H R Marateb reports grants or contracts from Universitat Politècnica de Catalunya · Barcelona Tech – UPC; outside the submitted work. R Maude reports support for the present manuscript from the Wellcome Trust (grant number 220211) as it provides core funding for Mahidol Oxford Tropical Medicine Research and contributes to R Maude's salary. R Maude is required by Wellcome to acknowledge this grant in all publications. S A Meo reports grants or contracts from the Ongoing Research Funding Program (ORF-2025-47), King Saud University (Riyadh, Saudi Arabia); outside the submitted work. L Monasta reports support for the present manuscript from the Italian Ministry of Health (Ricerca Corrente 34/2017), payments made to the Institute for Maternal and Child Health IRCCS Burlo Garofolo. R Moreira reports grants or contracts from the CNPq Research Productivity Scholarship as a research productivity fellow by CNPq (National Council for Scientific and Technological Development), scholarship registration number 316607/2021-5; outside the submitted work. J Mosser reports support for the present manuscript from the Gates Foundation; grant or contract funding from Gavi; and support for attending meetings and/or travel from the Gates Foundation. F Mughal reports support for the present manuscript from NIHR (the views expressed in this manuscript are those of the authors and not those of the NIHR, NHS, or Department for Health and Social Care). S Nomura reports support for the present manuscript from the Ministry of Education, Culture, Sports, Science and Technology of Japan (24H00663) Grant and the Precursory Research for Embryonic Science and Technology from the Japan Science and Technology Agency (JPMJPR22R8) Grant. B Oancea reports support for the present manuscript from the Ministry of Research, Innovation and Digitalization through the Core Program of the National Research, Development and Innovation Plan 2022–2027, project number PN 23-02-0101-contract number 7N/2023 PNRR/2022/C9/MCID/I8 project 760096. S Onie reports grants or contracts from Templeton World Charity Foundation; support for attending meetings and/or travel from Suicide Prevention Australia (Registration, Travel and Accommodation at National Suicide Prevention Conference 2025) and the International Association for Suicide Prevention (Registration for 2025 World Congress); leadership or fiduciary roles in board, society, committee, or advocacy groups, paid or unpaid as Founding President of the Indonesian Association for Suicide Prevention; stock or stock options in Wellspring Indonesia, a local mental health clinic; outside the submitted work. R Ornello reports grants or contracts from the Italian Ministry of Health as co-investigator for grant-funded research; consulting fees from AbbVie, Eli Lilly, Pfizer, and Teva; payment or honoraria from AbbVie, Bayer, Eli Lilly, Lundbeck, Novartis, Organon, Pfizer, and Teva; support for attending meetings and/or travel from Novartis and Teva; participation on a DSMB or advisory board from AbbVie, Eli Lilly, and Pfizer; outside the submitted work. A Ortiz reports grants or contracts from Sanofi (to institution: IIS-FJD UAM), grants to Universidad Autonoma de Madrid (UAM) as Director of the Catedra Astrazeneca-UAM of chronic kidney disease and electrolytes; consulting fees from Astellas, AstraZeneca, Bioporto, Boehringer Ingelheim, Fresenius Medical Care, GSK, Bayer, Sanofi-Genzyme, Lilly, Chiesi, Otsuka, Novo Nordisk, and Sysmex; payment or honoraria for lectures, presentations, speakers bureaus, manuscript writing, or educational events from Astellas, AstraZeneca, Bioporto, Boehringer Ingelheim, Fresenius Medical Care, GSK, Bayer, Sanofi-Genzyme, Sobi, Menarini, Lilly, Chiesi, Otsuka, Novo Nordisk, Sysmex, Vifor Fresenius Medical Care Renal Pharma, and Spafarma; support for attending meetings and/or travel from Astellas, AstraZeneca, Fresenius Medical Care, Boehringer Ingelheim, Bayer, Sanofi-Genzyme, Chiesi, Sobi, Bayer, and participation on a data safety monitoring board or advisory board with Astellas, AstraZeneca, Boehringer Ingelheim, Fresenius Medical Care, Bayer, Sanofi-Genzyme, Chiesi, Otsuka, Novo Nordisk, and Sysmex; and leadership or fiduciary roles in board, society, committee or advocacy groups, unpaid as Council ERA with SOMANE; outside the submitted work. R Palma-Alvarez reports payment or honoraria for lectures, presentations, speakers bureaus, manuscript writing or educational events from Casen Recordati, Angelini, Lundbeck, Rubió, Servier, Takeda, and Neuraxpharm; support for attending meetings and/or travel from Takeda, Advanz Pharma, Angelini, Italfarmaco, and Lundbeck; outside the submitted work. S Panda reports support for the present manuscript from Siksha ‘O’ Anusandhan (Deemed to be University); grants or contracts from file number 17-59/2023-24/CCRH/Tech./Coll./ICMR- Diabetes/960] as Co-Investigator, outside the submitted work. C I Panelo reports consulting fees from The Global Fund and the World Health Organization; participation on a DSMB or advisory board with University of the Philippines Manila Research Ethics Board: Member of Panel 2 (ongoing); leadership or fiduciary roles with the Culion Foundation as Elected Vice President and Board Member (2004 to present), Foundation for the Advancement of Clinical Epidemiology as Elected President (2018 to present); International Society for Pharmacoeconomics and Outcomes Research (ISPOR) Philippine Chapter as Elected member of the Board (2024–2026); outside the submitted work. R Passero reports leadership or fiduciary roles in board, society, committee, or advocacy groups, unpaid as Member of the EBMT Statistical Committee, European Society for Blood and Marrow Transplantation, Paris, France (no payment received) and as Past member 2020–2023 (biostatistician) of the IRB/IEC Comitato Etico AO SS. Antonio e Biagio Alessandria-ASL AL-VC, Italy (no payment received); participation on a DSMB or advisory board member of the data safety monitoring board dello studio “Consolidation with ADCT-402 (loncastuximab tesirine) after immunochemotherapy: a phase II study in BTKi- treated/ineligible Relapse/Refractory Mantle Cell Lymphoma (MCL) patients’ - FIL, Fondazione Italiana Linfomi, Alessandria, Italy (no payment received); outside the submitted work. A Peden reports support for the present manuscript from the Australian National Health and Medical Research Council (grant number: APP2009306). V C Pepito reports grants or contracts from Sanofi Consumer Healthcare grant to do a study on self-care in the Philippines and from the Zuellig Family Foundation grant to write manuscripts on health systems strengthening; outside the submitted work. M Piradov reports leadership or fiduciary roles in board, society, committee or advocacy groups, paid or unpaid as Editor-in-Chief of *Annals of Clinical and Experimental Neurology*; outside the submitted work. S Rege reports leadership or fiduciary roles in board, society, committee, or advocacy groups, paid or unpaid as the Operational Lead – International Society for Pharmacoeconomics and Outcomes Research (ISPOR) Medication Adherence and Persistence (MAP) Special Interest Group (SIG), Review Editor – Editorial Board of Pharmacoepidemiology section within *Frontiers in Pharmacology*, Academic Editor – *PLoS One* Editorial Board, and Editorial Board Member – *Pain Management*; outside the submitted work. M Rolfzen reports grants or contracts from the Society of Cardiovascular Anesthesiologists in-training grant; outside the submitted work. L Ronfani reports support for the present manuscript from the Italian Ministry of Health (Ricerca Corrente 34/2017, payments made to the Institute for Maternal and Child Health IRCCS Burlo Garofolo. P Sachdev reports grants or contracts from the National Health and Medical Research Council of Australia, APP1169489 (payment to institution) and National Institutes of Health, USA; grants 1RF1AG057531-01 and 2R01AG057531-02A1 (payment to institution); leadership or fiduciary roles in board, society, committee or advocacy groups, unpaid with the International Neuropsychiatric Association as Executive Board member, and World Psychiatric Association on the Planning Committee; payment or honoraria for lectures, presentations, speakers bureaus, manuscript writing, or educational events from Alkem Labs for a lecture as part of the Frontiers of Psychiatry 2023 seminar, Mumbai, India, June 2023; support for attending meetings and/or travel for attendance of four meetings; participation on a DSMB or advisory board with Biogen Australia Medical Advisory committee in 2020 and 2021 Roche Australia Medical Advisory Committee in 2022 and Eli Lilly, Expert Advisory Panel, 2025; outside the submitted work. Y L Samodra reports grants or contracts from Taipei Medical University (Taiwan), Type A Doctoral Scholarship, EPM NTU & NSTC, Taiwan, postdoctoral fellow contract; leadership or fiduciary roles in board, society, committee, or advocacy groups, paid or unpaid with Benang Merah Research Center, Indonesia (benangmerah.net); outside the submitted work. H Sarma reports support for the present manuscript from Bodoland University, Assam, India. A Schutte reports consulting fees from Medtronic, AstraZeneca, Sky Labs, and Omron; payment or honoraria for lectures, presentations, speakers bureaus, manuscript writing, or educational events from Medtronic, AstraZeneca, Servier, Sanofi, Abbott, Omron, and Aktiaa; support for attending meetings and/or travel from Servier and Medtronic; outside the submitted work. F Shahkarami reports grants or contracts and payment or honoraria from Tehran University of Medical Sciences, School of Medicine, Department of Internal Medicine; outside the submitted work. V Sharma reports grants or contracts from DFSS (MHA)‘s research project (DFSS28(1)2019/EMR/6) at Institute of Forensic Science & Criminology, Panjab University, Chandigarh, India; outside the submitted work. V Shivarov reports one patent from the Bulgarian patent office (#BG113116A); and other financial support from ICON plc (salary); outside the submitted work. L M L R da Silva reports grants or contracts from SPRINT - Sport Physical Activity and Health Research & Innovation Center, Polytechnic of Guarda, 6300-559 Guarda, Portugal and RISE-Health, Faculty of Health Sciences, University of Beira Interior, 6201-506 Covilhã, Portugal; outside the submitted work. J Singh reports consulting fees from ROMTech, Atheneum, ClearView Healthcare Partners, American College of Rheumatology, Yale, Hulio, Horizon Pharmaceuticals, DINORA, ANI/Exeltis, USA Inc., Frictionless Solutions, Schipher, Crealta/Horizon, Medisys, Fidia, PK Med, Two Labs Inc., Adept Field Solutions, Clinical Care options, Putnam Associates, Focus Forward, Navigant Consulting, Spherix, MedIQ, Jupiter Life Science, UBM LLC, Trio Health, Medscape, WebMD, and Practice Point Communications; and the National Institutes of Health; payment or honoraria for lectures, presentations, speakers bureaus, manuscript writing, or educational events from Simply Speaking; support for attending meetings and/or travel from Simply Speaking; leadership or fiduciary role in other board, society, committee, or advocacy group, paid or unpaid as a past steering committee member of the OMERACT, an international organisation that develops measures for clinical trials and receives arm's length funding from 12 pharmaceutical companies, and as a Chair of the Veterans Affairs Rheumatology Field Advisory Committee, and as editor and the Director of the UAB Cochrane Musculoskeletal Group Satellite Center on Network Meta-analysis; stock or stock options in Atai Life Sciences, Kintara Therapeutics, Intelligent Biosolutions, Acumen Pharmaceutical, TPT Global Tech, Vaxart Pharmaceuticals, Atyu Biopharma, Adaptimmune Therapeutics, GeoVax Labs, Pieris Pharmaceuticals, Enzolytics Inc., Seres Therapeutics, Tonix Pharmaceuticals Holding Corp., Aebona Pharmaceuticals, and Charlotte's Web Holdings, Inc., and previously owned stock options in Amarin, Viking, and Moderna Pharmaceuticals; outside the submitted work. I Soyiri reports leadership or fiduciary roles in board, society, committee or advocacy groups, unpaid as Trustee of the Citizens Advice Bureau for Hull & East Riding, UK; outside the submitted work. J D Stanaway reports support for the present manuscript from the Gates Foundation (grants paid to institution); and grants or contracts from Novo Nordisk Foundation (grants paid to institution), outside the submitted work. R Tabares-Seisdedos reports grants or contracts from Valencian Regional Government's Ministry of Education (PROMETEO/CIPROM/2022/58 (the funders were not involved in the design of the manuscript or decision to submit the manuscript for publication, nor will they be involved in any aspect of the study's conduct) and grants or contracts from the Spanish Ministry of Science, Innovation and Universities (PID2021-129099OB-I00; the funders were not involved in the design of the manuscript or decision to submit the manuscript for publication, nor will they be involved in any aspect of the study's conduct); outside the submitted work. T Tabuchi reports grants or contracts from Daiichi Sankyo Healthcare, Workout-Plus LLC, Johnson & Johnson, EMMA, and Data Seed; outside the submitted work. J H V Ticoalu reports leadership or fiduciary roles in board, society, committee, or advocacy groups, paid or unpaid as co-founder of Benang Merah Research Center, Indonesia (benangmerah.net); outside the submitted work. M Titova reports support for the present manuscript from the state assignment of the Ministry of Science and Higher Education of the Russian Federation (themes number 122042600086-7 and number 122042700043-9). S Tromans reports grants or contracts from part of the 2023/4 Adult Psychiatric Morbidity Survey team, collecting epidemiological data on community-based adults living in England. This is a contracted study from NHS Digital, via the Department of Health and Social Care. S Tromans reports contributions and data collection for the 2023/4 Adult Psychiatric Morbidity Survey report; payments made to University of Leicester; being lead on a study funded by the National Institute for Health and Care Research Clinical Research Network, on optimizing the survey design for people with learning disability and autism; leadership or fiduciary roles in board, society, committee, or advocacy groups, paid or unpaid as Academic Secretary for the Neurodevelopmental Psychiatry Special Interest Group and Psychiatry of Intellectual Disability Faculty at the Royal College of Psychiatrists; as Editorial Board Member for *Progress in Neurology and Psychiatry*, *Advances in Mental Health and Intellectual Disabilities*, *Advances in Autism*, *BMC Psychiatry*, and *BJPsych Open*, and as Editor of *Psychiatry of Intellectual Disability Across Cultures* (Oxford University Press); support for attending meetings and/or travel from the Royal College of Psychiatrists; outside the submitted work. G Tse reports leadership or fiduciary roles in board, society, committee, or advocacy groups, paid or unpaid with the International Society of Electrocardiology and the International Society for Holter and Noninvasive Electrocardiology; participation on a DSMB or advisory board as a member of the Core Steering Committee meeting for the International Cardiovascular and Respiratory Alliance and the International Society of Electrocardiology, International Society for Holter and Noninvasive Electrocardiology; outside the submitted work. E Upadhyay reports the following patents planned, issued or pending with The Office of the Controller General of Patents, Designs & Trade Marks (CGPDTM; https://iprsearch.ipindia.gov.in/PublicSearch/PublicationSearch/ApplicationStatus): “Eco-friendly bio-shoe polish from banana and turmeric” (Filed 202511021382), “Honey-based polyherbal syrup composition to treat air pollution-induced inflammation and preparation method thereof” (Filed 202511035171), “Process for preparing a caffeine free, antioxidant and nutrient rich beverage” (Filed 202511042794), “A system and method of reusable filters for anti-pollution mask” (Published 202011003559), “A system and method for electricity generation through crop stubble by using microbial fuel cells” (Published 202011008531), “A system for disposed personal protection equipment (PPE) into biofuel through pyrolysis and method” (Published 202111005659), “A novel herbal pharmaceutical aid for formulation of gel and method thereof” (Published 202111023333), “Herbal drug formulation for treating lung tissue degenerated by particulate matter exposure” (Published 202311035276), “A method to transform cow dung into the wall paint by using natural materials and composition thereof” (Filed 202311085452), “Biodegradable packaging composition and method of preparation thereof” (Filed 202511017848); leadership or fiduciary role in other board, society, committee, or advocacy group as an Executive Council Member for the Indian Meteorological Society, Jaipur Chapter (India) and a Member Secretary for the DSTPURSE Program; outside the submitted work. E Vounzoulaki reports grants or contracts from an NIHR DSE Award until July 2026; outside the submitted work. P Willeit reports consulting fees from Novartis Pharmaceuticals; outside the submitted work. J Wu reports grants or contracts from the National Heart, Lung, and Blood Institute (R38HL167238) and acknowledges previous funding from the American Society of Hematology Opportunities for the Next Generation of Research Scientists (HONORS) Award, outside the submitted work. Y Yasufuku reports grants or contracts from Shionogi & Co., Ltd; employment expenses are paid from the joint research fund provided by this pharmaceutical company to The University of Osaka; outside the submitted work. S Zadey reports leadership or fiduciary roles in board, society, committee or advocacy groups, paid or unpaid on the Board of the Association for Socially Applicable Research Fellow, the Lancet Citizens’ Commission on Reimagining India's Health System Chair, G4 Alliance Asia Working Group and as Fellow, Blood DESERT Coalition; payment or honoraria for lectures, presentations, speakers bureaus, manuscript writing, or educational events from Think Global Health and Hindu; participation on a DSMB or advisory board with Nivarana - Advisory Board; outside the submitted work. G Zamagni reports support for the present manuscript from the Italian Ministry of Health through the contribution given to the Institute for Maternal and Child Health IRCCS Burlo Garofolo, Trieste – Italy. L Zuhlke reports royalties or license from Up to Date; consulting fees from Le DucQ; and leadership or fiduciary roles in board, society, committee or advocacy groups, unpaid with Food Forward SA; outside the submitted work. All other authors declare no competing interests.

## References

[1] UN (July 2024). World Population Prospects 2024: summary of results. https://desapublications.un.org/publications/world-population-prospects-2024-summary-results.

[2] WHO (May 21, 2024). World health statistics 2024: monitoring health for the SDGs, sustainable development goals. https://www.who.int/publications/i/item/9789240094703.

[3] OECD (Nov 7, 2023). Health at a glance 2023: OECD indicators.

[4] eurostat (March 2025). Mortality and life expectancy statistics. https://ec.europa.eu/eurostat/statistics-explained/index.php?title=Mortality_and_life_expectancy_statistics.

[5] Murray CJL (2022). The Global Burden of Disease Study at 30 years. Nat Med.

[6] Wang H, Dwyer-Lindgren L, Lofgren KT (2012). Age-specific and sex-specific mortality in 187 countries, 1970–2010: a systematic analysis for the Global Burden of Disease Study 2010. Lancet.

[7] GBD 2013 Mortality and Causes of Death Collaborators (2015). Global, regional, and national age-sex specific all-cause and cause-specific mortality for 240 causes of death, 1990–2013: a systematic analysis for the Global Burden of Disease Study 2013. Lancet.

[8] GBD 2015 Mortality and Causes of Death Collaborators (2016). Global, regional, and national life expectancy, all-cause mortality, and cause-specific mortality for 249 causes of death, 1980–2015: a systematic analysis for the Global Burden of Disease Study 2015. Lancet.

[9] GBD 2016 Mortality Collaborators (2017). Global, regional, and national under-5 mortality, adult mortality, age-specific mortality, and life expectancy, 1970–2016: a systematic analysis for the Global Burden of Disease Study 2016. Lancet.

[10] GBD 2017 Mortality Collaborators (2018). Global, regional, and national age-sex-specific mortality and life expectancy, 1950–2017: a systematic analysis for the Global Burden of Disease Study 2017. Lancet.

[11] GBD 2019 Demographics Collaborators (2020). Global age-sex-specific fertility, mortality, healthy life expectancy (HALE), and population estimates in 204 countries and territories, 1950–2019: a comprehensive demographic analysis for the Global Burden of Disease Study 2019. Lancet.

[12] GBD 2021 Demographics Collaborators (2024). Global age-sex-specific mortality, life expectancy, and population estimates in 204 countries and territories and 811 subnational locations, 1950–2021, and the impact of the COVID-19 pandemic: a comprehensive demographic analysis for the Global Burden of Disease Study 2021. Lancet.

[13] Coale A, Demeny P (1966).

[14] Brass W, Brass W (1971). Biological aspects of demography.

[15] Murray C, Ferguson B, Lopez A, Guillot M, Salomon J, Ahmad O (2003). Modified logit life table system: principles, empirical validation, and application. Popul Stud.

[16] Wilmoth J, Zureick S, Canudas-Romo V, Inoue M, Sawyer C (2012). A flexible two-dimensional mortality model for use in indirect estimation. Popul Stud.

[17] Clark S (May 2014). A singular value decomposition-based factorization and parsimonious component model of demographic quantities correlated by age: predicting complete demographic age schedules with few parameters. https://csss.uw.edu/research/working-papers/singular-value-decomposition-based-factorization-and-parsimonious-component.

[18] UN (July 2024). World Population Prospects 2024: methodology of the United Nations population estimates and projections. https://population.un.org/wpp/assets/Files/WPP2024_Methodology-Report_Final.pdf.

[19] Bendavid E, Seligman B, Kubo J (2011). Comparative analysis of old-age mortality estimations in Africa. PLoS One.

[20] Ouedraogo S. Levels and trends of older adult mortality in Sub-Saharan Africa: comparison of sources and estimates. PhD thesis, Université Panthéon-Sorbonne-Paris I, 2022.

[21] IHME (June 4, 2024). Global Burden of Diseases, Injuries, and Risk Factors Study (GBD) Protocol. https://www.healthdata.org/sites/default/files/2024-06/GBD%20Protocol%20060424.pdf.

[22] UNICEF (March 24, 2025). Levels and trends in child mortality. United Nations Inter-Agency Group for Child Mortality Estimation (UN IGME), Report 2024. https://data.unicef.org/resources/levels-and-trends-in-child-mortality-2024/.

[23] UN Population Division (2024). World Population Prospects - Population Division - United Nations. https://population.un.org/wpp/.

[24] Masquelier B, Hug L, Sharrow D, the United Nations Inter-agency Group for Child Mortality Estimation (2018). Global, regional, and national mortality trends in older children and young adolescents (5–14 years) from 1990 to 2016: an analysis of empirical data. Lancet Glob Health.

[25] Preston SH, Heuveline P, Guillot M (2001).

[26] Zheng P, Barber R, Sorensen RJD, Murray CJL, Aravkin AY (2021). Trimmed constrained mixed effects models: formulations and algorithms. J Comput Graph Stat.

[27] Stevens GA, Alkema L, Black RE, The GATHER Working Group (2016). Guidelines for Accurate and Transparent Health Estimates Reporting: the GATHER statement. Lancet.

[28] GBD 2023 Causes of Death Collaborators (2025). Global burden of 292 causes of death in 204 countries and territories and 660 subnational locations, 1990–2023: a systematic analysis for the Global Burden of Disease Study 2023. Lancet.

[29] Phillips DE, AbouZahr C, Lopez AD (2015). Are well functioning civil registration and vital statistics systems associated with better health outcomes?. Lancet.

[30] AbouZahr C, Rampatige R, Lopez A, deSavigny D (2012). When civil registration is inadequate: interim methods for generating vital statistics. Pac Health Dialog.

[31] Carshon-Marsh R, Aimone A, Ansumana R (2022). Child, maternal, and adult mortality in Sierra Leone: nationally representative mortality survey 2018–20. Lancet Glob Health.

[32] Jiwani SS, Mavie VA, Williams E, Kante AM, Amouzou A (2023). Implementing the countrywide mortality surveillance in action in Mozambique: how much did it cost?. Am J Trop Med Hyg.

[33] Zambia National Public Health Institute (Oct 26, 2023). Mortality surveillance meeting on development of a costed plan for ICT use with linkages to CRV's and data warehouse meeting. https://w2.znphi.co.zm/2023/10/26/mortality-surveillance-meeting-on-development-of-a-costed-plan-for-ict-use-with-linkages-to-crvs-and-data-warehouse-meeting/.

[34] Mandavilli A (Feb 26, 2025). https://www.nytimes.com/2025/02/26/health/usaid-global-health-surveys.html.

[35] Ouellette N, Barbieri M, Wilmoth JR (2014). Period-based mortality change: turning points in trends since 1950. Popul Dev Rev.

[36] Case A, Deaton A (2017). Mortality and morbidity in the 21st century. Brookings Pap Econ Act.

[37] van Raalte AA (2021). What have we learned about mortality patterns over the past 25 years?. Popul Stud.

[38] Castro MC, Turra CM, Ponmattam J (2025). Trends and decomposition of changes in mortality in low- and middle-income countries, 1950–2019. Popul Dev Rev.

[39] UN Goal 3: Ensure healthy lives and promote well-being for all at all ages. United Nations Sustainable Development. https://www.un.org/sustainabledevelopment/health/.

[40] Burstein R, Henry NJ, Collison ML (2019). Mapping 123 million neonatal, infant and child deaths between 2000 and 2017. Nature.

[41] IHME (May 14, 2024). Financing global health 2023: the future of health financing in the post-pandemic era. https://www.healthdata.org/sites/default/files/2024-05/FGH_2023_Accessible_Digital_Version_with_Translations_2024.05.13.pdf.

[42] Lay K (March 2, 2025). ‘This will cost lives’: cuts to UK aid budget condemned as ‘betrayal’ by international development groups. The Observer. https://www.theguardian.com/global-development/2025/mar/02/this-will-cost-lives-cuts-to-uk-aid-budget-condemned-as-betrayal-by-international-development-groups.

[43] GBD 2021 Causes of Death Collaborators (2024). Global burden of 288 causes of death and life expectancy decomposition in 204 countries and territories and 811 subnational locations, 1990–2021: a systematic analysis for the Global Burden of Disease Study 2021. Lancet.

[44] Rehder K, Lusk J, Chen JI (2021). Deaths of despair: conceptual and clinical implications. Cogn Behav Pract.

[45] Filip R, Gheorghita Puscaselu R, Anchidin-Norocel L, Dimian M, Savage WK (2022). Global challenges to public health care systems during the COVID-19 pandemic: a review of pandemic measures and problems. J Pers Med.

[46] Mustafa S, Zhang Y, Zibwowa Z (2022). COVID-19 Preparedness and Response Plans from 106 countries: a review from a health systems resilience perspective. Health Policy Plan.

[47] LaRotta J, Escobar O, Ávila-Aguero ML (2023). COVID-19 in Latin America: a snapshot in time and the road ahead. Infect Dis Ther.

[48] Amul GG, Ang M, Kraybill D, Ong SE, Yoong J (2022). Responses to COVID-19 in Southeast Asia: diverse paths and ongoing challenges. Asian Econ Policy Rev.

[49] Schumacher AE, McCormick TH, Wakefield J (2022). A flexible Bayesian framework to estimate age- and cause-specific child mortality over time from sample registration data. Ann Appl Stat.

[50] Golding N, Burstein R, Longbottom J (2017). Mapping under-5 and neonatal mortality in Africa, 2000–15: a baseline analysis for the Sustainable Development Goals. Lancet.

[51] Ho JY, MacKellar L, Friedman R (2021). Covid-19 and the global demographic research agenda.

[52] Hill K, Queiroz B (2010). Adjusting the general growth balance method for migration. Rev Bras Estud Popul.

[53] Monti A, Drefahl S, Mussino E, Härkönen J (2020). Over-coverage in population registers leads to bias in demographic estimates. Popul Stud.

[54] GBD 2021 Forecasting Collaborators (2024). Burden of disease scenarios for 204 countries and territories, 2022–2050: a forecasting analysis for the Global Burden of Disease Study 2021. Lancet.

[55] GBD 2021 Fertility and Forecasting Collaborators (2024). Global fertility in 204 countries and territories, 1950–2021, with forecasts to 2100: a comprehensive demographic analysis for the Global Burden of Disease Study 2021. Lancet.

[56] Vollset SE, Goren E, Yuan C-W (2020). Fertility, mortality, migration, and population scenarios for 195 countries and territories from 2017 to 2100: a forecasting analysis for the Global Burden of Disease Study. Lancet.

