# Supplementary material for: Global age-sex-specific all-cause mortality and life expectancy estimates for 204 countries and territories and 660 subnational locations, 1950–2023: a demographic analysis for the Global Burden of Disease Study 2023
Source: Lancet. 2025 Oct 18;406(10513):1731–810. doi: 10.1016/S0140-6736(25)01330-3 (PMC12535839; doi:10.1016/S0140-6736(25)01330-3)
Supplement: Supplementary appendix 1 [file mmc1.pdf]

THE LANCET

Supplementary appendix 1

This appendix formed part of the original submission and has been peer reviewed. We post it as supplied by the authors.

Supplement to: GBD 2023 Demographics Collaborators. Global age-sex-specific all-cause mortality and life expectancy estimates for 204 countries and territories and 660 subnational locations, 1950–2023: a demographic analysis for the Global Burden of Disease Study 2023. *Lancet* 2025; published online Oct 12. https://doi.org/10.1016/S0140-6736(25)01330-3.

Appendix 1: methods appendix to "Global age-sex-specific all-cause mortality estimates for 204 countries and territories and 660 subnational locations, 1950–2023: a comprehensive demographic analysis for the Global Burden of Disease Study 2023"

This appendix provides further methodological detail for "Global age-sex-specific all-cause mortality estimates for 204 countries and territories and 660 subnational locations, 1950–2023: a comprehensive demographic analysis for the Global Burden of Disease Study 2023."

Preamble

This appendix provides further methodological detail for "Global age-sex-specific all-cause mortality estimates for 204 countries and territories and 660 subnational locations, 1950–2023: a comprehensive demographic analysis for the Global Burden of Disease Study 2023." This study complies with the Guidelines for Accurate and Transparent Health Estimates Reporting (GATHER) recommendations.[1] It includes detailed tables and information on data in an effort to maximise transparency in our estimation processes and provide a comprehensive description of analytical steps. We intend this appendix to be a living document, to be updated with each iteration of the Global Burden of Disease Study.

Portions of this appendix have been reproduced or adapted from appendix 1 of GBD 2021 Demographics Collaborators.[2] References are provided for reproduced or adapted sections.

Table of Contents

Preamble 2

List of appendix figures and tables 5

Appendix figures: 5

Appendix tables: 5

Section 1: GBD overview[2] 6

Section 1.1: Global Burden of Diseases, Injuries, and Risk Factors Study 2023 6

Section 1.2: Harmonising estimation of mortality, population, and fertility 6

Section 1.3: Geographical locations of the analysis 6

Section 1.4: Time period of the analysis 6

Section 1.5: Statement of GATHER compliance 6

Section 1.6: List of abbreviations 6

Section 1.7: GBD results overview 8

Section 1.8: Data input sources overview 8

Section 1.9: Funding sources 9

Section 2: Modelling all-cause mortality using OneMod 9

Section 2.1: All-cause mortality data sources[2] 9
Section 2.1.1: Overview 9
Section 2.1.2: VR/SRS/DSP/DSS 9
Section 2.1.3: CBH microdata 9
Section 2.1.4: CBH tabulated data 10
Section 2.1.5: SBH microdata 10
Section 2.1.6: Sibling survival histories 10
Section 2.1.7: Household recall of deaths 10
Section 2.1.8: Under-5 populations and livebirths 10

Section 2.2: All-cause mortality data processing 10
Section 2.2.1: VR prioritisation[2] 10
Section 2.2.2: CBH method[2] 10
Section 2.2.3: SBH method[2] 11
Section 2.2.4: Sibling survival method 12
Section 2.2.5: Initial identification and removal of outliers 15
Section 2.2.6: Sex and Age Splitting 15
Section 2.2.7: Addressing fatal discontinuities (shocks) 16
Section 2.2.8: VR under-enumeration for bias correction[2] 16
Section 2.2.9: Additional round of identifying and removing outliers 17

Section 2.3: Modelling all-cause mortality 17
Section 2.3.1: OneMod introduction 18
Section 2.3.2: Stagewise binomial modelling using offsets 18
Section 2.3.3: SpXMod: functional specification of complex GLMs 19
Section 2.3.4: Kronecker-factored multivariate kernel regression (Kreg) 20
Section 2.3.5: OneMod for mortality envelope estimation 23

Section 2.4: PyDisagg for age-sex splitting 28

Section 2.5: Life table and death number estimation 28

Section 2.5.1: Raking subnational estimates 28
Section 2.5.2: Adding fatal discontinuities[2] 28
Section 2.5.3: Life table calculation[2] 29
Section 2.5.4: Death number calculation 29

Section 2.6: Fatal discontinuities[2] 29
Section 2.6.1: Input data 30
Section 2.6.2: Location mapping 32
Section 2.6.3: Side splitting 32
Section 2.6.4: Prioritisation 32
Section 2.6.5: Age-sex splitting 32
Section 2.6.6: Assigning uncertainty and generating draws 33

Section 3: Fertility[2] 33

Section 3.1: Overview 33

Section 3.2: Modelling fertility 33
Section 3.2.1: Fertility data source types 33
Section 3.2.2: Fertility data identification and synthesis 33
Section 3.2.3: Age-specific fertility rate estimation for 15 to 49 years 34
Section 3.2.3: Data source adjustment 36
Section 3.2.4: Hyperparameter selection 36
Section 3.2.5: SBH methods 38
Section 3.2.6: Age-specific fertility rate estimation for 10- to 14-year-olds and 50- to 54-year-olds 38
Section 3.2.7: Fertility metrics 39

Section 3.3: Sex ratio at birth 39
Section 3.3.1: Overview 39
Section 3.3.2: Modelling approach 39

Section 4: Population[2] 39

Section 4.1: Overview 39

Section 4.2: Data sources and processing 40
Section 4.2.1: Census and registry lists 40
Section 4.2.2: Data extraction 40
Section 4.2.3: Data processing 40

Section 4.3: Model inputs 43
Section 4.3.1: Migration 43
Section 4.3.2: Mortality 44
Section 4.3.3: Fertility 44
Section 4.3.4: Sex ratio at birth 44

Section 4.4: Modelling strategy 44
Section 4.4.1: CCMPP 44
Section 4.4.2: Bayesian demographic balancing model 44
Section 4.4.3: Model description 45
Section 4.4.4: Population uncertainty 46
Section 4.4.5: GBD world population age standard 47

Section 5: Socio-demographic Index (SDI) analysis[2] 47

Section 5.1: Overview 47

Section 5.2: SDI calculation 47

Section 6: Additional methods[2] 48

Section 6.1: Correlation estimation 48
Section 6.2: Annualised rates of change 48
Section 6.3: Gross domestic product (GDP) and lag-distributed income 49
Section 6.4: Educational attainment covariate 49
Section 7: References 51
Section 8: Tables and figures 55

List of appendix figures and tables

See section 8 for all appendix figures and tables listed here.

Appendix figures:

Appendix Figure S1. Analytical flowchart for the estimation of all-cause mortality by age and sex for GBD 2023

Appendix Figure S2. Estimated completeness of death registration, 1950−2023

Appendix tables:

Appendix Table S1. GBD location hierarchy with levels

Appendix Table S2. GATHER checklist

Appendix Table S3. Number of all-cause mortality data sources by type and location, 1950–2023

Appendix Table S4. Number of all-cause mortality data sources by type and year, 1950–2023

Appendix Table S5. New data sources added for GBD 2023 mortality estimation

Section 1: GBD overview[2]

Section 1.1: Global Burden of Diseases, Injuries, and Risk Factors Study 2023

The Global Burden of Diseases, Injuries, and Risk Factors Study (GBD) is a collaborative research effort aimed at estimating worldwide population, fertility, morbidity, and mortality. The GBD collaborator network draws on the expertise of over 14 000 contributors from more than 160 countries and territories around the world. For this paper, we estimated mortality, population, and fertility by age, sex, and location from 1950 to 2023. This study supersedes previous GBD rounds, most recently GBD 2021 Demographics Collaborators[2] and GBD 2021 Fertility and Forecasting Collaborators.[3]

Section 1.2: Harmonising estimation of mortality, population, and fertility

The GBD demographic analytical framework is unique in that mortality and population estimates are generated using a closely interconnected modelling process that also incorporates estimation of fertility metrics. For GBD 2023, we first generated age-specific mortality and fertility estimates separately using the analytical approaches described in the sections that follow. In these steps, population estimates were used as an input for the calculation of age-specific mortality rates and fertility rates from vital and civil registration systems. The outputs from these two processes were used to generate age-sex-specific population estimates by location and year using a Bayesian hierarchical cohort component model for population projection. Once the new population numbers were estimated, they were returned as inputs to the same mortality and fertility estimation models that were run again in a second loop. To maintain consistency between mortality, population, and fertility in count space, we updated estimates of number of deaths and number of births using the rates estimated in the second loop and the final population estimates.

Section 1.3: Geographical locations of the analysis

We produced estimates for 204 countries and territories that were grouped into 21 regions and seven super-regions. GBD regions were designated based on two criteria: epidemiological similarity and geographic closeness of countries and territories. Super-regions were grouped based on cause of death patterns among regions. GBD 2023 includes subnational analyses for 20 countries (Brazil, China, Ethiopia, India, Indonesia, Iran, Italy, Japan, Kenya, Mexico, New Zealand, Nigeria, Norway, Pakistan, the Philippines, Poland, Russia, South Africa, Sweden, the UK, and the USA). All analyses are at the first or second level of administrative organisation within each country except for New Zealand, which is by Māori ethnicity or not. In the present publication, subnational results are presented in appendix 2 instead of the main text, given space constraints. Note that estimates are only published for a subset of these countries and territories, per agreements with country partners. A list of locations can be found in appendix table S1 (section 8).

Section 1.4: Time period of the analysis

We estimated numbers and rates of all-cause mortality, population, and fertility for the years 1950–2023.

Section 1.5: Statement of GATHER compliance

This study complies with the Guidelines for Accurate and Transparent Health Estimates Reporting (GATHER) recommendations.[1] We have documented the steps involved in our analytical procedures and detailed the data sources used. See appendix table S2 (section 8) for the GATHER checklist.

The GATHER recommendations can be found here: http://gather-statement.org/

Section 1.6: List of abbreviations

Abbreviation	Full phrase
$_{1}q_{0}$	probability of death from birth to age 1 year
$_{45}m_{15}$	mortality rate between age 15 years and 60 years
$_{45}q_{15}$	probability of death between age 15 years and 60 years
$_{4}q_{1}$	probability of death between age 1 year and 5 years
$_{5}m_{0}$	mortality rate between birth and age 5 years
$_{5}q_{0}$	probability of death between birth and age 5 years, also commonly known as under-5 mortality rate (U5MR)
ACLED	Armed Conflict Location and Event Database

ART	antiretroviral therapy
ASFR	age-specific fertility rate
BCCMP	Bayesian hierarchical cohort component model for population projection
BMA	Bayesian model averaging
BTL	International Classification of Diseases Basic Tabulation List
CBH	complete birth history
CCMPP	cohort component method for population projection
CD4	CD4+ T lymphocyte; an indicator of immune function
CEB	children ever born
CIBA	cohort incidence bias adjustment
CIBA-SPECTRUM	cohort incidence bias adjusted spectrum output
CoD	causes of death
CODEm	Cause of Death Ensemble model
COVID-19	coronavirus disease 2019
DALYs	disability-adjusted life-years
DDM	death distribution methods
DHS	Demographic and Health Surveys
DSP	Disease Surveillance Points System
DYB	Demographic Yearbook
EDU15+	Mean years of education for those aged 15 and older
EM-DAT	Centre for Research on the Epidemiology of Disasters' International Disaster Database
enn	early neonatal
EPP	Estimation and Projection Package
GATHER	Guidelines for Accurate and Transparent Health Estimates Reporting
GBD	Global Burden of Diseases, Injuries, and Risk Factors Study
GEMS	Global Enteric Multicenter Study
GGB	generalised growth balance method
GGBSEG	combined generalised growth balance and synthetic extinct generation method
GHDx	Global Health Data Exchange
GIDEON	Global Infectious Diseases and Epidemiology Network
GK	Gakidou-King
GLM	generalised linear model
GLMM	Gulf Labour Markets, Migration, and Population
GPR	Gaussian process regression
HDI	human development index
HFC	Human Fertility Collection
HIV	human immunodeficiency virus
HIV CDR	crude death rate due to HIV/AIDS
HMD	Human Mortality Database
IBC	Iraq Body Count
ICD	International Classification of Disease
IPUMS	Integrated Public Use Microdata Series
KReg	Kronecker-factored multivariate kernel regression
LDI	lag-distributed income per capita
lnn	late neonatal
LSMS	Living Standards and Measurement Surveys
MAD	median absolute deviation
MCCD	Medical Certification of Causes of Death
MCMC	Markov Chain Monte Carlo
MICS	Multiple Indicator Cluster Surveys
MPIDR	Max Planck Institute for Demographic Research
${}_nm_x$	mortality rate between age x and x+n

NCD	non-communicable disease
OneMod	orchestration tool that allows fitting stagewise statistical models with multiple components
OPRM	other pandemic-related mortality
PCVA	Physician Certified Verbal Autopsy
PES	post-enumeration survey
pnn	post neonatal
PRIO	Peace Research Institute Oslo
$_nq_x$	probability of death between age x and x+n
RHS	Reproductive Health Surveys
SBH	summary birth history
SCAD	Social Conflict Analysis Database
SDI	Socio-demographic Index
SEER	Surveillance, Epidemiology, and End Results Program
SEG	synthetic extinct generation
SpXMod	functional specification of complex GLM models
SRS	Sample Registration System
ST-GPR	spatiotemporal Gaussian process regression
TFO30	total fertility over age 30
TFR	total fertility rate
TFU25	total fertility under age 25
TMB	template model builder
U5MR	under-5 mortality rate
UCDP	Uppsala Conflict Data Program
UI	uncertainty interval
UN	United Nations
UNAIDS	Joint United Nations Programme on HIV/AIDS
UNHCR	United Nations High Commissioner for Refugees
UNICEF	United Nations International Children's Emergency Fund
UNPD	United Nations Population Division
UNSD	United Nations Statistics Division
USAID	United States Agency for International Development
VR	vital registration
WFS	World Fertility Survey
WHO	World Health Organization
YLDs	years lived with disability
YLLs	years of life lost

Section 1.7: GBD results overview

Supplementary results are available in appendix 2.

Visualisations for all results specific to demographic mortality measures for GBD 2023 are also available at https://vizhub.healthdata.org/mortality/ (note to editors and reviewers that this link will be made available for GBD 2023 prior to publication).

Section 1.8: Data input sources overview

GBD 2023 synthesises a large and growing number of data input sources including surveys, censuses, vital statistics, and other health-related data sources. The data from these sources are used to estimate population; fertility; all-cause mortality; causes of death, disease, and injury; and attributable risk for 204 countries and territories from 1990 to 2023 (and from 1950 to 2023 for population, fertility, and all-cause mortality). The input sources are accessible through an interactive citation tool, the GBD Sources Tool, on the GHDx: https://ghdx.healthdata.org/gbd-2023/sources (note to editors and reviewers that this link is currently password protected, at *GBDSources2023*. The link will be made public at publication).

This tool allows users to view and access GHDx records for input sources and export a CSV file that includes metadata, citations, and information about where the data were used in GBD. As required by GATHER, additional metadata for input sources are available through the citation tool as well. While we reviewed and extracted all input sources included in this tool, some sources were not included in our models as a result of data deduplication and outliering in the modelling process.

Section 1.9: Funding sources

Research reported in this publication was supported by the Gates Foundation (OPP1152504), the UK Departments of Health and Social Care, the Norwegian Institute of Public Health, and the New Zealand Ministry of Health. The content is solely the responsibility of the authors and does not necessarily represent the official views of the Gates Foundation. The funders of the study had no role in study design, data collection, data analysis, data interpretation, or writing of the report. Lead and senior authors as well as members of the core research team had full access to all data in the study and had final responsibility for the decision to submit for publication.

Section 2: Modelling all-cause mortality using OneMod

Section 2.1: All-cause mortality data sources[2]

Section 2.1.1: Overview

For the estimation of all-cause mortality, we used data from vital registration (VR) systems, Sample Registration Systems (SRS), Disease Surveillance Points Systems (DSP), household surveys (complete birth histories [CBH], summary birth histories [SBH], sibling survival modules [SIBS]), censuses (SBHs, CBHs on rare occasions), and Demographic Surveillance Sites (DSS). It was important that each data point was fully representative of the given location-year and age-sex group.

The GBD's standard set of exhaustive, non-overlapping age groups is early neonatal (0-6 days), late neonatal (7-27 days), 1-5 months, 6-11 months, 12-23 months, 2-4 years, 5-year age groups from 5 to 94, and 95+ years. Some country-years of data had less specific age groups, with wider age groups such as the early and late neonatal age groups combined or the under-1 or under-5 age groups combined. While we preferred data that aligned with the standard set of age groups over broader age groups, all data were included (after some processing). These steps are described in section 2.2.

Section 2.1.2: VR/SRS/DSP/DSS

We endeavored to incorporate all available data from VR systems as inputs in our all-cause mortality estimation process. This included death counts from multi-country VR sources such as the WHO Mortality Database, United Nations Demographic Yearbooks, Human Mortality Database (HMD), country statistics offices, and the Organisation for Economic Co-operation and Development (OECD) databases. We updated data from these sources in our systems as new releases occurred as well. Wherever possible, we also catalogued all data sources from each system for ongoing national VR systems (eg, the USA National Vital Statistics System [NVSS]).

When VR data were not available, we extracted data from the SRS from India and the DSP from China. Data from Demographic Surveillance Sites (DSS), such as Health and Demographic Surveillance System (HDSS) data, were also used.

Section 2.1.3: CBH microdata

Where VR data were unavailable or unreliable, CBHs were the preferred method for data collection on child mortality. CBHs are surveys conducted among mothers about all livebirths they have ever had by the time of the survey regardless of the child's survival status, including birthdate, survival status, and date of death, if deceased. Since CBH data include the age at which mothers gave birth to each child, we could calculate period and age-specific mortality rates in the years before the survey, assuming no survivor, migration-related, or recall bias. Many routine survey series, including the Demographic and Health Surveys (DHS), World Fertility Surveys (WFS), Multiple Indicator Cluster Surveys (MICS), and other national surveys, contain CBH modules. When available, we downloaded and used microdata that included individual level survey responses as opposed to the tabulated results which generally report values for three five-year periods (DHS) or one three-year period (MICS).

CBHs from surveys provide another important source on the distribution of mortality among under-5 age groups. These data reflect age patterns primarily from low-income countries and countries with higher levels of under-5 mortality. We only used datapoints from the 15 years leading up to the survey, so children who were born to mothers more than 15 years before the interview were not included in our analysis.

Section 2.1.4: CBH tabulated data

Some surveys release tabulated results before microdata, so we included these tabulated datapoints in our probability of death from birth to age 5 years (${}_5q_0$) database. However, once we were able to obtain microdata from the same survey, the tabulated report estimates were deprioritised in favor of the point estimates from the processed microdata.

Section 2.1.5: SBH microdata

SBH questionnaires are shorter and less detailed than CBHs and lack information on dates of birth and death of children. They simply ask mothers the number of livebirths they have ever had and how many of those children have died, as well as the age of the mother at the time of the interview. For this study, we collected all available SBH data with microdata or those that reported data on the proportion of a mother's children who died, by maternal age group. GBD has developed its own summary birth history method to generate under-5 mortality rate (U5MR) using the aforementioned SBH data (section 2.2.3).

Section 2.1.6: Sibling survival histories

We also acquired sibling survival history data from surveys where participants are asked about the status of their siblings (alive or dead). Surveys were deemed usable if they asked participants to provide a full account of all siblings (children born to the same mother), including each sibling's sex, year of birth, year of death and age at death (if applicable).

Section 2.1.7: Household recall of deaths

Household death recall modules can be found in censuses and surveys, where the information on the number of deaths that occurred in each household over a specific recall period is collected. Information on the number of usual residents in the household for the same recall period is collected as well. Such information, combined, can be used to adjust data deemed incomplete in the recall of household deaths to generate sex- and age-specific mortality rates.

Section 2.1.8: Under-5 populations and livebirths

Livebirths and population in the under-5 age groups were produced as part of the fertility and population estimation process, described in sections 3 and 4.

Section 2.2: All-cause mortality data processing

Section 2.2.1: VR prioritisation[2]

As we worked with a variety of VR sources, we developed a hierarchy of preferred sources. For a given location-year, our first preference was to use WHO data from GBD cause-specific mortality estimation, followed by unadjusted WHO data, country-specific civil registration/vital statistics data, the Human Mortality Database (HMD) data, and lastly, UN Demographic Yearbook data. We made exceptions in some cases, however. We assessed single-country VR data sources according to whether there were inconsistencies with other data sources or VR system documentation and based on the length of the time series provided by each source. We also added all ages and all sexes data from a variety of country-specific reports. This data was assigned the lowest priority level and only used when no other data was available for a given location in recent years.

Section 2.2.2: CBH method[2]

We used CBH microdata from unpooled surveys to calculate age-specific mortality rates for the 15 years prior to the survey. In some cases, we were able to calculate mortality rates for the under-5 age group up to 25 years prior.

We analysed each survey separately (eg, Kenya DHS 2014; Bangladesh MICS 2012–2013) instead of pooling all DHS or MICS CBH surveys from a single country (eg, Nigeria MICS surveys from 2007, 2011, and 2016–2017) so that we could better understand and address data quality issues in specific surveys.

To calculate age-specific mortality rates, from surveys while accounting for sampling design, we used the *demogsurv* package in R (https://github.com/ihmeuw-demographics/demogsurv). This uses standard statistical methodology to calculate sampling probability-weighted means stratified by time, age, and location. We specified yearly sex-specific estimates for age groups 0-6 days, 7 days - 1 month 1-5 months, 6-11 months, 12-23 months, 2-4 years, 5-9 years, and 10-14 years at the national level, as well as administrative level 1 for countries for which we produce subnational estimates. The package uses survival analysis methods and appropriately accounts for sampling design in both point estimates and standard errors. For standard errors, we used Taylor linearization. The method tabulates deaths by location-year-age-sex, and calculates survey-design weighted aggregations of person-years of exposure from the individual-level data. Mortality rates are calculated as the deaths divided by person-years. Full descriptions of the methods used are available on the linked GitHub page above.

The CBH mortality rates were calculated for the preferred set of age groups up to 20-24 using standard demographic methods that account for survey design. However, the 15-19 and 20-24 mortality rate values were not included in the final dataset due to expected incompleteness and bias. Our comparison of these age groups to other data types (VR and SIBS), by calculating average mortality rates between overlapping location-years, identified that CBH was slightly lower, on average (Table A).

Table A

Comparison	Age group	Avg percent difference
CBH vs. VR	15-19	CBH 5% lower
	20-24	CBH 9% lower
CBH vs. SIBS	15-19	CBH 2% lower
	20-24	CBH 8% lower

Section 2.2.2.1: Processing of tabular CBH

In some instances, microdata were not available for certain CBH surveys. We still added results from these surveys to our database if survey reports provided $_5q_0$ datapoint estimates based on CBH.

Section 2.2.3: SBH method[2]

We used a method developed by Rajaratnam and colleagues to estimate $_5q_0$ from SBH data from censuses and surveys.[4] We found this method to be more accurate and provide more timely estimates than previous methods. Here, we provide a concise summary of the methods. Full details on the calculations used for this method, can be found in this paper by Rajaratnam and colleagues

For most SBH data, we use the Combined bethod described below to estimate child mortality, which is a combination of the standard indirect method, maternal age cohort-derived method, time since first birth cohort-derived method, maternal age period-derived method, and time since first birth period-derived method. In cases where the only data available were tabular data of the number of livebirths ever born and number of children who died, aggregated by the mother's age, we applied the Maternal Age Cohort-Derived method.[4]

Here is a summary of the methods:

Standard Indirect Method

The standard indirect method, classified as a maternal age cohort-derived approach, estimates child mortality using regression equations fitted to simulated data. Developed from refinements of Brass' methods and contributions by Trussell and Feeney, it is widely applied using the United Nations' Manual X and software package QFIVE. The method uses two regression equations:

1. Reference Time Estimation: This equation computes the reference time for age group (i) based on parity ratios between maternal age groups (e.g., 15–19 y vs. 20–24 y) and standard model coefficients (Manual X, Table 48).
2. Child Mortality Estimation: The second equation predicts child mortality ((q(x))) using the ratio of children dead to children ever born ((CD/CEB)), parity ratios, and coefficients (Manual X, Table 47). Mortality estimates from maternal age groups are converted to under-five mortality ((5q0)) using Coale-Demeny model life tables, with the West model life table applied for generalizability.

Maternal Age Cohort-Derived Method (MAC)

The MAC method modifies the standard indirect method in three ways:

3. Direct estimation of (5q0) for all maternal age groups using logit transformations of (CD/CEB). Uncertainty intervals are derived through back-transformation.
4. Inclusion of country-level random effects to account for systematic variation across countries.
5. Use of empirical datasets rather than simulated data, eliminating reliance on model life tables.

The MAC method estimates reference time from complete birth history data, linking observed reference time to (CD/CEB), parity ratios, and fertility patterns. Predicted (5q0) values are derived using Bayesian posterior estimates of country-level random effects.

Time since First Birth Cohort-Derived Method (TFBC)

The TFBC method groups mothers by time since first birth (in 5-year increments) rather than maternal age. The time localization and (5q0) estimation equations are identical to the MAC method but stratify data by time since first birth. This method leverages datasets, such as MICS-3, that include time since first birth as a survey variable.

Maternal Age Period-Derived Method (MAP)

The MAP method addresses limitations in cohort-derived approaches by estimating period-specific (CD/CEB) ratios for each year prior to the survey. Responses from mothers are pooled by region, age group, and children ever born (CEB) bands, ensuring at least 500 observations per group. Using fine age groupings (e.g., 2-year bands), frequency distributions of births and deaths are applied to generate expected time allocations. These are regressed to predict (5q0) for single years of calendar time, up to 25 years prior to the survey, where the relationship between (CD/CEB) and (5q0) remains robust ((R^2 > 90%)).

Time since First Birth Period-Derived Method (TFBP)

The TFBP method mirrors the MAP approach but groups mothers by time since first birth rather than age. Frequency distributions for births and deaths based on time since first birth are applied to generate period-specific (CD/CEB) measures, which are then modeled to predict (5q0).

Combined Method

To generate a summary measure, estimates from the above methods are combined using Loess local regression, excluding results from the 15–19 year maternal age group due to upward bias in MAC estimates. An alpha value of 0.5 is applied to balance local fluctuations with smoothing.

Section 2.2.4: Sibling survival method

Section 2.2.4.1: GBD 2021 Method[2]

While VR systems are the preferred sources for adult mortality rates, in countries with few to no data from a working VR system, adult mortality rates can be derived from sibling survival modules, which provide much-needed information on the level and trends of adult mortality. We define a sibship as a group of offspring who have the same biological parents. Without necessary adjustment, adult mortality estimates from sibling survival modules are biased in four primary ways, resulting from the design and implementation of the method:[5]

6. Selection bias (under-representation of siblings from sibships with high mortality)
7. Zero reporter bias (sibships not represented in the survey due to sex composition and/or absence of any alive sibling of a sibship by the time of the interview)
8. Sparse data
9. Recall bias (under-reporting of deaths of siblings living in different places, having died in the distant past, or for any other reason that the respondent could not recall the death).

The technique we used to estimate adult mortality from sibling survival data and minimise bias was based on methods in Obermeyer and colleagues,[5] with the following changes to their methods:

10. Incorporated appropriate survival weights that accounted for the study design
11. Implemented a zero-survivor correction that accounted for mortality in families who were under-represented in the data because none of the siblings were alive to participate in the survey
12. Refined recall bias adjustment.

The correction to account for mortality in sibships with high mortality rates, proposed by Gakidou and King,[6] incorporates a sibship-level weight:

$$W_j = \frac{B_j}{S_j}$$

Where

j is a given sibship

B_j is the original size of sibship j

S_j is the number of siblings in sibship j who survive to the time of the survey

We used the Gakidou-King (GK) weight on observations being analysed at the sibship level to compute the weighted average of the proportions of siblings who had died, as reported by each respondent. The weight corrected for under-representation of high-mortality sibships.

Since we analyse data at the sibling level (with one observation for each sibling instead of for each sibship), we also used the following sibling-level weight:

$$W_i = \frac{1}{S_j}$$

for sibling i in sibship j. [7,8]

Previously, we applied the GK sibship-level weight to data that had been disaggregated to the sibling level, which led to inaccuracies in the estimates. Our sibling-level weight overcomes this challenge.

Eligibility criteria for different surveys also needed to be factored into the correction.[7] In GK's survivorship correction, $\frac{S_j}{B_j}$ refers to the probability that a sibling in sibship j survived and was eligible to be selected as a survey participant. For instance, only women between the ages of 15 and 49 are eligible to participate in DHSs, so for DHS data, S_j refers to the number of surviving women in sibship j who were aged 15-49 at the time of the survey. For this study, we made the S_j value consistent with the eligibility criteria for each survey.

As previously discussed, the sampled population in sibling histories did not include sibships that had no eligible siblings to participate in the surveys. As a result, these sibships are not present in the data. To account for this, we estimated the number of deaths among missing sibships by age and sibship size using a zero-survivor correction. We then added these estimated siblings to the observed sample before calculating age-specific mortality rates.

We applied the zero-survivor correction to sibships with one or two female siblings. It factored in the true number of sibships with one (or two) females as it related to the cumulative probability of dying before the time of the survey. The result of the correction was an estimate of the number of missing sibling deaths. For one-sibling sibships, we used the following formula:

$$K_{obs}^1 = K_{true}^1 * (1 - {}_a q_0^1)$$

$$K_{miss}^1 = K_{true}^1 * {}_a q_0^1$$

Where

K_{obs}^1 is the number of sibships with one sister that were observed in the sample

K_{true}^1 is the true number of sibships with one sister in the sample

K_{miss}^{1} is the number of sibships with one sister that were not represented in the sample due to no surviving sibling

${}_{a}q_{0}^{1}$ is the probability of death between birth and age a

$(1 - {}_{a}q_{0}^{1})$ is the probability that the sister survived to the time of the survey

We were able to determine that the number of sibships with only one sister that were not represented in the sampled population as a result of zero-survivor bias was:

$$K_{miss}^{1} = \frac{K_{obs}^{1}}{1 - {}_{a}q_{0}^{1}} \times {}_{a}q_{0}^{1}$$

We then multiplied the estimated number of missing sibships (K_{miss}^{1}) by the number of females in the sibship (one, in this instance) to estimate the number of females missing from each age group because they had died. We made each missing female one observation point, assigned birth and death dates based on the distribution in the sampled population, and added them to the dataset. We conducted the same process with families with two sisters using the formula below:

$$K_{obs}^{2} = K_{true}^{2} * (1 - {}_{a}q_{0}^{1} * {}_{a}q_{0}^{2})$$

$$K_{miss}^{2} = K_{true}^{2} * {}_{a}q_{0}^{1} * {}_{a}q_{0}^{2}$$

$$\therefore K_{miss}^{2} = \frac{K_{obs}^{2}}{1 - {}_{a}q_{0}^{1} * {}_{a}q_{0}^{2}} * {}_{a}q_{0}^{1} * {}_{a}q_{0}^{2}$$

Where

K_{obs}^{2} is the number of sibships with two sisters that were observed in the sampled population

K_{true}^{2} is the true number of sibships with two sisters in the population

K_{miss}^{2} is the number of sibships with two sisters that were not represented in the sampled population due to zero-survivor bias

${}_{a}q_{0}^{1}$ is the probability of death for the first sister between birth and age a

${}_{a}q_{0}^{2}$ is the probability of death for the second sister between birth and age a.

In two-sister sibships with only one 15- to 49-year-old sister, we used the following equation to account for the second sister not contributing to the probability of the sibship being observed in the sample:

$$K_{obs}^{2} = K_{true}^{2} * (1 - {}_{a}q_{0}^{1})$$

$$K_{miss}^{2} = K_{true}^{2} * {}_{a}q_{0}^{1} * {}_{a}q_{0}^{2} + K_{true}^{2} * {}_{a}q_{0}^{1} * (1 - {}_{a}q_{0}^{2})$$

$$\therefore K_{miss}^{2} = \frac{K_{obs}^{2}}{1 - {}_{a}q_{0}^{1}} * {}_{a}q_{0}^{1} * {}_{a}q_{0}^{2} * \frac{K_{obs}^{2}}{1 - {}_{a}q_{0}^{1}} * {}_{a}q_{0}^{1} * (1 - {}_{a}q_{0}^{2})$$

The original methods developed by GK and Obermeyer and colleagues used a logistic regression to account for bias in the time period before the survey.[5,6] For this study, however, we used an updated method to adjust for recall bias. Once we had estimated the probability of death between 15 and 60 years (${}_{45}q_{15}$) from each survey, we paired up estimates that overlapped

the same time period. Overlaps occurred in countries and territories where at least two surveys were conducted within 15 years of one another, since we included 15 years of recall. For each year with overlapping surveys, we computed the difference in the years of recall as the number of years between surveys. We quantified the relationship between years of recall and level of mortality for each sex of sibling using the following linear regression model:

$$\Delta({}_{45}q_{15})_{i,j} = \beta \times \Delta(survey\ date)_{i,j} + \xi$$

Where

$\Delta({}_{45}q_{15})_{i,j}$ is the difference in ${}_{45}q_{15}$

$\Delta(survey\ date)_{i,j}$ is the difference in survey date

j is the survey pair

i is the country

We also calculated 95% UIs.

We adjusted the ${}_{45}q_{15}$ estimates to account for recall bias using the period coefficient.

Section 2.2.4.2: GBD 2023 Updates
We calculated mx values from the SIBS microdata for 5-year age groups from 15 to 59. These values were prioritised over the 45q15 values generated in the description above and adjusted with the same period coefficients to address recall bias applied to each 5-year age group.

The SIBS estimates were calculated for the preferred set of adult age groups, but the 50-54 and 55-59 mx values were not included in the final dataset due to expected incompleteness and bias. Our comparison of these age groups to VR data, by calculating average mortality rates between overlapping location-years, identified that SIBS was slightly lower, on average (Table B).

Table B

Sex	Age group	Avg percent difference
Female	50-54	SIBS 5% lower
	55-59	SIBS 7% lower
Male	50-54	SIBS 2% lower
	55-59	SIBS 3% lower

Section 2.2.5: Initial identification and removal of outliers
We carefully reviewed the mortality data with standard GBD age groups to ensure quality. This included data with quality concerns, such as the Botwana Family Health Survey 2007-2008 where the survey documentation flags known incompleteness and non-representativeness.

Section 2.2.6: Sex and Age Splitting
All VR/SRS/DSP and survey (CBH, SIBS) data that were sex-specific and assigned to standard age groups informed an initial model to generate a sex-specific age pattern, as described in section 2.4.

Section 2.2.6.1: VR/SRS/DSP Data
Aggregate VR data that were not sex-specific were first split into sex-specific data using the sex pattern. Then, data with too wide of an age group was split into standard age groups. Both splitting steps were performed using a tool called PyDisagg,[9] which utilised the sex-specific age pattern to split the aggregate groups present in the dataset. The PyDisagg tool is described in section 2.4.

Section 2.2.6.2: Survey Data

There were a few types of survey data that needed to be split to be sex-specific or standardised to the preferred GBD age groups. This included neonatal and under-5 mortality rates from CBH sources, under-5 mortality rates from SBH sources, and a small number of SIBS sources with mx estimates for the 15-60 age group. Similarly to VR, the sex-specific age pattern informed the PyDisagg tool which performed the splitting.[9]

Section 2.2.7: Addressing fatal discontinuities (shocks)

We excluded location-years of survey data with substantial fatal discontinuities (shocks) and subtracted these deaths from VR because our objective was to identify the underlying mortality risk, which would be skewed by mortality data from large stochastic events. We reincorporated fatal discontinuities later in the estimation process (see section 2.6).

Section 2.2.8: VR under-enumeration for bias correction[2]

Child and adult VR completeness were estimated in the same way as previous iterations of GBD and used to inform VR data bias adjustments. For this study, we applied a suite of death distribution methods (DDMs) to make this assessment.[10] We used the three DDMs most common in demography: generalised growth balance (GGB), synthetic extinct generation (SEG), and a combined approach (GGBSEG),[11–15] which estimate completeness by comparing the age distribution of the population between two censuses with the age distribution of deaths between those same censuses. Starting in GBD 2019,[16] we also used two additional methods that utilise the GBD Bayesian Population Model, which only differ in whether the proportion of net migrants is also estimated in addition to completeness.

To test performance of the various methods, we simulated census and VR data while varying the level of VR completeness and factors that may bias completeness estimates, including total population size and age-structure, census completeness, net migration, and extent of age-misreporting in census and VR data. We used the Cohort Component Method of Population Projection (CCMPP) in the GBD Bayesian Population Model to estimate completeness of VR in between censuses.[10] This ages a population forward in time using CCMPP with the input mortality data and estimates of completeness in order to match the population at the second time point. For each of the five methods (new and traditional) we tested 105 age trims ranging from a starting age of 5 and an end age of 95. Table C shows the top ten age trims by method.

Table C

Rank	CCMP fixed migration		CCMP not fixed migration		GGB		GGBSEG		SEG	
	Start age trim	End age trim	Start age trim	End age trim	Start age trim	End age trim	Start age trim	End age trim	Start age trim	End age trim
1	55	95	25	95	5	70	5	70	45	85
2	65	95	25	90	15	65	10	70	40	85
3	55	90	35	95	10	65	5	75	50	95
4	65	90	30	95	20	65	15	65	45	95
5	45	95	20	95	5	85	20	65	55	85
6	60	95	15	95	5	60	15	70	50	85
7	50	95	35	90	10	85	10	60	40	90
8	45	90	45	95	10	70	10	65	60	85
9	50	90	30	90	15	85	15	60	40	95
10	60	90	20	90	10	60	10	75	35	85

From the simulation testing, we found that no single method performed significantly better than the other methods, but certain age trims for each method performed significantly better than other age trims. To account for this when producing

point estimates of completeness for each pair of censuses and VR data in between censuses, we excluded the highest and lowest point estimates across the five methods and only kept the three middle estimates. For each method, we only kept the top-performing age trim as ranked in our simulation testing. These three-point estimates of completeness for each census pair were then combined to form a full time series of completeness using a spatiotemporal regression model, as described below.

Our assessment used a two-stage model. We first used estimated child completeness to predict adult completeness, followed by a spatiotemporal regression model to incorporate estimates of adult completeness from the DDMs to generate a series of estimates from 1950 to 2023 of source- and country-specific adult death registration completeness. We assume that the completeness of a system changes gradually, so within the GBD standard locations, we used DDM estimates from neighbouring years to help assess the completeness of data from any given year in the study period. We also assumed that completeness was likely to be similar between countries in the same region, so we factored in completeness estimates from nearby countries as well.

We calculated child completeness as the ratio of observed child mortality to estimated child mortality in a given source, country, and year, using the estimation process described in previous iterations of GBD.[2] While data were only available from certain years for an individual country-source, we made a complete time series of child completeness estimates using a smoothing process. When a country-source had no more than three years of data, we assumed a constant level of child completeness that matched the mean of the available years' completeness. When a country-source had more than three years of data, we used a Loess regression to fill in gaps in the time series. In cases where we had to make out-of-sample estimates (country-sources without data from 1950 or 2023), we held child completeness constant before the first and after the last observation instead of using the Loess regression to predict completeness levels. When a country-source had a gap of more than five years between data, we filled in the gap with a linear interpolation of the two closest observations instead of using the Loess regression.

After estimating child completeness, we conducted our first-stage regression using simple linear regression of calculated adult completeness on child completeness, and predicted adult completeness using the obtained coefficients for VR, SRS, DSP, medical certification of causes of death (MCCD), and civil registration (CR) sources in log space, using the following formula:

$$\hat{\mu}(\log(Adult\ completeness)) = \hat{\beta}_0 + \hat{\beta}_1 * \log(Child\ completeness)$$

For other sources, we assumed completeness for the first stage due to lack of child mortality data. In our second-stage model, we took the residuals from the first-stage regression and applied spatiotemporal smoothing to the residuals, in order to borrow strength across the full time series and nearby locations. We then produced draw level estimates of completeness using the calculated prediction from stage 1 as the mean, and the standard deviation calculated from the smoothed residuals for stage 2 assuming a normal distribution. After setting logged completeness to 0 for estimates greater than 0, we produced final estimates and uncertainty from the anti-logged individual truncated draws.

For VR, SRS, and DSP sources, we considered country-years that had completeness estimates of 95% or higher to be 100% complete. We then scaled completeness values between 90% and 95% up to 100% as follows:

$$scaled\ completeness = 0.9 + 2\ (estimated\ completeness - 0.9).$$

We also assumed that if a country-source was complete across the full time series, its subnational locations were complete as well. Our exceptions to this assumption were Brazil and Iran, where we found that level of completeness differed across subnational locations, despite a complete and accurate time series at the national level.

Section 2.2.9: Additional round of identifying and removing outliers

We carefully reviewed the entire mortality dataset. This included checking and outliering data with quality concerns, and carefully reviewing all split data to ensure the sex- and age-pattern produced reasonable trends.

Section 2.3: Modelling all-cause mortality

To model all-cause mortality, we developed a new method to use all available data at the most granular level to estimate mortality rates using a single model. This is called the OneMod tool. In order to produce estimates of all-cause mortality, the strategy we developed is as follows: (1) compile all data at the finest age-sex granularity for GBD age groups and sexes; (2)

fit a OneMod model using these data to estimate mortality rates for all location-year-age-sexes in the GBD; (3) use these age-sex patterns of mortality to split data in more aggregated age-sex groups; (4) compile these data together with the original most-granular data in step 1; (5) fit a OneMod model using this full dataset to re-estimate mortality rates for all location-year-age-sexes in the GBD; (6) produce life tables and calculate death counts.

Section 2.3.1: OneMod introduction

We aimed to generate the most accurate all-cause mortality estimates for all GBD age groups, by sex, for all 925 locations in the GBD 2023 location hierarchy, for a 74-year time series from 1950 to 2023.

Here, we describe the OneMod tool and its application to the mortality envelope analysis. OneMod provides an implementation of a stagewise statistical model, with several components including parametric models, using covariates, intercepts, trends, and splines, as well as nonparametric fitting using kernel methods. The models are connected using an over-arching likelihood that links these stages, using priors and offsets to pass information from stage to stage. For mortality estimation, binomial likelihood (a particular example of general linear models)[17] is used throughout.

We describe the basics of stagewise modelling within binomial models, specify key parametric and nonparametric modelling components, and then provide a detailed explanation of how these components were used for the mortality envelope analysis.

Section 2.3.2: Stagewise binomial modelling using offsets

Given an observation p_i, associated weight N_i, and feature vector $a_i \in \mathbb{R}^n$, the contribution to the binomial negative log-likelihood is given by

$$\ell(y_i; p_i, N_i) = -N_i[y_i \log(p_i) - (1 - y_i)\log(1 - p_i)]. \quad (1)$$

The generalised modelling framework supposes a shared linear predictor between all the p_i, with

$$p_i(\theta) = \text{expit}(a_i^T \theta) = \frac{1}{1 + \exp(-a_i^T \theta)}.$$

An **offset** is a constant term within the linear predictor. This simple idea enables stagewise model fitting. For example, suppose we partition the parameter θ into

$$\theta = \begin{bmatrix} \theta_1 \\ \theta_2 \end{bmatrix}.$$

where θ_1 and θ_2 are two blocks of covariates. It may be desirable to first fit a model using θ_1. For example, θ_1 may include global information that can be borrowed across location, such as covariates, while θ_2 may include location-specific information, such as intercepts and trends.

The predictor for the first stage can now be written as

$$p_i(\theta_1) = \text{expit}(a_{i,1}^T \theta_1).$$

We can then fit

$$\hat{\theta}_1 = \arg\min_{\theta_1} \sum_i \ell(y_i; p_i(\theta_1), N_i)$$

Having obtained $\hat{\theta}_1$, we can lock the vector to the estimate, and for the second stage, consider

$$p_i(\theta_2 \mid \hat{\theta}_1) = \text{expit}(a_{i,1}^T \hat{\theta}_1 + a_{i,2}^T \theta_2) \quad (2)$$

where the term $a_{i,1}^T \hat{\theta}_1$ is the offset, and we now fit the remaining components of the model by solving

$$\hat{\theta}_2 = \arg\min_{\theta_2} \sum_i \ell(y_i; p_i(\theta_2 \mid \hat{\theta}_1), N_i)$$

The stagewise approach is a key technique for modelling outcomes informed by differential levels of sparsity, and offers great flexibility in combining results from different model types, such as parametric and nonparametric pieces as described below.

Section 2.3.3: SpXMod: functional specification of complex GLMs

The SpXMod tool allows specification of complex regularised generalised linear models (GLMs), allowing the user to provide dimensions of variation for intercepts, coefficients, and splines in addition to coefficient-valued splines.

Section 2.3.3.1: Group-specific coefficients and similarity priors

SpXMod provides a flexible modelling interface to allow group-specific effects of covariates, as well as similarity priors that can smooth these same effects across the dimension of interest.

For example, consider the dimension of **age** in the model, parametrised by age group $j = 1, \dots \dots, M$. Consider now that the effective parameters of interest, θ_j, may be age-specific, and that data $y_{i,j}$ and features $a_{i,j}$ are age-specific as well. Then we can specify a model that allows age-specific effects but imposes a relational prior:

$$\min_{\theta_1,\dots,\theta_M} \sum_{i,j} \ell(y_{i,j}; p_{i,j}, N_{i,j}) + \frac{1}{2}\theta^T C \theta.$$

where

$$p_{i,j} = \operatorname{expit}(a_{i,j}^T \theta_{i,j}).$$

and C is a matrix that enforces a similarity prior on the θ parameters. For example, suppose we assume that adjacent age groups should not vary significantly. This corresponds to a regularisation term of the form

$$\gamma \sum_j \|\theta_j - \theta_{j-1}\|^2$$

and so we could have

$$C = \gamma \begin{bmatrix} -I & I & 0 & \dots & 0 \\ 0 & -I & I & 0 & \dots \\ \ddots & \ddots & \ddots & \ddots & \\ 0 & 0 & \dots & -I & I \end{bmatrix} \tag{3}$$

While we used age in the construction, coeffients can vary across any relevant dimension or dimension combination. Key examples we come back to later on include location, where intercepts are location-specific, and location-age, where intercepts are specific to both location and age. The smoothing priors become ever more important in constraining the problems as there is more freedom for coefficients and intercepts to vary across multiple dimensions. To make this point more clear, one can view imposing a large penalty, for example, a large γ in (3), as a way to impose coefficients and intercepts that are constant across relevant dimensions such as age. Thus, the framework can interpolate between more common assumptions where all data are used to inform a single intercept and feature-specific multipliers and, on the other end, complete flexibility where the model does not borrow strength across groups in fitting group-specific intercepts and covariate multipliers.

Section 2.3.3.2: Spline specification

The coefficients θ_j may be understood as covariate multipliers for specific covariates. However, they may also correspond to splines, as detailed in this section. Taken together, the two sections show that SpXMod can specify correlated splines that vary across dimensions of interest.

For general background on splines and spline regression, see de Boor[18] and Friedman.[19]

For a spline with polynomial degree p and k knots placed at locations along the domain $x, t_0, \dots, t_k$, we need $p + k$ basis elements to construct the spline basis, denoted s_j^p. These basis elements can be constructed recursively.

The standard B-spline basis is generated recursively. By default, we assume $c_0^i = \emptyset, c_{i+k+1}^i = \emptyset$ and $s_0^i(t) = 0, s_{i+k+1}^i(t) = 0$, for all $i \geq 0$.

- $i = 0]$

$$c_j^0 = [t_{j-1}, t_j), s_j^0(t) = \delta_{c_j^0}(t), j = 1, \dots, k$$

- $i > 0$

$$c_j^i = c_{j-1}^{i-1} \cup c_j^{i-1}, \qquad s_j^i(t) = s_{j-1}^{i-1}(t) 1(t; c_{j-1}^{i-1}) + s_j^{i-1}(t) r(t; c_j^{i-1}), \qquad j = 1, \dots, i + k$$

where

$$l(t;c) = \begin{cases} (t - \inf c)/(\sup c - \inf c), & c \neq \emptyset \\ 0, & c = \emptyset \end{cases}$$

$$r(t;c) = \begin{cases} (t - \sup c)/(\inf c - \sup c), & c \neq \emptyset \\ 0, & c = \emptyset \end{cases}$$

as illustrated in figure A.

With knowledge of the degree and knots placement, we can construct a *design matrix X* based on an input vector x, where ethe j^{th} column of the design matrix is given by the expression

$$X_{\cdot,j} = \begin{bmatrix} s_j^p(t_0) \\ \vdots \\ s_j^p(t_k) \end{bmatrix} \tag{4}$$

Intercepts and trends can be seen as specific basis functions, and play a key role in the demographics analysis.

Figure A. Generation of B-spline basis elements (orders 0, 1, 2)

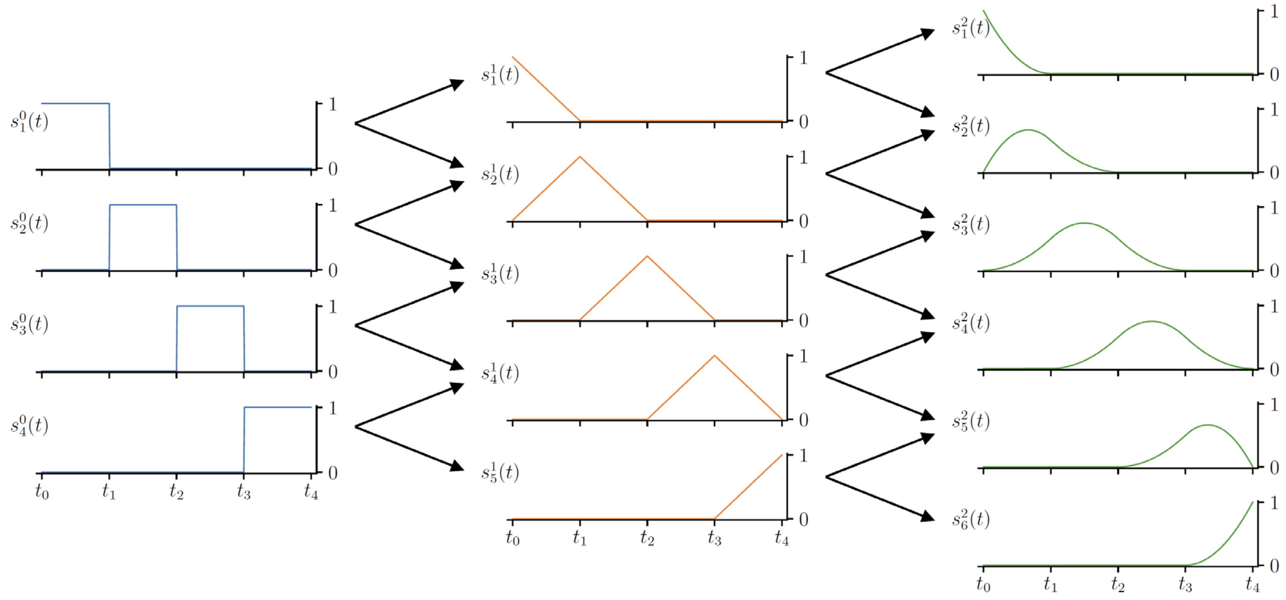

Section 2.3.4: Kronecker-factored multivariate kernel regression (Kreg)

Kernel regression[20] allows us to fit observations directly, balancing available observations with similarity across dimensions. We solve

$$\hat{f} = \arg\min_{f} \sum_{i=1}^{n} \ell(f_i \mid y_i, N_i) + \frac{\lambda}{2} f^T P f$$

where f is the entire vector of linear predictions of interest and $P = K^{-1}$, with K the kernel matrix that encodes the covariance structure through similarities across dimensions, such as age, time, and location.

This model corresponds to computing the MAP estimator of f given a Gaussian process prior

$$f \sim GP(0, \frac{1}{\lambda} K),$$

with fixed covariance K, and where all available information is communicated to the model through the kernel matrix K. In the following subsections, we discuss several useful kernels, and show how to efficiently model the action of the kernels across dimensions in higher-dimensional analysis.

Section 2.3.4.1: Specific kernels

KReg makes use of several standard kernels, and can incorporate user defined kernels. In the demographics analysis, we rely on different versions of the Matern kernel for age and time dimensions. For comparison, we also specify the Radial Basis Function (RBF)[21] kernel familiar to many readers; see eg, Rasmussen and Williams[22] for an accessible overview.

For all kernels, define

$$d = \|\mathbf{x} - \mathbf{y}\|_2 = \sqrt{\sum_{i=1}^{n} (x_i - y_i)^2}. \tag{5}$$

Radial basis function (RBF) kernel

$$k(\boldsymbol{x}, \boldsymbol{y}) = \exp\left(-\frac{d^2}{2\gamma^2}\right), \tag{6}$$

with d defined in (5). This kernel is widely used to model very smooth functions, and the underlying assumption that the unknown function is infinitely differentiable is not always desirable, and can induce oscillations when fitting it to data arising from more realistic processes.

Matern kernel

The Matern $3/2$ kernel function is defined as:

$$k(\boldsymbol{x}, \boldsymbol{y}) = \left(1 + \frac{\sqrt{3}d}{p}\right) \exp\left(-\frac{\sqrt{3}d}{p}\right), \tag{7}$$

with d defined in (5). In dimension 1, this equation models functions that are one-time differentiable and can help avoid overshoot/oscillations. The Matern kernel is frequently used in model-based geostatistics for this reason.[23]

Linear kernel

The linear kernel is parameterised by location and scale, and is defined by

$$k(x, y) = (x - a)^T (y - a)/b^2 \tag{8}$$

Section 2.3.4.2: Efficient kernel regression through Kronecker product

The vector f is inherently high-dimensional, as it includes all estimates of interest. For example, in the demographics case, the dimension would be

$$D = 2 \times 70 \times 20 \times 905 = 2\,534\,000,$$

where we consider 2 sexes, 70 years, 20 age groups, and 905 locations. Thus, a naïve application of Kernel regression with coupling across all dimensions would require computing and inverting a $2.5M \times 2.5M$ dense matrix at each iteration, which is not feasible. However, by imposing a product structure on our choice of covariance kernel and assuming fixed grids for each dimension, the resulting kernel matrix is available in Kronecker factored form. This greatly reduces memory and computational cost and makes the fully coupled dense problem computationally tractable.

Kronecker product

The Kronecker product is an operation on two matrices A and B, resulting in a larger block matrix. If A is an $m \times n$ matrix and B is a $p \times q$ matrix, their Kronecker product $A \otimes B$ is an $(mp) \times (nq)$ matrix defined as:

$$A \otimes B = \begin{bmatrix} a_{11}B & a_{12}B & \cdots & a_{1n}B \\ a_{21}B & a_{22}B & \cdots & a_{2n}B \\ \vdots & \vdots & \ddots & \vdots \\ a_{m1}B & a_{m2}B & \cdots & a_{mn}B \end{bmatrix}$$

Each element a_{ij} of matrix A is multiplied by the entire matrix B, resulting in a block matrix.

While we handle arbitrarily many blocks of variables, we detail the case of two blocks clarity. The Kronecker structure for kernels[24] arises from the following assumptions:

13. x can be partitioned into two groups of variables, $x = (a, b)$, so that $x_i = (a_i, b_i)$.
14. Assume observations lie on a uniform grid in terms of a_i, b_i, so that there are grids of values $(a_k)_{k=1}^{n_a}$, $(b_j)_{j=1}^{n_b}$, and $x_{kn_a+j} = (a_k, b_j)$ for $k = 1, \dots, n_a, j = 1, \dots, n_b$.
15. The covariance function $k(x_i, x_j)$ factorises into $k(x_i, x_j) = k\left((a_i, b_i), (a_j, b_j)\right) = k_a(a_i, a_j)k_b(b_i, b_j)$.

Using this factorisation, we can write:

$$K = K_a \otimes K_b, K_a = \left(k_a(a_i, a_j)\right)_{i,j=1}^{n_a}, K_b = \left(k_b(b_i, b_j)\right)_{i,j=1}^{n_b}$$

By properties of the Kronecker product,

$$P = P_a \otimes P_b, P_a = K_a^{-1}, P_b = K_b^{-1}$$

The optimisation problem then becomes:

$$f^* = \arg\min_f \left(\sum_{i=1}^{n} \ell_i(f_i) + \frac{\lambda}{2} f^\top (P_a \otimes P_b) f \right)$$

The Kronecker factorisation simplifies both direct and indirect approaches to the optimisation problem.

Decoupling

In practice, we often assume further decoupling across sexes and locations, which instead leads to 1810 separate estimation problems of size 1400 (ie, 1400 variables for each of the 1810 problems). This allows us to adapt hyperparameters to different locations as needed for a more desirable model fit. Indeed, conditioning on other features and the coupling induced by SpXMod, the additional variation that we want to fit using KReg doesn't appear to benefit much from coupling across locations.

Section 2.3.4.3: KReg with offsets

KReg can communicate with previous model stages (such as SpXMod) using offsets. For each reported observation y_i, we let g_i represent the predicted value from any previous steps.

The offset kernel regression model is given by

$$\hat{f} = \arg\min_{f} \sum_{i=1}^{n} \ell(f_i + g_i \mid y_i, N_i) + \frac{\lambda}{2} f^T P f. \tag{9}$$

Specifically, for any elements of f with observations, we shift the prediction f_i from the kernel regression in the likelihood by the offset g_i predicted from the previous model step.

In the case a grid point i is unobserved, the model effectively sets $N_i = 0$, weighting the negative log likelihood by zero there.

In the limiting case of no observations, then f will revert to zero, and our prediction reverts to g.

Section 2.3.5: OneMod for mortality envelope estimation

Section 2.3.5.1: Data preprocessing

Weight Adjustment

The sample size column corresponding to each datapoint controls the relative weight of each point in the model. However, the available sample size does not capture non-sampling variation, which can be significant depending on the data source. For example, we expect incomplete data (those where completeness is known to be low) to be noisier than complete data, and we know some data types in general and within location to be noisy. We make the following weight adjustments:

- Multiply sample size by completeness measure, so datapoints with larger completeness measures are weighted more heavily.
- Multiply 'Census' and 'DHP' weights by 0.005.
- Multiply 'Survey' and 'HHD' weights by 0.1.
- Multiply VR weights by 0.01 in Kenya, Botswana, Namibia, and Zimbabwe.
- Increase weights in early age groups for Greenland, Iran, United Arab Emirates, Oman, Croatia, Luxembourg, Barbados, Belize, Cyprus, Algeria, and Morocco.

Compactification

We model the mortality rate (deaths divided by population). For most age groups and years, this quantity is less than 1, suggesting a binomial model. However, for youngest (particularly early neonatal and late neonatal) and oldest age groups (particularly 90–94 and 95+) this may not hold, since we use mid-year populations for each age group, and the populations are also estimated, introducing further potential for reported rates above 1.

To address this issue, we used a compactification technique. Specifically, we first scaled all the observations down by a factor of 4, outliering all observations above 4. We then modelled the resulting observations using a binomial likelihood, and re-scaled the estimates by 4 after the analysis.

Section 2.3.5.2: Covariates

We used five GBD covariates to model mortality rate:

16. `sdi` (SDI)
17. `hiv` (HIV/AIDS mortality rate)
18. `covid_asdr` (age-standardised COVID-19 mortality rate)
19. `is_island` (a binary covariate indicating whether each location is an island)
20. `paf_stand_all` (an all-risk population attributable fraction covariate)

Covariates were chosen in part based on their availability over the entire time series, since GBD produces a limited number of covariates for 1950–2023. We tested models using lag-distributed income (LDI) per capita and education together without SDI, and they did not perform as well as models that included SDI (but excluded LDI and education, since these are components of SDI). We used HIV/AIDS and COVID-19 mortality rate covariates to model the important effects of these causes. Finally, we chose the all-risk PAF covariate as a summary measure of underlying risk factors that influence mortality.

The first four covariates were available for all locations for the entire time series (1950–2023). The last covariate, however, was only available from 1990 onward.

SDI

The Socio-demographic Index (SDI) is a composite indicator of background social and economic conditions that influence health outcomes in each location. In short, it is the geometric mean of 0 to 1 indices of total fertility rate (TFR) for those younger than 25 years old (TFU25), mean education for those 15 years old and older (EDU15+), and LDI per capita. After calculating SDI, values are multiplied by 100 for a scale of 0 to 100. See section 5 of this appendix for more details.

HIV

To calculate HIV age-specific mortality rates, we modified two tools developed by the Joint United Nations Programme on HIV/AIDS (UNAIDS):

EPP and EPP-Age Sex Model (EPP-ASM)

These tools generate HIV burden estimates in line with observed prevalence data. They incorporate many of the same assumptions as Spectrum. EPP fits a simpler model to HIV prevalence data from representative surveys and surveillance sites to estimate prevalence and incidence for the 15–49-year age groups. EPP-ASM incorporates the full population project of Spectrum and produces age-sex-specific prevalence, incidence, and mortality.

We modified EPP-ASM by building a paediatric module that mirrored that of Spectrum. Perinatal and breastfeeding transmission was calculated as a function of prevalence among pregnant women and maternal-to-child transmission programme data. We additionally improved the fit to prevalence data by allowing flexibility in the age distribution of incidence over time. We parameterised the ratio of incidence among ages 15–24:25+ as a constant before year 2000 and a linear regression thereafter. This allowed for the shifts in the age distribution of incidence observed over the course of the HIV epidemic to be reflected in our results.

Spectrum natural history model

This model generates HIV incidence, prevalence, and death by age, sex, and year using time series estimates of HIV incidence, demographic inputs (such as HIV-free mortality and population), assumptions about CD4 progression rates, and assumptions about on-antiretroviral therapy (ART) and off-ART HIV mortality rates by age, sex, and CD4 rate. We used treatment coverage time series from UNAIDS.

We made several modifications after creating a replica of Spectrum in Python (starting with GBD 2013). First, we sex-split incidence based on a model that was fit to the sex ratio of observed prevalence in countries and territories with nationally representative surveys. Second, our child model included CD4 progression and CD4-specific mortality rates that came from a model fit to survival data from the International epidemiology Databases to Evaluate AIDS (IeDEA), as well as ART distribution data from IeDEA. Additionally, we scaled all input values by a uniformly sampled factor between 0.9 and 1.1 to generate estimates with realistic ranges of uncertainty.

Age-standardised COVID-19 mortality rate

We used modelled age-specific mortality rates due to COVID-19 for years 2020–2023. Data on COVID-19 mortality rates were the same as for GBD 2021 with numerous additions for years 2022 and 2023. The model used is part of the causes of death estimation process for GBD 2023.[25]

Island indicator

We reviewed all locations in the GBD 2023 location hierarchy and selected any locations that were fully bordered by water and had no shared land border with another country. We considered these locations to be islands.

All-risk population attributable fraction

We calculated the total risk population attributable fraction (PAF) for age-standarised all-cause mortality, excluding HIV and shock deaths, using standard epidemiological methods. These PAF values were estimated as part of the GBD 2023 risk factor analysis.[25]

Section 2.3.5.3: SpXMod stage-wise model

The SpXMod stage-wise model captures variation due to covariates, followed by location and year intercepts and trends. Two identical models were fit for 1950+ and 1990+ datasets, using the available covariate sets. The only difference is that the 1990+ model also used the `paf_stand_all` covariate.

Each stage-wise models used four stages, described below.

Stage 1: Global covariate model

We fit an age-specific covariate model, along with intercepts by age and location. Specifically, we use the binomial model (1) where the feature a_i contains

21. age-specific covariate features (20×4 for the 1950 + model and 20×5 for the 1990 + model)
- global, super-region, and region-specific sets of age-specific intercepts

We specify a relational structure across age for covariate multipliers and the intercepts, penalising differences across adjacent age groups.

We place sign priors on the coefficients, requiring `covid_asdr`, `hiv`, and `paf_stand_all` to have a nondecreasing impact on mortality, and `is_island` and `sdi` to have a non-increasing impact.

We also impose a monotonically increasing prior specifically for overall mortality rates in the three oldest age groups for global, super-region, and region intercepts.

The explicit formulation for the combined model can be written as follows. Let M denote the total number of age groups and K the total number of locations. Let θ denote all unknown parameters, including age-specific covariate multipliers and region and age-specific intercepts:

$$\theta = \begin{bmatrix} \theta_1^C \\ \vdots \\ \theta_M^C \\ \theta_1^I \\ \vdots \\ \theta_{KM}^I \end{bmatrix}$$

where superscript C corresponds to covariate multipliers while superscript I corresponds to intercepts. We have $\theta \in \mathbb{R}^{M+KM}$.

Let i index all the observations, with a feature row a_i that links the observation to the corresponding covariates and location-specific intercepts, so that the data for the i th training point can be written as a tuple (y_i, a_i), with $a_i \in \mathbb{R}^{M+KM}$ just as θ. Just as in any GLM, the quantity $a_i^T \theta$ serves as a linear predictor, which is mapped to the binomial datapoint through a link function.

Specification of how strength is borrowed across dimensions is specified using the matrix C, which penalises differences in inferred covariate multipliers across adjacent age groups, and intercepts in adjacent age groups and locations. We specify C through is action on the parameter vector:

$$\theta^T C\theta := \sum_{k,l} \gamma_C \|\theta_k^c - \theta_{k+1}^c\|^2 + \gamma_I \|\theta_l^I - \theta_{l+1}^I\|^2 + \gamma_{CI} \left\|\theta_{k,l}^I - \theta_{k+1,l+1}^I\right\|^2$$

Finally, let D impose any inequality constraints on the covariate multipliers (eg, non-positivity of mortality rates with respect to increasing SDI). Each constraint for each covariate multiplier forms a row in the matrix D that acts on θ, specifying the element that should be non-positive, eg,

$$D\theta^C = \begin{bmatrix} \theta_1^{sdi} \\ \vdots \\ \theta_M^{sdi} \\ -\theta_1^{hiv} \\ \vdots \\ -\theta_m^{hiv} \\ \vdots \end{bmatrix}$$

Then the full inference problem can now be compactly written as

$$\min_{\theta} \sum_{i} \ell(y_i; p_i, N_i) + \frac{1}{2}\theta^T C\theta \quad (10)$$
$$\text{subject to } D\theta^C \leq 0$$

where we have

$$p_i = \text{expit}(a_i^T \theta_i)$$

and the negative log-likelihood ℓ is as in equation (1).

Stage 2: Global time model

We use the result from the global covariate model as a constant offset in equation (2).

We then fit region- and age-specific trends and intercepts for global, as well as the central Europe, eastern Europe, and central Asia; sub-Saharan Africa; Latin America and the Caribbean; and north Africa and the Middle East super-regions.

The general form of the model is similar in (10). To avoid confusion, we denote the parameter vector by β, and it now contains region- and age-specific slopes. Abusing notation here to denote the number of regions by K, we have

$$\beta = \begin{bmatrix} \beta_1^C \\ \vdots \\ \beta_M^C \\ \beta_1^R \\ \vdots \\ \beta_K^R \end{bmatrix}$$

We do not impose any constraints through D, and so we can write the time model as

$$\min_{\theta} \sum_{i} \ell\big(y_i; p_i(\beta \mid \hat{\theta}), N_i\big) + \frac{1}{2}\theta^T C\theta \quad (11)$$

where

$$p_i(\beta \mid \hat{\theta}) = \text{expit}\big(a_i^T \hat{\theta} + b_i^T \hat{\beta}\big), \quad (12)$$

with $\hat{\theta}$ the age model inferred from (10) and b_i the feature vector comprising a vector of 1s and elapsed time, so that β can be interpreted as intercepts and trends that are age- and region-specific.

Stage 3: National model

We use the result from the Global time model as a constant offset in (2). We then add a national- and age-specific intercept, with offsets from Stage 2 that are input analogously to (12), with estimates from Stage 2 now readily available, and the unknown parameters in this stage corresponding to the national- and age-specific intercepts.

Here, we use the C matrix analogously to (10), but now pulling the intercepts towards the region-level intercept, with a variable strength depending on the super-region:

$$(\theta^I)^T C\theta^I = \sum_{r} \gamma_R \left\|\theta_r^I - \hat{\theta}_R^I\right\|^2$$

where $\hat{\theta}_R^I$ is the estimate for the super-region intercept, and r indexes the corresponding estimates for the nations within that super region.

We also add a monotonically increasing constraint specifically for the three oldest age groups for each nation. The constraints are specified using the matrix D just as in (10), and corresponds to the requirement that

$$\theta^I_{M-2} \leq \theta^I_{M-1} \leq \theta^I M$$

for each national intercept.

Stage 4: Subnational model

If we have sub-national locations, we fit another model. We use the result from the National model (Stage 3) as a constant offset in (2). We then fit sub-national age-specific intercepts, along with a monotonically increasing prior specifically for the three oldest age groups for each nation.

The specific structure of this model is equivalent to the Stage 3 National model detailed above. The subnational intercepts pull towards the national-level intercept with the same strength as the national model prior in Stage 3. The constraints on the three final age groups are specified through the matrix D.

Section 2.3.5.4: Reconciling 1990+ and 1950+ models

After fitting separately for 1990+ (using sdi, hiv, covid_asdr, is_island, and paf_stand_all) and for 1950+ (as above but excluding paf_stand_all, which is not available), we create a 'spliced' model that matches at 1990 and gradually to the trend of the 1950+ model. The purpose of the spliced model is to interleave the 1990+ model (which we use for 1990 onward) with the results for 1950–1990 from the 1950+ model. Practically, in logit space, we use the years from 1990–2000 to understand the average gap between the two models. For $t \in [1950{,}1990]$ we then take

$$f(t) = \beta_0 w + \beta_2(1-2) + y_{1990} + (\beta_1 v)t$$

where β_0 is the gap between the models at 1990, β_1 is the average slope of $y_{1990} - y_{1950} - \beta_0$ over the interval [1990, 2000], and β_2 is the mean gap between 1990 and 2000, while w and v are weights that go from 1 to 0 and from 0 to 1 respectively between 1950 and 1990.

The spliced model itself is used as an offset to KReg in the next stage, meaning that the final model is fit to data from this baseline.

Section 2.3.5.5: KReg model

The spliced predictions are used as an offset for KReg following (9)

$$\hat{f} = \arg\min_f \sum_{i=1}^{n} \ell(f_i + g_i \mid y_i, N_i) + \frac{\lambda}{2} f^T P f.$$

where we fit for f with the offset g set to be the spliced model predictions discussed in the previous section.
As discussed in section 2.4.3, the final estimate follows the prior g when there is no data, and otherwise fits to the available observations y_i with g_i as baseline. 'Better' that are able to predict closely to y_i means that it is easier for the kernel to adapt to the remaining signal.

The remaining model specification is all in building the prior smoothing matrix λP that is the 'information' of the kernel process. Our kernels are independent across locations, and for each location we use a 2-dimensional kernel over age and year, see section 2.4.2. For the year, we use the sum of three kernels: two Matern $3/2$ kernels (7) with length scales 12 and 6, and a linear kernel (8). For age, we use a single Matern $3/2$ kernel with length scale 8. The purpose of Matern kernels is to capture correlation without assuming as smooth a model as the classic RBF kernel (6). The linear kernel is able to extrapolate trends. Year and age kernels combine through the Kronecker product as described in section 2.4.2.

Section 2.3.5.6: Uncertainty calibration

The model (9) provides classic asymptotic uncertainty for the estimates f_i :

$$f_i \sim N\left(\hat{f}_i, \nabla^2 \ell|_{\hat{f}_i} + \lambda P\right)^{-1} \quad (13)$$

However, from the form of the variance, it is clear that as we strengthen the prior kernel smoothing matrix P, we decrease the posterior variance as well.

To mitigate the effect of the prior, we use a calibration strategy for uncertainty analogous to the one frequently employed in weather prediction.[26]

Once we obtain model-based asymptotic uncertainty for each f_i, we pick a granularity (either location or region) and scale each set of uncertainties so that residuals corresponding to observations has variance 1.

Specifically, the variance of the prediction intervals for the kernel regression model is given by

$$V_p = V(f_i) + \frac{1}{\hat{p}(1-\hat{p})w}$$

where the second term corresponds to the reported variance from a binomial model. We pick α so that the observed residuals, when scaled by $\sqrt{\alpha V_p}$, have variance 1.

Section 2.4: PyDisagg for age-sex splitting

After fitting the first stage OneMod model on data at the most granular age-sex level, we used these mortality rate estimates as the age-sex pattern of mortality to split more aggregated data. This was done using PyDisagg. This tool disaggregates an estimated count observation into buckets based on the assumption that the rate (in a suitably transformed space) is proportional to some baseline rate. The most functionality is to perform disaggregation under the rate multiplicative model that is currently in use.

The setup is as follows: Let D_1,...,D_k be an aggregated measurement across groups g_1,...,g_k, where the population of each is p_i,...,p_k. Let f_1,...,f_k be the baseline pattern of the rates across groups, which could have potentially been estimated on a larger dataset or a population in which have higher quality data on. Using this data, we generate estimates for D_i, the number of events in group g_i and f_i^, the rate in each group in the population of interest by combining D_1,...,D_k with f_1,...,f_k to make the estimates self-consistent. Mathematically, in the simpler rate multiplicative model, we find β such that D_1,...,D_k=$\sum f_i$ ^ $\cdot p_i$. This yields the estimates for the per-group event count, D_i=$f_i \cdot p_i$. For the current models in use, we assume that each rate is a constant multiplied by the overall rate pattern level.

We use the mortality rates from fitting OneMod in stage 1 to thus split mortality rates from data that are in aggregated age-sex groups into the most granular detailed GBD age-sex groups. These split rates are used as data in the second stage OneMod model, along with the original data at the most detailed age-sex level.

Section 2.5: Life table and death number estimation

Section 2.5.1: Raking subnational estimates

For all countries and territories, we raked subnational-level estimated mortality rates from OneMod to separately estimated national-level mortality rates. This is done because subnational mortality rates must aggregate to national estimates, and we generally had more data at the national level or else the data at the national level were more robust due to larger sample sizes.

Section 2.5.2: Adding fatal discontinuities[2]

We excluded data from years with fatal discontinuities from our mortality estimation process because we did not want sudden and temporary jumps in mortality to distort long-term mortality trends in that country or territory. The following section explains how we added deaths caused by fatal discontinuities to the all-cause mortality envelope and life tables. Section 2.6 provides more information about how we estimated the fatal discontinuities mortality.

To add fatal discontinuities to the mortality rates, we first produced full single-year life mortality rates (as detailed in section 2.5.3), to which the fatal discontinuity death rates would later be added. In addition to the single-year death rates, early neonatal, late neonatal, and post-neonatal age groups were used instead of the under-1 age group. The fatal discontinuity death rates come from 1000 draws of deaths due to fatal discontinuities, transformed by our estimates of population. We then calculated fatal discontinuity mortality rates for each single-year age by assuming the same mortality rate for each year in an age group. This preserved the original abridged fatal discontinuity deaths. In cases where all-cause mortality probabilities would be greater than 1 if fatal discontinuities were added, we lowered the all-cause mortality rate by a corresponding amount to prevent this from happening. We then added the 1000 draws of fatal discontinuity mortality rates to the 1000 draws of single-year mortality rates for each location, sex, and age group, creating mortality rates that had fatal discontinuities included. We calculated 95% UIs in the same way as for other estimates, using the 2.5% and 97.5% percentiles of the summed draws.

Section 2.5.3: Life table calculation[2]

We calculated both with-shock (mortality rates inclusive of fatal discontinuities) and without-shock (mortality rates without fatal discontinuities) life tables. The same calculations were done for both. Using the results from section 2.5.1 and 2.5.2, we interpolated the mortality rates above age 1 into single-year age groups (up to 95+) using a linear interpolation on the log of the mortality rate. We then scaled the single-year mortality rates such that they would be equal to the values of the GBD age groups when aggregated. We calculated ${}_na_x$ (defined as the average number of years lived in the age interval, from among those who died in that age interval) as 0.5 for all single-year age groups and using a standard demographic formula which approaches half of the age group length for the detailed under-1 ages (e.g. early neonatal, late neonatal, and 2 post-neonatal age groups). We then used these ${}_nm_x$ and ${}_na_x$ values to calculate full life tables for single years of age (and more detailed under-1 ages) using standard demographic methods. We then calculated abridged life tables for standard GBD age groups by aggregating ${}_nm_x$, ${}_nq_x$, ${}_nL_x$, and ${}_na_x$ using standard demographic methods to the abridged age groups and keeping other parameters like life expectancy constant based on the starting year of the age group.

For aggregate locations (ie, regions, super-regions, and global), we produced aggregate ${}_nm_x$ and ${}_na_x$ by weighting ${}_nm_x$ by population and ${}_na_x$ by deaths:

$$ {}_nm_x \times population $$

We used these death- and population-weighted ${}_nm_x$ and ${}_na_x$ values to produce life tables at the aggregate level.

Section 2.5.4: Death number calculation

We calculated without-shock and with-shock death numbers for standard GBD age groups by taking the corresponding life table ${}_nm_x$ values and multiplying them by our population estimates for each location, year, sex, and age group. Compared to GBD 2021, both life tables and population are estimated to a terminal age of 95+, so no different approach is required to calculate death numbers in the open age interval.

Section 2.6: Fatal discontinuities[2]

Figure B

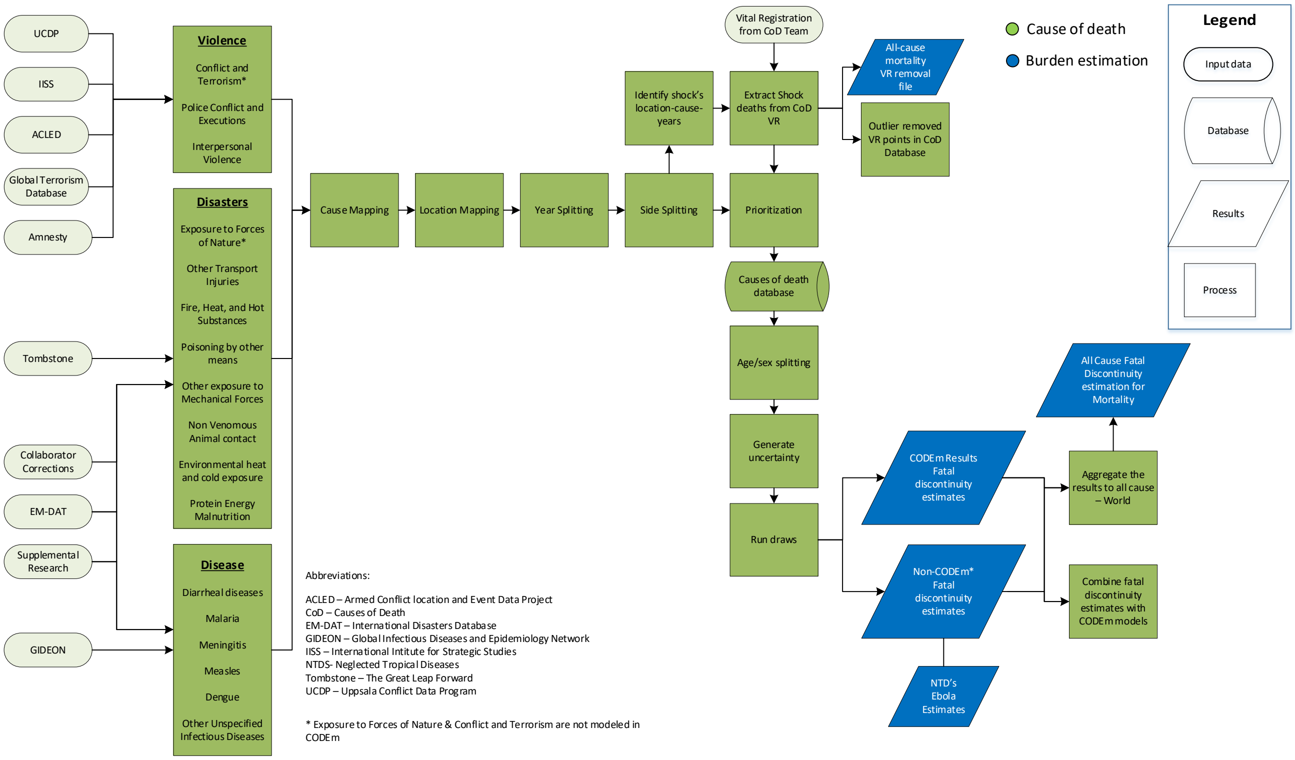

Fatal discontinuities are defined as events that are stochastic in nature and cannot be modelled using cause of death ensemble modelling (CODEm) because they do not have a predictable time trend. Some causes have both fatal discontinuities as well

as a continuous background mortality that has a smooth time trend and can be modelled using CODEm. These include police violence and executions; interpersonal violence; other transport injuries; fire, heat, and hot substances; poisoning by other means; other exposure to mechanical forces; non-venomous animal contact; environmental heat and cold exposure; protein-energy malnutrition; diarrhoeal disease; meningitis; encephalitis; diphtheria; measles; dengue; and other unspecified infectious diseases. Causes without a continuous background mortality that are exclusively estimated using the fatal discontinuity method are conflict and terrorism and exposure to forces of nature. Any other causes are not captured in fatal discontinuities.

Section 2.6.1: Input data

Section 2.6.1.1: Overall

We collected data on fatal discontinuities from a range of sources, namely from country-level VR systems and international databases that reported several cause-specific fatal discontinuities. We also collected supplemental data that have known issues related to quality, representativeness, or time lags in reporting. We describe the different fatal discontinuity data sources, presented by fatal discontinuity sub-cause, below.

Section 2.6.1.2: Discontinuities only (non-CODEm)

For causes not modelled in the Cause of Death Ensemble model (CODEm), all deaths captured in VR were considered to be fatal discontinuities. Deaths that were extracted from cause-specific VR were then subtracted from the all-cause VR data used in the all-cause mortality estimation process.

Conflict and terrorism

In GBD 2023, war is defined as "a state of armed conflict between states, governments, societies, and paramilitary groups." It is "generally characterised by extreme violence, aggression, destruction, and mortality, and the use of regular or irregular military forces." Terrorism is defined as "the unlawful use or threatened use of force or violence against individuals or property in an attempt to coerce or intimidate governments or societies to achieve political, religious, or ideological objectives." We used conflict and terrorism data from the Uppsala Conflict Data Program (UCDP), International Institute for Strategic Studies (IISS), Armed Conflict Location & Event Data Project (ACLED), Global Terrorism Database (GTD), and VR systems and other supplemental data sources (table D). A case was assigned for each event using the source's cause coding and any description from available notes.

Table D. Conflict and terrorism data sources

Data source name	Date accessed	Years of data downloaded	Type of data included
**Uppsala Conflict Data Program**[27]			
Georeferenced Event Dataset, Version 23.1	3/5/2024	1989-2022	UCDP battles, non-state, and one-sided conflict deaths with the most disaggregated location information available
PRIO Battles Deaths Dataset, Version 23.1	3/5/2024	1946-2021	Armed conflict (civil wars, etc.)
**International Institute for Strategic Studies**			
Armed Conflict Dataset	11/17/2016	1997-2016	Insurgency, Inter-state, Intra-state conflict deaths
**Robert S. Strauss Center for International Security and Law**			
Armed Conflict Location and Event Dataset (ACLED)	3/3/2024	1997-2024	Actions of opposition groups, governments, and militias in selected locations in Africa, Asia, and the Middle East specifying the exact location and date of battle events, transfers of military control, headquarter establishment, civilian violence, and rioting
**University of Maryland, Global Terrorism Database**			
Global Terrorism Database (GTD)	9/12/2023	1970-2020	Attacks aimed at attaining political, economic, religious, or social goal, includes evidence of intention to coerce, action was outside precepts of International Humanitarian Law

**University of Chicago, Chicago Project on Security and Threats**			
Suicide Attack Database (CPOST SAD)	11/26/2017	1982-2016	Attacks in which an attacker kills him/herself in a deliberate attempt to kill others, includes only attacks perpetrated by non-state actors
**Amnesty International**			
Amnesty	9/12/2023	1991-2022	Police conflict and executions

Four major conflicts were identified that were not represented in these databases: genocide in Bangladesh in 1971,[28] genocide in Burundi in 1972 and 1993,[29] and civil conflict in Albania in 1997.[30] In these cases, literature sources were used to account for these fatal discontinuities.

Deaths of migrants who die en route are split into their respective countries of origin using patterns found from the Missing Migrants Project.[31]

Exposure to forces of nature

In GBD 2023, exposure to forces of nature is defined as "a force that is beyond human control." The Centre for Research on the Epidemiology of Disasters' International Disaster Database (EM-DAT)[32] served as the primary non-VR source of fatal discontinuities due to exposure to forces of nature (ie, natural disasters, lightning, earthquakes, volcanic eruptions, avalanches, storms, and floods). Data from EM-DAT were last accessed February 28, 2024. Supplemental online research was conducted for events where EM-DAT and VR were not up-to-date.

Section 2.6.1.3: Partial discontinuity (CODEm)

For causes modelled in CODEm with fatal discontinuities obscured in the time trend, a process was established to avoid duplication of fatal discontinuity deaths in CODEm and the fatal discontinuity estimates. First, location-cause-years were identified through outside non-VR sources. If these location-cause-years also had VR death estimates that were greater than the average of the immediate surrounding years, the difference between the identified year and the average of the surrounding years was included in the relevant cause for the fatal discontinuities database. The extracted deaths for all fatal discontinuity causes from VR were then subtracted from the all-cause VR data used in the all-cause mortality estimation process.

Police conflict and executions

In GBD 2023, police conflict and executions are defined as "the lawful use or threatened use of force or violence against individuals or groups of people or property in an attempt to achieve political or socioeconomic objectives for a state." Data for police conflict and executions came primarily from Amnesty International, but other sources such as UCDP, ACLED, and VR that reported deaths due to legal intervention were also cause-mapped to police conflict and executions.

Homicide

In GBD 2023, homicide is defined as "the use of violence against an individual or group of people in an attempt to achieve non-political, religious, or ideological objectives." Data for homicide came from VR, IISS, GED, ACLED, and other supplements. Events were mapped to homicide where the notes found in the raw data indicated gang violence. Deaths from IISS, GED, and ACLED were then split among three homicide sub-types; physical violence by firearms, physical violence by sharp object, and physical violence by other means, based on the rates calculated from VR by country and territory if available, and by region if country VR was unavailable.

Protein-energy malnutrition (PEM)

In GBD 2023, protein-energy malnutrition is defined as "a lack of dietary protein and/or energy" and covers famines as well as severe droughts. The primary source for PEM data, other than VR, was EM-DAT. Supplemental online research was conducted for events where EM-DAT and VR were not up to date. The Tombstone report was used to estimate deaths attributed to the famine during the Great Leap Forward in China in the 1960s.[33]

Other injury causes

Other injury causes include other transport injuries (eg, plane, train, and boat accidents); poisonings; fire, heat, and hot substances; and other exposure to mechanical forces (eg, building collapse). The primary data source for these events other

than VR was EM-DAT. Supplemental online research was conducted for events where EM-DAT and VR were not up to date.

Meningococcal meningitis and other diseases

In GBD 2023, fatal discontinuities due to a subset of infectious diseases were estimated, including meningococcal meningitis (or meningococcal infection), diarrhoeal disease caused by cholera and dengue. These infectious diseases were first included on the fatal discontinuity cause list for GBD 2016 because (1) their current modelling strategies in CODEm did not optimally capture the potentially highly variable—or epidemic—mortality levels and trends characteristic of these causes; and (2) they can contribute to significant total fatalities in a given location-year. Other infectious diseases for which the latter is true—high death rates in the presence of an outbreak or epidemic—are currently modelled with alternative cause of death methods (eg, natural history models for measles and yellow fever), which allow for greater variation year-over-year if or when outbreaks occur.

The Global Infectious Diseases and Epidemiology Network (GIDEON) and EM-DAT served as the primary data sources for collating cholera and meningococcal meningitis or meningococcal infection death reports.[34,35] For any year that cholera or meningococcal meningitis deaths were recorded in a country or territory covered by GBD, reported deaths were directly extracted from 1950 to 2021. If GIDEON or EMDAT had reporting gaps in cholera or meningococcal meningitis deaths, and WHO reports had coverage for those years, the WHO reports were used. For the Yemen Cholera outbreak in 2016 and 2017, estimates from local collaborators were used in the absence of other data sources.

Section 2.6.2: Location mapping

Every event in the fatal discontinuities database was mapped to a GBD location using a four-step process that includes the following steps in succession: (1) manual mapping, (2) string matching, (3) GPS overlay, and (4) geocoding. If an event was manually mapped, the location was assigned without the use of any other map types. In manual mapping, events are manually assigned to locations by matching the location provided in the raw data to a GBD location. During string matching, an event's location strings were directly compared to the GBD ASCII location names. During GPS overlay, events that have GPS coordinates provided were overlaid onto a map of GBD locations. If the event was placed over a GBD most-detailed location, the event was assigned to that location. During geocoding, the event's location string was entered into Open Street Maps, which returns GPS coordinates. These coordinates were processed using GPS overlay to return GBD locations. This hierarchy provides results where the results of manual mappings are considered the most reliable, followed successively by string matching, GPS coordinates, and then geocoding.

Section 2.6.3: Side splitting

Many fatal discontinuities, such as war, have deaths that are reported across multiple locations. In these instances, deaths were split between the population from both locations, unless estimates by side were provided. If the resulting locations were at the most detailed GBD location level, no further splitting was needed. If a location was not most detailed, the deaths were distributed among the child locations by population.

Section 2.6.4: Prioritisation

Where multiple sources reported shock deaths for the same location-year-cause, a cause-specific prioritisation scheme was followed that reflected the available detail in the cause-specific datasets. For example, the Georeferenced Event Dataset from UCDP was prioritised above all other non-VR sources because it included detail on how deaths were distributed between multiple actors and locations in each conflict event. In most cases, VR from 4-star or 5-star locations was used where available. In some cases, VR from 4-star or 5-star locations was not chosen if there were well-known data quality issues or discrepancies in the cause of death data reporting related to a particular event (eg, supplemental death data for Louisiana were used for Hurricane Katrina because of established data reporting issues).

Section 2.6.5: Age-sex splitting

We ran all compiled data through the causes of death age-sex splitting process, except in cases where we had substantial and reliable information about the age distribution of specific events with high mortality, such as United States mortality during the Vietnam war and Iran mortality during the Iran-Iraq conflict in the early 1980s.[36]

Section 2.6.6: Assigning uncertainty and generating draws

Section 2.6.6.1: Uncertainty analysis

We generated UIs for deaths caused by conflict and terrorism using UCDP high and low mortality estimates, except for deaths in Iraq from 2003 to 2016. During this time period, deaths due to conflict and terrorism were estimated using a combination of supplemental sources. We used death counts from the Iraq Body Count (IBC),[37] which had the lowest number of deaths from among the sources we found, as the lower bound of the UI from 2003 to 2016. We used estimates from the Iraq Mortality Study (IMS) by Hagopian and colleagues[38] from 2003 to 2006, the deadliest years of the war, to scale deaths to produce the upper UI limits using the formula below:

$$deaths_{GBD\ 2017,high} = deaths_{IBC} \cdot \overline{\left[\frac{deaths_{IMS}}{deaths_{IBC}}\right]}_{2003-2006}$$

GBD 2023 used the average ratio between IMS and IBC reported deaths between 2003 and 2006, multiplied by the number of deaths reported by the IBC. This high estimate was carried forward through 2017 under the assumption that IBC similarly undercounts the number of deaths due to the ongoing civil war in Iraq. The final, best estimate for conflict and terrorism deaths in Iraq from 2003 to 2016 is the midpoint of the high and low estimates given above.

When high and low estimates were not included in the existing data, we applied the regional average UI to the available mortality estimate, for all fatal discontinuity causes.

We assumed a log-normal distribution using mean mortality rates and standard error based on high and low estimates. When the standard error was less than 10e-8, we set the draws equal to the mean rate. We then sampled 1000 draws from the log-normal distribution and converted the draws back to counts space. We used the count space draws to calculate final means and UIs.

Section 3: Fertility[2]

Section 3.1: Overview

Prior to GBD 2016, we used United Nations World Population Prospects fertility estimates for all countries and territories.[39] Starting with GBD 2016, we estimated fertility rates within the GBD framework. We based our estimate of total fertility rate (TFR) on a systematic synthesis of all data available for all GBD 2016 locations, using the age-specific fertility pattern from World Population Prospects (WPP).[40] Starting in GBD 2017, we estimated age-specific fertility rates (ASFRs) for ages 10 to 54 years based on a systematic synthesis of all available data for all GBD locations. We calculated TFR as a function of the ASFRs.[41] We compiled a total of 53 082 unique location-source-years of data for females aged 10–54 for the period between 1950 and 2023.

Section 3.2: Modelling fertility

Section 3.2.1: Fertility data source types

We sought to use accurate and complete accounts of livebirths reported according to the age of mothers. Complete livebirth registration reports are designed to account for all births in a single country, territory, or subnational location in a single year, which makes them the gold standard for fertility data. Most high-income countries and territories maintain high-quality VR systems with information on dates and locations for all births as well as demographic characteristics of each mother. Many lower-income countries and territories, however, rely on birth registries with incomplete data coverage or interrupted and/or delayed reporting. In these locations, we used household surveys with birth history information for women aged 15 to 49 at the time of the survey, but had to use birth registries for females aged 10–14 and 50–54 since most household surveys do not collect birth histories from those age groups.

For triangulating the level and age-pattern of fertility, we had to rely on other types of data sources, primarily household surveys and censuses in areas, where birth registration data quality and completeness were low. Household surveys and censuses contained two primary types of fertility information: CBHs and SBHs. See sections 2.1–2.2 for more information about CBHs and SBHs.

Section 3.2.2: Fertility data identification and synthesis

We obtained VR data from the UN Demographic Yearbook (DYB) from the UN Statistics Division (UNSD),[42] the Human Fertility Collection (HFC) and Human Fertility Database (HFD) from the Max Planck Institute for Demographic Research

(MPIDR),[43,44] the WHO mortality database,[45] official publications, online data portals of national statistical offices, and international collaborators. The HFC, HFD, and DYB are compilations of registry-based fertility data from national statistical offices and research institutes. We obtained DYB data on live births by age of mother for every year available from 1950 to 2022. We obtained the complete set of age-specific empirical data from HFC up to 2022 and from HFD up to 2022 but excluded country-year-ages already accounted for by the DYB. In addition to DYB, HFC, and HFD data, we obtained data from SRSs where available, primarily in South Asian countries such as India, Pakistan, and Bangladesh. In total, at the national level, we obtained 9217 unique country-source-years of VR data, with 2496 from before 1970 and 3038 from 2000 onwards. We also had 33 country-source-years of data from SRS.

We identified fertility data from censuses and household surveys using the Global Health Data Exchange (ger) by searching for "complete birth history," "summary birth history," and "fertility" from among the records categorised as "survey" and "census." Research team members reviewed these data to verify whether they included the necessary information for GBD analysis. We then conducted additional research to identify and fill gaps in data, primarily by data seeking on country statistical office websites and seeking recently released surveys such as DHS, MICS, WFS, and Reproductive Health Surveys (RHS). In-country collaborators also assisted in acquiring data that were not publicly available. For low-income locations (especially in sub-Saharan Africa), we sought out colonial censuses from the 1950s and 1960s with SBH data. For sources that contained microdata, we computed period ASFRs every three years over a 15-year recall using CBH data and calculated the average number of children ever born (CEB) for each year of mother's age, which we later split by cohort age patterns from the first modelling stage (see section 3.2.5), using SBH data. For sources that did not contain microdata, we extracted period ASFR or average CEB by mother's age from reports or other publications. In total, we used 796 CBH and 917 SBH sources from surveys and censuses. We were occasionally unable to identify whether a survey that contained tabulated period ASFRs was a CBH or SBH survey, but these data only accounted for 144 country-source-years from 27 sources. We then estimated fertility rates for the 10–14-year age group as a function of estimated fertility in the 15–19-year age group and for the 50–54-year age group as a function of the estimated fertility in the 45–49-year group. We provide more information about the age-specific fertility estimation process below. After estimating ASFRs, we computed summary measures of fertility including TFR and total fertility under age 25 (TFU25).

Section 3.2.3: Age-specific fertility rate estimation for 15 to 49 years

As stated above, we used ST-GPR to estimate ASFR for age groups 15–19, 20–24, 25–29, 30–34, 35–39, 40–44, and 45–49. The methods for this process have been described in full elsewhere,[16] but in short, we did the following:

(1) estimated ASFR for the 20–24 age group using age-specific data from CBH and VR sources, using mean years of education in that age group as a covariate; (2) estimated ASFR for the other age groups using age-specific data from CBH and VR sources as well as age-specific mean years of education and the 20–24 age group ASFR; (3) split SBH and other total births data by age and period using estimated location, time, and ASFR for each age group; (4) re-estimated ASFR for the 20–24 age group using CBH, VR, and period-age-split SBH data; and (5) re-estimated ASFR for the other age groups using CBH, VR, and the period-age-split SBH data.

We implemented the ST-GPR models for ASFR as explained below. The first stage of our mixed effect regression was fit in bounded logit space:

$$Logit\left(\frac{ASFR\ data - lower\ bound_{age}}{upper\ bound_{age} - \ lower\ bound_{age}}\right)$$

We set the lower bound as the minimum fertility by age across time and location and the upper bound, after dropping implausibly high ASFRs over 0·5, as the 99·3 percentile of fertility by age across time and location. The upper bound set an implied maximum TFR of 9·35.

We used the following formula for our mixed effects regression:

$$logit_{bound}\left(ASFR_{20\text{–}24}\right)_{c,t,s,i} = \beta_0 + \beta_1 * female\ education_{c,t} + \gamma_{cs} + \varepsilon_{c,t,s,i}$$

$$logit_{bound}\left(ASFR_{n\text{–}n+4}\right)_{c,t,s,i} = \beta_0 + \beta_1 * fem\ edu_{c,t} + spline(ASFR_{20\text{–}24,c,t}) + \gamma_{cs} + \varepsilon_{c,t,s,i}$$

$$\gamma_{cs} \sim N(0, \sigma^2_{\gamma cs})$$

$\varepsilon_{c,t,s,i} \sim N(0, \sigma_\varepsilon^2)$

Where

c is location, t is time, s is source of datapoint i

n is between 15 and 45

β_0 is the intercept

β_1 is the coefficient on female education

γ_{CS} is a location-source random intercept

ε is the residual

Female education and the 20–24 age group ASFR estimates were specific to each country or territory and year.

We only used female education as a covariate in high-income locations for the 20–24 age group, not for the other age groups. We fit separate models for the high-income, sub-Saharan Africa, central Europe, eastern Europe, and central Asia super-regions to factor in the differences in the relationships between the 20–24 age group ASFR and the ASFR of other age groups. We selected the knots in the linear spline (in logit space) by super-region and age group, as outlined in table E below.

Table E. Knots on ASFR 20–24

Region	Age	Knot
Central Europe, eastern Europe, and central Asia	15	NA
Central Europe, eastern Europe, and central Asia	25	-1·5
Central Europe, eastern Europe, and central Asia	30	-2
Central Europe, eastern Europe, and central Asia	35	-1·75
Central Europe, eastern Europe, and central Asia	40	-1·75
Central Europe, eastern Europe, and central Asia	45	-2
High-income	15	NA
High-income	25	NA
High-income	30	-2·25
High-income	35	-2
High-income	40	-2·25
High-income	45	-2·25
Others	15	NA
Others	25	-1·5
Others	30	-1·3
Others	35	-1·3
Others	40	-2
Others	45	-2·5
Sub-Saharan Africa	15	NA
Sub-Saharan Africa	25	-1·75
Sub-Saharan Africa	30	-1·25
Sub-Saharan Africa	35	-1·3
Sub-Saharan Africa	40	-1·5
Sub-Saharan Africa	45	-1·75

We outliered data that reported improbably high ASFR (ie, ASFR over 0·5); had 0 values as a result of sampling error, particularly in the 45–49-year age group; reflected an undercounting of births, when we could not adjust the data using other

sources; or reported implausibly high mortality levels or trends compared to complete VR data or other more reliable sources.

Section 3.2.3: Data source adjustment

After running the mixed effects model, we adjusted data to a reference source using the random intercept on the concatenation of location and source. To get the adjustment factor, we did the following using the equation below: (1) calculated the difference between the fixed and random effects of the reference source, (2) calculated the difference between the fixed and random effects of the datapoint for the specific source, (3) added the two differences together. We then added this adjustment factor to the data to get an adjusted value.

$$Adjustment\ Factor = (Location\ Source\ RE_{ref} - Location\ Source\ RE_{data\ point})$$

where RE represents a random intercept of either a reference source or a datapoint-specific location-source.

When we had more than one reference source for a single location, we averaged the values of the location source random effects from all the reference sources and used that for the $Location\ Source\ REref$ part of the equation.

We primarily chose reference sources as those that met one of the following criteria: (1) complete VR for locations with complete VR, (2) an average of complete birth history sources for locations with one or more complete birth histories, and (3) an average of all the sources for each location for locations without complete VR or complete birth histories. We considered a location-year of VR to be complete if the estimated completeness of child death registrations was over 95% according to the previous round of GBD.[16] We also chose reference sources for some locations using expert judgement. For example, the 1950s and 1960s censuses in sub-Saharan Africa are widely viewed as an accurate reflection of depressed fertility in that region at that time, so we used those as reference sources.

Section 3.2.4: Hyperparameter selection

We used the outputs of the previous processes to implement residual smoothing and GPR. We chose hyperparameters for these steps based on a location-age-specific data density score. We calculated data density scores based on the years for which VR sources were available plus the number of unique CBH and SBH sources available for the given location, using the following computational methods for each type of data source:

22. **Complete VR sources:** calculated as the number of years for which VR data were available. If the number of births in the age group was below 100, this part of the score was down-weighted by the ratio between the number of births and 100.
23. **Incomplete VR sources:** calculated the same way as with complete VR data, but down-weighted by 0·5.
24. **Total CBH sources:** the number of unique complete birth histories for a single location.
25. **Total SBH sources:** the number of unique summary birth histories for a single location. We calculated the data density score using the following equation:

$$DD\ Score_{loc,age} = Complete\ VR\ years_{loc,age} + (2 * Number\ CBH\ Sources_{loc,age})$$

$$+ (0.25 * Number\ SBH\ sources_{loc,age}) + (0.5 * Incomplete\ VR\ years_{loc,age})$$

$$+ Number\ Other\ Sources_{loc,ag}$$

Where

DD is the data density

CBH is complete birth history

SBH is summary birth history

In this round of GBD, we updated our time weights to use a beta density function. We assigned hyperparameters α and β for the beta density function, generally based on final data densities, as shown in table F. However, there were exceptions where we manually assigned a different set of hyperparameters.

Table F. Hyperparameter values by data density

Data density	Alpha	Beta	Zeta	Scale
Over 50	500	500	0·99	5
Between 30 and 50	100	100	0·9	10
Between 20 and 29	20	20	0·8	15
Between 10 and 19	15	15	0·7	15
Under 10	10	10	0·6	15

Where the time weights were calculated as:

$$w_t = \frac{\frac{\Gamma(\alpha+\beta)}{\Gamma(\alpha)\Gamma(\beta)} x^{\alpha-1}(1-x)^{\beta-1}}{\frac{\Gamma(\alpha+\beta)}{\Gamma(\alpha)\Gamma(\beta)} 0.5^{\alpha-1}(1-0.5)^{\beta-1}} = \frac{x^{\alpha-1}(1-x)^{\beta-1}}{0.5^{\alpha+\beta-2}}$$

And:

$$x = \frac{(t+72)}{144}$$

In cases of incomplete VR sources, we defined data variance as the difference between the spatiotemporal prediction and the unadjusted data. Some location-ages had very little data, resulting in implausible variance. As such, for location-ages with fewer than five datapoints, we used the maximum data variance in the location's GBD region.

For complete VR sources, we assumed that non-sampling variance was 0. We calculated sampling variance for these sources using the following binomial equation:

$$Sampling\ Variance = \frac{ASFR * (1 - ASFR)}{Births}$$

We then calculated amplitude and applied it to all locations other than high-income VR-only national locations with 40+ years of VR data. For these locations, we took the mean of the location-specific variance of the difference between the data and the spatiotemporal smoothing. However, we only included national locations from 1990 to 2023.

Section 3.2.5: SBH methods

For the earlier years of the study period, CEB data were more readily available from SBHs than from CBHs. While there are numerous methods for calculating period-age-specific fertility from SBH information, the Brass Parity/Fertility ratio method is used most often. However, this method assumes a constant ASFR over time. We wanted a dynamic measure of cohort age patterns over time, so we instead used ASFR estimates from CBH and VR data from the first run-through of ST-GPR described above to split SBH into period ASFR. We used these estimates to compute implied annualised fertility for all the five-year birth cohorts represented in a given SBH from age 10 through whichever came sooner: age 54 or the year of the survey. To account for births that occurred in years when part of the initial age group had moved into the next age group, we calculated the weighted average of estimated ASFRs in the age groups on either side of the selected age group and took that average as the fertility experienced by that hypothetical cohort in that year, assuming a uniform age distribution within the age group. For example, for a cohort of women aged 20–24 in 1984 with ASFR F, we would compute the ASFR experienced by this cohort as women aged 21–25 in 1985 as

$$.8 * {}_5F_{20}^{1984} + .2 * {}_5F_{25}^{1989}$$

since 20% of the cohort had aged into the 25–29 age group by the following year.

We then used the implied annualised cohort ASFR to calculate cumulative cohort fertility up to the age of each cohort at the time of the survey. We compared implied cumulative fertility to observed cumulative fertility (average CEB from SBHs) by each cohort to get a scaling factor. We then applied this scaling factor to the original implied cohort age pattern, which distributed CEB back across time and age. This method only covered birth cohorts between 1940 (who began to experience ASFR 10–14 in 1950, the first year of our study period) and 2013 (who began to experience ASFR 10–14 in 2023, at the end of our study period).

Splitting of total birth and historic location aggregate data

A large portion of the data could only be obtained already aggregated by age and/or location (eg, total live births instead of births by mother's age; former USSR prior to its dissolution). For these data, we split just the CBH and VR data using the age and location proportions designated in the first ST-GPR run-through. After splitting the data, we reran the estimation process describe above using all CBH, VR, and period and age-split data as well as location and age-split miscellany. This improved the availability of past data and gave us more information about aggregate levels of fertility over time.

Section 3.2.6: Age-specific fertility rate estimation for 10- to 14-year-olds and 50- to 54-year-olds

We estimated ASFR for the 10–14 and 50–54-year age groups separately because data for these age groups in locations without VR systems were scarce. We used the relationship between ASFR in sequential age groups to estimate both age groups. For the 10–14 age group, we ran a mixed effects regression on the log of the ratio of ASFR 10–14 over ASFR 15–19, and used ASFR 15–19 and nested random intercepts by super-region, region, and location as predictors, as defined by the equation below:

$$\log\left(\frac{ASFR\ 10-14}{ASFR\ 15-19}\right) = \beta_0 + \beta_1 \log(ASFR\ 15-19) + \gamma_{k[j]} + \gamma_{jk[i]} + \gamma_{ijk}$$

$$\gamma_{k[j]} \sim N(0,\sigma^2_{\gamma_{k[j]}})$$

$$\gamma_{jk[i]} \sim N(0,\sigma^2_{\gamma_{jk[i]}})$$

$$\gamma_{ijk} \sim N(0,\sigma^2_{\gamma_{ijk}})$$

Where

i is location, j is region, k is super-region

β_i is a fixed covariate coefficient

$\gamma_{k[j]}$, $\gamma_{jk[i]}$, and γ_{ijk} are nested super-region, region, and location random intercepts

For ASFR in the 50–54 age group, we instead estimated a regression on the log ratio of ASFR 50–54 over ASFR 45–49 with a constant. We did this because there was not a clear relationship between this ratio and ASFR 45–49. We produced 1000 draws from the variance-covariance matrix to generate uncertainty using methods described previously.

Section 3.2.7: Fertility metrics

We calculated TFR as the time-weighted sum of the ASFRs from each age group. To do this, we added ASFR for each five-year age group and multiplied that by the five years spent in each age group. We calculated TFU25 and TFO30 in the same way, but with only the relevant age groups included. We defined livebirths as the sum of ASFR from all age groups multiplied by the 10- to 54-year-old female population from the population model described in section 4.

Section 3.3: Sex ratio at birth

Section 3.3.1: Overview

Another component of population structures and reproductive capacity is the sex ratio at birth (SRB). For the GBD 2023 analysis, we defined SRB as the ratio of total male to total female livebirths in each location in a given calendar year. The naturally occurring SRB is generally approximately 1·05 males per female, with some location-specific variations.[46] Since the introduction of ultrasound technologies and the ability to conduct sex-selective abortions, previously stable SRBs have shifted in some locations as a result of systematic sex preferences for children.[46] SRBs in recent years are particularly skewed in the Caucasus, south Asia, and east Asia. To reflect historic equilibria and recent shifts in SRB, we developed a model to estimate SRB in all GBD countries and territories, Hong Kong, and Macau from 1950 to 2023.

Section 3.3.2: Modelling approach

For GBD 2023, we used the approach for estimating sex ratio at birth that was introduced in GBD 2021.[2] We implemented ST-GPR to estimate a complete time series of the sex ratio at birth for all GBD locations and years. The main differences from previous GBD rounds are additional VR and CBH data, updated outliering decisions following thorough model review, and the use of higher values of the beta hyperparameter for locations with more data. The beta hyperparameter change lowers the smoothness of the model fit, allowing the model to follow the data more closely. See the section 3 fertility sections above for more details on the methodology.

Section 4: Population[2]

Section 4.1: Overview

To estimate population size over a period of time, it is necessary to estimate the initial population and how many people are entering and leaving the population through births, deaths, and migration. The demographic balancing equation expresses how population (N) changes between two points in time.

$$N(T) = N(0) + B[0,T] - D[0,T] + I[0,T] - O[0,T]$$

People can only enter a population through birth (B) or immigration (I) and exit through death (D) or emigration (O). In this analysis, immigration and emigration are collapsed into net migration (G), where net immigration is indicated by a positive value and net emigration is indicated by a negative value.

$$N(T) = N(0) + B[0,T] - D[0,T] + G[0,T]$$

The Bayesian population model used in this study reconciles data on population size obtained through population censuses and registries with GBD fertility and mortality estimates. Below we describe each data source used as an input to the model and then we describe the modelling processes used to estimate migration and population.

Section 4.2: Data sources and processing

Section 4.2.1: Census and registry lists

Population censuses, which are generally conducted in most countries every ten years, are the primary source of data for population size by age and sex. In some countries, continuous population registries are also maintained. We synthesised population census data by first compiling a list of censuses as documented by the United Nations Statistics Division (UNSD),[47] United Nations Population Division (UNPOP),[48] United Nations Demographic Yearbook (UN DYB),[42] the Integrated Public Use Microdata Series (IPUMS),[49] and the Population Research Center at The University of Texas at Austin.[50]

Section 4.2.2: Data extraction

We extracted age-sex-specific population census data and registry counts from the UN DYB,[42] IPUMS,[49] national statistic websites, and by searching the WorldCat catalog.[51] Occasionally, multiple sources of population counts for a given census or registry-year were available. In these instances, preference was given to registry data rather than census data, data where population counts were reported as de facto (when people are counted based on place of enumeration) over de jure (when people are counted based on place of usual residence), and data reporting population by more granular age and sex groups. In certain cases, only a portion of the total census population was reported with more granular detail for age and sex (with a sample ranging from 1% to 20% of the total census). In these instances, we assumed that the age and sex structure of the sub-sample was identical to that of the total census. Then, we scaled up the sub-sample counts using the total census population counts.

In total, data were extracted for 1374 censuses. Of the censuses extracted, 702 were de facto, 582 were de jure, and in 89 instances we were unable to determine whether the data were de facto or de jure. We were unable to extract some censuses because results were either never or have not yet released by the coordinating organisation. In total, 837 location-years of registry data was extracted for 25 locations.

Any instance of published limitations of the census was also noted, including both limitations identified by media and independent experts in addition to limitations provided by the country's statistical division upon publication. In total, data were outliered from 92 censuses because they were representative of only a subset of the population, utilised questionable methods, or were inconsistent with adjacent data. Some censuses were excluded because countries may have artificially inflated population counts or undercounted minorities for political reasons. Others were excluded because they failed to enumerate the non-white population (for example, Rwanda in 1953 and Burundi in both 1952 and 1958).

Section 4.2.3: Data processing

Population data could be inaccurate due to age misreporting, representativeness of the de facto population, and under/over-enumeration. While identifying censuses, we preferentially utilised raw census and registry population counts. Then, we corrected for the issues mentioned by applying a standardised set of methods.

The first step was to distribute counts of individuals for whom age and/or sex were unknown. The age-sex structure of the remaining data from that particular census was used to distribute the counts of this group of individuals.

The next step was to adjust for age heaping that occurs when, instead of reporting their exact age, individuals report their age as a round number (usually ending in 0 or 5). Population totals were aggregated first to the largest age interval length originally in the data, so each population count was in standardised age groups and the censuses with unusually wide age groups were collapsed to total population by sex only. For each census, the age ratio score (for males and females), sex ratio score, and joint score (JS) were calculated. These measures are explained in detail in the US Census Bureau Population Analysis System documentation.[52]

We used a combination of three previously developed age-heaping methods for this analysis. One of these, the Feeney correction, which is applicable only in census counts with single-year age groups, redistributes people

reporting their age as multiples of five to the eight adjacent single-year age groups, resulting in corrected counts with proportional adjacent age groups that then form a linear progression. The corrected counts are reported in five-year age groups. The Arriaga correction takes input census counts in five-year age groups and smooths them by combining the three nearest ten-year age groups with a second-degree polynomial. The advantage of using this technique instead of other similar methods is that it also smooths the youngest and oldest age groups. The Arriaga strong method averages three consecutive ten-year age groups, which addresses cases with severe age misreporting. These three methods were used in countries outside of the high-income and central Europe, eastern Europe, and central Asia super-regions according to the age group length of the population counts and the JS, as seen below in table G.

Table G. Age heaping corrections

Age group length	Joint score (JS)	Age heaping correction
1	JS <= 20	None
1	JS > 20	Feeney
5	JS <= 20	None
5	20 < JS >= 40	Arriaga
5	JS > 40	Arriaga strong
10	JS <= 20	None
10	JS > 20	Arriaga strong
Total population	Not available	None

After performing a visual inspection, 36 censuses were identified as being incorrectly scored or poor-quality, and these data were not corrected for age heaping. This was predominantly due to sex ratio scores that were higher than normal, but still thought to be a real phenomenon, occurring among the Persian Gulf states, for example. 21 censuses were also identified as exhibiting age heaping but were not corrected due to being in either the high-income or the central Europe, eastern Europe, and central Asia super-regions. After applying age heaping corrections, we aggregated the single-year age group population counts conducted outside of the high-income and the central Europe, eastern Europe, and central Asia super-regions into five-year age groups.

For the third step, we corrected data in cases that were not representative of the de facto population of interest, either due to the coverage of geographical area, subpopulations, or a combination of both. A number of countries that exist in 2023 are combinations of two or more countries that existed in the past, such as Germany. In other countries, semi-autonomous territories have conducted separate censuses, such as Transnistria, and others still have contemporary states that are part of historical aggregates, such as the states that were previously part of Yugoslavia. Censuses for fragmented or semi-autonomous areas often occurred in different years and collected information about different age groups.

We estimated the population of the complete modern geographic unit for which we had a time series of separate censuses from all constituent historical or disputed territories. All available censuses for the constituent parts were collapsed to the most granular set of common age groups. Age-sex-specific populations were interpolated after standardisation using the age-sex-specific annual rate of change to generate annual time series spanning the available census data for each constituent. Finally, for all original census years corresponding to the largest constituent where there was also overlap with the estimated populations from the constituents that were smaller, the populations of all the constituents were summed at the age-sex level to produce a census estimate that was representative of the entire modern geography. This method was utilised in the combination of censuses from East and West Germany, Moldova and Transnistria, Cyprus and the Turkish Republic of Northern Cyprus, Serbia and Kosovo, and Malaysia Peninsular, Sarawak, and Sabah. For modern countries formerly part of Yugoslavia, only historical census data that were reported according to ethnic (rather than geographical) affiliation were able to be extracted. In these cases, the estimated age-sex proportions of the total historical aggregate population from the previous iteration of the Bayesian demographic balancing model were applied to create modern geographies from

the historic census data. These split data were used only for Serbia, as census data for the five other modern countries that were formerly part of Yugoslavia were obtained.

Singapore censuses only contained granular age and sex population counts for residents, but non-residents accounted for a significant portion of the de facto population, while registry data for Singapore residents and non-residents were only available in broad age groups (0–14, 15–64, 65+). Using available empirical death counts for both residents and non-residents, and assuming that both subpopulations have similar mortality rates, the relative age-pattern in the death counts was utilised to scale the resident population from the census data to the total population. These age-sex-specific population counts were then scaled to the broad age group data from the population registries in Singapore.

A fourth step was to correct for improper enumeration. Many censuses suffer from under-enumeration, since it is logistically very difficult to reach every single member of the population except in the smallest and most navigable countries. To address this issue, national statistical offices will often conduct post-enumeration surveys (PES) shortly after the completion of censuses to assess the degree to which individuals were either missed or double-counted. The PES quantifies the overall bias of a given census, and population totals can be adjusted using those results. We searched for PES using the UNSD's list of known or planned PES efforts in the 2000, 2010, and 2020 census rounds. We looked for PES corresponding to any known census by looking through country statistical websites, academic publications, the WorldCat catalog, and data presented by countries during UN Stats symposia on the topic of PES. 157 PES were identified that reported a net under-enumeration percentage, of which 36 reported under-enumeration percentage by age and/or sex. As most extracted census data were unadjusted and a corresponding PES was unable to be identified for many cases, a simple linear regression was used to predict percentage adjustment for all ages and both sexes on the basis of the Socio-demographic Index (SDI) (section 5).

$$totalPctAdjust = \beta_0 + \beta_1 SDI + \varepsilon$$

We used the variance-covariance matrix from this model to simulate 1000 draws of the predicted total percentage adjustment ($totalPctAdjust$) for every possible value of SDI. We incorporated the variance of the 1000 draws simulated from the model into the overall calculation of population uncertainty (section 4.4.4).

Based on observations that over- and under-enumeration varies significantly depending on age and sex, the 36 PES that reported under-enumeration by age and/or sex were used to obtain an under-enumeration global age pattern. In order to address the issue of PES reporting inconsistent age groups, we used DisMod-MR, an age-integrating Bayesian meta-regression tool widely used in GBD processes[53,54] and included SDI as a predictor in the model. The age-sex pattern was then shifted up or down to equal the predicted mean total percentage adjustment and was applied to all census counts where prior adjustment was not identified.

The Bayesian demographic balancing model and the cohort component method of population projection (CCMPP) requires a starting population as an input from which it can project populations forward in time, but most countries did not conduct a census in 1950. Therefore, the last step of data processing was to produce a prior estimate of the population in 1950 in each location in single-year age groups for each sex. In instances where post-processed censuses were available for 1950 with continuous single-year age groups up to the age group 95+, the census was used directly. In all other cases, aggregate age groups were split in the oldest census available with person-years lived (${}_nL_x$) in single-year age intervals from the estimated life table for that location in 1950.[55] Then, this age pattern was smoothed using a local first-degree polynomial with bandwidth equal to 2 if the original age groups were in age interval lengths of 1 or 5 and bandwidth equal to 5 if in larger age groups.[56] If this oldest census was not from 1950, we projected the census backward using an inverse version of CCMPP (assuming zero migration). A description of standard CCMPP can be found in section 4.4.1; backward CCMPP solves for the population at the previous time point rather than projecting to the next time point.

$${}_1N_{x-1}(t) = {}_1N_x\ (t+1) \cdot \frac{{}_1L_{x-1}(t)}{{}_1L_x\ (t)}$$

The equation for the open-ended age group is indeterminate and leaves what we call the upper missing triangle as the backward projection continues further back in time. The missing age groups are estimated using the 1950 ${}_nL_x$ age pattern found in the estimated life tables. In certain locations, as the census counts are projected backward, age misreporting in the older age groups becomes extremely evident and leads to implausible age patterns; we addressed this by collapsing the original census to a lower open-ended age group, thereby ignoring the information for the oldest age groups in the original census. 31 countries used annualised rate of change between the oldest two censuses to back-project the total population, then scaled the back-projected age-specific population to the back-projected total population. In 35 countries, the assumption of zero migration used in backward CCMPP is not appropriate, or the oldest census was not close enough temporally to 1950 to back-project a reasonable age pattern; in these countries we instead used previous GBD population estimates in 1950 as the baseline population. The 1950 populations were used as the prior for the Bayesian demographic balancing model but were input with considerable uncertainty to account for the fact that a census did not occur in 1950 in most countries.

Section 4.3: Model inputs

Section 4.3.1: Migration

The demographic balancing equation has four main components: population, births, deaths, and migration. Except in high-income countries, migration was not measured as well as the other components, thus impacting CCMPP which requires either the net migration proportion or net number of migrations by age, sex, and year. In locations with frequent censuses, we set the prior for net migration to zero in the Bayesian population model (further described below). However, we recognise the model is not able to estimate migration accurately when large migration occurred between censuses or in the years after the last census was conducted. Therefore, we describe our process of replacing the zero prior in these specific types of locations by identifying and extracting migration data below.

For migration, we utilised a refugee stock dataset from the United Nations High Commissioner for Refugees (UNHCR)[57] which included end-of-year (EOY) counts of refugees residing in each country of destination (D), organised by country of origin (O), to calculate net flow of refugees in or out of countries. For example, change in the number of refugees residing in Rwanda in 1994 was calculated by taking the number of refugees in the country at the EOY in 1994 $D_{RWA}(EOY\ 1994)$ and subtracting the number of refugees at the EOY in 1993 $D_{RWA}(EOY\ 1993)$.

$$\Delta D_{RWA}[1994] = D_{RWA}(EOY\ 1994) - D_{RWA}(EOY\ 1993)$$

Likewise, change in the number of refugees originating from Rwanda who had relocated elsewhere in 1994 was calculated by taking the number of refugees originating from Rwanda at the EOY in 1994 $O_{RWA}\ (EOY\ 1994)$ and substracting the number of refugees originating from Rwanda at the EOY in 1993 $O_{RWA}(EOY\ 1993)$.

$$\Delta O_{RWA}[1994] = O_{RWA}(EOY\ 1994) - O_{RWA}(EOY\ 1993)$$

Net refugee migration for Rwanda in 1994 was calculated as:

$$\text{Net refugee migration}[1994] = (\Delta D_{RWA}[1994] - \Delta O_{RWA}[1994])$$

We converted net refugee migration to mid-year migration totals by averaging totals for adjacent years. While the UNHCR dataset also reported totals for internally displaced persons, these types of individuals were excluded from our analysis. In location-years of refugee crises, the total number of migrants were primarily made up of the large influxes or outfluxes of people, replacing the zero prior with the UNHCR data.

There are certain countries, such as Germany, Romania, the United Arab Emirates, and Bahrain, where large non-refugee migrations have taken place too recently to have been captured by the last census, so they were not accounted for in the Bayesian population model. In locations where this was the case, migration data was taken from the statistical office of the European union (EUROSTAT),[58] the Gulf Labour Markets, Migration and Population (GLMM) programme,[59] or national statistics websites.

EUROSTAT was the only source that provided age-sex-specific migration data; the other sources reported total number of migrants over specific time periods. Since age-sex-specific migration is required as an input to CCMPP,

we scaled age-sex patterns of migration to the input net migrant totals. The aggregated EUROSTAT age-sex pattern was used for most locations, but we used the Bayesian demographic balancing model's age-sex pattern for Qatar in places where the majority of migration has occurred among young adult male temporary workers (Saudi Arabia, Bahrain, United Arab Emirates, Oman, and Kuwait). Qatar was chosen because its most recent census was conducted in 2015 and captured this recent uptick in migration. Lastly, given the genocide in Rwanda in 1994, we assumed the migrant age pattern would be dissimilar to the typical migrant age pattern reported in the European Union and the age pattern of migratory workers in Qatar, so we instead used an age pattern that was uniform across age and sex for the country.

Section 4.3.2: Mortality

The GBD 2023 mortality model produced yearly complete period life tables with single-year age groups up to age 95+, as described in section 2.

Section 4.3.3: Fertility

The GBD fertility model produced single-year age group fertility rates between ages 10 and 54 as described in section 3.

Section 4.3.4: Sex ratio at birth

The GBD sex ratio at birth (SRB) model produces yearly sex ratio estimates, which we used to split total livebirths into sex-specific livebirths during CCMPP. See section 4.4.1 for more information.

Section 4.4: Modelling strategy

Section 4.4.1: CCMPP

CCMPP allows projection of population forward in discrete time intervals by age and sex by incorporating mortality, fertility, and migration estimates. Since more information about this method is provided in Preston,[13] we only present the fundamental equation here:

$$ {}_1N_x\ (t+1) = \left[\left({}_1N_{x-1}(t) + \frac{{}_1G_{x-1}[t, t+1]}{2} \right) \cdot \frac{{}_1L_x(t)}{{}_1L_{x-1}(t)} \right] + \frac{{}_1G_{x-1}[t, t+1]}{2} $$

The survivorship ratio, $\frac{{}_1L_x}{{}_1L_{x-1}}$, which is derived from the GBD period life tables, is used to survive any age group (except the youngest and oldest age groups, which are slightly different) forward by one time interval. One key assumption made was that the number of migrants was evenly split, with half migrating during the beginning of the time interval and the other half migrating at the end of the time interval. Although G was defined by Preston as the net flow of migrants during the projection period in the age interval x - 1 to x,[13] we defined ${}_1G_{x-1}$ as the net flow of migrants during the projection period for the cohort initially between age x – 1 and x at the beginning of the projection period because we used the GBD Bayesian demographic balancing model (described below) to estimate migration. Net migration proportions were estimated by this model instead of counts. We defined these proportions as the ratio of net migrants in a cohort to the population at the beginning of the projection period.

$$ {}_1g_x = \frac{{}_1G_x}{{}_1N_x} $$

This equation is for ages above 0 years, as it does not include fertility. Fertility rates are used along with populations sizes in childbearing ages to calculate the age 0 population.

Section 4.4.2: Bayesian demographic balancing model

A Bayesian hierarchical model (popReconstruct) which reconstructs population by age and sex back in time was previously developed by Wheldon and colleagues.[60] Baseline population counts, mortality, fertility, migration, and

sex ratio at birth were simultaneously estimated by the popReconstruct model to reconcile CCMPP's population projections with recent census data.

In Wheldon's version, bias-reduced input values reported by the United Nations were used for the necessary components of population change which included net migration. In our application of the model, we used initial GBD estimates of fertility and mortality which were derived from replicable methods. Since GBD did not produce net migration estimates due to a lack of migration data and difficulties in directly modelling the variable, we modified Wheldon's popReconstruct model to estimate net migration indirectly, consistent with available census data and the input demographic estimates.

Section 4.4.3: Model description

In both popReconstruct and our demographic balancing model, CCMPP is embedded into a Bayesian hierarchical model. To keep the variables consistent with the popReconstruct model, n, g, s, f, and srb were used to symbolise population, the net migration proportion, survivorship ratio, age-specific fertility rate, and sex-ratio at birth, respectively. We indexed the variables by sex (l), single-year age groups (a) and single calendar years (t). Initial parameter values are indicated by an asterisk ($*$).

Level 1: The percentage difference between non-baseline sex-age-year-specific census counts ($n^*_{l,a,t}$) and the corresponding projected sex-age-year-specific populations counts ($n_{l,a,t}$) from CCMPP in Level 2 was modelled. We modelled percentage difference to ensure the variance ($\sigma^2_{n^*_{l,a,t}}$) associated with each census datapoint was estimated on a consistent scale across age groups and locations for a variety of population magnitudes.

$$Level\ 1\text{:}\ \frac{n^*_{l,a,t} - n_{l,a,t}}{n_{l,a,t}} \sim Normal(0, \sigma^2_{n^*_{l,a,t}})$$

Level 2: The model inputs were transformed into projected sex-age-year-specific population counts ($n_{l,a,t}$).

$$Level\ 2\text{:}\ n_{l,a,t} = CCMPP(n_{l,a,t_0}, g_{l,a,t}, s^*_{l,a,t}, f^*_{l,a,t}, srb^*_t)$$

Level 3: The net migration proportion ($g_{l,a,t}$) and baseline population in 1950 (n_{l,a,t_0}) were modelled. Like in level 1, the baseline population was modelled as the percentage difference between the estimated baseline population in 1950 and the input values. To avoid major discontinuities in the age pattern and time series, net migration proportion was modelled as an autoregressive process over age and time. Contrary to the popReconstruct model, we did not also estimate ASFR ($f^*_{l,a,t}$), survival ratios ($s^*_{l,a,t}$) and sex ratio at birth (srb^*_t) because the zero prior for migration makes it difficult to jointly estimate all components.

$$Level\ 3\text{:}\ \frac{n_{l,a,t_0} - n^*_{l,a,t_0}}{n^*_{l,a,t_0}} \sim Normal\left(0, \sigma^2_{n^*_{l,a,t_0}}\right)$$

$$g_{l,a,t} - g^*_{l,a,t} \sim AR1\text{:}\ AR1(\sigma_g, \rho_{g_a}, \rho_{g_t})$$

Level 4: Hyper-priors were defined for the prior distribution on the net migration proportion. We specified greater correlation over time than age for the net migration proportion correlation.

$$Level\ 4\text{:}\ \log(\sigma_g) \sim Normal(-5, 9)$$

$$logit(\rho_{g_a}) \sim Normal(-3, 0.01)$$

$$logit(\rho_{g_t}) \sim Normal(3, 0.01)$$

Rather than estimating one variance term for all census counts, the initial standard deviation for every age-sex-year-specific census datapoint ($\sigma_{n^*_{l,a,t}}$) was set to 0.01 for locations in the high-income super-region, 0.03 for locations in the central Europe, eastern Europe, and central Asia super-region, and 0.05 for locations in the other super-regions. Several issues were addressed by multiplying the standard deviation by scalars in order to adjust the weight of a

datapoint. These issues included: 1) We doubled the standard deviation of the 1950 baseline counts so that the estimated baseline count could change considerably from the input 1950 counts from census processing. 2) Our model was constructed to compare projected population counts to the available census age groups, whether that was single-year age groups or just total population. When a census only had less granular age groups, there were fewer datapoints included, which gave less weight to the census year as a whole. To resolve this, the standard deviation for each census datapoint was divided by the age interval's width, resulting in all censuses being assigned similar weights. 3) Age misreporting among older ages was accounted for by multiplying the standard deviation by a scalar which increased linearly from 1 among 50-year-olds to 3 among 95+-year-olds in the terminal age group. 4) Under-enumeration in the under-5 age group was addressed by multiplying standard deviation of population counts by a linear scalar that increased from 1 for age 5 to 3 for age 0. For the locations not included in the high-income or central Europe, eastern Europe, and central Asia super-regions, non-baseline under-5 population counts were not input for all censuses, making under-5 populations dependent on the fertility estimates, under-5 mortality estimates, and census counts from older age groups.

Older age misreporting seemed to be quite common, demonstrated by the unrealistically large immigration values produced by the model to compensate for the high counts of individuals among the older age groups. This problem was partly addressed by the methods we described above which gave less weight to census counts among the older age groups. In addition, we ran the model multiple times for each location with each version excluding data above a certain maximum age between 55 and 95. Upon completion of each model version for a location, we calculated: 1) mean average percentage error (MAPE) to compare input census data to the estimated population for each datapoint that was included in the model and 2) the weighted mean of the absolute value of the net migration proportions for five-year age groups between 55 and 95 by assigning a weight equal to 1 for age group 55, 2 for age group 60, and so on, capping at a weight of 9 assigned to age group 95+. Then, we selected the version with the highest maximum age that was within 5% of the minimum MAPE value from the various model versions and within 0.005 of the minimum weighted mean net migration proportion for older age groups. By using this method, we were able to balance decreasing migration among the oldest age groups and ensure a good fit to the census data we included in our model.

For each location, we separately fit the Bayesian demographic balancing model from 1950 to 2023 with single-year age groups up to the terminal age group of 95+. CCMPP was the used to derive final population estimates using GBD estimates of mortality, fertility, and sex ratio at birth as well as posterior estimates of baseline population and the net migration proportion.

Section 4.4.4: Population uncertainty

After estimating a complete time series of age-sex-specific population with CCMPP, we estimated population uncertainty using a combination of out-of-sample predictive validity and uncertainty in the adjustment for census completeness (section 4.2.3).

A major cause of uncertainty was related to the PES results and the census completeness model, leading to additional uncertainty in population estimates for all years, since censuses are used to inform population size in both census and non-census years. Using our census completeness model, we calculated the variance of the percentage adjustment for completeness ($Var_{completeness}$) that was associated with each location-year and dependent on the SDI value.

There was additional uncertainty in our population estimates for non-census years which was related to the amount of time to the closest census and GBD estimates of mortality, fertility, and migration. To measure this, we withheld census data in the 143 national locations where there were a minimum of five censuses with age-sex-specific population totals after undergoing post-census processing (section 4.2.3). For each location, the model was fit 20 times using a random subset of one to five of the location's censuses. Percentage error between the held-out census data and the projected posterior population estimates were compiled across locations by the absolute number of years from the closest census. We then fit a linear regression on the root mean squared percentage error ($RMSPE$) as a function of the number of years to the closest census ($YearsToCensus$).

$$RMSPE = \beta_0 + \beta_1 YearsToCensus + \varepsilon_i$$

For each location-year, we then had the predicted variance of the percentage adjustment for completeness ($Var_{completeness}$) based on the specific year's SDI and the predicted out-of-sample percentage error based on the distance from the closest census ($RMSPE$). We combined these sources of uncertainty in variance space.

$$Var_{pop} = Var_{completeness} + RMSPE^2$$

We used the total variance for population to take simulated draws of the population percentage error ($PopPctError$) by location-year.

$$PopPctError \sim Normal(0, Var_{pop})$$

Finally, these percentage errors were used to calculate new population draws which incorporated both PES adjustment uncertainty and also out-of-sample variance associated with estimation errors in the GBD fertility, mortality, and migration estimates and the amount of time to the closest census.

$$Pop_{draw} = Pop_{mean} + (Pop_{mean} * PopPctError_{draw})$$

Section 4.4.5: GBD world population age standard

The GBD world population age standard was used to calculate age-standardised rates presented throughout GBD. In previous GBD years, 2013, 2015, and 2016, specifically, a standard population age structure was generated by taking the non-weighted mean of the 2010 to 2035 age-specific proportional distributions for national locations reported by the UNPD World Population Prospects 2012 revision. Beginning in GBD 2017, we used the non-weighted mean of the GBD year's age-specific proportional distributions for national locations with populations greater than 5 million in the GBD year to update the world population age standard.

Section 5: Socio-demographic Index (SDI) analysis[2]

Section 5.1: Overview

The Socio-demographic Index (SDI) is a summary indicator created to get at the background social and economic conditions that shape health outcomes in a given location. Introduced in GBD 2015,[39] and further refined in GBD 2019,[16] it represents an update to the Human Development Index (HDI), while also providing an alternative to outdated development language.

SDI includes three components. It includes an economic indicator, lag-distributed income (LDI)[16] per capita, as well as two demographic indicators, total fertility rate under the age of 25 (TFU25) and mean educational attainment for those aged 15 and older (EDU15+). TFU25 is intended to serve as an indicator of the status of women. Beginning in GBD 2017, we began using TFU25 instead of total fertility rate (TFR), because we found it to be a better proxy for women's status.[41]

To create the index, we rescaled each component to obtain a value between 0 and 1. We set this scale using selected health indicators. At the low end of this scale, 0 represents the minimum level of each covariate input past which selected health outcomes do not get worse. At the high end of this scale, 1 represents the maximum level of each covariate input past which selected health outcomes do not improve. Finally, we calculated SDI by taking the geometric mean of those rescaled values.

Section 5.2: SDI calculation

Further refinements to the method for calculating SDI have been implemented since the original development in the 2015 cycle. Beginning in GBD 2017, we decided to use TFU25 instead of the TFR component. The rationale for this was to attempt to better capture women's social status, given that it covers ages when women tend to enter the workforce and pursue further educational opportunities. It is also important that there has been a consistent decline in TFU25 over time in highly developed countries. In contrast, there have been rebounds in TFR driven by increasing fertility in older ages. The concordance correlation coefficient was 0.981 between SDI using the GBD 2016 method and the updated GBD 2017 method.[40,55]

In order to improve the stability of the interpretation of SDI over time, we switched from relative index scales to absolute index scales during GBD 2016 when we noticed the introduction of subnational units led to stretched empirical minima and maxima.[40] The minima and maxima of the scales were selected by looking at the relationships between each of the inputs and life expectancy at birth and under-5 mortality, then identifying points of limiting returns at both high and low values, if they occurred prior to theoretical limits (eg, EDU15+ of 0).

An index score of 0 therefore represents the point at which decreasing each covariate does not worsen selected health outcomes and an index score of 1 represents the level at which increasing the level of each covariate does not improve selected health outcomes. The means that a location with an SDI of 1 would have the theoretical maximum level of development relevant to these selected health outcomes. A location with an SDI of 0, on the other hand, would have the theoretical minimum level of development relevant to these selected health outcomes.

Using scales defining the upper and lower bound for each input, we computed the index scores underlying SDI as follows:

$$I_{Cly} = \frac{(C_{ly} - C_{low})}{(C_{high} - C_{low})}$$

Where I_{cly} – the index for covariate *C*, location *l*, and year *y* – is equal to the quotient of the difference between the value of that covariate in that location-year and the lower bound of the covariate and the difference between the upper and lower bounds for that covariate. If the values of input covariates fell above the upper bounds or below the lower bounds, they were set equal to the respective upper or lower bounds. The index value for TFU25 was computed as $1 - I_{TFU25ly}$, to account for the negative relationship between TFU25 and development, and thus between TFU25 and index score. For GBD 2023, SDI was computed for 934 national and subnational locations spanning the time period 1950 to 2023.

The composite SDI was calculated as the geometric mean of these three indices (LDI, TFU25, EDU15+) for a given location-year. The cutoff values used to determine quintiles for analysis were then computed using country-level estimates of SDI for the year 2023, excluding countries with populations less than 200 million. Annual SDI values can be found in appendix 2 tables S6A–6C.

Section 6: Additional methods[2]

Section 6.1: Correlation estimation

To estimate correlation, we used the Pearson correlation coefficient, defined by the function below:[61]

$$\hat{\rho} = \frac{\sum_{i=1}^{n} w_i (x_i - \bar{x})(y_i - \bar{y})}{\sqrt{\sum_{i-1}^{n} w_i (x_i - \bar{x})^2} \sqrt{\sum_{i-1}^{n} w_i (y_i - \bar{y})^2}}$$

Where

w_i are the weights, if specified, or $w_i = 1$ if weights are not specified

$\bar{x} = (\sum w_i x_i)/(\sum w_i)$ is the mean of x

$\bar{y}$ is similarly defined

Section 6.2: Annualised rates of change

We calculated annualised rates of change by taking the log of ${}_5q_0$ in the target year $(y + t)$, dividing that by the ${}_5q_0$ at the baseline year y, and dividing that by the difference in years between the target and baseline years (t), using the following equation:

$$aroc_{{}_5q_0} = \frac{ln\left(\frac{{}_5q_{0\,y+t}}{{}_5q_{0\,y}}\right)}{t}$$

Section 6.3: Gross domestic product (GDP) and lag-distributed income

We used five gross domestic product (GDP) per capita series to calculate GDP per capita: World Bank World Development Indicators, International Monetary Fund World Economic Outlook report, Angus Maddison's research homepage at the University of Groningen Department of Economics, the Penn World Table, and the United Nations Statistics Division National Accounts Main Aggregates Database .[62–66] We imputed each series separately using growth regressions and then averaged the four series to make a unified GDP per capita series for GBD. We used mixed effects regression with region-specific random effects to interpolate or extrapolate any country data that were missing from all four series. These methods are described in full in by James and colleagues.[67]

Lag-distributed income (LDI) per capita is a moving average transformation of GDP per capita. We calculated LDI, a ten-year lagged average of GDP, with the formula below:

$$LDI_{pc,t} = \frac{1}{5.5}\left(GDPpc_t + \sum_{i=1}^{9} GDPpc_{t-i} \cdot \left(1 - \frac{i}{10}\right)\right)$$

where LDI at time t is a function of current GDP (at t) and the previous nine years of GDP with an inverse moving weight and a normalisation factor (the sum of all weights).

Section 6.4: Educational attainment covariate

We based our estimates of average years of education on a collection of 6072 censuses and household surveys. The approach we used to estimate educational attainment, which we describe below, has been published[68] and draws upon previously established methods.[13,69] Educational data sources used have information about the distribution of educational attainment and must be granular by country or territory, year, sex, and five-year or ten-year age groups. Some sources only provided education data for multi-year bins (e.g., the percentage of the population with between two and five years of completed schooling), which were probabilistically split into single-year proportions using a previously published crosswalk model.[70] Data were top coded to 18 years of education.

We then adjusted for systematic biases between data providers in a regional and location-specific fashion. We used a mixed-effects regression model with random effects for data provider and nested random effects for data provider within country to adjust the quantity of interest, either proportion of the population with no education or the mean years of educational attainment. Next, we used age-cohort imputation[68] to carry educational attainment in observed cohorts forward through time, since education levels are relatively constant after age 25. To model the changes by age within cohorts, we used data from all available cohorts with multiple observations at or after age 25. For datapoints from cohorts aged 25 or older, we extrapolated the data forward and backward in time so all year-age combinations in that cohort contained that data (e.g., a datapoint for a cohort aged 40–44 in 1995 was projected forward for 45–49-year-olds in 2000, 50–54-year-olds in 2005, etc. and backward for 35–39-year-olds in 1990, 30–34-year-olds in 1985, etc.). The details of both the adjustment and cohort extrapolation models are described elsewhere.[68]

After adjustment and imputation, GPR was used to ensure final model results were consistent with input data and to incorporate model and data uncertainty to produce uncertainty intervals. This allowed us to estimate a complete single-year series of educational attainment from 1950 through 2021 by age, sex, and location. GPR assumes that the trend in the underlying data follows a Gaussian process, which is defined using a mean function m(·) and a covariance function Cov(·). To define the mean function, we calculated the mean level of educational attainment of the country-age-year-specific population, $Edu_{c,a,s,t}$, using the following formula:

$$logit\left(\frac{Edu_{c,a,s,t}}{Edu_{max_a}}\right) = \beta_{s,r} + \delta_{s,r}\,Year + I_{s,r} + \propto_{c,a,s}$$

$\alpha_{c,a,s} \sim N(0, \sigma_\alpha^2)$

where:
c is location, a is age, s is sex, t is time

Edu_{max_a} is the maximum mean educational attainment for each age group, defined as 3 for ages 5-9, 8 for ages 10-14, 13 for ages 15-19, and 18 for all age groups 20-24 and up

$\beta_{s,r}$ is a sex- and region-specific intercept

$\delta_{s,r}$ captures the linear secular trend for each sex and region

$I_{s,r}$ is a natural spline on age to capture the non-linear age pattern by sex and region, with knots at 45 and 65 years of age

$\propto_{c,s}$ is a country-sex-specific random intercept.

The covariance function of the model was derived using a Matérn covariance function, consistent with prior applications of GPR:

$$M(y, y') = \sigma^2 \frac{2^{1-\nu}}{\Gamma(\nu)} \left(\frac{d(y, y')\sqrt{2\nu}}{\iota} \right)^{\nu} \kappa_{\nu} \left(\frac{d(y, y')\sqrt{2\nu}}{\iota} \right)$$

Where:

$d(\cdot)$ is a distance function

σ^2 is the marginal variance

ν is a smoothness hyper parameter defining the differentiability of the function

ι is a link-scale parameter approximately equivalent to the number of years at which two points are no longer correlated

κ_{ν} is the Bessel function

$\Gamma(\cdot)$ is the gamma function

Similar to previous applications of GPR, we approximated σ_p^2 as the super-region and sex-specific residual from the mean function, with ν set to 2 and l to 40, to reflect the inherent smoothness of educational attainment trends over time.

Lastly, we used GPR to smooth the age-period model residuals. This step allowed us to account for uncertainty in each datapoint and combine data and model uncertainty to estimate UIs. Final covariates are aggregated to the age-sex of interest.

Section 7: References

1 Stevens GA, Alkema L, Black RE, *et al.* Guidelines for Accurate and Transparent Health Estimates Reporting: the GATHER statement. *Lancet* 2016; **388**: e19–23.

2 GBD 2021 Demographics Collaborators. Global age-sex-specific mortality, life expectancy, and population estimates in 204 countries and territories and 811 subnational locations, 1950-2021, and the impact of the COVID-19 pandemic: a comprehensive demographic analysis for the Global Burden of Disease Study 2021. *Lancet* 2024; **403**: 1989–2056.

3 GBD 2021 Fertility and Forecasting Collaborators. Global fertility in 204 countries and territories, 1950-2021, with forecasts to 2100: a comprehensive demographic analysis for the Global Burden of Disease Study 2021. *Lancet* 2024; **403**: 2057–99.

4 Rajaratnam JK, Tran LN, Lopez AD, Murray CJL. Measuring under-five mortality: validation of new low-cost methods. *PLOS Med* 2010; **7**: e1000253.

5 Obermeyer Z, Rajaratnam JK, Park CH, *et al.* Measuring adult mortality using sibling survival: a new analytical method and new results for 44 countries, 1974–2006. *PLoS Med* 2010; **7**: e1000260.

6 Gakidou E, King G. Death by survey: estimating adult mortality without selection bias from sibling survival data. *Demography* 2006; **43**: 569–85.

7 Masquelier B. Adult mortality from sibling survival data: a reappraisal of selection biases. *Demography* 2013; **50**: 207–28.

8 Rogers RG, Crimmins EM, editors. International handbook of adult mortality. Springer, 2011.

9 Hsu A, Zheng P, Maass K, Aravkin A, Ali S. pyDisagg: Dissaggregation under Generalized Proportionality Assumptions. 2025; published online Jan 13. DOI:10.5281/zenodo.14641582.

10 Murray CJL, Rajaratnam JK, Marcus J, Laakso T, Lopez AD. What can we conclude from death registration? Improved methods for evaluating completeness. *PLoS Med* 2010; **7**: e1000262.

11 Hill K. Estimating census and death registration completeness. *Asian Pac Popul Forum* 1987; **1**: 8–13, 23–4.

12 Brass W, Coale AJ. Methods of analysis and estimation. In: Smith DP, Keyfitz N, eds. Mathematical Demography: Selected Papers. Berlin, Heidelberg: Springer, 1977: 307–13.

13 Preston SH. The changing relation between mortality and level of economic development. *Popul Stud* 1975; **29**: 231–48.

14 Preston S, Coale AJ, Trussell J, Weinstein M. Estimating the completeness of reporting of adult deaths in populations that are approximately stable. *Popul Index* 1980; **46**: 179–202.

15 Bennett NG, Horiuchi S. Estimating the completeness of death registration in a closed population. *Popul Index* 1981; **47**: 207–21.

16 GBD 2019 Demographics Collaborators. Global age-sex-specific fertility, mortality, healthy life expectancy (HALE), and population estimates in 204 countries and territories, 1950-2019: a comprehensive demographic analysis for the Global Burden of Disease Study 2019. *Lancet Lond Engl* 2020; **396**: 1160–203.

17 Nelder JA, Wedderburn RWM. Generalized linear models. *J R Stat Soc Ser Gen* 1972; **135**: 370–84.

18 de Boor C. A practical guide to splines. New York, NY, 1978 https://link.springer.com/book/9780387953663 (accessed Jan 13, 2025).

19 Friedman JH. Multivariate adaptive regression splines. *Ann Stat* 1991; **19**: 1–67.

20 Hofmann T, Schölkopf B, Smola AJ. Kernel methods in machine learning. *Ann Stat* 2008; **36**: 1171–220.

21 Buhmann MD. Radial basis functions. *Acta Numer* 2000; **9**: 1–38.

22 Rasmussen CE, Williams CKI. Gaussian processes for machine learning. The MIT Press, 2005 DOI:10.7551/mitpress/3206.001.0001.

23 Diggle PJ, Tawn JA, Moyeed RA. Model-based geostatistics. *J R Stat Soc Ser C Appl Stat* 1998; **47**: 299–350.

24 Airola A, Pahikkala T. Fast kronecker product kernel methods via generalized vec trick. *IEEE Trans Neural Netw Learn Syst* 2018; **29**: 3374–87.

25 GBD 2023 Disease and Injury and Risk Factor Collaborators.).

26 Slingo J, Palmer T. Uncertainty in weather and climate prediction. *Philos Trans R Soc Math Phys Eng Sci* 2011; **369**: 4751–67.

27 Themnér L. UCDP/PRIO Armed Conflict Dataset codebook version 18.1. Uppsala Conflict Data Program (UCDP) and Center for the Study of Civil Wars - International Peace Research Institute (PRIO), 2012.

28 Obermeyer Z, Murray CJL, Gakidou E. Fifty years of violent war deaths from Vietnam to Bosnia: analysis of data from the world health survey programme. *BMJ* 2008; **336**: 1482–6.

29 Leitenberg M. Rwanda, 1994: International incompetence produces genocide. *Peacekeeping Int Relat* 1994; **23**: 6.

30 Jarvis C. The rise and fall of Albania's pyramid schemes. *Finance Dev* 2000; **37**.

31 International Organization for Migration. Missing Migrants Project. 2025. https://missingmigrants.iom.int/ (accessed May 19, 2021).

32 Centre for Research on the Epidemiology of Disaster (CRED). EM-DAT: The OFDA/CRED International Disaster Database. Brussels: Catholic University of Leuven.

33 Jisheng Y, Friedman E, Guo J, Mosher S. Tombstone: the great Chinese famine, 1958-1962. New York: Farrar, Straus and Giroux (Macmillan), 2012.

34 Inc G, Berger D. Bacterial meningitis: global status: 2017 edition. GIDEON Informatics Inc, 2017.

35 Inc G, Berger D. Cholera: global status: 2017 edition. GIDEON Informatics Inc, 2017.

36 GBD 2021 Causes of Death Collaborators. Global burden of 288 causes of death and life expectancy decomposition in 204 countries and territories and 811 subnational locations, 1990-2021: a systematic analysis for the Global Burden of Disease Study 2021. *Lancet* 2024; **403**: 2100–32.

37 Iraq Body Count. Documented civilian deaths from violence. 2024. https://www.iraqbodycount.org/ (accessed Sept 9, 2024).

38 Hagopian A, Flaxman AD, Takaro TK, *et al.* Mortality in Iraq associated with the 2003-2011 war and occupation: findings from a national cluster sample survey by the university collaborative Iraq Mortality Study. *PLoS Med* 2013; **10**: e1001533.

39 GBD 2015 Mortality and Causes of Death Collaborators. Global, regional, and national life expectancy, all-cause mortality, and cause-specific mortality for 249 causes of death, 1980-2015: a systematic analysis for the Global Burden of Disease Study 2015. *Lancet Lond Engl* 2016; **388**: 1459–544.

40 GBD 2016 Mortality Collaborators. Global, regional, and national under-5 mortality, adult mortality, age-specific mortality, and life expectancy, 1970-2016: a systematic analysis for the Global Burden of Disease Study 2016. *Lancet Lond Engl* 2017; **390**: 1084–150.

41 GBD 2017 Population and Fertility Collaborators. Population and fertility by age and sex for 195 countries and territories, 1950-2017: a systematic analysis for the Global Burden of Disease Study 2017. *Lancet Lond Engl* 2018; **392**: 1995–2051.

42 United Nations Department of Economic and Social Affairs. United Nations Demographic Yearbook. New York: United Nations, 2020 https://unstats.un.org/unsd/demographic-social/products/dyb/dyb_2020/.

43 Max Planck Institute for Demographic Research (Germany), Vienna Institute of Demography (Austria). Human Fertility Collection. www.fertilitydata.org (accessed July 6, 2021).

44 Max Planck Institute for Demographic Research (Germany), Vienna Institute of Demography (Austria). Human Fertility Database. www.fertilitydata.org (accessed Sept 13, 2022).

45 World Health Organization. WHO Mortality Database. https://www.who.int/data/data-collection-tools/who-mortality-database (accessed Sept 1, 2023).

46 Preston S, Heuveline P, Guillot M. Demography: measuring and modeling population processes. Malden, MA: Blackwell Publishers, 2001.

47 United Nations Department of Economic and Social Affairs Statistics Division. Welcome to UNSD. 2020. https://unstats.un.org/home/ (accessed Sept 10, 2023).

48 United Nations Department of Economic and Social Affairs. Population. http://www.un.org/en/development/desa/population/ (accessed Sept 10, 2023).

49 IPUMS USA. U.S. census data for social, economic, and health research. 2019. https://usa.ipums.org/usa/ (accessed Sept 10, 2023).

50 The University of Texas at Austin College of Liberal Arts. Welcome to the Population Research Center (PRC). 2020. https://liberalarts.utexas.edu/prc/ (accessed Sept 10, 2023).

51 WorldCat. WorldCat. https://www.worldcat.org/ (accessed Sept 10, 2023).

52 Arriaga EE, Johnson PD, Jamison E. Population analysis with microcomputers - Volume 1 Presentation of Techniques. 1994; published online Nov. https://www2.census.gov/software/pas/documentation/pamvi-archive.pdf.

53 GBD 2019 Diseases and Injuries Collaborators. Global burden of 369 diseases and injuries in 204 countries and territories, 1990-2019: a systematic analysis for the Global Burden of Disease Study 2019. *Lancet Lond Engl* 2020; **396**: 1204–22.

54 Flaxman AD, Vos T, Murray CJL, editors. An Integrative Metaregression Framework for Descriptive Epidemiology, 1st edition. Seattle: University of Washington Press, 2015.

55 GBD 2017 Mortality Collaborators. Global, regional, and national age-sex-specific mortality and life expectancy, 1950–2017: a systematic analysis for the Global Burden of Disease Study 2017. *The Lancet* 2018; **392**: 1684–735.

56 Wand M, src/d*) CM (LINPACK routines in, updates) BR (R port and. KernSmooth: Functions for Kernel Smoothing Supporting Wand & Jones (1995). 2024; published online May 17. https://cran.r-project.org/web/packages/KernSmooth/index.html (accessed Sept 9, 2024).

57 UNCHR. UNHCR - The UN Refugee Agency. 2001; published online 2020. http://www.unhcr.org/en-us/ (accessed Sept 10, 2023).

58 European Commission. EUROSTAT: your key to European statistics. 2020. http://ec.europa.eu/eurostat (accessed Sept 10, 2023).

59 European University Institute and Gulf Research Center. Gulf Labour Markets, Migration, and Population (GLMM) Programme. GLMM. 2020. http://gulfmigration.eu/ (accessed Sept 10, 2023).

60 Wheldon MC, Raftery AE, Clark SJ, Gerland P. Reconstructing past populations with uncertainty from fragmentary data. *J Am Stat Assoc* 2013; **108**: 96–110.

61 Stata:Corp. Stata: release 13. College Station, TX: StataCorp LP, 2013.

62 Groningen Growth and Development Centre: Faculty of Economics and Business. The Database | Penn World Table version 10.0. https://www.rug.nl/ggdc/productivity/pwt/ (accessed Feb 4, 2022).

63 The World Bank. DataBank | The World Bank. http://databank.worldbank.org/data/home.aspx (accessed Feb 4, 2022).

64 International Monetary Fund. World Economic Outlook, April 2022: War Sets Back the Global Recovery. International Monetary Fund. https://www.imf.org/en/Publications/WEO/Issues/2022/04/19/world-economic-outlook-april-2022 (accessed June 8, 2022).

65 Groningen Growth and Development Centre: Faculty of Economics and Business. Maddison Project Database 2020. https://www.rug.nl/ggdc/historicaldevelopment/maddison/releases/maddison-project-database-2020 (accessed Feb 4, 2022).

66 United Nations Statistics Division - National Health Accounts. National Accounts Main Aggregates Database. https://unstats.un.org/unsd/snaama/ (accessed Feb 4, 2022).

67 James SL, Gubbins P, Murray CJ, Gakidou E. Developing a comprehensive time series of GDP per capita for 210 countries from 1950 to 2015. *Popul Health Metr* 2012; **10**: 12.

68 Friedman J, York H, Graetz N, *et al.* Measuring and forecasting progress towards the education-related SDG targets. *Nature* 2020; **580**: 636–9.

69 Gakidou E, Cowling K, Lozano R, Murray CJL. Increased educational attainment and its effect on child mortality in 175 countries between 1970 and 2009: a systematic analysis. *Lancet Lond Engl* 2010; **376**: 959–74.

70 Friedman J, Graetz N, Gakidou E. Improving the estimation of educational attainment: New methods for assessing average years of schooling from binned data. *PLoS ONE* 2018; **13**: e0208019.

Section 8: Tables and figures

Appendix 1 Figure S1: Detailed mortality flowchart for GBD 2023 with direct estimation

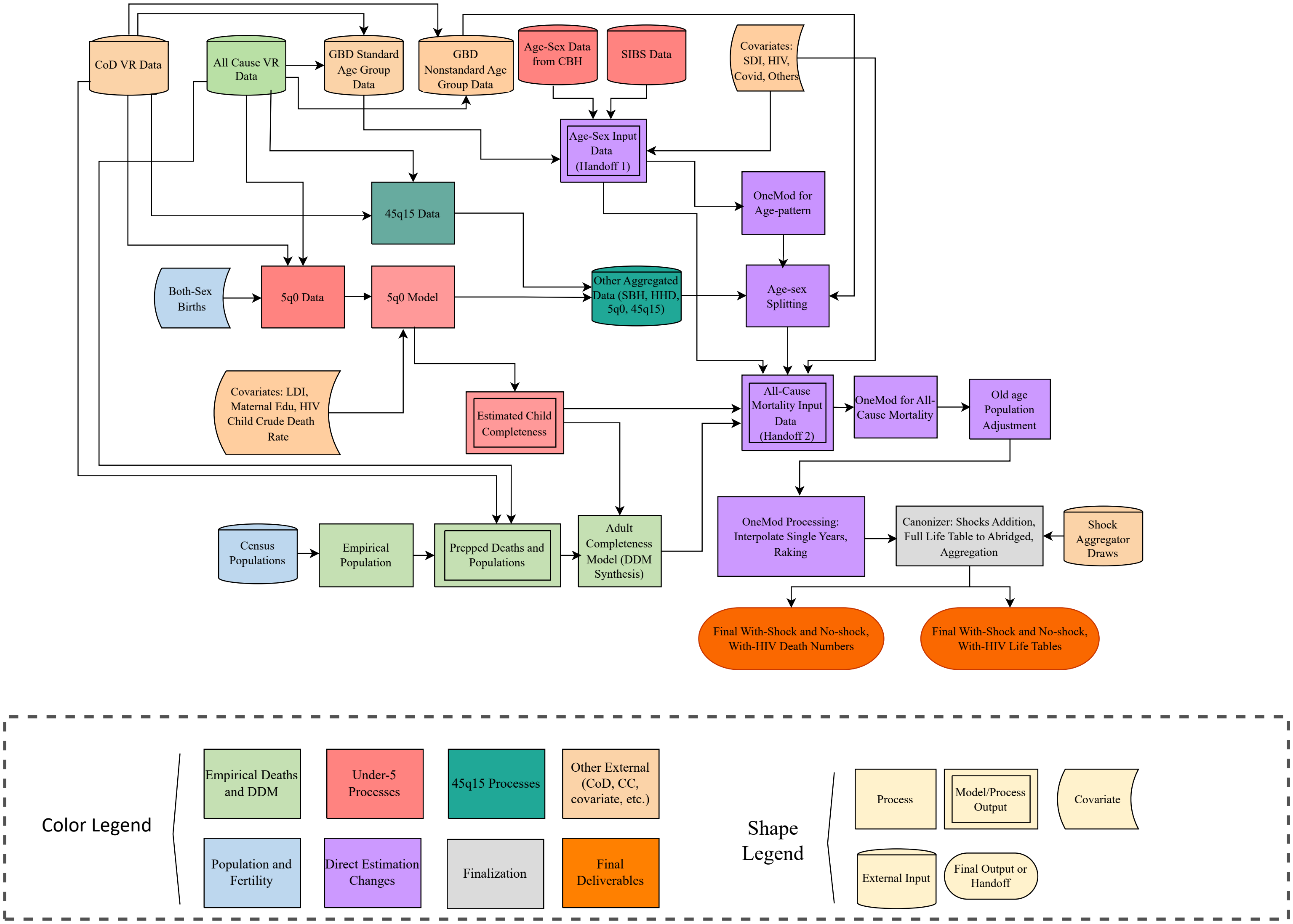

Appendix Figure S2: Estimated completeness of death registration, 1950–2023

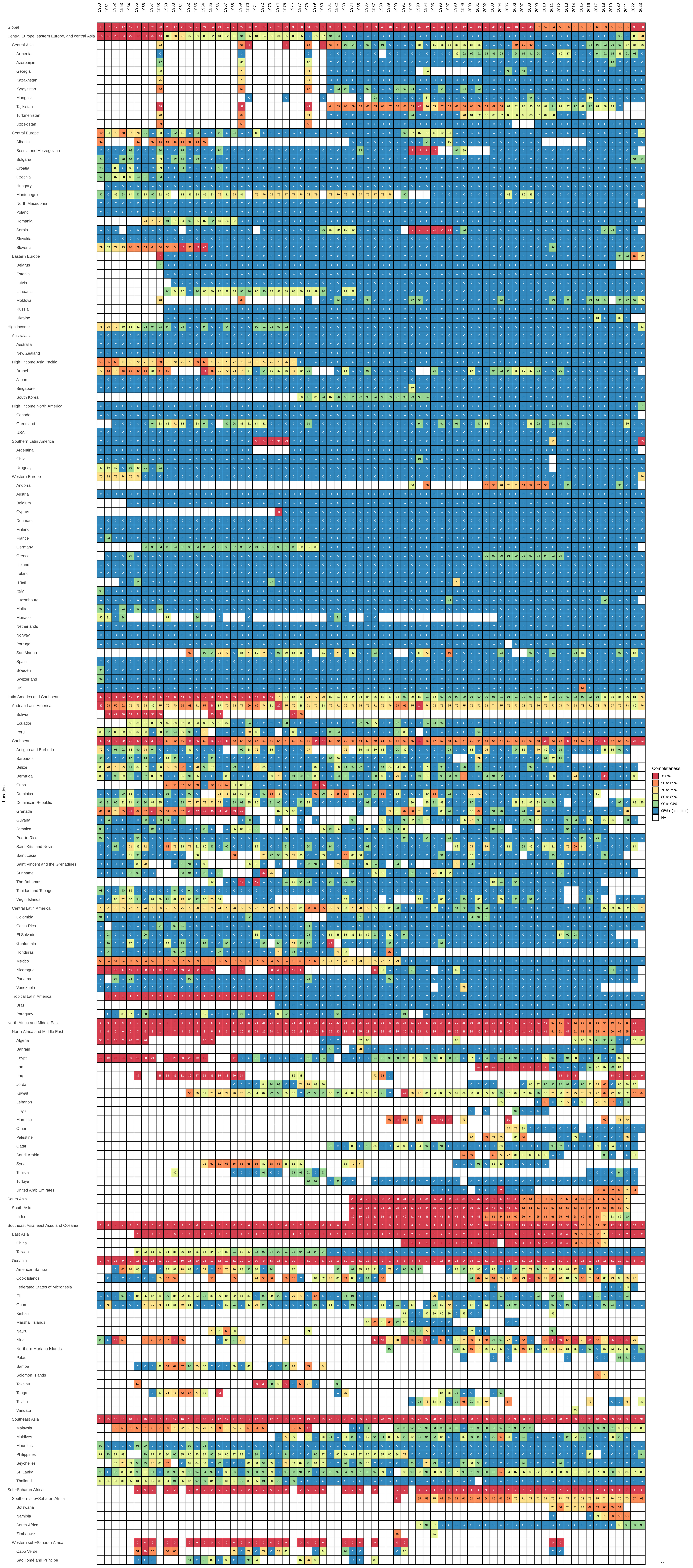

Appendix 1 Table S1: GBD location hierarchy with levels

Location	Level
Global	0
Central Europe, eastern Europe, and central Asia	1
Central Asia	2
Armenia	3
Azerbaijan	3
Georgia	3
Kazakhstan	3
Kyrgyzstan	3
Mongolia	3
Tajikistan	3
Turkmenistan	3
Uzbekistan	3
Central Europe	2
Albania	3
Bosnia and Herzegovina	3
Bulgaria	3
Croatia	3
Czechia	3
Hungary	3
Montenegro	3
North Macedonia	3
Poland	3
Romania	3
Serbia	3
Slovakia	3
Slovenia	3
Eastern Europe	2
Belarus	3
Estonia	3
Latvia	3
Lithuania	3
Moldova	3
Russia	3
Ukraine	3
High income	1
Australasia	2
Australia	3
New Zealand	3
High-income Asia Pacific	2
Brunei	3

Japan	3
Aichi	4
Akita	4
Aomori	4
Chiba	4
Ehime	4
Fukui	4
Fukuoka	4
Fukushima	4
Gifu	4
Gunma	4
Hiroshima	4
Hokkaidō	4
Hyōgo	4
Ibaraki	4
Ishikawa	4
Iwate	4
Kagawa	4
Kagoshima	4
Kanagawa	4
Kōchi	4
Kumamoto	4
Kyōto	4
Mie	4
Miyagi	4
Miyazaki	4
Nagano	4
Nagasaki	4
Nara	4
Niigata	4
Ōita	4
Okayama	4
Okinawa	4
Ōsaka	4
Saga	4
Saitama	4
Shiga	4
Shimane	4
Shizuoka	4
Tochigi	4
Tokushima	4
Tōkyō	4

Tottori	4
Toyama	4
Wakayama	4
Yamagata	4
Yamaguchi	4
Yamanashi	4
Singapore	3
South Korea	3
High-income North America	2
Canada	3
Greenland	3
USA	3
Alabama	4
Alaska	4
Arizona	4
Arkansas	4
California	4
Colorado	4
Connecticut	4
Delaware	4
Florida	4
Georgia	4
Hawaii	4
Idaho	4
Illinois	4
Indiana	4
Iowa	4
Kansas	4
Kentucky	4
Louisiana	4
Maine	4
Maryland	4
Massachusetts	4
Michigan	4
Minnesota	4
Mississippi	4
Missouri	4
Montana	4
Nebraska	4
Nevada	4
New Hampshire	4
New Jersey	4

New Mexico	4
New York	4
North Carolina	4
North Dakota	4
Ohio	4
Oklahoma	4
Oregon	4
Pennsylvania	4
Rhode Island	4
South Carolina	4
South Dakota	4
Tennessee	4
Texas	4
Utah	4
Vermont	4
Virginia	4
Washington	4
Washington, DC	4
West Virginia	4
Wisconsin	4
Wyoming	4
Southern Latin America	2
Argentina	3
Chile	3
Uruguay	3
Western Europe	2
Andorra	3
Austria	3
Belgium	3
Cyprus	3
Denmark	3
Finland	3
France	3
Germany	3
Greece	3
Iceland	3
Ireland	3
Israel	3
Italy	3
Luxembourg	3
Malta	3
Monaco	3

Netherlands	3
Norway	3
Agder	4
Innlandet	4
Møre og Romsdal	4
Nordland	4
Oslo	4
Rogaland	4
Troms og Finnmark	4
Trøndelag	4
Vestfold og Telemark	4
Vestland	4
Viken	4
Portugal	3
San Marino	3
Spain	3
Sweden	3
Switzerland	3
UK	3
England	4
East Midlands	5
East of England	5
Greater London	5
North East England	5
North West England	5
South East England	5
South West England	5
West Midlands	5
Yorkshire and the Humber	5
Northern Ireland	4
Scotland	4
Wales	4
Latin America and Caribbean	1
Andean Latin America	2
Bolivia	3
Ecuador	3
Peru	3
Caribbean	2
Antigua and Barbuda	3
Barbados	3
Belize	3
Bermuda	3

Cuba	3
Dominica	3
Dominican Republic	3
Grenada	3
Guyana	3
Haiti	3
Jamaica	3
Puerto Rico	3
Saint Kitts and Nevis	3
Saint Lucia	3
Saint Vincent and the Grenadines	3
Suriname	3
The Bahamas	3
Trinidad and Tobago	3
Virgin Islands	3
Central Latin America	2
Colombia	3
Costa Rica	3
El Salvador	3
Guatemala	3
Honduras	3
Mexico	3
Aguascalientes	4
Baja California	4
Baja California Sur	4
Campeche	4
Chiapas	4
Chihuahua	4
Coahuila	4
Colima	4
Durango	4
Guanajuato	4
Guerrero	4
Hidalgo	4
Jalisco	4
México	4
Mexico City	4
Michoacán de Ocampo	4
Morelos	4
Nayarit	4
Nuevo León	4
Oaxaca	4

Puebla	4
Querétaro	4
Quintana Roo	4
San Luis Potosí	4
Sinaloa	4
Sonora	4
Tabasco	4
Tamaulipas	4
Tlaxcala	4
Veracruz de Ignacio de la Llave	4
Yucatán	4
Zacatecas	4
Nicaragua	3
Panama	3
Venezuela	3
Tropical Latin America	2
Brazil	3
Acre	4
Alagoas	4
Amapá	4
Amazonas	4
Bahia	4
Ceará	4
Distrito Federal	4
Espírito Santo	4
Goiás	4
Maranhão	4
Mato Grosso	4
Mato Grosso do Sul	4
Minas Gerais	4
Pará	4
Paraíba	4
Paraná	4
Pernambuco	4
Piauí	4
Rio de Janeiro	4
Rio Grande do Norte	4
Rio Grande do Sul	4
Rondônia	4
Roraima	4
Santa Catarina	4
São Paulo	4

Sergipe	4
Tocantins	4
Paraguay	3
North Africa and Middle East	1
North Africa and Middle East	2
Afghanistan	3
Algeria	3
Bahrain	3
Egypt	3
Iran	3
Alborz	4
Ardebil	4
Bushehr	4
Chahar Mahaal and Bakhtiari	4
East Azarbayejan	4
Fars	4
Gilan	4
Golestan	4
Hamadan	4
Hormozgan	4
Ilam	4
Isfahan	4
Kerman	4
Kermanshah	4
Khorasan-e-Razavi	4
Khuzestan	4
Kohgiluyeh and Boyer-Ahmad	4
Kurdistan	4
Lorestan	4
Markazi	4
Mazandaran	4
North Khorasan	4
Qazvin	4
Qom	4
Semnan	4
Sistan and Baluchistan	4
South Khorasan	4
Tehran	4
West Azarbayejan	4
Yazd	4
Zanjan	4
Iraq	3

Jordan	3
Kuwait	3
Lebanon	3
Libya	3
Morocco	3
Oman	3
Palestine	3
Qatar	3
Saudi Arabia	3
Sudan	3
Syria	3
Tunisia	3
Türkiye	3
United Arab Emirates	3
Yemen	3
South Asia	1
South Asia	2
Bangladesh	3
Bhutan	3
India	3
Andhra Pradesh	4
Arunachal Pradesh	4
Assam	4
Bihar	4
Chhattisgarh	4
Delhi	4
Goa	4
Gujarat	4
Haryana	4
Himachal Pradesh	4
Jammu & Kashmir and Ladakh	4
Jharkhand	4
Karnataka	4
Kerala	4
Madhya Pradesh	4
Maharashtra	4
Manipur	4
Meghalaya	4
Mizoram	4
Nagaland	4
Odisha	4
Other Union Territories	4

Punjab	4
Rajasthan	4
Sikkim	4
Tamil Nadu	4
Telangana	4
Tripura	4
Uttar Pradesh	4
Uttarakhand	4
West Bengal	4
Nepal	3
Pakistan	3
Azad Jammu & Kashmir	4
Balochistan	4
Gilgit-Baltistan	4
Islamabad Capital Territory	4
Khyber Pakhtunkhwa	4
Punjab	4
Sindh	4
Southeast Asia, east Asia, and Oceania	1
East Asia	2
China	3
North Korea	3
Taiwan*	3
Oceania	2
American Samoa	3
Cook Islands	3
Federated States of Micronesia	3
Fiji	3
Guam	3
Kiribati	3
Marshall Islands	3
Nauru	3
Niue	3
Northern Mariana Islands	3
Palau	3
Papua New Guinea	3
Samoa	3
Solomon Islands	3
Tokelau	3
Tonga	3
Tuvalu	3
Vanuatu	3

Southeast Asia	2
Cambodia	3
Indonesia	3
Aceh	4
Bali	4
Bangka-Belitung Islands	4
Banten	4
Bengkulu	4
Gorontalo	4
Jakarta	4
Jambi	4
West Java	4
Central Java	4
East Java	4
West Kalimantan	4
South Kalimantan	4
Central Kalimantan	4
East Kalimantan	4
North Kalimantan	4
Riau Islands	4
Lampung	4
Maluku	4
North Maluku	4
West Nusa Tenggara	4
East Nusa Tenggara	4
Papua	4
West Papua	4
Riau	4
West Sulawesi	4
South Sulawesi	4
Central Sulawesi	4
Southeast Sulawesi	4
North Sulawesi	4
West Sumatra	4
South Sumatra	4
North Sumatra	4
Yogyakarta	4
Laos	3
Malaysia	3
Maldives	3
Mauritius	3
Myanmar	3

Philippines	3
Abra	4
Agusan Del Norte	4
Agusan Del Sur	4
Aklan	4
Albay	4
Antique	4
Apayao	4
Aurora	4
Basilan	4
Bataan	4
Batanes	4
Batangas	4
Benguet	4
Biliran	4
Bohol	4
Bukidnon	4
Bulacan	4
Cagayan	4
Camarines Norte	4
Camarines Sur	4
Camiguin	4
Capiz	4
Catanduanes	4
Cavite	4
Cebu	4
Cotabato (North Cotabato)	4
Davao de Oro	4
Davao Del Norte	4
Davao Del Sur	4
Davao Occidental	4
Davao Oriental	4
Dinagat Islands	4
Eastern Samar	4
Guimaras	4
Ifugao	4
Ilocos Norte	4
Ilocos Sur	4
Iloilo	4
Isabela	4
Kalinga	4
La Union	4

Laguna	4
Lanao Del Norte	4
Lanao Del Sur	4
Leyte	4
Maguindanao	4
Marinduque	4
Masbate	4
Misamis Occidental	4
Misamis Oriental	4
Mountain Province	4
National Capital Region	4
Negros Occidental	4
Negros Oriental	4
Northern Samar	4
Nueva Ecija	4
Nueva Vizcaya	4
Occidental Mindoro	4
Oriental Mindoro	4
Palawan	4
Pampanga	4
Pangasinan	4
Quezon	4
Quirino	4
Rizal	4
Romblon	4
Samar (Western Samar)	4
Sarangani	4
Siquijor	4
Sorsogon	4
South Cotabato	4
Southern Leyte	4
Sultan Kudarat	4
Sulu	4
Surigao Del Norte	4
Surigao Del Sur	4
Tarlac	4
Tawi-Tawi	4
Zambales	4
Zamboanga Del Norte	4
Zamboanga Del Sur	4
Zamboanga Sibugay	4
Seychelles	3

Sri Lanka	3
Thailand	3
Timor-Leste	3
Viet Nam	3
Sub-Saharan Africa	1
Central sub-Saharan Africa	2
Angola	3
Central African Republic	3
Congo (Brazzaville)	3
DR Congo	3
Equatorial Guinea	3
Gabon	3
Eastern sub-Saharan Africa	2
Burundi	3
Comoros	3
Djibouti	3
Eritrea	3
Ethiopia	3
Addis Ababa	4
Afar	4
Amhara	4
Benishangul-Gumuz	4
Dire Dawa	4
Gambella	4
Harari	4
Oromia	4
Sidama	4
Somali	4
South West	4
Southern Nations, Nationalities, and Peoples	4
Tigray	4
Kenya	3
Baringo	4
Bomet	4
Bungoma	4
Busia	4
Elgeyo Marakwet	4
Embu	4
Garissa	4
Homa Bay	4
Isiolo	4
Kajiado	4

Kakamega	4
Kericho	4
Kiambu	4
Kilifi	4
Kirinyaga	4
Kisii	4
Kisumu	4
Kitui	4
Kwale	4
Laikipia	4
Lamu	4
Machakos	4
Makueni	4
Mandera	4
Marsabit	4
Meru	4
Migori	4
Mombasa	4
Murang'a	4
Nairobi	4
Nakuru	4
Nandi	4
Narok	4
Nyamira	4
Nyandarua	4
Nyeri	4
Samburu	4
Siaya	4
Taita Taveta	4
Tana River	4
Tharaka Nithi	4
Trans Nzoia	4
Turkana	4
Uasin Gishu	4
Vihiga	4
Wajir	4
West Pokot	4
Madagascar	3
Malawi	3
Mozambique	3
Rwanda	3
Somalia	3

South Sudan	3
Uganda	3
Tanzania	3
Zambia	3
Southern sub-Saharan Africa	2
Botswana	3
Eswatini	3
Lesotho	3
Namibia	3
South Africa	3
Eastern Cape	4
Free State	4
Gauteng	4
KwaZulu-Natal	4
Limpopo	4
Mpumalanga	4
North West	4
Northern Cape	4
Western Cape	4
Zimbabwe	3
Western sub-Saharan Africa	2
Benin	3
Burkina Faso	3
Cabo Verde	3
Cameroon	3
Chad	3
Côte d'Ivoire	3
Ghana	3
Guinea	3
Guinea-Bissau	3
Liberia	3
Mali	3
Mauritania	3
Niger	3
Nigeria	3
Abia	4
Adamawa	4
Akwa Ibom	4
Anambra	4
Bauchi	4
Bayelsa	4
Benue	4

Borno	4
Cross River	4
Delta	4
Ebonyi	4
Edo	4
Ekiti	4
Enugu	4
FCT (Abuja)	4
Gombe	4
Imo	4
Jigawa	4
Kaduna	4
Kano	4
Katsina	4
Kebbi	4
Kogi	4
Kwara	4
Lagos	4
Nasarawa	4
Niger	4
Ogun	4
Ondo	4
Osun	4
Oyo	4
Plateau	4
Rivers	4
Sokoto	4
Taraba	4
Yobe	4
Zamfara	4
São Tomé and Príncipe	3
Senegal	3
Sierra Leone	3
The Gambia	3
Togo	3

*United Nations convention recognises Taiwan as a province of China

Appendix 1 Table S2: GATHER checklist

Item #	Checklist item	Reporting location
**Objectives and funding**		
**1**	Define the indicator(s), populations (including age, sex, and geographic entities), and time period(s) for which estimates were made.	Main text (Summary—Methods section); Main text (Methods section); appendix 1 (sections 1.1–1.4)
**2**	List the funding sources for the work.	Main text (Summary—Funding section); appendix 1 (section 1.9)
**Data Inputs**		
For all data inputs from multiple sources that are synthesized as part of the study:		
**3**	Describe how the data were identified and how the data were accessed.	Main text (Methods); appendix 1 (section 1.8, section 2.1, section 2.6.1.1, section 3.2, sections 4.2–4.3)
**4**	Specify the inclusion and exclusion criteria. Identify all ad-hoc exclusions.	Main text (Methods); appendix 1 (section 1.8, section 2.1, sections 2.2.1–2.2.9, section 2.5.2, section 3.2.2, section 4.2-4.3)
**5**	Provide information on all included data sources and their main characteristics. For each data source used, report reference information or contact name/institution, population represented, data collection method, year(s) of data collection, sex and age range, diagnostic criteria or measurement method, and sample size, as relevant.	Online data citation tool: https://ghdx.healthdata.org/gbd-2023/sources
**6**	Identify and describe any categories of input data that have potentially important biases (e.g., based on characteristics listed in item 5).	Appendix 1 (section 1.8, section 2.1, sections 2.2.1-2.2.9, section 3.2, sections 4.2-4.4, section 6.4)
For data inputs that contribute to the analysis but were not synthesized as part of the study:		
**7**	Describe and give sources for any other data inputs.	Online data citation tool: https://ghdx.healthdata.org/gbd-2023/sources
For all data inputs:		
**8**	Provide all data inputs in a file format from which data can be efficiently extracted (e.g., a spreadsheet rather than a PDF), including all relevant meta-data listed in item 5. For any data inputs that cannot be shared because of ethical or legal reasons, such as third-party ownership, provide a contact name or the name of the institution that retains the right to the data.	Online data citation tool: https://ghdx.healthdata.org/gbd-2023/sources. Availability of data is dependent on data use agreements; contact information provided when not directly available.
**Data analysis**		
**9**	Provide a conceptual overview of the data analysis method. A diagram may be helpful.	Main text (Methods); appendix 1 (sections 2–6; figure S1)
**10**	Provide a detailed description of all steps of the analysis, including mathematical formulae. This description should cover, as relevant, data cleaning, data pre-processing, data adjustments and weighting of data sources, and mathematical or statistical model(s).	Main text (Methods); appendix 1 (sections 2–6)
**11**	Describe how candidate models were evaluated and how the final model(s) were selected.	Appendix 1 (sections 2.2.4, section -2.2.8, section 2.3, sections 3.2-3.3, section 4.4, section 6)

**12**	Provide the results of an evaluation of model performance, if done, as well as the results of any relevant sensitivity analysis.	Appendix 1 (sections 2.2.1-2.2.8, sections 3.2-3.3, section 4.4, section 6)
**13**	Describe methods for calculating uncertainty of the estimates. State which sources of uncertainty were, and were not, accounted for in the uncertainty analysis.	Appendix 1 (section 2.2.8, section 2.3.2, section 2.3.5, section 2.6.6, section 3.2.6, section 4.2.3, section 4.4.4, section 6.4)
**14**	State how analytic or statistical source code used to generate estimates can be accessed.	Code provided in online repository: https://ghdx.healthdata.org/gbd-2023/code
**Results and Discussion**		
**15**	Provide published estimates in a file format from which data can be efficiently extracted.	Manuscript, supplementary results, and online data tools (data visualization tools, data query tools, and the Global Health Data Exchange); https://vizhub.healthdata.org/gbd-results/
**16**	Report a quantitative measure of the uncertainty of the estimates (e.g. uncertainty intervals).	UIs given in main text, appendix 1, and appendix 2, including in figures (as appropriate) and tables; UIs also given for all results in our online results tool: https://vizhub.healthdata.org/gbd-results/
**17**	Interpret results in light of existing evidence. If updating a previous set of estimates, describe the reasons for changes in estimates.	Main text (Discussion)
**18**	Discuss limitations of the estimates. Include a discussion of any modelling assumptions or data limitations that affect interpretation of the estimates.	Main text (Discussion—Limitations and future directions section)

Appendix 1 Table S3. Number of all-cause mortality data sources by type and location, 1950-2023						
**Location**	**SBH**	**CBH**	**HH**	**SIBS**	**VR/SRS/DSP**	**HDSS**
Armenia	2	4	0	0	45	0
Azerbaijan	2	3	0	0	46	0
Georgia	1	3	0	0	38	0
Kazakhstan	6	2	0	0	46	0
Kyrgyzstan	4	5	0	0	46	0
Mongolia	8	4	0	0	41	0
Tajikistan	6	4	0	0	43	0
Turkmenistan	1	3	0	0	38	0
Uzbekistan	2	3	0	0	41	0
Albania	3	3	0	0	44	0
Bosnia and Herzegovina	0	0	0	0	68	0
Bulgaria	0	0	0	0	74	0
Croatia	0	0	0	0	74	0
Czechia	0	0	0	0	74	0
Hungary	0	0	0	0	73	0
Montenegro	1	0	0	0	67	0
North Macedonia	0	0	0	0	73	0
Poland	0	0	0	0	74	0
Romania	0	0	0	0	68	0
Serbia	0	0	0	0	69	0
Slovakia	0	0	0	0	74	0
Slovenia	0	0	0	0	74	0
Belarus	2	0	0	0	62	0
Estonia	0	0	0	0	65	0
Latvia	0	0	0	0	64	0
Lithuania	0	0	0	0	65	0
Moldova	2	2	0	0	46	0
Russia	0	0	0	0	65	0
Ukraine	0	1	0	0	63	0
Australia	0	0	0	0	74	0
New Zealand	0	0	0	0	74	0
Brunei	0	0	0	0	64	0
Japan	0	0	0	0	74	0
Aichi	0	0	0	0	45	0
Akita	0	0	0	0	45	0
Aomori	0	0	0	0	45	0
Chiba	0	0	0	0	45	0
Ehime	0	0	0	0	45	0
Fukui	0	0	0	0	45	0
Fukuoka	0	0	0	0	45	0
Fukushima	0	0	0	0	45	0
Gifu	0	0	0	0	45	0
Gunma	0	0	0	0	45	0
Hiroshima	0	0	0	0	45	0
Hokkaidō	0	0	0	0	45	0
Hyōgo	0	0	0	0	45	0
Ibaraki	0	0	0	0	45	0
Ishikawa	0	0	0	0	45	0
Iwate	0	0	0	0	45	0
Kagawa	0	0	0	0	45	0
Kagoshima	0	0	0	0	45	0
Kanagawa	0	0	0	0	45	0

Appendix 1 Table S3. Number of all-cause mortality data sources by type and location, 1950-2023

Location	SBH	CBH	HH	SIBS	VR/SRS/DSP	HDSS
Kōchi	0	0	0	0	45	0
Kumamoto	0	0	0	0	45	0
Kyōto	0	0	0	0	45	0
Mie	0	0	0	0	45	0
Miyagi	0	0	0	0	45	0
Miyazaki	0	0	0	0	45	0
Nagano	0	0	0	0	45	0
Nagasaki	0	0	0	0	45	0
Nara	0	0	0	0	45	0
Niigata	0	0	0	0	45	0
Ōita	0	0	0	0	45	0
Okayama	0	0	0	0	45	0
Okinawa	0	0	0	0	45	0
Ōsaka	0	0	0	0	45	0
Saga	0	0	0	0	45	0
Saitama	0	0	0	0	45	0
Shiga	0	0	0	0	45	0
Shimane	0	0	0	0	45	0
Shizuoka	0	0	0	0	45	0
Tochigi	0	0	0	0	45	0
Tokushima	0	0	0	0	45	0
Tōkyō	0	0	0	0	45	0
Tottori	0	0	0	0	45	0
Toyama	0	0	0	0	45	0
Wakayama	0	0	0	0	45	0
Yamagata	0	0	0	0	45	0
Yamaguchi	0	0	0	0	45	0
Yamanashi	0	0	0	0	45	0
Singapore	0	0	0	0	74	0
South Korea	5	1	0	0	47	0
Canada	0	0	0	0	73	0
Greenland	0	0	0	0	71	0
USA	0	0	0	0	74	0
Alabama	0	0	0	0	65	0
Alaska	0	0	0	0	65	0
Arizona	0	0	0	0	65	0
Arkansas	0	0	0	0	65	0
California	0	0	0	0	65	0
Colorado	0	0	0	0	65	0
Connecticut	0	0	0	0	65	0
Delaware	0	0	0	0	65	0
Florida	0	0	0	0	65	0
Georgia	0	0	0	0	65	0
Hawaii	0	0	0	0	65	0
Idaho	0	0	0	0	65	0
Illinois	0	0	0	0	65	0
Indiana	0	0	0	0	65	0
Iowa	0	0	0	0	65	0
Kansas	0	0	0	0	65	0
Kentucky	0	0	0	0	65	0
Louisiana	0	0	0	0	65	0
Maine	0	0	0	0	65	0

Appendix 1 Table S3. Number of all-cause mortality data sources by type and location, 1950-2023

Location	SBH	CBH	HH	SIBS	VR/SRS/DSP	HDSS
Maryland	0	0	0	0	65	0
Massachusetts	0	0	0	0	65	0
Michigan	0	0	0	0	65	0
Minnesota	0	0	0	0	65	0
Mississippi	0	0	0	0	65	0
Missouri	0	0	0	0	65	0
Montana	0	0	0	0	65	0
Nebraska	0	0	0	0	65	0
Nevada	0	0	0	0	65	0
New Hampshire	0	0	0	0	65	0
New Jersey	0	0	0	0	65	0
New Mexico	0	0	0	0	65	0
New York	0	0	0	0	65	0
North Carolina	0	0	0	0	65	0
North Dakota	0	0	0	0	65	0
Ohio	0	0	0	0	65	0
Oklahoma	0	0	0	0	65	0
Oregon	0	0	0	0	65	0
Pennsylvania	0	0	0	0	65	0
Rhode Island	0	0	0	0	65	0
South Carolina	0	0	0	0	65	0
South Dakota	0	0	0	0	65	0
Tennessee	0	0	0	0	65	0
Texas	0	0	0	0	65	0
Utah	0	0	0	0	65	0
Vermont	0	0	0	0	65	0
Virginia	0	0	0	0	65	0
Washington	0	0	0	0	65	0
Washington, DC	0	0	0	0	65	0
West Virginia	0	0	0	0	65	0
Wisconsin	0	0	0	0	65	0
Wyoming	0	0	0	0	65	0
Argentina	0	0	0	0	68	0
Chile	0	0	0	0	73	0
Uruguay	0	0	0	0	73	0
Andorra	0	0	0	0	23	0
Austria	0	0	0	0	74	0
Belgium	0	0	0	0	70	0
Cyprus	3	0	0	0	50	0
Denmark	0	0	0	0	74	0
Finland	0	0	0	0	74	0
France	0	0	0	0	74	0
Germany	0	0	0	0	67	0
Greece	0	0	0	0	73	0
Iceland	0	0	0	0	74	0
Ireland	0	0	0	0	74	0
Israel	0	0	0	0	71	0
Italy	0	0	0	0	74	0
Luxembourg	0	0	0	0	73	0
Malta	0	0	0	0	74	0
Monaco	0	0	0	0	33	0
Netherlands	0	0	0	0	74	0

Appendix 1 Table S3. Number of all-cause mortality data sources by type and location, 1950-2023						
**Location**	**SBH**	**CBH**	**HH**	**SIBS**	**VR/SRS/DSP**	**HDSS**
Norway	0	0	0	0	74	0
Agder	0	0	0	0	71	0
Innlandet	0	0	0	0	71	0
Møre og Romsdal	0	0	0	0	71	0
Nordland	0	0	0	0	71	0
Oslo	0	0	0	0	71	0
Rogaland	0	0	0	0	71	0
Troms og Finnmark	0	0	0	0	71	0
Trøndelag	0	0	0	0	71	0
Vestfold og Telemark	0	0	0	0	71	0
Vestland	0	0	0	0	71	0
Viken	0	0	0	0	71	0
Portugal	0	0	0	0	73	0
San Marino	0	0	0	0	56	0
Spain	0	0	0	0	74	0
Sweden	0	0	0	0	74	0
Switzerland	0	0	0	0	74	0
UK	0	0	0	0	74	0
England	0	0	0	0	43	0
East Midlands	0	0	0	0	42	0
East of England	0	0	0	0	42	0
Greater London	0	0	0	0	42	0
North East England	0	0	0	0	42	0
North West England	0	0	0	0	42	0
South East England	0	0	0	0	42	0
South West England	0	0	0	0	42	0
West Midlands	0	0	0	0	42	0
Yorkshire and the Humber	0	0	0	0	42	0
Northern Ireland	0	0	0	0	74	0
Scotland	0	0	0	0	74	0
Wales	0	0	0	0	43	0
Bolivia	20	6	2	3	12	0
Ecuador	10	7	0	0	70	0
Peru	9	15	0	4	71	0
Antigua and Barbuda	0	0	0	0	65	0
Barbados	0	0	0	0	62	0
Belize	4	3	0	0	73	0
Bermuda	0	0	0	0	73	0
Cuba	0	0	0	0	63	0
Dominica	0	0	0	0	66	0
Dominican Republic	5	14	0	2	70	0
Grenada	0	0	0	0	60	0
Guyana	3	5	0	0	53	0
Haiti	2	8	0	3	0	0
Jamaica	4	2	0	0	53	0
Puerto Rico	0	0	0	0	74	0
Saint Kitts and Nevis	0	0	0	0	72	0
Saint Lucia	0	0	0	0	62	0
Saint Vincent and the Grenadines	0	0	0	0	56	0
Suriname	2	0	0	0	64	0
The Bahamas	0	0	0	0	52	0
Trinidad and Tobago	3	3	0	0	67	0

Appendix 1 Table S3. Number of all-cause mortality data sources by type and location, 1950-2023

Location	SBH	CBH	HH	SIBS	VR/SRS/DSP	HDSS
Virgin Islands	0	0	0	0	56	0
Colombia	5	8	0	0	71	0
Costa Rica	0	0	0	0	73	0
El Salvador	4	5	0	3	70	0
Guatemala	4	6	0	0	71	0
Honduras	7	7	0	1	38	0
Mexico	20	4	0	0	74	0
Aguascalientes	13	2	0	0	45	0
Baja California	5	0	0	0	45	0
Baja California Sur	5	0	0	0	45	0
Campeche	13	2	0	0	45	0
Chiapas	13	2	0	0	45	0
Chihuahua	13	2	0	0	45	0
Coahuila	13	2	0	0	45	0
Colima	13	2	0	0	45	0
Durango	13	2	0	0	45	0
Guanajuato	13	2	0	0	45	0
Guerrero	13	2	0	0	45	0
Hidalgo	11	2	0	0	45	0
Jalisco	13	2	0	0	45	0
México	5	0	0	0	45	0
Mexico City	5	1	0	0	45	0
Michoacán de Ocampo	13	2	0	0	45	0
Morelos	13	2	0	0	45	0
Nayarit	13	2	0	0	45	0
Nuevo León	13	2	0	0	45	0
Oaxaca	13	2	0	0	45	0
Puebla	13	2	0	0	45	0
Querétaro	13	2	0	0	45	0
Quintana Roo	13	2	0	0	45	0
San Luis Potosí	13	2	0	0	45	0
Sinaloa	13	2	0	0	45	0
Sonora	13	2	0	0	45	0
Tabasco	13	2	0	0	45	0
Tamaulipas	13	2	0	0	45	0
Tlaxcala	13	2	0	0	45	0
Veracruz de Ignacio de la Llave	13	2	0	0	45	0
Yucatán	13	2	0	0	45	0
Zacatecas	13	2	0	0	45	0
Nicaragua	7	5	0	0	58	0
Panama	8	1	0	0	72	0
Venezuela	0	0	0	0	67	0
Brazil	26	3	0	0	50	0
Acre	8	1	0	0	45	0
Alagoas	20	3	0	0	44	0
Amapá	2	0	0	0	45	0
Amazonas	9	2	0	0	45	0
Bahia	20	3	0	0	42	0
Ceará	20	3	0	0	38	0
Distrito Federal	10	2	0	0	45	0
Espírito Santo	18	2	0	0	45	0
Goiás	20	2	0	0	35	0

Appendix 1 Table S3. Number of all-cause mortality data sources by type and location, 1950-2023

Location	SBH	CBH	HH	SIBS	VR/SRS/DSP	HDSS
Maranhão	20	3	0	0	39	0
Mato Grosso	20	2	0	0	45	0
Mato Grosso do Sul	10	2	0	0	44	0
Minas Gerais	20	2	0	0	45	0
Pará	9	2	0	0	41	0
Paraíba	20	3	0	0	44	0
Paraná	9	2	0	0	44	0
Pernambuco	20	3	0	0	44	0
Piauí	20	3	0	0	41	0
Rio de Janeiro	9	2	0	0	45	0
Rio Grande do Norte	20	3	0	0	44	0
Rio Grande do Sul	10	2	0	0	45	0
Rondônia	8	1	0	0	45	0
Roraima	8	1	0	0	45	0
Santa Catarina	9	2	0	0	45	0
São Paulo	9	2	0	0	45	0
Sergipe	20	3	0	0	45	0
Tocantins	8	1	0	0	34	0
Paraguay	8	7	0	0	69	0
Afghanistan	5	3	1	0	0	0
Algeria	2	4	0	0	25	0
Bahrain	2	2	0	0	40	0
Egypt	3	13	0	0	67	0
Iran	4	4	0	0	26	0
Alborz	3	1	0	0	26	0
Ardebil	3	1	0	0	26	0
Bushehr	3	1	0	0	26	0
Chahar Mahaal and Bakhtiari	3	1	0	0	26	0
East Azarbayejan	3	1	0	0	26	0
Fars	3	1	0	0	26	0
Gilan	3	1	0	0	26	0
Golestan	3	1	0	0	26	0
Hamadan	3	1	0	0	26	0
Hormozgan	3	1	0	0	26	0
Ilam	3	1	0	0	26	0
Isfahan	3	1	0	0	26	0
Kerman	3	1	0	0	26	0
Kermanshah	3	1	0	0	26	0
Khorasan-e-Razavi	3	1	0	0	26	0
Khuzestan	3	1	0	0	26	0
Kohgiluyeh and Boyer-Ahmad	3	1	0	0	26	0
Kurdistan	3	1	0	0	26	0
Lorestan	3	1	0	0	26	0
Markazi	3	1	0	0	26	0
Mazandaran	3	1	0	0	26	0
North Khorasan	3	1	0	0	26	0
Qazvin	3	1	0	0	26	0
Qom	3	1	0	0	26	0
Semnan	3	1	0	0	26	0
Sistan and Baluchistan	3	1	0	0	26	0
South Khorasan	3	1	0	0	26	0
Tehran	3	1	0	0	26	0

Appendix 1 Table S3. Number of all-cause mortality data sources by type and location, 1950-2023

Location	SBH	CBH	HH	SIBS	VR/SRS/DSP	HDSS
West Azarbayejan	3	1	0	0	26	0
Yazd	3	1	0	0	26	0
Zanjan	3	1	0	0	26	0
Iraq	2	8	0	1	26	0
Jordan	5	8	0	1	33	0
Kuwait	0	0	0	0	61	0
Lebanon	2	2	0	1	13	0
Libya	3	1	0	0	7	0
Morocco	3	7	0	2	12	0
Oman	4	2	0	0	18	0
Palestine	3	7	0	0	17	0
Qatar	0	2	0	0	42	0
Saudi Arabia	2	1	0	0	16	0
Sudan	4	7	0	1	0	0
Syria	3	4	0	0	30	0
Tunisia	2	7	0	0	20	0
Türkiye	8	7	0	0	41	0
United Arab Emirates	2	1	0	0	19	0
Yemen	2	7	0	0	0	0
Bangladesh	5	13	1	3	14	3
Bhutan	4	1	1	1	0	0
India	8	6	0	0	41	0
Andhra Pradesh	3	5	0	0	8	0
Arunachal Pradesh	7	6	0	0	0	0
Assam	9	6	0	0	26	0
Bihar	11	6	0	0	27	0
Chhattisgarh	9	5	0	0	18	0
Delhi	6	6	0	0	18	0
Goa	6	5	0	0	11	0
Gujarat	6	6	0	0	27	0
Haryana	7	6	0	0	27	0
Himachal Pradesh	7	6	0	0	41	0
Jammu & Kashmir and Ladakh	6	6	0	0	18	0
Jharkhand	9	5	0	0	18	0
Karnataka	7	5	0	0	27	0
Kerala	7	5	0	0	40	0
Madhya Pradesh	11	6	0	0	27	0
Maharashtra	6	6	0	0	35	0
Manipur	6	6	0	0	0	0
Meghalaya	6	6	0	0	0	0
Mizoram	7	6	0	0	0	0
Nagaland	4	5	0	0	0	0
Odisha	11	6	0	0	27	0
Other Union Territories	2	3	0	0	0	0
Punjab	6	6	0	0	27	0
Rajasthan	12	6	0	0	27	0
Sikkim	7	5	0	0	11	0
Tamil Nadu	7	5	0	0	27	0
Telangana	3	3	0	0	7	0
Tripura	6	6	0	0	0	0
Uttar Pradesh	11	6	0	0	27	0
Uttarakhand	8	5	0	0	8	0

Appendix 1 Table S3. Number of all-cause mortality data sources by type and location, 1950-2023

Location	SBH	CBH	HH	SIBS	VR/SRS/DSP	HDSS
West Bengal	7	6	0	0	27	0
Nepal	2	10	3	2	0	0
Pakistan	5	15	0	0	22	0
Azad Jammu & Kashmir	1	2	0	0	0	0
Balochistan	3	8	0	0	0	0
Gilgit-Baltistan	1	4	0	0	0	0
Islamabad Capital Territory	0	2	0	0	0	0
Khyber Pakhtunkhwa	2	9	0	0	0	0
Punjab	4	8	0	0	0	0
Sindh	3	8	0	0	0	0
China	4	3	4	0	51	0
North Korea	0	1	0	0	0	0
Taiwan	0	0	0	0	69	0
American Samoa	0	0	0	0	55	0
Cook Islands	1	0	0	0	51	0
Federated States of Micronesia	3	0	0	0	6	0
Fiji	6	2	0	0	62	0
Guam	0	0	0	0	73	0
Kiribati	6	2	0	0	12	0
Marshall Islands	2	1	0	0	14	0
Nauru	1	1	0	0	17	0
Niue	0	0	0	0	50	0
Northern Mariana Islands	0	0	0	0	27	0
Palau	2	0	0	0	10	0
Papua New Guinea	6	4	0	1	0	0
Samoa	5	3	1	0	22	0
Solomon Islands	4	2	0	0	2	0
Tokelau	0	0	0	0	11	0
Tonga	2	2	0	0	18	0
Tuvalu	0	1	0	0	17	0
Vanuatu	3	2	0	0	1	0
Cambodia	6	6	2	5	0	0
Indonesia	34	17	3	5	0	0
Aceh	28	5	0	4	0	0
Bali	31	6	0	5	0	0
Bangka-Belitung Islands	22	4	0	4	0	0
Banten	30	4	0	4	0	0
Bengkulu	31	6	0	5	0	0
Gorontalo	19	4	0	5	0	0
Jakarta	35	6	0	5	0	0
Jambi	30	5	0	5	0	0
West Java	35	6	0	5	0	0
Central Java	35	6	0	5	0	0
East Java	35	6	0	5	0	0
West Kalimantan	31	6	0	5	0	0
South Kalimantan	35	6	0	5	0	0
Central Kalimantan	32	5	0	5	0	0
East Kalimantan	30	5	0	5	0	0
North Kalimantan	17	3	0	2	0	0
Riau Islands	27	3	0	5	0	0
Lampung	34	6	0	5	0	0
Maluku	30	4	0	4	0	0

Appendix 1 Table S3. Number of all-cause mortality data sources by type and location, 1950-2023						
**Location**	**SBH**	**CBH**	**HH**	**SIBS**	**VR/SRS/DSP**	**HDSS**
North Maluku	22	3	0	3	0	0
West Nusa Tenggara	35	6	0	5	0	0
East Nusa Tenggara	30	5	0	5	0	0
Papua	28	4	0	4	0	0
West Papua	27	3	0	4	0	0
Riau	32	6	0	5	0	0
West Sulawesi	28	3	0	3	0	0
South Sulawesi	35	6	0	5	0	0
Central Sulawesi	31	6	0	5	0	0
Southeast Sulawesi	30	6	0	5	0	0
North Sulawesi	31	6	0	5	0	0
West Sumatra	35	6	0	5	0	0
South Sumatra	35	6	0	5	0	0
North Sumatra	35	6	0	5	0	0
Yogyakarta	35	6	0	5	0	0
Laos	1	3	0	1	0	0
Malaysia	0	0	0	0	55	0
Maldives	5	2	0	0	47	0
Mauritius	0	0	0	0	74	0
Myanmar	5	3	0	1	0	0
Philippines	6	10	0	0	71	0
Abra	1	7	0	0	25	0
Agusan Del Norte	1	7	0	0	24	0
Agusan Del Sur	1	7	0	0	24	0
Aklan	1	7	0	0	30	0
Albay	1	7	0	0	30	0
Antique	1	7	0	0	30	0
Apayao	1	6	0	0	18	0
Aurora	1	7	0	0	18	0
Basilan	1	7	0	0	23	0
Bataan	1	7	0	0	30	0
Batanes	2	5	0	0	30	0
Batangas	1	7	0	0	30	0
Benguet	1	7	0	0	18	0
Biliran	1	6	0	0	18	0
Bohol	1	7	0	0	30	0
Bukidnon	1	7	0	0	30	0
Bulacan	1	7	0	0	30	0
Cagayan	1	7	0	0	30	0
Camarines Norte	1	7	0	0	30	0
Camarines Sur	1	7	0	0	30	0
Camiguin	1	6	0	0	29	0
Capiz	1	7	0	0	30	0
Catanduanes	1	7	0	0	30	0
Cavite	1	7	0	0	30	0
Cebu	1	7	0	0	30	0
Cotabato (North Cotabato)	1	7	0	0	30	0
Davao de Oro	1	5	0	0	17	0
Davao Del Norte	1	7	0	0	29	0
Davao Del Sur	1	7	0	0	29	0
Davao Occidental	2	1	0	0	14	0
Davao Oriental	1	7	0	0	29	0

Appendix 1 Table S3. Number of all-cause mortality data sources by type and location, 1950-2023

Location	SBH	CBH	HH	SIBS	VR/SRS/DSP	HDSS
Dinagat Islands	1	2	0	0	18	0
Eastern Samar	1	7	0	0	29	0
Guimaras	2	6	0	0	18	0
Ifugao	1	7	0	0	29	0
Ilocos Norte	1	7	0	0	30	0
Ilocos Sur	1	7	0	0	30	0
Iloilo	1	7	0	0	30	0
Isabela	1	7	0	0	30	0
Kalinga	1	7	0	0	28	0
La Union	1	7	0	0	30	0
Laguna	1	7	0	0	30	0
Lanao Del Norte	1	7	0	0	30	0
Lanao Del Sur	1	7	0	0	18	0
Leyte	1	7	0	0	30	0
Maguindanao	1	7	0	0	23	0
Marinduque	1	7	0	0	30	0
Masbate	1	7	0	0	30	0
Misamis Occidental	1	7	0	0	30	0
Misamis Oriental	1	7	0	0	30	0
Mountain Province	1	7	0	0	29	0
National Capital Region	1	7	0	0	18	0
Negros Occidental	1	7	0	0	30	0
Negros Oriental	1	7	0	0	30	0
Northern Samar	1	7	0	0	29	0
Nueva Ecija	1	7	0	0	30	0
Nueva Vizcaya	1	7	0	0	30	0
Occidental Mindoro	1	7	0	0	30	0
Oriental Mindoro	1	7	0	0	30	0
Palawan	1	7	0	0	30	0
Pampanga	1	7	0	0	30	0
Pangasinan	1	7	0	0	30	0
Quezon	1	7	0	0	30	0
Quirino	1	7	0	0	24	0
Rizal	1	7	0	0	18	0
Romblon	1	7	0	0	30	0
Samar (Western Samar)	1	7	0	0	30	0
Sarangani	1	6	0	0	18	0
Siquijor	2	7	0	0	24	0
Sorsogon	1	7	0	0	30	0
South Cotabato	1	7	0	0	28	0
Southern Leyte	1	7	0	0	29	0
Sultan Kudarat	1	7	0	0	23	0
Sulu	1	7	0	0	15	0
Surigao Del Norte	1	7	0	0	30	0
Surigao Del Sur	1	7	0	0	30	0
Tarlac	1	7	0	0	30	0
Tawi-Tawi	1	7	0	0	22	0
Zambales	1	7	0	0	30	0
Zamboanga Del Norte	1	7	0	0	30	0
Zamboanga Del Sur	1	7	0	0	30	0
Zamboanga Sibugay	1	5	0	0	18	0
Seychelles	0	0	0	0	71	0

Appendix 1 Table S3. Number of all-cause mortality data sources by type and location, 1950-2023

Location	SBH	CBH	HH	SIBS	VR/SRS/DSP	HDSS
Sri Lanka	0	2	0	0	73	0
Thailand	9	2	0	0	73	0
Timor-Leste	3	6	0	4	0	0
Viet Nam	12	5	2	0	0	0
Angola	1	4	0	1	0	0
Central African Republic	4	2	1	1	0	0
Congo (Brazzaville)	2	3	0	3	0	0
DR Congo	3	4	0	2	0	0
Equatorial Guinea	1	1	0	0	0	0
Gabon	0	3	0	2	0	0
Burundi	6	3	2	2	0	0
Comoros	0	1	1	2	0	0
Djibouti	2	2	0	0	0	0
Eritrea	1	2	0	1	0	0
Ethiopia	4	6	0	4	0	1
Addis Ababa	1	6	0	4	0	0
Afar	1	6	0	4	0	0
Amhara	1	6	0	4	0	1
Benishangul-Gumuz	1	6	0	4	0	0
Dire Dawa	1	6	0	4	0	0
Gambella	1	6	0	4	0	0
Harari	1	6	0	4	0	1
Oromia	1	6	0	4	0	2
Sidama	0	5	0	4	0	0
Somali	1	6	0	4	0	0
South West	0	5	0	4	0	0
Southern Nations, Nationalities, and Peoples	0	5	0	4	0	0
Tigray	1	6	0	4	0	1
Kenya	12	8	0	4	0	0
Baringo	6	6	0	2	0	0
Bomet	7	4	0	2	0	0
Bungoma	10	8	0	2	0	0
Busia	9	7	0	2	0	0
Elgeyo Marakwet	9	7	0	2	0	0
Embu	10	8	0	2	0	0
Garissa	8	4	0	2	0	0
Homa Bay	7	5	0	2	0	0
Isiolo	7	5	0	2	0	0
Kajiado	9	7	0	2	0	0
Kakamega	9	8	0	2	0	0
Kericho	10	7	0	2	0	0
Kiambu	10	7	0	2	0	0
Kilifi	10	7	0	2	0	0
Kirinyaga	9	7	0	2	0	0
Kisii	9	8	0	2	0	0
Kisumu	9	8	0	2	0	2
Kitui	11	8	0	2	0	0
Kwale	9	7	0	2	0	0
Laikipia	10	7	0	2	0	0
Lamu	8	5	0	2	0	0
Machakos	11	8	0	2	0	0

Appendix 1 Table S3. Number of all-cause mortality data sources by type and location, 1950-2023

Location	SBH	CBH	HH	SIBS	VR/SRS/DSP	HDSS
Makueni	8	5	0	2	0	0
Mandera	8	4	0	2	0	0
Marsabit	6	5	0	2	0	0
Meru	8	8	0	2	0	0
Migori	7	5	0	2	0	0
Mombasa	9	8	0	2	0	0
Murang'a	9	7	0	2	0	0
Nairobi	9	6	0	2	0	0
Nakuru	9	7	0	2	0	0
Nandi	10	7	0	2	0	0
Narok	9	7	0	2	0	0
Nyamira	9	5	0	1	0	0
Nyandarua	8	7	0	2	0	0
Nyeri	8	7	0	2	0	0
Samburu	6	4	0	1	0	0
Siaya	10	8	0	2	0	1
Taita Taveta	9	7	0	2	0	0
Tana River	7	5	0	2	0	0
Tharaka Nithi	7	5	0	2	0	0
Trans Nzoia	9	7	0	2	0	0
Turkana	7	5	0	2	0	0
Uasin Gishu	9	7	0	2	0	0
Vihiga	7	4	0	2	0	0
Wajir	8	4	0	2	0	0
West Pokot	9	7	0	2	0	0
Madagascar	6	6	1	5	0	0
Malawi	13	10	4	6	0	2
Mozambique	4	6	1	4	0	2
Rwanda	7	8	1	5	0	0
Somalia	1	1	0	0	0	0
South Sudan	1	1	0	0	0	0
Uganda	11	7	0	5	0	2
Tanzania	7	8	1	5	0	2
Zambia	6	7	0	6	0	0
Botswana	7	3	2	0	10	0
Eswatini	1	4	2	1	0	0
Lesotho	6	6	1	3	0	0
Namibia	2	4	2	4	7	0
South Africa	8	2	4	2	31	0
Eastern Cape	6	2	3	2	27	0
Free State	6	2	3	2	27	0
Gauteng	6	2	3	2	27	1
KwaZulu-Natal	7	2	3	2	27	2
Limpopo	6	2	3	2	27	2
Mpumalanga	6	2	3	2	27	2
North West	6	2	3	2	27	0
Northern Cape	5	2	3	2	27	0
Western Cape	6	2	3	2	27	0
Zimbabwe	1	11	2	7	2	0
Benin	2	8	2	4	0	0
Burkina Faso	8	6	1	4	0	2
Cabo Verde	2	2	0	0	22	0

Appendix 1 Table S3. Number of all-cause mortality data sources by type and location, 1950-2023						
Location	SBH	CBH	HH	SIBS	VR/SRS/DSP	HDSS
Cameroon	4	7	1	4	0	0
Chad	2	4	0	3	0	0
Côte d'Ivoire	1	11	3	4	0	1
Ghana	10	13	1	2	0	2
Guinea	1	6	1	4	0	0
Guinea-Bissau	3	2	0	1	0	0
Liberia	3	5	1	3	0	0
Mali	4	8	4	5	0	0
Mauritania	3	6	0	3	0	0
Niger	2	4	0	3	0	0
Nigeria	16	10	1	3	0	0
Abia	9	7	0	3	0	0
Adamawa	9	8	0	3	0	0
Akwa Ibom	9	8	0	3	0	0
Anambra	11	8	0	3	0	0
Bauchi	9	8	0	3	0	0
Bayelsa	8	7	0	3	0	0
Benue	9	8	0	3	0	0
Borno	9	8	0	3	0	0
Cross River	9	8	0	3	0	0
Delta	9	7	0	3	0	0
Ebonyi	8	7	0	3	0	0
Edo	9	8	0	3	0	0
Ekiti	6	7	0	3	0	0
Enugu	8	7	0	3	0	0
FCT (Abuja)	10	7	0	3	0	0
Gombe	7	7	0	3	0	0
Imo	8	8	0	3	0	0
Jigawa	9	7	0	3	0	0
Kaduna	11	8	0	3	0	0
Kano	11	8	0	3	0	0
Katsina	9	8	0	3	0	0
Kebbi	9	7	0	3	0	0
Kogi	9	7	0	3	0	0
Kwara	9	8	0	3	0	0
Lagos	11	8	0	3	0	0
Nasarawa	10	7	0	3	0	0
Niger	9	8	0	3	0	0
Ogun	9	8	0	3	0	0
Ondo	9	9	0	3	0	0
Osun	9	7	0	3	0	0
Oyo	9	8	0	3	0	0
Plateau	9	8	0	3	0	0
Rivers	10	8	0	3	0	0
Sokoto	9	8	0	3	0	0
Taraba	11	7	0	3	0	0
Yobe	9	7	0	3	0	0
Zamfara	8	7	0	3	0	1
São Tomé and Príncipe	4	3	1	2	19	0
Senegal	3	17	0	4	0	1
Sierra Leone	4	4	0	3	0	0
The Gambia	3	5	0	2	0	1

Appendix 1 Table S3. Number of all-cause mortality data sources by type and location, 1950-2023

Location	SBH	CBH	HH	SIBS	VR/SRS/DSP	HDSS
Togo	5	5	0	2	0	0

Appendix 1 Table S4. Number of all-cause mortality data sources by type and year, 1950-2023						
Year	SBH	CBH	HH	SIBS	VR/SRS/DSP	HDSS
1950	0	0	0	0	76	0
1951	0	0	0	0	78	0
1952	0	0	0	0	95	0
1953	0	0	0	0	95	0
1954	0	0	0	0	95	0
1955	0	0	0	0	103	0
1956	0	0	0	0	104	0
1957	0	0	0	0	103	0
1958	0	0	1	0	111	0
1959	0	0	0	0	160	0
1960	2	0	0	0	159	0
1961	0	0	0	0	159	0
1962	0	0	0	0	160	0
1963	0	0	0	0	161	0
1964	0	0	0	0	162	0
1965	0	0	1	0	212	0
1966	1	0	0	0	159	0
1967	2	0	0	0	156	0
1968	0	0	0	0	225	0
1969	2	0	0	0	255	0
1970	6	0	2	0	241	0
1971	29	0	0	0	241	0
1972	1	0	0	0	241	0
1973	2	0	0	0	242	0
1974	4	3	0	0	247	0
1975	1	6	0	0	186	0
1976	3	4	0	0	257	0
1977	1	5	0	0	258	0
1978	2	7	1	0	264	0
1979	2	4	0	0	343	0
1980	73	4	0	0	300	0
1981	64	2	0	0	317	0
1982	35	0	1	0	321	0
1983	3	1	0	0	322	0
1984	6	0	1	0	328	0
1985	40	1	0	0	331	0
1986	23	31	0	0	330	0
1987	6	34	3	0	335	0
1988	40	46	1	0	332	0
1989	5	4	2	1	406	0
1990	141	32	30	0	406	20
1991	10	51	1	1	407	0
1992	57	91	0	6	410	0
1993	113	124	0	2	409	0
1994	122	9	0	35	413	0
1995	95	17	0	3	462	0
1996	71	44	1	6	466	0
1997	135	43	2	37	476	0
1998	251	230	2	22	477	0

Appendix 1 Table S4. Number of all-cause mortality data sources by type and year, 1950-2023

Year	SBH	CBH	HH	SIBS	VR/SRS/DSP	HDSS
1999	149	12	1	3	480	0
2000	257	61	32	24	480	0
2001	158	16	13	3	511	0
2002	135	37	3	30	514	0
2003	95	184	0	61	516	0
2004	56	14	2	6	558	0
2005	191	124	0	22	594	0
2006	145	27	3	8	643	0
2007	272	124	13	39	643	0
2008	151	200	0	51	643	0
2009	118	15	2	3	646	0
2010	379	36	35	23	642	0
2011	252	108	12	7	645	0
2012	170	58	0	40	681	0
2013	42	138	3	46	673	0
2014	17	94	6	64	679	0
2015	129	111	0	5	686	15
2016	48	117	1	31	657	1
2017	16	139	0	3	622	1
2018	1	58	1	43	625	0
2019	0	138	0	6	621	0
2020	59	2	1	0	622	0
2021	0	46	2	3	583	0
2022	0	57	1	3	500	0
2023	1	41	0	0	454	0
2024	1	2	0	0	0	0

Appendix 1 Table S5: New data sources added for GBD 2023 mortality estimation

Location	Year	Sources
Afghanistan	2022-2023	Afghanistan Multiple Indicator Cluster Survey 2022-2023
Albania	2002-2002	Albania Living Standards Measurement Survey 2002
Algeria	1990-2017	Algeria Demography 2017
Algeria	1990-2018	Algeria Demography 2018
Algeria	1989-2040	Algeria Demography 2019
Algeria	2002-2020	Algeria Demography 2020
Algeria	2007-2023	Algeria Demography 2020-2023
American Samoa	2021-2021	American Samoa NVSS Custom Mortality Data 2021
American Samoa	2022-2022	American Samoa NVSS Custom Mortality Data 2022
American Samoa	2023-2023	American Samoa NVSS Custom Mortality Data 2023
Andorra	1986-1986	Andorra Data Bank - Deaths by Parish, Age (10-Year Age Group) and Sex
Angola	2023-2024	Angola Demographic and Health Survey 2023-2024
Antigua and Barbuda	2010-2010	Antigua and Barbuda Vital Registration - Deaths 2010 ICD10
Antigua and Barbuda	2011-2011	Antigua and Barbuda Vital Registration - Deaths 2011 ICD10
Antigua and Barbuda	2018-2018	Antigua and Barbuda Vital Registration - Deaths 2018 ICD10
Antigua and Barbuda	2019-2019	Antigua and Barbuda Vital Registration - Deaths 2019 ICD10
Antigua and Barbuda	2020-2020	Antigua and Barbuda Vital Registration - Deaths 2020 ICD10
Antigua and Barbuda	2021-2021	Antigua and Barbuda Vital Registration - Deaths 2021 ICD10
Argentina	2020-2020	Argentina Vital Registration - Deaths 2020 ICD10
Argentina	2021-2021	Argentina Vital Registration - Deaths 2021 ICD10
Argentina	1990-2021	Argentina Vital Statistics 2021
Argentina	1990-2022	Argentina Vital Statistics 2022
Armenia	1990-2022	Armenia Demographic Handbook 2023
Armenia	2022-2023	Armenia Socio-Economic Situation 2023
Armenia	2020-2020	Armenia Vital Registration - Deaths 2020 ICD10
Armenia	2021-2021	Armenia Vital Registration - Deaths 2021 ICD10
Armenia	2022-2022	Armenia Vital Registration - Deaths 2022 ICD10
Australia	2022-2022	Australia Deaths, Year of Registration, Age at Death, Age-Specific Death Rates, Sex, States and Territories 2022
Australia	2020-2023	Australia Provisional Mortality Statistics 2023
Australia	2019-2019	Australia Vital Registration - Deaths 2019 ICD10
Australia	2020-2020	Australia Vital Registration - Deaths 2020 ICD10
Australia	2021-2021	Australia Vital Registration - Deaths 2021 ICD10
Australia	2022-2022	Australia Vital Registration - Deaths 2022 ICD10
Austria	2000-2000	Austria Deceased by Calendar Week and 5-Year Groups
Austria	2021-2021	Austria Vital Registration - Deaths 2021 ICD10
Azerbaijan	2021-2021	Azerbaijan Demographic Indicators 2022
Azerbaijan	1926-2024	Azerbaijan Demographic Indicators 2024
Bahrain	2013-2020	Bahrain Health Statistics 2020
Bangladesh	2022-2022	Bangladesh Demographic and Health Survey 2022
Belgium	1992-2023	Belgium Distribution of Deaths by Age and Sex 1992-2023 (Provisional)
Belgium	2019-2019	Belgium Vital Registration - Deaths 2019 ICD10
Belgium	2020-2020	Belgium Vital Registration - Deaths 2020 ICD10
Belize	2022-2022	Belize Deaths by Sex, Age Group, District, and Cities, Villages, Towns 2022
Benin	2021-2022	Benin Multiple Indicator Cluster Survey 2021-2022

Benin	1992-1992	Benin Population and Housing Census 1992 - IPUMS
Benin	2002-2002	Benin Population and Housing Census 2002 - IPUMS
Bermuda	2017-2022	Bermuda Registry General Annual Report of Births, Marriages, Domestic Partnerships and Deaths 2022
Bhutan	2016-2022	Bhutan Vital Statistics Report 2023
Bolivia	2021-2021	Bolivia Household Survey 2021
Bosnia and Herzegovina	2022-2022	Bosnia and Herzegovina Demography 2022
Bosnia and Herzegovina	2022-2023	Bosnia and Herzegovina Natural Population Change and Marriages 2023, Quarter 4
Bosnia and Herzegovina	2021-2021	Bosnia and Herzegovina Vital Registration - Deaths 2021 ICD10
Bosnia and Herzegovina	2022-2022	Bosnia and Herzegovina Vital Registration - Deaths 2022 ICD10
Brazil	2022-2022	Brazil Mortality Information System - Deaths 2022
Brazil	2023-2023	Brazil Mortality Information System - Deaths 2023
Brunei	2017-2022	Brunei Vital Statistics 2022
Bulgaria	2017-2017	Bulgaria Deaths by Week, Sex and 5-year Age Group - Eurostat
Bulgaria	2015-2015	Bulgaria Deaths by Weeks, Age, Sex, and Districts
Bulgaria	2020-2020	Bulgaria Vital Registration - Deaths 2020 ICD10
Bulgaria	2021-2021	Bulgaria Vital Registration - Deaths 2021 ICD10
Burkina Faso	2021-2021	Burkina Faso Demographic and Health Survey 2021
Cabo Verde	2018-2018	Cape Verde Demographic and Reproductive Health Survey 2018
Cambodia	2021-2022	Cambodia Demographic and Health Survey 2021-2022
Cambodia	2004-2004	Cambodia Inter-Censal Population Survey 2004 - IPUMS
Cambodia	2013-2013	Cambodia Inter-Censal Population Survey 2013 - IPUMS
Cameroon	2017-2018	Cameroon Population-Based HIV Impact Assessment 2017-2018
Canada	2020-2020	Canada Vital Registration - Deaths 2020 ICD10
Canada	2021-2021	Canada Vital Registration - Deaths 2021 ICD10
Canada	2022-2022	Canada Vital Registration - Deaths 2022 ICD10
Chile	1990-1990	Chile Vital Statistics - Deaths 1990
Chile	1991-1991	Chile Vital Statistics - Deaths 1991
Chile	1992-1992	Chile Vital Statistics - Deaths 1992
Chile	1993-1993	Chile Vital Statistics - Deaths 1993
Chile	1994-1994	Chile Vital Statistics - Deaths 1994
Chile	1995-1995	Chile Vital Statistics - Deaths 1995
Chile	1996-1996	Chile Vital Statistics - Deaths 1996
Chile	2012-2022	Chile Vital Statistics Yearbook 2022 (Provisional)
Chile	2012-2023	Chile Vital Statistics Yearbook 2023 (Provisional)
China	2017-2017	China Disease Surveillance Points 2017 - China CDC
China	2018-2018	China Disease Surveillance Points 2018 - China CDC
China	2019-2019	China Disease Surveillance Points 2019 - China CDC
China	2020-2020	China Disease Surveillance Points 2020 - China CDC
China	2021-2021	China Disease Surveillance Points 2021 - China CDC
China	2015-2015	China Intercensal Population Sample Survey of One-Percent 2015
China	2020-2020	China National Population Census 2020
China	2012-2013	China Sample Survey on Population Changes 2013
China	2013-2014	China Sample Survey on Population Changes 2014
China	2015-2016	China Sample Survey on Population Changes 2016
China	2016-2017	China Sample Survey on Population Changes 2017
China	2017-2018	China Sample Survey on Population Changes 2018

China	2018-2019	China Sample Survey on Population Changes 2019
China	2020-2021	China Sample Survey on Population Changes 2021
China	2021-2022	China Sample Survey on Population Changes 2022
China	2017-2022	Hong Kong Annual Digest of Statistics 2023
China	2018-2023	Hong Kong Monthly Digest of Statistics February 2024
China	2018-2018	Hong Kong Vital Registration - Deaths 2018 ICD10
China	2019-2019	Hong Kong Vital Registration - Deaths 2019 ICD10
China	2020-2020	Hong Kong Vital Registration - Deaths 2020 ICD10
China	2021-2021	Hong Kong Vital Registration - Deaths 2021 ICD10
China	2018-2022	Macao Demographics Statistics 2022
China	2021-2023	Macao Monthly Bulletin of Statistics January 2024
Colombia	2021-2021	Colombia Vital Statistics - Deaths 2021
Colombia	2022-2022	Colombia Vital Statistics - Deaths 2022
Colombia	2023-2023	Colombia Vital Statistics - Deaths 2023
Comoros	2022-2022	Comoros Multiple Indicator Cluster Survey 2022
Cook Islands	2013-2017	Cook Islands Population Estimate and Vital Statistics: December Quarter 2017
Cook Islands	2018-2022	Cook Islands Population Estimate and Vital Statistics: December Quarter 2022
Costa Rica	2000-2023	Costa Rica Main Demographic Indicators Preliminary Data 2000-2023
Costa Rica	2020-2020	Costa Rica Registered Deaths 2020
Costa Rica	2021-2021	Costa Rica Registered Deaths 2021
Costa Rica	2022-2022	Costa Rica Registered Deaths 2022
Croatia	1998-1998	Croatia Deaths by Sex, Age, Towns and Municipalities
Croatia	2011-2023	Croatia Natural Change in Population Provisional Data December 2023
Croatia	2018-2018	Croatia Vital Registration - Deaths 2018 ICD10
Croatia	2020-2020	Croatia Vital Registration - Deaths 2020 ICD10
Croatia	2021-2021	Croatia Vital Registration - Deaths 2021 ICD10
Cuba	2020-2020	Cuba Vital Registration - Deaths 2020 ICD10
Cuba	2021-2021	Cuba Vital Registration - Deaths 2021 ICD10
Cyprus	2019-2019	Cyprus Vital Registration - Deaths 2019 ICD10
Cyprus	2020-2020	Cyprus Vital Registration - Deaths 2020 ICD10
Cyprus	2021-2021	Cyprus Vital Registration - Deaths 2021 ICD10
Czechia	2021-2021	Czech Republic Vital Registration - Deaths 2021 ICD10
Czechia	2017-2017	Czechia Deaths by Week, Sex and 5-year Age Group - Eurostat
Côte d'Ivoire	2021-2021	Côte d'Ivoire Demographic and Health Survey 2021
Côte d'Ivoire	2021-2021	Côte d'Ivoire Population and Housing Census 2021
Côte d'Ivoire	2017-2018	Côte d'Ivoire Population-Based HIV Impact Assessment 2017-2018
DR Congo	2023-2024	Democratic Republic of the Congo Demographic and Health Survey 2023-2024
Denmark	2019-2019	Denmark Vital Registration - Deaths 2019 ICD10
Denmark	2020-2020	Denmark Vital Registration - Deaths 2020 ICD10
Denmark	2021-2021	Denmark Vital Registration - Deaths 2021 ICD10
Djibouti	2012-2012	Djibouti Family Health Survey 2012
Dominica	2016-2016	Dominica Vital Registration - Deaths 2016 ICD10
Dominica	2017-2017	Dominica Vital Registration - Deaths 2017 ICD10
Dominican Republic	2001-2022	Dominican Republic Vital Statistics Yearbook 2022
Ecuador	2021-2021	Ecuador General Deaths 2021
Ecuador	2022-2022	Ecuador General Deaths 2022

Egypt	1997-2022	Egypt Statistical Yearbook 2023
Estonia	2017-2017	Estonia Deaths by Week, Reference period, and Sex
Estonia	2017-2017	Estonia Deaths by Week, Sex and 5-year Age Group - Eurostat
Estonia	2021-2021	Estonia Vital Registration - Deaths 2021 ICD10
Estonia	2022-2022	Estonia Vital Registration - Deaths 2022 ICD10
Eswatini	2021-2022	Eswatini Multiple Indicator Cluster Survey 2021-2022
Eswatini	2016-2017	Swaziland HIV Incidence Measurement Survey 2 2016-2017
Federated States of Micronesia	2018-2018	Federated States of Micronesia Vital Registration - Deaths 2018 ICD10
Federated States of Micronesia	2019-2019	Federated States of Micronesia Vital Registration - Deaths 2019 ICD10
Federated States of Micronesia	2020-2020	Federated States of Micronesia Vital Registration - Deaths 2020 ICD10
Federated States of Micronesia	2021-2021	Federated States of Micronesia Vital Registration - Deaths 2021 ICD10
Federated States of Micronesia	2022-2022	Federated States of Micronesia Vital Registration - Deaths 2022 ICD10
Fiji	2021-2021	Fiji Multiple Indicator Cluster Survey 2021
Fiji	2012-2017	Fiji Vital Statistics Report 2012-2017
Fiji	2016-2021	Fiji Vital Statistics Report 2016-2021
Finland	2023-2023	Finland Preliminary Vital Statistics by Area
Finland	2019-2019	Finland Vital Registration - Deaths 2019 ICD10
Finland	2020-2020	Finland Vital Registration - Deaths 2020 ICD10
Finland	2021-2021	Finland Vital Registration - Deaths 2021 ICD10
France	2017-2017	France Deaths by Week, Sex and 5-year Age Group - Eurostat
France	1957-2024	France Demographic Assessment 2023
France	2017-2017	France Vital Registration - Deaths 2017 ICD10
France	2018-2018	France Vital Registration - Deaths 2018 ICD10
France	2019-2019	France Vital Registration - Deaths 2019 ICD10
France	2020-2020	France Vital Registration - Deaths 2020 ICD10
Gabon	2019-2021	Gabon Demographic and Health Survey 2019-2021
Georgia	1994-2023	Georgia Basic Demographic Statistics: Births, Deaths, Marriages, Divorces 2023
Georgia	1994-2022	Georgia Deaths by Age and Sex 1994-2022
Georgia	2021-2021	Georgia Vital Registration - Deaths 2021 ICD10
Germany	2020-2020	Germany Causes of Death 2020
Germany	2021-2021	Germany Causes of Death 2021
Germany	2017-2017	Germany Deaths by Week, Sex and 5-year Age Group - Eurostat
Ghana	2022-2023	Ghana Demographic and Health Survey 2022-2023
Greece	2017-2023	Greece Data on Weekly Deaths: Period 1st to 52nd Week 2023
Greece	2000-2021	Greece Deaths by Sex and Age of the Deceased 2000-2021
Greece	2017-2017	Greece Deaths by Week, Sex and 5-year Age Group - Eurostat
Greece	2020-2020	Greece Vital Registration - Deaths 2020 ICD10
Grenada	2019-2019	Grenada Vital Registration - Deaths 2019 ICD10
Grenada	2020-2020	Grenada Vital Registration - Deaths 2020 ICD10
Grenada	2021-2021	Grenada Vital Registration - Deaths 2021 ICD10
Guam	2021-2021	Guam NVSS Custom Mortality Data 2021
Guam	2022-2022	Guam NVSS Custom Mortality Data 2022
Guam	2023-2023	Guam NVSS Custom Mortality Data 2023
Guatemala	2010-2010	Guatemala Vital Statistics 2010
Guatemala	2011-2011	Guatemala Vital Statistics 2011
Guatemala	2003-2012	Guatemala Vital Statistics 2012

Guatemala	2021-2021	Guatemala Vital Statistics 2021
Guatemala	2022-2022	Guatemala Vital Statistics 2022
Guinea	2014-2014	Guinea Population and Housing Census 2014
Guyana	2015-2015	Guyana Vital Registration - Deaths 2015 ICD10
Guyana	2016-2016	Guyana Vital Registration - Deaths 2016 ICD10
Guyana	2017-2017	Guyana Vital Registration - Deaths 2017 ICD10
Guyana	2018-2018	Guyana Vital Registration - Deaths 2018 ICD10
Guyana	2019-2019	Guyana Vital Registration - Deaths 2019 ICD10
Guyana	2009-2022	Guyana Yearly and Quarterly Deaths by Region and by Sex 2009-2022
Hungary	2023-2023	Hungary Population and Vital Events, December 2023
Hungary	2020-2020	Hungary Vital Registration - Deaths 2020 ICD10
Hungary	2021-2021	Hungary Vital Registration - Deaths 2021 ICD10
Hungary	2022-2022	Hungary Vital Registration - Deaths 2022 ICD10
Iceland	2021-2021	Iceland Causes of Death Register 2021
Iceland	2022-2022	Iceland Causes of Death Register 2022
Iceland	2017-2017	Iceland Deaths by Week, Sex and 5-year Age Group - Eurostat
India	2021-2021	India - Bihar Registration of Births and Deaths Report 2021
India	2022-2022	India - Bihar Registration of Births and Deaths Report 2022
India	2018-2018	India - Delhi Medical Certification of Cause of Death Report 2018
India	2019-2019	India - Delhi Medical Certification of Cause of Death Report 2019
India	2005-2020	India - Delhi Medical Certification of Cause of Death Report 2020
India	2021-2021	India - Delhi Registration of Births and Deaths Report 2021
India	2022-2022	India - Delhi Registration of Births and Deaths Report 2022
India	2005-2023	India - Delhi Registration of Births and Deaths Report 2023
India	2022-2022	India - Goa Medical Certification of Cause of Death Report 2022
India	2022-2022	India - Goa Registration of Births and Deaths Report 2022
India	2023-2023	India - Goa Registration of Births and Deaths Report 2023
India	2022-2023	India - Haryana Statistical Abstract 2022-2023
India	1991-2022	India - Himachal Pradesh Vital Statistics Annual Report 2022
India	1991-2023	India - Himachal Pradesh Vital Statistics Annual Report 2023
India	2018-2018	India - Karnataka Medical Certification of Cause of Death Report 2018
India	2019-2019	India - Karnataka Medical Certification of Cause of Death Report 2019
India	2020-2020	India - Karnataka Medical Certification of Cause of Death Report 2020
India	2021-2021	India - Karnataka Registration of Births and Deaths Report 2021
India	2022-2022	India - Karnataka Registration of Births and Deaths Report 2022
India	2021-2021	India - Kerala Annual Vital Statistics Report 2021
India	2018-2018	India - Kerala Medical Certification of Cause of Death Report 2018
India	2020-2020	India - Kerala Medical Certification of Cause of Death Report 2020
India	2014-2014	India - Maharashtra Survey of Causes of Deaths 2014
India	2023-2023	India - Meghalaya Registration of Births, Deaths, Infant Deaths, and Stillbirths 2023
India	2018-2018	India - Mizoram Medically Certified Causes of Death 2018
India	2019-2019	India - Mizoram Medically Certified Causes of Death 2019
India	2020-2020	India - Mizoram Medically Certified Causes of Death 2020
India	2021-2021	India - Mizoram Medically Certified Causes of Death 2021
India	2022-2022	India - Mizoram Medically Certified Causes of Death 2022
India	2021-2021	India - Mizoram Registration of Births and Deaths Report 2021

India	2022-2022	India - Mizoram Registration of Births and Deaths Report 2022
India	2023-2023	India - Mizoram Registration of Births and Deaths Report 2023
India	2014-2014	India - Odisha Medical Certification of Cause of Death Data 2014
India	2015-2015	India - Odisha Medical Certification of Cause of Death Data 2015
India	2016-2016	India - Odisha Medical Certification of Cause of Death Data 2016
India	2017-2017	India - Odisha Medical Certification of Cause of Death Data 2017
India	2018-2018	India - Odisha Medical Certification of Cause of Death Data 2018
India	2020-2020	India - Odisha Medical Certification of Cause of Death Data 2020
India	2021-2021	India - Odisha Registration of Births and Deaths 2021
India	2022-2022	India - Odisha Registration of Births and Deaths 2022
India	2019-2019	India - Rajasthan Medical Certification of Cause of Deaths 2019
India	2021-2021	India - Uttarakhand Civil Registration System Report of Births and Deaths 2021
India	2020-2021	India Every Newborn Health Assessment and Neonatal Care Evaluation (ENHANCE) 2020-2021
India	2004-2005	India Human Development Survey 2004-2005 - ICPSR
India	2009-2021	India SRS Statistical Report 2021
India	2007-2007	India WHO Study on Global Ageing and Adult Health 2007
India	2011-2013	Population-based study of neonatal mortality and maternal care utilization in the Indian state of Bihar
India	2016-2016	Vital Statistics of India Based on the Civil Registration System 2016
India	2017-2017	Vital Statistics of India Based on the Civil Registration System 2017
India	1987-2018	Vital Statistics of India Based on the Civil Registration System 2018
India	1988-2019	Vital Statistics of India Based on the Civil Registration System 2019
India	1989-2020	Vital Statistics of India Based on the Civil Registration System 2020
Indonesia	2014-2015	Indonesia Family Life Survey 2014-2015
Iran	2017-2017	Iran Civil Registration Recorded Deaths by Age Group, Sex, and Province 2017
Iran	2018-2018	Iran Civil Registration Recorded Deaths by Age Group, Sex, and Province 2018
Iran	2019-2019	Iran Civil Registration Recorded Deaths by Age Group, Sex, and Province 2019
Iran	2020-2020	Iran Civil Registration Recorded Deaths by Age Group, Sex, and Province 2020
Iran	1956-2018	Iran Statistical Yearbook 2017-2018
Iran	1956-2019	Iran Statistical Yearbook 2018-2019
Iran	1956-2020	Iran Statistical Yearbook 2019-2020
Iran	1956-2022	Iran Statistical Yearbook 2021-2022
Iran	2017-2017	Iran Vital Registration - Deaths 2017 ICD10
Iraq	2020-2020	Iraq Annual Statistical Report 2020
Iraq	2021-2021	Iraq Annual Statistical Report 2021
Iraq	2005-2023	Iraq Annual Statistical Report 2023
Iraq	2022-2022	Iraq Annual Statistical Report with Kurdistan Region 2022
Iraq	2012-2013	Iraq Household Socioeconomic Survey 2012-2013
Ireland	2016-2016	Ireland Vital Registration - Deaths 2016 ICD10
Ireland	2017-2017	Ireland Vital Registration - Deaths 2017 ICD10
Ireland	2019-2019	Ireland Vital Registration - Deaths 2019 ICD10
Ireland	2020-2020	Ireland Vital Registration - Deaths 2020 ICD10
Israel	2000-2022	Israel Deaths by Sub-District, Sex, and Age Group 2000-2022
Israel	1996-2023	Israel Monthly Bulletin of Statistics February 2024
Israel	2019-2019	Israel Vital Registration - Deaths 2019 ICD10
Israel	2020-2020	Israel Vital Registration - Deaths 2020 ICD10
Italy	2011-2023	Italy Municipal Deaths by Month, Sex and Age Group 2011-2023

Italy	2021-2021	Italy Vital Registration - Deaths 2021
Jamaica	2015-2015	Jamaica Vital Registration Tables 2015
Jamaica	2016-2016	Jamaica Vital Registration Tables 2016
Jamaica	2017-2017	Jamaica Vital Registration Tables 2017
Jamaica	2018-2018	Jamaica Vital Registration Tables 2018
Japan	2021-2021	Japan Vital Registration - Deaths 2021
Japan	2022-2022	Japan Vital Registration - Deaths 2022
Jordan	2023-2023	Jordan Demographic and Health Survey 2023
Jordan	2000-2022	Jordan Registered Deaths 2000-2022
Kazakhstan	2018-2022	Kazakhstan Demographic Yearbook 2018-2022
Kazakhstan	2022-2023	Kazakhstan Natural Movement of the Population Deaths by Month 2023
Kazakhstan	2021-2021	Kazakhstan Vital Registration - Deaths 2021 ICD10
Kenya	2022-2022	Kenya Demographic and Health Survey 2022
Kenya	2015-2016	Kenya Integrated Household Budget Survey 2015-2016
Kosovo	2019-2020	Kosovo Multiple Indicator Cluster Survey 2019-2020
Kuwait	2021-2021	Kuwait Annual Bulletin for Vital Statistics - Births and Deaths 2021
Kuwait	2018-2022	Kuwait Annual Bulletin for Vital Statistics - Births and Deaths 2022
Kuwait	2023-2023	Kuwait Annual Bulletin for Vital Statistics - Births and Deaths 2023
Kyrgyzstan	2018-2022	Kyrgyzstan Demographic Yearbook 2018-2022
Kyrgyzstan	2011-2023	Kyrgyzstan Number of Deaths 2011-2023
Latvia	1950-1950	Latvia Deaths by Sex and Month
Latvia	2017-2017	Latvia Deaths by Week, Sex and 5-year Age Group - Eurostat
Latvia	2021-2021	Latvia Vital Registration - Deaths 2021 ICD10
Lebanon	2020-2020	Lebanon Vital Registration - Deaths 2020 ICD10
Lebanon	2021-2021	Lebanon Vital Registration - Deaths 2021 ICD10
Lesotho	2023-2024	Lesotho Demographic and Health Survey 2023-2024
Lithuania	2000-2000	Lithuania Deaths by Age Group, Gender, and Week
Lithuania	2017-2017	Lithuania Deaths by Week, Sex and 5-year Age Group - Eurostat
Lithuania	2021-2021	Lithuania Vital Registration - Deaths 2021 ICD10
Lithuania	2022-2022	Lithuania Vital Registration - Deaths 2022 ICD10
Luxembourg	2017-2017	Luxembourg Deaths by Week, Sex and 5-year Age Group - Eurostat
Luxembourg	2018-2018	Luxembourg Vital Registration - Deaths 2018 ICD10
Luxembourg	2020-2020	Luxembourg Vital Registration - Deaths 2020 ICD10
Luxembourg	2021-2021	Luxembourg Vital Registration - Deaths 2021 ICD10
Luxembourg	2022-2022	Luxembourg Vital Registration - Deaths 2022 ICD10
Malawi	2024-2024	Malawi Demographic and Health Survey 2024
Malawi	2010-2016	Malawi Integrated Household Panel Survey, Long-Term Panel, 2010-2016
Malawi	2010-2019	Malawi Integrated Household Panel Survey, Long-Term Panel, 2010-2019
Malawi	2004-2005	Malawi Integrated Household Survey 2004-2005
Malawi	2018-2018	Malawi Population and Housing Census 2018
Malawi	2015-2016	Malawi Population-Based HIV Impact Assessment 2015-2016
Malaysia	1998-1998	Malaysia - Peninsular Vital Statistics - Deaths 1998
Malaysia	2023-2023	Malaysia Demographic Statistics Quarter 1 2023
Malaysia	2023-2023	Malaysia Demographic Statistics Quarter 2 2023
Malaysia	2023-2023	Malaysia Demographic Statistics Quarter 3 2023
Malaysia	2023-2023	Malaysia Demographic Statistics Quarter 4 2023

Malaysia	2000-2000	Malaysia Vital Registration - Deaths 2000 ICD10
Malaysia	2001-2001	Malaysia Vital Registration - Deaths 2001 ICD10
Malaysia	2002-2002	Malaysia Vital Registration - Deaths 2002 ICD10
Malaysia	2003-2003	Malaysia Vital Registration - Deaths 2003 ICD10
Malaysia	2004-2004	Malaysia Vital Registration - Deaths 2004 ICD10
Malaysia	2005-2005	Malaysia Vital Registration - Deaths 2005 ICD10
Malaysia	2006-2006	Malaysia Vital Registration - Deaths 2006 ICD10
Malaysia	2007-2007	Malaysia Vital Registration - Deaths 2007 ICD10
Malaysia	2008-2008	Malaysia Vital Registration - Deaths 2008 ICD10
Malaysia	2015-2015	Malaysia Vital Registration - Deaths 2015 ICD10
Malaysia	2016-2016	Malaysia Vital Registration - Deaths 2016 ICD10
Malaysia	2017-2017	Malaysia Vital Registration - Deaths 2017 ICD10
Malaysia	2018-2018	Malaysia Vital Registration - Deaths 2018 ICD10
Malaysia	2019-2019	Malaysia Vital Registration - Deaths 2019 ICD10
Malaysia	2020-2020	Malaysia Vital Registration - Deaths 2020 ICD10
Malaysia	2021-2021	Malaysia Vital Statistics 2022
Malaysia	2022-2022	Malaysia Vital Statistics 2023
Maldives	1911-2030	Maldives Statistical Yearbook 2022
Mali	2023-2024	Mali Demographic and Health Survey 2023-2024
Malta	2018-2018	Malta Vital Registration - Deaths 2018 ICD10
Malta	2019-2019	Malta Vital Registration - Deaths 2019 ICD10
Malta	2020-2020	Malta Vital Registration - Deaths 2020 ICD10
Mauritius	2000-2022	Mauritius Digest of Demographic Statistics 2022
Mauritius	2009-2023	Mauritius Digest of Demographic Statistics 2023
Mauritius	2021-2021	Mauritius Vital Registration - Deaths 2021 ICD10
Mauritius	2022-2022	Mauritius Vital Registration - Deaths 2022 ICD10
Mexico	1997-1997	Mexico National Survey of Demographic Dynamics 1997
Mexico	2023-2023	Mexico National Survey of Demographic Dynamics 2023
Mexico	2022-2022	Mexico Vital Registration - Deaths 2022
Mexico	2023-2023	Mexico Vital Registration - Deaths 2023
Mexico	2022-2022	Mexico Vital Registration - Multiple Causes of Death 2022
Monaco	2005-2021	Monaco Deaths by Five-Year Age Group and Sex 2005-2021
Monaco	2022-2022	Monaco Deaths by Five-Year Age Group and Sex 2022
Monaco	2004-2013	Monaco In Figures 2014
Monaco	2005-2014	Monaco In Figures 2015
Monaco	2006-2015	Monaco In Figures 2016
Monaco	2007-2016	Monaco In Figures 2017
Monaco	2008-2017	Monaco In Figures 2018
Monaco	2009-2018	Monaco In Figures 2019
Monaco	2010-2019	Monaco In Figures 2020
Monaco	2011-2020	Monaco In Figures 2021
Monaco	2012-2021	Monaco In Figures 2022
Monaco	2014-2023	Monaco In Figures 2024
Mongolia	2023-2023	Mongolia Births and Deaths in Population 2023
Mongolia	2017-2017	Mongolia Vital Registration - Deaths 2017 ICD10
Mongolia	2021-2021	Mongolia Vital Registration - Deaths 2021

Mongolia	2022-2022	Mongolia Vital Registration - Deaths 2022
Morocco	1971-2021	Morocco Statistical Yearbook 2022
Mozambique	2018-2022	Mozambique Countrywide Mortality Surveillance for Action Data 2018-2022
Mozambique	2022-2023	Mozambique Demographic and Health Survey 2022-2023
Mozambique	2007-2007	Mozambique Population and Housing Census 2007
Myanmar	2006-2021	Myanmar Statistical Yearbook 2022
Myanmar	2009-2009	Myanmar Vital Registration - Deaths by State/Region, Sex, Age, and Cause 2009
Myanmar	2010-2010	Myanmar Vital Registration - Deaths by State/Region, Sex, Age, and Cause 2010
Myanmar	2011-2011	Myanmar Vital Registration - Deaths by State/Region, Sex, Age, and Cause 2011
Myanmar	2012-2012	Myanmar Vital Registration - Deaths by State/Region, Sex, Age, and Cause 2012
Myanmar	2013-2013	Myanmar Vital Registration - Deaths by State/Region, Sex, Age, and Cause 2013
Myanmar	2014-2014	Myanmar Vital Registration - Deaths by State/Region, Sex, Age, and Cause 2014
Myanmar	2015-2015	Myanmar Vital Registration - Deaths by State/Region, Sex, Age, and Cause 2015
Myanmar	2016-2016	Myanmar Vital Registration - Deaths by State/Region, Sex, Age, and Cause 2016
Myanmar	2017-2017	Myanmar Vital Registration - Deaths by State/Region, Sex, Age, and Cause 2017
Namibia	2016-2017	Namibia Mortality and Causes of Death Report 2016-2017
Namibia	2018-2021	Namibia Mortality and Causes of Death Report 2018-2021
Namibia	2017-2017	Namibia Population-Based HIV Impact Assessment 2017
Nepal	2022-2022	Nepal Demographic and Health Survey 2022
Nepal	2021-2021	Nepal Population and Housing Census 2021
Netherlands	2023-2023	Netherlands Number of Deaths 2023
Netherlands	2021-2021	Netherlands Vital Registration - Deaths 2021 ICD10
Netherlands	2022-2022	Netherlands Vital Registration - Deaths 2022 ICD10
New Zealand	2023-2023	New Zealand Births and Deaths 2023
New Zealand	2019-2019	New Zealand Mortality Collection 2019
New Zealand	2023-2023	New Zealand Mortality Collection 2023 (Provisional)
Nicaragua	2020-2021	Nicaragua Compendium of Vital Statistics 2020-2021
Nicaragua	2021-2022	Nicaragua Compendium of Vital Statistics 2021-2022
Nicaragua	2020-2020	Nicaragua Vital Registration - Deaths 2020 ICD10
Nicaragua	2021-2021	Nicaragua Vital Registration - Deaths 2021 ICD10
Nigeria	2023-2024	Nigeria Demographic and Health Survey 2023-2024
Nigeria	2009-2009	Nigeria General Household Survey 2009
Nigeria	2010-2011	Nigeria General Household Survey 2010-2011
Nigeria	2015-2016	Nigeria General Household Survey Panel 2015-2016 Wave 3
Nigeria	2012-2013	Nigeria General Household Survey, Panel 2012-2013, Wave 2
Nigeria	2021-2021	Nigeria Multiple Indicator Cluster Survey and National Immunization Coverage Survey 2021
Nigeria	2016-2017	Nigeria Multiple Indicator Cluster Survey with National Immunization Coverage Survey Supplement 2016-2017 - National Bureau of Statistics
Niue	2012-2016	Niue Vital Statistics Report 2012-2016
Niue	2021-2021	Niue Vital Statistics Report 2021
Niue	2022-2022	Niue Vital Statistics Report 2022
Niue	2017-2017	Niue Vital Statistics Report December 2017
Niue	2018-2018	Niue Vital Statistics Report December 2018
Niue	2019-2019	Niue Vital Statistics Report December 2019
Niue	2020-2020	Niue Vital Statistics Report December 2020
Niue	2017-2017	Niue Vital Statistics Report June 2017
Niue	2018-2018	Niue Vital Statistics Report June 2018

Niue	2019-2019	Niue Vital Statistics Report June 2019
Niue	2020-2020	Niue Vital Statistics Report June 2020
North Macedonia	2018-2019	North Macedonia - Roma Settlements Multiple Indicator Cluster Survey 2018-2019
North Macedonia	2018-2019	North Macedonia Multiple Indicator Cluster Survey 2018-2019
North Macedonia	1972-2022	North Macedonia Statistical Yearbook 2023
North Macedonia	2021-2021	North Macedonia Vital Registration - Deaths 2021 ICD10
Northern Mariana Islands	2021-2021	Northern Mariana Islands NVSS Custom Mortality Data 2021
Northern Mariana Islands	2022-2022	Northern Mariana Islands NVSS Custom Mortality Data 2022
Northern Mariana Islands	2023-2023	Northern Mariana Islands NVSS Custom Mortality Data 2023
Norway	2021-2021	Norway Cause of Death Registry 2021
Norway	2022-2022	Norway Cause of Death Registry 2022
Oman	2006-2022	Oman Population Deaths (No.) by Region, Nationality, Sex, and Age Groups 2006-2022
Oman	2014-2023	Oman Statistical Yearbook 2024
Oman	2018-2018	Oman Vital Registration - Deaths 2018 ICD10
Oman	2019-2019	Oman Vital Registration - Deaths 2019 ICD10
Oman	2021-2021	Oman Vital Registration - Deaths 2021 ICD10
Pakistan	2020-2021	Pakistan - Azad Jammu and Kashmir Multiple Indicator Cluster Survey 2020-2021
Pakistan	2019-2020	Pakistan - Balochistan Multiple Indicator Cluster Survey 2019 - 2020
Pakistan	2019-2019	Pakistan - Khyber Pakhtunkhwa Multiple Indicator Cluster Survey 2019
Pakistan	2019-2019	Pakistan Special Demographic and Health Survey 2019
Palau	2005-2021	Palau Statistical Yearbook 2021
Palau	1995-2022	Palau Statistical Yearbook 2022
Palau	2005-2023	Palau Statistical Yearbook 2023
Palestine	2019-2019	Palestine - Gaza Strip Mortality by Sex, Age, and Cause 2019
Palestine	2020-2020	Palestine - Gaza Strip Mortality by Sex, Age, and Cause 2020
Palestine	2021-2021	Palestine - Gaza Strip Mortality by Sex, Age, and Cause 2021
Palestine	2019-2019	Palestine - West Bank Mortality by Sex, Age, and Cause 2019
Palestine	2020-2020	Palestine - West Bank Mortality by Sex, Age, and Cause 2020
Palestine	2021-2021	Palestine - West Bank Mortality by Sex, Age, and Cause 2021
Palestine	2005-2022	Palestine Health Annual Report 2022
Panama	2021-2021	Panama Vital Registration - Deaths 2021 ICD10
Panama	2021-2021	Panama Vital Statistics, Volume III - Deaths 2021
Panama	2018-2022	Panama Vital Statistics, Volume III - Deaths 2022
Papua New Guinea	2022-2022	Papua New Guinea Vital Registration - Deaths 2022 ICD10
Paraguay	2004-2004	Paraguay Vital Registration - Deaths 2004
Paraguay	2005-2005	Paraguay Vital Registration - Deaths 2005
Paraguay	2006-2006	Paraguay Vital Registration - Deaths 2006
Paraguay	2007-2007	Paraguay Vital Registration - Deaths 2007
Paraguay	2008-2008	Paraguay Vital Registration - Deaths 2008
Paraguay	2009-2009	Paraguay Vital Registration - Deaths 2009
Paraguay	2010-2010	Paraguay Vital Registration - Deaths 2010
Paraguay	2011-2011	Paraguay Vital Registration - Deaths 2011
Paraguay	2012-2012	Paraguay Vital Registration - Deaths 2012
Paraguay	2013-2013	Paraguay Vital Registration - Deaths 2013
Paraguay	2014-2014	Paraguay Vital Registration - Deaths 2014
Paraguay	2020-2020	Paraguay Vital Registration - Deaths 2020 ICD10

Paraguay	2021-2021	Paraguay Vital Registration - Deaths 2021 ICD10
Paraguay	2010-2021	Paraguay Vital Statistics 2021
Peru	2017-2023	Peru Death Information System (SINADEF) - System Report of Deaths 2023
Peru	2018-2018	Peru Demographic and Family Health Survey 2018
Peru	2019-2019	Peru Demographic and Family Health Survey 2019
Peru	2020-2020	Peru Demographic and Family Health Survey 2020
Peru	2021-2021	Peru Demographic and Family Health Survey 2021
Peru	2022-2022	Peru Demographic and Family Health Survey 2022
Peru	2019-2019	Peru Vital Registration - Deaths 2019 ICD10
Peru	2020-2020	Peru Vital Registration - Deaths 2020 ICD10
Peru	2021-2022	Perú Birth, Mortality and Marriage, 2022
Philippines	2022-2023	Philippines Birth, Marriage, and Death Statistics for 2023 (Provisional, as of 31 May 2024)
Philippines	2022-2022	Philippines Demographic and Health Survey 2022
Philippines	1993-1993	Philippines In Depth Demographic and Health Survey 1993
Philippines	2021-2021	Philippines Vital Registration - Deaths 2021
Philippines	2022-2022	Philippines Vital Registration - Deaths 2022
Poland	2023-2023	Poland Deaths by Week and Age, Sex of the Deceased and 73 Subregions 2023
Poland	2017-2017	Poland Deaths by Week, Sex and 5-year Age Group - Eurostat
Poland	2020-2020	Poland Vital Registration - Causes of Death Data 2020
Poland	2021-2021	Poland Vital Registration - Causes of Death Data 2021
Poland	2022-2022	Poland Vital Registration - Causes of Death Data 2022
Portugal	2023-2023	Portugal Deaths by Sex and Month
Portugal	1980-1980	Portugal Vital Registration - Deaths 1980
Portugal	1981-1981	Portugal Vital Registration - Deaths 1981
Portugal	1982-1982	Portugal Vital Registration - Deaths 1982
Portugal	1983-1983	Portugal Vital Registration - Deaths 1983
Portugal	2019-2019	Portugal Vital Registration - Deaths 2019 ICD10
Puerto Rico	2022-2022	Puerto Rico Islands NVSS Custom Mortality Data 2022
Puerto Rico	2021-2021	Puerto Rico NVSS Custom Mortality Data 2021
Puerto Rico	2023-2023	Puerto Rico NVSS Custom Mortality Data 2023
Qatar	2021-2021	Qatar Vital Registration - Deaths 2021 ICD10
Qatar	2022-2022	Qatar Vital Registration - Deaths 2022 ICD10
Qatar	2012-2022	Qatar Vital Statistics Annual Bulletin 2022
Romania	2023-2023	Romania Demographic Events in 2023
Russia	2015-2017	Russia Mortality Rates by Region, 1-Year Age Groups, and Sex 2015-2017
Russia	2021-2021	Russia Mortality by Region, Age, Sex, and Cause of Death 2021
Russia	2022-2022	Russia Mortality by Region, Age, Sex, and Cause of Death 2022
Russia	2022-2023	Russia Natural Movement of the Population 2023
Russia	2004-2004	Russia Vital Registration - Deaths 2004
Russia	2005-2005	Russia Vital Registration - Deaths 2005
Russia	2006-2006	Russia Vital Registration - Deaths 2006
Russia	2007-2007	Russia Vital Registration - Deaths 2007
Russia	2008-2008	Russia Vital Registration - Deaths 2008
Russia	2009-2009	Russia Vital Registration - Deaths 2009
Russia	2010-2010	Russia Vital Registration - Deaths 2010
Russia	2011-2011	Russia Vital Registration - Deaths 2011

Russia	2012-2012	Russia Vital Registration - Deaths 2012
Russia	2013-2013	Russia Vital Registration - Deaths 2013
Russia	2014-2014	Russia Vital Registration - Deaths 2014
Rwanda	2018-2019	Rwanda Population-Based HIV Impact Assessment 2018-2019
Saint Kitts and Nevis	2017-2017	Saint Kitts and Nevis Reported Deaths by Parish 2017
Saint Kitts and Nevis	2018-2018	Saint Kitts and Nevis Reported Deaths by Parish 2018
Saint Kitts and Nevis	2019-2019	Saint Kitts and Nevis Reported Deaths by Parish 2019
Saint Kitts and Nevis	2020-2020	Saint Kitts and Nevis Reported Deaths by Parish 2020
Saint Kitts and Nevis	2021-2021	Saint Kitts and Nevis Reported Deaths by Parish 2021
Saint Kitts and Nevis	2022-2022	Saint Kitts and Nevis Reported Deaths by Parish 2022
Saint Lucia	2016-2016	Saint Lucia Vital Registration - Deaths 2016 ICD10
Saint Lucia	2017-2017	Saint Lucia Vital Registration - Deaths 2017 ICD10
Saint Lucia	2018-2018	Saint Lucia Vital Registration - Deaths 2018 ICD10
Saint Lucia	2019-2019	Saint Lucia Vital Registration - Deaths 2019 ICD10
Saint Lucia	2020-2020	Saint Lucia Vital Registration - Deaths 2020 ICD10
Saint Vincent and the Grenadines	2018-2018	Saint Vincent and the Grenadines Vital Registration - Deaths 2018 ICD10
Saint Vincent and the Grenadines	2019-2019	Saint Vincent and the Grenadines Vital Registration - Deaths 2019 ICD10
Saint Vincent and the Grenadines	2020-2020	Saint Vincent and the Grenadines Vital Registration - Deaths 2020 ICD10
Saint Vincent and the Grenadines	2021-2021	Saint Vincent and the Grenadines Vital Registration - Deaths 2021 ICD10
Samoa	2016-2016	Samoa Population and Housing Census 2016
Samoa	1906-2017	Samoa Statistical Abstract 2017
Samoa	1966-2020	Samoa Statistical Abstract 2020
Samoa	1966-2022	Samoa Statistical Abstract 2022
San Marino	2023-2023	San Marino Deaths by Age and Gender 2023
San Marino	2018-2022	San Marino Supplement to the Statistical Bulletin 2022
Saudi Arabia	2016-2016	Saudi Arabia Demographic Survey 2016
Saudi Arabia	2019-2019	Saudi Arabia Vital Registration - Deaths 2019
Saudi Arabia	2021-2021	Saudi Arabia Vital Registration - Deaths 2021
Saudi Arabia	2022-2022	Saudi Arabia Vital Registration - Deaths 2022
Senegal	2023-2023	Senegal Continuous Demographic and Health Survey 2023
Senegal	1999-2000	Senegal Demographic and Health Survey 1999-2000
Senegal	2023-2023	Senegal Population and Housing Census 2023
Serbia	2023-2024	Serbia Population Statistics - Live Births and Deaths January 2024
Serbia	2021-2021	Serbia Vital Registration - Deaths 2021 ICD10
Serbia	2022-2022	Serbia Vital Registration - Deaths 2022 ICD10
Seychelles	1980-2023	Seychelles Statistical Bulletin: Population and Vital Statistics 2023
Sierra Leone	2016-2020	Sierra Leone Healthy Sierra Leone (HEAL-SL) Data on Mortality and Causes of Death 2016-2020
Singapore	2012-2012	Singapore Causes of Death 2012
Singapore	2013-2013	Singapore Causes of Death 2013
Singapore	2014-2014	Singapore Causes of Death 2014
Singapore	2015-2015	Singapore Causes of Death 2015
Singapore	2015-2023	Singapore Demographic Bulletin 1st Quarter January-March 2023
Singapore	2015-2023	Singapore Demographic Bulletin 2nd Quarter April-June 2023
Singapore	2016-2023	Singapore Demographic Bulletin 3rd Quarter July-September 2023
Singapore	2016-2023	Singapore Demographic Bulletin 4th Quarter October-December 2023
Singapore	2003-2022	Singapore Report on Registration of Births and Deaths 2022

Singapore	2021-2021	Singapore Vital Registration - Deaths 2021 ICD10
Singapore	2022-2022	Singapore Vital Registration - Deaths 2022 ICD10
Slovakia	2000-2000	Slovakia Deaths by Month of Death, Age, Sex and Causes of Death - SR, Areas, Regions
Slovakia	2016-2016	Slovakia Vital Registration - Deaths 2016 ICD10
Slovakia	2017-2017	Slovakia Vital Registration - Deaths 2017 ICD10
Slovakia	2018-2018	Slovakia Vital Registration - Deaths 2018 ICD10
Slovakia	2019-2019	Slovakia Vital Registration - Deaths 2019 ICD10
Slovakia	2020-2020	Slovakia Vital Registration - Deaths 2020 ICD10
Slovakia	2021-2021	Slovakia Vital Registration - Deaths 2021 ICD10
Slovakia	2022-2022	Slovakia Vital Registration - Deaths 2022
Slovenia	2000-2000	Slovenia Deaths by Day of Death, Monthly
South Africa	2016-2016	South Africa General Household Survey 2016
South Africa	2015-2024	South Africa Monthly Report on Weekly Numbers of Deaths December 2024
South Africa	2008-2008	South Africa National Income Dynamics Study - Wave 1 2008
South Africa	2012-2012	South Africa National Income Dynamics Study - Wave 3 2012
South Africa	2019-2023	South Africa Report on Weekly Deaths - Epi Week 52 - December 2023
South Africa	2018-2018	South Africa Vital Registration - Causes of Death 2018
South Africa	2019-2019	South Africa Vital Registration - Causes of Death 2019
South Africa	2007-2008	South Africa WHO Study on Global AGEing and Adult Health 2007-2008
South Korea	1981-1981	South Korea KOSIS Database - Vital Statistics by Month for Provinces
South Korea	1997-1997	South Korea Vital Statistics Causes of Death Data 1997
South Korea	1998-1998	South Korea Vital Statistics Causes of Death Data 1998
South Korea	1999-1999	South Korea Vital Statistics Causes of Death Data 1999
South Korea	2009-2009	South Korea Vital Statistics Causes of Death Data 2009
South Korea	2010-2010	South Korea Vital Statistics Causes of Death Data 2010
South Korea	2011-2011	South Korea Vital Statistics Causes of Death Data 2011
South Korea	2012-2012	South Korea Vital Statistics Causes of Death Data 2012
South Korea	2013-2013	South Korea Vital Statistics Causes of Death Data 2013
South Korea	2014-2014	South Korea Vital Statistics Causes of Death Data 2014
South Korea	2015-2015	South Korea Vital Statistics Causes of Death Data 2015
South Korea	2016-2016	South Korea Vital Statistics Causes of Death Data 2016
South Korea	2017-2017	South Korea Vital Statistics Causes of Death Data 2017
South Korea	2018-2018	South Korea Vital Statistics Causes of Death Data 2018
South Korea	2019-2019	South Korea Vital Statistics Causes of Death Data 2019
South Korea	2020-2020	South Korea Vital Statistics Causes of Death Data 2020
South Korea	2021-2021	South Korea Vital Statistics Causes of Death Data 2021
Spain	2009-2009	Spain Monthly Accumulated Deaths and Absolute Difference in Accumulated Deaths by Sex and Age, National Total and Provinces
Spain	2021-2021	Spain Vital Registration - Deaths 2021 ICD10
Sri Lanka	1968-2020	Sri Lanka Statistical Abstract 2021
Sri Lanka	2015-2015	Sri Lanka Vital Registration - Deaths 2015 ICD10
Sri Lanka	2019-2019	Sri Lanka Vital Registration - Deaths 2019 ICD10
Sri Lanka	2009-2009	Sri Lanka Vital Statistics - Births and Deaths 2009
Sri Lanka	2015-2015	Sri Lanka Vital Statistics - Deaths 2015
Sri Lanka	2019-2019	Sri Lanka Vital Statistics - Deaths 2019
Sri Lanka	2019-2023	Sri Lanka Vital Statistics - Number of Births, Deaths and Marriages by District 2019-2023
Sri Lanka	2019-2024	Sri Lanka Vital Statistics - Number of Births, Deaths and Marriages by District 2019-2024

Sudan	2008-2008	Sudan Population and Housing Census 2008
Suriname	2000-2022	Suriname Statistical Yearbook 2020-2022
Sweden	1980-1980	Sweden Cause of Death Register 1980
Sweden	1981-1981	Sweden Cause of Death Register 1981
Sweden	1982-1982	Sweden Cause of Death Register 1982
Sweden	1983-1983	Sweden Cause of Death Register 1983
Sweden	1984-1984	Sweden Cause of Death Register 1984
Sweden	1985-1985	Sweden Cause of Death Register 1985
Sweden	1986-1986	Sweden Cause of Death Register 1986
Sweden	2000-2000	Sweden Population Statistics by Region, Sex, Month
Sweden	2020-2020	Sweden Vital Registration - Deaths 2020 ICD10
Sweden	2021-2021	Sweden Vital Registration - Deaths 2021 ICD10
Sweden	2022-2022	Sweden Vital Registration - Deaths 2022 ICD10
Switzerland	2023-2023	Switzerland Deaths per Week by 5-Year Age Group, Sex and Canton 2023
Switzerland	2019-2019	Switzerland Vital Registration - Deaths 2019 ICD10
Switzerland	2020-2020	Switzerland Vital Registration - Deaths 2020 ICD10
Switzerland	2021-2021	Switzerland Vital Registration - Deaths 2021 ICD10
Taiwan*	2023-2023	Taiwan Demographic Statistics Quarterly (October-December 2023)
Tajikistan	2023-2023	Tajikistan Demographic and Health Survey 2023
Tajikistan	2000-2020	Tajikistan Number of Deaths, Thousand People 2000-2020
Tanzania	2022-2022	Tanzania Demographic and Health Survey 2022
Tanzania	2016-2017	Tanzania HIV Impact Survey 2016-2017
Tanzania	2019-2020	Tanzania National Panel Survey (NPS) 2019-2020 Extended Panel with Sex Disaggregated Data
Tanzania	2010-2011	Tanzania National Panel Survey 2010-2011
Tanzania	2012-2013	Tanzania National Panel Survey 2012-2013
Tanzania	2014-2016	Tanzania National Panel Survey 2014-2016
Tanzania	2020-2022	Tanzania National Panel Survey 2020-2022
Thailand	2020-2020	Thailand Annual Number of Deaths by Age 2020
Thailand	2021-2021	Thailand Annual Number of Deaths by Age 2021
Thailand	2022-2022	Thailand Annual Number of Deaths by Age 2022
Thailand	2023-2023	Thailand Statistics on Number of Deaths 2023
The Bahamas	2004-2017	Bahamas Vital Statistics Report 2017
Trinidad and Tobago	2022-2022	Trinidad and Tobago Multiple Indicator Cluster Survey 2022
Tunisia	2021-2023	Tunisia Monthly Statistics Bulletin May 2023
Tunisia	2023-2023	Tunisia Multiple Indicator Cluster Survey 2023
Tunisia	1970-2018	Tunisia Statistical Yearbook 2013-2017
Tunisia	1970-2020	Tunisia Statistical Yearbook 2015-2019
Tunisia	1970-2022	Tunisia Statistical Yearbook 2017-2021
Tunisia	1970-2022	Tunisia Statistical Yearbook 2018-2022
Tunisia	2020-2020	Tunisia Vital Registration - Deaths 2020 ICD10
Tunisia	2021-2021	Tunisia Vital Registration - Deaths 2021 ICD10
Tuvalu	2018-2020	Tuvalu Civil Registration and Vital Statistics Report 2018–2020
Tuvalu	2021-2021	Tuvalu Civil Registration and Vital Statistics Report 2021
Tuvalu	2018-2023	Tuvalu Social Statistics Report 2023 - Release 2-2024
Türkiye	2018-2019	Turkey Demographic and Health Survey 2018-2019
Türkiye	2009-2021	Turkey Mortality and Cause of Death Statistics 2021

Türkiye	2009-2022	Turkey Mortality and Cause of Death Statistics 2022
Türkiye	2009-2023	Turkey Mortality and Cause of Death Statistics 2023
Türkiye	2017-2017	Turkey Vital Registration - Deaths 2017 ICD10
Türkiye	2018-2018	Turkey Vital Registration - Deaths 2018 ICD10
UK	2023-2023	United Kingdom - England and Wales Deaths Registered Monthly December 2023
UK	2022-2022	United Kingdom - England and Wales Mortality Statistics 2022
UK	2010-2021	United Kingdom - Northern Ireland Registrar General Annual Report 2021
UK	2010-2022	United Kingdom - Northern Ireland Registrar General Annual Report 2022
UK	2020-2020	United Kingdom - Northern Ireland Vital Registration - Deaths 2020 ICD10
UK	2021-2021	United Kingdom - Northern Ireland Vital Registration - Deaths 2021 ICD10
UK	2023-2023	United Kingdom - Northern Ireland Weekly Death Registrations 2023
UK	2005-2023	United Kingdom - Scotland Monthly Mortality Analysis as of December 2023
UK	2022-2022	United Kingdom - Scotland Vital Events Reference Tables 2022
UK	2020-2020	United Kingdom - Scotland Vital Registration - Deaths 2020 ICD10
UK	2021-2021	United Kingdom - Scotland Vital Registration - Deaths 2021 ICD10
UK	2023-2023	United Kingdom - Wales Deaths Registered Weekly 2023 (Provisional)
USA	2023-2023	United States CDC Wonder Provisional Mortality Statistics 2023
USA	2021-2021	United States NVSS Custom Mortality Data 2021
USA	2022-2022	United States NVSS Custom Mortality Data 2022
USA	2023-2023	United States NVSS Custom Mortality Data 2023
USA	2018-2018	United States NVSS Mortality Data 2018
USA	1979-1979	United States Vital Registration - Deaths 1979 ICD9
Uganda	2024-2024	Uganda Population and Housing Census 2024
Uganda	2016-2017	Uganda Population-Based HIV Impact Assessment 2016-2017
Ukraine	2021-2021	Ukraine - Republic of Crimea Mortality by Region, Age, Sex, and Cause of Death 2021
Ukraine	2022-2022	Ukraine - Republic of Crimea Mortality by Region, Age, Sex, and Cause of Death 2022
Ukraine	1991-2022	Ukraine Demographic Yearbook 2021
Ukraine	2013-2013	Ukraine Vital Registration - Deaths 2013
United Arab Emirates	2018-2018	United Arab Emirates Vital Registration - Deaths 2018 ICD10
United Arab Emirates	1977-2022	United Arab Emirates Vital Statistics - Births and Deaths 2022
Uruguay	2014-2023	Uruguay Birth and Infant Mortality Preliminary Report 2023
Uruguay	1980-2022	Uruguay National Statistical Yearbook 2023
Uruguay	2020-2020	Uruguay Vital Registration - Deaths 2020 ICD10
Uzbekistan	2021-2022	Uzbekistan Multiple Indicator Cluster Survey 2021-2022
Uzbekistan	2000-2022	Uzbekistan Number of Deceased Men 2000-2022
Uzbekistan	2010-2023	Uzbekistan Number of Deceased Men 2010-2023
Uzbekistan	2000-2022	Uzbekistan Number of Deceased Women 2000-2022
Uzbekistan	2010-2023	Uzbekistan Number of Deceased Women 2010-2023
Vanuatu	2023-2023	Vanuatu Multiple Indicator Cluster Survey 2023
Viet Nam	2020-2021	Vietnam Multiple Indicator Cluster Survey 2020-2021
Virgin Islands	2021-2021	United States Virgin Islands NVSS Custom Mortality Data 2021
Virgin Islands	2022-2022	United States Virgin Islands NVSS Custom Mortality Data 2022
Virgin Islands	2023-2023	United States Virgin Islands NVSS Custom Mortality Data 2023
Yemen	2022-2023	Yemen Multiple Indicator Cluster Survey 2022-2023
Zambia	2024-2024	Zambia Demographic and Health Survey 2024
Zambia	2016-2016	Zambia Population-Based HIV Impact Assessment 2016

Zambia	2013-2016	Zambia Vital Statistics Report 2016
Zambia	2013-2017	Zambia Vital Statistics Report 2017
Zambia	2016-2020	Zambia Vital Statistics Report 2020
Zimbabwe	2023-2024	Zimbabwe Demographic and Health Survey 2023-2024
Zimbabwe	2022-2022	Zimbabwe Population and Housing Census 2022
Zimbabwe	2015-2016	Zimbabwe Population-Based HIV Impact Assessment 2015-2016
Zimbabwe	2019-2020	Zimbabwe Population-Based HIV Impact Assessment 2019-2020
Zimbabwe	2022-2022	Zimbabwe Vital Statistics Report 2022

*United Nations convention recognises Taiwan as a province of China
