# Supplementary material for: Global age-sex-specific all-cause mortality and life expectancy estimates for 204 countries and territories and 660 subnational locations, 1950–2023: a demographic analysis for the Global Burden of Disease Study 2023
Source: Lancet. 2025 Oct 18;406(10513):1731–810. doi: 10.1016/S0140-6736(25)01330-3 (PMC12535839; doi:10.1016/S0140-6736(25)01330-3)
Supplement: Supplementary appendix 3 [file mmc3.pdf]

THE LANCET

Supplementary appendix 3

This appendix formed part of the original submission and has been peer reviewed. We post it as supplied by the authors.

Supplement to: GBD 2023 Demographics Collaborators. Global age-sex-specific all-cause mortality and life expectancy estimates for 204 countries and territories and 660 subnational locations, 1950–2023: a demographic analysis for the Global Burden of Disease Study 2023. *Lancet* 2025; published online Oct 12. https://doi.org/10.1016/S0140-6736(25)01330-3.

Appendix 3: Authorship appendix to "Global age-sex-specific all-cause mortality and life expectancy estimates for 204 countries and territories and 660 subnational locations, 1950– 2023: a demographic analysis for the Global Burden of Disease Study 2023"

This appendix provides further authorship detail for "Global age-sex-specific all-cause mortality and life expectancy estimates for 204 countries and territories and 660 subnational locations, 1950– 2023: a demographic analysis for the Global Burden of Disease Study 2023"

Table of Contents

GBD 2023 Demographics Collaborators 2

Affiliations 12

Authors' Contributions 66

- Managing the overall research enterprise 66
- Writing the first draft of the manuscript 66
- Primary responsibility for applying analytical methods to produce estimates 66
- Primary responsibility for seeking, cataloguing, extracting, or cleaning data; designing or coding figures and tables 66
- Providing data or critical feedback on data sources 66
- Developing methods or computational machinery 71
- Providing critical feedback on methods or results 72
- Drafting the work or revising it critically for important intellectual content 83
- Managing the estimation or publications process 90

GBD 2023 Demographics Collaborators

Austin E Schumacher*, Peng Zheng*, Ryan M Barber*, Bhoomadevi A, Mohammad Amin Aalipour, Hasan Aalruz, Hazim S Ababneh, Ukachukwu O Abaraogu, Cristiana Abbafati, Nasir Abbas, Mitra Abbasifard, Faezeh Abbaspour, Abdallah H A Abd Al Magied, Samar Abd ElHafeez, Mohammed Altigani Abdalla, Emad M Abdallah, Nadin M I Abdel Razeq, Reda Abdel-Hameed, Wael M Abdel-Rahman, Sherief Abd-Elsalam, Omar Ahmed Abdelwahab, Parsa Abdi, Arash Abdollahi, Meriem Abdoun, Arman Abdous, Deldar Morad Abdulah, Rizwan Suliankatchi Abdulkader, Auwal Abdullahi, Abdullahi Salahudeen Abdulraheem, Habtamu Abebe Abebe Getahun, Parisa Abedi, Armita Abedi, Asrat Agalu Abejew, Roberto Ariel Abeldaño Zuñiga, Syed Hani Abidi, Alemwork Abie, Olumide Abiodun, Olugbenga Olusola Abiodun, Richard Gyan Aboagye, Shady Abohashem, Ulric Sena Abonie, Nagah M Abourashed, Mohamed Abouzid, Dmitry Abramov, Lucas Guimarães Abreu, Dariush Abtahi, Rana Kamal Abu Farha, Fuad Hamdi A Abuadas, Aminu Kende Abubakar, Bilyaminu Abubakar, Eman Abu-Gharbieh, Sawsan Abuhammad, Ahmad Y Abuhelwa, Hana J Abukhadijah, Salahdein Aburuz, Dina Abushanab, Ahmed Abu-Zaid, Anirudh Balakrishna Acharya, Meshack Achore, Juan Manuel Acuna, Tim Adair, Lisa C Adams, Oladimeji Muritala Adebayo, Tajudeen Adesanmi Adebisi, David Adedia, Kamoru Ademola Adedokun, Oluwatobi E Adegbile, Nurudeen A Adegoke, Olumide Thomas Adeleke, Miracle Ayomikun Adesina, Isaac Ayodeji Adesina, Olatunji O Adetokunboh, Temitayo Esther Adeyeoluwa, Mache Tsadik Adhana, Kishor Adhikari, Ripon Kumar Adhikary, Usha Adiga, Tanin Adl Parvar, Mohd Adnan, Qorinah Estiningtyas Sakilah Adnani, Leticia Akua Adzigbli, David Adzrago, Giuseppina Affinito, Aanuoluwapo Adeyimika Afolabi, Rotimi Felix Afolabi, Saira Afzal, Gizachew Beykaso Agafari, Navidha Aggarwal, Mahdi Aghaalikhani, Sepehr Aghajanian, Seyed Mohammad Kazem Aghamir, Feleke Doyore Agide, Mary Dada Agoi, César Agostinis Sobrinho, Anurag Agrawal, Williams Agyemang-Duah, Bright Opoku Ahinkorah, Rabbiya Ahmad, Danish Ahmad, Faisal Ahmad, Aqeel Ahmad, Muayyad M Ahmad, Khurshid Ahmad, Tauseef Ahmad, Waqas Ahmad, Aram Mahmood Ahmed, Muktar Beshir Ahmed, Ayman Ahmed, Ali Ahmed, Anisuddin Ahmed, Mushood Ahmed, Naveed Ahmed, Oli Ahmed, Meqdad Saleh Ahmed, Akeem Olayiwola Ahmed, Mehrunnisha Sharif Ahmed, Syed Anees Ahmed, Gasha Salih Ahmed, Shabbir Ahmed, Haroon Ahmed, Luai A Ahmed, Gulzhanat Aimagambetova, Janardhana P Aithala, Marjan Ajami, Budi Aji, Hossein Akbarialiabad, Saeid Akbarifard, Oluwasefunmi Akeju, Roland Eghoghosoa Akhigbe, Muhammad Nadeem Akhtar, Karolina Akinosoglou, Yagiz Matthew Akiska, Mohammed Ahmed Akkaif, Wole Akosile, Hammad Akram, Ashley E Akrami, Hanadi Al Hamad, Syed Mahfuz Al Hasan, Mohammad Khaled Al Nawayseh, Omar Al Omari, Mohammad Al Qadire, Zain Al Ta'ani, Yazan Al Thaher, Omar Ali Mohammed Al Zaabi, Mohammad Ahmmad Mahmoud Al Zoubi, Mousa Ali Al-Abbadi, Tariq A Alalwan, Ziyad Al-Aly, Mohammad Khursheed Alam, Khurshid Alam, Mostafa Alam, Manjurul Alam, Rasmieh Mustafa Al-Amer, Abebaw Alamrew, Amani Alansari, Turki M Alanzi, Fahmi Y Al-Ashwal, Mohammed Albashtawy, Khalifah A Aldawsari, Mohammed S Aldossary, Robert W Aldridge, Shereen M Aleidi, Bezawit Abeje Alemayehu, Tekletsadik Tekleslassie Alemayehu, Fentahun Alemnew, Ayman Al-Eyadhy, Ali M Alfalki, Abdelazeem M Algammal, Fadwa Naji Alhalaiqa, Mohammed Khaled Al-Hanawi, Aminu Alhassan Alhassan Ibrahim, Ashraf Alhumaidi, Fahad A Alhumaydhi, Shahid Ali, Mohammed Usman Ali, Kamran Ali, Mohammad Daud Ali, Irfan Ali, Syed Shujait Ali, Waad Ali, Haroon Muhammad Ali, Amjad Ali, Rafat Ali, Maratab Ali, Syed Yusuf Ali, Sameer Afif Ali, Akram Al-Ibraheem, Gianfranco Alicandro, Montaha Al-Iede, Sheikh Mohammad Alif, Hamid Alinejad Rokny, Samah W Al-Jabi, Mohamad Aljofan, Moath Saleh Aljohani, Adel Al-Jumaily, Syed Mohamed Aljunid, Ahmad Alkhatib, Mustafa Alkhawam, Atefeh Allahbakhshian, Mohammed Z Allouh, Wesam Taher Almagharbeh, Wael Almahmeed, Md. Al-Mamun, Sabah Al-Marwani, Joseph Uy Almazan, Hesham M Al-Mekhlafi, Omar Almidani, Amr Almobayed, Khaldoon Aied

Alnawafleh, Hasan Yaser Alniss, Margret Beaula Alocious Sukumar, Mohammad R Alosta, Saleh A Alqahtani, Jaber S Alqahtani, Mohammad R Alqudimat, Ahmad Rajeh Al-Qudimat, Ahmad Alrawashdeh, Rami H Al-Rifai, Intima Alrimawi, Sahel Majed Alrousan, Salman Khalifah Al-Sabah, Mohammed A Alsabri, Najim Z Alshahrani, Zaid Altaany, Awais Altaf, Alaa B Al-Tammemi, Jaffar A Al-Tawfiq, Malik A Althobiani, Khalid A Altirkawi, Javier Alvarez-Galvez, Nelson Alvis-Guzman, Mohammad Al-Wardat, Yaser Mohammed Al-Worafi, Hany Aly, Mohammad Sharif Ibrahim Alyahya, Karem H Alzoubi, Md. Akib Al-Zubayer, Uchenna Anderson Amaechi, Joy Amafah, Ekiyor Joseph Amafah, Masoud Aman Mohammadi, Faten Amer, Bardia Amidi, Tarek Tawfik Amin, Amr Amin, Alireza Amindarolzarbi, Saeed Amini, Ehsan Amini-Salehi, Nafiu Aminu, Majid Aminzare, Sohrab Amiri, Mohammad Hosein Amirzade-Iranaq, Joanne O Amlag, Dickson A Amugsi, Ganiyu Adeniyi Amusa, Filippos Anagnostakis, Roshan A Ananda, Nazanin Anaraki, Robert Ancuceanu, Deanna Anderlini, David B Anderson, Tudorel Andrei, Song Peng Ang, Nguyen Hoang Anh, Samuel Egyakwa Ankomah, Kabilan Annadurai, Amir Anoushiravani, Sumbul Ansari, Umair Ansari, Alireza Ansari-Moghaddam, Catherine M Antony, Ernoiz Antriyandarti, Boluwatife Stephen Anuoluwa, Iyadunni Adesola Anuoluwa, Saleha Anwar, Sumadi Lukman Anwar, Razique Anwer, Shahnawaz Anwer, Anayochukwu Edward Anyasodor, Geminn Louis Carace Apostol, Juan Pablo Arab, Hossein Arabi, Jalal Arabloo, Mosab Arafat, Demelash Areda, Abdulfatai Aremu, Jorge Arias de la Torre, Ghazal Arjmand, Benedetta Armocida, Johan Ärnlöv, Jesu Arockiaraj, Mahwish Arooj, Anton A Artamonov, Deepavalli Arumuganainar, Nurila Aryntayeva, Mahsa Asadi Anar, Majid Asadi-Samani, Syed Mohammed Basheeruddin Asdaq, Saeed Asgary, Mohammad Asghari-Jafarabadi, Tahira Ashraf, Muhammad Abdul Basit Ashraf, Syed Amir Ashraf, Mitra Ashrafi, Milad Ashrafizadeh, Bernard Kwadwo Yeboah Asiamah-Asare, Muhammad Shahzad Aslam, Saeed Aslani, Yuni Asri, Anil Raj Assariparambil, Dereje Zewdu Assefa, Batyrbek Assembekov, Thomas Astell-Burt, Mirbahador Athari, Maha Moh'd Wahbi Atout, Alok Atreya, Julie Alaere Atta, Zeenah A Atwan, Marcel Ausloos, Abolfazl Avan, Núbia Carelli Pereira Avelar, Sana Javaid Awan, Amlaku Mulat Aweke, Babafela B Awosile, Adedapo Wasiu Awotidebe, Beatriz Paulina Ayala Quintanilla, Fekadu Belay Ayalew, Lemessa Assefa A Ayana, Haleh Ayatollahi, Olatunde O Ayinde, Yusuf Oloruntoyin Ayipo, Berrak Itır Itır Aylı, Seyed Mohammad Ayyoubzadeh, Sina Azadnajafabad, Arian Azadnia, James Mba Azam, Alireza Azarboo, Ali Azargoonjahromi, Gulrez Shah Azhar, Farya Azimi, Sadat Abdulla Aziz, Mohd Yusmaidie Aziz, Amin Azizan, Ahmed Y Azzam, Domenico Azzolino, Shahram Babadoust, Abraham Samuel Babu, Giridhara Rathnaiah Babu, Ashish D Badiye, Hunter Southwick Baggen, Elahe Baghizadeh, Sana Baghizadeh, Khlood K Baghlaf, Ahmed Salem BaHammam, Najmeh Bahmanziari, Razieh Bahreini, Yogesh Bahurupi, Ruhai Bai, Atif Amin Baig, Arun Balachandran, Wondu Feyisa Balcha, Maher Balkis, Jose Balmori-de-la-Miyar, Mohammadreza Balooch Hasankhani, Ovidiu Constantin Baltatu, Soham Bandyopadhyay, Palash Chandra Banik, Rajon Banik, Angelo Barbato, Suzanne Lyn Barker-Collo, Hiba Jawdat Barqawi, Amadou Barrow, Sandra Barteit, Zarrin Basharat, Shahid Bashir, Azadeh Bashiri, Guido Basile, Pritish Baskaran, Mohammad-Mahdi Bastan, Abdul-Monim Batiha, Kavita Batra, Bernhard T Baune, Mahdis Bayat, Mohammad Amin Bayat Tork, Mohsen Bayati, Mulat Tirfie Bayih, Thomas Beaney, Neeraj Bedi, Narasimha M Beeraka, Jina Behjati, Babak Behnam, Payam Behzadi, Diana Fernanda Bejarano Ramirez, Bezawit K Bekele, Almaz Nibret Belay, Melesse Belayneh, Asnake Gashaw Belayneh, Gokce Belge Bilgin, Bashir Bello, Muhammad Bashir Bello, Umar Muhammad Bello, Olorunjuwon Omolaja Bello, Luis Belo, Apostolos Beloukas, Riyad Bendardaf, Samiun Nazrin Bente Kamal Tune, Habib Benzian, Maria Bergami, Alemshet Yirga Berhie, Abiye Assefa Berihun, Amiel Nazer C Bermudez, Robert S Bernstein, Ajeet Singh Bhadoria, Akshaya Srikanth Bhagavathula, Jeetendra Bhandari, Charmi Bhanushali, Pankaj Bhardwaj, Nikha Bhardwaj, Ashish Bhargava, Sonu Bhaskar, Arushee Bhatnagar, Shuvarthi Bhattacharjee,

Priyadarshini Bhattacharjee, Manpreet S Singh Bhatti, Rajbir Bhatti, Gurjit Kaur Bhatti, Jasvinder Singh Bhatti, Soumitra S Bhuyan, Sibhatu Kassa Biadgilign, Raluca Bievel-Radulescu, Can Bilgin, Naif Kandash Binsaleh, Catherine Bisignano, Raaj Kishore Biswas, Mohammad Shahangir Biswas, Bijit Biswas, Ahmad Naoras Bitar, Molalegne Bitew, Bruno Bizzozero-Peroni, Virginia Bodolica, Mahmut Bodur, Lucimere Bohn, Obasanjo Afolabi Bolarinwa, Archith Boloor, Paria Bolourinejad, Sri Harsha Boppana, Berrak Bora Basara, Hamed Borhany, Arturo Borzutzky, Alejandro Botero Carvajal, Souad Bouaoud, Soufiane Boufous, Rupert R A Bourne, Christopher Boxe, Nicola Luigi Bragazzi, Dejana Braithwaite, Susanne Breitner, Hermann Brenner, Edmond D Brewer, Gabrielle Britton, Julie Brown, Annie J Browne, Raffaele Bugiardini, Linh Phuong Bui, Tsion Samuel Bunare, Richard A Burns, Felix Busch, Reinhard Busse, Yasser Bustanji, Zahid A Butt, Nadeem Shafique Butt, Lucero Cahuana-Hurtado, Tianji Cai, Rose Cairns, Daniela Calina, Luis Alberto Cámera, Luciana Aparecida Campos, Ismael Campos-Nonato, Si Cao, Yuchen Cao, Chao Cao, Angelo Capodici, Giulia Carreras, Austin Carter, Andrea Carugno, Márcia Carvalho, Andre F Carvalho, Ana Paula Carvalho-e-Silva, Joao Mauricio Castaldelli-Maia, Carlos A Castañeda-Orjuela, Giulio Castelpietra, Ferrán Catalá-López, Alberico L Catapano, Maria Sofia Cattaruzza, Luca Cegolon, Francieli Cembranel, Muthia Cenderadewi, Ester Cerin, Pamela Roxana Chacón-Uscamaita, Chiranjib Chakraborty, Sandip Chakraborty, Jeffrey Shi Kai Chan, Joht Singh Chandan, Rama Mohan Chandika, Miyuru Chandradasa, Jung-Chen Chang, Vijay Kumar Chattu, Victoria Chatzimavridou-Grigoriadou, Sirshendu Chaudhuri, Akhilanand Chaurasia, Galmesa Bekana Chemeda, An-Tian Chen, Hui Chen, Haowei Chen, Xiang Chen, Hana Chen, Meng Xuan Chen, Haojin Cheng, Ka Ching Cheung, Nicholas WS Chew, Fatemeh Chichagi, Ju-Huei Chien, Odgerel Chimed-Ochir, William C S Cho, Daniel Youngwhan Cho, Bryan Chong, Hitesh Chopra, Shivani Chopra, Sonali Gajanan Choudhari, Shanjida Chowdhury, Mohiuddin Ahsanul Kabir Chowdhury, Sreshtha Chowdhury, Dinh-Toi Chu, Hongyuan Chu, Isaac Sunday Chukwu, Stephen Chukwudeh, Eric Chung, Sheng-Chia Chung, Erin Chung, Sunghyun Chung, Cain C T Clark, Alyssa Columbus, Haley Comfort, Joao Conde, Nathalie Conrad, Samuele Cortese, Paolo Angelo Cortesi, Claudia Cosma, Michael H Criqui, Natalia Cruz-Martins, Garland T Culbreth, Nour Dababo, Ali Dabbagh, Omid Dadras, Zainab Umar Dahiru, Tukur Dahiru, Xiaochen Dai, Mayank Dalakoti, Koustuv Dalal, Gloria Dalla Costa, Emanuele D'Amico, Rakhi Dandona, Lalit Dandona, Lucio D'Anna, Pojsakorn Danpanichkul, Samuel E Danso, Samuel Demissie Darcho, Latefa Ali Dardas, Chengetai Dare, Jai K Das, Barbara A D'Avanzo, Claudio Alberto Dávila-Cervantes, Dimash Davletov, Kairat Davletov, Fernando Pio De la Hoz, Alejandro de la Torre-Luque, Edward Christopher Dee, Sindhura Deekonda, Louisa Degenhardt, Paria Dehesh, Lee Deitesfeld, Denise Myriam Dekker, Pouria Delbari, Mohammad Delsoz, Dessalegn Demeke, Andreas K Demetriades, Edgar Denova-Gutiérrez, Ismail Dergaa, Kebede Deribe, Hunegnaw Almaw Derseh, Emina Dervišević, Hardik Dineshbhai Desai, Abraham Aregay Desta, Vinoth Gnana Chellaiyan Devanbu, Pradeep Kumar Devarakonda, Devananda Devegowda, Arkadeep Dhali, Kuldeep Dhama, Rajinder K Dhamija, Samath Dhamminda Dharmaratne, Meghnath Dhimal, Bibha Dhungel, Marcello Di Pumpo, Diana Dias da Silva, Luis Antonio Diaz, Daniel Diaz, Diego Diaz-Milanes, Elangovan Dilipan, Lauren K Dillard, Zhendong Ding, Xueting Ding, M Ashworth Dirac, Huyen Do, Thao Huynh Phuong Do, Phidelia Theresa Doegah, Sushil Dohare, Klara Georgieva Dokova, Regina-Mae Villanueva Dominguez, Francesco Dondi, Mario D'Oria, Fariba Dorostkar, Ojas Prakashbhai Doshi, Robert Kokou Dowou, Menayit Tamrat Dresse, Tim Robert Driscoll, Jiang Du, Judy R Dubno, Emeka W Dumbili, Samuel C Dumith, Bruce B Duncan, Jennifer Dunne, Andre Rodrigues Duraes, Senbagam Duraisamy, Oyewole Christopher Durojaiye, Siddhartha Dutta, Angel Belle Cheng Dy, Abdel Rahman E'mar, Osamudiamen Ebohon, Ejemai Eboreime, Mohammad Hossein Ebrahimi, Abdelaziz Ed-Dra, David Edvardsson, Ferry Efendi, Behrad Eftekhari, Foolad Eghbali, Shayan Eghdami, Fatemeh Ehsani, Ashkan Eighaei Sedeh, Terje

Andreas Eikemo, Ebrahim Eini, Michael Ekholuenetale, Temitope Cyrus Ekundayo, Rabie Adel El Arab, Maysaa El Sayed Zaki, Mohamed Ahmed Eladl, Reza Elahi, Said El-Ashker, Rana Elbeshbeishy, Faris El-Dahiyat, Marwa Eldegwi, Marwan El-Deyarbi, Noha Mousaad Elemam, Ghada Metwally Tawfik ElGohary, Muhammed Elhadi, Mohamed Elhoumed, Waseem El-Huneidi, Omar Abdelsadek Abdou Elmeligy, Mohamed A Elmonem, Adel B Elmoselhi, Mohamed Hassan Elnaem, Mohammed Elshaer, Ibrahim Elsohaby, Chadi Eltaha, Abdelgawad Salah Abdelgawad Eltahawy, Tadele Emagneneh, Syed Emdadul Haque, Theophilus I Emeto, Victor Oghenekparobo Emojevwe, Stanley Chinedu Eneh, Christopher Imokhuede Esezobor, Babak Eshrati, Sharareh Eskandarieh, Majid Eslami, Rafaela Cavalheiro do Espírito Santo, Kara Estep, Elochukwu Ezenwankwo, Natalia Fabin, Heidar Fadavian, Adeniyi Francis Fagbamigbe, Ayesha Fahim, Razana Faiz, Ildar Ravisovich Fakhradiyev, Aliasghar Fakhri-Demeshghieh, Luca Falzone, Qiping Fan, Mohammad Farahmand, Seyed Nooreddin Faraji, Ali Faramarzi, Mohammad Fareed, Andre Faro, Syed Muhammad Yousaf Farooq, Fatemeh Farshad, Farima Farsi, Md. Omar Faruk, Abidemi Omolara Fasanmi, Folorunso Oludayo Fasina, Modupe Margaret Fasina, Ali Fatehizadeh, Davood Fathi, Zareen Fatima, Timur Fazylov, Valery L Feigin, maryam feili, Alireza Feizkhah, Ginenus Fekadu, Xiaoqi Feng, Talukdar Raian Ferdous, Seyed-Mohammad Fereshtehnejad, Nuno Ferreira, Bikila Regassa Feyisa, Alexander Finnemore, Claudio Fiorilla, Ida Fitriana, Luisa S Flor, Artem Alekseevich Fomenkov, Marco Fonzo, Arianna Fornari, Behzad Foroutan, Daniela Fortuna, Matteo Foschi, Maryam Fotouhi, Kayode Raphael Fowobaje, Richard Charles Franklin, Alberto Freitas, Takeshi Fukumoto, Ami Fukunaga, John E Fuller, Nancy Fullman, Blima Fux, Sridevi G, Peter Andras Gaal, Muktar A Gadanya, Dominic Dormenyo Gadeka, Márió Gajdács, Emmanuela Gakidou, Yaseen Galali, Silvano Gallus, Dhanraj Ganapathy, Balasankar Ganesan, Shivaprakash Gangachannaiah, Xiang Gao, Yijie Gao, Bashiru Garba, Miguel Garcia-Argibay, David Garcia-Azorin, Jacopo Garlasco, Zisis Gatzioufas, Rupesh K Gautam, Prem Gautam, Bamba Gaye, Federica Gazzelloni, Hong-Han Ge, Feven Sahle Gebre, Miglas Welay Gebregergis, Haftay Gebremedhin Gebreslassie, Miesa Gelchu, Stefano Gelibter, Nsikakabasi Samuel George, Ali Gerami Matin, Lemma Getacher, Genanew K Getahun, Kalab Yigermal Gete, Peter W Gething, Delaram J Ghadimi, Keyghobad Ghadiri, Arin Ghamkhar, Ali Ghandili, Mohammad-Reza Ghasemi, Moein Ghasemi, Shakiba Ghasemi Assl, Fariba Ghassemi, Ramy Mohamed Ghazy, Nermin Ghith, Zainab Gholami, Nasim Gholizadeh, Elena Ghotbi, Arun Ghuge, Alessandro Gialluisi, Konstantinos Giannakis, Ruth Margaret Gibson, Syed Abdullah Gilani, Tiffany K Gill, Alem Abera Girmay, Alessandro Girombelli, Laszlo Göbölös, Rajesh Kumar Goel, Anil Kumar Goel, Archit Goel, Kimiya Gohari, Mahaveer Golechha, Ali Golestani, Mohsen Golkar, Nelson G M Gomes, Philimon N Gona, Wenping Gong, Sameer Vali Gopalani, Giuseppe Gorini, Yitayal Ayalew Goshu, Alessandra C Goulart, Ayman Grada, Simon Matthew Matthew Graham, Michal Grivna, Ashna Grover, Habtamu Alganeh Guadie, Bin Guan, Shi-Yang Guan, Giovanni Guarducci, Mohammed Ibrahim Mohialdeen Gubari, Avirup Guha, Stefano Guicciardi, Zhifeng Guo, Xingzhi Guo, Zhaoyu Guo, Cui Guo, Zheng Guo, Rajat Das Gupta, Rajeev Gupta, Sapna Gupta, Lalit Gupta, Himanshu Gupta, Roberth Steven Gutiérrez-Murillo, Jose Guzman-Esquivel, Abrham Tesfaye Tesfaye Habteyes, Awoke Derbie Derbie Habteyohannes, Tesfahun Simon Hadaro, Zahra Hadian, Sarah Hafsia, Faraidoon Haghdoost, Arian Haghtalab, Nguyen Hai Nam, Arvin Haj-Mirzaian, Pritam Halder, Sebastian Haller, Rabih Halwani, Islam M Hamad, Randah R Hamadeh, Nadia M Hamdy, Samer Hamidi, Erin B Hamilton, Ahmad Hammoud, Mohammad Hamza, Didem Han Yekdeş, Asif Hanif, Nasrin Hanifi, Graeme J Hankey, Fahad Hanna, Md Nuruzzaman Haque, Ashanul Haque, Obaid I Haque, Harapan Harapan, Hilda L Harb, Arief Hargono, Andy Martahan Andreas Hariandja, Josep Maria Haro, Eka Mishbahatul Marah Has, Ahmed I Hasaballah, Md Kamrul Hasan, Faizul Hasan, Towhid Hasan, Hamidreza Hasani, Ali Hasanpour-Dehkordi, Arezou Hashem Zadeh, Mohammad Hashem Hashempur, Nada Tawfig Hashim, Ammarah

Hasnain, Ikrama Hassan, Ibrahim Nagmeldin Hassan, Nageeb Hassan, Yusuf Wada Hassan Wada, Mahgol Sadat Hassan Zadeh Tabatabaei, Rasmus J Havmoeller, Simon I Hay, Khezar Hayat, Jiawei He, Jeffrey J Hebert, Mohammad Heidari, Golnaz Heidari, Mehdi Hemmati, Claire A Henson, Claudiu Herteliu, Hamed Hesami, Sumudu Avanthi Hewage, Majid Heydari, Zahra Heydarifard, Yuta Hiraike, Ramesh Holla, Nobuyuki Horita, Md Belal Hossain, Md Mahbub Hossain, Md Sabbir Hossain, Alamgir Hossain, Mohammad Bellal Hossain, Lubna Hossain, Sorin Hostiuc, Mihaela Hostiuc, Peter J Hotez, Jada Averianna Houser, Amir Human Hoveidaei, Hanno Hoven, Alexander Win Hsu, Chengxi Hu, Guoqing Hu, Yefei Huang, Zhenyao Huang, Junjie Huang, Weijun Huang, Mega Hasanul Huda, Ayesha Humayun, Waqar Husain, Kiavash Hushmandi, Javid Hussain, Nawfal R Hussein, Mohamed Ibrahim Husseiny, Hong-Han Huynh, Bing-Fang Hwang, Luigi Francesco Iannone, Segun Emmanuel Ibitoye, Umar Idris Ibrahim, Ismail A. Atef Ismail Ahmed Ibrahim, Ramzi Ibrahim, Anel Ibrayeva, Fidelia Ida, Pulwasha Maria Iftikhar, Adalia Ikiroma, Kevin S Ikuta, Olayinka Stephen Ilesanmi, Irena M Ilic, Milena D Ilic, Masoud Imani, Mustapha Immurana, Lucius Chidiebere Imoh, Leeberk Raja Inbaraj, Arit Inok, Mujahid Iqbal, Muhammad Iqhrammullah, Mustafa Alhaji Isa, Benni Iskandar, Teresa R Iskander, Dr. Md. Shahinul Islam, Md Rabiul Islam, Md Shariful Islam, Farhad Islami, Nahlah Elkudssiah Ismail, Faisal Ismail, Yerlan Ismoldayev, Gaetano Isola, Masao Iwagami, Ihoghosa Osamuyi Iyamu, Mahalaxmi Iyer, Vinothini J, Jalil Jaafari, Udeme Samuel Jacob, Kathryn H Jacobsen, Ali Jadidi, Mohammadsadegh Jafari, Ali Jafari-Khounigh, Morteza Jafarinia, Vennila Jaganathan, Haitham Jahrami, Ayushi Jain, Ammar Abdulrahman Jairoun, Mihajlo Jakovljevic, Ali Jaliliyan, Mohamed Jalloh, Qazi Mohammad Sajid Jamal, Armaan Jamal, Jazlan Jamaluddin, Melika Jameie, Jerin James, Safayet Jamil, Masoud Jamshidi, Shaghayegh JamshidiRastabi, Esmaeil Jarrahi, Tahereh Javaheri, Syed Sarmad Javaid, Anita Javanmardi, Javad Javidnia, Shubha Jayaram, Ruwan Duminda Jayasinghe, Yovanthi Anurangi Jayasinghe, Achala Upendra Jayatilleke, Felix K Jebasingh, Jayakumar Jeganathan, Seogsong Jeong, Bijay Mukesh Jeswani, Zixiang Ji, Min Jiang, Wenyi Jin, Shuai Jin, Mohammad Jokar, Jost B Jonas, Darwin Phan Jones, Tamas Joo, Abu Jor, Nitin Joseph, Abel Joseph, Charity Ehimwenma Joshua, Katie Joskowitz, Kripa Josten, George Joy, Jacek Jerzy Jozwiak, Malik E Juweid, Vaishali K, Zubair Kabir, Dler H. Hussein Kadir, Ashish Kumar Kakkar, Pradnya Vishal Kakodkar, Khalil Kalavani, Sanjay Kalra, Md Moustafa Kamal, Mehnaz Kamal, Sivesh Kathir Kamarajah, Rajesh Kamath, Saltanat Kamenova, Arun Kamireddy, Ramat T Kamorudeen, Devanish Narasimhasanth Kamtam, Naser Kamyari, Oleksandr Kamyshnyi, Mona Kanaan, Saddam Fuad Kanaan, Jiseung Kang, Samuel Berchi Kankam, Kehinde Kazeem Kanmodi, Rami S Kantar, Neeti Kapoor, Sujita Kumar Kar, Paschalis Karakasis, Reema A Karasneh, Mohammad Amin Karimi, Salah Eddin Karimi, Mohmed Isaqali Karobari, Tomasz M Karpiński, Sadanand Karun, Manoj Kumar Kashyap, Eden Asmare Kassahun, Nigussie Assefa Kassaw, Nicholas J Kassebaum, Molly B Kassel, Adarsh Katamreddy, Kanica Kaushal, Foad Kazemi, Nastaran Kazemi rad, Sina Kazemian, Hafte Kahsay Kebede, Chukwudi Keke, John H Kempen, Jessica A Kerr, Emmanuelle Kesse-Guyot, Reza Khademi, Inn Kynn Khaing, Himanshu Khajuria, Nauman Khalid, Sidra Khalid, Hazim O Khalifa, Anas Husam Khalifeh, Anees Ahmed Khalil, Pantea Khalili, Anita Khalili, Alireza Khalilian, Ghazaleh Khalili-Tanha, Mohamed khalis, Faham Khamesipour, Muhammad Mueed Khan, Zahid Khan, Zahid Khan, Abdul A Khan, Md Abdullah Saeed Khan, Serab Khan, Yusuf Saleem Khan, Ramsha Mushtaq Khan, Ajmal Khan, Mohammad Jobair Khan, Muhammad Hamza Khan, Iman Waheed Khan, Muhammad Umer Khan, Sumaiya Khan, Maseer Khan, Fayaz Khan, Srijana Khanal, Shaghayegh Khanmohammadi, Zenith Khashim, Khaled Khatab, Haitham Khatatbeh, Moawiah Mohammad Khatatbeh, Kavin Khatri, Hamid Reza Khayat Kashani, Khalid A Kheirallah, Sunil Kumar Khokhar, Mohammad Saeid Khonji, Zahra Khorrami, Najmaddin Salih Husen S.H. Khoshnaw, Atulya Aman Khosla, Sepehr Khosravi, Majid Khosravi, Mahmood Khosrowjerdi, Jagdish Khubchandani, Zemene Demelash

Kifle, Min Seo Kim, Hye Jun Kim, Jinho Kim, Yun Jin Kim, Ruth W Kimokoti, Yohannes Kinfu, Sanjay Kini B, Mary Kirk, Adnan Kisa, Sezer Kisa, Ladli Kishore, Juniper Boroka Kiss, Mika Kivimäki, Shivakumar KM, Ann Kristin Skrindo Knudsen, Nazarii Kobyliak, Sonali Kochhar, Michail Kokkorakis, Ali-Asghar Kolahi, Diana Gladys Kolieghu Tcheumeni, Farzad Kompani, Aida Kondybayeva, Anastasios Georgios Panagiotis Konstas, Isaac Koomson, Gerbrand Koren, Tapos Kormoker, Oleksii Korzh, Karel Kostev, Archana Koul, Sindhura Lakshmi Koulmane Laxminarayana, James-Paul Kretchy, Irene Akwo Kretchy, Kewal Krishan, Chong-Han Kua, Ananya Kuanar, Barthelemy Kuate Defo, Mohammed Kuddus, Ilari Kuitunen, Shikha Kukreti, Mukhtar Kulimbet, Vishnutheertha Kulkarni, Shweta Kulshreshtha, Sanjay Kirshan Kumar, Manasi Kumar, Dewesh Kumar, Tushar Kumar, Vijay Kumar, Jogender Kumar, Nithin Kumar, G Anil Kumar, Avinash Kumar, Jibin Kunjavara, Setor K Kunutsor, Almagul Kurmanova, Maria Dyah Kurniasari, Pramod Kumar Kushawaha, Asep Kusnali, Christina Yeni Yeni Kustanti, Dian Kusuma, Tezer Kutluk, Assylkhan Kuttybayev, Wai Hang Patrick Kwong, Grace Kwakyewaa Kyei, Evans F Kyei, Frank Kyei-Arthur, Ville Kytö, Hmwe Hmwe Kyu, Pallavi L C, Adriano La Vecchia, Carlo La Vecchia, Alessio Lachi, Muhammad Awwal Ladan, Lucie Laflamme, Chandrakant Lahariya, Daphne Teck Ching Lai, Balzhan Lakanova, Anita Lakhani, Dharmesh Kumar Lal, Ratilal Lalloo, Tea Lallukka, Iván Landires, Berthold Langguth, Ariane Laplante-Lévesque, Dylan Lasher, Kamaluddin Latief, Mahrukh Latif, Colleen L L Lau, Saheed Akinmayowa Lawal, Aliyu Lawan, Trang Diep Thanh Le, Huu-Hoai Le, Nhi Huu Hanh Le, Minh Huu Nhat Le, Thao Thi Thu Le, Caterina Ledda, Wei-Chen Lee, Ivan Lee, Seung Won Lee, Yo Han Lee, Vasileios Leivaditis, Matthew J Lennon, Matilde Leonardi, Elvynna Leong, An Li, Jinbo Li, Hui Li, Yongze Li, Jianan Li, Ming-Chieh Li, Wei Li, Wei Li, Chengfeng Li, Wang-Zhong Li, Zhengrui Li, Zhaolong Adrian Li, Weilong Li, Jiaying Li, Yanxue Lian, Xue-Zhen Liang, Virendra S Ligade, Stephen S Lim, Ro-Ting Lin, Queran Lin, Shuzhi Lin, Jialing Lin, Daniel Lindholm, Yuewei Ling, Xuefeng Liu, Xiaofeng Liu, Haipeng Liu, Xianliang Liu, Zhe Liu, Jue Liu, Gang Liu, Yubo Liu, Erand Llanaj, Michael J Loftus, Valerie Lohner, José Francisco López-Gil, Masoud Lotfizadeh, Surbala Devi Lourembam, Rafael Lozano, Shanjie Luan, Jailos Lubinda, Giancarlo Lucchetti, Susu Luo, Jay B Lusk, Angelina M Lutambi, Miltiadis D Lytras, Ellina Lytvyak, Hawraz Ibrahim M. Amin, Zheng Feei Ma, Kevin Sheng-Kai Ma, Kelsey Lynn Maass, Mahmoud Mabrok, Nikolaos Machairas, Monika Machoy, Firoozeh Madadi, Seyed Ataollah Madinezad, Christian Madsen, Aurea Marilia Madureira-Carvalho, Pasquale Maffia, Azzam A Maghazachi, D. R. Mahadeshwara Prasad, Sasikumar Mahalingam, Preeti Maharjan, Mina Maheri, Nozad Hussein Mahmood, Alireza Mahmoudi, Farhad Mahmoudi, Panagiota Maikanti-Charalampous, Rituparna Maiti, Marek Majdan, Abdelrahman M Makram, Reza Malekzadeh, Hardeep Singh Malhotra, Farihah Malik, Ahmad Azam Malik, Deborah Carvalho Malta, Mustapha Mangdow, Emery Manirambona, Lokesh Manjani, Yosef Manla, Fahmida Mannan, Kamaruddeen Mannethodi, Farheen Mansoor, Marjan Mansourian, Mohammad Ali Mansournia, Lorenzo Giovanni Mantovani, Changkun Mao, Tahir Maqbool, Hamid Reza Marateb, Joemer C Maravilla, Konstantinos Margetis, Mirko Marino, Adilson Marques, Gabriel Martinez, Bernardo Alfonso Martinez-Guerra, Ramon Martinez-Piedra, Daniela Martini, Francisco Rogerlândio Martins-Melo, Miquel Martorell, Roy Rillera Marzo, Sammer Marzouk, Sugeng Mashudi, Soroush Masrouri, Clara N Matei, Yasith Mathangasinghe, Stephanie Mathieson, Alexander G Mathioudakis, Medha Mathur, Neeta Mathur, Fernanda Penido Matozinhos, Rita Mattiello, Khurshid A Mattoo, Richard James Maude, Pallab K Maulik, Erin A May, Mahsa Mayeli, Mohsen Mazidi, John J McGrath, Martin McKee, Steven M McPhail, Michael A McPhail, Enkeleint A Mechili, Rishi P Mediratta, Riffat Mehboob, Ravi Mehrotra, Vini Mehta, Tesfahun Mekene Meto, Berhanu Abebaw Mekonnen, Hadush Negash Meles, Addisu Melese, Satish Melwani, Walter Mendoza, Godfred Antony Menezes, Emiru Ayalew Mengistie, George A Mensah, Sultan Ayoub Ayoub Meo, Michelangelo Mercogliano, Tuomo J Meretoja, Atte Meretoja, Tomislav

Mestrovic, Chamila Dinushi Kukulege Mettananda, Sachith Mettananda, Mohamed M M Metwally, Tomasz Miazgowski, Irmina Maria Michalek, Andrea Michelerio, Hiwot Soboksa Mideksa, Ted R Miller, Giuseppe Minervini, GK Mini, Mojgan Mirghafourvand, Seyed Ali Mirshahvalad, Mizan Kiros Mirutse, Maryam Mirzaei, Awoke Misganaw, Archana Mishra, Philip B Mitchell, Sayan Mitra, Chaitanya Mittal, Malihe Moazeni, Shivani Modi, Nouh Saad Mohamed, Mona Gamal Mohamed, Jama Mohamed, Khabab Abbasher Hussien Mohamed Ahmed, Taj Mohammad, Sakineh Mohammad-Alizadeh-Charandabi, Abdollah Mohammadian-Hafshejani, Saeed Mohammadpour, Ibrahim Mohammadzadeh, Shafiu Mohammed, Yahaya Mohammed, Hussen Mohammed, Abdulwase Mohammed, Omer Mohammed, Mustapha Mohammed, Suleiman Mohammed, Ammas Siraj Mohammed, Mohammad Mohseni, Ali H Mokdad, Sabrina Molinaro, Amirabbas Mollaei, Shaher Momani, Lorenzo Monasta, Himel Mondal, Stefania Mondello, Mohammad Ali Moni, Marco Montalti, Yousef Moradi, Maziar Moradi-Lakeh, Paula Moraga, Rafael Silveira Moreira, Shane Douglas Morrison, Mahmoud M Morsy, Reza Mosaddeghi Heris, Jonathan F Mosser, Elias Mossialos, Simin Mouodi, Mariana Mourgova, Asma Mousavi, Seyede Zohre Mousavi, Amin Mousavi Khaneghah, Seyed Mohamad Sadegh Mousavi Kiasary, Amanda Movo, Hagar Lotfy Mowafy, Kimia Mozahheb Yousefi, Matías Mrejen, Rabia Mubarak, Faraz Mughal, Syed Aun Muhammad, Oscar J Mujica, Sumoni Mukherjee, Sukhes Mukherjee, Amartya Mukhopadhyay, George Duke Mukoro, M A Muktadir, Francesk Mulita, Chalie Mulugeta, Mulyadi Mulyadi, Malaisamy Muniyandi, Kavita Munjal, Yanjinlkham Munkhsaikhan, Javier Muñoz Laguna, Michio Murakami, Efren Murillo-Zamora, B.V. Murlimanju, Sani Musa, Ali Mushtaq, Sherzad Ibrahim Mustafa, Mubarak Taiwo Mustapha, Sathish Muthu, Saravanan Muthupandian, Claude Mambo Muvunyi, Muhammad Muzaffar, Woojae Myung, Amin Nabavi, Ahamarshan Jayaraman Nagarajan, Shankar Prasad Nagaraju, Mohsen Naghavi, Ganesh R Naik, Firzan Nainu, Hastyar Hama Rashid Najmuldeen, Noureddin Nakhostin Ansari, Gopal Nambi, Vinay Nangia, Jobert Richie Nansseu, Yvonne Nartey, Bruno Ramos Nascimento, Gustavo G Nascimento, Abdallah Y Naser, Abdulqadir J Nashwan, Hamide Nasiri, Mahmoud Nassar, Zuhair S Natto, Zakira Naureen, Samidi Nirasha Kumari Navaratna, Nawsherwan , Biswa Prakash Nayak, Shalini Ganesh Nayak, Smitha Nayak, Shumaila Naz, G. Takop Nchanji, Amanuel Tebabal Nega, Masoud Negahdary, Wubshet D Negash, Ruxandra Irina Negoi, Ionut Negoi, Jalil Nejati, Nikita A Nekliudov, Samata Nepal, Henok Biresaw Netsere, Charles Richard James Newton, Marie Ng, Georges Nguefack-Tsague, Josephine W Ngunjiri, The Phuong Nguyen, Van Thanh Nguyen, Dang Nguyen, Long Nguyen, Nghia Phu Nguyen, Tu Anh Nguyen, Cuong Tat Nguyen, Ambe Marius Ngwa, Robina Khan Niazi, Luciano Nieddu, Ali Nikoobar, Vikram Niranjan, Abebe Melis Nisro, Jan Rene Nkeck, Chukwudi A Nnaji, Shuhei Nomura, Syed Toukir Ahmed Noor, Sana Noreen, Masoud Noroozi, Jean Jacques Noubiap, Valentine C Nriagu, Chisom Adaobi Nri-Ezedi, Jean Claude Nshimiyimana, Mpiko Ntsekhe, Fred Nugen, Atoma Negera Nugusa, Mengistu H Nunemo, Nurfatimah Nurfatimah, Dieta Nurrika, Sylvester Dodzi Dodzi Nyadanu, Felix Kwasi Nyande, Ogochukwu Janet Nzoputam, Bogdan Oancea, Fabio Massimo Oddi, Ismail A Odetokun, Oluwakemi Ololade Odukoya, Joseph Kojo Oduro, Michael Safo Oduro, Akinyemi O D Ofakunrin, Onome Bright Oghenetega, Oluwafunmilayo Tosin Ogundeko-Olugbami, In-Hwan Oh, Sarah Oh, Edel T O'Hagan, Hassan Okati-Aliabad, Sylvester Reuben Okeke, Deborah Oluwatosin Okeke-Obayemi, Olalekan John Okesanya, Osaretin Christabel Okonji, Oluwaseyi Isaiah Olabisi, Andrew T Olagunju, Oladotun Victor Olalusi, Matthew Idowu Olatubi, Arão Belitardo Oliveira, Gláucia Maria Moraes Oliveira, Abdulhakeem Abayomi Olorukooba, Oluseye Olalekan Oludoye, Jacob Olusegun Olusanya, Bolajoko Olubukunola Olusanya, Goran Latif Omer, Sandersan Onie, Obinna E Onwujekwe, Marcel Opitz, Aksoltan Shyhdurdyevna Oradova, Michal Ordak, Verner N Orish, Raffaele Ornello, Atakan Orscelik, Alberto Ortiz, Esteban Ortiz-Prado, Augustus Osborne, John W Ostrominski, Uchechukwu Levi

Osuagwu, Olayinka Osuolale, Godfred Otchere, Elham H Othman, Adrian Otoiu, Oche Joseph Otorkpa, Abdu Oumer, Jerry John Ouner, Amel Ouyahia, Mayowa O Owolabi, Irene Amoakoh Owusu, Kolapo Oyebola, Tope Oyelade, Oyetunde T Oyeyemi, Ilker Ozsahin, Mahesh P A, Alicia Padron-Monedero, Jagadish Rao Padubidri, Dimpal Manilal Paija, Keyvan Pakshir, Tamás Palicz, Raffaele Palladino, Raul Felipe Palma-Alvarez, Tejasri Paluvai, Feng Pan, Sujogya Kumar Panda, Songhomitra Panda-Jonas, Seithikurippu R Pandi-Perumal, Carlo Irwin Able Panelo, Helena Ullyartha Pangaribuan, Georgios D Panos, Leonidas D Panos, Ioannis Pantazopoulos, Anca Pantea Stoian, Giovanni Paolino, Ilias Papadimopoulos, Paraskevi Papadopoulou, Parinaz Paranjkhoo, Shahina Pardhan, Peyvand Parhizkar Roudsari, Romil R Parikh, Chulwoo Park, Eun-Kee Park, Seoyeon Park, Arpit Parmar, Swapnil Parve, Maja Pasovic, Roberto Passera, Jay Patel, Satyananda Patel, Mitesh Patel, Sangram Kishor Patel, Hemal M Patel, Bhumi Hemal Patel, Heta Pavan Patel, Riya Jayesh Patel, Neel Navinkumar Patel, Angel J Paternina-Caicedo, Bharat Smita Umakant Patil, Shankargouda Patil, Ashlesh Patil, Apurba Patra, Venkata Suresh Patthipati, Shubhadarshini Pawar, Shrikant Pawar, Hamidreza Pazoki Toroudi, Spencer A Pease, Amy E Peden, Paolo Pedersini, Jarmila Pekarcikova, Veincent Christian Filipino Pepito, Prince Peprah, Emmanuel K Peprah, João Perdigão, Gavin Pereira, Maria Odete Pereira, Pablo Perez-Lopez, Arokiasamy Perianayagam, Norberto Perico, Simone Perna, Pavlo Petakh, Olumuyiwa James Peter, Fanny Emily Petermann-Rocha, Hoang Nhat Pham, Tung Thanh Pham, Hoang Tran Pham, Nhat Truong Pham, Anil K Philip, Michael R Phillips, Zayar Phyo, David M Pigott, Zahra Zahid Piracha, Edoardo Pirera, Moein Piroozkhkah, Enrico Pisoni, Florian Ploeckl, Evgenii Plotnikov, Dimitri Poddighe, Roman V Polibin, Ramesh Poluru, Ville T Ponkilainen, Ion Popa, Djordje S Popovic, Sajjad Pourasghary, Reza Pourbabaki, Farzad Pourghazi, Naeimeh Pourtaheri, Sergio I Prada, Pranil Man Singh Pradhan, Jalandhar Pradhan, Akila Prashant, Elton Junio Sady Prates, Harsh Priya, Nicola Riccardo Pugliese, Hery Purnobasuki, Shuby Puthussery, Jagadeesh Puvvula, Nameer Hashim Qasim, Zhipeng Qi, Xiang Qi, Jia-Yong Qiu, Zahiruddin Syed Quazi, Basuki Rachmat, Raghu Anekal Radhakrishnan, Hadi Raeisi Shahraki, Alberto Raggi, Pracheth Raghuveer, Hawbash Mohammed-Amin Rahim, Sajjad Rahimi, Vafa Rahimi-Movaghar, Muhammad Aziz Rahman, Mahbubur Rahman, Fryad Majeed Rahman, Md Mijanur Rahman, Mohammad Hifz Ur Rahman, Md. Mosfequr Rahman, Mosiur Rahman, Amir Masoud Rahmani, Saeed Rahmani, Masoud Rahmati, Ghasem Rahmatpour Rokni, Hakim Rahmoune, Ivano Raimondo, Diego Raimondo, Sunil Kumar Raina, Jeffrey Pradeep Raj, Adarsh Raja, Sandesh Raja, Erta Rajabi, Gunaseelan Rajendran, Judah Rajendran, Mohammad Amin Rajizadeh, Mahmoud Mohammed Ramadan, Majed Ramadan, Kadar Ramadhan, Chitra Ramasamy, Shakthi Kumaran Ramasamy, Sheena Ramazanu, Zahra Ramezani, Marzieh Ramezani Farani, Juwel Rana, Chhabi Lal Ranabhat, Nemanja Rancic, Smitha Rani, Kumuda Rao, Mithun Rao, Chythra R Rao, Davide Rasella, Vahid Rashedi, Mohammad-Mahdi Rashidi, Ashkan Rasouli-Saravani, Prateek Rastogi, Azad Rasul, Devarajan Rathish, Abdur Rauf, Santosh Kumar Rauniyar, Ilari Rautalin, Ramin Ravangard, Dhwani Ravi, David Laith Rawaf, Reza Rawassizadeh, Ramu Rawat, Bahman Razi, Christian Razo, Murali Mohan Rama Krishna Reddy, Elrashdy Redwan, Sanika Rege, Wajiha Rehman, Rainer Reile, Giuseppe Remuzzi, Bhageerathy Reshmi, Stefano Restaino, Mina Rezaei, Marzieh Rezaei, Nazila Rezaei, Mohsen Rezaeian, Taeho Gregory Rhee, Antonio Luiz P Ribeiro, Tércia Moreira Ribeiro da Silva, Jennifer Rickard, Hannah Elizabeth Robinson-Oden, Hermano Alexandre Lima Rocha, João Rocha Rocha-Gomes, Alfonso J. Rodriguez-Morales, Leonardo Roever, Ravi Rohilla, Iftitakhur Rohmah, Susanne Röhr, David Rojas-Rueda, Megan L Rolfzen, Debby Syahru Romadlon, Michele Romoli, Luca Ronfani, Moustaq Karim Khan Rony, Jennifer Jacqueline Rosauer, Emily Rosenblad, Amirhossein Roshanshad, Morteza Rostamian, Kunle Rotimi, Himanshu Sekhar Rout, Shiva Rouzbahani, Reza Rouzbahani, Hanieh Rouzbahani, Adrija Roy, Nitai Roy, Priyanka Roy, Parimal Roy, Bedanta Roy, Sharmistha Roy, Simanta

Roy, Simanta Roy, Shubhanjali Roy, Parameswari Royapuram Parthasarathy, Enrico Rubagotti, Susan Fred Rumisha, Michele Russo, Godfrey M Rwegerera, Poorvikha S, Chandan S N, Aly M A Saad, Zahra Saadatian, Michela Sabbatucci, Korosh Saber, Maha Mohamed Saber-Ayad, Cameron John Sabet, Siamak Sabour, Perminder S Sachdev, Kabir P Sadarangani, Seyed Kiarash Sadat Rafiei, Basema Ahmad Saddik, Adam Saddler, Bashdar Abuzed Sadee, Tarannom Sadegh, Erfan Sadeghi, Ehsan Sadeghi, Fatemeh Sadeghi-Ghyassi, Umar Saeed, Mohd Saeed, Maryam Saeedi, Mehdi Safari, Mahdi Safdarian, Sher Zaman Safi, Rajesh Sagar, Mastooreh Sagharichi, Amene Saghazadeh, Dominic Sagoe, Indranil Saha, Nondo Saha, Narjes Saheb Sharif-Askari, Fatemeh Saheb Sharif-Askari, Amirhossein Sahebkar, Biniyam Sahiledengle, Gülsüm Şahin Bodur, Pragyan Monalisa Sahoo, Zahra Saif, S Mohammad Sajadi, Md Refat Uz Zaman Sajib, Mirza Rizwan Sajid, Payman Salamati, Luciane B Salaroli, Mohamed A Saleh, Mahdi Salehi, Mohammed Z Y Salem, Marwa Rashad Salem, Dauda Salihu, Sohrab Salimi, Pegah Salimi Pormehr, Malik Sallam, Hossein Samadi Kafil, Saad Samargandy, Yoseph Leonardo Samodra, Abdallah M Samy, Sandeep G Sangle, Elaheh Sanjari, Sathish Sankar, Francesca Sanna, Lucas H C C Santos, Milena M Santric-Milicevic, Haaris Saqib, Sivan Yegnanarayana Iyer Saraswathy, Jacob Owusu Owusu Sarfo, Yaser Sarikhani, Tanmay Sarkar, Hemen Sarma, Mohammad Sarmadi, Sachin C Sarode, Gargi Sachin Sarode, Benn Sartorius, Arash Sarveazad, Michele Sassano, Brijesh Sathian, Mukesh Kumar Sathya Narayanan, Maheswar Satpathy, Jennifer Saulam, Mehrdad Savabi Far, Kimia Savoji, Monika Sawhney, Ganesh Kumar Saya, Abu Sayeed, Christophe Schinckus, Jurgen Carlo Schmidt, Maria Inês Schmidt, Aletta Elisabeth Schutte, Ghil Schwarz, David C Schwebel, Falk Schwendicke, Catherine Schwinger, Mario Šekerija, Siddharthan Selvaraj, Yuliya Semenova, Mohammad H Semreen, Ashenafi Kibret Sendekie, Yigit Can Senol, Subramanian Senthilkumaran, Sadaf G Sepanlou, Edson Serván-Mori, Yashendra Sethi, Seyed Mohammad Seyed Alshohadaei, Allen Seylani, Abubakar Sha'aban, Mahan Shafie, Arezoo Shafieioun, Muhammad Shahab, Shazlin Shaharudin, Muhammad Shahbaz, Syed Ahsan Shahid, Samiah Shahid, Wajeehah Shahid, Endrit Shahini, Farshad Shahkarami, Fatemeh Shahrahmani, Hamid R Shahsavari, Moyad Jamal Shahwan, Masood Ali Shaikh, Nafhat Shaikh, Alireza Shakeri, Ali Shakerimoghaddam, Ali S Shalash, Sunder Sham, Muhammad Aaqib Shamim, Mehran Shams-Beyranvand, Anas Shamsi, Alfiya Shamsutdinova, Dan Shan, Mohd Shanawaz, Abhishek Shankar, Ben David Geller Shapiro, Amin Sharifan, Javad Sharifi Rad, Manoj Sharma, Ravi Kumar Sharma, Bunty Sharma, Ujjawal Sharma, Avimanu Sharma, Bhoopesh Kumar Sharma, Vishal Sharma, Armin Shavandi, Ramzi Shawahna, Maryam Shayan, Ali Sheidaei, Aziz Sheikh, Mahabalesh Shetty, Suraj S Shetty, Lin-Hong Shi, Fang Shi, Belayneh Fentahun Shibesh, Desalegn Shiferaw, Tariku Shimels, Md Monir Hossain Shimul, Min-Jeong Shin, Rahman Shiri, Reza Shirkoohi, Aminu Shittu, Abdul-karim Olayinka Shitu, Ivy Shiue, Velizar Shivarov, Ambreen Shoaib, Shayan Shojaei, Sina Shool, Seyed Afshin Shorofi, Sunil Shrestha, Suleiman Adeiza Adeiza Shuaibu, Kerem Shuval, Nicole R S Sibuyi, Emmanuel Edwar Siddig, Mohammad Sidiq, Luís Manuel Lopes Rodrigues Silva, Diego Augusto Santos Silva, Noah Joseph Bernard Silva de Leonardi, Biagio Simonetti, Amit Singh, Balbir Bagicha Singh, Jasvinder A Singh, Baljinder Singh, Harmanjit Singh, Narinder Pal Singh, Puneetpal Singh, Satwinder Singh, Poornima Suryanath Singh, Akanksha Singh, Harpreet Singh, Surendra Singh, Bhim Pratap Singh, Kalpana Singh, Samer Singh, Abhinav Singh, Mukesh Kumar Sinha, Robert Sinto, Freddy Sitas, Dagne Feleke Siyoum, Natia Skhvitaridze, Valentin Yurievich Skryabin, Anna Aleksandrovna Skryabina, David A Sleet, Aalam Sohal, Md.Salman Sohel, Somaye Sohrabi, Anton Sokhan, Shipra Solanki, Solikhah Solikhah, Sameh S M Soliman, Aayushi Sood, Prashant Sood, Soroush Soraneh, Joan B Soriano, Michele Sorrentino, Fernando Sousa, Ireneous N Soyiri, Michael Spartalis, Chandrashekhar T Sreeramareddy, Bahadar S Srichawla, Shyamkumar Sriram, Devin Bailey Srivastava, Jeffrey D Stanaway, Nicholas Steel, Aleksandar Stevanović, Sebastian Straube, Peter Stubbs, Omer

Subasi, Narayan Subedi, Vetriselvan Subramaniyan, Hasnat Sujon, Thitiporn Sukaew, Surajo Kamilu Sulaiman, Auwal Garba Suleiman, Muhammad Suleman, Desy Sulistiyorini, Mark J M Sullman, Jing Sun, Haitong Zhe Sun, Xiaohui Sun, Mao-ling Sun, Zhuanlan Sun, Suraj Sundaragiri, David Sunkersing, Sumam Sunny, Chandan Kumar Swain, Tasmin L Symons, Lukasz Szarpak, Mindy D Szeto, Sree Sudha T Y, Payam Tabaee Damavandi, Rafael Tabarés-Seisdedos, Seyyed Mohammad Tabatabaei, Fatemeh Sadat Tabatabaei, Seyed Shahaboddin Tabatabaei, Shima Tabatabai, Celine Tabche, Ramin Tabibi, Mohammad Tabish, Takahiro Tabuchi, Santosh Kumar Tadakamadla, Buhari Abdullahi Tafida, Farzad Taghizadeh-Hesary, Zanan Mohammed-Ameen Taha, Yasaman Taheri Abkenar, Shima Tajabadi, Iman M Talaat, Mircea Tampa, Jacques Lukenze Tamuzi, Ker-Kan Tan, Shynar Tanabayeva, Haosu Tang, Guodong Tang, Mohsan Tanveer, Sarvenaz Taridashti, Ingan Ukur Tarigan, Mengistie Kassahun Tariku, Saba Tariq, Anika Tasnim, Seyed Mohammad Tavangar, Mebrahtu G. Tedla, Mohamad-Hani Temsah, Reem Temsah, Masayuki Teramoto, Azimeraw Arega Tesfu, Jay Tewari, Alireza Teymouri, Chandan Kumar Thakur, Rekha Thapar, Ismaeel Tharwat, Samar Tharwat, Hadiza Theyra-Enias, Arun James Thirunavukarasu, Muthu Thiruvengadam, Manuel Sebastian Thomas, Jansje Henny Vera Ticoalu, Mariya Vladimirovna Titova, Yves Joel Tochie Noutakdie, Sojit Tomo, Marcello Tonelli, Roman Topor-Madry, Ali Torkashvand, Mathilde Touvier, Marcos Roberto Tovani-Palone, Khaled Trabelsi, Thang Huu Tran, Quynh Thuy Huong Tran, Tam Quoc Minh Tran, Mai Thi Ngoc Tran, Nguyen Tran Minh Duc, Domenico Trico, Indang Trihandini, Samuel Joseph Tromans, Claudia Truppa, Gary Tse, Evangelia Eirini Tsermpini, Munkhtuya Tumurkhuu, Zhouting Tuo, Biruk Shalmeno Tusa, Sok Cin Tye, Stefanos Tyrovolas, Aniefiok John Udoakang, Himayat Ullah, Saeed Ullah, Atta Ullah, Riaz Ullah, Muhammad Umair, Lawan Umar, Muhammad Umar, Muhammad Umar, Bhaskaran Unnikrishnan, Dinesh Upadhya, Era Upadhyay, Dipan Uppal, Jibrin Sammani Usman, Kelechi Julian Uzor, Hande Uzunçıbuk, Pratyusha Vadagam, Asokan Govindaraj Vaithinathan, Pascual R Valdez, Mario Valenti, Zahir Vally, Jef Van den Eynde, Javad Varasteh, Joe Varghese, Priya Vart, Santosh Varughese, Tommi Juhani Vasankari, Sampara Vasishta, Srivatsa Surya Vasudevan, Alireza Vaysi, Ashleigh S Vella, Balachandar Vellingiri, Gowri Venkatraman Subramanian, Narayanaswamy Venketasubramanian, Nicholas Alexander Verghese, Poonam Verma, Megan Verma, Madhur Verma, Massimiliano Veroux, Georgios-Ioannis Verras, Dominique Vervoort, Ramesh Vidavalur, Simone Villa, Jorge Hugo Villafañe, David Villarreal-Zegarra, Francesco S Violante, Sharath Chaitanya Vipparthy, Luciano Magalhães Vitorino, Stein Emil Vollset, Avina Vongpradith, Theo Vos, Elpida Vounzoulaki, Linh Vu, Henok Toga Wada, Yasir Waheed, Megha Walia, Lindsey E Wallace, Agnes Wamuyu Wamai, Jin-Yi Wan, Arvinder Wander, Shu Wang, Ruixuan Wang, Xing Wang, Shaopan Wang, Qingzhi Wang, Jinyu Wang, Fang Wang, Wei Wang, Yanzhong Wang, Denny Wang, Youxin Wang, Wanzhou Wang, Yuan-Pang Wang, Mary Njeri Wanjau, Ahmed Bilal Waqar, Muhammad Waqas, Paul Ward, Stefanie Watson, Kosala Gayan Weerakoon, Fei-Long Wei, Xueying Wei, Robert G Weintraub, Daniel J Weiss, Ronny Westerman, Joanna L Whisnant, Taweewat Wiangkham, Yohanes Cakrapradipta Wibowo, Anggi Lukman Wicaksana, Nuwan Darshana Darshana Wickramasinghe, Dakshitha Praneeth Wickramasinghe, Angga Wilandika, Peter Willeit, Andrew Awuah Wireko, Gemechu Kumera Wirtu, Charles Shey Wiysonge, Abay Tadesse Woday, Marcin W Wojewodzic, Axel Walter Wolf, Tewodros Eshete Wonde, Yen Jun Wong, Daniel Tarekegn Worede, Minichil Chanie Chanie Worku, James Fan Wu, Zenghong Wu, Jinyi Wu, Felicia Wu, Peng Wu, Yihun Miskir Wubie, Qing Xia, Zhijia Xia, Hong Xiao, Lishun Xiao, Guangqin Xiao, Na Xiao, Wanqing Xie, Wanqing Xu, Xiaoyue Xu, Suowen Xu, Wang-Dong Xu, Site Xu, Mingyang Xue, Mukesh Kumar Yadav, Vikas Yadav, Sajad Yaghoubi, Saba Yahoo (Syed), Kazumasa Yamagishi, Xinxin Yang, Haibo Yang, Yuichiro Yano, Haiqiang Yao, Laiang Yao, Amir Yarahmadi, Habib Yaribeygi, Haya Yasin, Mohamed A Yassin, Yuichi Yasufuku, Sanni Yaya, Pengpeng Ye, Meghdad Yeganeh,

Ali Cem Yekdeş, Mohammad Hossein YektaKooshali, Getaneh Atikilt Yemata, Subah Abderehim Yesuf, Saber Yezli, Siyan Yi, Muluken Yigezu, Dehui Yin, Yazachew Engida Yismaw, Malede Berihun Yismaw, Dong Keon Yon, Naohiro Yonemoto, Mustafa Z Younis, Abdilahi Yousuf, Jian Yu, Yong Yu, Chuanhua Yu, Hui Yuan, Ghazala Yunus, Umar Yunusa, Siddhesh Zadey, Vesna Zadnik, Mubashir Zafar, Manijeh Zaghampour, Mondal Hasan Zahid, Emilia Zainal Abidin, Fathiah Zakham, Giulia Zamagni, Sojib Bin Zaman, Abu Sarwar Zamani, Hussaini Zandam, Alireza Zangeneh, Aurora Zanghì, Iman Zare, Kourosh Zarea, Shirin Zaresharifi, Michael Zastrozhin, Mohammed Zawiah, Mohammed G M Zeariya, Dawit Zemedikun, Abay Mulu Zenebe, Sebastian Zensen, Eyael M Zeru, Tiansong Zhan, Yongle Zhan, Xiaoyi Zhang, Liqun Zhang, Haijun Zhang, Yunquan Zhang, Beijian Zhang, Ning Zhang, Xiu-Hang Zhang, Meixin Zhang, Zhiqiang Zhang, Casper J P Zhang, Jinpeng Zhang, Zhongyi Zhao, Shenglin Zhao, Sheng Zhao, Ming-Hua Zheng, Anthony Zhong, Claire Chenwen Zhong, Juexiao Zhou, Jiayan Zhou, Bin Zhu, Abzal Zhumagaliuly, Hafsa Zia, Ghazal Zoghi, Mohamed Ali Zoromba, Rafat Mohammad Zrieq, Liesl J Zuhlke, Lilik Zuhriyah, Alimuddin Zumla, Sa'ed H Zyoud, Shaher H Zyoud, Ahed H Zyoud, Aleksandr Y Aravkin**, Christopher J L Murray**.

*Joint first authors
**Joint senior authors

Affiliations

Institute for Health Metrics and Evaluation (A E Schumacher PhD, P Zheng PhD, R M Barber BS, R W Aldridge PhD, S A Ali MS, J O Amlag MPH, C M Antony MA, C Bisignano MPH, E D Brewer, A Carter MPH, E Chung MD, H Comfort MPH, G T Culbreth PhD, X Dai PhD, Prof R Dandona PhD, Prof L Dandona MD, Prof L Degenhardt PhD, L Deitesfeld MA, Prof S D Dharmaratne MD, M A Dirac MD, R V Dominguez BS, K Estep MPA, Prof V L Feigin PhD, L S Flor MPH, J E Fuller MLIS, N Fullman MPH, Prof E Gakidou PhD, E B Hamilton MPH, Prof S I Hay FMedSci, J He MSc, C A Henson MPH, J A Houser BA, A W Hsu MS, K S Ikuta MD, D P Jones BS, K Joskowitz MAS, N J Kassebaum MD, M B Kassel BA, M Kirk MPH, H H Kyu PhD, D Lasher MS, Prof S S Lim PhD, Prof R Lozano MD, K L Maass PhD, E A May MS, T Mestrovic PhD, Prof A H Mokdad PhD, J F Mosser MD, A Movo MPH, Prof M Naghavi PhD, M Ng PhD, M Pasovic MEd, S A Pease BS, D M Pigott PhD, C Razo PhD, H E Robinson-Oden MLIS, J J Rosauer BA, E Rosenblad MPH, H Saqib MA, B D G Shapiro MA, N J B Silva de Leonardi PhD, J D Stanaway PhD, N A Verghese BA, M Verma MSc, Prof S E Vollset DrPH, A Vongpradith BA, Prof T Vos PhD, L E Wallace MPA, D Wang BA, S Watson MS, J L Whisnant MPH, M Zhang MS, A Y Aravkin PhD, Prof C J L Murray DPhil), Department of Health Metrics Sciences, School of Medicine (P Zheng PhD, R W Aldridge PhD, X Dai PhD, Prof R Dandona PhD, Prof S D Dharmaratne MD, M A Dirac MD, L S Flor MPH, Prof E Gakidou PhD, Prof S I Hay FMedSci, N J Kassebaum MD, H H Kyu PhD, Prof S S Lim PhD, Prof R Lozano MD, A Misganaw PhD, Prof A H Mokdad PhD, Prof M Naghavi PhD, D M Pigott PhD, B Sartorius PhD, J D Stanaway PhD, Prof S E Vollset DrPH, Prof T Vos PhD, A Y Aravkin PhD, Prof C J L Murray DPhil), Department of Pediatrics (E Chung MD), Department of Family Medicine (M A Dirac MD), Department of Applied Mathematics (A W Hsu MS, A Y Aravkin PhD), Population, Fertility, and Mortality Team (K Joskowitz MAS), Department of Anesthesiology & Pain Medicine (N J Kassebaum MD), Department of Global Health (S Kochhar MD), Department of Epidemiology (H Zia BDS), University of Washington, Seattle, WA, USA; Amity Institute of Public Health (Prof B A PhD), Amity University, Uttar Pradesh, India; Shahid Beheshti University of Medical sciences (M Aalipour MD), Department of Anesthesiology (Prof D Abtahi MD, S Salimi MD, S Seyed Alshohadaei MD, A Shakeri MD), National Nutrition and Food Technology Research Institute (M Ajami PhD, Z Hadian PhD),

School of Medicine (G Arjmand MD, M Asadi Anar MD, J Behjati MD, S Madinezad MD, S Sadat Rafiei MD), Research Institute of Dental Sciences (Prof S Asgary MSc), Shahid Rajii Hospital (E Baghizadeh MD), Cancer Research Center (M Bayat MD), Internal Medicine Department (H Borhany MD), Department of Anesthsia, Critical Care and Pain Medicine (Prof A Dabbagh MD), Department of Medical Genetics (M Ghasemi PhD), Center for Comprehensive Genetic Services (M Ghasemi PhD), Obstetrics and Gynecology Department (E Ghotbi MD), Department of Oral and Maxillofacial Surgery (M Golkar MSc), Obesity Research Center (A Haj-Mirzaian MD), Urology and Nephrology Research Center (H Hesami MD), Ophthalmic Research Center (ORC) (H Hesami MD, M Shayan MD), Department of general medicine (M Karimi MD), Department of Neurosurgery (H Khayat Kashani MD), Ophthalmic Epidemiology Research Center (Z Khorrami PhD), Social Determinants of Health Research Center (Prof A Kolahi MD, A Nikoobar BSc, M Rashidi MD), Anesthesiology Research Center (F Madadi MD), Research Institute for Endocrine Sciences (S Masrouri MD), Skull Base Research Center (I Mohammadzadeh MD), Department of audiology, school of rehabilitation (S Mousavi PhD), Department of Immunology (A Rasouli-Saravani PhD), Department of Epidemiology (Prof S Sabour PhD), school of public health (S Sadat Rafiei MD), Department of Health (M Safari PhD), Faculty of Medicine (M Sagharichi HSdipl), School of Medical Education and Learning Technologies (S Sohrabi PhD), Department of Medical Education (S Tabatabai PhD), Department of Dermatology (S Zaresharifi MD), Shahid Beheshti University of Medical Sciences, Tehran, Iran; Department of Nursing (H Aalruz PhD), Al Zaytoonah University of Jordan, Amman, Jordan; Department of Radiation Oncology (H S Ababneh MD), Cardiovascular Research Center (S Abohashem MPH), Department of Radiology (A Haj-Mirzaian MD, X Liu PhD), Department of Anesthesia, Critical Care and Pain Medicine (Prof J Kang PhD), Department of Orthopaedics (O Subasi PhD), Massachusetts General Hospital, Boston, MA, USA (A Eighaei Sedeh MD, M Kim MD); School of Health & Life Sciences (U O Abaraogu PhD), University of the West of Scotland, Paisley, UK; Department of Medical Rehabilitation (U O Abaraogu PhD), Department of Pharmacology and Therapeutics (Prof O E Onwujekwe PhD), University of Nigeria Nsukka, Enugu, Nigeria; Department of Legal and Economic Studies (Prof C Abbafati PhD), Department of Public Health and Infectious Diseases (M S Cattaruzza PhD), La Sapienza University, Rome, Italy; Centre for Regenerative Medicine and Health (N Abbas PhD), Chinese Academy of Sciences, Hong Kong, China; Department of Neuroscience (N Abbas PhD), Department of Infectious Diseases and Public Health (I Elsohaby PhD, G Fekadu PhD), Department of Biomedical Sciences (W Jin MD), City University of Hong Kong, Hong Kong, China; Department of Internal Medicine (M Abbasifard MD), Clinical Research Development Unit (M Abbasifard MD), Department of Epidemiology and Biostatistics (Prof M Rezaeian PhD), Rafsanjan University of Medical Sciences, Rafsanjan, Iran; Department of Medicine (F Abbaspour MD), Department of Global Health Sciences (S Ghasemi Assl MD), Division of Cardiology (J Noubiap MD), Department of Neurosurgery (A Orscelik MD, Y Senol MD), School of Nursing (J Ouner PhD), Department of Bioengineering and Therapeutical Sciences (Prof M Zastrozhin PhD), University of California San Francisco, San Francisco, CA, USA; College of Pharmacy (A H A Abd Al Magied MSc), Nonlinear Dynamics Research Center (NDRC) (Prof S Momani PhD), Center for Medical and Bio-Allied Health Sciences Research (Prof M J Shahwan PhD, A Shamsi PhD), College of Pharmacy and Health Science (H Yasin PhD), Ajman University, Ajman, United Arab Emirates (Prof N Hassan PhD); Department of Epidemiology (S Abd ElHafeez DrPH), Pediatric Dentistry and Dental Public Health Department (Prof O A A Elmeligy PhD), Tropical Health Department (R M Ghazy PhD), Department of Pathology (Prof I M Talaat PhD), Alexandria University, Alexandria, Egypt; Hull York Medical School (M A Abdalla PhD), University of Hull, Hull, UK; Department of Biology (Prof E M Abdallah PhD), College of Applied Medical Sciences (Prof F A Alhumaydhi PhD), Department of Health Informatics (Q Jamal PhD), Qassim

University, Buraydah, Saudi Arabia; School of Nursing (Prof N M I Abdel Razeq PhD), The Univeristy of Jordan, Amman, Jordan; Basic Science Department (Prof R Abdel-Hameed PhD), University of Ha'il, Hail, Saudi Arabia; Chemistry Department (Prof R Abdel-Hameed PhD), Internal Medicine Department (O A Abdelwahab MD), Department of Zoology and Entomology (A I Hasaballah PhD, M G M Zeariya PhD), Al-Azhar University, Cairo, Egypt; Department of Medical Laboratory Science (Prof W M Abdel-Rahman PhD), Clinical Sciences Department (Prof E Abu-Gharbieh PhD, H J Barqawi MPhil, Prof R Halwani PhD, Prof A A Maghazachi PhD, Prof M M Saber-Ayad PhD, N Saheb Sharif-Askari PhD, Prof I M Talaat PhD), Department of Nursing (Prof S Abuhammad PhD), Department Pharmacy Practice and Pharmacotherapeutics (A Y Abuhelwa PhD), Department of Restorative Dentistry (A B Acharya PhD), College of pharmacy (S M Aleidi PhD), College of Pharmacy (H Y Alniss PhD, Prof M H Semreen PhD), Department of Pharmacy Practice and Pharmacotherapeutics (Prof K H Alzoubi PhD), College of Medicine (Prof A Amin PhD, Prof R Halwani PhD, Prof M A Saleh PhD), Center of Excellence of Cancer Research (Prof R Bendardaf PhD), Department of Basic Biomedical Sciences (Prof Y Bustanji PhD), Department of Basic Medical Sciences (M A Eladl PhD, Prof W El-Huneidi PhD), Sharjah Institute for Medical Research (N M Elemam PhD), Basic Medical Sciences Department (A B Elmoselhi PhD), Research Institute of Medical & Health Sciences (A B Elmoselhi PhD, Prof M H Semreen PhD), College of Health Sciences (S V Gopalani PhD), Department of Clinical Sciences (Prof M M Ramadan PhD), Sharjah Institute of Medical Sciences (F Saheb Sharif-Askari PhD), Department of Medicinal Chemistry (S S M Soliman PhD), University of Sharjah, Sharjah, United Arab Emirates (K A Altirkawi MD); Department of Tropical Medicine and Infectious Diseases (S Abd-Elsalam PhD), Tanta University, Tanta, Egypt; Department of Medicine (P Abdi BEng), Memorial University, St. John's, NL, Canada; Minimally Invasive Surgery Research Center (A Abdollahi MD), Neuroscience Research Center (S Aghajanian MD, M Jameie MD), Health Management and Economics Research Center (J Arabloo PhD, H Ayatollahi PhD), Department of Health Information Management (H Ayatollahi PhD), School of Medicine (M Bastan MD, S Eghdami MD), Department of Medical Laboratory Sciences (F Dorostkar PhD), Iran University of Medical Sciences (F Eghbali MD), Preventive Medicine and Public Health Research Center (Prof B Eshrati PhD, Prof M Moradi-Lakeh MD), Department of Medicine (M Fotouhi MD), Department of Ophthalmology (H Hasani MD), Department of Biostatistics (M Imani MSc), Department of Surgery (A Jaliliyan MD), Department of Obstetrics & Gynecology (P Khalili MD), Bone and Joint Reconstruction Research Center (M Khonji MD), Department of Health Economics (M Khosravi PhD), Gastrointestinal and Liver Diseases Research Center (Prof M Moradi-Lakeh MD), Antimicrobial Resistance Research Center (K Mozahheb Yousefi MD), Hazrat-e Rasool General Hospital (K Mozahheb Yousefi MD), Physiology Research Center (H Pazoki Toroudi PhD), Department of Physiology (H Pazoki Toroudi PhD), Colorectal Research Center (A Sarveazad PhD), Center for Technology and Innovation in Cardiovascular Informatics (S Shool MD), The Five Senses Health Institute (F Taghizadeh-Hesary MD), Iran University of Medical Sciences, Tehran, Iran; Department of Medicine (Prof M Abdoun PhD), Faculty of Medicine (H Rahmoune MD), LIRSSEI Research Lab (H Rahmoune MD), University of Setif Algeria, Sétif, Algeria; Department of Health, Sétif, Algeria (Prof M Abdoun PhD); Faculty of Veterinary Medicine (A Abdous MD), Young Researchers and Elite Club (M Jokar DVM), Islamic Azad University, Karaj, Iran; Community and Maternity Nursing Unit (D M Abdulah MPH), Department of Pathology and Microbiology (M S Ahmed PhD), Duhok Research Centre (Z M Taha PhD), University of Duhok, Duhok, Iraq; National Institute of Epidemiology (R Abdulkader PhD), Indian Council of Medical Research, Chennai, India; Department of Physiotherapy (A Abdullahi PhD, A W Awotidebe PhD, J S Usman PhD), Department of Community Medicine (Prof M A Gadanya MD), Department of Nursing Science (M Ladan PhD), Bayero University Kano, Kano, Nigeria; Department of

Physiotherapy (A Abdullahi PhD), Federal University Wukari, Wukari, Nigeria; Department of Pharmacognosy Faculty of Pharmacy (A S Abdulraheem BSc), Department of Paediatrics (C I Esezobor MB), Department of Psychiatry (Prof A T Olagunju PhD), University of Lagos, Lagos, Nigeria; Department of Epidemiology and Biostatistics (H A Abebe Getahun MSc), Department of Pharmacology (Z D Kifle MSc), Department of Health Systems and Policy (W D Negash MPH), School of Nursing (H B Netsere MSc), Department of Clinical Pharmacy (A K Sendekie MSc), Department of Pharmacy (M C Worku MSc), University of Gondar, Gondar, Ethiopia; Yale School of Medicine (P Abedi MD), Iran University of Medical Sciences, New Haven, USA; Neuroendocrine Department (P Abedi MD), Department of Radiology (S Abohashem MPH), Dana-Farber Cancer Institute (C Cao PhD), Nutrition Department (G Dalla Costa MD), T. H. Chan School of Public Health (S B Kankam MD), Department of Ophthalmology (Prof J H Kempen PhD, M Shayan MD), Department of Medicine (M Kokkorakis BSc, F Tabatabaei MD), Department of Health Policy and Oral Epidemiology (Z S Natto DrPH), Department of Global Health and Social Medicine (S Onie PhD), Cardiovascular Division (J W Ostrominski MD), T.H. Chan School of Public Health (P M S Pradhan MD), Division of General Internal Medicine (Prof A Sheikh MD), Joslin Diabetes Center (S Tye PhD), Department of Social and Behavioral Sciences (W Xu MPH), Harvard Medical School (A Zhong MA), Harvard University, Boston, MA, USA; Department of Emergency Medicine (A Abedi MD), Department of Food Safety and Hygiene (M Aminzare PhD), School of Medicine (M Ashrafi MD, H Nasiri MD), Department of Critical Care and Emergency Nursing (N Hanifi PhD), Zanjan University of Medical Sciences, Zanjan, Iran; School of Pharmacy (A Abejew MSc, T T Alemayehu MSc), Department of Midwifery (A Abie MSc, B A Alemayehu MSc, F Alemnew MSc, A M Aweke MSc, W F Balcha MSc, E A Kassahun MSc, A T Nega MSc, A A Tesfu MSc), Department Nutrition and Dietetics (M T Bayih MSc), College of Medicine and Health Science (A N Belay MSc, K Y Gete MD), Department of Public Health (M Belayneh PhD), Department of Emergency and Critical Care Nursing (A G Belayneh MSc), Department of Nursing (A Y Berhie MSc), Department of Environmental Health (T S Bunare MPH), Department of Physiology (D Demeke MSc), Department of Nutrition and Dietetics (H A Derseh MPH), Department of Health Informatics (H A Guadie MPH), Department of Medical Microbiology (A D Habteyohannes PhD), School of Public Health (B A Mekonnen MPH), Department of Medical Laboratory Science (A Melese MSc), Department of Adult Health Nursing (E A Mengistie MSc), College of Medicine and Health Sciences (H B Netsere MSc), Department of Emergency Medicine and Critical Care Nursing (Y M Wubie MPH), Department of Pharmacology (Y E Yismaw MSc), Department of Pharmacy (M Yismaw MSc), Bahir Dar University, Bahir Dar, Ethiopia; Postgraduate Department (Prof R Abeldaño Zuñiga PhD), University of Sierra Sur, Miahuatlan de Porfirio Diaz, Mexico; Yhteiskuntadatatieteen Keskus (Centre for Social Data Science) (Prof R Abeldaño Zuñiga PhD), Department of Public Health (Prof M Kivimäki PhD, Prof T Lallukka PhD), University of Helsinki, Helsinki, Finland (T J Meretoja MD); Department of Biomedical Sciences (S Abidi PhD), Nazarbayev University School of Medicine, Astana, Kazakhstan; Department of Community Medicine (Prof O Abiodun MPH), Babcock University, Ilishan-Remo, Nigeria; Department of Internal Medicine (O O Abiodun FWACP), Federal Medical Centre, Abuja, Nigeria; Department of Family and Community Health (R G Aboagye MPH), School of Basic and Biomedical Sciences (D Adedia PhD), Department of Epidemiology and Biostatistics (L A Adzigbli BSc, R K Dowou MPhil), Institute of Health Research (P T Doegah PhD, M Immurana PhD), Department of Nursing (F K Nyande PhD), Department of Microbiology and Immunology (Prof V N Orish PhD), University of Health and Allied Sciences, Ho, Ghana; School of Population Health (R G Aboagye MPH, X Feng PhD, Prof B A Saddik PhD, Prof A E Schutte PhD, X Xu PhD), St George and Sutherland Clinical School (H Akbarialiabad MD), The Graduate School of Biomedical Engineering (Prof H Alinejad Rokny PhD), Transport and Road Safety (TARS) Research Centre

(S Boufous PhD), National Drug and Alcohol Research Centre (Prof L Degenhardt PhD), The George Institute for Global health (F Haghdoost PhD), International Centre for Future Health Systems (J Lin PhD), School of Medicine (Prof P K Maulik PhD), Discipline of Psychiatry and Mental Health (Prof P B Mitchell MD), Centre for Social Research in Health (S R Okeke PhD), Black Dog Institute (S Onie PhD), Centre for Healthy Brain Ageing (CHeBA) (S Röhr PhD, S Röhr PhD), School of Psychiatry (Prof P S Sachdev MD), Centre for Primary Health Care and Equity (CPHCE) (F Sitas PhD), Centre for Healthy Brain Ageing (A S Vella PhD), The George Institute for Global Health (P Ye PhD), University of New South Wales, Sydney, NSW, Australia; Department of Sport, Exercise and Rehabilitation (U S Abonie PhD), Northumbria University, Newcastle, UK; Basic Science Department, Preparatory Year (N M Abourashed PhD), University of Ha'il, Ha'il, Saudi Arabia; Zoology Department, Faculty of Science (N M Abourashed PhD), Benha University, Benha, Egypt; Department of Physical Pharmacy and Pharmacokinetics (M Abouzid PharmD), Chair and Department of Medical Microbiology (Prof T M Karpiński DDS), Poznan University of Medical Sciences, Poznan, Poland; Cardiovascular Disease (D Abramov MD), Loma Linda University Medical Center, Loma Linda, CA, USA; Department of Pediatric Dentistry (Prof L Abreu PhD), Department of Maternal-Child Nursing and Public Health (Prof D C Malta PhD, Prof F P Matozinhos PhD, E J S Prates BS), Department of Clinical Medicine (Prof B R Nascimento PhD), Clinical Hospital (Prof B R Nascimento PhD), Department of Applied Nursing (Prof M O Pereira PhD), Department of Internal Medicine (Prof A P Ribeiro MD), Centre of Telehealth (Prof A P Ribeiro MD), Escola de Enfermagem da UFMG (Prof T M Ribeiro da Silva PhD), Federal University of Minas Gerais, Belo Horizonte, Brazil; Clinical Pharmacy and Therapeutics Department (Prof R K Abu Farha PhD), Applied Science Research Center (A B Al-Tammemi MPH), Faculty of Nursing (E H Othman PhD), Applied Science Research Centre (R M Zrieq PhD), Applied Science Private University, Amman, Jordan; Community Helth Nursing Department (F H A Abuadas PhD), Preventive Dentistry Department (Prof M K Alam PhD), Jouf University, Sakaka, Saudi Arabia; Graduate School of Public Health (A K Abubakar MPH), St. Luke's International University, Tokyo, Japan; Division of Population Data Science (A K Abubakar MPH), National Cancer Center, Tokyo, Japan; Department of Pharmacology and Toxicology (B Abubakar PhD), Department of Pharmaceutics and Pharmaceutical Technology (N Aminu PhD), Department of Veterinary Microbiology (M B Bello PhD), Department of Veterinary Public Health and Preventive Medicine (B Garba PhD, A Shittu MSc), Medical Microbiology Department (Prof Y Mohammed FWACP), Clinical Pharmacy and Pharmacy Practice (S A Shuaibu PhD), Usmanu Danfodiyo University, Sokoto, Sokoto, Nigeria; Department of Biochemistry and Nutrition (K Oyebola PhD), Nigerian Institute of Medical Research, Lagos, Nigeria (B Abubakar PhD); Department of Biopharmaceutics and Clinical Pharmacy (Prof E Abu-Gharbieh PhD), College of Pharmacy (Prof S Aburuz PhD), School of Nursing (Prof M M Ahmad PhD), The University of Jordan School of Medicine (Prof M A Al-Abbadi MD), Department of Radiology and Nuclear Medicine (Prof M E Juweid MD), University of Jordan, Amman, Jordan; Maternal and Child Health Nursing (Prof S Abuhammad PhD), Jordan University of Science and Technology, Irbid, Jordan; Medical Research Center (H J Abukhadijah MPH), Department of Pharmacy (D Abushanab MSc), Department of Geriatric and Long Term Care (H Al Hamad MD, B Sathian PhD), Rumailah Hospital (H Al Hamad MD), Department of Surgery (A Alansari MD), Surgical Research Section/ Surgery Department (A R Al-Qudimat MPH), Nursing and midwifery Research Department (G Joy MSc), Corporate Nursing and midwifery Research (J Kunjavara PhD), Corporate Nursing and Midwifery Research Department (K Mannethodi MPH), Nursing & Midwifery Research Department (NMRD) (A J Nashwan PhD), Research Department (K Singh PhD), Hematology Section (Prof M A Yassin MD), Hamad Medical Corporation, Doha, Qatar; Department of Pharmacology and Therapeutics (Prof S Aburuz PhD), Institute of Public Health (Prof L A Ahmed PhD, R H Al-Rifai PhD, Prof

M Grivna PhD), College of Medicine and Health Sciences (Prof M Z Allouh PhD), Pharmacology and Therapeutic Department (M El-Deyarbi PhD), Department of Veterinary Medicine (H O Khalifa PhD), United Arab Emirates University, Al Ain, United Arab Emirates; Department of Biochemistry and Molecular Medicine (A Abu-Zaid PhD), College of Medicine (Prof O Baltatu PhD), College of Pharmacy (R Temsah PharmD), Alfaisal University, Riyadh, Saudi Arabia; College of Graduate Health Sciences (A Abu-Zaid PhD), Ophthalmology Department (M Delsoz MD), Department of Ophthalmology (A Nabavi MD), University of Tennessee, Memphis, TN, USA; Department of Population Health (M Achore PhD), Hofstra University, Hempstead, NY, USA; Department of Clinical Medicine (Prof J M Acuna MD), American University of Antigua, Coolidge, Antigua and Barbuda; FIU Robert Stempel College of Public Health & Social Work (Prof J M Acuna MD), Robert Stemple College of Public Health and Social Work (S Chowdhury MPH), Department of Epidemiology (S Roy MPH, S Roy MPH), Florida International University, Miami, FL, USA; Melbourne School of Population and Global Health (T Adair PhD), Population Interventions Unit (B Dhungel DrPH), Department of Medicine (Prof F K Jebasingh DM), School of Health Sciences (A Meretoja MD), University of Melbourne, Melbourne, VIC, Australia; Department of Diagnostic and Interventional Radiology (L C Adams PhD), School of Medicine and Health (F Busch MD), Technical University of Munich, Munich, Germany; Department of Medicine (R M Gibson PhD), Cardiothoracic Surgery (D N Kamtam MS), Division of Pediatric Hospital Medicine (R P Mediratta MD), School of Medicine (J Zhou PhD), Stanford University, Palo Alto, CA, USA (L C Adams PhD); Institute of Cardiovascular Diseases (O M Adebayo MD), Department of Physiotherapy (M A Adesina BPT), Department of Veterinary Medicine (T E Adeyeoluwa PhD), Department of Epidemiology and Medical Statistics (R F Afolabi PhD, A F Fagbamigbe PhD), Psychiatry Department (O O Ayinde MD), Department of Health Promotion and Education (S Ibitoye PhD), Department of Special Education (U Jacob PhD), Counselling and Human Development Studies (D O Okeke-Obayemi BSc), College of Medicine (O I Olabisi PhD), Department of Medicine (O V Olalusi MD, Prof M O Owolabi DrM), University of Ibadan, Ibadan, Nigeria; Department of Community Medicine (O S Ilesanmi PhD), Department of Neurology (O V Olalusi MD), Department of Medicine (Prof M O Owolabi DrM), University College Hospital, Ibadan, Ibadan, Nigeria (O M Adebayo MD); Department of Microbiology (T A Adebisi BSc), Ladoke Akintola University, Osogbo, Nigeria; NMC Healthcare (T A Adebisi BSc), Independent Consultant, Sharjah, United Arab Emirates; Department of Immunology (K A Adedokun MSc), Roswell Park Comprehensive Cancer Center, Buffalo, NY, USA; Graduate Program Division (K A Adedokun MSc), University at Buffalo, Buffalo, NY, USA; Department of Pediatrics (O E Adegbile MD), East Tennessee State University, Johnson City, TN, USA; Center for Cardiovascular Risk Research (O E Adegbile MD), Center for Cardiovascular Risk Research, Johnson City, TN, USA; Translational Research Team (N A Adegoke PhD), Melanoma Institute Australia (N A Adegoke PhD), The University of Sydney, Sydney, NSW, Australia; Department of Family Medicine (O T Adeleke MD), College of Health Sciences (O I Olabisi PhD), Bowen University, Iwo, Nigeria; Department of Family Medicine (O T Adeleke MD), Bowen University Teaching Hospital, Ogbomoso, Nigeria; Slum and Rural Health Initiative Research Academy (M A Adesina BPT), Slum and Rural Health Initiative, Ibadan, Nigeria; Department of Microbiology (I A Adesina PhD), Department of Biosciences and Biotechnology (M D Agoi MSc), University of Medical Sciences, Ondo, Ondo City, Nigeria; Division of Biostatistics and Epidemiology (O O Adetokunboh PhD), Department of Epidemiology (J L Tamuzi MSc), Department of Global Health (Prof C S Wiysonge MD), Stellenbosch University, Cape Town, South Africa; Department of Pharmacology and Therapeutics (T E Adeyeoluwa PhD), Department of Environmental and Occupational Health (B S Anuoluwa MPH), Department of Microbiology (I A Anuoluwa PhD, O O Bello PhD, T C Ekundayo PhD), Department of Physiology (V O Emojevwe PhD), Department of

Biosciences and Biotechnology (O T Oyeyemi PhD, A J Udoakang PhD), Mathematical and Computer Sciences (O Peter PhD), University of Medical Sciences, Ondo, Ondo, Nigeria; School of Public Health (M T Adhana PhD), Mekelle University, Mekelle, Ethiopia; Department of Community Medicine (Prof K Adhikari PhD), Tribhuvan University, Bharatpur, Nepal; Public Health Section (Prof K Adhikari PhD), Himalayan Environment and Public Health Network (HEPHN), Chitwan, Nepal; Department of Fisheries and Marine Bioscience (R K Adhikary PhD), Jashore University of Science and Technology, Jashore, Bangladesh; Research School of Population Health (R K Adhikary PhD), School of Medicine and Psychology (D Ahmad PhD), National Centre for Epidemiology and Population Health (R A Burns PhD), Australian National University, Canberra, ACT, Australia; Apollo Institute Of Medical Sciences & Research Chittoor (Prof U Adiga PhD), Apollo Hospital, Chittoor, India; School of Medicine (T Adl Parvar MD, A Azarboo MD, N Kazemi rad MD, S Khanmohammadi MD), Urology Research Center (Prof S Aghamir PhD), Universal Scientific Education and Research Network (USERN) (M Amirzade-Iranaq DDS), Orthopedic Department (N Anaraki MD), Digestive Diseases Research Institute (A Anoushiravani MD, S G Sepanlou MD), Department of Health Information Management (S Ayyoubzadeh PhD), Rheumatology Research Center (A Azizan PhD), School of Public Health (N Bahmanziari PhD, A Sheidaei PhD), Non-communicable Diseases Research Center (M Bastan MD, M Rashidi MD, N Rezaei MD), Pastor Institute (M Bayat MD), Department of Scientific Research (F Chichagi MD), Iranian Research Center for HIV/AIDS (IRCHA) (O Dadras PhD), Department of Neurosurgery (P Delbari MD), Department of Radiology (R Elahi MD, A Teymouri MD), Multiple Sclerosis Research Center (S Eskandarieh PhD), Pediatric Infectious Disease Research Center (M Farahmand PhD), Dentistry Research Institute (F Farshad DDS), Obesity and Eating Habits Research Center (F Farsi MD), Neurology Department of Imam Khomeini hospital (M Ghasemi MD), Department of Ophthalmology (Prof F Ghassemi MD, A Mahmoudi MD), Sina Trauma and Surgery Research Center (M Hassan Zadeh Tabatabaei MD, M Jalloh MD, Prof V Rahimi-Movaghar MD, Z Ramezani MD, Prof P Salamati MD, S Shool MD), Iranian Center of Neurological Research (M Jameie MD), Cardiac Primary Prevention Research Center (S Kazemian MD), Department of Cardiac Electrophysiology (S Kazemian MD), Children's Medical Center (Prof F Kompani MD), Digestive Diseases Research Institute (DDRI) (Prof R Malekzadeh MD), Department of Epidemiology and Biostatistics (M Mansournia PhD), Tehran Heart Center, Cardiovascular Diseases Research Institute (A Mousavi MD, S Shojaei MD), Department of Physiotherapy (Prof N Nakhostin Ansari PhD), Research Center for War-affected People (Prof N Nakhostin Ansari PhD), Digestive Diseases Research Center (P Parhizkar Roudsari MD), Cardiac Research Center (P Parhizkar Roudsari MD), Department of Bioinformatics (M Piroozkhkah MD), Department of Infectious Diseases and Tropical Medicine (E Rajabi MD), Research Center for Immunodeficiencies (A Saghazadeh MD), Department of Research and Development (P Salimi Pormehr MSc), Epidemiology and Biostatistics (E Sanjari PhD, E Sanjari PhD), Department of Neurology (M Shafie MD), Department of Internal Medicine (F Shahkarami MD), Cancer Research Center (R Shirkoohi PhD), Cancer Biology Research Center (R Shirkoohi PhD), Student Scientific Research Center (S Shojaei MD), Department of Pathology (Prof S Tavangar MD), Tehran University of Medical Sciences, Tehran, Iran (A Azizan PhD); Department of Biology (Prof M Adnan PhD, Prof M Saeed PhD), College of Applied Medical Science (S Ashraf PhD), Medical Laboratory Sciences (N K Binsaleh PhD), Department of Chemistry (A Haque PhD), College of Medicine (Y S Khan MD), Department of Biochemistry (Prof M Kuddus PhD), Medical and Diagnostic Research Centre (Prof C T Sreeramareddy MD), Department of Basic Science (G Yunus PhD), Family and Community Medicine Department (M Zafar PhD), Department of Public Health (M G M Zeariya PhD), Department of Medical-Surgical Nursing (R M Zrieq PhD), University of Hail, Hail, Saudi Arabia; Department of Public Health (Q Adnani PhD), Universitas Padjadjaran (Padjadjaran

University), Bandung, Indonesia; National Institute on Minority Health and Health Disparities (D Adzrago PhD), National Human Genome Research Institute (NHGRI) (N Horita PhD), Center for Translation Research and Implementation Science (G A Mensah MD), National Institutes of Health, Bethesda, MD, USA (A Grover MD); Department of Public Health and Preventive Medicine (G Affinito PhD), Department of Public Health (C Fiorilla MD, R Palladino MD, M Sorrentino MD), University of Naples "Federico II", Naples, Italy; Technical Services Directorate (A A Afolabi MPH), MSI Nigeria Reproductive Choices, Abuja, Nigeria; Department of Community Medicine (Prof S Afzal PhD), King Edward Memorial Hospital, Lahore, Pakistan; Department of Public Health (Prof S Afzal PhD), Public Health Institute, Lahore, Pakistan; Department of Public Health (G B Agafari PhD, M H Nunemo MPH), Department of Health Education and Health Promotion (F D Agide PhD), Wachemo University, Hossana, Ethiopia; MM College of Pharmacy (N Aggarwal PhD), Maharishi Markandeshwar (Deemed to be University), Ambala, India; Department of Orthopedic Surgery and Sports Medicine (M Aghaalikhani MD), Boston Children's Hospital, boston, MA, USA; Department of Neurosurgery (S Aghajanian MD), School of Medicine (M Shams-Beyranvand MSc), Alborz University of Medical Sciences, Karaj, Iran; Health Research and Innovation Sciences Center (C Agostinis Sobrinho PhD), Health Research and Innovation Science Centre (R C D Espírito Santo PhD), Klaipeda University, Klaipeda, Lithuania; SPRINT Sport Physical Activity and Health Research & Innovation Center (C Agostinis Sobrinho PhD), Sport Physical Activity and Health Research & Innovation Center (SPRINT) (Prof L M L R Silva PhD), Polytechnic Institute of Guarda, Guarda, Portugal; Trivedi School of Biosciences (Prof A Agrawal PhD), Ashoka University, Sonipat, India; Department of Public Health Sciences (W Agyemang-Duah PhD), Queen's University, Kingston, ON, Canada; School of Public Health (B O Ahinkorah MPhil), School of Life Sciences (G Liu PhD), Discipline of Physiotherapy (P Stubbs PhD), University of Technology Sydney, Sydney, NSW, Australia; Department of Clinical Pharmacy (R Ahmad PhD), Advanced Medical & Dental Institute (M Aziz PhD), Universiti Sains Malaysia, Penang, Malaysia; Department of Pharmacy Practice (R Ahmad PhD), The Islamia University of Bahawalpur, Bahawalpur, Pakistan; Health Research Institute (D Ahmad PhD), University of Canberra, Canberra,ACT, NSW, Australia; Biological Production Unit National Institute of Health Islamabad Pakistan (F Ahmad PhD), National Institute of Health, Islamabad, Pakistan; World Health Organization (F Ahmad PhD), World Health Organisation, Islamabad, Pakistan; College of Medicine (A Ahmad PhD, M Tabish MPharm, H Ullah MBBS), Shaqra University, Shaqra, Saudi Arabia; Department of Health Informatics (K Ahmad PhD), Qassim University, Buraidha, Saudi Arabia; School of Public Health (T Ahmad PhD), Zhejiang University, Hangzhou, China; College of Medicine (W Ahmad PhD), University of Cincinnati, Cincinnati, OH, USA; Institute of Research and Development (A Ahmed PhD), Duy Tan University, Da Nang, Viet Nam; Centre for Research Impact & Outcome (A Ahmed PhD), Chitkara University, Punjab, India; College of Medicine and Public Health (M B Ahmed PhD, G R Naik PhD), Department of Nursing and Health Sciences (S Shorofi PhD), Flinders University, Adelaide, SA, Australia; Faculty of Public Health (M B Ahmed PhD), Department of Health Behavior and Society (L A A Ayana MPH), School of Pharmacy (H K Kebede MSc), Department of Epidemiology (D Shiferaw MPH), Jimma University, Jimma, Ethiopia (B Feyisa MPH); Institute of Endemic Diseases (A Ahmed MSc), Department of Oral Rehabilitation (N T Hashim PhD), Faculty of Medicine (K A H Mohamed Ahmed MD), Unit of Basic Medical Sciences (E E Siddig MD), University of Khartoum, Khartoum, Sudan; Swiss Tropical and Public Health Institute (A Ahmed MSc), Department of Ophthalmology (Prof Z Gatzioufas PhD), University of Basel, Basel, Switzerland; Department of Pharmacy Practice (A Ahmed PhD), Riphah Institute of Pharmaceutical Sciences, Islamabad, Pakistan; Division of Infectious Diseases and Global Public Health (IDGPH) (A Ahmed PhD), Moores Cancer Center (S Luo PhD), University of California San Diego, San Diego, CA, USA;

Maternal and Child Health Division (A Ahmed MS, S Ahmed MDS, R Banik MS, S Noor MS, N Saha MSc), Maternal and Child Health Division (MCHD) (M Al-Zubayer MSc, A Sayeed MSc), Department of Maternal and Child Health (L Hossain MPH), International Centre for Diarrhoeal Disease Research, Bangladesh, Dhaka, Bangladesh; Department of Women's and Children's Health (A Ahmed MS), Department of Medical Sciences (D Lindholm MD), Uppsala University, Uppsala, Sweden; Department of Medicine (M Ahmed MD), Rawalpindi Medical University, Rawalpindi, Pakistan; Department of Assistance Medical Sciences (N Ahmed PhD), Faculty of Nursing (W T Almagharbeh PhD), Prince Fahad bin Sultan Chair for Biomedical Research (S Muthupandian PhD), University of Tabuk, Tabuk, Saudi Arabia (S Muthupandian PhD); Department of Psychology (O Ahmed MSc), University of Chittagong, Chattogram, Bangladesh; Department of Veterinary Microbiology (A O Ahmed PhD), Department of Veterinary Pharmacology and Toxicology (A Aremu PhD), Department of Veterinary Public Health and Preventive Medicine (I A Odetokun PhD), University of Ilorin, Ilorin, Nigeria; College of Nursing (M S Ahmed MSc), Majmaah University, Al Majmaah, Saudi Arabia; Brody School of Medicine (S Ahmed PhD), East Carolina University, Greenville, NC, USA; Medical Laboratory Science Department (G S Ahmed MSc, H M Rahim MSc), University of Human Development, Sulaymaniyah, Iraq; Department of Biosciences (H Ahmed PhD), COMSATS Institute of Information Technology, Islamabad, Pakistan; School of Medicine (G Aimagambetova PhD, Y Semenova PhD), Department of Biomedical Sciences (M Aljofan PhD), Department of Medicine (J U Almazan PhD), Nazarbayev University, Astana, Kazakhstan; Clinical Academic Department of Women's Health (G Aimagambetova PhD), University Medical Center, NU Medicine, Astana, Kazakhstan; Department of Orthopedics (Prof J P Aithala DNB), Yenepoya Medical College, Mangalore, India; Faculty of Medicine and Public Health (B Aji DrPH), Jenderal Soedirman University, Purwokerto, Indonesia; Department of Water Engineering (S Akbarifard PhD), Graduate University of Advanced Technology, Kerman, Iran; Oxford Vaccine Group (O Akeju MPH), Nuffield Department of Surgical Sciences (O Almidani MSc, S Bandyopadhyay MPH), Nuffield Department of Orthopaedics, Rheumatology, and Musculoskeletal Sciences (S M Graham PhD), Big Data Institute, Nuffield Department of Population Health (Z Guo PhD), Nuffield Department of Medicine (Prof R J Maude PhD, B Sartorius PhD), Department of Psychiatry (Prof C R J Newton MD), Centre for Global Epilepsy (M Romoli MD), University of Oxford, Oxford, UK; Department of Physiology (R E Akhigbe PhD), Department of Medicine (A O Shitu MBBS), Ladoke Akintola University, Ogbomoso, Nigeria; University Institute of Diet and Nutritional Sciences (M Akhtar PhD, A Khalil PhD), Institute of Molecular Biology and Biotechnology (A Altaf PhD, M Khan PhD, T Maqbool PhD, S Shahid PhD), University College of Medicine & Dentistry (Prof M Arooj PhD), University Institute of Food Science and Technology (S Bashir PhD), University Institute of Radiological Sciences and Medical Imaging Technology (Prof Z Fatima PhD, M Latif PhD), University Institute of Public Health (F Malik PhD), Department of Technology (M Muzaffar MBA), Research Centre for Health Sciences (RCHS) (M Muzaffar MBA, S Shahid PhD, M Umar MBA), Department of Physics (W Shahid PhD), Lahore Business School (M Umar MBA), Faculty of Sciences (Prof A B Waqar PhD), The University of Lahore, Lahore, Pakistan; Department of Internal Medicine (K Akinosoglou PhD), Department of Cardiothoracic Surgery (V Leivaditis PhD), University of Patras, Patras, Greece; Department of Internal Medicine and Infectious Diseases (K Akinosoglou PhD), University General Hospital of Patras, Patras, Greece; Biomedical Engineering Department (Y M Akiska BS), School of Dentistry (M Chen DDS), University of Michigan, Ann Arbor, MI, USA; School of Medicine (Y M Akiska BS), Milken Institute of Public Health (B K Bekele MPH), Department of Global Health (R S Bernstein MD), School of Engineering and Applied Science (A Gerami Matin PhD), George Washington University, Washington, DC, USA; Department of Cardiology, Heart, Vascular, and Thoracic Institute (M Akkaif PhD),

Fudan University, Shanghai, China; Faculty of Health and Behavioural Sciences (W Akosile PhD), Centre for the Business and Economics of Health (I Koomson PhD), The University of Queensland, Brisbane, Queensland (QLD), Australia; Department of Infection Prevention & Control (H Akram MD), Baylor Scott & White Health, Frisco, TX, USA; VentrureBlick, (H Akram MD); Chicago College of Osteopathic Medicine (A E Akrami BS), Midwestern University, Downers Grove, IL, USA; Feinberg School of Medicine (A E Akrami BS, D B Srivastava BA), Department of Microbiology and Immunology (O Ebohon MPH), Medical Scientist Training Program (S Marzouk MA), Department of Radiology (A Shafieioun MD), Department of Preventive Medicine (M Teramoto MD), Northwestern University, Chicago, IL, USA (M D Szeto MS); Department of Surgery (S Al Hasan PhD, S Azadnajafabad MD), Department of Research and Development (Z Al-Aly MD), Department of Psychiatry (Z Li BA), Washington University in St. Louis, St. Louis, MO, USA; The University of Jordan (M K Al Nawayseh PhD), Jordanian Public Health Society, Amman, Jordan; American University in the Emirates (M K Al Nawayseh PhD), Dubai, United Arab Emirates; Fundamentals and Administration Department (Prof O Al Omari PhD), Department of Adult Health and Critical Care (O A M Al Zaabi PhD), Department of Geography (W Ali PhD), Sultan Qaboos University, Muscat, Oman; Al Al-Bayt University, Mafraq, Jordan (Prof M Al Qadire PhD); Jordan Medical Association, Amman, Jordan (Z Al Ta'ani MD); Faculty of Pharmacy (Y Al Thaher PhD), Faculty of Nursing (M M W Atout PhD), Philadelphia University, Amman, Jordan; School of Pharmacy (Y Al Thaher PhD), Division of Population Medicine (A Sha'aban PhD), Cardiff University, Cardiff, UK; School of Public Health (M A M Al Zoubi PhD, H Theyra-Enias MD), Management Policy and Community Health (J A Atta MPH), University of Texas, Houston, TX, USA; Department of Biology (T A Alalwan PhD), College of Health and Sport Sciences (A G Vaithinathan MSc), University of Bahrain, Zallaq, Bahrain; Clinical Epidemiology Center (Z Al-Aly MD), US Department of Veterans Affairs (VA), St. Louis, MO, USA; Murdoch Business School (K Alam PhD), Murdoch University, Perth, WA, Australia; Department of Oral and Maxillofacial Surgery (M Alam MSc), Neurology Department (S Tabatabaei MD), Shahid Beheshti University of Medical Sciences, Tehran, Iran; Department of Bioengineering (M Alam PhD), Department of Nutrition and Food Studies (S Tyrovolas PhD), George Mason University, Fairfax, VA, USA; School of Nursing (R M Al-Amer PhD), Department of Basic Sciences (Z Altaany PhD), Department of Basic Medical Sciences (R A Karasneh PhD, Prof M M Khatatbeh PhD), Faculty of Nursing (H Khatatbeh PhD), Yarmouk University, Irbid, Jordan; School of Nursing and Midwifery (R M Al-Amer PhD), Western Sydney University, Sydney, NSW, Australia; Department of Nursing and Midwifery (A Alamrew MSc), Woldia University, Woldia, Ethiopia; Department of Health Information Management and Technology (Prof T M Alanzi PhD), Deanship of Preparatory Year and Supporting Studies (Prof S El-Ashker PhD), Imam Abdulrahman Bin Faisal University, Dammam, Saudi Arabia; Department of Clinical Pharmacy (F Y Al-Ashwal PhD), Al-Ayen Iraqi University, Thi-Qar, Iraq; Department of Clinical Pharmacy and Pharmacy Practice (F Y Al-Ashwal PhD), University of Science and Technology, Sana'a, Yemen; Department of Community and Mental Health (Prof M Albashtawy PhD), Al al-Bayt University, Mafraq, Jordan; Division of Pediatric Cardiology (K A Aldawsari MD), University of Colorado, Aurora, CO, USA; Heart Center (K A Aldawsari MD), Liver, Digestive, and Lifestyle Health Research Section (S A Alqahtani MD), Biostatics, Epidemiology, and Science Computing Department (S Yezli PhD), King Faisal Specialist Hospital & Research Center, Riyadh, Saudi Arabia; General Directorate of Research and Studies (M S Aldossary MClinDent), Ministry of Health, Riyadh, Saudi Arabia; Institute of Health Informatics (R W Aldridge PhD), Department of Health Informatics (S Chung PhD), Department of Brain Sciences (Prof M Kivimäki PhD), Division of Medicine (T Oyelade PhD), Department of Population Health Sciences (D Sunkersing PhD), Center for Clinical Microbiology (Prof A Zumla PhD), University College London, London, UK; School of Pharmacy (S M

Aleidi PhD, Prof Y Bustanji PhD), Department of Diagnostic Radiology and Nuclear Medicine (Prof A Al-Ibraheem MD), The University of Jordan (Prof L A Dardas PhD), Department of Mathematics (Prof S Momani PhD), Department of Pathology, Microbiology and Forensic Medicine (M Sallam PhD), Department of Clinical Laboratories and Forensic Medicine (M Sallam PhD), Department of Movement Sciences and Sports Training (K Trabelsi PhD), The University of Jordan, Amman, Jordan; Pediatric Intensive Care Unit (A Al-Eyadhy MD, Prof M Temsah MD), Department of Medicine (Prof A S BaHammam MD), Section of Adult Hematology (Prof G M T ElGohary MD), Department of Physiology (Prof S A Meo PhD), Research Chair for Evidence-Based Health Care and Knowledge Translation (Prof M Temsah MD), King Saud University, Riyadh, Saudi Arabia; Department of Epidemiology and Biostatistics (A M Alfalki MPH), University of South Carolina, Columbia SC, SC, USA; Department of Bacteriology, Immunology, and Mycology (Prof A M Algammal PhD), Faculty of Veterinary Medicine (M Mabrok PhD), Suez Canal University, Ismailia, Egypt; College of Nursing (Prof F N Alhalaiqa PhD), College of Dental Medicine (Prof K Ali PhD), Department of Population Medicine (Prof G Babu PhD), Department of Rehabilitation Sciences (S F Kanaan PhD), College of Medicine (Prof Y Kinfu PhD, Prof M A Yassin MD), Biomedical Research Center, QU Health (M Mohammed PhD), Social and Economic Survey Research Institute (SESRI) (Prof A Perianayagam PhD), Qatar University, Doha, Qatar; Department of Health Services and Hospital Administration (M K Al-Hanawi PhD), Health Economics Research Group (M K Al-Hanawi PhD), Department of Respiratory Therapy (M A Althobiani PhD), Respiratory Therapy Unit (M A Althobiani PhD), Pediatric Dentistry Department (K K Baghlaf PhD), Department of Family and Community Medicine (Prof N S Butt PhD), Department of Physical Therapy (F Khan PhD), Rabigh Faculty of Medicine (Prof A Malik PhD), Department of Dental Public Health (Z S Natto DrPH), Department of Community Medicine (S Samargandy PhD), King Abdulaziz University, Jeddah, Saudi Arabia; Faculty of Applied Health Sciences (Physiotherapy) (A A Alhassan Ibrahim PhD), Department of Physiotherapy (S K Sulaiman PhD), Tishk International University, Erbil, Iraq; Faculty of Dentistry (A Alhumaidi DDS), Ibn Al-Nafis University for Medical Sciences, Sana'a, Yemen; Centre for Biotechnology and Microbiology (S Ali PhD), University of Swat, Charbagh, Pakistan; Department of Medical Rehabilitation (Physiotherapy) (M U Ali PhD), Department of Microbiology (M A Isa PhD), University of Maiduguri, Maiduguri, Nigeria; Department of Rehabilitation Sciences (M U Ali PhD, S Anwer PhD, K Cheung MSc, W P Kwong PhD, J S Usman PhD), Department of Biomedical Engineering (A Jor MSc), School of Nursing (S Tyrovolas PhD), Hong Kong Polytechnic University, Hong Kong, China; Faculty of Health (Prof K Ali PhD), Plymouth University, Plymouth, UK; Department of Pharmacy (M Ali PhD), Mohammed Al-Mana College for Medical Sciences, Dammam, Saudi Arabia; Department of Statistics and Operations Research (I Ali PhD), Aligarh Muslim University, Aligarh, India; Center for Biotechnology and Microbiology (S S Ali PhD, M Suleman PhD), University of Swat, Swat, Pakistan; Department of Biotechnology (H M Ali MS), University of Malakand, Chakdara, Pakistan; Department of Biotechnology and Genetic Engineering (A Ali PhD, M Waqas PhD), Hazara University Mansehra, Mansehra, Pakistan; Department of Biosciences (R Ali PhD), Centre for Interdisciplinary Research in Basic Sciences (CIRBSc) (S Anwar PhD), Centre for Interdisciplinary Research in Basic Sciences (S Khan MSc), Centre For Interdisciplinary Research In Basic Sciences (CIRBSc) (A Shamsi PhD), Jamia Millia Islamia, New Delhi, India; School of Food and Agricultural Sciences (M Ali PhD, N Khalid PhD), Department of Life Sciences (Prof M Umair PhD), University of Management and Technology, Lahore, Pakistan; Biomedical Engineering Department (S Ali MS), Bloomberg School of Public Health (F B Ayalew MD), School of Public Health (A A Berihun MA), Department of Anesthesia and Critical Care Medicine (S Boppana MD), Department of Biostatistics (A Columbus MS), institute of radiology and radiological sciences (A Hashem Zadeh MD), Russell H. Morgan

Department of Radiology and Radiological Science (A Kamireddy MD), Department of Neurosurgery (F Kazemi MD), School of Nursing (J Li PhD), Department of Health Policy and Management (D Vervoort MD), Department of Psychiatry (E M Zeru MPH), Department of International Health (H Zhang PhD), Johns Hopkins University, Baltimore, MD, USA; Department of Nuclear Medicine (Prof A Al-Ibraheem MD), King Hussein Cancer Center, Amman, Jordan; Department of Pathophysiology and Transplantation (G Alicandro PhD), Università degli Studi di Milano (University of Milan), Milan, Italy; Cystic Fibrosis Center (G Alicandro PhD), Fondazione IRCCS Ospedale Maggiore Policlinico (IRCCS "Ca' Granda Maggiore Policlinico" Hospital Foundation), Milan, Italy; The School of Medicine (M Al-Iede MD), The University of Jordan, Amman, New South Wales (NSW), Jordan; Institute of Health and Wellbeing (S M Alif PhD), Federation University Australia, Melbourne, VIC, Australia; School of Public Health and Preventive Medicine (S M Alif PhD, Prof M Asghari-Jafarabadi PhD, P Maharjan MPH), Department of Infectious Diseases (M J Loftus MBBS), Department of General Practice (S Melwani PhD), School of Primary and Allied Health Care (F Sousa PhD), Monash University, Melbourne, VIC, Australia; Department of Clinical and Community Pharmacy (Prof S W Al-Jabi PhD, Prof S H Zyoud PhD), Department of Pharmacy (F Amer PhD), Department of Physiology, Pharmacology, and Toxicology (Prof R Shawahna PhD), Department of Chemistry (Prof A H Zyoud PhD), An-Najah National University, Nablus, Palestine; Family and Community Medicine Department (M S Aljohani MD), Qassim University, Al Qassim, Saudi Arabia; School of Physics, Mathematics and Computing (Prof A Al-Jumaily PhD), Cardiovascular Epidemiology Research Centre (CERC) (D Zemedikun PhD), University of Western Australia, Perth, WA, Australia; Information and Communication Technology Research Pole (Lab-STICC) (Prof A Al-Jumaily PhD), ENSTA Bretagne, Brest, France; Department of Public Health and Community Medicine (Prof S M Aljunid PhD, Prof C T Sreeramareddy MD), International Medical University, Kuala Lumpur, Malaysia; International Centre for Casemix and Clinical Coding (Prof S M Aljunid PhD), National University of Malaysia, Bandar Tun Razak, Malaysia; College of Life Sciences (Prof A Alkhatib PhD, C C T Clark PhD, D Islam PhD), Birmingham City University, Birmingham, UK; Cardiovascular Divivsion (M Alkhawam MD), Department of Psychology (D C Schwebel PhD), University of Alabama, Birmingham, AL, USA; Tabriz University of Medical Sciences (A Allahbakhshian PhD), Department of Statistics and Epidemiology (A Jafari-Khounigh PhD), Social Determinants of Health Research Center (S Karimi PhD, Prof S Mohammad-Alizadeh-Charandabi PhD), Faculty of Nursing and Midwifery (Prof M Mirghafourvand PhD), Midwifery Department (Prof S Mohammad-Alizadeh-Charandabi PhD), Neurosciences Research Center (NSRC) (R Mosaddeghi Heris MD), Student Research Committee (R Mosaddeghi Heris MD), Iranian Research Center for Evidence-based Medicine (F Sadeghi-Ghyassi PhD), Drug Applied Research Center (H Samadi Kafil PhD), Tabriz University of Medical Sciences, Tabriz, Iran; Faculty of Medicine (Prof M Z Allouh PhD, Prof M S I Alyahya PhD), Department of Allied Medical Sciences (A Alrawashdeh PhD), Department of Rehabilitation Sciences (M Al-Wardat PhD), Department of Clinical Pharmacy (Prof K H Alzoubi PhD), Department of Public Health (Prof K A Kheirallah PhD), Jordan University of Science and Technology, Irbid, Jordan; Department of Cardiology, Heart, Vascular, and Thoracic Institute (Prof W Almahmeed MD), Department of Urology (O Almidani MSc), Research Department (N Dababo MD), Department of Cardiac Surgery (Prof L Göbölös PhD), Cleveland Clinic Abu Dhabi, Abu Dhabi, United Arab Emirates; College of Medicine and Health Sciences Academic Programs (Prof W Almahmeed MD), Department of Public Health and Epidemiology (Prof B A Saddik PhD), Khalifa University, Abu Dhabi, United Arab Emirates; BRAC Institute of Governance and Development (BIGD) (M Al-Mamun MS), BRAC James P Grant School of Public Health (S Bente Kamal Tune MPH), Centre for Noncommunicable Diseases and Nutrition (R Gupta MPH), School of Pharmacy (M Islam PhD), BRAC University, Dhaka, Bangladesh; Department of Public and Community

Health (M Al-Mamun MS), Frontier University Garowe (FUG), Puntland, Somalia; Independent Consultant, Amman, Jordan (S Al-Marwani MSc); Department of Parasitology (Prof H M Al-Mekhlafi PhD), University of Malaya, Kuala Lumpur, Malaysia; Department of Parasitology (Prof H M Al-Mekhlafi PhD), Sana'a University, Sana'a, Yemen; Ophthalmology Department (A Almobayed MD), Neurology Department (F Mahmoudi MD), Department of Ophthalmology (S Rouzbahani MD), University of Miami, Miami, FL, USA; university of tabuk-nursing faculty (K A Alnawafleh PhD), King Saud bin Abdulaziz University for Health Sciences, tabuk, Saudi Arabia; School of Public Health (M Alocious Sukumar MPH), Institute of Science and Technology, CHENNAI, India; Faculty of Nursing (M R Alosta PhD), Department of Nursing (A H Khalifeh PhD), Zarqa University, Zarqa, Jordan; Division of Gastroenterology and Hepatology (S A Alqahtani MD), Weill Cornell Medicine, New York, NY, USA; Department of Respiratory Care (J S Alqahtani PhD), Prince Sultan Military College of Health Sciences, Dammam, Saudi Arabia; American University of the Middle East, Egaila, Kuwait (M R Alqudimat PhD); Department of Nursing (I Alrimawi PhD), Department of Medicine (M Hemmati MD, C J Sabet MA), Georgetown University, Washington, DC, USA; Macro-Fiscal Policy Department (S M Alrousan PhD), Ministry of Finance, Dubai, United Arab Emirates; Department of Surgery (S K Al-Sabah MD), Kuwait University, Kuwait, Kuwait; Jaber Al Ahmad Al Sabah Hospital (S K Al-Sabah MD), Ministry of Health, Kuwait, Kuwait; Department of Emergency Medicine (M A Alsabri MD), Sana'a University, Sanaa, Yemen; Pediatric Emergency Medicine Department (M A Alsabri MD), Drexel Dornsife School of Public Health (E Ezenwankwo MPH), School of Biomedical Engineering, Science and Health Systems (M Noroozi BSc), Drexel University, Philadelphia, PA, USA; Department of Family and Community Medicine (N Z Alshahrani MD), University of Jeddah, Jeddah, Saudi Arabia; Faculty of Health Sciences (A Altaf PhD), Equator University of Science and Technology, Uganda, Masaka, Uganda; Research, Policy, and Training Directorate (A B Al-Tammemi MPH), Jordan Center for Disease Control, Amman, Jordan; Department of Specialty Internal Medicine (Prof J A Al-Tawfiq MD), Johns Hopkins Aramco Healthcare, Dhahran, Saudi Arabia; Department of Medicine (Prof J A Al-Tawfiq MD), Indiana University School of Medicine, Indianapolis, IN, USA; Faculty of Health Sciences (J Alvarez-Galvez PhD), University of Cadiz, Cadiz, Spain; Research Group in Health Economics (Prof N Alvis-Guzman PhD), Universidad de Cartagena (University of Cartagena), Cartagena, Colombia; Research Group in Hospital Management and Health Policies (Prof N Alvis-Guzman PhD), Universidad de la Costa (University of the Coast), Barranquilla, Colombia; Department of Medical Sciences (Prof Y M Al-Worafi PhD), Azal University for Human Development, Sana'a, Yemen; Department of Clinical Sciences (Prof Y M Al-Worafi PhD), University of Science and Technology of Fujairah, Fujairah, United Arab Emirates; Department of Pediatrics (Prof H Aly MD, A E’mar MD), Lerner College of Medicine (M Balkis MD), Lerner Research Institute (Prof X Liu PhD), Department of Internal Medicine (A Mushtaq MD), Department of Cardiovascular Medicine (J Rajendran MD), Cleveland Clinic, Cleveland, OH, USA; Evaluation Unit (U A Amaechi BDS), Global Alliance for Vaccines and Immunisations, Geneva, Switzerland; Global Health Advocacy Incubator (GHAI) (J Amafah MPH), University of Central Nicaragua, Washington, DC, USA; London School of Hygiene and Tropical Medicine (E J Amafah MSc), University of London, London, UK; Food and Beverages Safety Research Center (M Aman Mohammadi PhD), School of Medicine (A Haghtalab MD, S Pourasghary MD, S Soraneh MD), Department of Public Health (M Maheri PhD), Urmia University of Medical Sciences, Urmia, Iran; Student Research Committee (B Amidi MD), Department of Virology (Z Heydarifard PhD), Lorestan University of Medical Sciences, Khorramabad, Iran; Health Policy Research Center (B Amidi MD, R Khademi MD, Y Sarikhani PhD), Department of Nursing (A Azargoonjahromi BSc), Health Information Management (A Bashiri PhD), Health Human Resources Research Center (M Bayati PhD), Department of Pathology (S Faraji PhD), Student Research

Committee (A Faramarzi MD), Department of Otolaryngology (A Faramarzi MD), cardiovascular department (M feili MD), Research Center for Traditional Medicine and History of Medicine (Prof M Hashempur PhD), Shiraz Neuroscience Research Center (M Jafarinia PhD), Non-communicable Disease Research Center (Prof R Malekzadeh MD), Department of Medical Mycology and Parasitology (Prof K Pakshir PhD), Department of Occupational Health and Safety Engineering (R Pourbabaki PhD), Department of Epidemiology and Biostatistics (H Raeisi Shahraki PhD), Department of Health Services Management (Prof R Ravangard PhD), Department of Biostatistics (E Sadeghi PhD), Shiraz University of Medical Sciences, Shiraz, Iran; Public Health and Community Medicine Department (Prof T T Amin MD), Department of Clinical and Chemical Pathology (Prof M A Elmonem PhD), Medical Microbiology and Immunology Department (H L Mowafy MD), Cairo University, Cairo, Egypt; Department of Radiology and Radiological Science (A Amindarolzarbi MD), University of Maryland, Baltimore, MD, USA; Department of Health and Management Sciences (S Amini PhD), Khomein University of Medical Sciences, Khomein, Iran; Gastrointestinal and Liver Diseases Research Center (E Amini-Salehi MD), Gastrointestinal and Liver Disease Research Center (B Eftekhari MD), Department of Social Medicine and Epidemiology (A Feizkhah MD), Department of Environmental Health Engineering (J Jaafari PhD), Department of Medicine (A Khalili MD), Medical Biotechnology Research Center (M YektaKooshali PhD), Guilan University of Medical Sciences, Rasht, Iran; School of Pharmacy (N Aminu PhD), Department of Internal Medicine (G M Rwegerera MD), University of Botswana, Gaborone, Botswana; Spiritual Health Research Center (S Amiri PhD), Nephrology and Urology Research Center (K Hushmandi PhD), Baqiyatallah University of Medical Sciences, Tehran, Iran; Department of Health and Wellbeing (D A Amugsi PhD), African Population and Health Research Center, Nairobi, Kenya; Department of Medicine (G A Amusa MD), Department of Chemical Pathology (L C Imoh MPH), Department of Pediatrics (A O D Ofakunrin MD), University of Jos, Jos, Nigeria; Department of Internal Medicine (G A Amusa MD), Department of Chemical Pathology (L C Imoh MPH), Department of Pediatrics (A O D Ofakunrin MD), Jos University Teaching Hospital, Jos, Nigeria; Center for Biomedical Image Computing & Analytics (F Anagnostakis MD), Penn Medicine (S K Khokhar PhD), Population Studies Center (W Li PhD), Perelman School of Medicine (K Ma DDS), Department of Biostatistics, Epidemiology, and Informatics (J Puvvula PhD), University of Pennsylvania, Philadelphia, PA, USA; Dipartimento di Scienze Mediche e Chirurgiche (M Bergami PhD), Department of Medical and Surgical Sciences (Prof R Bugiardini MD, M Sassano MD, Prof F S Violante MD), Department of Biomedical and Neuromotor Sciences (S Guicciardi MD), Department of Medicine and Surgery (I Papadimopoulos MD), University of Bologna, Bologna, Italy (F Anagnostakis MD); Department of General Medicine (R A Ananda MD), Eastern Health, Box Hill, VIC, Australia; Faculty of Pharmacy (Prof R Ancuceanu PhD), Department of Legal Medicine and Bioethics (Prof S Hostiuc PhD), Department of Internal Medicine (M Hostiuc PhD), Department of Dermatology (C N Matei PhD, M Tampa PhD), Department of Anatomy and Embryology (R I Negoi PhD), Department of General Surgery (I Negoi PhD), Department of Diabetes, Nutrition and Metabolic Diseases (Prof A Pantea Stoian PhD), Carol Davila University of Medicine and Pharmacy, Bucharest, Romania; Centre for Sensorimotor Performance (D Anderlini MD), Department of Urology (Prof E Chung MD), School of Public Health (M Islam MSc, J C Maravilla PhD), School of Dentistry (R Lalloo PhD), Centre for Clinical Research, Faculty of Health, Medicine and Behavioural Sciences (Prof C L Lau PhD), Queensland Brain Institute (Prof J J McGrath MD), Faculty of Medicine (B Sartorius PhD), The University of Queensland, Brisbane, QLD, Australia (M Moni PhD); Neurology Department (D Anderlini MD), Royal Brisbane and Women's Hospital, Brisbane, QLD, Australia; Faculty of Medicine and Health (D B Anderson PhD, M M Kamal MPH), Sydney Musculoskeletal Health (D B Anderson PhD, S Mathieson PhD), School of Architecture, Design, and

Planning (Prof T Astell-Burt PhD), Charles Perkins Centre (R Biswas PhD), School of Health Science (A Carvalho-e-Silva PhD), School of Public Health (Prof T R Driscoll PhD), University of Sydney (S Mathieson PhD), Westmead Applied Research Center (E T O'Hagan PhD), School of Veterinary Science (B B Singh PhD), Menzies Centre for Health Policy (F Sitas PhD), University of Sydney, Sydney, NSW, Australia (S R Okeke PhD); Department of Statistics and Econometrics (Prof T Andrei PhD, Prof M Ausloos PhD, Prof C Herteliu PhD, A Otoiu PhD), Management Department (Prof I Popa PhD), Bucharest University of Economic Studies, Bucharest, Romania; Department of Internal Medicine (S Ang MD), Rutgers University, Toms River, NJ, USA; Sarver Heart Center (S Ang MD), College of Medicine (M Asadi Anar MD), Department of Internal Medicine (H Pham MD), University of Arizona, Tucson, AZ, USA; Department of General Medicine (N Anh MD), Thai Binh University of Medicine and Pharmacy in Vietnam, Thai Binh City, Viet Nam; Department of Management (S E Ankomah PhD, S E Ankomah PhD), Department of Population and Health (J K Oduro PhD), Department of Health, Physical Education and Recreation (J O Sarfo PhD), University of Cape Coast, Cape Coast, Ghana; Department of Public Health (K Annadurai PhD), The Apollo University, Chittoor, India; Department of Physiotherapy (S Ansari PhD, M Sidiq PhD), Galgotias Multidisciplinary Research & Development Cell (M Sidiq PhD), Galgotias University, Greater Noida, India; Pharmacy Department, Critical Care (U Ansari PharmD), Cleveland Clinic Abu Dhabi, Abu Dhabi, United Arab Emirates; Department of Epidemiology and Biostatistics (Prof A Ansari-Moghaddam PhD), Health Promotion Research Center (J Nejati PhD, H Okati-Aliabad PhD), Zahedan University of Medical Sciences, Zahedan, Iran; Agribusiness Study Program (E Antriyandarti DrAgrSc), Sebelas Maret University, Surakarta, Indonesia; School of Chemical and Life Sciences (SCLS) (S Anwar PhD), Jamia Hamdard, New Delhi, India; Department of Surgery (S Anwar PhD), Department of Pharmacology (I Fitriana PhD), Department of Medical Surgical Nursing (A L Wicaksana MS), Gadjah Mada University, Yogyakarta, Indonesia; Department of Pathology (R Anwer PhD), Imam Mohammad Ibn Saud Islamic University, Riyadh, Saudi Arabia; Rural Health Research Institute (A E Anyasodor PhD, Prof J Sun PhD), Charles Sturt University, Orange, NSW, Australia; School of Medicine and Public Health (G C Apostol MD), Ateneo Center for Research and Innovation (A C Dy MD), Center for Research and Innovation (V F Pepito MSc), Ateneo De Manila University, Pasig City, Philippines; Inter-Agency Committee on Environmental Health (G C Apostol MD), Department of Health Philippines, Manila, Philippines; Division of Gastroenterology, Hepatology, and Nutrition (J Arab MD), Virginia Commonwealth University, Richmond, VA, USA; Gastroenterology Department (J Arab MD), Department of Gastroenterology (L Diaz MD), Pontifical Catholic University of Chile, Santiago, Chile; Geneva University Hospital (H Arabi PhD), University of Geneva, Geneva, Switzerland; College of Pharmacy (M Arafat PhD), AAU Health and Biomedical Research Center (Prof F El-Dahiyat PhD), Al Ain University, Abu Dhabi, United Arab Emirates; College of Art and Science (D Areda PhD), Ottawa University, Surprise, AZ, USA; School of Life Sciences (D Areda PhD), Arizona State University, Tempe, AZ, USA; Care in Long Term Conditions Research Division (J Arias de la Torre PhD), School of Life Course and Population Sciences (Prof Y Wang PhD), King's College London, London, UK; CIBER Epidemiology and Public Health (CIBERESP), Madrid, Spain (J Arias de la Torre PhD); Department of Cardiovascular, Endocrine-Metabolic Diseases and Aging (B Armocida MD), Istituto Superiore di Sanità (ISS), Rome, Italy; Department of Neurobiology, Care Sciences and Society (Prof J Ärnlöv PhD), Karolinska Institutet, Stockholm, Sweden; School of Health and Social Studies (Prof J Ärnlöv PhD), Dalarna University, Falun, Sweden; Department of Biotechnology (Prof J Arockiaraj PhD), Sri Ramaswamy Memorial Institute of Science and Technology, Kattankulathur, India; Institute for Biomedical Problems (A A Artamonov PhD), K.A. Timiryazev Institute of Plant Physiology (M V Titova PhD), Russian Academy of Sciences, Moscow, Russia; Department of

Periodontics (D Arumuganainar PhD), Department of Biosciences (S Chopra MPH), Department of Physiology (E Dilipan PhD), Department of Prosthodontics (D Ganapathy PhD), Department of Oral Medicine and Periodontology (Prof R D Jayasinghe MS), Saveetha Medical College and Hospital (Prof M Karobari PhD), Saveetha Dental College and Hospitals (G Minervini PhD, M Tovani-Palone PhD), Department of Biochemistry (P Royapuram Parthasarathy PhD), Center for Global Health Research (Prof A Sahebkar PhD), Department of Microbiology (S Sankar PhD), Saveetha University, Chennai, India; Department of Public Health (N Aryntayeva MSPH, A Kuttybayev MSc), Atchabar Scientific Research Institute (B Assembekov PhD), Atchabarov Scientific-Research Institute of Fundamental and Applied Medicine (D Davletov MD, A Zhumagaliuly MD), Population Health Research Center (Prof K Davletov PhD), Director of the Scientific and Technological Park (I R Fakhradiyev PhD), Laboratory of Experimental Medicine (T Fazylov MD), Science and Technology Park (A Ibrayeva PhD), Department of Urology (Y Ismoldayev PhD), Department of General Medical Practice No. 2 (Prof S Kamenova DMedSc), Scientific and Educational Center for Neurology and Applied Neuroscience (A Kondybayeva PhD), Research and Publication Activity Division (M Kulimbet MSc), Department of Research (B Lakanova MD), Scientific laboratory "Center for Collective Use" (A S Oradova PhD), Science Department (A Shamsutdinova MD), Department of Medicine (S Tanabayeva PhD), Kazakh National Medical University, Almaty, Kazakhstan; Department of Clinical Disciplines (N Aryntayeva MSPH), Department of Clinical Subjects (A Kurmanova MD), Al Farabi Kazakh National University, Almaty, Kazakhstan; Basic Health Sciences Institute (M Asadi-Samani PhD), Community-Oriented Nursing Midwifery Research Center (M Heidari PhD), Department of Community Health (M Lotfizadeh PhD), Social Determinants of Health Research Center (M Lotfizadeh PhD), Modeling in Health Research Center (A Mohammadian-Hafshejani PhD), Shahrekord University of Medical Sciences, Shahrekord, Iran; Department of Pharmacy Practice (Prof S Asdaq PhD), College of Medicine (M Fareed PhD), AlMaarefa University, Riyadh, Saudi Arabia; National Agency for Strategic Research in Medical Education (NASRME) (Prof S Asgary MSc), National Agency for Strategic Research in Medical Sciences Education (M Heydari PhD), Halal Research Center of IRI (Prof A Mousavi Khaneghah PhD), Ministry of Health and Medical Education, Tehran, Iran; Cabrini Research (Prof M Asghari-Jafarabadi PhD), Cabrini Health, Malvern, VIC, Australia; Pioneer Journal of Biostatistics and Medical Research (PJBMR), Pakistan, Pakistan (T Ashraf PhD); Department of Physiology (M A B Ashraf PhD), Faisalabad Medical University, Faisalabad, Pakistan; Department of Radiation Oncology (M Ashrafizadeh DVM), Shandong University, Shandong, China; Deakin Health Economics/School of Health and Social Development (B K Y Asiamah-Asare PhD), Deakin University, Melbourne, Victoria (VIC), Australia; School of Traditional Chinese Medicine (M Aslam PhD, Y Kim PhD), Xiamen University Malaysia, Sepang, Malaysia; Faculty of Medicine, Nursing, and Health Sciences (S Aslani PhD), Monash Addiction Research Center (D Z Assefa MSc), Monash University, Melbourne, Victoria (VIC), Australia; Nursing Department (Y Asri PhD), Faculty of Health Science (Y Asri PhD), Institute of Technology and Health Science RS dr Soepraoen, Malang, Indonesia; Department of Medical-Surgical Nursing (A Assariparambil PhD), Manipal College of Health Professions (V Jaganathan PhD, K Josten MSc), Kasturba Medical College, Manipal (S Koulmane Laxminarayana MD), Manipal Academy of Higher Education, Udupi, India; Keck School of Medicine (M Athari MD), Department of Radiology (M Fotouhi MD), University of Southern California, Los Angeles, CA, USA; Department of Forensic Medicine (A Atreya MD), Department of Community Medicine (S Nepal MD), Lumbini Medical College, Palpa, Nepal; College of Medicine (Prof Z A Atwan PhD), University of Basrah, Basrah, Iraq; School of Business (Prof M Ausloos PhD), Department of Health Sciences (S J Tromans PhD), Diabetes Research Centre (E Vounzoulaki PhD), University of Leicester, Leicester, UK; Robarts Research Institute (A Avan MD), The University of Western Ontario, London, ON,

Canada; Departament of Physiotherapy (N C P Avelar DSc), Federal University of Santa Catarina, Araranguá, Brazil; IMBB (S J Awan PhD), The University of Lahore, lahore, Pakistan; School of Veterinary Medicine (Prof B B Awosile PhD), Texas Tech University, Amarillo, TX, USA; School of Nursing and Public Health (A W Awotidebe PhD), University of KwaZulu-Natal, Durban, South Africa; The Judith Lumley Centre (B Ayala Quintanilla PhD), School of Nursing and Midwifery (Prof D Edvardsson PhD, Prof M Rahman PhD), La Trobe University, Melbourne, VIC, Australia; Universidad de San Martin de Porres, Lima, Peru (B Ayala Quintanilla PhD); Department of Public Health (L A A Ayana MPH), Department of Pharmacy (G Fekadu PhD), Institute of Health Sciences (B Feyisa MPH), Wollega University, Nekemte, Ethiopia; Medicinal Chemistry Unit (Y O Ayipo PhD), Kwara State University, Malete, Ilorin, Nigeria; Centre for Drug Research (Y O Ayipo PhD), Universiti Sains Malaysia, Pinang, Malaysia; Department of Public Health (B I Aylı MD), Hacettepe University, ankara, Turkiye; Department of Nephrology (B I Aylı MD), Etlik City Hospital, Ankara, Turkiye; Research and Technology Deputy (A Azadnia PhD), Department of Epidemiology and Biostatistics (Y Moradi PhD), Kurdistan University of Medical Sciences, Sanandaj, Iran; Department of Infectious Disease Epidemiology (J M Azam PhD), Department and Demography and Health (A C Dy MD), Department of Non-Communicable Disease Epidemiology (M Iwagami PhD), Department of Health Services Research and Policy (Prof M McKee DSc), International Centre for Eye Health (A J Thirunavukarasu MA), London School of Hygiene & Tropical Medicine, London, UK; Department of Applied Mathematics (J M Azam PhD), Stellenbosch University, Stellenbosch, South Africa; Consultant, Washington, DC, USA (G S Azhar PhD); Department of Psychiatry (F Azimi MD), University of Social Welfare and Rehabilitation Sciences, tehran, Iran; Department of Anesthesia (S A Aziz PhD), Research Center (N H Mahmood PhD), College of Health Sciences (H H R Najmuldeen PhD), Cihan University, Sulaymaniyah, Iraq; Department of Basic Sciences (S A Aziz PhD), University of Sulaimani, Sulaymaniyah, Iraq (H H R Najmuldeen PhD); ASIDE Healthcare, Lewes, DE, USA (A Y Azzam MD); Faculty of Medicine (A Y Azzam MD), The Orthopaedic Department (A M Makram MD), October 6 University, 6th of October City, Egypt; Geriatric Unit (D Azzolino PhD), Fondazione IRCCS Ca' Granda Ospedale Maggiore Policlinico, Milan, Italy; Department of Medical Biochemical Analysis (S Babadoust PhD, A Ghandili PhD, H I M. Amin PhD), Department of Nutrition and Dietetics (Y Galali ResM, B A Sadee PhD), Department of Business Administrations (Prof D H Kadir PhD), Cihan University, Erbil, Iraq; Department of Physiotherapy (A S Babu PhD, Prof V K PhD, M K Sinha PhD), Department of Pharmacology (S Gangachannaiah MD), Kasturba Medical College, Mangalore (R Holla MD), Prasanna School of Public Health (R Kamath MHA), Department of Community Medicine (S Kini B MD, C R Rao MD), Kasturba Medical College, Manipal (P L C MD), Department of Pharmaceutical Regulatory Affairs and Management (V S Ligade PhD), Department of Nephrology (Prof S Nagaraju DM), Manipal College of Nursing (S Nayak PhD), Manipal Institute of Management (S Nayak PhD), Manipal College of Dental Sciences (Prof R A Radhakrishnan PhD), Kasturba Medical College Manipal (J P Raj DM), Kasturba Medical College Mangalore (M Rao MD, Prof B Unnikrishnan MD), Department of Health Information Management (B Reshmi PhD), Kasturba Medical College (D Upadhya PhD), Manipal Academy of Higher Education, Manipal, India; Department of Forensic Science (A D Badiye PhD, N Kapoor PhD), Government Institute of Forensic Science Nagpur, Nagpur, India; Rashtrasant Tukadoji Maharaj Nagpur University, Nagpur, India (A D Badiye PhD); The Malaria Atlas Project (H S Baggen MPhil, S Hafsia PhD, M A McPhail PhD, S F Rumisha PhD, T L Symons PhD), Child Health Analytics Research Program (Prof P W Gething PhD, F Sanna PhD, D J Weiss PhD), Geospatial Health and Development Team-Child Health Analytics (J Lubinda PhD), Geospatial Health and Development Team (A Saddler PhD), Telethon Kids Institute, Perth, WA, Australia; Dental Material Research Center (S Baghizadeh DDS), Islamic Azad

University, tehran, Iran; Academic Affairs and Prince Naif Health Research Center (Prof A S BaHammam MD), King Khalid University Hospital, Riyadh, Saudi Arabia; College of Optometry (R Bahreini MS), Pacific University, Forest Grove, OR, USA; Department of Community Medicine (Y Bahurupi MD), Department of Physiology (A Patil MD), All India Institute of Medical Sciences, Nagpur, India; Clinical Research Center (Prof R Bai MD), Nanjing Children's Hospital, Nanjing, China; International Medical School (A A Baig PhD), Management and Science University, Alam, Malaysia; Robert N Butler Aging Center (A Balachandran PhD), Columbia University Medical Center, New York, NY, USA; Population Research Centre (A Balachandran PhD), Institute for Social and Economic Change, Bengaluru, India; Chen Senior Medical Center, Tamarac, FL, USA (M Balkis MD); Anahuac Business School (J Balmori-de-la-Miyar PhD), Universidad Anahuac Mexico, Mexico City, Mexico; Department of Epidemiology and Biostatistics (M Balooch Hasankhani PhD), Department of Biostatistics and Epidemiology (P Dehesh PhD), Physiology Research Center (M Rajizadeh PhD), Kerman University of Medical Sciences, Kerman, Iran; Center of Innovation, Technology and Education (CITE) (Prof O Baltatu PhD), Anhembi Morumbi University, São José dos Campos, Brazil; Department of Neurosurgery (S Bandyopadhyay MPH), School of Psychology (Prof S Cortese PhD), Centre for Innovation in Mental Health (M Garcia-Argibay PhD), Department of Surgery (G Verras MSc), University of Southampton, Southampton, UK; Department of Non-communicable Diseases (P C Banik MPhil), Bangladesh University of Health Sciences, Dhaka, Bangladesh; Department of Health Policy (A Barbato MD), Mario Negri Institute for Pharmacological Research, Milano, Italy; Scientific Advisory Board (A Barbato MD), Institut d'Investigació Biomèdica de Girona Dr. Josep Trueta, Girona, Spain; School of Psychology (Prof S L Barker-Collo PhD), University of Auckland, Auckland, New Zealand; Department of Public and Environmental Health (A Barrow MPH), University of The Gambia, Banjul, The Gambia; Department of Epidemiology (A Barrow MPH, D Braithwaite PhD), Department of Health Services Research, Management and Policy (R Wang PhD), Biology & Emerging Pathogens Institute (M H Zahid PhD), University of Florida, Gainesville, FL, USA; Heidelberg Institute of Global Health (HIGH) (S Barteit PhD), Heidelberg University Hospital, Heidelberg, Germany; Alpha Genomics Private Limited, Islamabad, Pakistan (Z Basharat PhD); Department of General Surgery and Medical-Surgical Specialties (Prof G Basile MD, Prof G Isola PhD), Department of Medical and Surgical Sciences and Advanced Technologies "GF Ingrassia" (Prof E D'Amico MD, Prof M Veroux PhD), Department of Biomedical and Biotechnological Sciences (L Falzone PhD), Department of Clinical and Experimental Medicine (Prof C Ledda PhD), University of Catania, Catania, Italy; Department of Community Medicine (P Baskaran MD), Emergency Medicine Department (G Rajendran MD), Sri Manakula Vinayagar Medical College and Hospital, Puducherry, India; Faculty of Nursing (Prof A Batiha PhD), King Khalid University, Mahyil Asir, Saudi Arabia; Department of Medical Education (K Batra PhD), Department of Social and Behavioral Health (Prof M Sharma PhD), University of Nevada Las Vegas, Las Vegas, NV, USA; Department of Psychiatry (Prof B T Baune PhD), University of Münster, Münster, Germany; Department of Psychiatry (Prof B T Baune PhD), Melbourne Medical School, Melbourne, VIC, Australia; Biological Science Division (M Bayat Tork MD), Public Health Sciences (A Jamal BS), Pritzker School of Medicine (Prof H Yao PhD), University of Chicago, Chicago, IL, USA (Prof J Wan PhD); The George Institute for Global Health (T Beaney PhD, Prof S Yaya PhD), Department of Brain Sciences (L D'Anna PhD), WHO Collaborating Centre for Public Health Education and Training (Q Lin MPH, D L Rawaf MD), School of Public Health (A M Makram MD), Department of Surgery and Cancer (Prof E Mossialos PhD), Department of Primary Care and Public Health (R Palladino MD, C Tabche MSc), Imperial College London, London, UK; School of Public Health (Prof N Bedi MD), Dr. D. Y. Patil University, Mumbai, India; Clinical Nutrition Department (R M Chandika PhD), Department of Public Health (S Dohare MD, W

Rehman MS), Epidemiology Program (M Khan MD), Department of Prosthetic dental sciences (Substitutive Dental Sciences) (K A Mattoo MD), Health Education & Promotion, College of Nursing and Health Sciences (M Shanawaz MD), Department of Clinical Practice (A Shoaib PhD), College of Public Health and Tropical Medicine (J Varghese PhD), Jazan University, Jazan, Saudi Arabia (Prof N Bedi MD); Department of Human Anatomy and Histology (Prof N Beeraka PhD), Clinical Medicine (General Medicine profile) (N A Nekliudov MD), Department of Epidemiology and Evidence-Based Medicine (R V Polibin PhD), I.M. Sechenov First Moscow State Medical University, Moscow, Russia; Avicenna Biotech Research, Germantown, MD, USA (B Behnam MD); Department of Regulatory Affairs (B Behnam MD), Amarex Clinical Research, Germantown, MD, USA; Department of Microbiology (P Behzadi PhD), Islamic Azad University, Shahr-e-Qods, Iran; Transplant and Hepatobiliary Surgery Service (D F Bejarano Ramirez MSc), Hospital Universitario Fundación Santa Fe de Bogotá (University Hospital Santa Fe Foundation of Bogotá), Bogota, Colombia; Subdirectorate of Clinical Studies and Clinical Epidemiology (D F Bejarano Ramirez MSc), Hospital Universitorio Fundación Santa Fe de Bogotá, Bogotá, Colombia; Department of Public Health (M Belayneh PhD), Institute for Social and Health Sciences (Prof L Laflamme PhD), University of South Africa, Pretoria, South Africa; Department of Radiology (G Belge Bilgin MD, F Nugen PhD), Physiology and Biomedical engineering (Z Khashim PhD), Department of Cardiovascular Medicine (H Pham MD), Department of Physiology and Biomedical Engineering (F Pourghazi MD), Department of Endocrinology (M Salehi MD), Section of Advanced Heart Failure and Transplant Cardiology (D Uppal MD), Mayo Clinic, Rochester, MN, USA; Bayero University Kano (Prof B Bello PhD), Higher National School of Veterinary Medicine, Kano, Nigeria; North-West University, Mafikeng, South Africa (Prof B Bello PhD); Infectious Disease Research Department (M B Bello PhD), Medical Genomics Research Department (Prof M Umair PhD), King Abdullah International Medical Research Center, Riyadh, Saudi Arabia; Department of Physiotherapy and Paramedicine (U M Bello PhD), Glasgow Caledonian University, Glasgow, UK; Department of Biological Sciences (Prof L Belo PhD), Research Unit on Applied Molecular Biosciences (UCIBIO) (Prof L Belo PhD), Research Centre for Physical Activity, Health, and Leisure (L Bohn PhD), Associated Laboratory for Green Chemistry (LAQV) (M Carvalho PhD, N G M Gomes PhD), Institute for Research and Innovation in Health (i3S) (Prof N Cruz-Martins PhD), UCIBIO Applied Molecular Biosciences Unit (Prof D Dias da Silva PhD), MEDCIDS, Faculty of Medicine of the University of Porto (A Freitas PhD), Faculty of Medicine (J R Rocha-Gomes MD), University of Porto, Porto, Portugal; Department of Biomedical Sciences (Prof A Beloukas PhD), National AIDS Reference Center of Southern Greece (Prof A Beloukas PhD), University of West Attica, Athens, Greece; Department of Epidemiology and Health Promotion (Prof H Benzian PhD), Department of Child and Adolescent Psychiatry (Prof S Cortese PhD), Institute for Excellence in Health Equity (M Kumar PhD), School of Global Public Health (E K Peprah PhD), Rory Meyers College of Nursing (X Qi PhD), New York University, New York, NY, USA; Department of Epidemiology and Biostatistics (A C Bermudez MD), Department of Clinical Epidemiology (C A Panelo MA), University of the Philippines Manila, Manila, Philippines; Department of Epidemiology (A C Bermudez MD), Brown University, Providence, RI, USA; Hubert Department of Global Health (R S Bernstein MD), School of Medicine (A O Fasanmi PhD), Rollins School of Public Health (Prof D A Sleet PhD), Emory University, Atlanta, GA, USA; Department of Community Medicine and Family Medicine (A S Bhadoria MD), All India Institute of Medical Sciences, Rishikesh, India; Community Health Department (A S Bhadoria MD), University of South Wales, South Wales, UK; Department of Public Health (A S Bhagavathula PhD), North Dakota State University, Fargo, ND, USA; Department of General Practice and Emergency Medicine (J Bhandari MD), Karnali Academy of Health Sciences, Jumla, Nepal; Internal Medicine (C Bhanushali MD), Saint Vincent Hospital, Worcester,

Worcester, MA, USA; Department of Community Medicine and Family Medicine (Prof P Bhardwaj MD), School of Public Health (Prof P Bhardwaj MD), Department of Anatomy (N Bhardwaj MD), Department of Pharmacology (M Shamim MBBS), Department of Biochemistry (S Tomo MD), All India Institute of Medical Sciences, Jodhpur, India; Department of Internal Medicine (A Bhargava MD), Wayne State University, Gross Pointe Woods, MI, USA; Global Health Neurology Lab (S Bhaskar MD), NSW Brain Clot Bank, Sydney, NSW, Australia; Division of Cerebrovascular Medicine and Neurology (S Bhaskar MD), National Cerebral and Cardiovascular Center, Suita, Japan; Department of Family Medicine (A Bhatnagar MD), Texas Tech University, el paso, TX, USA; School of Sport & Health Sciences (S Bhattacharjee MPH), University of Brighton, Brighton, UK; Department of Public Health Research (S Bhattacharjee MPH), Bengal Rural Welfare Service (BRWS), Kolkata, West Bengal, India; Translational and Clinical Research Institute (P Bhattacharjee MD), Newcastle University, Newcastle upon Tyne, UK; Department of Botanical and Environmental Sciences (Prof M S S Bhatti PhD), Department of Pharmaceutical Sciences (Prof R Bhatti PhD), Guru Nanak Dev University, Amritsar, India; Department of Medical Lab Technology (Prof G K Bhatti PhD), Centre for Research and Development (Prof S R Pandi-Perumal MSc), Department of University Institute of Biotechnology (R Sharma PhD), Chandigarh University, Punjab, India; Laboratory of Translational Medicine and Nanotherapeutics (Prof J S Bhatti PhD), Department of Microbiology (P K Kushawaha PhD, A Singh PhD, M Yadav PhD), Department of Human Genetics and Molecular Medicine (U Sharma PhD), Department of Biochemistry (B Singh PhD), Department of Computer Science & Engineering (Prof S Singh PhD), Department of Zoology (B Vellingiri PhD), Central University of Punjab, Bathinda, India; Department of Health Administration (S S Bhuyan PhD), Institute for Health, Health Care Policy and Aging Research (S Rege PhD), Rutgers University, New Brunswick, NJ, USA; Independent Consultant, Addis Ababa, Ethiopia (S K Biadgilign PhD); Fondazione Banca Degli Occhi Del Veneto (R Bievel-Radulescu MD), Carol Davila University of Medicine and Pharmacy, Venice, Italy; TAF Uludag Winter Training Center (C Bilgin MD), Turkish Ministry of Defence, Bursa, Turkiye; Clinical Research Centre (R Biswas PhD), Sydney Local Health District, Sydney, NSW, Australia; Department of Biochemistry and Biotechnology (M Biswas PhD), University of Science and Technology Chittagong, Chittagong, Bangladesh; Department of Community Medicine and Family Medicine (B Biswas MD), Department of Physiology (H Mondal MD), Department of Pharmacology (S T Y MD), All India Institute of Medical Sciences, Deoghar, India; Department of Clinical Pharmacy (A Bitar PhD), Universiti Sultan Zainal Abidin, Besut, Malaysia; Health Biotechnology Directorate at Bio and Emerging Technology Institute (M Bitew PhD), School of Public Health (K Deribe PhD), College of Health Sciences (F S Gebre MD, A M Zenebe MSc), Department of Reproductive, Family and Population Health (N A Kassaw MPH), Department of Medical Physiology (H T Wada MSc), Addis Ababa University, Addis Ababa, Ethiopia; Department of Neurobiology, Care Sciences and Society (B Bizzozero-Peroni PhD), Department of Neurobiology, Care Sciences, and Society (S Fereshtehnejad PhD), Clinical Epidemiology Division (KEP) (K R Fowobaje PhD), KI Solna (A Javanmardi MD), Department of Global Public Health (Prof L Laflamme PhD), Karolinska Institute, Stockholm, Sweden; Department of Physical Education and Health (B Bizzozero-Peroni PhD), Universidad de la República, Rivera, Uruguay; School of Business Administration (Prof V Bodolica PhD), American University of Sharjah, Sharjah, United Arab Emirates; Department of Nutrition and Dietetics (M Bodur PhD), Ankara University, Ankara, Turkiye; Faculty of Psychology, Education, and Sport (L Bohn PhD), University Lusofona, Porto, Portugal; Department of Demography and Population Studies (O A Bolarinwa MSc), University of Witwatersrand, Johannesburg, South Africa; Department of Internal Medicine (A Boloor MD), Department of General Medicine (J Jeganathan MD), Department of Community Medicine (N Joseph MD, N Kumar MD, R Thapar MD), Kasturba Medical

College (G A Menezes PhD), Department of Anatomy (B Murlimanju MD), Department of Forensic Medicine and Toxicology (Prof J Padubidri MD, Prof P Rastogi MD), Department of Conservative dentistry and Endodontics (M S Thomas MDS), Manipal Academy of Higher Education, Mangalore, India; Ophthalmology Department (P Bolourinejad MD), Department of Epidemiology and Biostatistics (Prof M Mansourian PhD), Environment Research Center (M Moazeni PhD), Department of Environmental Health Engineering (M Moazeni PhD), Department of Health Services Management (M Mohseni PhD), Department of Obstetrics and Gynecology - Infertility Clinic (M Rezaei MD), Family and Prevention Medicine (R Rouzbahani MD), Department of Medical Physics (K Saber PhD), Isfahan University of Medical Sciences, Isfahan, Iran; General Directorate of Health Information Systems (B Bora Basara PhD), Ministry of Health, Ankara, Turkiye; Pediatric Infectious Diseases and Immunology (A Borzutzky MD), Pontificia Universidad Católica de Chile (Pontifical Catholic University of Chile), Santiago, Chile; Facultad de Salud (Faculty of Health) (Prof A Botero Carvajal PhD), Universidad Santiago de Cali, Cali, Colombia; Department of Medicine (Prof S Bouaoud DrPH), Faculty of Medicine (Prof A Ouyahia PhD), University Ferhat Abbas of Setif, Sétif, Algeria; Department of Epidemiology and Preventive Medicine (Prof S Bouaoud DrPH), University Hospital Saadna Abdenour, Sétif, Algeria; Vision and Eye Research Institute (Prof R R A Bourne FRCOphth, Prof S Pardhan PhD), Anglia Ruskin University, Cambridge, UK; Department of Earth, Environment, and Equity (C Boxe PhD), Howard University, Washington, DC, USA; University of Genoa, Genoa, Italy (N L Bragazzi PhD); Cancer Population Sciences Program (D Braithwaite PhD), University of Florida Health Cancer Center, Gainesville, FL, USA; Institute for Medical Information Processing, Biometry, and Epidemiology (S Breitner DSc), LMU Munich, Neuherberg, Germany; Institute of Epidemiology (S Breitner DSc), Helmholtz Zentrum München (German Research Center for Environmental Health), Neuherberg, Germany; Division of Clinical Epidemiology and Aging Research (Prof H Brenner MD), German Cancer Research Center, Heidelberg, Germany; Center for Neuroscience (G Britton PhD), Institute for Scientific Research and High Technology Services, Panama City, Panama; Gorgas Memorial Institute for Health Studies, Panama City, Panama (G Britton PhD); Department of Injury (J Brown PhD), The George Institute for Global Health, Newtown, NSW, Australia; Faculty of Medicine (J Brown PhD), School of Population Health (A E Peden PhD), University of New South Wales, Kensington, NSW, Australia; Malaria Atlas Project (A J Browne DPhil), Telethon Kids Institute, Nedlands, WA, Australia; College of Health Sciences (L P Bui PhD, T T Pham PhD), College of Health Sciences (CHS) (Prof D Poddighe PhD), VinUniversity, Hanoi, Viet Nam; Research Advancement Consortium in Health, Hanoi, Viet Nam (L P Bui PhD, T T Pham PhD); Department of Radiology (F Busch MD), Department of Public Health and Primary Care (M Dalakoti MPH, Prof P Willeit PhD), University of Cambridge, Cambridge, UK; Department of Health Care Management (Prof R Busse PhD), Technische Universität Berlin, Berlin, Germany; School of Public Health Sciences (Z A Butt PhD), University of Waterloo, Waterloo, ON, Canada; Al Shifa School of Public Health (Z A Butt PhD), Al Shifa Trust Eye Hospital, Rawalpindi, Pakistan; School of Public Health and Administration (L Cahuana-Hurtado PhD), Peruvian University Cayetano Heredia, Lima, Peru; Department of Sociology (Prof T Cai PhD), University of Macau, Macau, China; Faculty of Medicine and Health (R Cairns PhD), Institute for Musculoskeletal Health (M Jamshidi PhD), Central Clinical School, Faculty of Medicine and Health (S Mitra PhD), University of Sydney, Sydney, New South Wales (NSW), Australia; The Children's Hospital at Westmead (R Cairns PhD), New South Wales Poisons Information Centre, Sydney, New South Wales (NSW), Australia; Department of Clinical Pharmacy (Prof D Calina PhD), University of Medicine and Pharmacy of Craiova, Craiova, Romania; Department of Internal and Geriatric Medicine (Prof L A Cámera MD), Hospital Italiano de Buenos Aires (Italian Hospital of Buenos Aires), Buenos Aires, Argentina; Board of Directors

(Prof L A Cámera MD), Argentine Society of Medicine, Buenos Aires, Argentina; Center of Innovation, Technology and Education (CITE) (Prof L A Campos PhD), Anhembi Morumbi University, Sao Jose dos Campos, Brazil; Center for Nutrition and Health Research (I Campos-Nonato PhD), Public Health Intelligence Unit (Prof D Diaz PhD), Center for Health Systems Research (E Serván-Mori PhD), National Institute of Public Health, Cuernavaca, Mexico; Department of Anesthesiology (S Cao MD), Third Xiangya Hospital of Central South University, Changsha, China; Department of Surgery (Y Cao MD), Fuwai Hospital (S Zhao MD), Chinese Academy of Medical Sciences, Beijing, China; Unit of Hygiene and Public Health (A Capodici MD), Romagna Local Health Authority, Forlì-Cesena, Italy; Interdisciplinary Research Center for Health Science (A Capodici MD), Sant'Anna School of Advanced Studies, Pisa, Italy; Oncological Network, Prevention and Research Institute (G Gorini MD), Institute for Cancer Research, Prevention and Clinical Network, Florence, Italy (G Carreras PhD); Department of Medicine and Surgery (A Carugno PhD), University of Insubria, Varese, Italy; Faculty of Health Sciences (M Carvalho PhD), University Fernando Pessoa, Porto, Portugal; IMPInstitute for Mental and Physical Health and Clinical Translation (IMPACT) (A F Carvalho MD), Deakin University, Geelong, VIC, Australia; Education Center of Australia (A Carvalho-e-Silva PhD), Health Science College, Sydney, NSW, Australia; Department of Psychiatry (Prof J Castaldelli-Maia PhD), University of Sao Paulo, São Paulo, Brazil; Public Health Department (C A Castañeda-Orjuela PhD), Epidemiology and Public Health Evaluation Group (C A Castañeda-Orjuela PhD), Department of Public Health (Prof F P De la Hoz PhD), National University of Colombia, Bogota, Colombia; Division of Country Health Policies and Systems (CPS) (G Castelpietra PhD), World Health Organisation, Italy; Mental Health Flagship (G Castelpietra PhD), World Health Organization (WHO), Copenhagen, Denmark; Institute of Public Goods and Policies (IPP) (F Catalá-López PhD), Spanish National Research Council, Madrid, Spain; Centre for Biomedical Research in Mental Health Network (CIBERSAM) (F Catalá-López PhD), National School of Public Health (A Padron-Monedero PhD), Institute of Health Carlos III, Madrid, Spain; Department of Pharmacological and Biomolecular Sciences (Prof A L Catapano PhD), Department of Clinical Sciences and Community Health (Prof C La Vecchia MD), Department of Food, Environmental and Nutritional Sciences (Prof D Martini PhD), University of Milan, Milan, Italy; MultiMedica Sesto San Giovanni IRCCS, Sesto San Giovanni, Italy (Prof A L Catapano PhD); Department of Medical, Surgical, and Health Sciences (Prof L Cegolon PhD, Prof M D'Oria MD), University of Trieste, Trieste, Italy; Public Health Unit (Prof L Cegolon PhD), University Health Agency Giuliano-Isontina (ASUGI), Trieste, Italy; Department of Nutrition (Prof F Cembranel DSc), Department of Physical Education (Prof D A S Silva PhD), Federal University of Santa Catarina, Florianópolis, Brazil; College of Public Health, Medical, and Veterinary Sciences (M Cenderadewi MPHTM, A E Peden PhD), Department of Public Health and Tropical Medicine (T I Emeto PhD), College of Medicine, Dentistry and Public Health (Prof R C Franklin PhD), James Cook University, Townsville, QLD, Australia; Department of Public Health (M Cenderadewi MPHTM), University of Mataram, Mataram, Indonesia; Mary MacKillop Institute for Health Research (Prof E Cerin PhD), Australian Catholic University, Melbourne, VIC, Australia; School of Public Health (Prof E Cerin PhD, C J P Zhang PhD), Department of Urban Planning and Design (Prof C Guo PhD), Department of Surgery (Y Zhan PhD), University of Hong Kong, Hong Kong, China; Posgrado de Medicina, Facultad de Ciencias de la Salud (P Chacón-Uscamaita DDS), Faculty of Health Sciences (A Rodriguez-Morales DSc), Universidad Cientifica del Sur, Lima, Peru; Department of Biotechnology (Prof C Chakraborty PhD), Adamas University, Kolkata, India; Institute for Skeletal Aging & Orthopedic Surgery (Prof C Chakraborty PhD), Hallym University, Chuncheon, South Korea; State Disease Investigation Laboratory (S Chakraborty MVSc), Animal Resources Development Department, Agartala, India; Cardio-Oncology Research Unit (J Chan MPH),

Cardiovascular Analytics Group, Canterbury, UK; School of Nursing and Health Studies (J Chan MPH), School of Nursing and Health Sciences (X Liu PhD), School of Nursing & Health Sciences (S Ramazanu PhD), Hong Kong Metropolitan University, Hong Kong, China; Department of Applied Health Sciences (Prof J S Chandan PhD), NIHR Global Health Research Unit on Global Surgery (S K Kamarajah MD), Department of Metabolism and Systems Science (S Tariq PhD), University of Birmingham, Birmingham, UK; Department of Psychiatry (Prof M Chandradasa MD), Department of Pharmacology (Prof C D K Mettananda PhD), Department of Paediatrics (Prof S Mettananda DPhil), University of Kelaniya, Ragama, Sri Lanka; University Psychiatry Unit (Prof M Chandradasa MD), Clinical Medicine Department (Prof C D K Mettananda PhD), University Paediatrics Unit (Prof S Mettananda DPhil), Colombo North Teaching Hospital, Ragama, Sri Lanka; College of Medicine (J Chang PhD), Institute of Epidemiology and Preventive Medicine (Y L Samodra PhD), National Taiwan University, Taipei, Taiwan; Department of Nursing (J Chang PhD), National Taiwan University Hospital, Taipei, Taiwan; Department of Epidemiology and Biostatistics (V Chattu PhD), Semey Medical University (SMU), Semey, Kazakhstan; Department of Community Medicine (V Chattu PhD), Datta Meghe Institute of Medical Sciences, Sawangi, India; Department of Endocrinology (V Chatzimavridou-Grigoriadou MD), Department of Cardiovascular Science (F Mannan MD), Division of Immunology, Immunity to Infection and Respiratory Medicine (A G Mathioudakis PhD), Division of Psychology and Mental Health (F Mughal FRCGP), University of Manchester, Manchester, UK; Department of Endocrinology (V Chatzimavridou-Grigoriadou MD), Christie Hospital NHS Foundation Trust, Manchester, UK; Department of Public Health (S Chaudhuri MD), Indian Institute of Public Health, Hyderabad, India; Department of Oral Medicine and Radiology (Prof A Chaurasia MD), Department of Oral Pathology and Microbiology (A Jain MDS), Department of Psychiatry (S K Kar MD), Department of Neurology (Prof H S Malhotra D.M.), Internal Medicine Department (J Tewari MBBS), King George's Medical University, Lucknow, India; EPI (G B Chemeda MPH), Oromia Health Bureau, Addis Ababa, Ethiopia; Peking Union Medical College Hospital (A Chen PhD), Chinese Academy of Medical Sciences & Peking Union Medical College, Beijing, China; Science and Technology Department (H Chen Mmed), Northern Jiangsu People's Hospital, yangzhou, China; Clinical Research Center (H Chen PhD), Zhujiang Hospital of Southern Medical University, Guangzhou, China; Department of Computer, Electrical and Mathematical Sciences and Engineering (X Chen MSc), Computer, Electrical, and Mathematical Sciences and Engineering Division (P Moraga PhD), King Abdullah University of Science and Technology, Thuwal, Saudi Arabia; Faculty of Humanities and Health Sciences (H Chen MSc), Curtin University, Miri, Malaysia; School of Chinese Medicine (Teaching and Research Division) (H Cheng BSc), Hong Kong Baptist University, Hong Kong, China; Yong Loo Lin School of Medicine (N W Chew MD, M Ng PhD, Prof H Z Sun PhD, Prof N Venketasubramanian MSc), Department of Medicine (B Chong MBBS), Cardiovascular Metabolic Translational Research Program (M Dalakoti MPH), Saw Swee Hock School of Public Health (S Ramazanu PhD, Prof S Yi PhD), Department of Surgery (K Tan PhD), National University of Singapore, Singapore, Singapore; Department of Laboratory Medicine (J Chien PhD), Taichung Tzu-Chi Hospital Buddhist Tzu-Chi Medical Foundation, Tanzih, Taiwan; Department of Medical Laboratory Science and Biotechnology (J Chien PhD), Central Taiwan University of Science and Technology, Taiwan; Department of Public Health and Health Policy (O Chimed-Ochir PhD, A Fukunaga PhD, I Khaing MPH), Department of Epidemiology (Z Phyo MD), Hiroshima University, Hiroshima, Japan; Department of Clinical Oncology (W C S Cho PhD), Queen Elizabeth Hospital, Hong Kong, China; Division of Plastic Surgery (D Y Cho MD), University of Wisconsin, Madison, WI, USA; Centre for Research Impact & Outcome (H Chopra PhD), Chitkara University, Rajpura, India; Department of Community Medicine (Prof S G Choudhari MD), Global Consortium for Public Health and Research (Prof

Z Quazi PhD), Jawaharlal Nehru Medical College, Wardha, India; Southeast University (S Chowdhury PhD), Southeast University, Dhaka, Bangladesh; Department of Public Health (M Chowdhury PhD), Asian University for Women, Chittagong, Bangladesh; The Interdisciplinary Research Group on Biomedicine and Health (D Chu PhD), Faculty of Applied Sciences (D Chu PhD), VNU International School (VNUIS), Hanoi, Viet Nam; Department of Pediatrics (H Chu PhD), Department of Epidemiology and Biostatistics (Prof J Liu PhD), school of public health (Y Wang MD), National Institute of Health Data Science (W Wang PhD), School of Public Health (H Zhang PhD), Peking University, Beijing, China; Department of Paediatric Surgery (I S Chukwu BMedSc), Federal Medical Centre, Umuahia, Nigeria; Faculty of Social Sciences (S Chukwudeh PhD), Federal University Oye-Ekiti Nigeria, Oye-Ekiti, Nigeria; Department of AndroUrology (Prof E Chung MD), AndroUrology Centre, Brisbane, QLD, Australia; Health Data Research UK, London, UK (S Chung PhD); Department of Health Behavior (S Chung MPH), Department of Biomedical Engineering (M Negahdary PhD), Center for Remote Health Technologies & Systems (M Negahdary PhD), Texas A&M University, College Station, TX, USA; Nova Medical School (Prof J Conde PhD), Nova University of Lisbon, Lisbon, Portugal; Department of Cardiovascular Sciences (N Conrad PhD, J Van den Eynde BSc), Department of Abdominal Surgery (A Teymouri MD), Katholieke Universiteit Leuven, Leuven, Belgium; Research Center on Public Health (CESP) (P Cortesi PhD), School of Medicine and Surgery (Prof L G Mantovani DSc), University of Milan Bicocca, Monza, Italy; Laboratory of Public Health (P Cortesi PhD), Instituto Auxologico Italiano IRCCS (Italian Auxological Institute), Milan, Italy; Department of Health Sciences (C Cosma MD), University of Florence, Florence, Italy; Department of Family Medicine and Public Health (Prof M H Criqui MD), University of California San Diego, La Jolla, CA, USA (L Diaz MD); Life and Health Sciences Research Institute (ICVS) (Prof N Cruz-Martins PhD), University of Minho, Braga, Portugal; Faculty of Medicine (N Dababo MD), University of Aleppo, Aleppo, Syria; Research Center for Child Psychiatry (O Dadras PhD), Heart Center (V Kytö MD), University of Turku, Turku, Finland; Departartment of Allied health sciences (Z U Dahiru MSc), Federal University of Health Science Azare, Katagum-Azare, Nigeria; Department of Community Medicine (Prof T Dahiru MA, A A Olorukooba MD), Health Systems and Policy Research Unit (Prof S Mohammed PhD), Department of Paediatrics (S Musa MSc), Ahmadu Bello University, Zaria, Nigeria; Institute for Health Sciences (Prof K Dalal PhD), Mid Sweden University, Sundsvall, Sweden; Public Health Foundation of India, Gurugram, India (Prof R Dandona PhD, Prof L Dandona MD, G Kumar PhD); Department of Internal Medicine (P Danpanichkul MD), Texas Tech University, Lubbock, TX, USA; Ga East Municipal Hospital (S E Danso MPH), Ghana Health Service, Accra, Ghana; Department of Public Health (S D Darcho MPH), School of Pharmacy (A S Mohammed BA), School of Public Health (A Oumer PhD), Department of Epidemiology and Biostatistics (B S Tusa MPH), Haramaya University, Harar, Ethiopia; School of Public Health (C Dare PhD), University of the Witwatersrand, Johannesburg, South Africa; Division of Women and Child Health (J K Das MD), Aga Khan University, Karachi, Pakistan; Health Policy (B A D'Avanzo MA), Department of Medical Epidemiology (S Gallus PhD), Mario Negri Institute for Pharmacological Research, Milan, Italy; Department of Population and Development (C A Dávila-Cervantes PhD), Latin American Faculty of Social Sciences Mexico, Mexico City, Mexico; Department of Legal Medicine, Psychiatry and Pathology (A de la Torre-Luque PhD), Universidad Complutense de Madrid (Complutense University of Madrid), Madrid, Spain; Memorial Sloan Kettering Cancer Center (E Dee MD), Memorial Sloan Kettering Cancer Center, New York, NY, USA; Department of Pediatrics (S Deekonda MD), Brookdale University Hospital Medical Center, Brooklyn, NY, USA; Department of Implementation Research (D M Dekker PhD), Bernhard Nocht Institute for Tropical medicine, Hamburg, Germany; Department of Neurosurgery (A K Demetriades MD), Centre for Medical Informatics (Prof A Sheikh MD), College of Medicine and

Veterinary Medicine (G Verras MSc), University of Edinburgh, Edinburgh, UK; Department of Neurosurgery (A K Demetriades MD), National Health Service (NHS) Scotland, Edinburgh, UK; Dirección de Nutrición (E Denova-Gutiérrez DSc), Salvador Zubiran National Institute of Medical Sciences and Nutrition, Mexico City, Mexico; Department of Biological Sciences (I Dergaa PhD), University of Manouba, Manouba, Tunisia; Department of Social Sciences (I Dergaa PhD), University of Jendouba, El Kef, Tunisia; Wellcome Trust Brighton and Sussex Centre for Global Health Research (K Deribe PhD), Brighton and Sussex Medical School, Brighton, UK; Department of Forensic Medicine (E Dervišević PhD), University of Sarajevo, Sarajevo, Bosnia and Herzegovina; Clinical and Public Health Research (H Desai MBBS), Independent Clinician Scientist and Public Health Researcher, Ahmedabad, India; Department of Statistics, Computer Science, Applications "G. Parenti" (DiSIA) (A Desta MSc), University of Florence and University of Palermo, Florence, Italy; Chettinad Hospital & Research Institute (Prof V Devanbu MD), Chettinad Academy of Research and Education, Chennai, India; Department of Cardiology (P Devarakonda MD), Department of Neurosurgery (K Margetis MD), Icahn School of Medicine at Mount Sinai, New York, NY, USA; JSS Medical College Department of Biochemistry (D Devegowda PhD), Jagadguru Sri Shivarathreeswara Academy of Health Education and Research, Mysuru, India; Sheffield Teaching Hospitals NHS Foundation Trust, Sheffield, UK (A Dhali MBBS); Division of Pathology (K Dhama PhD), ICAR-Indian Veterinary Research Institute, Bareilly, India; Neurology Department Institute of Human Behavior and Allied Sciences (Prof R K Dhamija MD), University of Delhi, New Delhi, India; Department of Community Medicine (Prof S D Dharmaratne MD), Department of Oral Medicine and Periodontology (Prof R D Jayasinghe MS), Faculty of Dental Sciences (Y A Jayasinghe BSc), University of Peradeniya, Peradeniya, Sri Lanka; Research Department (M Dhimal PhD), Nepal Health Research Council, Kathmandu, Nepal; Institute of Occupational, Social and Environmental Medicine (M Dhimal PhD), Goethe University, Frankfurt am Main, Germany; Department of Life Science and Public Health (M Di Pumpo DrPH), Università Cattolica del Sacro Cuore (Catholic University of the Sacred Heart), Rome, Italy; Escola Superior de Saúde (Higher School of Health) (Prof D Dias da Silva PhD), Instituto Politécnico do Porto (Polytechnic Institute of Porto), Porto, Portugal; Department of Quantitative Methods (D Diaz-Milanes PhD), Loyola University Andalusia, Sevilla, Spain; Health Research Institute (D Diaz-Milanes PhD), University of Canberra, Canberra, New South Wales (NSW), Australia; Department of Otolaryngology - Head and Neck Surgery (L K Dillard PhD), Department of Otolaryngology-Head and Neck Surgery (J R Dubno PhD), Medical University of South Carolina, Charleston, SC, USA; Department of Anesthesiology (Prof Z Ding PhD), Department of Epidemiology and Health Statistics (Prof G Hu PhD), Xiangya Hospital (Y Liu MSc), Central South University, Changsha, China; Joe C. Wen School of Population & Public Health (X Ding MA), University of California Irvine, Irvine, CA, USA; Research Institute for Advanced Nursing (RIAN) (H Do PhD), Dong Nai Technology University, Dong Nai Province, Viet Nam; Institute of Health Economics and Technology (iHEAT), Hanoi, Viet Nam (H Do PhD); Department of Medicine (T H P Do MD), Can Tho University of Medicine and Pharmacy, Can Tho, Viet Nam; Department of Social Medicine and Health Care Organisation (Prof K G Dokova PhD), Medical University of Varna, Varna, Bulgaria; Nuclear Medicine Deparment (F Dondi MD), ASST Spedali Civili di Brescia and Università degli Studi di Brescia, Brescia, Italy; Cardio-Thoraco-Vascular Department (Prof M D'Oria MD), Azienda Sanitaria Universitaria Giuliano Isontina, Trieste, Italy; Independent Consultant, Bridgewater, NJ, USA (O P Doshi MS); Department of Epidemiology (M Dresse MD), University of Pittsburgh, Pittsburgh, PA, USA; Department of Psychiatry (M Dresse MD), University of Pittsburgh Medical Center, Pittsburgh, PA, USA; Department of Pathology (Prof J Du PhD), China Medical University, Liaoning, China; School of Sociology (E W Dumbili PhD), University College Dublin, Dublin, Ireland; Postgraduate Program in Health Sciences

(S C Dumith PhD), Federal University of Rio Grande do Sul, Rio Grande, Brazil; Postgraduate Program in Epidemiology (Prof B B Duncan MD, Prof M I Schmidt MD), Department of Social Medicine (R Mattiello PhD), Federal University of Rio Grande do Sul, Porto Alegre, Brazil; School of Population Health (J Dunne PhD, Prof P W Gething PhD, S D Nyadanu PhD, D J Weiss PhD), School of Pharmacy (A K Sendekie MSc), Curtin University, Perth, WA, Australia; School of Medicine (Prof A R Duraes PhD), Institute of Collective Health (Prof D Rasella PhD), Federal University of Bahia, Salvador, Brazil; Department of Internal Medicine (Prof A R Duraes PhD), Escola Bahiana de Medicina e Saúde Pública (Bahiana School of Medicine and Public Health), Salvador, Brazil; Faculty of Science and Humanities (S Duraisamy PhD), SRM Institute of Science and Technology, Kattankulathur, India; Department of Infection and Tropical Medicine (O C Durojaiye MPH), School of Medicine and Population Health (N S George MPH), University of Sheffield, Sheffield, UK; Department of Pharmacology (S Dutta MD), All India Institute of Medical Sciences, Rajkot, India; Department of Biological and Chemical Sciences (O Ebohon MPH), Michael and Cecilia Ibru University, Delta State, Nigeria; Department of Psychiatry (E Eboreime PhD, E Tsermpini PhD), Dalhousie University, Halifax, NS, Canada; Department of Psychiatry (E Eboreime PhD), Department of Medicine (E Lytvyak MD, Prof S Straube DPhil), University of Alberta, Edmonton, AB, Canada; Environmental and Occupational Health Research Center (M Ebrahimi MD), Shahroud University of Medical Sciences, Shahroud, Iran; Higher School of Technology (Prof A Ed-Dra PhD), Sultan Moulay Slimane University, Beni Mellal, Morocco; Advanced Nursing Department (F Efendi PhD), Department of Epidemiology Population Biostatistics and Health Promotion (A Hargono PhD), Department of Advanced Nursing (E M M Has PhD), Department of Biology (Prof H Purnobasuki PhD), Universitas Airlangga (Airlangga University), Surabaya, Indonesia; Semnan University of Medical Sciences and Health (F Ehsani PhD), Samara University, Semnan, Iran; Isenberg School of Management (A Eighaei Sedeh MD), University of Massachusetts Amherst, Amherst, MA, USA; Centre for Global Health Inequalities Research (CHAIN) (Prof T Eikemo PhD, H Hoven DrPH), Norwegian University of Science and Technology, Trondheim, Norway; Private Orthodontist, Ahvaz, Iran (E Eini MSD); Faculty of Science and Health (M Ekholuenetale PhD), University of Portsmouth, Hampshire, UK; Almoosa College of Health Sciences, Al Ahsa, Saudi Arabia (R A El Arab PhD); Clinical Pathology Department-Faculty of Medicine (Prof M El Sayed Zaki PhD), Department of Anatomy and Embryology (M A Eladl PhD), Department of Clinical Pathology (Prof M Elshaer PhD), Department of Cardiology (Prof M M Ramadan PhD), Faculty of Pharmacy (Prof M A Saleh PhD), Rheumatology and Immunology Unit (Prof S Tharwat MD), Faculty of Nursing (M Zoromba PhD), Mansoura University, Mansoura, Egypt; College of Medicine (Prof R Elbeshbeishy PhD), RAK College of Nursing (M Mohamed PhD), RAK Medical and Health Sciences University, Ras Al-Khaimah, United Arab Emirates; Faculty of Medicine (Prof R Elbeshbeishy PhD), Department of Internal Medicine (Prof G M T ElGohary MD), Biochemistry Department (Prof N M Hamdy PhD), Department of Entomology (A M Samy PhD), Medical Ain Shams Research Institute (MASRI) (A M Samy PhD), Neurology Department (Prof A S Shalash PhD), Ain Shams University, Cairo, Egypt; Clinical Pharmacy Program (Prof F El-Dahiyat PhD), Al Ain University, Al Ain, United Arab Emirates; Pediatrics and Neonatology Department (M Eldegwi MD), Kafr Elshiekh University, Kafr Elshiekh, Egypt; College of Medicine (M Elhadi MD, Prof S Jeong PhD), Department of Medicine (I R Fakhradiyev PhD), School of Health and Environmental Science (Prof J Kang PhD), Department of Health Policy and Management (Prof J Kim PhD), Department of Preventive Medicine (Prof Y Lee PhD), Korea University, Seoul, South Korea (Prof M Shin PhD); Houston Methodist Hospital, Houston, TX, USA (M Elhadi MD); the National Institute of Public health Research (M Elhoumed PhD), Ministry of Health, Nouakchott, Mauritania; School of Pharmacy and Pharmaceutical Sciences (M Elnaem PhD), Ulster University, Coleraine, UK;

Department of Animal Medicine (I Elsohaby PhD), Department of Pathology (Prof M M M Metwally PhD), Cardiovascular Department (Prof A M A Saad MD), Zagazig University, Zagazig, Egypt (Prof M I Husseiny PhD); Department of Pediatrics (C Eltaha MD), University of Texas, Dallas, TX, USA; Faculty of Veterinary Medicine (Prof A S A Eltahawy PhD), Damanhour University, Damanhur, Egypt; Department of Midwifery (T Emagneneh MSc), Woldia University, Addis Ababa, Ethiopia; Department of Research (S Emdadul Haque PhD), UChicago Research Bangladesh, Dhaka, Bangladesh; Department of Community Health (S C Eneh MPH), Obafemi Awolowo University, Ile-Ife, Nigeria; Ivan Research Institute (S C Eneh MPH), University of Nigeria, Enugu, Nigeria; Department of Paediatrics (C I Esezobor MB), Lagos University Teaching Hospital, Lagos, Nigeria; Department of Bacteriology and Virology (M Eslami PhD), Research Center of Physiology (H Yaribeygi PhD), Semnan University of Medical Sciences, Semnan, Iran; Cancer Research Center (M Eslami PhD), Semnan University of Medical Sciences, Semnan, Iran; Department of Biomedical Sciences (N Fabin MD, M Valenti MD), Humanitas University, Milan, Italy; Dermatology Unit (M Valenti MD), IRCCS Humanitas Research Hospital, Milan, Italy (N Fabin MD); Department of Electrical and Computer Engineering (H Fadavian MSc), Department of Electrical and Computer Engineering (ECE) (Prof D Fathi PhD), Department of Biostatistics (K Gohari MS), Departmant of Hematology (B Razi PhD), Tarbiat Modares University, Tehran, Iran; Research Centre for Healthcare and Community (A F Fagbamigbe PhD), Centre for Intelligent Healthcare (H Liu PhD), Coventry University, Coventry, UK; Department of Oral Biology (A Fahim PhD), Riphah International University, Islamabad, Pakistan (Z Z Piracha PhD); The Maldives National University (R Faiz MPH), The Maldives National University, Male, Maldives; Department of Food Hygiene and Quality Control (A Fakhri-Demeshghieh PhD), School of Biotechnology (M Yeganeh PhD), University of Tehran, Tehran, Iran; Epidemiology and Biostatistics Unit (L Falzone PhD), IRCCS Pascale, Naples, Italy; Department of Public Health Sciences (Q Fan DrPH), School of Computing (K Savoji MS), Clemson University, Clemson, SC, USA; Saveetha Medical College and Hospital (M Fareed PhD), Department of Public Health Dentistry (Prof G Mini PhD), Saveetha Institute of Medical and Technical Sciences (SIMATS), Chennai, India; Department of Psychology (A Faro PhD), Federal University of Sergipe, São Cristóvão, Brazil; Department of Radiography and Imaging Technology (S Farooq PhD), Green International University, Lahore, Pakistan; Department of Clinical Psychology (M Faruk MSc), Department of Population Sciences (Prof M B Hossain PhD), University of Dhaka, Dhaka, Bangladesh; Community-based Inclusive Mental Health Department (M Faruk MSc), Centre for Disability in Development (CDD), Dhaka, Bangladesh; Satcher Health Leadership Institute (A O Fasanmi PhD), Morehouse School of Medicine, Atlanta, GA, USA; Department of Veterinary Tropical Diseases (Prof F O Fasina PhD), University of Pretoria, Pretoria, South Africa; Animal Production and Health Division (EMPRES) (Prof F O Fasina PhD), Food and Agriculture Organization of the United Nations, Rome, Italy; Charité University Berlin (M M Fasina MSc), Charité Universitätsmedizin Berlin (Charité University Medical Center Berlin), Berlin, Germany; School of Engineering (A Fatehizadeh PhD), Edith Cowan University, Joondalup, WA, Australia; National Institute for Stroke and Applied Neurosciences (Prof V L Feigin PhD), The National Institute for Stroke and Applied Neurosciences (I Rautalin PhD), Auckland University of Technology, Auckland, New Zealand; Research Center of Neurology, Moscow, Russia (Prof V L Feigin PhD); National Institute of Environmental Health (X Feng PhD), National Center for Chronic and Noncommunicable Disease Control and Prevention (P Ye PhD), Chinese Center for Disease Control and Prevention, Beijing, China; Department of Biomedical Engineering (T Ferdous MSc), Department of Decision and Information Sciences (M Hossain DrPH), Department of Biology and Biochemistry (S Ullah MSc), University of Houston, Houston, TX, USA; Division of Neurology (S Fereshtehnejad PhD), University Health Network (S Mirshahvalad MD),

University of Toronto, Toronto, ON, Canada; Department of Social Sciences (Prof N Ferreira PhD, Prof M J M Sullman PhD), Department of Life and Health Sciences (Prof M J M Sullman PhD), University of Nicosia, Nicosia, Cyprus; Medical School (A Finnemore HSdipl), Universidad de Navarra, Pamplona, Spain; Department of Cell Biology and Biotechnology (A A Fomenkov PhD), K.A. Timiryazev Institute of Plant Physiology, Moscow, Russia; Department of Cardiac, Thoracic, Vascular Sciences and Public Health (M Fonzo MD), University of Padova, Italy, Padova, Italy; Department of Neurology, Public Health and Disability (A Fornari PhD), Fondazione IRCCS Istituto Neurologico Carlo Besta, Milano, Italy; Department of Pharmacology (Prof B Foroutan PhD), Iranshahr University of Medical Sciences, Iranshahr, Iran; Innovation in Healthcare and Social Services Department (D Fortuna MSc), Emilia-Romagna Region, Bologna, Italy; Department of Neuroscience (M Foschi MD), Multiple Sclerosis Research Center, Ravenna, Italy; Department of Biotechnological and Applied Clinical Sciences (M Foschi MD), University of L'Aquila, L'Aquila, Italy; Center for Health Technology and Services Research (CINTESIS), Porto, Portugal (A Freitas PhD); Department of Dermatology (Prof T Fukumoto PhD), Kyoto Prefectural University of Medicine, Kyoto, Japan; Department of Pathology (Prof B Fux PhD), Department of Integrated Health Education (Prof L B Salaroli PhD), Federal University of Espirito Santo, Vitória, Brazil; Department of Community Medicine and Family Medicine (S G MD, V J MD), All India Institute of Medical Sciences, Gorakhpur, India; Health Services Management Training Centre (Prof P A Gaal PhD, T Joo PhD, T Palicz MD), Semmelweis University, Budapest, Hungary; Department of Applied Social Sciences (Prof P A Gaal PhD), Sapientia Hungarian University of Transylvania, Târgu-Mureş, Romania; Department of Community Medicine (Prof M A Gadanya MD), Aminu Kano Teaching Hospital, Kano, Nigeria; School of Public Health (D Gadeka PhD), School of Pharmacy (Prof I A Kretchy PhD), West African Center for Cell Biology of Infectious Pathogens (I A Owusu PhD), University of Ghana, Legon, Ghana; Department of Oral Biology and Experimental Dental Research (M Gajdács PhD), University of Szeged, Szeged, Hungary; Department of Food Technology (Y Galali ResM), Department of Chemistry (H I M. Amin PhD), Salahaddin University-Erbil, Erbil, Iraq; School of American Education, Institute of Health & Management (B Ganesan PhD), Institute of Health & Management, Australia, Melbourne, VIC, Australia; Swinburne University of Technology (B Ganesan PhD), School of Engineering, Melbourne, VIC, Australia; Department of Biostatistics (Prof X Gao PhD), Key Lab of Environment and Health (Prof X Gao PhD), School of Public Health (Y Huang PhD, Z Huang PhD, W Li PhD, Q Wang PhD, F Wang PhD, Prof W Wang PhD, L Xiao PhD, X Yang PhD, T Zhan PhD), Xuzhou Medical University (M Jiang PhD), Department of Epidemiology (D Yin DrPH), Xuzhou Medical University, Xuzhou, China; Joint Surgery and Sports Medicine (Y Gao MD), Institute of Science Tokyo, Tokyo, Japan; Department of Public Health (B Garba PhD), SIMAD University Mogadishu. Somalia, Mogadishu, Somalia; School of Medicine (M Garcia-Argibay PhD), Orebro University, Orebro, Sweden; Department of Medicine (Prof D Garcia-Azorin MD), University of Valladolid, Valladolid, Spain; Department of Neurology (Prof D Garcia-Azorin MD), Hospital Universitario Rio Hortega, Valladolid, Spain; Infectious Diseases Unit (J Garlasco MD), University of Verona, Verona, Italy; Department of Pharmacology (Prof R K Gautam PhD), IES Institute of Pharmacy, Bhopal, India; Professional Services Division (P Gautam PhD), Texas State Board of Pharmacy, Austin, TX, USA; Institute of Health and Development (ISED) (Prof B Gaye PhD), Alliance for Medical Research in Africa (AMedRA), Dakar, Senegal; Independent Consultant, Rome, Italy (F Gazzelloni MSc); School of Public Health (H Ge MD), Shandong First Medical University and Shandong Academy of Medical Sciences, Jinan, China; Department of Midwifery (M W Gebregergis MSc), School of Public Health (H G Gebreslassie MPH), Department of Medical Laboratory Sciences (H N Meles MSc), Adigrat University, Adigrat, Ethiopia; School of Public Health (M Gelchu MPH), Bule Hora University, Bule Hora, Ethiopia;

Department of Neurosciences, Neurology and Stroke Unit (S Gelibter MD), Stroke Unit and Neurology Unit (G Schwarz MD), ASST Grande Ospedale Metropolitano Niguarda, Milan, Italy; Institute of Public Health (N S George MPH), Jagiellonian University Medical College, Krakow, Poland; Department of Public Health (L Getacher PhD), Debre Berhan University, Debre Berhan, Ethiopia; Department of Public Health (G K Getahun MPH), Menelik II Medical and Health Science College, Addis Ababa, Ethiopia; Mayo Clinic, rochester, MN, USA (D J Ghadimi MD); Infectious Disease Research Center (Prof K Ghadiri MD), Pediatric Department (Prof K Ghadiri MD), Department of Rehabilitation and Sports Medicine (M Mirzaei MSc), Research Center for Environmental Determinants of Health (Prof E Sadeghi PhD), Social Development and Health Promotion Research Center (A Zangeneh MSc), Kermanshah University of Medical Sciences, Kermanshah, Iran; Research Committee of qom university of medical sciences (A Ghamkhar BSc), Department of Health Management and Economics (M Khosravi PhD), Qom University of Medical Sciences, Qom, Iran; Family and Community Medicine Department (R M Ghazy PhD), King Khalid University, Abha, Saudi Arabia; Research Group for Childhood Cancer (N Ghith PhD), Danish Cancer Research Institute, Copenhagen, Denmark; Department of Physics (Z Gholami PhD), University of Zanjan, Zanjan, Iran; Department of Dermatology (N Gholizadeh MD, G Rahmatpour Rokni MD), Invasive Fungi Research Center (J Javidnia PhD), Department of Medical Mycology (J Javidnia PhD), Department of Biostatistics (Prof A Khalilian PhD), Department of Medical-Surgical Nursing (S Shorofi PhD), Mazandaran University of Medical Sciences, Sari, Iran; Department of Biology (A Ghuge MPhil), Government Institute of Science, Nagpur, India; Department of Clinical Research (A Ghuge MPhil), National Institute For Research In Reproductive and Child Health, Mumbai, India; Department of Epidemiology and Prevention (A Gialluisi PhD), IRCCS Neuromed, Pozzilli, Italy; GBD Collaborating Unit (K Giannakis, Prof S E Vollset DrPH), Centre for Disease Burden (A S Knudsen PhD), Department of Disease Burden (C Madsen PhD, C Schwinger PhD), Norwegian Institute of Public Health, Bergen, Norway; Department of Biological Sciences and Chemistry (DBSC) (S A Gilani PhD), Department of Biological Sciences and Chemistry (Prof J Hussain PhD), Natural and Medical Sciences Research Center (A Khan PhD, S Shahid MPhil, A Ullah MS), Dept of Biological Sciences and Chemistry (Z Naureen PhD), School of Pharmacy (A K Philip PhD), University of Nizwa, Nizwa, Oman; Adelaide Medical School (T K Gill PhD), School of Economics and Public Policy (F Ploeckl PhD), University of Adelaide, Adelaide, SA, Australia; Department of Nursing (A A Girmay MSc), Aksum University, Aksum, Ethiopia; Department of Anesthesiology and Critical Care Medicine (A Girombelli MD), Ospedale SS Annunziata Savigliano, Savigliano, Italy; Lerner College of Medicine (Prof L Göbölös PhD), Harrington Heart and Vascular Institute (A Guha MD), Department of Quantitative Health Science (Prof X Liu PhD), Department of Endocrinology (A Sood MD), Case Western Reserve University, Cleveland, OH, USA; Department of Pharmaceutical Sciences and Drug Research (Prof R K Goel PhD), Department of Human Genetics (P Singh PhD), Punjabi University Patiala, Patiala, India; Department of Radiation Oncology (Prof A K Goel MD), All India Institute of Medical Sciences, Punjab, India; Department of Medicine (A Goel MBBS), Department of Human Anatomy (A Patra MD), Department of Community Medicine and Family Medicine (M Verma MD), Department of Paediatrics (A Wander DM), All India Institute of Medical Sciences, Bathinda, India; Quantitative Department (K Gohari MS, A Sheidaei PhD), Non-Communicable Diseases Research Center (NCDRC) (A Golestani MD), Department of Epidemiology (S Khanmohammadi MD), Department of Epidemiology and Biostatistics (S Khosravi MD), Non-Communicable Diseases Research Center (NCDRC), Tehran, Iran; Department of Health Systems and Policy Research (Prof M Golechha PhD), Indian Institute of Public Health, Gandhinagar, India; Laboratório de Farmacognosia (N G M Gomes PhD), REQUIMTE/LAQV, Porto, Portugal; Department of Urban Public Health (Prof P N Gona PhD), Department of Nursing (G K Kyei BSc,

E F Kyei PhD), University of Massachusetts Boston, Boston, MA, USA; Senior Department of Tuberculosis (Prof W Gong PhD), The Eighth Medical Center of PLA General Hospital, Beijing, China; Midwifery Department (Y A Goshu MSc), Department of Epidemiology and Biostatistics (G A Yemata MPH), Debre Tabor University, Debre Tabor, Ethiopia; Department of Epidemiology (Prof A C Goulart PhD), Universidade de São Paulo (University of São Paulo), São Paulo, Brazil; Department of Dermatology (A Grada MD), Case Western Reserve University, Libertyville, IL, USA; Liverpool Orthopaedic and Trauma Service (S M Graham PhD), University of Liverpool, Liverpool, UK; Department of Public Health and Preventive Medicine (Prof M Grivna PhD), Charles University, Prague, Czech Republic; Tianjin Medical University General Hospital (B Guan MD), Department of Cardiology (Prof G Tse PhD), Tianjin Medical University, Tianjin, China; Cheeloo College of Medicine (B Guan MD), School of Nursing and Rehabilitation (N Xiao BS), Shandong University, Jinan, China; Department of Epidemiology and Biostatistics (S Guan MD), Department of Urology (C Mao MSc), Anhui Medical University, Hefei, China; Health Direction (G Guarducci MD), Local Health Authority of Ferrara, Ferrara, Italy; Department of Clinical Science (M I M Gubari PhD), University Of Sulaimani, Sulaimani, Iraq; Division of Cardiovascular Medicine (A Guha MD), Center for Tobacco Research (C Keke PhD), Ohio State University, Columbus, OH, USA; Department of the Health Directorate (S Guicciardi MD), Local Health Authority of Bologna, Bologna, Italy; Group Health Department (Z Guo MPH), Nanyang Central Hospital, Nanyang, China; Department of Geriatric Neurology (X Guo PhD), Shaanxi Provincial People’s Hospital, Xi'an, China; Divison of Epidemology (Z Guo PhD), Vanderbilt University Medical Center, Nashville, TN, USA; Department of Epidemiology and Biostatistics (R Gupta MPH), University of South Carolina, Columbia, SC, USA; Department of Preventive Cardiology & Medicine (Prof R Gupta MD), Eternal Heart Care Centre & Research Institute, Jaipur, India; Department of Medicine (Prof R Gupta MD), Mahatma Gandhi University Medical Sciences, Jaipur, India; Department of Toxicology (S Gupta PhD), Shriram Institute for Industrial Research, Delhi, India; Department of Anaesthesia (Prof L Gupta MD), Maulana Azad Medical College, New Delhi, India; College of Medicine and Public Health (H Gupta PhD), Flinders University, Melbourne/Darwin, Victoria (VIC), Australia; Doctoral Program in Biomedical Gerontology (R S Gutiérrez-Murillo PhD), Pontifical Catholic University of Rio Grande do Sul, Porto Alegre, Brazil; Research Unit in Epidemiology Clinic (J Guzman-Esquivel DSc), Mexican Institute of Social Segurity, Colima, Mexico; College of Health Science (A T Habteyes MPH), Dilla University, Dilla, Ethiopia; Department of Midwifery (T S Hadaro MSc), Department of Public Health (T Mekene Meto MPH), Department of Biomedical Sciences (H T Wada MSc), Arba Minch University, Arba Minch, Ethiopia; School of Medicine (A Haghtalab MD), Hamedan University of Medical Sciences, Hamedan, Iran; Department of Liver Tumor, Cancer Center (N Hai Nam PhD), Liver Transplant Unit (N Hai Nam PhD), Cho Ray Hospital, Ho Chi Minh City, Viet Nam; Department of Community Medicine (P Halder MD), Department of Pharmacology (A K Kakkar MD), Department of Pediatrics (J Kumar MD), Post Graduate Institute of Medical Education and Research, Chandigarh, India; Centre for Community Medicine (P Halder MD), Department of Biophysics (T Mohammad PhD), Centre for Dental Education and Research (H Priya MDS), Department of Psychiatry (Prof R Sagar MD), Department of Radiation Oncology (A Shankar MD), Department of Laboratory Medicine (A Singh PhD), All India Institute of Medical Sciences, New Delhi, India; Department of Infectious Disease Epidemiology (S Haller MD), Robert Koch Institute, Berlin, Germany; Department of Public Health (S Haller MD), Charité Institute of Public Health, Berlin, Germany; Department of Pharmacy (Prof I M Hamad PhD), American University of Madaba, Amman, Jordan; Department of Family and Community Medicine (Prof R R Hamadeh PhD), College of Medicine and Health Sciences (H Jahrami PhD), Arabian Gulf University, Manama, Bahrain; School of Health and Environmental Studies (Prof S

Hamidi DrPH), Hamdan Bin Mohammed Smart University, Dubai, United Arab Emirates; Department of Medical and Technical Information Technology (A Hammoud PhD), Bauman Moscow State Technical University, Moscow, Russia; Guthrie Medical Group (M Hamza MD), Guthrie Medical Group, Cortland, NY, USA; Edirne Public Health Center (D Han Yekdeş MD), Edirne Provincial Health Directorate, Edirne, Turkiye; Sakarya University, Sakarya, Turkiye (A Hanif PhD); Centre for Neuromuscular and Neurological Disorders (Perron Institute) (Prof G J Hankey MD), The University of Western Australia, Perth, WA, Australia; Stroke Research Centre (Prof G J Hankey MD), Perron Institute for Neurological and Translational Science, Perth, WA, Australia; Department of Health and Education (F Hanna PhD), Torrens University Australia, Melbourne, VIC, Australia; Department of Population Science and Human Resource Development (Prof M Haque PhD, Prof M Rahman PhD, Prof M Rahman DrPH), Department of Physics (A Hossain PhD), University of Rajshahi, Rajshahi, Bangladesh; Department of Medicine (O I Haque MD), MedStar Health, Baltimore, MD, USA; Medical Research Unit (H Harapan PhD), Universitas Syiah Kuala (Syiah Kuala University), Banda Aceh, Indonesia; Vital and Health Statistics (H L Harb MPH), Ministry of Health, Beirut, Lebanon; Department for Health (H L Harb MPH), University of Bath, Bath, UK; Directorate General of Health Human Resources (A M A Hariandja DrPH), Ministry of Health, Jakarta, Indonesia (H U Pangaribuan MSc); Research Unit (J M Haro MD), Parc Sanitari Sant Joan de Deu, Barcelona, Spain; Department of Mental Health (J M Haro MD), Carlos III Health Institute (Prof R Tabarés-Seisdedos PhD), Biomedical Research Networking Center for Mental Health Network (CiberSAM), Madrid, Spain; School of Nursing and Midwivery (E M M Has PhD), La Trobe University, Bundoora, VIC, Australia; Department of Health Research Methods, Evidence, and Impact (M Hasan MPH), Population Health Research Institute (PHRI) (F Mannan MD), Department of Psychiatry and Behavioural Neurosciences (Prof A T Olagunju PhD), McMaster University, Hamilton, ON, Canada; Department of Biochemistry and Molecular Biology (M Hasan MPH), Tejgaon College, Dhaka, Bangladesh; Faculty of Nursing (F Hasan PhD, D S Romadlon PhD), Chulalongkorn University, Bangkok, Thailand; Department of Food Technology and Nutrition Science (T Hasan PhD), Noakhali Science and Technology University, Noakhali, Bangladesh; Department of Medical Surgical (Prof A Hasanpour-Dehkordi PhD), Shahroud University of Medical Sciences, Shahrekord, Iran; institute of radiology and radiological sciences (A Hashem Zadeh MD), Tehran University of Medical Sciences, tehran, Iran; Department of Periodontics (N T Hashim PhD), RAK Medical & Health Sciences University, Ras Al-Khaimah, United Arab Emirates; Department of Biotechnology (A Hasnain PhD), Lahore University of Biological and Applied Sciences, Lahore, Pakistan; Community Medicine (I Hassan PhD), Federal University Teaching Hospital, Lafia, Nigeria; Department of Epidemiology and Community Medicine (I Hassan PhD), Federal University of Lafia, Lafia, Nigeria; Department of Medicine (I Hassan MD), University of Khartoum Faculty of Medicine, Khartoum, Sudan; Health Policy and Financing (Y W Hassan Wada MPH), Society for Family Health, Abuja, Nigeria; Skaane University Hospital (R J Havmoeller PhD), Skaane County Council, Malmö, Sweden; Institute of Pharmaceutical Sciences (K Hayat MS), University of Veterinary and Animal Sciences, Lahore, Pakistan; Department of Pharmacy Administration and Clinical Pharmacy (K Hayat MS), Xian Jiaotong University, Xian, China; Faculty of Kinesiology (Prof J J Hebert PhD), University of New Brunswick, Fredericton, NB, Canada; School of Allied Health (Prof J J Hebert PhD), Murdoch University, Murdoch, WA, Australia; Independent Consultant, Santa Clara, CA, USA (G Heidari MD); Department of Medicine (M Hemmati MD), Internal Medicine Department (L Manjani MD), MedStar Health, Washington, DC, USA; Babes-Bolyai University, Cluj-Napoca, Romania (Prof C Herteliu PhD); Australian Centre for Health Service Innovations (S A Hewage MD), Queensland University of Technology, Brisbane, Queensland (QLD), Australia; Graduate School of Medicine (Y Hiraike

PhD), Department of Global Health Policy (S K Rauniyar PhD), University of Tokyo, Tokyo, Japan; Department of Pulmonology (N Horita PhD), Yokohama City University, Yokohama, Japan; School of Population and Public Health (M Hossain PhD, I O Iyamu MD), University of British Columbia, Vancouver, BC, Canada; Centre for Advancing Health Outcomes, Vancouver, BC, Canada (M Hossain PhD); Public Health Research Group (M Hossain DrPH), Nature Study Society of Bangladesh, Khulna, Bangladesh; Department of Statistics (M Hossain MSc, S Noor MS), Shahjalal University of Science and Technology, Sylhet, Bangladesh; Department of Clinical Legal Medicine (Prof S Hostiuc PhD), National Institute of Legal Medicine Mina Minovici, Bucharest, Romania; National School of Tropical Medicine (Prof P J Hotez PhD), School of Medicine (Prof J A Singh MD), Baylor College of Medicine, Houston, TX, USA; Rubin Institute for Advanced Orthopedics (A Hoveidaei MD), Sinai Hospital of Baltimore, Baltimore, MD, USA; Institute for Occupational and Maritime Medicine (ZfAM) (H Hoven DrPH), University Medical Center Hamburg-Eppendorf (UKE), Hamburg, Germany; Department of Psychological and Cognitive Sciences (C Hu PhD), School of Clinical Medicine (X Sun PhD), Department of Neurology (X Sun PhD), Tsinghua University, Beijing, China; Faculty of Medicine (J Huang MD), Department of Medicine & Therapeutics (L Shi PhD), School of Public Health and Primary Care (L Yao MSc), Jockey Club School of Public Health and Primary Care (C Zhong PhD), The Chinese University of Hong Kong, Hong Kong, China; Department of Otorhinolaryngology Head and Neck Surgery (W Huang PhD), School of Medicine (Z Li PhD), Shanghai Mental Health Center (Prof M R Phillips MD), Ruijin Hospital (S Xu MPH), Shanghai Jiao Tong University, Shanghai, China; Pediatric Nursing Department (M H Huda PhD), Faculty of Public Health (D Kusuma DSc, Prof I Trihandini PhD), Centre for Family Welfare (K Latief PhD), University of Indonesia, Depok, Indonesia; Department of Public Health and Community Medicine (Prof A Humayun PhD), Shaikh Zayed Postgraduate Medical Institute, Lahore, Pakistan; Department of Humanities (W Husain PhD), COMSATS University Islamabad, Islamabad, Pakistan; Department of Biomolecular Sciences (Prof N R Hussein PhD), University of Zakho, Zakho, Iraq; Artur Riggs Diabetes & Metabolism Research Institute (Prof M I Husseiny PhD), Cancer Prevention and Research Institute, Duarte, CA, USA; International Master Program for Translational Science (H Huynh BS), School of Pharmacy (B Iskandar PhD), School of Nursing (M Kurniasari PhD, I Rohmah MSN, A L Wicaksana MS), Department of Global Health and Health Security (K Latief PhD), International Ph.D. Program in Medicine (M H N Le MD), Research Center for Artificial Intelligence in Medicine (M H N Le MD), Taipei Medical University, Taipei, Taiwan; Department of Occupational Safety and Health (Prof B Hwang PhD), College of Public Health (R Lin PhD), China Medical University, Taiwan, Taichung, Taiwan; Department of Occupational Therapy (Prof B Hwang PhD), Asia University, Taiwan, Taichung, Taiwan; Department of Biomedical, Metabolic, and Neural Science (L F Iannone MD), University of Modena and Reggio Emilia, Modena, Italy; Faculty of Pharmacy (U I Ibrahim PhD), Sultan Zainal Abidin University, Malaysia, Terengganu, Malaysia; Fenerbahce University, Istanbul, Turkiye (I A I A Ibrahim BSc); Department of Cardiovascular Medicine (R Ibrahim MD), Mayo Clinic, Phoenix, AZ, USA; Pharmacoepidemiology Department (F Ida PhD), Sanofi, Cambridge, MA, USA; Health Policy and Management Department (P M Iftikhar MD), City University of New York, New York, NY, USA; Collaborative Alliance Research and Education (CARE) Programme (A Ikiroma PhD), Episcope Research Service, Aberdeen, Scotland; Division of Infectious Diseases (K S Ikuta MD), Veterans Affairs Greater Los Angeles, Los Angeles, CA, USA; West Africa RCC (O S Ilesanmi PhD), Africa Centre for Disease Control and Prevention, Abuja, Nigeria; Faculty of Medicine (I M Ilic PhD, Prof M M Santric-Milicevic PhD, A Stevanović MD), School of Public Health and Health Management (Prof M M Santric-Milicevic PhD), University of Belgrade, Belgrade, Serbia; Faculty of Medical Sciences (Prof M D Ilic PhD), University of Kragujevac, Kragujevac, Serbia; Department of Health Research (L R Inbaraj MD), ICMR National Institute

for Research in Tuberculosis, Chennai, India; Faculty of Health and Life Sciences (A Inok PhD), Living Systems Institute (Y Taheri Abkenar PharmD), University of Exeter, Exeter, UK; Department of Psychology (M Iqbal PhD), Department of Orthopedics (W Jin MD), Department of Epidemiology and Biostatistics (Prof C Yu PhD), Wuhan University, Wuhan, China; Faculty of Public Health (M Iqhrammullah PhD), Universitas Muhammadiyah Aceh (Muhammadiyah University of Aceh), Banda Aceh, Indonesia; Department of Biotechnology (M A Isa PhD), Sharda University, Greater Noida, India; Department of Pharmaceutical Technology (B Iskandar PhD), Sekolah Tinggi Ilmu Farmasi Riau, Pekanbaru, Indonesia; Independent Researcher, Cairo, Egypt (T R Iskander BSc); 4-Green Research Society (D Islam PhD), Journal of Biological Sciences and Public Health, Dhaka, Dhaka, Bangladesh; Department of Surveillance and Health Equity Science (F Islami PhD), American Cancer Society, Atlanta, GA, USA; Department of Clinical Pharmacy & Pharmacy Practice (Prof N E Ismail PhD), Asian Institute of Medicine, Science and Technology, Bedong, Malaysia; Malaysian Academy of Pharmacy, Puchong, Malaysia (Prof N E Ismail PhD); Clinical Laboratory Department (F Ismail PhD), Tobruk University, Tobruk, Libya; Department of Blood Transmitted Diseases (F Ismail PhD), National Centre for Disease Control (NCDC), Tobruk, Libya; Department of Health Services Research (M Iwagami PhD), Department of Public Health Medicine (Prof K Yamagishi MD), University of Tsukuba, Tsukuba, Japan; Knowledge Translation Program (I O Iyamu MD), Centre for Health Evaluation and Outcome Sciences, Vancouver, BC, Canada; Department of Biotechnology (M Iyer PhD), Karpagam Academy of Higher Education, Coimbatore, Coimbatore, India; Department of Health Studies (K H Jacobsen PhD), University of Richmond, Richmond, VA, USA; Department of Nursing (A Jadidi PhD), Arak University of Medical Sciences, Arak, Iran; School of Medicine (M Jafari MD), Volgograd state medical university, Volgograd, Russia; Government Hospitals, Manama, Bahrain (H Jahrami PhD); Department of Health and Safety (A A Jairoun PhD), Dubai Municipality, Dubai, United Arab Emirates; UNESCO-TWAS Section of Economic & Social Sciences, Humanities & Arts (Prof M Jakovljevic PhD), The World Academy of Sciences UNESCO-TWAS, Trieste, Italy; Shaanxi University of Technology, Hanzhong, China (Prof M Jakovljevic PhD); Department of Neurosurgery (M Jalloh MD), Division of Hematology and Oncology (J F Wu MD), Medical College of Wisconsin, Milwaukee, WI, USA; Department of Primary Care Medicine (J Jamaluddin MMed), Universiti Malaya, Kuala Lumpur, Malaysia; SRM Medical College Hospital and Research Centre (J James MD), Sri Ramaswamy Memorial Institute of Science and Technology, Chengelpet, India; Department of Public Health (S Jamil MPH, M Shimul MPH), Department of Development Studies (M Sohel MPH), Daffodil International University, Dhaka, Bangladesh; Department of Public and Community Health (S Jamil MPH), Frontier University Garowe, Puntland, Somalia; Shahrekord University of Medical Science (S JamshidiRastabi MSc), Shahrekord University of Medical Science, Shahrekord, Iran; Department of Stem Cells and Developmental Biology (E Jarrahi MSc, M Piroozkhkah MD), Royan Institution, Tehran, Iran; Health Informatic Lab (T Javaheri PhD), Department of Computer Science (R Rawassizadeh PhD), Boston University, Boston, MA, USA; Department of Medicine (S Javaid MD), University of Mississippi Medical Center, Jackson, MS, USA; Department of Medicine (S Javaid MD), Jinnah Sindh Medical University, Karachi, Pakistan; Department of Biochemistry (Prof S Jayaram MD), Government Medical College, Mysuru, India; Department of Research (Y A Jayasinghe BSc, Prof K K Kanmodi MPH), Faculty of Dentistry (Prof S Selvaraj PhD), University of Puthisastra, Phnom Penh, Cambodia; Postgraduate Institute of Medicine (A U Jayatilleke PhD, Prof S N K Navaratna MD), Department of Anatomy, Genetics and Biomedical Informatics (Y Mathangasinghe PhD), Department of Surgery (D P Wickramasinghe MD), University of Colombo, Colombo, Sri Lanka; Faculty of Graduate Studies (A U Jayatilleke PhD), Institute for Violence and Injury Prevention, Colombo, Sri Lanka; Department of Endocrinology, Diabetes and

Metabolism (Prof F K Jebasingh DM), Department of Nephrology (Prof S Varughese FRCP), Christian Medical College and Hospital (CMC), Vellore, India; Department of Internal Medicine (B M Jeswani MBBS), GCS Medical College, Hospital & Research Centre, Ahmedabad, India; Department of Public Health (Z Ji MD), Tongji University, Shanghai, China; School of Biology and Engineering (School of Health Medicine Modern Industry) (S Jin MPH), Guizhou Medical University, Guiyang, China; Faculty of Veterinary Medicine (M Jokar DVM), Cumming School of Medicine (M I Olatubi PhD), Department of Medicine (Prof M Tonelli MD), University of Calgary, Calgary, AB, Canada; Rothschild Foundation Hospital (Prof J B Jonas MD), Institut Français de Myopie, Paris, France; Singapore Eye Research Institute (Prof J B Jonas MD), Singapore Eye Research Institute, Singapore, Singapore; Hungarian Health Management Association, Budapest, Hungary (T Joo PhD, T Palicz MD); Department of Gastroenterology and Hepatology (A Joseph MD), Management Science and Engineering (Y Ling MS), Department of Biomedical Data Science (S Park MD), Department of Radiology (S Ramasamy MD), Stanford University, Stanford, CA, USA; Department of Economics (C E Joshua BSc), National Open University, Benin City, Nigeria; Department of Family Medicine and Public Health (J J Jozwiak PhD), University of Opole, Opole, Poland; Research Department (Z Kabir PhD), TobaccoFree Research Institute Ireland, Dublin, Ireland; School of Public Health (Z Kabir PhD), University College Cork, Cork, Ireland; Department of Statistics (Prof D H Kadir PhD), Department of Food Technology (B A Sadee PhD), Salahaddin University, Erbil, Iraq; Independent Consultant, Pune, India (P V Kakodkar MDS); Department of Health (K Kalavani PhD), Khoy Medical Sciences, Khoy, Iran; Department of Endocrinology (S Kalra DM), Bharti Hospital Karnal, Karnal, India; University Centre for Research and Development (S Kalra DM), Chandigarh University, Mohali, India; Canberra Business School (M M Kamal MPH), University of Canberra, Hawker, ACT, Australia; College of Pharmacy (M Kamal PhD), Jamia Hamdard, Al Kharj, Saudi Arabia; Care and Public Health Research Institute (CAPHRI) (R Kamath MHA), Maastricht University, Maastricht, Netherlands; Department of Public Health (R T Kamorudeen MPH), South Wales University, Treforest, UK; Osun State Hospital Management Board, (R T Kamorudeen MPH); Department of Biostatistics and Epidemiology (N Kamyari PhD), Abadan University of Medical Sciences, Abadan, Iran; Microbiology, Virology and Immunology Department (Prof O Kamyshnyi DSc), I. Horbachevsky Ternopil National Medical University, Ternopil, Ukraine; Department of Health Sciences (Prof M Kanaan PhD), University of York, York, UK; Office of the Executive Director (Prof K K Kanmodi MPH), Cephas Health Research Initiative Inc, Ibadan, Nigeria; The Hansjörg Wyss Department of Plastic and Reconstructive Surgery (R S Kantar MD), NYU Langone Health, New York, NY, USA; Cleft Lip and Palate Surgery Division (R S Kantar MD), Global Smile Foundation, Norwood, MA, USA; 2nd Department of Cardiology (P Karakasis MD), Department of Ophthalmology (Prof A G P Konstas PhD), First Department of Ophthalmology (Prof G D Panos MD), Aristotle University of Thessaloniki, Thessaloniki, Greece; Department of Public Health and Mortality Studies (S Karun MA), Department of Mathematical Demography & Statistics (R Rawat PhD), International Institute for Population Sciences, Mumbai, India; Amity Stem Cell Institute (ASCI) (Prof M K Kashyap PhD), Amity University Haryana, Gurugram, India; Department of Medicine (A Katamreddy MD), Jacobi Medical Center, New York, NY, USA; Department of Clinical Research and Epidemiology (K Kaushal MD), Institute of Liver and Biliary Sciences, New Delhi, India; Eye Unit (Prof J H Kempen PhD), MyungSung Medical College, Addis Ababa, Ethiopia; Centre for Adolescent Health (J A Kerr PhD), Department of Critical Care and Neurosciences (Prof R G Weintraub MB), Murdoch Childrens Research Institute, Parkville, VIC, Australia; Department of Psychological Medicine (J A Kerr PhD), University of Otago, Christchurch, New Zealand; Department of Human Nutrition (E Kesse-Guyot PhD), National Research Institute for Agriculture, Food and Environment, Jouy-en-Josas, France; Department of Health,

Medicine and Human Biology (M Touvier PhD), Sorbonne Paris Nord University, Bobigny, France (E Kesse-Guyot PhD); Faculty of Medicine (R Khademi MD, F Shahrahmani MD), Department of Medical Genetics and Molecular Medicine (G Khalili-Tanha PhD), Emam-Reza Hospital (S Mohammadpour PhD), Biotechnology Research Center (Prof A Sahebkar PhD), Department of Medical Informatics (S Tabatabaei PhD), Applied Biomedical Research Center (S Tabatabaei PhD), Department of Medicine (A Yarahmadi PhD), Mashhad University of Medical Sciences, Mashhad, Iran; Amity Institute of Forensic Sciences (H Khajuria PhD, B P Nayak PhD), Amity Institute of Pharmacy (K Munjal PhD), Amity institute of Public Health and Hospital Administration (P S Singh PhD, A Singh PhD), Amity University, Noida, India; College of Health Sciences (N Khalid PhD), Abu Dhabi University, Abu Dhabi, United Arab Emirates; Lahore Medical Research Center (S Khalid PhD), Research and Development Department (Prof R Mehboob PhD), Lahore Medical Research Center, Lahore, Pakistan; Faculty of Veterinary Medicine (H O Khalifa PhD), Kafrelsheikh University, Kafrelsheikh, Egypt; Department of Public Health (Prof M khalis PhD), Mohammed VI Center for Research and Innovation, Rabat, Morocco; Higher Institute of Nursing Professions and Health Techniques, Rabat, Morocco (Prof M khalis PhD); Halal Research Center of the Islamic Republic of Iran (IRI) (F Khamesipour PhD), Iran Food and Drug Administration, Tehran, Iran; Center for Atmospheric Particle Studies (CAPS) (M Khan MSc), Department of Mechanical Engineering (MechE) (M Khan MSc), Carnegie Mellon University, Pittsburgh, PA, USA; Department of Cardiology (Z Khan MD, Z Khan MD), University of South Wales, Treforest, UK; Department of Cardiology (Z Khan MD, Z Khan MD), University of Buckingham, Buckingham, UK; NITVAR (A Khan PhD), Indian Council of Medical Research, Pune, India; Academy of Scientific and Innovative Research (AcSIR) (A Khan PhD), Ghaziabad, India; Department of Community Medicine (M Khan MPH), National Institute of Preventive and Social Medicine, Dhaka, Bangladesh; International Center for Chemical and Biological Sciences (S Khan PhD), International Center for Chemical and Biological Sciences, Karachi, Pakistan; Department of Community and Preventive Medicine (R Khan MD), King Edward Medical University, Lahore, Pakistan; BDStatistics Center for Research, Dhaka, Bangladesh (M Khan MPH); Karachi Medical and Dental College, Karachi, Pakistan (M Khan MBBS); Internal Medicine Department (I Khan MD), Reading Hospital Tower Health, Reading, PA, USA; Central Department of zoology (S Khanal MSc), Department of Community Medicine and Public Health (P M S Pradhan MD), Tribhuvan University, Kathmandu, Nepal; College of Health, Wellbeing and Life Sciences (Prof K Khatab PhD), Sheffield Hallam University, Sheffield, UK; College of Arts and Sciences (Prof K Khatab PhD), Ohio University, Zanesville, OH, USA; Department of Orthopaedics (K Khatri MS), Postgraduate Medical Institute, Sangrur, India; University of Sulaimani College of Medicine (N S H S Khoshnaw PhD), Sulaimani Polytechnic University, Sulaymaniyah, Iraq; Department of Internal Medicine (A A Khosla MD), Corewell Health East William Beaumont University Hospital, Royal Oak, MI, USA; Department of Medical Oncology (A A Khosla MD), Miami Cancer Institute, Miami, FL, USA; Department of Clinical Research (S Khosravi MD), Icahn School of Medicine at Mount Sinai, New York City, NY, USA; Research Department (M Khosrowjerdi PhD), University of Inland Norway, Elverum, Norway; Department of Public Health (J Khubchandani PhD, S Roy MD), New Mexico State University, Las Cruces, NM, USA; Cardiovascular Disease Initiative (M Kim MD), Broad Institute of MIT and Harvard, Cambridge, MA, USA; Department of Biomedical Sciences (H Kim MS), Department of Psychiatry (W Myung PhD), Seoul National University, Seoul, South Korea; Health and Healing Research, Education, and Service, Inc., Boston, MA, USA (R W Kimokoti MD); Millennium Prevention, Inc., Westwood, MA, USA (R W Kimokoti MD); The Pacific Community, Noumea, New Caledonia (Prof Y Kinfu PhD); School of Health Sciences (Prof A Kisa PhD), Kristiania University College, Oslo, Norway; Department of International Health and Sustainable Development (Prof A Kisa PhD), Tulane University,

New Orleans, LA, USA (E Lytvyak MD); Department of Nursing and Health Promotion (S Kisa PhD), Faculty of Health Sciences (Prof A W Wolf PhD), Oslo Metropolitan University, Oslo, Norway; School of Pharmacy and Emerging Sciences (L Kishore PhD), Baddi University of Emerging Sciences & Technology, Himachal Pradesh, India; Child Health Analytics (J B Kiss Mres), The Kids Research Institute Australia, Nedlands, WA, Australia; Department of Public Health Dentistry (Prof S KM PhD), Krishna Vishwa Vidyapeeth (Deemed to be University), Karad, India; Endocrinology Department (Prof N Kobyliak DSc), Bogomolets National Medical University, Kyiv, Ukraine; Scientific Department (Prof N Kobyliak DSc), Medical Laboratory CSD, Kyiv, Ukraine; Global Healthcare Consulting, New Delhi, India (S Kochhar MD); Mycobacteriology Unit (D Kolieghu Tcheumeni MSc), Center for Health Promotion and Research, Bamenda, Cameroon; Copernicus Institute of Sustainable Development (G Koren PhD), Utrecht University, Utrecht, Netherlands; Department of Science and Environmental Studies (T Kormoker PhD), The Education University of Hong Kong, Hong Kong, China; Department of General Practice and Family Medicine (Prof O Korzh DSc), Department of Infectious Diseases (Prof A Sokhan PhD), Kharkiv National Medical University, Kharkiv, Ukraine; Department of Epidemiology (Prof K Kostev PhD), IQVIA, Frankfurt am Main, Germany; University Hospital Marburg, Marburg, Germany (Prof K Kostev PhD); Amity institute of Public Health and Hospital administration (A Koul MHA), Amity University Noida, Noida, India; Department of Public Health (J Kretchy PhD), Central University, Accra, Ghana (J Kretchy PhD); Department of Anthropology (Prof K Krishan PhD), Institute of Forensic Science & Criminology (V Sharma PhD), Panjab University, Chandigarh, India; School of Applied Science (C Kua PhD), Republic Polytechnic, Singapore, Singapore; centre for biotechnology (A Kuanar PhD), Centre for Biotechnology (S Patel PhD, P Verma PhD), Siksha 'O' Anusandhan Deemed to be University, Bhubaneswar, India; Department of Demography (Prof B Kuate Defo PhD), Department of Social and Preventive Medicine (Prof B Kuate Defo PhD), University of Montreal, Montreal, QC, Canada; Department of Pediatrics (I Kuitunen PhD), Kuopio University Hospital, Kuopio, Finland; Institute of Clinical Medicine (I Kuitunen PhD), University of Eastern Finland, Kuopio, Finland; College of Medicine (S Kukreti PhD), National Cheng Kung University, Tainan, Taiwan; Center of Medicine and Public Health (M Kulimbet MSc), Asfendiyarov Kazakh National Medical University, Almaty, Kazakhstan; Department of Medicine (V Kulkarni MS), Digital Health and Informatics Directorate (Prof S M McPhail PhD), Queensland Health, Brisbane, QLD, Australia; Amity Centre for Water Studies and Research (S Kulshreshtha PhD), Amity Institute of Biotechnology (E Upadhyay PhD), Amity University Rajasthan, Jaipur, India; Gastroenterology Department (S Kumar FCPS), Ahalia Hospital, Abu Dhabi, United Arab Emirates; Allied Health Sciences (S Kumar FCPS), Bahria University Medical and Dental College, Karachi, Pakistan; Department of Psychiatry (M Kumar PhD), Population Studies and Research Institute (A W Wamai MSc), School of Nursing Sciences (M N Wanjau PhD), University of Nairobi, Nairobi, Kenya; Department of Community Medicine (D Kumar MD), Rajendra Institute of Medical Sciences, Ranchi, India; Department of Anaesthesiology (T Kumar DNB), Rajendra Institute of Medical Sciences, Ranchi, Ranchi, India; Department of Economics (V Kumar PhD), Manipal University, Jaipur, Jaipur, India; Department of Community Medicine (A Kumar MD), Jawaharlal Institute of Postgraduate Medical Education and Research, Karaikal, India; Section of Cardiology (Prof S K Kunutsor PhD), University of Manitoba, Winnipeg, MB, Canada; Translational Health Sciences (Prof S K Kunutsor PhD), Bristol Medical School, Population Health Sciences (Y Nartey PhD), University of Bristol, Bristol, UK; Faculty of Medicine and Health Science (M Kurniasari PhD), Universitas Kristen Satya Wacana (Satya Wacana Christian University), Salatiga, Indonesia; National Research and Innovation Agency (BRIN), Jakarta, Indonesia (A Kusnali MA); Institute for Health Sciences (C Y Kustanti PhD), STIKES Bethesda Yakkum Yogyakarta Indonesia, Yogyakarta, Indonesia; Department of Public Health and Epidemiology (D

Kusuma DSc), Khalifa University of Science and Technology, Abu Dhabi, United Arab Emirates; Department of Pediatric Oncology (Prof T Kutluk MD), Medicana Health International, Istanbul, Turkiye; Department of Pediatric Oncology (Prof T Kutluk MD), Hacettepe University, Ankara, Turkiye; Department of Environment and Public Health (F Kyei-Arthur PhD), University of Environment and Sustainable Development, Somanya, Ghana; Clinical Research Center (V Kytö MD), Turku University Hospital, Turku, Finland; Department of Medicine and Surgery (A La Vecchia MD), University of Milano - Bicocca, Milan, Italy; Pediatric Emergency Department (A La Vecchia MD), Fondazione IRCCS Ospedale Maggiore Policlinico, Milan, Italy; Department of Medicine (A Lachi PhD), UniCamillus University, Rome, Italy; Division of Evidence Synthesis (C Lahariya MD), Foundation for People-centric Health Systems, New Delhi, India; Division of Lifestyle Medicine (C Lahariya MD), Centre for Health: The Specialty Practice, New Delhi, India; School of Digital Science (D Lai PhD), Institute of Applied Data Analytics (D Lai PhD), Faculty of Science (E Leong PhD), Universiti Brunei Darussalam (University of Brunei Darussalam), Bandar Seri Begawan, Brunei; Department of Chemistry (Prof A Lakhani PhD), Dayalbagh Educational Institute, Agra, India; Indian Council of Medical Research, New Delhi, India (D K Lal MD); Unidad de Genética y Salud Pública (Prof I Landires MD), Instituto de Ciencias Médicas, Las Tablas, Panama; Ministry of Health (Prof I Landires MD), Hospital Joaquín Pablo Franco Sayas, Las Tablas, Panama; Department of Psychiatry and Psychotherapy (B Langguth PhD), University of Regensburg, Regensburg, Germany; Department of Behavioural Sciences and Learning (Prof A Laplante-Lévesque PhD), Linköping University, Linköping, Sweden; Health Systems, Administration and Management (S A Lawal PhD), Babcock University, Sagamu, Nigeria; Health Services Management Programme (S A Lawal PhD), Plasma University, Mogadishu, Somalia; School of Physical Therapy (A Lawan PhD), The University of Western Ontario, London, Ontario (ON), Canada; Faculty of Medicine (H Le MD, N Le MD), Department of General Medicine (V T Nguyen MD), Department of Internal Medicine (T H Tran MD), University of Medicine and Pharmacy at Ho Chi Minh City, Ho Chi Minh City, Viet Nam (T D T Le MD, T T T Le MD); Independent Consultant, Ho Chi Minh City, Viet Nam (T D T Le MD); Department of Cardiovascular Research (H Le MD, N Le MD), Methodist Hospital, Merrillville, IN, USA; Department of Family Medicine (W Lee PhD), University of Texas Medical Branch, Galveston, TX, USA; STEM (Prof I Lee PhD), University of South Australia, Adelaide, SA, Australia; Department of Precision Medicine (Prof S Lee MD), Department of Integrative Biotechnology (N Pham MS), Sungkyunkwan University, Suwon, South Korea; Department of Cardiothoracic and Vascular Surgery (V Leivaditis PhD), Westpfalz Klinikum, Kaiserslautern, Germany; Centre for Healthy Brain Ageing (M J Lennon PhD), University of New South Wales, Sydney, New South Wales (NSW), Australia; SC Neurologia, Salute Pubblica e Disabilità (Neurology, Public Health, Disability Unit) (M Leonardi MD), UO Neurologia, Salute Pubblica e Disabilità (The Neurology, Public Health and Disability Unit) (A Raggi PhD), Fondazione IRCCS Istituto Neurologico Carlo Besta (IRCCS Foundation Carlo Besta Neurological Institute), Milan, Italy; Center for Dentistry and Oral Hygiene (A Li PhD), Department of Internal Medicine (P Vart PhD), University of Groningen, Groningen, Netherlands; Stomatological Hospital (A Li PhD), Southern Medical University, Guangzhou, China; Shanxi Medical University, Taiyuan, China (J Li MD); Department of Rheumatology and Immunology (H Li MD), The People's Hospital of Baoan Shenzhen, Shenzhen, China; Department of Endocrinology and Metabolism (Prof Y Li PhD), The First Hospital of China Medical University, Shenyang, China; School of Public Health (Prof J Li PhD), Xuzhou medical university, Xuzhou, China; Department of Health Promotion and Health Education (M Li PhD), National Taiwan Normal University, Taipei, Taiwan; First Clincal Medicine (W Li BSc), Shandong University of Traditional Chinese Medicine, Jinan, China; Nutrition & Health Innovation Research Institute (C Li MPH), Edith Cowan University, Perth, WA, Australia; The First Affiliated Hospital

of Guangzhou Medical University (W Li MD), Guangzhou Medical University, Guangzhou, China; Discipline of Physiology (Y Lian MA), National University of Ireland - Galway, Galway, Ireland (D Shan MD); First Clinical Medical College (X Liang DrPH), Shandong University of Chinese Medicine, Jinan, China; Asbestos Diseases Research Institute, Concord, NSW, Australia (R Lin PhD); Department of Food Science and Human Nutrition (Q Lin MPH), Iowa State University, Ames, IA, USA; The Center for Drug Safety and Policy Research (S Lin PhD), Xi'an Jiaotong University, Xi'an, China; Department of Medicine (D Lindholm MD), Norrtälje Hospital (Tiohundra), Norrtälje, Sweden; Department of Radiology and Biomedical Imaging (X Liu PhD, M Mayeli MD, S Rahmani MD), Department of Genetics (S Pawar PhD), Department of Psychiatry (T Rhee PhD), Yale University, New Haven, CT, USA; College of Mathematics and Computer (Prof Z Liu PhD), Xinyu University, Xinyu, China; Department of Molecular Epidemiology (E Llanaj PhD), German Institute of Human Nutrition Potsdam-Rehbrücke, Potsdam, Germany; German Center for Diabetes Research (DZD), München-Neuherberg, Germany (E Llanaj PhD); Department of Infectious Diseases (M J Loftus MBBS), Alfred Health, Melbourne, VIC, Australia; Department of Cardiology (V Lohner PhD), University of Cologne, Cologne, Germany; School of Medicine (J López-Gil PhD), Universidad Espíritu Santo, Samborondón, Ecuador (J Sharifi Rad PhD); Vicerrectoría de Investigación y Postgrado (J López-Gil PhD), Universidad de Los Lagos, Osorno, Chile; Ashok & Rita Patel Institute of Physiotherapy (S D Lourembam PhD, D M Paija MPT, H P Patel PhD, S Sunny PhD), Department of Physiotherapy (H M Patel PhD), Charotar University of Science and Technology, Anand, India; School of Medicine (Prof R Lozano MD), National Autonomous University of Mexico, Mexico City, Mexico; Department of Spine Surgery (S Luan MD), Qingdao Municipal Hospital Group, Qingdao, China; Scientific Research and Surveillance Systems (J Lubinda PhD), Macha Research Trust, Choma, Zambia; School of Medicine (Prof G Lucchetti PhD), Federal University of Juiz de Fora, Juiz de Fora, Brazil; The Third Department of Hepatic Surgery (S Luo PhD), Eastern Hepatobiliary Surgery Hospital, Shanghai, China; Department of Population Health Sciences (J B Lusk MD), Duke University, Durham, NC, USA; Dodoma Medical Research Centre (A M Lutambi PhD), National Institute for Medical Research in Tanzania, Dodoma, Tanzania; College of Engineering (Prof M D Lytras PhD), Effat University, Jeddah, Saudi Arabia; Management of Information Systems Department (Prof M D Lytras PhD), The American College of Greece, Aghia Paraskevi, Greece; Centre for Public Health and Wellbeing (Z Ma PhD), University of the West of England, Bristol, UK; Department of Microbiology and Parasitology (M Mabrok PhD), King Salman International University, South of Sinai, Egypt; 2nd Department of Propaedeutic Surgery (N Machairas PhD), Department of Biophysics (Prof P Papadopoulou PhD), 3rd Department of Cardiology (M Spartalis PhD), University of Athens, Athens, Greece; Department of Periodontology (Prof M Machoy PhD), Department of Propedeutics of Internal Diseases & Arterial Hypertension (Prof T Miazgowski MD), Pomeranian Medical University, Szczecin, Poland; Associate Laboratory i4HB (A M Madureira-Carvalho PhD), University Institute of Health Sciences - CESPU, Gandra, Portugal; UCIBIO Research Unit on Applied Molecular Biosciences (A M Madureira-Carvalho PhD), University Institute of Health Sciences, Gandra, Portugal; School of Infection & Immunity (Prof P Maffia PhD), School of Cardiovascular and Metabolic Health (F E Petermann-Rocha PhD), University of Glasgow, Glasgow, UK; Department of Pharmacy (Prof P Maffia PhD), University of Naples Federico II, Naples, Italy; Department of Forensic Medicine & Toxicology (D Mahadeshwara Prasad MD), Mysore Medical College & Research Institute, Mysooru, India; Department of Health & Family Welfare (D Mahadeshwara Prasad MD), Government of Karnataka, Bangalore, India; Department of Emergency Medicine (S Mahalingam MD), Sri Lakshmi Narayana Institute of Medical Science, Puducherry, Pondicherry, India; Microbiology Department (P Maikanti-Charalampous MD), Nicosia General Hospital, Nicosia, Cyprus; Department of

Pharmacology (Prof R Maiti MD, A Mishra DM), Department of Psychiatry (A Parmar DM), All India Institute of Medical Sciences, Bhubaneswar, India; Department of Public Health (M Majdan PhD, J Pekarcikova PhD), Trnava University, Trnava, Slovakia; University of Kansas Medical Center (M Mangdow MSc), A.T. Still University, Kansas City, KS, USA; College of Medicine and Health Sciences (E Manirambona MD, Prof C M Muvunyi PhD), University of Rwanda, Kigali, Rwanda; Internal Medicine Department (Y Manla MD), Eisenhower Health, Palm Desert, CA, USA; International Center for Chemical and Biological Sciences (F Mansoor MS, S Ullah MSc), H.E.J. Research Institute of Chemistry (M Shahbaz MPH), University of Karachi, Karachi, Pakistan; Biomedical Engineering Research Center (CREB) (Prof M Mansourian PhD), Automatic Control Department (H Marateb PhD), Universitat Politècnica de Catalunya (Barcelona Tech - UPC), Barcelona, Spain; Laboratory of Public Health (Prof L G Mantovani DSc), IRCCS Istituto Auxologico Italiano, Milan, Italy; Department of Biomedical Engineering (H Marateb PhD, M Noroozi BSc), University of Isfahan, Isfahan, Iran; Far Eastern University, Manila, Philippines (J C Maravilla PhD); Department of Food, Environmental and Nutritional Sciences (M Marino PhD, Prof S Perna PhD), Department of Biomedical Sciences for Health (S Villa MD), University of Milan, Milano, Italy; Faculty of Human Kinetics (Prof A Marques PhD), University of Lisbon, Lisbon, Portugal; Department of Economics (Prof G Martinez PhD), Instituto Tecnologico Autonomo de Mexico, Mexico City, Mexico; Department of Infectious Diseases (B A Martinez-Guerra MSc), Instituto Nacional de Nutrición Salvador Zubirán, Mexico City, Mexico; Department of Non-communicable Diseases and Mental Health (R Martinez-Piedra BSc), Department of Evidence and Intelligence for Action in Health (O J Mujica MD), Pan American Health Organization, Washington, DC, USA; Campus Fortaleza (F R Martins-Melo PhD), Federal Institute of Education, Science and Technology of Ceará, Fortaleza, Brazil; Department of Nutrition and Dietetics (M Martorell PhD), Centre for Healthy Living (M Martorell PhD), University of Concepción, Concepción, Chile; Faculty of Humanities and Health Sciences (Prof R R Marzo MD), Curtin University, Sarawak, Malaysia; Jeffrey Cheah School of Medicine and Health Sciences (Prof R R Marzo MD), School of Pharmacy (Y Wong PhD), Monash University, Subang Jaya, Malaysia; Department of Nursing (S Mashudi PhD), Muhammadiyah University of Surakarta, Ponorogo, Indonesia; Board of Directors (C N Matei PhD), Association of Resident Physicians, Bucharest, Romania; Department of Anatomy and Developmental Biology (Y Mathangasinghe PhD), Monash University, Clayton, VIC, Australia; North West Lung Centre (A G Mathioudakis PhD), Manchester University NHS Foundation Trust, Manchester, UK; Department of Community Medicine (M Mathur MD), Geetanjali Medical College and Hospital, Udaipur, India; Department of Community Medicine (N Mathur MD), Apollo Institute of Medical Sciences and Research, Hyderabad, India; Department of Epidemiology (Prof R J Maude PhD), Mahidol-Oxford Tropical Medicine Research Unit, Bangkok, Thailand; Research Division (Prof P K Maulik PhD), The George Institute for Global Health, New Delhi, India; Nuffield Department of Population Health (M Mazidi PhD), University of Oxford, London, UK; National Centre for Register-based Research (Prof J J McGrath MD), Aarhus University, Aarhus, Denmark; Australian Centre for Health Services Innovation (Prof S M McPhail PhD), Queensland University of Technology, Kelvin Grove, QLD, Australia; Department of Healthcare (Prof E A Mechili PhD), University of Vlora, Vlora City, Albania; Clinic of Social and Family Medicine (Prof E A Mechili PhD), University of Crete, Heraklion, Greece; National Heart, Lung and Blood Institute (Prof R Mehboob PhD), National Heart, Lung, and Blood Institute, Bethesda, MD, USA; Centre for Health Innovation and Policy, Noida, India (Prof R Mehrotra PhD); Department of Dental Research Cell (Prof V Mehta PhD), Dr. D. Y. Patil University, Pune, India; École de Santé Publique (B A Mekonnen MPH), Université libre de Bruxelles (ULB), Brussels, Belgium; Dirección General de Investigación, Desarrollo e Innovación (DGIDI) (W Mendoza MD), Universidad Científica del

Sur (University of the South), Lima, Peru; Department of Medical Microbiology and Immunology (G A Menezes PhD), Trinity Medical Sciences University, St. Vincent, Saint Vincent and the Grenadines; Department of Medicine (G A Mensah MD, Prof L J Zuhlke PhD), Technical Department (C A Nnaji PhD), School of Public Health and Family Medicine (C A Nnaji PhD), Division of Cardiology (Prof M Ntsekhe PhD), Department of Paediatrics and Child Health (Prof L J Zuhlke PhD), University of Cape Town, Cape Town, South Africa; Department of Public Health (M Mercogliano MD), University "Federico II" of Naples, Naples, Italy; Comprehensive Cancer Center (T J Meretoja MD), General Administration Department (A Meretoja MD), Department of Neurosurgery (I Rautalin PhD), Helsinki University Hospital, Helsinki, Finland; University Centre Varazdin (T Mestrovic PhD), University North, Varazdin, Croatia; Faculty of Veterinary Medicine (Prof M M M Metwally PhD), King Salman International University, Ras Sedr, Egypt; Department of Pathology (I Michalek PhD), Maria Sklodowska-Curie National Research Institute of Oncology, Warsaw, Poland; Dermatology Unit (A Michelerio PhD), Fondazione IRCCS Policlinico San Matteo, Pavia, Italy; Department of Oncology (H S Mideksa MD), Addis Ababa University, Addis Abeba, Ethiopia; College of Human Medicine (T R Miller PhD), Michigan State University, Flint, MI, USA; Multidisciplinary Department of Medical-Surgical and Dental Specialties (G Minervini PhD), University of Campania Luigi Vanvitelli, Naples, Italy; Global Institute of Public Health (Prof G Mini PhD), Ananthapuri Hospitals and Research Institute, Trivandrum, India; Department of Radiology (S Mirshahvalad MD), Health Sciences North, Sudbury, ON, Canada; Bergen Center for Ethics and Priority Setting (M K Mirutse PhD), Department of Psychosocial Science (Prof D Sagoe PhD), Center for International Health (C Schwinger PhD), University of Bergen, Bergen, Norway; National Data Management Center for Health (A Misganaw PhD), Ethiopian Public Health Institute, Addis Ababa, Ethiopia; Department of Forensic Medicine and Toxicology (C Mittal MD), All India Institute of Medical Sciences, Patna, India; Department of Internal Medicine (S Modi MD), Albert Einstein Hospital, Philadelphia, PA, USA; Molecular Biology Unit (N S Mohamed MSc), Bio-Statistical and Molecular Biology Department (N S Mohamed MSc), Sirius Training and Research Centre, Khartoum, Sudan; Nursing College (M Mohamed PhD), Sohag University, Sohag, Egypt; College of Applied and Natural Science (J Mohamed MSc), University of Hargeisa, Hargeisa, Somalia; Centre for Interdisciplinary Research in Basic Sciences (T Mohammad PhD), Jamia Millia Islamia, New Delhi, Delhi, India; Health Economics Division (S Mohammadpour PhD), Ministry of Health and Medical Education, Mashhad, Iran; Heidelberg Institute of Global Health (HIGH) (Prof S Mohammed PhD), Department of Ophthalmology (S Panda-Jonas MD), Heidelberg University, Heidelberg, Germany; Medical Microbiology Department (Prof Y Mohammed FWACP), Usmanu Danfodiyo University Teaching Hospital, Sokoto, Nigeria; Department of Public Health (H Mohammed PhD, A Oumer PhD), Dire Dawa University, Dire Dawa, Ethiopia; University of Gondar (A Mohammed MSc), university of Gondar, Gondar, Ethiopia; Department of Medicine (O Mohammed MBBS), Government Medical College Kozhikode, Kozhikode, India; Department of health sciences azare Bauchi State -Nigeria (S Mohammed MSc), National Institute for Research in Tribal Health, Bauchi, Nigeria; Department of Health Services Management (M Mohseni PhD), Iran University of Medical Sciences, Iran, Iran; Institute of Clinical Physiology (S Molinaro PhD), National Research Council, Pisa, Italy; Department Medical-Surgical Nursing (A Mollaei PhD), Golestan University of Medical Sciences, Gorgan, Iran; Clinical Epidemiology and Public Health Research Unit (L Monasta DSc, L Ronfani PhD, G Zamagni MSc), Burlo Garofolo Institute for Maternal and Child Health, Trieste, Italy; Department of Biomedical and Dental Sciences and Morphofunctional Imaging (Prof S Mondello MD), Messina University, Messina, Italy; AI & Cyber Futures Institute (M Moni PhD), Charles Sturt University, Bathurst, NSW, Australia; Department of Collective Prevention and Public Health (M Montalti MD), General

Directorate for Personal Care, Health, and Welfare, Bologna, Italy; Department of Public Health (Prof R S Moreira PhD), Oswaldo Cruz Foundation, Recife, Brazil; Department of Public Health (Prof R S Moreira PhD), Federal University of Pernambuco, Recife, Brazil; Division of Plastic and Reconstructive Surgery (S D Morrison MD), University of Washington Medical Center, Seattle, WA, USA; Faculty of Medicine (M Morsy MD), October 6 University, Giza, Egypt; Department of Health Policy (Prof E Mossialos PhD), London School of Economics and Political Science, London, UK; Social Determinants of Health Research Center (S Mouodi PhD), School of Medicine (S Soraneh MD), Babol University of Medical Sciences, Babol, Iran; Department for Statistics and Econometrics (M Mourgova PhD), University of National and World Economy, Sofia, Bulgaria; Faculty of Biotechnologies (BioTech) (Prof A Mousavi Khaneghah PhD), ITMO University, Saint Petersburg, Russia; Department of Physical and Environmental Sciences (S Mousavi Kiasary DVM-MPH), Texas A&M University, Corpus Christi, TX, USA; Shiraz University of Medical Sciences, Shiraz, Shiraz, Iran (S Mousavi Kiasary DVM-MPH); René Rachou Institute (M Mrejen PhD), Oswaldo Cruz Foundation, Belo Horizonte, Brazil; Arid Agriculture University Rawalpindi, Pakistan (R Mubarak MSc), PMAS Arid Agriculture University Rawalpindi, Pakistan, Rawalpindi, Pakistan; School of Medicine (F Mughal FRCGP), School of Medicine (T Oyelade PhD), Keele University, Keele, UK; Institute of Molecular Biology and Biotechnology (S Muhammad PhD), Bahauddin Zakariya University Multan, Multan, Pakistan; Knowledge Management Department (S Mukherjee PhD), Prahlad Omkarwati Foundation (POF), Mumbai, India; Changescape Consulting (S Mukherjee PhD), Independent Consultant, New Delhi, India (P Sood PhD); Department of Biochemistry (S Mukherjee PhD), Department of Dentistry (Prof A Singh MDS), All India Institute of Medical Sciences, Bhopal, India; Department of Medicine (A Mukhopadhyay MD), National University Health System, Singapore, Singapore; Department of Surgery (G D Mukoro MD), Ahmadu Bello University Teaching Hospital, Zaria, Nigeria; Department of Mechanical Engineering (M Muktadir PhD), North Carolina Agricultural and Technical State University, Greensboro, NC, USA; Department of Surgery (F Mulita PhD), General University Hospital of Patras, Patras, Greece; Faculty of Medicine (F Mulita PhD), Department of Emergency Medicine (Prof I Pantazopoulos PhD), University of Thessaly, Larissa, Greece; College of Health Science (C Mulugeta MSc), Woldia University, woldia, Ethiopia; Department of Nursing (M Mulyadi PhD), Sam Ratulangi University, Manado, Indonesia; Department of Health Economics (M Muniyandi PhD), Department of Epidemiology (M Sathya Narayanan MBBS), National Institute for Research in Tuberculosis, Chennai, India; Department of Community and Global Health (Y Munkhsaikhan MD), The University of Tokyo, Tokyo, Japan; Epidemiology, Biostatistics and Prevention Institute (EBPI) (J Muñoz Laguna MSc), University of Zürich, Zurich, Switzerland; Center for Infectious Disease Education and Research (Prof M Murakami PhD), Department of Biostatistics and Data Science (Y Yasufuku MSc), The University of Osaka, Suita, Japan; Clinical Epidemiology Research Unit (E Murillo-Zamora PhD), Mexican Institute of Social Security, Villa de Alvarez, Mexico; Postgraduate in Medical Sciences (E Murillo-Zamora PhD), Universidad de Colima, Colima, Mexico; Department of Pathology and Microbiology (S I Mustafa PhD), Duhok University, Duhok, Iraq; Operational Research Center in Healthcare (M T Mustapha PhD), Near East University, Nicosia, Cyprus; Department of Research Methods (S Muthu PhD), Orthopaedic Research Group, Coimbatore, India; Central Research Laboratory (S Muthu PhD), Meenakshi Medical College Hospital and Research Institute, Chennai, Tamil Nadu, India; Director General (Prof C M Muvunyi PhD), Rwanda Biomedical Centre, Kigali, Rwanda; Department of Neuropsychiatry (W Myung PhD), Seoul National University Bundang Hospital, Seongnam, South Korea; Research and Analytics Department (A J Nagarajan MTech), Initiative for Financing Health and Human Development, Chennai, India; Department of Research and Analytics (A J Nagarajan MTech), Bioinsilico Technologies, Chennai, India; Department of Computer

Science and IT (G R Naik PhD), Torrens University, Adelaide, SA, Australia; Faculty of Pharmacy (Prof F Nainu PhD), Hasanuddin University, Makassar, Indonesia; Department of Health and Rehabilitation Sciences (Prof G Nambi PhD), Department of Computer and Self Development (A S Zamani PhD), Prince Sattam bin Abdulaziz University, Al Kharj, Saudi Arabia; Suraj Eye Institute, Nagpur, India (V Nangia PhD); Department for the Control of Disease, Epidemics, and Pandemics (J Nansseu MD), Ministry of Public Health, Yaoundé, Cameroon; Department of Public Heath (J Nansseu MD), Department of Public Health (G Nguefack-Tsague PhD), University of Yaoundé I, Yaoundé, Cameroon; National Dental Research Institute Singapore (G G Nascimento PhD), Duke-NUS Medical School, Singapore, Singapore; Department of Applied Pharmaceutical Sciences and Clinical Pharmacy (A Y Naser PhD), Isra University, Amman, Jordan; Division of Endocrinology and Diabetes (M Nassar PhD), University of Vermont, South Burlington, VT, USA; Department of Community Medicine (Prof S N K Navaratna MD), University of Peradeniya, Kandy, Sri Lanka; Xiamen Cardiovascular Hospital of Xiamen University (N PhD), Fujian Branch of National Clinical Research Center for Cardiovascular Diseases, Xiamen, China; Department of Biological Sciences (S Naz PhD, R Ullah PhD), National University of Medical Sciences (NUMS), Rawalpindi, Pakistan; Department of Research (G Nchanji PhD), TroDDIVaT Initiative, Buea, Cameroon; Department of Microbiology and Parasitology (G Nchanji PhD), University of Buea, Buea, Cameroon; Department of Cardiology (R I Negoi PhD), Cardio-Aid, Bucharest, Romania; Department of General Surgery (I Negoi PhD), Emergency University Hospital of Bucharest, Bucharest, Romania; Department of Neurosciences (Prof C R J Newton MD), Kenya Medical Research Institute/Wellcome Trust Research Programme, Kilifi, Kenya; Department of Biological Sciences (J W Ngunjiri PhD), University of Embu, Embu, Kenya; Hitotsubashi Institute for Advanced Study (HIAS) (T Nguyen DrPH), Hitotsubashi University, Tokyo, Japan; Institute for Cancer Control (T Nguyen DrPH), National Cancer Center, Chuo-ku, Japan; Tuberculosis Group (V T Nguyen MD), Oxford University Clinical Research Unit, Vietnam, Ho Chi Minh City, Viet Nam; Harvard T.H. Chan School of Public Health (D Nguyen BS), Department of Orthopaedics (O Subasi PhD), Harvard Kennedy School (K J Uzor MD), Harvard University, Cambridge, MA, USA; Department of Medical Engineering (D Nguyen BS), University of South Florida, Tampa, FL, USA; Faculty of Public Health (L Nguyen PhD, L Vu PhD), International Institute for Training and Research (INSTAR) (L Nguyen PhD, L Vu PhD), VNU University of Medicine and Pharmacy, Hanoi, Viet Nam; Nam Can Tho Health Science Institute (N P Nguyen MD), Nam Can Tho University, Can Tho, Viet Nam; Department of Pediatrics (T Nguyen MD), New York Medical College, New York, NY, USA; Institute for Global Health Innovations (C T Nguyen MPH), Duy Tan University, Hanoi, Viet Nam; Department of Public Health (A M Ngwa MSc), University of Bamenda, Bamenda, Cameroon; International Islamic University Islamabad, Islamabad, Pakistan (R K Niazi PhD); Department of Humanities and Social Science (L Nieddu PhD), University for International Studies in Rome, Rome, Italy; School of Medicine (V Niranjan PhD), University of Limerick, Limerick, Ireland; Department of Public Health (V Niranjan PhD), UNICAF, Larnaca, Cyprus; Department of Pathology (A M Nisro MD), Hawassa University, Hawassa, Ethiopia; Department of Internal Medicine and Specialties (J Nkeck MD), University of Yaoundé I, Yaounde, Cameroon; Global Research Institute (Prof S Nomura PhD), Keio University, Tokyo, Japan; University Institute of Diet and Nutritional Sciences (S Noreen PhD), The University of Lahore, LAHORE, Pakistan; Internal Medicine Department (V C Nriagu MD), Maimonides Medical Center, Brooklyn, NY, USA; Department of Paediatrics (C A Nri-Ezedi PhD), Nnamdi Azikiwe University, Awka, Nigeria; Global Health Department (J Nshimiyimana MPH), Euclid University, Banqui, Central African Republic; The Cardiac Clinic (Prof M Ntsekhe PhD), Groote Schuur Hospital, Cape Town, South Africa; School of Information (F Nugen PhD), University of California Berkeley, Berkeley, CA, USA; Department of Public Health (A N

Nugusa MPH), Mattu University, Mattu, Ethiopia; Midwifery Department (N Nurfatimah MPH), Poltekkes Kemenkes Palu, Palu, Indonesia; Department of Public Health (D Nurrika PhD), Banten School of Health Science, South Tangerang, Indonesia; Ministry of Research, Technology and Higher Education (D Nurrika PhD), Higher Education Service Institutions (LL-DIKTI) Region IV, Bandung, Indonesia; Department of Physiology (O J Nzoputam PhD), University of Benin, Edo, Nigeria; Department of Physiology (O J Nzoputam PhD), Benson Idahosa University, Benin City, Nigeria; Department of Applied Economics and Quantitative Analysis (Prof B Oancea PhD), University of Bucharest, Bucharest, Romania; Bioinformatics Department (Prof B Oancea PhD), National Institute of Research and Development for Biological Sciences, Bucharest, Romania; Department of Biomedicine and Prevention (F Oddi PhD), University of Rome "Tor Vergata", Rome, Italy; Department of Community Health and Primary Care (Prof O O Odukoya MSc), University of Lagos, Idi Araba, Nigeria; Department of Family and Preventive Medicine (Prof O O Odukoya MSc), Department of Biomedical Informatics (D Villarreal-Zegarra MPH), University of Utah, Salt Lake City, UT, USA; PSSM Data Sciences, Pfizer Research & Development (M Oduro PhD), Pfizer Inc., Groton, CT, USA; Department of Physiology (O B Oghenetega PhD), Babcock University, Ilisan-Remo, Nigeria; Technical Unit (O T Ogundeko-Olugbami MSc), Malaria Consortium, London, UK; Department of Preventive Medicine (Prof I Oh MD), University of Ulsan, Seoul, South Korea; Institute for Global Engagement & Empowerment (Prof S Oh PhD), Yonsei University, Seoul, South Korea; Faculty of Medicine (O J Okesanya MPH), University of Thessaly, Volos, Greece; Department of Medical Laboratory Science (O J Okesanya MPH), Federal Neuropsychiatric Hospital, Abeokuta, Nigeria; School of Pharmacy (O C Okonji MSc), University of the Western Cape, Cape Town, South Africa; Department of Nursing Science (M I Olatubi PhD), Bowen University Iwo, Iwo, Nigeria; Center for Clinical and Epidemiological Research (A B Oliveira PhD), Department of Psychiatry (Y Wang PhD), University of São Paulo, São Paulo, Brazil; Associação Brasileira de Cefaleia em Salvas e Enxaqueca (ABRACES), São Paulo, Brazil (A B Oliveira PhD); Cardiology Department (Prof G M M Oliveira PhD), Federal University of Rio de Janeiro, Rio de Janeiro, Brazil; School of Health and Life Sciences (O O Oludoye PhD), Teesside University, Middlesbrough, UK; Research Policy & Administration (J O Olusanya MBA), Executive Director (B O Olusanya PhD), Centre for Healthy Start Initiative, Lagos, Nigeria; Surgery Department (G L Omer MD), Sulaimani University, Sulaimani, Iraq; ENT Department (G L Omer MD), Tor Vergata University of Rome, Rome, Italy; Institute of Diagnostic and Interventional Radiology and Neuroradiology (M Opitz MD), University Hospital Essen, Essen, Germany; Department of Pharmacotherapy and Pharmaceutical Care (M Ordak PhD), Medical University of Warsaw, Warsaw, Poland; Sickle Cell Unit (Prof V N Orish PhD), Ho Teaching Hospital, Ho, Ghana; Department of Biotechnological and Applied Clinical Sciences (R Ornello PhD), University of L'Aquila, L'Aquila, Italy; Department of Neurology (R Ornello PhD), ASL Avezzano-Sulmona-L'Aquila, L'Aquila, Italy; Department of Nephrology and Hypertension (Prof A Ortiz MD), IIS-Fundacion Jimenez Diaz, Madrid, Spain; Department of Medicine (Prof A Ortiz MD), Faculty of Medicine (P Perez-Lopez MD), Autonomous University of Madrid, Madrid, Spain; One Health Global Research Group (Prof E Ortiz-Prado PhD), Universidad de las Americas (University of the Americas), Quito, Ecuador; Department of Biological Sciences (A Osborne MSc), Njala University, Freetown, Sierra Leone; School of Medicine (U L Osuagwu PhD), Western Sydney University, Bathurst, NSW, Australia; Department of Optometry and Vision Science (U L Osuagwu PhD), University of KwaZulu-Natal, KwaZulu-Natal, South Africa; Department of Biological Sciences (O Osuolale PhD), Elizade University, Ilara-Mokin, Nigeria; Department of Preventive and Social Medicine (G Otchere MSc), University of Otago, Dunedin, New Zealand; School of Public Health (O J Otorkpa PhD), Texila American University, Georgetown, Guyana; Division of Infectious Diseases (Prof A Ouyahia PhD), University Hospital of Setif, Sétif, Algeria;

Operational Research Center in Healthcare (I Ozsahin PhD, Prof U Saeed PhD), Near East University, Nicosia, Turkiye; Department of Mathematical Sciences (I Ozsahin PhD), Saveetha School of Engineering, SIMATS, Chennai, India; Department of Respiratory Medicine (Prof M P A DNB), Department of Forensic Medicine and Toxicology (S Rani MD), Department of Oral and Maxillofacial Surgery (C S N PhD), Jagadguru Sri Shivarathreeswara University, Mysore, India; Department of Mental Health (R F Palma-Alvarez PhD), Hospital Universitari Vall d'Hebron (CIBERSAM), Barcelona, Spain; Biomedical Network Research Centre on Mental Health (CIBERSAM), Barcelona, Spain (R F Palma-Alvarez PhD); Primary Health Center (T Paluvai MBBS), Directorate of Public Health and Family Welfare, Eluru district, India; Menzies Institute for Medical Research (F Pan PhD), University of Tasmania, Hobart, TAS, Australia; Centre for Biotechnology (S K Panda PhD), Siksha 'O' Anusandhan (Deemed to be University), Bhubaneswar, India; Division of Research and Development (Prof S R Pandi-Perumal MSc), Lovely Professional University, Phagwara, India; Division of Ophthalmology & Visual Sciences (Prof G D Panos MD), University of Nottingham, Nottingham, UK; Department of Neurology (L D Panos MD), University of Bern, Biel/Biene, Switzerland; Department of Neurology (L D Panos MD), University of Cyprus, Nicosia, Cyprus; Department of Emergency Medicine (Prof I Pantazopoulos PhD), University of Bern, Bern, Switzerland; Unit of Dermatology (G Paolino PhD), IRCCS Ospedale San Raffaele, Milano, Italy; Medical University of Vienna, Vienna, Austria (I Papadimopoulos MD); Department of Science and Mathematics (Prof P Papadopoulou PhD), Deree-The American College of Greece, Athens, Greece; Ottawa Hospital Research Institute, Ottawa, ON, Canada (P Paranjkhoo MD); Division of Health Policy and Management (R R Parikh MD), Department of Surgery (J Rickard MD), University of Minnesota, Minneapolis, MN, USA; Department of Sociology, Anthropology, and Public Health (C Park DrPH), University of Maryland, Baltimore County, Baltimore, MD, USA; Department of Medical Humanities and Social Medicine (Prof E Park PhD), Kosin University, Busan, South Korea; Department of Primary Care and General Practice (S Parve PhD), Kazan State Medical University, Kazan, Russia; Department of Cardiology (S Parve PhD), Parve Nursing Home, Sindkhed Raja, India; Department of Medical Sciences (R Passera PhD), University of Torino, Torino, Italy; Department of Imaging (R Passera PhD), AOU Città della Salute e della Scienza di Torino (AOU City of Health and Science of Turin), Torino, Italy; Faculty of Medicine and Health (J Patel MChD), University of Leeds, Leeds, UK; Research and Development Cell (M Patel PhD), Parul University, Vadodara, India; Department of Research and Training (S K Patel PhD), Population Council Institute, New Delhi, India; Institute of Physiotherapy (B H Patel MPT), Ashok and Rita Patel Institute of Physiotherapy, Anand, India; Roswell Park Comprehensive Cancer Center (R J Patel MD), The State University of New York at Buffalo, Buffalo, NY, USA; Department of Cardiovascular Medicine (N N Patel MD), University of Tennessee, Nashville, TN, USA; School of Medicine (A J Paternina-Caicedo MSc), University of Sinu, Cartagena, Colombia; Mahatma Gandhi Institute of Medical Sciences, Sevagram (B S U Patil MD), Maharashtra University of Health Sciences, Wardha, India; College of Dental Medicine (Prof S Patil PhD), Roseman University of Health Sciences, South Jordan, UT, USA; Department of Internal Medicine (V Patthipati MD), Advent Health, Palm Coast, FL, USA; Department of Hospital Medicine (V Patthipati MD), Sound Physicians, Palm Coast, FL, USA; Department of Interventional Cardiology (S Pawar MD), Cedars Sinai Medical Center, Los Angeles, CA, USA; IRCCS Fondazione Don Carlo Gnocchi, Milan, Italy (P Pedersini MSc); Department of Clinical and Experimental Sciences (P Pedersini MSc), University of Brescia, Brescia, Italy; Australian Institute of Health Innovation (P Peprah MSc), Macquarie University, Sydney, NSW, Australia; Research Institute for Medicines (Prof J Perdigão PhD), Universidade de Lisboa (University of Lisbon), Lisbon, Portugal; School of Population Health (Prof G Pereira PhD), Curtin University, Bentley, WA, Australia; Centre for Fertility and Health (Prof G Pereira PhD), Department of

Chemical Toxicology (M W Wojewodzic PhD), Norwegian Institute of Public Health, Oslo, Norway; Mario Negri Institute for Pharmacological Research, Bergamo, Italy (N Perico MD, Prof G Remuzzi MD); Department of Biochemistry and Pharmacology (P Petakh PhD), Uzhhorod National University, Uzhhorod, Ukraine; Facultad de Medicina (Faculty of Medicine) (F E Petermann-Rocha PhD), Universidad Diego Portales (Diego Portales University), Santiago, Chile; Department of Internal Medicine (H Pham MD), Weiss Memorial Hospital, Chicago, IL, USA; Departments of Psychiatry and Epidemiology (Prof M R Phillips MD), Columbia University, New York, NY, USA (D Shan MD); International Center of Medical Sciences Research (Z Z Piracha PhD), International Center of Medical Sciences Research, Islamabad, Pakistan (Prof U Saeed PhD); Department of Promoting Health, Maternal-Infant, Excellence and Internal and Specialized Medicine (PROMISE) G. D'Alessandro (E Pirera MD), University of Palermo, Palermo, Italy; Air and Climate Unit (E Pisoni PhD), European Commission, Ispra, Italy; Mental Health Research Institute (E Plotnikov PhD), Tomsk National Research Medical Center, Tomsk, Russia; Siberian State Medical University, Tomsk, Russia (E Plotnikov PhD); Department of Data Management and Analysis (R Poluru PhD), The INCLEN Trust International, New Delhi, India; Department of Ortopedics and Traumatology (V T Ponkilainen PhD), University of Tampere, Tampere, Finland; Academy of Romanian Scientists, Bucharest, Romania (Prof I Popa PhD); Department of Internal Medicine (D S Popovic PhD), University of Novi Sad, Novi Sad, Serbia; Clinic for Endocrinology, Diabetes and Metabolic Disorders (D S Popovic PhD), Clinical Center of Vojvodina, Novi Sad, Serbia; Non-communicable Diseases Research Center (N Pourtaheri PhD), Bam University of Medical Sciences, Bam, Iran; Centro de Investigaciones Clinicas (Clinical Research Center) (S I Prada PhD), Fundación Valle del Lili (Valle del Lili Foundation), Cali, Colombia; Centro PROESA (S I Prada PhD), Departamento de Ciencias Básicas Médicas (E Rubagotti PhD), Universidad ICESI, Cali, Colombia; Department of Humanities and Social Sciences (Prof J Pradhan PhD), National Institute of Technology Rourkela, Rourkela, India; Department of Biochemistry (Prof A Prashant PhD), JSS Academy of Higher Education and Research, Mysuru, India; Department of Clinical and Experimental Medicine (Prof N Pugliese PhD, D Trico MD), University of Pisa, Pisa, Italy; Institute for Health Research (S Puthussery DrPH), University of Bedfordshire, Luton, UK; Department of Medical instrumentation Techniques Engineering (N H Qasim DSc), Al-Rafidain University College, Baghdad, Iraq; Department of Cybersecurity (N H Qasim DSc), Kyiv National University of Construction and Architecture, Kyiv, Ukraine; School of Public Health (Prof Z Qi PhD), 徐州医科大学公共卫生学院 (Xuzhou Medical University), Xuzhou, China; Department of Cardiovascular Medicine (J Qiu MD), Guangdong Cardiovascular Institute, Guangdong Provincial People's Hospital, Guangzhou, China; Department of Respiratory and Critical Care Medicine (J Qiu MD), The First Affiliated Hospital, and College of Clinical Medicine of Henan University of Science and Technology, Luoyang, China; Research Center for Public Health and Nutrition (B Rachmat MPH), National Research and Innovation Agency of Indonesia, Jakarta, Indonesia; Oman Dental College, Oman (Prof R A Radhakrishnan PhD); Department of Epidemiology (P Raghuveer MD), National Institute of Mental Health and Neurosciences, Bengaluru, India; Department of Environmental Health Engineering (S Rahimi PhD), Health Science Research Centre (S Rahimi PhD), Torbat Heydariyeh University of Medical Sciences, Torbat Heydariyeh, Iran; Institute of Health and Wellbeing (Prof M Rahman PhD), Federation University Australia, Berwick, VIC, Australia; Department of Epidemiology (M Rahman PhD), Institute of Epidemiology, Disease Control and Research (IEDCR), Dhaka, Bangladesh; Department of Pathobiology and Population Sciences (PPS) (M Rahman PhD), Royal Veterinary College (RVC), London, UK; Faculty of Health Sciences (F M Rahman PhD), Qaiwan International University, Sulaymaniyah, Iraq; College of Science (F M Rahman PhD), University of Sulaimani, Sulaymaniyah, KRG, Iraq; Collaboration for Cancer Outcomes Research and Evaluation

(CCORE) (M Rahman PhD), University of New South Wales, Liverpool, NSW, Australia; School of Medicine and Public Health (M Rahman PhD), University of Sydney, Wollongong, NSW, Australia; College of Medicine and Health Sciences (M Rahman PhD), National University of Science and Technology, Sohar, Oman; Future Technology Research Center (A Rahmani PhD), National Yunlin University of Science and Technology, Yunlin, Taiwan; Health Service Research and Quality of Life Center (CEReSS) (Prof M Rahmati PhD), Aix-Marseille University, Marseille, France; Department of Medical, Surgical and Experimental Sciences (I Raimondo MD), University of Sassari, Sassari, Italy; Gynecology and Breast Care Center (I Raimondo MD), Mater Olbia Hospital, Olbia, Italy; Division of Gynecology and Human Reproduction Physiopathology (D Raimondo PhD), IRCCS Azienda Ospedaliero-Universitaria di Bologna, Bologna, Italy; Dr. Rajendra Prasad Government Medical College, Tanda, Kangra, India (Prof S K Raina MD); Department of Cardiology (A Raja MD), Department of Medicine (S Raja MD), Dow University of Health Sciences, Karachi, Pakistan; Department of Population Health (M Ramadan DrPH), King Saud bin Abdulaziz University for Health Sciences, Jeddah, Saudi Arabia; Department of Midwifery (K Ramadhan MPH), Ministry of Health of the Republic of Indonesia, Palu, Indonesia; Department of Anatomy (C Ramasamy MD), Govt. Siddhartha Medical College, Vijayawada, India; Biological Science and Bioengineering (M Ramezani Farani PhD), Inha University, Incheon, South Korea; South Asian Institute for Social Transformation (SAIST), Dhaka, Bangladesh (J Rana MPH); Department of Epidemiology, Biostatistics and Occupational Health (J Rana MPH), McGill University, Montreal, QC, Canada; Department of Research (C L Ranabhat PhD), Eastern Scientific LLC, Richmond, KY, USA; Planetary Health Research Centre (PHRC), Kathmandu, Nepal (C L Ranabhat PhD); Centre for Clinical Pharmacology (N Rancic PhD), University of Defence in Belgrade, Belgrade, Serbia; Centre for Clinical Pharmacology (N Rancic PhD), Medical College of Georgia at Augusta University, Belgrade, Serbia; Department of Oral Medicine and Radiology (K Rao PhD), Nitte (deemed to be) University, Mangalore, India; Barcelona Institute for Global Health, Barcelona, Spain (Prof D Rasella PhD); Iranian Research Center on Aging (V Rashedi PhD), University of Social Welfare and Rehabilitation Sciences, Tehran, Iran; Department of Geography (A Rasul PhD), Soran University, Soran, Iraq; Department of Family Medicine (Prof D Rathish PhD), Department of Parasitology (Prof K G Weerakoon PhD), Department of Community Medicine (N D Wickramasinghe MD), Rajarata University of Sri Lanka, Anuradhapura, Sri Lanka; University of Swabi (A Rauf PhD), University of swabi, Swabi, Pakistan; Department of Psychiatry (D Ravi MBBS), Department of Medicine (P S MBBS), St. John's National Academy of Health Sciences, Bangalore, India; Inovus Medical, St Helens, UK (D L Rawaf MD); Department of Hematology (B Razi PhD), North Khorasan University of Medical Sciences, Bojnurd, Iran; Department of Internal Medicine (M M R K Reddy MD), Northwest Health, Porter, Valparaiso, IN, USA; Department of Biological Sciences (Prof E Redwan PhD), King Abdulaziz University, Jeddah, Egypt; Department of Protein Research (Prof E Redwan PhD), Research and Academic Institution, Alexandria, Egypt; The School of Pharmaceutical Sciences (W Rehman MS), University of Science Malaysia, Penang, Malaysia; Department for Epidemiology and Biostatistics (R Reile PhD), National Institute for Health Development, Tallinn, Estonia; Department of Obstetrics and Gynecology (S Restaino MD), Azienda Sanitaria Universitaria Friuli Centrale, Udine, Italy; School of Environment (M Rezaei PhD), Tehran University, Tehran, Iran; Department of Public Health Sciences (T Rhee PhD), University of Connecticut, Farmington, CT, USA; Department of Surgery (J Rickard MD), University Teaching Hospital of Kigali, Kigali, Rwanda; Community Health Department (Prof H A L Rocha PhD), Federal University of Ceará, Fortaleza, Brazil; Faculty of Medicine (A Rodriguez-Morales DSc), Fundacion Universitaria Autonoma de las Americas, Pereira, Colombia; Department of Clinical Research (Prof L Roever PhD), University of Sao Paulo, Ribeirão Preto, Brazil; Gilbert and Rose-Marie Chagoury

School of Medicine (Prof L Roever PhD), Lebanese American University, Beirut, Lebanon; Department of Community Medicine (R Rohilla MD), Government Medical College, Chandigarh, India; Department of Environmental and Radiological Health Sciences (Prof D Rojas-Rueda PhD), Colorado State University, Fort Collins, CO, USA; Department of Anesthesiology (M L Rolfzen MD), University of Nebraska Medical Center, Omaha, NE, USA; Department of Neurosciences (M Romoli MD), Maurizio Bufalini Hospital, Cesena, Italy; Action Research Bangladesh, Dhaka, Bangladesh (M Rony MPH); Department of Ophthalmology and Visual Sciences (A Roshanshad MD), University of Wisconsin-Madison, Madison, WI, USA; School of Medicine (M Rostamian PhD), Faculty of Medicine (Z Saadatian PhD), Infectious Diseases Research Center (Z Saadatian PhD), Gonabad University of Medical Sciences, Gonabad, Iran; Department of Pharmacy Services (K Rotimi MSc), Alberta Health Services, Edmonton, AB, Canada; West African Postgraduate College of Pharmacists, Lagos, Nigeria (K Rotimi MSc); Department of Analytical and Applied Economics (Prof H Rout PhD, P Sahoo MA, C Swain MPhil), RUSA Centre of Excellence in Public Policy and Governance (Prof H Rout PhD), UGC Centre of Advanced Study in Psychology (Prof M Satpathy PhD), Utkal University, Bhubaneswar, India; Isfahan University of Medical Sciences (H Rouzbahani MD), Islamic Azad University, Isfahan, Iran; Department of Community Medicine (A Roy MD), RVM Medical College and Research Centre, Hyderabad, Hyderabad, India; Achutha Menon Centre for Health Science Studies (A Roy MD), Sree Chitra Tirunal Institute for Medical Sciences and Technology, Thiruvananthapuram, India; Department of Biochemistry and Food Analysis (N Roy PhD), Department of Post-Harvest Technology and Marketing (A Sayeed MSc), Patuakhali Science and Technology University, Patuakhali, Bangladesh; Department of Labour (P Roy PhD), Government of West Bengal, Kolkata, India; Department of Veterinary Microbiology (P Roy PhD), College of Veterinary Science and Animal Husbandry, Agartala, India; Faculty of Medicine (B Roy PhD), Quest International University Perak, Ipoh, Malaysia; Research Department (S Roy MSc), Indian Institute of Public Health, Delhi, India; Department of Health Statistics (S F Rumisha PhD), National Institute for Medical Research, Dar es Salaam, Tanzania; Department of Cardiology (M Russo PhD), SS. Annunziata Hospital - ASL2 Abruzzo, Chieti, Italy; Department Infectious Diseases (M Sabbatucci PhD), National Institute of Health, Rome, Italy; Department for Health Prevention (M Sabbatucci PhD), Ministry of Health, Rome, Italy; Department of Medical Pharmacology (Prof M M Saber-Ayad PhD), Public Health and Community Medicine Department (M R Salem MD), Cairo University, Giza, Egypt; Neuropsychiatric Institute (Prof P S Sachdev MD), Prince of Wales Hospital, Randwick, NSW, Australia; Escuela de Kinesiología (Prof K P Sadarangani PhD), Diego Portales University, Santiago de Chile, Chile; Universidad Autónoma de Chile, Santiago de Chile, Chile (Prof K P Sadarangani PhD); Department of Computer (T Sadegh MSc), University of Science and Culture, Tehran, Iran; Department of Nursing and Midwifery (M Saeedi PhD), Saveh University of Medical Sciences, Saveh, Iran; Department of Neurology (M Safdarian MD), Christian-Doppler University Hospital, Salzburg, Austria; Spinal Cord Injury and Tissue Regeneration Center Salzburg (SCI-TReCS) (M Safdarian MD), Paracelsus Medical University, Salzburg, Austria; Faculty of Medicine, Bioscience and Nursing (S Safi PhD), MAHSA University, Selangor, Malaysia; Interdisciplinary Research Centre in Biomedical Materials (IRCBM) (S Safi PhD), COMSATS Institute of Information Technology, Lahore, Pakistan; ICMR - National Institute for Research in Bacterial Infections (Prof I Saha PhD), Indian Council of Medical Research, Kolkata, India; Department of Public Health (B Sahiledengle MPH), Madda Walabu University, Bale Robe, Ethiopia; Research Centre for Public Health, Equity and Human Flourishing (B Sahiledengle MPH), Torrens University Australia, Adelaide, SA, Australia; Faculty of Health Sciences (G Şahin Bodur PhD), Çankırı Karatekin University, Çankırı, Turkiye; Department of Psychiatry (Z Saif MBA), Ministry of Health, Manama, Bahrain; College of Pharmacy (Prof S Sajadi PhD), Al-Hadba University,

Mosul, Iraq; Department of Health and Kinesiology (M Sajib BDS), University of Illinois, Urbana-Champaign, IL, USA; Department of Statistics (M R Sajid PhD), University of Gujrat, Gujrat, Pakistan; Student Research Committee (M Salehi MD), Kashan University of Medical Sciences, Kashan, Iran; Technology Management Department (Prof M Z Y Salem PhD), University College of Applied Sciences, Gaza, Palestine; School of Economics and Management (Prof M Z Y Salem PhD), University of Kassel, Kassel, Germany; College of Nursing (D Salihu PhD), Jouf University, Jouf, Saudi Arabia; Benang Merah Research Center (Y L Samodra PhD), Benang Merah Research Center (BMRC), Minahasa Utara, Indonesia; Department of Forensic Biology (S G Sangle PhD), Government Institute of Forensic Science Chhatrapati Sambhajinagar, Chhatrapati Sambhajinagar Maharashtra, India; University of São Paulo City, São Paulo, Brazil (L H C C Santos MSc); Independent Consultant, Thiruvananthapuram, India (S Y I Saraswathy PhD); Department of Public Health (Y Sarikhani PhD), Jahrom University of Medical Sciences, Jahrom, Iran; Department of Food Processing Technology (T Sarkar PhD), West Bengal State Council of Technical Education, Malda, India; Bodoland Univeisty (H Sarma PhD), Botany Department, Kokrajhar, India; Faculty of Science (M Sarmadi MSc), Australian Centre for Health Services Innovation (Q Xia PhD), Queensland University of Technology, Brisbane, QLD, Australia; Health Sciences Research Center (M Sarmadi MSc), Torbat Heydariyeh University of Medical Sciences, Torbat Heydariyeh, Razavi Khorasan Province, Iran; Department of Oral Pathology and Microbiology (Prof S C Sarode PhD, Prof G S Sarode PhD), Dr. D. Y. Patil Dental College & Hospital (Prof S Selvaraj PhD), Dr. D. Y. Patil Vidyapeeth, Pune (Deemed to be University), Pune, India; Faculty of Health & Social Sciences (B Sathian PhD), Bournemouth University, Bournemouth, UK; Udyam-Global Association for Sustainable Development, Bhubaneswar, India (Prof M Satpathy PhD); Department of Medical Informatics (J Saulam MSc), Kagawa University, Miki-cho, Japan; Food Processing and Nutrition (J Saulam MSc), Karnataka State Akkamahadevi Women's University, Vijayapura, India; Precision Medicine Department (M Savabi Far MD, S Tajabadi MSc), Università degli studi della Campania Luigi Vanvitelli (University of Campania Luigi Vanvitelli), Naples, Italy; Department of Public Health Sciences (M Sawhney PhD), University of North Carolina at Charlotte, Charlotte, NC, USA; Department of Preventive and Social Medicine (G Saya MD), Jawaharlal Institute of Postgraduate Medical Education and Research, Puducherry, India; Faculty of Business and Computing (Prof C Schinckus PhD), University of the Fraser Valley, Abbotsford, BC, Canada; Department of Finance (Prof C Schinckus PhD), International School of Management, Paris, France; Chief Data Officer Directorate (J C Schmidt MSc), Office for Health Improvement and Disparities (Prof N Steel PhD), UK Department of Health and Social Care, London, UK; Cardiovascular Program (X Xu PhD), The George Institute for Global Health, Sydney, NSW, Australia (Prof A E Schutte PhD); Clinic for Conservative Dentistry and Periodontology (Prof F Schwendicke PhD), University Hospital of the Ludwig-Maximilians-University Munich, Munich, Germany; Department of Medical Statistics (M Šekerija PhD), University of Zagreb, Zagreb, Croatia; Department of Epidemiology and Prevention of Chronic Noncommunicable Diseases (M Šekerija PhD), Croatian Institute of Public Health, Zagreb, Croatia; Emergency Department (S Senthilkumaran PhD), Manian Medical Centre, Erode, India; Department of Medicine (Y Sethi MD), Swami Vivekanand Subharti University, Meerut, India; National Heart, Lung, and Blood Institute (A Seylani MD), National Institutes of Health, Rockville, MD, USA; Dongguan Key Laboratory of Computer-Aided Drug Design (M Shahab PhD), Key Laboratory of Computer-Aided Drug Design (M Waqas PhD), Guangdong Medical University, Dongguan, China; State Key Laboratories of Chemical Resources Engineering (M Shahab PhD), Beijing University Of Chemical Technology, Beijing, China; School of Health Sciences (S Shaharudin PhD), Universiti Sains Malaysia, Kota Bharu, Malaysia; Department of Biotechnology (S Shahid MPhil), Quaid-i-Azam University Islamabad, Islamabad, Pakistan;

Gastroenterology Unit (E Shahini MD), IRCCS, Castellana Grotte (Bari), Italy; Department of Chemistry (H R Shahsavari PhD), Institute for Advanced Studies in Basic Sciences (IASBS), Zanjan, Iran; Independent Consultant, Karachi, Pakistan (M A Shaikh MD); Department of Medicine (N Shaikh MBBS), Liaquat University Of Medical and Health Sciences, Jamshoro, Pakistan; Noncommunicable Diseases Research Center (A Shakerimoghaddam PhD), Department of Basic Medical Sciences (S Yaghoubi PhD), Neyshabur University of Medical Sciences, Neyshabur, Iran; Department of Pathology and Laboratory Medicine (S Sham MD), Northwell Health, New York, NY, USA; Department for Evidence-based Medicine and Evaluation (A Sharifan PharmD), University for Continuing Education Krems, Krems, Austria; Department of Biotechnology (B Sharma PhD), Graphic Era (Deemed to be University), Dehradun, India; Amity Institute of Biotechnology (A Sharma PhD), Amity University Rajasthan, Rajasthan, India; Department of Forensic Science (Prof B K Sharma PhD, M Walia MPhil), Faculty of Medicine and Health Sciences (Prof N P Singh MD), Shree Guru Gobind Singh Tricentenary University, Gurugram, India; Department of Engineering (A Shavandi PhD), Free University of Brussels, Brussels, Belgium; K S Hegde Medical Academy (Prof M Shetty MD), KS Hegde Medical Academy (S S Shetty MD), Nitte University, Mangalore, India; Department of Epidemiology and Health Statistics (F Shi PhD), Wenzhou Medical University, Wenzhou, China; HIV/AIDS Prevention and Control (B F Shibesh MPH), Amahara Regional Sate Health Bureau, Bahir Dar, Ethiopia; Department of Public Health (D Shiferaw MPH), Dambi Dollo University, Dembi Dollo, Ethiopia; Department of Pharmacology (T Shimels MSc), Saint Paul's Hospital Millennium Medical College, Addis Ababa, Ethiopia; Finnish Institute of Occupational Health, Helsinki, Finland (R Shiri PhD); Oulu Business School (I Shiue PhD), Martti Ahtisaari Institute (I Shiue PhD), University of Oulu, Oulu, Finland; Department of Experimental Research (V Shivarov PhD), Medical University Pleven, Pleven, Bulgaria; Department of Genetics (V Shivarov PhD), Sofia University "St. Kliment Ohridiski", Sofia, Bulgaria; Department of Research and Academics (S Shrestha PhD), Kathmandu Cancer Center, Bhaktapur, Nepal; Person-Centered Research (S Shrestha PhD), Monash University, Box Hill, VIC, Australia; Kenneth H. Cooper Institute (Prof K Shuval PhD), Texas Tech University Health Sciences Center, Dallas, TX, USA; Advanced Materials Division (N R S Sibuyi PhD), Mintek, Randburg, South Africa; Department of Biotechnology (N R S Sibuyi PhD), University of the Western Cape, Bellville, South Africa; Department of Medical Microbiology and Infectious Diseases (E E Siddig MD), Erasmus University, Rotterdam, Netherlands; RISE Health (Prof L M L R Silva PhD), University of Beira Interior, Covilhã, Portugal; Department of Law, Economics, Management and Quantitative Methods (Prof B Simonetti PhD), University of Sannio, Benevento, Italy; WSB University in Gdańsk, Gdańsk, Poland (Prof B Simonetti PhD); School of Public Health & Zoonoses (B B Singh PhD), Guru Angad Dev Veterinary & Animal Sciences University, Ludhiana, India; Department of Medicine Service (Prof J A Singh MD), US Department of Veterans Affairs (VA), Houston, TX, USA; Department of Pharmacology (H Singh DM), Government Medical College and Hospital, Chandigarh, India; School of Pharmaceutical Sciences (Faculty of Pharmacy) (H Singh PhD), IFTM University, Moradabad, India; Department of Community Medicine (S Singh MD), Veer Chandra Singh Garhwali Government Institute of Medical Science and Research, Srinagar Garhwal, India; Institute of National Importance on Food Technology (Prof B P Singh PhD), National Institute of Food Technology Entrepreneurship and Management, Sonipat, India; Institute of Medical Sciences (S Singh PhD), Banaras Hindu University, Varanasi, India; Department of Internal Medicine (R Sinto MD), University of Indonesia, Jakarta Pusat, Indonesia; Department of Internal Medicine (R Sinto MD), Dr. Cipto Mangunkusumo National Hospital, Jakarta Pusat, Indonesia; Department of Anesthesiology (D Siyoum MD), New York Medical College, Passaic, NJ, USA; Global and European Health Education and Study Institute (Prof N Skhvitaridze PhD), University of Georgia, Tbilisi,

Georgia; NCDC (Prof N Skhvitaridze PhD), National Center for Disease Control and Public Health, Tbilisi, Georgia; Books Committee (V Y Skryabin MD), Royal College of Psychiatrists, London, UK; Royal college of psychiatrists, London, UK (V Y Skryabin MD); Department of Infectious Diseases and Epidemiology (A A Skryabina MD), Pirogov Russian National Research Medical University, Moscow, Russia; Division of Injury Prevention (Prof D A Sleet PhD), The Bizzell Group, Atlanta, GA, USA; Department of Gastroenterology (A Sohal MD), Creighton University, Phoenix, AZ, USA; Clinical Science Line (Prof A Sokhan PhD), Ludwig Boltzmann Institute of Osteologie, Vienna, Austria; Department of Biochemistry (S Solanki MD), American University of Integrative Sciences, Bridgetown, Barbados; Faculty of Public Health (Prof S Solikhah DrPH), Universitas Ahmad Dahlan, Yogyakarta, Indonesia; Hospital Universitario de La Princesa (Prof J B Soriano MD), Universidad Autónoma de Madrid (Autonomous University of Madrid), Madrid, Spain; Centro de Investigación Biomédica en Red Enfermedades Respiratorias (CIBERES), Madrid, Spain (Prof J B Soriano MD); Department of Public Health, Experimental and Forensic Medicine (M Sorrentino MD), University of Pavia, Pavia, Italy; Hull York Medical School (I N Soyiri PhD), University of Hull, Hull City, UK; Department of Neurology (B S Srichawla MD), University of Massachusetts Medical School, Worcester, MA, USA; College of Health and Public Service (S Sriram PhD), University of North Texas, Denton, TX, USA; Department of Primary Care and Public Health (Prof N Steel PhD), University of East Anglia, Norwich, UK; School of Public Health (Prof S Straube DPhil), University of Alberta, Edmonton, Alberta (AB), Canada; Research Department (N Subedi PhD), Nepal Development Society, Kathmandu, Nepal; School of Exercise and Nutrition Sciences (N Subedi PhD), Deakin University, Melbourne, VIC, Australia; Department of Medical Sciences (Prof V Subramaniyan PhD), Sunway University, Subang Jaya, Malaysia; Clinical Research Unit (H Sujon MSc), Projahnmo Research Foundation, Dhaka, Bangladesh; Praboromarajchanok Institute (T Sukaew PhD), Ministry of Public Health, Nonthaburi, Thailand; Department of Community Medicine (A G Suleiman MPH), Ahmadu Bello University, Kaduna State, Nigeria; School of Life Sciences (M Suleman PhD), Department of Artificial Intelligence (S Wang PhD), Xiamen University, Xiamen, China; Faculty of Health Science (D Sulistiyorini MSc), Universitas Indonesia Maju, Jakarta, Indonesia; Institute of Integrated Intelligence and Systems (Prof J Sun PhD), Griffith University, Brisbane, QLD, Australia; Yusuf Hamied Department of Chemistry (Prof H Z Sun PhD), University of Cambridge, Cambridgeshire, UK; The First Hospital of China Medical University (M Sun MM), China Medical University, Shenyang, China; High-Quality Development Evaluation Research Institute (Z Sun PhD), Nanjing University of Posts and Telecommunications, Nanjing, China; Gandhi Medical College (S Sundaragiri MD), Kaloji Narayana Rao University of Health Sciences (KNRUHS), Secunderabad, India; School of Population Health (T L Symons PhD), Curtin University, Perth, VIC, Australia; Department of Clinical Research and Development (Prof L Szarpak PhD), LUXMED Group, Warsaw, Poland; Collegium Medicum (Prof L Szarpak PhD), John Paul II Catholic University of Lublin, Lublin, Poland; Department of Neurology (P Tabaee Damavandi MD), Neurocenter of Southern Switzerland (NSI), Lugano, Switzerland; Department of Medicine (Prof R Tabarés-Seisdedos PhD), University of Valencia, Valencia, Spain; Department of Health, Safety, and Environmental Management (R Tabibi PhD), Abadan School of Medical Sciences, Abadan, Iran; Saveetha Medical College and Hospital (M Tabish MPharm), Saveetha Institute of Medical and Technical Sciences, Chennai, India; Division of Epidemiology (T Tabuchi MD), Tohoku University, Sendai, Japan; School of Dentistry and Oral Health (S K Tadakamadla PhD), School of Medicine and Dentistry (M N Wanjau PhD), Griffith University, Gold Coast, QLD, Australia; Department of Physiotherapy (B A Tafida MSc), A.T. Still University, Azare, Nigeria; Department of Dermato-Venereology (M Tampa PhD), Dr. Victor Babes Clinical Hospital of Infectious Diseases and Tropical Diseases, Bucharest, Romania; Department of Medicine (J L Tamuzi MSc),

Northlands Medical Group, Omuthiya, Namibia; State Key Laboratory of Numerical Modeling for Atmospheric Sciences and Geophysical Fluid Dynamics (LASG) (H Tang PhD), Chinese Academy of Sciences, Beijing, China; Department of Optometry (G Tang MS), Eye Hospital of Shandong University of Traditional Chinese Medicine, Jinan, China; Department of Computer and Software Engineering (M Tanveer PhD), NUST School of Health Sciences (Prof Y Waheed PhD), National University of Science and Technology (NUST), Islamabad, Pakistan; Department of Psychology (S Taridashti MA), Montclair State University, Montclair, NJ, USA; National Research and Innovation Agency, Jakarta, Indonesia (I U Tarigan PhD); Department of Public Health (M K Tariku MPH), School of Public Health (G K Wirtu PhD), Debre Markos University, Debre Markos, Ethiopia; Department of Pharmacology and Therapeutics (S Tariq PhD), The University of Faisalabad, Faisalabad, Pakistan; Department of Public Health and Informatics (A Tasnim MPH), Bangladesh Medical University, Dhaka, Bangladesh; Indiana University School of Medicine (M G Tedla PhD), University of Missouri, Indianapolis, IN, USA; Clinical Microbiology (C Thakur PhD), Karnali Academy of Health Sciences (KAHS), Jumla, Nepal; Department of Economics (I Tharwat PhD), The American University in Cairo, Cairo, Egypt; Department of Radiology (H Theyra-Enias MD), Kaduna State University, Kaduna, Nigeria; Department of Applied Bioscience (Prof M Thiruvengadam PhD), Konkuk University, Seoul, South Korea; Faculty of Public Health (J H V Ticoalu MPH), Universitas Sam Ratulangi (Sam Ratulangi University), Manado, Indonesia; Douala Laquintinie Hospital, Douala, Cameroon (Y Tochie Noutakdie MD), University of Yaoundé I, Douala, Cameroon; Interdisciplinary Health Data Center (R Topor-Madry PhD), Jagiellonian University Medical College, Kraków, Poland; Merilyn and Glick Eye Institute (A Torkashvand MD), University of Indiana, Indianapolis, IN, USA; Nutritional Epidemiology Research Team (EREN) (M Touvier PhD), National Institute for Health and Medical Research (INSERM), Paris, France; High Institute of Sport and Physical Education of Sfax (K Trabelsi PhD), University of Sfax, Sfax, Tunisia; Department of Business Analytics (T H Tran MD), University of Massachusetts Dartmouth, Dartmouth, MA, USA; Second Department of Internal Medicine (Q T H Tran MD), Kansai Medical University, Osaka, Japan; John T. Milliken Department of Medicine (T Q M Tran MSc), Washington University in St. Louis, Saint Louis, MO, USA; School of Medicine and Dentistry (M T N Tran PhD), Griffith University, Gold Coast, QLD, Australia; Molecular Neuroscience Research Center (N Tran Minh Duc MD), Shiga University of Medical Science, Shiga, Japan; ALS Vietnam Research and Advocacy Initiative (N Tran Minh Duc MD), ALS Vietnam, Quang Ngai, Viet Nam; Adult Learning Disability Service (S J Tromans PhD), Leicestershire Partnership National Health Service Trust, Leicester, UK; CRIMEDIM Center for Research and Training in Global Health, Humanitarian Aid and Disaster Medicine (C Truppa MD), University of Eastern Piedmont, Novara, Italy; Department of Primary Care (C Truppa MD), Geneva University Hospital, Geneva, Switzerland; Kent and Medway Medical School (Prof G Tse PhD), Kent and Medway Medical School, Canterbury, UK; Department of Internal Medicine (M Tumurkhuu PhD), Wake Forest University, Winston-Salem, NC, USA; Department of Urology (Z Tuo MS), The Second Hospital of Tianjin Medical University, Tianjin, China; Hayatabad Medical Complex (H Ullah MBBS), Postgraduate Medical Institute, Peshawar, Pakistan; Federal University of Health Sciences Azare (L Umar PhD), Federal Teaching Hospital, Azare, Nigeria; Federal Teaching Hospital Azare (L Umar PhD), Federal Medical Centre, Azare, Bauchi-State, Nigeria; Department of Medicine (M Umar MBBS), Khairpur Medical College, Khairpur, Pakistan; Department of Orthodontics (H Uzunçıbuk PhD), University of Trakya, Edirne, Turkiye; Johnson & Johnson (P Vadagam MS), Duquesne University, Pittsburgh, PA, USA; Sociedad Argentina de Medicina, Buenos Aires, Argentina (Prof P R Valdez PhD); Hospital Vélez Sarsfield, Buenos Aires, Argentina (Prof P R Valdez PhD); Department of Psychology (Z Vally PhD), Zayed University, Abu Dhabi, United Arab Emirates; Faculty of Sciences (J Varasteh MSc), University of Guilan,

Rasht, Iran; UKK Institute, Tampere, Finland (Prof T J Vasankari PhD); Faculty of Medicine and Health Technology (Prof T J Vasankari PhD), Tampere University, Tampere, Finland; Department of Biochemistry (S Vasishta PhD), Apollo Institute of Medical Sciences and Research Chittoor, Chittoor, India; Department of Otolaryngology Head and Neck Surgery (S Vasudevan MS), Louisiana State University Health Sciences Center, Shreveport, LA, USA; Biomedical Engineering Department (A Vaysi MSc), University of Texas, Arlington, TX, USA; Department of Human Genetics & Molecular Biology (B Vellingiri PhD), Bharathiar University, Coimbatore, India; PG & Research Department of Chemistry (G Venkatraman Subramanian PhD), Auxilium College (Autonomous), Vellore, India; Raffles Neuroscience Centre (Prof N Venketasubramanian MSc), Raffles Hospital, Singapore, Singapore; Department of Pediatrics (Prof R Vidavalur MD), Cornell University, Ithaca, NY, USA; Department of Physiotherapy (J H Villafañe PhD), Universidad Europea de Madrid (European University of Madrid), Villaviciosa de Odón, Spain; Digital Health Research Center (D Villarreal-Zegarra MPH), Instituto Peruano de Orientación Psicológica, Lima, Peru; Occupational Medicine Unit (Prof F S Violante MD), Sant'Orsola Malpighi Hospital, Bologna, Italy; Cardiac Electrophysiology (S C Vipparthy MD), St Bernard’s Medical Center, Jonesboro, AR, USA; Faculty of Medicine of Itajubá, Brazil (L M Vitorino PhD), Faculty of Medicine of Itajubá, Brazil, Itajubá, Brazil; Széchenyi István University, Gyor, Hungary (Prof Y Waheed PhD); Department of Social Sciences (A W Wamai MSc), Chuka University, Kenya, Nairobi, Kenya; School of Chinese Medicine (Prof J Wan PhD), School of Traditional Chinese Medicine (Prof H Yao PhD), Beijing University of Chinese Medicine, Beijing, China; Department of Neurosurgery (S Wang MD), Capital Medical University, Beijing, China; Department of Neurosurgery (S Wang MD), Beijing Tiantan Hospital, Beijing, China; College of Agriculture (X Wang PhD), Northwest A&F University, Xianyang City, China; Department of Rehabilitation (J Wang PhD), Southeast University, Nanjing, China; Centre for Health Policy Research (Prof P Ward PhD), Adelaide, SA, Australia; Department of Orthopaedics (F Wei PhD), General Hospital of Central Theater Command, Wuhan, China; Fourth Military Medical University, Xi'an, China (F Wei PhD); Department of Geriatrics (X Wei MS), The Eighth Affiliated Hospital of Sun Yat-sen University, Shenzhen, China; Cardiology Department (Prof R G Weintraub MB), Royal Children's Hospital, Melbourne, VIC, Australia; Competence Center of Mortality-Follow-Up of the German National Cohort (R Westerman DSc), Federal Institute for Population Research, Wiesbaden, Germany; Department of Physical Therapy (T Wiangkham PhD), Naresuan University, Phitsanulok, Thailand; Department of Experimental Pharmacology (Y Wibowo MD), Heidelberg University, Mannheim, Germany; Department of Nursing (A Wilandika PhD), Universitas Aisyiyah Bandung, Bandung, Indonesia; Institute of Clinical Epidemiology (Prof P Willeit PhD), Medical University Innsbruck, Innsbruck, Austria; Research Organisation (A Wireko MD), Inter-Continental Omni-Research in Medicine Collaborative, Berlin, Germany; School of Public Health (G K Wirtu PhD), University of Technology Sydney, Sydney, New South Wales (NSW), Australia; Cochrane South Africa (Prof C S Wiysonge MD), South African Medical Research Council, Cape Town, South Africa; Department of Public Health (A T Woday MPH), Samara University, Samara, Ethiopia; Department of Research (M W Wojewodzic PhD), Cancer Registry of Norway, Oslo, Norway; Institute of Health and Care Sciences (Prof A W Wolf PhD), University of Gothenburg, Gothenburg, Sweden; Faculty of Health (T E Wonde MPH), University of Technology Sydney, Australia, NSW, Australia; Department of Theory and Empiricism of Healthcare (D T Worede MSc), Universität Kassel, Kassel, Germany; Division of Gastroenterology (Prof Z Wu PhD), Tongji Medical College (G Xiao MD), Huazhong University of Science and Technology, Wuhan, China; Department of Public Health (Prof J Wu MPH), Wuhan fourth hospital, Wuhan, China; Department of Food Science and Human Nutrition (Prof F Wu PhD), Michigan State University, East Lansing, MI, USA; Shenzhen Institute of Advanced

Technology (P Wu PhD), Chinese Academy of Sciences, Shenzhen, China; Western Institute of Digital-Intelligent Medicine (Z Xia MD), Chongqing Medical University, Chongqing, China; School of Public Health (H Xiao PhD), Zhejiang University, Zhejiang, China; Department of Public Health Science (H Xiao PhD), Fred Hutchinson Cancer Research Center, Seattle, WA, USA; Department of Intelligent Medical Engineering (Prof W Xie DrPH), Anhui Medical University, Anhui, China; Department of Surgery (Prof W Xie DrPH), The First Affiliated Hospital of Anhui Medical University, Hefei, Anhui, China; Department of Nutrition (W Xu MPH), Tufts University, Boston, MA, USA; Department of Endocrinology (Prof S Xu PhD), University of Science and Technology of China, Hefei, China; School of Medicine (Prof S Xu PhD), University of Rochester, Rochester, NY, USA; School of Public Health (Prof W Xu MD), Southwest Medical University, luzhou, China; School of Medicine (M Xue MSc), Kunming University of Science and Technology, Kunming, China; Department of Environmental Health and Epidemiology (V Yadav MD), National Institute for Research in Environmental Health, Bhopal, India; Department of Community Medicine (S Yahoo (Syed) MD), Apollo Institute of Medical Sciences and Research, Hyderabad, India; Department of Public Health (Prof K Yamagishi MD, Prof N Yonemoto PhD), Faculty of Medicine (Y Yano MD), Juntendo University, Tokyo, Japan; Department of Public Health Administration (H Yang MD), Linyi People's Hospital, Linyi, China; Department of Medicine (A Yarahmadi PhD), Thomas Jefferson University, Philadelphia, PA, USA; Department of Public Health (A Yekdeş MD), Trakya University, Edirne, Turkiye; Department of Family Medicine (S A Yesuf MSc), St. Paul's Hospital Millennium Medical College, Addis Ababa, Ethiopia; Family Medicine Department (S A Yesuf MSc), St. Peter's Specialized Hospital, Addis Ababa, Ethiopia; KHANA Center for Population Health Research, Phnom Penh, Cambodia (Prof S Yi PhD); Public Health Department (Public health nutrition unit) (M Yigezu MPH), Dire Dawa University, Dire Dawa Administration, Ethiopia; Pharmacy Department (Y E Yismaw MSc), Alkan Health Science, Business and Technology College, Bahir Dar, Ethiopia; Department of Pediatrics (Prof D Yon MD), Kyung Hee University, Seoul, South Korea; Department of Biostatistics (Prof N Yonemoto PhD), University of Toyama, Toyama, Japan; Department of Health Policy and Management (Prof M Z Younis PhD), Jackson State University, Jackson, MS, USA; School of Business & Economics (Prof M Z Younis PhD), Universiti Putra Malaysia (University of Putra Malaysia), Kuala Lumpur, Malaysia; Department of Public Health (A Yousuf PhD), Jigjiga University, Jigjiga, Ethiopia; Sichuan Provincial Center for Mental Health (J Yu MD), University of Electronic Science and Technology of China, Chengdu, China; Key Laboratory of Psychosomatic Medicine (J Yu MD), Chinese Academy of Medical Sciences, Chengdu, China; School of Public Health (Prof Y Yu MS), Hubei University of Medicine, Shiyan, China; Southeast University Affiliated Xuzhou Central Hospital (H Yuan PhD), Clinical Hospital, Xuzhou, China; Department of Nursing Science (U Yunusa PhD), Bayero University, Kano, Nigeria; Faculty of Nursing (U Yunusa PhD), University of Alberta, Edmonton, AB, Nigeria; Association for Socially Applicable Research (ASAR), Pune, India (S Zadey MS); Department of Emergency Medicine (S Zadey MS), Global Emergency Medicine Innovation and Implementation (GEMINI) Research Center, Durham, NC, USA; Epidemiology and Cancer Registry Sector (Prof V Zadnik PhD), Institute of Oncology Ljubljana, Ljubljana, Slovenia; Islamic Azad University, Tehran, Iran (M Zaghampour MD); Faculty of Medicine and Health Sciences (E Zainal Abidin PhD), Universiti Putra Malaysia (Putra University of Malaysia), UPM Serdang, Malaysia; Faculty of Medicine and Health Sciences (F Zakham PhD), Hodeidah University, Hodeidah, Yemen; Department of Health Sciences (S Zaman PhD), James Madison University, Harrisonburg, VA, USA; The Heller School for Social Policy and Management (H Zandam PhD), Brandeis University, Waltham, MA, USA; Sant'Elia Hospital (A Zanghì MD), University of Catania, Caltanissetta, Italy; Research and Development Department (I Zare BSc), Sina Medical Biochemistry Technologies, Shiraz, Iran; Nursing Care Research Center in Chronic

Diseases (Prof K Zarea PhD), Ahvaz Jundishapur University of Medical Sciences, Ahvaz, Iran; Department of Administration (Prof M Zastrozhin PhD), PGxAI, San Francisco, CA, USA; Department of Clinical Practice (M Zawiah PhD), Northern Border University, Rafha, Saudi Arabia; Cardiology Population Health Laboratory (D Zemedikun PhD), Victor Chang Cardiac Research Institute (VCCRI), Perth, WA, Australia; Institute of Diagnostic and Interventional Radiology and Neuroradiology (S Zensen MD), University of Duisburg-Essen, Essen, Germany; Department of Internal Medicine (X Zhang MD), Jacobi Medical Center, Bronx, NY, USA; Department of Internal Medicine (X Zhang MD), Albert Einstein College of Medicine, Bronx, NY, USA; Medical Oncology Department of Gastrointestinal Cancer (L Zhang MS), Cancer Hospital of Dalian University of Technology, Shenyang, China; School of Biomedical Engineering (L Zhang MS), Dalian University of Technology, Dalian, China; School of Public Health (Y Zhang PhD), Hubei Province Key Laboratory of Occupational Hazard Identification and Control (Y Zhang PhD), Wuhan University of Science and Technology, Wuhan, China; Department of Cardiology (B Zhang PhD), Zhongshan Hospital, Fudan University, Shanghai, China; Department of Obstetrics and Gynecology (N Zhang BS), Frist Affiliated Hospital of Anhui Medical University, Hefei, China; Burn Surgery Department (X Zhang PhD), The First Hospital of Jilin University, Changchun, China; Tianjin Medical University General Hospital (Z Zhang MD), Tianjin Centers for Disease Control and Prevention, Tianjin, China; XuZhou Medical University (Prof J Zhang DrPH), University of Medicine, XuZhou, China; Department of Health Management (Z Zhao PhD), Shengjing Hospital of China Medical University, Shenyang, China; Office of Chongqing Cancer Prevention and Treatment (S Zhao MPH), Chongqing University Cancer Hospital, Chongqing, China; Department of Hepatology (Prof M Zheng PhD), Wenzhou Medical University, wenzhou, China; School of Data Science (J Zhou PhD), The Chinese University of Hong Kong, Shenzhen, Shenzhen, China; School of Public Health and Emergency Management (B Zhu PhD), Southern University of Science and Technology, Shenzhen, China; Institute of Public Health and Social Sciences (H Zia BDS), Khyber Medical University, Peshawar, Pakistan; Endocrinology and Metabolism Research Center (G Zoghi MD), Hormozgan University of Medical Sciences, Bandar Abbas, Iran; College of Nursing (M Zoromba PhD), Prince Sattam bin Abdulaziz University, Al-Kharj, Saudi Arabia; Department of Public Health (L Zuhriyah PhD), Universitas Brawijaya, Malang, Indonesia; NIHR-Biomedical Research Centre (NIHR-BRC) (Prof A Zumla PhD), University College London Hospitals, London, UK; Clinical Research Centre (Prof S H Zyoud PhD), An-Najah National University Hospital, Nablus, Palestine; Department of Building Engineering and Environment (S H Zyoud PhD), Civil Engineering and Sustainable Structures (S H Zyoud PhD), Palestine Technical University (Kadoorie), Tulkarem, Palestine

Authors' Contributions

Managing the overall research enterprise

Austin E Schumacher, Katie Joskowitz, Christopher J L Murray

Writing the first draft of the manuscript

Catherine Bisignano, Simon I Hay, Austin E Schumacher, Aleksandr Aravkin, Peng Zheng, Christopher J L Murray

Primary responsibility for applying analytical methods to produce estimates

Haley Comfort, Erin A May, Spencer A Pease, Benjamin David Geller Shapiro, Peng Zheng, Darwin P Jones, Ryan M Barber

Primary responsibility for seeking, cataloguing, extracting, or cleaning data; designing or coding figures and tables

Haley Comfort, John E Fuller, Hannah Elizabeth Robinson-Oden, Haaris Saqib, Denny Wang, Stefanie Watson, Nicholas Verghese

Providing data or critical feedback on data sources

Bhoomadevi A, Mohammad Amin Aalipour, Ukachukwu O Abaraogu, Cristiana Abbafati, Abdallah H A Abd Al Magied, Samar Abd ElHafeez, Mohammed Altigani Abdalla, Emad M. Abdallah, Nadin M. I. Abdel Razeq, Reda Abdel-Hameed, Parsa Abdi, Arash Abdollahi, Rizwan Suliankatchi Abdulkader, Auwal Abdullahi, Abdullahi Salahudeen Abdulraheem, Armita Abedi, Roberto Ariel Abeldaño Zuñiga, Olugbenga Olusola Abiodun, Richard Gyan Aboagye, Shady Abohashem, Lucas Guimarães Abreu, Fuad Hamdi A. Abuadas, Bilyaminu Abubakar, Sawsan Abuhammad, Hana J Abukhadijah, Salahdein Aburuz, Dina Abushanab, Ahmed Abu-Zaid, Anirudh Balakrishna Acharya, Juan Manuel Acuna, Tim Adair, Oladimeji Muritala Adebayo, Tajudeen Adesanmi Adebisi, Kamoru Ademola Adedokun, Oluwatobi E Adegbile, Olumide Thomas Adeleke, Olatunji O Adetokunboh, Mohd Adnan, Qorinah Estiningtyas Sakilah Adnani, Leticia Akua Adzigbli, David Adzrago, Aanuoluwapo Adeyimika Afolabi, Saira Afzal, Gizachew Beykaso Agafari, Mahdi Aghaalikhani, Feleke Doyore Agide, César Agostinis Sobrinho, Anurag Agrawal, Williams Agyemang-Duah, Bright Opoku Ahinkorah, Aqeel Ahmad, Muayyad M Ahmad, Rabbiya Ahmad, Tauseef Ahmad, Ali Ahmed, Aram Mahmood Ahmed, Ayman Ahmed, Gasha Salih Ahmed, Haroon Ahmed, Muktar Beshir Ahmed, Naveed Ahmed, Oli Ahmed, Shabbir Ahmed, Gulzhanat Aimagambetova, Janardhana P Aithala, Budi Aji, Hossein Akbarialiabad, Roland Eghoghosoa Akhigbe, Muhammad Nadeem Akhtar, Hanadi Al Hamad, Omar Al Omari, Zain Al Ta'ani, Yazan Al Thaher, Omar Ali Mohammed Al Zaabi, Mohammad Ahmmad Mahmoud Al Zoubi, Rasmieh Mustafa Al-Amer, Abebaw Alamrew, Turki M Alanzi, Mohammed Albashtawy, Robert W Aldridge, Tekletsadik Tekleslassie Alemayehu, Abdelazeem M Algammal, Aminu Alhassan Alhassan Ibrahim, Shahid Ali, Syed Shujait Ali, Montaha Al-Iede, Sheikh Mohammad Alif, Hamid Alinejad Rokny, Samah W Al-Jabi, Mohamad Aljofan, Syed Mohamed Aljunid, Mustafa Alkhawam, Wesam Taher Almagharbeh, Wael Almahmeed, Md. Al-Mamun, Sabah Al-Marwani, Joseph Uy Almazan, Hesham M Al-Mekhlafi, Omar Almidani, Amr Almobayed, Khaldoon Aied Alnawafleh, Hasan Yaser Alniss, Jaber S Alqahtani, Saleh A Alqahtani, Ahmad Rajeh Al-

Qudimat, Intima Alrimawi, Salman Khalifah Al-Sabah, Mohammed A Alsabri, Awais Altaf, Alaa B Al-Tammemi, Nelson Alvis-Guzman, Mohammad Al-Wardat, Hany Aly, Tarek Tawfik Amin, Alireza Amindarolzarbi, Saeed Amini, Ehsan Amini-Salehi, Nafiu Aminu, Majid Aminzare, Dickson A Amugsi, Ganiyu Adeniyi Amusa, Filippos Anagnostakis, Nazanin Anaraki, Deanna Anderlini, Song Peng Ang, Nguyen Hoang Anh, Samuel Egyakwa Ankomah, Kabilan Annadurai, Sumbul Ansari, Ernoiz Antriyandarti, Saleha Anwar, Sumadi Lukman Anwar, Razique Anwer, Geminn Louis Carace Apostol, Jalal Arabloo, Benedetta Armocida, Jesu Arockiaraj, Mahwish Arooj, Anton A Artamonov, Deepavalli Arumuganainar, Nurila Aryntayeva, Mahsa Asadi Anar, Majid Asadi-Samani, Muhammad Abdul Basit Ashraf, Tahira Ashraf, Mitra Ashrafi, Muhammad Shahzad Aslam, Yuni Asri, Anil Raj Assariparambil, Batyrbek Assembekov, Mirbahador Athari, Maha Moh'd Wahbi Atout, Alok Atreya, Marcel Ausloos, Núbia Carelli Pereira Avelar, Sana Javaid Awan, Beatriz Paulina Ayala Quintanilla, Fekadu Belay Ayalew, Olatunde O Ayinde, Arian Azadnia, James Mba Azam, Farya Azimi, Mohd Yusmaidie Aziz, Ahmed Y Azzam, Giridhara Rathnaiah Babu, Ashish D Badiye, Hunter Southwick Baggen, Ahmed Salem BaHammam, Atif Amin Baig, Ovidiu Constantin Baltatu, Palash Chandra Banik, Ryan M Barber, Hiba Jawdat Barqawi, Amadou Barrow, Shahid Bashir, Mohammad-Mahdi Bastan, Abdul-Monim Batiha, Mulat Tirfie Bayih, Narasimha M Beeraka, Jina Behjati, Babak Behnam, Diana Fernanda Bejarano Ramirez, Bezawit K Bekele, Almaz Nibret Belay, Melesse Belayneh, Bashir Bello, Olorunjuwon Omolaja Bello, Apostolos Beloukas, Samiun Nazrin Bente Kamal Tune, Robert S Bernstein, Akshaya Srikanth Bhagavathula, Sonu Bhaskar, Arushee Bhatnagar, Priyadarshini Bhattacharjee, Gurjit Kaur Bhatti, Jasvinder Singh Bhatti, Sibhatu Kassa Biadgilign, Raluca Bievel-Radulescu, Bijit Biswas, Ahmad Naoras Bitar, Molalegne Bitew, Lucimere Bohn, Obasanjo Afolabi Bolarinwa, Archith Boloor, Sri Harsha Boppana, Berrak Bora Basara, Hamed Borhany, Souad Bouaoud, Soufiane Boufous, Rupert R A Bourne, Nicola Luigi Bragazzi, Dejana Braithwaite, Edmond D Brewer, Annie J Browne, Linh Phuong Bui, Felix Busch, Nadeem Shafique Butt, Tianji Cai, Luis Alberto Cámera, Luciana Aparecida Campos, Joao Mauricio Castaldelli-Maia, Carlos A Castañeda-Orjuela, Ferrán Catalá-López, Francieli Cembranel, Chiranjib Chakraborty, Vijay Kumar Chattu, Victoria Chatzimavridou-Grigoriadou, Akhilanand Chaurasia, Hui Chen, Nicholas WS Chew, William C S Cho, Bryan Chong, Hitesh Chopra, Shivani Chopra, Dinh-Toi Chu, Hongyuan Chu, Stephen Chukwudeh, Sunghyun Chung, Alyssa Columbus, Haley Comfort, Joao Conde, Paolo Angelo Cortesi, Michael H Criqui, Natalia Cruz-Martins, Xiaochen Dai, Mayank Dalakoti, Gloria Dalla Costa, Lalit Dandona, Rakhi Dandona, Lucio D'Anna, Samuel Demissie Darcho, Chengetai Dare, Fernando Pio De la Hoz, Alejandro de la Torre-Luque, Sindhura Deekonda, Denise Myriam Dekker, Pouria Delbari, Andreas K Demetriades, Ismail Dergaa, Kebede Deribe, Hunegnaw Almaw Derseh, Hardik Dineshbhai Desai, Abraham Aregay Desta, Vinoth Gnana Chellaiyan Devanbu, Pradeep Kumar Devarakonda, Devananda Devegowda, Rajinder K K Dhamija, Samath Dhamminda Dharmaratne, Meghnath Dhimal, Marcello Di Pumpo, Diana Dias da Silva, Xueting Ding, M Ashworth Dirac, Huyen Do, Thao Huynh Phuong Do, Klara Georgieva Dokova, Regina-Mae Villanueva Dominguez, Ojas Prakashbhai Doshi, Robert Kokou Dowou, Tim Robert Driscoll, Judy R. Dubno, Bruce B Duncan, Jennifer Dunne, Andre Rodrigues Duraes, Senbagam Duraisamy, Fatemeh Ehsani, Ashkan Eighaei Sedeh, Ebrahim Eini, Michael Ekholuenetale, Temitope Cyrus Ekundayo, Rabie Adel El Arab, Maysaa El Sayed Zaki, Rana Elbeshbeishy, Faris El-Dahiyat, Ibrahim Elsohaby, Chadi Eltaha, Abdelgawad Salah Abdelgawad Eltahawy, Victor Oghenekparobo Emojevwe, Sharareh Eskandarieh, Majid Eslami, Heidar Fadavian, Adeniyi Francis Fagbamigbe, Ayesha Fahim, Ildar Ravisovich Fakhradiyev, Aliasghar Fakhri-Demeshghieh, Luca Falzone, Qiping Fan, Syed Muhammad Yousaf Farooq, Fatemeh Farshad, Abidemi Omolara Fasanmi, Ali Fatehizadeh, Davood Fathi, Valery L Feigin, maryam feili, Alireza Feizkhah, Ginenus Fekadu, Seyed-Mohammad Fereshtehnejad, Luisa S Flor, Artem Alekseevich Fomenkov, Arianna Fornari, Matteo Foschi, Maryam Fotouhi, Kayode Raphael Fowobaje, Richard Charles Franklin, Takeshi Fukumoto,

John E Fuller, Blima Fux, Sridevi G, Peter Andras Gaal, Muktar A Gadanya, Yaseen Galali, Silvano Gallus, Dhanraj Ganapathy, Balasankar Ganesan, David Garcia-Azorin, Zisis Gatzioufas, Bamba Gaye, Ali Gerami Matin, Lemma Getacher, Kalab Yigermal Gete, Peter W Gething, Keyghobad Ghadiri, Arin Ghamkhar, Shakiba Ghasemi Assl, Fariba Ghassemi, Ramy Mohamed Ghazy, Zainab Gholami, Elena Ghotbi, Arun Ghuge, Syed Abdullah Gilani, Alem Abera Girmay, Laszlo Göbölös, Archit Goel, Rajesh Kumar Goel, Mahaveer Golechha, Nelson G M Gomes, Wenping Gong, Yitayal Ayalew Goshu, Ayman Grada, Simon Matthew Matthew Graham, Habtamu Alganeh Guadie, Shi-Yang Guan, Zheng Guo, Rajat Das Gupta, Rajeev Gupta, Sapna Gupta, Awoke Derbie Derbie Habteyohannes, Tesfahun Simon Hadaro, Sarah Hafsia, Arian Haghtalab, Nguyen Hai Nam, Arvin Haj-Mirzaian, Pritam Halder, Sebastian Haller, Islam M Hamad, Nadia M Hamdy, Mohammad Hamza, Nasrin Hanifi, Obaid I Haque, Andy Martahan Andreas Hariandja, Josep Maria Haro, Eka Mishbahatul Marah Has, Faizul Hasan, Ali Hasanpour- Dehkordi, Arezou Hashem Zadeh, Nada Tawfig Hashim, Ammarah Hasnain, Ibrahim Nagmeldin Hassan, Yusuf Wada Hassan Wada, Mahgol Sadat Hassan Zadeh Tabatabaei, Simon I Hay, Jiawei He, Jeffrey J Hebert, Golnaz Heidari, Mehdi Hemmati, Claire A Henson, Claudiu Herteliu, Sumudu Avanthi Hewage, Nobuyuki Horita, Alamgir Hossain, Md Mahbub Hossain, Mohammad Bellal Hossain, Jada Averianna Houser, Chengxi Hu, Weijun Huang, Nawfal R Hussein, Mohamed Ibrahim Husseiny, Hong-Han Huynh, Bing-Fang Hwang, Segun Emmanuel Ibitoye, Ismail A. Atef Ismail Ahmed Ibrahim, Ramzi Ibrahim, Pulwasha Maria Iftikhar, Kevin S Ikuta, Olayinka Stephen Ilesanmi, Lucius Chidiebere Imoh, Benni Iskandar, Teresa R Iskander, Nahlah Elkudssiah Ismail, Gaetano Isola, Mahalaxmi Iyer, Jalil Jaafari, Mohammadsadegh Jafari, Morteza Jafarinia, Haitham Jahrami, Mihajlo Jakovljevic, Ali Jaliliyan, Mohamed Jalloh, Armaan Jamal, Jazlan Jamaluddin, Jerin James, Tahereh Javaheri, Syed Sarmad Javaid, Shubha Jayaram, Yovanthi Anurangi Jayasinghe, Achala Upendra Jayatilleke, Bijay Mukesh Jeswani, Wenyi Jin, Jost B Jonas, Darwin Phan Jones, Tamas Joo, Abel Joseph, Charity Ehimwenma Joshua, Jacek Jerzy Jozwiak, Zubair Kabir, Dler H. Hussein Kadir, Ashish Kumar Kakkar, Pradnya Vishal Kakodkar, Khalil Kalavani, Sivesh Kathir Kamarajah, Saltanat Kamenova, Ramat T. Kamorudeen, Devanish Narasimhasanth Kamtam, Oleksandr Kamyshnyi, Jiseung Kang, Rami S Kantar, Neeti Kapoor, Sujita Kumar Kar, Salah Eddin Karimi, Mohmed Isaqali Karobari, Tomasz M Karpiński, Sadanand Karun, Manoj Kumar Kashyap, Nicholas J Kassebaum, Adarsh Katamreddy, Kanica Kaushal, Foad Kazemi, Nastaran Kazemi rad, Emmanuelle Kesse-Guyot, Himanshu Khajuria, Nauman Khalid, Hazim O. Khalifa, Anas Husam Khalifeh, Anees Ahmed Khalil, Pantea Khalili, Faham Khamesipour, Ajmal Khan, Iman Waheed Khan, Maseer Khan, Muhammad Mueed Khan, Muhammad Umer Khan, Ramsha Mushtaq Khan, Zahid Khan, Zahid Khan, Zenith Khashim, Khaled Khatab, Khalid A Kheirallah, Sunil Kumar Khokhar, Mohammad Saeid Khonji, Atulya Aman Khosla, Majid Khosravi, Jagdish Khubchandani, Zemene Demelash Kifle, Jinho Kim, Yun Jin Kim, Yohannes Kinfu, Sanjay Kini B, Mary Kirk, Adnan Kisa, Sezer Kisa, Ladli Kishore, Juniper Boroka Kiss, Shivakumar KM, Ann Kristin Skrindo Knudsen, Michail Kokkorakis, Diana Gladys Kolieghu Tcheumeni, Aida Kondybayeva, Tapos Kormoker, Oleksii Korzh, Archana Koul, Sindhura Lakshmi Koulmane Laxminarayana, Irene Akwo Kretchy, Kewal Krishan, Chong-Han Kua, Barthelemy Kuate Defo, Shikha Kukreti, Shweta Kulshreshtha, Dewesh Kumar, G Anil Kumar, Jogender Kumar, Manasi Kumar, Sanjay Kirshan Kumar, Vijay Kumar, Jibin Kunjavara, Maria Dyah Kurniasari, Asep Kusnali, Dian Kusuma, Assylkhan Kuttybayev, Ville Kytö, Hmwe Hmwe Kyu, Pallavi L C, Adriano La Vecchia, Muhammad Awwal Ladan, Chandrakant Lahariya, Anita Lakhani, Dharmesh Kumar Lal, Tea Lallukka, Iván Landires, Kamaluddin Latief, Mahrukh Latif, Saheed Akinmayowa Lawal, Aliyu Lawan, Huu-Hoai Le, Minh Huu Nhat Le, Nhi Huu Hanh Le, Thao Thi Thu Le, Trang Diep Thanh Le, Caterina Ledda, Seung Won Lee, Wei-Chen Lee, Vasileios Leivaditis, Matthew J Lennon, Chengfeng Li, Weilong Li, Yongze Li, Zhengrui Li, Yanxue Lian, Virendra S Ligade, Stephen S Lim, Queran Lin, Gang Liu, Jue Liu, Xuefeng Liu, Zhe Liu, Erand Llanaj, Michael J Loftus, Rafael Lozano, Jailos Lubinda, Jay B

Lusk, Angelina M Lutambi, Miltiadis D Lytras, Ellina Lytvyak, Kevin Sheng-Kai Ma, Zheng Feei Ma, Monika Machoy, Seyed Ataollah Madinezad, Christian Madsen, Aurea Marilia Madureira-Carvalho, D. R. Mahadeshwara Prasad, Sasikumar Mahalingam, Preeti Maharjan, Nozad Hussein Mahmood, Alireza Mahmoudi, Rituparna Maiti, Ahmad Azam Malik, Deborah Carvalho Malta, Emery Manirambona, Lokesh Manjani, Mohammad Ali Mansournia, Changkun Mao, Joemer C Maravilla, Adilson Marques, Bernardo Alfonso Martinez-Guerra, Francisco Rogerlândio Martins-Melo, Roy Rillera Marzo, Sammer Marzouk, Sugeng Mashudi, Clara N Matei, Alexander G Mathioudakis, Medha Mathur, Rita Mattiello, Erin A May, Michael A McPhail, Steven M McPhail, Enkeleint A Mechili, Vini Mehta, Tesfahun Mekene Meto, Walter Mendoza, Godfred Antony Menezes, Atte Meretoja, Tomislav Mestrovic, Chamila Dinushi Kukulege Mettananda, Sachith Mettananda, Irmina Maria Michalek, Andrea Michelerio, Ted R Miller, Seyed Ali Mirshahvalad, Awoke Misganaw, Archana Mishra, Chaitanya Mittal, Mona Gamal Mohamed, Nouh Saad Mohamed, Khabab Abbasher Hussien Mohamed Ahmed, Abdollah Mohammadian-Hafshejani, Abdulwase Mohammed, Mustapha Mohammed, Shafiu Mohammed, Yahaya Mohammed, Ali H Mokdad, Shaher Momani, Lorenzo Monasta, Yousef Moradi, Mahmoud M Morsy, Jonathan F Mosser, Mariana Mourgova, Rabia Mubarak, Faraz Mughal, Syed Aun Muhammad, Oscar J Mujica, Sukhes Mukherjee, Sumoni Mukherjee, George Duke Mukoro, Francesk Mulita, Chalie Mulugeta, Yanjinlkham Munkhsaikhan, Efren Murillo-Zamora, Christopher J L Murray, Ali Mushtaq, Sherzad Ibrahim Mustafa, Mubarak Taiwo Mustapha, Saravanan Muthupandian, Muhammad Muzaffar, Ahamarshan Jayaraman Nagarajan, Shankar Prasad Nagaraju, Mohsen Naghavi, Ganesh R Naik, Yvonne Nartey, Bruno Ramos Nascimento, Hamide Nasiri, Mahmoud Nassar, Zuhair S Natto, Zakira Naureen, Biswa Prakash Nayak, Masoud Negahdary, Ionut Negoi, Ruxandra Irina Negoi, Henok Biresaw Netsere, Georges Nguefack-Tsague, Josephine W Ngunjiri, Cuong Tat Nguyen, Dang Nguyen, Long Nguyen, Tu Anh Nguyen, Van Thanh Nguyen, Ambe Marius Ngwa, Robina Khan Niazi, Luciano Nieddu, Chukwudi A Nnaji, Shuhei Nomura, Syed Toukir Ahmed Noor, Masoud Noroozi, Jean Jacques Noubiap, Fred Nugen, Nurfatimah Nurfatimah, Dieta Nurrika, Ogochukwu Janet Nzoputam, Bogdan Oancea, Ismail A Odetokun, Michael Safo Oduro, Onome Bright Oghenetega, Sarah Oh, Andrew T Olagunju, Oladotun Victor Olalusi, Matthew Idowu Olatubi, Bolajoko Olubukunola Olusanya, Jacob Olusegun Olusanya, Sandersan Onie, Obinna E Onwujekwe, Marcel Opitz, Atakan Orscelik, Alberto Ortiz, Olayinka Osuolale, Adrian Otoiu, Oche Joseph Otorkpa, Abdu Oumer, Jerry John Ouner, Amel Ouyahia, Mayowa O Owolabi, Mahesh P A, Jagadish Rao Padubidri, Dimpal Manilal Paija, Keyvan Pakshir, Tejasri Paluvai, Sujogya Kumar Panda, Songhomitra Panda-Jonas, Seithikurippu R Pandi-Perumal, Leonidas D Panos, Anca Pantea Stoian, Paraskevi Papadopoulou, Parinaz Paranjkhoo, Shahina Pardhan, Romil R Parikh, Chulwoo Park, Seoyeon Park, Swapnil Parve, Maja Pasovic, Bhumi Hemal Patel, Hemal M Patel, Heta Pavan Patel, Mitesh Patel, Neel Navinkumar Patel, Sangram Kishor Patel, Ashlesh Patil, Shankargouda Patil, Apurba Patra, Shrikant Pawar, Shubhadarshini Pawar, Spencer A Pease, Paolo Pedersini, Prince Peprah, Gavin Pereira, Arokiasamy Perianayagam, Simone Perna, Hoang Nhat Pham, Hoang Tran Pham, Nhat Truong Pham, David M Pigott, Zahra Zahid Piracha, Florian Ploeckl, Ramesh Poluru, Sajjad Pourasghary, Naeimeh Pourtaheri, Sergio I Prada, Jalandhar Pradhan, Elton Junio Sady Prates, Harsh Priya, Jagadeesh Puvvula, Xiang Qi, Zhipeng Qi, Zahiruddin Syed Quazi, Hadi Raeisi Shahraki, Pracheth Raghuveer, Hawbash Mohammed-Amin Rahim, Sajjad Rahimi, Vafa Rahimi-Movaghar, Amir Masoud Rahmani, Masoud Rahmati, Ghasem Rahmatpour Rokni, Diego Raimondo, Sunil Kumar Raina, Jeffrey Pradeep Raj, Adarsh Raja, Sandesh Raja, Judah Rajendran, Mohammad Amin Rajizadeh, Mahmoud Mohammed Ramadan, Kadar Ramadhan, Chitra Ramasamy, Shakthi Kumaran Ramasamy, Sheena Ramazanu, Marzieh Ramezani Farani, Chhabi Lal Ranabhat, Nemanja Rancic, Smitha Rani, Chythra R Rao, Kumuda Rao, Vahid Rashedi, Santosh Kumar Rauniyar, Ilari Rautalin, David Laith Rawaf, Reza Rawassizadeh, Ramu Rawat, Christian Razo, Wajiha

Rehman, Bhageerathy Reshmi, Stefano Restaino, Marzieh Rezaei, Mina Rezaei, Taeho Gregory Rhee, Antonio Luiz P Ribeiro, Tércia Moreira Ribeiro da Silva, Jennifer Rickard, Hannah Elizabeth Robinson-Oden, Hermano Alexandre Lima Rocha, João Rocha Rocha-Gomes, Alfonso J. Rodriguez-Morales, Leonardo Roever, Debby Syahru Romadlon, Luca Ronfani, Moustaq Karim Khan Rony, Amirhossein Roshanshad, Kunle Rotimi, Hanieh Rouzbahani, Reza Rouzbahani, Shiva Rouzbahani, Priyanka Roy, Sharmistha Roy, Shubhanjali Roy, Parameswari Royapuram Parthasarathy, Enrico Rubagotti, Michele Russo, Godfrey M Rwegerera, Aly M A Saad, Zahra Saadatian, Michela Sabbatucci, Korosh Saber, Cameron John Sabet, Siamak Sabour, Kabir P Sadarangani, Seyed Kiarash Sadat Rafiei, Basema Ahmad Saddik, Adam Saddler, Bashdar Abuzed Sadee, Tarannom Sadegh, Umar Saeed, Maryam Saeedi, Mehdi Safari, Mahdi Safdarian, Sher Zaman Safi, Rajesh Sagar, Mastooreh Sagharichi, Nondo Saha, Fatemeh Saheb Sharif-Askari, Narjes Saheb Sharif-Askari, Pragyan Monalisa Sahoo, Zahra Saif, S Mohammad Sajadi, Mirza Rizwan Sajid, Mahdi Salehi, Marwa Rashad Salem, Malik Sallam, Hossein Samadi Kafil, Abdallah M Samy, Sathish Sankar, Lucas H C C Santos, Milena M Santric-Milicevic, Jacob Owusu Owusu Sarfo, Yaser Sarikhani, Tanmay Sarkar, Mohammad Sarmadi, Arash Sarveazad, Brijesh Sathian, Maheswar Satpathy, Monika Sawhney, Christophe Schinckus, Jurgen Carlo Schmidt, Maria Inês Schmidt, Austin E Schumacher, Ghil Schwarz, David C Schwebel, Siddharthan Selvaraj, Yigit Can Senol, Subramanian Senthilkumaran, Edson Serván-Mori, Yashendra Sethi, Allen Seylani, Arezoo Shafieioun, Muhammad Shahab, Muhammad Shahbaz, Samiah Shahid, Endrit Shahini, Farshad Shahkarami, Masood Ali Shaikh, Ali Shakerimoghaddam, Sunder Sham, Muhammad Aaqib Shamim, Mehran Shams-Beyranvand, Anas Shamsi, Alfiya Shamsutdinova, Dan Shan, Amin Sharifan, Javad Sharifi Rad, Avimanu Sharma, Ravi Kumar Sharma, Vishal Sharma, Ramzi Shawahna, Maryam Shayan, Mahabalesh Shetty, Lin-Hong Shi, Belayneh Fentahun Shibesh, Md Monir Hossain Shimul, Aminu Shittu, Shayan Shojaei, Sunil Shrestha, Mohammad Sidiq, Diego Augusto Santos Silva, Luís Manuel Lopes Rodrigues Silva, Noah Joseph Bernard Silva de Leonardi, Abhinav Singh, Amit Singh, Baljinder Singh, Harmanjit Singh, Jasvinder A Singh, Kalpana Singh, Narinder Pal Singh, Samer Singh, Valentin Yurievich Skryabin, Anna Aleksandrovna Skryabina, David A Sleet, Md.Salman Sohel, Solikhah Solikhah, Aayushi Sood, Fernando Sousa, Ireneous N Soyiri, Michael Spartalis, Chandrashekhar T Sreeramareddy, Devin Bailey Srivastava, Peter Stubbs, Vetriselvan Subramaniyan, Muhammad Suleman, Haitong Zhe Sun, Xiaohui Sun, Suraj Sundaragiri, David Sunkersing, Chandan Kumar Swain, Tasmin L Symons, Lukasz Szarpak, Mindy D Szeto, Sree Sudha T Y, Rafael Tabarés-Seisdedos, Fatemeh Sadat Tabatabaei, Seyed Shahaboddin Tabatabaei, Seyyed Mohammad Tabatabaei, Shima Tabatabai, Celine Tabche, Takahiro Tabuchi, Zanan Mohammed-Ameen Taha, Yasaman Taheri Abkenar, Mircea Tampa, Ker-Kan Tan, Shynar Tanabayeva, Anika Tasnim, Jay Tewari, Chandan Kumar Thakur, Manuel Sebastian Thomas, Mariya Vladimirovna Titova, Roman Topor-Madry, Mathilde Touvier, Marcos Roberto Tovani-Palone, Mai Thi Ngoc Tran, Quynh Thuy Huong Tran, Tam Quoc Minh Tran, Domenico Trico, Indang Trihandini, Munkhtuya Tumurkhuu, Sok Cin Tye, Muhammad Umair, Muhammad Umar, Bhaskaran Unnikrishnan, Era Upadhyay, Jef Van den Eynde, Joe Varghese, Tommi Juhani Vasankari, Sampara Vasishta, Srivatsa Surya Vasudevan, Ashleigh S Vella, Balachandar Vellingiri, Narayanaswamy Venketasubramanian, Nicholas Alexander Verghese, Georgios-Ioannis Verras, David Villarreal-Zegarra, Sharath Chaitanya Vipparthy, Luciano Magalhães Vitorino, Avina Vongpradith, Linh Vu, Yasir Waheed, Megha Walia, Lindsey E Wallace, Agnes Wamuyu Wamai, Shu Wang, Youxin Wang, Yuan-Pang Wang, Stefanie Watson, Kosala Gayan Weerakoon, Daniel J Weiss, Ronny Westerman, Joanna L Whisnant, Taweewat Wiangkham, Dakshitha Praneeth Wickramasinghe, Angga Wilandika, Peter Willeit, Andrew Awuah Wireko, Gemechu Kumera Wirtu, Charles Shey Wiysonge, Axel Walter Wolf, Tewodros Eshete Wonde, Yen Jun Wong, Daniel Tarekegn Worede, Minichil Chanie Chanie Worku, Felicia Wu, Peng Wu, Zenghong Wu, Yihun Miskir Wubie, Hong Xiao, Na Xiao, Suowen Xu, Wanqing Xu, Mingyang Xue, Sajad

Yaghoubi, Kazumasa Yamagishi, Laiang Yao, Amir Yarahmadi, Habib Yaribeygi, Sanni Yaya, Pengpeng Ye, Mohammad Hossein YektaKooshali, Getaneh Atikilt Yemata, Siyan Yi, Muluken Yigezu, Malede Berihun Yismaw, Naohiro Yonemoto, Mustafa Z Younis, Abdilahi Yousuf, Chuanhua Yu, Yong Yu, Hui Yuan, Siddhesh Zadey, Vesna Zadnik, Mubashir Zafar, Emilia Zainal Abidin, Iman Zare, Kourosh Zarea, Michael Zastrozhin, Sebastian Zensen, Beijian Zhang, Jinpeng Zhang, Xiaoyi Zhang, Zhongyi Zhao, Anthony Zhong, Juexiao Zhou, Abzal Zhumagaliuly, Mohamed Ali Zoromba, Liesl J Zuhlke, Alimuddin Zumla.

Developing methods or computational machinery

Sameer Afif Ali, Aleksandr Y Aravkin, Edmond D Brewer, Austin Carter, Haley Comfort, Garland T Culbreth, Xiaochen Dai, Simon I Hay, Jiawei He, Claire A Henson, Alexander Win Hsu, Darwin Phan Jones, Nicholas J Kassebaum, Dylan Lasher, Stephen S Lim, Kelsey Lynn Maass, Erin A May, Ali H Mokdad, Christopher J L Murray, Mohsen Naghavi, Spencer A Pease, Haaris Saqib, Austin E Schumacher, Jeffrey D Stanaway, Megan Verma, Joanna L Whisnant, Peng Zheng

Providing critical feedback on methods or results

Bhoomadevi A, Mohammad Amin Aalipour, Hazim S Ababneh, Ukachukwu O Abaraogu, Cristiana Abbafati, Nasir Abbas, Mitra Abbasifard, Faezeh Abbaspour, Abdallah H A Abd Al Magied, Samar Abd ElHafeez, Mohammed Altigani Abdalla, Emad M. Abdallah, Nadin M. I. Abdel Razeq, Reda Abdel-Hameed, Wael M Abdel-Rahman, Sherief Abd-Elsalam, Omar Ahmed Abdelwahab, Parsa Abdi, Arash Abdollahi, Meriem Abdoun, Arman Abdous, Deldar Morad Abdulah, Rizwan Suliankatchi Abdulkader, Auwal Abdullahi, Abdullahi Salahudeen Abdulraheem, Habtamu Abebe Abebe Getahun, Armita Abedi, Parisa Abedi, Asrat Agalu Abejew, Roberto Ariel Abeldaño Zuñiga, Syed Hani Abidi, Alemwork Abie, Olugbenga Olusola Abiodun, Richard Gyan Aboagye, Shady Abohashem, Ulric Sena Abonie, Nagah M. Abourashed, Mohamed Abouzid, Lucas Guimarães Abreu, Dariush Abtahi, Rana Kamal Abu Farha, Fuad Hamdi A. Abuadas, Bilyaminu Abubakar, Eman Abu-Gharbieh, Sawsan Abuhammad, Ahmad Y Abuhelwa, Hana J Abukhadijah, Salahdein Aburuz, Dina Abushanab, Ahmed Abu-Zaid, Anirudh Balakrishna Acharya, Meshack Achore, Juan Manuel Acuna, Tim Adair, Lisa C Adams, Oladimeji Muritala Adebayo, Tajudeen Adesanmi Adebisi, David Adedia, Kamoru Ademola Adedokun, Oluwatobi E Adegbile, Nurudeen A Adegoke, Olumide Thomas Adeleke, Isaac Ayodeji Adesina, Miracle Ayomikun Adesina, Olatunji O Adetokunboh, Temitayo Esther Adeyeoluwa, Mache Tsadik Adhana, Kishor Adhikari, Ripon Kumar Adhikary, Mohd Adnan, Qorinah Estiningtyas Sakilah Adnani, Leticia Akua Adzigbli, David Adzrago, Giuseppina Affinito, Aanuoluwapo Adeyimika Afolabi, Rotimi Felix Afolabi, Saira Afzal, Gizachew Beykaso Agafari, Navidha Aggarwal, Mahdi Aghaalikhani, Sepehr Aghajanian, Seyed Mohammad Kazem Aghamir, Feleke Doyore Agide, César Agostinis Sobrinho, Anurag Agrawal, Williams Agyemang-Duah, Bright Opoku Ahinkorah, Aqeel Ahmad, Danish Ahmad, Faisal Ahmad, Khurshid Ahmad, Muayyad M Ahmad, Rabbiya Ahmad, Tauseef Ahmad, Waqas Ahmad, Ali Ahmed, Anisuddin Ahmed, Aram Mahmood Ahmed, Ayman Ahmed, Gasha Salih Ahmed, Haroon Ahmed, Luai A Ahmed, Mehrunnisha Sharif Ahmed, Muktar Beshir Ahmed, Mushood Ahmed, Naveed Ahmed, Oli Ahmed, Shabbir Ahmed, Syed Anees Ahmed, Gulzhanat Aimagambetova, Janardhana P Aithala, Budi Aji, Hossein Akbarialiabad, Saeid Akbarifard, Oluwasefunmi Akeju, Roland Eghoghosoa Akhigbe, Muhammad Nadeem Akhtar, Karolina Akinosoglou, Yagiz Matthew Akiska, Wole Akosile, Hammad Akram, Ashley E Akrami, Hanadi Al Hamad, Syed Mahfuz Al Hasan, Mohammad Khaled Al Nawayseh, Omar Al Omari, Mohammad Al Qadire, Zain Al Ta'ani, Yazan Al Thaher, Omar Ali Mohammed Al Zaabi, Mohammad Ahmmad Mahmoud Al Zoubi, Mousa Ali Al-Abbadi, Ziyad Al-Aly, Khurshid Alam, Manjurul Alam, Mohammad Khursheed Alam, Mostafa Alam, Rasmieh Mustafa Al-Amer, Abebaw Alamrew, Amani Alansari, Turki M Alanzi, Fahmi Y Al-Ashwal, Mohammed Albashtawy, Khalifah A Aldawsari, Robert W Aldridge, Shereen M Aleidi, Bezawit Abeje Alemayehu, Tekletsadik Tekleslassie Alemayehu, Fentahun Alemnew, Ali M Alfalki, Abdelazeem M Algammal, Mohammed Khaled Al-Hanawi, Aminu Alhassan Alhassan Ibrahim, Ashraf Alhumaidi, Fahad A. Alhumaydhi, Haroon Muhammad Ali, Irfan Ali, Kamran Ali, Maratab Ali, Mohammad Daud Ali, Mohammed Usman Ali, Rafat Ali, Shahid Ali, Syed Shujait Ali, Syed Yusuf Ali, Waad Ali, Akram Al-Ibraheem, Gianfranco Alicandro, Montaha Al-Iede, Sheikh Mohammad Alif, Hamid Alinejad Rokny, Samah W Al-Jabi, Mohamad Aljofan, Moath Saleh Aljohani, Adel Al-Jumaily, Syed Mohamed Aljunid, Mustafa Alkhawam, Atefeh Allahbakhshian, Mohammed Z. Allouh, Wesam Taher Almagharbeh, Wael Almahmeed, Sabah Al-Marwani, Joseph Uy Almazan, Hesham M Al-Mekhlafi,

Omar Almidani, Amr Almobayed, Khaldoon Aied Alnawafleh, Hasan Yaser Alniss, Margret Beaula Alocious Sukumar, Mohammad R Alosta, Jaber S Alqahtani, Saleh A Alqahtani, Mohammad R Alqudimat, Ahmad Rajeh Al-Qudimat, Rami H Al-Rifai, Intima Alrimawi, Salman Khalifah Al-Sabah, Mohammed A Alsabri, Najim Z. Alshahrani, Zaid Altaany, Awais Altaf, Alaa B Al-Tammemi, Jaffar A Al-Tawfiq, Malik A Althobiani, Khalid A Altirkawi, Javier Alvarez-Galvez, Nelson Alvis-Guzman, Mohammad Al-Wardat, Yaser Mohammed Al-Worafi, Hany Aly, Mohammad Sharif Ibrahim Alyahya, Karem H Alzoubi, Md. Akib Al-Zubayer, Uchenna Anderson Amaechi, Ekiyor Joseph Amafah, Joy Amafah, Masoud Aman Mohammadi, Faten Amer, Amr Amin, Tarek Tawfik Amin, Alireza Amindarolzarbi, Saeed Amini, Ehsan Amini-Salehi, Majid Aminzare, Sohrab Amiri, Dickson A Amugsi, Ganiyu Adeniyi Amusa, Filippos Anagnostakis, Roshan A Ananda, Nazanin Anaraki, Robert Ancuceanu, Deanna Anderlini, David B Anderson, Tudorel Andrei, Song Peng Ang, Nguyen Hoang Anh, Samuel Egyakwa Ankomah, Kabilan Annadurai, Amir Anoushiravani, Sumbul Ansari, Umair Ansari, Alireza Ansari-Moghaddam, Ernoiz Antriyandarti, Boluwatife Stephen Anuoluwa, Iyadunni Adesola Anuoluwa, Saleha Anwar, Sumadi Lukman Anwar, Razique Anwer, Shahnawaz Anwer, Anayochukwu Edward Anyasodor, Geminn Louis Carace Apostol, Hossein Arabi, Jalal Arabloo, Mosab Arafat, Demelash Areda, Jorge Arias de la Torre, Benedetta Armocida, Jesu Arockiaraj, Mahwish Arooj, Anton A Artamonov, Deepavalli Arumuganainar, Mahsa Asadi Anar, Majid Asadi-Samani, Syed Mohammed Basheeruddin Asdaq, Saeed Asgary, Mohammad Asghari-Jafarabadi, Muhammad Abdul Basit Ashraf, Syed Amir Ashraf, Tahira Ashraf, Mitra Ashrafi, Milad Ashrafizadeh, Bernard Kwadwo Yeboah Asiamah-Asare, Muhammad Shahzad Aslam, Saeed Aslani, Yuni Asri, Anil Raj Assariparambil, Dereje Zewdu Assefa, Batyrbek Assembekov, Thomas Astell-Burt, Mirbahador Athari, Maha Moh'd Wahbi Atout, Alok Atreya, Julie Alaere Atta, Zeenah A Atwan, Marcel Ausloos, Abolfazl Avan, Núbia Carelli Pereira Avelar, Sana Javaid Awan, Amlaku Mulat Aweke, Babafela B Awosile, Adedapo Wasiu Awotidebe, Beatriz Paulina Ayala Quintanilla, Fekadu Belay Ayalew, Lemessa Assefa A Ayana, Haleh Ayatollahi, Olatunde O Ayinde, Yusuf Oloruntoyin Ayipo, Berrak Itır Itır Aylı, Sina Azadnajafabad, Arian Azadnia, James Mba Azam, Alireza Azarboo, Ali Azargoonjahromi, Gulrez Shah Azhar, Farya Azimi, Mohd Yusmaidie Aziz, Sadat Abdulla Aziz, Amin Azizan, Ahmed Y Azzam, Domenico Azzolino, Abraham Samuel Babu, Giridhara Rathnaiah Babu, Ashish D Badiye, Elahe Baghizadeh, Sana Baghizadeh, Khlood K Baghlaf, Ahmed Salem BaHammam, Najmeh Bahmanziari, Razieh Bahreini, Yogesh Bahurupi, Ruhai Bai, Atif Amin Baig, Arun Balachandran, Wondu Feyisa Balcha, Jose Balmori-de-la-Miyar, Mohammadreza Balooch Hasankhani, Ovidiu Constantin Baltatu, Palash Chandra Banik, Rajon Banik, Ryan M Barber, Hiba Jawdat Barqawi, Amadou Barrow, Sandra Barteit, Zarrin Basharat, Shahid Bashir, Guido Basile, Pritish Baskaran, Mohammad-Mahdi Bastan, Abdul-Monim Batiha, Kavita Batra, Bernhard T Baune, Mahdis Bayat, Mohammad Amin Bayat Tork, Mohsen Bayati, Mulat Tirfie Bayih, Thomas Beaney, Narasimha M Beeraka, Jina Behjati, Babak Behnam, Diana Fernanda Bejarano Ramirez, Bezawit K Bekele, Almaz Nibret Belay, Asnake Gashaw Belayneh, Melesse Belayneh, Gokce Belge Bilgin, Bashir Bello, Olorunjuwon Omolaja Bello, Umar Muhammad Bello, Apostolos Beloukas, Riyad Bendardaf, Samiun Nazrin Bente Kamal Tune, Habib Benzian, Maria Bergami, Alemshet Yirga Berhie, Abiye Assefa Berihun, Amiel Nazer C Bermudez, Robert S Bernstein, Ajeet Singh Bhadoria, Akshaya Srikanth Bhagavathula, Jeetendra Bhandari, Charmi Bhanushali, Nikha Bhardwaj, Pankaj

Bhardwaj, Ashish Bhargava, Sonu Bhaskar, Arushee Bhatnagar, Priyadarshini Bhattacharjee, Shuvarthi Bhattacharjee, Gurjit Kaur Bhatti, Jasvinder Singh Bhatti, Manpreet S Singh Bhatti, Rajbir Bhatti, Soumitra S Bhuyan, Sibhatu Kassa Biadgilign, Raluca Bievel-Radulescu, Can Bilgin, Bijit Biswas, Mohammad Shahangir Biswas, Raaj Kishore Biswas, Ahmad Naoras Bitar, Molalegne Bitew, Bruno Bizzozero-Peroni, Virginia Bodolica, Mahmut Bodur, Lucimere Bohn, Obasanjo Afolabi Bolarinwa, Archith Boloor, Paria Bolourinejad, Sri Harsha Boppana, Hamed Borhany, Arturo Borzutzky, Alejandro Botero Carvajal, Souad Bouaoud, Rupert R A Bourne, Christopher Boxe, Nicola Luigi Bragazzi, Dejana Braithwaite, Hermann Brenner, Gabrielle Britton, Julie Brown, Annie J Browne, Raffaele Bugiardini, Linh Phuong Bui, Tsion Samuel Bunare, Richard A Burns, Felix Busch, Reinhard Busse, Yasser Bustanji, Nadeem Shafique Butt, Zahid A Butt, Lucero Cahuana-Hurtado, Tianji Cai, Rose Cairns, Luciana Aparecida Campos, Ismael Campos-Nonato, Chao Cao, Si Cao, Yuchen Cao, Angelo Capodici, Andre F Carvalho, Márcia Carvalho, Ana Paula Carvalho-e-Silva, Joao Mauricio Castaldelli-Maia, Giulio Castelpietra, Ferrán Catalá-López, Luca Cegolon, Francieli Cembranel, Muthia Cenderadewi, Ester Cerin, Pamela Roxana Chacón-Uscamaita, Chiranjib Chakraborty, Joht Singh Chandan, Rama Mohan Chandika, Jung-Chen Chang, Vijay Kumar Chattu, Victoria Chatzimavridou-Grigoriadou, Sirshendu Chaudhuri, Akhilanand Chaurasia, Galmesa Bekana Chemeda, An-Tian Chen, Hana Chen, Haowei Chen, Hui Chen, Meng Xuan Chen, Xiang Chen, Haojin Cheng, Nicholas WS Chew, Fatemeh Chichagi, Odgerel Chimed-Ochir, Daniel Youngwhan Cho, William C S Cho, Bryan Chong, Hitesh Chopra, Shivani Chopra, Sonali Gajanan Choudhari, Mohiuddin Ahsanul Kabir Chowdhury, Shanjida Chowdhury, Sreshtha Chowdhury, Dinh-Toi Chu, Hongyuan Chu, Isaac Sunday Chukwu, Stephen Chukwudeh, Eric Chung, Erin Chung, Sheng-Chia Chung, Sunghyun Chung, Cain C T Clark, Alyssa Columbus, Haley Comfort, Joao Conde, Nathalie Conrad, Samuele Cortese, Michael H Criqui, Natalia Cruz-Martins, Garland T Culbreth, Nour Dababo, Ali Dabbagh, Omid Dadras, Tukur Dahiru, Zainab Umar Dahiru, Xiaochen Dai, Mayank Dalakoti, Koustuv Dalal, Gloria Dalla Costa, Lalit Dandona, Rakhi Dandona, Lucio D'Anna, Pojsakorn Danpanichkul, Samuel E Danso, Samuel Demissie Darcho, Latefa Ali Dardas, Jai K Das, Dimash Davletov, Kairat Davletov, Alejandro de la Torre-Luque, Edward Christopher Dee, Sindhura Deekonda, Louisa Degenhardt, Paria Dehesh, Pouria Delbari, Mohammad Delsoz, Dessalegn Demeke, Andreas K Demetriades, Ismail Dergaa, Hunegnaw Almaw Derseh, Emina Dervišević, Hardik Dineshbhai Desai, Abraham Aregay Desta, Vinoth Gnana Chellaiyan Devanbu, Pradeep Kumar Devarakonda, Devananda Devegowda, Arkadeep Dhali, Kuldeep Dhama, Rajinder K K Dhamija, Samath Dhamminda Dharmaratne, Meghnath Dhimal, Bibha Dhungel, Marcello Di Pumpo, Diana Dias da Silva, Daniel Diaz, Diego Diaz-Milanes, Elangovan Dilipan, Lauren K Dillard, Xueting Ding, Zhendong Ding, M Ashworth Dirac, Huyen Do, Thao Huynh Phuong Do, Klara Georgieva Dokova, Mario D'Oria, Fariba Dorostkar, Ojas Prakashbhai Doshi, Robert Kokou Dowou, Judy R. Dubno, Emeka W Dumbili, Samuel C Dumith, Jennifer Dunne, Senbagam Duraisamy, Oyewole Christopher Durojaiye, Siddhartha Dutta, Angel Belle Cheng Dy, Abdel Rahman E’mar, Osamudiamen Ebohon, Ejemai Eboreime, Abdelaziz Ed-Dra, David Edvardsson, Ferry Efendi, Shayan Eghdami, Fatemeh Ehsani, Ashkan Eighaei Sedeh, Terje Andreas Eikemo, Ebrahim Eini, Michael Ekholuenetale, Temitope Cyrus Ekundayo, Rabie Adel El Arab, Maysaa El Sayed Zaki, Mohamed Ahmed Eladl, Reza Elahi, Said El-Ashker, Rana Elbeshbeishy, Faris El-Dahiyat, Marwa

Eldegwi, Marwan El-Deyarbi, Noha Mousaad Elemam, Muhammed Elhadi, Mohamed Elhoumed, Waseem El-Huneidi, Omar Abdelsadek Abdou Elmeligy, Mohamed A Elmonem, Adel B Elmoselhi, Mohamed Hassan Elnaem, Mohammed Elshaer, Ibrahim Elsohaby, Chadi Eltaha, Abdelgawad Salah Abdelgawad Eltahawy, Tadele Emagneneh, Syed Emdadul Haque, Theophilus I Emeto, Victor Oghenekparobo Emojevwe, Stanley Chinedu Eneh, Babak Eshrati, Sharareh Eskandarieh, Majid Eslami, Elochukwu Ezenwankwo, Natalia Fabin, Heidar Fadavian, Adeniyi Francis Fagbamigbe, Ayesha Fahim, Razana Faiz, Ildar Ravisovich Fakhradiyev, Aliasghar Fakhri-Demeshghieh, Luca Falzone, Qiping Fan, Mohammad Farahmand, Seyed Nooreddin Faraji, Ali Faramarzi, Mohammad Fareed, Andre Faro, Syed Muhammad Yousaf Farooq, Fatemeh Farshad, Farima Farsi, Abidemi Omolara Fasanmi, Folorunso Oludayo Fasina, Ali Fatehizadeh, Davood Fathi, Zareen Fatima, Timur Fazylov, Valery L Feigin, maryam feili, Alireza Feizkhah, Ginenus Fekadu, Xiaoqi Feng, Talukdar Raian Ferdous, Seyed-Mohammad Fereshtehnejad, Bikila Regassa Feyisa, Alexander Finnemore, Claudio Fiorilla, Ida Fitriana, Artem Alekseevich Fomenkov, Arianna Fornari, Matteo Foschi, Maryam Fotouhi, Kayode Raphael Fowobaje, Richard Charles Franklin, Alberto Freitas, Takeshi Fukumoto, Nancy Fullman, Blima Fux, Sridevi G, Peter Andras Gaal, Muktar A Gadanya, Márió Gajdács, Emmanuela Gakidou, Yaseen Galali, Dhanraj Ganapathy, Balasankar Ganesan, Xiang Gao, Yijie Gao, Bashiru Garba, Miguel Garcia-Argibay, David Garcia-Azorin, Jacopo Garlasco, Zisis Gatzioufas, Rupesh K Gautam, Bamba Gaye, Federica Gazzelloni, Hong-Han Ge, Feven Sahle Gebre, Miglas Welay Gebregergis, Haftay Gebremedhin Gebreslassie, Miesa Gelchu, Stefano Gelibter, Nsikakabasi Samuel George, Ali Gerami Matin, Lemma Getacher, Genanew K Getahun, Kalab Yigermal Gete, Delaram J Ghadimi, Arin Ghamkhar, Ali Ghandili, Moein Ghasemi, Mohammad-Reza Ghasemi, Shakiba Ghasemi Assl, Fariba Ghassemi, Ramy Mohamed Ghazy, Zainab Gholami, Elena Ghotbi, Arun Ghuge, Alessandro Gialluisi, Konstantinos Giannakis, Ruth Margaret Gibson, Syed Abdullah Gilani, Tiffany K Gill, Alem Abera Girmay, Alessandro Girombelli, Laszlo Göbölös, Anil Kumar Goel, Archit Goel, Rajesh Kumar Goel, Kimiya Gohari, Mahaveer Golechha, Ali Golestani, Mohsen Golkar, Nelson G M Gomes, Philimon N Gona, Wenping Gong, Giuseppe Gorini, Yitayal Ayalew Goshu, Alessandra C Goulart, Ayman Grada, Simon Matthew Matthew Graham, Michal Grivna, Ashna Grover, Habtamu Alganeh Guadie, Bin Guan, Shi-Yang Guan, Mohammed Ibrahim Mohialdeen Gubari, Avirup Guha, Stefano Guicciardi, Cui Guo, Xingzhi Guo, Zhaoyu Guo, Zheng Guo, Himanshu Gupta, Lalit Gupta, Rajat Das Gupta, Sapna Gupta, Roberth Steven Gutiérrez-Murillo, Jose Guzman-Esquivel, Abrham Tesfaye Tesfaye Habteyes, Awoke Derbie Derbie Habteyohannes, Tesfahun Simon Hadaro, Zahra Hadian, Faraidoon Haghdoost, Arian Haghtalab, Nguyen Hai Nam, Arvin Haj-Mirzaian, Pritam Halder, Rabih Halwani, Islam M Hamad, Randah R Hamadeh, Nadia M Hamdy, Samer Hamidi, Erin B Hamilton, Ahmad Hammoud, Mohammad Hamza, Didem Han Yekdeş, Asif Hanif, Nasrin Hanifi, Fahad Hanna, Ashanul Haque, Md Nuruzzaman Haque, Obaid I Haque, Harapan Harapan, Hilda L Harb, Arief Hargono, Eka Mishbahatul Marah Has, Ahmed I Hasaballah, Faizul Hasan, Md Kamrul Hasan, Towhid Hasan, Hamidreza Hasani, Ali Hasanpour- Dehkordi, Arezou Hashem Zadeh, Mohammad Hashem Hashempur, Nada Tawfig Hashim, Ammarah Hasnain, Ibrahim Nagmeldin Hassan, Ikrama Hassan, Yusuf Wada Hassan Wada, Mahgol Sadat Hassan Zadeh Tabatabaei, Rasmus J Havmoeller, Simon I Hay, Khezar Hayat, Jeffrey J Hebert, Golnaz Heidari, Mohammad Heidari,

Mehdi Hemmati, Claudiu Herteliu, Hamed Hesami, Sumudu Avanthi Hewage, Majid Heydari, Zahra Heydarifard, Yuta Hiraike, Ramesh Holla, Nobuyuki Horita, Alamgir Hossain, Lubna Hossain, Md Belal Hossain, Md Mahbub Hossain, Md Sabbir Hossain, Mohammad Bellal Hossain, Mihaela Hostiuc, Peter J Hotez, Amir Human Hoveidaei, Hanno Hoven, Alexander Win Hsu, Chengxi Hu, Guoqing Hu, Junjie Huang, Weijun Huang, Yefei Huang, Zhenyao Huang, Mega Hasanul Huda, Ayesha Humayun, Waqar Husain, Kiavash Hushmandi, Javid Hussain, Nawfal R Hussein, Mohamed Ibrahim Husseiny, Hong-Han Huynh, Bing-Fang Hwang, Luigi Francesco Iannone, Segun Emmanuel Ibitoye, Ismail A. Atef Ismail Ahmed Ibrahim, Ramzi Ibrahim, Umar Idris Ibrahim, Fidelia Ida, Kevin S Ikuta, Olayinka Stephen Ilesanmi, Irena M Ilic, Milena D Ilic, Masoud Imani, Mustapha Immurana, Lucius Chidiebere Imoh, Leeberk Raja Inbaraj, Arit Inok, Mujahid Iqbal, Mustafa Alhaji Isa, Teresa R Iskander, Dr. Md. Shahinul Islam, Md Rabiul Islam, Md Shariful Islam, Farhad Islami, Nahlah Elkudssiah Ismail, Gaetano Isola, Masao Iwagami, Ihoghosa Osamuyi Iyamu, Mahalaxmi Iyer, Vinothini J, Udeme Samuel Jacob, Kathryn H. Jacobsen, Mohammadsadegh Jafari, Ali Jafari-Khounigh, Morteza Jafarinia, Vennila Jaganathan, Haitham Jahrami, Ayushi Jain, Ammar Abdulrahman Jairoun, Mihajlo Jakovljevic, Ali Jaliliyan, Mohamed Jalloh, Armaan Jamal, Melika Jameie, Jerin James, Safayet Jamil, Masoud Jamshidi, Shaghayegh JamshidiRastabi, Esmaeil Jarrahi, Tahereh Javaheri, Syed Sarmad Javaid, Anita Javanmardi, Javad Javidnia, Shubha Jayaram, Ruwan Duminda Jayasinghe, Yovanthi Anurangi Jayasinghe, Achala Upendra Jayatilleke, Felix K Jebasingh, Jayakumar Jeganathan, Seogsong Jeong, Bijay Mukesh Jeswani, Zixiang Ji, Min Jiang, Shuai Jin, Wenyi Jin, Mohammad Jokar, Jost B Jonas, Darwin Phan Jones, Tamas Joo, Abu Jor, Abel Joseph, Nitin Joseph, Charity Ehimwenma Joshua, Kripa Josten, George Joy, Jacek Jerzy Jozwiak, Malik E Juweid, Zubair Kabir, Dler H. Hussein Kadir, Ashish Kumar Kakkar, Pradnya Vishal Kakodkar, Khalil Kalavani, Md Moustafa Kamal, Mehnaz Kamal, Sivesh Kathir Kamarajah, Rajesh Kamath, Saltanat Kamenova, Ramat T. Kamorudeen, Devanish Narasimhasanth Kamtam, Naser Kamyari, Oleksandr Kamyshnyi, Mona Kanaan, Jiseung Kang, Samuel Berchi Kankam, Kehinde Kazeem Kanmodi, Rami S Kantar, Neeti Kapoor, Sujita Kumar Kar, Paschalis Karakasis, Mohammad Amin Karimi, Salah Eddin Karimi, Mohmed Isaqali Karobari, Tomasz M Karpiński, Sadanand Karun, Manoj Kumar Kashyap, Eden Asmare Kassahun, Nigussie Assefa Kassaw, Nicholas J Kassebaum, Adarsh Katamreddy, Kanica Kaushal, Foad Kazemi, Nastaran Kazemi rad, Sina Kazemian, Chukwudi Keke, John H Kempen, Jessica A Kerr, Emmanuelle Kesse-Guyot, Reza Khademi, Himanshu Khajuria, Nauman Khalid, Hazim O. Khalifa, Anas Husam Khalifeh, Anees Ahmed Khalil, Pantea Khalili, Alireza Khalilian, Ghazaleh Khalili-Tanha, Mohamed khalis, Faham Khamesipour, Abdul A Khan, Ajmal Khan, Fayaz Khan, Iman Waheed Khan, Maseer Khan, Md Abdullah Saeed Khan, Mohammad Jobair Khan, Muhammad Hamza Khan, Muhammad Mueed Khan, Muhammad Umer Khan, Ramsha Mushtaq Khan, Serab Khan, Sumaiya Khan, Zahid Khan, Zahid Khan, Srijana Khanal, Shaghayegh Khanmohammadi, Zenith Khashim, Khaled Khatab, Haitham Khatatbeh, Moawiah Mohammad Khatatbeh, Kavin Khatri, Khalid A Kheirallah, Sunil Kumar Khokhar, Mohammad Saeid Khonji, Zahra Khorrami, Najmaddin Salih Husen S.H. Khoshnaw, Atulya Aman Khosla, Majid Khosravi, Mahmood Khosrowjerdi, Jagdish Khubchandani, Zemene Demelash Kifle, Hye Jun Kim, Jinho Kim, Min Seo Kim, Yun Jin Kim, Ruth W Kimokoti, Yohannes Kinfu, Sanjay Kini B, Adnan Kisa, Sezer Kisa, Ladli Kishore, Juniper Boroka Kiss, Mika

Kivimäki, Shivakumar KM, Ann Kristin Skrindo Knudsen, Sonali Kochhar, Michail Kokkorakis, Ali-Asghar Kolahi, Diana Gladys Kolieghu Tcheumeni, Farzad Kompani, Aida Kondybayeva, Anastasios Georgios Panagiotis Konstas, Isaac Koomson, Tapos Kormoker, Oleksii Korzh, Archana Koul, Sindhura Lakshmi Koulmane Laxminarayana, Irene Akwo Kretchy, James-Paul Kretchy, Kewal Krishan, Chong-Han Kua, Barthelemy Kuate Defo, Mohammed Kuddus, Ilari Kuitunen, Shikha Kukreti, Shweta Kulshreshtha, Avinash Kumar, Dewesh Kumar, G Anil Kumar, Jogender Kumar, Manasi Kumar, Nithin Kumar, Tushar Kumar, Vijay Kumar, Jibin Kunjavara, Setor K Kunutsor, Maria Dyah Kurniasari, Pramod Kumar Kushawaha, Asep Kusnali, Christina Yeni Yeni Kustanti, Dian Kusuma, Tezer Kutluk, Evans F Kyei, Grace Kwakyewaa Kyei, Frank Kyei-Arthur, Ville Kytö, Hmwe Hmwe Kyu, Pallavi L C, Adriano La Vecchia, Carlo La Vecchia, Alessio Lachi, Muhammad Awwal Ladan, Chandrakant Lahariya, Daphne Teck Ching Lai, Anita Lakhani, Dharmesh Kumar Lal, Ratilal Lalloo, Tea Lallukka, Iván Landires, Berthold Langguth, Dylan Lasher, Kamaluddin Latief, Mahrukh Latif, Colleen L L Lau, Saheed Akinmayowa Lawal, Aliyu Lawan, Huu-Hoai Le, Minh Huu Nhat Le, Nhi Huu Hanh Le, Thao Thi Thu Le, Trang Diep Thanh Le, Caterina Ledda, Ivan Lee, Seung Won Lee, Wei-Chen Lee, Yo Han Lee, Vasileios Leivaditis, Matthew J Lennon, Elvynna Leong, An Li, Chengfeng Li, Hui Li, Jianan Li, Jiaying Li, Jinbo Li, Ming-Chieh Li, Wang-Zhong Li, Wei Li, Wei Li, Weilong Li, Yongze Li, Zhaolong Adrian Li, Zhengrui Li, Yanxue Lian, Xue-Zhen Liang, Virendra S Ligade, Stephen S Lim, Jialing Lin, Queran Lin, Ro-Ting Lin, Shuzhi Lin, Daniel Lindholm, Yuewei Ling, Gang Liu, Haipeng Liu, Jue Liu, Xianliang Liu, Xiaofeng Liu, Xuefeng Liu, Yubo Liu, Zhe Liu, Erand Llanaj, Valerie Lohner, José Francisco López-Gil, Masoud Lotfizadeh, Surbala Devi Lourembam, Rafael Lozano, Shanjie Luan, Jailos Lubinda, Giancarlo Lucchetti, Angelina M Lutambi, Miltiadis D Lytras, Ellina Lytvyak, Hawraz Ibrahim M. Amin, Kevin Sheng-Kai Ma, Zheng Feei Ma, Kelsey Lynn Maass, Mahmoud Mabrok, Nikolaos Machairas, Monika Machoy, Firoozeh Madadi, Seyed Ataollah Madinezad, Aurea Marilia Madureira-Carvalho, Pasquale Maffia, Azzam A Maghazachi, D. R. Mahadeshwara Prasad, Sasikumar Mahalingam, Preeti Maharjan, Mina Maheri, Nozad Hussein Mahmood, Alireza Mahmoudi, Farhad Mahmoudi, Rituparna Maiti, Abdelrahman M Makram, Reza Malekzadeh, Hardeep Singh Malhotra, Ahmad Azam Malik, Farihah Malik, Deborah Carvalho Malta, Mustapha Mangdow, Lokesh Manjani, Yosef Manla, Fahmida Mannan, Kamaruddeen Mannethodi, Farheen Mansoor, Marjan Mansourian, Mohammad Ali Mansournia, Lorenzo Giovanni Mantovani, Changkun Mao, Tahir Maqbool, Hamid Reza Marateb, Joemer C Maravilla, Konstantinos Margetis, Mirko Marino, Adilson Marques, Bernardo Alfonso Martinez-Guerra, Ramon Martinez-Piedra, Daniela Martini, Francisco Rogerlândio Martins-Melo, Miquel Martorell, Roy Rillera Marzo, Sammer Marzouk, Sugeng Mashudi, Soroush Masrouri, Clara N Matei, Yasith Mathangasinghe, Stephanie Mathieson, Alexander G Mathioudakis, Medha Mathur, Neeta Mathur, Fernanda Penido Matozinhos, Rita Mattiello, Khurshid A Mattoo, Richard James Maude, Erin A May, Mahsa Mayeli, Mohsen Mazidi, John J McGrath, Martin McKee, Michael A McPhail, Steven M McPhail, Enkeleint A Mechili, Rishi P Mediratta, Riffat Mehboob, Ravi Mehrotra, Vini Mehta, Tesfahun Mekene Meto, Berhanu Abebaw Mekonnen, Hadush Negash Meles, Addisu Melese, Satish Melwani, Walter Mendoza, Godfred Antony Menezes, Emiru Ayalew Mengistie, Sultan Ayoub Ayoub Meo, Michelangelo Mercogliano, Atte Meretoja, Tuomo J Meretoja, Tomislav Mestrovic, Chamila Dinushi Kukulege Mettananda, Sachith Mettananda, Mohamed M. M. Metwally,

Tomasz Miazgowski, Irmina Maria Michalek, Andrea Michelerio, Hiwot Soboksa Mideksa, Ted R Miller, Giuseppe Minervini, GK Mini, Seyed Ali Mirshahvalad, Mizan Kiros Mirutse, Maryam Mirzaei, Awoke Misganaw, Archana Mishra, Philip B Mitchell, Sayan Mitra, Chaitanya Mittal, Shivani Modi, Jama Mohamed, Mona Gamal Mohamed, Nouh Saad Mohamed, Khabab Abbasher Hussien Mohamed Ahmed, Taj Mohammad, Sakineh Mohammad-Alizadeh-Charandabi, Abdollah Mohammadian-Hafshejani, Ibrahim Mohammadzadeh, Abdulwase Mohammed, Ammas Siraj Mohammed, Mustapha Mohammed, Omer Mohammed, Shafiu Mohammed, Suleiman Mohammed, Yahaya Mohammed, Mohammad Mohseni, Ali H Mokdad, Sabrina Molinaro, Amirabbas Mollaei, Shaher Momani, Himel Mondal, Stefania Mondello, Mohammad Ali Moni, Marco Montalti, Yousef Moradi, Maziar Moradi-Lakeh, Paula Moraga, Rafael Silveira Moreira, Mahmoud M Morsy, Reza Mosaddeghi Heris, Jonathan F Mosser, Elias Mossialos, Simin Mouodi, Mariana Mourgova, Asma Mousavi, Seyede Zohre Mousavi, Amin Mousavi Khaneghah, Seyed Mohamad Sadegh Mousavi Kiasary, Hagar Lotfy Mowafy, Kimia Mozahheb Yousefi, Rabia Mubarak, Faraz Mughal, Syed Aun Muhammad, Oscar J Mujica, Sukhes Mukherjee, Sumoni Mukherjee, Amartya Mukhopadhyay, George Duke Mukoro, M A Muktadir, Francesk Mulita, Chalie Mulugeta, Mulyadi Mulyadi, Malaisamy Muniyandi, Kavita Munjal, Yanjinlkham Munkhsaikhan, Javier Muñoz Laguna, Michio Murakami, Efren Murillo-Zamora, Christopher J L Murray, Ali Mushtaq, Sherzad Ibrahim Mustafa, Mubarak Taiwo Mustapha, Sathish Muthu, Saravanan Muthupandian, Claude Mambo Muvunyi, Muhammad Muzaffar, Amin Nabavi, Ahamarshan Jayaraman Nagarajan, Shankar Prasad Nagaraju, Mohsen Naghavi, Ganesh R Naik, Firzan Nainu, Hastyar Hama Rashid Najmuldeen, Gopal Nambi, Vinay Nangia, Jobert Richie Nansseu, Yvonne Nartey, Bruno Ramos Nascimento, Abdallah Y Naser, Abdulqadir J Nashwan, Hamide Nasiri, Mahmoud Nassar, Zuhair S Natto, Zakira Naureen, Samidi Nirasha Kumari Navaratna, Biswa Prakash Nayak, Shalini Ganesh Nayak, Smitha Nayak, Shumaila Naz, Amanuel Tebabal Nega, Masoud Negahdary, Wubshet D Negash, Ionut Negoi, Ruxandra Irina Negoi, Jalil Nejati, Nikita A Nekliudov, Henok Biresaw Netsere, Charles Richard James Newton, Marie Ng, Josephine W Ngunjiri, Cuong Tat Nguyen, Dang Nguyen, Long Nguyen, Nghia Phu Nguyen, The Phuong Nguyen, Tu Anh Nguyen, Van Thanh Nguyen, Ambe Marius Ngwa, Robina Khan Niazi, Luciano Nieddu, Ali Nikoobar, Vikram Niranjan, Abebe Melis Nisro, Chukwudi A Nnaji, Shuhei Nomura, Syed Toukir Ahmed Noor, Sana Noreen, Masoud Noroozi, Jean Jacques Noubiap, Valentine C Nriagu, Chisom Adaobi Nri-Ezedi, Jean Claude Nshimiyimana, Mpiko Ntsekhe, Fred Nugen, Atoma Negera Nugusa, Mengistu H Nunemo, Sylvester Dodzi Dodzi Nyadanu, Felix Kwasi Nyande, Ogochukwu Janet Nzoputam, Bogdan Oancea, Ismail A Odetokun, Joseph Kojo Oduro, Michael Safo Oduro, Akinyemi O D Ofakunrin, Onome Bright Oghenetega, Oluwafunmilayo Tosin Ogundeko-Olugbami, Sarah Oh, Hassan Okati-Aliabad, Olalekan John Okesanya, Osaretin Christabel Okonji, Oluwaseyi Isaiah Olabisi, Andrew T Olagunju, Oladotun Victor Olalusi, Matthew Idowu Olatubi, Gláucia Maria Moraes Oliveira, Abdulhakeem Abayomi Olorukooba, Oluseye Olalekan Oludoye, Bolajoko Olubukunola Olusanya, Jacob Olusegun Olusanya, Goran Latif Omer, Sandersan Onie, Obinna E Onwujekwe, Marcel Opitz, Aksoltan Shyhdurdyevna Oradova, Michal Ordak, Atakan Orscelik, John W Ostrominski, Uchechukwu Levi Osuagwu, Olayinka Osuolale, Godfred Otchere, Elham H Othman, Adrian Otoiu, Oche Joseph Otorkpa, Abdu Oumer, Jerry John Ouner, Amel Ouyahia, Mayowa O Owolabi, Irene

Amoakoh Owusu, Kolapo Oyebola, Tope Oyelade, Oyetunde T Oyeyemi, Ilker Ozsahin, Mahesh P A, Jagadish Rao Padubidri, Dimpal Manilal Paija, Tamás Palicz, Tejasri Paluvai, Feng Pan, Sujogya Kumar Panda, Songhomitra Panda-Jonas, Seithikurippu R Pandi-Perumal, Carlo Irwin Able Panelo, Georgios D Panos, Leonidas D Panos, Ioannis Pantazopoulos, Anca Pantea Stoian, Giovanni Paolino, Ilias Papadimopoulos, Paraskevi Papadopoulou, Parinaz Paranjkhoo, Shahina Pardhan, Peyvand Parhizkar Roudsari, Romil R Parikh, Chulwoo Park, Eun-Kee Park, Seoyeon Park, Arpit Parmar, Swapnil Parve, Maja Pasovic, Roberto Passera, Bhumi Hemal Patel, Hemal M Patel, Heta Pavan Patel, Mitesh Patel, Neel Navinkumar Patel, Riya Jayesh Patel, Sangram Kishor Patel, Satyananda Patel, Angel J Paternina-Caicedo, Ashlesh Patil, Shankargouda Patil, Apurba Patra, Venkata Suresh Patthipati, Shrikant Pawar, Shubhadarshini Pawar, Spencer A Pease, Amy E Peden, Paolo Pedersini, Jarmila Pekarcikova, Emmanuel K Peprah, Prince Peprah, Gavin Pereira, Maria Odete Pereira, Pablo Perez-Lopez, Pavlo Petakh, Olumuyiwa James Peter, Fanny Emily Petermann-Rocha, Hoang Nhat Pham, Hoang Tran Pham, Nhat Truong Pham, Tung Thanh Pham, Anil K Philip, Michael R Phillips, Zayar Phyo, David M Pigott, Zahra Zahid Piracha, Moein Piroozkhkah, Enrico Pisoni, Florian Ploeckl, Evgenii Plotnikov, Roman V Polibin, Ramesh Poluru, Ville T Ponkilainen, Ion Popa, Sajjad Pourasghary, Reza Pourbabaki, Farzad Pourghazi, Naeimeh Pourtaheri, Sergio I Prada, Jalandhar Pradhan, Pranil Man Singh Pradhan, Akila Prashant, Elton Junio Sady Prates, Harsh Priya, Nicola Riccardo Pugliese, Hery Purnobasuki, Shuby Puthussery, Jagadeesh Puvvula, Nameer Hashim Qasim, Xiang Qi, Zhipeng Qi, Jia-Yong Qiu, Zahiruddin Syed Quazi, Basuki Rachmat, Raghu Anekal Radhakrishnan, Hadi Raeisi Shahraki, Pracheth Raghuveer, Hawbash Mohammed-Amin Rahim, Sajjad Rahimi, Vafa Rahimi-Movaghar, Fryad Majeed Rahman, Mahbubur Rahman, Md Mijanur Rahman, Md. Mosfequr Rahman, Mohammad Hifz Ur Rahman, Mosiur Rahman, Muhammad Aziz Rahman, Amir Masoud Rahmani, Saeed Rahmani, Masoud Rahmati, Ghasem Rahmatpour Rokni, Hakim Rahmoune, Diego Raimondo, Sunil Kumar Raina, Jeffrey Pradeep Raj, Adarsh Raja, Sandesh Raja, Erta Rajabi, Gunaseelan Rajendran, Judah Rajendran, Mahmoud Mohammed Ramadan, Majed Ramadan, Kadar Ramadhan, Chitra Ramasamy, Shakthi Kumaran Ramasamy, Zahra Ramezani, Juwel Rana, Chhabi Lal Ranabhat, Nemanja Rancic, Smitha Rani, Chythra R Rao, Kumuda Rao, Mithun Rao, Vahid Rashedi, Mohammad-Mahdi Rashidi, Ashkan Rasouli-Saravani, Prateek Rastogi, Azad Rasul, Devarajan Rathish, Abdur Rauf, Santosh Kumar Rauniyar, Ilari Rautalin, Ramin Ravangard, David Laith Rawaf, Reza Rawassizadeh, Ramu Rawat, Bahman Razi, Christian Razo, Murali Mohan Rama Krishna Reddy, Elrashdy Redwan, Sanika Rege, Wajiha Rehman, Rainer Reile, Bhageerathy Reshmi, Stefano Restaino, Marzieh Rezaei, Mina Rezaei, Nazila Rezaei, Mohsen Rezaeian, Taeho Gregory Rhee, Tércia Moreira Ribeiro da Silva, Jennifer Rickard, Hermano Alexandre Lima Rocha, João Rocha Rocha-Gomes, Alfonso J. Rodriguez-Morales, Leonardo Roever, Ravi Rohilla, Iftitakhur Rohmah, Susanne Röhr, David Rojas-Rueda, Megan L Rolfzen, Debby Syahru Romadlon, Michele Romoli, Moustaq Karim Khan Rony, Amirhossein Roshanshad, Kunle Rotimi, Himanshu Sekhar Rout, Hanieh Rouzbahani, Reza Rouzbahani, Shiva Rouzbahani, Adrija Roy, Parimal Roy, Priyanka Roy, Sharmistha Roy, Shubhanjali Roy, Simanta Roy, Simanta Roy, Parameswari Royapuram Parthasarathy, Enrico Rubagotti, Susan Fred Rumisha, Michele Russo, Godfrey M Rwegerera, Poorvikha S, Aly M A Saad, Zahra Saadatian, Michela Sabbatucci, Korosh Saber, Maha Mohamed Saber-Ayad, Cameron John

Sabet, Siamak Sabour, Perminder S Sachdev, Kabir P Sadarangani, Seyed Kiarash Sadat Rafiei, Basema Ahmad Saddik, Adam Saddler, Bashdar Abuzed Sadee, Tarannom Sadegh, Ehsan Sadeghi, Erfan Sadeghi, Fatemeh Sadeghi-Ghyassi, Mohd Saeed, Umar Saeed, Maryam Saeedi, Mehdi Safari, Mahdi Safdarian, Sher Zaman Safi, Rajesh Sagar, Mastooreh Sagharichi, Dominic Sagoe, Nondo Saha, Fatemeh Saheb Sharif-Askari, Narjes Saheb Sharif-Askari, Gülsüm Şahin Bodur, Pragyan Monalisa Sahoo, Zahra Saif, S Mohammad Sajadi, Md Refat Uz Zaman Sajib, Mirza Rizwan Sajid, Payman Salamati, Mohamed A Saleh, Mahdi Salehi, Marwa Rashad Salem, Sohrab Salimi, Pegah Salimi Pormehr, Malik Sallam, Hossein Samadi Kafil, Saad Samargandy, Yoseph Leonardo Samodra, Abdallah M Samy, Elaheh Sanjari, Sathish Sankar, Francesca Sanna, Lucas H C C Santos, Milena M Santric-Milicevic, Sivan Yegnanarayana Iyer Saraswathy, Jacob Owusu Owusu Sarfo, Yaser Sarikhani, Tanmay Sarkar, Hemen Sarma, Mohammad Sarmadi, Gargi Sachin Sarode, Sachin C Sarode, Michele Sassano, Brijesh Sathian, Mukesh Kumar Sathya Narayanan, Maheswar Satpathy, Jennifer Saulam, Mehrdad Savabi Far, Kimia Savoji, Monika Sawhney, Ganesh Kumar Saya, Abu Sayeed, Christophe Schinckus, Austin E Schumacher, Aletta Elisabeth Schutte, Ghil Schwarz, David C Schwebel, Falk Schwendicke, Catherine Schwinger, Mario Šekerija, Siddharthan Selvaraj, Yuliya Semenova, Mohammad H Semreen, Ashenafi Kibret Sendekie, Yigit Can Senol, Subramanian Senthilkumaran, Sadaf G Sepanlou, Edson Serván-Mori, Yashendra Sethi, Seyed Mohammad Seyed Alshohadaei, Abubakar Sha'aban, Mahan Shafie, Arezoo Shafieioun, Muhammad Shahab, Shazlin Shaharudin, Muhammad Shahbaz, Samiah Shahid, Syed Ahsan Shahid, Wajeehah Shahid, Endrit Shahini, Farshad Shahkarami, Fatemeh Shahrahmani, Hamid R Shahsavari, Masood Ali Shaikh, Nafhat Shaikh, Alireza Shakeri, Ali Shakerimoghaddam, Ali S Shalash, Muhammad Aaqib Shamim, Mehran Shams-Beyranvand, Anas Shamsi, Dan Shan, Mohd Shanawaz, Abhishek Shankar, Amin Sharifan, Javad Sharifi Rad, Avimanu Sharma, Bhoopesh Kumar Sharma, Bunty Sharma, Ravi Kumar Sharma, Ujjawal Sharma, Vishal Sharma, Armin Shavandi, Ramzi Shawahna, Maryam Shayan, Ali Sheidaei, Aziz Sheikh, Fang Shi, Lin-Hong Shi, Belayneh Fentahun Shibesh, Desalegn Shiferaw, Tariku Shimels, Md Monir Hossain Shimul, Min-Jeong Shin, Rahman Shiri, Reza Shirkoohi, Aminu Shittu, Ivy Shiue, Ambreen Shoaib, Shayan Shojaei, Sina Shool, Seyed Afshin Shorofi, Sunil Shrestha, Suleiman Adeiza Adeiza Shuaibu, Kerem Shuval, Nicole R S Sibuyi, Emmanuel Edwar Siddig, Mohammad Sidiq, Luís Manuel Lopes Rodrigues Silva, Abhinav Singh, Amit Singh, Balbir Bagicha Singh, Baljinder Singh, Harmanjit Singh, Harpreet Singh, Jasvinder A Singh, Kalpana Singh, Narinder Pal Singh, Samer Singh, Satwinder Singh, Surendra Singh, Mukesh Kumar Sinha, Freddy Sitas, Dagne Feleke Siyoum, Natia Skhvitaridze, Valentin Yurievich Skryabin, Anna Aleksandrovna Skryabina, David A Sleet, Md.Salman Sohel, Solikhah Solikhah, Sameh S M Soliman, Aayushi Sood, Prashant Sood, Soroush Soraneh, Joan B Soriano, Fernando Sousa, Michael Spartalis, Chandrashekhar T Sreeramareddy, Shyamkumar Sriram, Devin Bailey Srivastava, Nicholas Steel, Aleksandar Stevanović, Sebastian Straube, Peter Stubbs, Omer Subasi, Vetriselvan Subramaniyan, Hasnat Sujon, Muhammad Suleman, Desy Sulistiyorini, Mark J M Sullman, Haitong Zhe Sun, Jing Sun, Mao-ling Sun, Xiaohui Sun, Zhuanlan Sun, Suraj Sundaragiri, David Sunkersing, Sumam Sunny, Chandan Kumar Swain, Lukasz Szarpak, Mindy D Szeto, Sree Sudha T Y, Payam Tabaee Damavandi, Rafael Tabarés-Seisdedos, Fatemeh Sadat Tabatabaei, Seyed Shahaboddin Tabatabaei, Seyyed Mohammad

Tabatabaei, Shima Tabatabai, Celine Tabche, Ramin Tabibi, Mohammad Tabish, Takahiro Tabuchi, Santosh Kumar Tadakamadla, Buhari Abdullahi Tafida, Farzad Taghizadeh-Hesary, Zanan Mohammed-Ameen Taha, Yasaman Taheri Abkenar, Shima Tajabadi, Iman M Talaat, Mircea Tampa, Jacques Lukenze Tamuzi, Ker-Kan Tan, Shynar Tanabayeva, Guodong Tang, Haosu Tang, Mohsan Tanveer, Sarvenaz Taridashti, Ingan Ukur Tarigan, Mengistie Kassahun Tariku, Saba Tariq, Anika Tasnim, Seyed Mohammad Tavangar, Mebrahtu G. Tedla, Mohamad-Hani Temsah, Masayuki Teramoto, Azimeraw Arega Tesfu, Jay Tewari, Alireza Teymouri, Rekha Thapar, Ismaeel Tharwat, Samar Tharwat, Hadiza Theyra-Enias, Arun James Thirunavukarasu, Manuel Sebastian Thomas, Jansje Henny Vera Ticoalu, Mariya Vladimirovna Titova, Yves Joel Tochie Noutakdie, Marcello Tonelli, Roman Topor-Madry, Ali Torkashvand, Mathilde Touvier, Marcos Roberto Tovani-Palone, Khaled Trabelsi, Mai Thi Ngoc Tran, Quynh Thuy Huong Tran, Tam Quoc Minh Tran, Nguyen Tran Minh Duc, Domenico Trico, Indang Trihandini, Samuel Joseph Tromans, Claudia Truppa, Gary Tse, Evangelia Eirini Tsermpini, Munkhtuya Tumurkhuu, Zhouting Tuo, Biruk Shalmeno Tusa, Sok Cin Tye, Stefanos Tyrovolas, Aniefiok John Udoakang, Atta Ullah, Himayat Ullah, Riaz Ullah, Saeed Ullah, Muhammad Umair, Muhammad Umar, Muhammad Umar, Bhaskaran Unnikrishnan, Dinesh Upadhya, Era Upadhyay, Dipan Uppal, Jibrin Sammani Usman, Kelechi Julian Uzor, Hande Uzunçıbuk, Pascual R Valdez, Zahir Vally, Jef Van den Eynde, Joe Varghese, Priya Vart, Santosh Varughese, Sampara Vasishta, Srivatsa Surya Vasudevan, Alireza Vaysi, Ashleigh S Vella, Balachandar Vellingiri, Gowri Venkatraman Subramanian, Narayanaswamy Venketasubramanian, Madhur Verma, Megan Verma, Massimiliano Veroux, Georgios-Ioannis Verras, Dominique Vervoort, Ramesh Vidavalur, Simone Villa, Jorge Hugo Villafañe, David Villarreal-Zegarra, Francesco S Violante, Sharath Chaitanya Vipparthy, Luciano Magalhães Vitorino, Stein Emil Vollset, Theo Vos, Elpida Vounzoulaki, Linh Vu, Henok Toga Wada, Yasir Waheed, Megha Walia, Agnes Wamuyu Wamai, Jin-Yi Wan, Arvinder Wander, Fang Wang, Jinyu Wang, Qingzhi Wang, Ruixuan Wang, Shaopan Wang, Shu Wang, Wanzhou Wang, Wei Wang, Xing Wang, Yanzhong Wang, Youxin Wang, Yuan-Pang Wang, Mary Njeri Wanjau, Ahmed Bilal Waqar, Muhammad Waqas, Paul Ward, Stefanie Watson, Kosala Gayan Weerakoon, Fei-Long Wei, Xueying Wei, Ronny Westerman, Joanna L Whisnant, Taweewat Wiangkham, Yohanes Cakrapradipta Wibowo, Anggi Lukman Wicaksana, Dakshitha Praneeth Wickramasinghe, Nuwan Darshana Darshana Wickramasinghe, Angga Wilandika, Peter Willeit, Andrew Awuah Wireko, Gemechu Kumera Wirtu, Charles Shey Wiysonge, Abay Tadesse Woday, Marcin W Wojewodzic, Axel Walter Wolf, Tewodros Eshete Wonde, Yen Jun Wong, Daniel Tarekegn Worede, Minichil Chanie Chanie Worku, Felicia Wu, James Fan Wu, Jinyi Wu, Peng Wu, Yihun Miskir Wubie, Qing Xia, Zhijia Xia, Guangqin Xiao, Hong Xiao, Lishun Xiao, Na Xiao, Wanqing Xie, Site Xu, Suowen Xu, Wanqing Xu, Xiaoyue Xu, Mingyang Xue, Mukesh Kumar Yadav, Haibo Yang, Yuichiro Yano, Haiqiang Yao, Laiang Yao, Amir Yarahmadi, Habib Yaribeygi, Haya Yasin, Mohamed A Yassin, Yuichi Yasufuku, Sanni Yaya, Pengpeng Ye, Meghdad Yeganeh, Ali Cem Yekdeş, Mohammad Hossein YektaKooshali, Getaneh Atikilt Yemata, Subah Abderehim Yesuf, Saber Yezli, Siyan Yi, Muluken Yigezu, Dehui Yin, Malede Berihun Yismaw, Yazachew Engida Yismaw, Dong Keon Yon, Naohiro Yonemoto, Mustafa Z Younis, Abdilahi Yousuf, Chuanhua Yu, Jian Yu, Yong Yu, Hui Yuan, Ghazala Yunus, Umar Yunusa, Siddhesh Zadey, Vesna Zadnik, Mubashir Zafar, Manijeh Zaghampour, Mondal Hasan Zahid, Emilia

Zainal Abidin, Fathiah Zakham, Giulia Zamagni, Sojib Bin Zaman, Abu Sarwar Zamani, Hussaini Zandam, Kourosh Zarea, Shirin Zaresharifi, Michael Zastrozhin, Mohammed Zawiah, Mohammed G M Zeariya, Dawit Zemedikun, Abay Mulu Zenebe, Sebastian Zensen, Eyael M Zeru, Tiansong Zhan, Yongle Zhan, Beijian Zhang, Casper J P Zhang, Haijun Zhang, Jinpeng Zhang, Liqun Zhang, Meixin Zhang, Ning Zhang, Xiaoyi Zhang, Xiu-Hang Zhang, Yunquan Zhang, Zhiqiang Zhang, Sheng Zhao, Shenglin Zhao, Zhongyi Zhao, Ming-Hua Zheng, Peng Zheng, Anthony Zhong, Claire Chenwen Zhong, Jiayan Zhou, Juexiao Zhou, Bin Zhu, Mohamed Ali Zoromba, Rafat Mohammad Zrieq, Liesl J Zuhlke, Lilik Zuhriyah, Alimuddin Zumla, Ahed H Zyoud, Sa'ed H Zyoud, Shaher H Zyoud, Nawsherwan

Drafting the work or revising it critically for important intellectual content

Bhoomadevi A, Mohammad Amin Aalipour, Hasan Aalruz, Hazim S Ababneh, Cristiana Abbafati, Mitra Abbasifard, Faezeh Abbaspour, Abdallah H A Abd Al Magied, Samar Abd ElHafeez, Emad M. Abdallah, Nadin M. I. Abdel Razeq, Wael M Abdel-Rahman, Sherief Abd-Elsalam, Omar Ahmed Abdelwahab, Parsa Abdi, Arman Abdous, Auwal Abdullahi, Armita Abedi, Parisa Abedi, Roberto Ariel Abeldaño Zuñiga, Olugbenga Olusola Abiodun, Olumide Abiodun, Shady Abohashem, Ulric Sena Abonie, Nagah M. Abourashed, Mohamed Abouzid, Dmitry Abramov, Lucas Guimarães Abreu, Rana Kamal Abu Farha, Fuad Hamdi A. Abuadas, Aminu Kende Abubakar, Bilyaminu Abubakar, Eman Abu-Gharbieh, Sawsan Abuhammad, Ahmad Y Abuhelwa, Hana J Abukhadijah, Salahdein Aburuz, Dina Abushanab, Ahmed Abu-Zaid, Anirudh Balakrishna Acharya, Meshack Achore, Juan Manuel Acuna, Oladimeji Muritala Adebayo, Tajudeen Adesanmi Adebisi, David Adedia, Kamoru Ademola Adedokun, Oluwatobi E Adegbile, Nurudeen A Adegoke, Olumide Thomas Adeleke, Isaac Ayodeji Adesina, Olatunji O Adetokunboh, Mache Tsadik Adhana, Usha Adiga, Tanin Adl Parvar, Mohd Adnan, Qorinah Estiningtyas Sakilah Adnani, David Adzrago, Aanuoluwapo Adeyimika Afolabi, Rotimi Felix Afolabi, Saira Afzal, Gizachew Beykaso Agafari, Navidha Aggarwal, Mahdi Aghaalikhani, Seyed Mohammad Kazem Aghamir, Feleke Doyore Agide, Mary Dada Agoi, César Agostinis Sobrinho, Bright Opoku Ahinkorah, Danish Ahmad, Muayyad M Ahmad, Rabbiya Ahmad, Tauseef Ahmad, Waqas Ahmad, Akeem Olayiwola Ahmed, Ali Ahmed, Anisuddin Ahmed, Aram Mahmood Ahmed, Ayman Ahmed, Gasha Salih Ahmed, Haroon Ahmed, Luai A Ahmed, Mehrunnisha Sharif Ahmed, Meqdad Saleh Ahmed, Muktar Beshir Ahmed, Mushood Ahmed, Naveed Ahmed, Shabbir Ahmed, Syed Anees Ahmed, Gulzhanat Aimagambetova, Janardhana P Aithala, Marjan Ajami, Hossein Akbarialiabad, Saeid Akbarifard, Roland Eghoghosoa Akhigbe, Yagiz Matthew Akiska, Mohammed Ahmed Akkaif, Wole Akosile, Hammad Akram, Ashley E Akrami, Mohammad Khaled Al Nawayseh, Omar Al Omari, Zain Al Ta'ani, Yazan Al Thaher, Omar Ali Mohammed Al Zaabi, Mohammad Ahmmad Mahmoud Al Zoubi, Tariq A Alalwan, Khurshid Alam, Mostafa Alam, Rasmieh Mustafa Al-Amer, Abebaw Alamrew, Amani Alansari, Fahmi Y Al-Ashwal, Mohammed Albashtawy, Khalifah A Aldawsari, Mohammed S Aldossary, Shereen M Aleidi, Tekletsadik Tekleslassie Alemayehu, Fentahun Alemnew, Ayman Al-Eyadhy, Ali M Alfalki, Abdelazeem M Algammal, Fadwa Naji Alhalaiqa, Mohammed Khaled Al-Hanawi, Aminu Alhassan Alhassan Ibrahim, Ashraf Alhumaidi, Fahad A. Alhumaydhi, Amjad Ali, Kamran Ali, Mohammed Usman Ali, Shahid Ali, Syed Shujait Ali, Waad Ali, Akram Al-Ibraheem, Gianfranco Alicandro, Montaha Al-Iede, Hamid Alinejad Rokny, Samah W Al-Jabi, Moath Saleh Aljohani, Ahmad Alkhatib, Mustafa Alkhawam, Atefeh Allahbakhshian, Mohammed Z. Allouh, Wael Almahmeed, Md. Al-Mamun, Hesham M Al-Mekhlafi, Omar Almidani, Amr Almobayed, Khaldoon Aied Alnawafleh, Hasan Yaser Alniss, Mohammad R Alosta, Jaber S Alqahtani, Saleh A Alqahtani, Mohammad R Alqudimat, Ahmad Rajeh Al-Qudimat, Ahmad Alrawashdeh, Rami H Al-Rifai, Intima Alrimawi, Sahel Majed

Alrousan, Najim Z. Alshahrani, Zaid Altaany, Awais Altaf, Alaa B Al-Tammemi, Jaffar A Al-Tawfiq, Malik A Althobiani, Nelson Alvis-Guzman, Mohammad Al-Wardat, Yaser Mohammed Al-Worafi, Hany Aly, Mohammad Sharif Ibrahim Alyahya, Karem H Alzoubi, Faten Amer, Bardia Amidi, Amr Amin, Tarek Tawfik Amin, Alireza Amindarolzarbi, Saeed Amini, Ehsan Amini-Salehi, Nafiu Aminu, Majid Aminzare, Sohrab Amiri, Mohammad Hosein Amirzade-Iranaq, Dickson A Amugsi, Ganiyu Adeniyi Amusa, Filippos Anagnostakis, Roshan A Ananda, Nazanin Anaraki, Robert Ancuceanu, Deanna Anderlini, David B Anderson, Song Peng Ang, Nguyen Hoang Anh, Samuel Egyakwa Ankomah, Amir Anoushiravani, Sumbul Ansari, Umair Ansari, Ernoiz Antriyandarti, Boluwatife Stephen Anuoluwa, Iyadunni Adesola Anuoluwa, Saleha Anwar, Shahnawaz Anwer, Anayochukwu Edward Anyasodor, Geminn Louis Carace Apostol, Juan Pablo Arab, Jalal Arabloo, Abdulfatai Aremu, Jorge Arias de la Torre, Ghazal Arjmand, Benedetta Armocida, Johan Ärnlöv, Jesu Arockiaraj, Mahwish Arooj, Deepavalli Arumuganainar, Mahsa Asadi Anar, Majid Asadi-Samani, Syed Mohammed Basheeruddin Asdaq, Muhammad Abdul Basit Ashraf, Mitra Ashrafi, Bernard Kwadwo Yeboah Asiamah-Asare, Muhammad Shahzad Aslam, Yuni Asri, Dereje Zewdu Assefa, Maha Moh'd Wahbi Atout, Alok Atreya, Marcel Ausloos, Abolfazl Avan, Núbia Carelli Pereira Avelar, Adedapo Wasiu Awotidebe, Beatriz Paulina Ayala Quintanilla, Fekadu Belay Ayalew, Lemessa Assefa A Ayana, Olatunde O Ayinde, Yusuf Oloruntoyin Ayipo, Seyed Mohammad Ayyoubzadeh, Sina Azadnajafabad, Arian Azadnia, James Mba Azam, Alireza Azarboo, Gulrez Shah Azhar, Farya Azimi, Sadat Abdulla Aziz, Ahmed Y Azzam, Domenico Azzolino, Shahram Babadoust, Abraham Samuel Babu, Giridhara Rathnaiah Babu, Ashish D Badiye, Elahe Baghizadeh, Sana Baghizadeh, Khlood K Baghlaf, Ahmed Salem BaHammam, Ruhai Bai, Atif Amin Baig, Maher Balkis, Jose Balmori-de-la-Miyar, Mohammadreza Balooch Hasankhani, Ovidiu Constantin Baltatu, Soham Bandyopadhyay, Palash Chandra Banik, Angelo Barbato, Ryan M Barber, Suzanne Lyn Barker-Collo, Hiba Jawdat Barqawi, Amadou Barrow, Shahid Bashir, Azadeh Bashiri, Guido Basile, Pritish Baskaran, Mohammad-Mahdi Bastan, Abdul-Monim Batiha, Bernhard T Baune, Mahdis Bayat, Mulat Tirfie Bayih, Thomas Beaney, Neeraj Bedi, Jina Behjati, Babak Behnam, Payam Behzadi, Bezawit K Bekele, Melesse Belayneh, Bashir Bello, Muhammad Bashir Bello, Olorunjuwon Omolaja Bello, Umar Muhammad Bello, Luis Belo, Apostolos Beloukas, Samiun Nazrin Bente Kamal Tune, Habib Benzian, Maria Bergami, Ajeet Singh Bhadoria, Akshaya Srikanth Bhagavathula, Jeetendra Bhandari, Ashish Bhargava, Sonu Bhaskar, Arushee Bhatnagar, Priyadarshini Bhattacharjee, Shuvarthi Bhattacharjee, Gurjit Kaur Bhatti, Jasvinder Singh Bhatti, Soumitra S Bhuyan, Raluca Bievel-Radulescu, Naif Kandash Binsaleh, Catherine Bisignano, Bijit Biswas, Mohammad Shahangir Biswas, Molalegne Bitew, Bruno Bizzozero-Peroni, Virginia Bodolica, Mahmut Bodur, Lucimere Bohn, Obasanjo Afolabi Bolarinwa, Paria Bolourinejad, Sri Harsha Boppana, Berrak Bora Basara, Hamed Borhany, Arturo Borzutzky, Alejandro Botero Carvajal, Souad Bouaoud, Soufiane Boufous, Rupert R A Bourne, Christopher Boxe, Nicola Luigi Bragazzi, Dejana Braithwaite, Susanne Breitner, Hermann Brenner, Gabrielle Britton, Julie Brown, Raffaele Bugiardini, Felix Busch, Yasser Bustanji, Lucero Cahuana-Hurtado, Rose Cairns, Daniela Calina, Luciana Aparecida Campos, Ismael Campos-Nonato, Angelo Capodici, Giulia Carreras, Andrea Carugno, Andre F Carvalho, Márcia Carvalho, Ana Paula Carvalho-e-Silva, Joao Mauricio Castaldelli-Maia, Carlos A Castañeda-Orjuela, Giulio Castelpietra, Ferrán Catalá-López, Alberico L Catapano, Maria Sofia Cattaruzza, Luca Cegolon, Francieli Cembranel, Muthia Cenderadewi, Ester Cerin, Pamela Roxana Chacón-Uscamaita, Sandip Chakraborty, Jeffrey Shi Kai Chan, Joht Singh Chandan, Rama Mohan Chandika, Miyuru Chandradasa, Jung-Chen Chang, Vijay Kumar Chattu, Victoria Chatzimavridou-Grigoriadou, Sirshendu Chaudhuri, Akhilanand Chaurasia, An-Tian Chen, Hana Chen, Haowei Chen, Hui Chen, Meng Xuan Chen, Haojin Cheng, Ka Ching Cheung, Nicholas WS Chew, Fatemeh Chichagi, Ju-Huei Chien, Daniel Youngwhan Cho, William C S Cho, Bryan Chong, Hitesh Chopra, Shivani Chopra, Sreshtha Chowdhury, Hongyuan Chu, Stephen Chukwudeh, Eric Chung, Sunghyun Chung, Cain C T Clark, Alyssa

Columbus, Haley Comfort, Joao Conde, Nathalie Conrad, Samuele Cortese, Paolo Angelo Cortesi, Claudia Cosma, Michael H Criqui, Natalia Cruz-Martins, Tukur Dahiru, Zainab Umar Dahiru, Mayank Dalakoti, Koustuv Dalal, Gloria Dalla Costa, Emanuele D'Amico, Samuel E Danso, Samuel Demissie Darcho, Latefa Ali Dardas, Chengetai Dare, Barbara A D'Avanzo, Claudio Alberto Dávila-Cervantes, Dimash Davletov, Alejandro de la Torre-Luque, Edward Christopher Dee, Louisa Degenhardt, Paria Dehesh, Andreas K Demetriades, Edgar Denova-Gutiérrez, Ismail Dergaa, Kebede Deribe, Hunegnaw Almaw Derseh, Emina Dervišević, Hardik Dineshbhai Desai, Abraham Aregay Desta, Pradeep Kumar Devarakonda, Devananda Devegowda, Arkadeep Dhali, Samath Dhamminda Dharmaratne, Meghnath Dhimal, Marcello Di Pumpo, Diana Dias da Silva, Daniel Diaz, Luis Antonio Diaz, Diego Diaz-Milanes, Elangovan Dilipan, Lauren K Dillard, M Ashworth Dirac, Huyen Do, Phidelia Theresa Doegah, Sushil Dohare, Francesco Dondi, Mario D'Oria, Ojas Prakashbhai Doshi, Menayit Tamrat Dresse, Tim Robert Driscoll, Jiang Du, Judy R. Dubno, Emeka W Dumbili, Samuel C Dumith, Bruce B Duncan, Jennifer Dunne, Senbagam Duraisamy, Oyewole Christopher Durojaiye, Abdel Rahman E'mar, Osamudiamen Ebohon, Ejemai Eboreime, Mohammad Hossein Ebrahimi, David Edvardsson, Behrad Eftekhari, Foolad Eghbali, Shayan Eghdami, Ashkan Eighaei Sedeh, Ebrahim Eini, Michael Ekholuenetale, Rabie Adel El Arab, Maysaa El Sayed Zaki, Reza Elahi, Said El-Ashker, Rana Elbeshbeishy, Faris El-Dahiyat, Marwa Eldegwi, Marwan El-Deyarbi, Noha Mousaad Elemam, Ghada Metwally Tawfik ElGohary, Muhammed Elhadi, Mohamed Elhoumed, Omar Abdelsadek Abdou Elmeligy, Mohamed A Elmonem, Adel B Elmoselhi, Mohamed Hassan Elnaem, Mohammed Elshaer, Chadi Eltaha, Abdelgawad Salah Abdelgawad Eltahawy, Syed Emdadul Haque, Theophilus I Emeto, Victor Oghenekparobo Emojevwe, Christopher Imokhuede Esezobor, Sharareh Eskandarieh, Majid Eslami, Rafaela Cavalheiro do Espírito Santo, Elochukwu Ezenwankwo, Natalia Fabin, Heidar Fadavian, Adeniyi Francis Fagbamigbe, Ayesha Fahim, Aliasghar Fakhri-Demeshghieh, Luca Falzone, Seyed Nooreddin Faraji, Ali Faramarzi, Mohammad Fareed, Andre Faro, Fatemeh Farshad, Farima Farsi, Md. Omar Faruk, Folorunso Oludayo Fasina, Modupe Margaret Fasina, Ali Fatehizadeh, Davood Fathi, Zareen Fatima, Valery L Feigin, maryam feili, Talukdar Raian Ferdous, Seyed-Mohammad Fereshtehnejad, Nuno Ferreira, Alexander Finnemore, Claudio Fiorilla, Ida Fitriana, Luisa S Flor, Marco Fonzo, Behzad Foroutan, Daniela Fortuna, Matteo Foschi, Maryam Fotouhi, Alberto Freitas, Takeshi Fukumoto, Ami Fukunaga, Blima Fux, Peter Andras Gaal, Muktar A Gadanya, Dominic Dormenyo Gadeka, Márió Gajdács, Emmanuela Gakidou, Silvano Gallus, Balasankar Ganesan, Shivaprakash Gangachannaiah, Xiang Gao, Bashiru Garba, Miguel Garcia-Argibay, David Garcia-Azorin, Jacopo Garlasco, Prem Gautam, Rupesh K Gautam, Bamba Gaye, Federica Gazzelloni, Miglas Welay Gebregergis, Haftay Gebremedhin Gebreslassie, Miesa Gelchu, Stefano Gelibter, Nsikakabasi Samuel George, Ali Gerami Matin, Lemma Getacher, Kalab Yigermal Gete, Delaram J Ghadimi, Arin Ghamkhar, Moein Ghasemi, Mohammad-Reza Ghasemi, Shakiba Ghasemi Assl, Fariba Ghassemi, Ramy Mohamed Ghazy, Nermin Ghith, Zainab Gholami, Nasim Gholizadeh, Elena Ghotbi, Alessandro Gialluisi, Ruth Margaret Gibson, Syed Abdullah Gilani, Tiffany K Gill, Alem Abera Girmay, Alessandro Girombelli, Laszlo Göbölös, Anil Kumar Goel, Archit Goel, Rajesh Kumar Goel, Ali Golestani, Mohsen Golkar, Nelson G M Gomes, Wenping Gong, Sameer Vali Gopalani, Yitayal Ayalew Goshu, Alessandra C Goulart, Ayman Grada, Michal Grivna, Ashna Grover, Shi-Yang Guan, Giovanni Guarducci, Avirup Guha, Stefano Guicciardi, Cui Guo, Zhaoyu Guo, Zheng Guo, Zhifeng Guo, Himanshu Gupta, Lalit Gupta, Rajat Das Gupta, Rajeev Gupta, Sapna Gupta, Roberth Steven Gutiérrez-Murillo, Awoke Derbie Derbie Habteyohannes, Tesfahun Simon Hadaro, Zahra Hadian, Faraidoon Haghdoost, Arian Haghtalab, Nguyen Hai Nam, Pritam Halder, Rabih Halwani, Islam M Hamad, Randah R Hamadeh, Nadia M Hamdy, Ahmad Hammoud, Mohammad Hamza, Didem Han Yekdeş, Graeme J Hankey, Ashanul Haque, Md Nuruzzaman Haque, Obaid I Haque, Harapan Harapan, Josep Maria Haro, Eka Mishbahatul Marah Has, Ahmed I Hasaballah, Faizul Hasan, Md Kamrul

Hasan, Towhid Hasan, Ali Hasanpour- Dehkordi, Arezou Hashem Zadeh, Mohammad Hashem Hashempur, Nada Tawfig Hashim, Ammarah Hasnain, Ibrahim Nagmeldin Hassan, Nageeb Hassan, Yusuf Wada Hassan Wada, Mahgol Sadat Hassan Zadeh Tabatabaei, Rasmus J Havmoeller, Simon I Hay, Jeffrey J Hebert, Golnaz Heidari, Mehdi Hemmati, Claudiu Herteliu, Hamed Hesami, Sumudu Avanthi Hewage, Yuta Hiraike, Ramesh Holla, Alamgir Hossain, Lubna Hossain, Md Mahbub Hossain, Md Sabbir Hossain, Mohammad Bellal Hossain, Sorin Hostiuc, Amir Human Hoveidaei, Hanno Hoven, Chengxi Hu, Weijun Huang, Nawfal R Hussein, Mohamed Ibrahim Husseiny, Hong-Han Huynh, Luigi Francesco Iannone, Segun Emmanuel Ibitoye, Ismail A. Atef Ismail Ahmed Ibrahim, Ramzi Ibrahim, Umar Idris Ibrahim, Anel Ibrayeva, Fidelia Ida, Pulwasha Maria Iftikhar, Adalia Ikiroma, Olayinka Stephen Ilesanmi, Irena M Ilic, Milena D Ilic, Masoud Imani, Mustapha Immurana, Lucius Chidiebere Imoh, Leeberk Raja Inbaraj, Arit Inok, Mujahid Iqbal, Muhammad Iqhrammullah, Mustafa Alhaji Isa, Benni Iskandar, Teresa R Iskander, Dr. Md. Shahinul Islam, Md Rabiul Islam, Farhad Islami, Faisal Ismail, Nahlah Elkudssiah Ismail, Yerlan Ismoldayev, Gaetano Isola, Ihoghosa Osamuyi Iyamu, Mahalaxmi Iyer, Udeme Samuel Jacob, Kathryn H. Jacobsen, Ali Jadidi, Mohammadsadegh Jafari, Morteza Jafarinia, Haitham Jahrami, Mihajlo Jakovljevic, Ali Jaliliyan, Mohamed Jalloh, Armaan Jamal, Qazi Mohammad Sajid Jamal, Jazlan Jamaluddin, Melika Jameie, Safayet Jamil, Masoud Jamshidi, Shaghayegh JamshidiRastabi, Esmaeil Jarrahi, Tahereh Javaheri, Syed Sarmad Javaid, Anita Javanmardi, Shubha Jayaram, Ruwan Duminda Jayasinghe, Yovanthi Anurangi Jayasinghe, Achala Upendra Jayatilleke, Felix K Jebasingh, Seogsong Jeong, Bijay Mukesh Jeswani, Shuai Jin, Wenyi Jin, Jost B Jonas, Tamas Joo, Abel Joseph, Nitin Joseph, Charity Ehimwenma Joshua, George Joy, Jacek Jerzy Jozwiak, Malik E Juweid, Vaishali K, Ashish Kumar Kakkar, Pradnya Vishal Kakodkar, Khalil Kalavani, Md Moustafa Kamal, Sivesh Kathir Kamarajah, Rajesh Kamath, Saltanat Kamenova, Arun Kamireddy, Ramat T. Kamorudeen, Devanish Narasimhasanth Kamtam, Oleksandr Kamyshnyi, Mona Kanaan, Saddam Fuad Kanaan, Jiseung Kang, Samuel Berchi Kankam, Kehinde Kazeem Kanmodi, Rami S Kantar, Neeti Kapoor, Paschalis Karakasis, Reema A Karasneh, Mohammad Amin Karimi, Mohmed Isaqali Karobari, Tomasz M Karpiński, Sadanand Karun, Manoj Kumar Kashyap, Adarsh Katamreddy, Kanica Kaushal, Foad Kazemi, Sina Kazemian, Hafte Kahsay Kebede, John H Kempen, Jessica A Kerr, Emmanuelle Kesse-Guyot, Inn Kynn Khaing, Himanshu Khajuria, Sidra Khalid, Hazim O. Khalifa, Anas Husam Khalifeh, Anees Ahmed Khalil, Anita Khalili, Pantea Khalili, Ghazaleh Khalili-Tanha, Ajmal Khan, Fayaz Khan, Iman Waheed Khan, Maseer Khan, Md Abdullah Saeed Khan, Muhammad Hamza Khan, Muhammad Mueed Khan, Muhammad Umer Khan, Ramsha Mushtaq Khan, Sumaiya Khan, Yusuf Saleem Khan, Zahid Khan, Zahid Khan, Srijana Khanal, Shaghayegh Khanmohammadi, Zenith Khashim, Haitham Khatatbeh, Moawiah Mohammad Khatatbeh, Hamid Reza Khayat Kashani, Sunil Kumar Khokhar, Atulya Aman Khosla, Majid Khosravi, Sepehr Khosravi, Mahmood Khosrowjerdi, Jagdish Khubchandani, Zemene Demelash Kifle, Hye Jun Kim, Jinho Kim, Min Seo Kim, Yun Jin Kim, Sanjay Kini B, Adnan Kisa, Sezer Kisa, Ladli Kishore, Mika Kivimäki, Shivakumar KM, Ann Kristin Skrindo Knudsen, Nazarii Kobyliak, Sonali Kochhar, Michail Kokkorakis, Diana Gladys Kolieghu Tcheumeni, Aida Kondybayeva, Gerbrand Koren, Tapos Kormoker, Oleksii Korzh, Karel Kostev, Sindhura Lakshmi Koulmane Laxminarayana, Irene Akwo Kretchy, James-Paul Kretchy, Kewal Krishan, Chong-Han Kua, Ananya Kuanar, Barthelemy Kuate Defo, Mohammed Kuddus, Ilari Kuitunen, Shikha Kukreti, Mukhtar Kulimbet, Vishnutheertha Kulkarni, Shweta Kulshreshtha, Avinash Kumar, Dewesh Kumar, Jogender Kumar, Tushar Kumar, Setor K Kunutsor, Almagul Kurmanova, Maria Dyah Kurniasari, Asep Kusnali, Christina Yeni Yeni Kustanti, Dian Kusuma, Tezer Kutluk, Wai Hang Patrick Kwong, Frank Kyei-Arthur, Ville Kytö, Pallavi L C, Adriano La Vecchia, Carlo La Vecchia, Muhammad Awwal Ladan, Lucie Laflamme, Chandrakant Lahariya, Daphne Teck Ching Lai, Balzhan Lakanova, Anita Lakhani, Tea Lallukka, Iván Landires, Berthold Langguth, Ariane Laplante-Lévesque, Kamaluddin Latief, Saheed Akinmayowa Lawal, Aliyu Lawan, Huu-Hoai Le, Minh

Huu Nhat Le, Nhi Huu Hanh Le, Thao Thi Thu Le, Caterina Ledda, Vasileios Leivaditis, Matilde Leonardi, Elvynna Leong, An Li, Hui Li, Jiaying Li, Wei Li, Wei Li, Zhaolong Adrian Li, Zhengrui Li, Yanxue Lian, Xue-Zhen Liang, Stephen S Lim, Queran Lin, Daniel Lindholm, Yuewei Ling, Jue Liu, Xianliang Liu, Yubo Liu, Zhe Liu, Erand Llanaj, Michael J Loftus, Valerie Lohner, José Francisco López-Gil, Surbala Devi Lourembam, Giancarlo Lucchetti, Susu Luo, Jay B Lusk, Angelina M Lutambi, Miltiadis D Lytras, Ellina Lytvyak, Kevin Sheng-Kai Ma, Zheng Feei Ma, Mahmoud Mabrok, Nikolaos Machairas, Monika Machoy, Seyed Ataollah Madinezad, Christian Madsen, Aurea Marilia Madureira-Carvalho, D. R. Mahadeshwara Prasad, Sasikumar Mahalingam, Preeti Maharjan, Farhad Mahmoudi, Rituparna Maiti, Marek Majdan, Abdelrahman M Makram, Reza Malekzadeh, Hardeep Singh Malhotra, Ahmad Azam Malik, Farihah Malik, Deborah Carvalho Malta, Mustapha Mangdow, Emery Manirambona, Lokesh Manjani, Kamaruddeen Mannethodi, Marjan Mansourian, Lorenzo Giovanni Mantovani, Changkun Mao, Tahir Maqbool, Hamid Reza Marateb, Mirko Marino, Adilson Marques, Gabriel Martinez, Bernardo Alfonso Martinez-Guerra, Ramon Martinez-Piedra, Daniela Martini, Francisco Rogerlândio Martins-Melo, Miquel Martorell, Roy Rillera Marzo, Sammer Marzouk, Sugeng Mashudi, Clara N Matei, Yasith Mathangasinghe, Stephanie Mathieson, Alexander G Mathioudakis, Medha Mathur, Fernanda Penido Matozinhos, Rita Mattiello, Khurshid A Mattoo, Pallab K Maulik, Mahsa Mayeli, Mohsen Mazidi, John J McGrath, Steven M McPhail, Enkeleint A Mechili, Rishi P Mediratta, Riffat Mehboob, Berhanu Abebaw Mekonnen, Hadush Negash Meles, Satish Melwani, Walter Mendoza, Godfred Antony Menezes, George A Mensah, Sultan Ayoub Ayoub Meo, Michelangelo Mercogliano, Atte Meretoja, Tuomo J Meretoja, Tomislav Mestrovic, Chamila Dinushi Kukulege Mettananda, Sachith Mettananda, Mohamed M. M. Metwally, Tomasz Miazgowski, Irmina Maria Michalek, Andrea Michelerio, Ted R Miller, Giuseppe Minervini, Mojgan Mirghafourvand, Seyed Ali Mirshahvalad, Awoke Misganaw, Archana Mishra, Philip B Mitchell, Chaitanya Mittal, Malihe Moazeni, Shivani Modi, Mona Gamal Mohamed, Nouh Saad Mohamed, Khabab Abbasher Hussien Mohamed Ahmed, Taj Mohammad, Sakineh Mohammad-Alizadeh-Charandabi, Abdollah Mohammadian-Hafshejani, Saeed Mohammadpour, Abdulwase Mohammed, Ammas Siraj Mohammed, Hussen Mohammed, Mustapha Mohammed, Shafiu Mohammed, Suleiman Mohammed, Yahaya Mohammed, Mohammad Mohseni, Ali H Mokdad, Sabrina Molinaro, Amirabbas Mollaei, Lorenzo Monasta, Himel Mondal, Stefania Mondello, Mohammad Ali Moni, Marco Montalti, Maziar Moradi-Lakeh, Paula Moraga, Rafael Silveira Moreira, Shane Douglas Morrison, Mahmoud M Morsy, Jonathan F Mosser, Simin Mouodi, Seyede Zohre Mousavi, Amin Mousavi Khaneghah, Seyed Mohamad Sadegh Mousavi Kiasary, Hagar Lotfy Mowafy, Matías Mrejen, Faraz Mughal, Syed Aun Muhammad, Oscar J Mujica, Sukhes Mukherjee, Amartya Mukhopadhyay, George Duke Mukoro, Francesk Mulita, Chalie Mulugeta, Malaisamy Muniyandi, Yanjinlkham Munkhsaikhan, Javier Muñoz Laguna, Michio Murakami, Efren Murillo-Zamora, B.V. Murlimanju, Christopher J L Murray, Sani Musa, Ali Mushtaq, Sathish Muthu, Saravanan Muthupandian, Claude Mambo Muvunyi, Muhammad Muzaffar, Woojae Myung, Amin Nabavi, Ahamarshan Jayaraman Nagarajan, Mohsen Naghavi, Noureddin Nakhostin Ansari, Gopal Nambi, Jobert Richie Nansseu, Bruno Ramos Nascimento, Gustavo G Nascimento, Abdallah Y Naser, Abdulqadir J Nashwan, Hamide Nasiri, Mahmoud Nassar, Zuhair S Natto, Samidi Nirasha Kumari Navaratna, Biswa Prakash Nayak, Shalini Ganesh Nayak, Smitha Nayak, Shumaila Naz, G. Takop Nchanji, Wubshet D Negash, Ionut Negoi, Ruxandra Irina Negoi, Jalil Nejati, Nikita A Nekliudov, Samata Nepal, Charles Richard James Newton, Georges Nguefack-Tsague, Josephine W Ngunjiri, Cuong Tat Nguyen, Dang Nguyen, Long Nguyen, Nghia Phu Nguyen, Tu Anh Nguyen, Van Thanh Nguyen, Ambe Marius Ngwa, Robina Khan Niazi, Luciano Nieddu, Vikram Niranjan, Abebe Melis Nisro, Jan Rene Nkeck, Masoud Noroozi, Jean Jacques Noubiap, Valentine C Nriagu, Chisom Adaobi Nri-Ezedi, Jean Claude Nshimiyimana, Mpiko Ntsekhe, Fred Nugen, Nurfatimah Nurfatimah, Dieta Nurrika, Sylvester Dodzi Dodzi Nyadanu, Ogochukwu Janet Nzoputam,

Bogdan Oancea, Fabio Massimo Oddi, Ismail A Odetokun, Oluwakemi Ololade Odukoya, Akinyemi O D Ofakunrin, Onome Bright Oghenetega, In-Hwan Oh, Sarah Oh, Edel T O'Hagan, Sylvester Reuben Okeke, Deborah Oluwatosin Okeke-Obayemi, Olalekan John Okesanya, Osaretin Christabel Okonji, Andrew T Olagunju, Oladotun Victor Olalusi, Matthew Idowu Olatubi, Arão Belitardo Oliveira, Abdulhakeem Abayomi Olorukooba, Oluseye Olalekan Oludoye, Bolajoko Olubukunola Olusanya, Jacob Olusegun Olusanya, Obinna E Onwujekwe, Marcel Opitz, Aksoltan Shyhdurdyevna Oradova, Michal Ordak, Verner N Orish, Raffaele Ornello, Atakan Orscelik, Alberto Ortiz, Esteban Ortiz-Prado, Augustus Osborne, John W Ostrominski, Uchechukwu Levi Osuagwu, Godfred Otchere, Elham H Othman, Adrian Otoiu, Oche Joseph Otorkpa, Jerry John Ouner, Amel Ouyahia, Mayowa O Owolabi, Irene Amoakoh Owusu, Kolapo Oyebola, Tope Oyelade, Ilker Ozsahin, Mahesh P A, Alicia Padron-Monedero, Jagadish Rao Padubidri, Tamás Palicz, Raffaele Palladino, Raul Felipe Palma-Alvarez, Tejasri Paluvai, Feng Pan, Sujogya Kumar Panda, Songhomitra Panda-Jonas, Seithikurippu R Pandi-Perumal, Helena Ullyartha Pangaribuan, Leonidas D Panos, Ioannis Pantazopoulos, Anca Pantea Stoian, Giovanni Paolino, Ilias Papadimopoulos, Paraskevi Papadopoulou, Parinaz Paranjkhoo, Shahina Pardhan, Romil R Parikh, Arpit Parmar, Swapnil Parve, Roberto Passera, Hemal M Patel, Heta Pavan Patel, Jay Patel, Mitesh Patel, Neel Navinkumar Patel, Riya Jayesh Patel, Satyananda Patel, Angel J Paternina-Caicedo, Bharat Smita Umakant Patil, Shankargouda Patil, Apurba Patra, Venkata Suresh Patthipati, Shrikant Pawar, Shubhadarshini Pawar, Hamidreza Pazoki Toroudi, Spencer A Pease, Amy E Peden, Paolo Pedersini, Veincent Christian Filipino Pepito, João Perdigão, Gavin Pereira, Arokiasamy Perianayagam, Norberto Perico, Simone Perna, Fanny Emily Petermann-Rocha, Hoang Nhat Pham, Hoang Tran Pham, Nhat Truong Pham, Michael R Phillips, Zahra Zahid Piracha, Edoardo Pirera, Moein Piroozkhkah, Florian Ploeckl, Dimitri Poddighe, Ville T Ponkilainen, Ion Popa, Djordje S Popovic, Sajjad Pourasghary, Sergio I Prada, Jalandhar Pradhan, Pranil Man Singh Pradhan, Akila Prashant, Elton Junio Sady Prates, Harsh Priya, Nicola Riccardo Pugliese, Shuby Puthussery, Jagadeesh Puvvula, Xiang Qi, Jia-Yong Qiu, Zahiruddin Syed Quazi, Raghu Anekal Radhakrishnan, Hadi Raeisi Shahraki, Alberto Raggi, Pracheth Raghuveer, Hawbash Mohammed-Amin Rahim, Sajjad Rahimi, Vafa Rahimi-Movaghar, Mahbubur Rahman, Md Mijanur Rahman, Mohammad Hifz Ur Rahman, Amir Masoud Rahmani, Masoud Rahmati, Ghasem Rahmatpour Rokni, Hakim Rahmoune, Diego Raimondo, Ivano Raimondo, Sunil Kumar Raina, Jeffrey Pradeep Raj, Sandesh Raja, Erta Rajabi, Gunaseelan Rajendran, Judah Rajendran, Mahmoud Mohammed Ramadan, Kadar Ramadhan, Chitra Ramasamy, Shakthi Kumaran Ramasamy, Zahra Ramezani, Chhabi Lal Ranabhat, Nemanja Rancic, Smitha Rani, Chythra R Rao, Kumuda Rao, Mithun Rao, Davide Rasella, Vahid Rashedi, Mohammad-Mahdi Rashidi, Ashkan Rasouli-Saravani, Prateek Rastogi, Devarajan Rathish, Ilari Rautalin, Ramin Ravangard, Dhwani Ravi, David Laith Rawaf, Reza Rawassizadeh, Ramu Rawat, Bahman Razi, Elrashdy Redwan, Sanika Rege, Wajiha Rehman, Rainer Reile, Giuseppe Remuzzi, Bhageerathy Reshmi, Stefano Restaino, Marzieh Rezaei, Mina Rezaei, Antonio Luiz P Ribeiro, Tércia Moreira Ribeiro da Silva, Jennifer Rickard, Hermano Alexandre Lima Rocha, João Rocha Rocha-Gomes, Alfonso J. Rodriguez-Morales, Leonardo Roever, Ravi Rohilla, Susanne Röhr, David Rojas-Rueda, Megan L Rolfzen, Debby Syahru Romadlon, Michele Romoli, Luca Ronfani, Moustaq Karim Khan Rony, Amirhossein Roshanshad, Morteza Rostamian, Kunle Rotimi, Hanieh Rouzbahani, Reza Rouzbahani, Shiva Rouzbahani, Bedanta Roy, Nitai Roy, Priyanka Roy, Sharmistha Roy, Shubhanjali Roy, Simanta Roy, Simanta Roy, Enrico Rubagotti, Susan Fred Rumisha, Michele Russo, Godfrey M Rwegerera, Chandan S N, Aly M A Saad, Michela Sabbatucci, Korosh Saber, Maha Mohamed Saber-Ayad, Cameron John Sabet, Perminder S Sachdev, Kabir P Sadarangani, Seyed Kiarash Sadat Rafiei, Basema Ahmad Saddik, Bashdar Abuzed Sadee, Tarannom Sadegh, Ehsan Sadeghi, Mohd Saeed, Umar Saeed, Maryam Saeedi, Mehdi Safari, Mahdi Safdarian, Rajesh Sagar, Mastooreh Sagharichi, Amene Saghazadeh, Dominic Sagoe, Indranil Saha,

Fatemeh Saheb Sharif-Askari, Amirhossein Sahebkar, Biniyam Sahiledengle, Gülsüm Şahin Bodur, Pragyan Monalisa Sahoo, Zahra Saif, Md Refat Uz Zaman Sajib, Mirza Rizwan Sajid, Luciane B Salaroli, Mahdi Salehi, Marwa Rashad Salem, Mohammed Z Y Salem, Dauda Salihu, Sohrab Salimi, Malik Sallam, Saad Samargandy, Abdallah M Samy, Sandeep G Sangle, Elaheh Sanjari, Sathish Sankar, Lucas H C C Santos, Milena M Santric-Milicevic, Jacob Owusu Owusu Sarfo, Tanmay Sarkar, Mohammad Sarmadi, Gargi Sachin Sarode, Sachin C Sarode, Benn Sartorius, Arash Sarveazad, Michele Sassano, Maheswar Satpathy, Ganesh Kumar Saya, Abu Sayeed, Maria Inês Schmidt, Austin E Schumacher, Aletta Elisabeth Schutte, Ghil Schwarz, David C Schwebel, Falk Schwendicke, Catherine Schwinger, Mario Šekerija, Siddharthan Selvaraj, Yuliya Semenova, Mohammad H Semreen, Ashenafi Kibret Sendekie, Yigit Can Senol, Sadaf G Sepanlou, Edson Serván-Mori, Yashendra Sethi, Seyed Mohammad Seyed Alshohadaei, Allen Seylani, Abubakar Sha'aban, Mahan Shafie, Muhammad Shahab, Shazlin Shaharudin, Samiah Shahid, Endrit Shahini, Farshad Shahkarami, Moyad Jamal Shahwan, Alireza Shakeri, Ali Shakerimoghaddam, Muhammad Aaqib Shamim, Mehran Shams-Beyranvand, Anas Shamsi, Alfiya Shamsutdinova, Dan Shan, Mohd Shanawaz, Abhishek Shankar, Amin Sharifan, Javad Sharifi Rad, Avimanu Sharma, Bunty Sharma, Manoj Sharma, Ravi Kumar Sharma, Ujjawal Sharma, Vishal Sharma, Ramzi Shawahna, Mahabalesh Shetty, Suraj S Shetty, Fang Shi, Lin-Hong Shi, Md Monir Hossain Shimul, Aminu Shittu, Abdul-karim Olayinka Shitu, Velizar Shivarov, Ambreen Shoaib, Sina Shool, Seyed Afshin Shorofi, Sunil Shrestha, Suleiman Adeiza Adeiza Shuaibu, Kerem Shuval, Emmanuel Edwar Siddig, Mohammad Sidiq, Diego Augusto Santos Silva, Luís Manuel Lopes Rodrigues Silva, Biagio Simonetti, Akanksha Singh, Balbir Bagicha Singh, Bhim Pratap Singh, Harmanjit Singh, Harpreet Singh, Jasvinder A Singh, Kalpana Singh, Narinder Pal Singh, Poornima Suryanath Singh, Puneetpal Singh, Samer Singh, Satwinder Singh, Surendra Singh, Robert Sinto, Natia Skhvitaridze, Valentin Yurievich Skryabin, Anna Aleksandrovna Skryabina, Aalam Sohal, Md.Salman Sohel, Somaye Sohrabi, Anton Sokhan, Shipra Solanki, Solikhah Solikhah, Sameh S M Soliman, Aayushi Sood, Prashant Sood, Joan B Soriano, Michele Sorrentino, Fernando Sousa, Ireneous N Soyiri, Michael Spartalis, Chandrashekhar T Sreeramareddy, Bahadar S Srichawla, Nicholas Steel, Sebastian Straube, Omer Subasi, Narayan Subedi, Vetriselvan Subramaniyan, Hasnat Sujon, Thitiporn Sukaew, Surajo Kamilu Sulaiman, Auwal Garba Suleiman, Muhammad Suleman, Desy Sulistiyorini, Mark J M Sullman, Haitong Zhe Sun, Mao-ling Sun, Xiaohui Sun, Zhuanlan Sun, Suraj Sundaragiri, David Sunkersing, Lukasz Szarpak, Sree Sudha T Y, Payam Tabaee Damavandi, Rafael Tabarés-Seisdedos, Seyed Shahaboddin Tabatabaei, Shima Tabatabai, Celine Tabche, Ramin Tabibi, Takahiro Tabuchi, Santosh Kumar Tadakamadla, Buhari Abdullahi Tafida, Farzad Taghizadeh-Hesary, Zanan Mohammed-Ameen Taha, Yasaman Taheri Abkenar, Iman M Talaat, Mircea Tampa, Jacques Lukenze Tamuzi, Ker-Kan Tan, Guodong Tang, Sarvenaz Taridashti, Saba Tariq, Anika Tasnim, Seyed Mohammad Tavangar, Mohamad-Hani Temsah, Reem Temsah, Azimeraw Arega Tesfu, Jay Tewari, Alireza Teymouri, Chandan Kumar Thakur, Ismaeel Tharwat, Samar Tharwat, Arun James Thirunavukarasu, Muthu Thiruvengadam, Manuel Sebastian Thomas, Yves Joel Tochie Noutakdie, Sojit Tomo, Marcello Tonelli, Roman Topor-Madry, Ali Torkashvand, Mathilde Touvier, Marcos Roberto Tovani-Palone, Khaled Trabelsi, Mai Thi Ngoc Tran, Tam Quoc Minh Tran, Thang Huu Tran, Nguyen Tran Minh Duc, Domenico Trico, Indang Trihandini, Samuel Joseph Tromans, Claudia Truppa, Gary Tse, Evangelia Eirini Tsermpini, Sok Cin Tye, Stefanos Tyrovolas, Aniefiok John Udoakang, Himayat Ullah, Muhammad Umair, Lawan Umar, Muhammad Umar, Bhaskaran Unnikrishnan, Dinesh Upadhya, Era Upadhyay, Dipan Uppal, Jibrin Sammani Usman, Hande Uzunçıbuk, Pratyusha Vadagam, Asokan Govindaraj Vaithinathan, Pascual R Valdez, Mario Valenti, Zahir Vally, Jef Van den Eynde, Javad Varasteh, Joe Varghese, Priya Vart, Tommi Juhani Vasankari, Sampara Vasishta, Srivatsa Surya Vasudevan, Alireza Vaysi, Ashleigh S Vella, Balachandar Vellingiri, Narayanaswamy Venketasubramanian, Nicholas Alexander Verghese, Madhur

Verma, Poonam Verma, Massimiliano Veroux, Georgios-Ioannis Verras, Dominique Vervoort, Simone Villa, Jorge Hugo Villafañe, David Villarreal-Zegarra, Sharath Chaitanya Vipparthy, Luciano Magalhães Vitorino, Stein Emil Vollset, Linh Vu, Henok Toga Wada, Yasir Waheed, Agnes Wamuyu Wamai, Jin-Yi Wan, Fang Wang, Jinyu Wang, Qingzhi Wang, Shaopan Wang, Shu Wang, Wanzhou Wang, Wei Wang, Xing Wang, Yanzhong Wang, Youxin Wang, Yuan-Pang Wang, Mary Njeri Wanjau, Ahmed Bilal Waqar, Paul Ward, Kosala Gayan Weerakoon, Fei-Long Wei, Robert G Weintraub, Ronny Westerman, Taweewat Wiangkham, Anggi Lukman Wicaksana, Dakshitha Praneeth Wickramasinghe, Nuwan Darshana Darshana Wickramasinghe, Angga Wilandika, Peter Willeit, Andrew Awuah Wireko, Gemechu Kumera Wirtu, Charles Shey Wiysonge, Abay Tadesse Woday, Marcin W Wojewodzic, Axel Walter Wolf, Tewodros Eshete Wonde, Yen Jun Wong, Daniel Tarekegn Worede, Minichil Chanie Chanie Worku, James Fan Wu, Jinyi Wu, Peng Wu, Yihun Miskir Wubie, Qing Xia, Na Xiao, Site Xu, Suowen Xu, Wang-Dong Xu, Wanqing Xu, Mingyang Xue, Mukesh Kumar Yadav, Vikas Yadav, Saba Yahoo (Syed), Kazumasa Yamagishi, Haibo Yang, Xinxin Yang, Yuichiro Yano, Haiqiang Yao, Laiang Yao, Amir Yarahmadi, Sanni Yaya, Meghdad Yeganeh, Ali Cem Yekdeş, Mohammad Hossein YektaKooshali, Saber Yezli, Muluken Yigezu, Malede Berihun Yismaw, Dong Keon Yon, Naohiro Yonemoto, Jian Yu, Yong Yu, Ghazala Yunus, Umar Yunusa, Siddhesh Zadey, Vesna Zadnik, Mubashir Zafar, Mondal Hasan Zahid, Emilia Zainal Abidin, Fathiah Zakham, Sojib Bin Zaman, Alireza Zangeneh, Aurora Zanghì, Iman Zare, Kourosh Zarea, Shirin Zaresharifi, Michael Zastrozhin, Mohammed Zawiah, Mohammed G M Zeariya, Dawit Zemedikun, Abay Mulu Zenebe, Sebastian Zensen, Beijian Zhang, Casper J P Zhang, Haijun Zhang, Ning Zhang, Xiaoyi Zhang, Xiu-Hang Zhang, Zhiqiang Zhang, Sheng Zhao, Shenglin Zhao, Zhongyi Zhao, Anthony Zhong, Claire Chenwen Zhong, Bin Zhu, Hafsa Zia, Ghazal Zoghi, Mohamed Ali Zoromba, Rafat Mohammad Zrieq, Liesl J Zuhlke, Alimuddin Zumla, Ahed H Zyoud, Sa'ed H Zyoud, Shaher H Zyoud, Nawsherwan

Managing the estimation or publications process

Joanne O Amlag, Catherine M Antony, Catherine Bisignano, Haley Comfort, Kara Estep, Erin B Hamilton, Simon I Hay, Katie Joskowitz, Nicholas J Kassebaum, Molly B Kassel, Stephen S Lim, Ali H Mokdad, Christopher J L Murray, Mohsen Naghavi, Amanda Novotney, Emily Rosenblad, Austin E Schumacher
