# Supplementary material for: Global age-sex-specific all-cause mortality and life expectancy estimates for 204 countries and territories and 660 subnational locations, 1950–2023: a demographic analysis for the Global Burden of Disease Study 2023
Source: Lancet. 2025 Oct 18;406(10513):1731–810. doi: 10.1016/S0140-6736(25)01330-3 (PMC12535839; doi:10.1016/S0140-6736(25)01330-3)

THE LANCET

Supplementary appendix 2

This appendix formed part of the original submission and has been peer reviewed. We post it as supplied by the authors.

Supplement to: GBD 2023 Demographics Collaborators. Global age-sex-specific all-cause mortality and life expectancy estimates for 204 countries and territories and 660 subnational locations, 1950–2023: a demographic analysis for the Global Burden of Disease Study 2023. *Lancet* 2025; published online Oct 12. https://doi.org/10.1016/S0140-6736(25)01330-3.

Appendix 2: supplementary results appendix to "Global age-sex-specific all-cause mortality and life expectancy estimates for 204 countries and territories and 660 subnational locations, 1950–2023: a demographic analysis for the Global Burden of Disease Study 2023"

This supplement provides additional figures containing more detailed results for "Global age-sex-specific all-cause mortality and life expectancy estimates for 204 countries and territories and 660 subnational locations, 1950–2023: a demographic analysis for the Global Burden of Disease Study 2023."

All supplementary tables can be accessed here https://ghdx.healthdata.org/record/ihme-data/gbd-2023-demographics-1950-2023

Figures

Appendix 2 Figure S1a: Most recent year of available vital registration, by country 2

Appendix 2 Figure S1b: Completeness of vital registration systems for the latest year of reporting, by country 3

Appendix 2 Figure S2a: Number of global deaths over time by age group, all sexes combined, 1950-2023 4

Appendix 2 Figure S2b: Number of global deaths over time by age group, females, 1950-2023 5

Appendix 2 Figure S2c: Number of global deaths over time by age group, males, 1950-2023 5

Appendix 2 Figure S3: Death structure by SDI in 2023 6

Appendix 2 Figure S4: National mortality rates across the lifespan in females and males, 1950-2023 7

Appendix 2 Figure S5: National estimated - expected life expectancy based on SDI, by sex, 1950-2023 211

Appendix 2 Figure S6: Comparison of under 5 mortality estimates for 2023 between GBD 2023 and UNICEF 2025, by location 212

Appendix 2 Figure S7: Comparison of life expectancy at birth estimates for 2023 between GBD 2023 and WPP 2024, by sex and location 213

Appendix 2 Figure S8: Comparison of life expectancy at birth estimates for countries in GBD 2023 and WPP 2024, all sexes combined, for every five years from 1950 to 2020 and 2023 214

Appendix 2 Figure S1a: Most recent year of available vital registration, by country

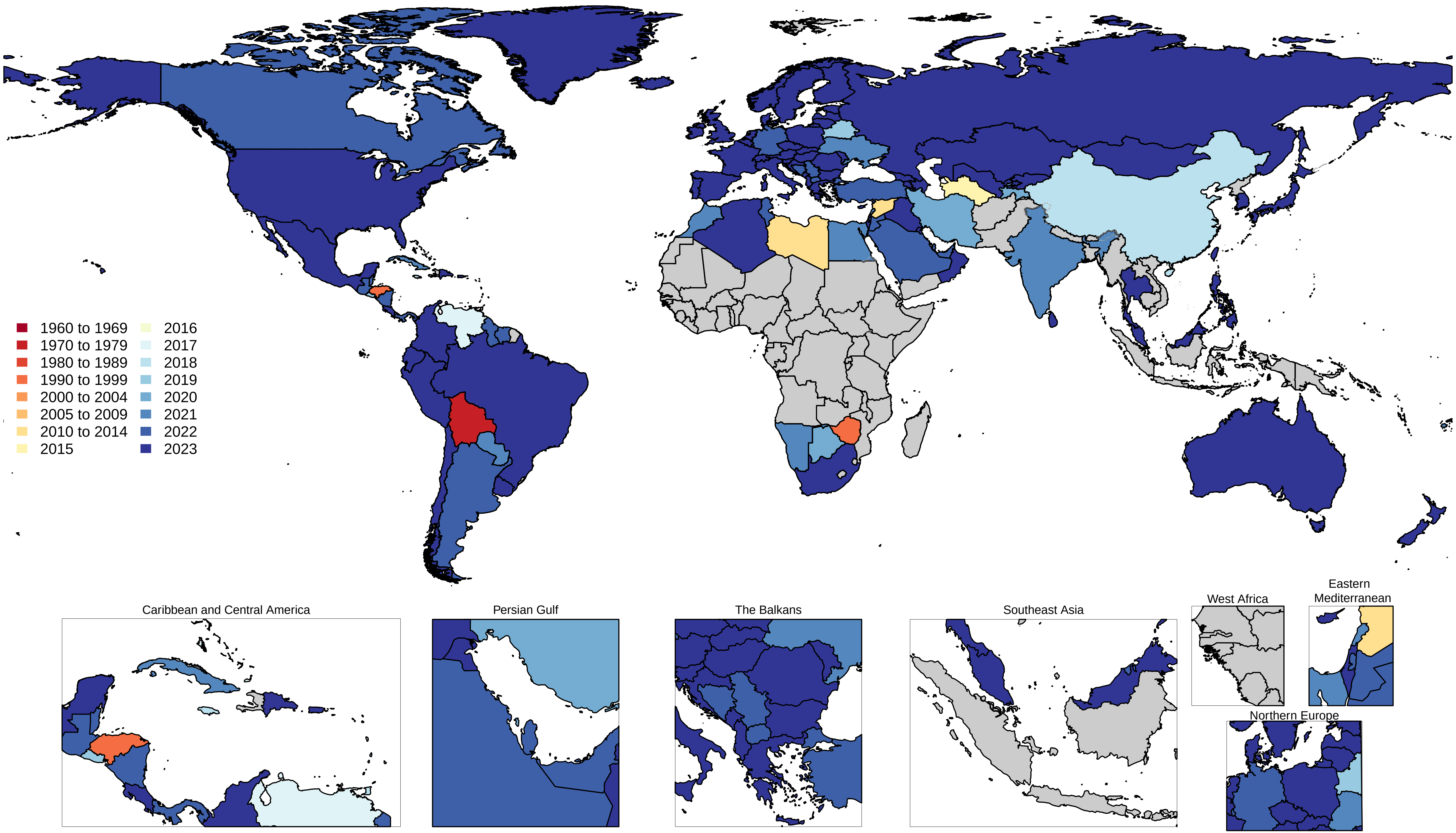

Appendix 2 Figure S1b: Completeness of vital registration systems for the latest year of reporting, by country

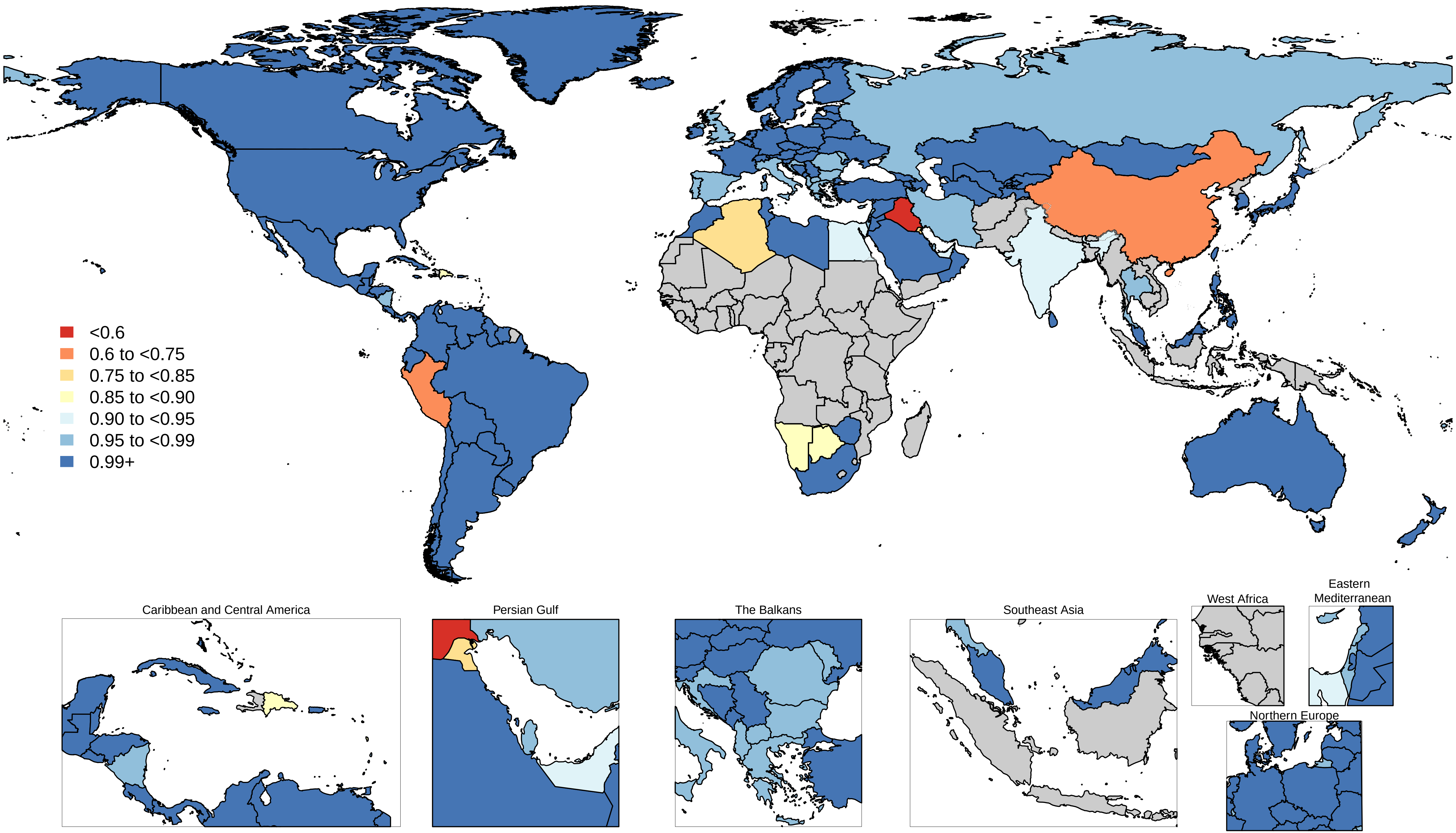

Appendix 2 Figure S2a: Number of global deaths over time by age group, all sexes combined, 1950−2023

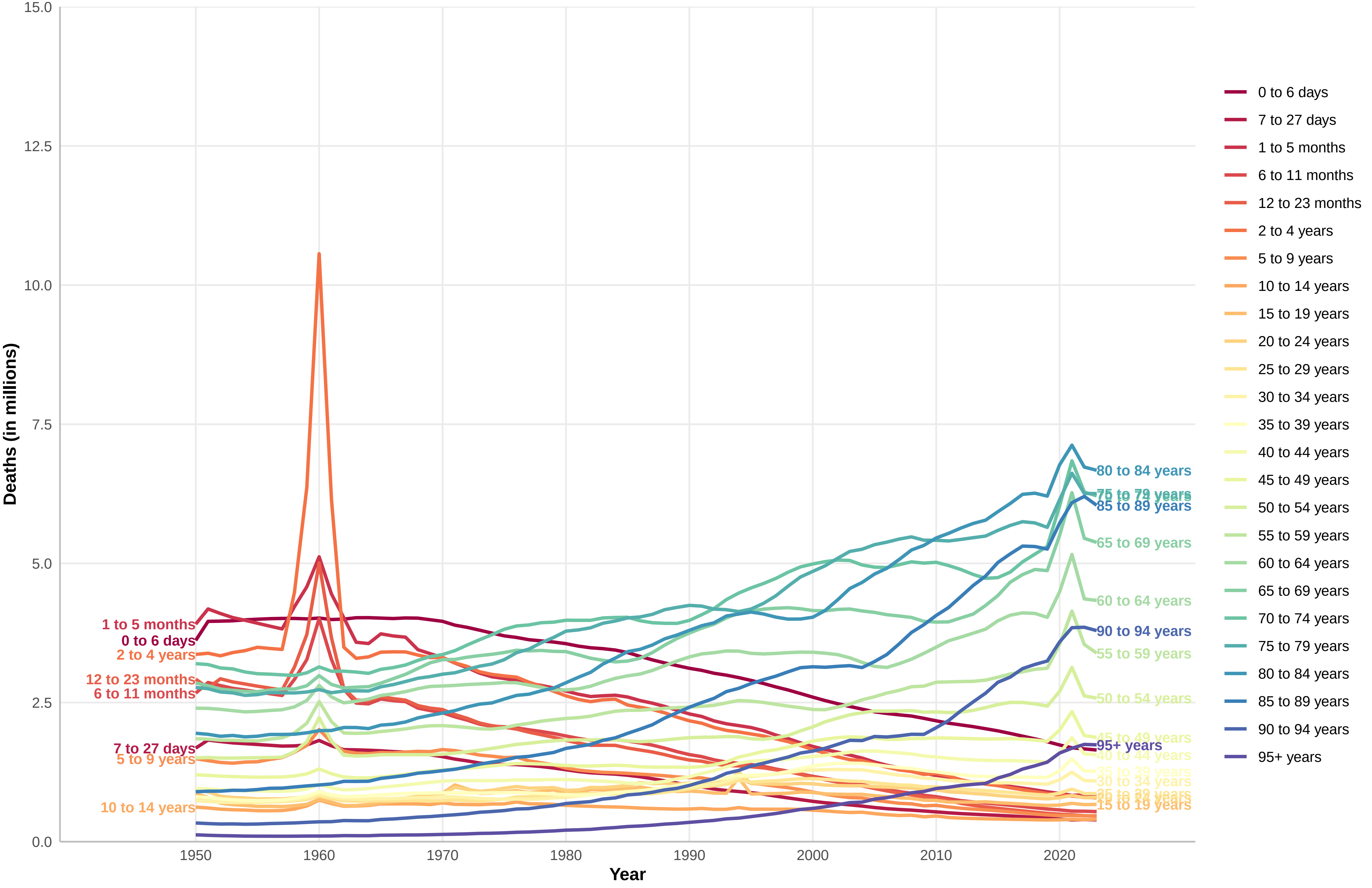

Appendix 2 Figure S2b: Number of global deaths over time by age group, females, 1950−2023

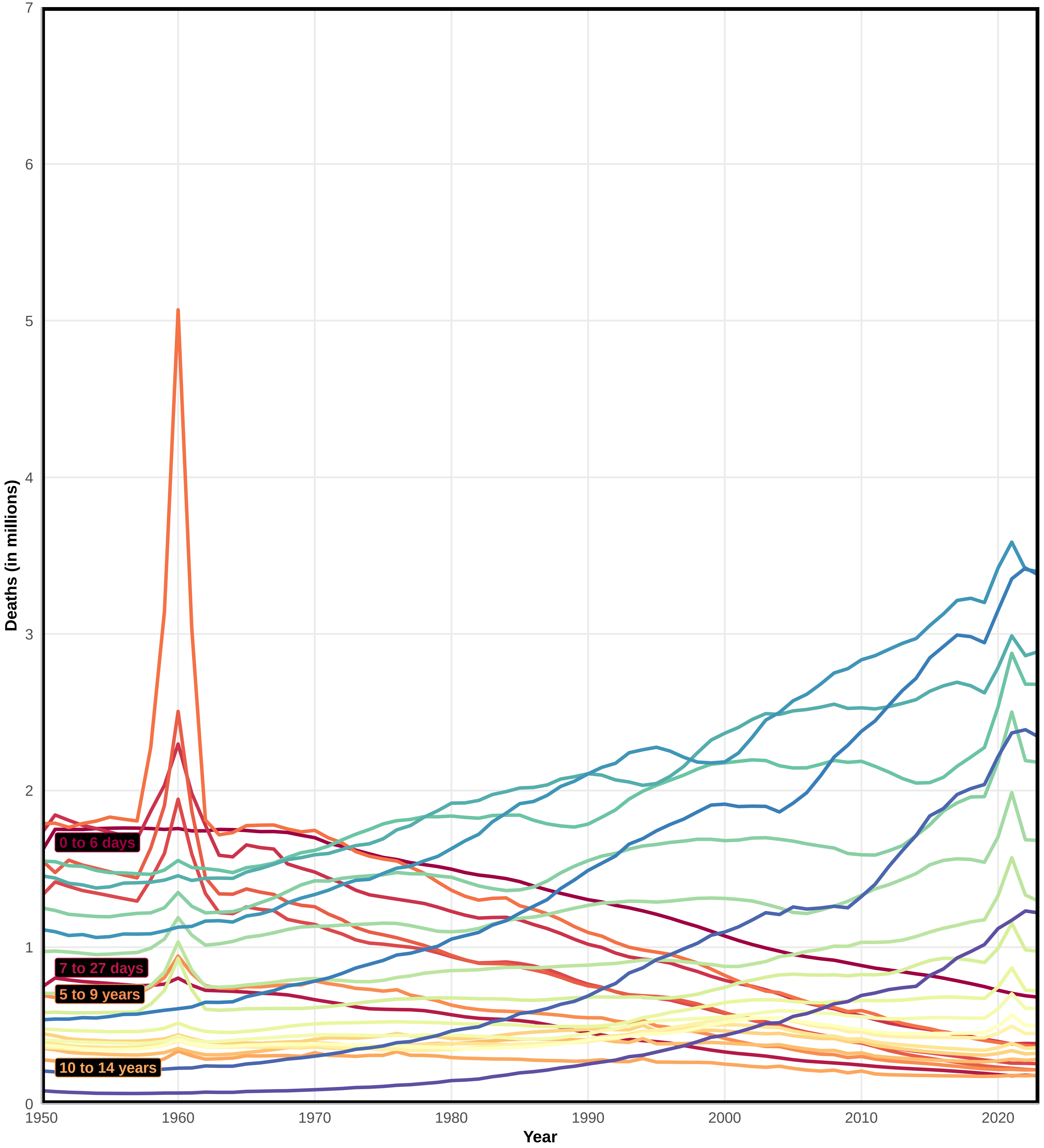

Appendix 2 Figure S2c: Number of global deaths over time by age group, males, 1950−2023

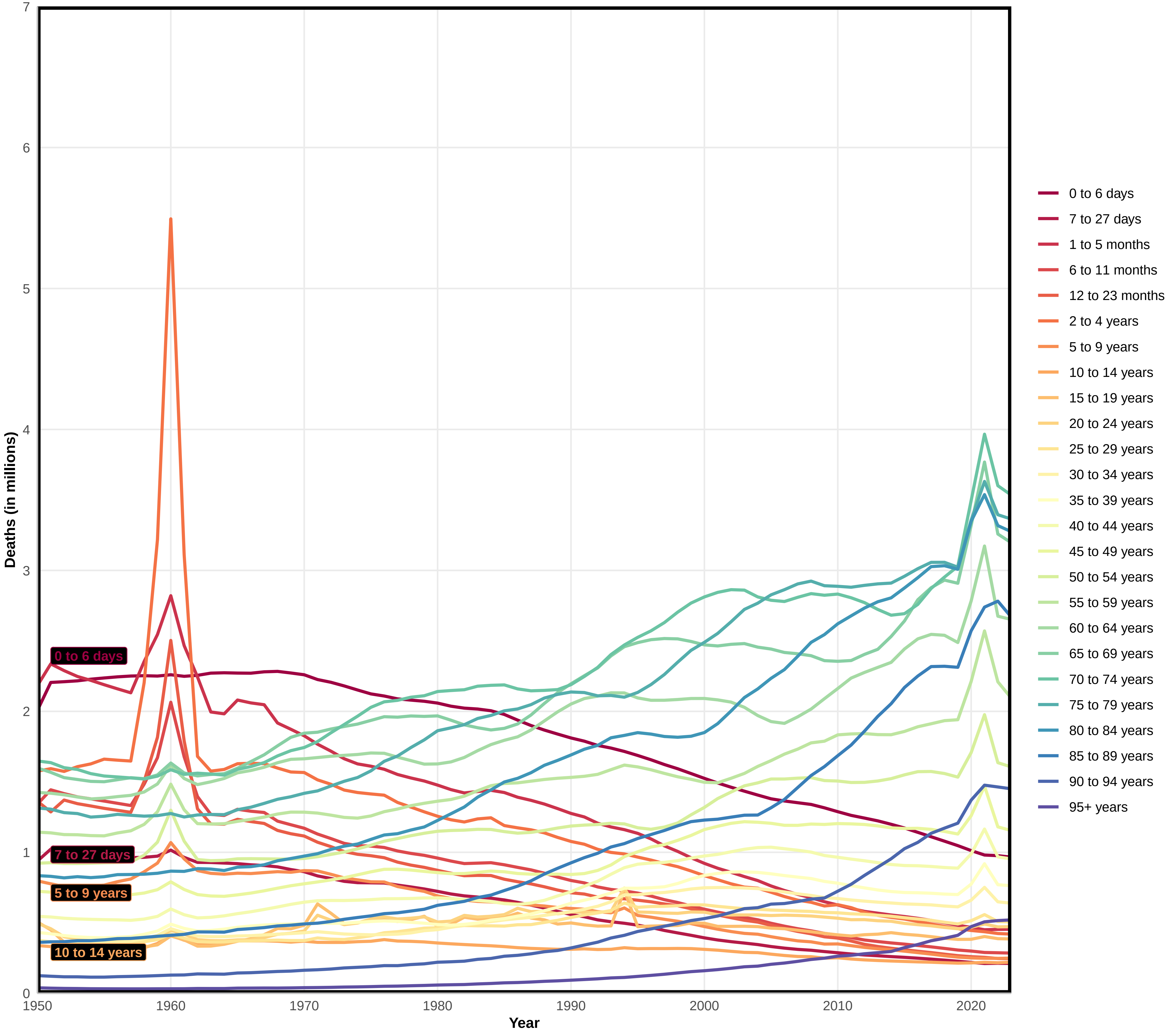

Appendix 2 Figure S3: Death Structure by SDI in 2023

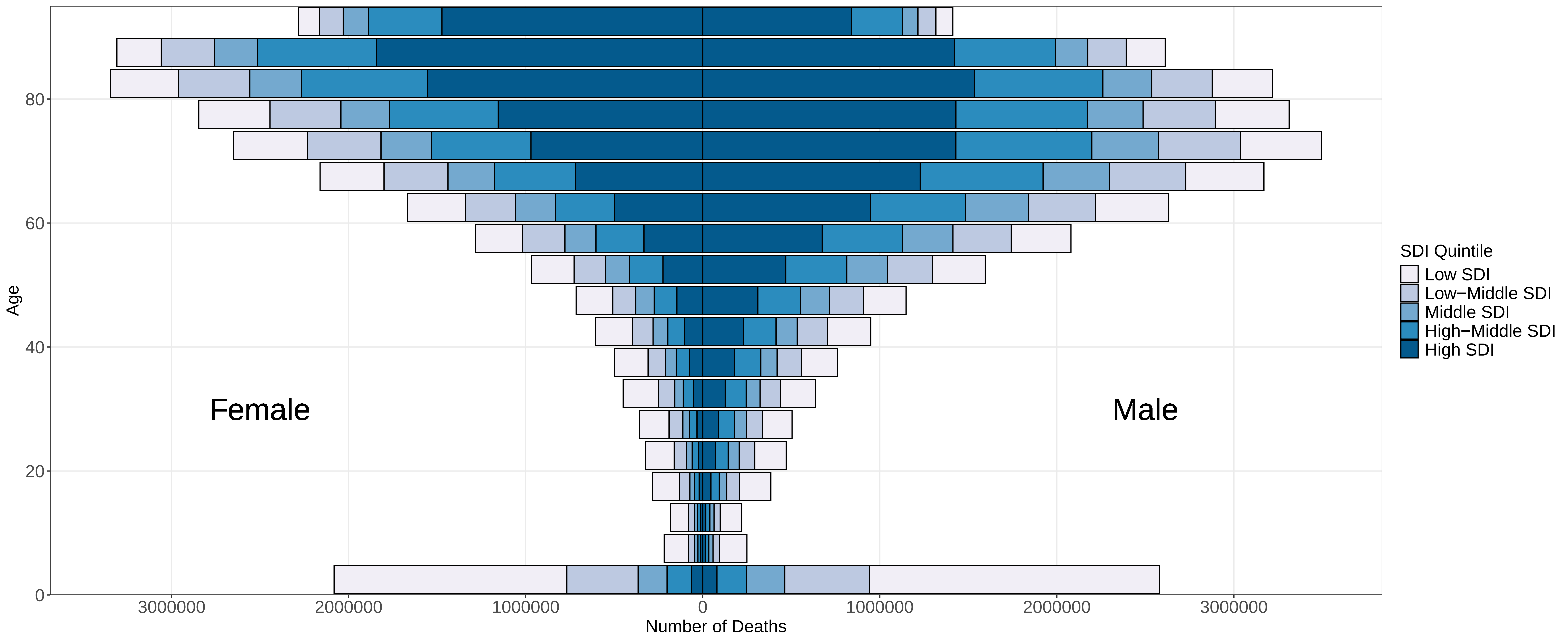

Appendix 2 Figure S4: Armenia mortality rates, 1950−2023

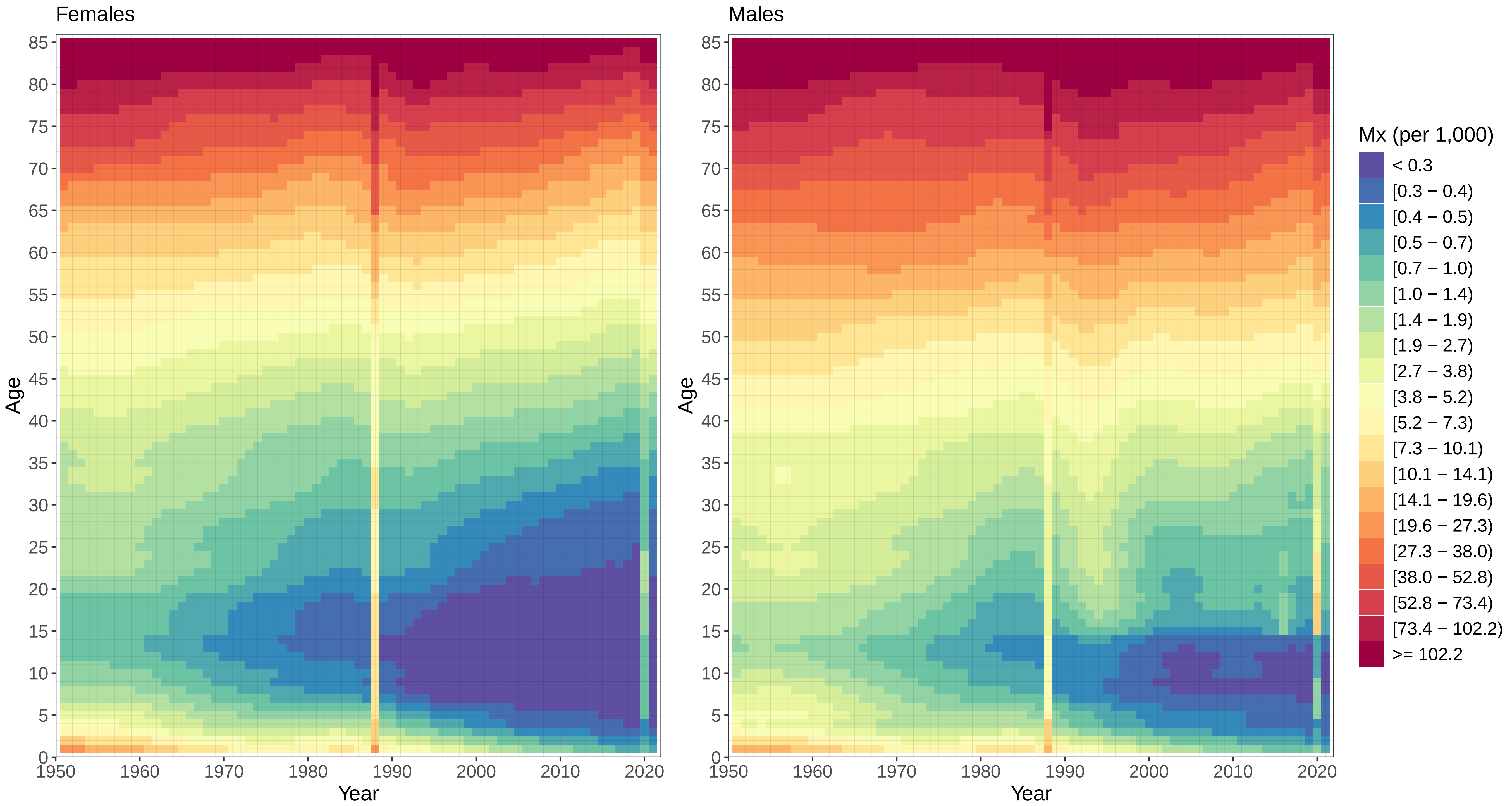

Appendix 2 Figure S4: Azerbaijan mortality rates, 1950−2023

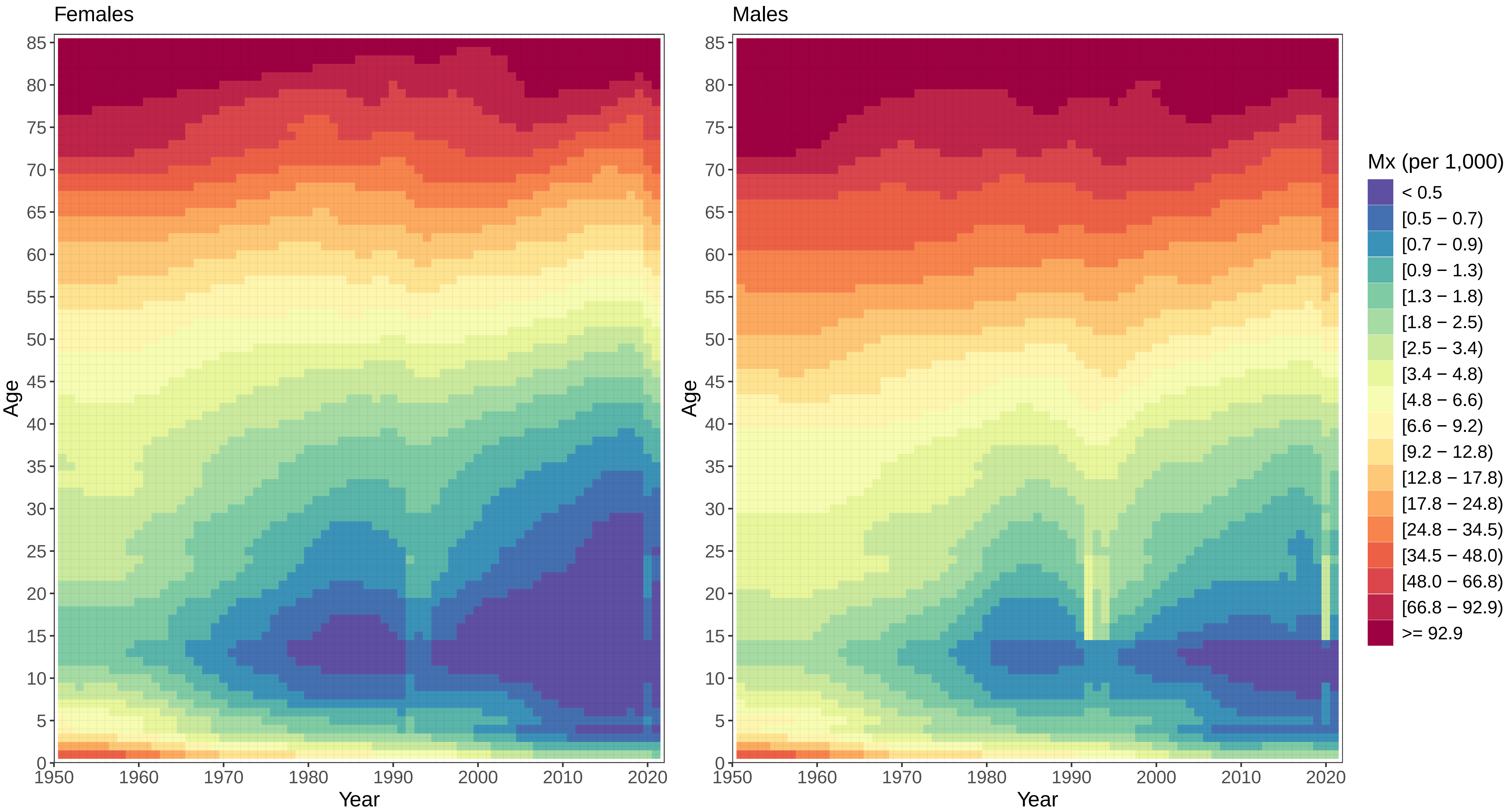

Appendix 2 Figure S4: Georgia mortality rates, 1950−2023

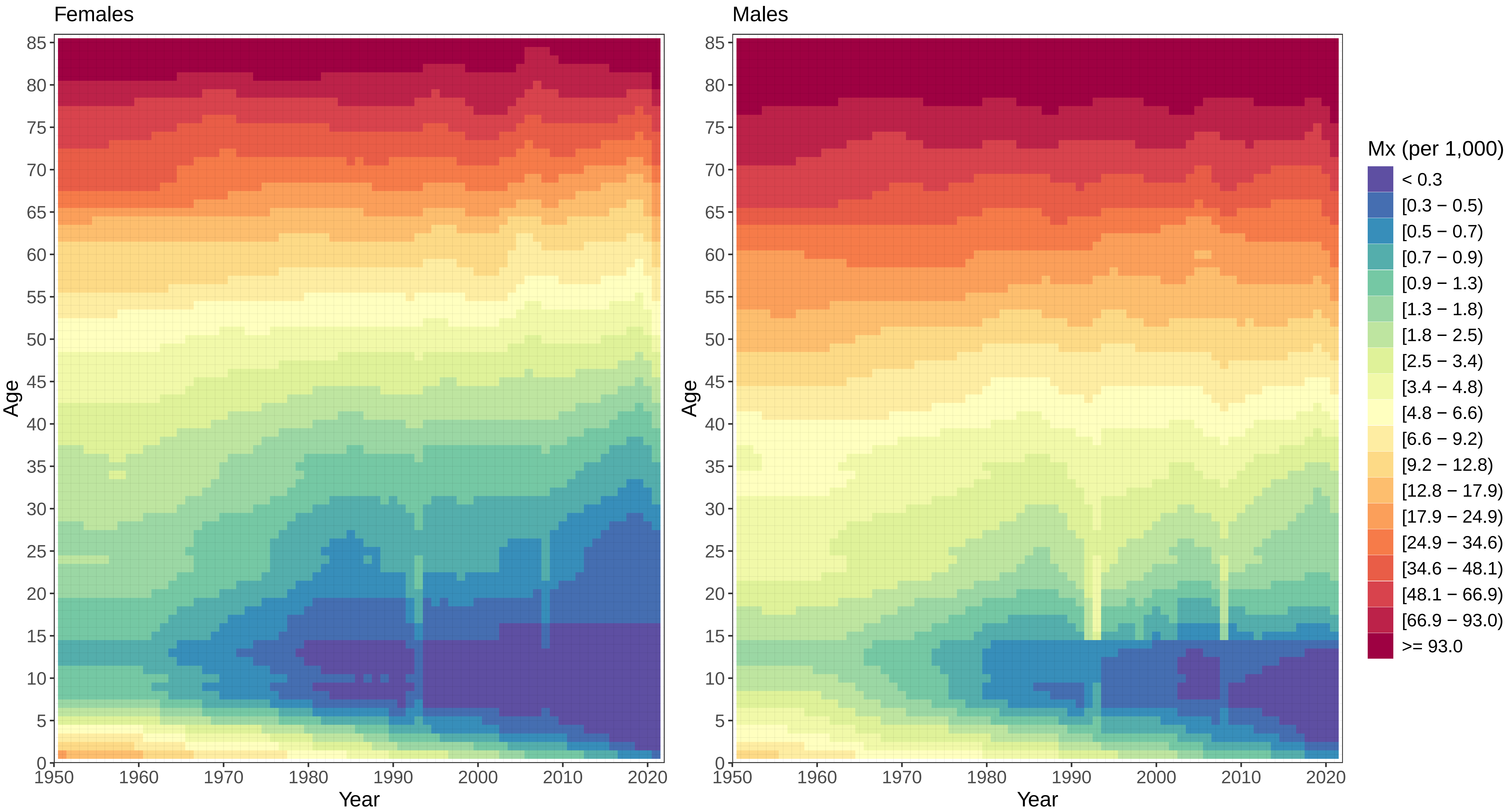

Appendix 2 Figure S4: Kazakhstan mortality rates, 1950−2023

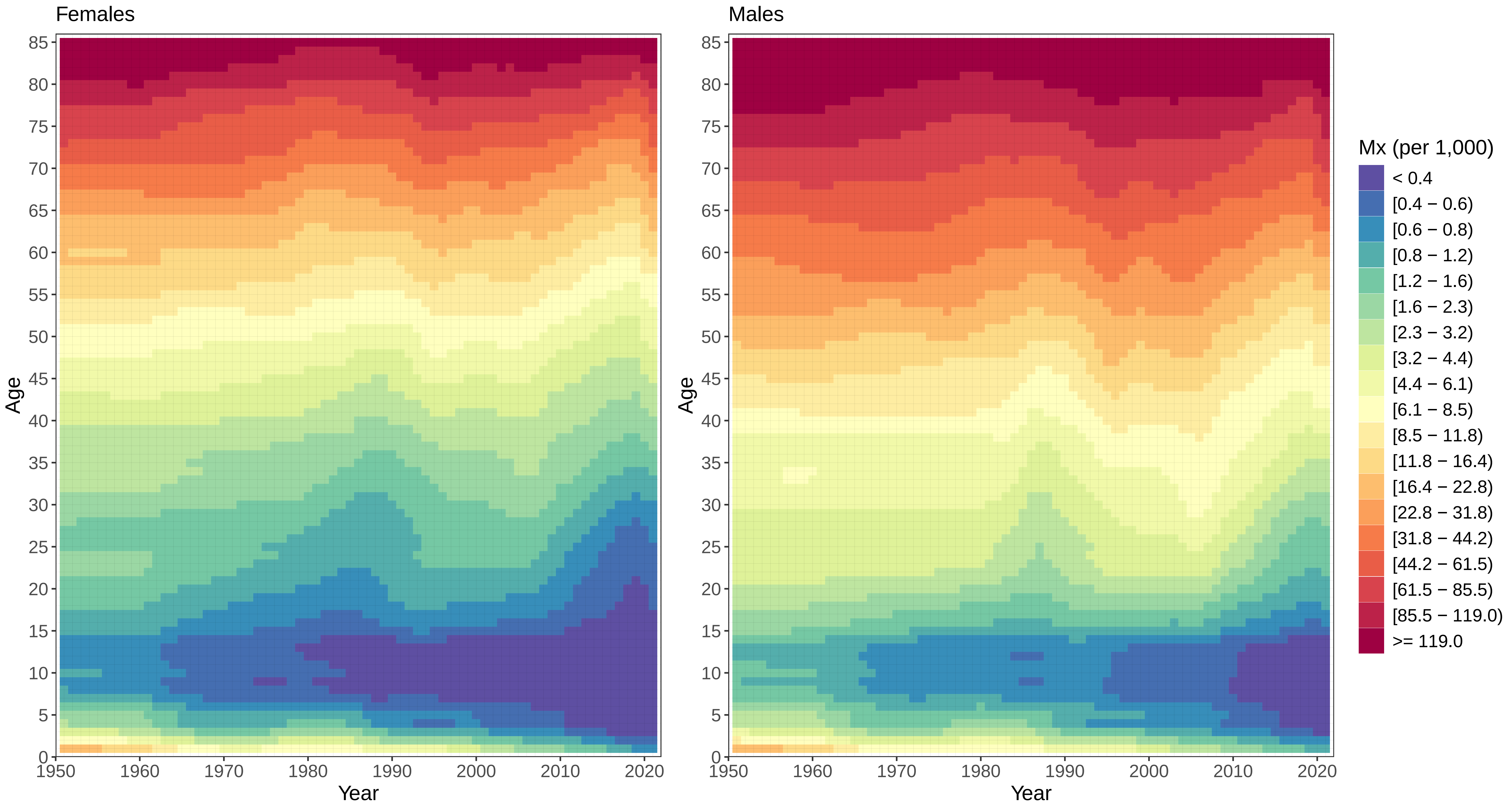

Appendix 2 Figure S4: Kyrgyzstan mortality rates, 1950−2023

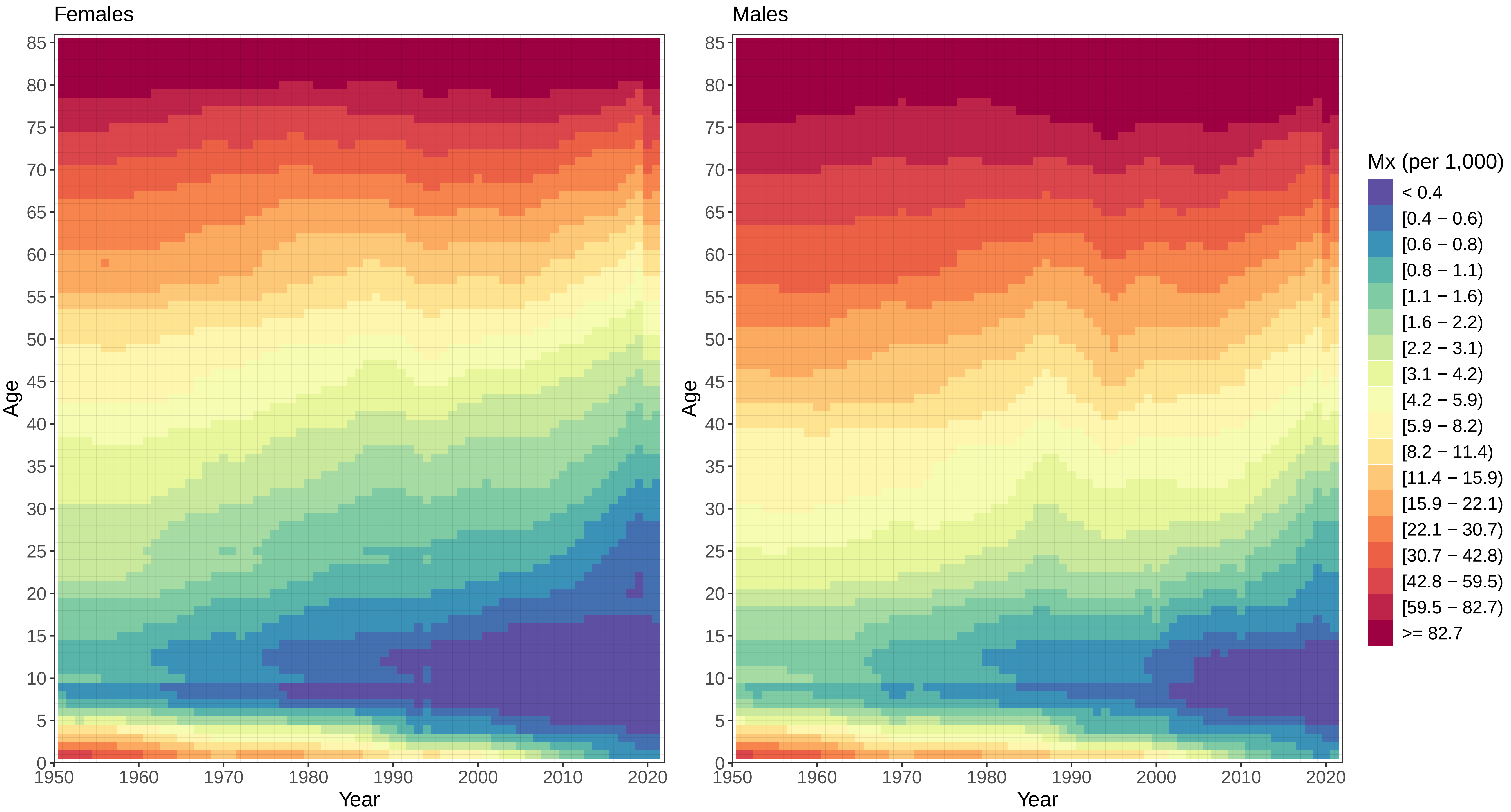

Appendix 2 Figure S4: Mongolia mortality rates, 1950−2023

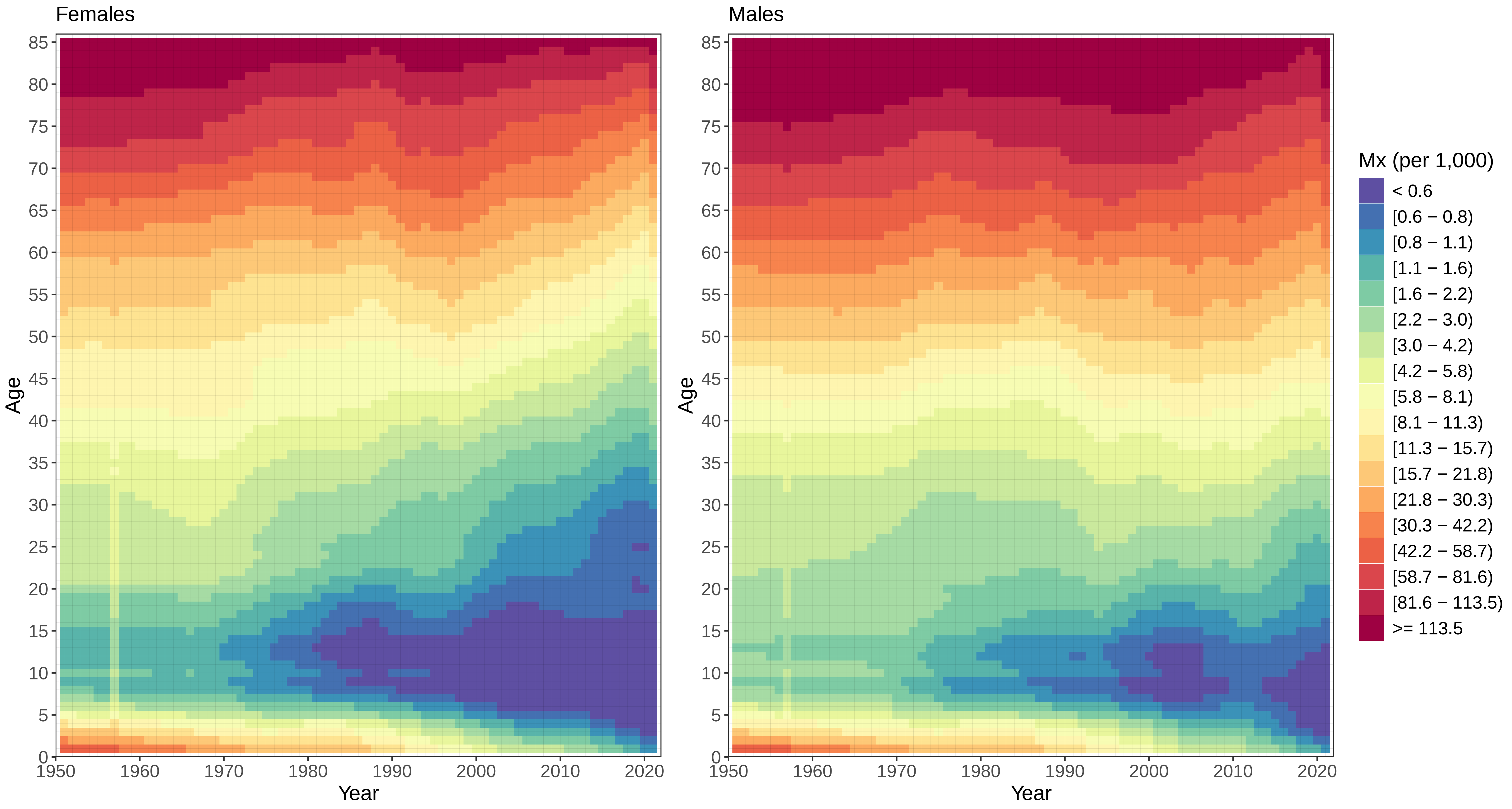

Appendix 2 Figure S4: Tajikistan mortality rates, 1950−2023

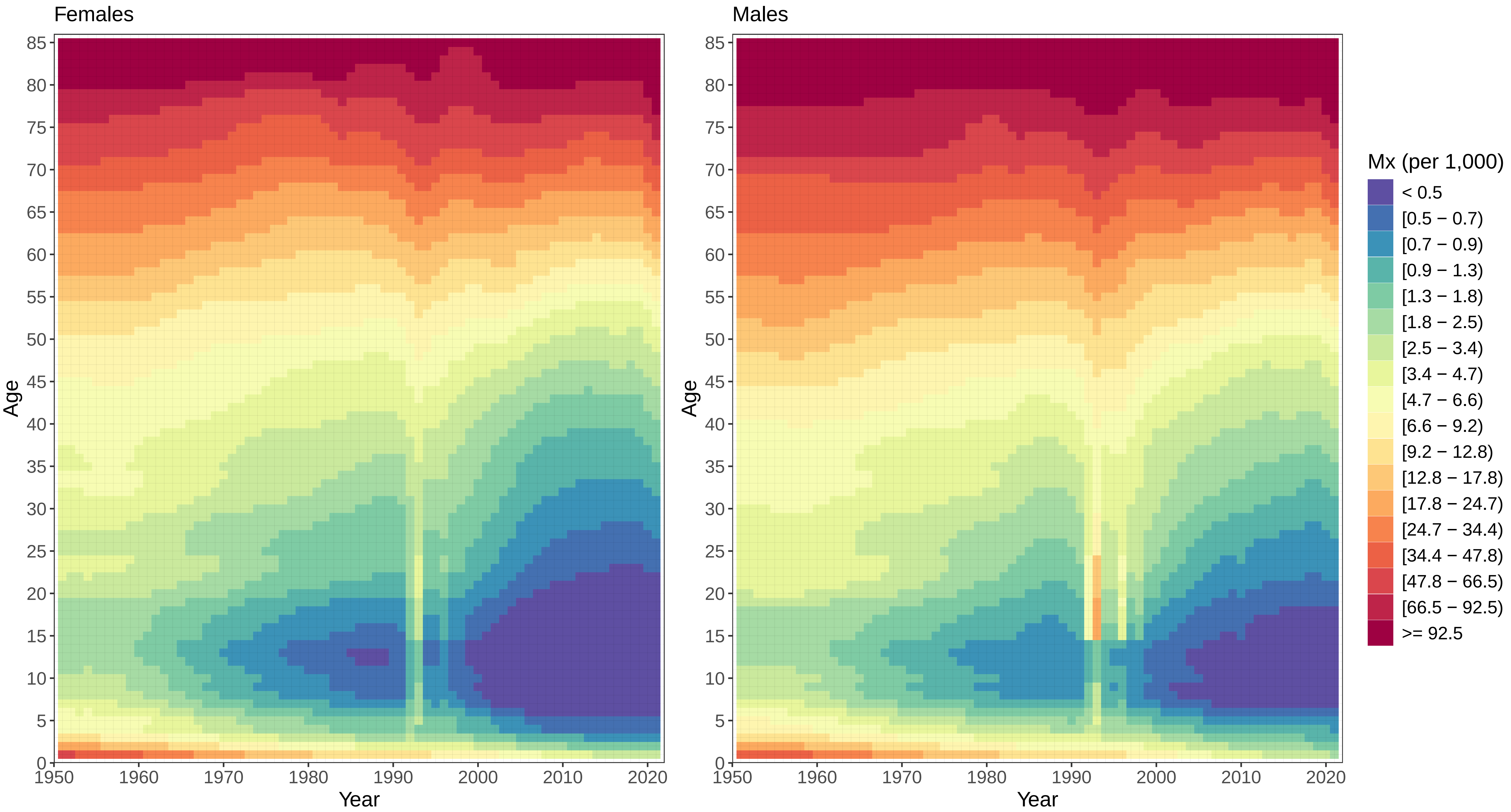

Appendix 2 Figure S4: Turkmenistan mortality rates, 1950−2023

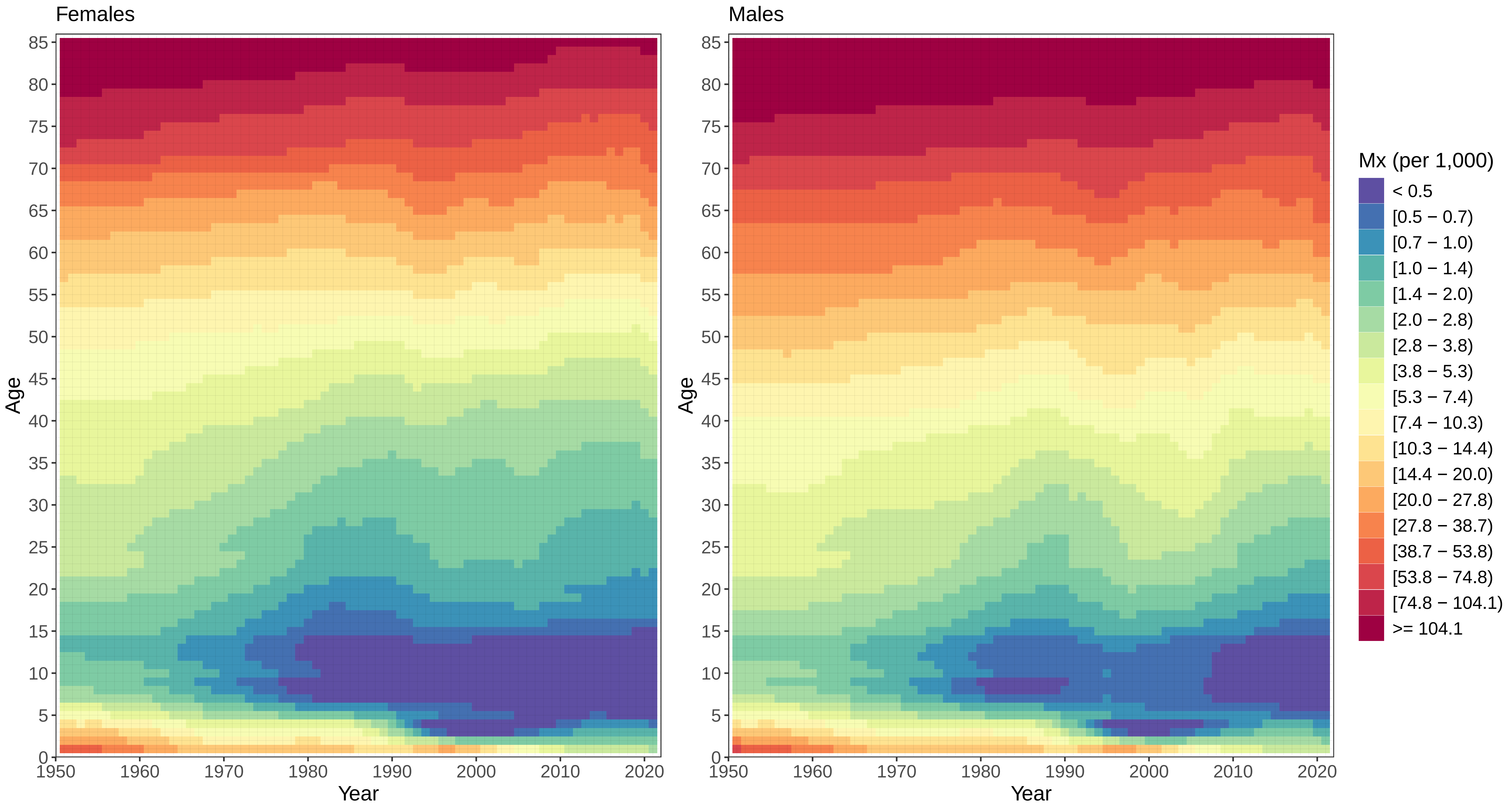

Appendix 2 Figure S4: Uzbekistan mortality rates, 1950−2023

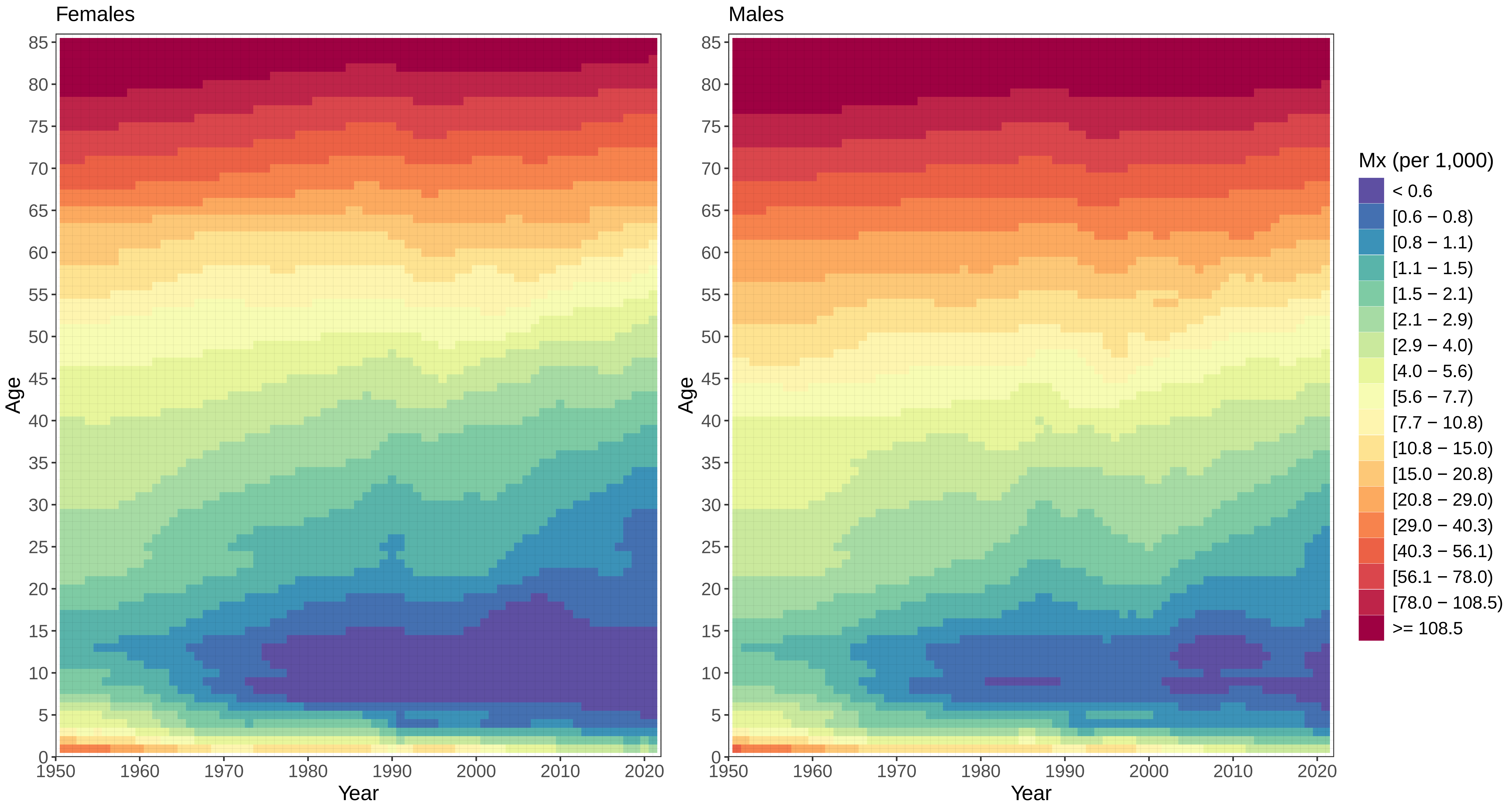

Appendix 2 Figure S4: Albania mortality rates, 1950−2023

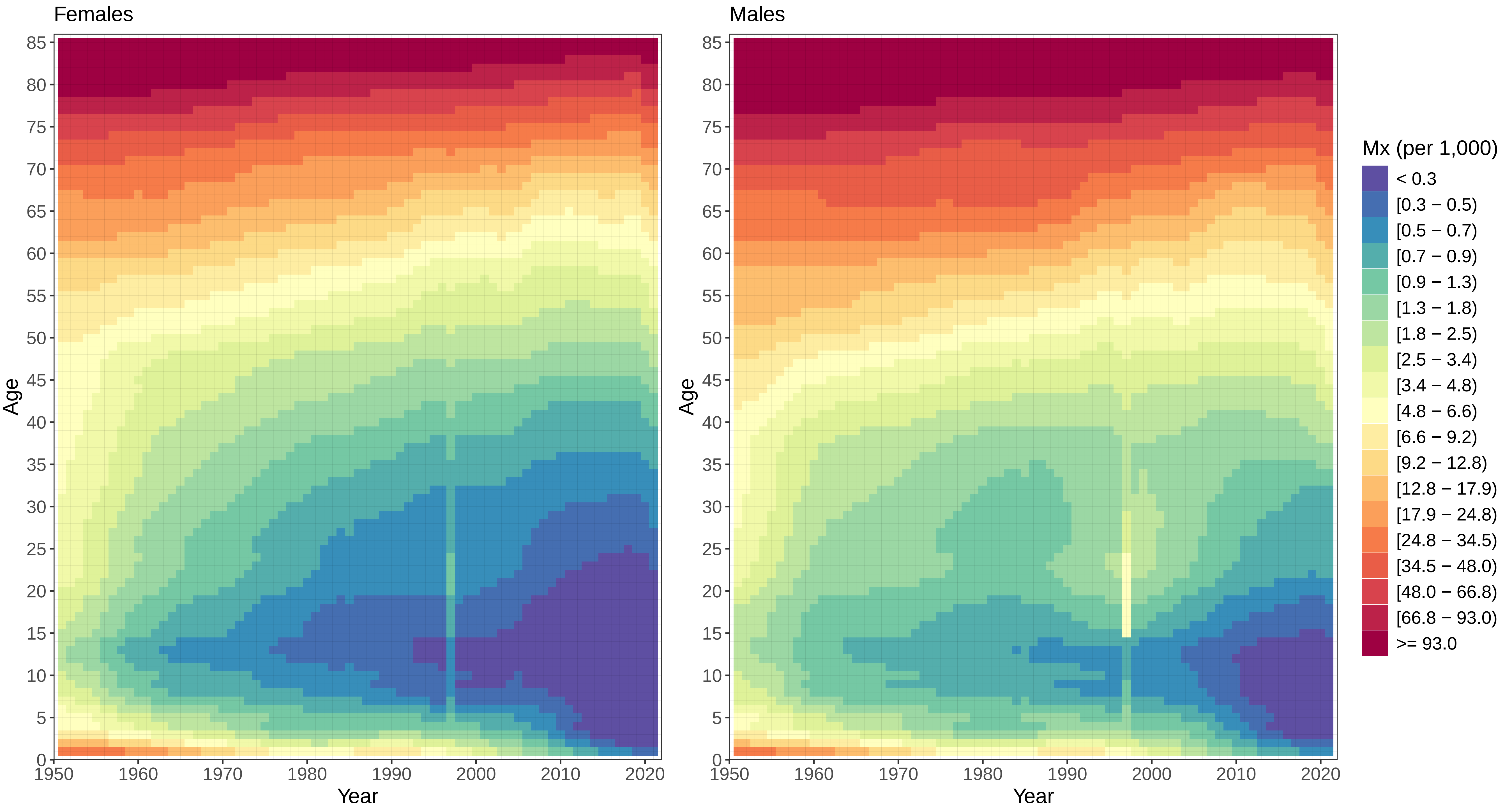

Appendix 2 Figure S4: Bosnia and Herzegovina mortality rates, 1950−2023

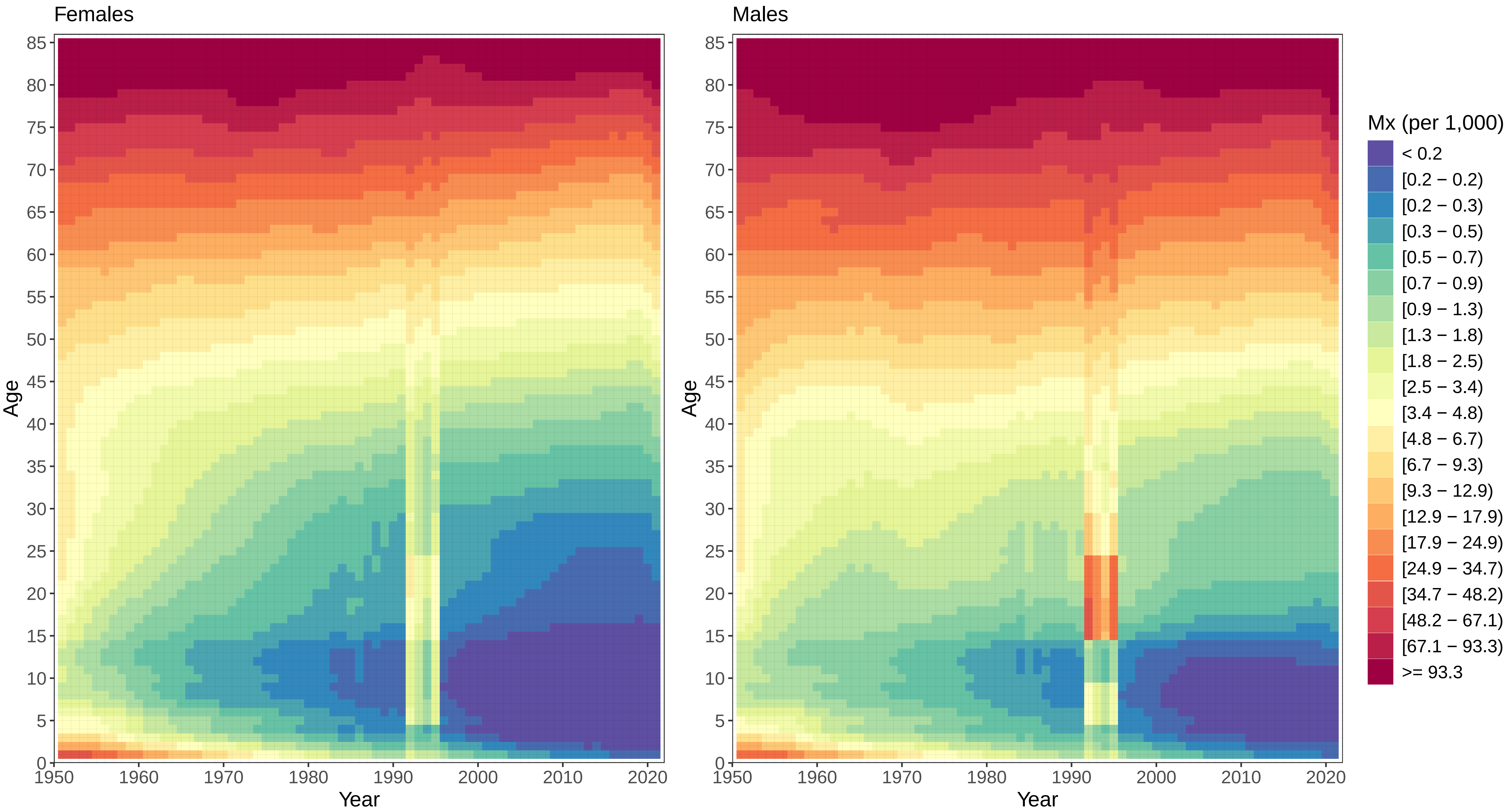

Appendix 2 Figure S4: Bulgaria mortality rates, 1950−2023

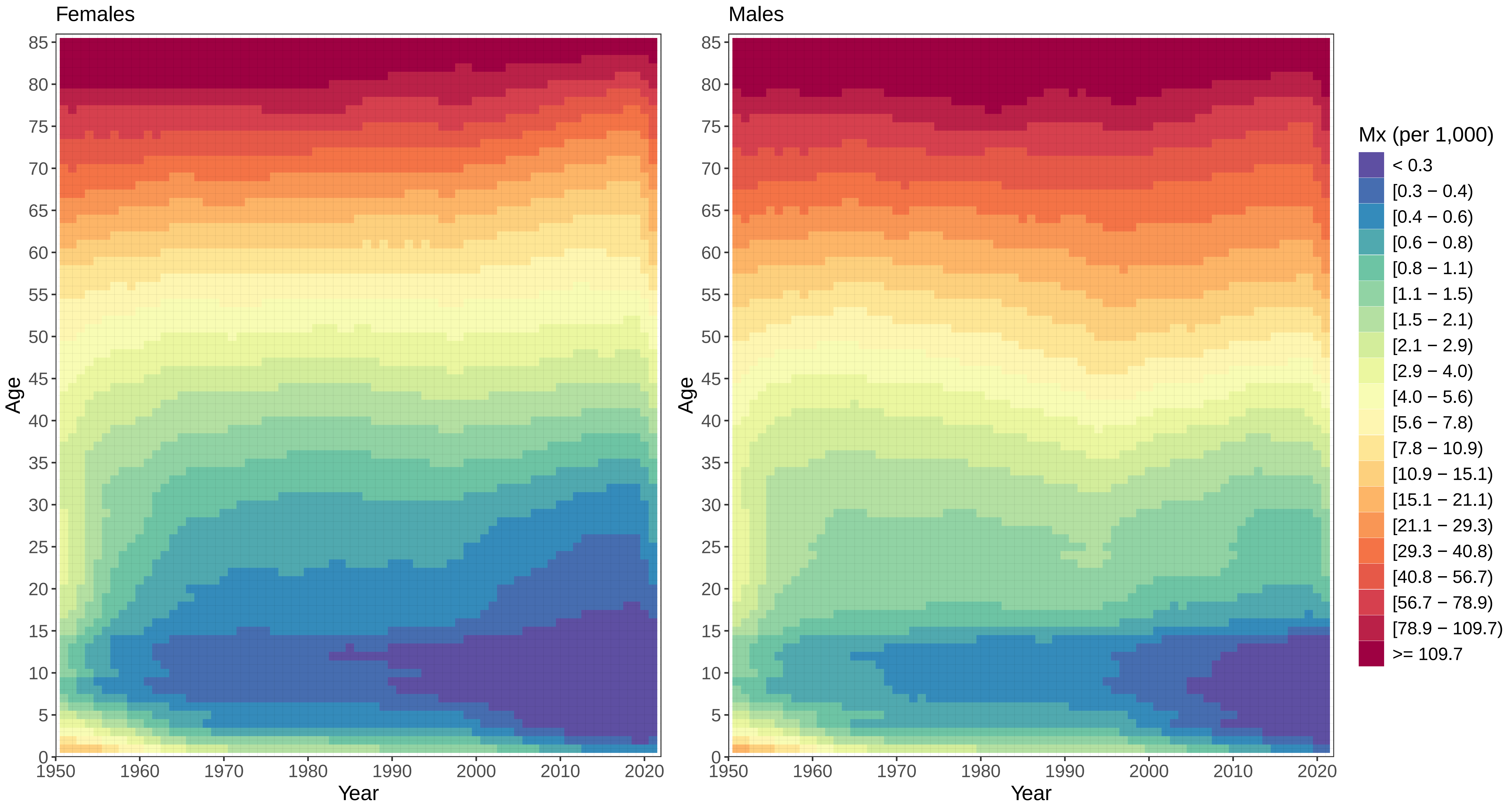

Appendix 2 Figure S4: Croatia mortality rates, 1950−2023

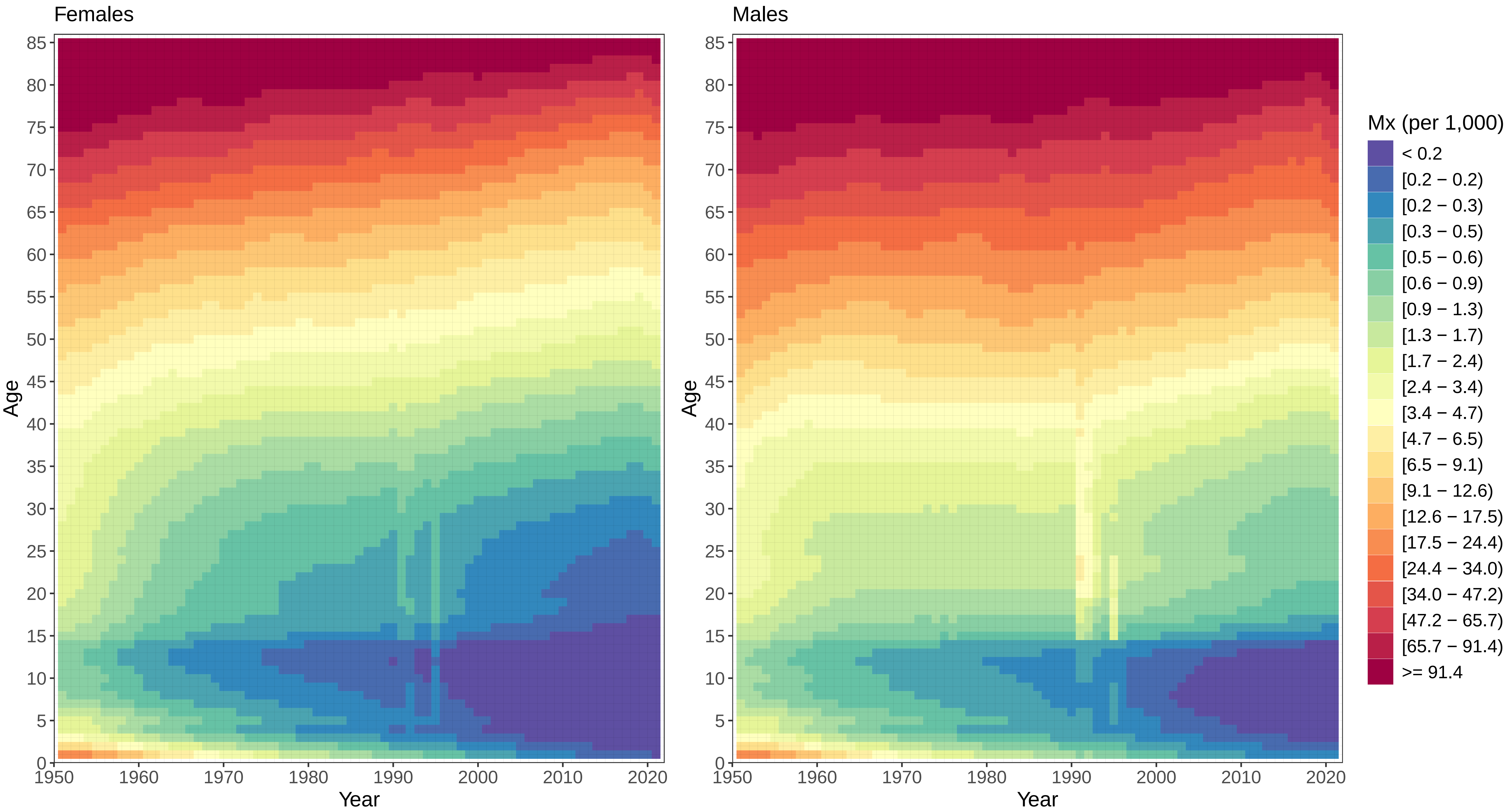

Appendix 2 Figure S4: Czechia mortality rates, 1950−2023

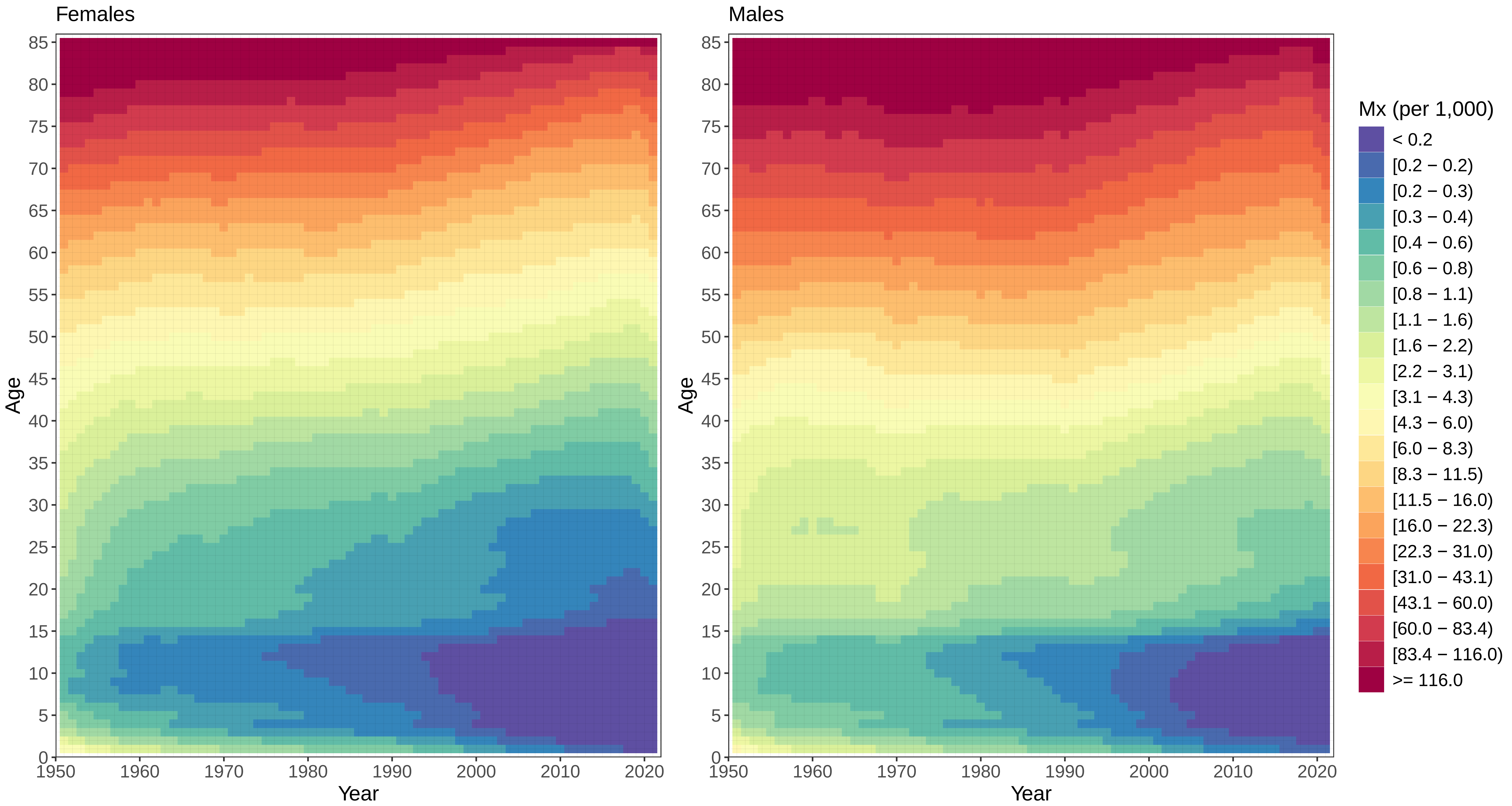

Appendix 2 Figure S4: Hungary mortality rates, 1950−2023

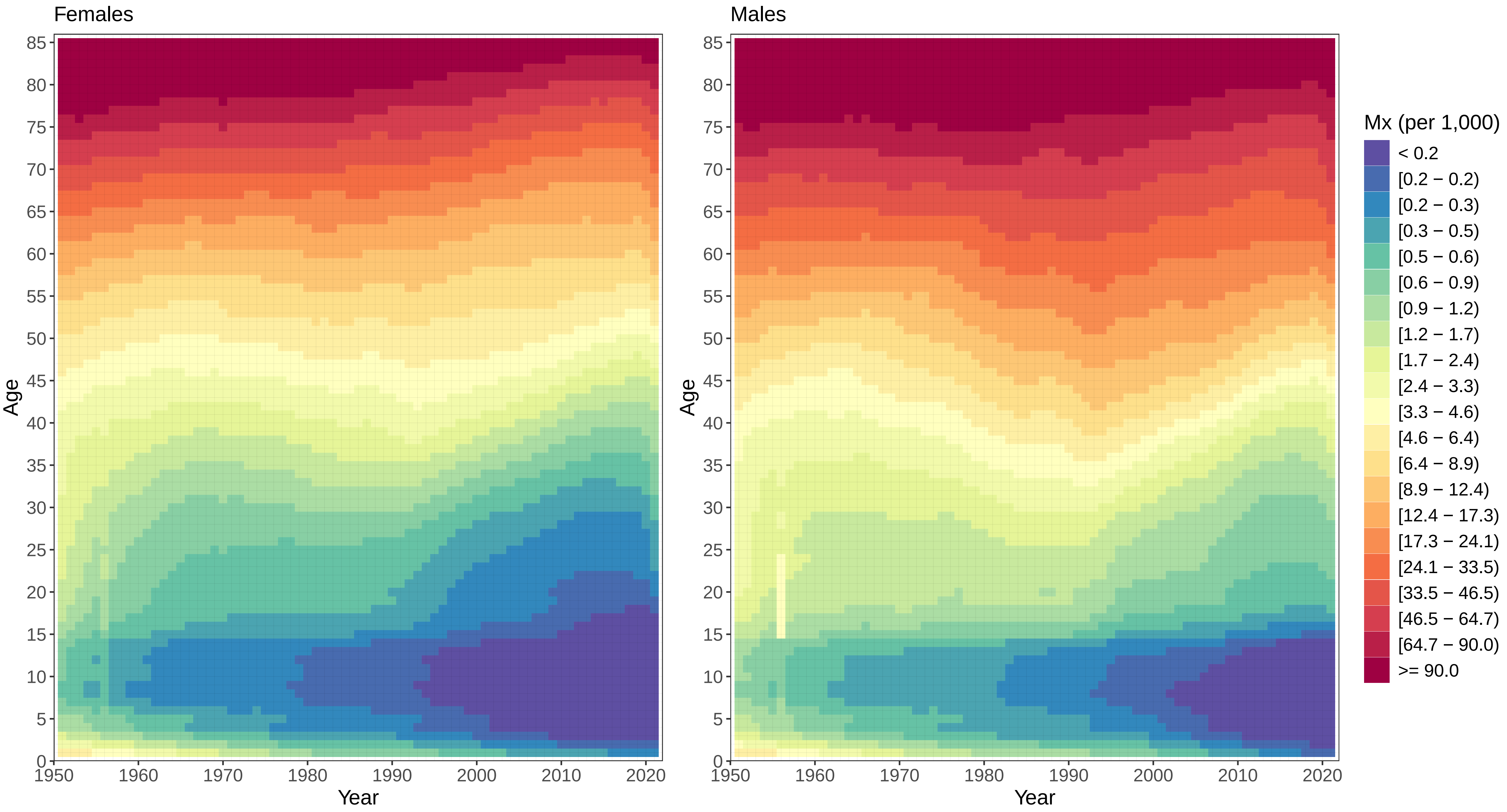

Appendix 2 Figure S4: Montenegro mortality rates, 1950−2023

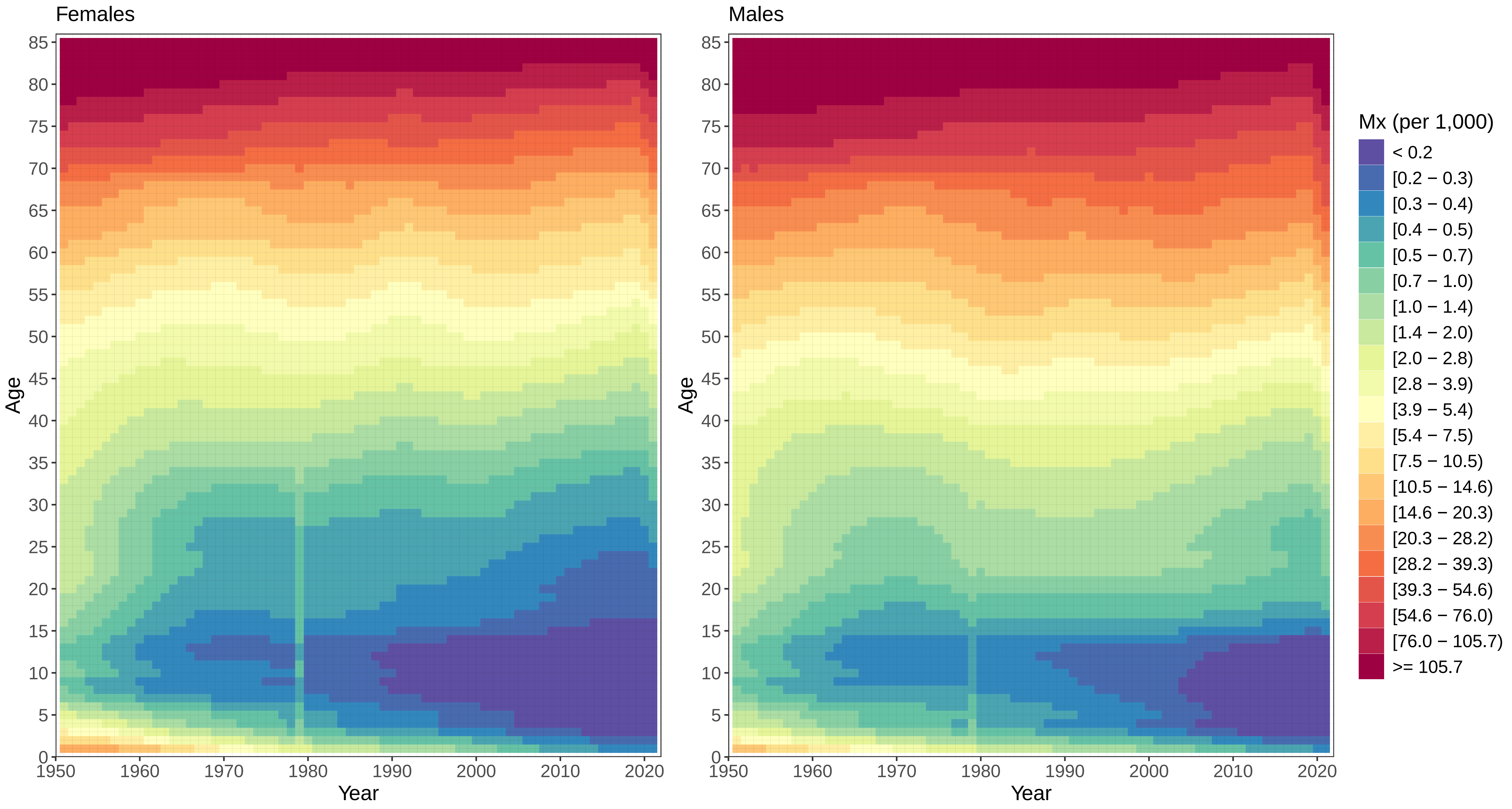

Appendix 2 Figure S4: North Macedonia mortality rates, 1950−2023

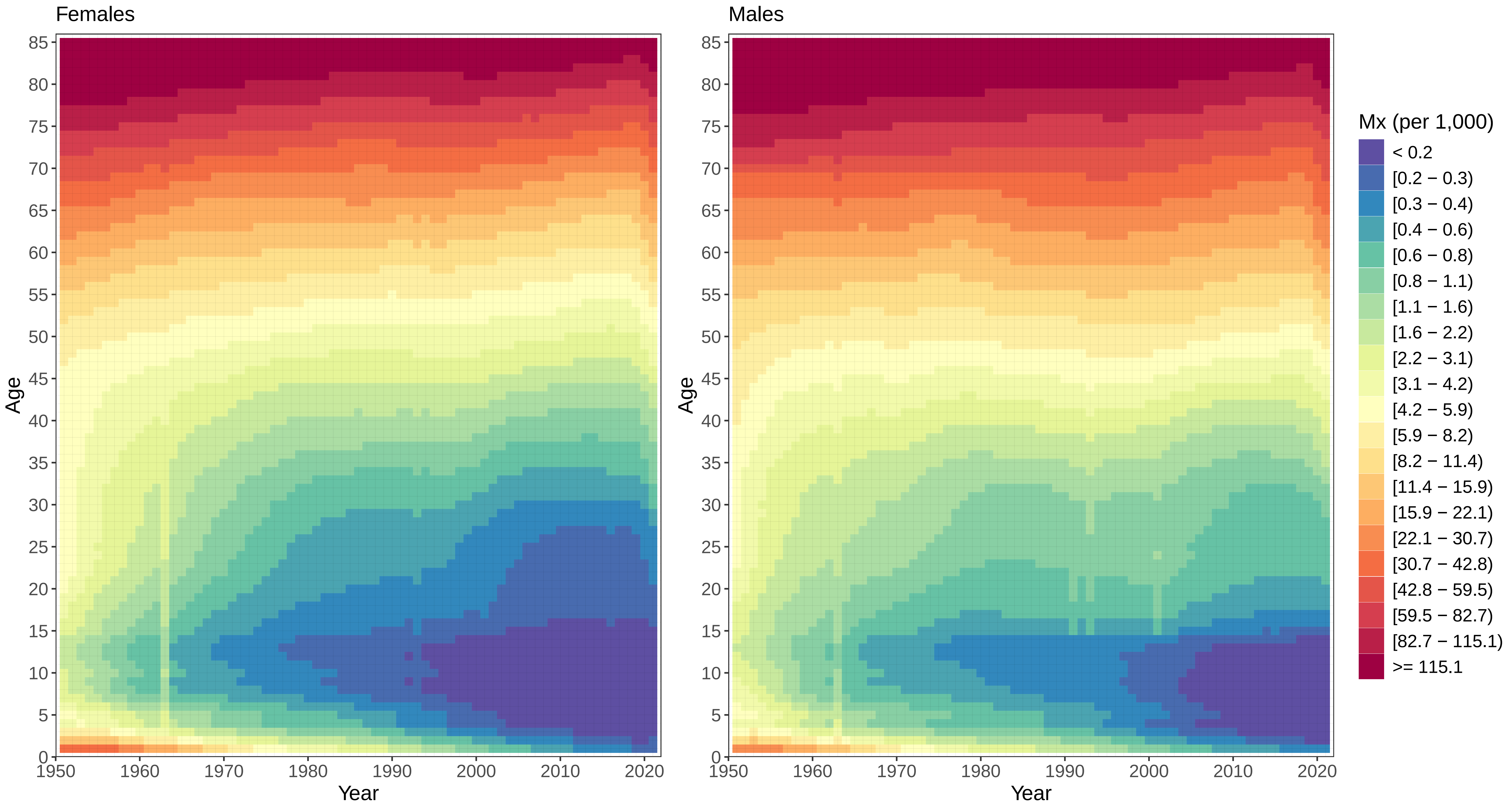

Appendix 2 Figure S4: Poland mortality rates, 1950−2023

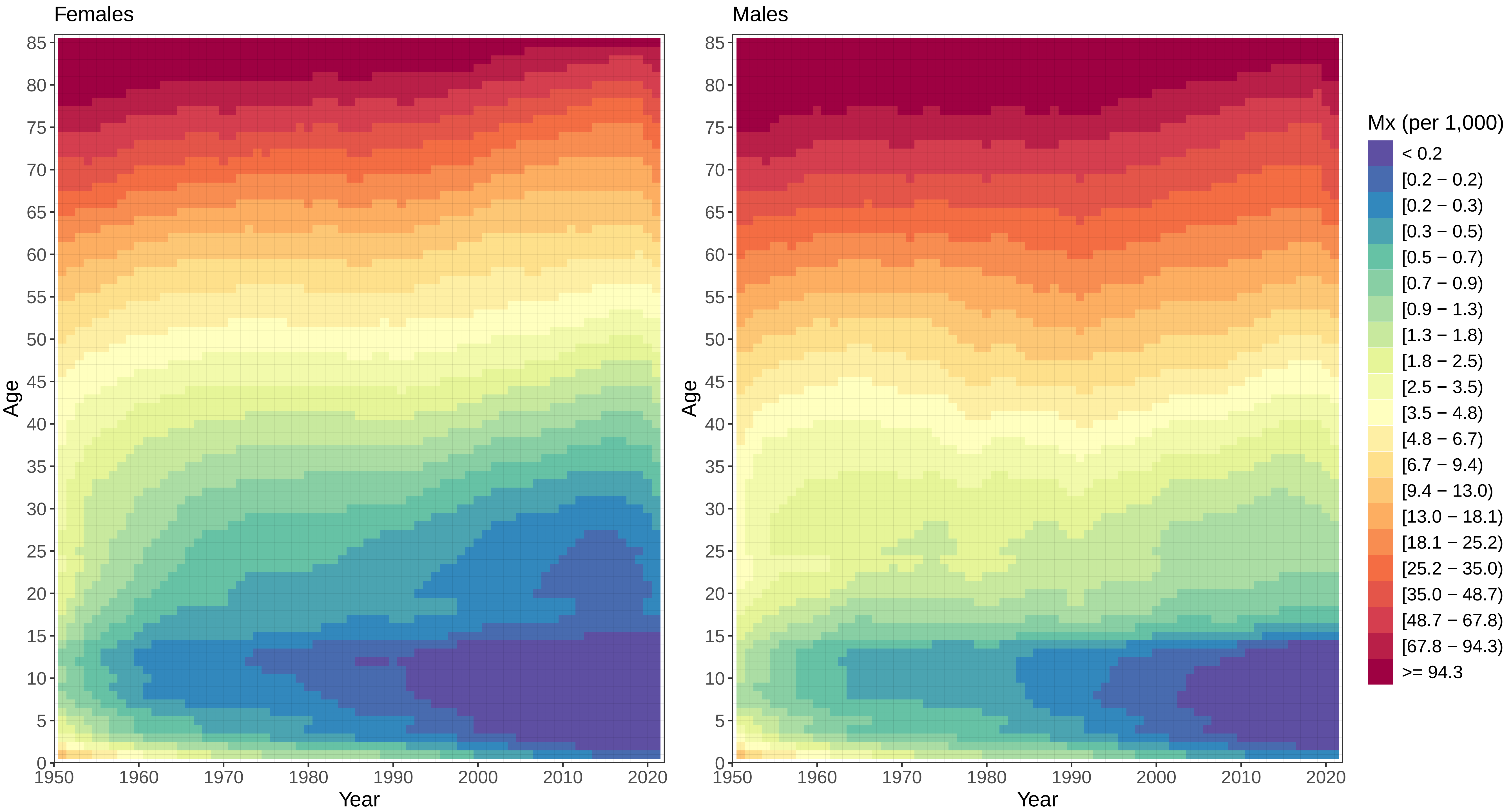

Appendix 2 Figure S4: Romania mortality rates, 1950−2023

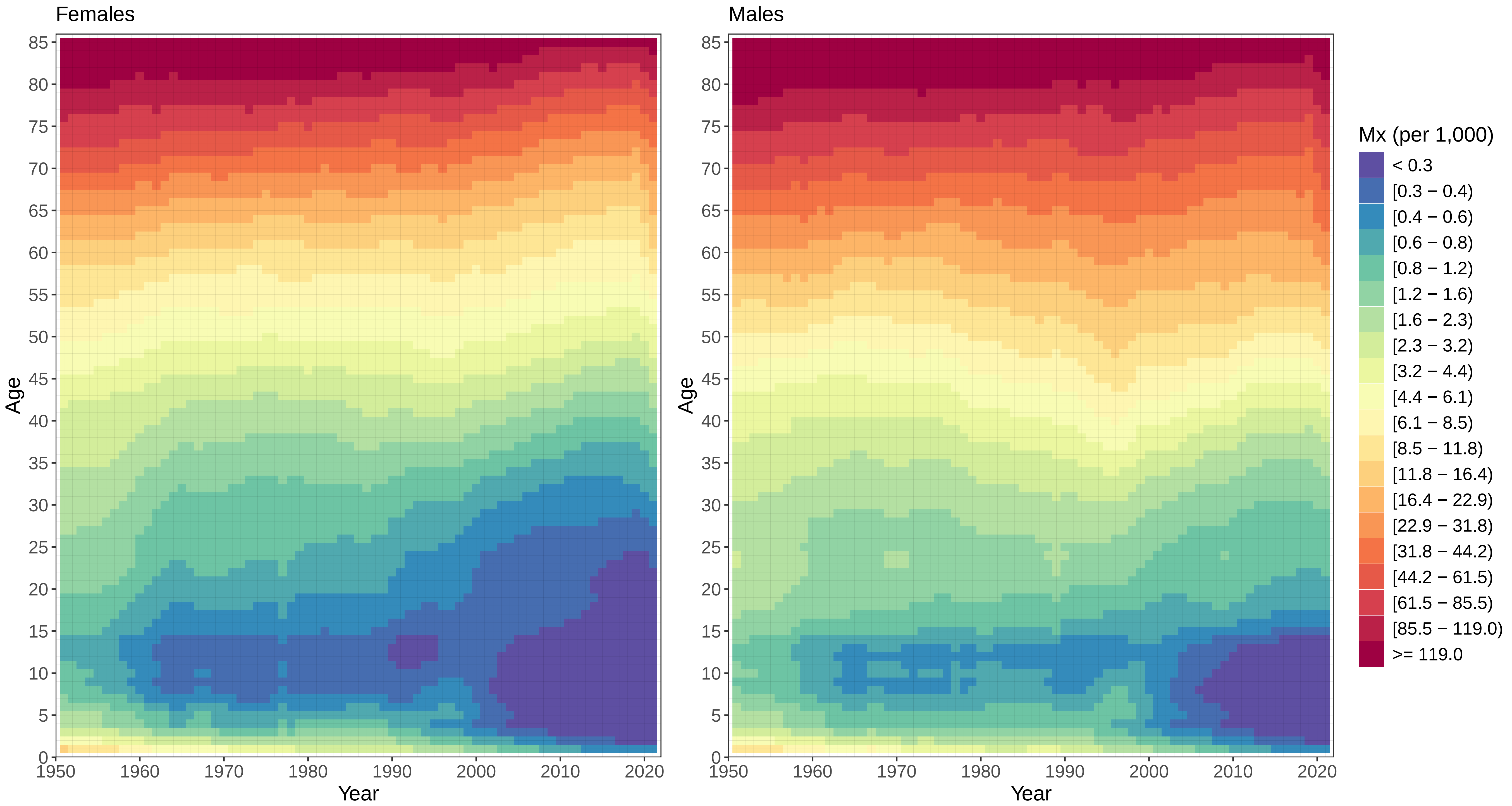

Appendix 2 Figure S4: Serbia mortality rates, 1950−2023

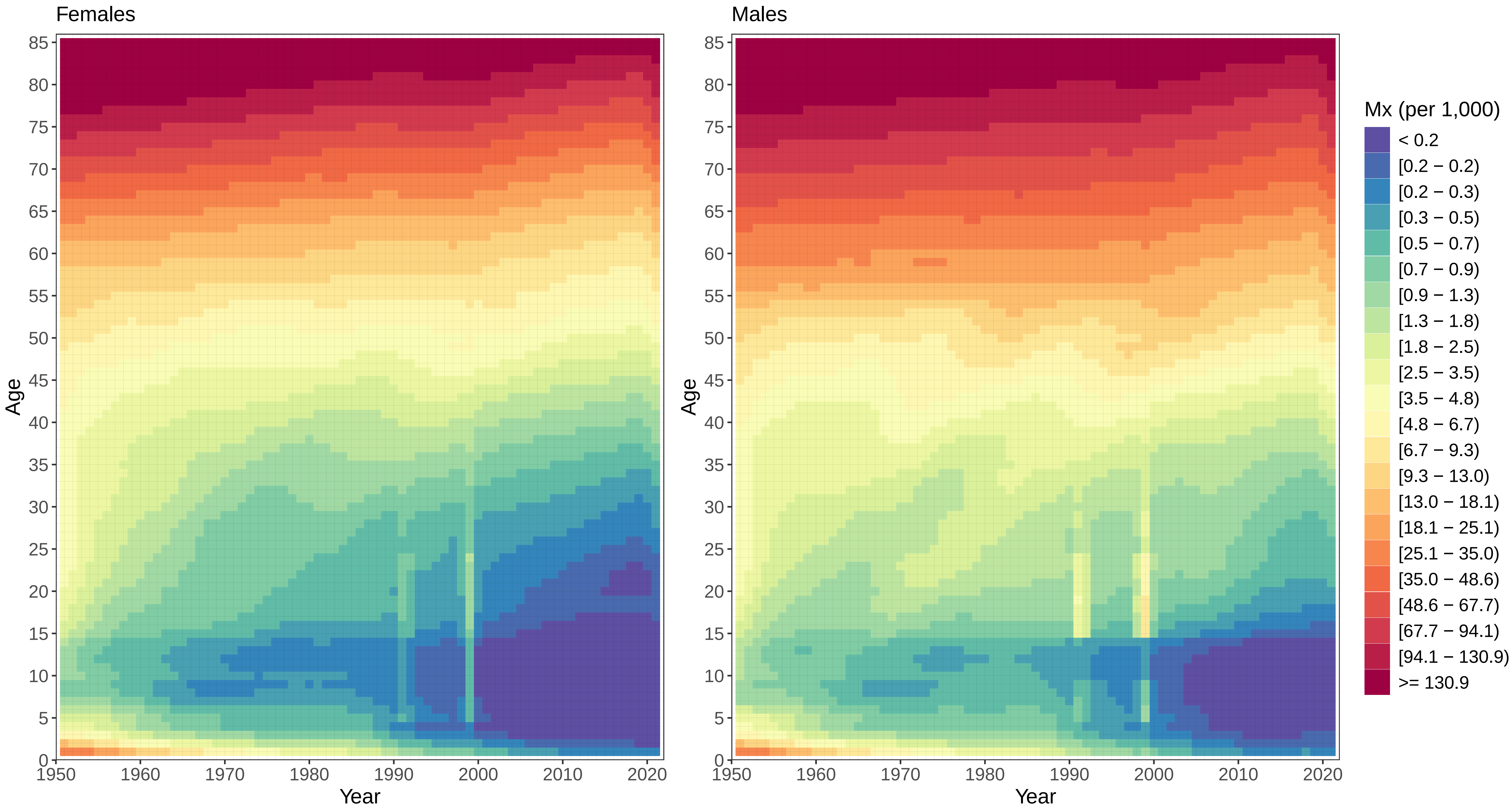

Appendix 2 Figure S4: Slovakia mortality rates, 1950−2023

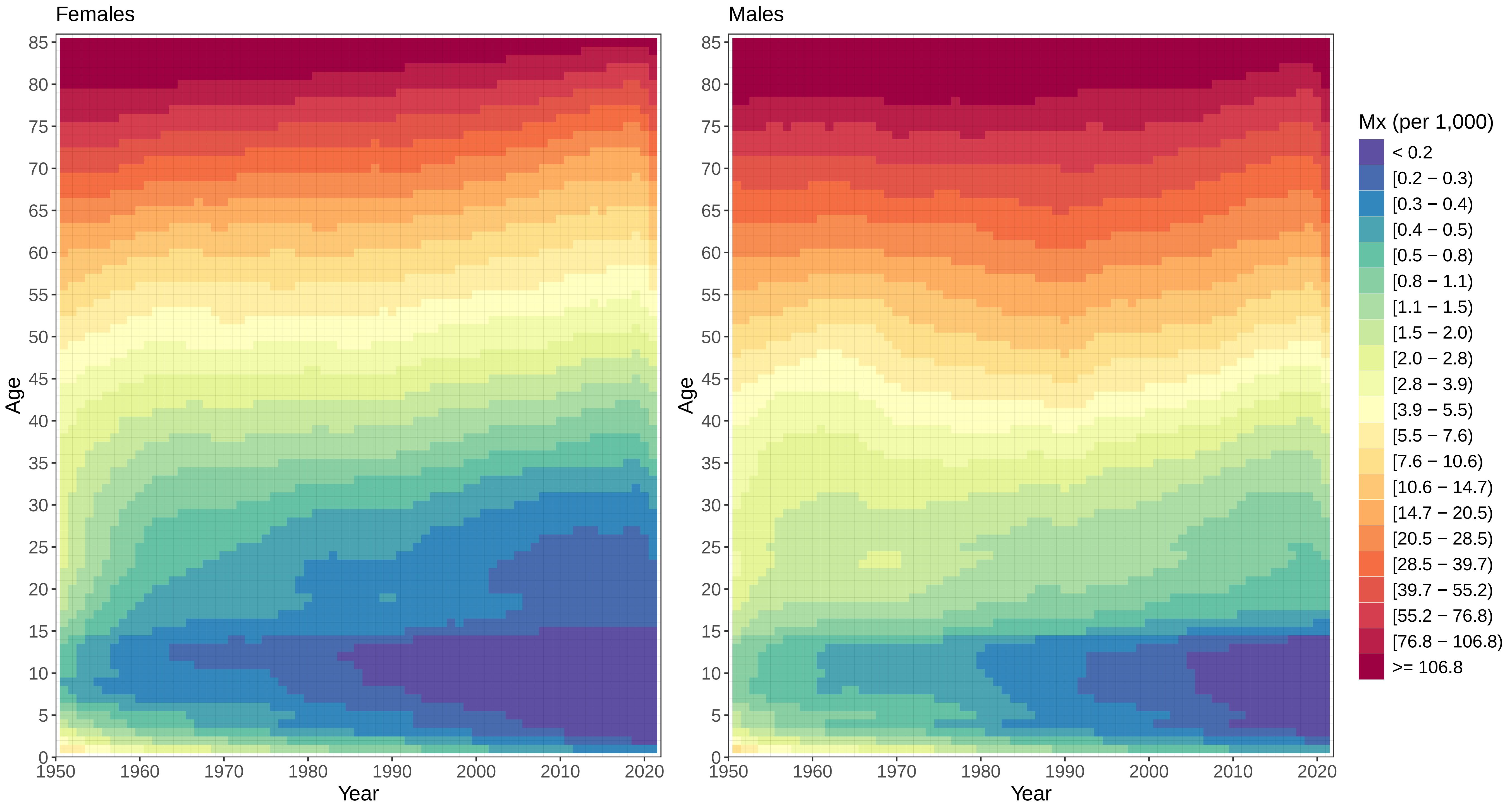

Appendix 2 Figure S4: Slovenia mortality rates, 1950−2023

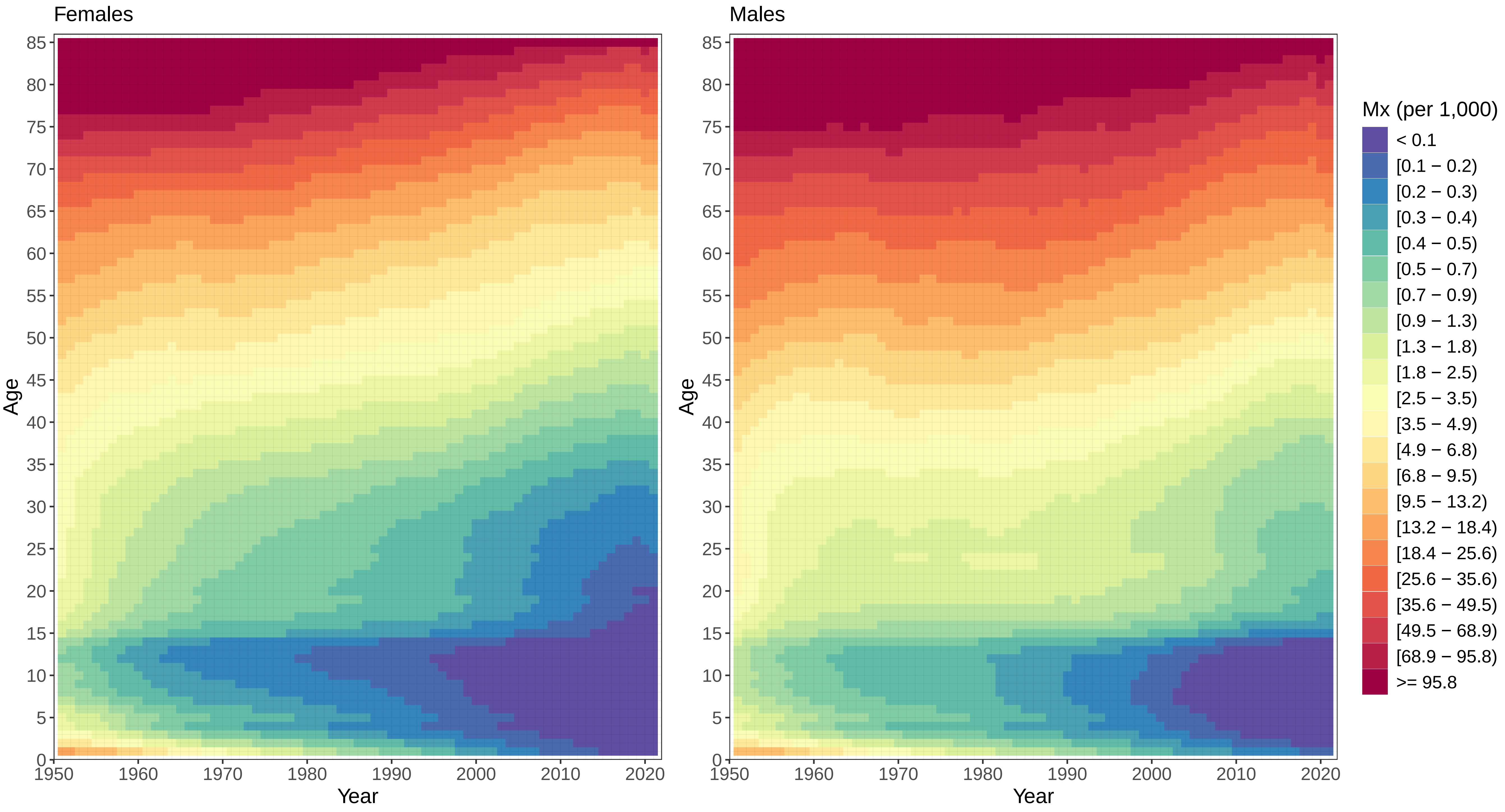

Appendix 2 Figure S4: Belarus mortality rates, 1950−2023

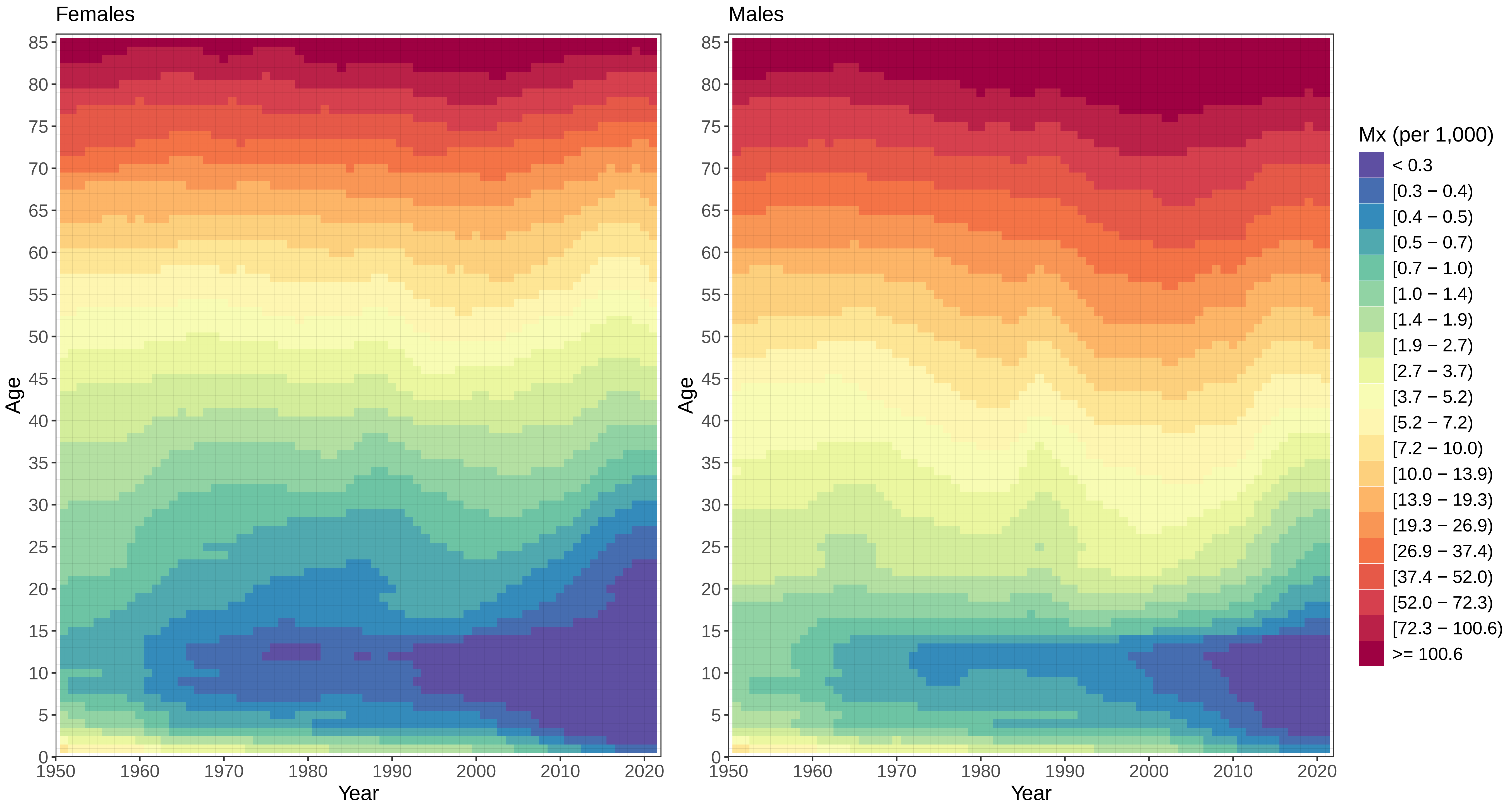

Appendix 2 Figure S4: Estonia mortality rates, 1950−2023

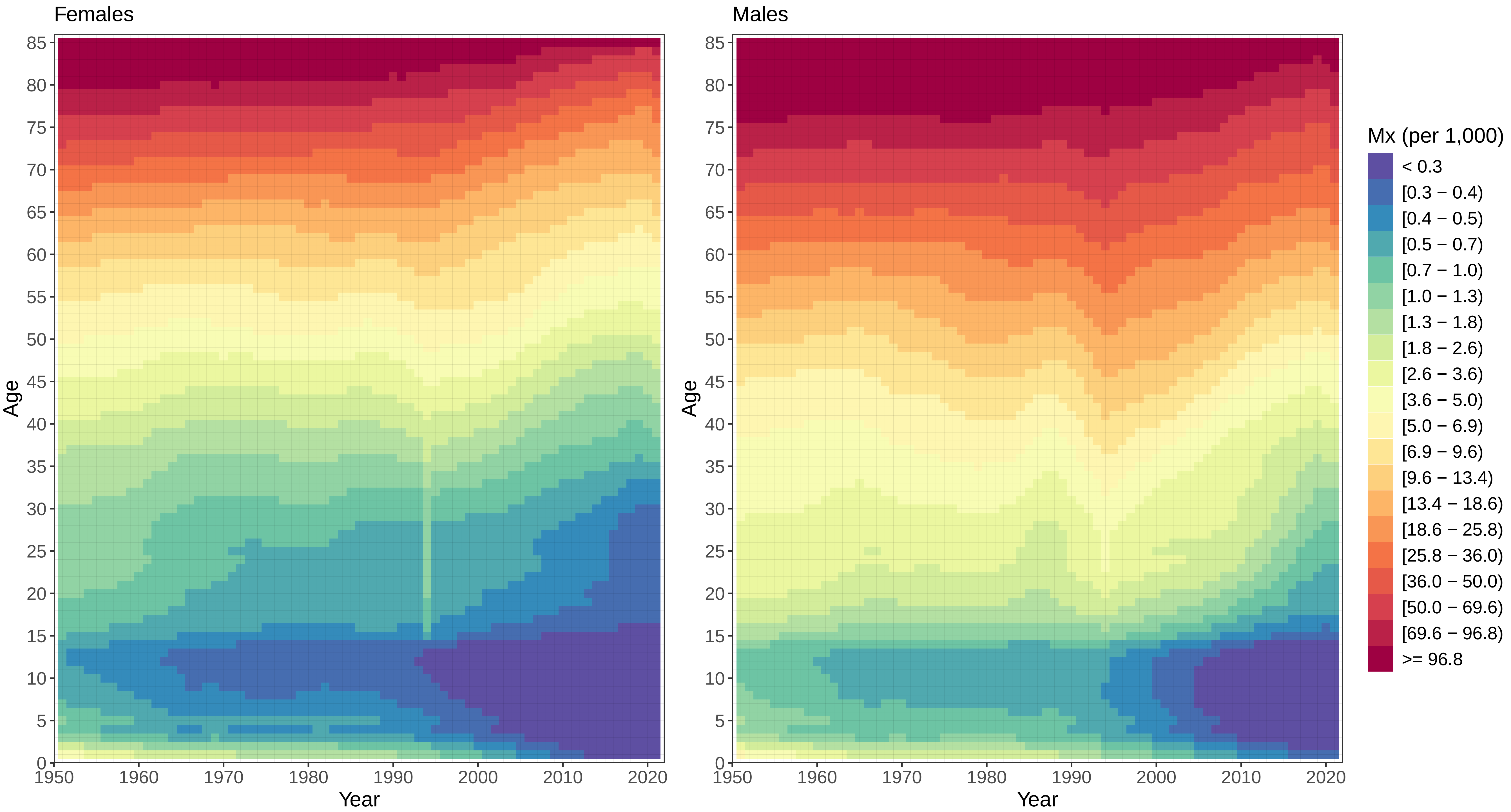

Appendix 2 Figure S4: Latvia mortality rates, 1950−2023

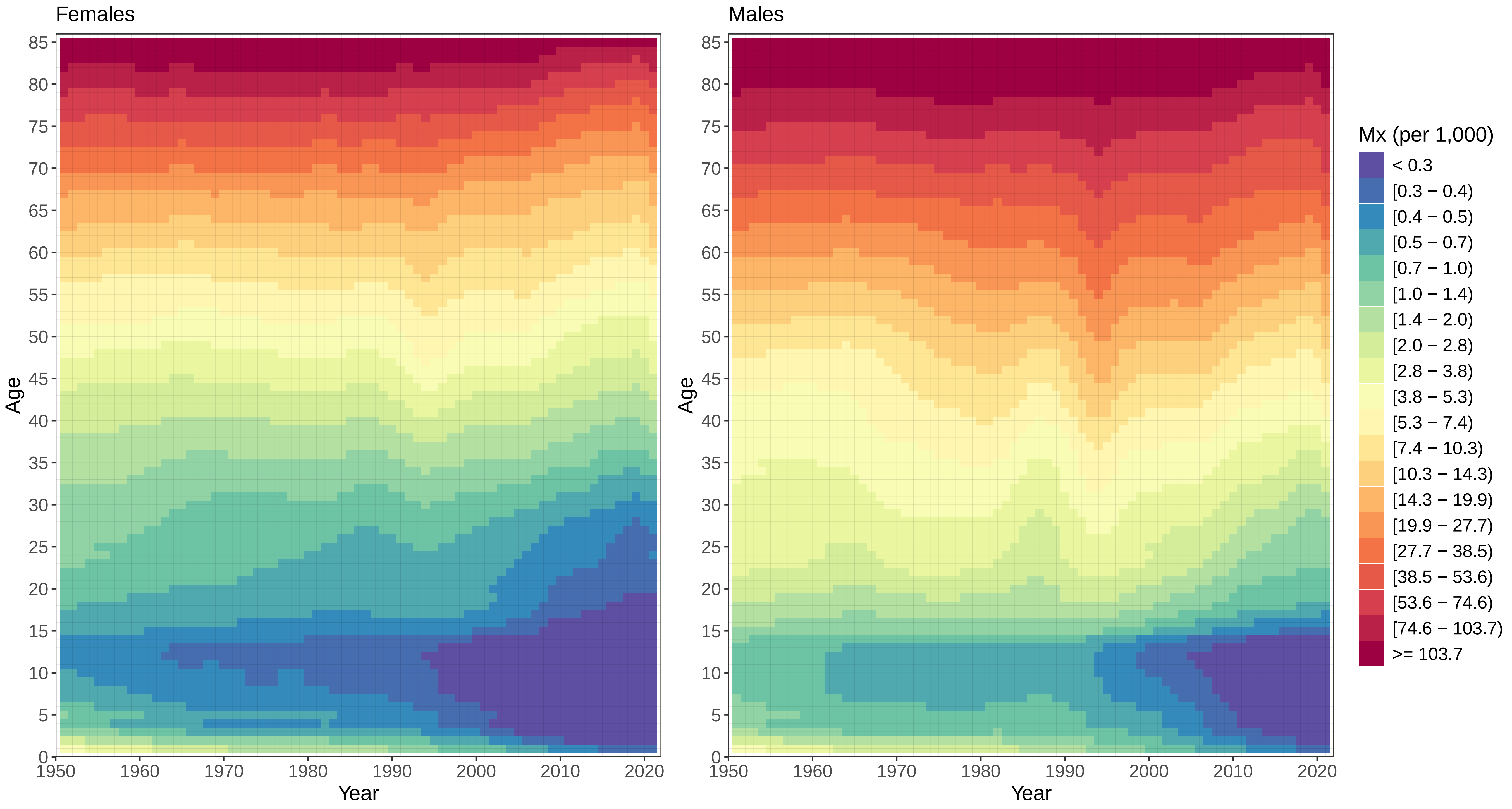

Appendix 2 Figure S4: Lithuania mortality rates, 1950−2023

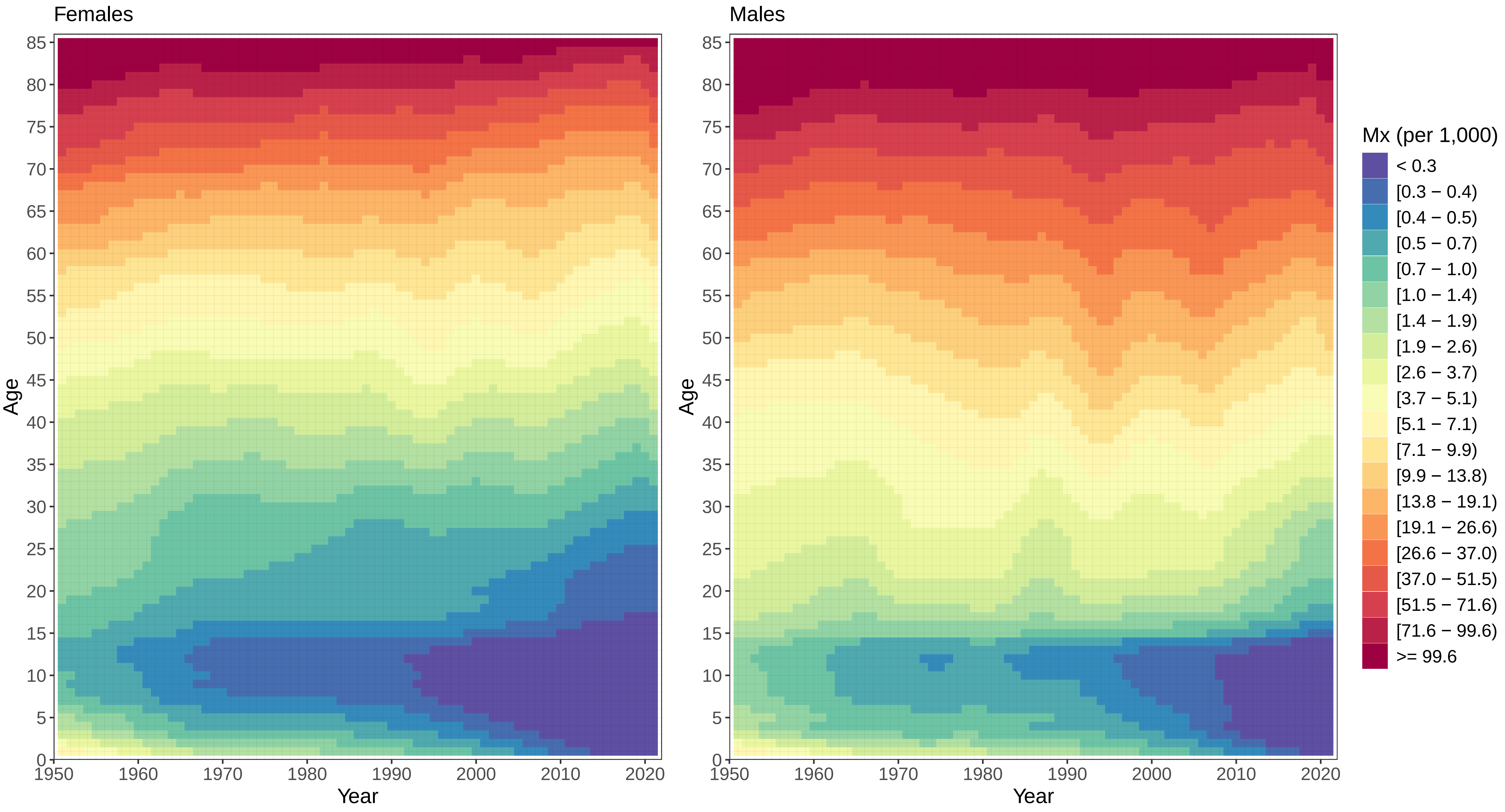

Appendix 2 Figure S4: Moldova mortality rates, 1950−2023

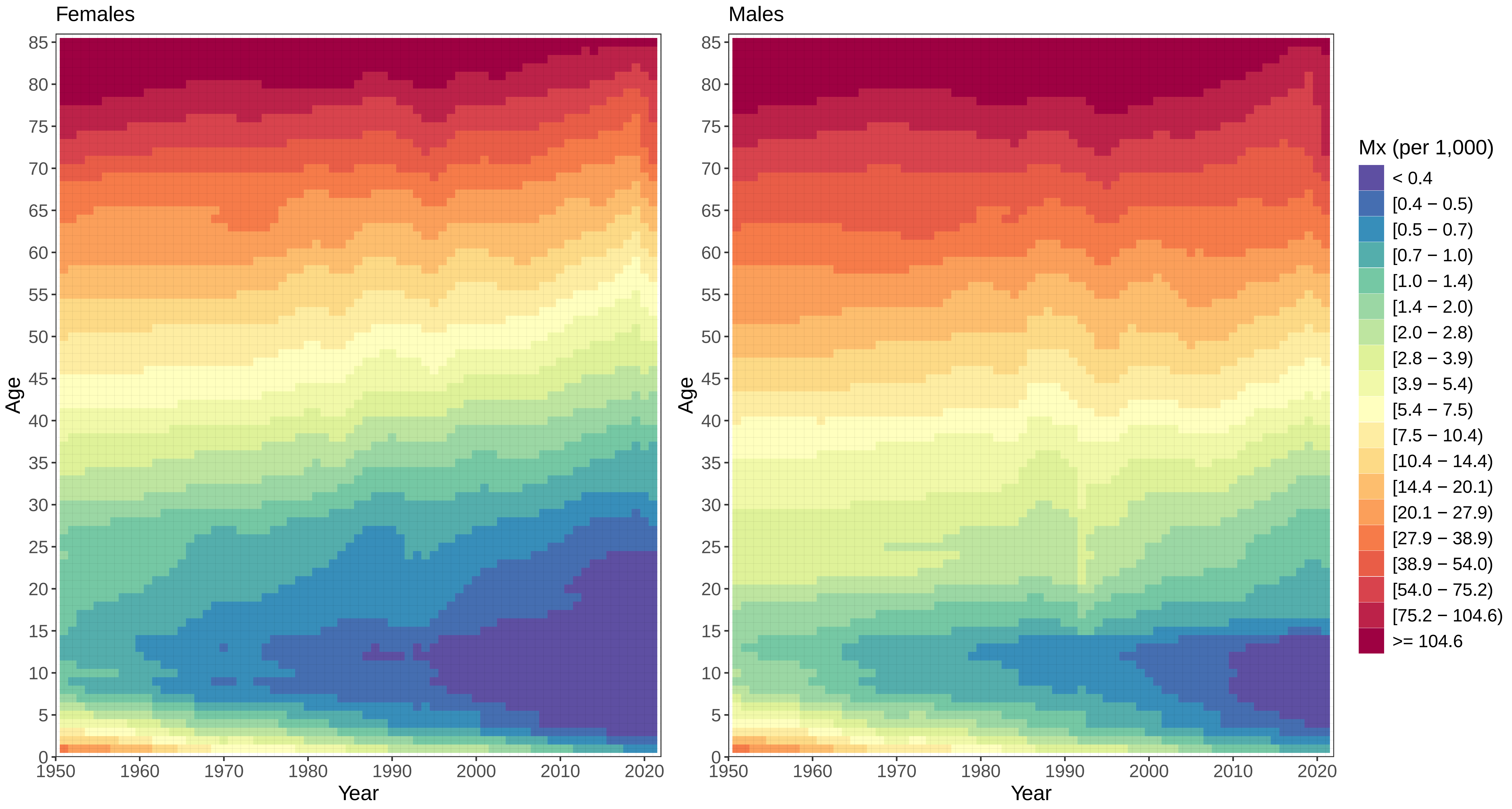

Appendix 2 Figure S4: Russia mortality rates, 1950−2023

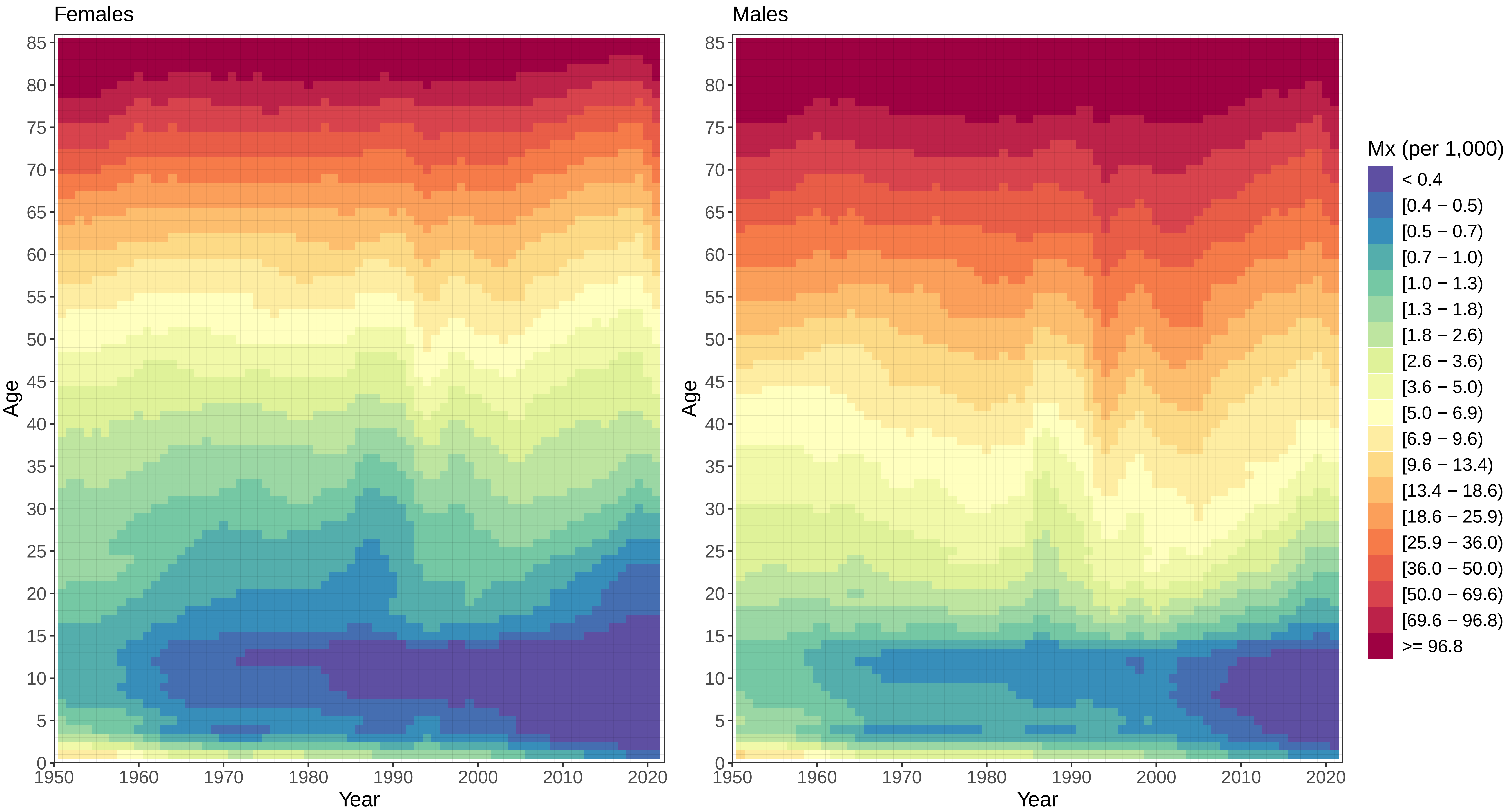

Appendix 2 Figure S4: Ukraine mortality rates, 1950−2023

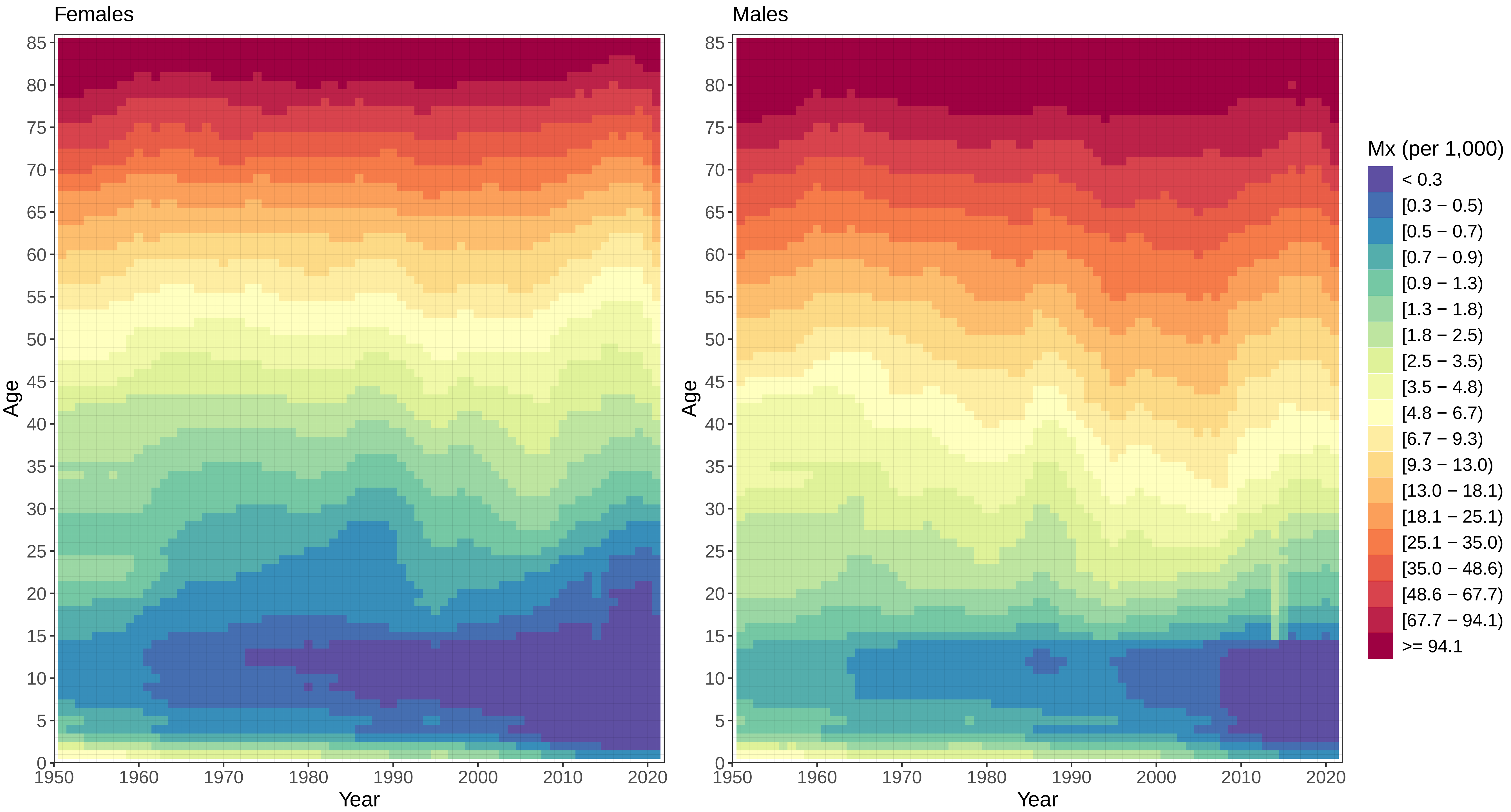

Appendix 2 Figure S4: Australia mortality rates, 1950−2023

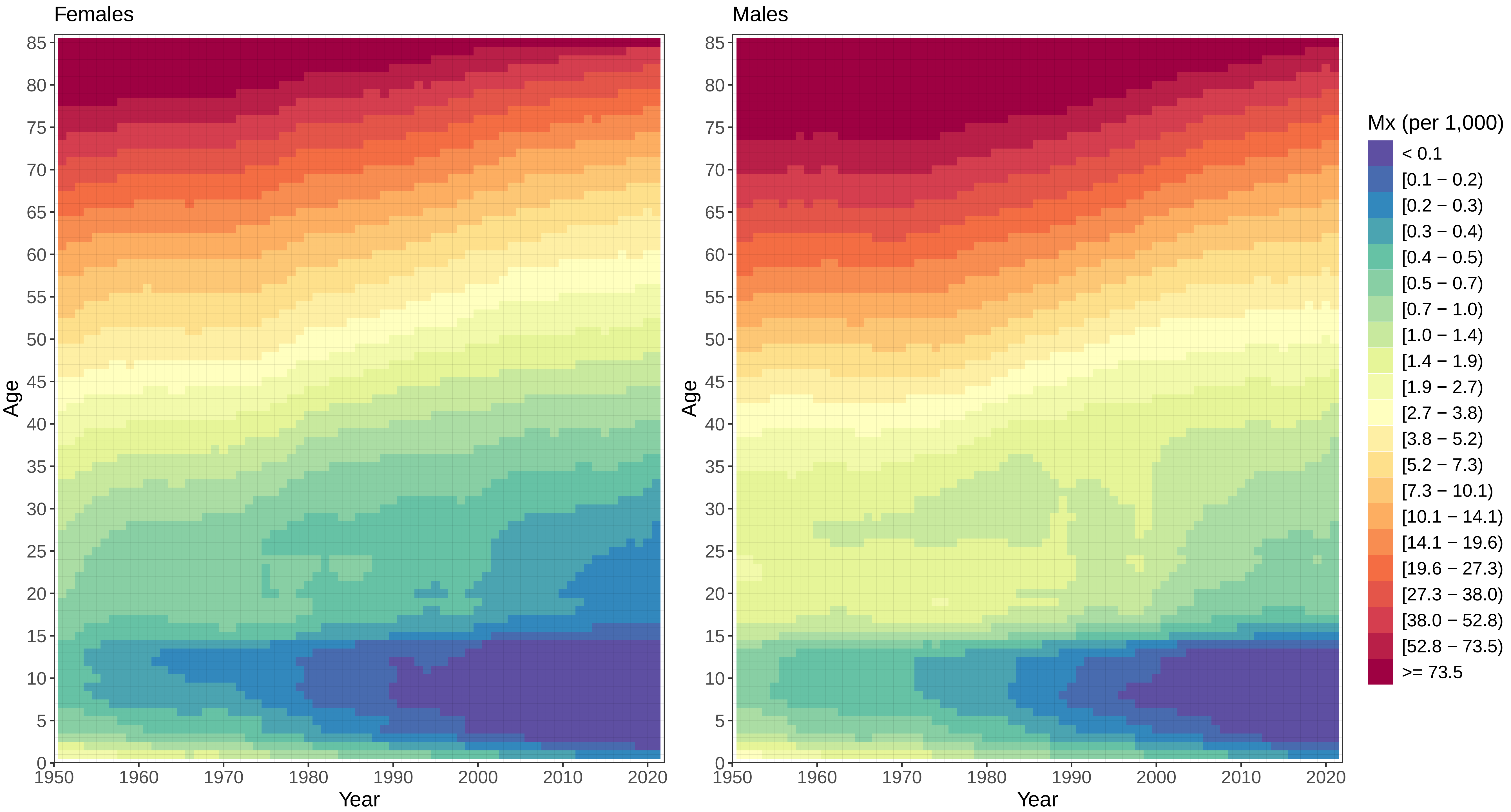

Appendix 2 Figure S4: New Zealand mortality rates, 1950−2023

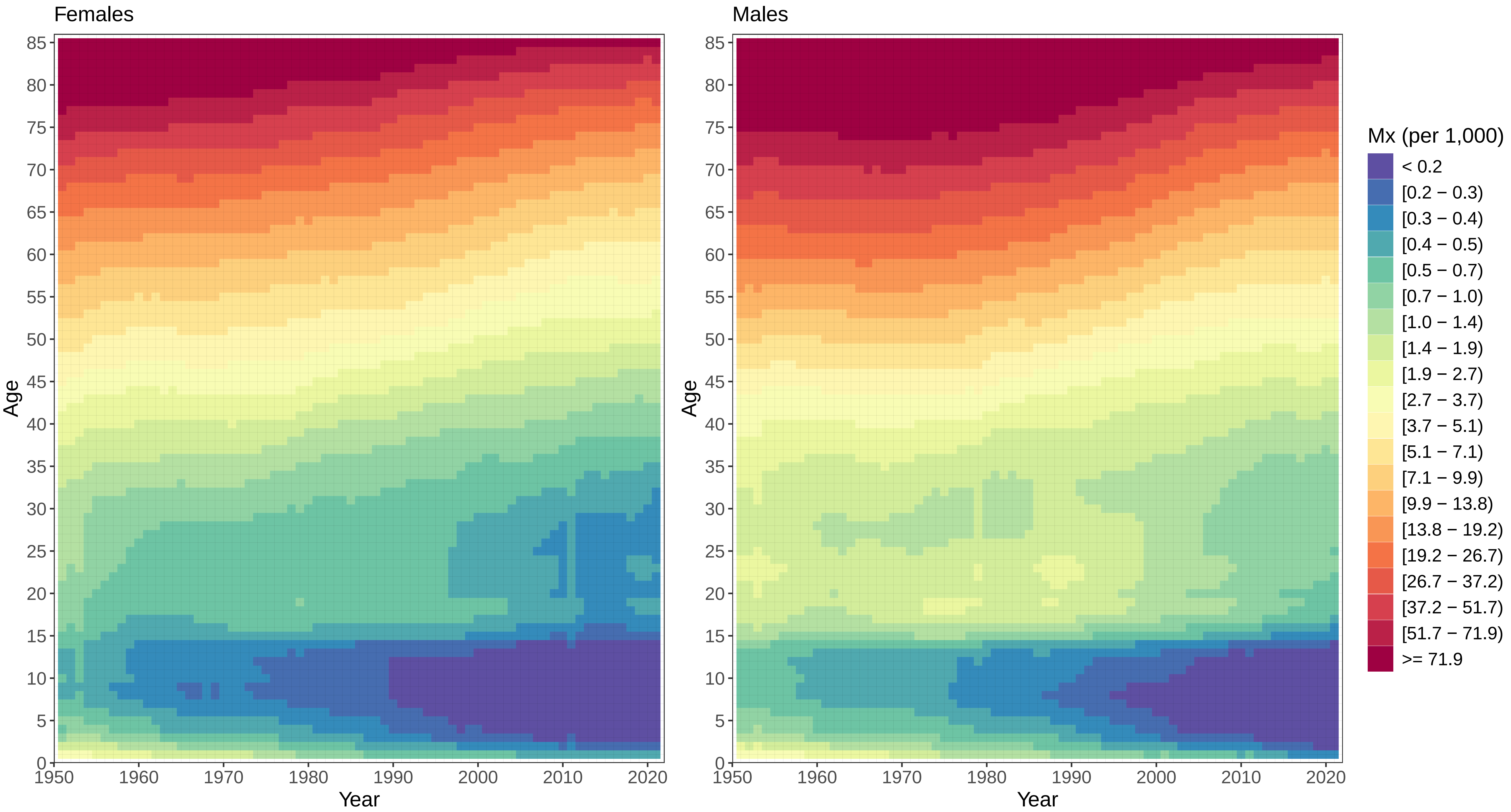

Appendix 2 Figure S4: Brunei mortality rates, 1950−2023

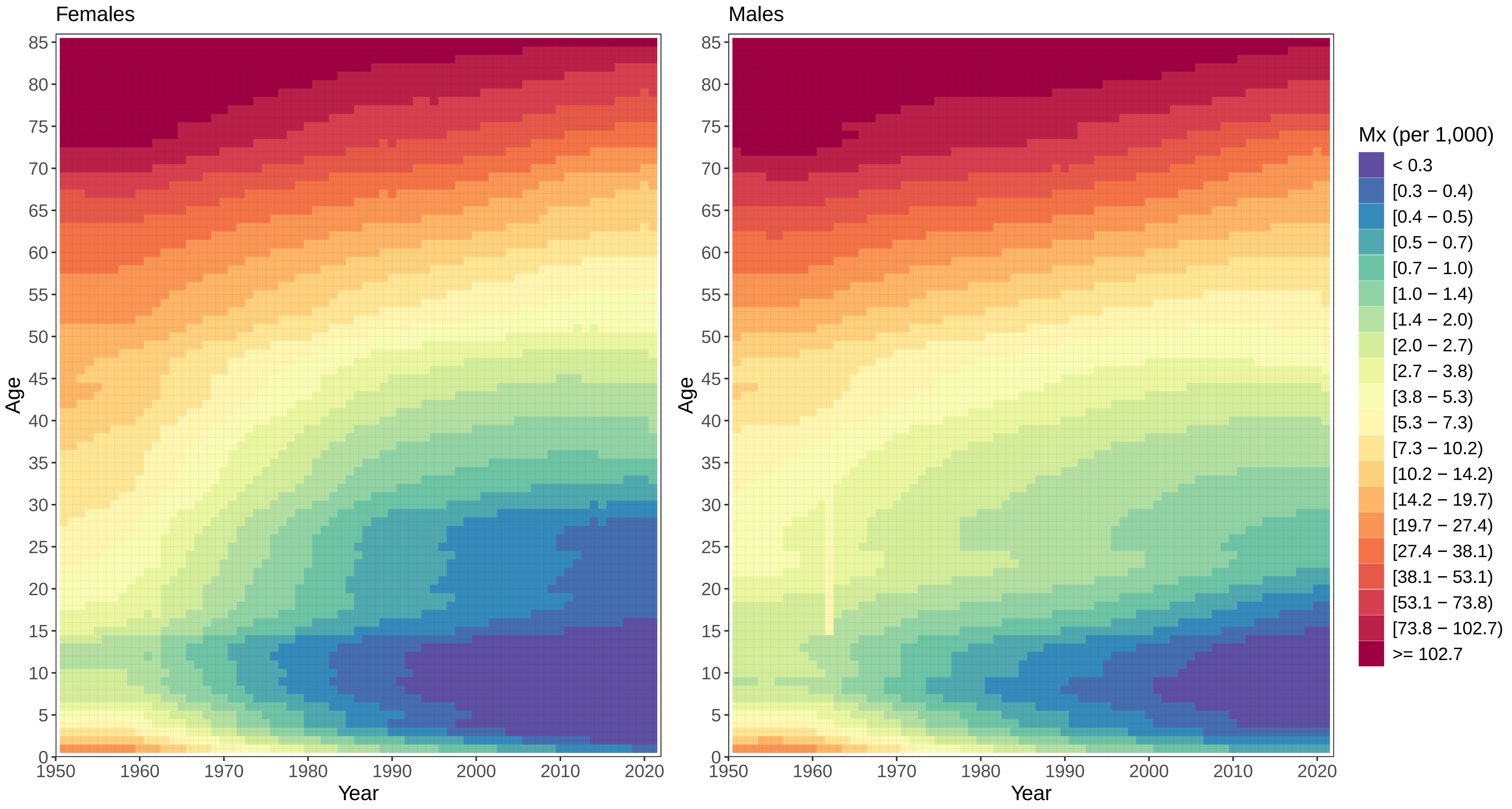

Appendix 2 Figure S4: Japan mortality rates, 1950−2023

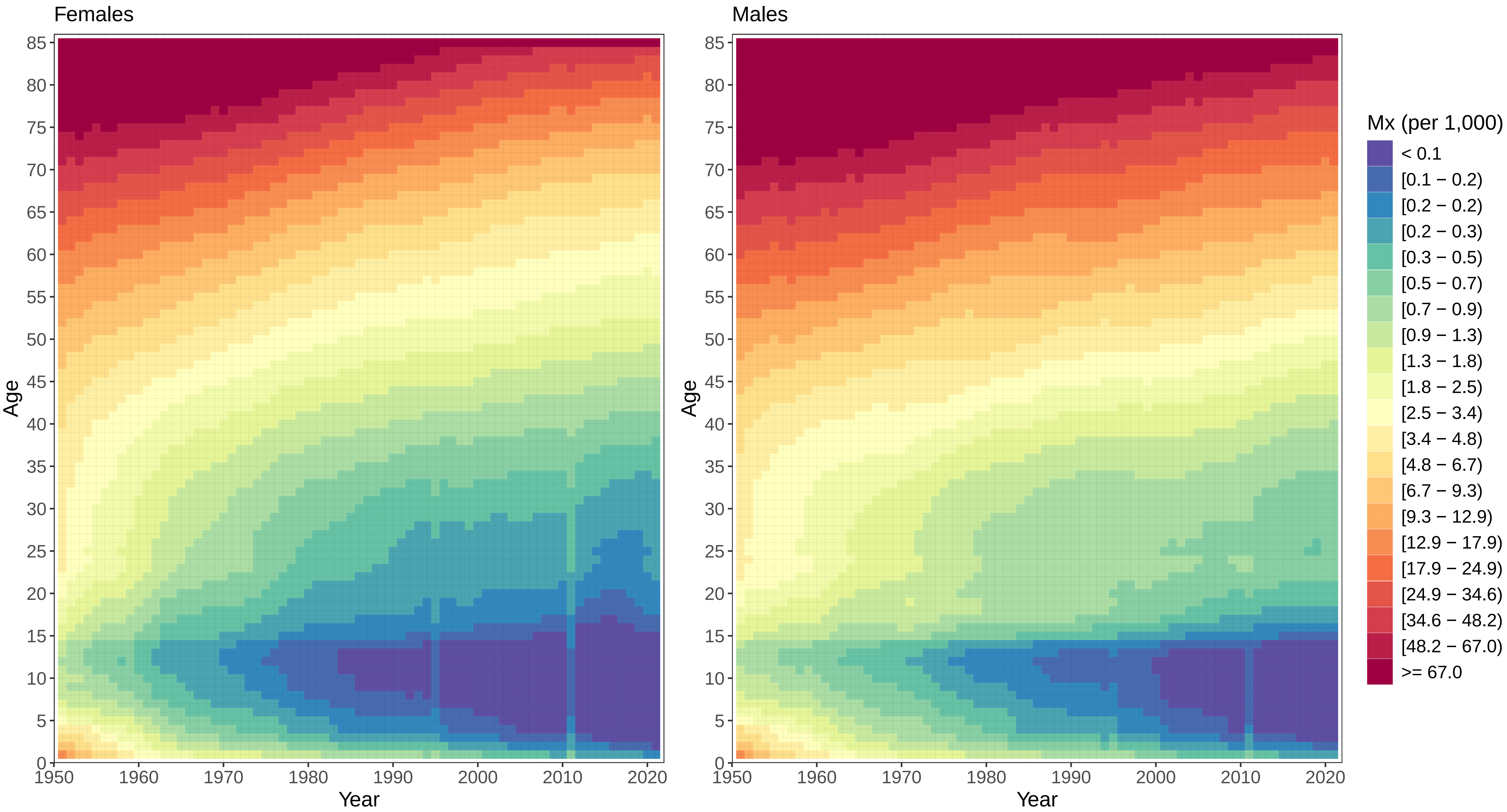

Appendix 2 Figure S4: Singapore mortality rates, 1950−2023

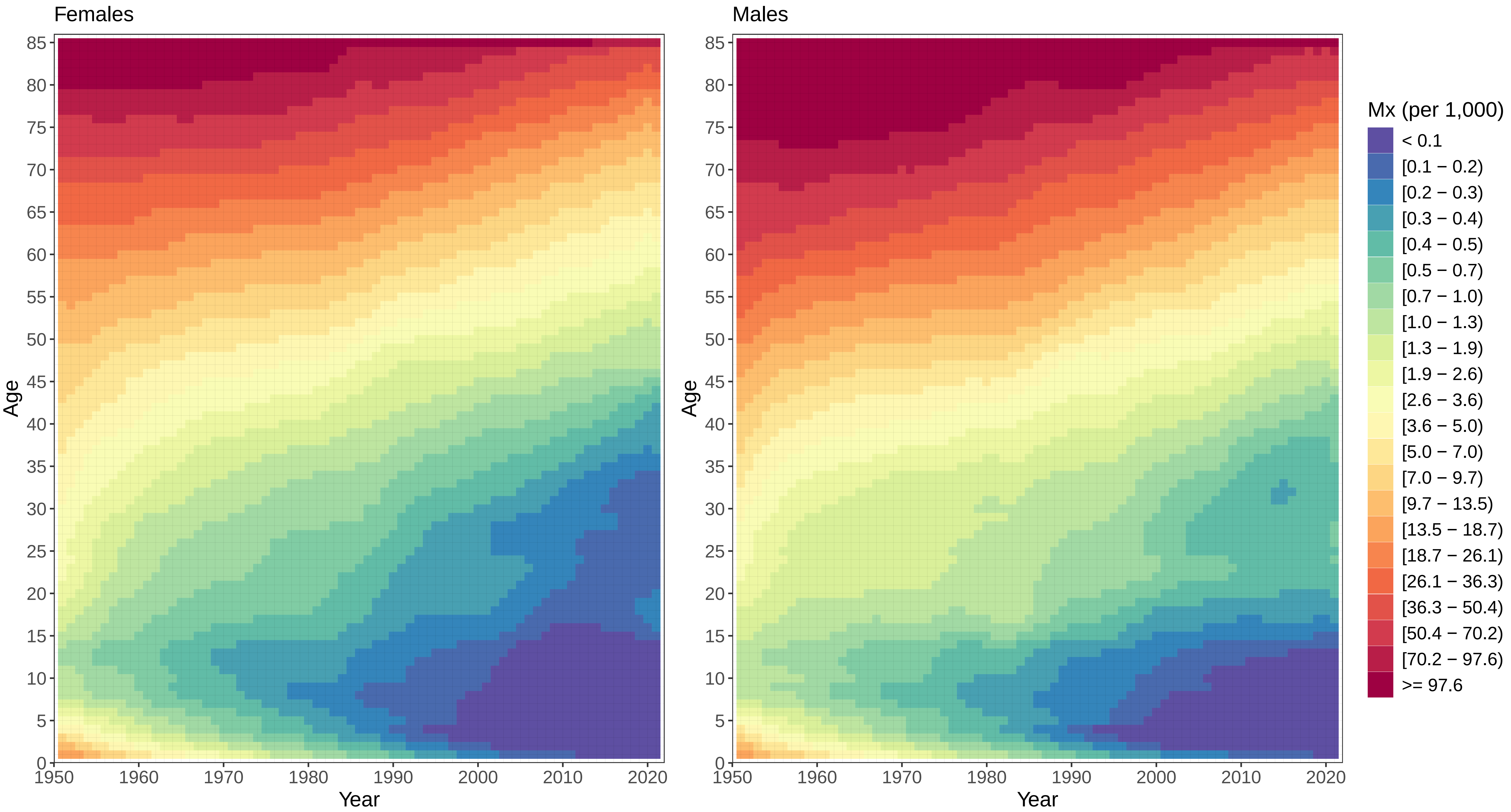

Appendix 2 Figure S4: South Korea mortality rates, 1950−2023

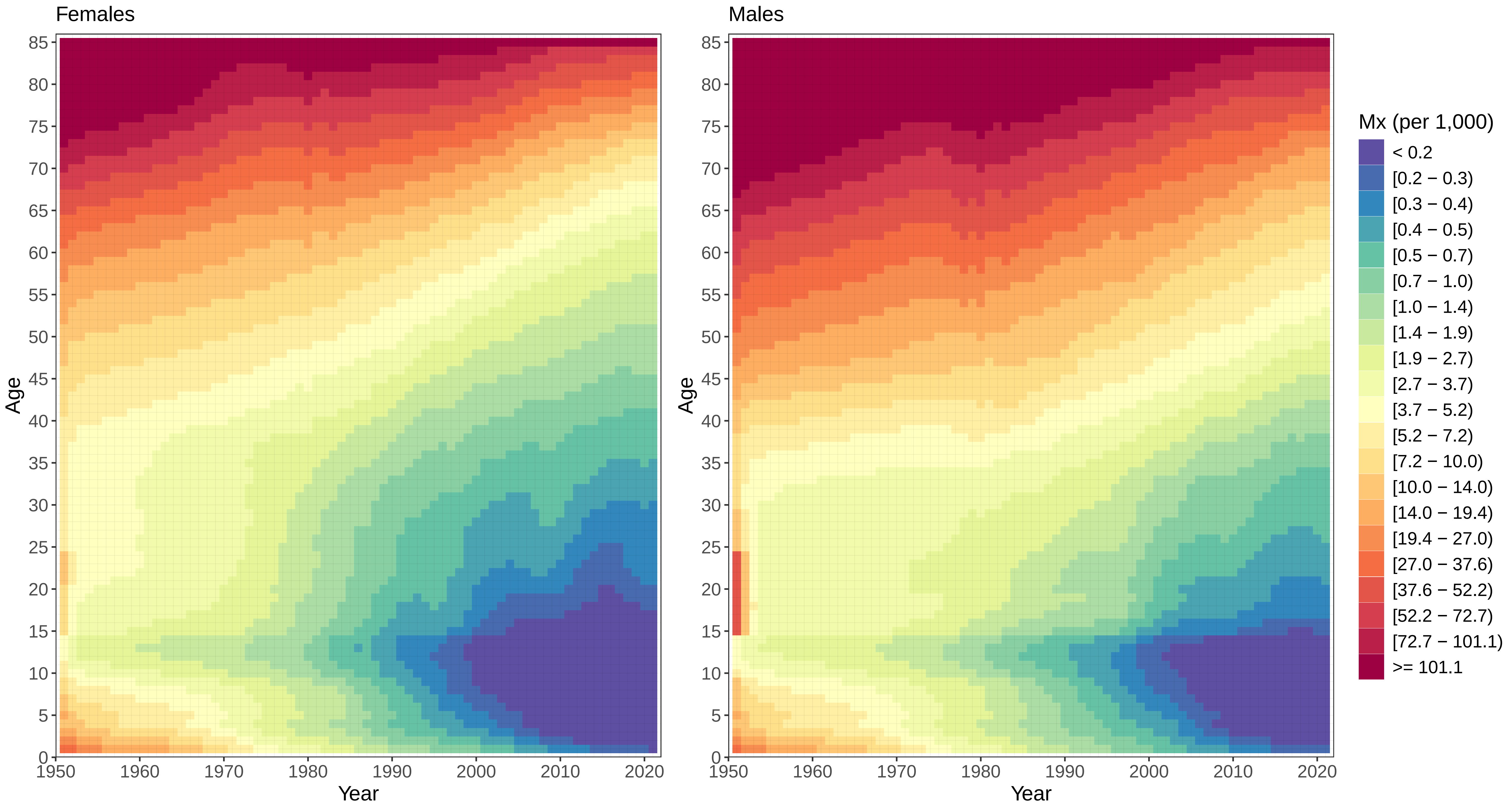

Appendix 2 Figure S4: Canada mortality rates, 1950−2023

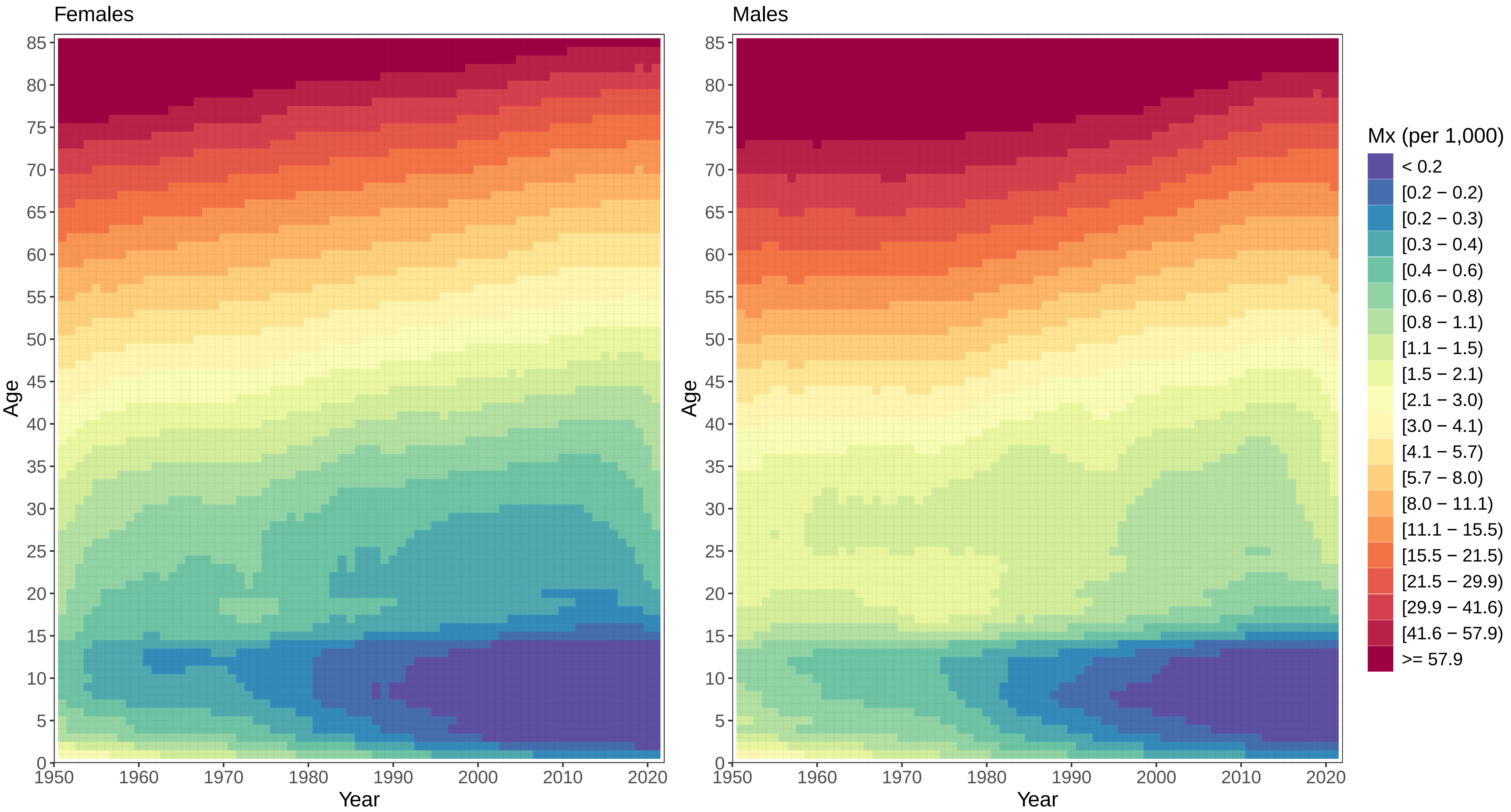

Appendix 2 Figure S4: Greenland mortality rates, 1950−2023

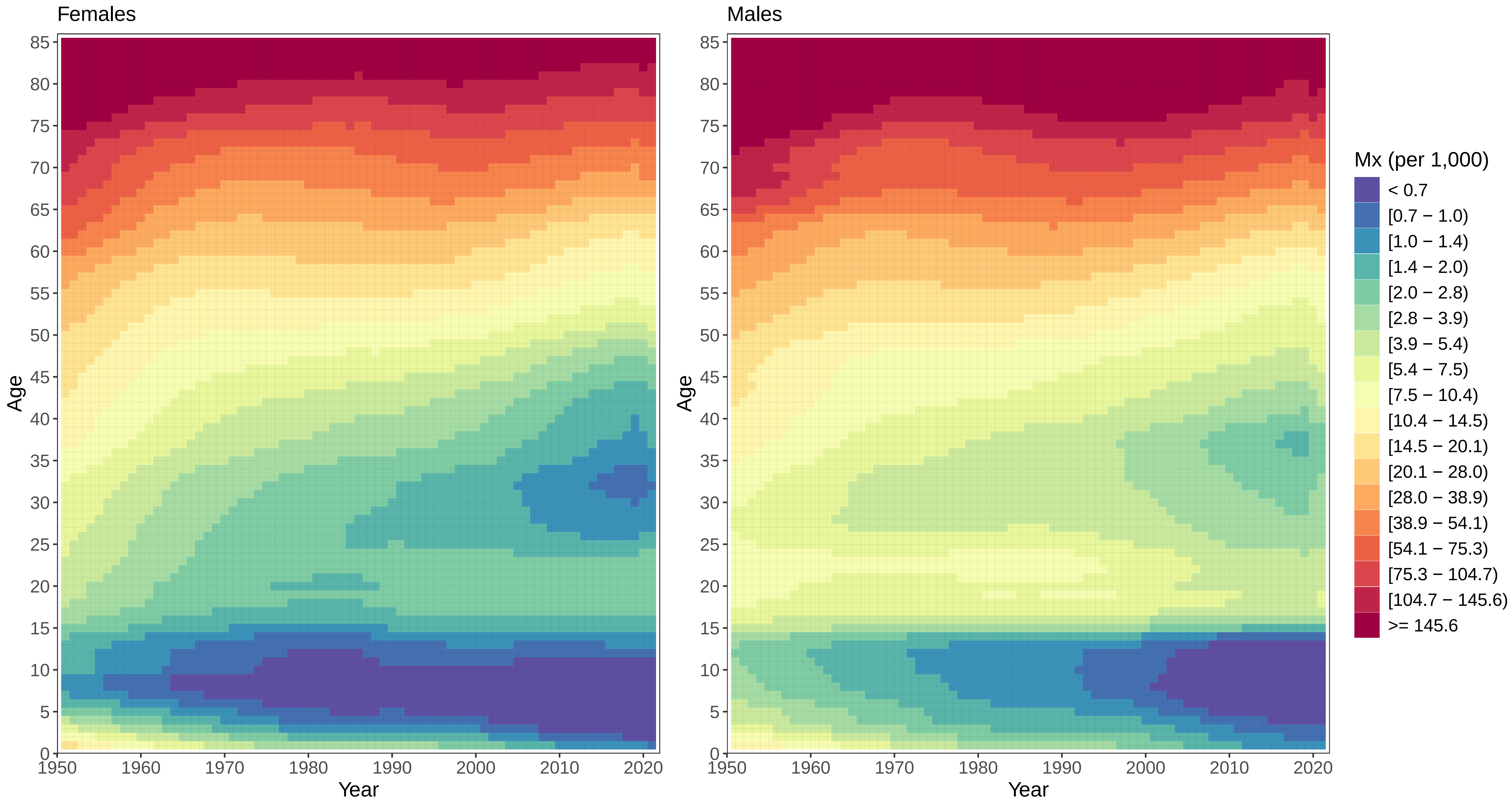

Appendix 2 Figure S4: USA mortality rates, 1950−2023

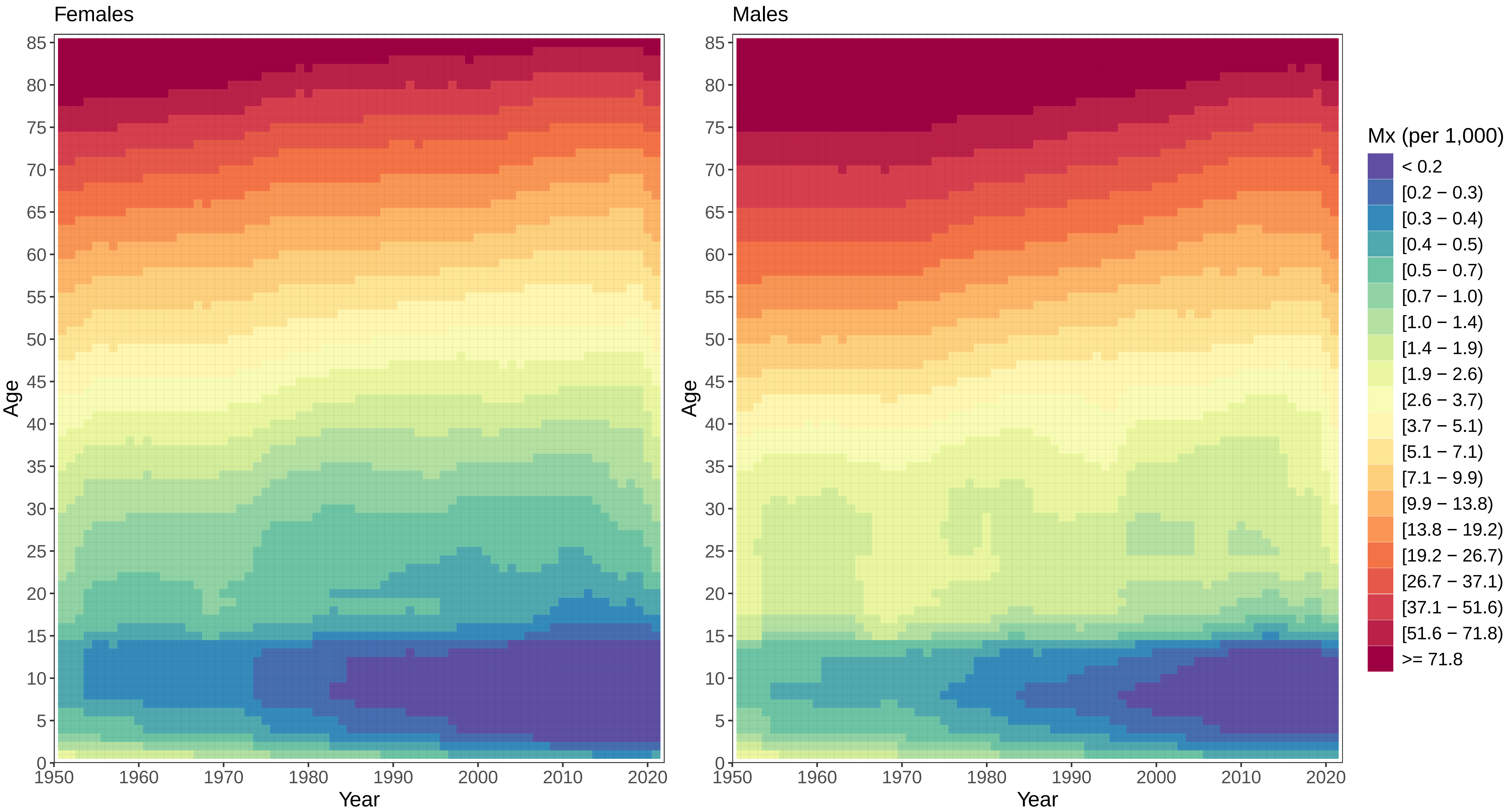

Appendix 2 Figure S4: Argentina mortality rates, 1950−2023

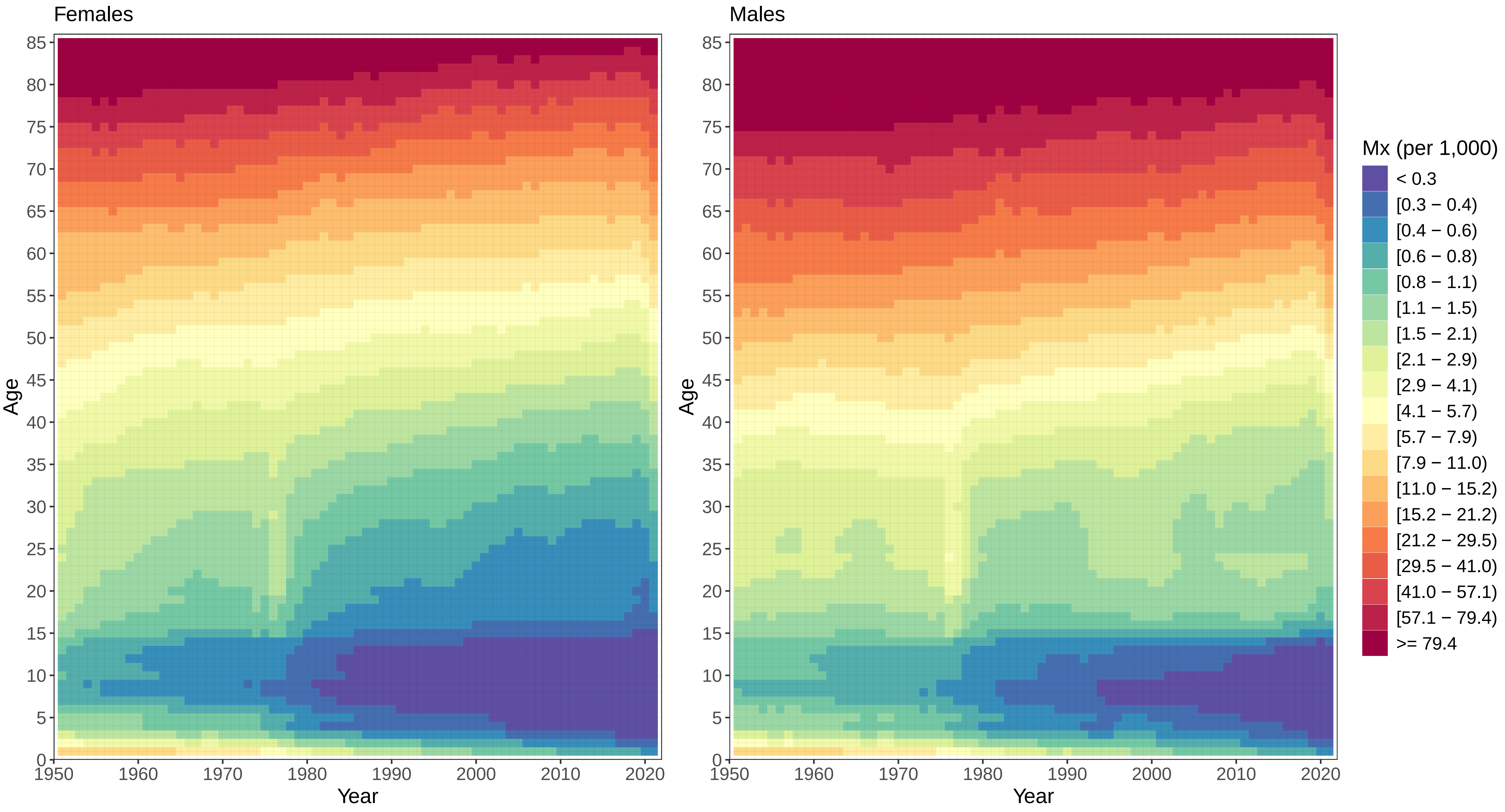

Appendix 2 Figure S4: Chile mortality rates, 1950−2023

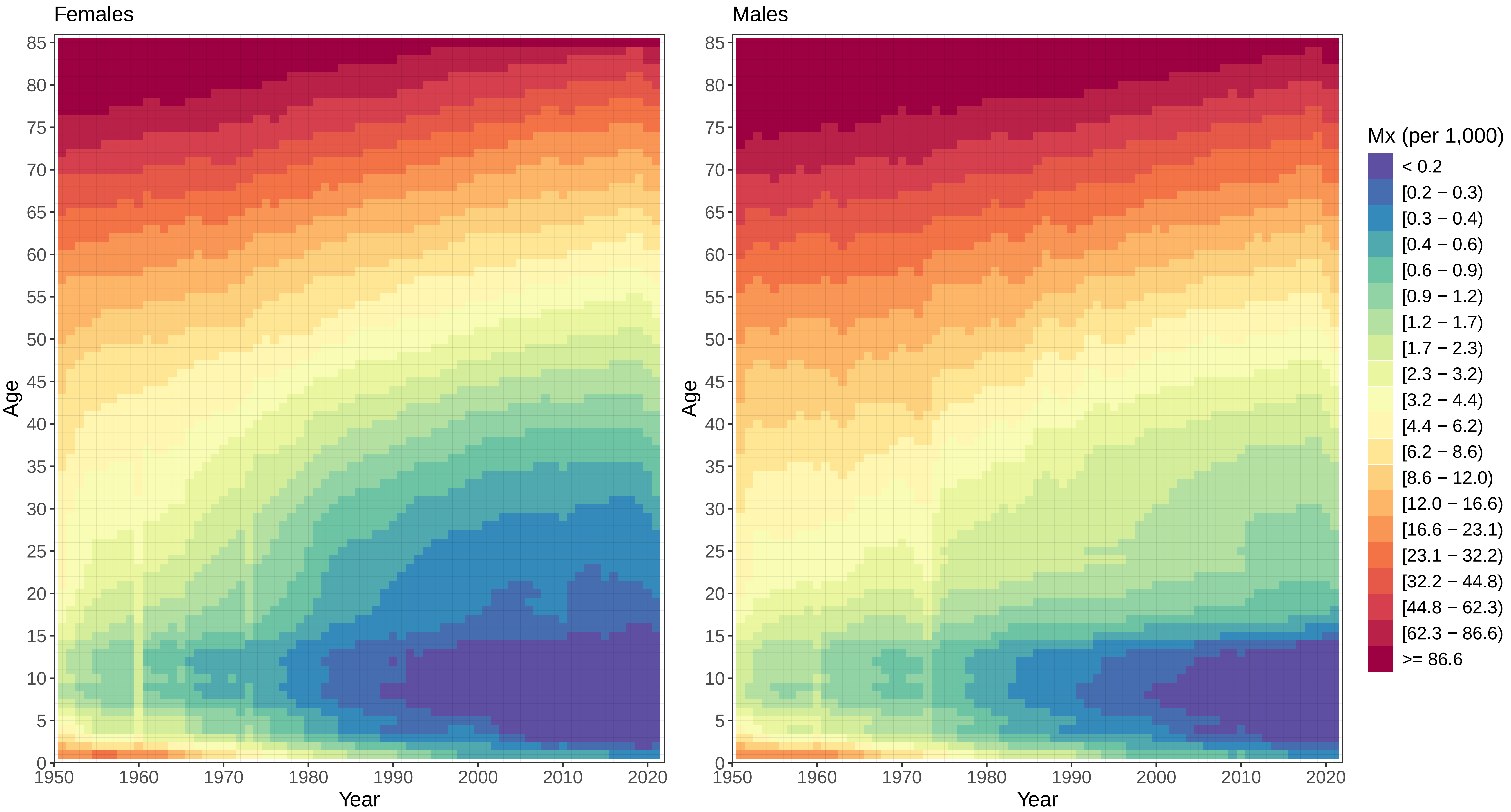

Appendix 2 Figure S4: Uruguay mortality rates, 1950−2023

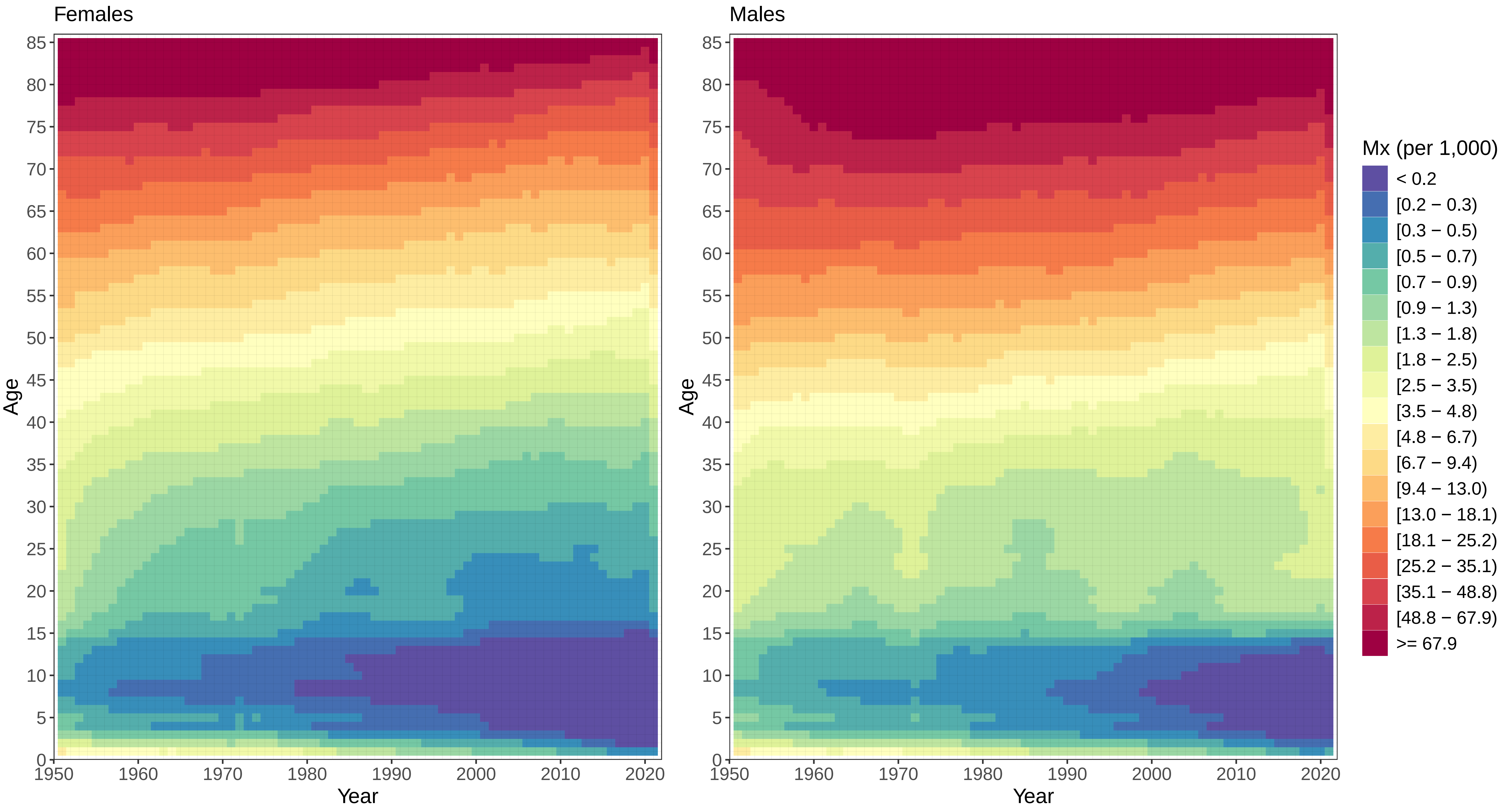

Appendix 2 Figure S4: Andorra mortality rates, 1950−2023

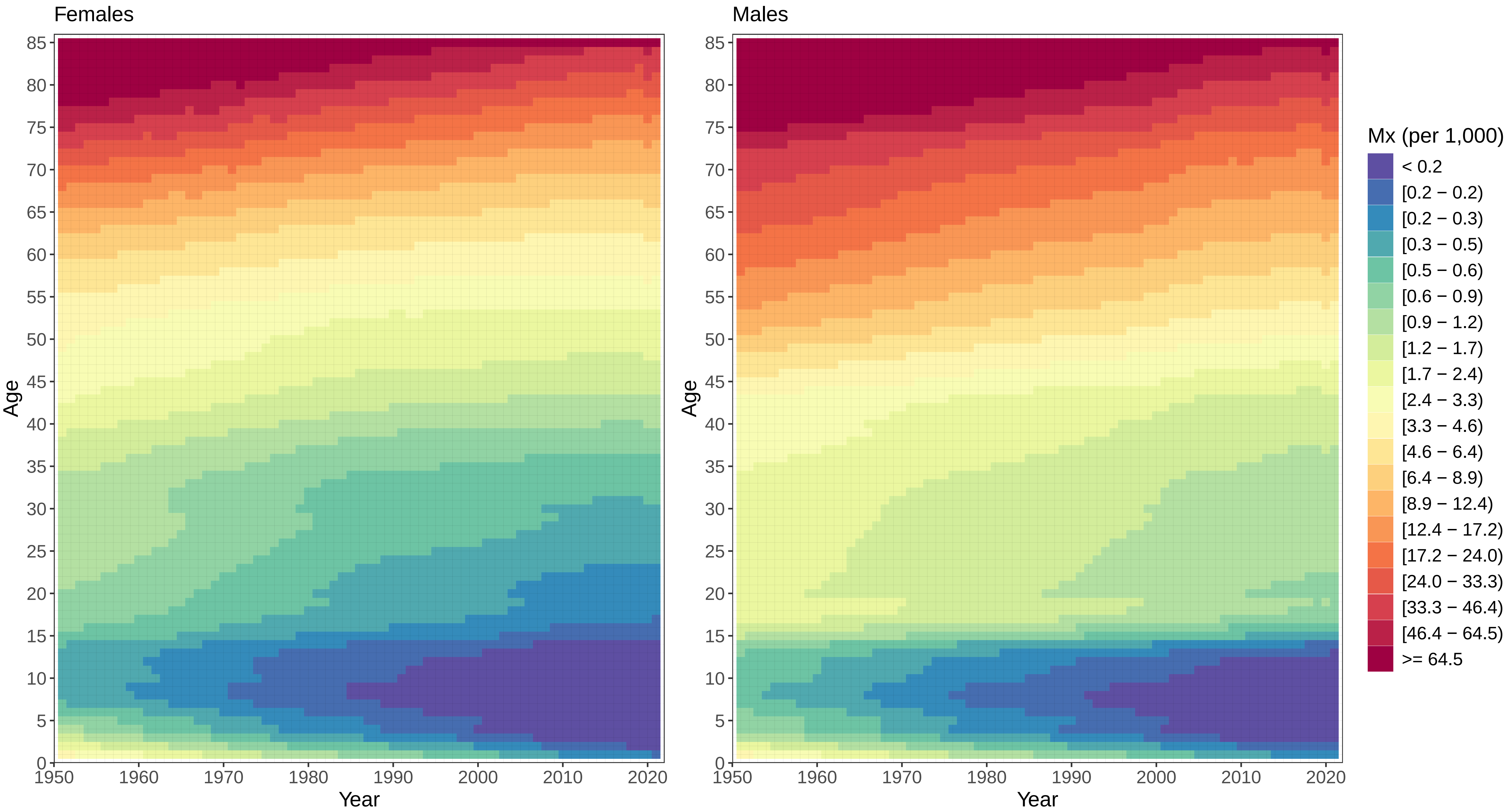

Appendix 2 Figure S4: Austria mortality rates, 1950−2023

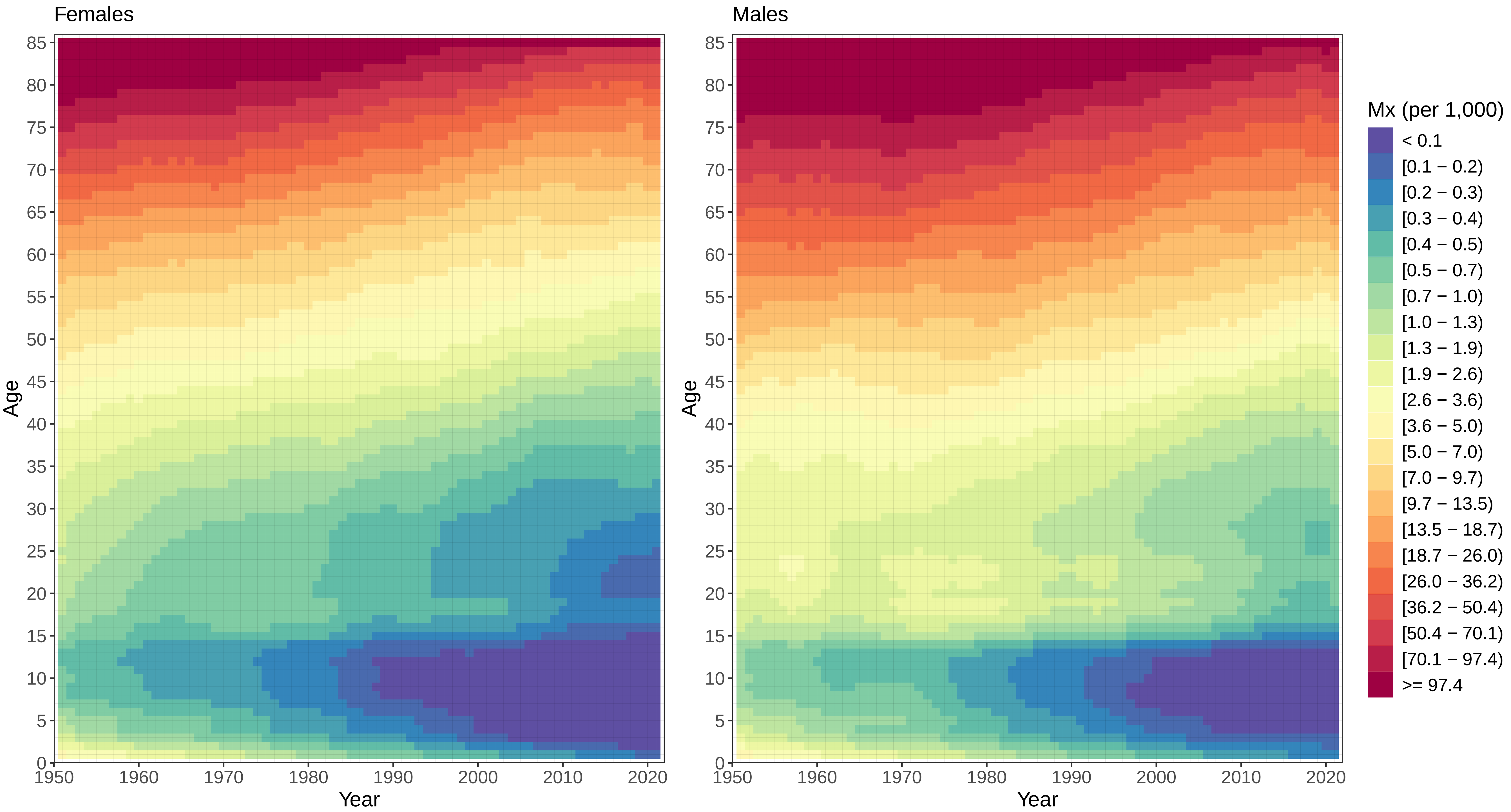

Appendix 2 Figure S4: Belgium mortality rates, 1950−2023

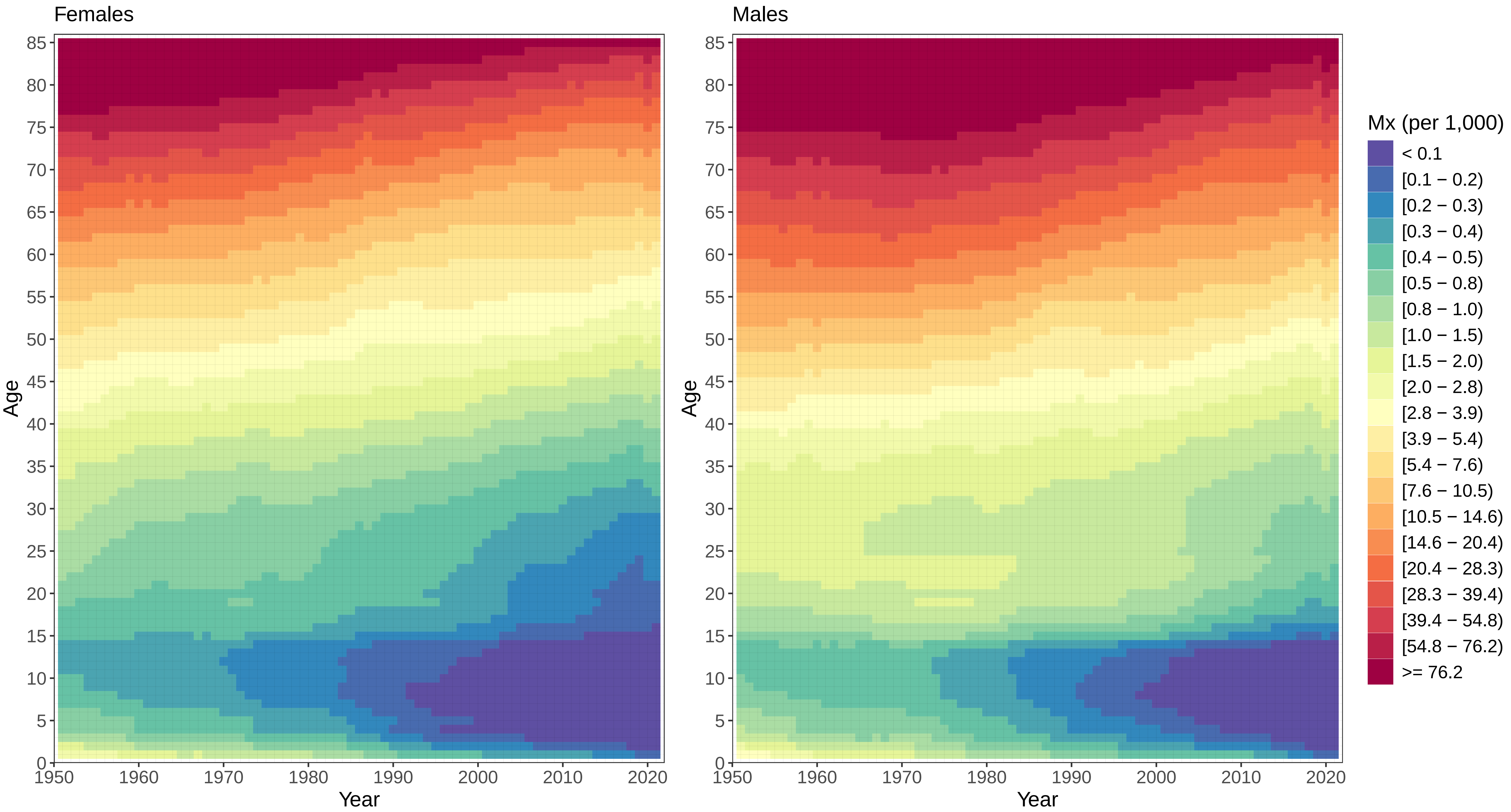

Appendix 2 Figure S4: Cyprus mortality rates, 1950−2023

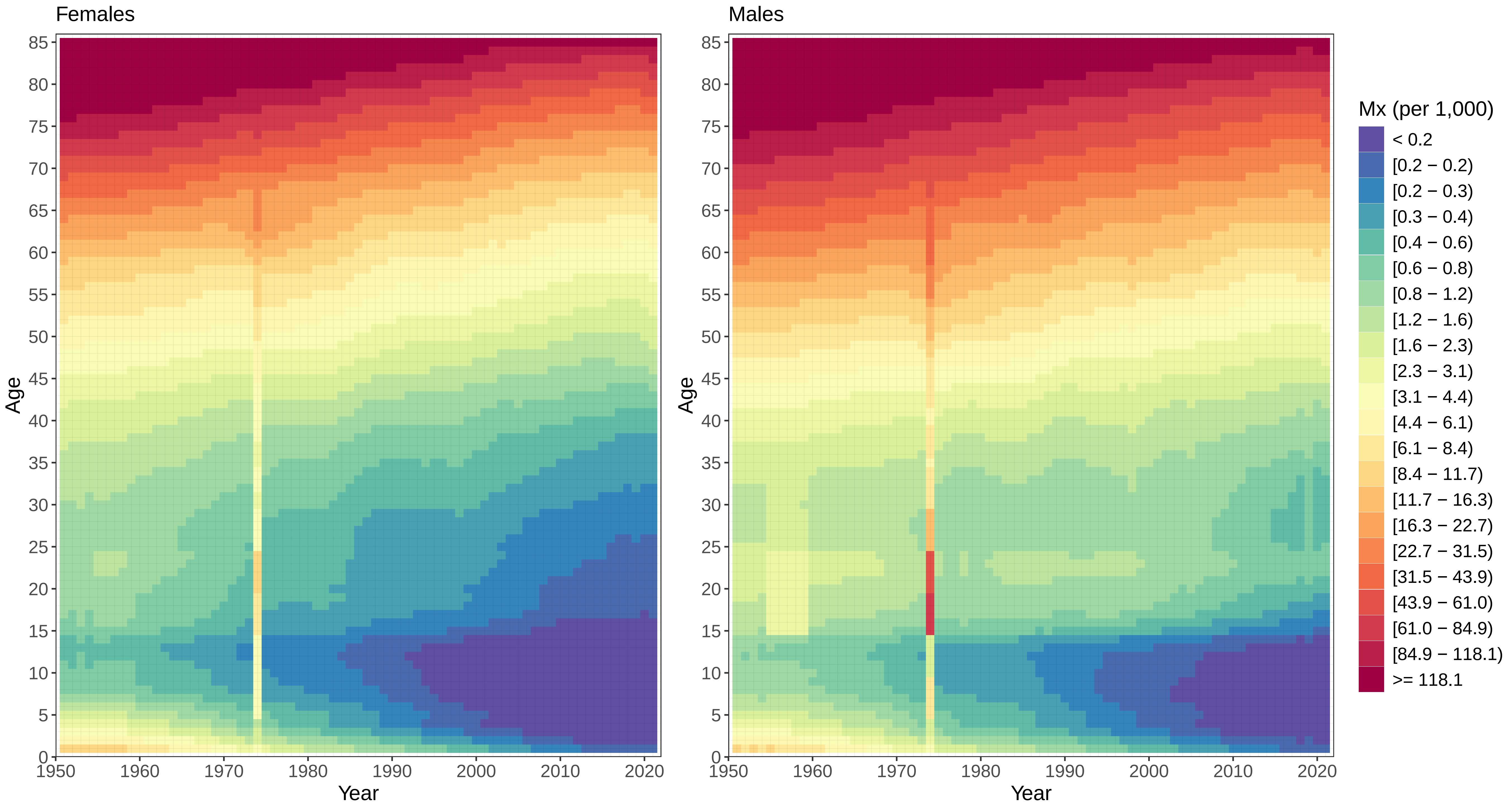

Appendix 2 Figure S4: Denmark mortality rates, 1950−2023

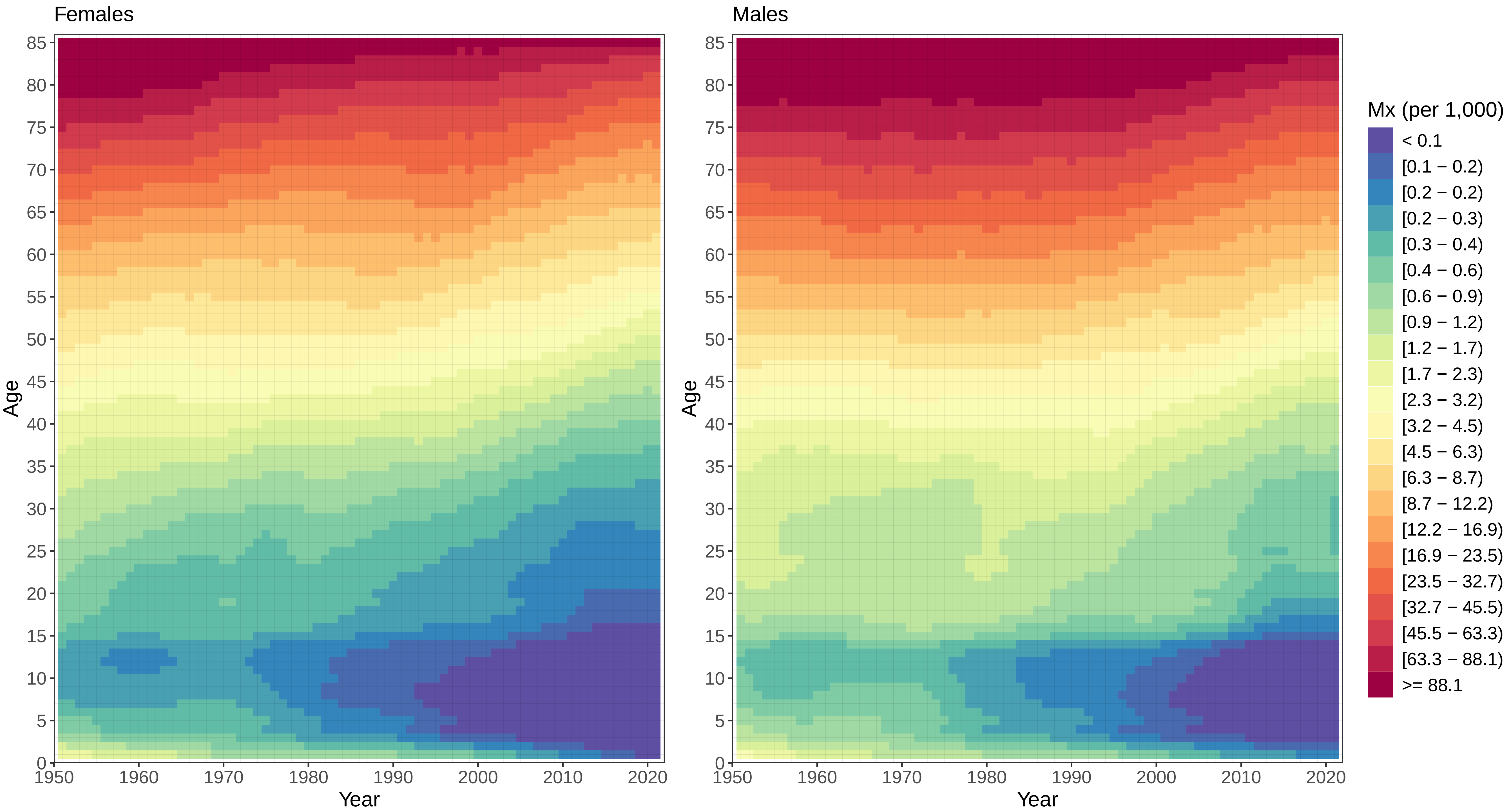

Appendix 2 Figure S4: Finland mortality rates, 1950−2023

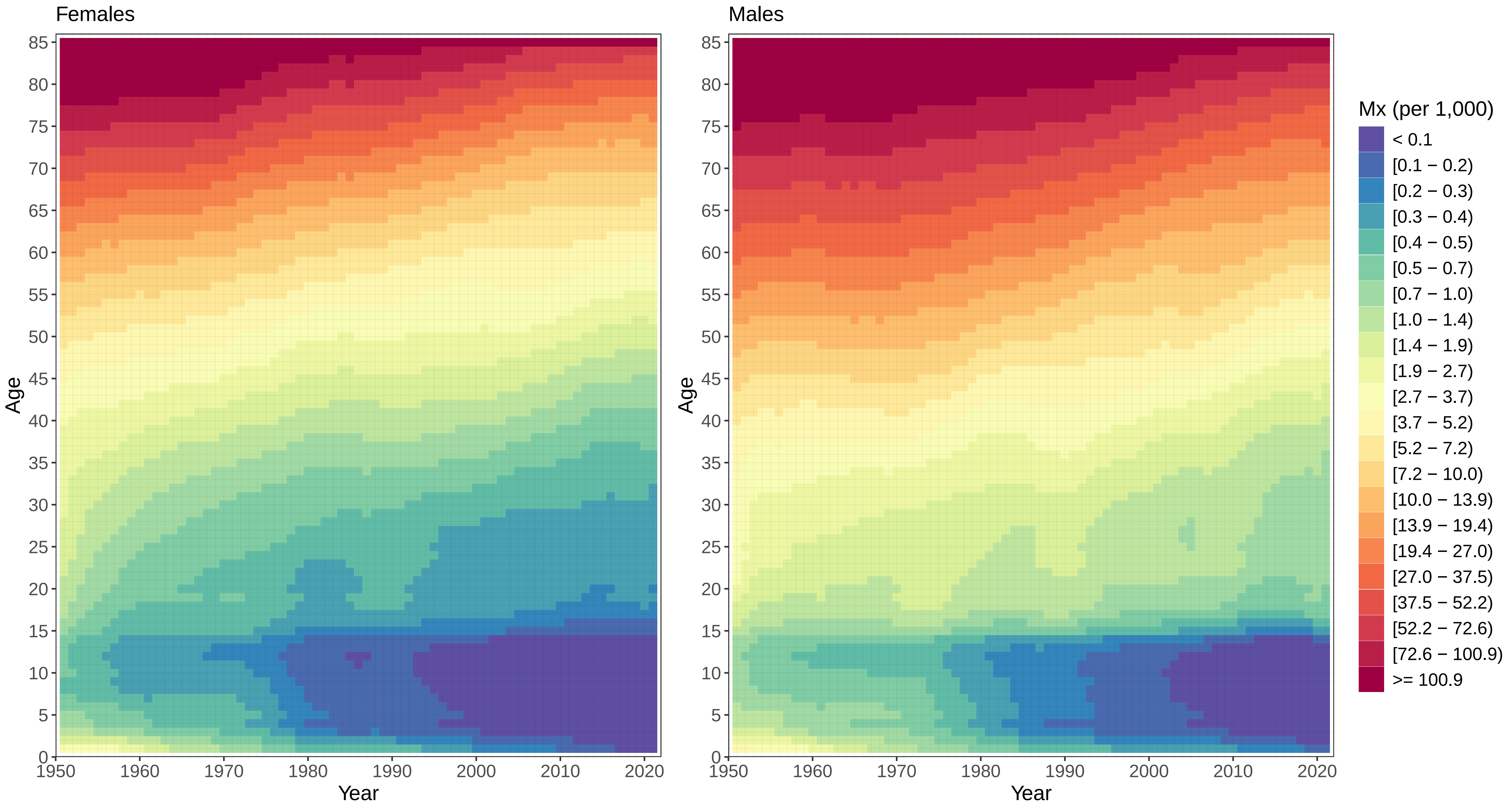

Appendix 2 Figure S4: France mortality rates, 1950−2023

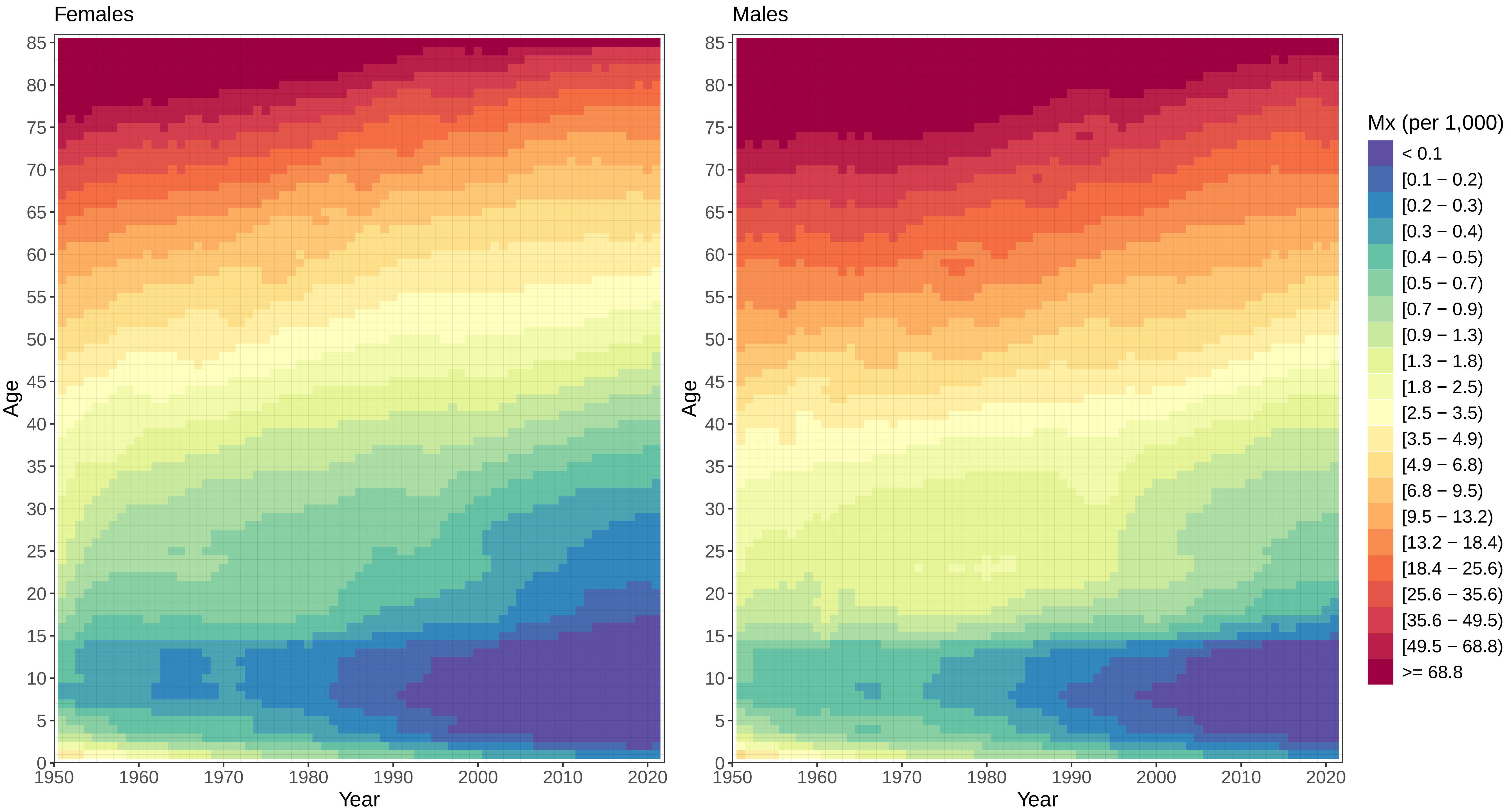

Appendix 2 Figure S4: Germany mortality rates, 1950−2023

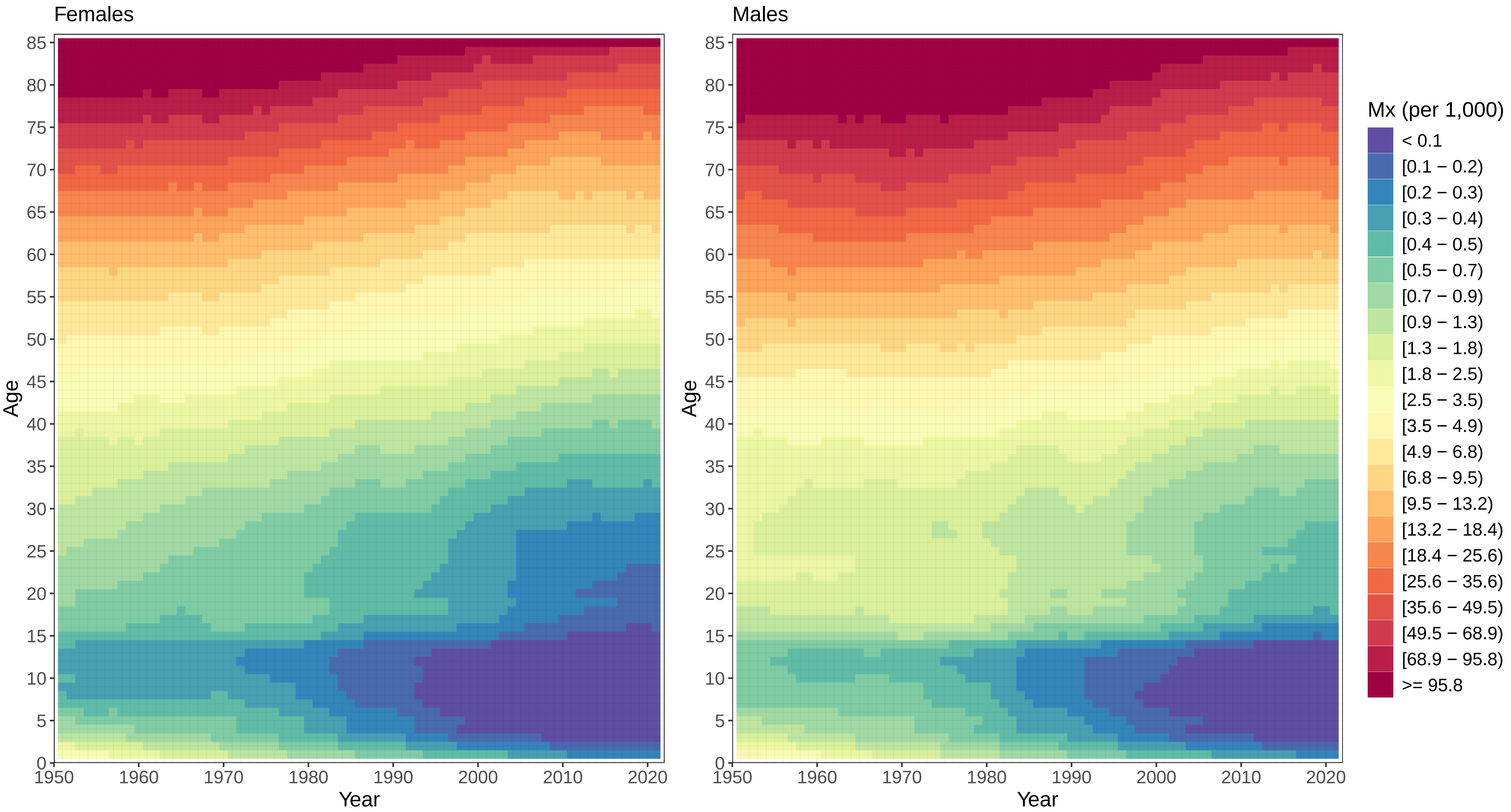

Appendix 2 Figure S4: Greece mortality rates, 1950−2023

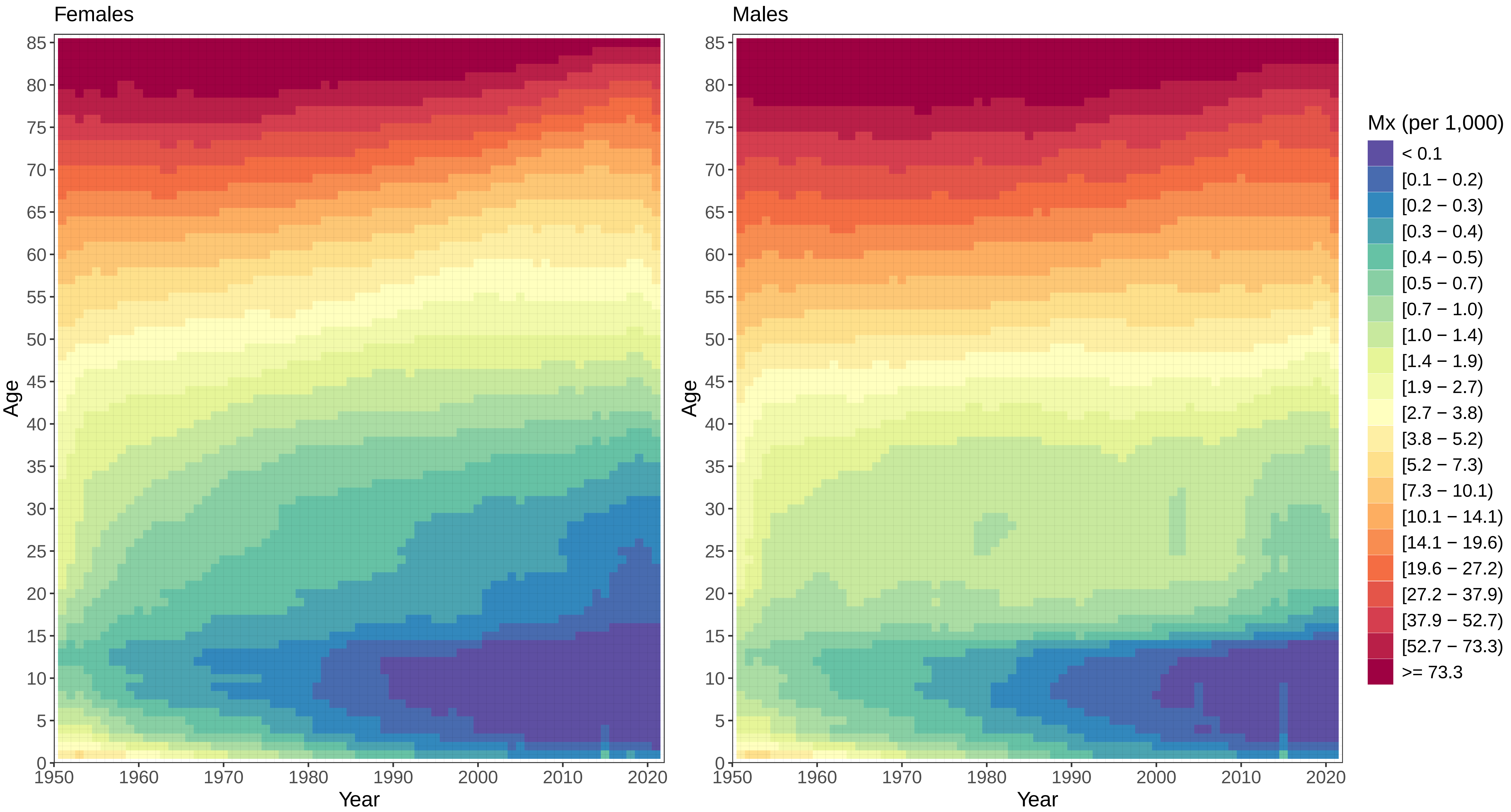

Appendix 2 Figure S4: Iceland mortality rates, 1950−2023

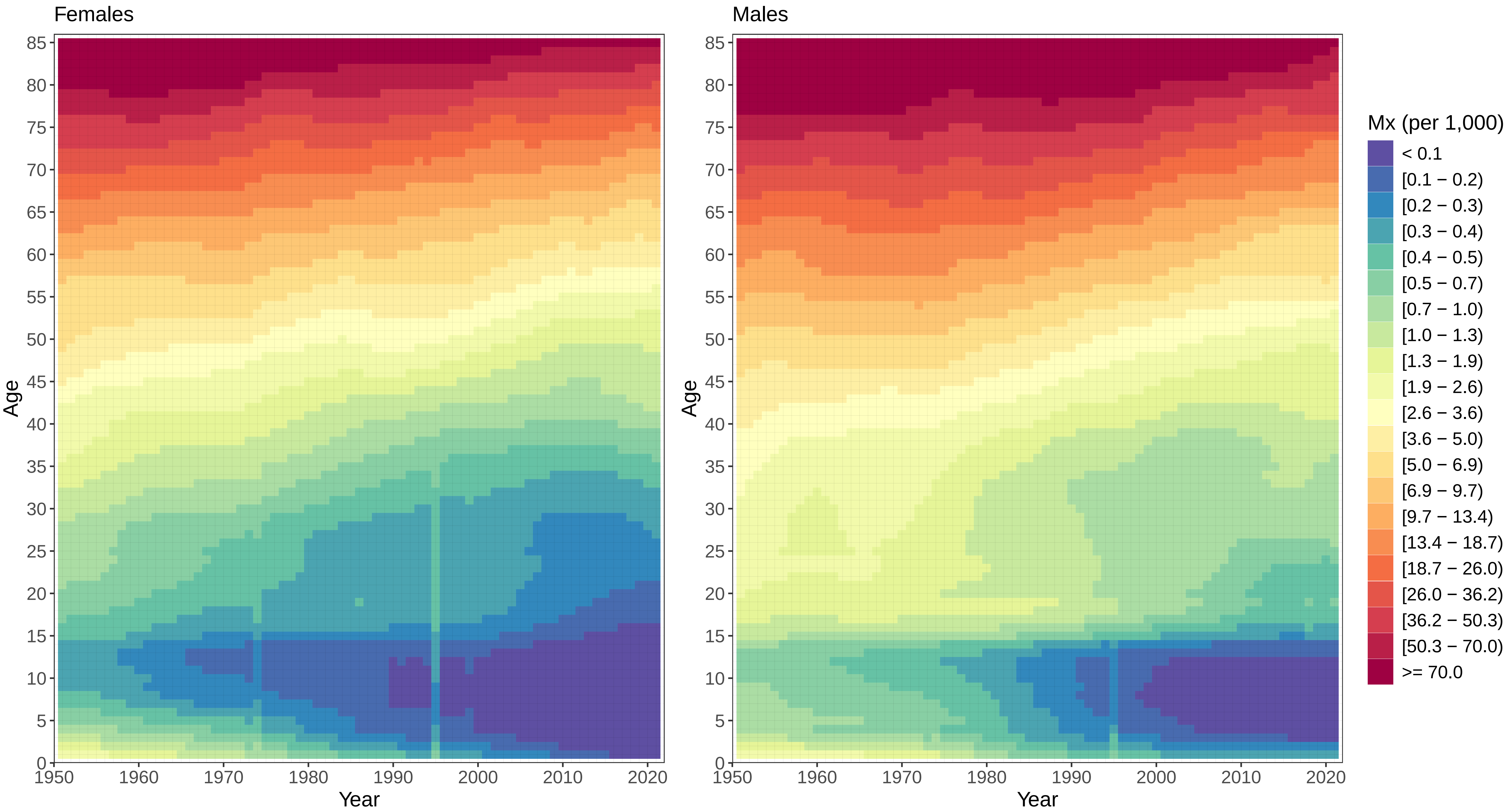

Appendix 2 Figure S4: Ireland mortality rates, 1950−2023

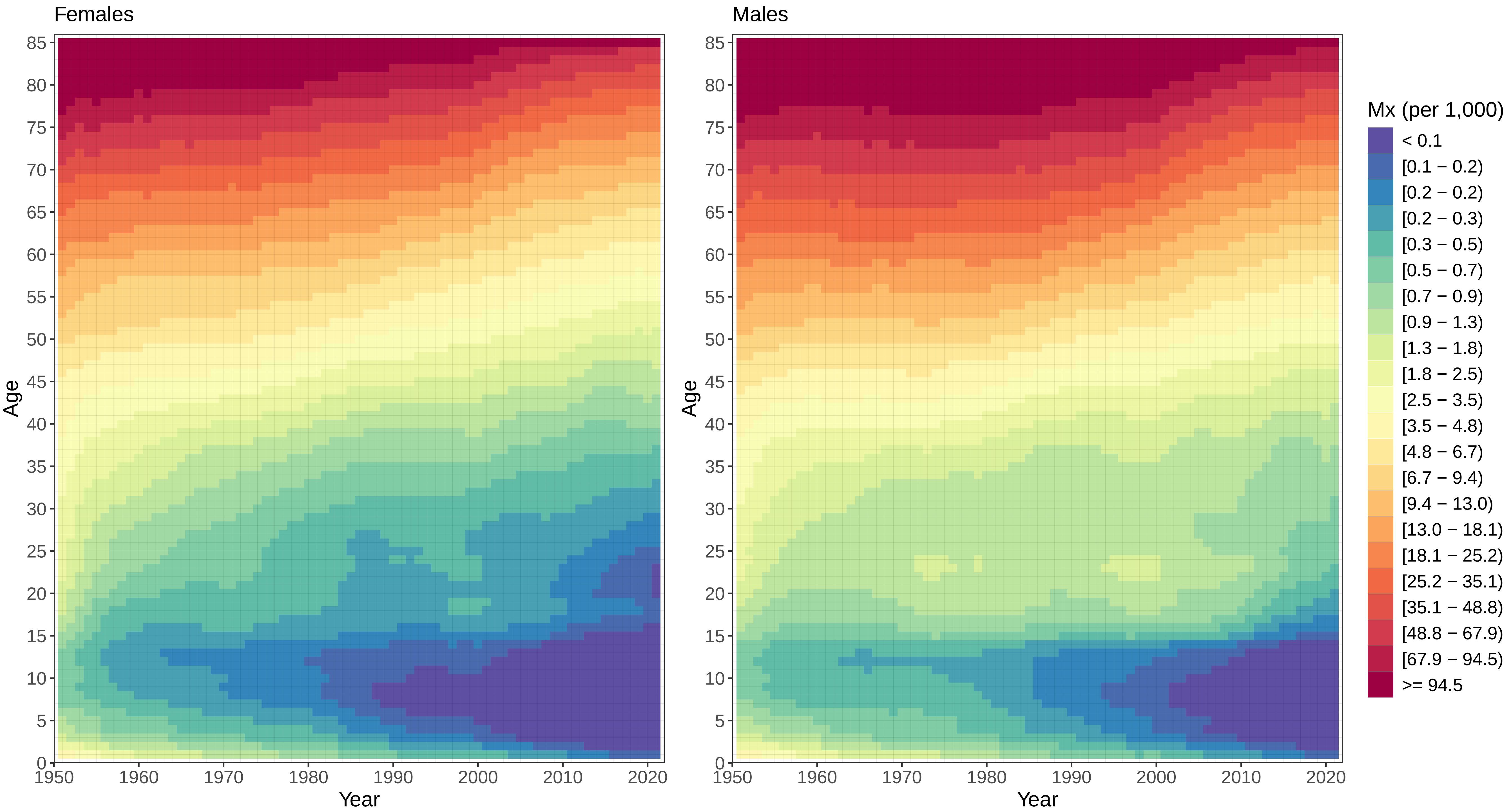

Appendix 2 Figure S4: Israel mortality rates, 1950−2023

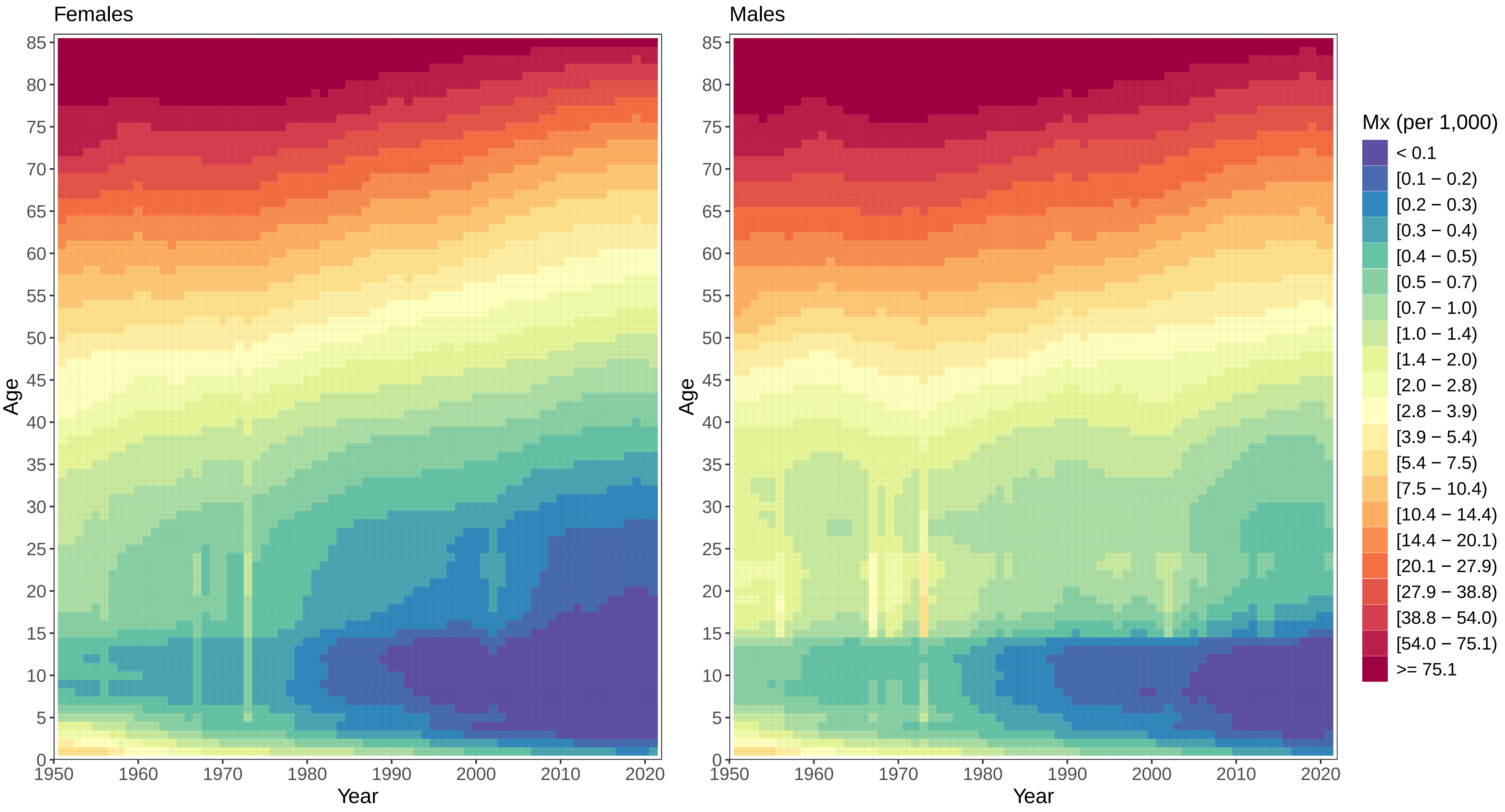

Appendix 2 Figure S4: Italy mortality rates, 1950−2023

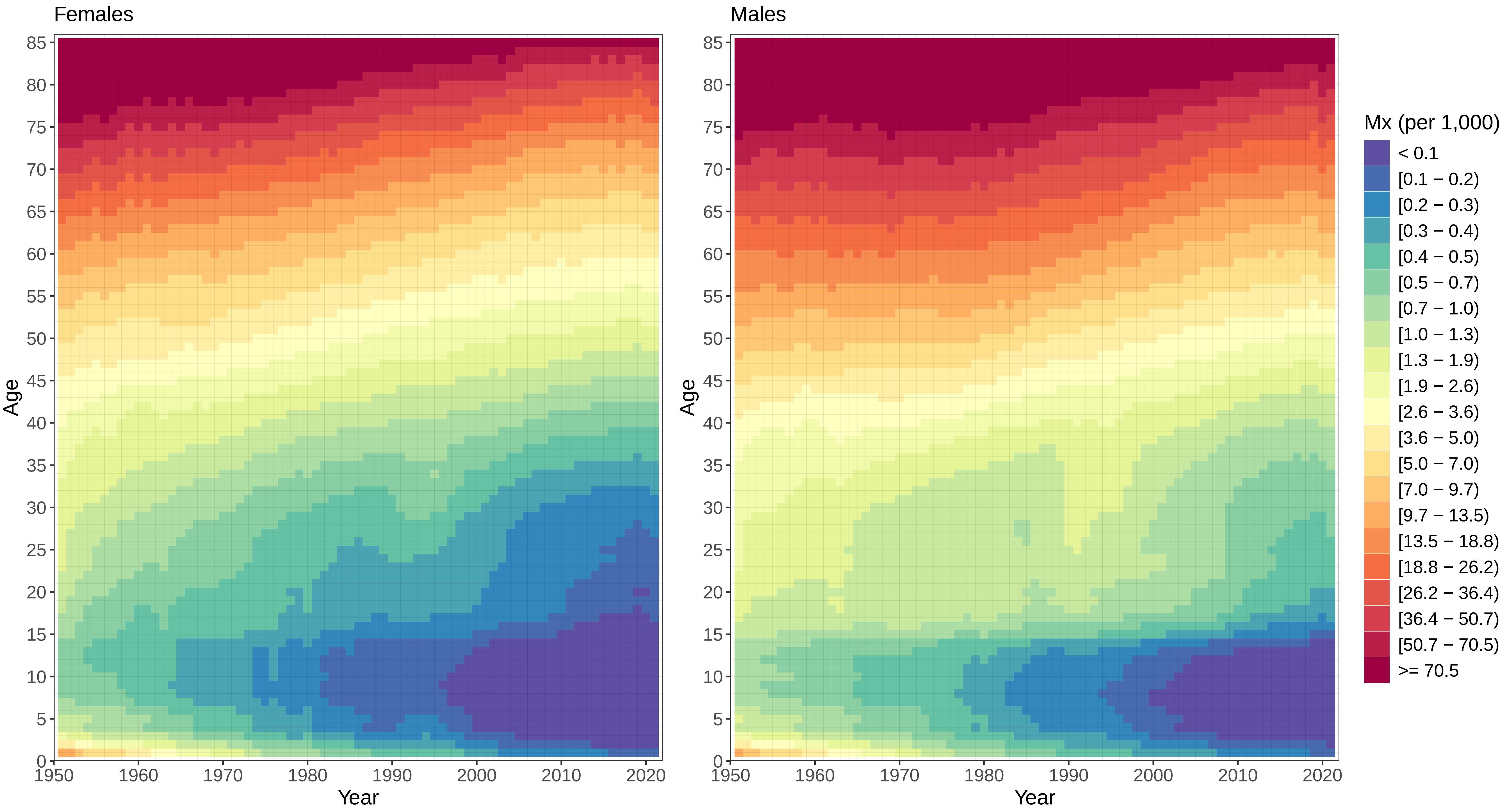

Appendix 2 Figure S4: Luxembourg mortality rates, 1950−2023

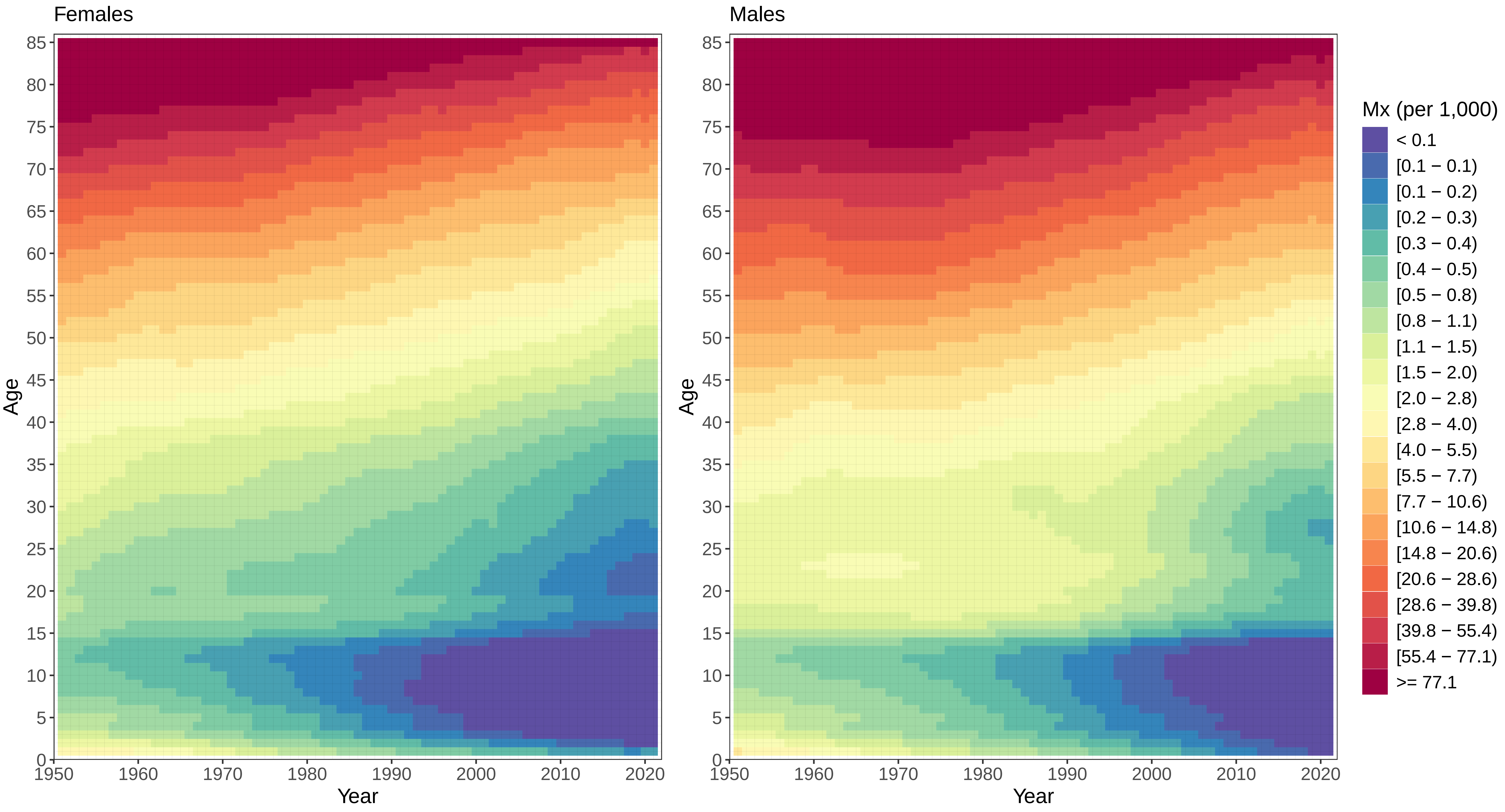

Appendix 2 Figure S4: Malta mortality rates, 1950−2023

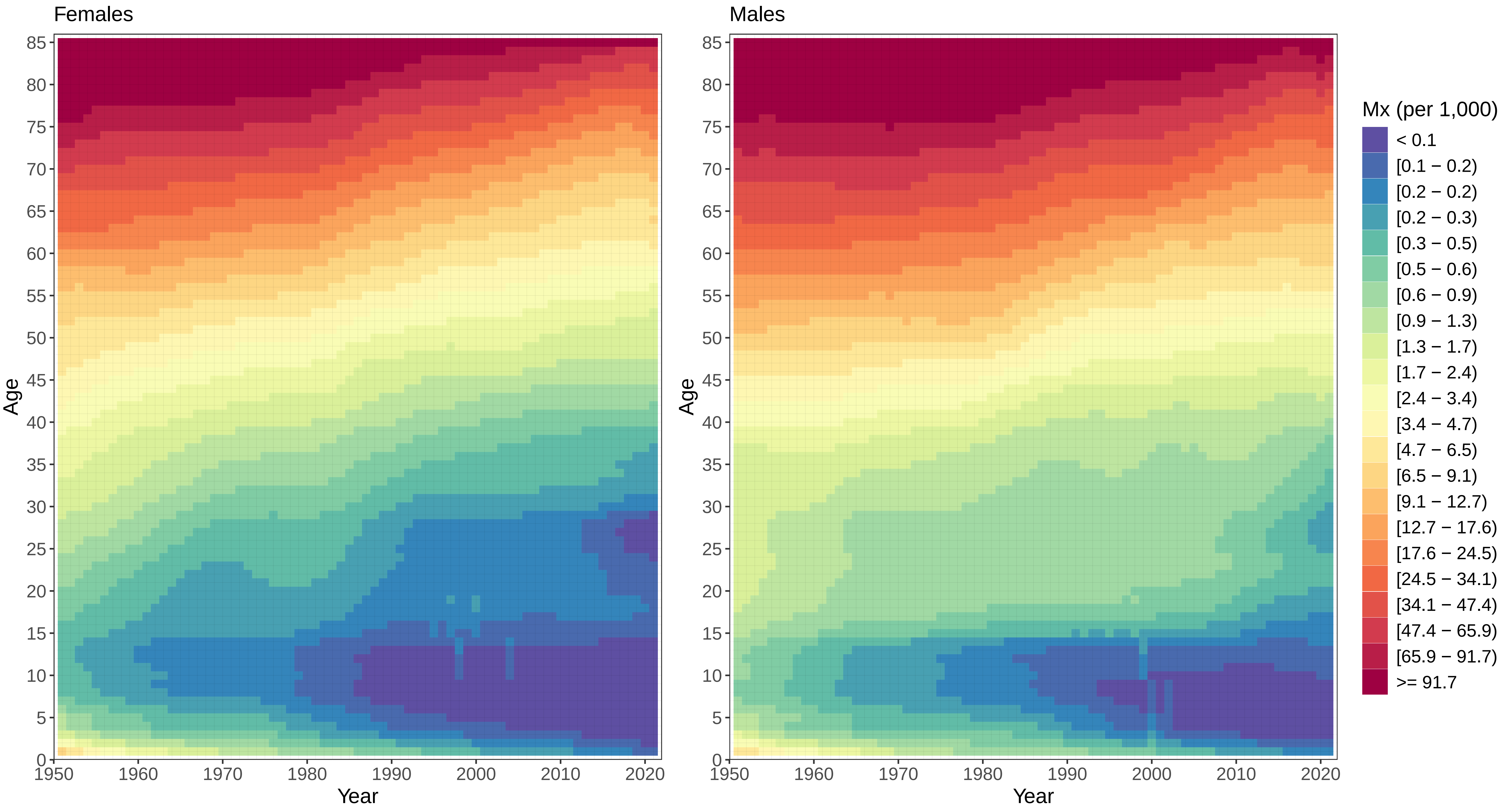

Appendix 2 Figure S4: Monaco mortality rates, 1950−2023

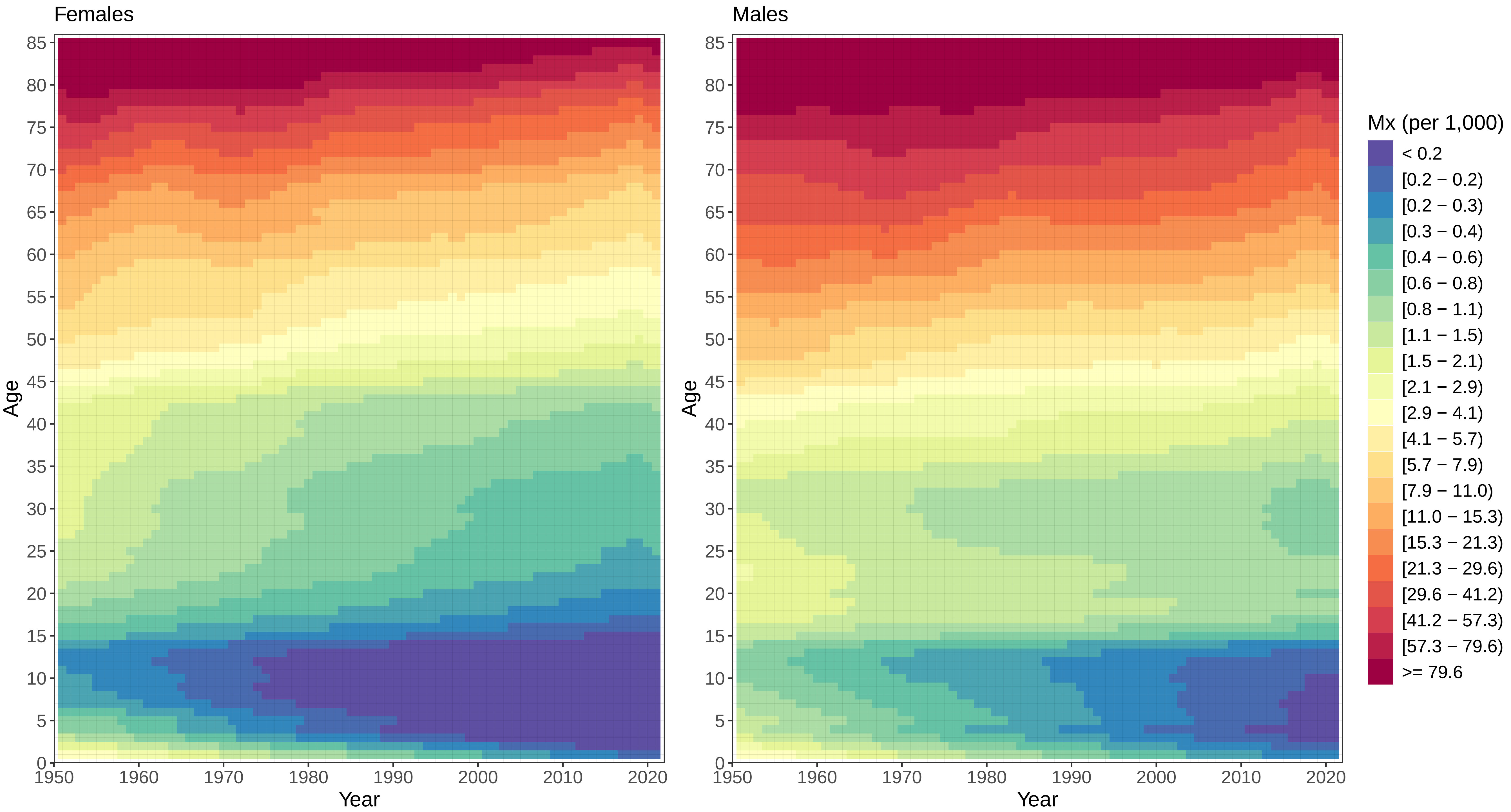

Appendix 2 Figure S4: Netherlands mortality rates, 1950−2023

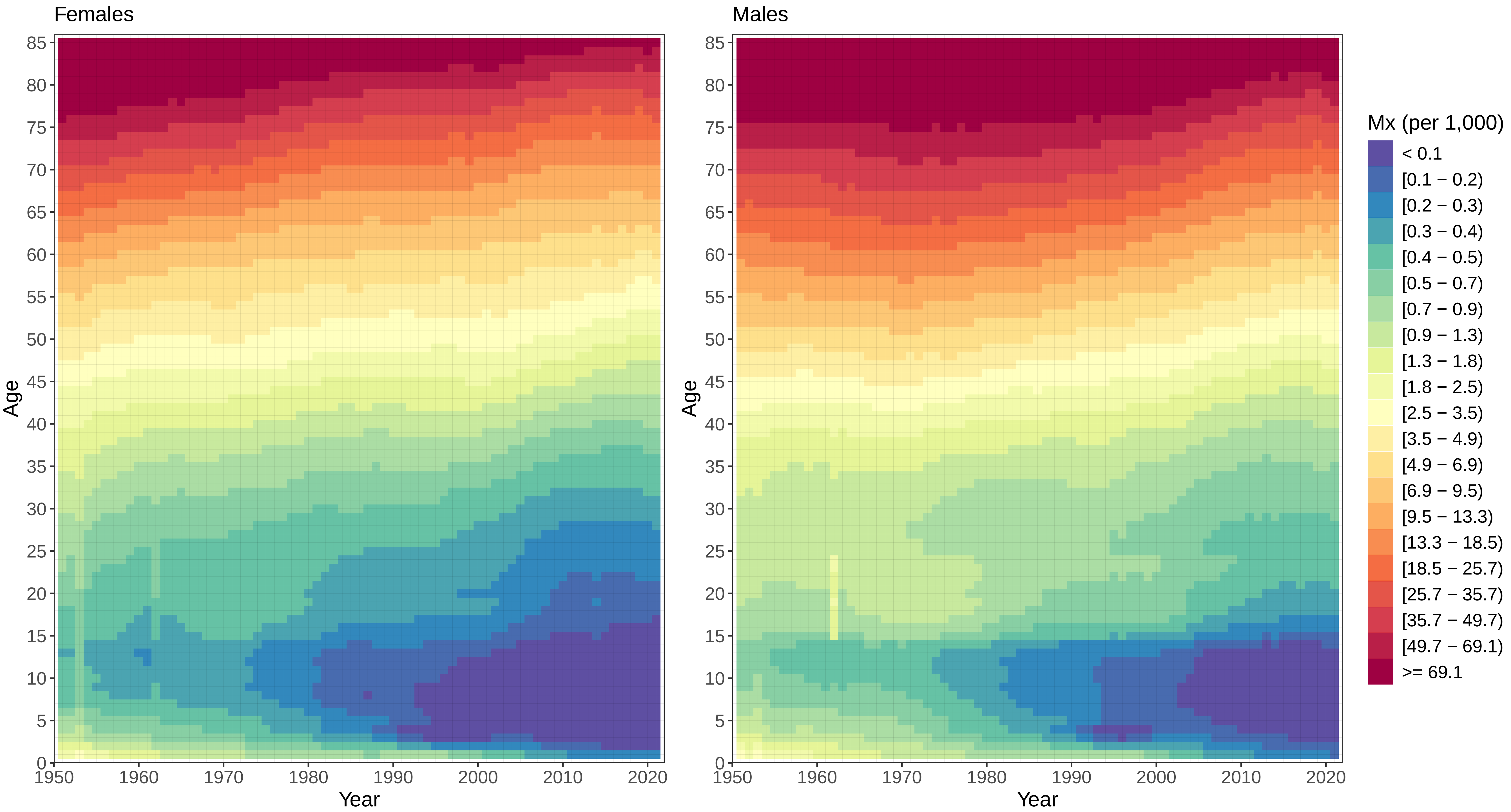

Appendix 2 Figure S4: Norway mortality rates, 1950−2023

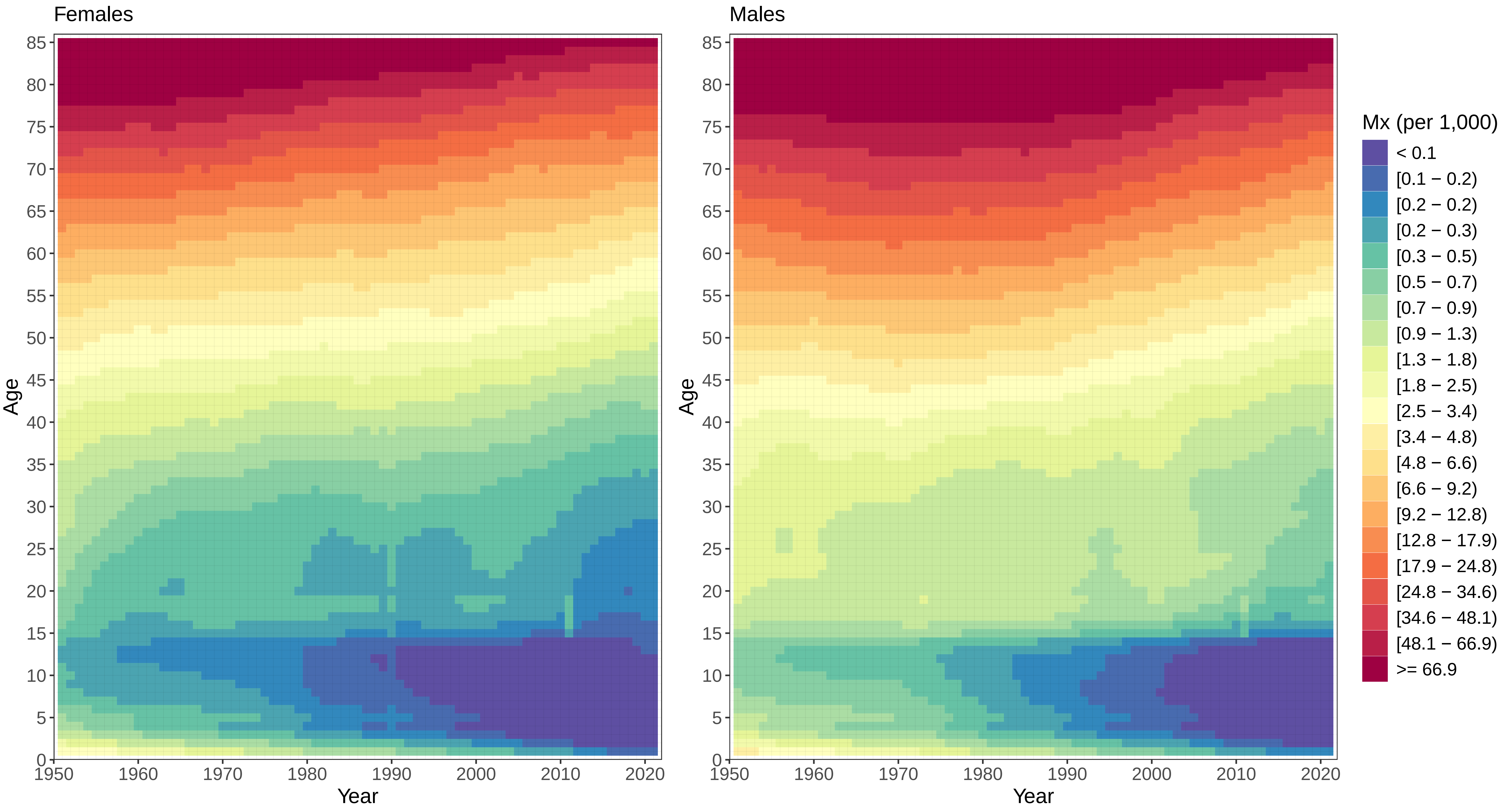

Appendix 2 Figure S4: Portugal mortality rates, 1950−2023

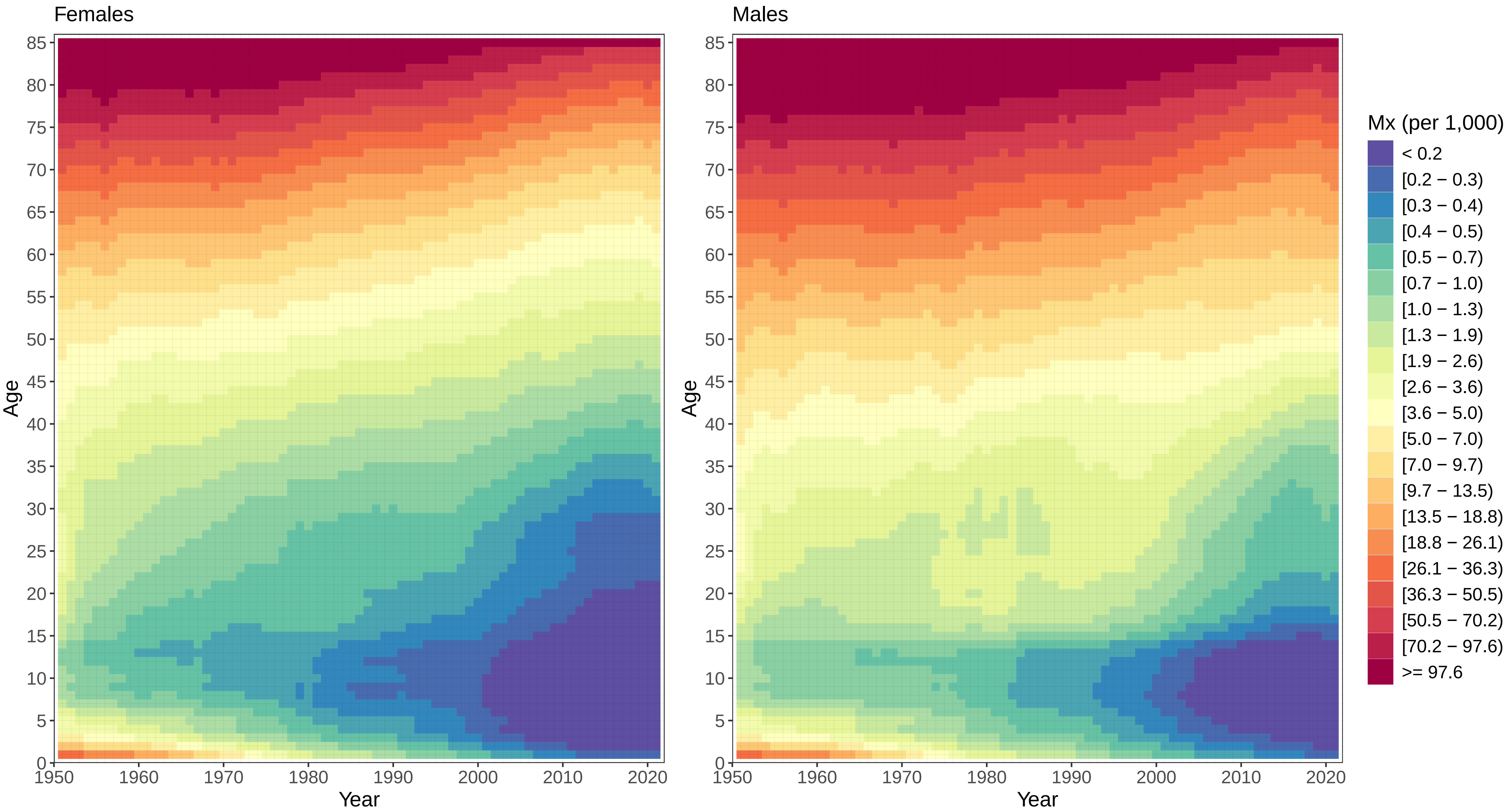

Appendix 2 Figure S4: San Marino mortality rates, 1950−2023

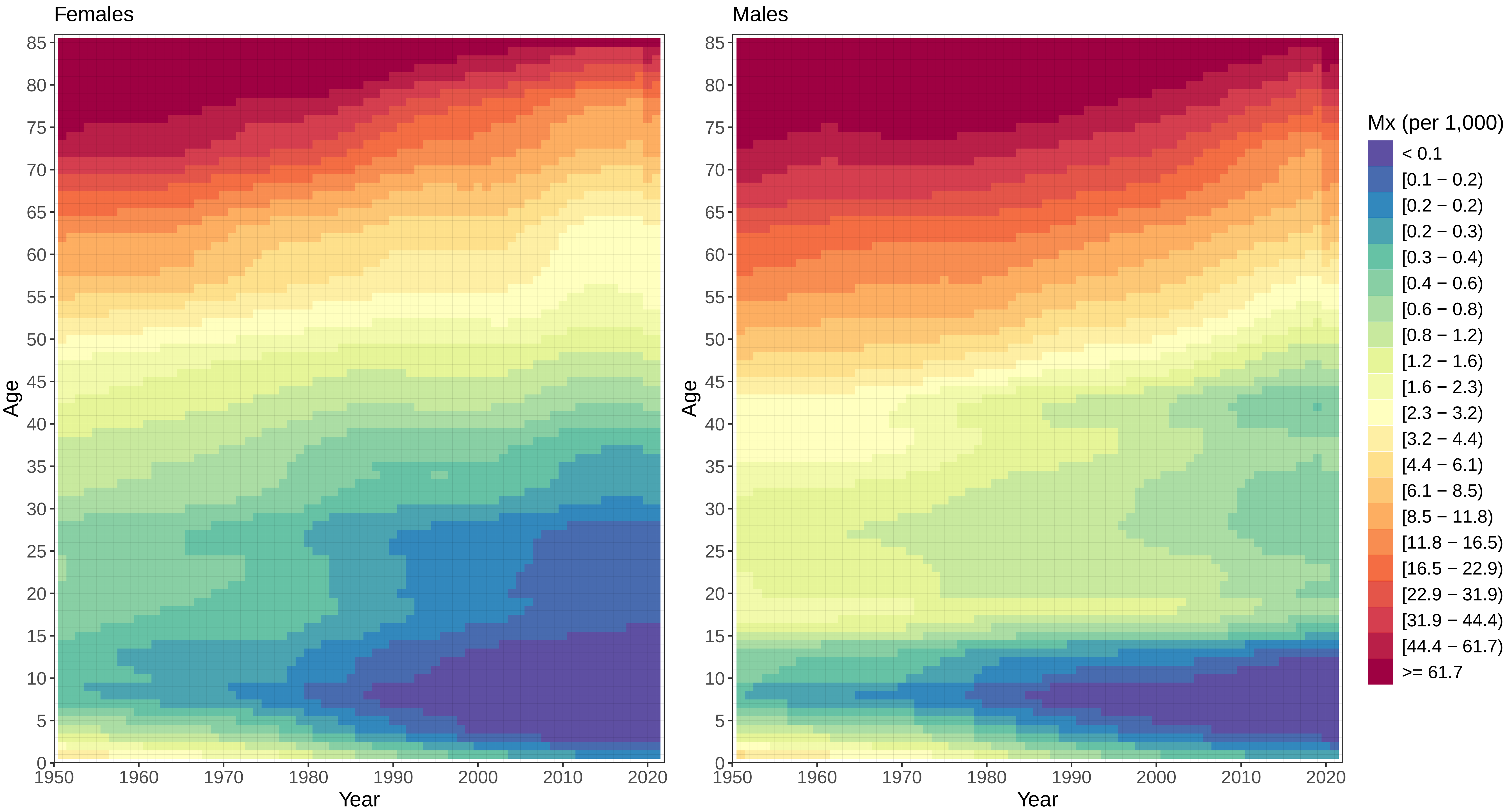

Appendix 2 Figure S4: Spain mortality rates, 1950−2023

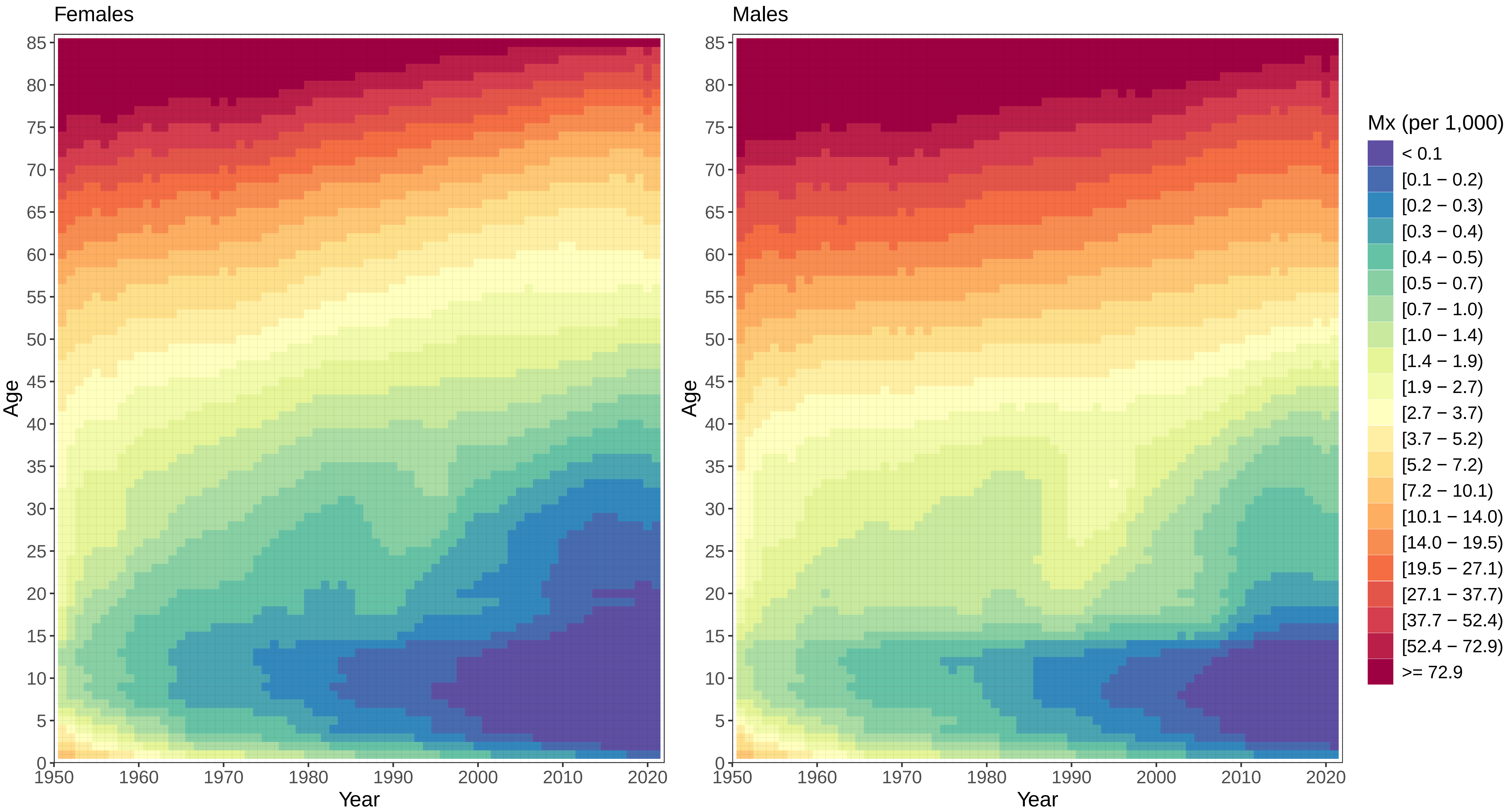

Appendix 2 Figure S4: Sweden mortality rates, 1950−2023

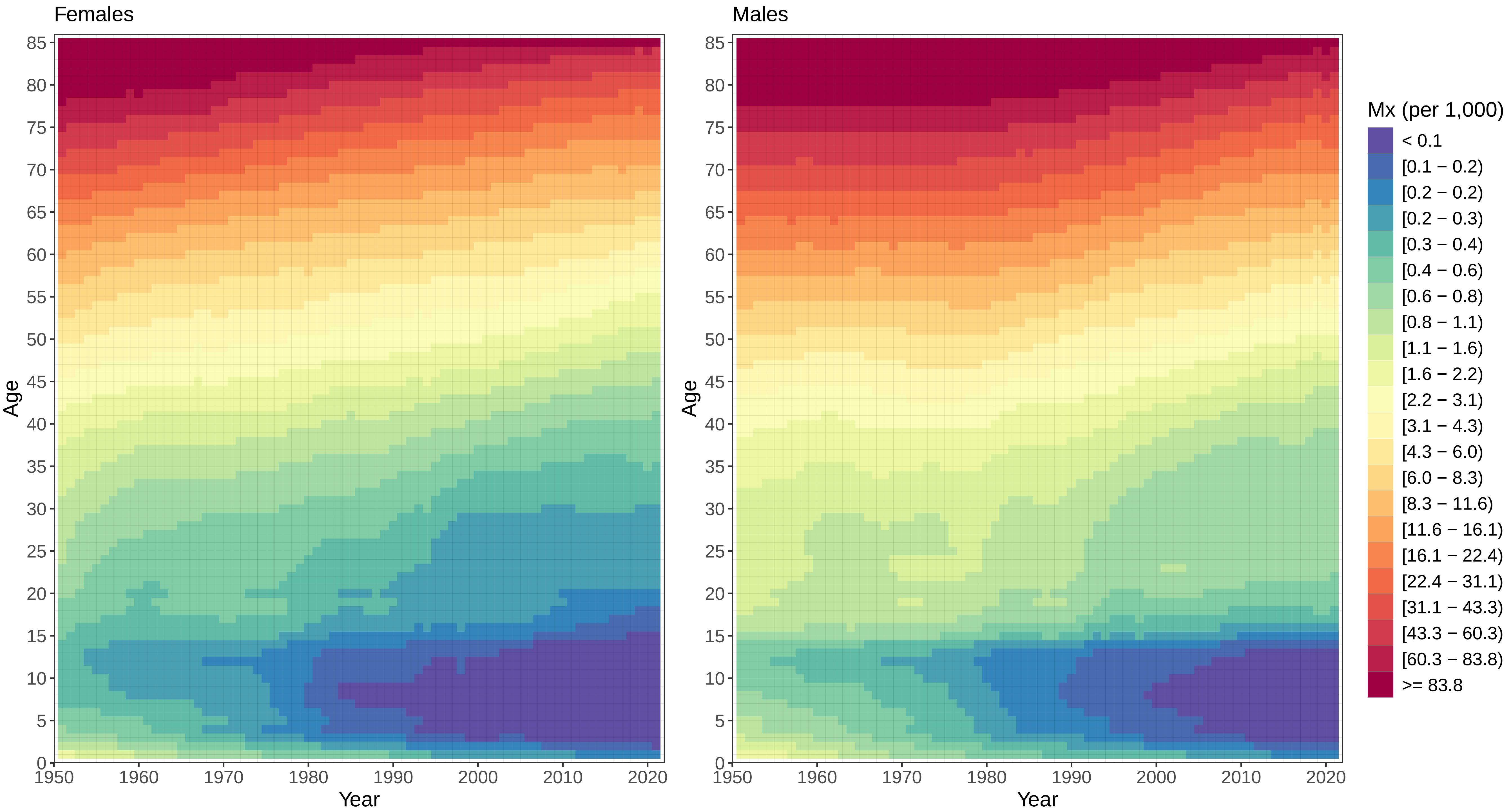

Appendix 2 Figure S4: Switzerland mortality rates, 1950−2023

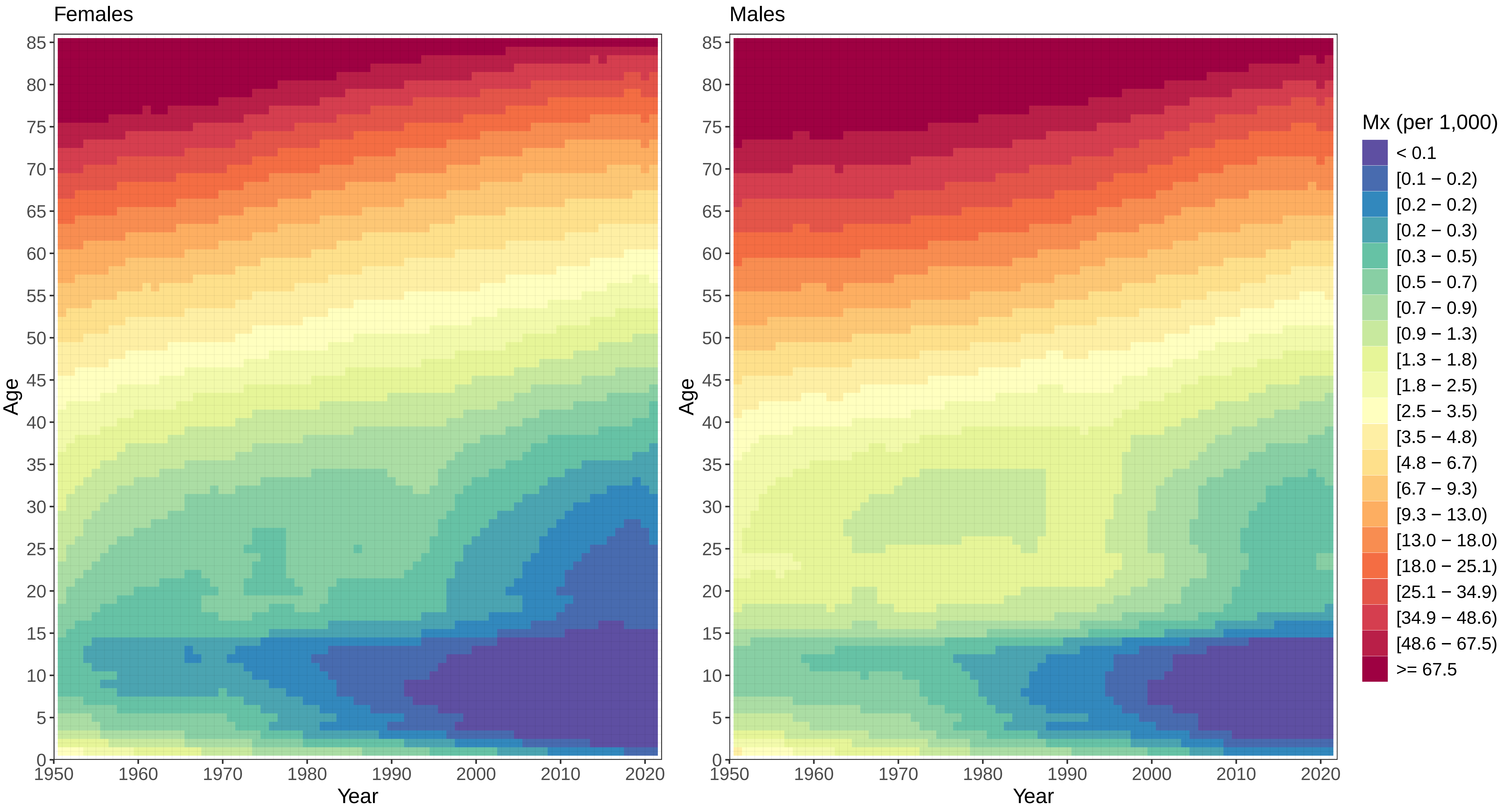

Appendix 2 Figure S4: UK mortality rates, 1950−2023

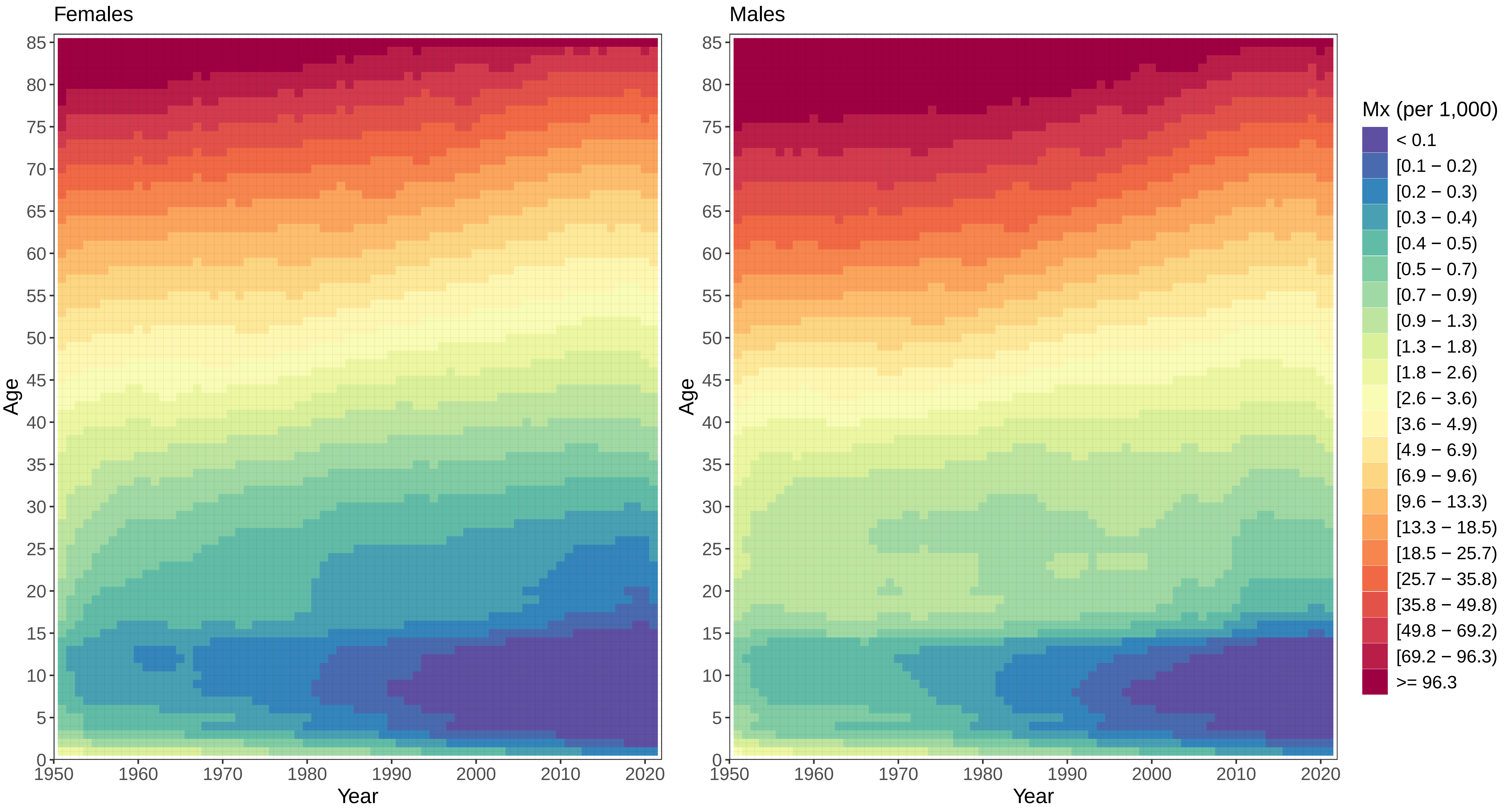

Appendix 2 Figure S4: Bolivia mortality rates, 1950−2023

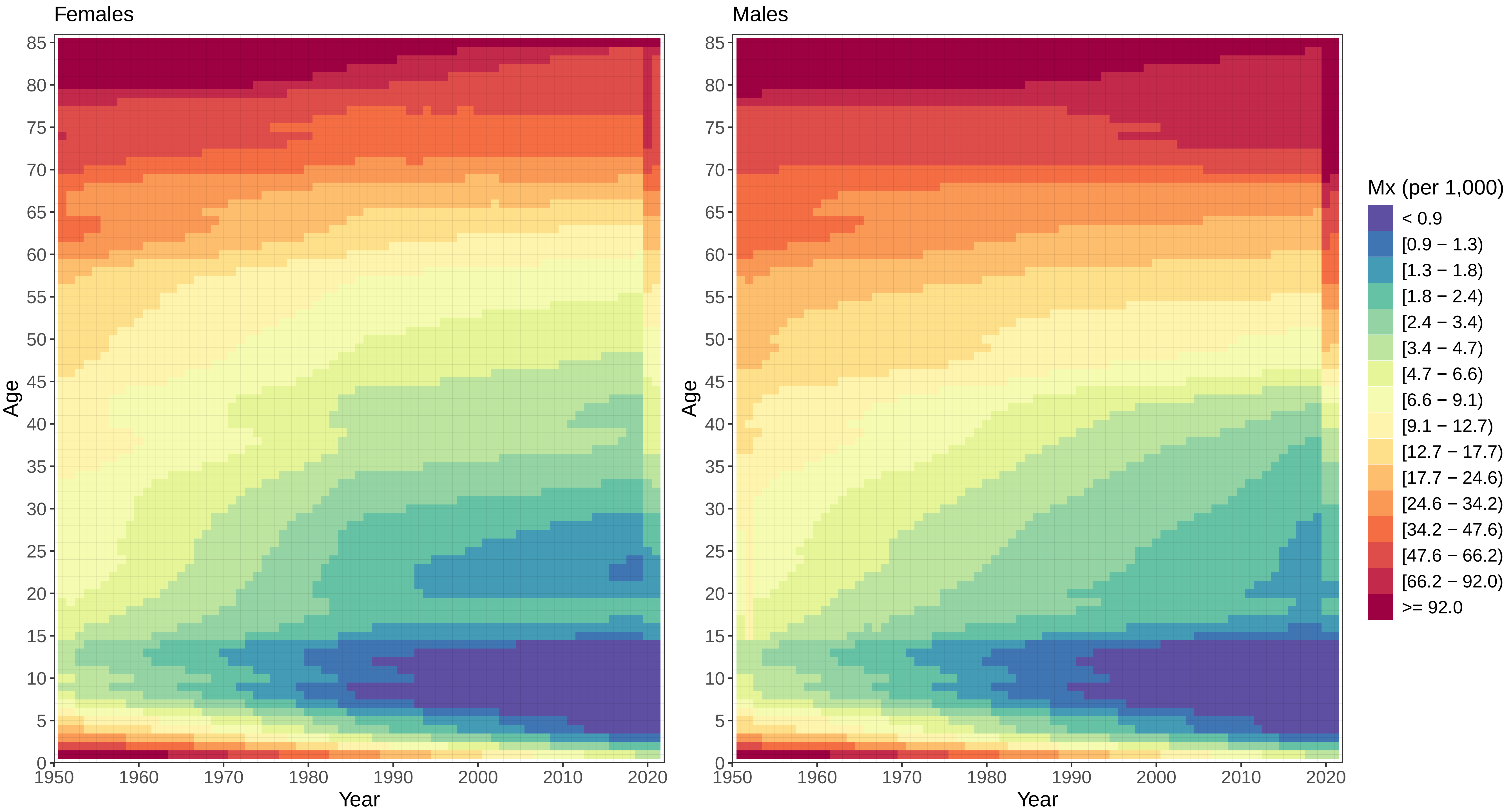

Appendix 2 Figure S4: Ecuador mortality rates, 1950−2023

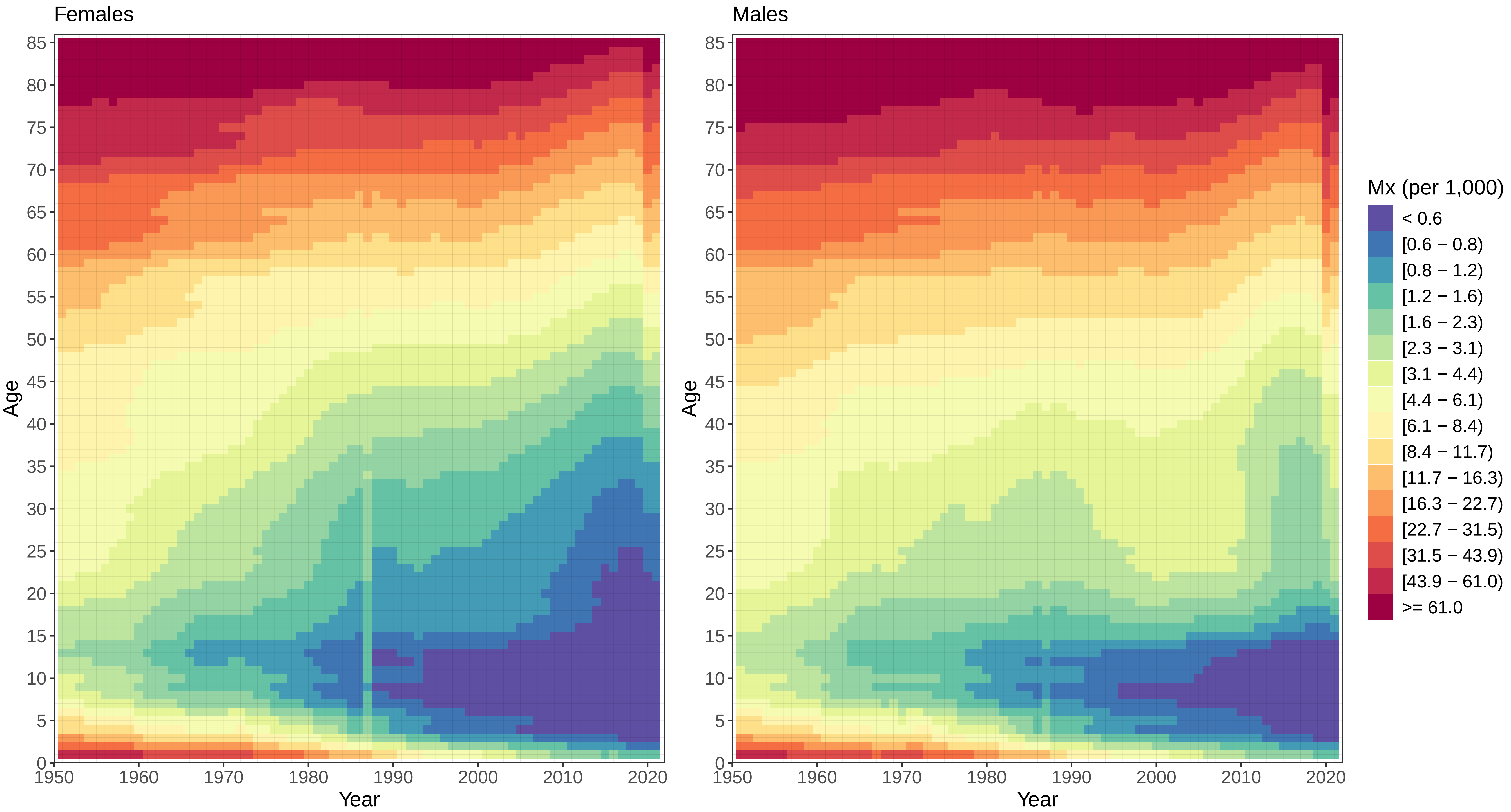

Appendix 2 Figure S4: Peru mortality rates, 1950−2023

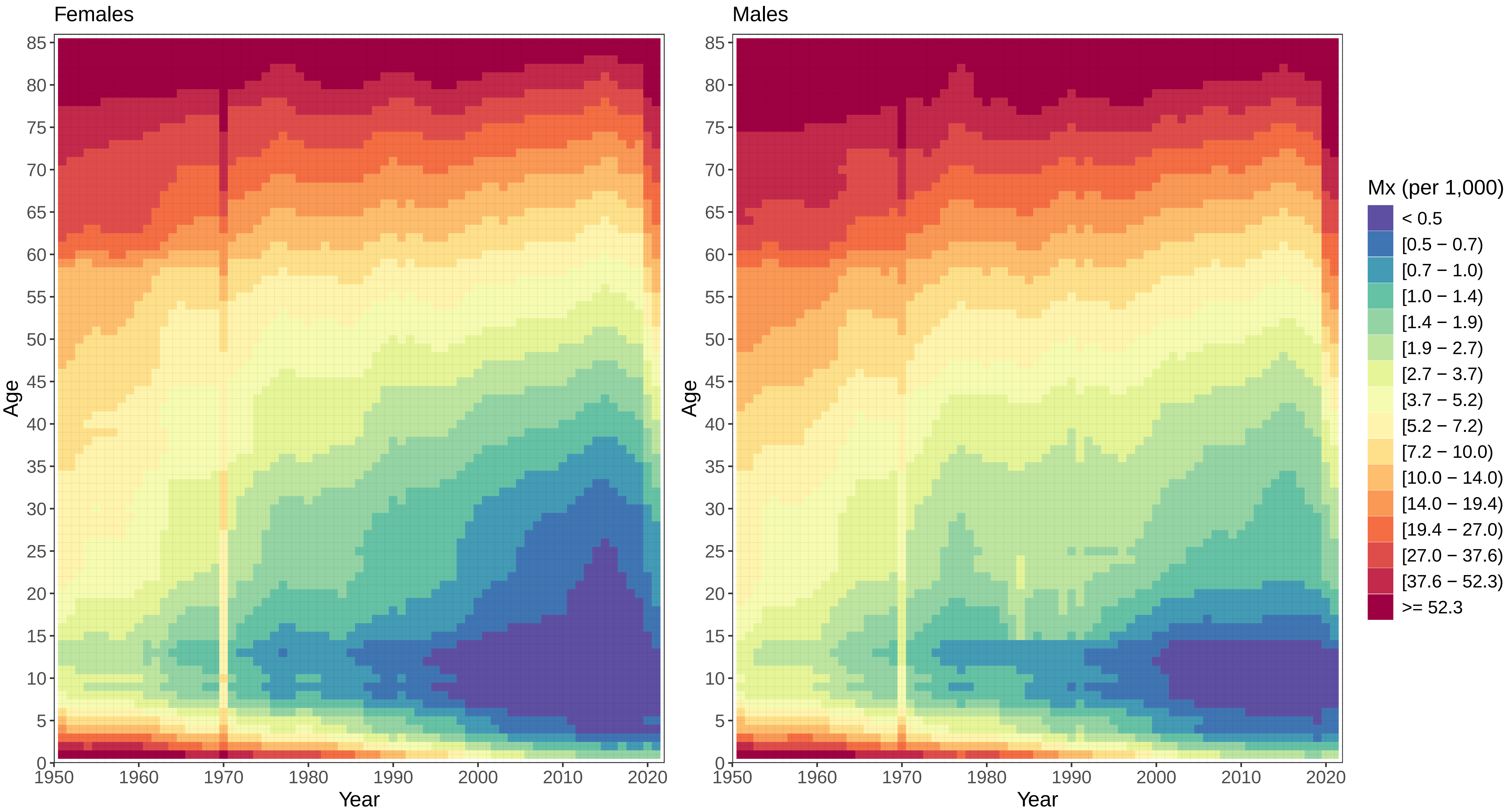

Appendix 2 Figure S4: Antigua and Barbuda mortality rates, 1950−2023

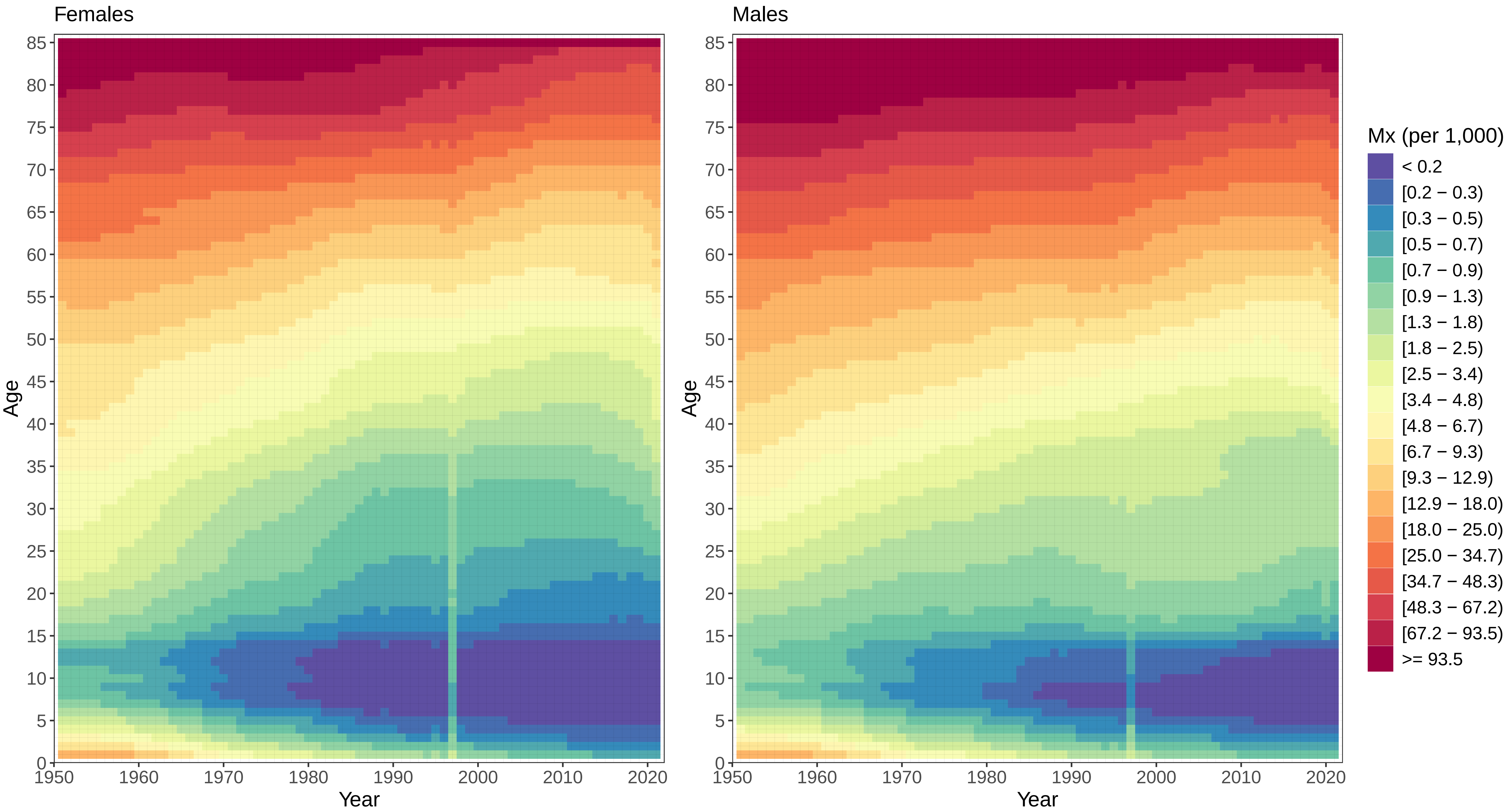

Appendix 2 Figure S4: Barbados mortality rates, 1950−2023

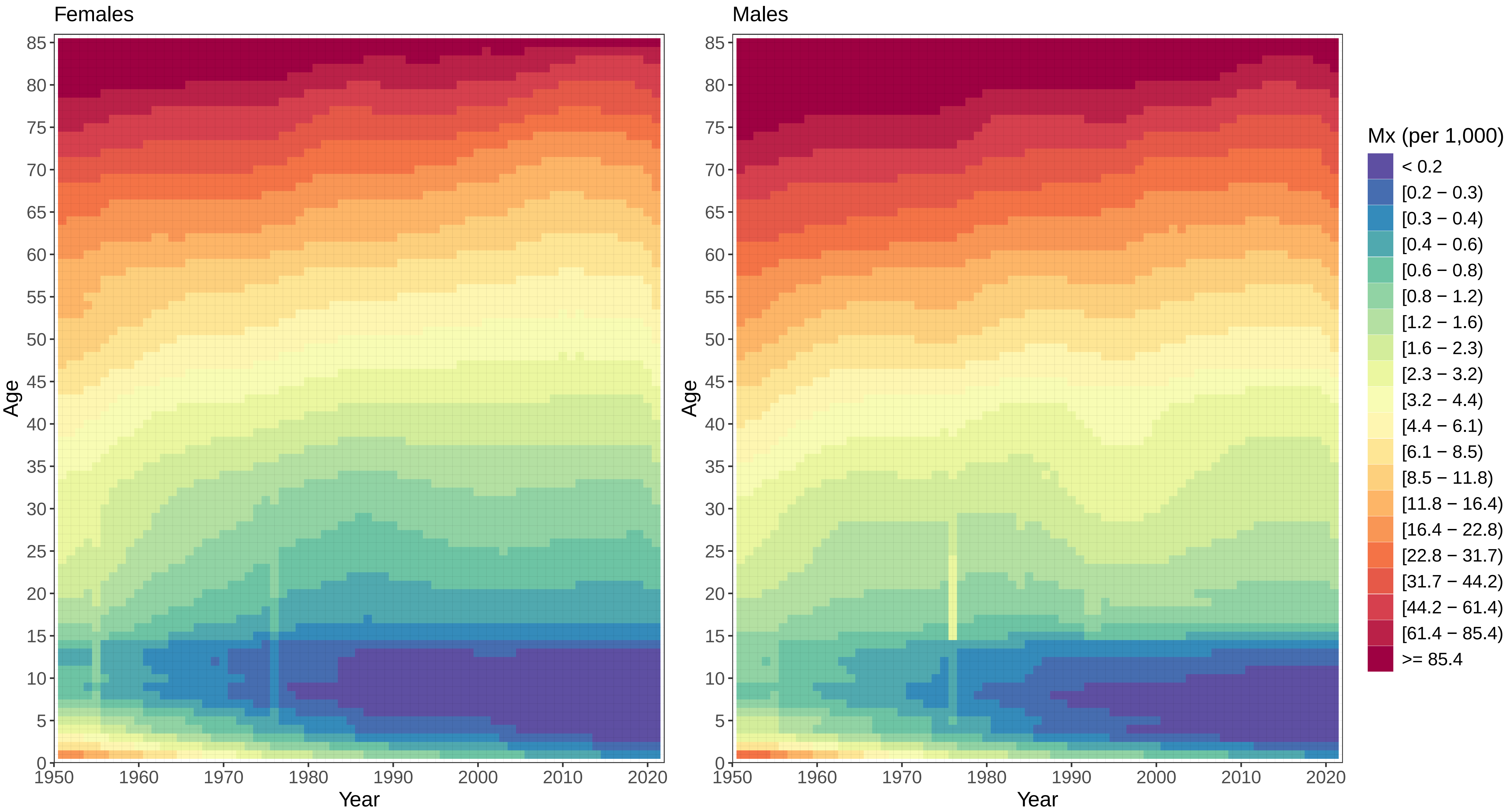

Appendix 2 Figure S4: Belize mortality rates, 1950−2023

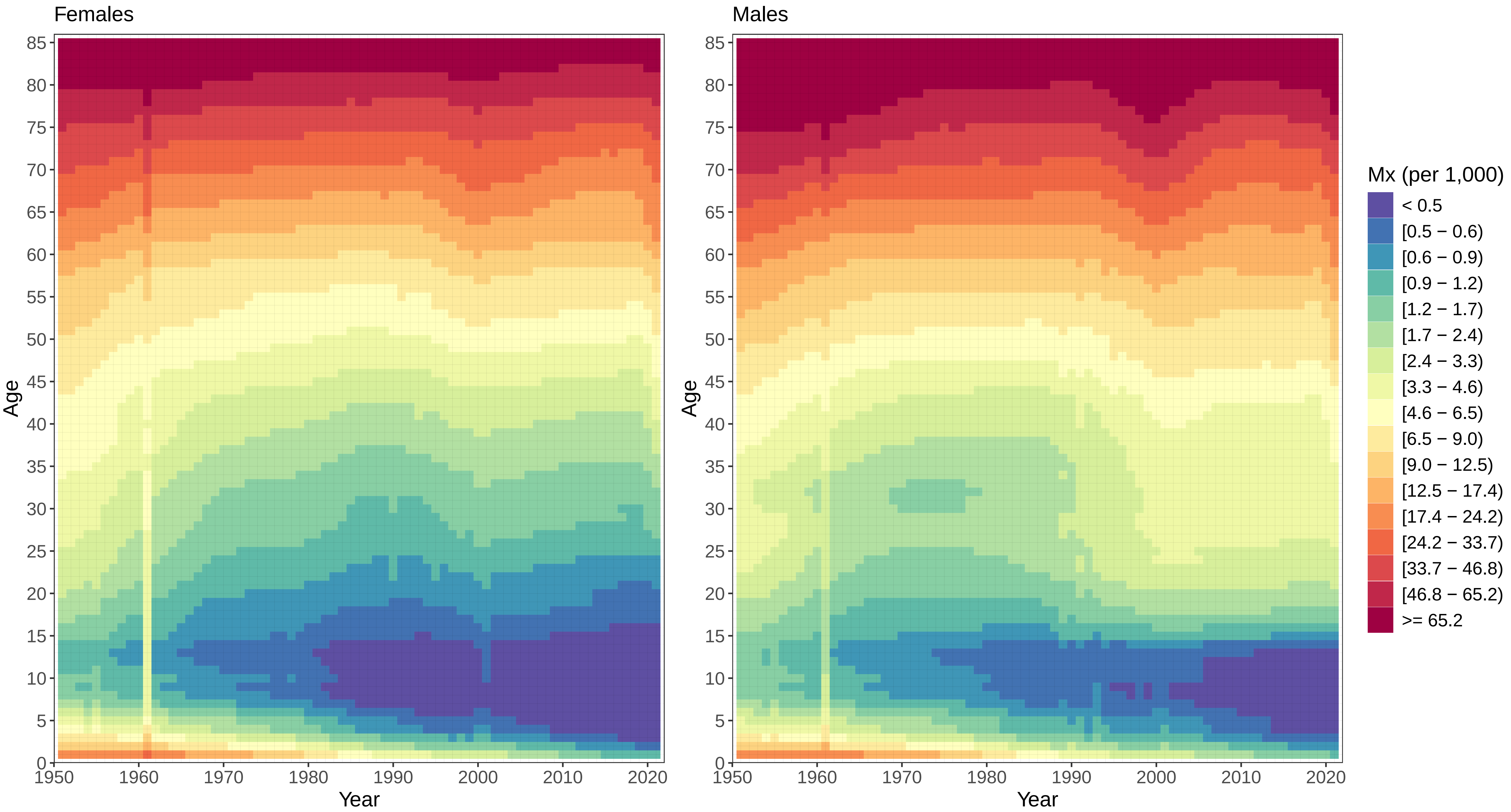

Appendix 2 Figure S4: Bermuda mortality rates, 1950−2023

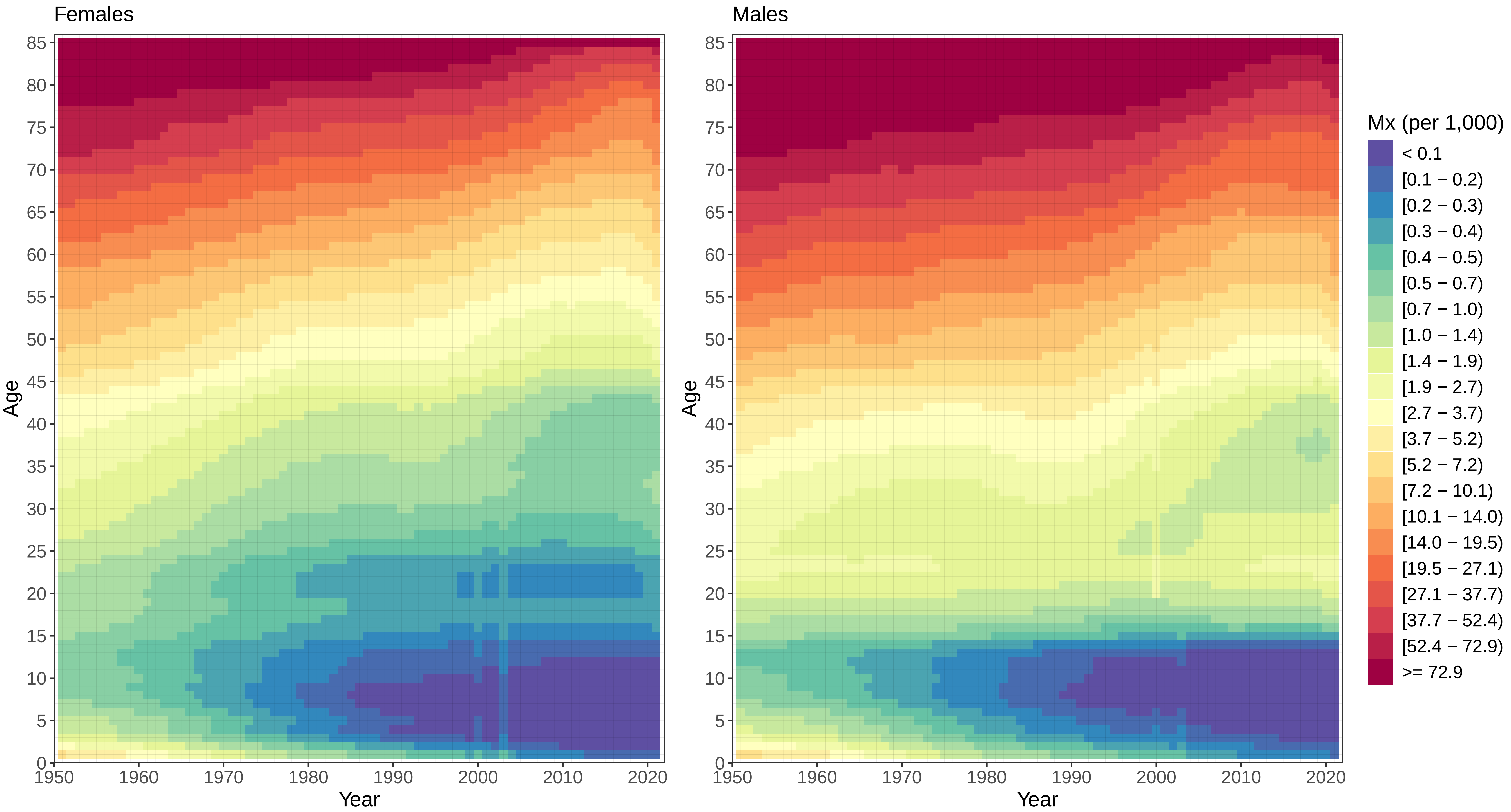

Appendix 2 Figure S4: Cuba mortality rates, 1950−2023

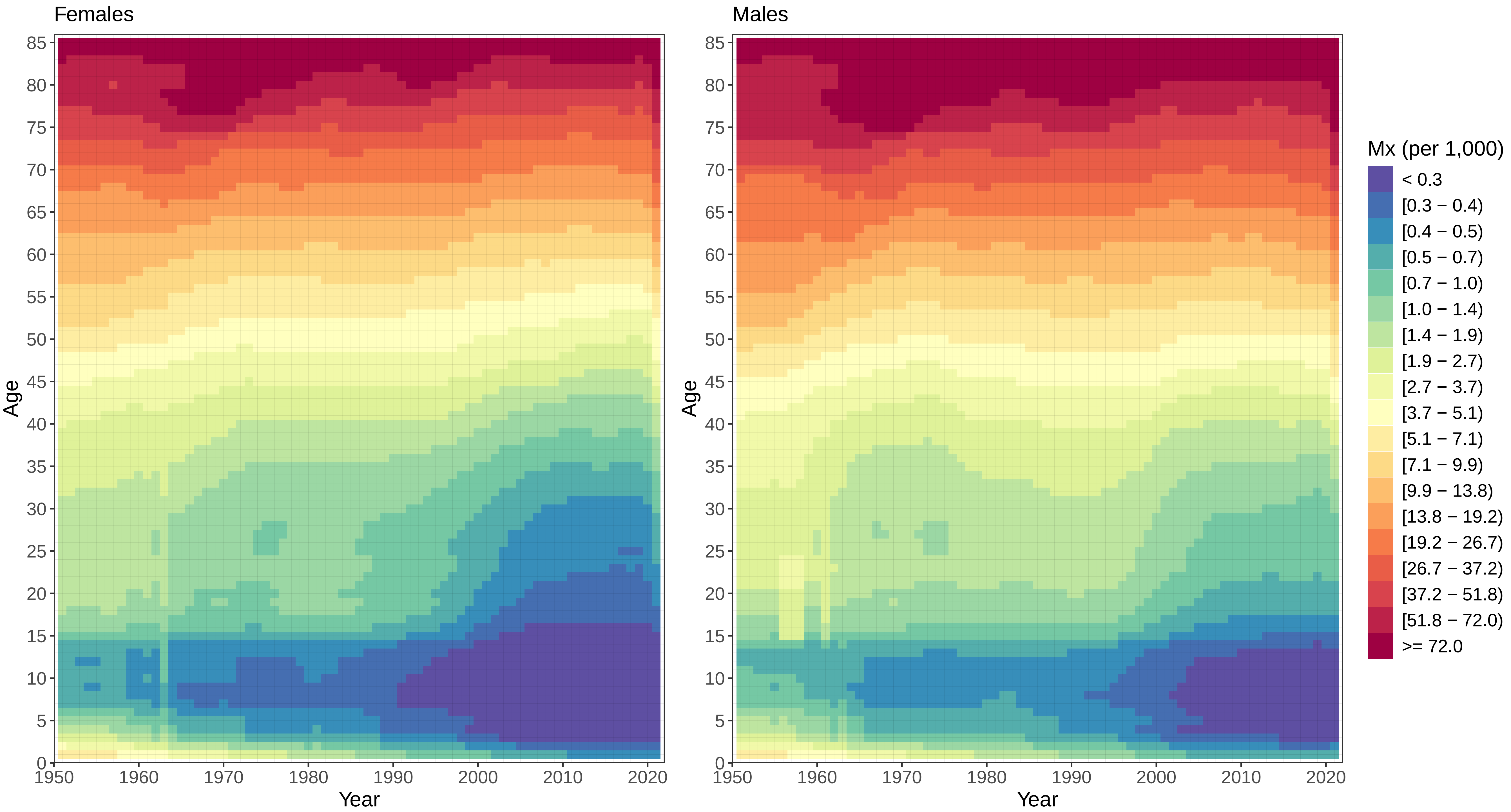

Appendix 2 Figure S4: Dominica mortality rates, 1950−2023

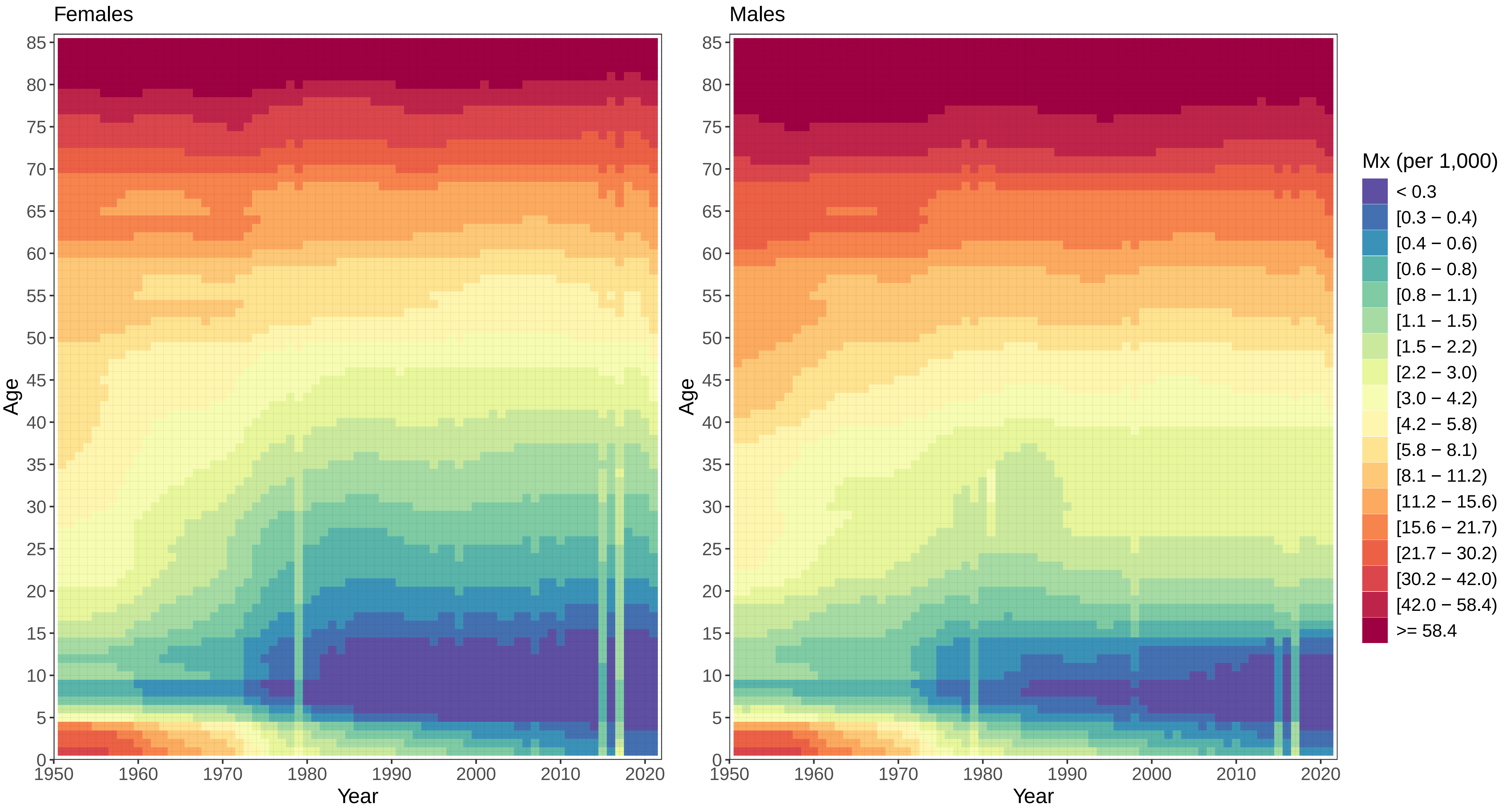

Appendix 2 Figure S4: Dominican Republic mortality rates, 1950−2023

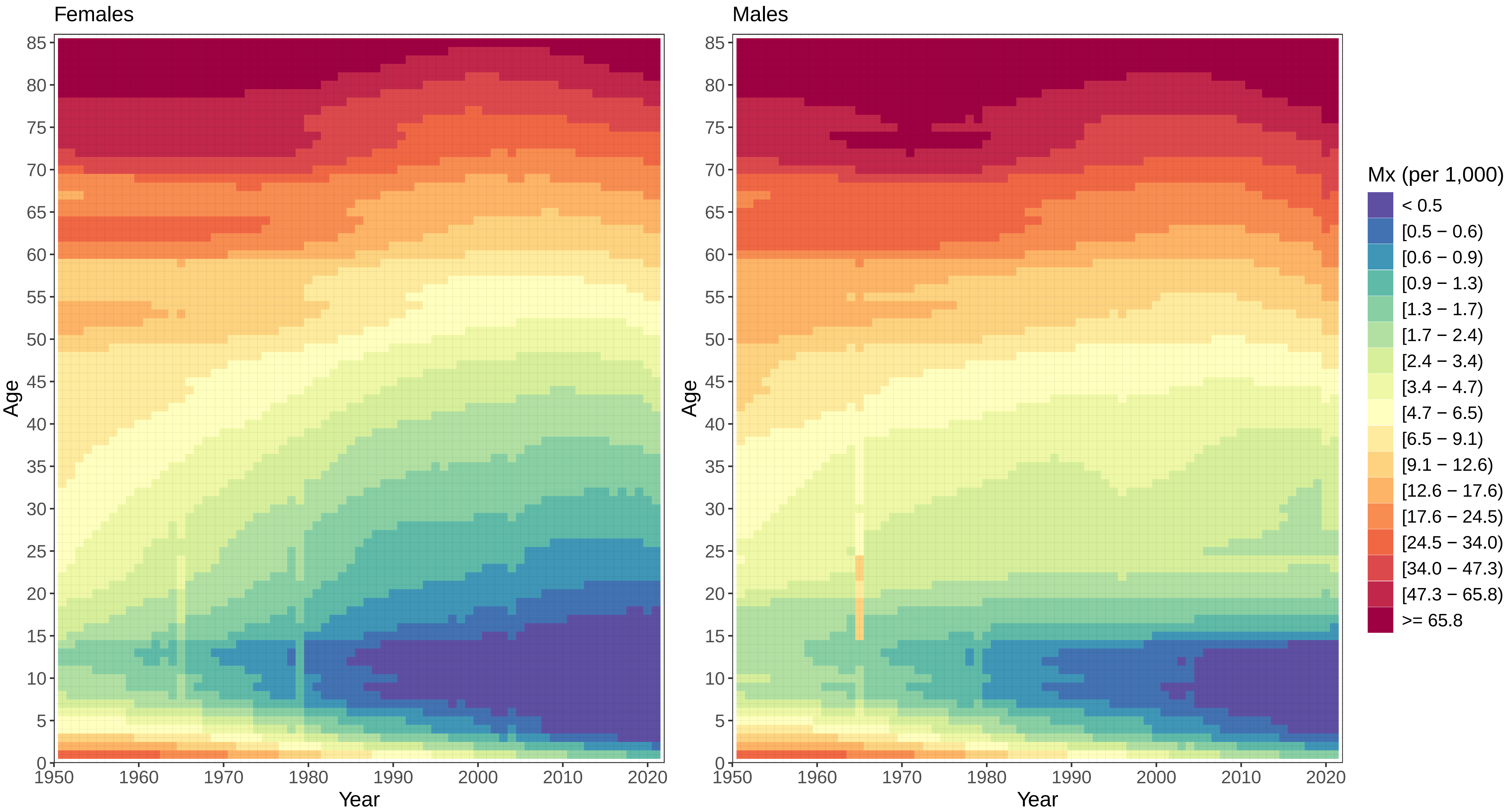

Appendix 2 Figure S4: Grenada mortality rates, 1950−2023

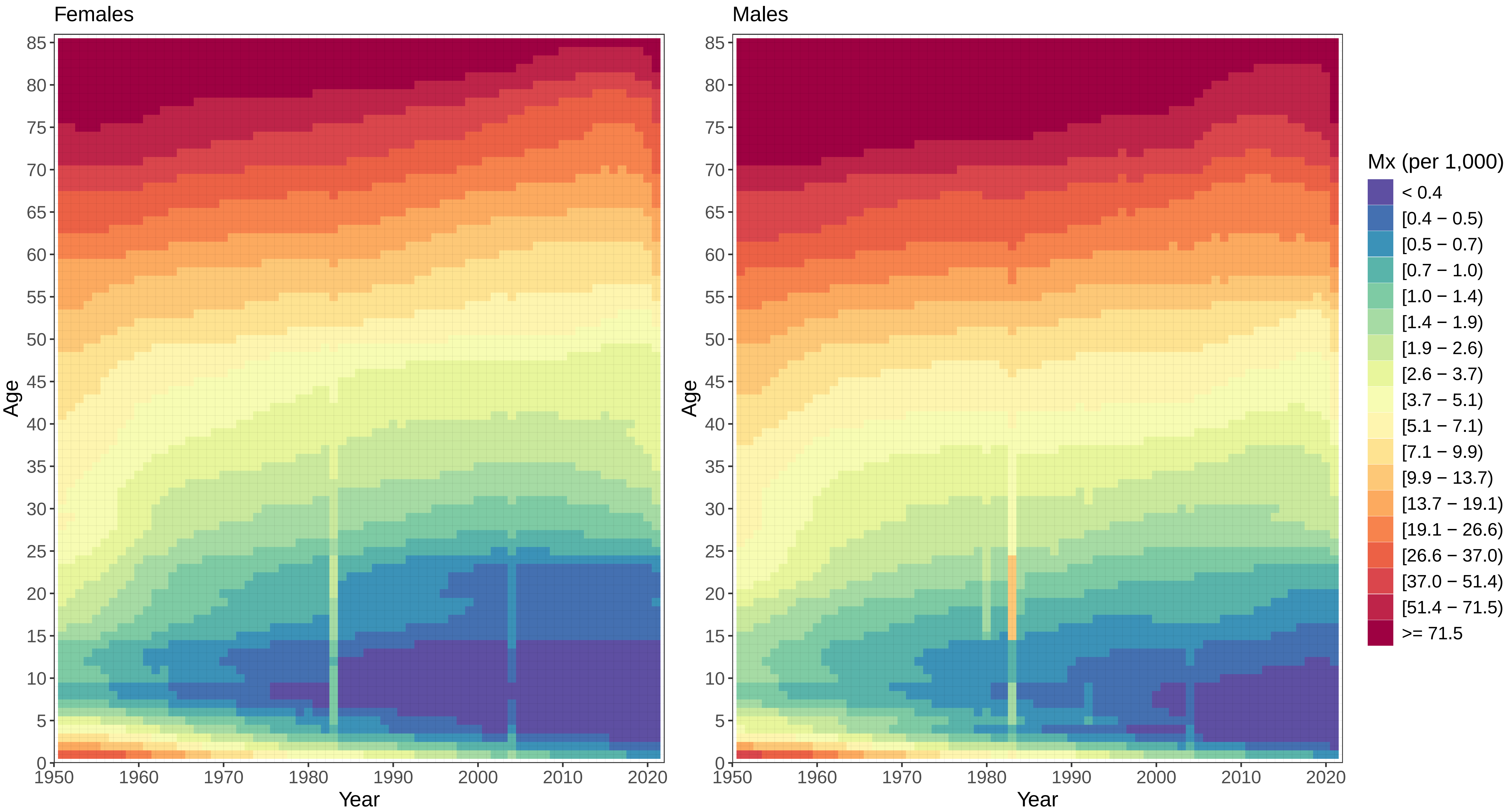

Appendix 2 Figure S4: Guyana mortality rates, 1950−2023

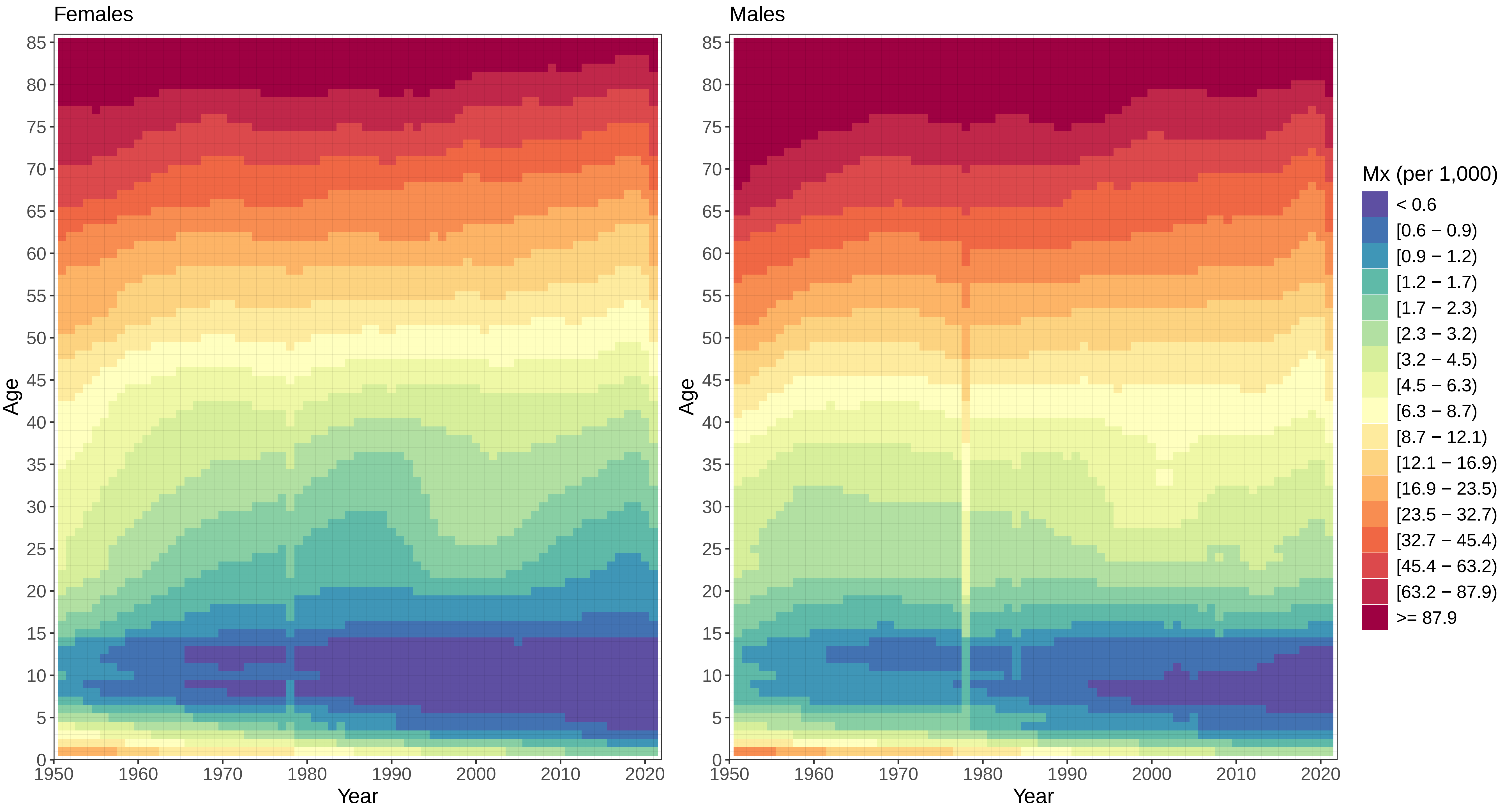

Appendix 2 Figure S4: Haiti mortality rates, 1950−2023

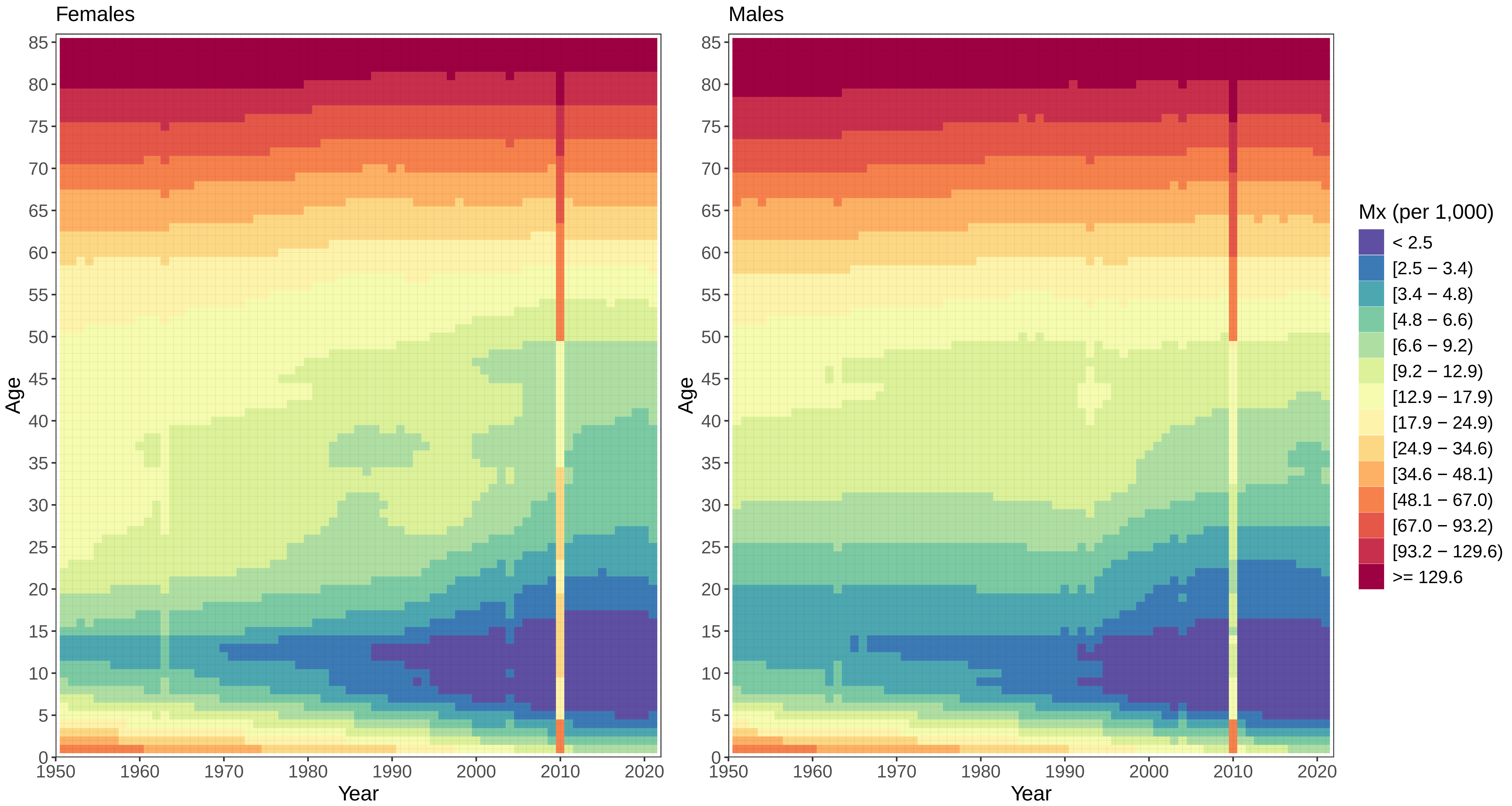

Appendix 2 Figure S4: Jamaica mortality rates, 1950−2023

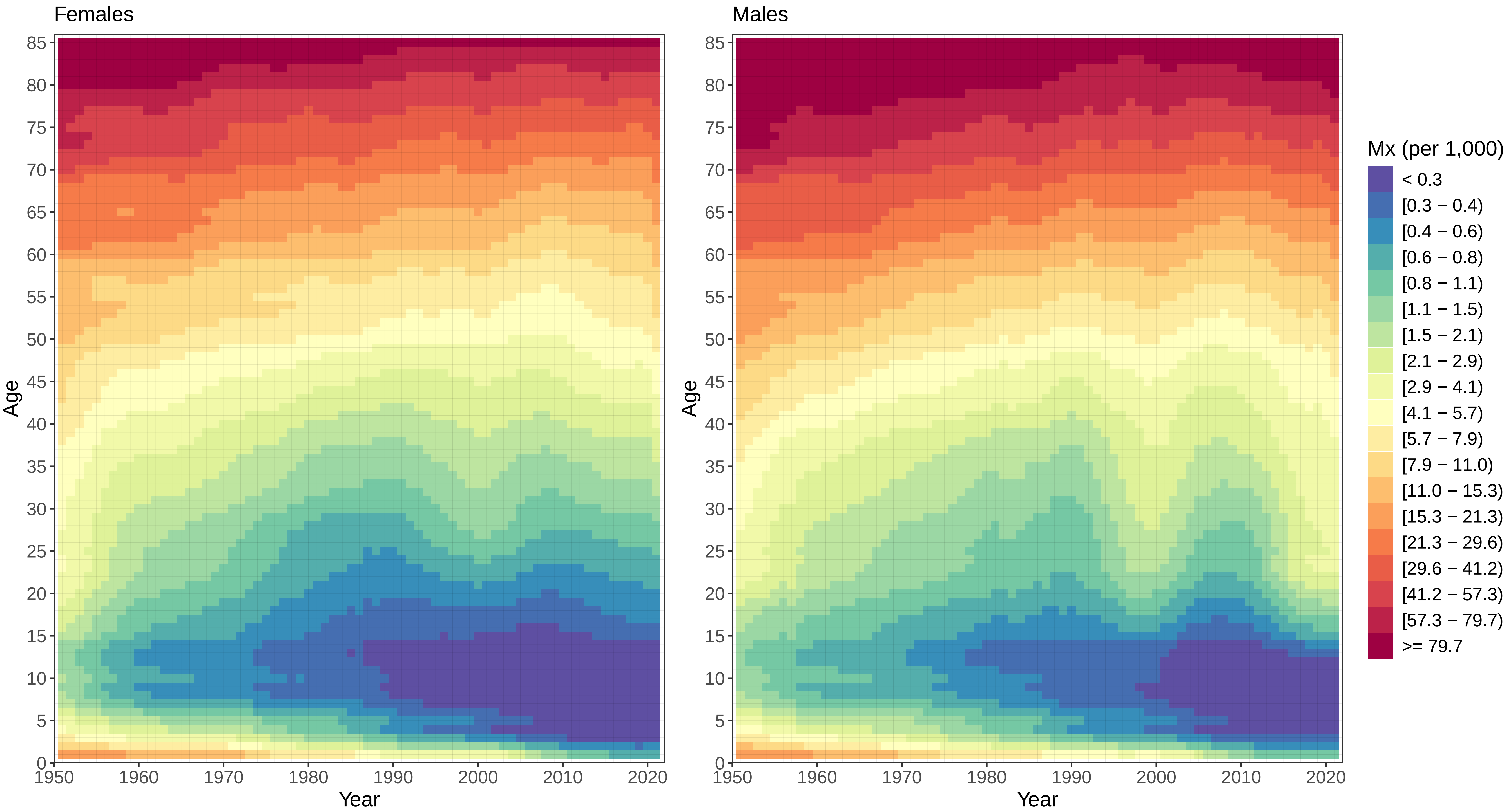

Appendix 2 Figure S4: Puerto Rico mortality rates, 1950−2023

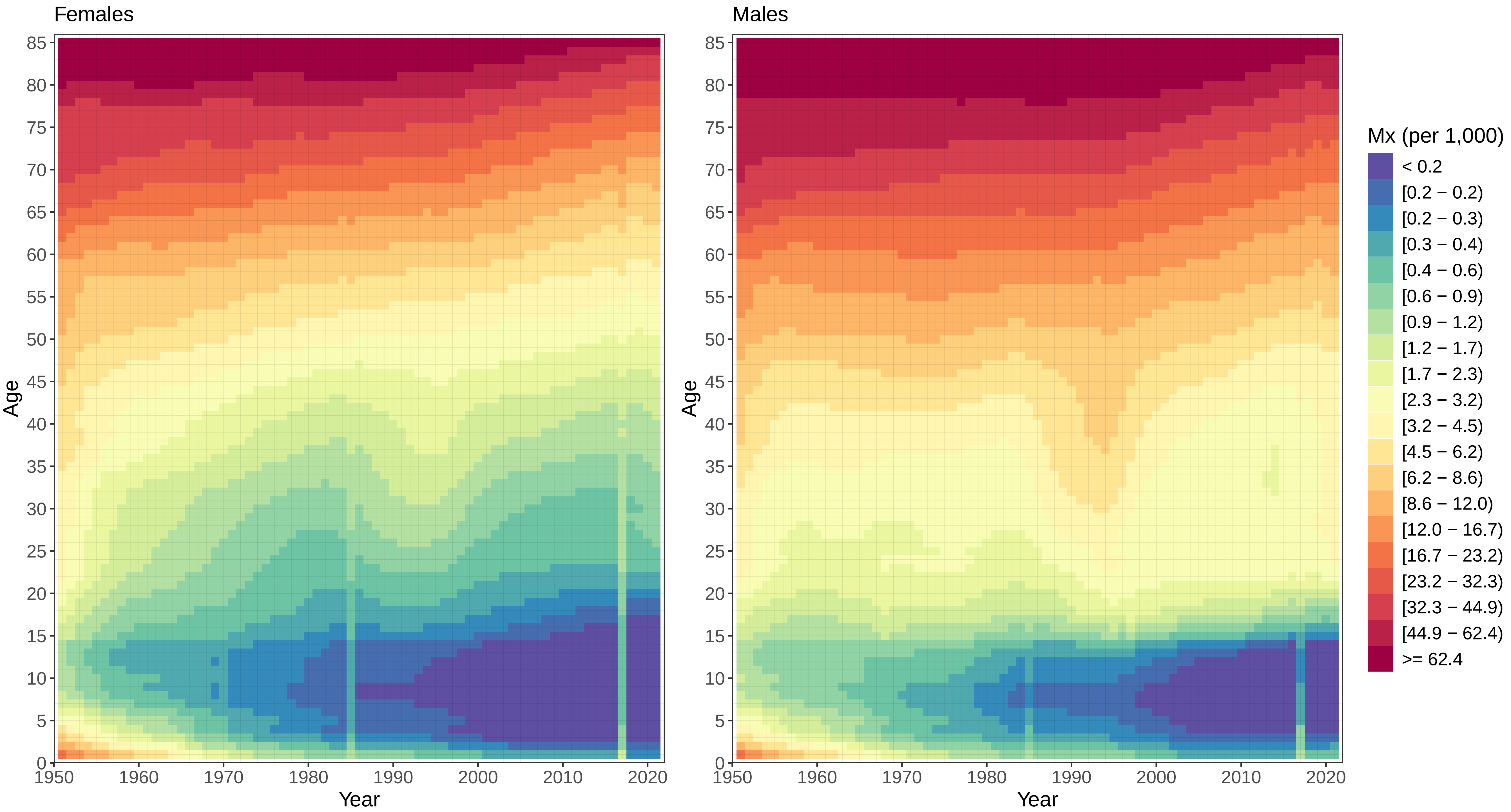

Appendix 2 Figure S4: Saint Kitts and Nevis mortality rates, 1950−2023

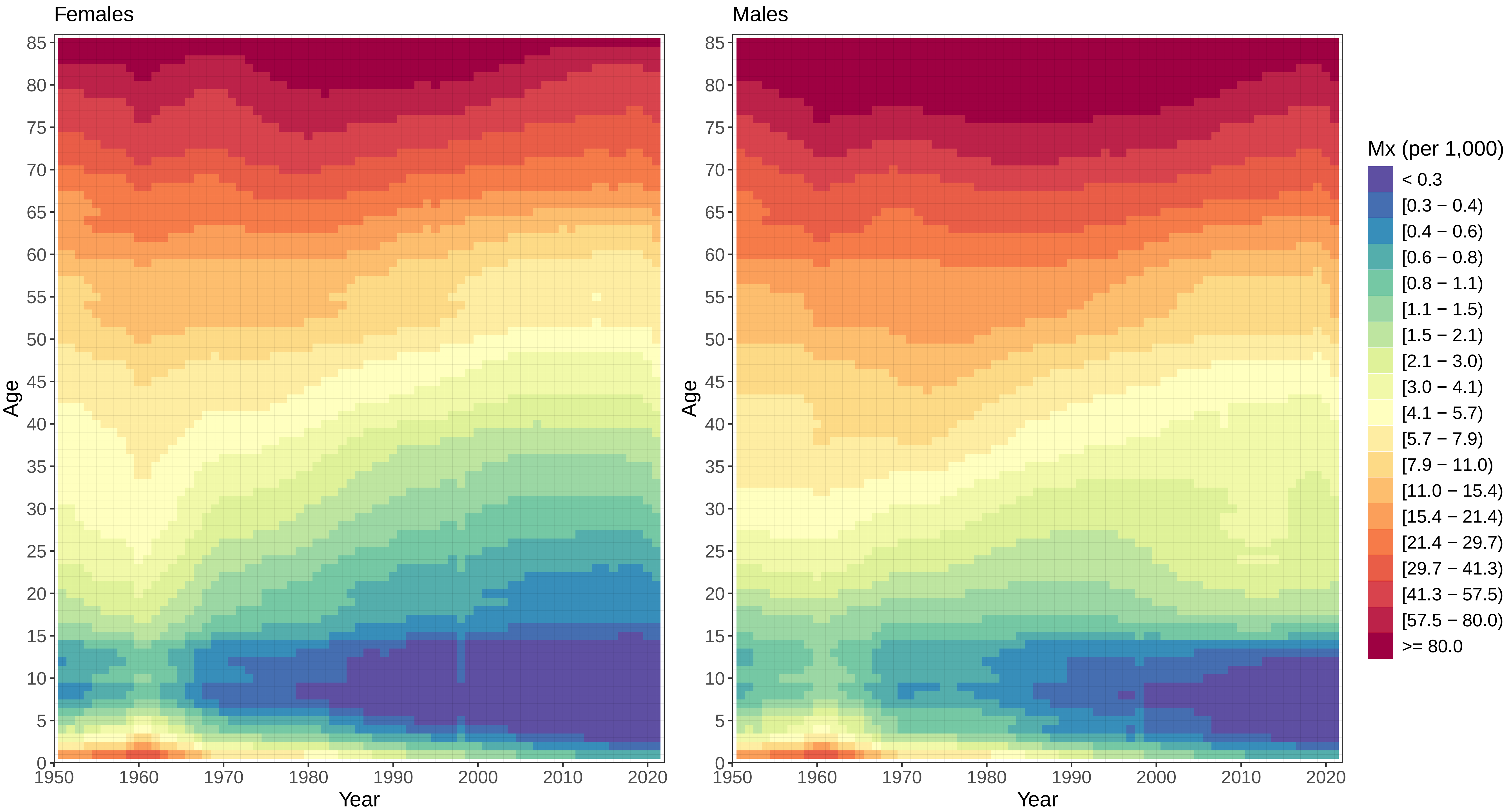

Appendix 2 Figure S4: Saint Lucia mortality rates, 1950−2023

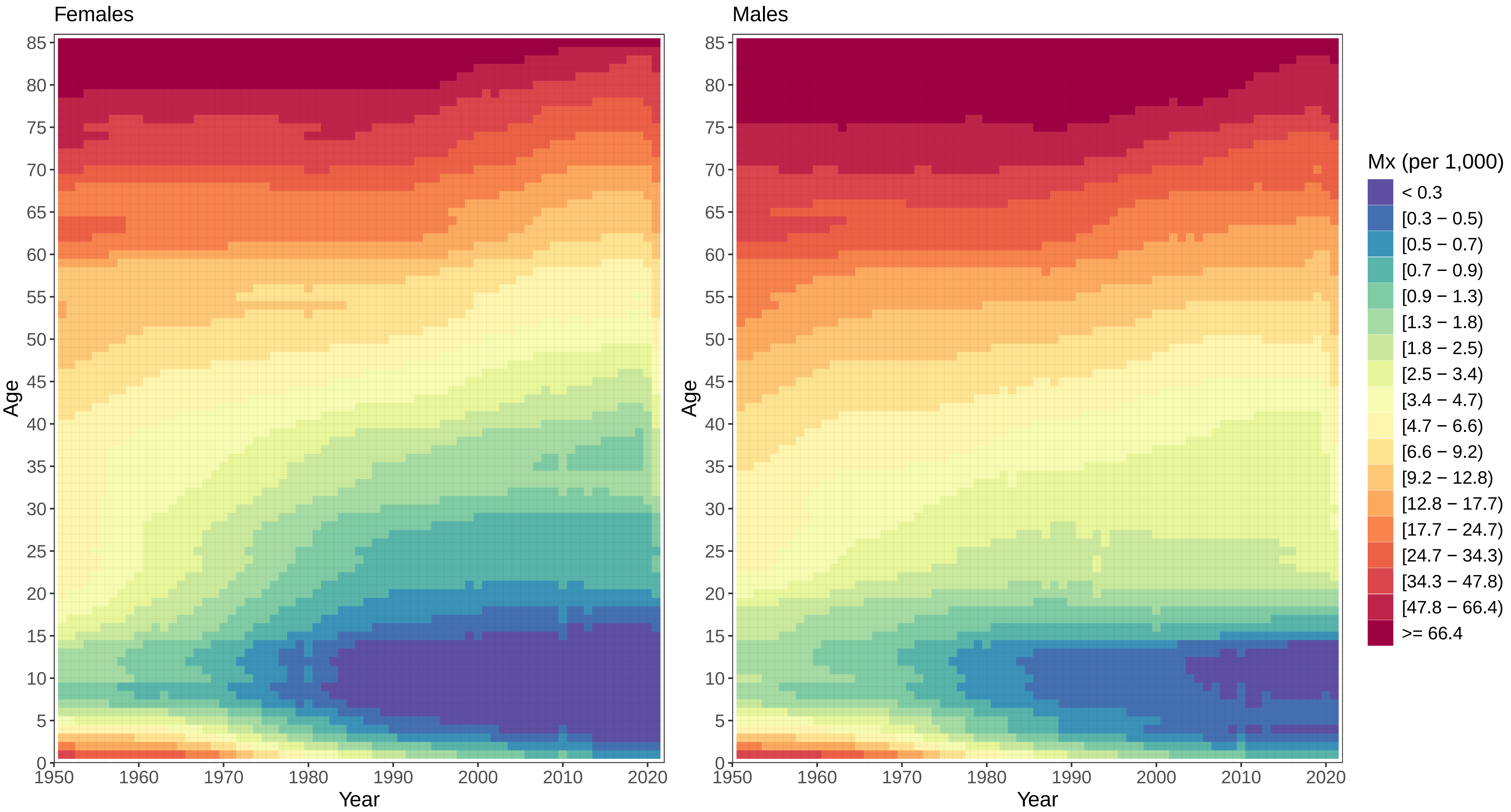

Appendix 2 Figure S4: Saint Vincent and the Grenadines mortality rates, 1950−2023

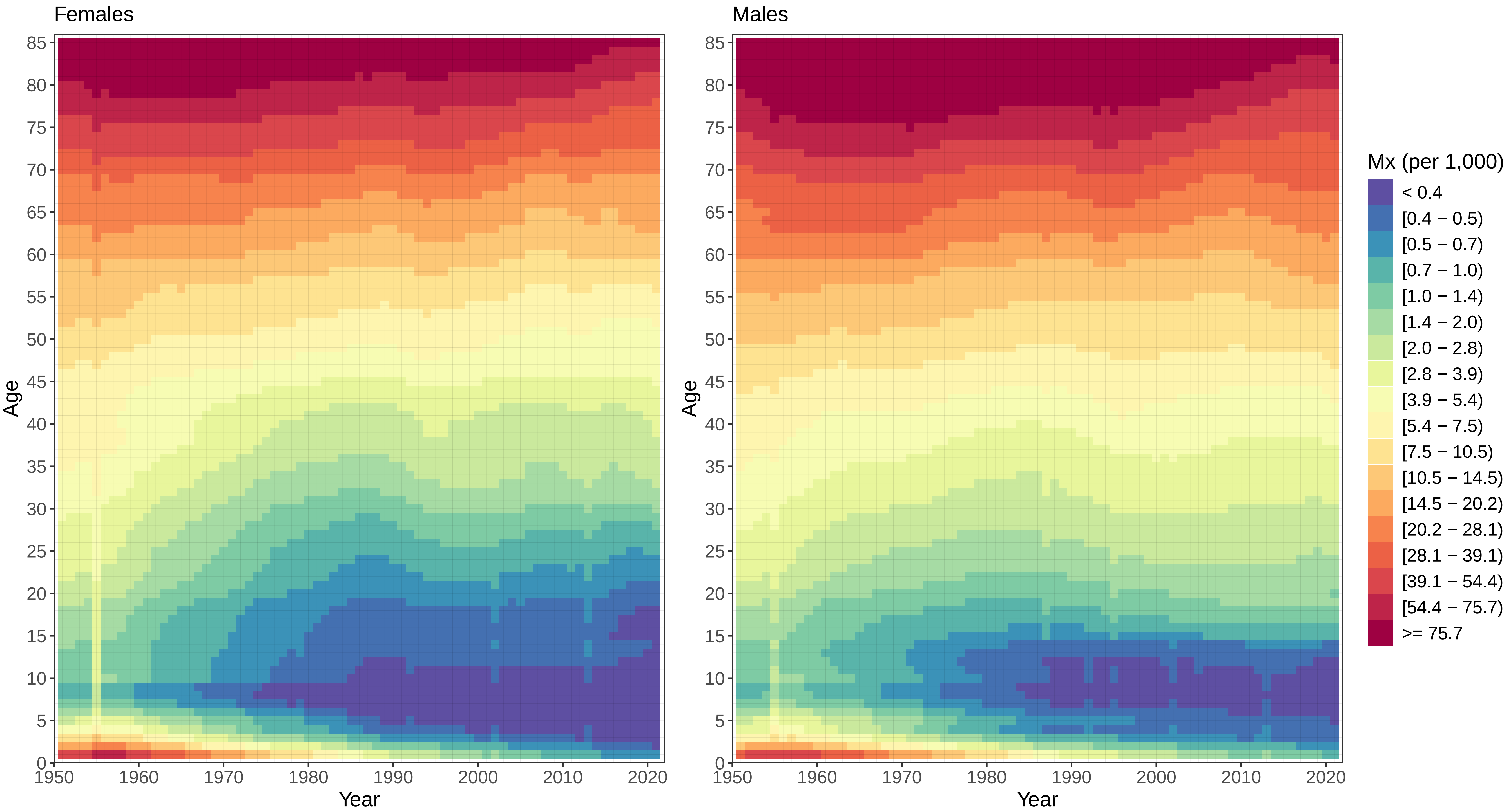

Appendix 2 Figure S4: Suriname mortality rates, 1950−2023

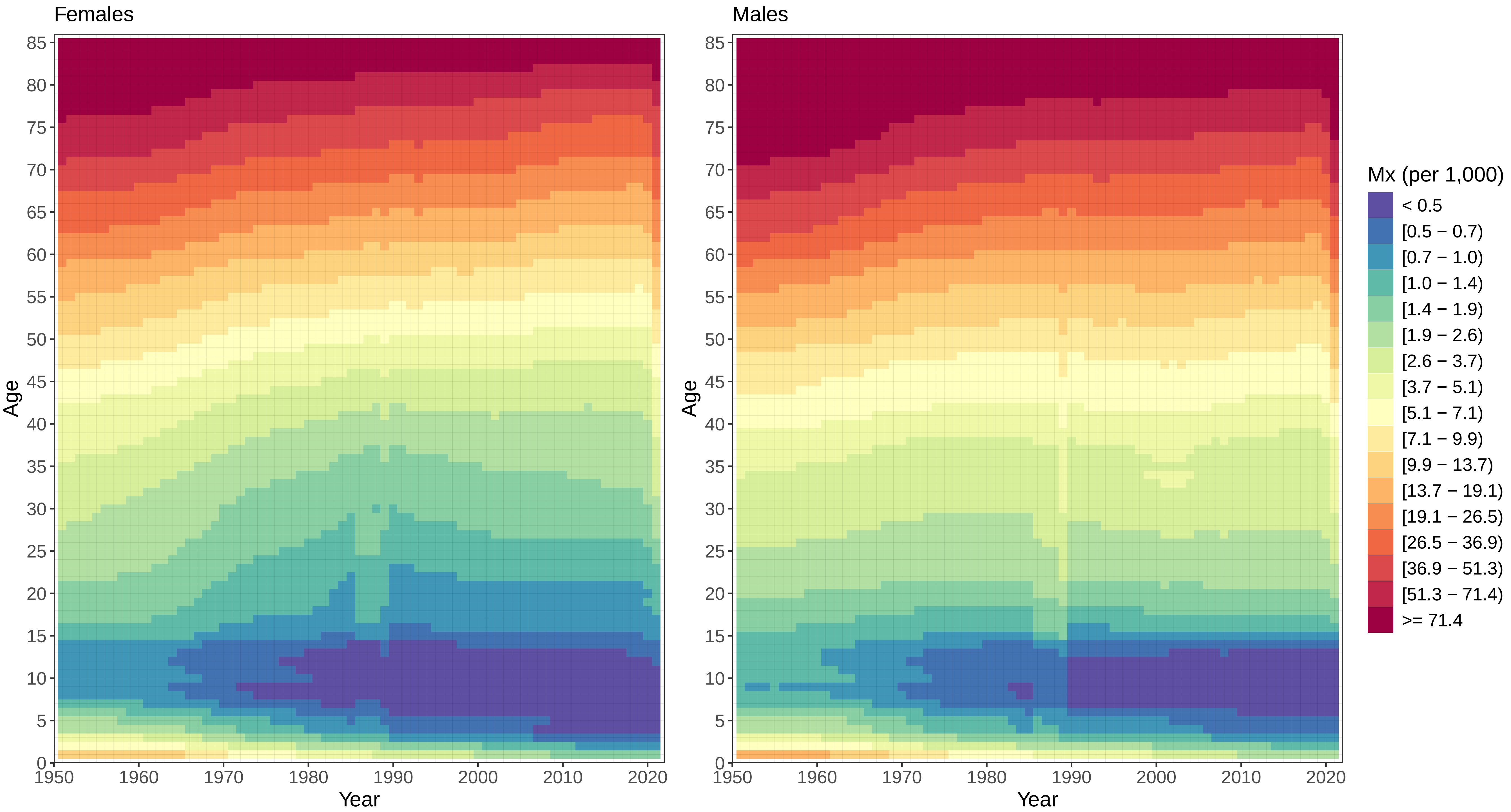

Appendix 2 Figure S4: The Bahamas mortality rates, 1950−2023

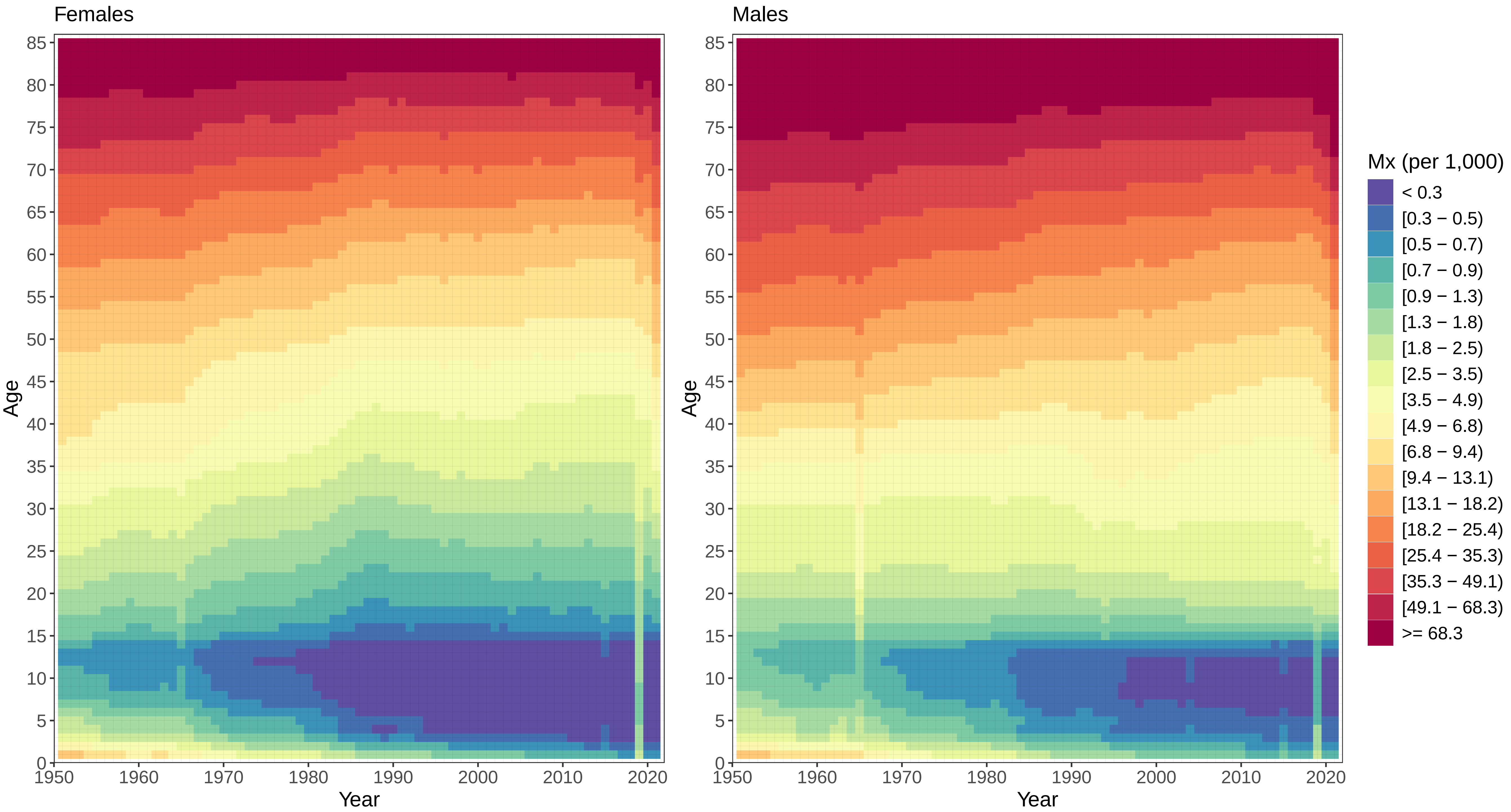

Appendix 2 Figure S4: Trinidad and Tobago mortality rates, 1950−2023

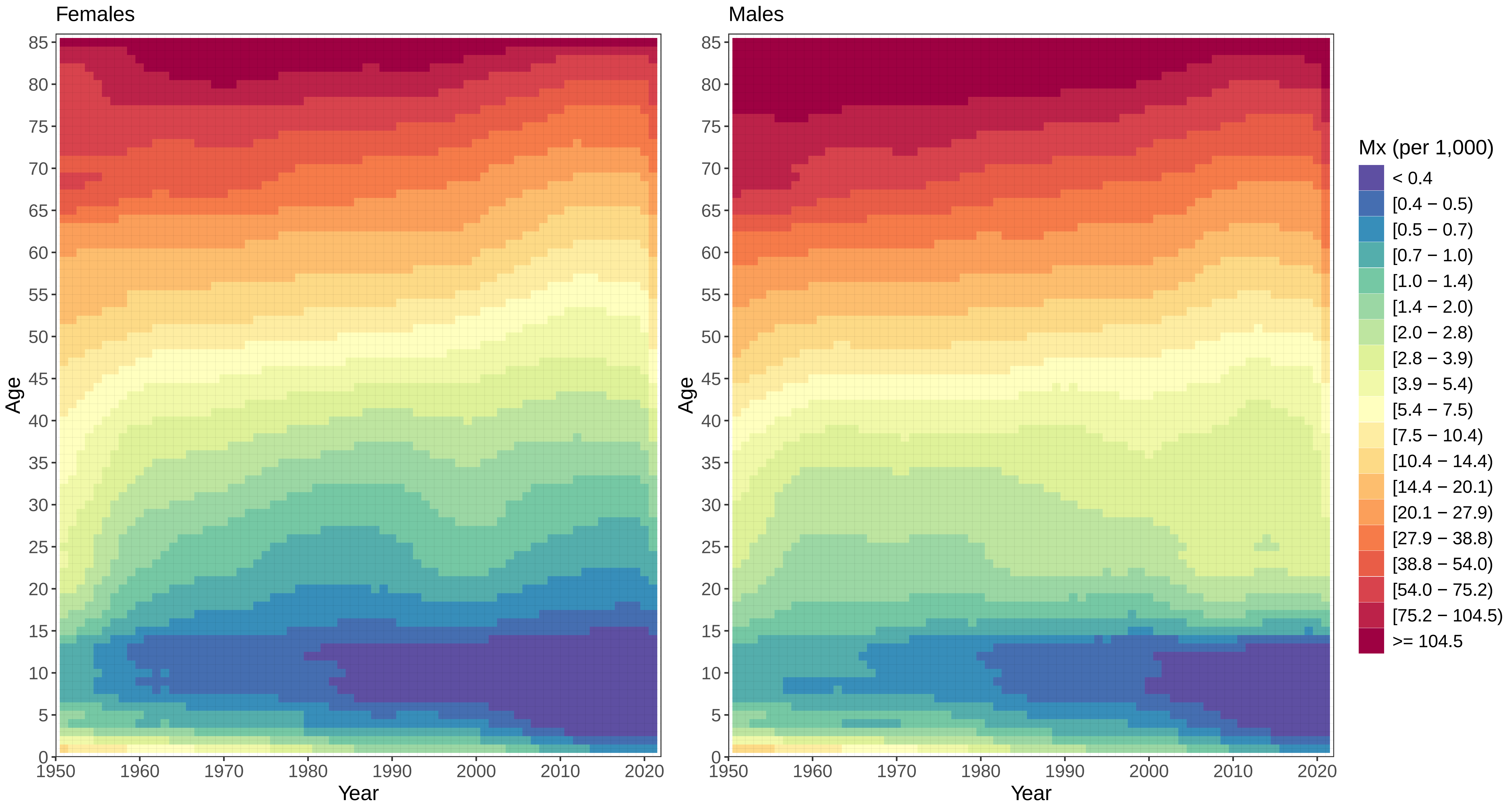

Appendix 2 Figure S4: Virgin Islands mortality rates, 1950−2023

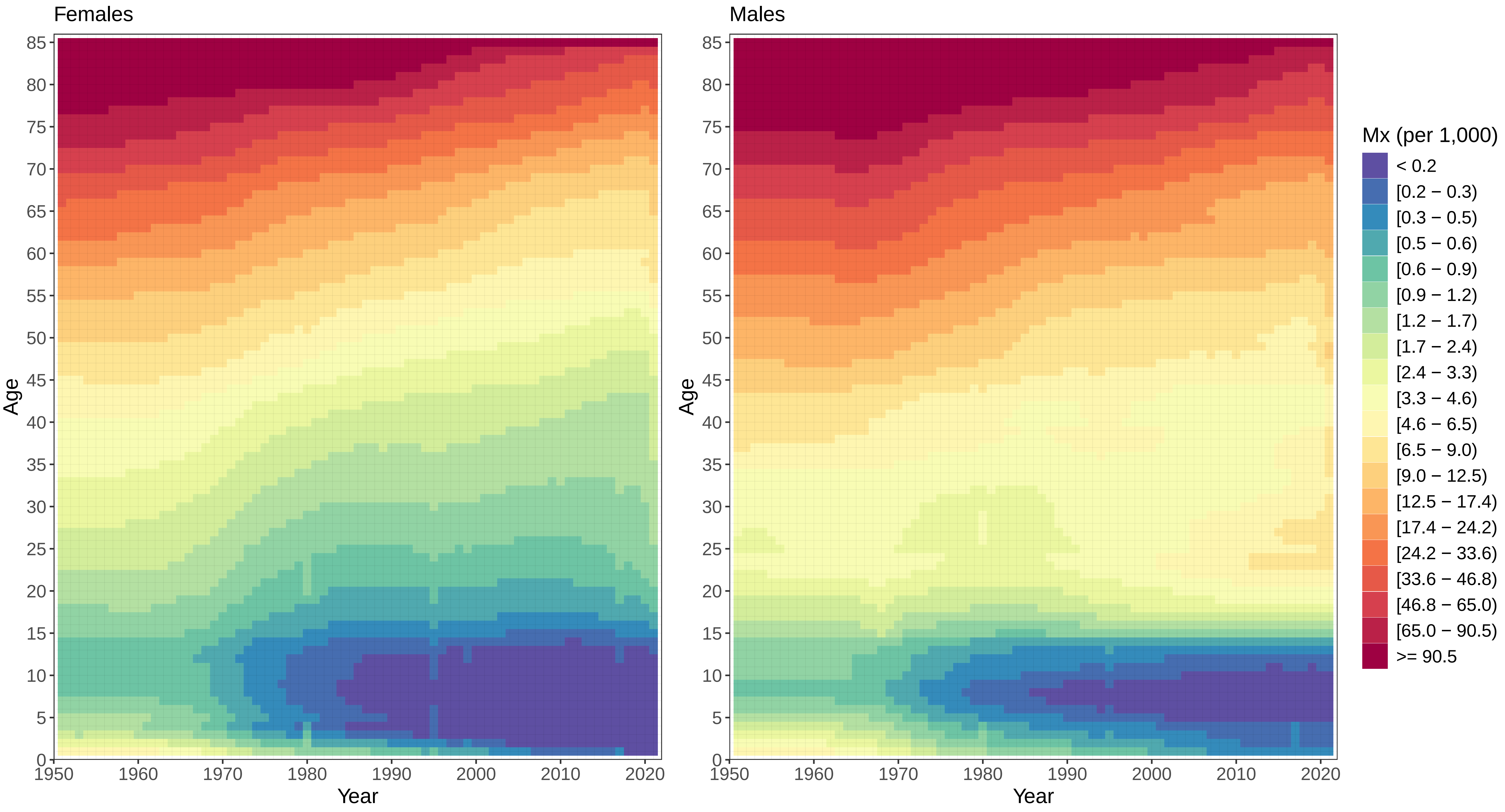

Appendix 2 Figure S4: Colombia mortality rates, 1950−2023

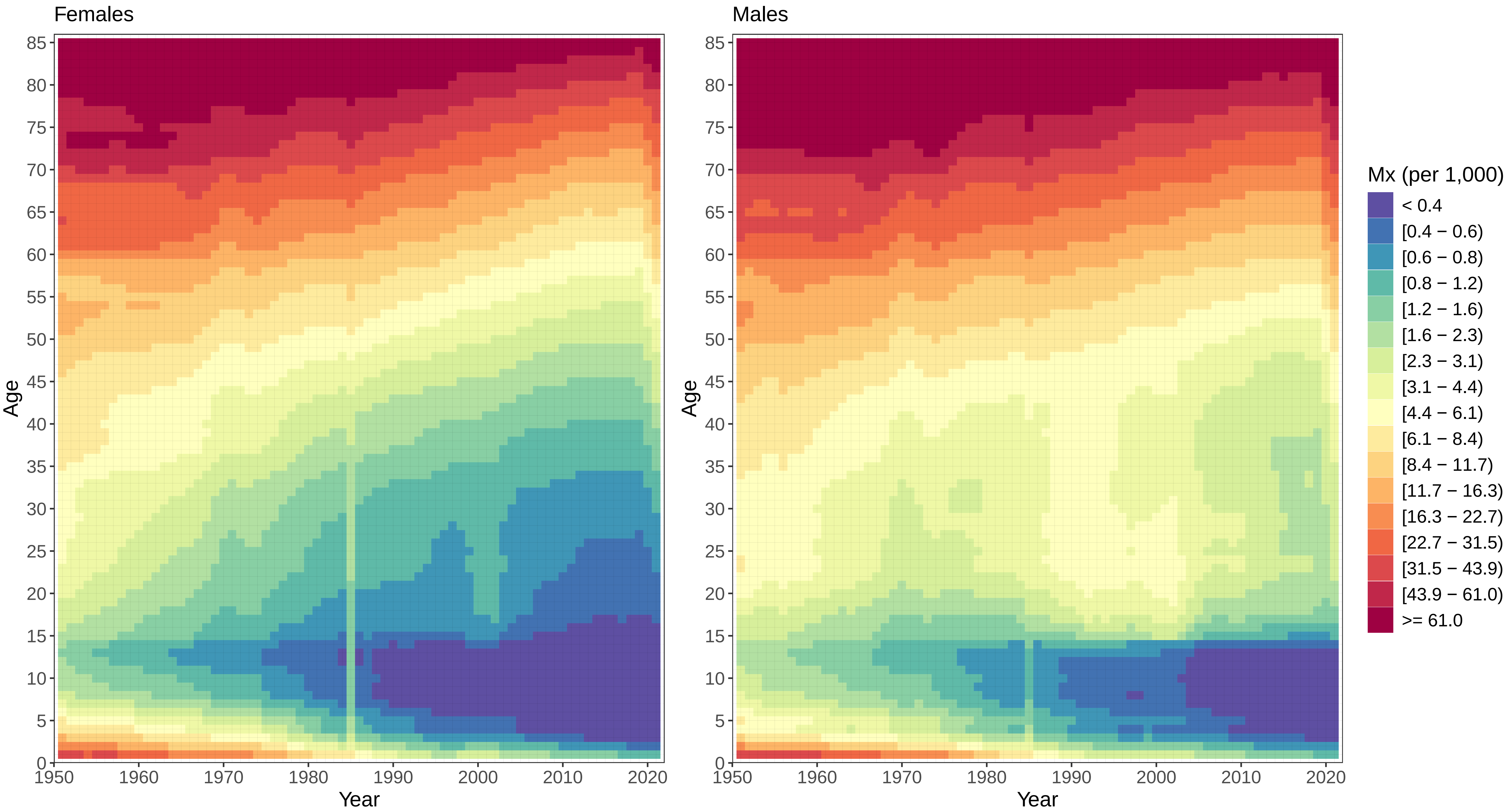

Appendix 2 Figure S4: Costa Rica mortality rates, 1950−2023

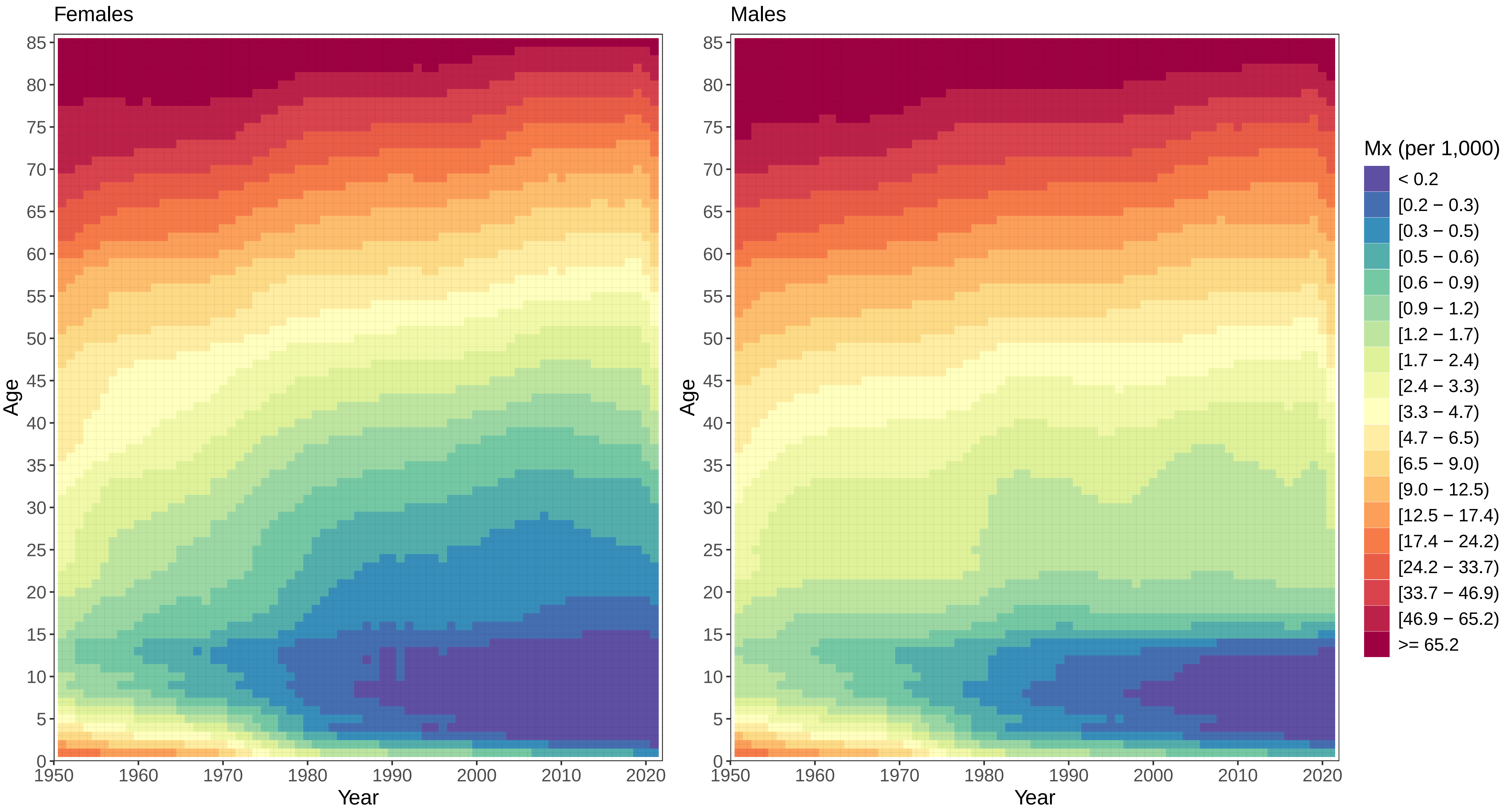

Appendix 2 Figure S4: El Salvador mortality rates, 1950−2023

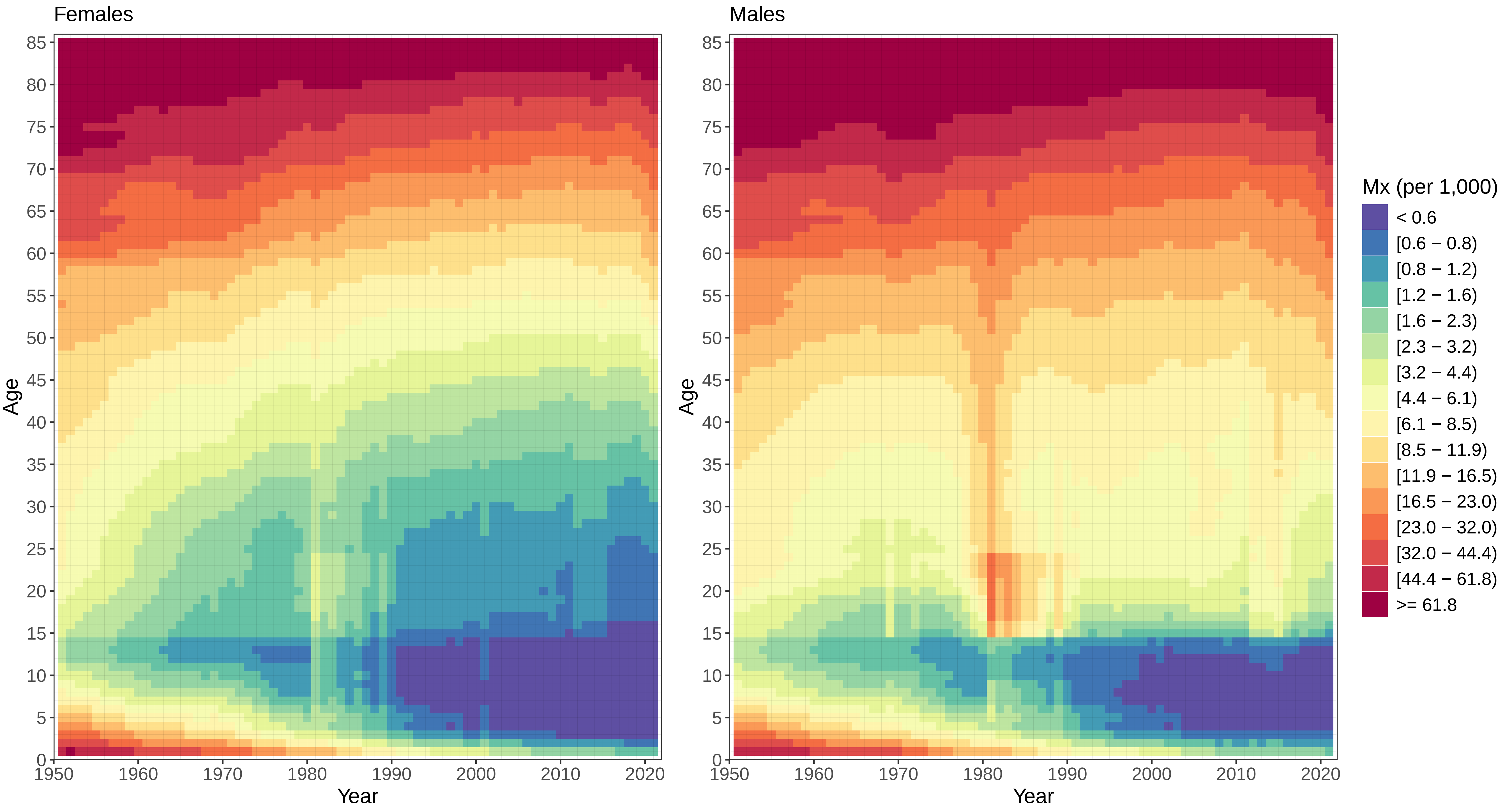

Appendix 2 Figure S4: Guatemala mortality rates, 1950−2023

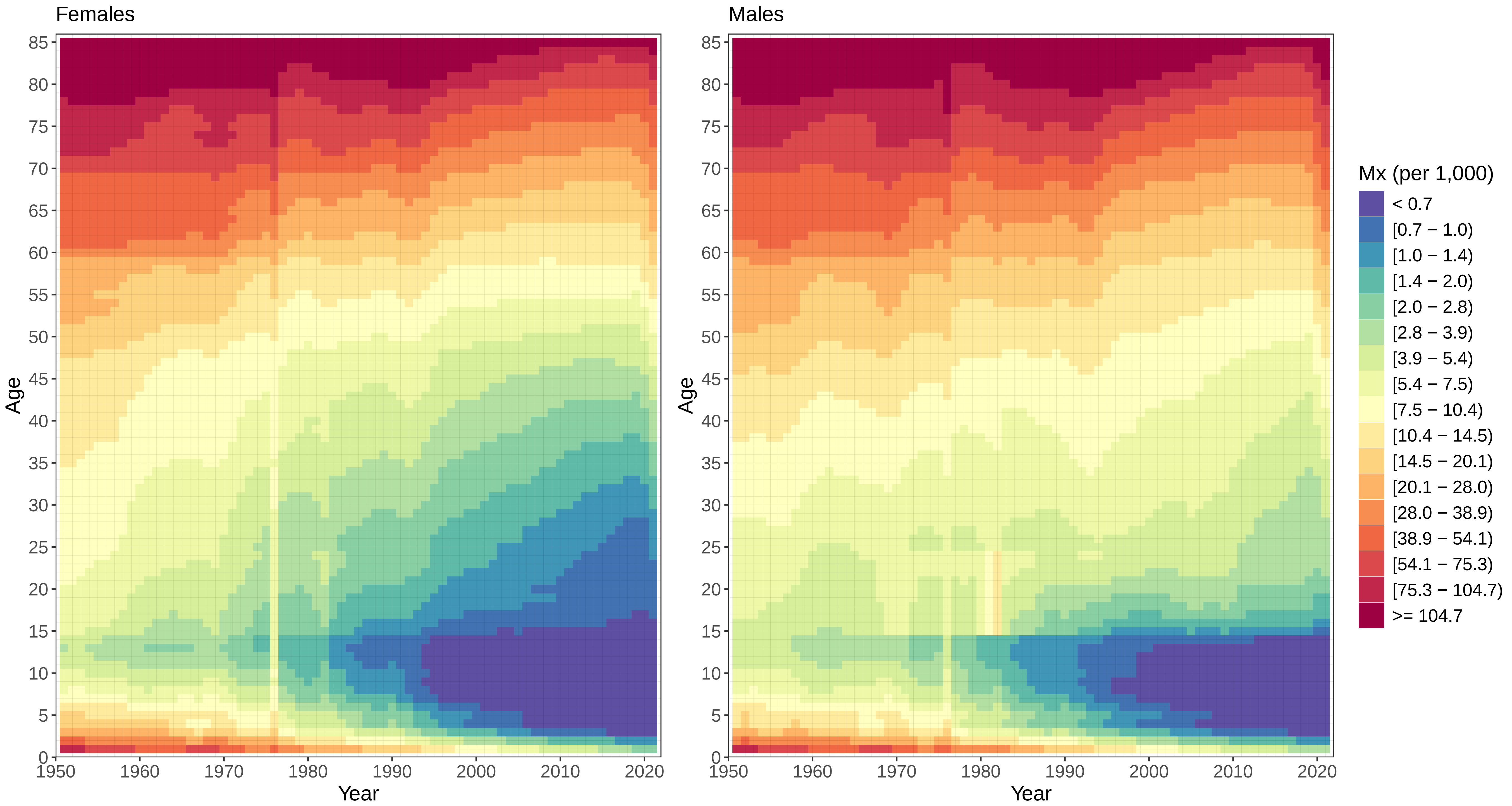

Appendix 2 Figure S4: Honduras mortality rates, 1950−2023

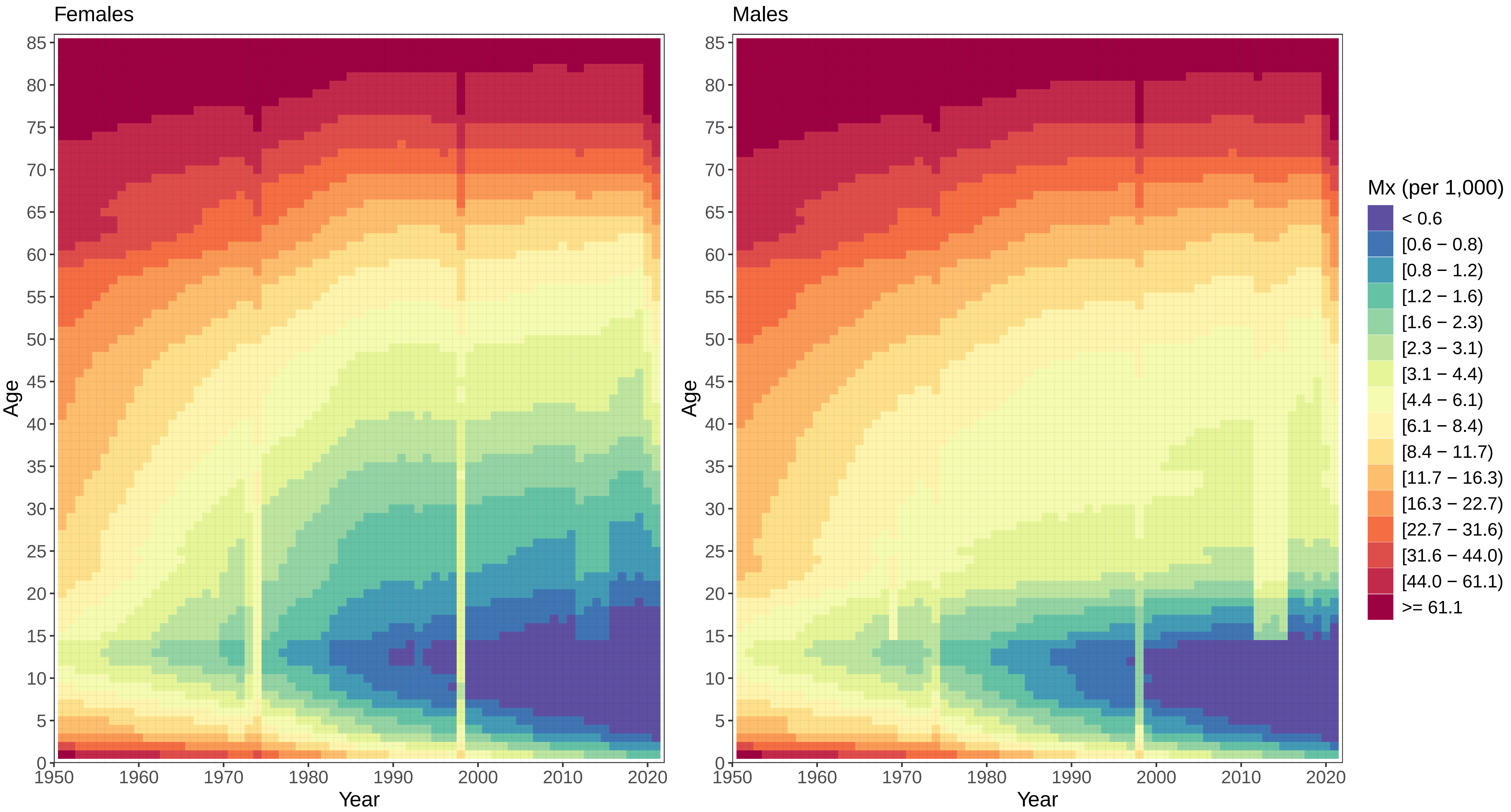

Appendix 2 Figure S4: Mexico mortality rates, 1950−2023

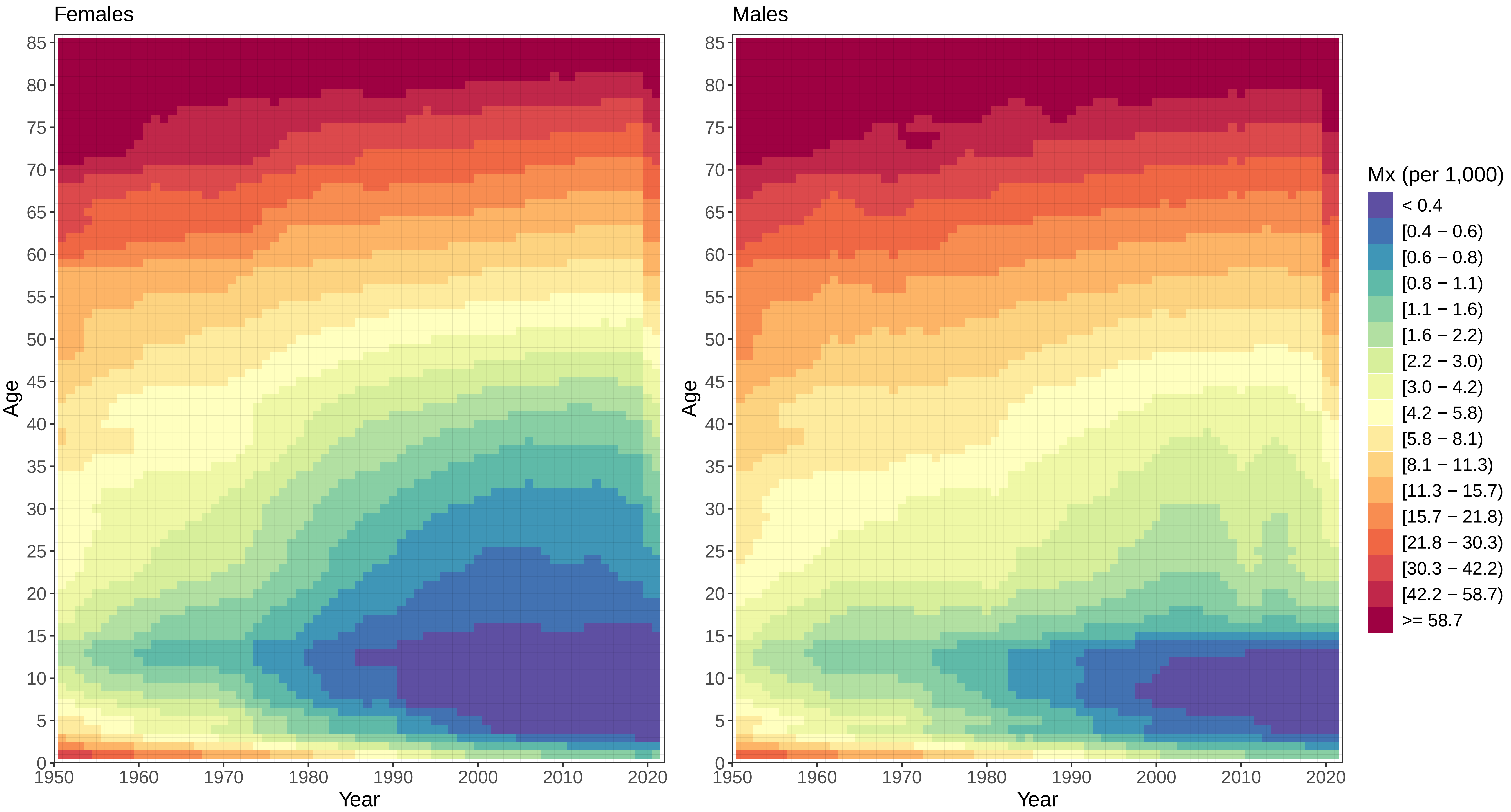

Appendix 2 Figure S4: Nicaragua mortality rates, 1950−2023

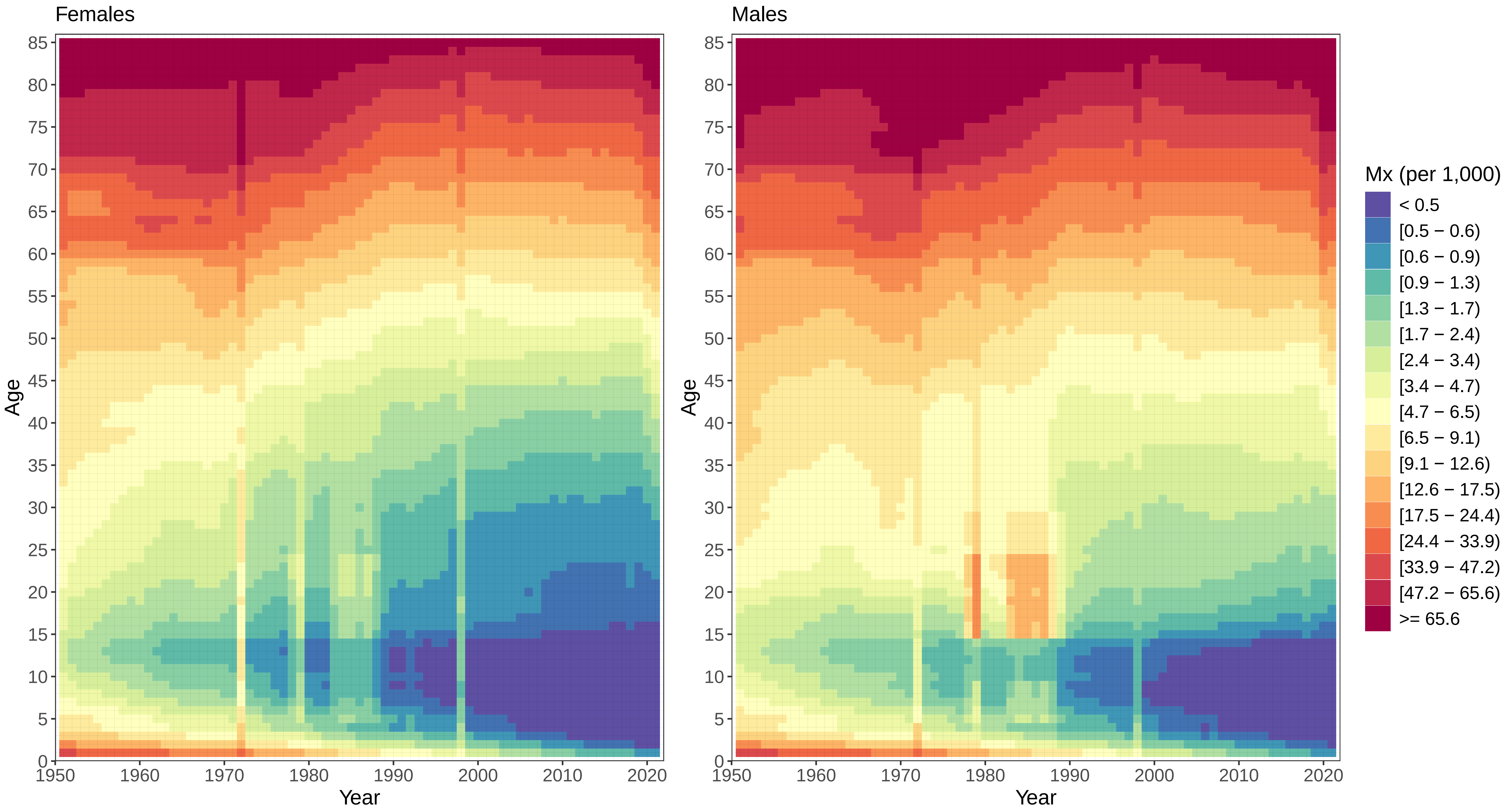

Appendix 2 Figure S4: Panama mortality rates, 1950−2023

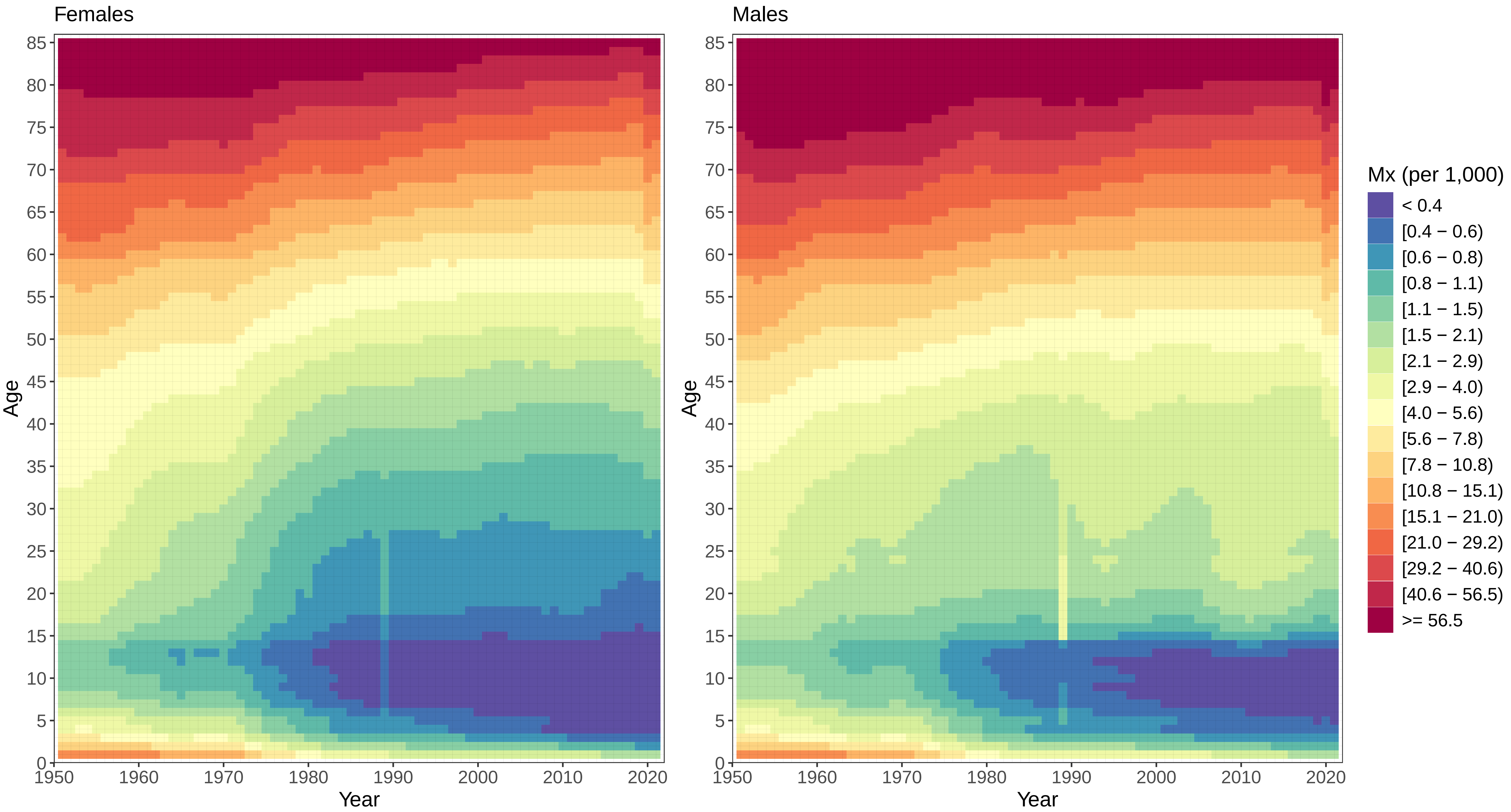

Appendix 2 Figure S4: Venezuela mortality rates, 1950−2023

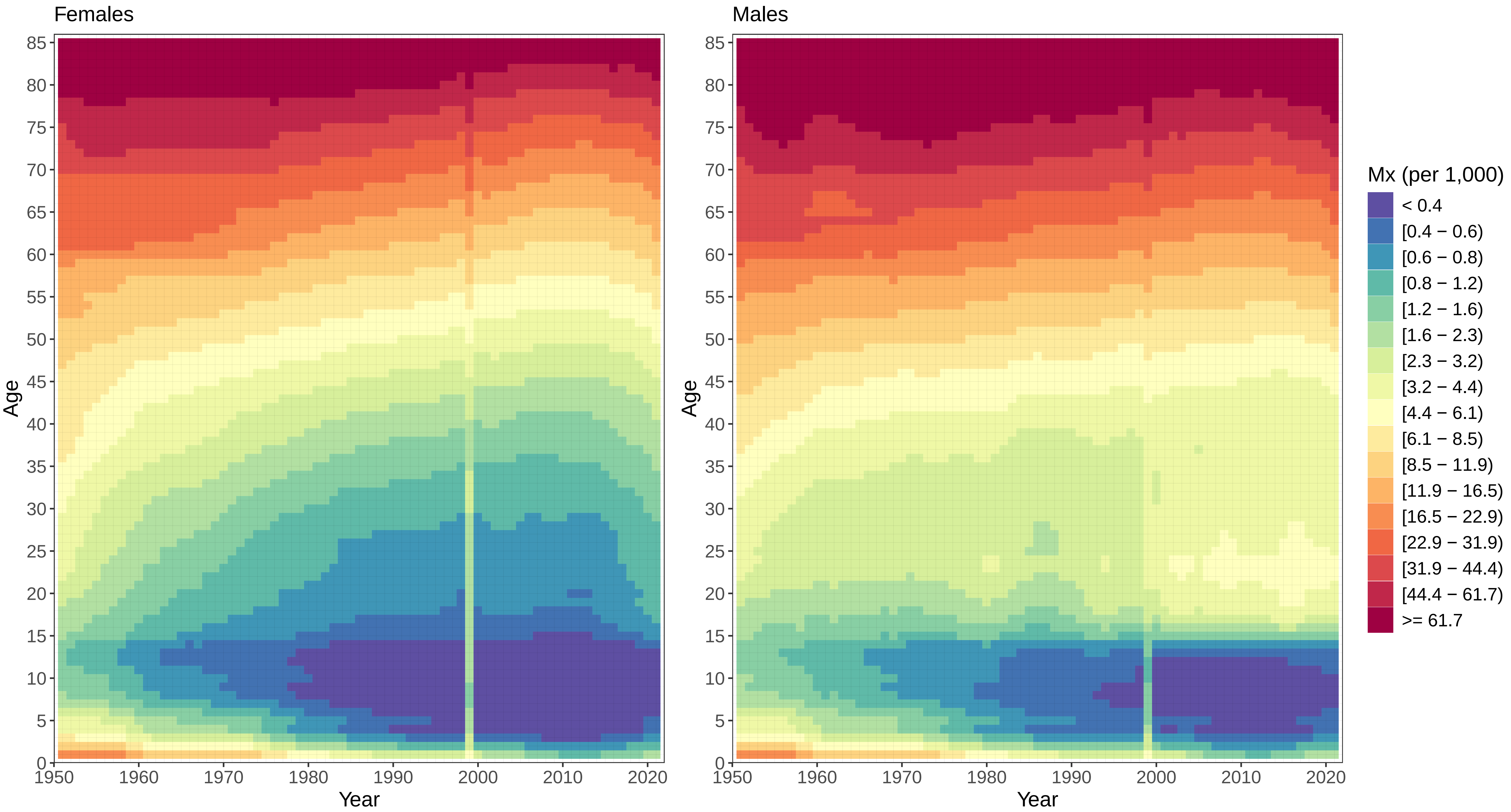

Appendix 2 Figure S4: Brazil mortality rates, 1950−2023

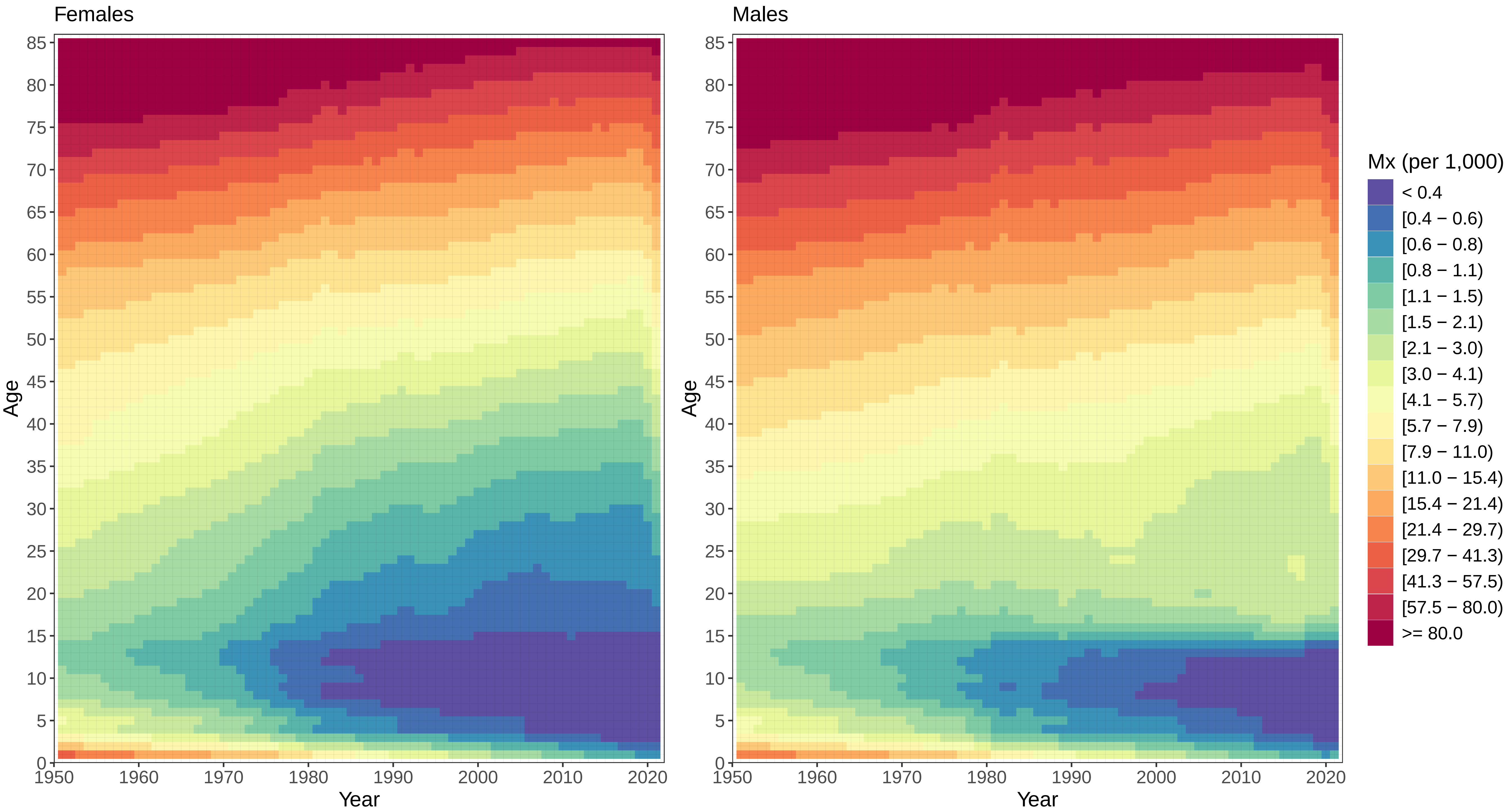

Appendix 2 Figure S4: Paraguay mortality rates, 1950−2023

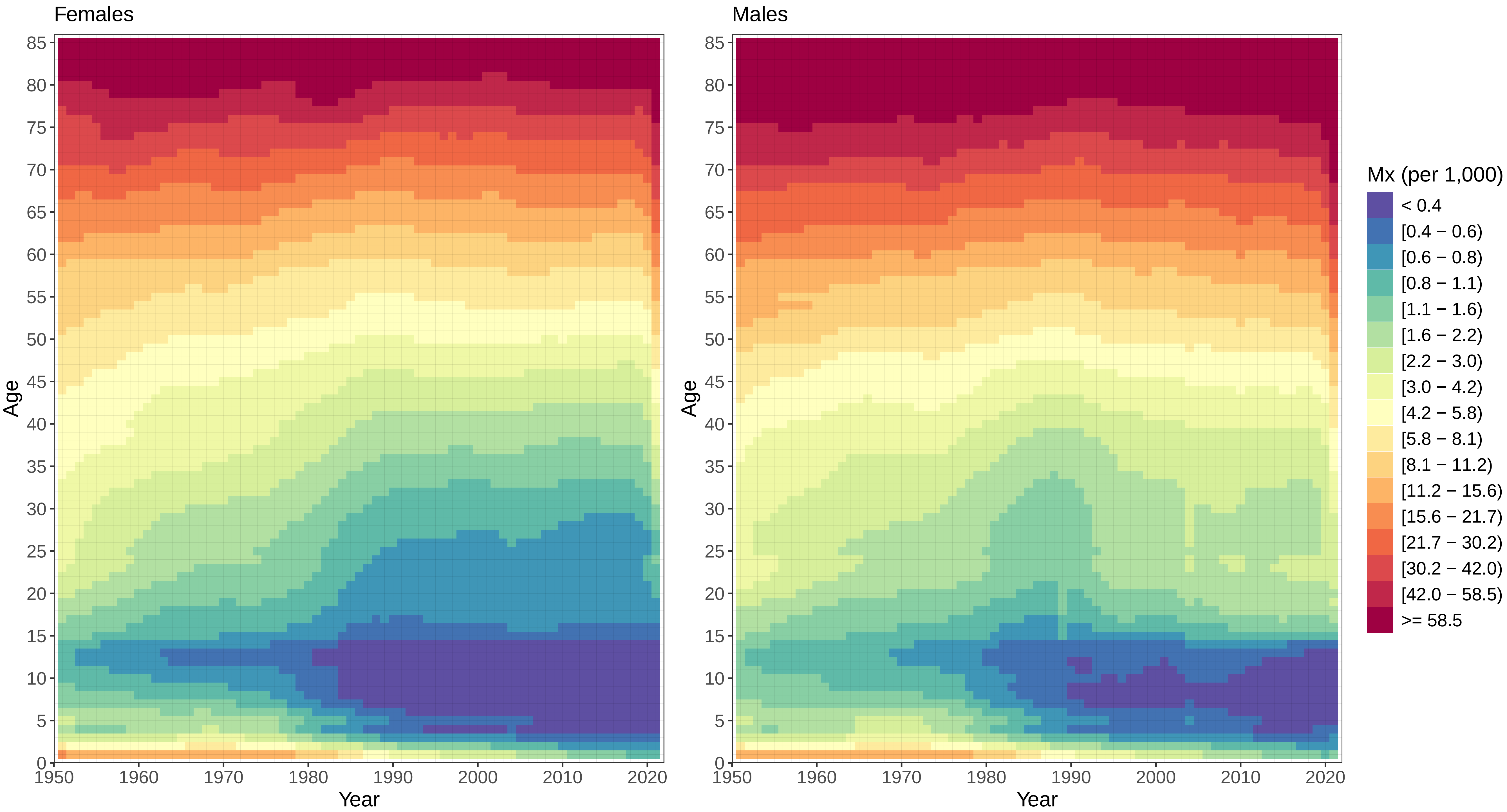

Appendix 2 Figure S4: Afghanistan mortality rates, 1950−2023

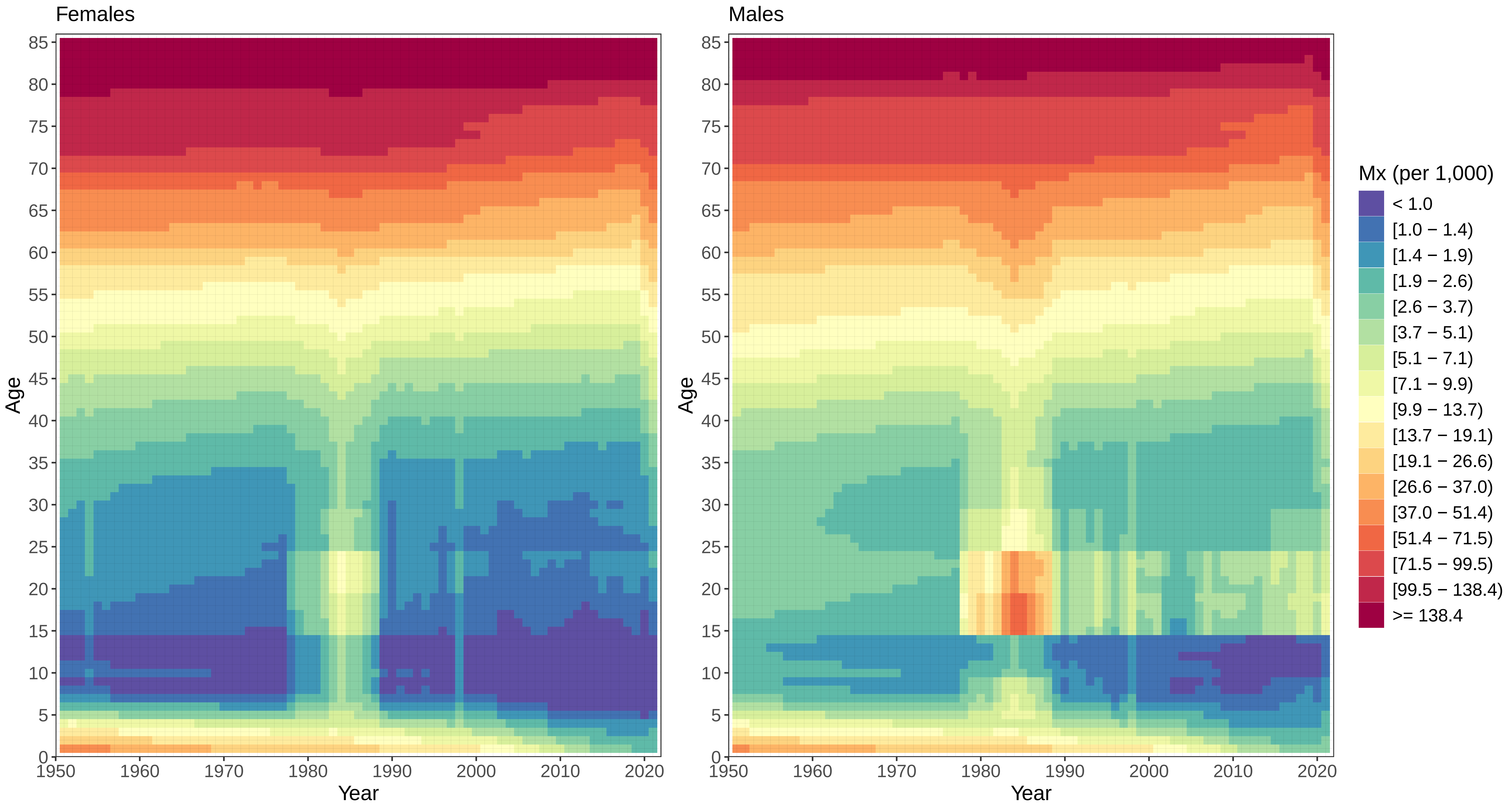

Appendix 2 Figure S4: Algeria mortality rates, 1950−2023

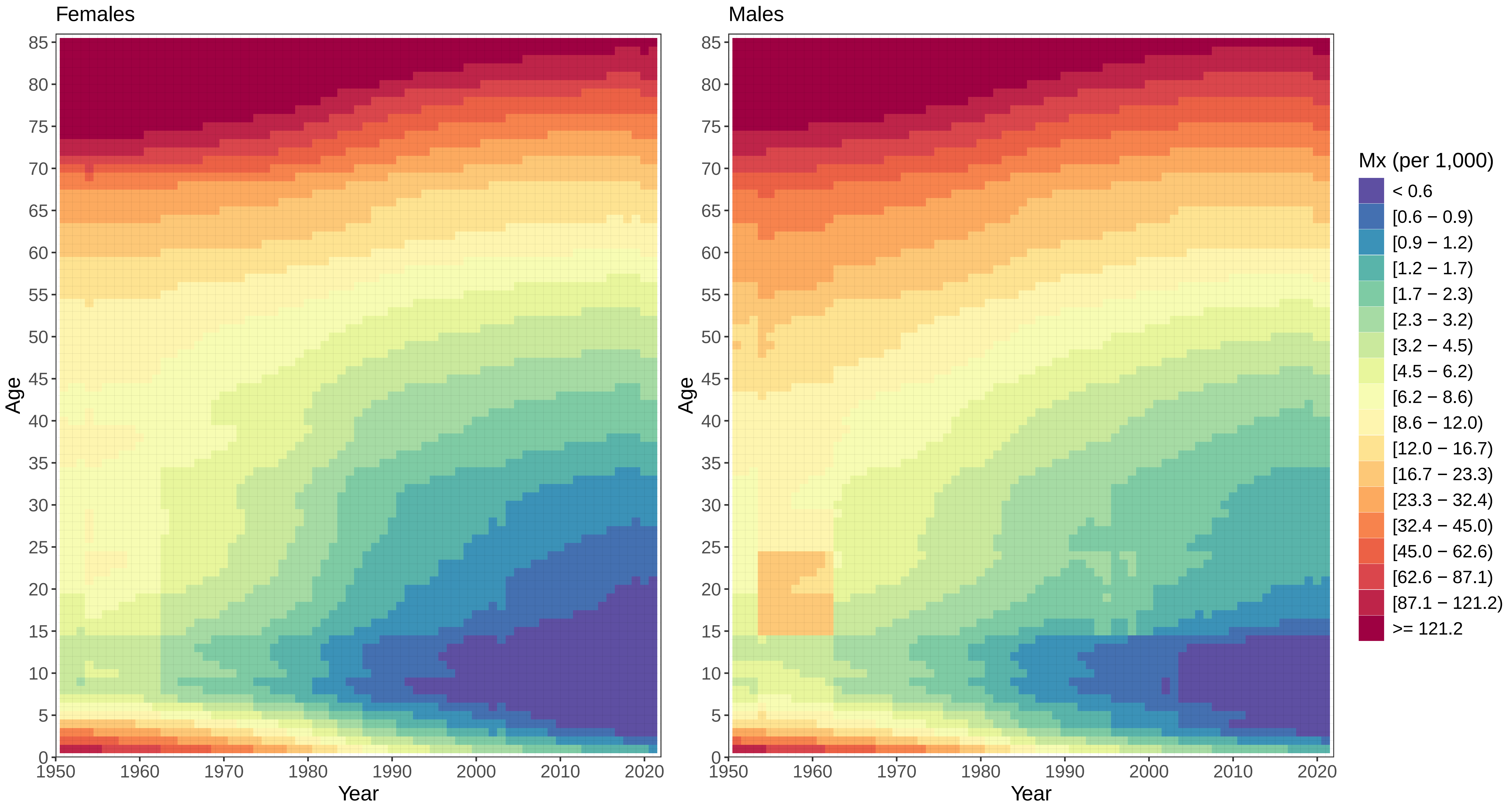

Appendix 2 Figure S4: Bahrain mortality rates, 1950−2023

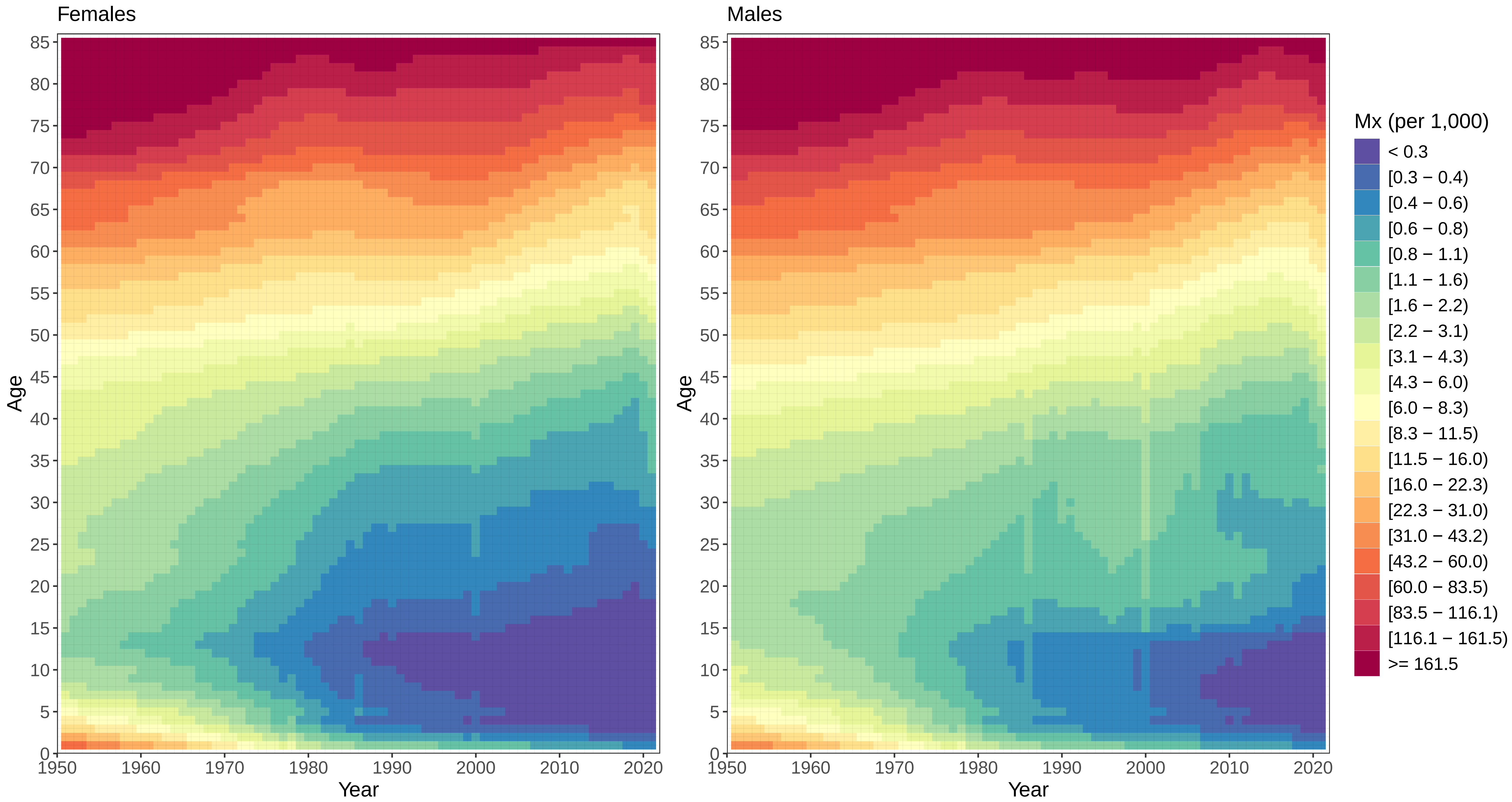

Appendix 2 Figure S4: Egypt mortality rates, 1950−2023

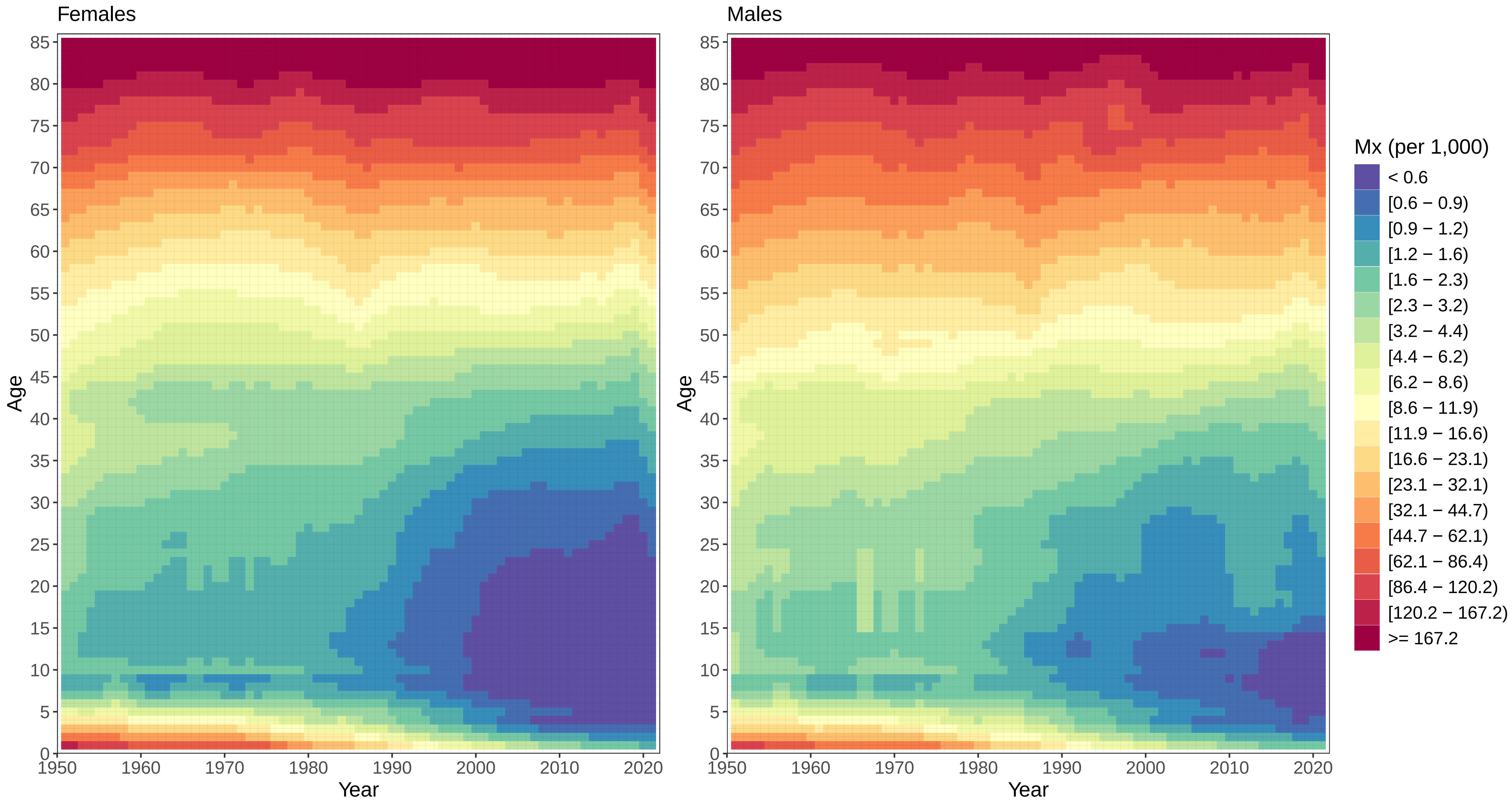

Appendix 2 Figure S4: Iran mortality rates, 1950−2023

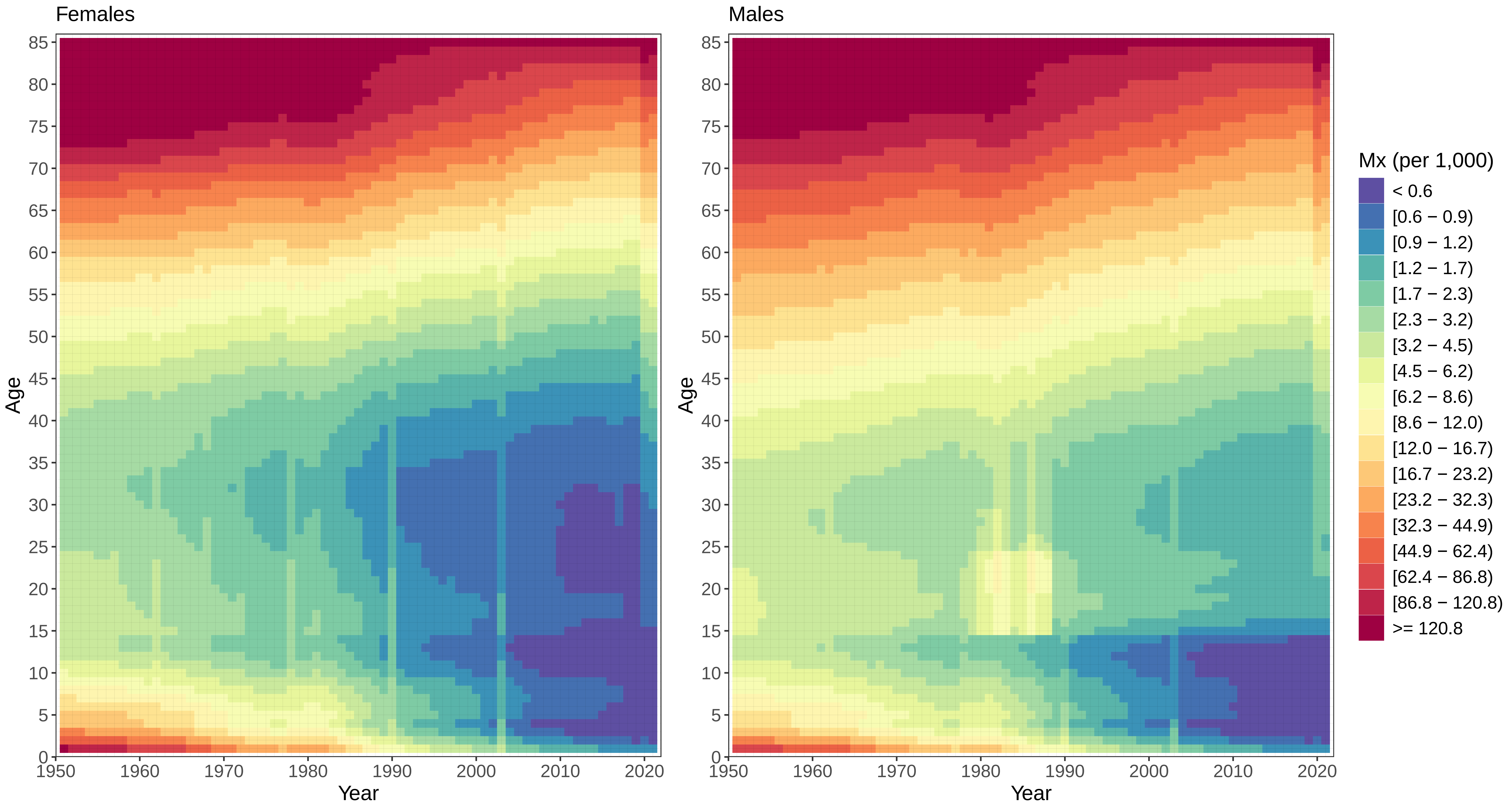

Appendix 2 Figure S4: Iraq mortality rates, 1950−2023

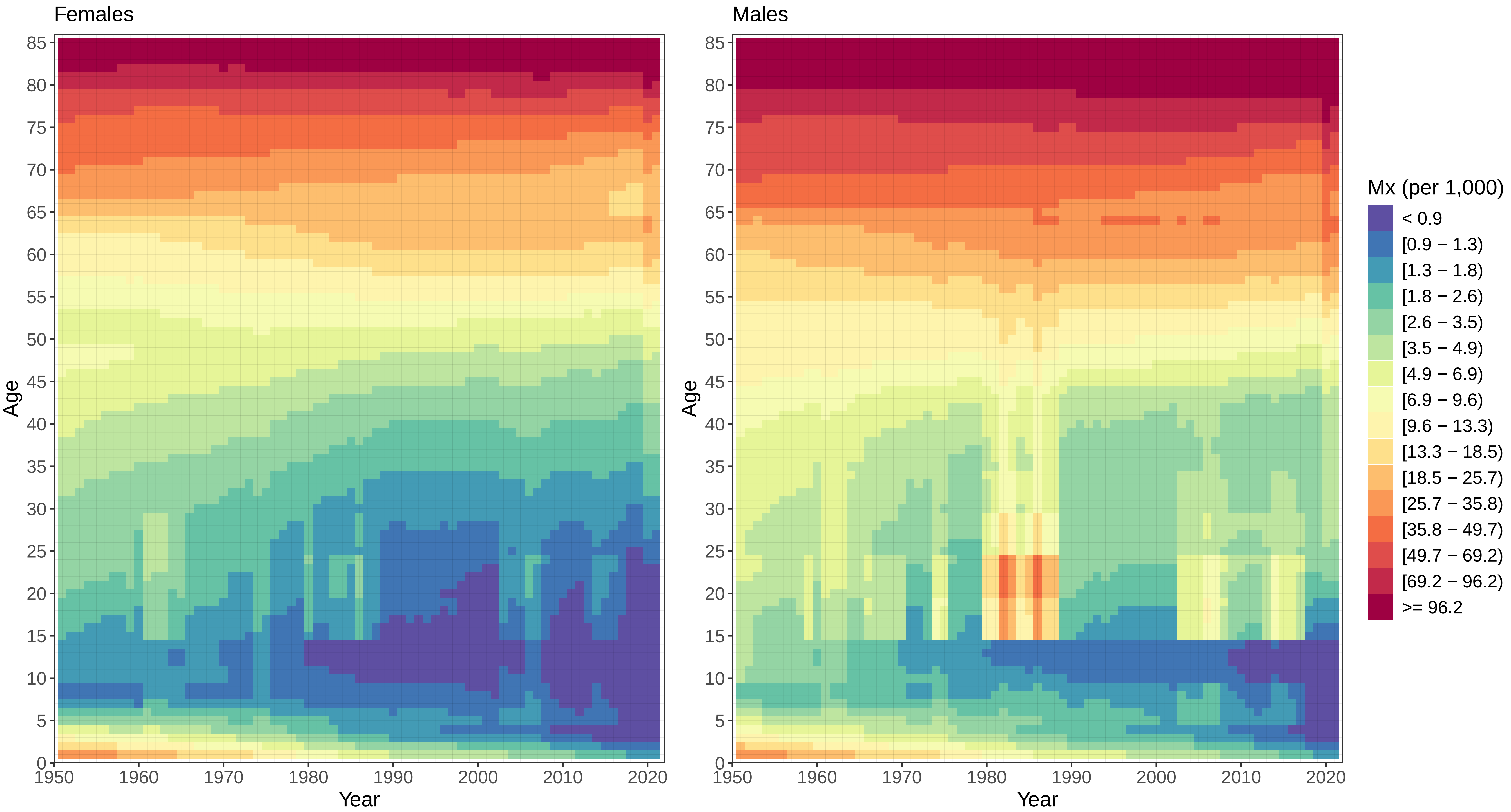

Appendix 2 Figure S4: Jordan mortality rates, 1950−2023

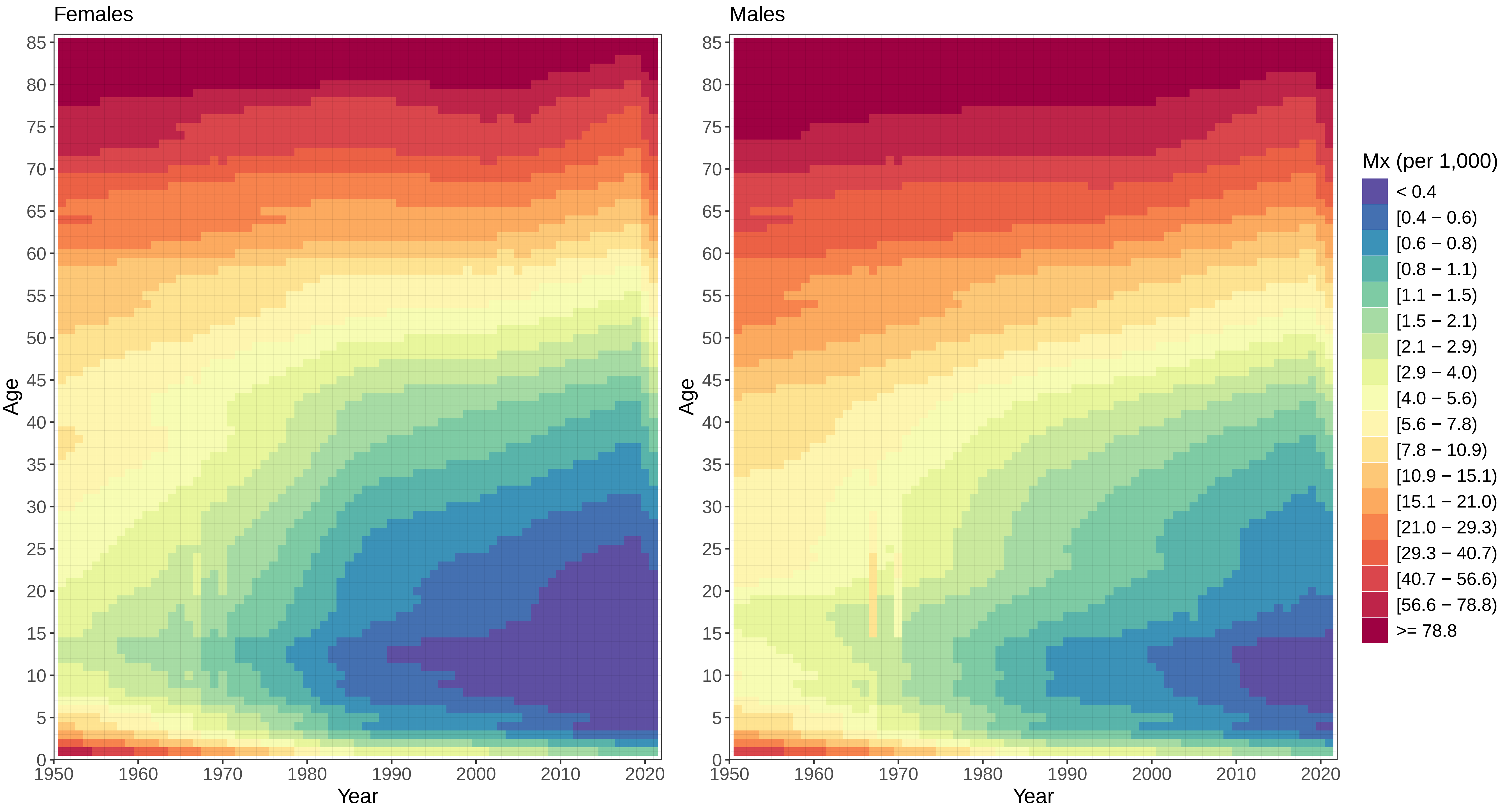

Appendix 2 Figure S4: Kuwait mortality rates, 1950−2023

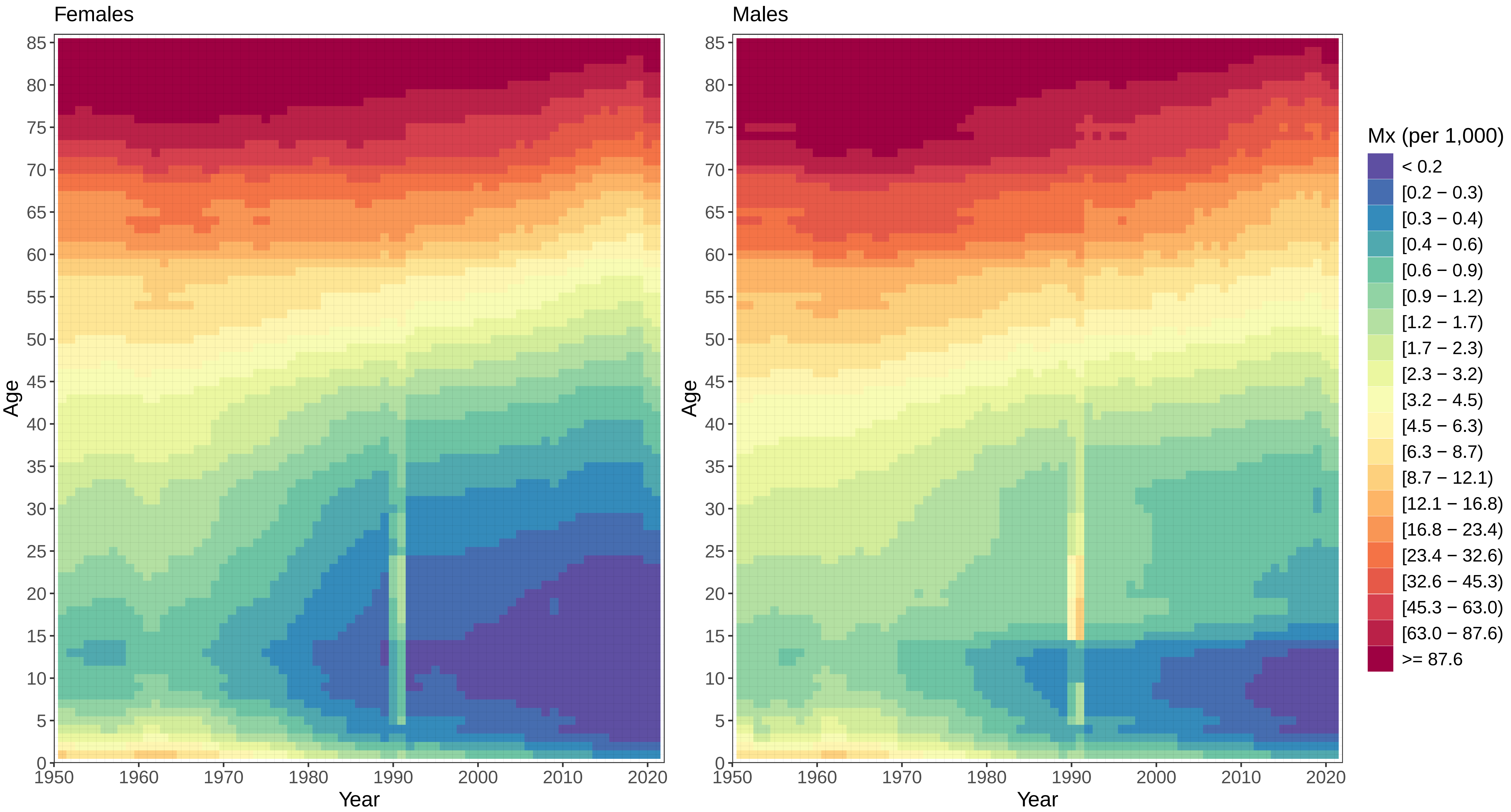

Appendix 2 Figure S4: Lebanon mortality rates, 1950−2023

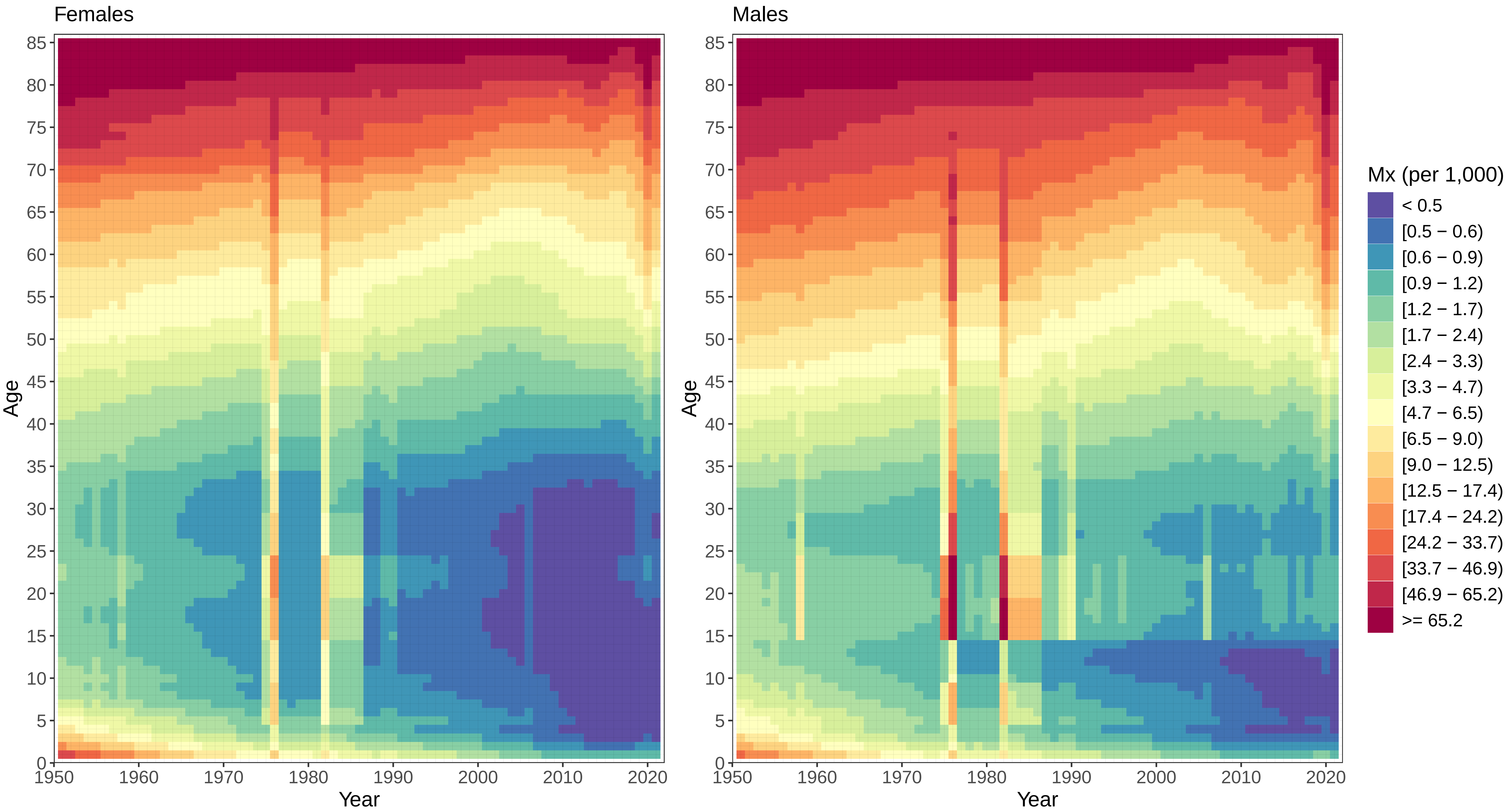

Appendix 2 Figure S4: Libya mortality rates, 1950−2023

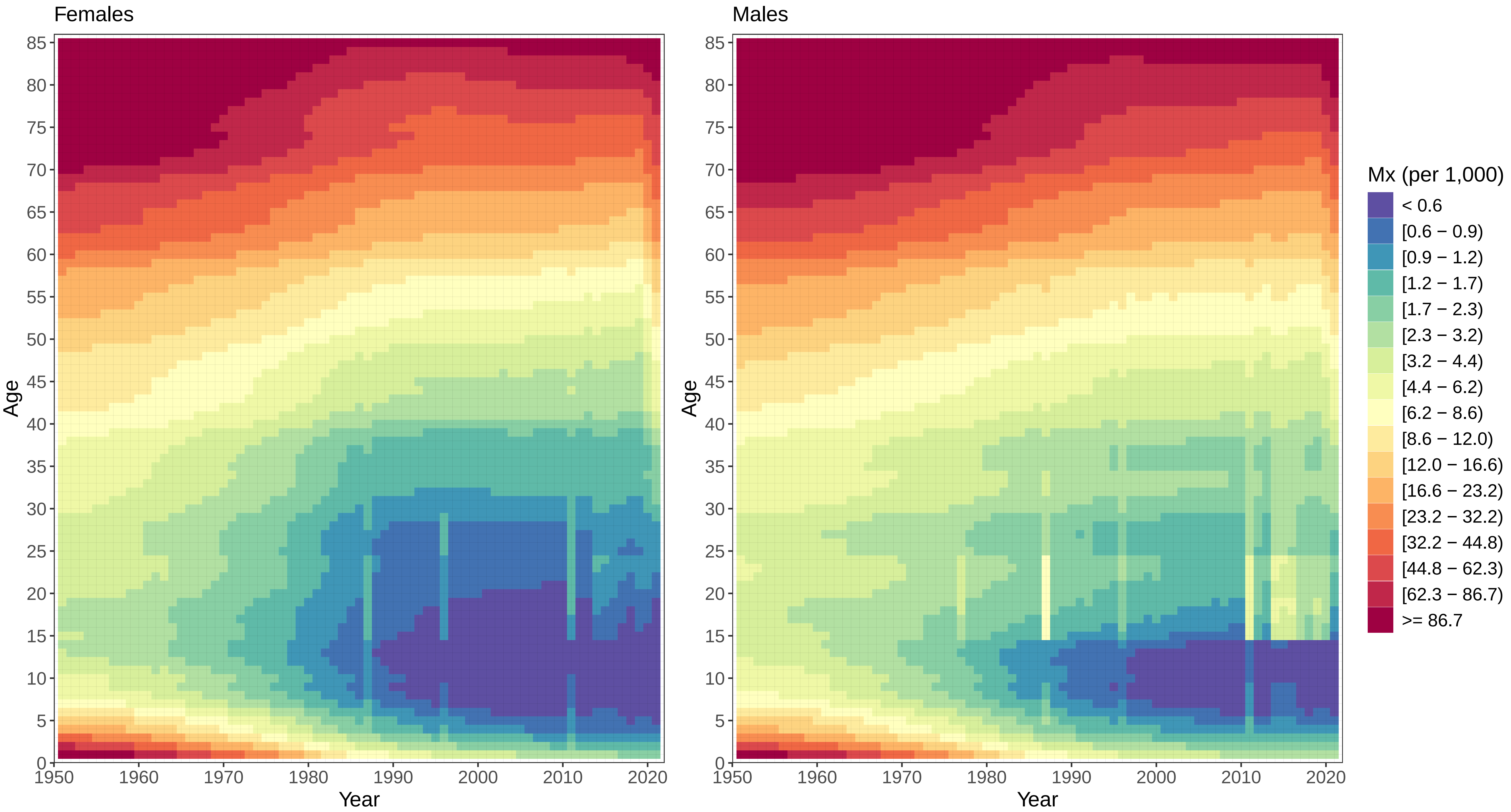

Appendix 2 Figure S4: Morocco mortality rates, 1950−2023

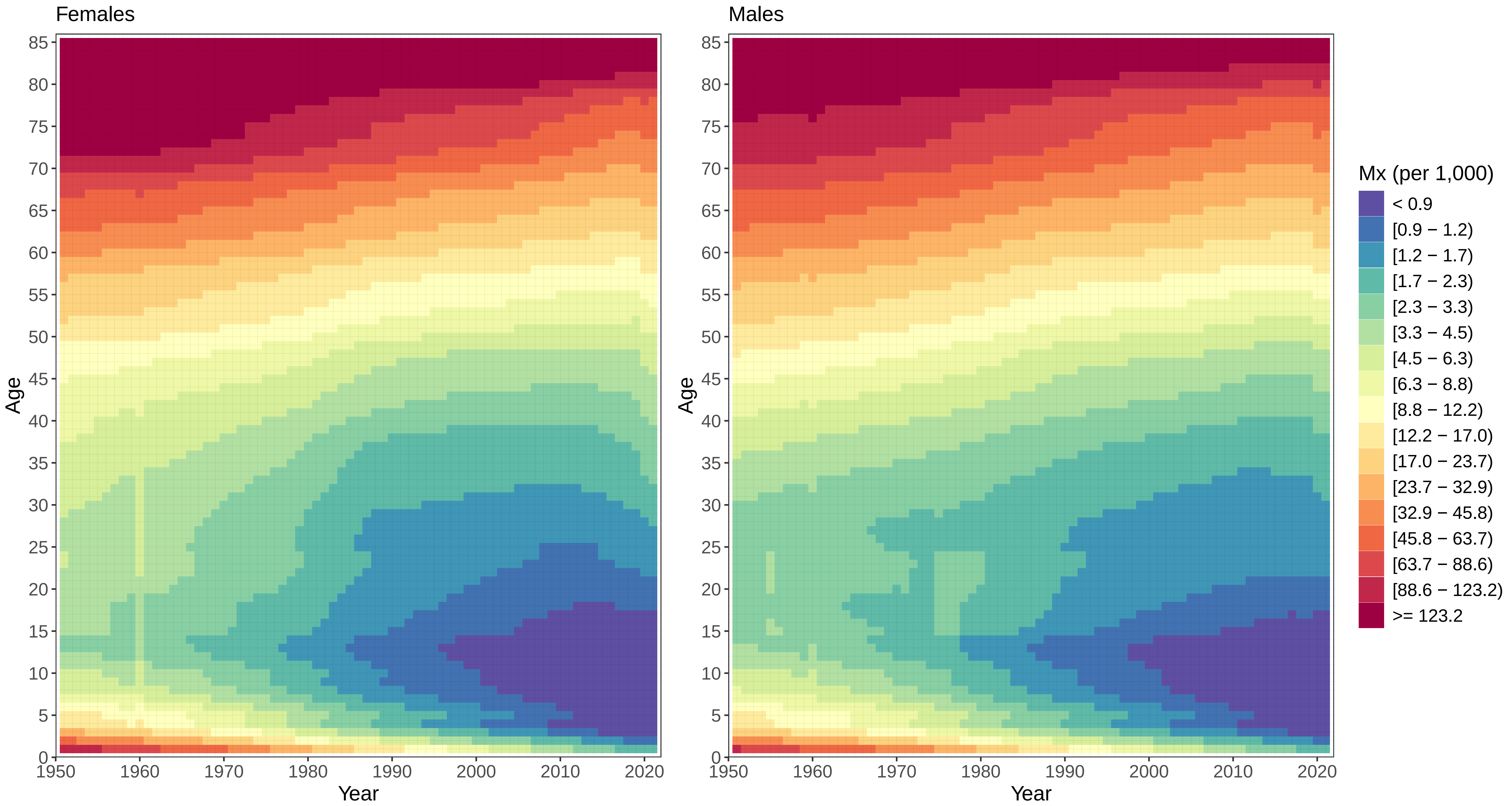

Appendix 2 Figure S4: Oman mortality rates, 1950−2023

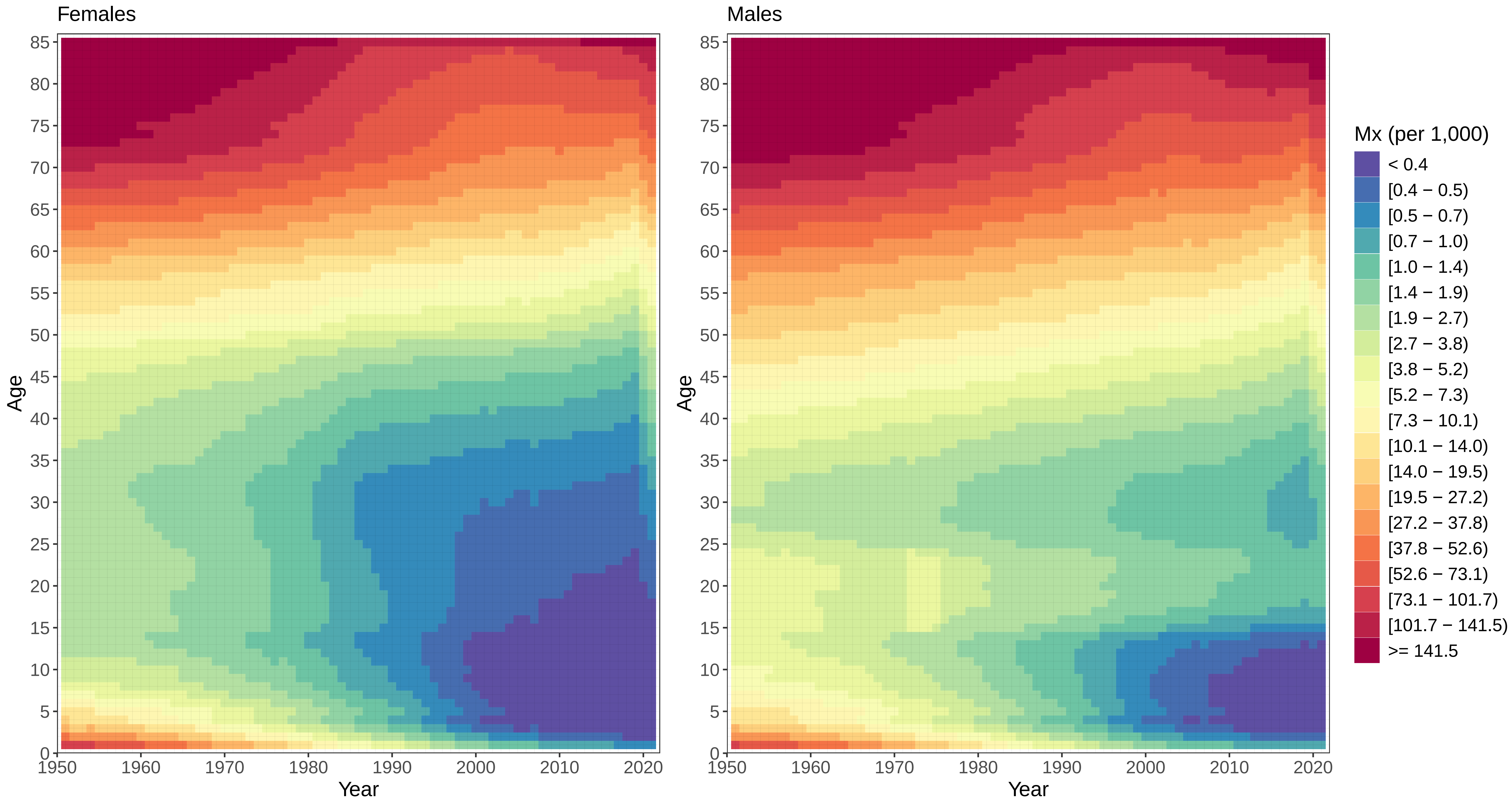

Appendix 2 Figure S4: Palestine mortality rates, 1950−2023

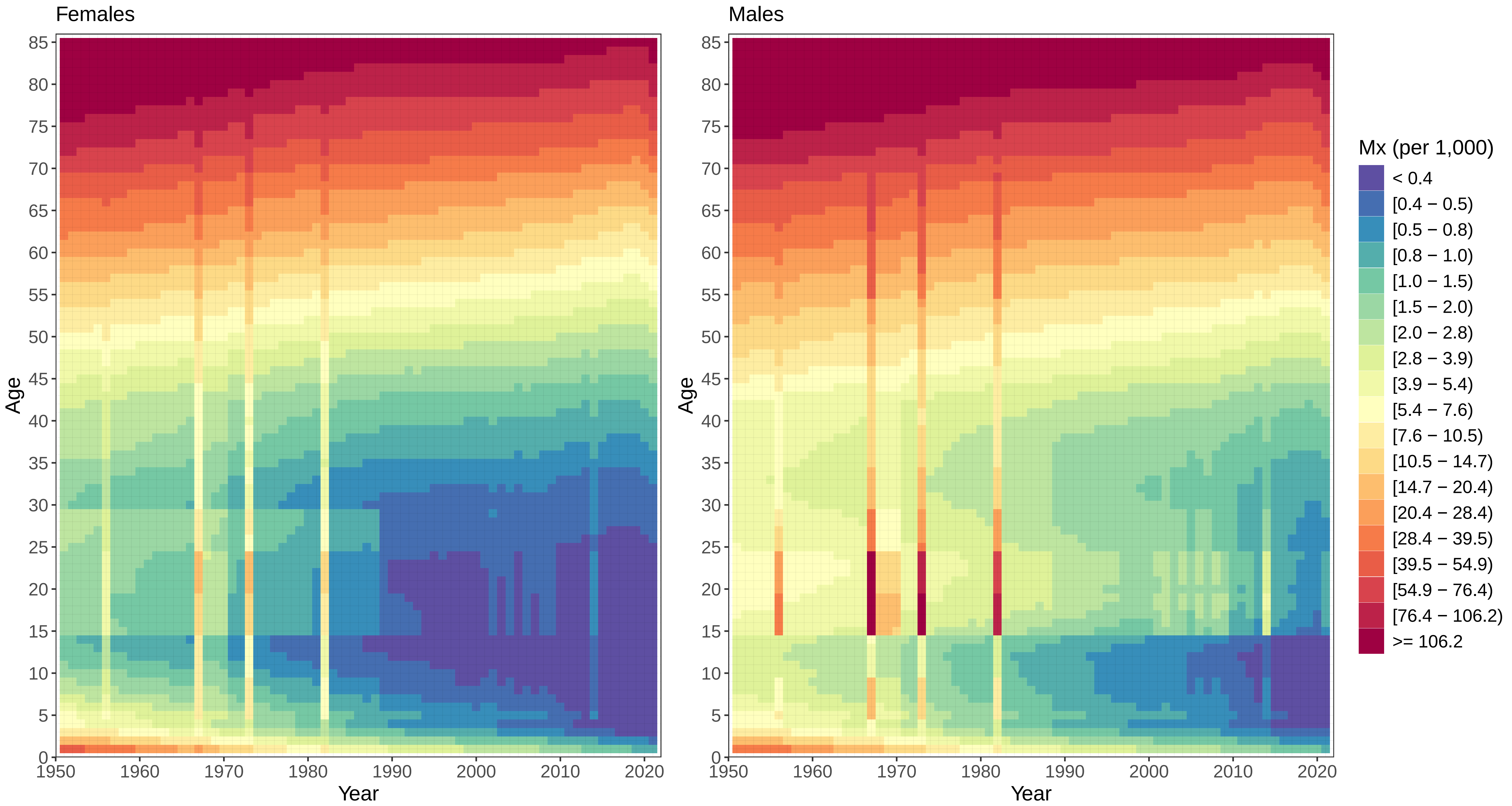

Appendix 2 Figure S4: Qatar mortality rates, 1950−2023

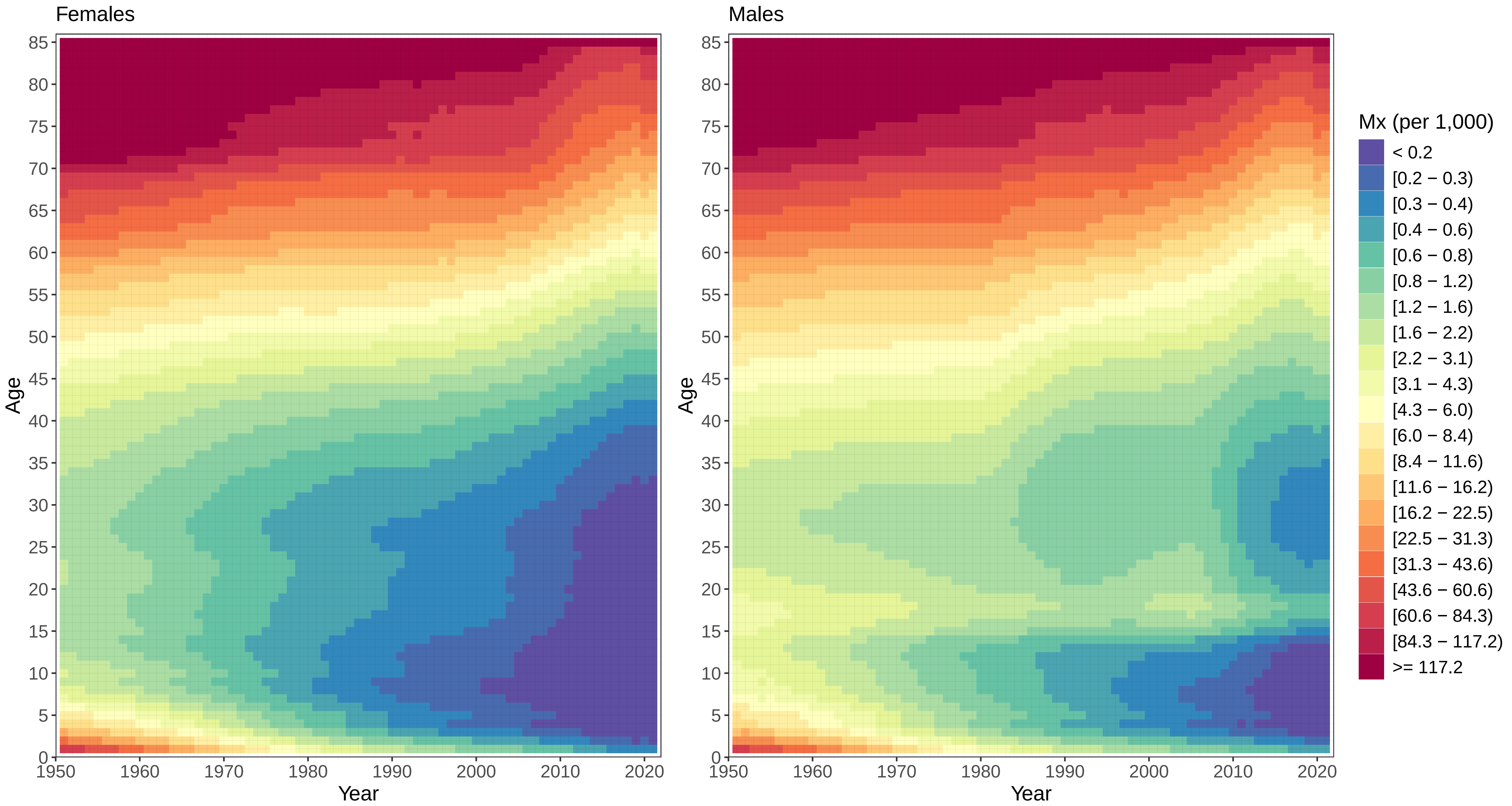

Appendix 2 Figure S4: Saudi Arabia mortality rates, 1950−2023

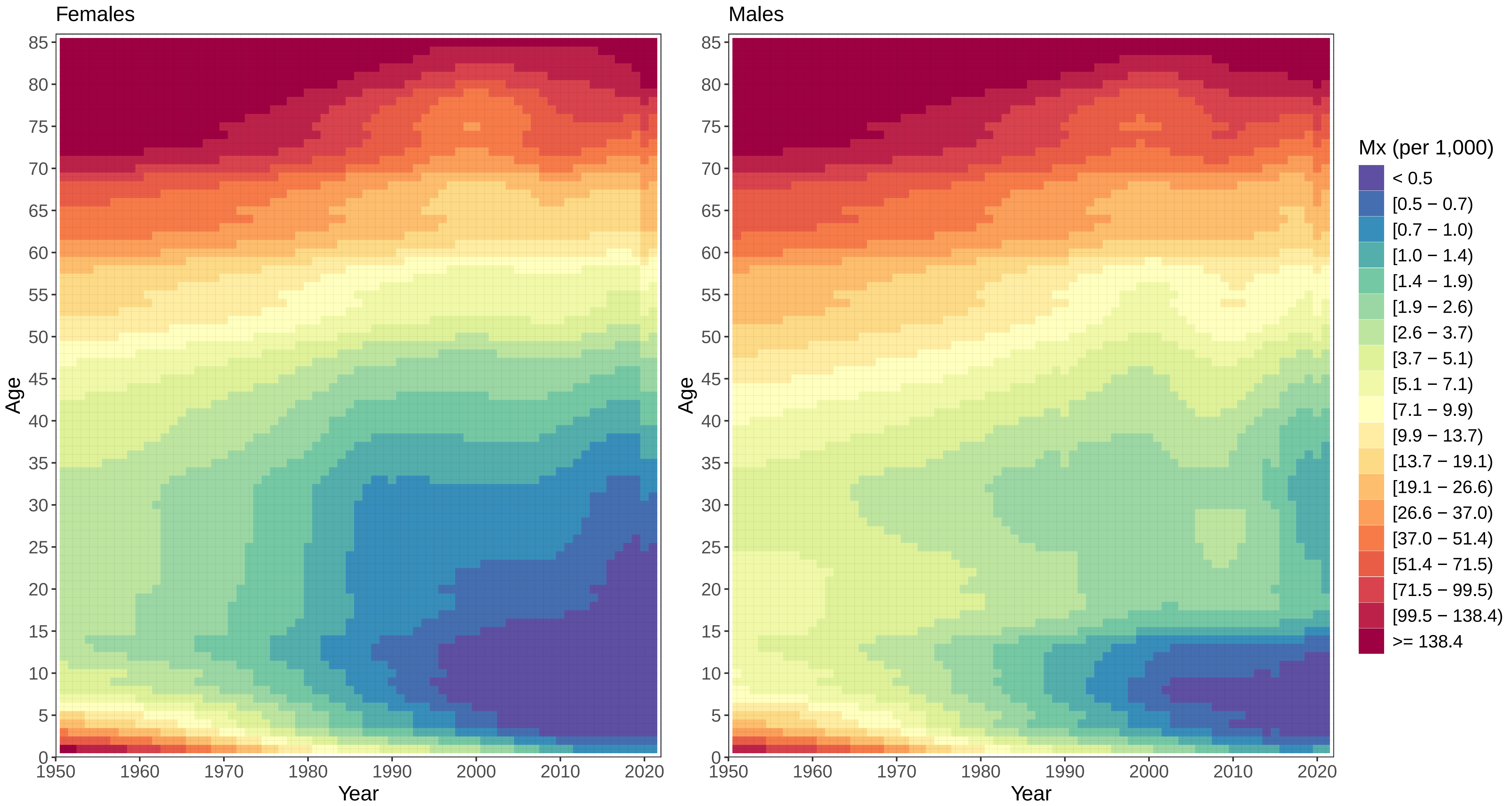

Appendix 2 Figure S4: Sudan mortality rates, 1950−2023

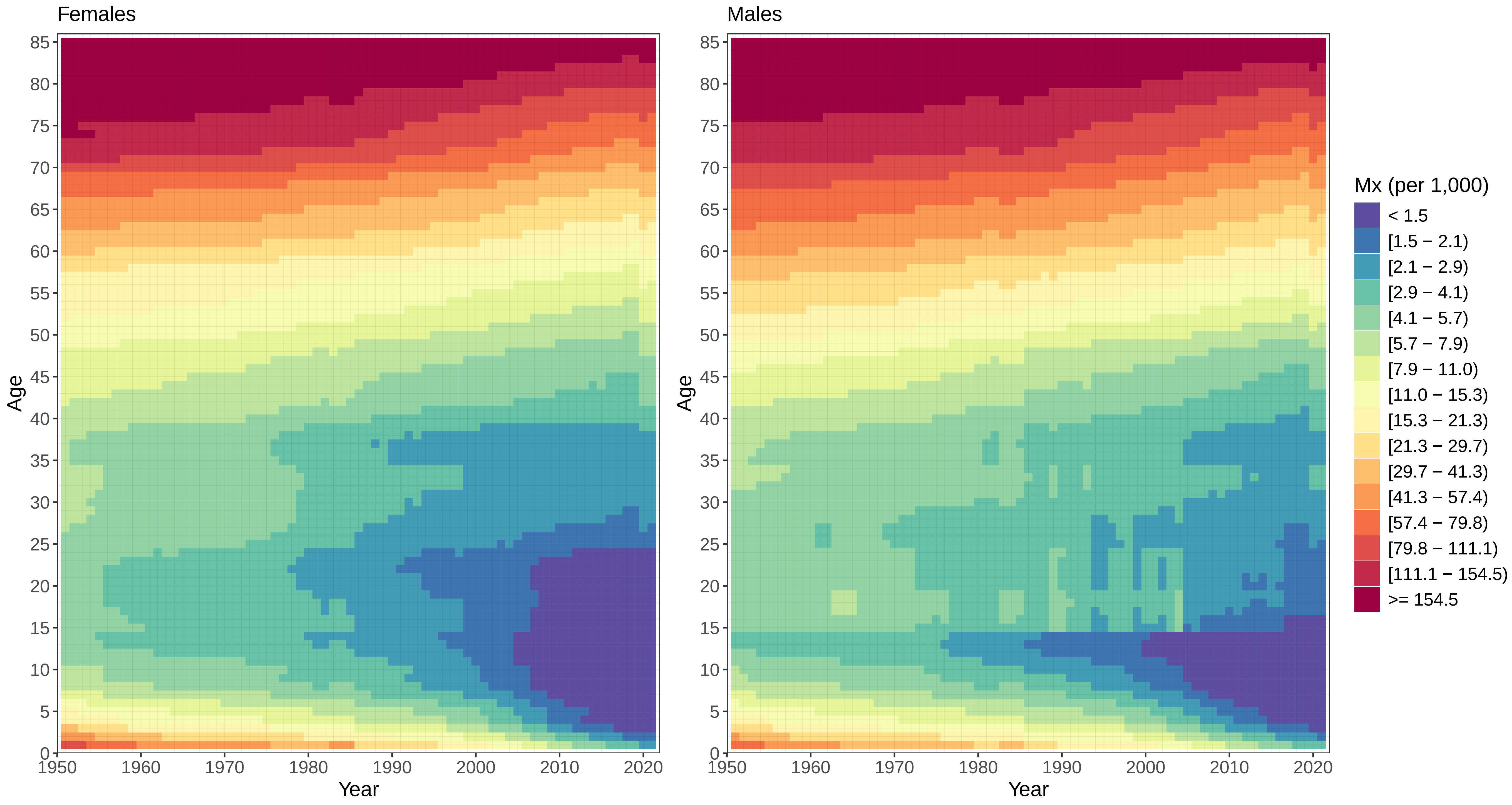

Appendix 2 Figure S4: Syria mortality rates, 1950−2023

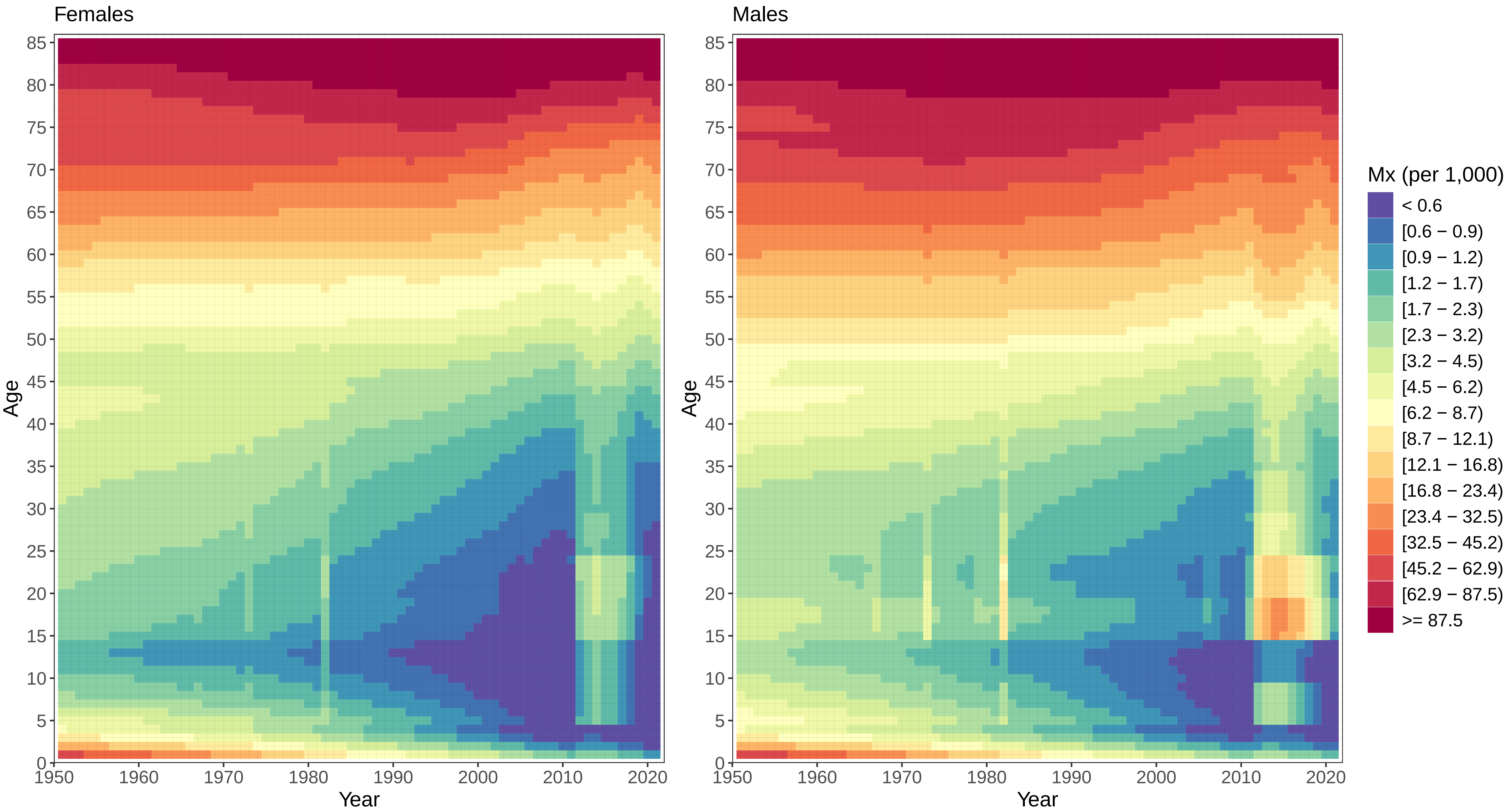

Appendix 2 Figure S4: Tunisia mortality rates, 1950−2023

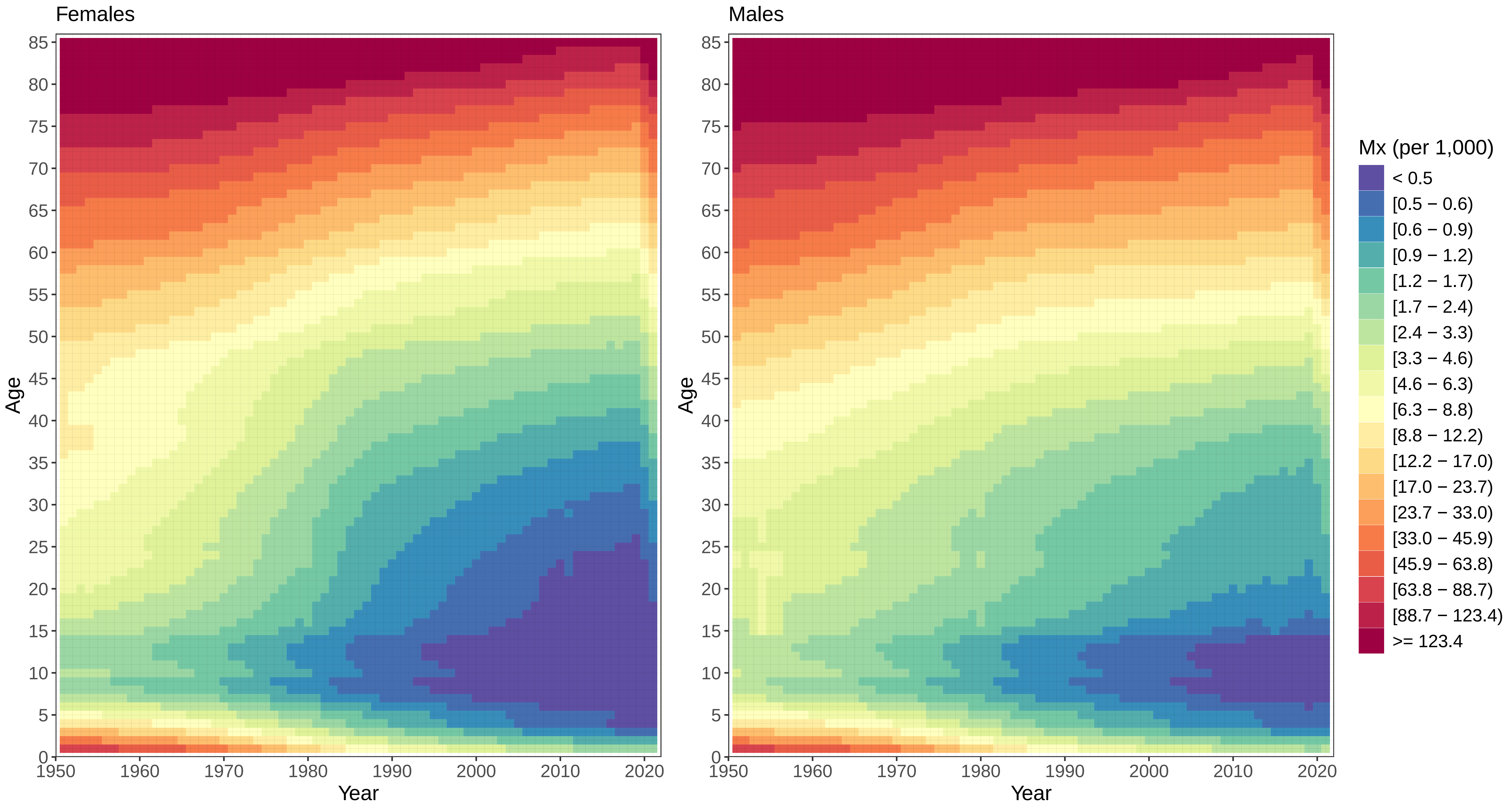

Appendix 2 Figure S4: Türkiye mortality rates, 1950−2023

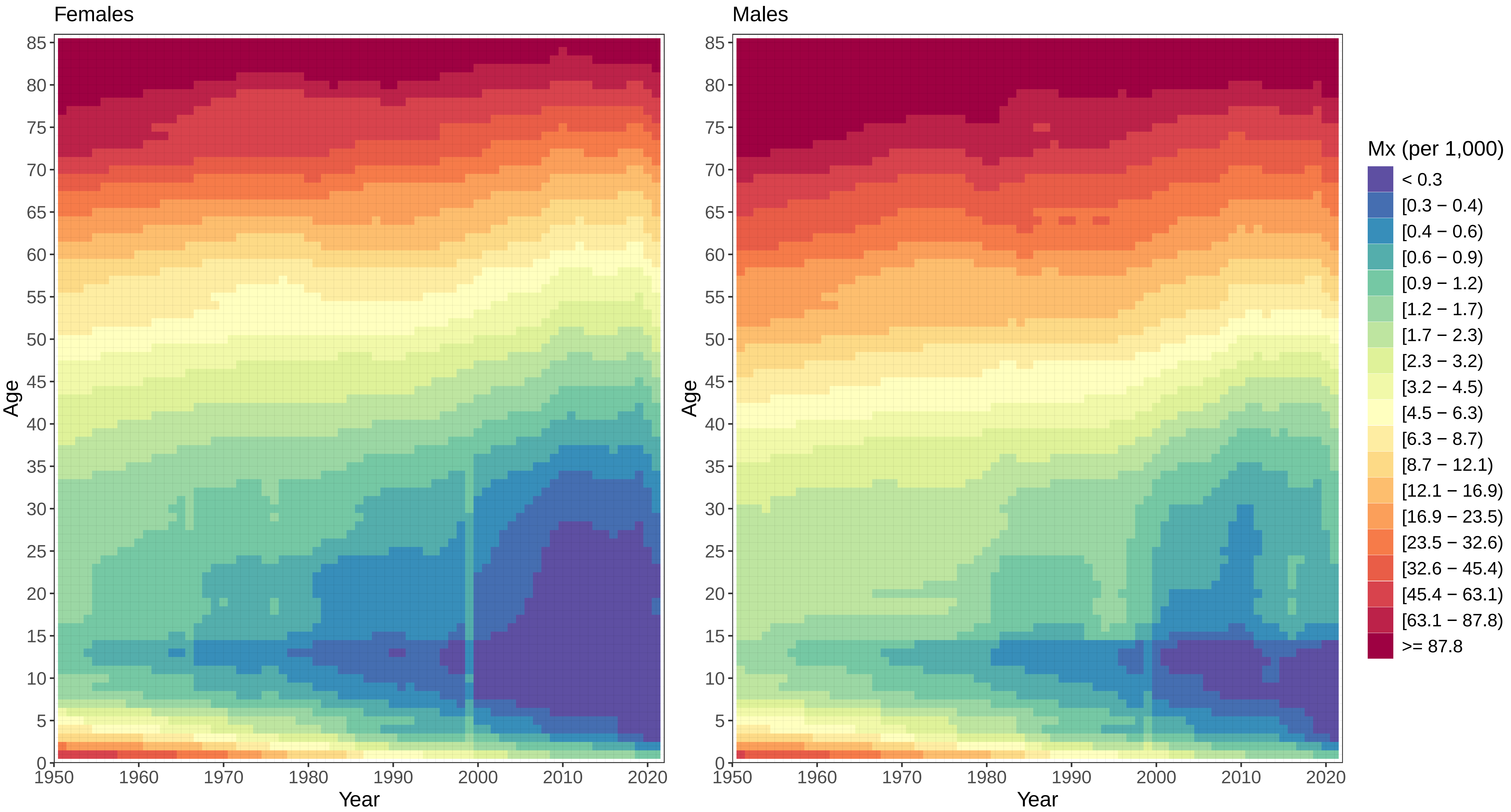

Appendix 2 Figure S4: United Arab Emirates mortality rates, 1950−2023

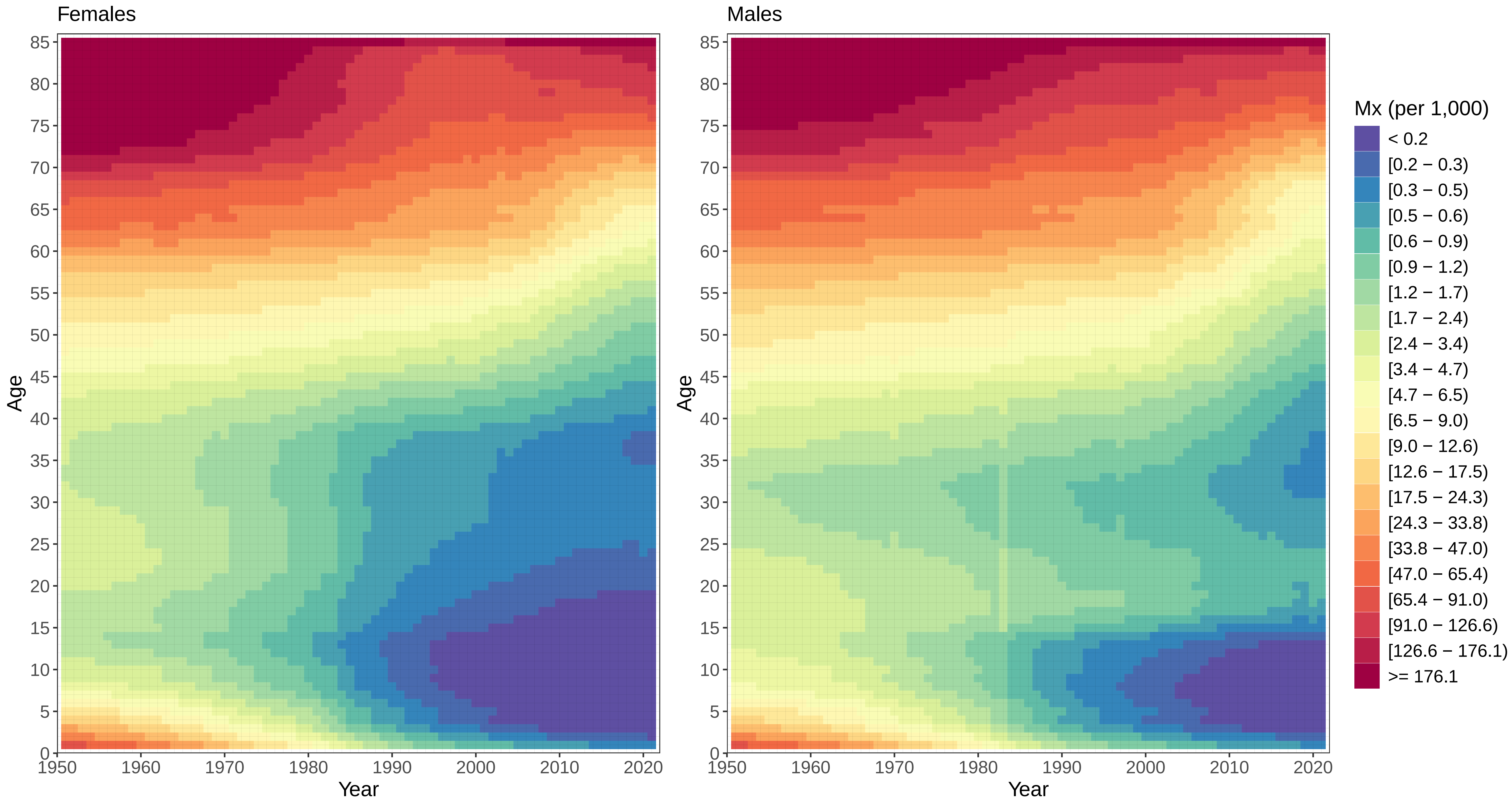

Appendix 2 Figure S4: Yemen mortality rates, 1950−2023

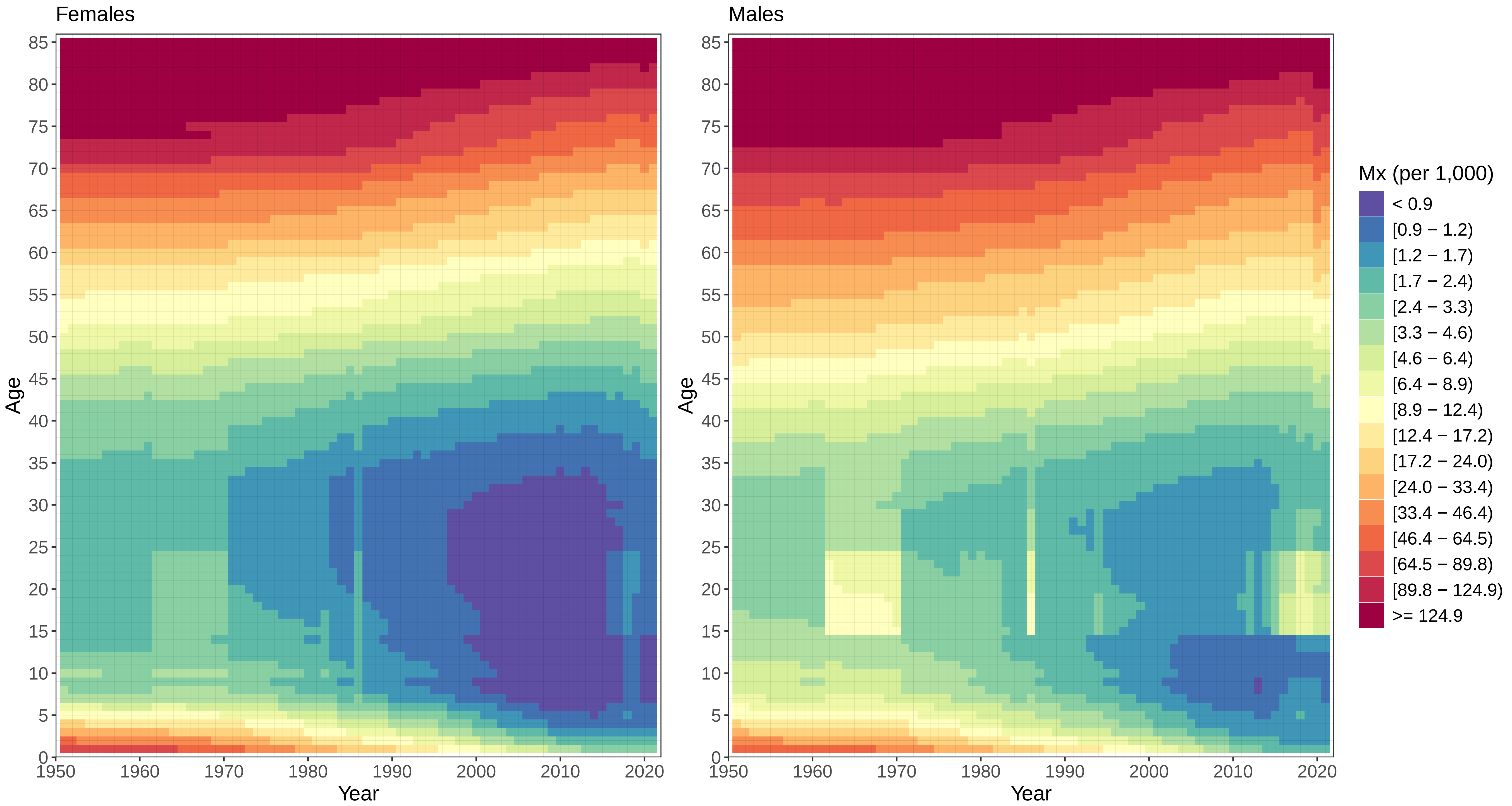

Appendix 2 Figure S4: Bangladesh mortality rates, 1950−2023

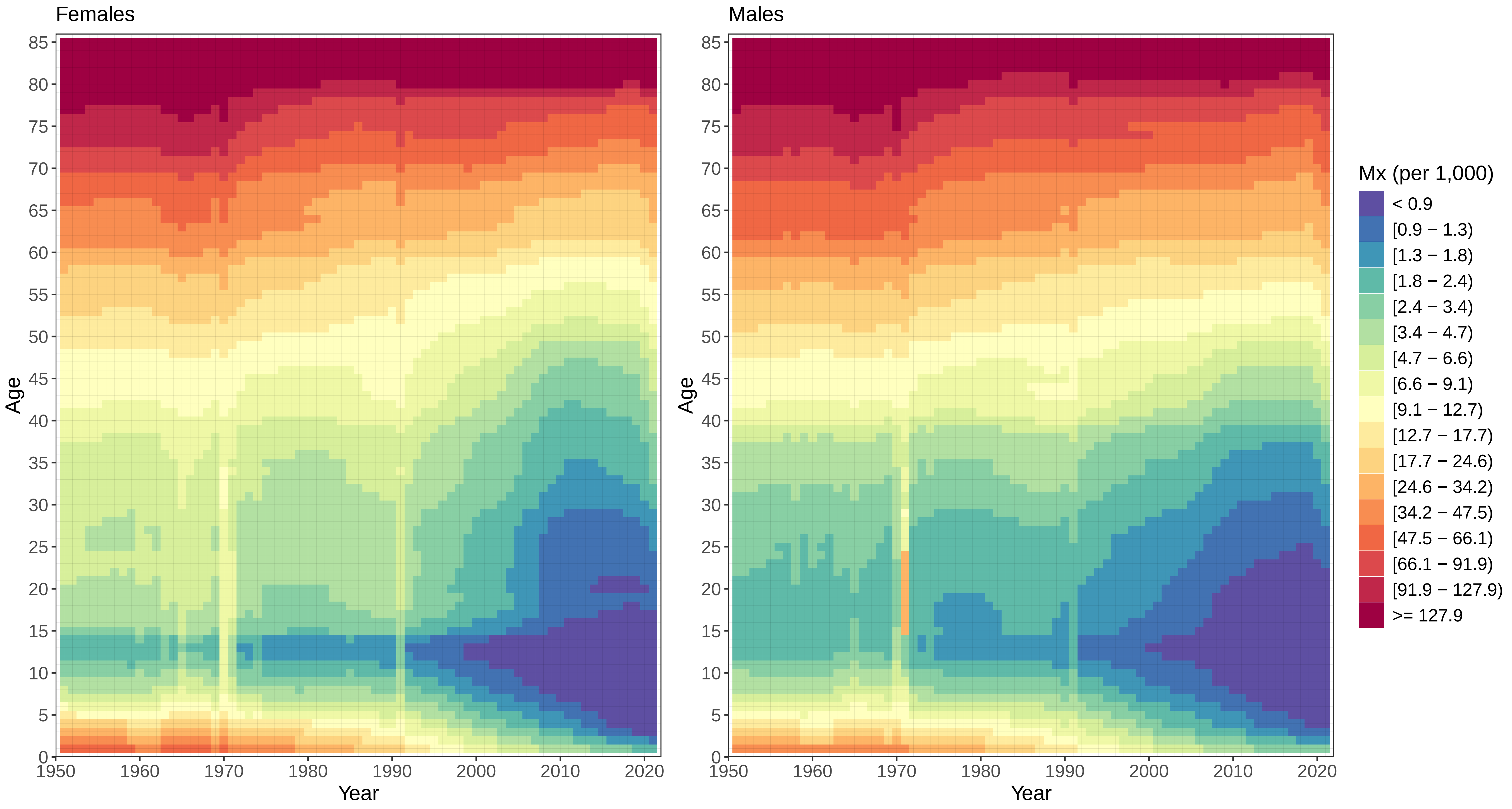

Appendix 2 Figure S4: Bhutan mortality rates, 1950−2023

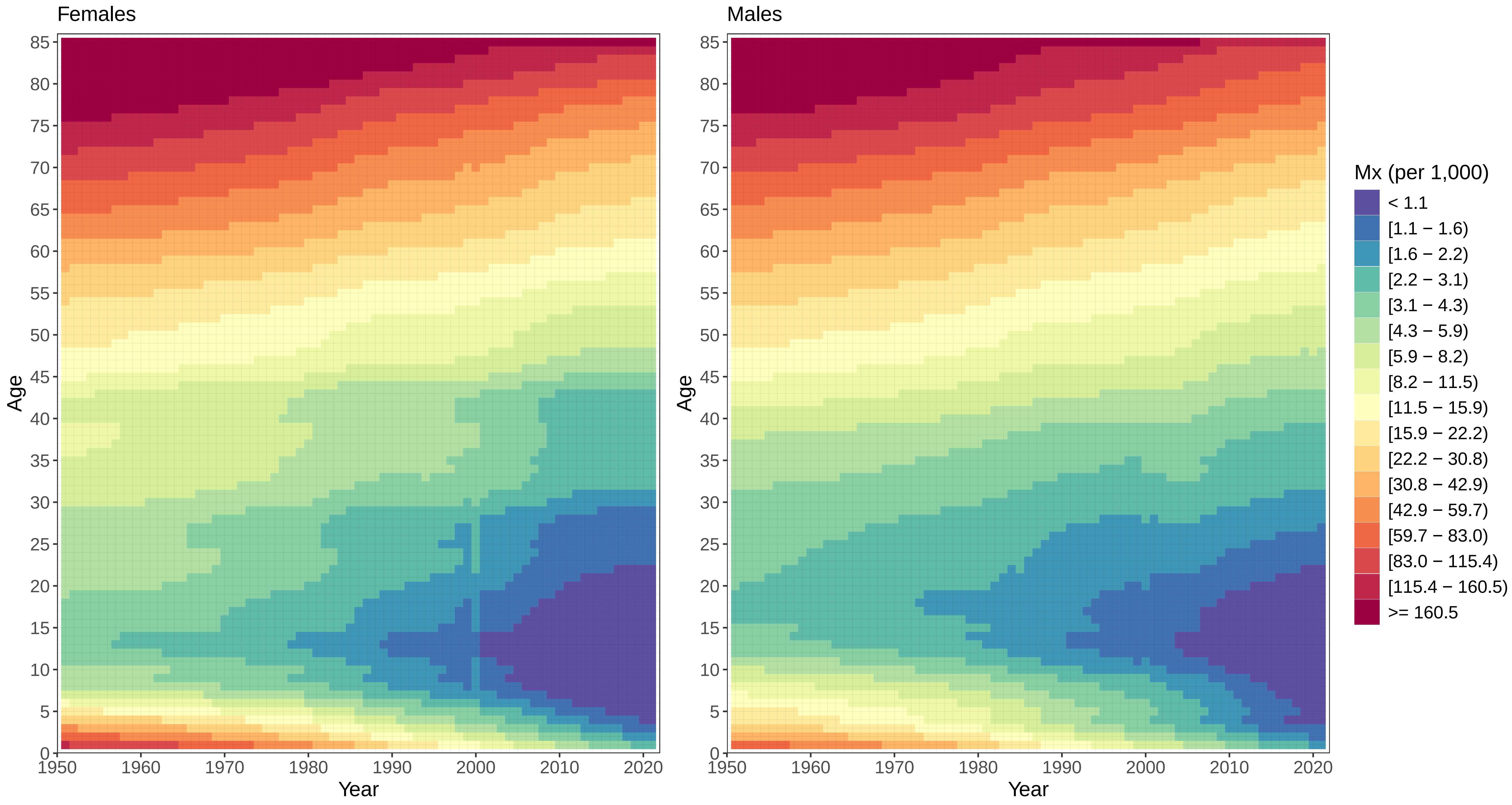

Appendix 2 Figure S4: India mortality rates, 1950−2023

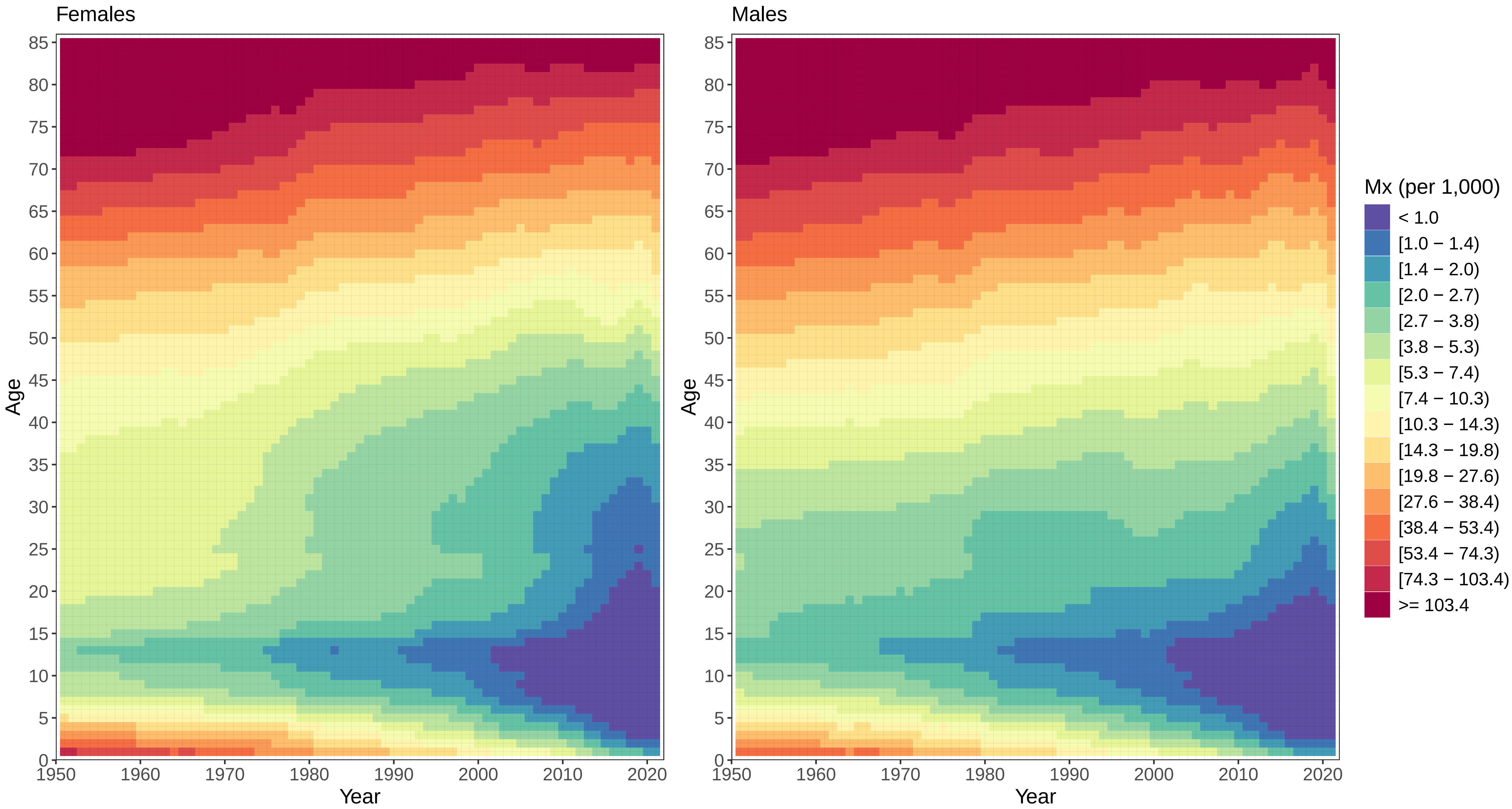

Appendix 2 Figure S4: Nepal mortality rates, 1950−2023

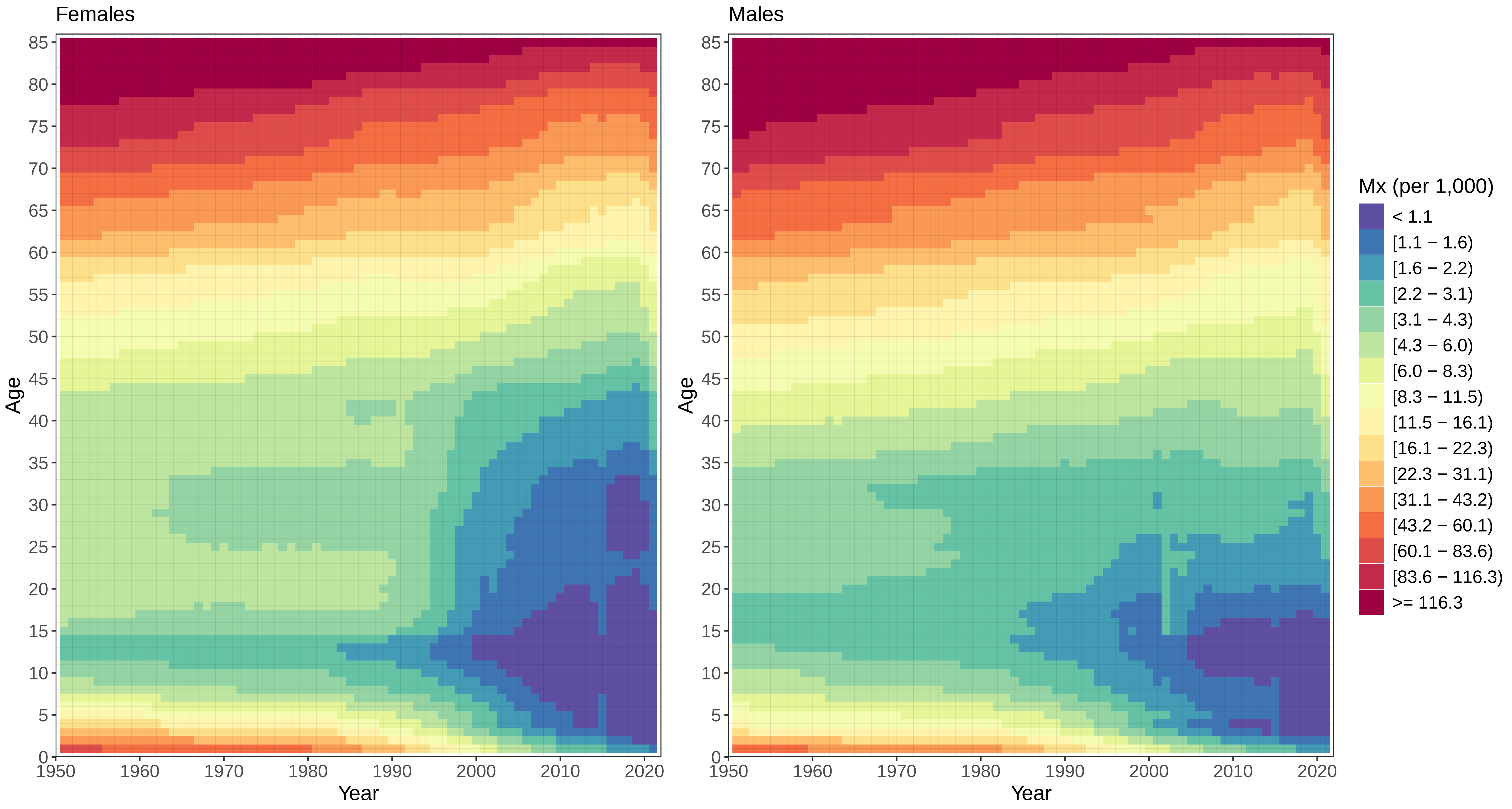

Appendix 2 Figure S4: Pakistan mortality rates, 1950−2023

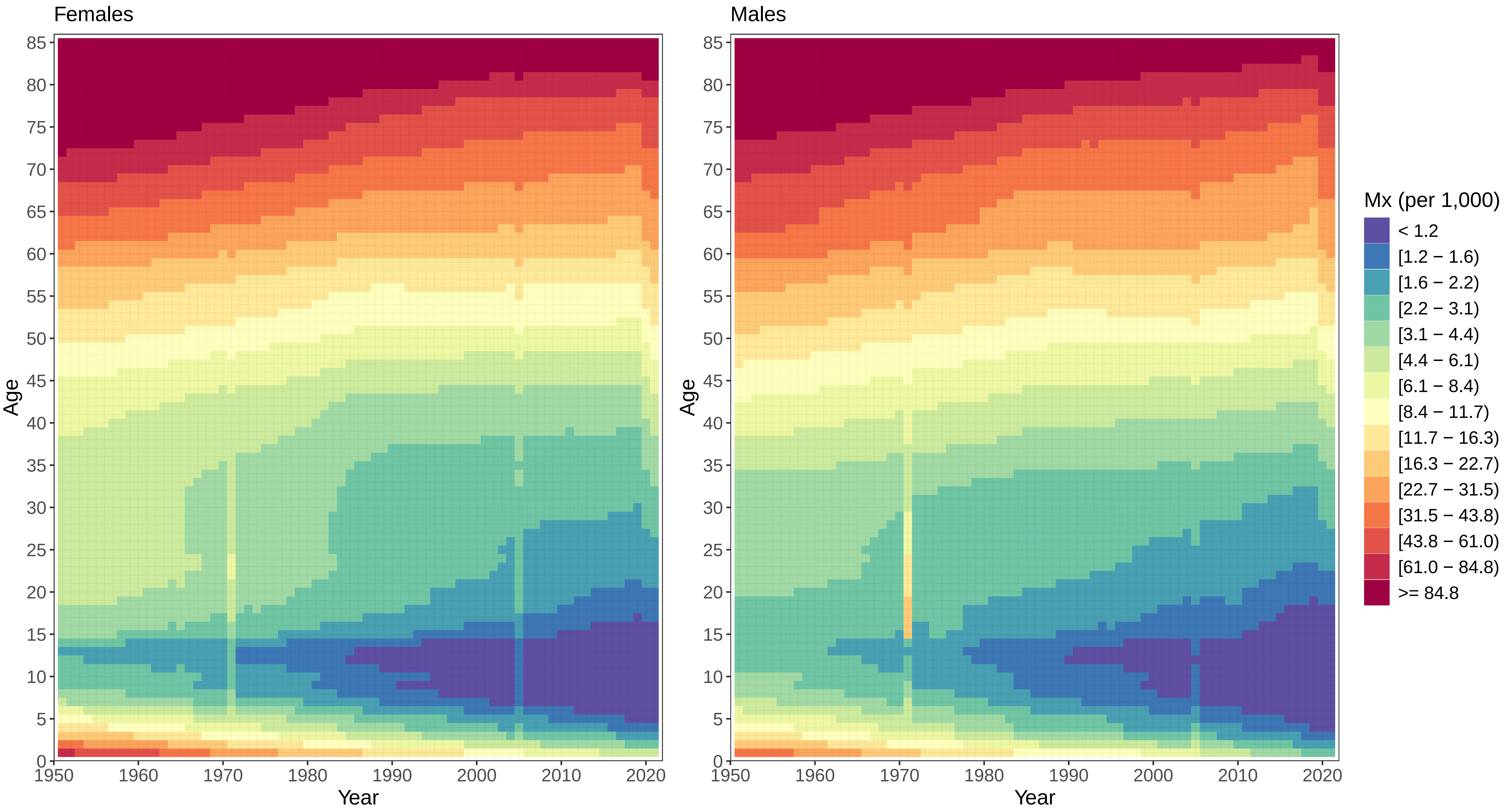

Appendix 2 Figure S4: China (without Hong Kong and Macao) mortality rates, 1950−2023

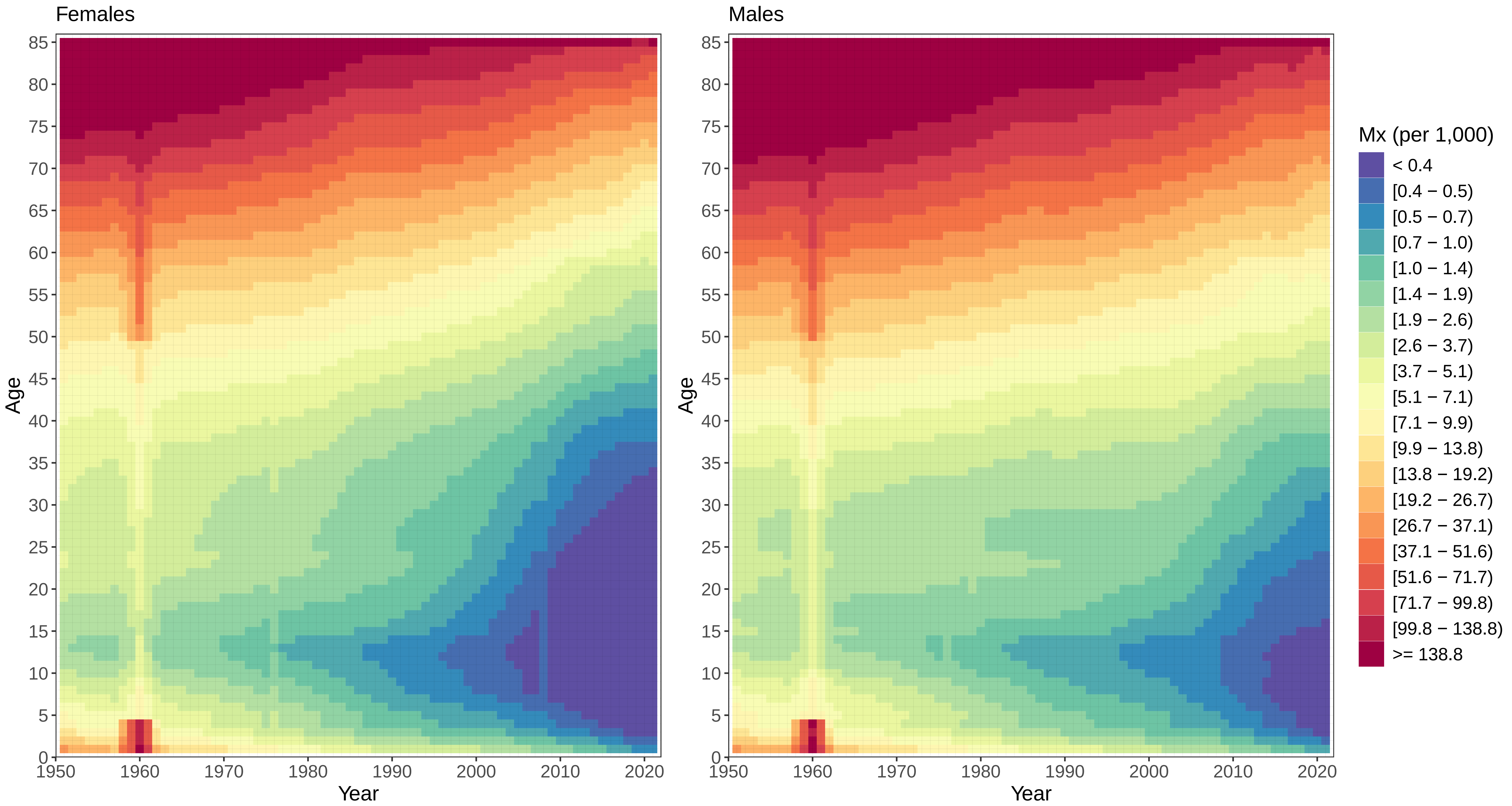

Appendix 2 Figure S4: North Korea mortality rates, 1950−2023

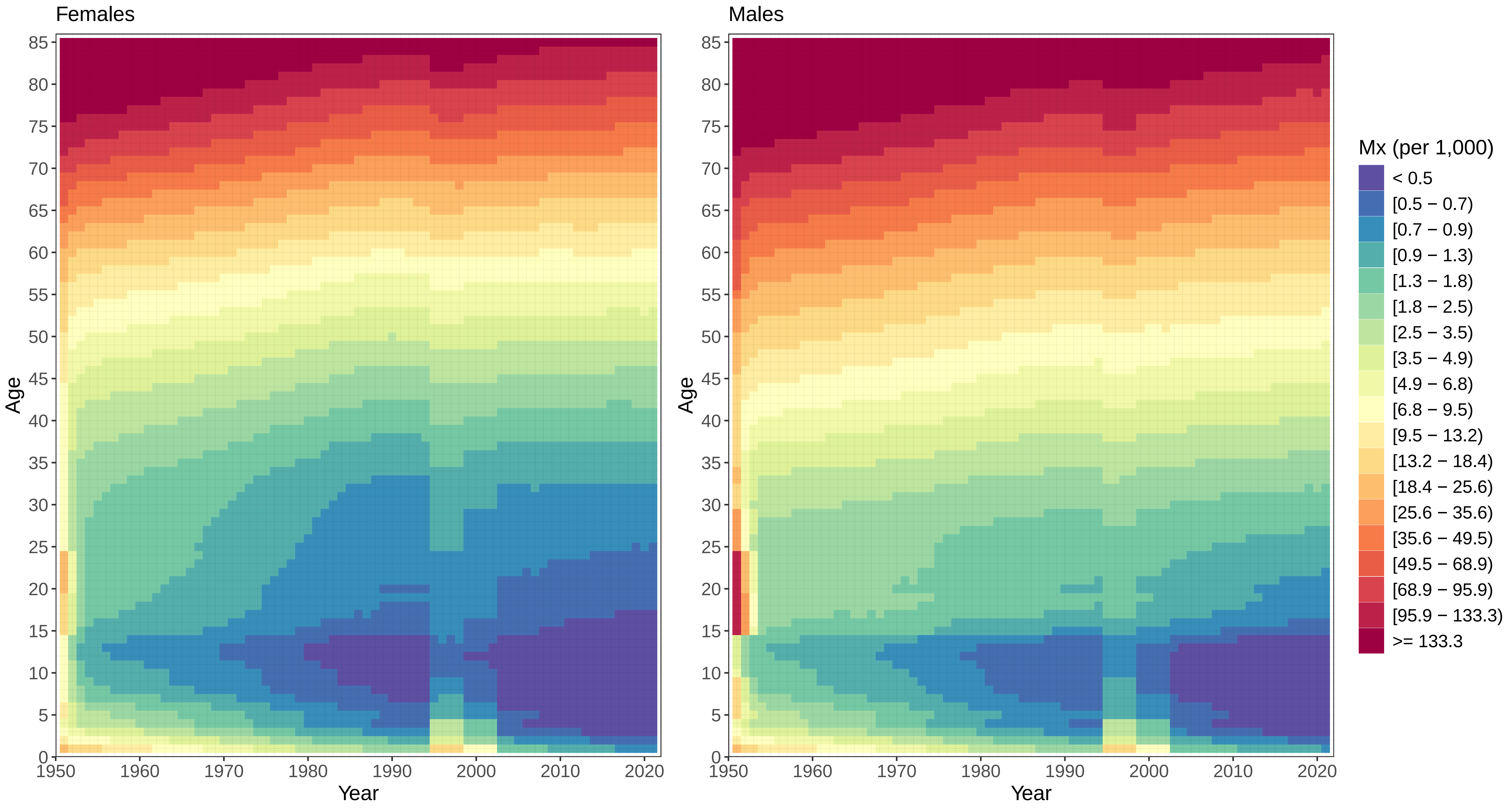

Appendix 2 Figure S4: Taiwan mortality rates, 1950−2023

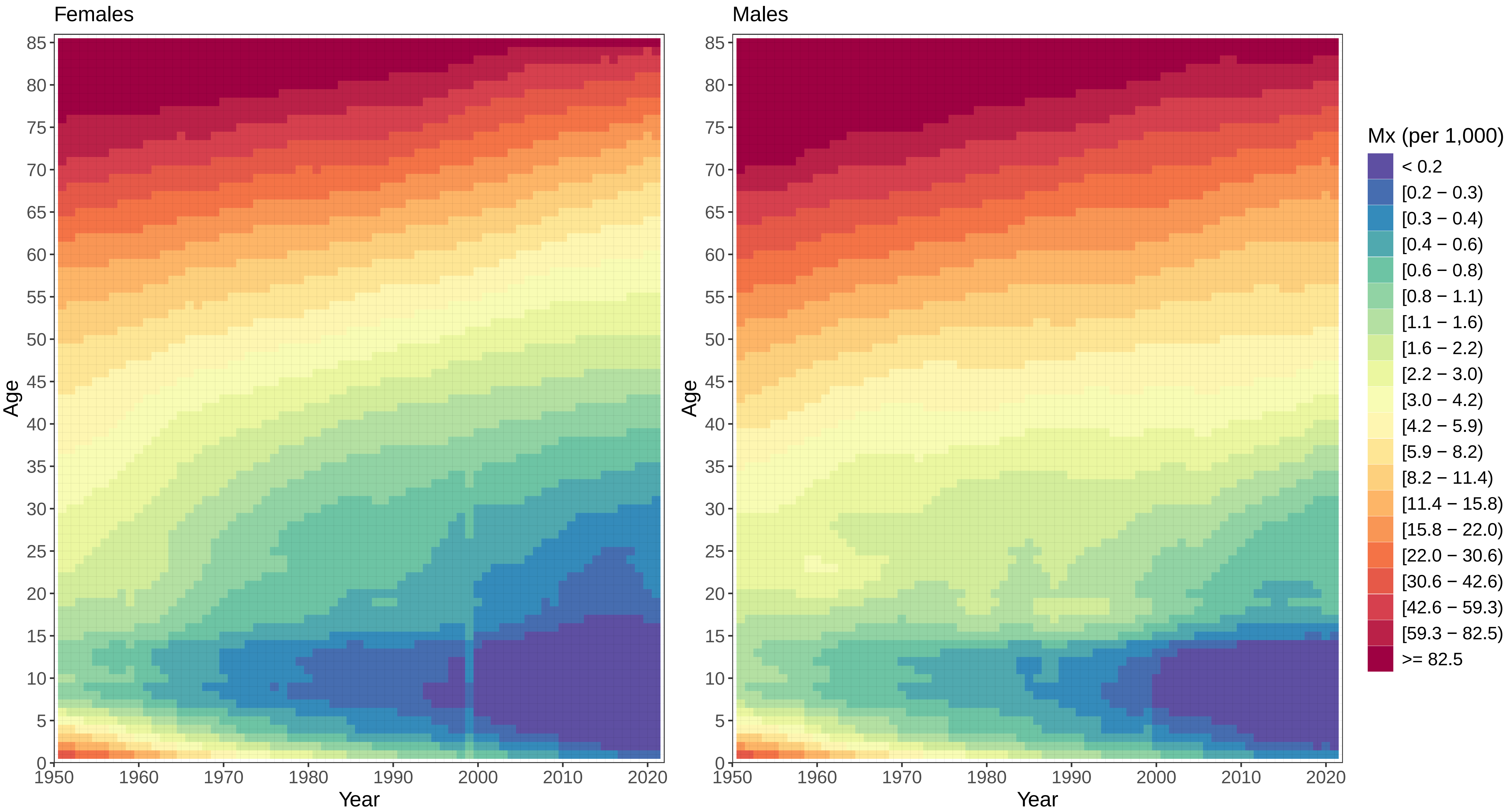

Appendix 2 Figure S4: American Samoa mortality rates, 1950−2023

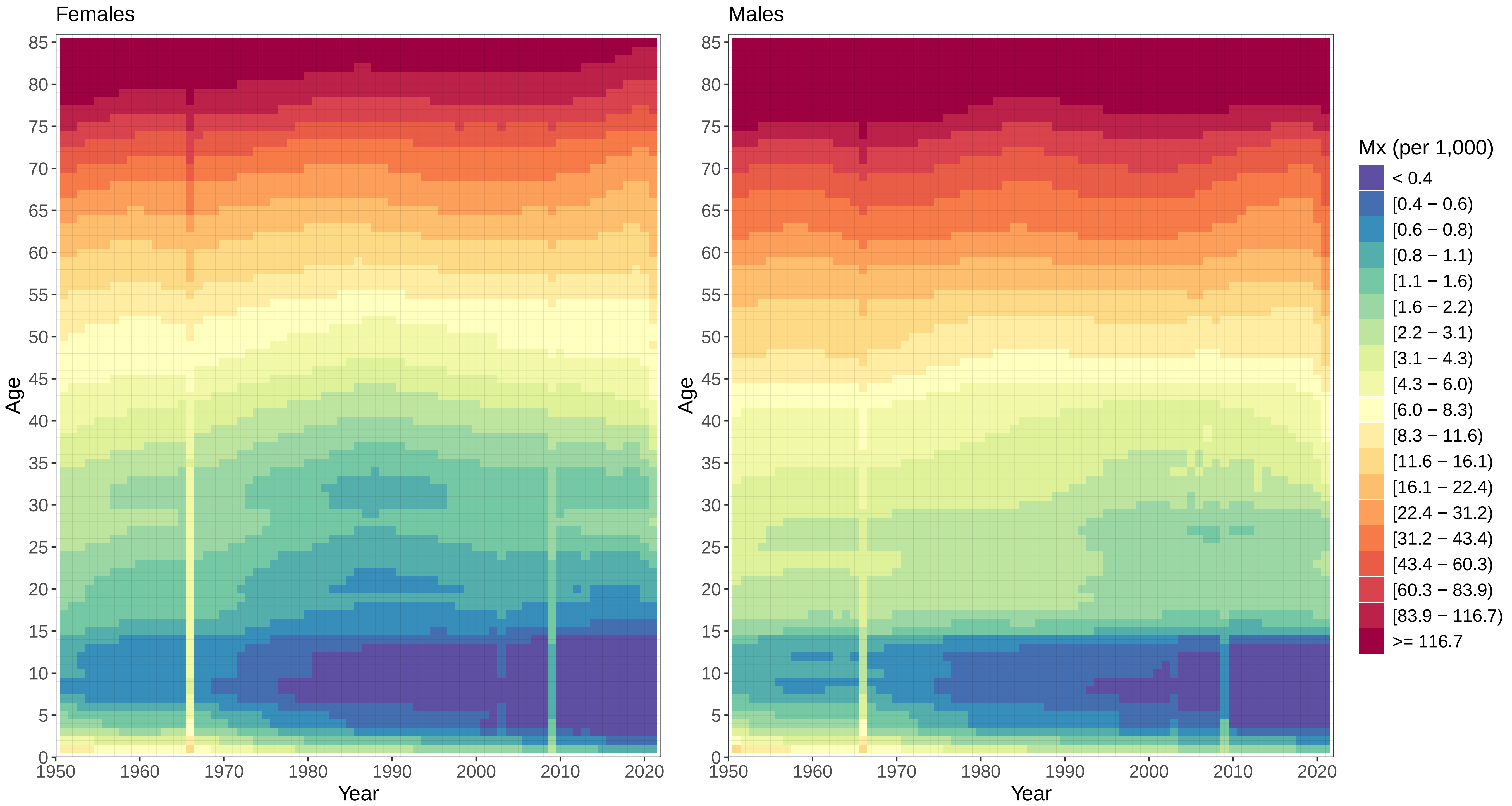

Appendix 2 Figure S4: Cook Islands mortality rates, 1950−2023

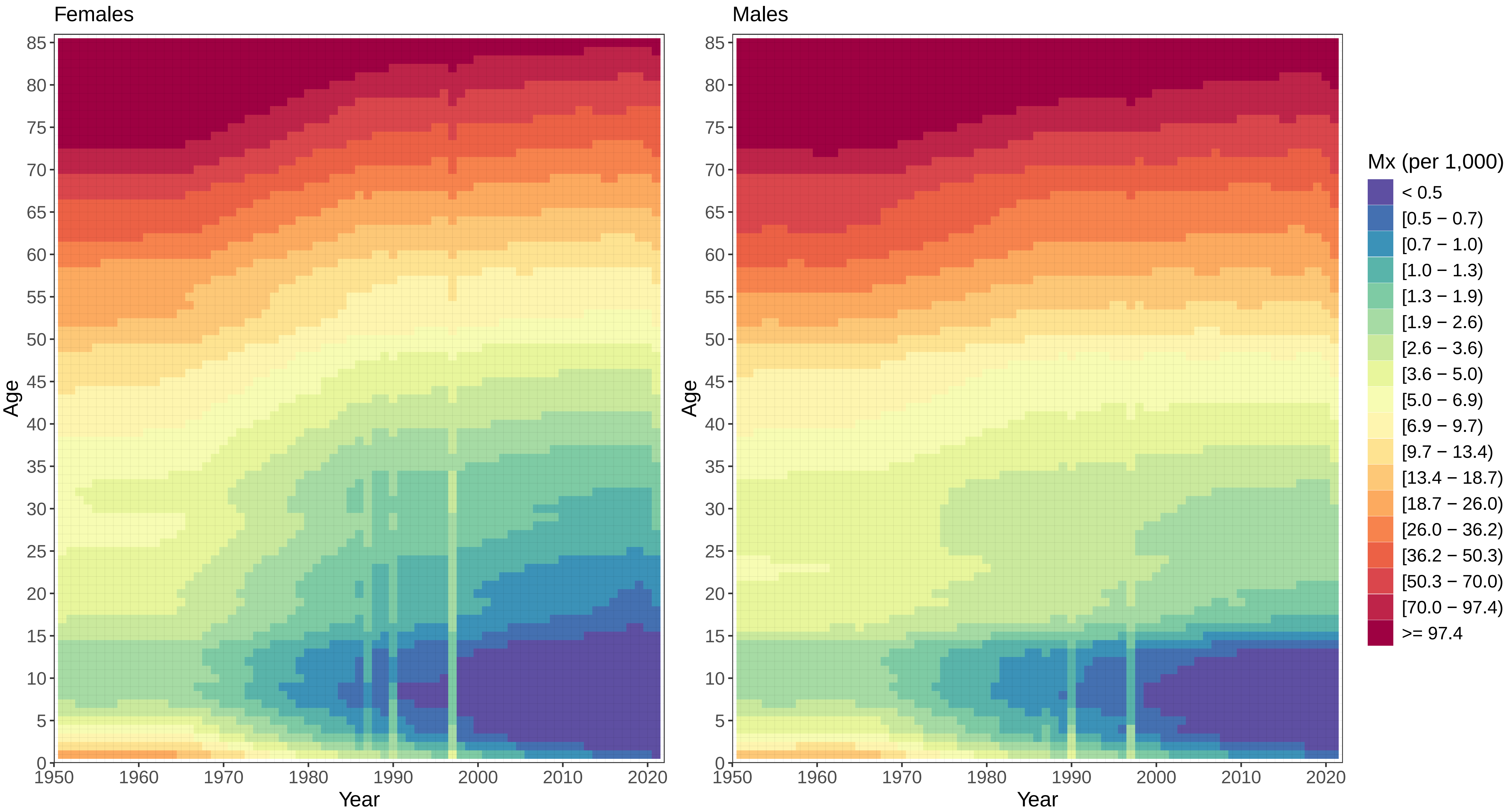

Appendix 2 Figure S4: Federated States of Micronesia mortality rates, 1950−2023

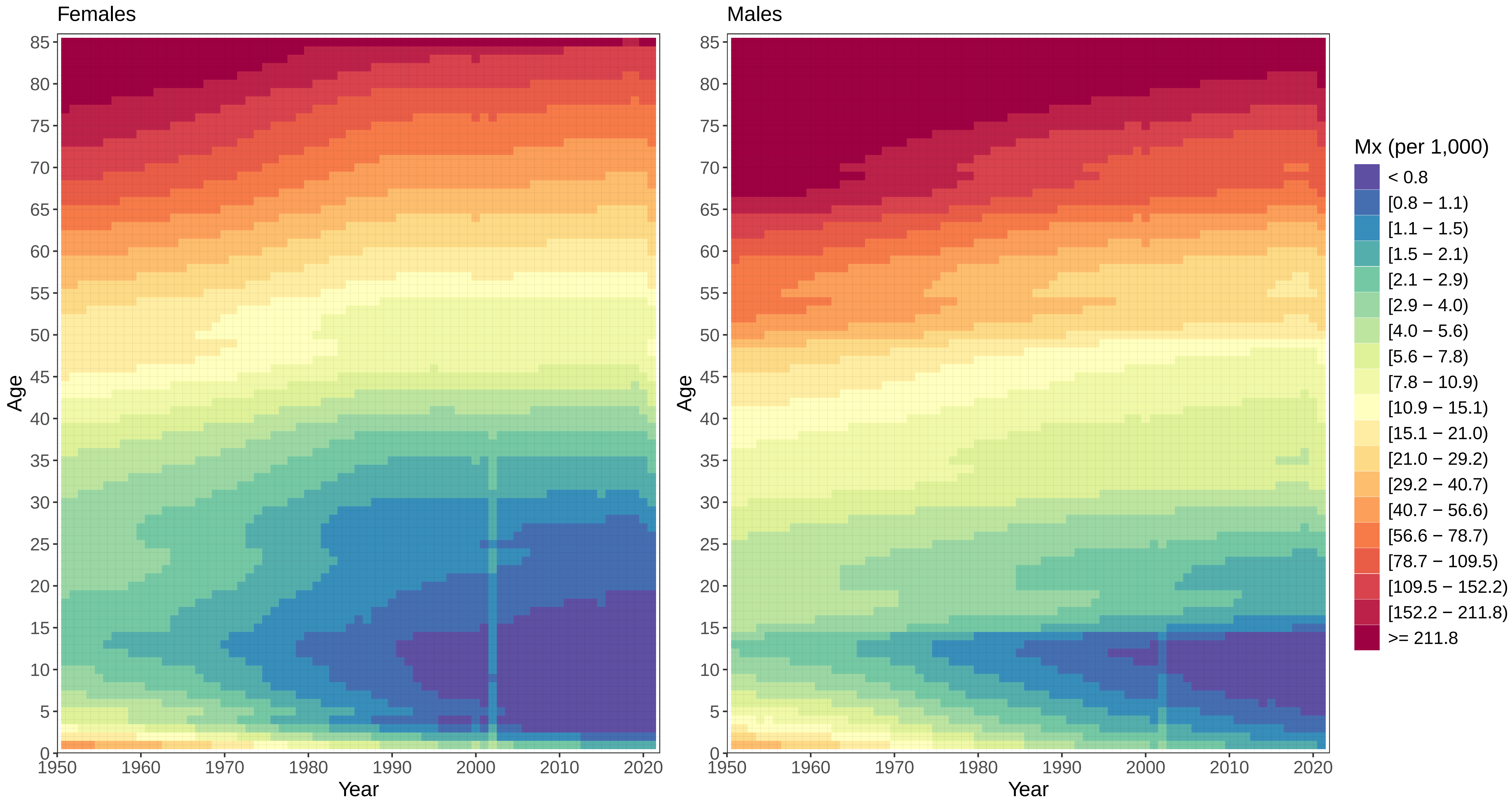

Appendix 2 Figure S4: Fiji mortality rates, 1950−2023

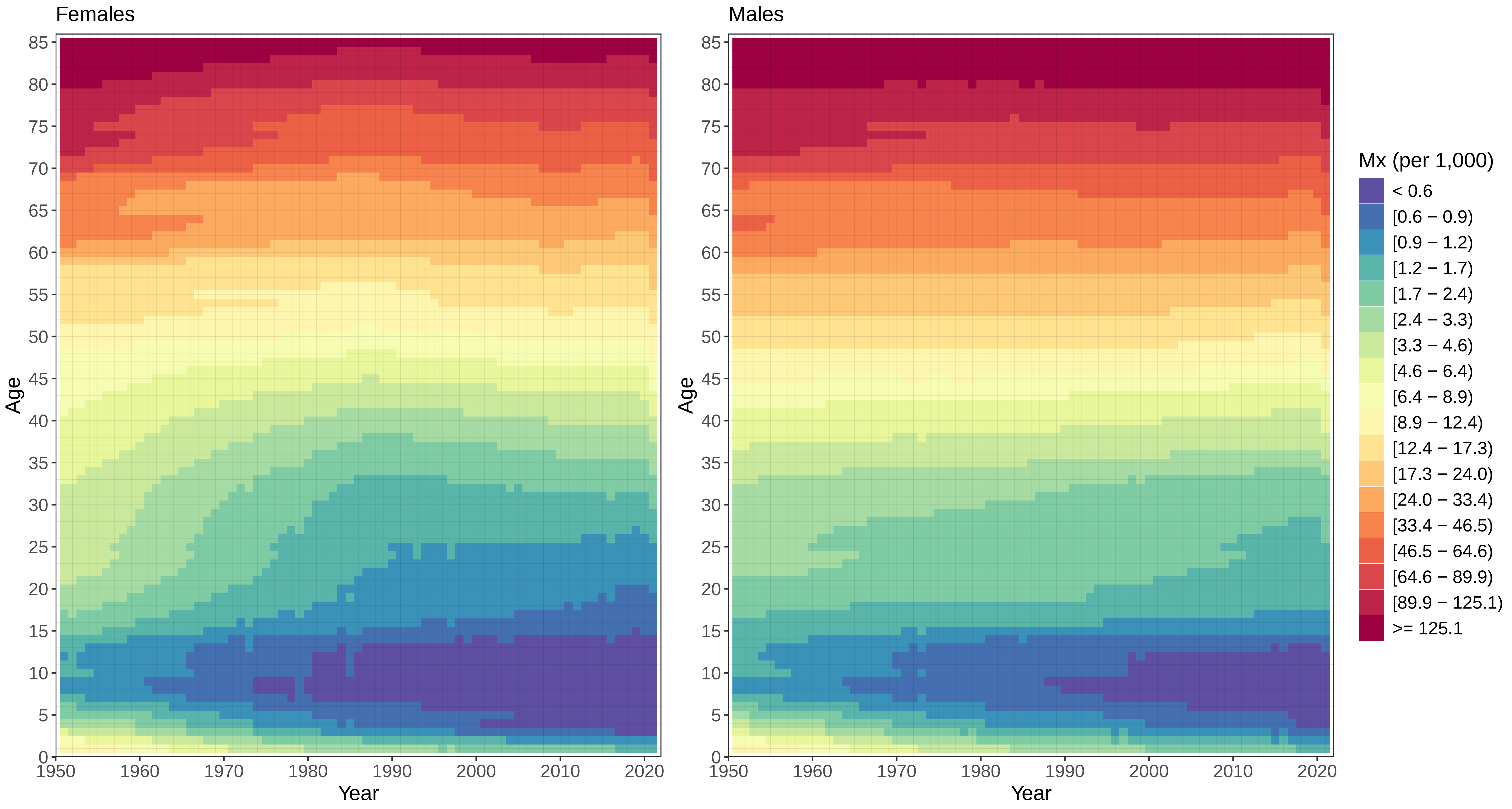

Appendix 2 Figure S4: Guam mortality rates, 1950−2023

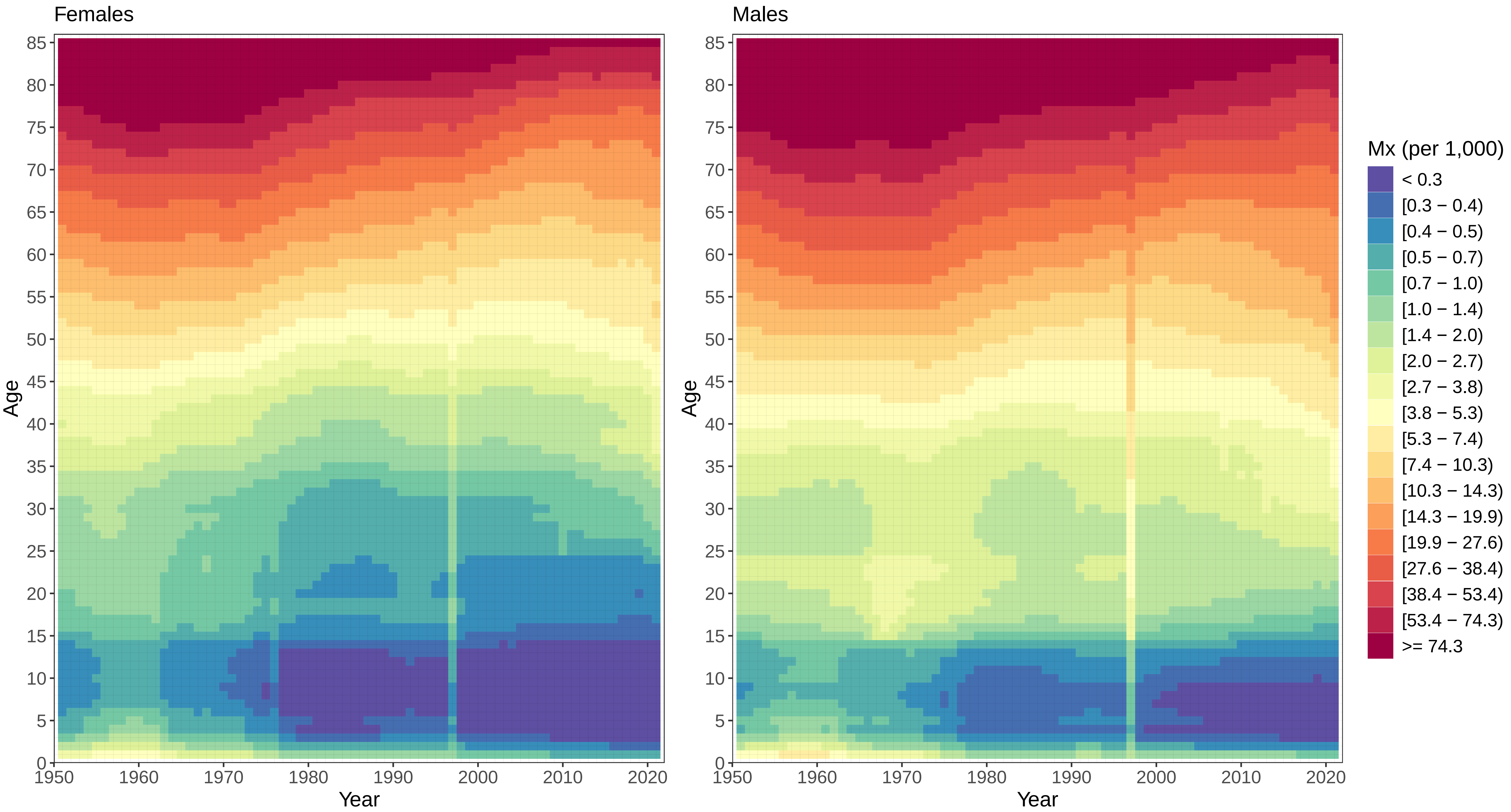

Appendix 2 Figure S4: Kiribati mortality rates, 1950−2023

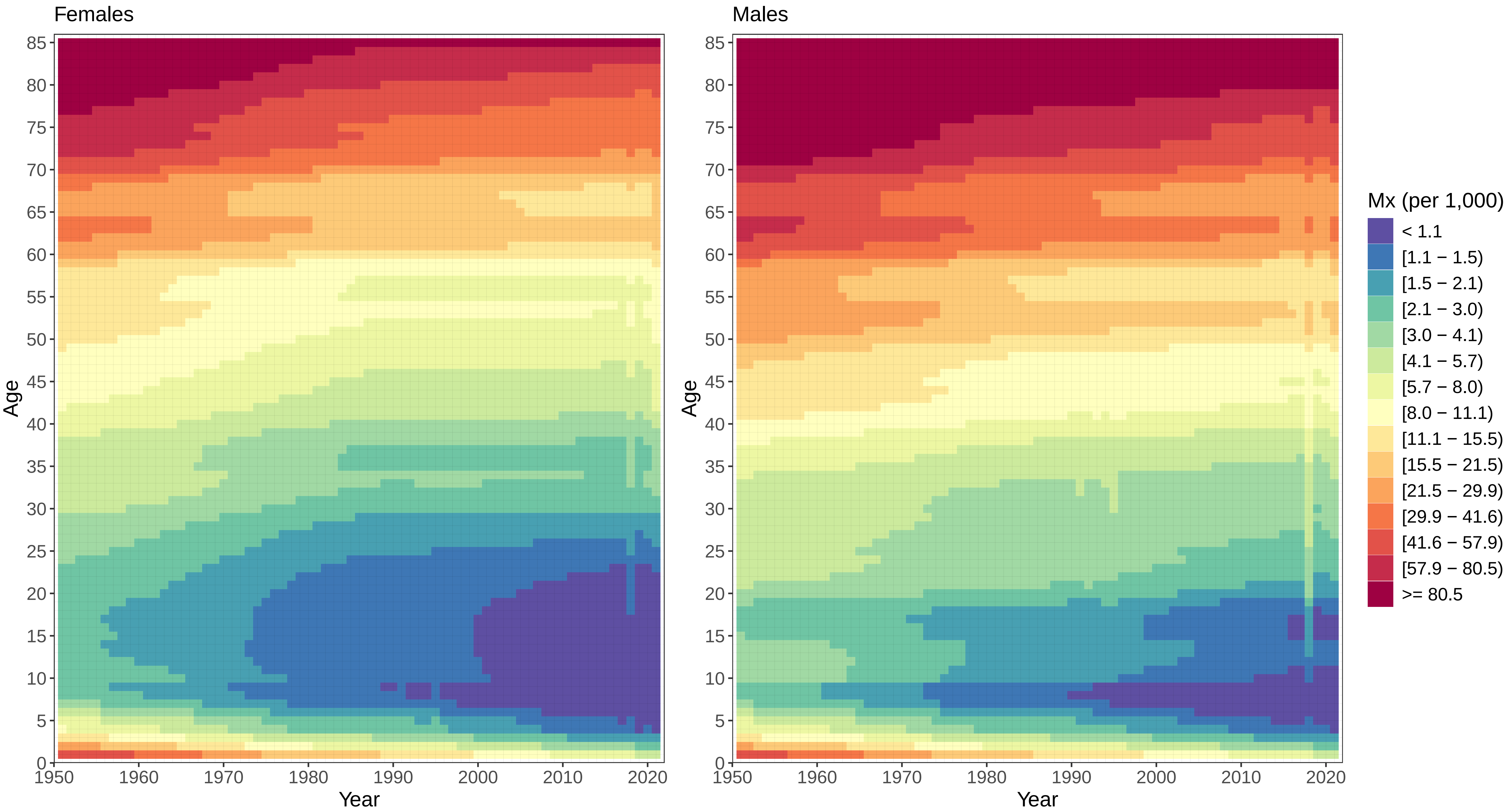

Appendix 2 Figure S4: Marshall Islands mortality rates, 1950−2023

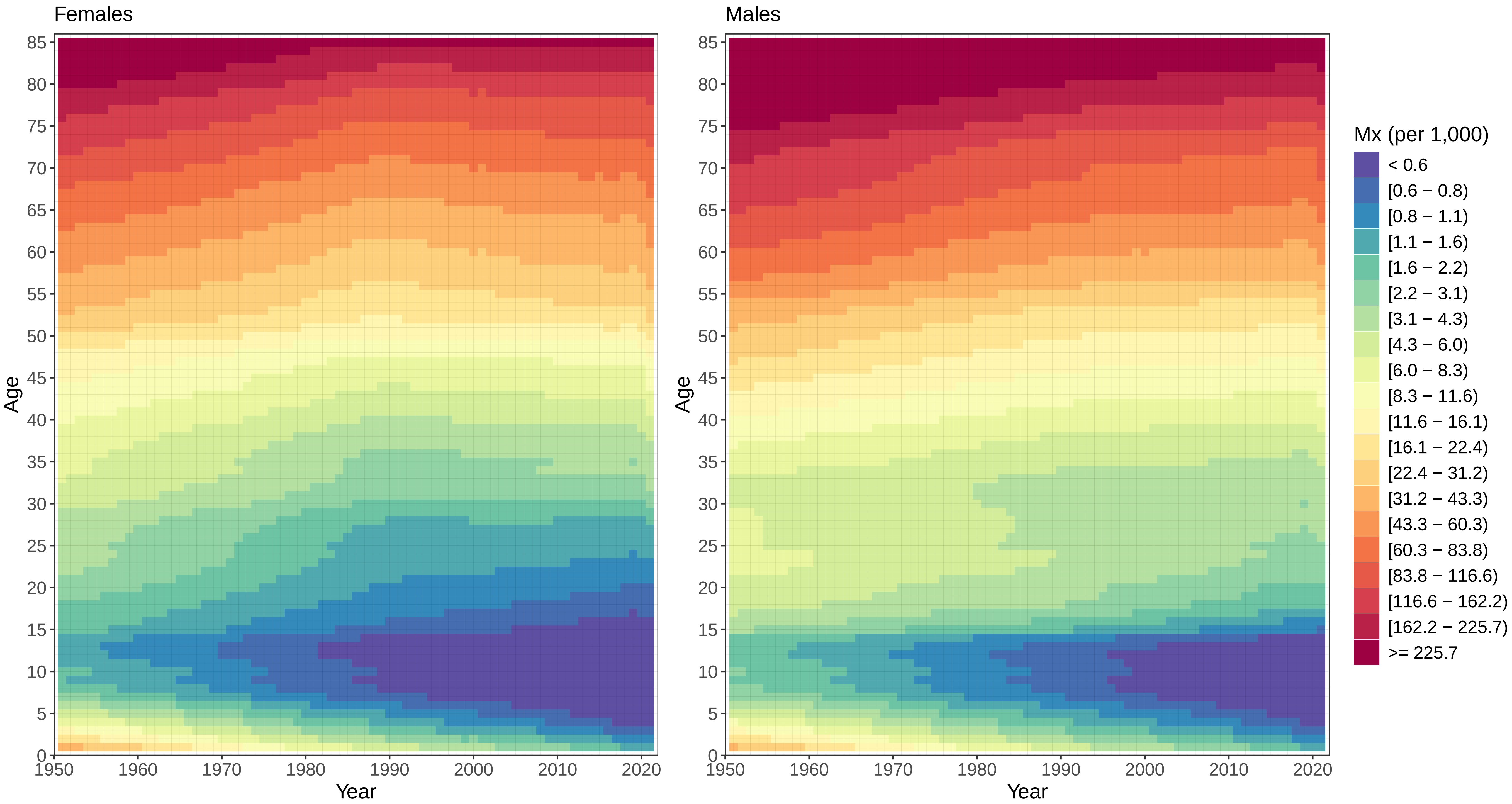

Appendix 2 Figure S4: Nauru mortality rates, 1950−2023

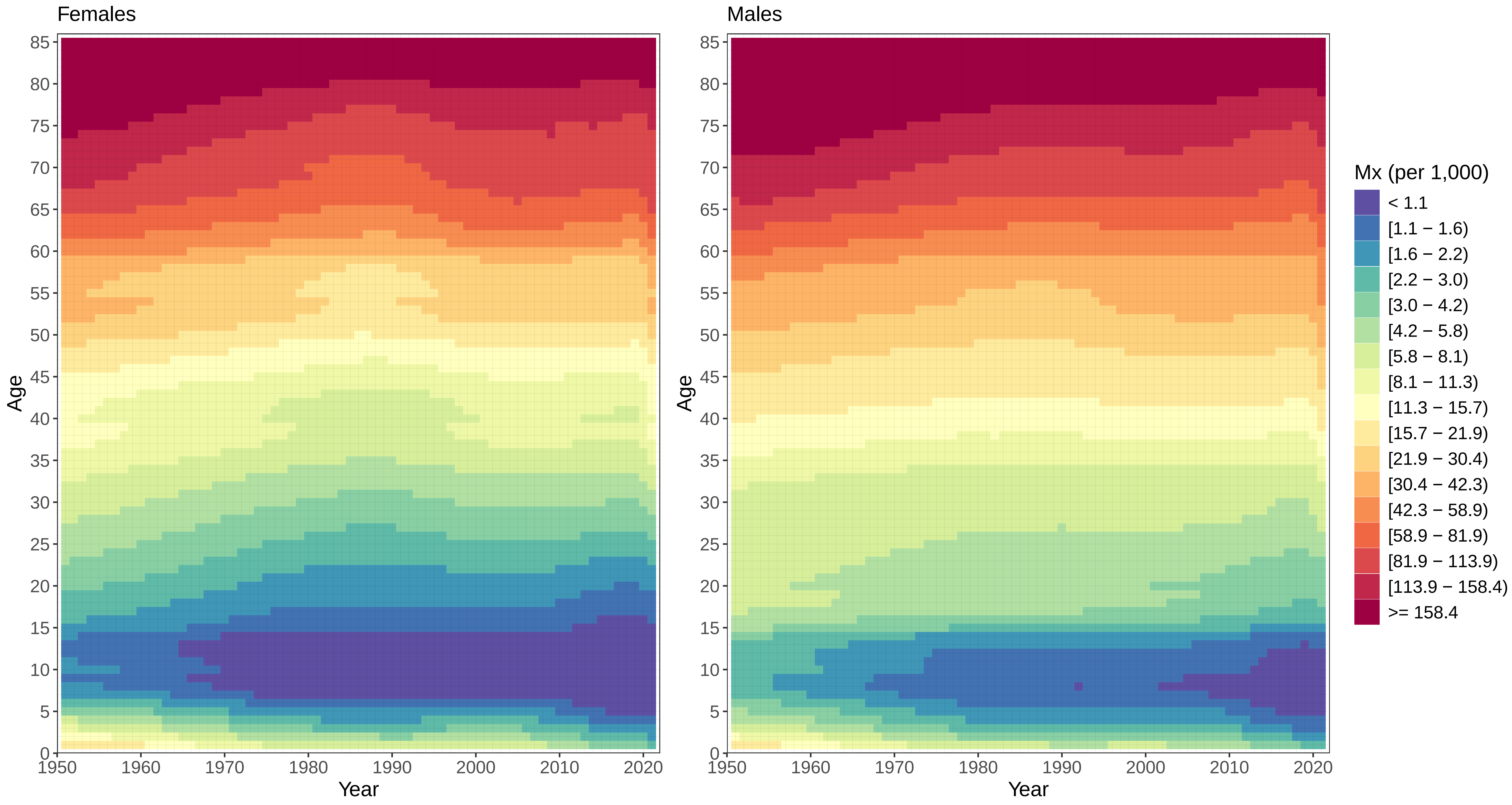

Appendix 2 Figure S4: Niue mortality rates, 1950−2023

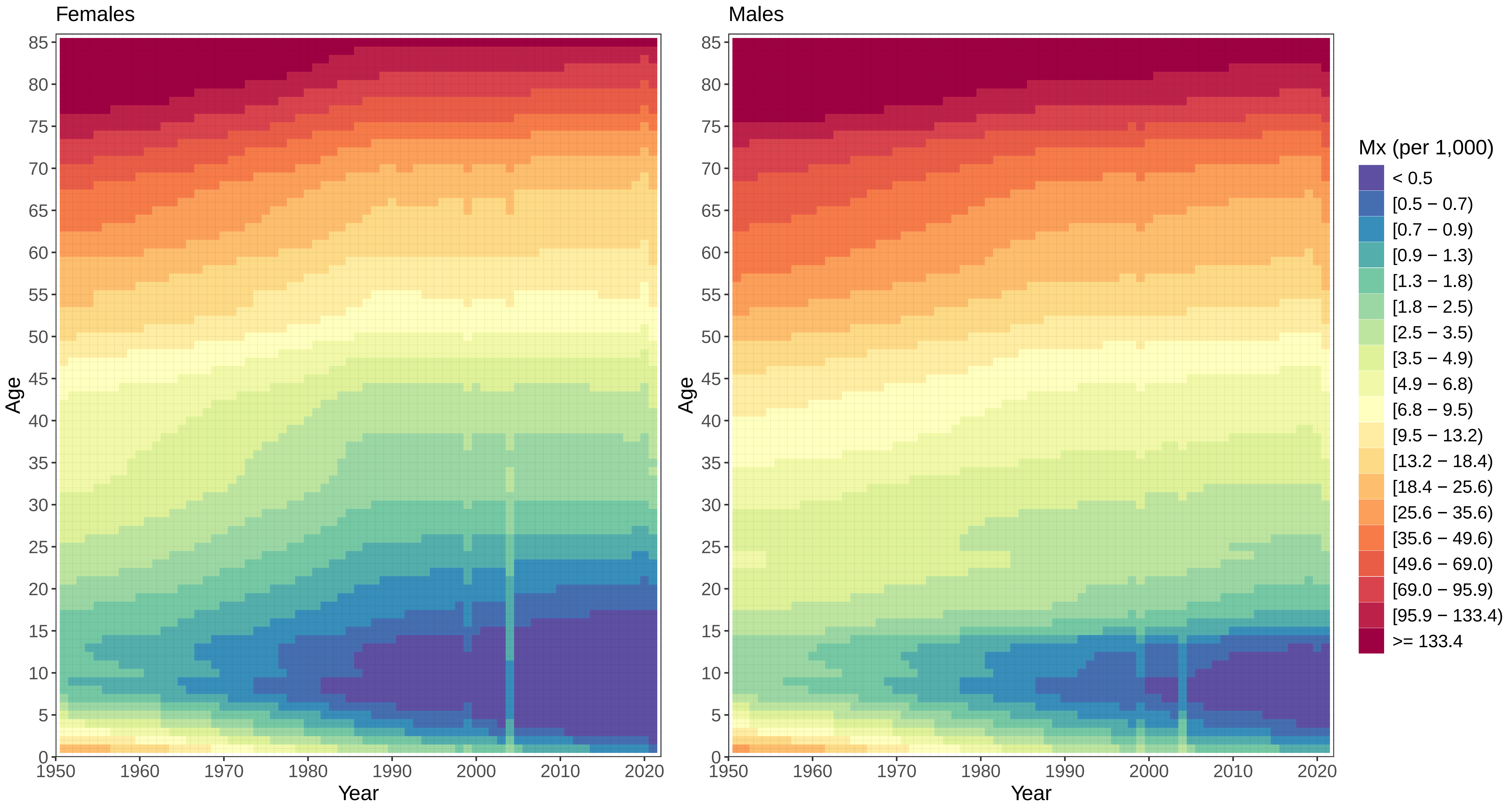

Appendix 2 Figure S4: Northern Mariana Islands mortality rates, 1950−2023

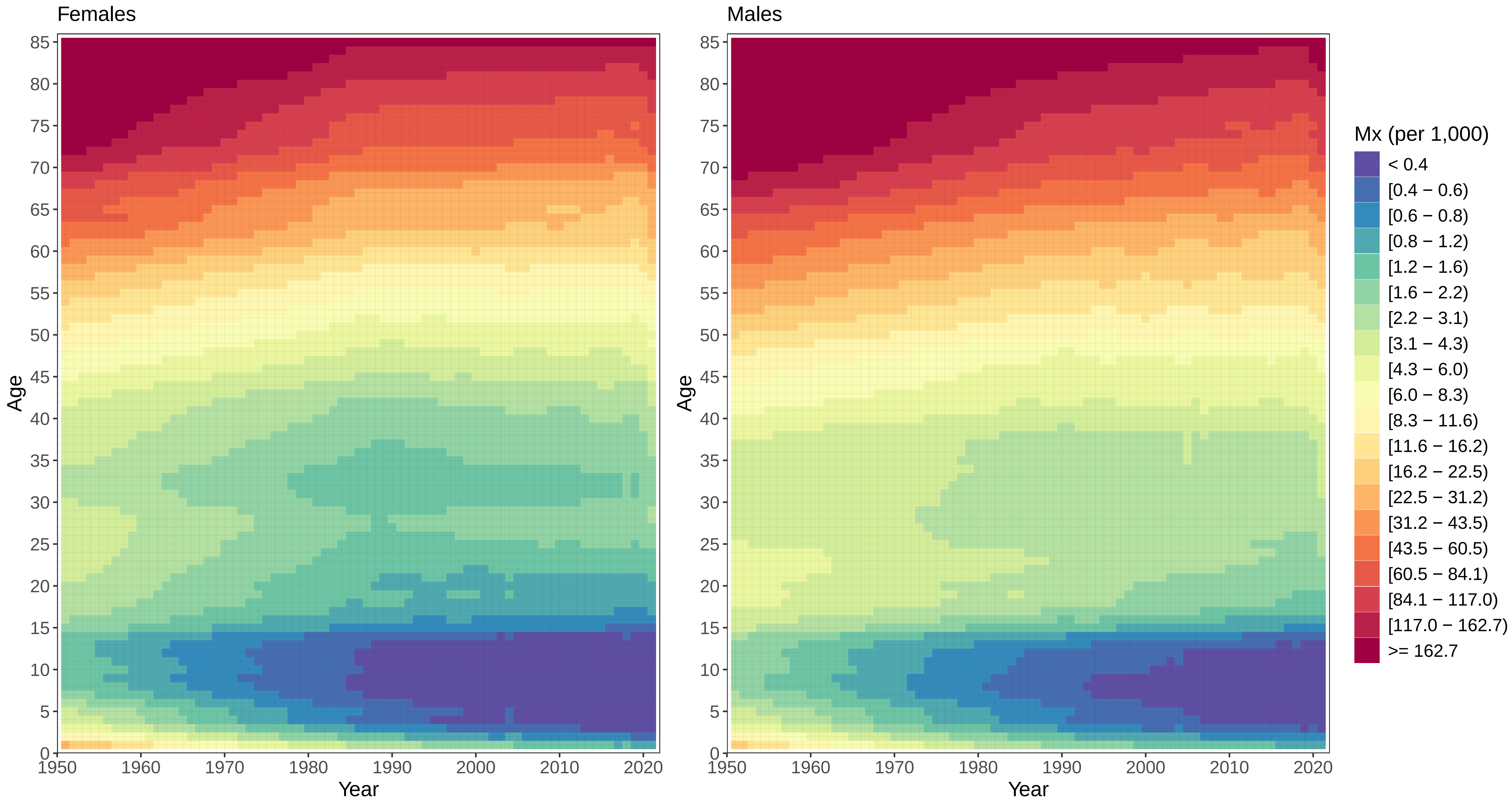

Appendix 2 Figure S4: Palau mortality rates, 1950−2023

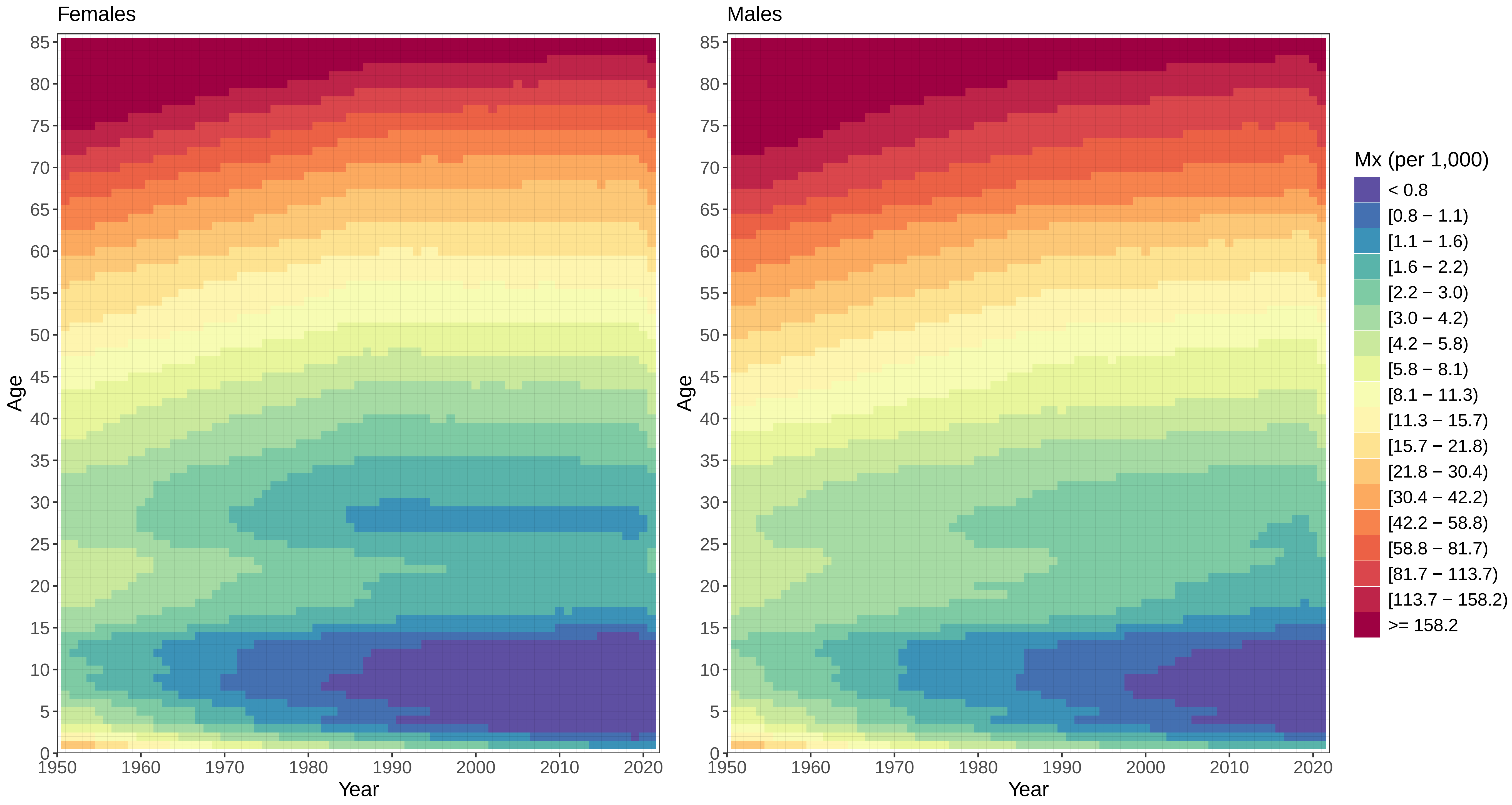

Appendix 2 Figure S4: Papua New Guinea mortality rates, 1950−2023

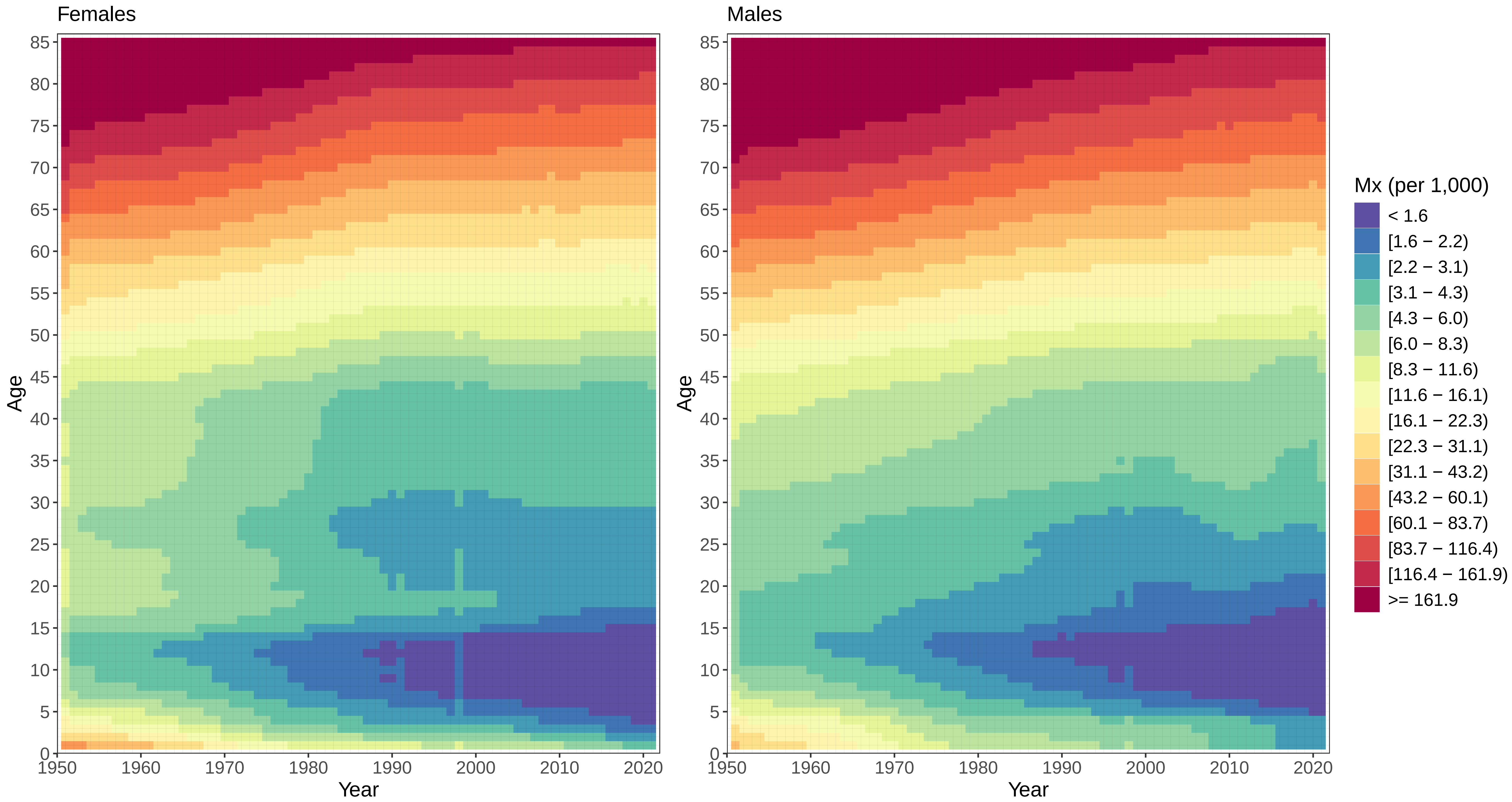

Appendix 2 Figure S4: Samoa mortality rates, 1950−2023

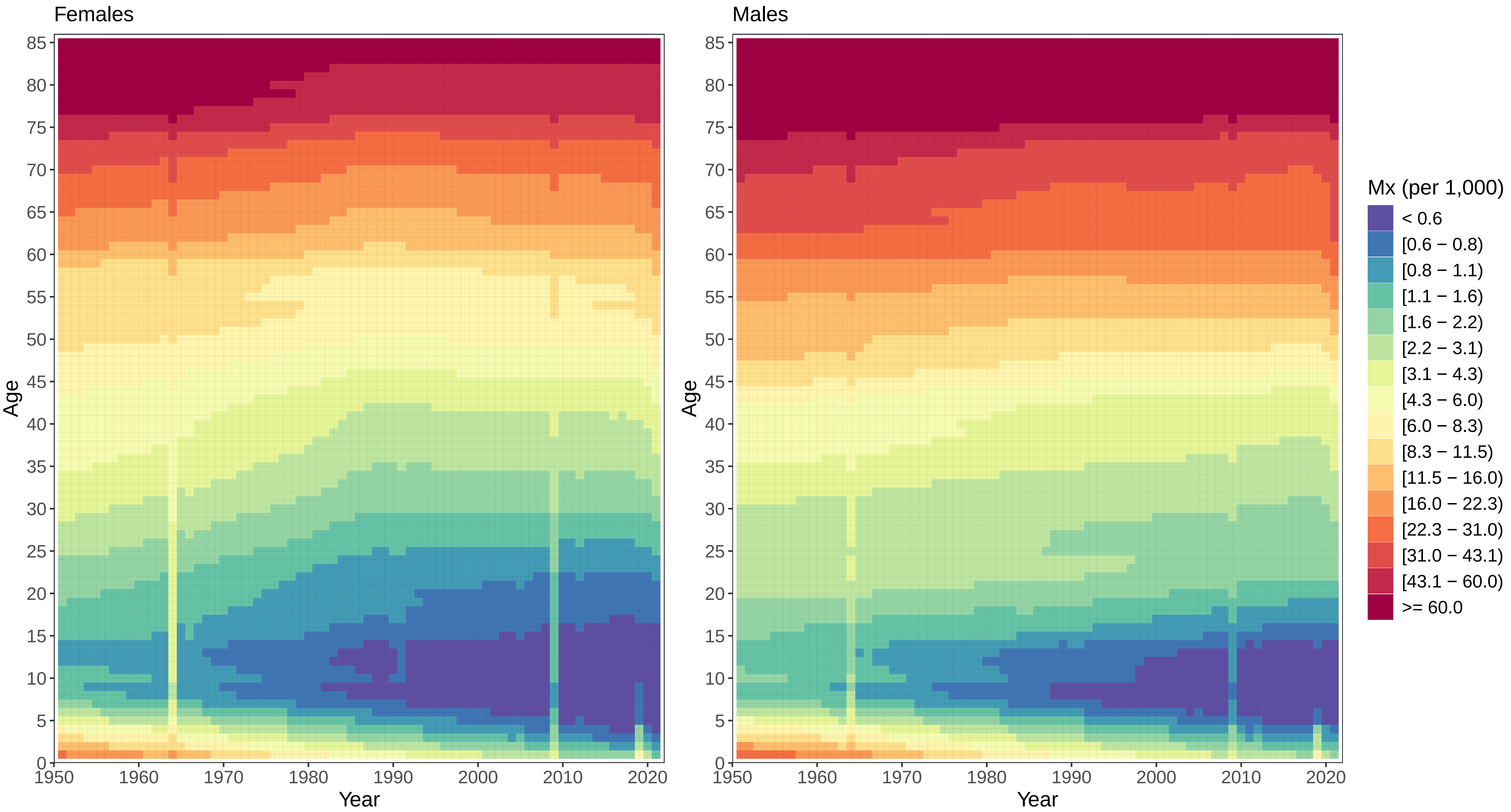

Appendix 2 Figure S4: Solomon Islands mortality rates, 1950−2023

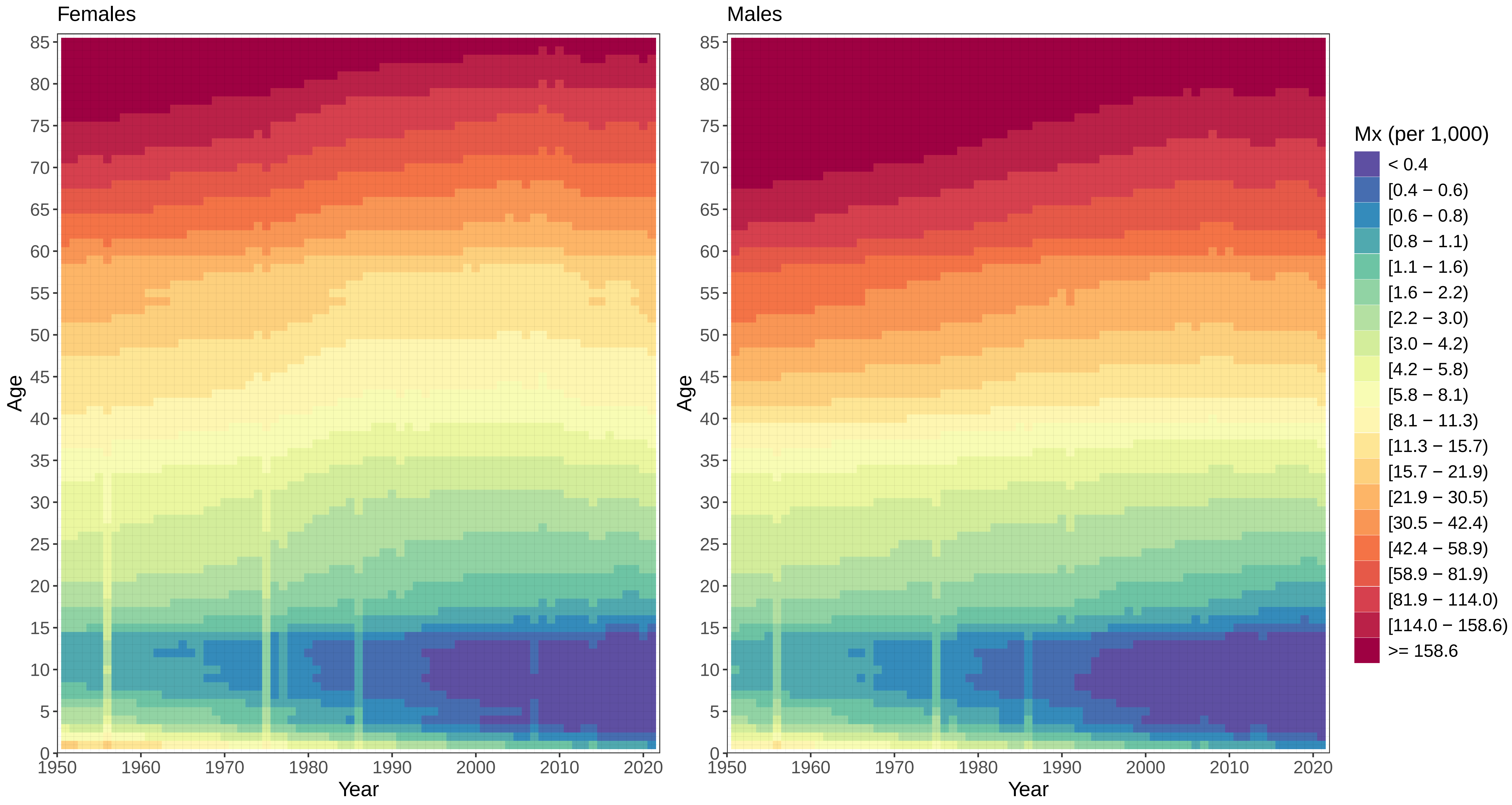

Appendix 2 Figure S4: Tokelau mortality rates, 1950−2023

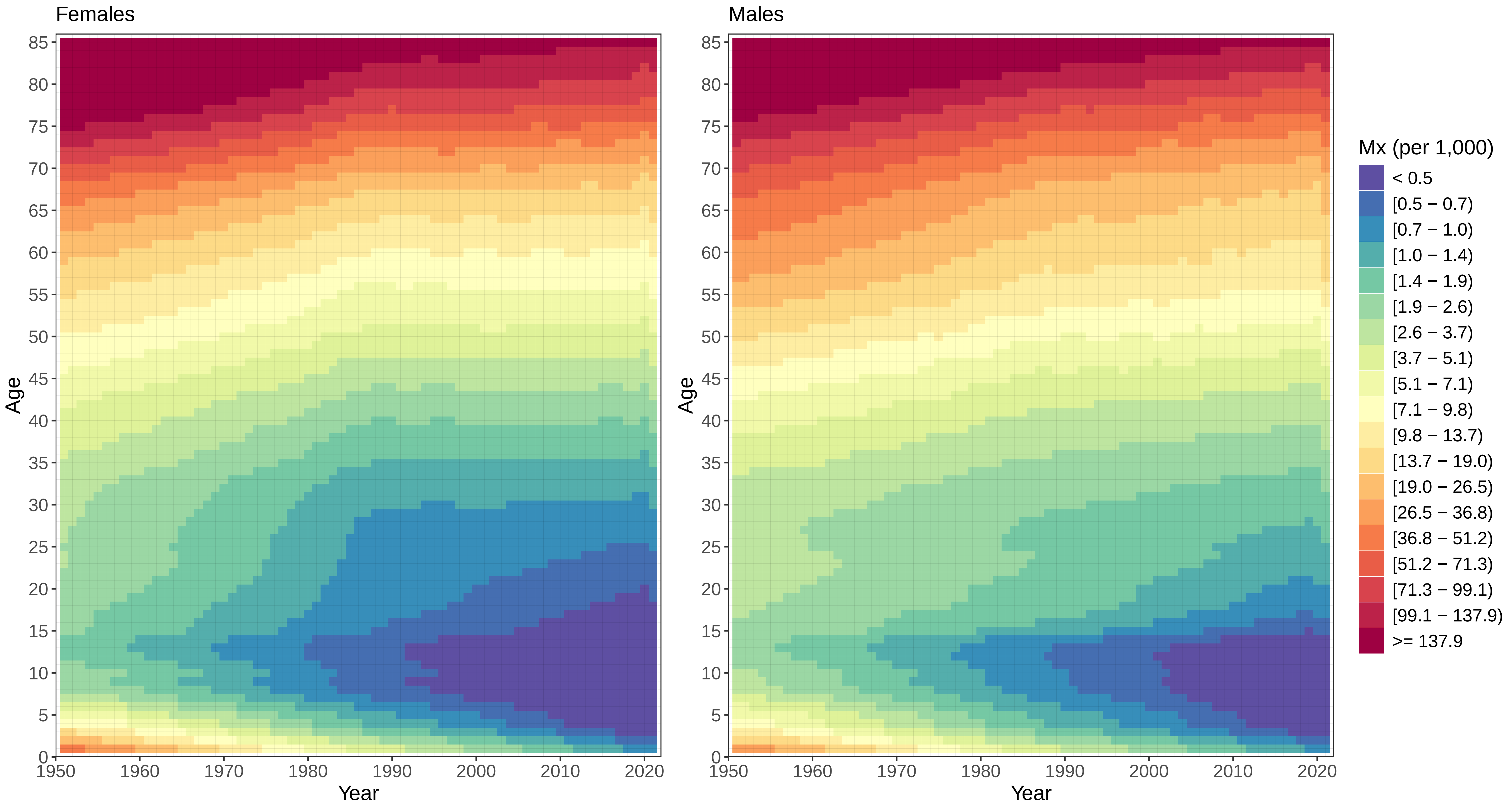

Appendix 2 Figure S4: Tonga mortality rates, 1950−2023

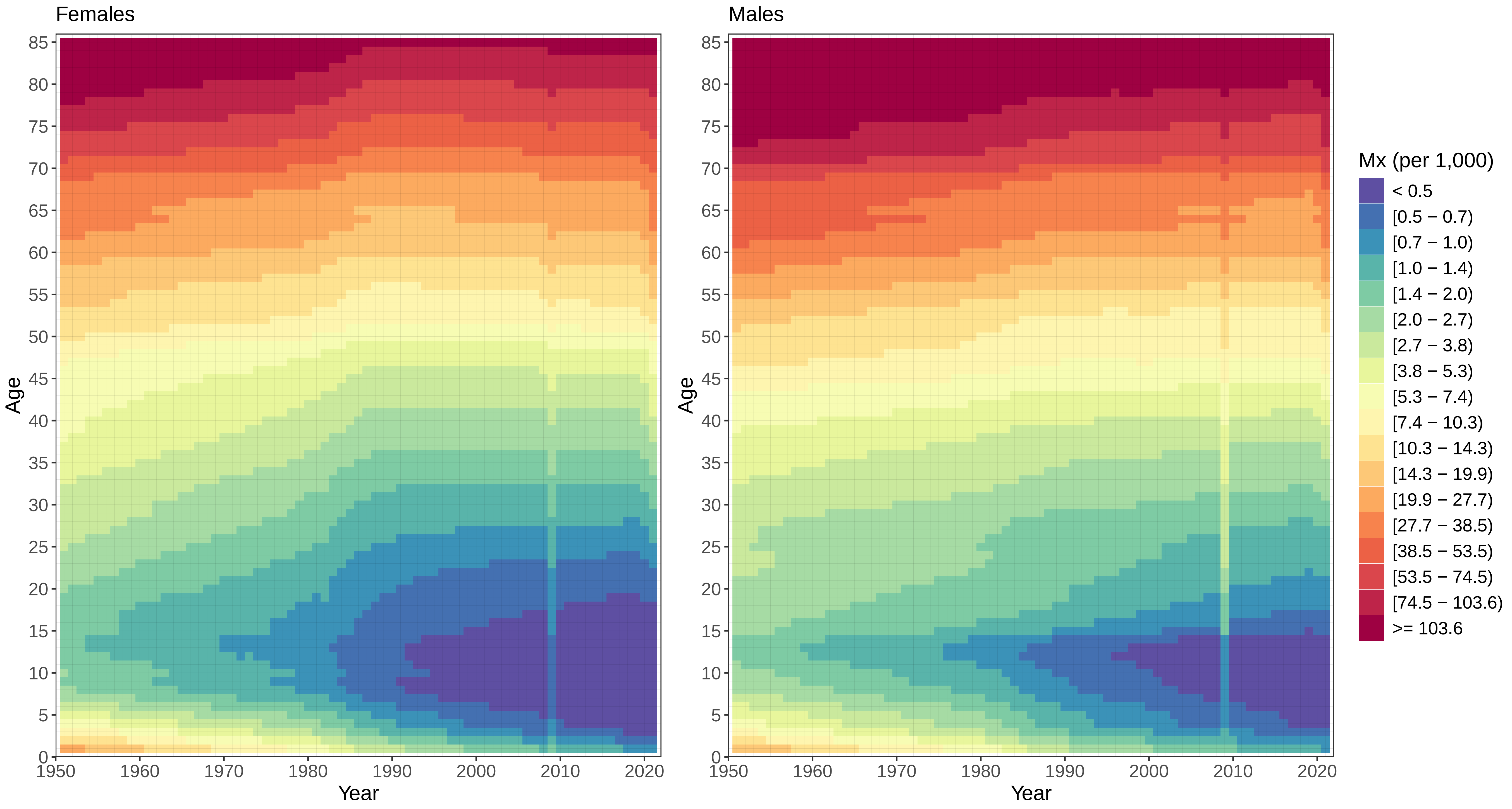

Appendix 2 Figure S4: Tuvalu mortality rates, 1950−2023

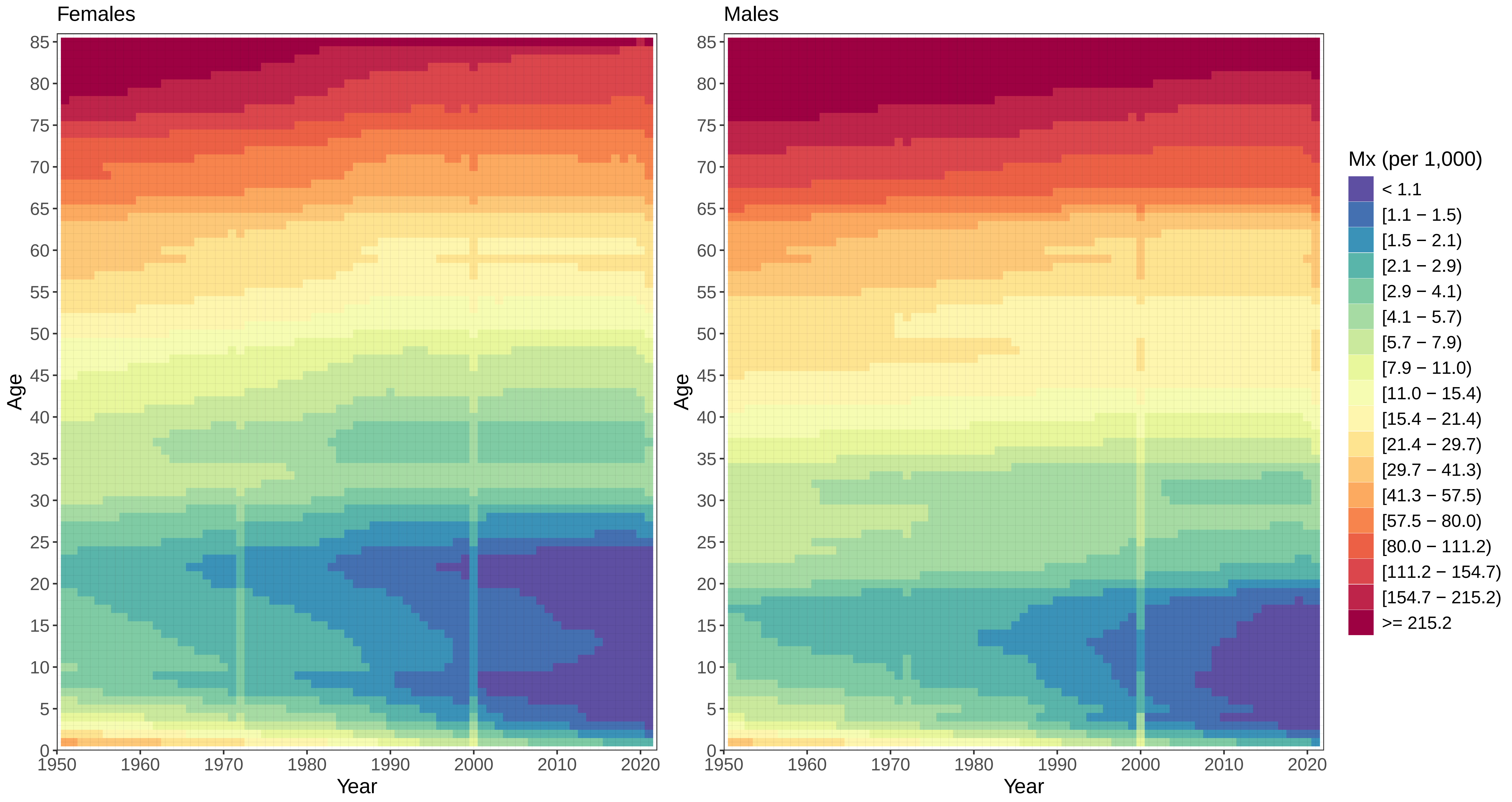

Appendix 2 Figure S4: Vanuatu mortality rates, 1950−2023

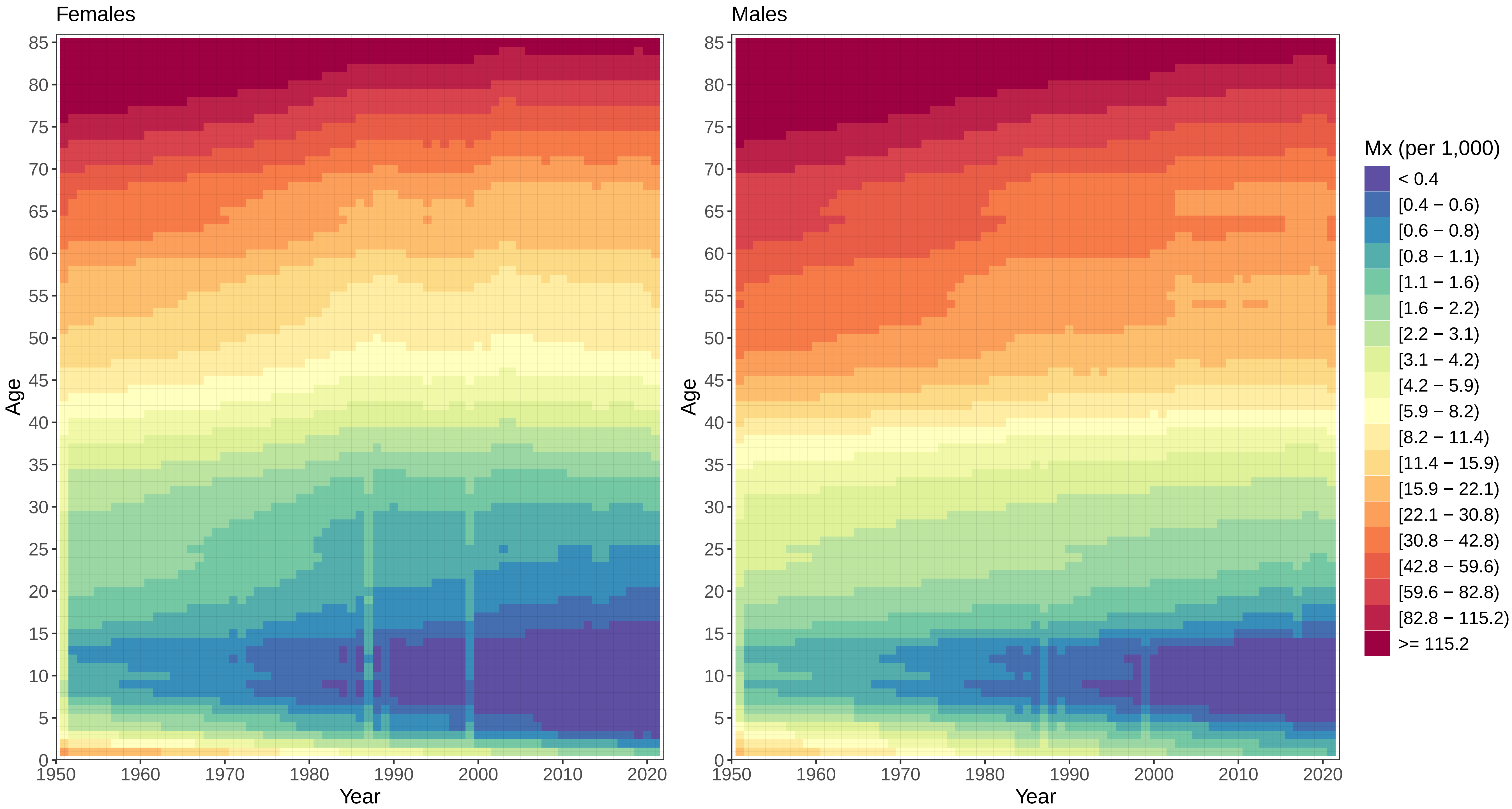

Appendix 2 Figure S4: Cambodia mortality rates, 1950−2023

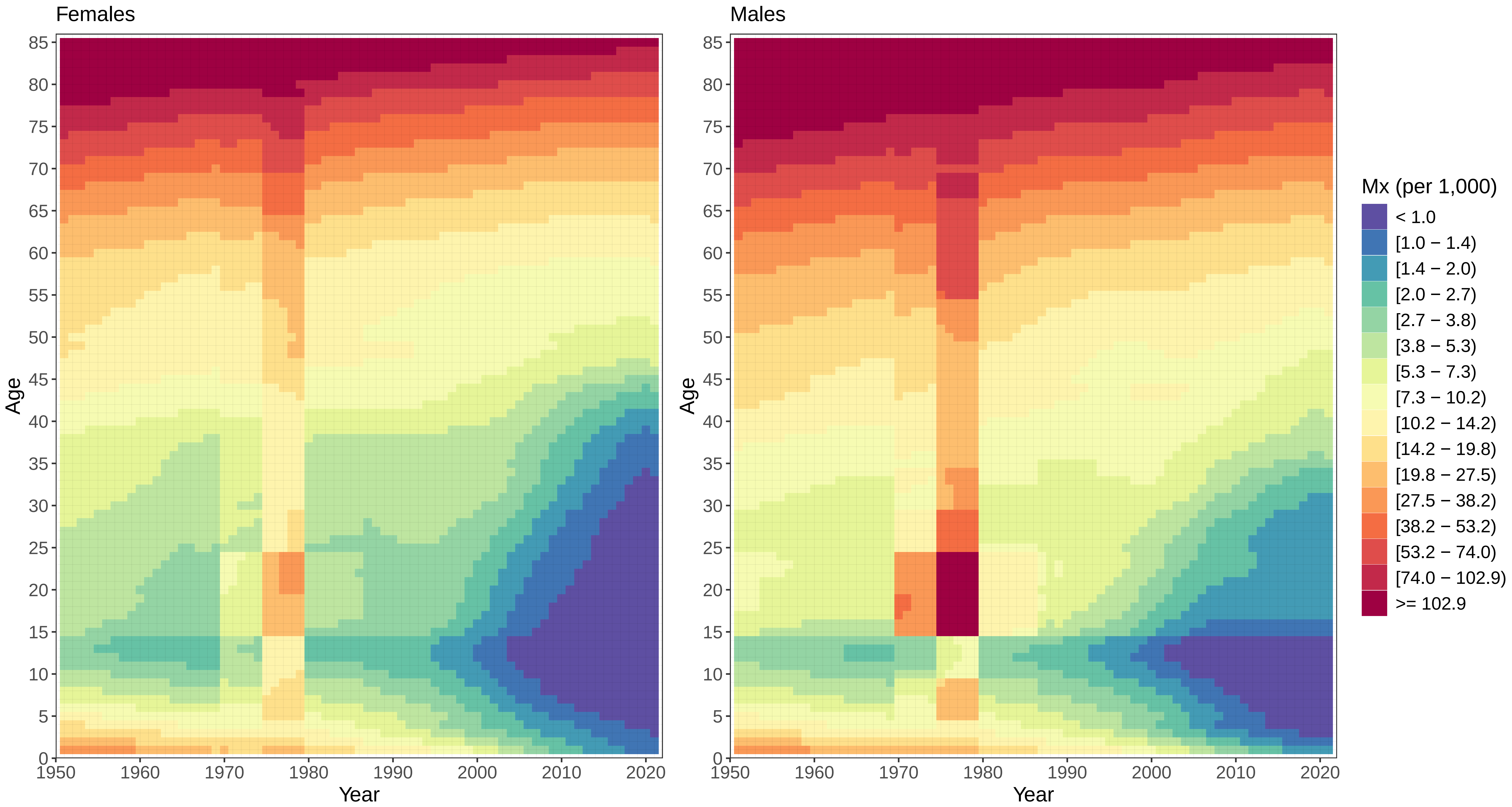

Appendix 2 Figure S4: Indonesia mortality rates, 1950−2023

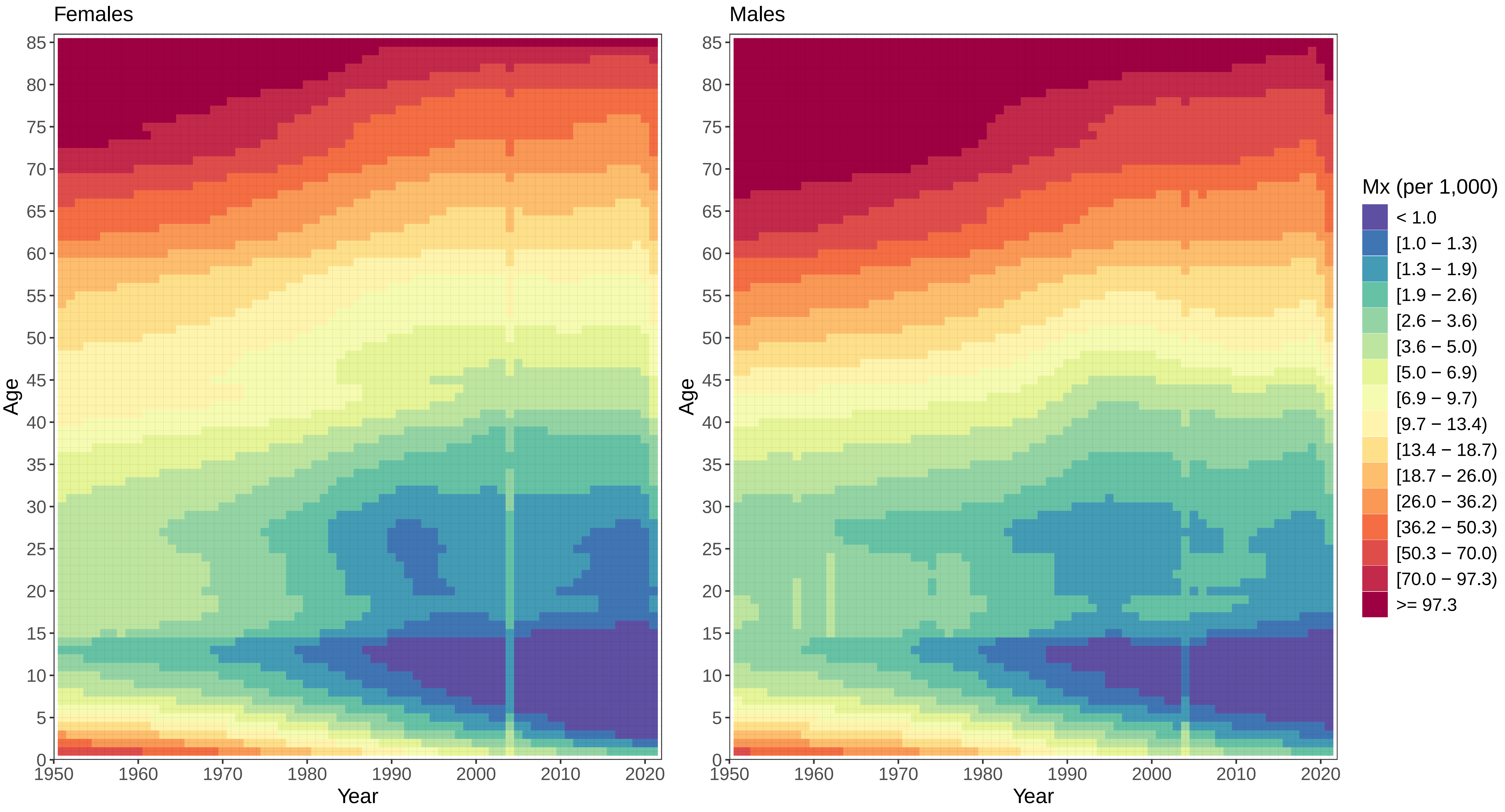

Appendix 2 Figure S4: Laos mortality rates, 1950−2023

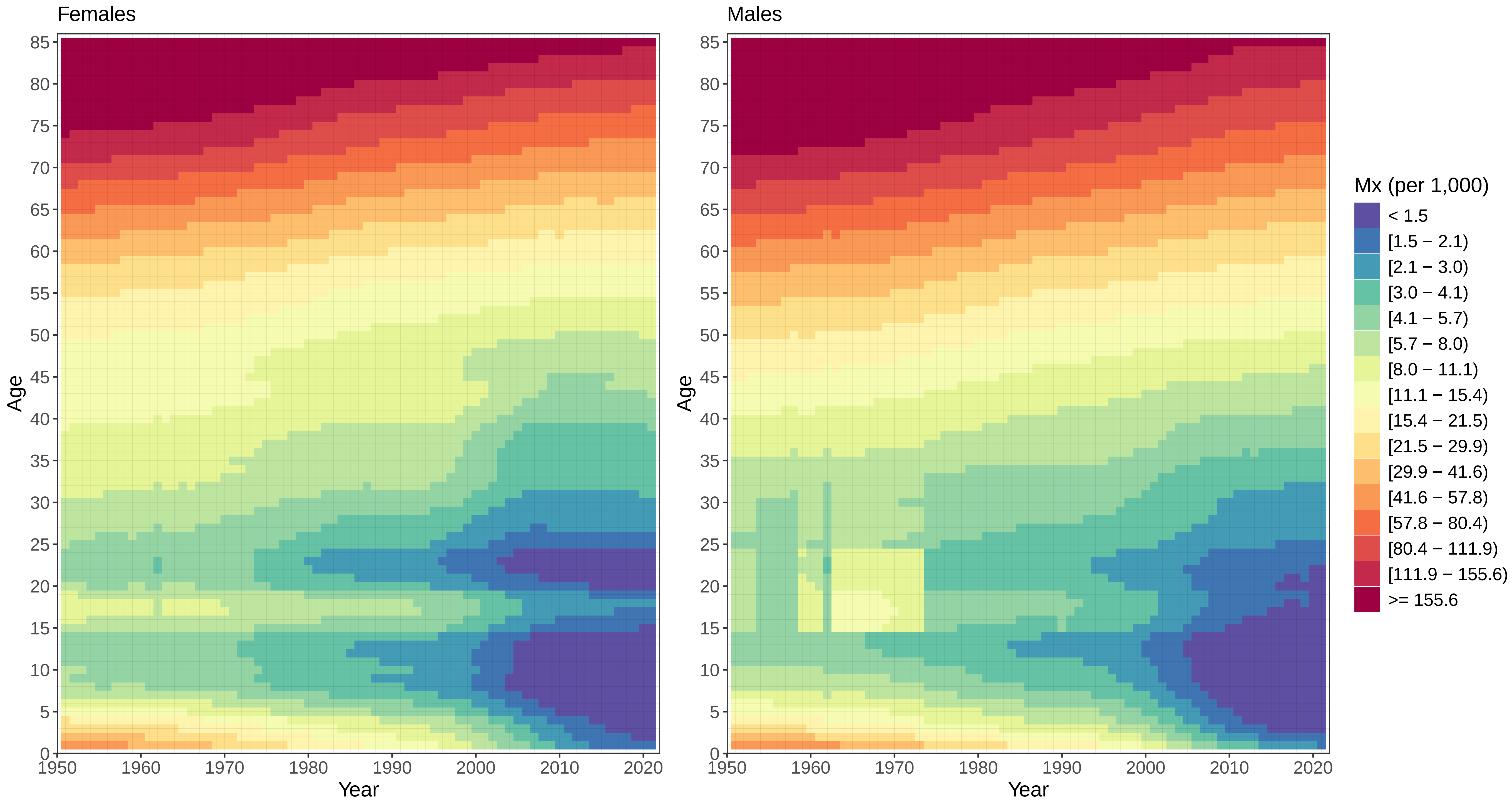

Appendix 2 Figure S4: Malaysia mortality rates, 1950−2023

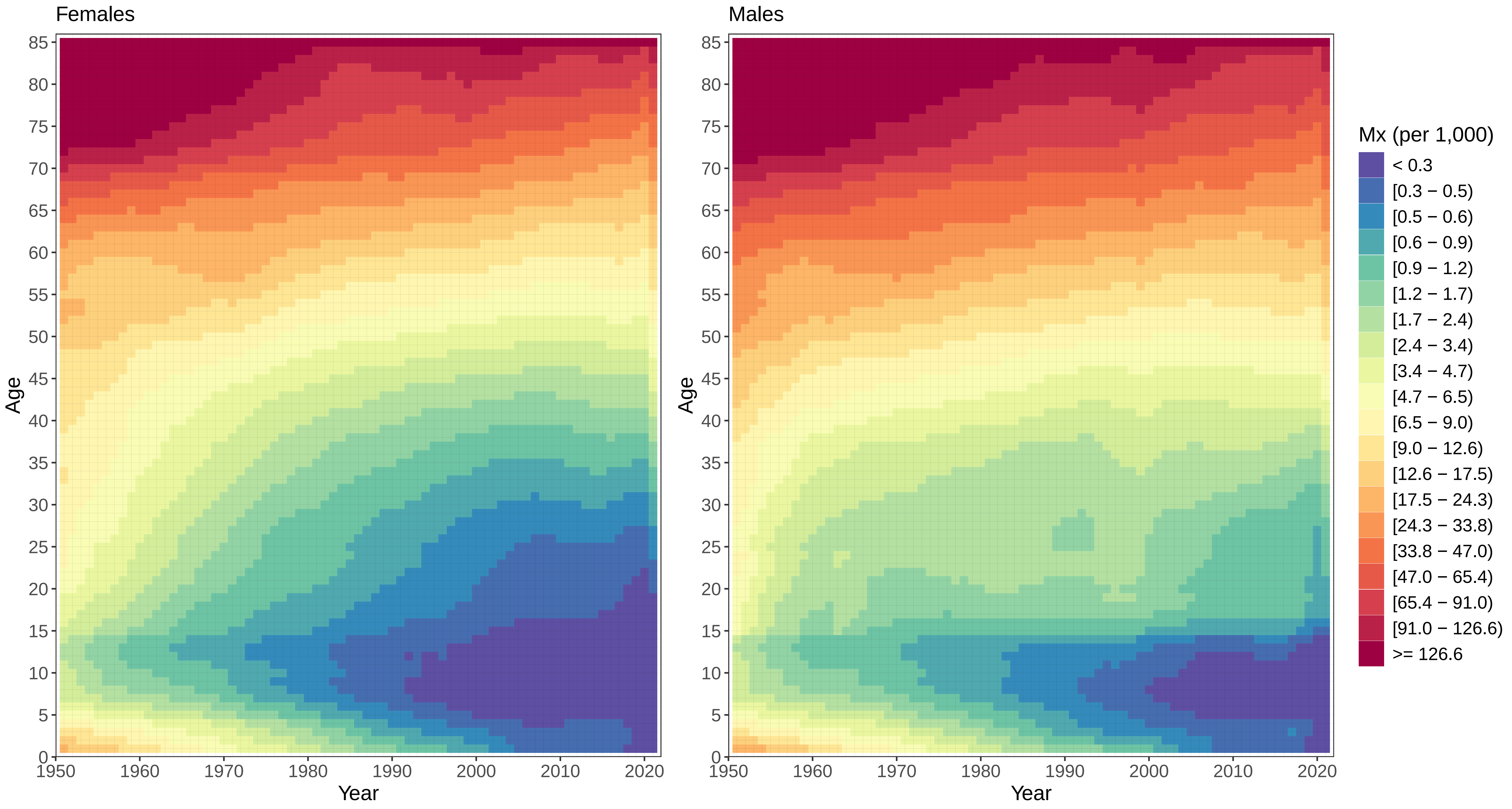

Appendix 2 Figure S4: Maldives mortality rates, 1950−2023

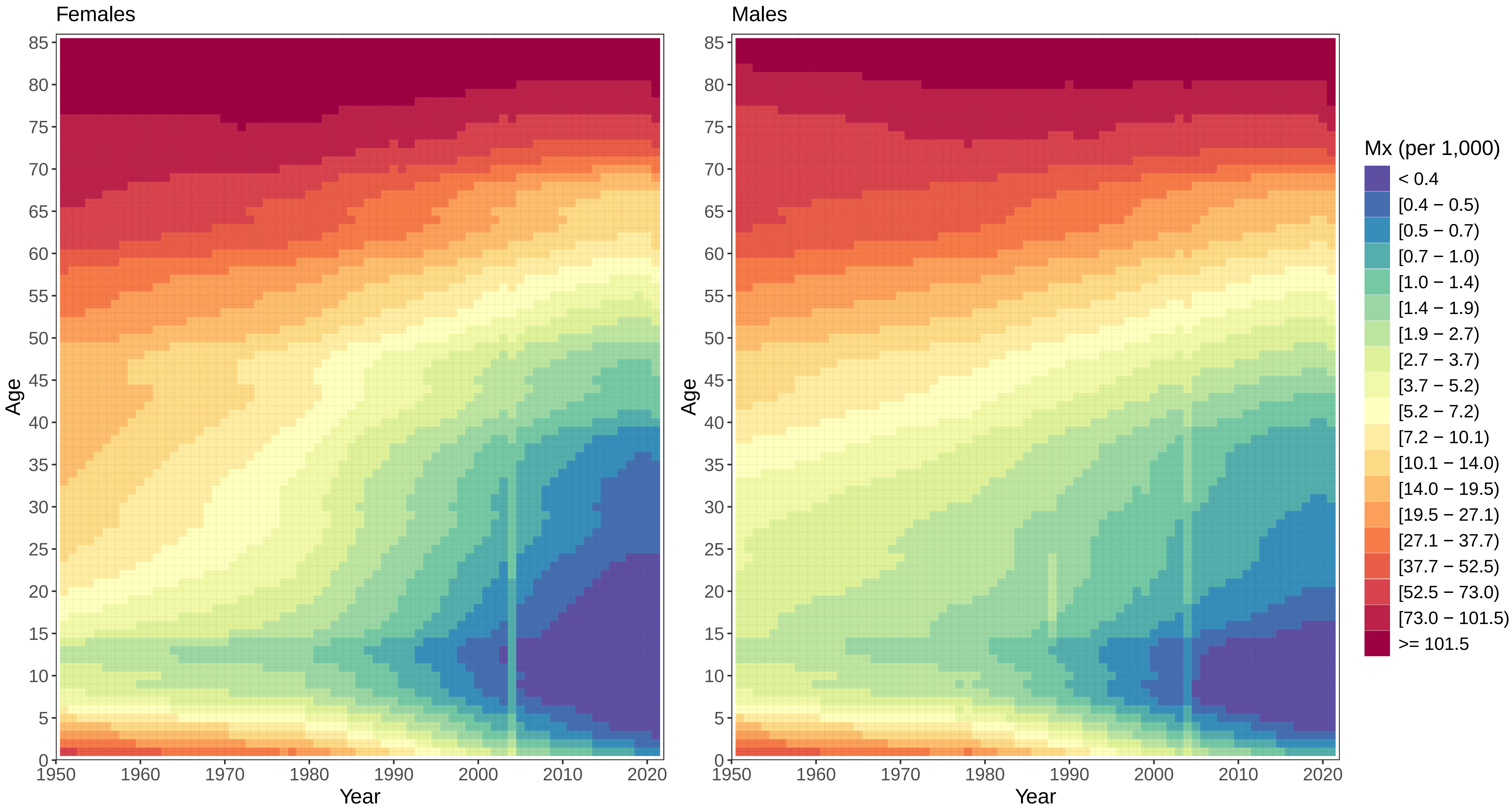

Appendix 2 Figure S4: Mauritius mortality rates, 1950−2023

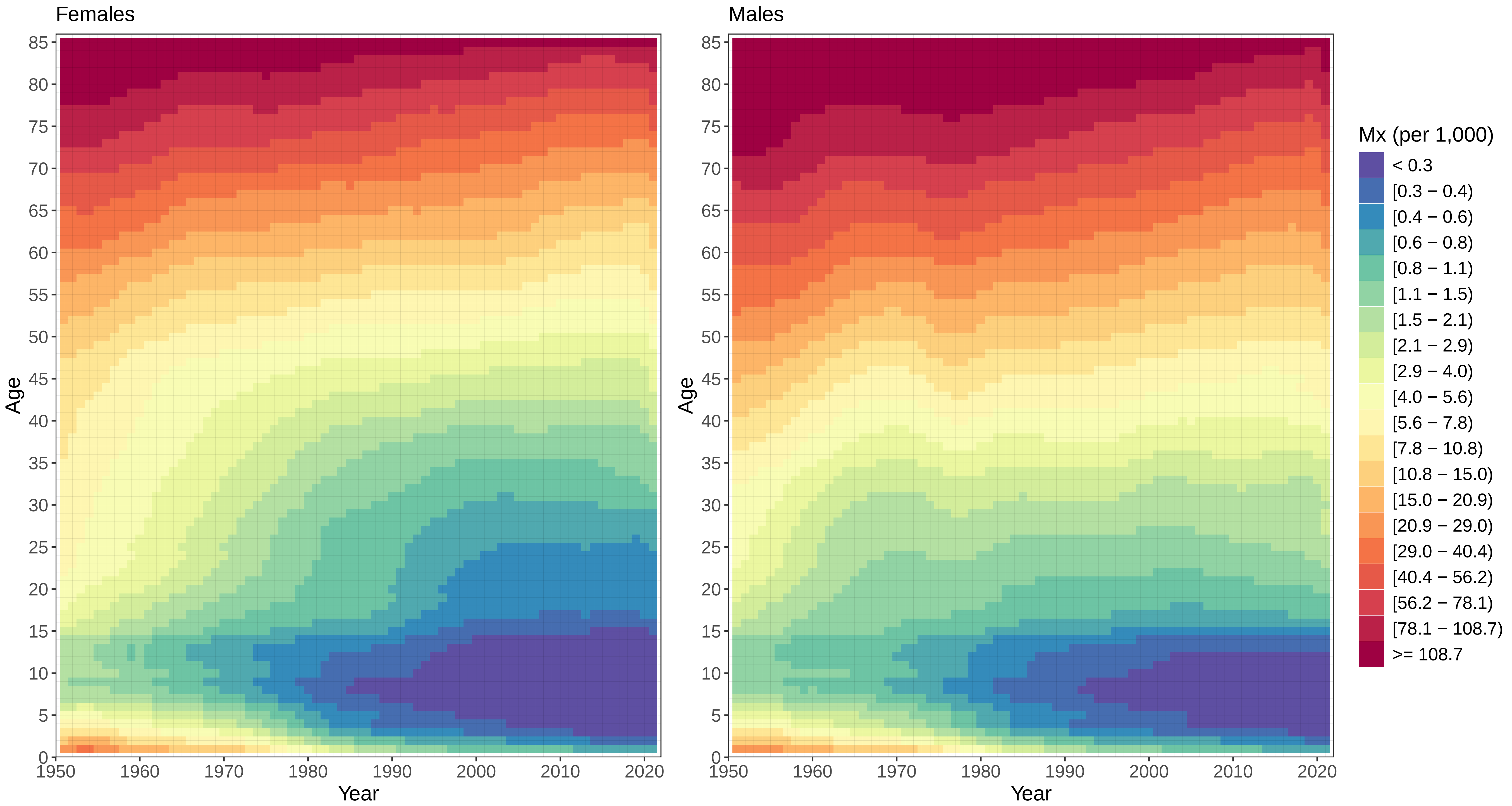

Appendix 2 Figure S4: Myanmar mortality rates, 1950−2023

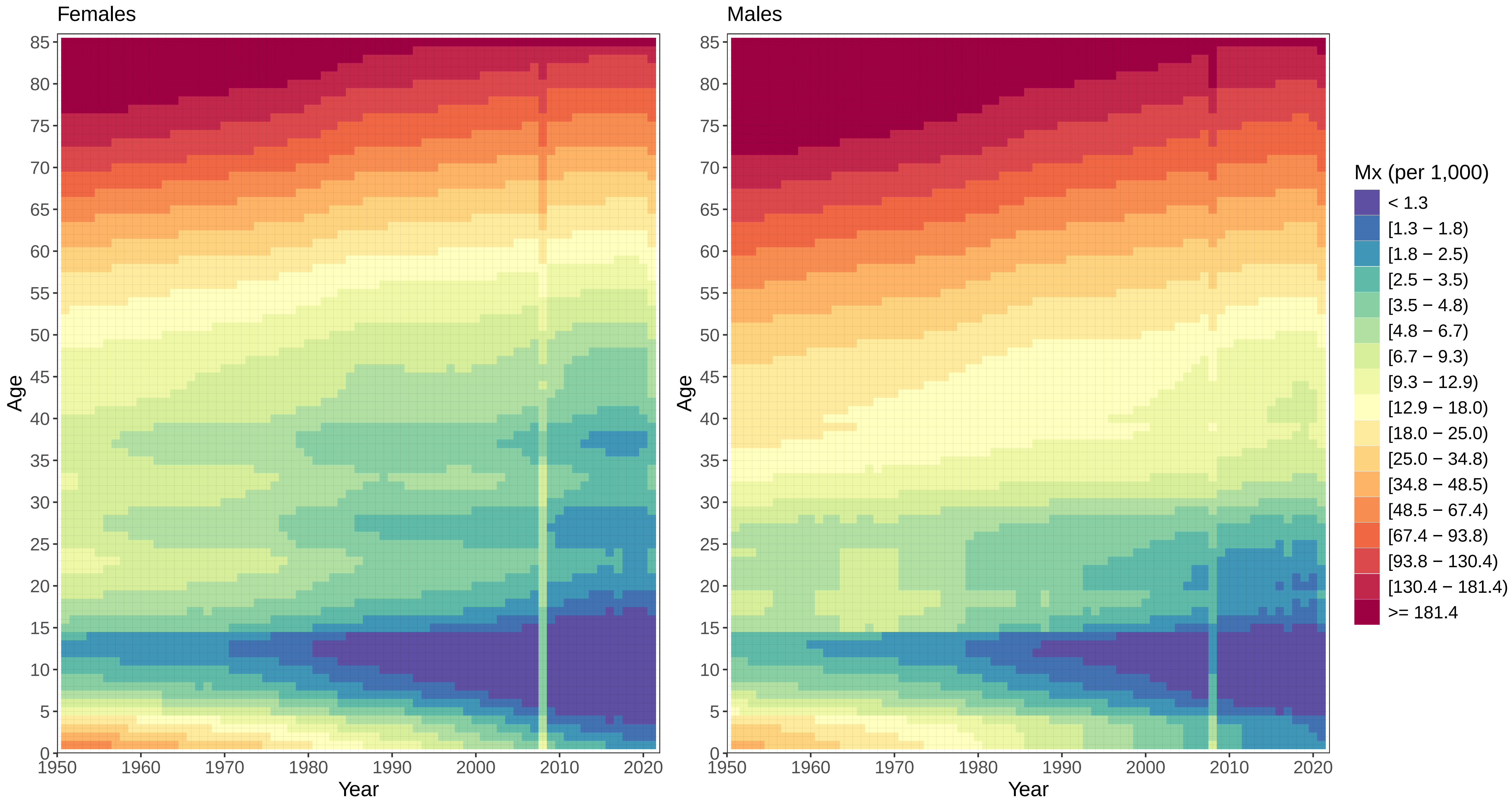

Appendix 2 Figure S4: Philippines mortality rates, 1950−2023

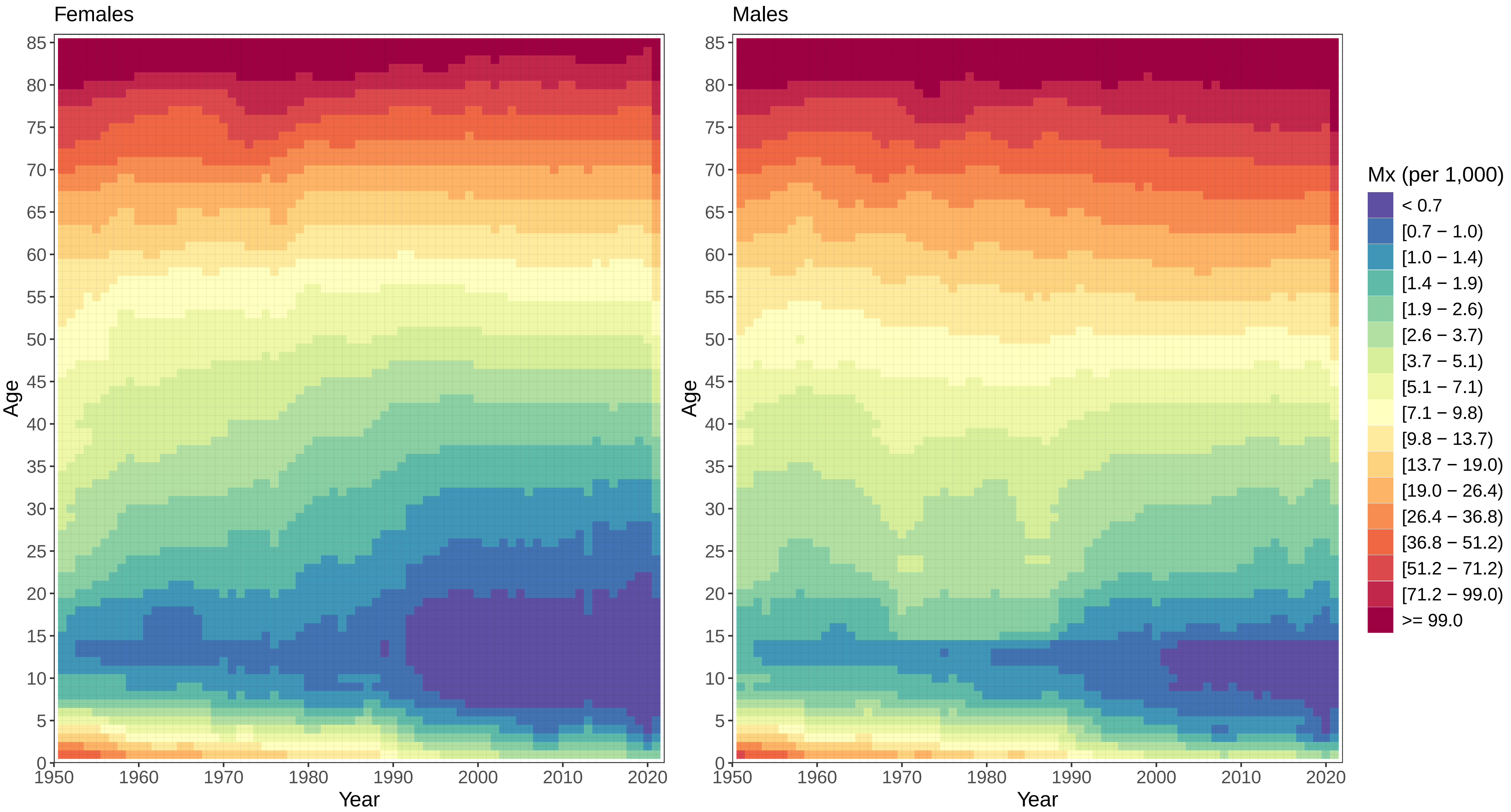

Appendix 2 Figure S4: Seychelles mortality rates, 1950−2023

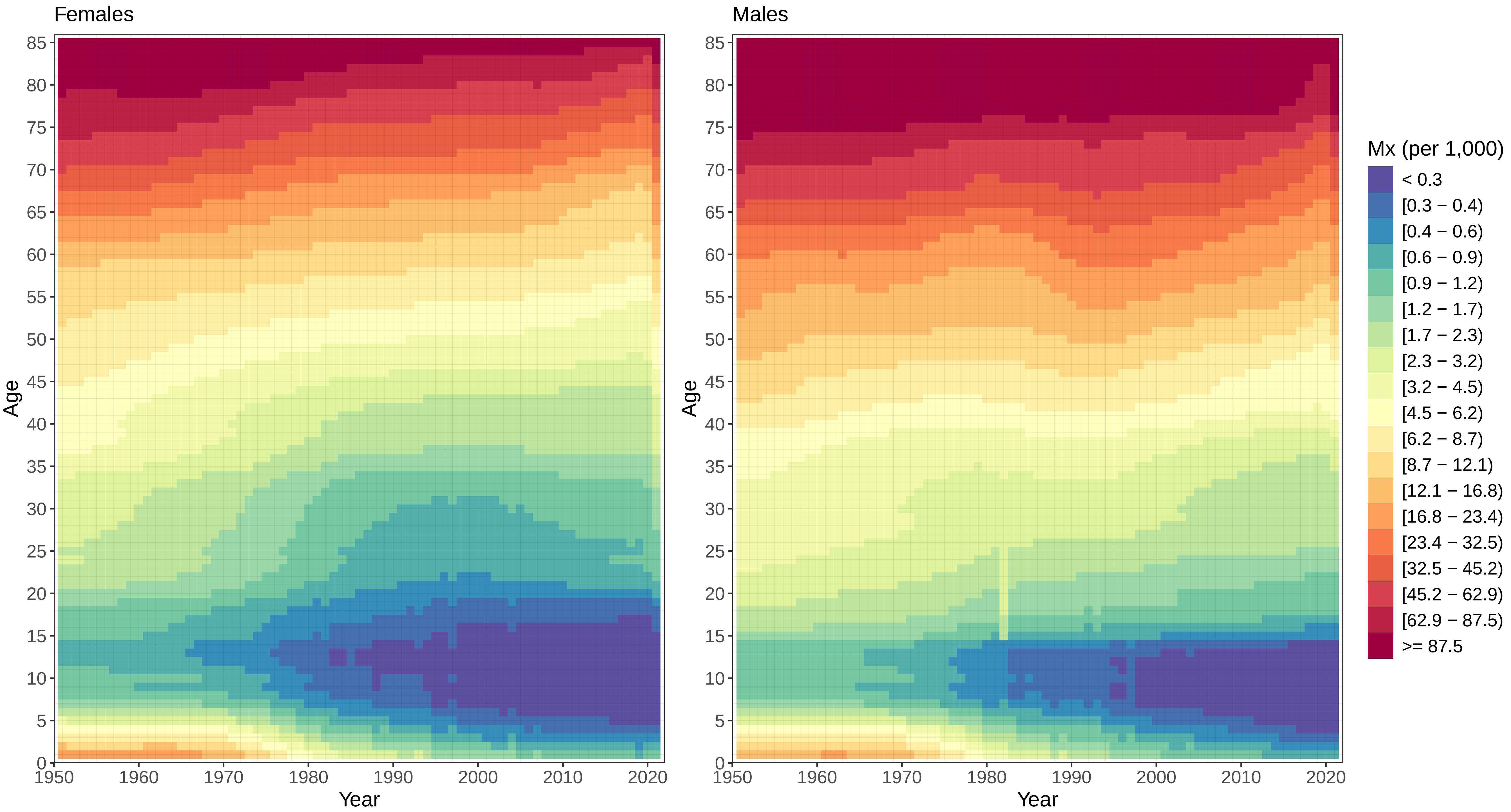

Appendix 2 Figure S4: Sri Lanka mortality rates, 1950−2023

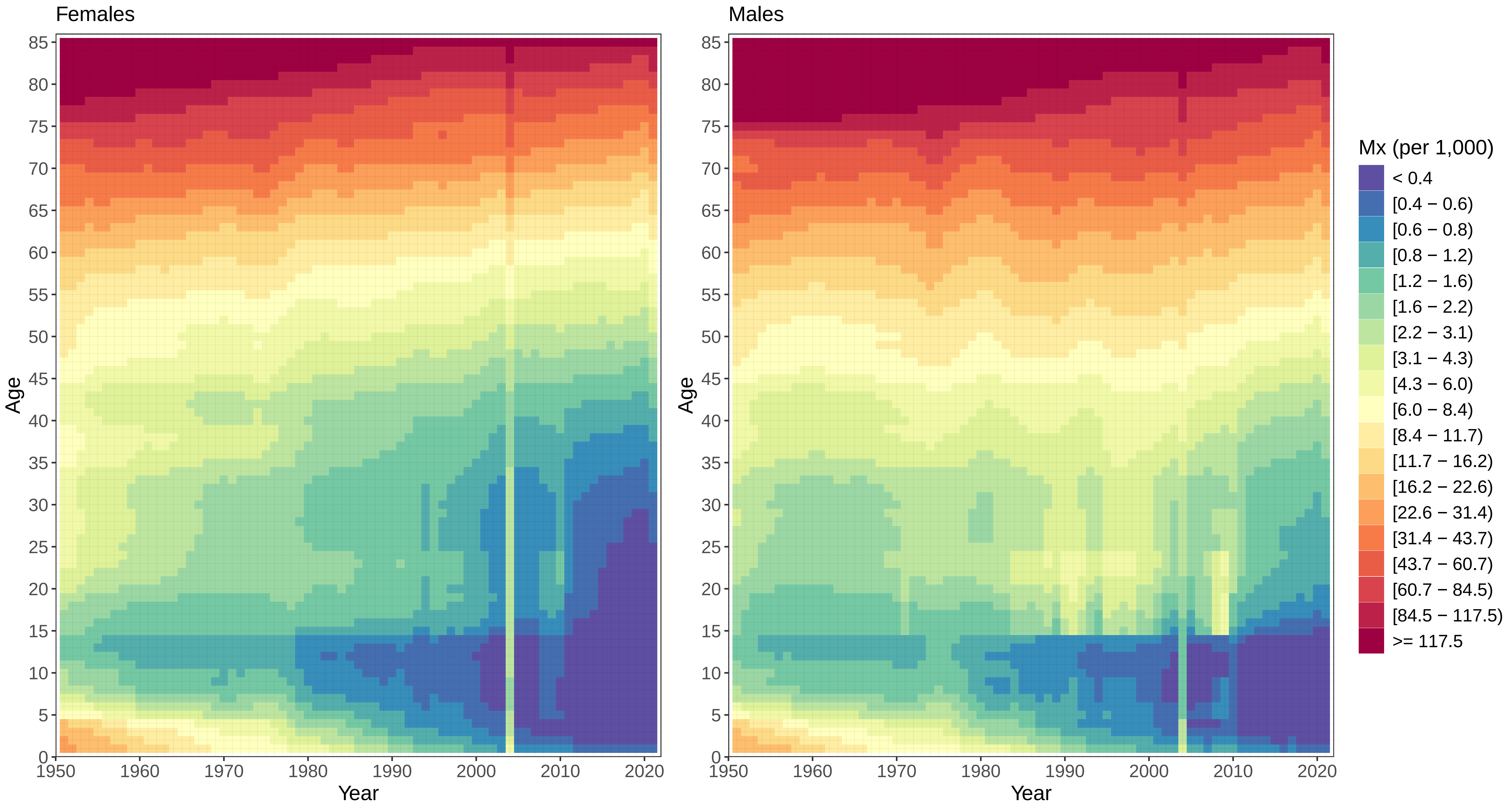

Appendix 2 Figure S4: Thailand mortality rates, 1950−2023

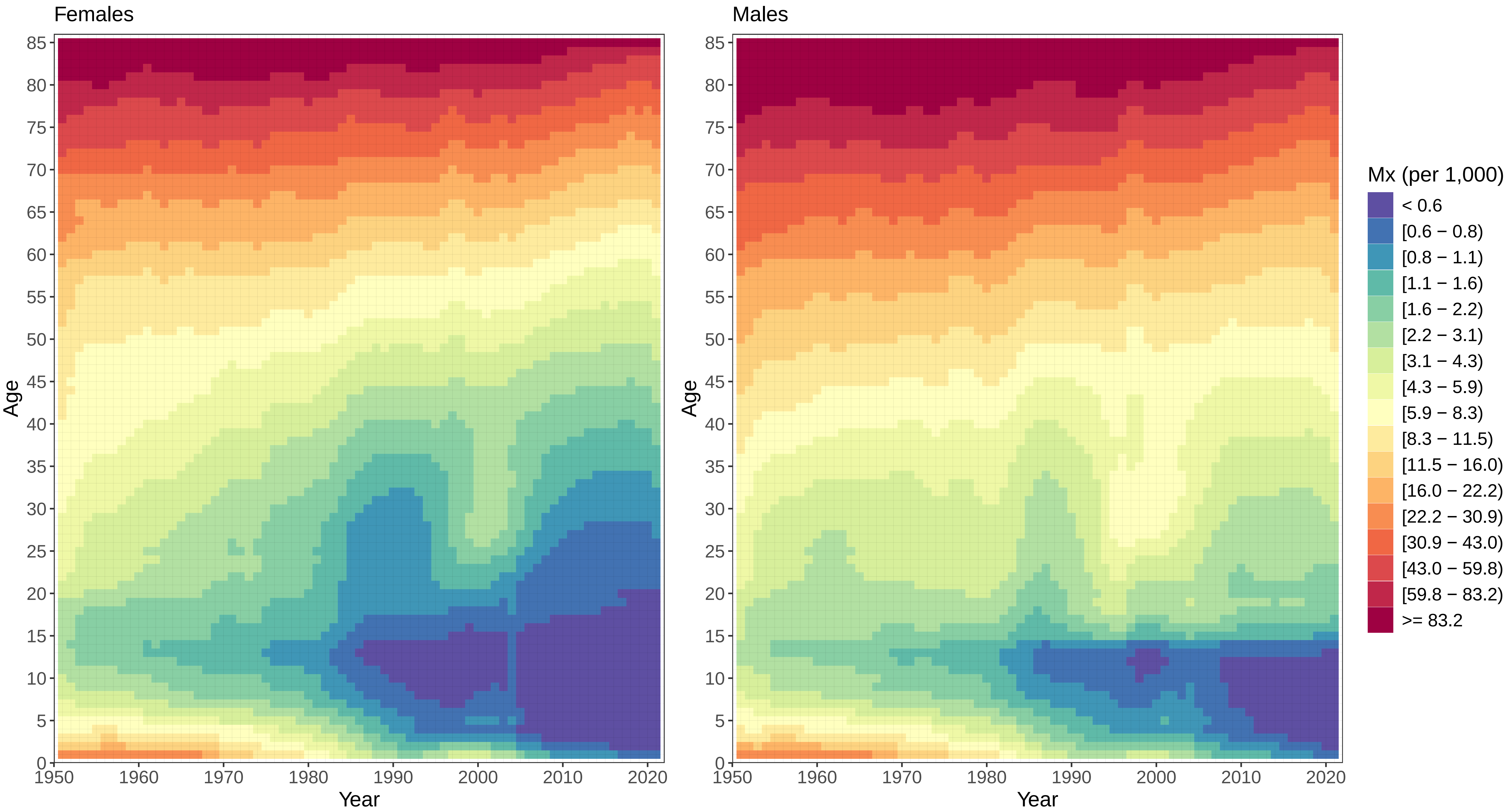

Appendix 2 Figure S4: Timor−Leste mortality rates, 1950−2023

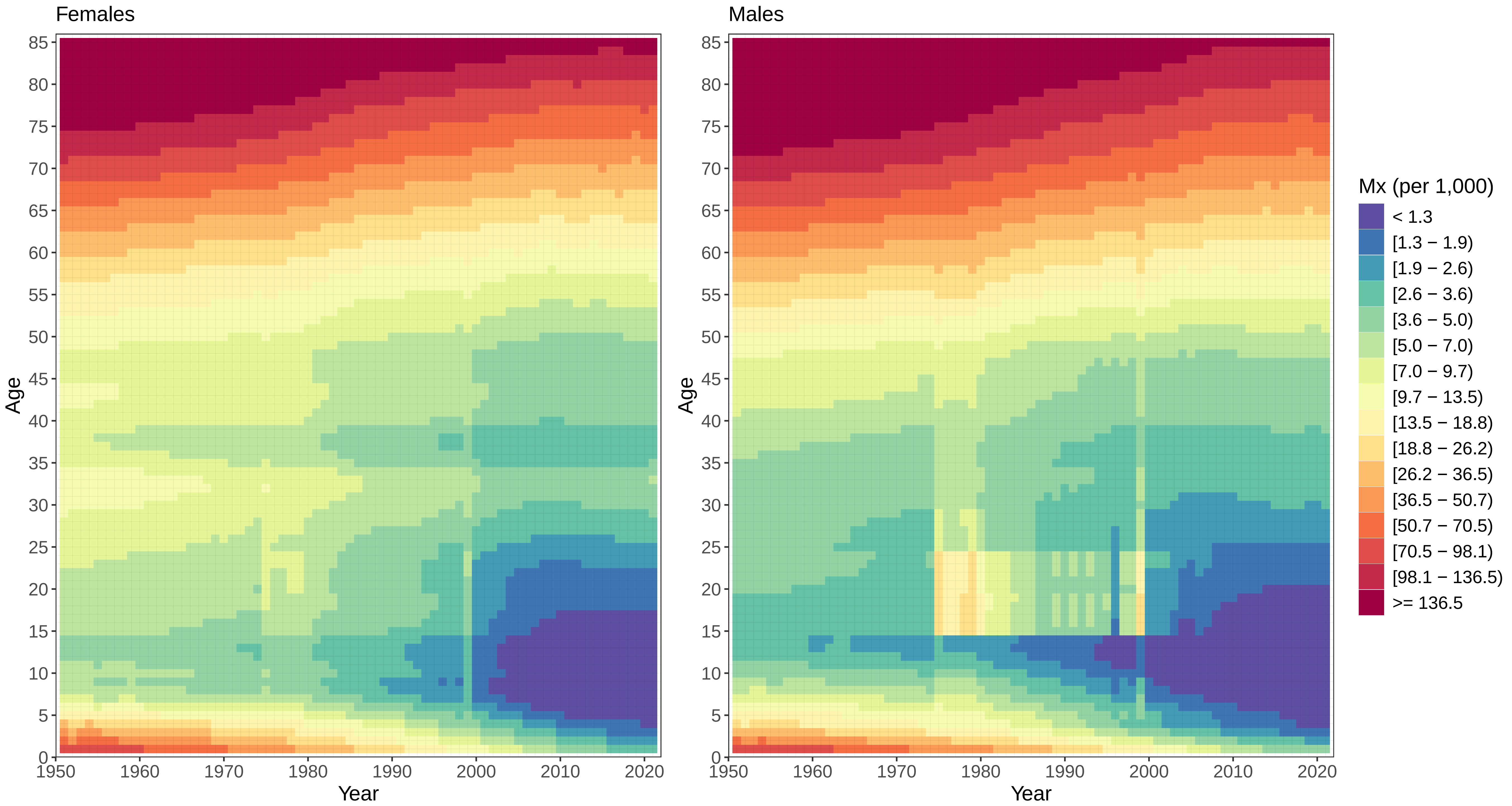

Appendix 2 Figure S4: Viet Nam mortality rates, 1950−2023

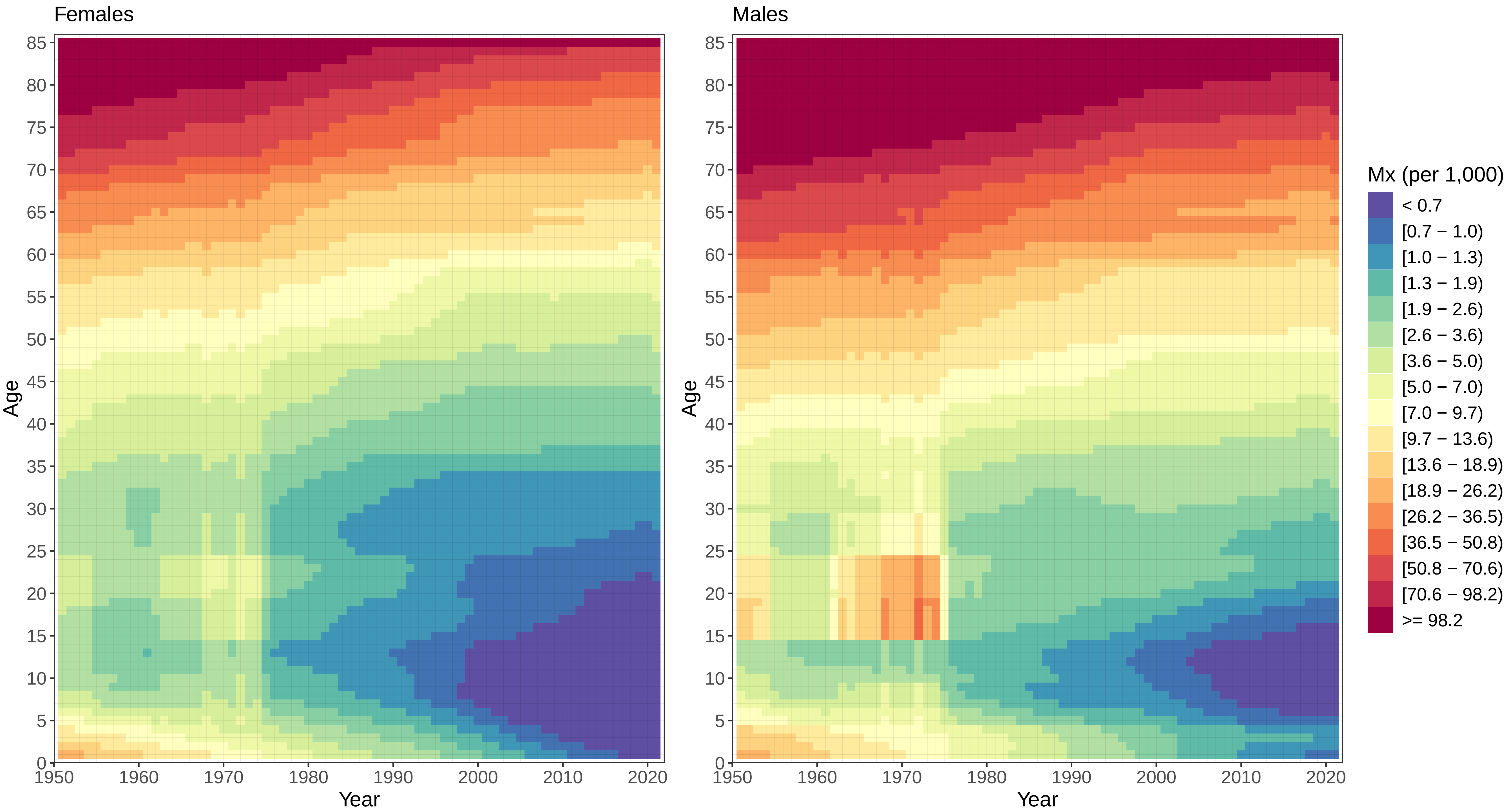

Appendix 2 Figure S4: Angola mortality rates, 1950−2023

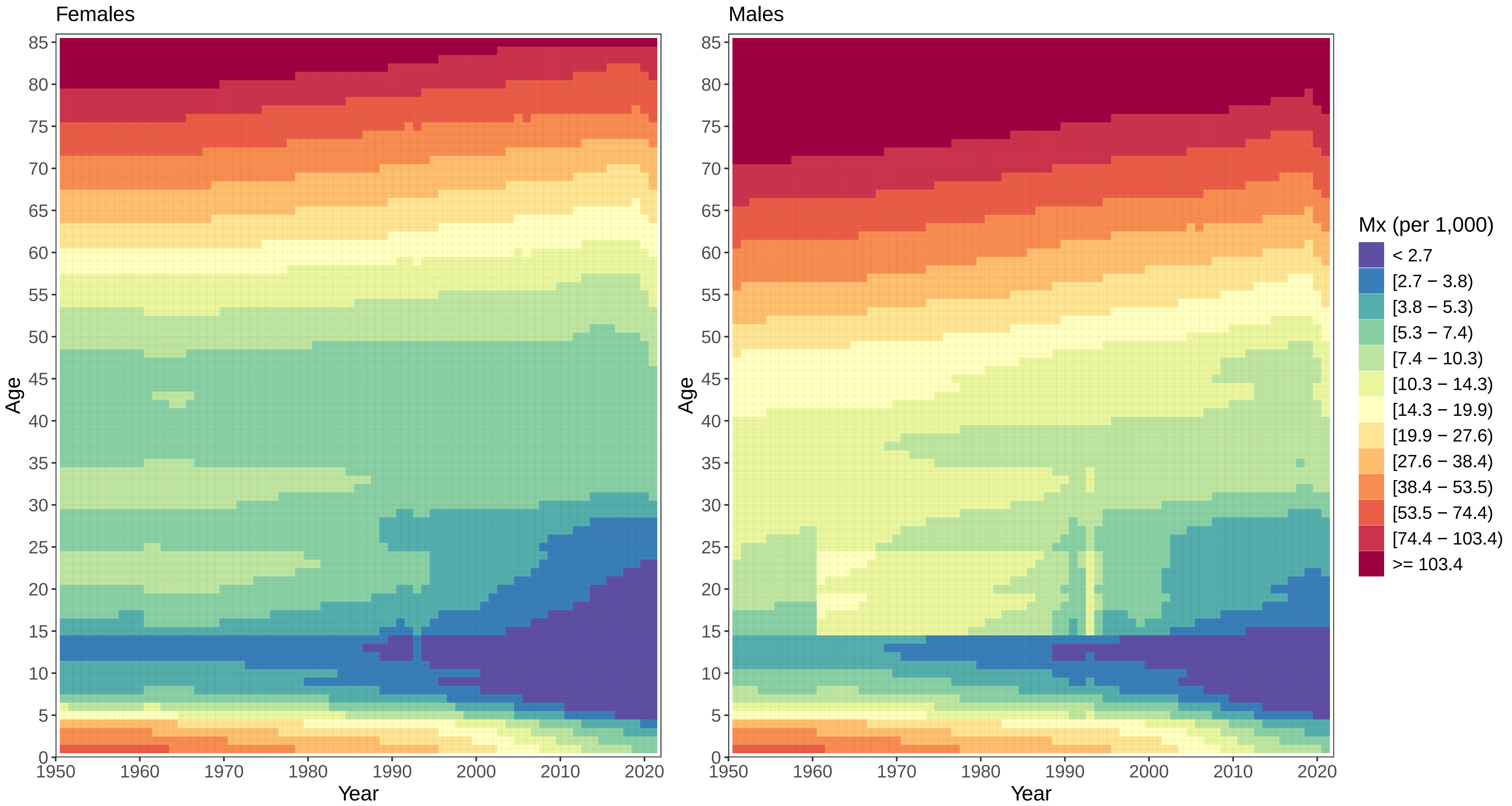

Appendix 2 Figure S4: Central African Republic mortality rates, 1950−2023

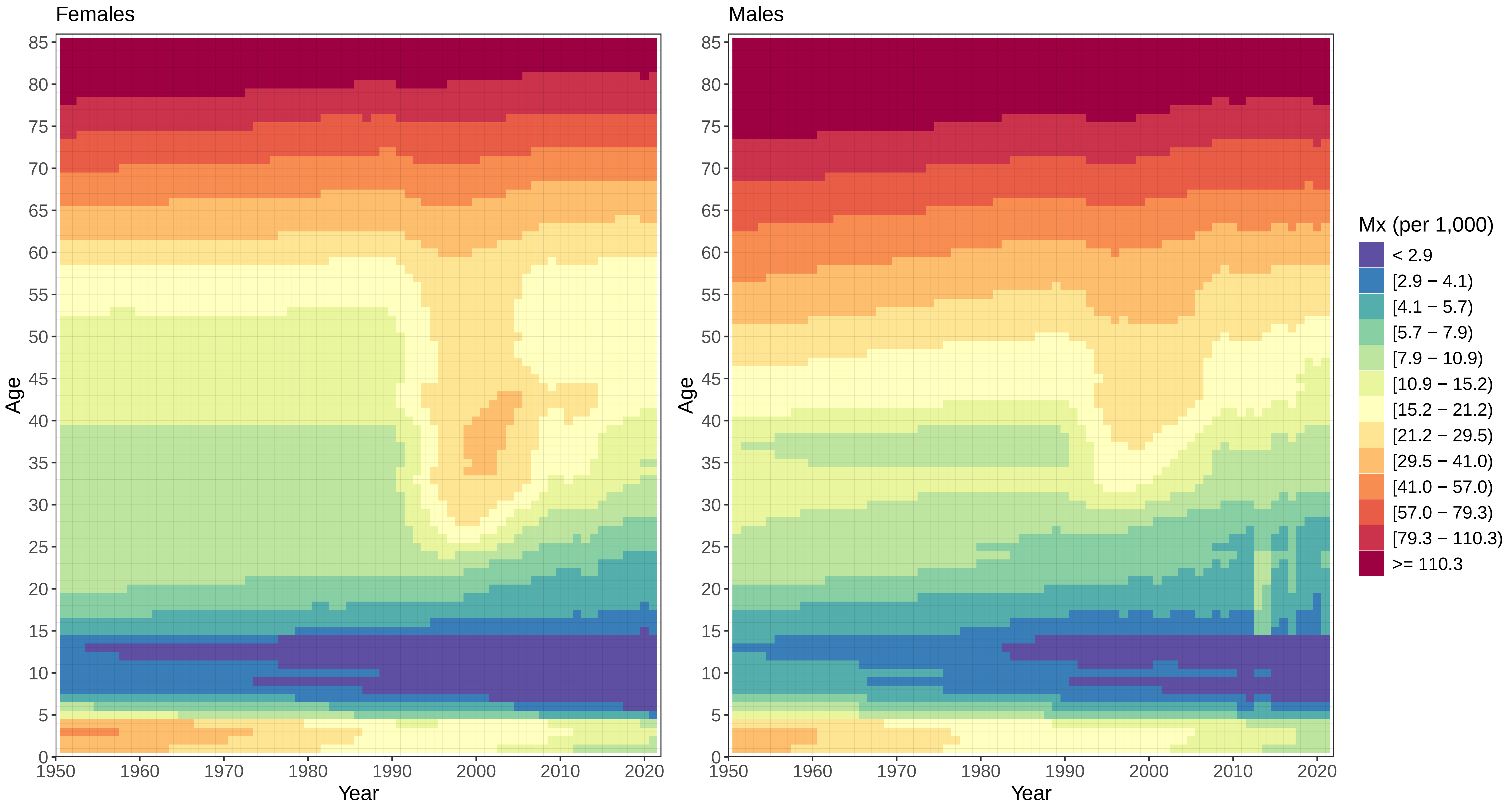

Appendix 2 Figure S4: Congo (Brazzaville) mortality rates, 1950−2023

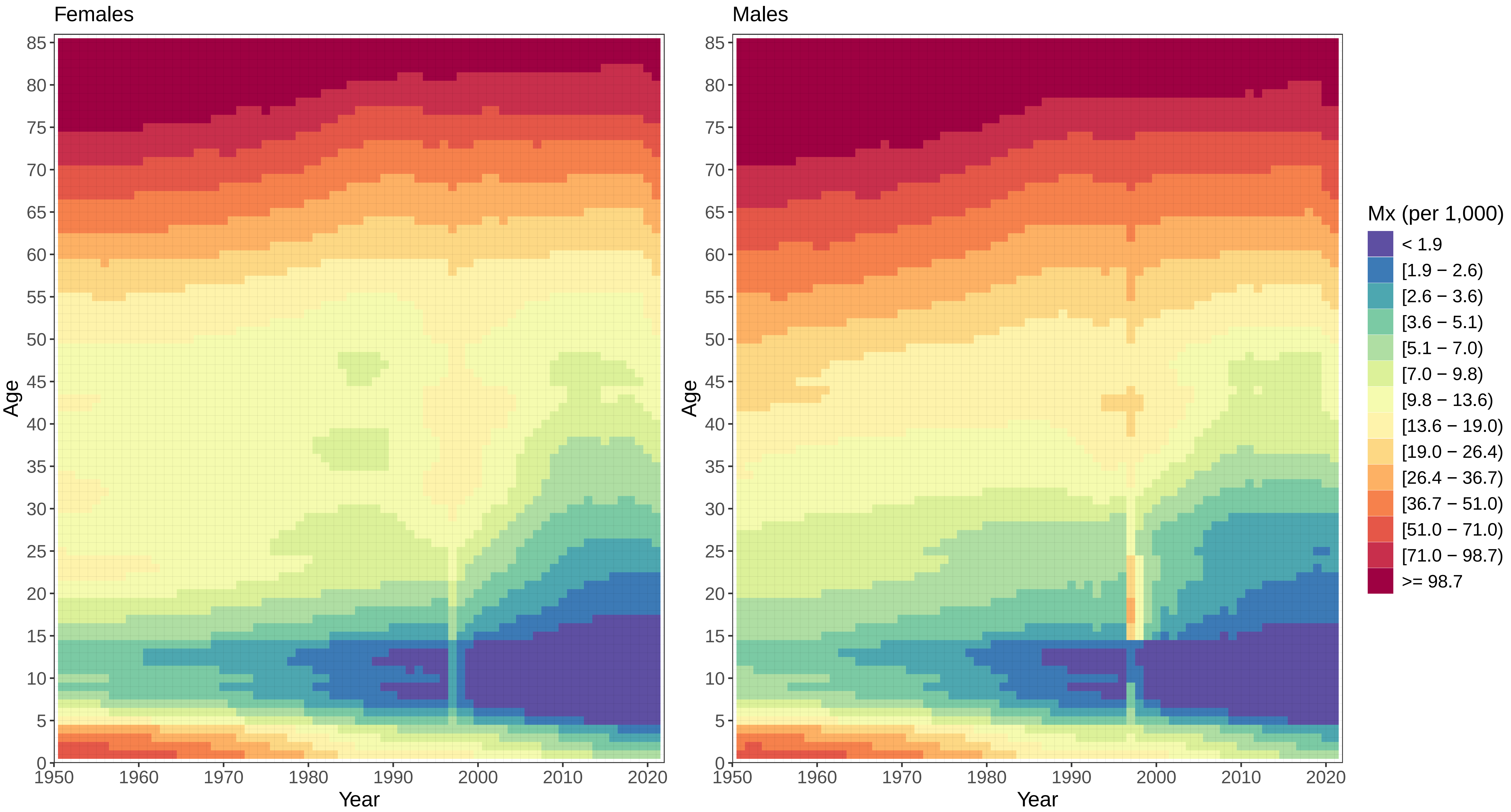

Appendix 2 Figure S4: DR Congo mortality rates, 1950−2023

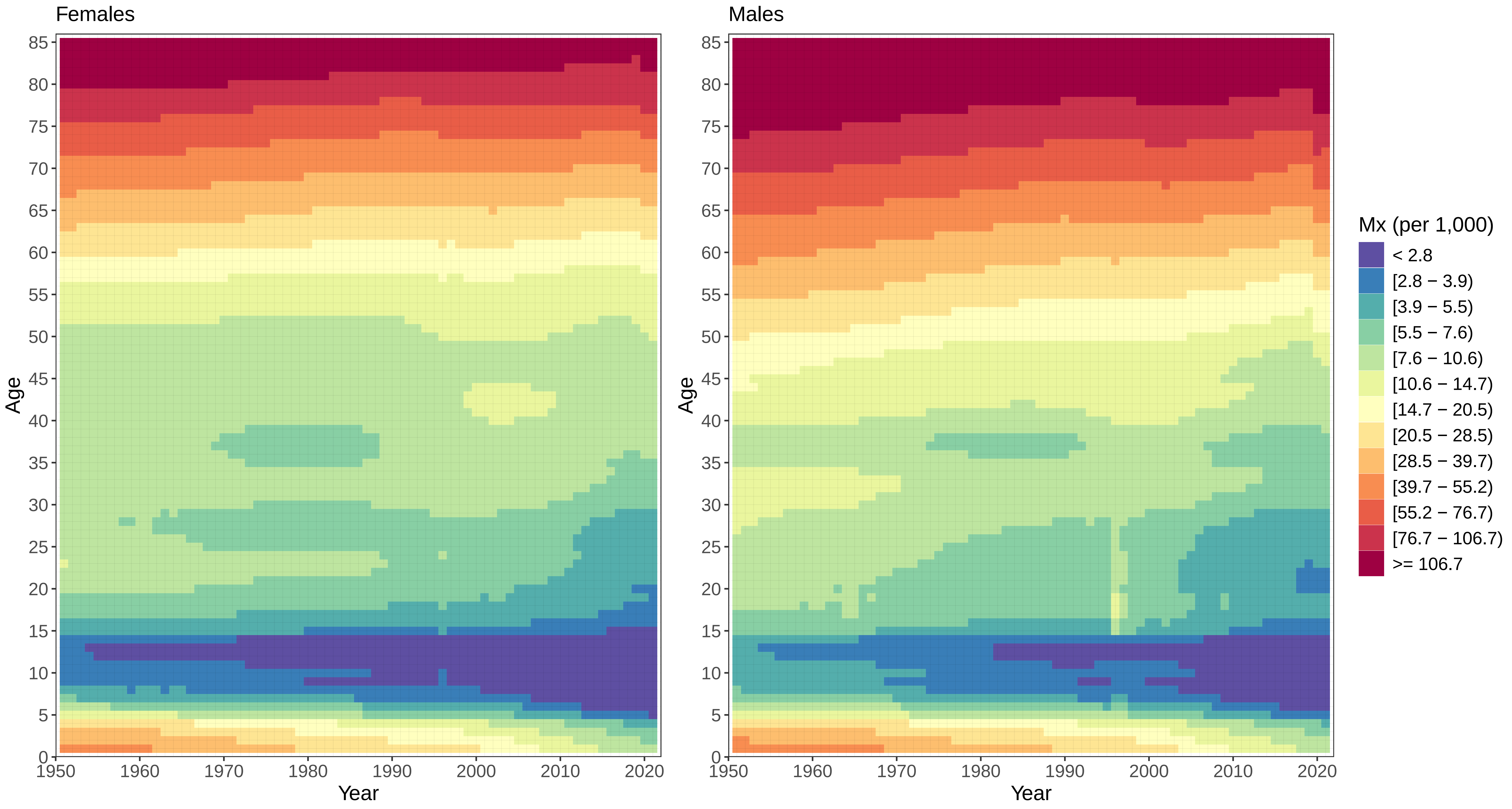

Appendix 2 Figure S4: Equatorial Guinea mortality rates, 1950−2023

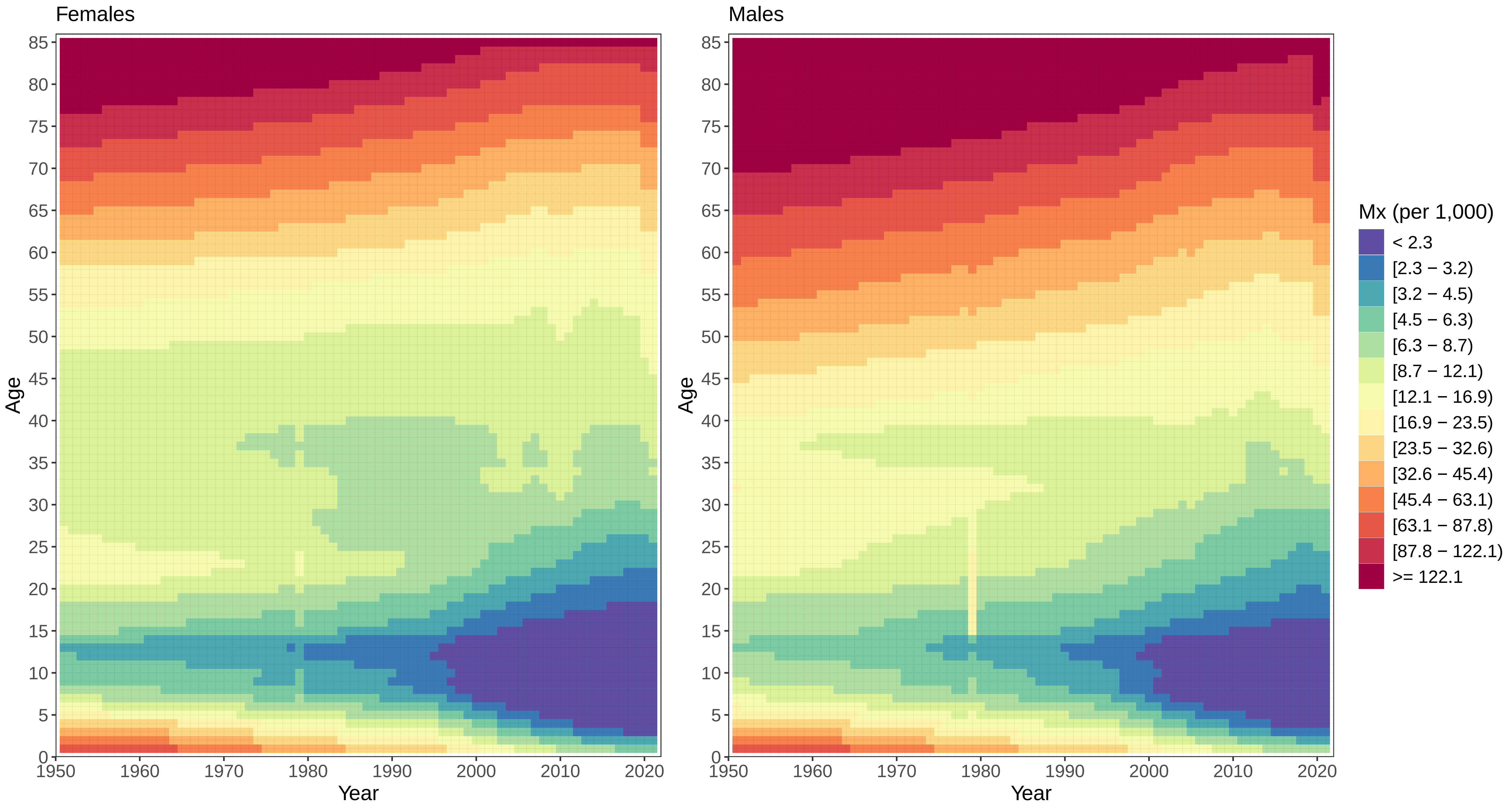

Appendix 2 Figure S4: Gabon mortality rates, 1950−2023

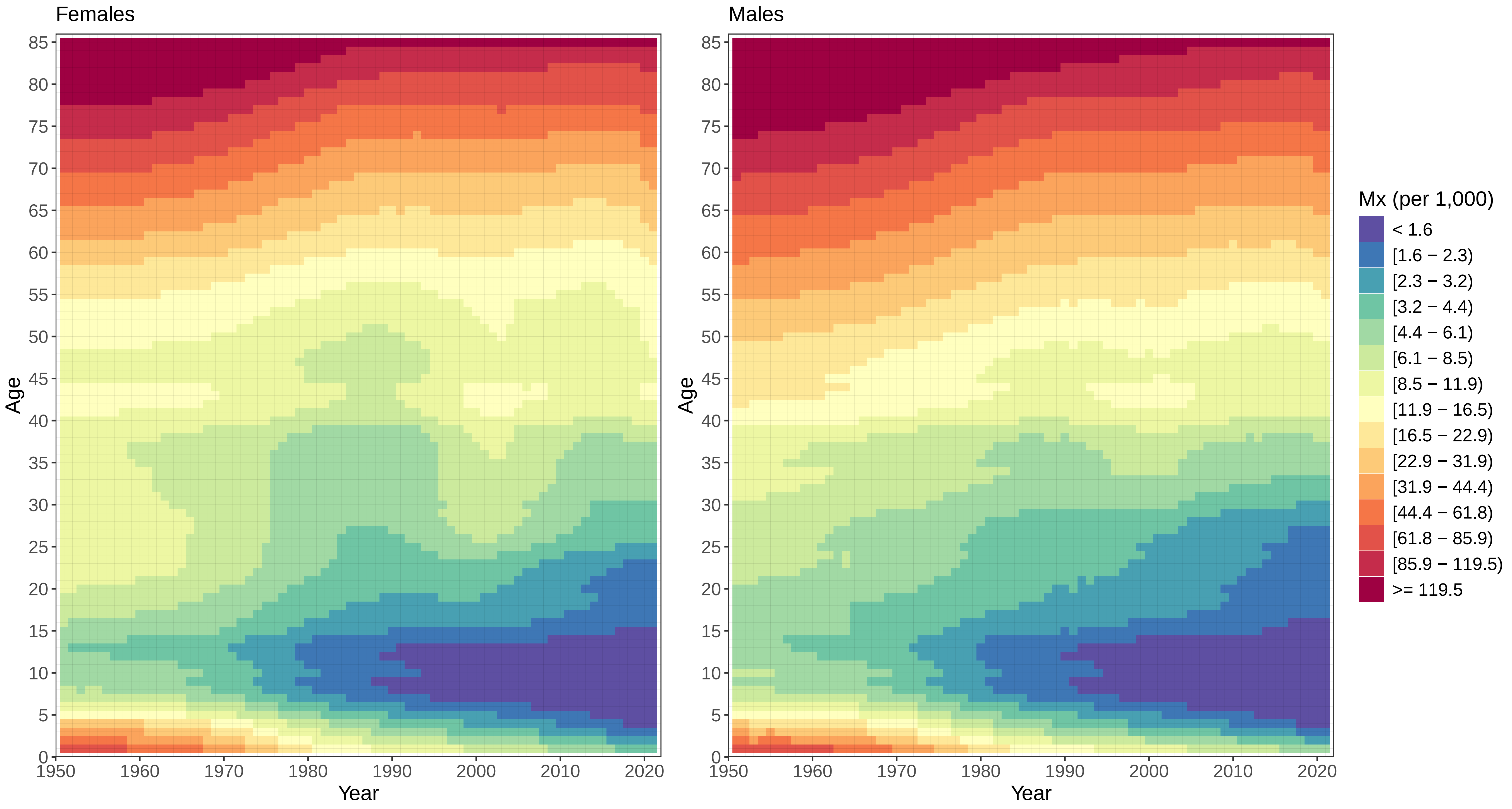

Appendix 2 Figure S4: Burundi mortality rates, 1950−2023

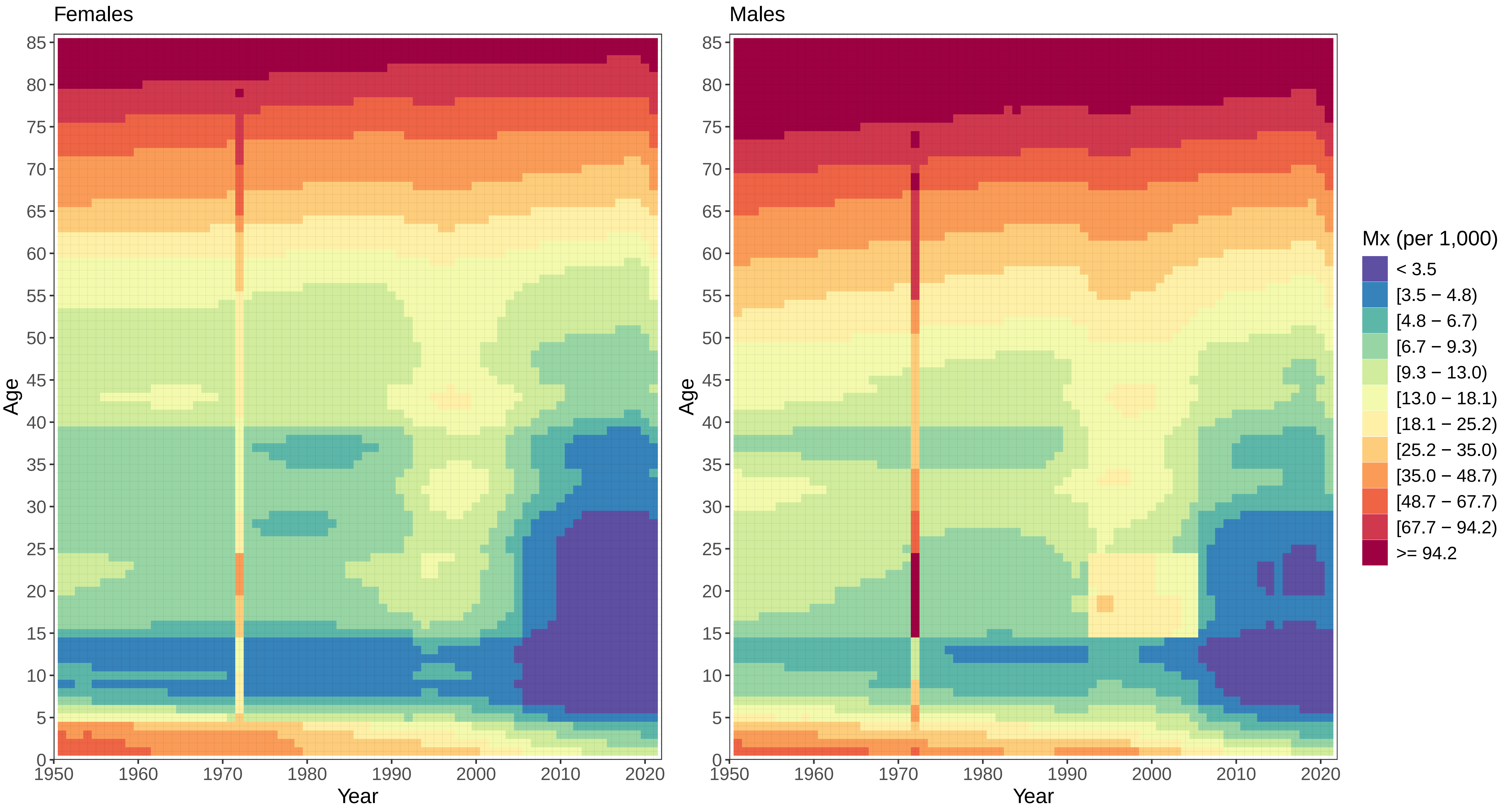

Appendix 2 Figure S4: Comoros mortality rates, 1950−2023

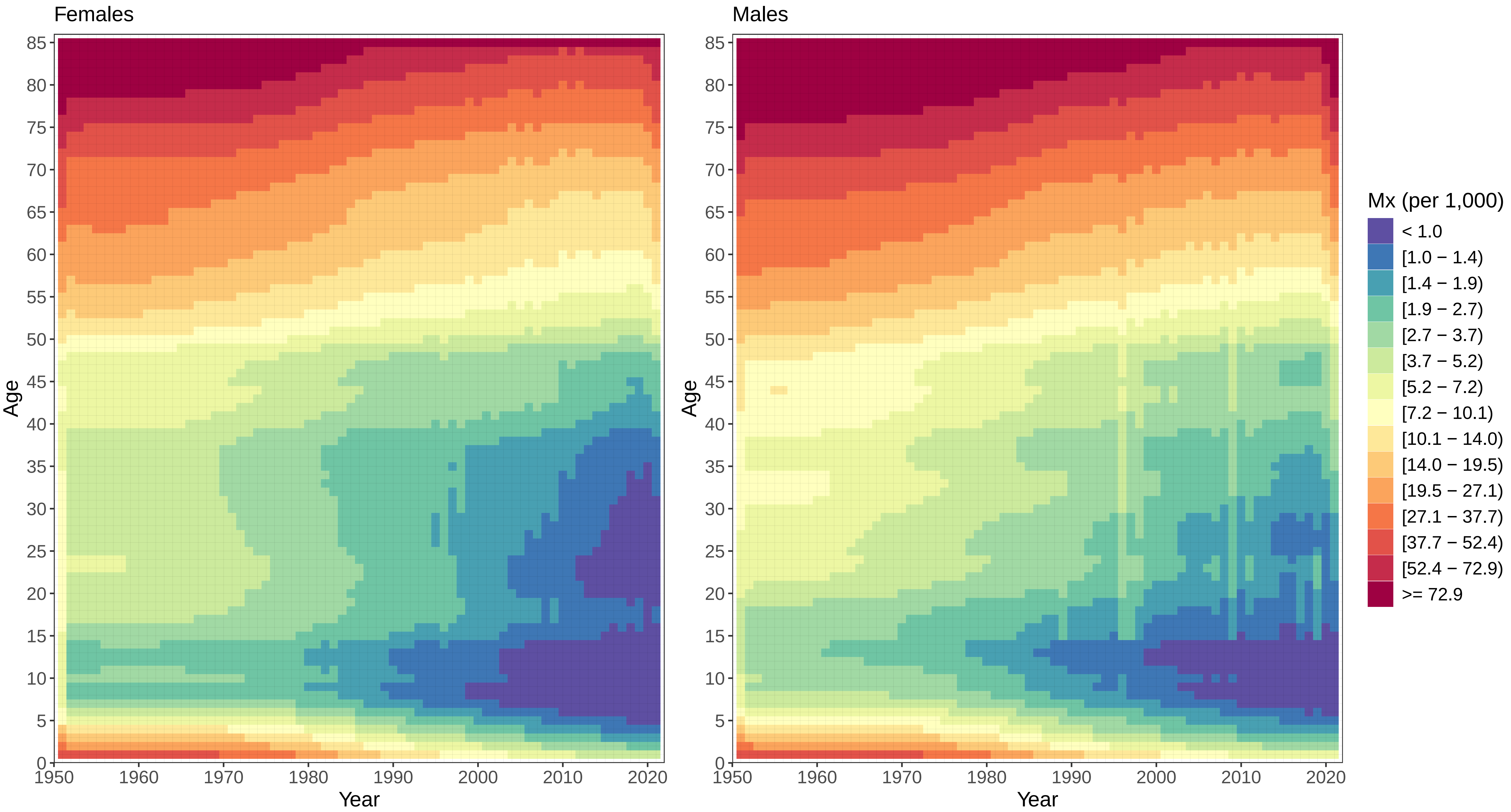

Appendix 2 Figure S4: Djibouti mortality rates, 1950−2023

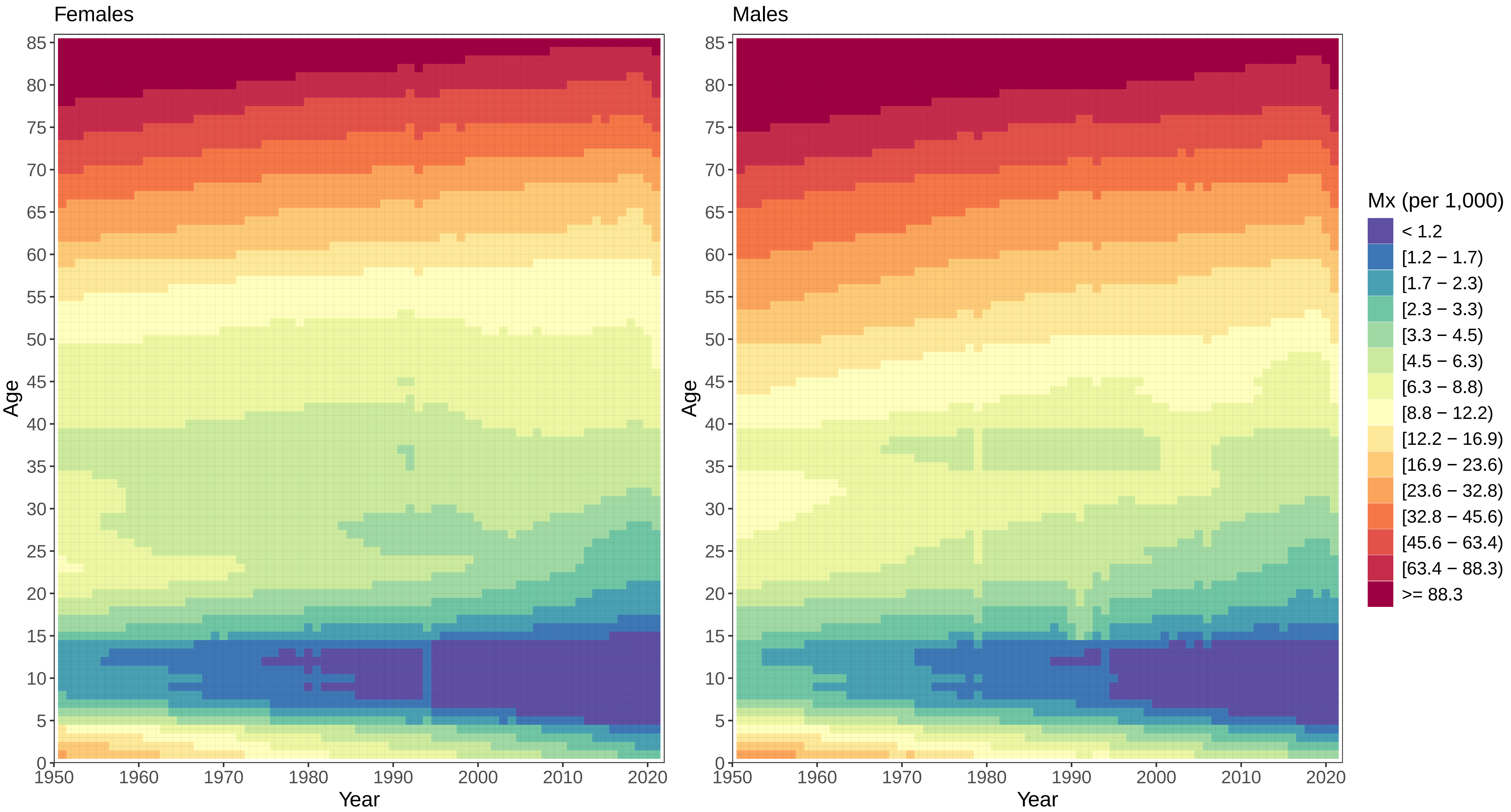

Appendix 2 Figure S4: Eritrea mortality rates, 1950−2023

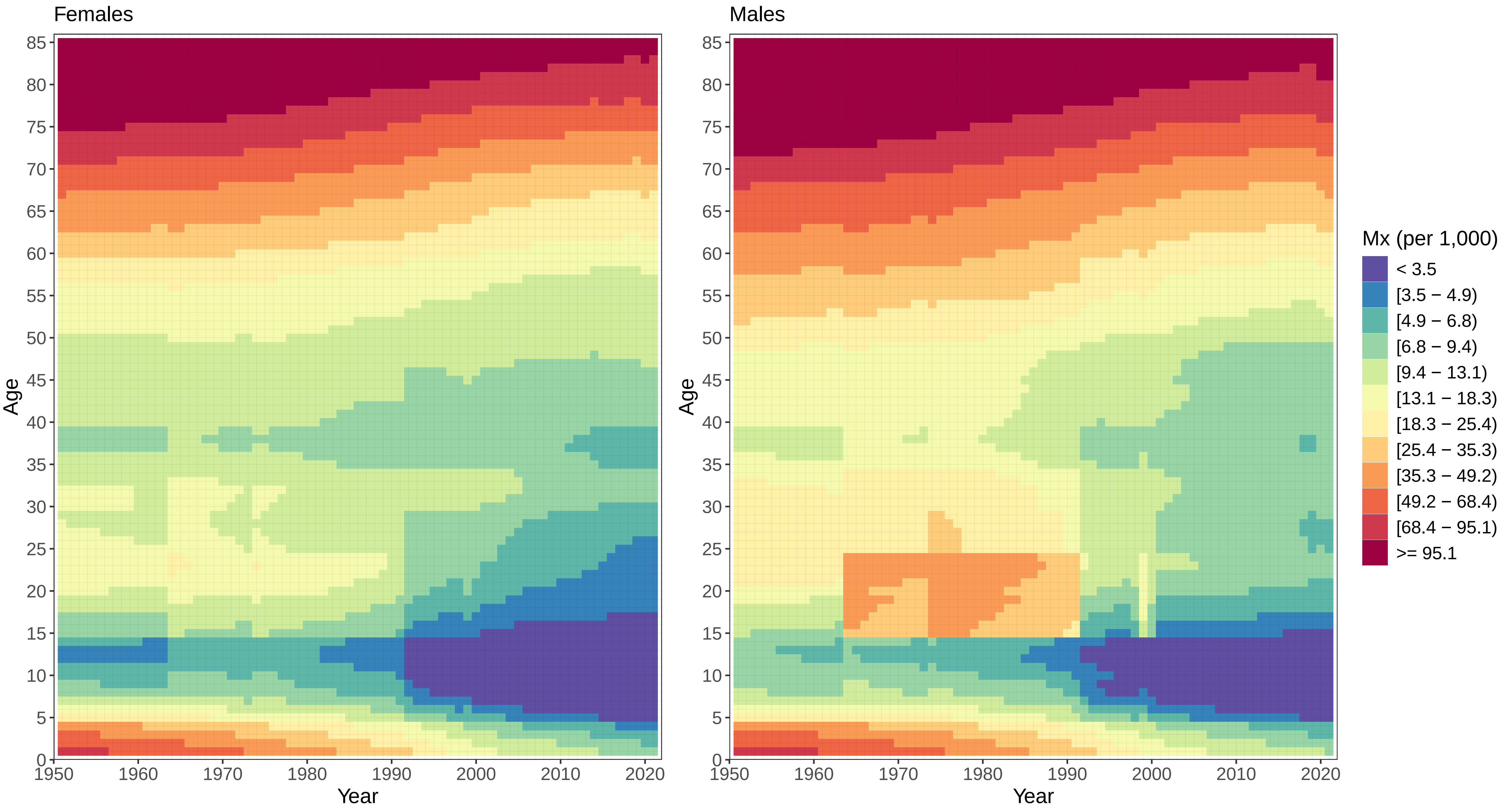

Appendix 2 Figure S4: Ethiopia mortality rates, 1950−2023

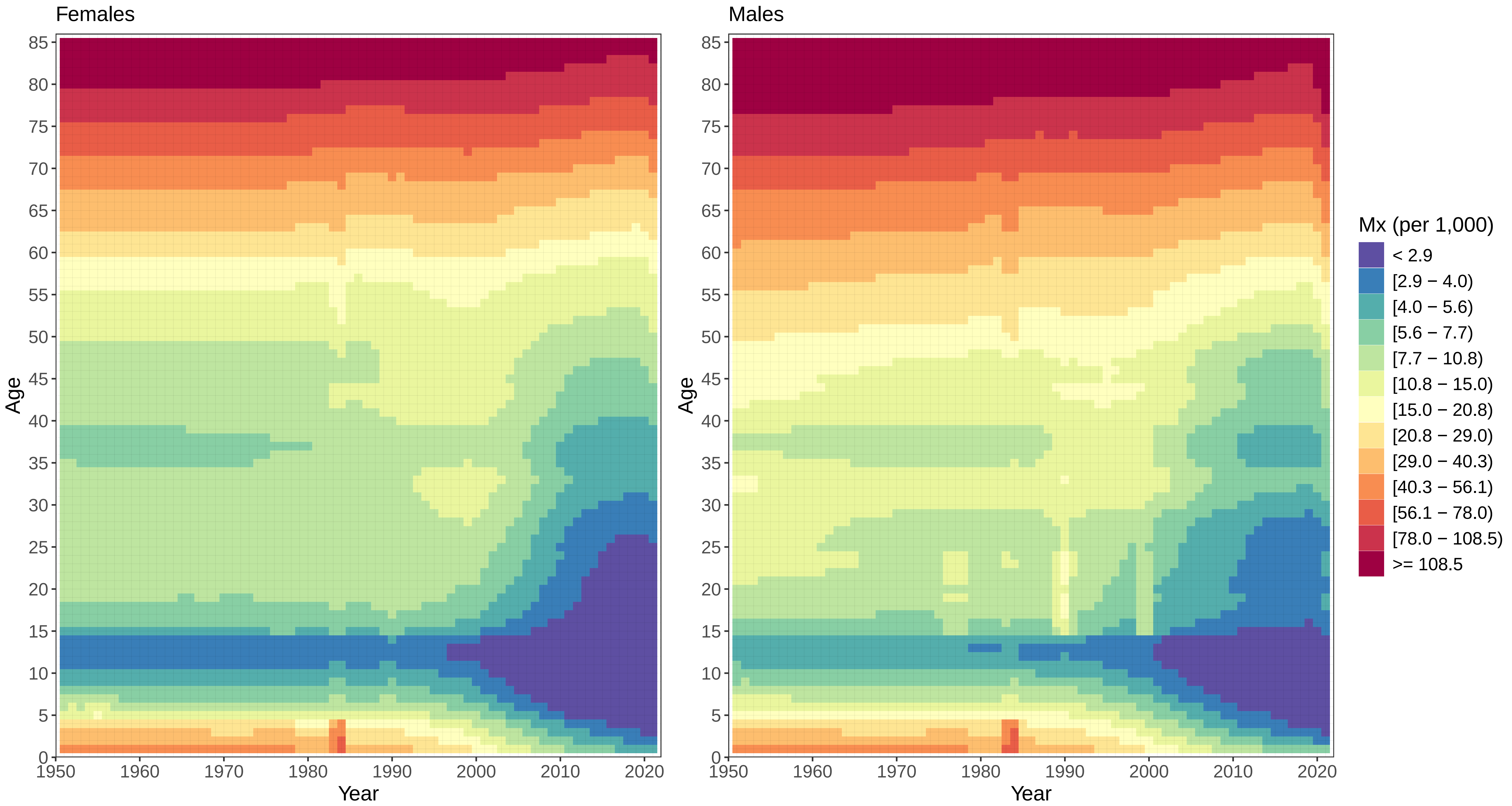

Appendix 2 Figure S4: Kenya mortality rates, 1950−2023

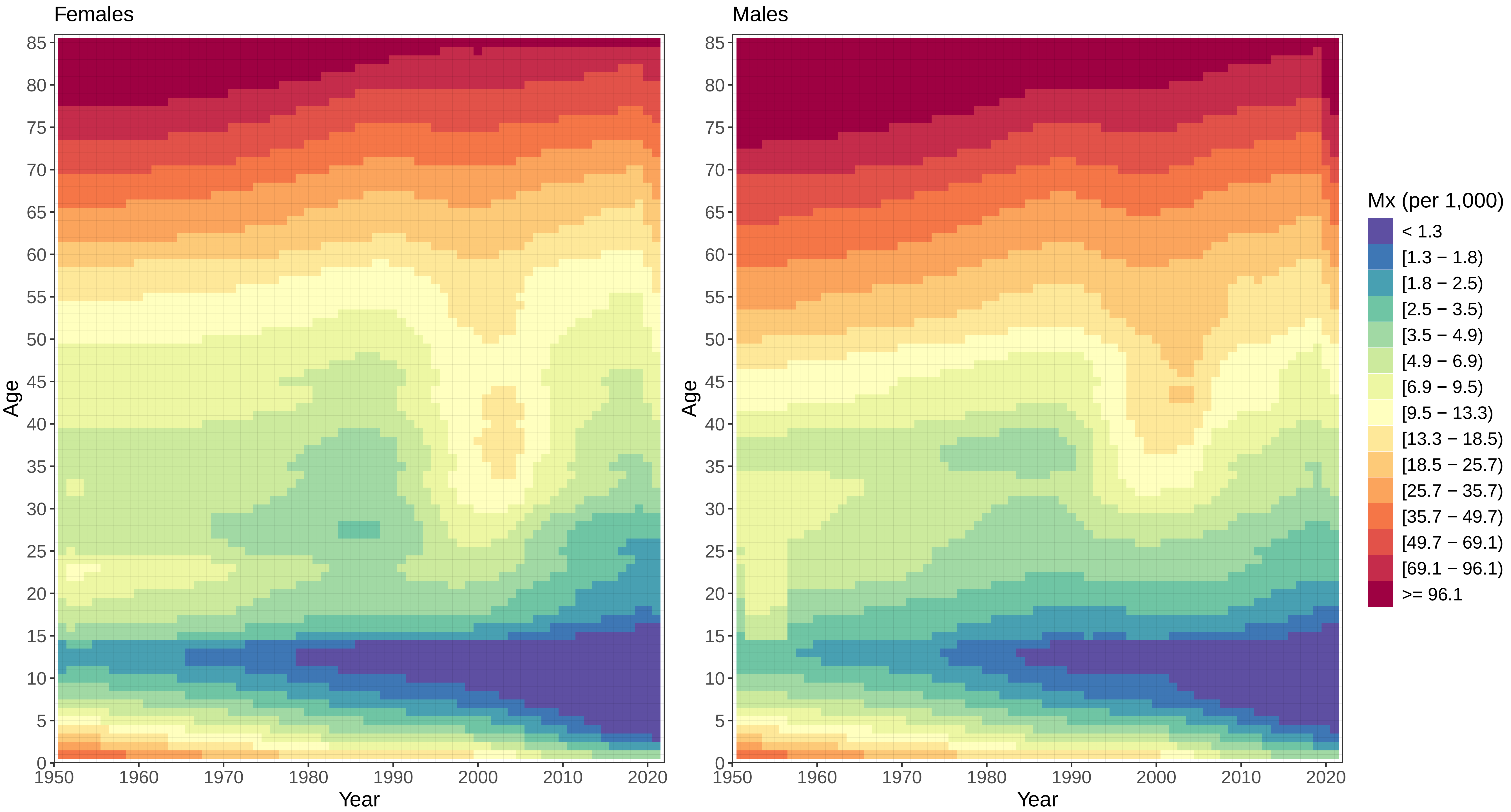

Appendix 2 Figure S4: Madagascar mortality rates, 1950−2023

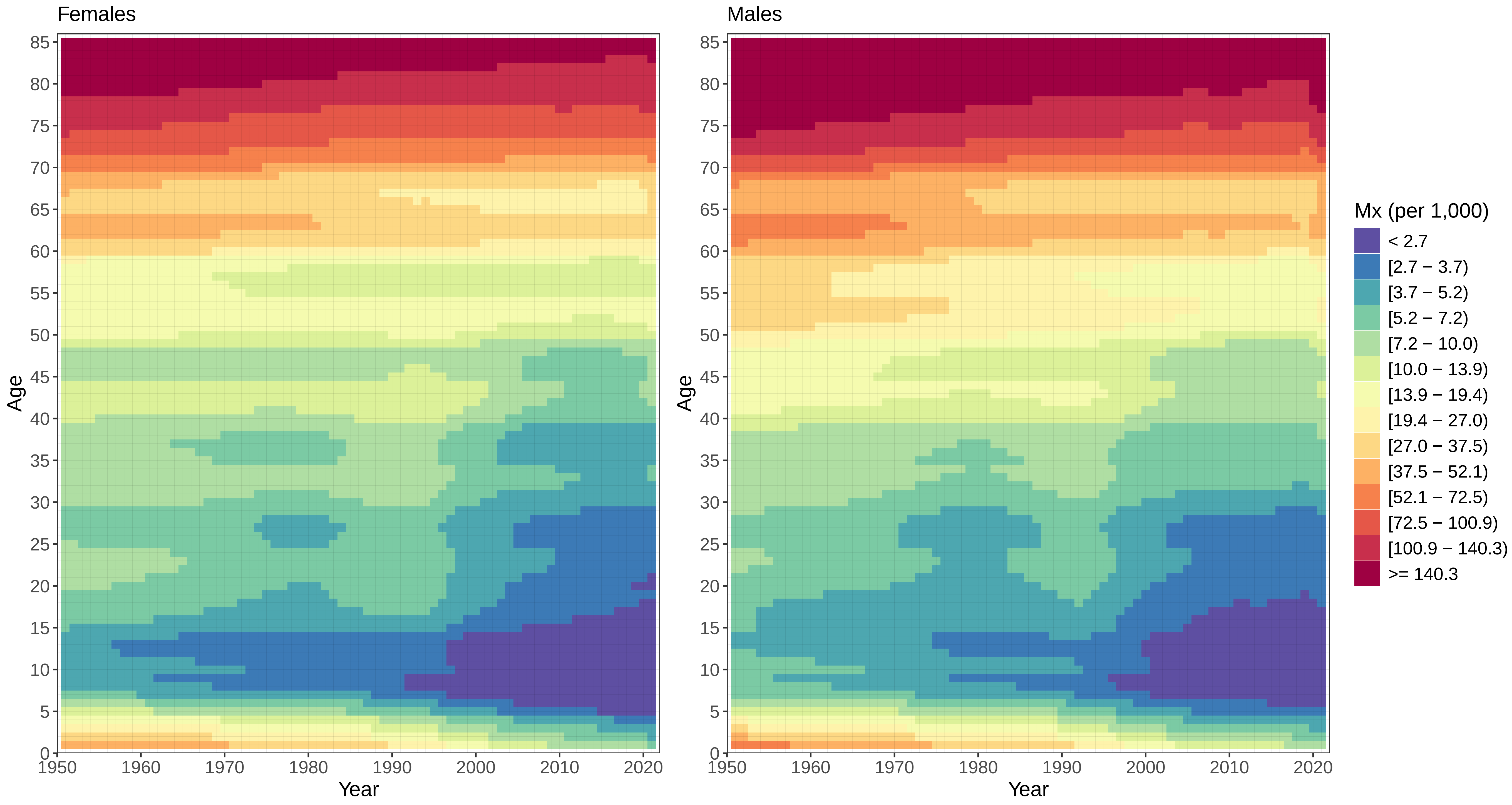

Appendix 2 Figure S4: Malawi mortality rates, 1950−2023

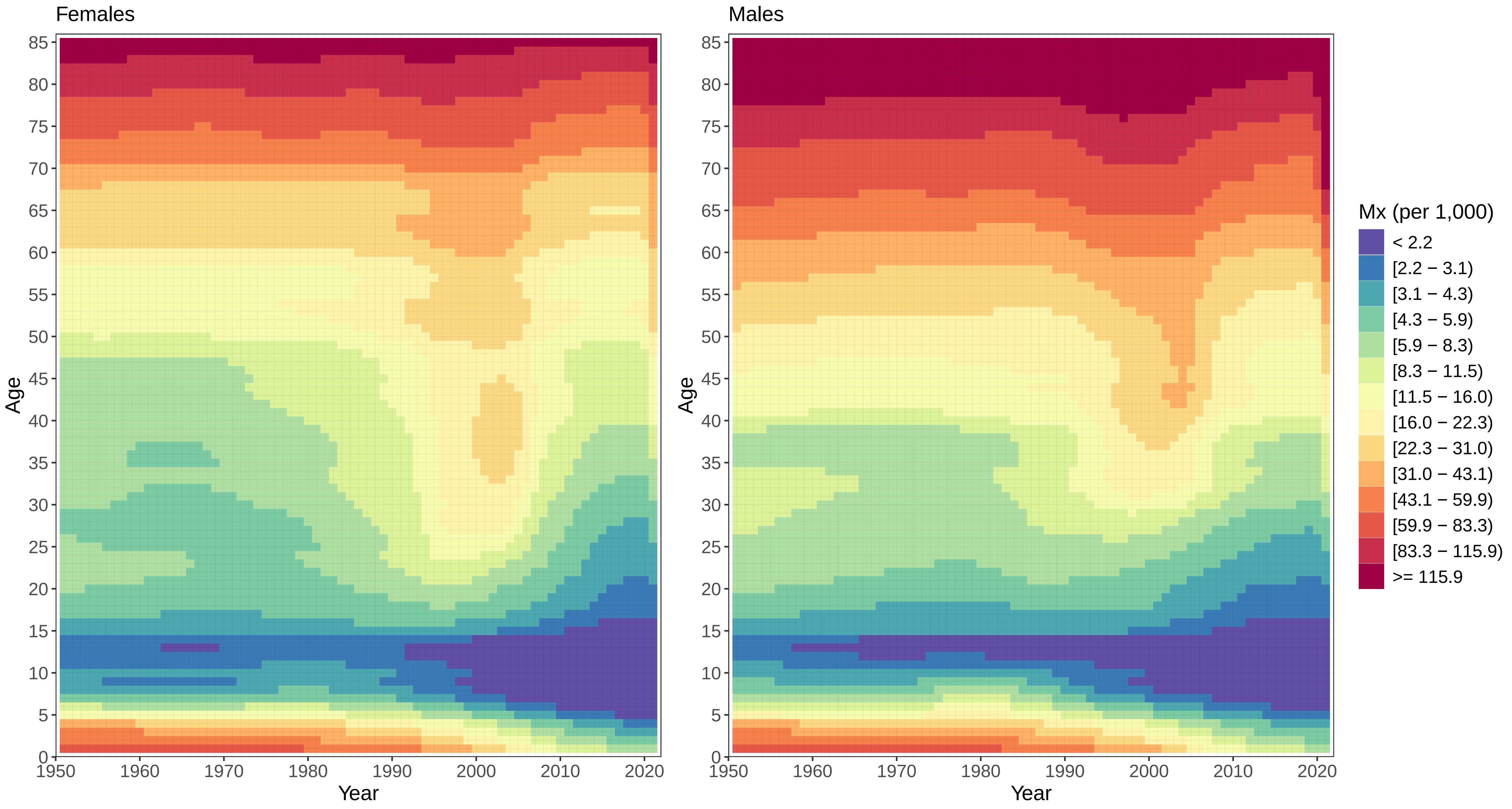

Appendix 2 Figure S4: Mozambique mortality rates, 1950−2023

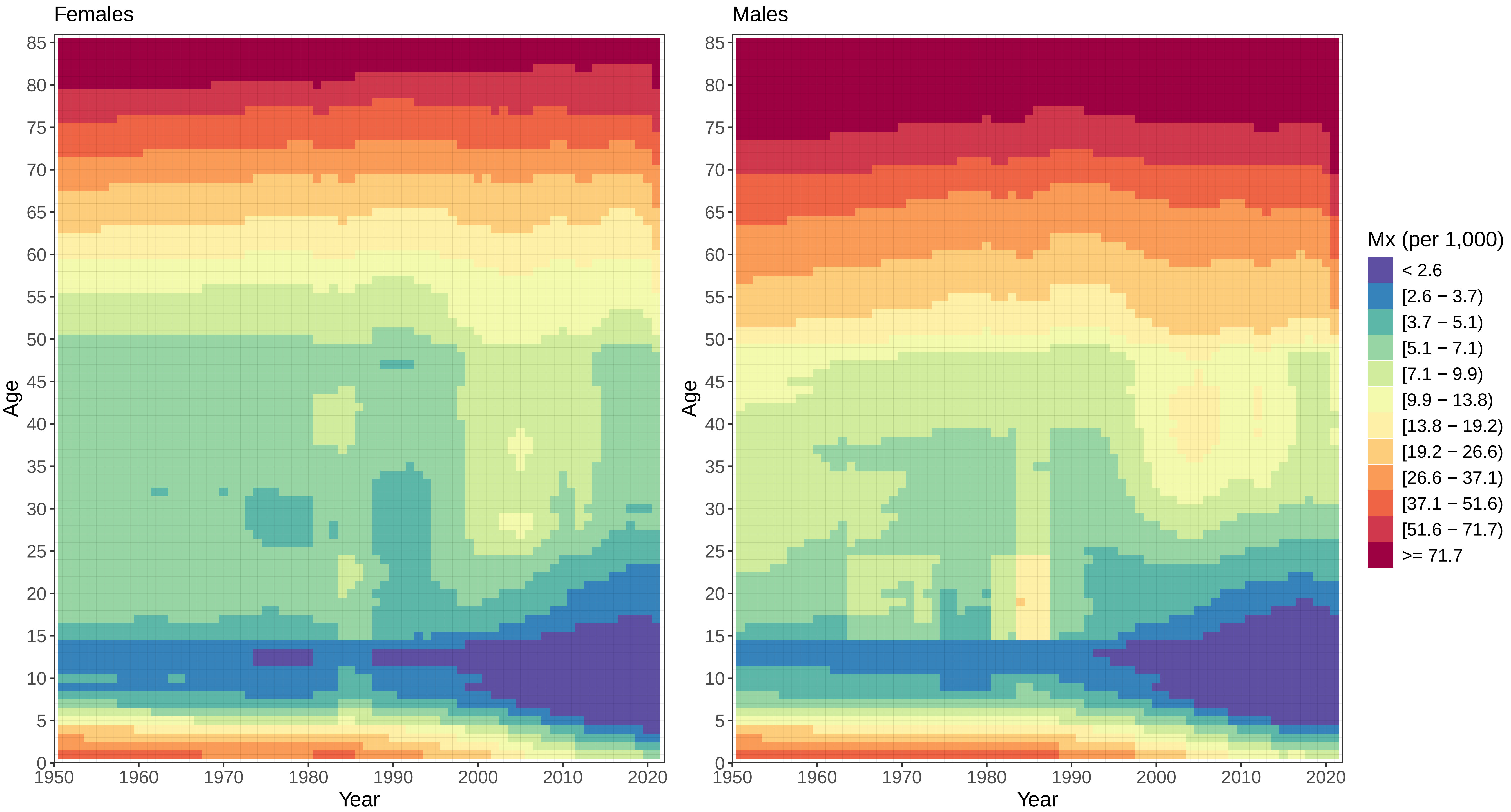

Appendix 2 Figure S4: Rwanda mortality rates, 1950−2023

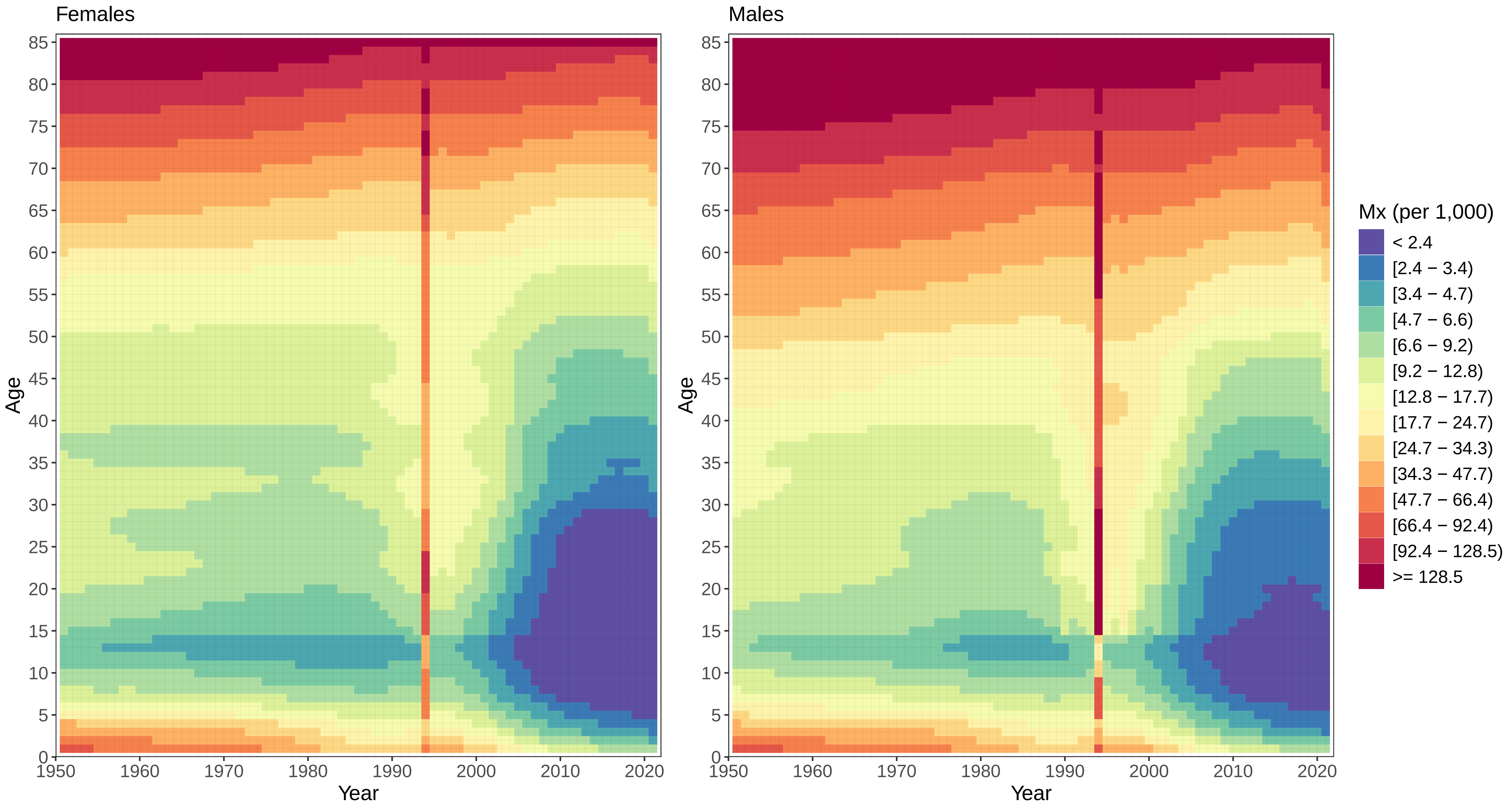

Appendix 2 Figure S4: Somalia mortality rates, 1950−2023

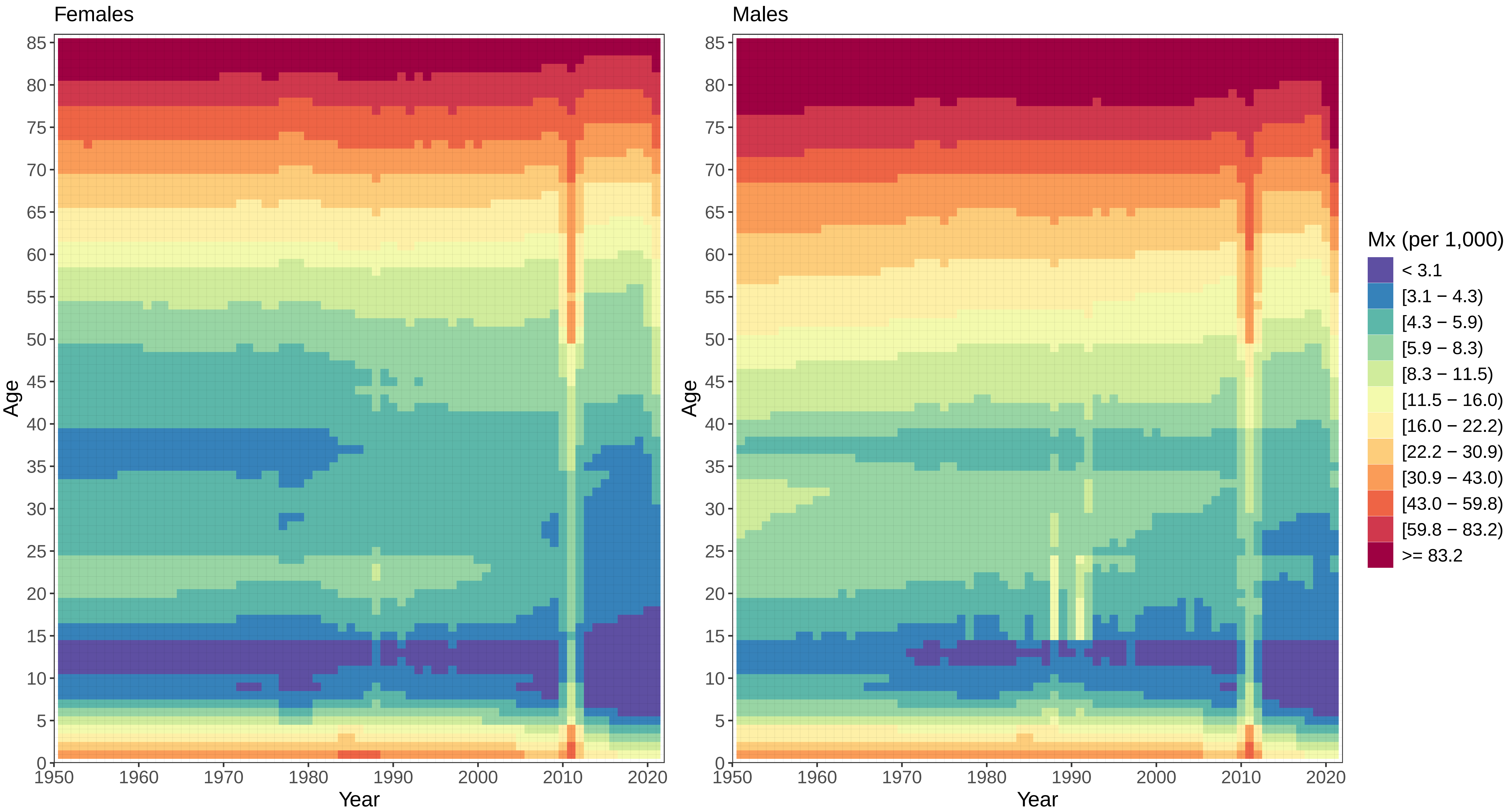

Appendix 2 Figure S4: South Sudan mortality rates, 1950−2023

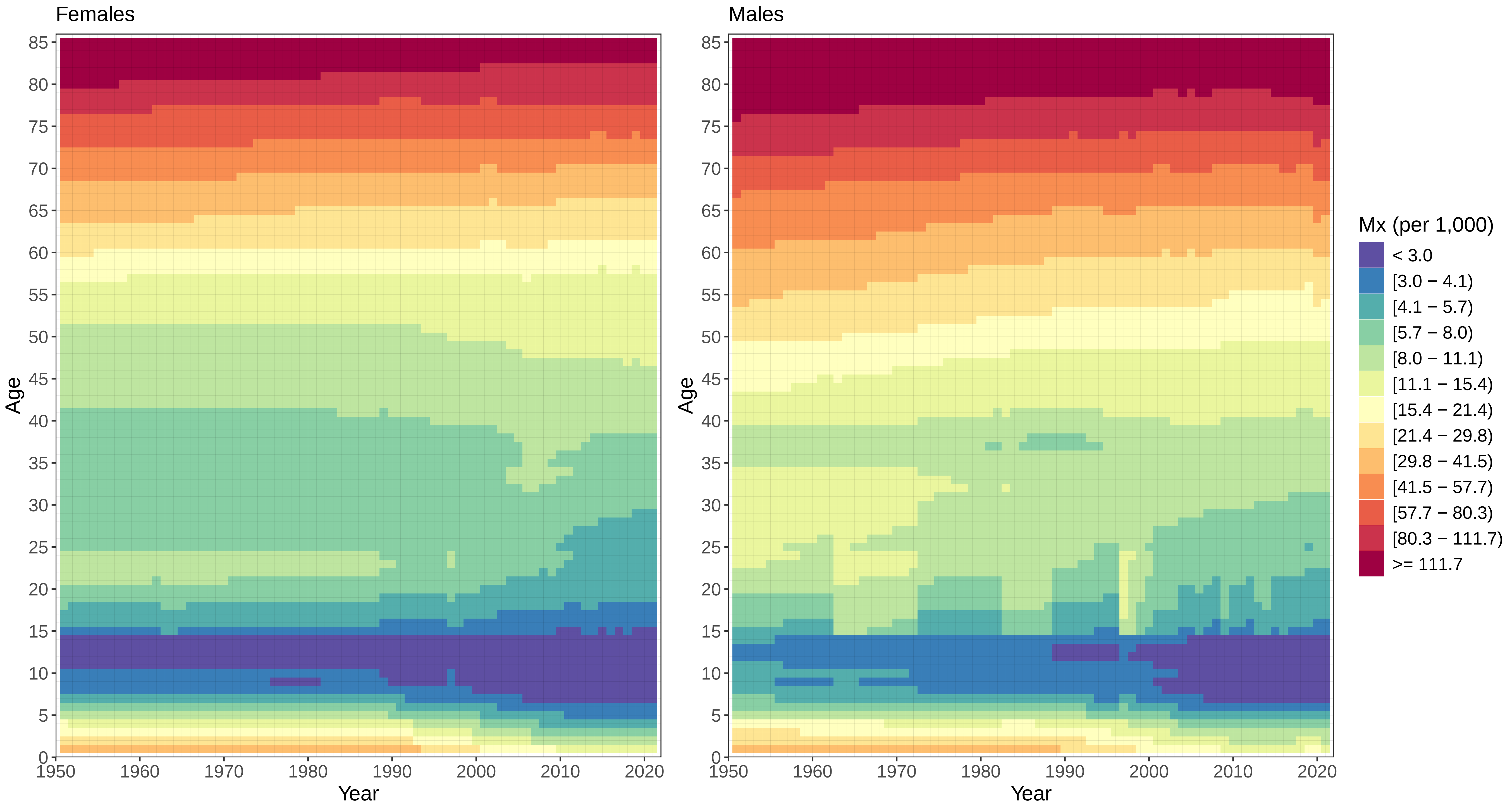

Appendix 2 Figure S4: Uganda mortality rates, 1950−2023

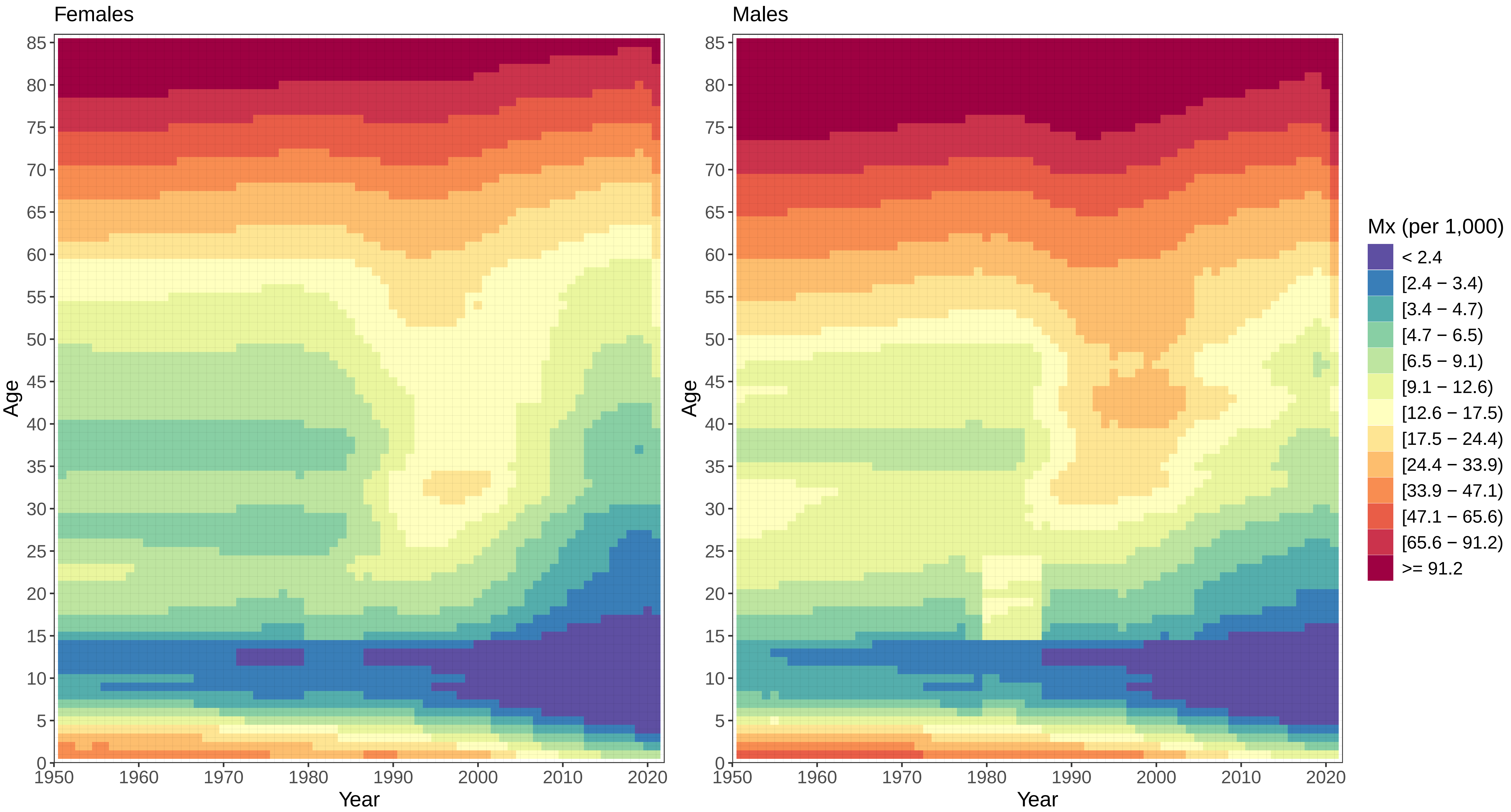

Appendix 2 Figure S4: Tanzania mortality rates, 1950−2023

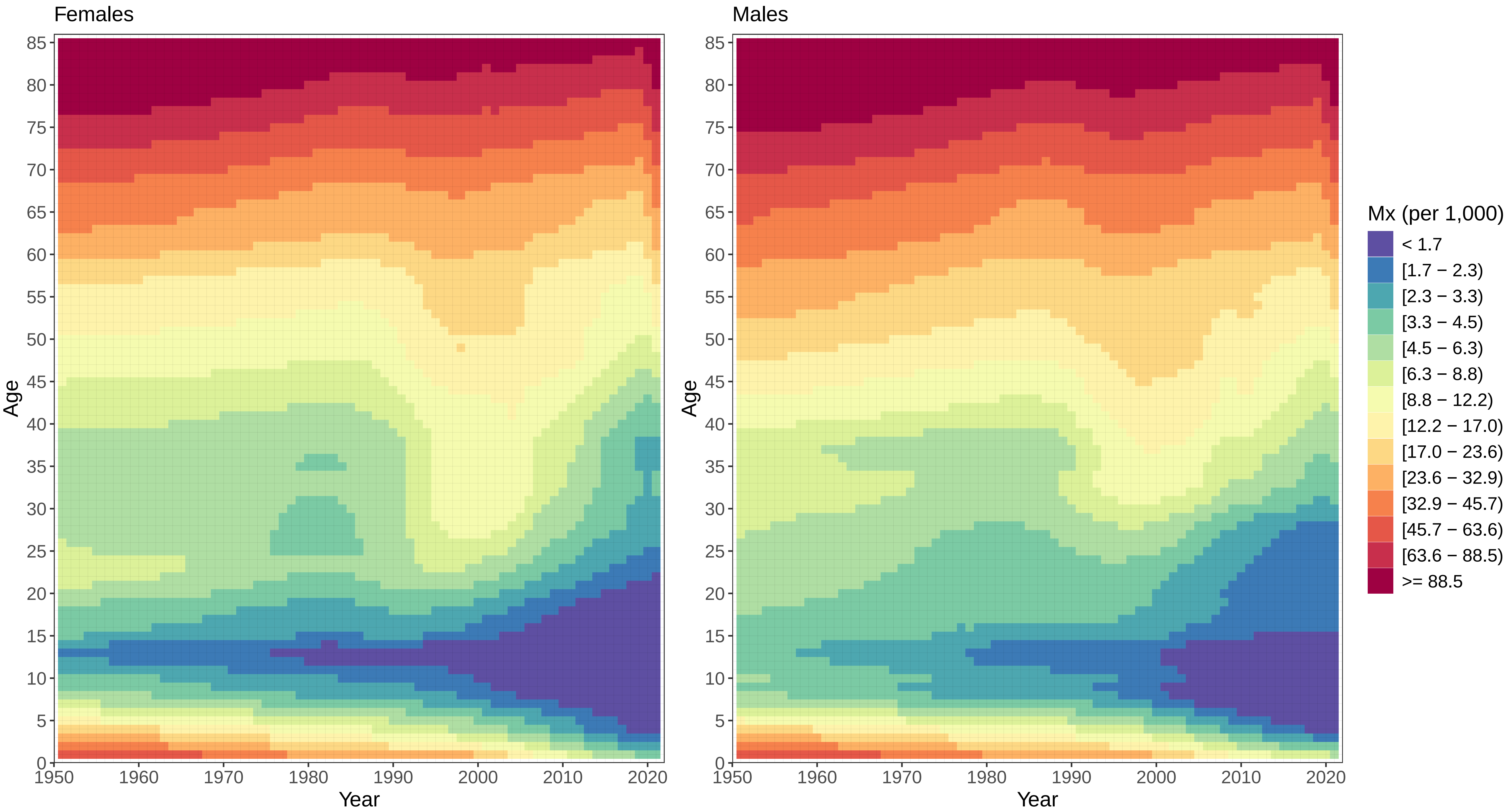

Appendix 2 Figure S4: Zambia mortality rates, 1950−2023

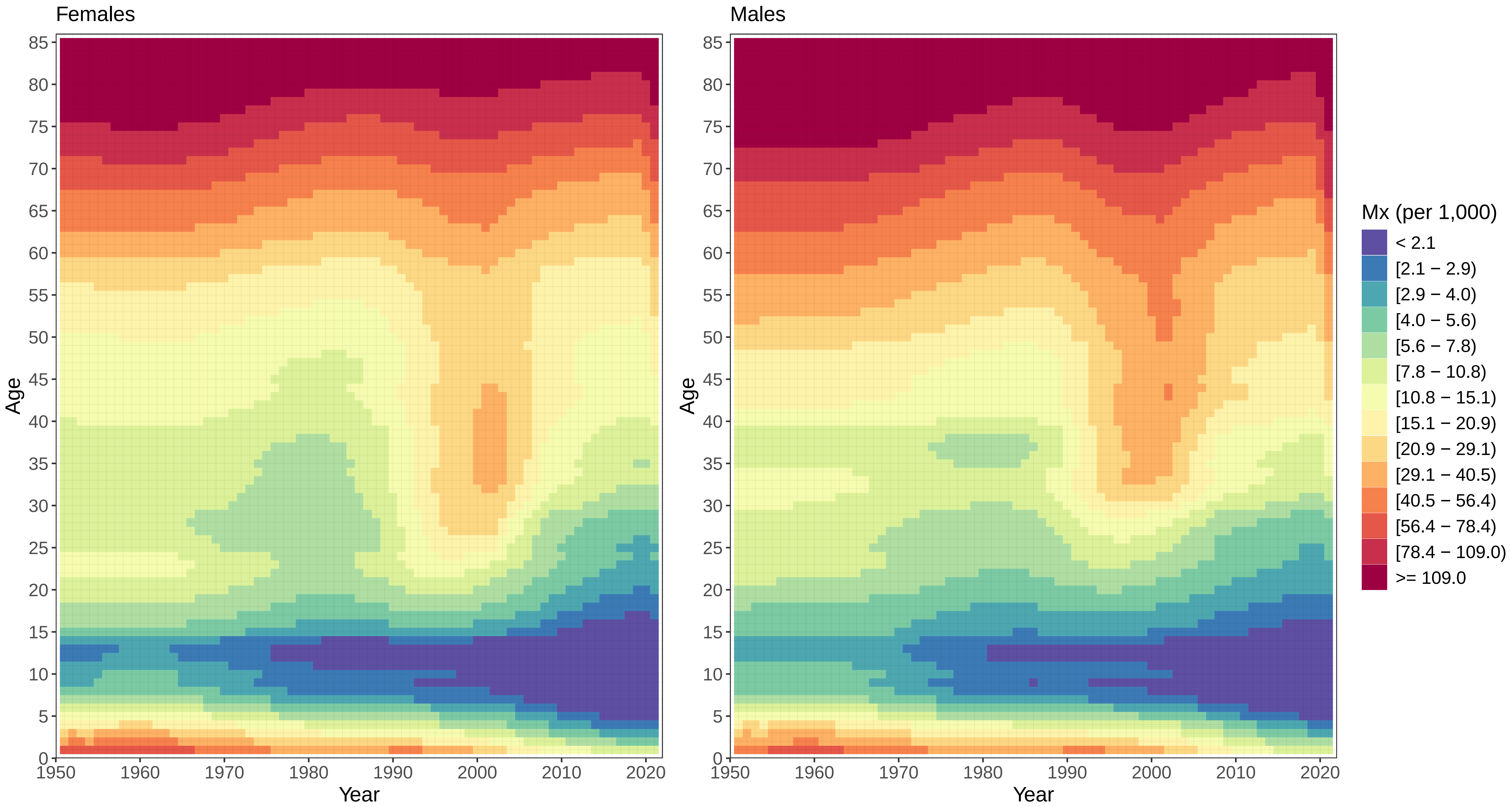

Appendix 2 Figure S4: Botswana mortality rates, 1950−2023

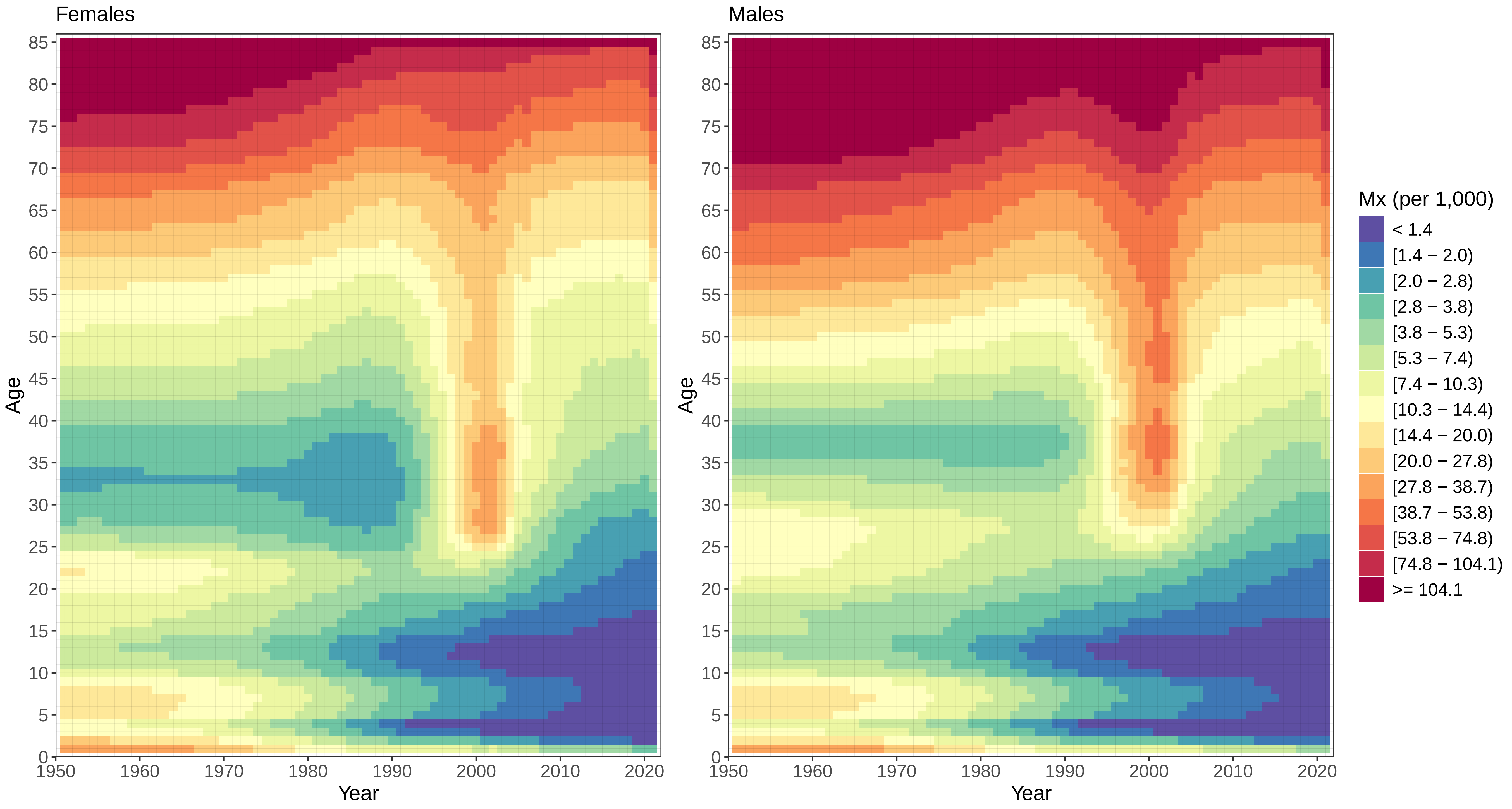

Appendix 2 Figure S4: Eswatini mortality rates, 1950−2023

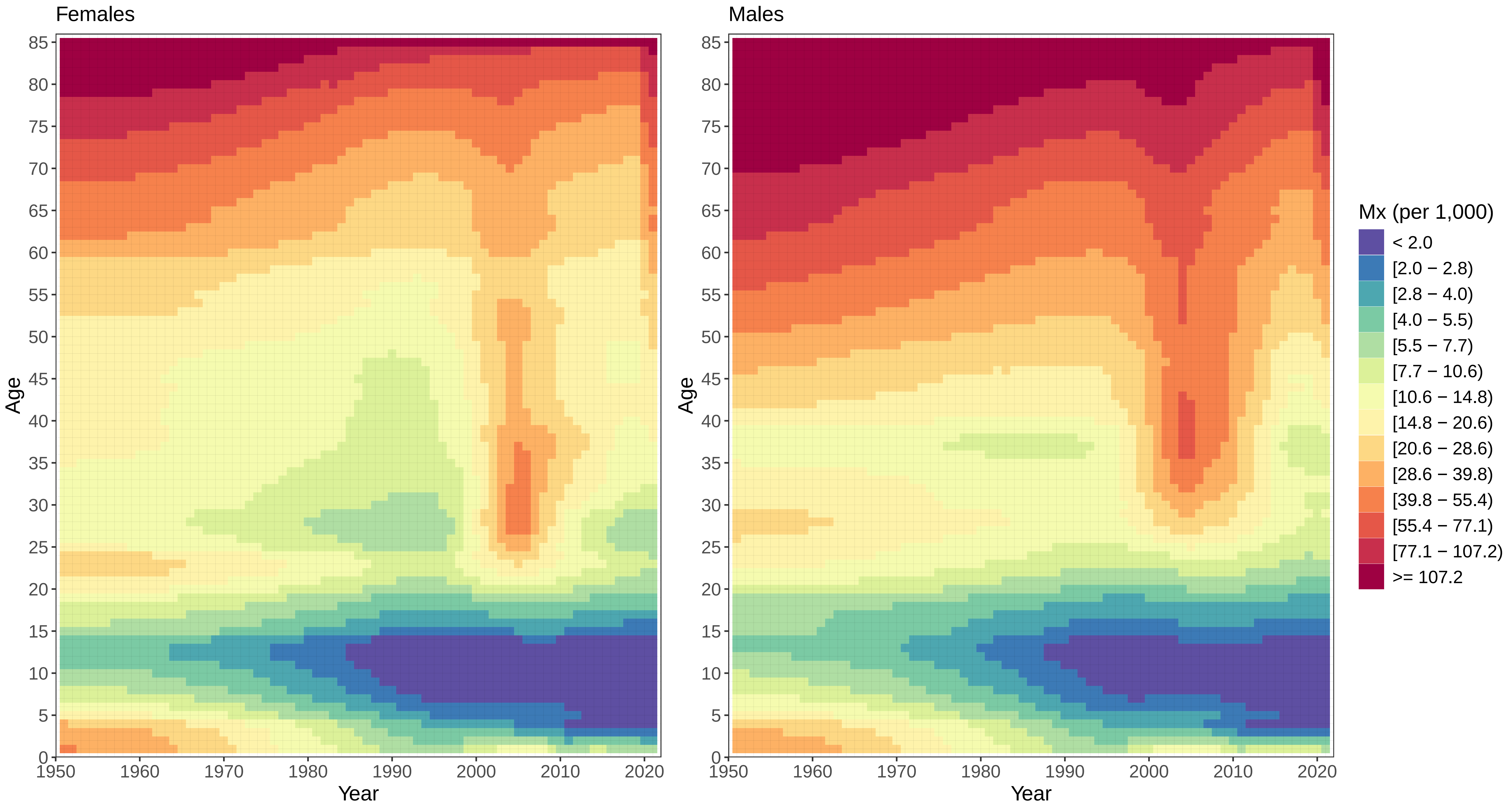

Appendix 2 Figure S4: Lesotho mortality rates, 1950−2023

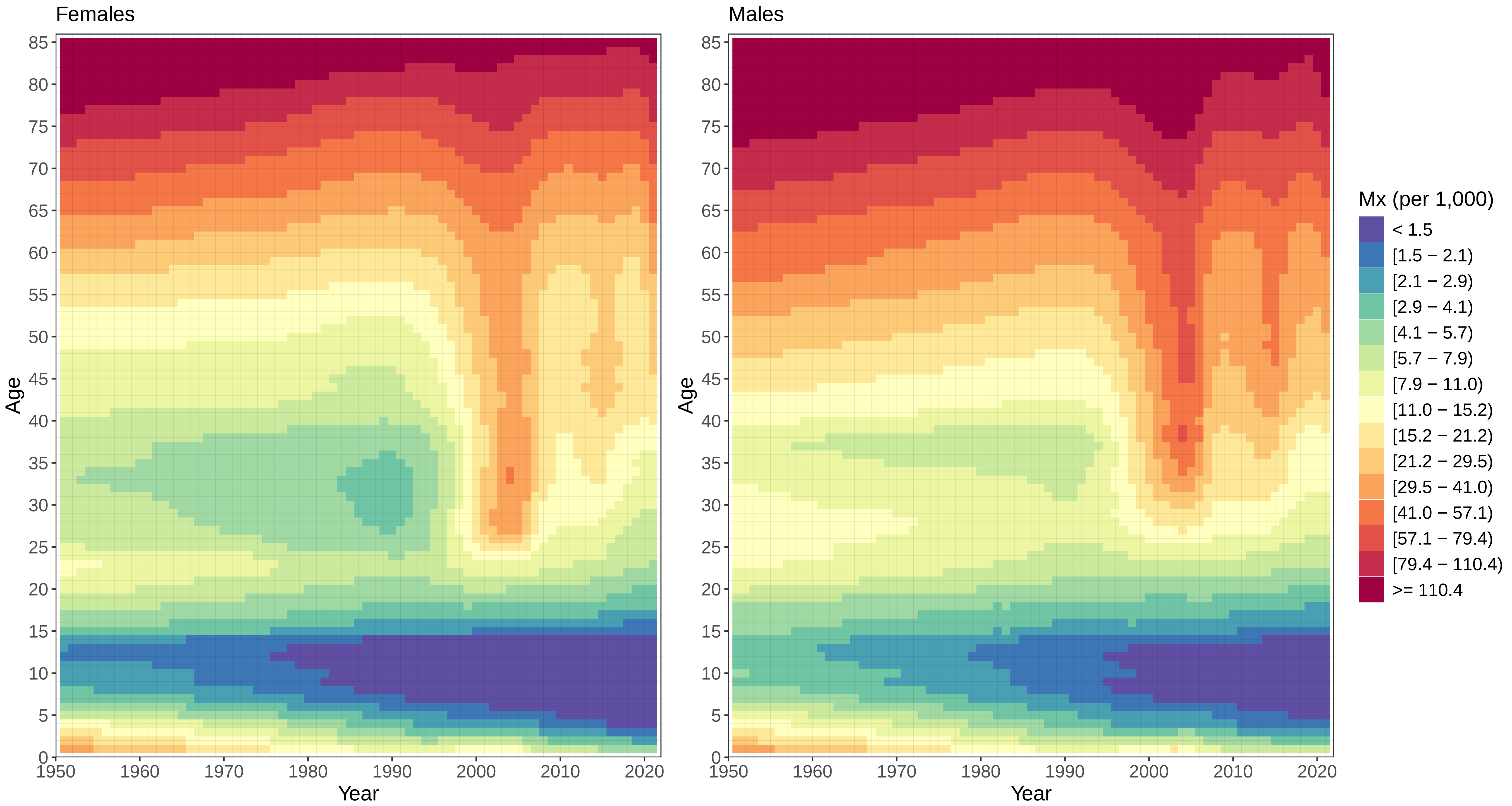

Appendix 2 Figure S4: Namibia mortality rates, 1950−2023

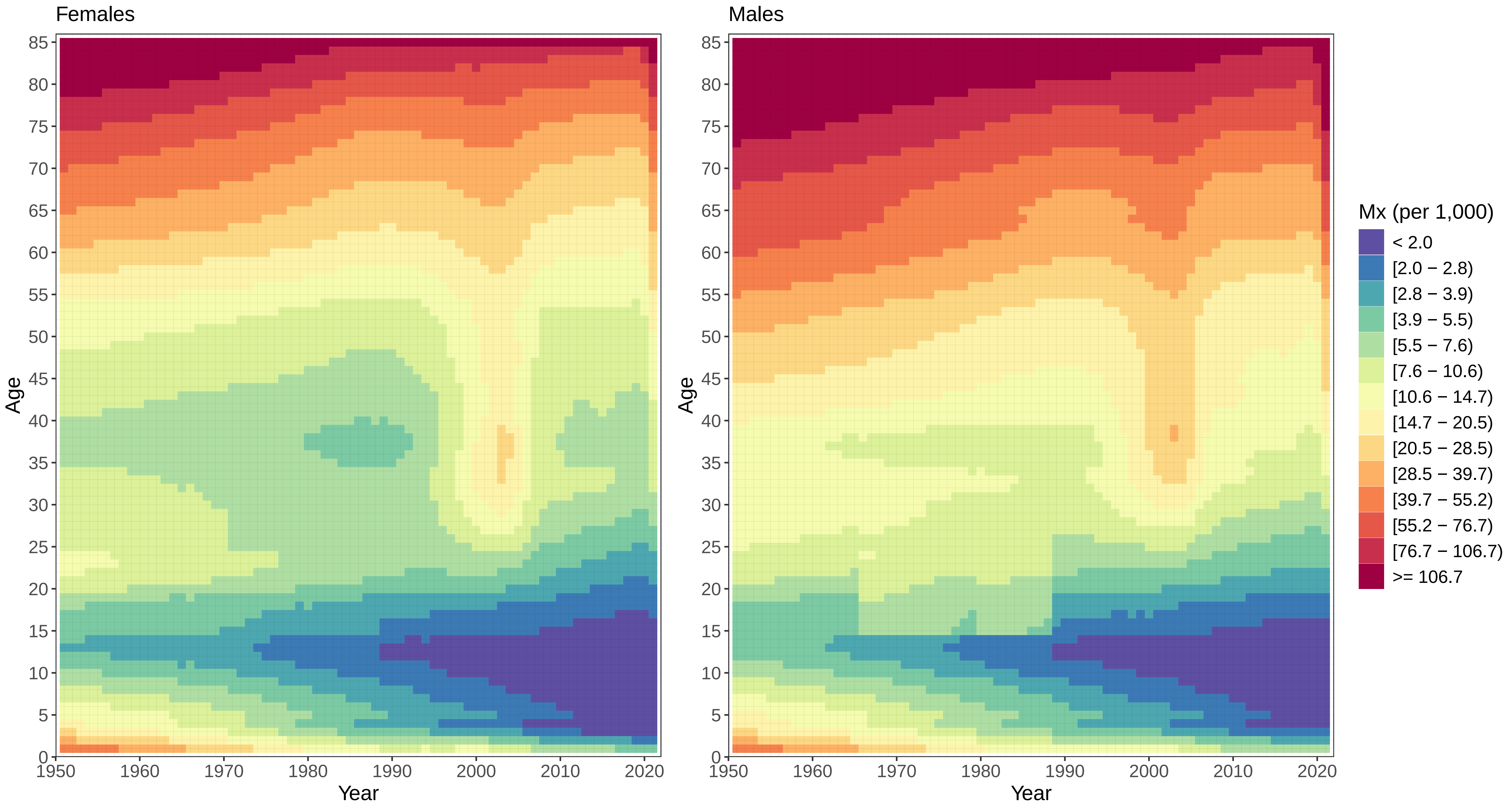

Appendix 2 Figure S4: South Africa mortality rates, 1950−2023

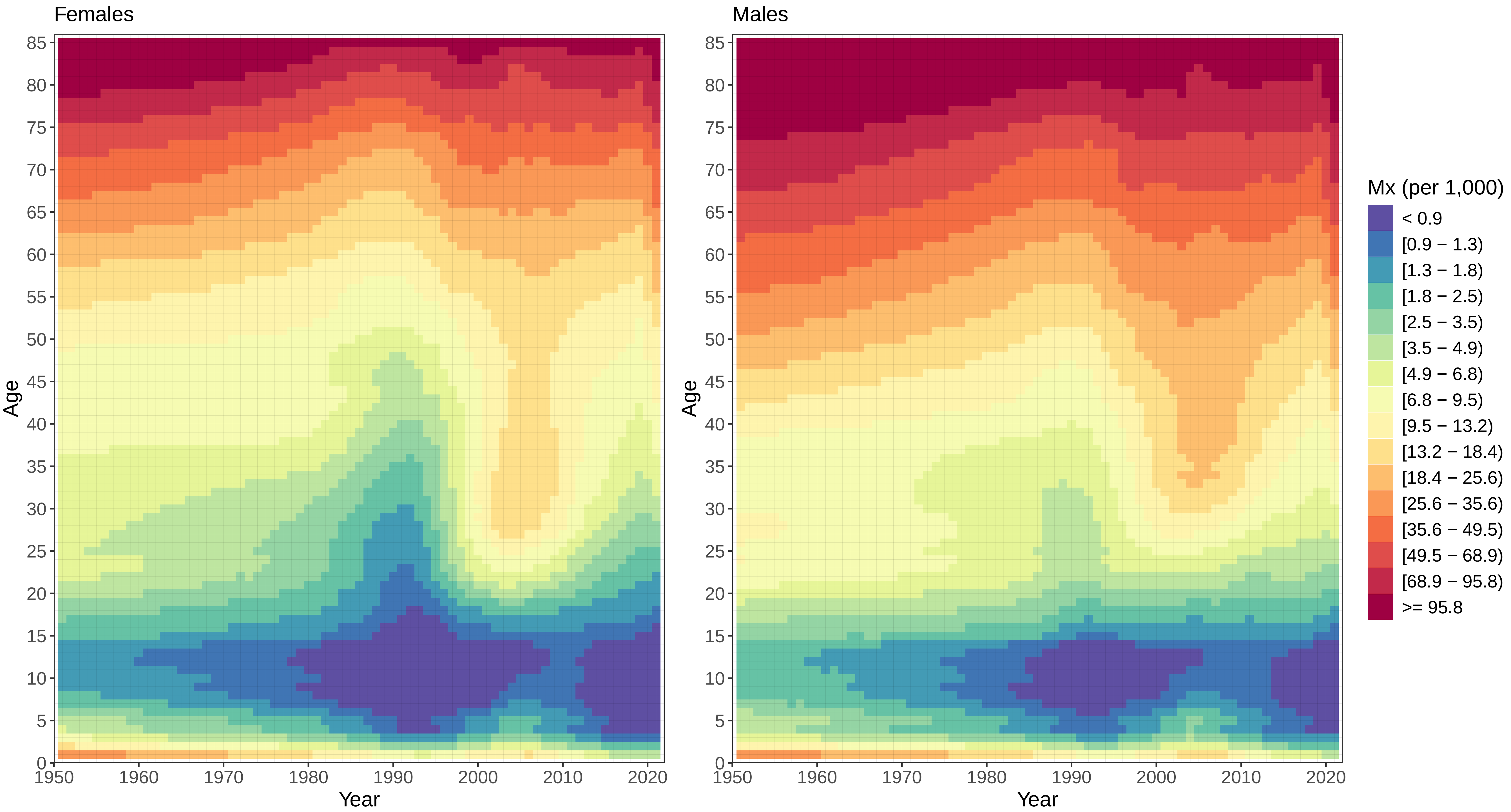

Appendix 2 Figure S4: Zimbabwe mortality rates, 1950−2023

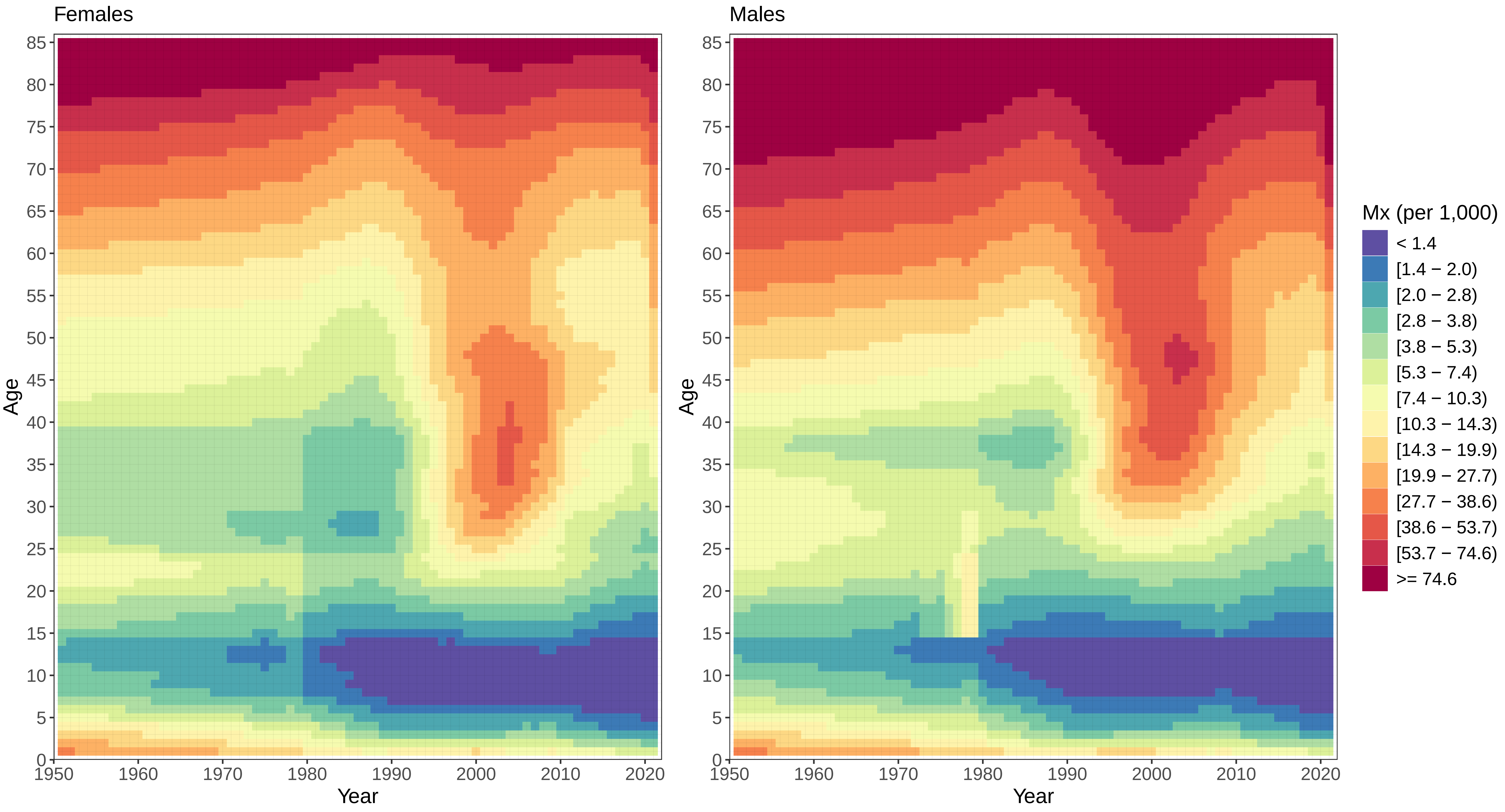

Appendix 2 Figure S4: Benin mortality rates, 1950−2023

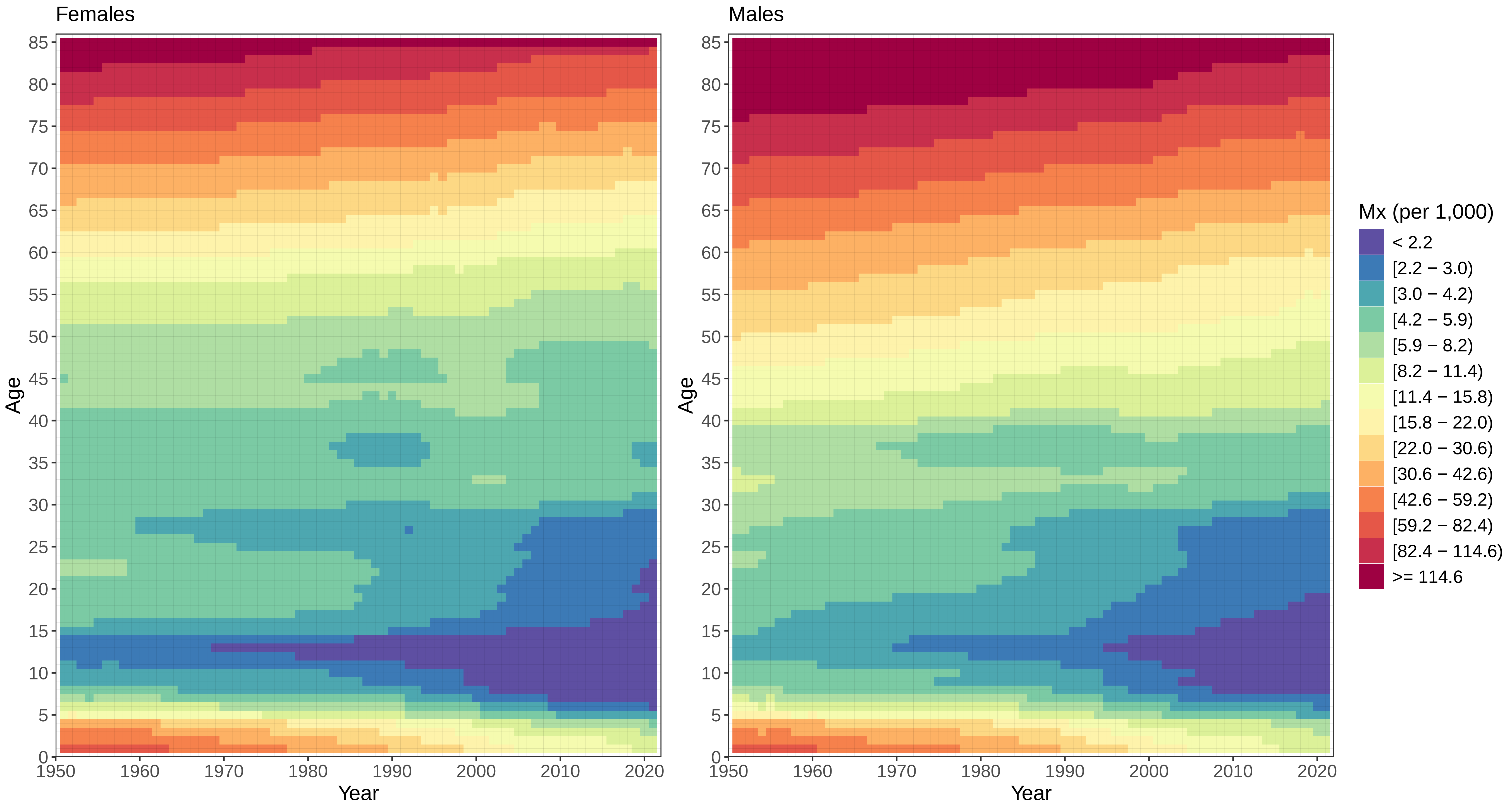

Appendix 2 Figure S4: Burkina Faso mortality rates, 1950−2023

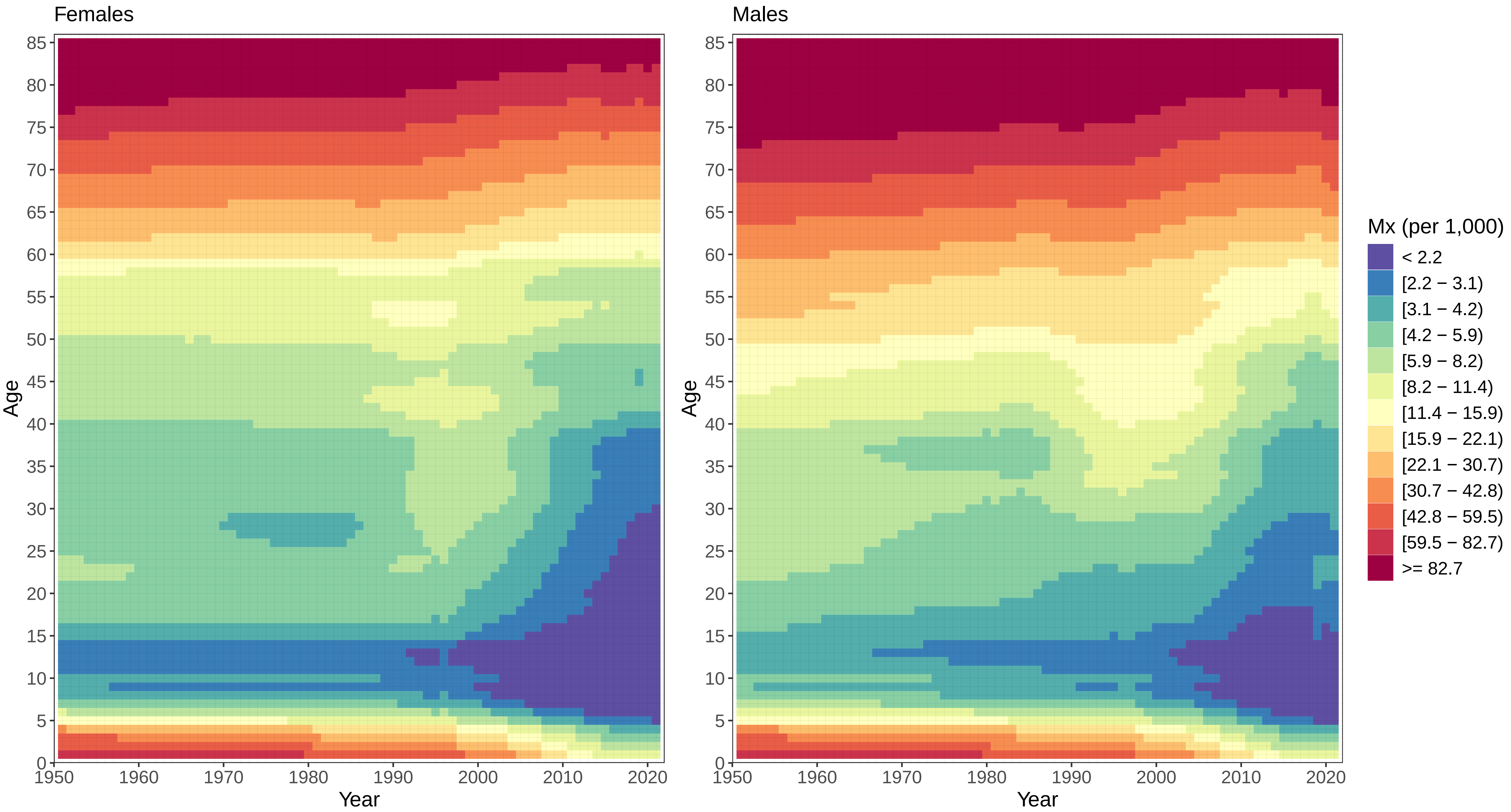

Appendix 2 Figure S4: Cabo Verde mortality rates, 1950−2023

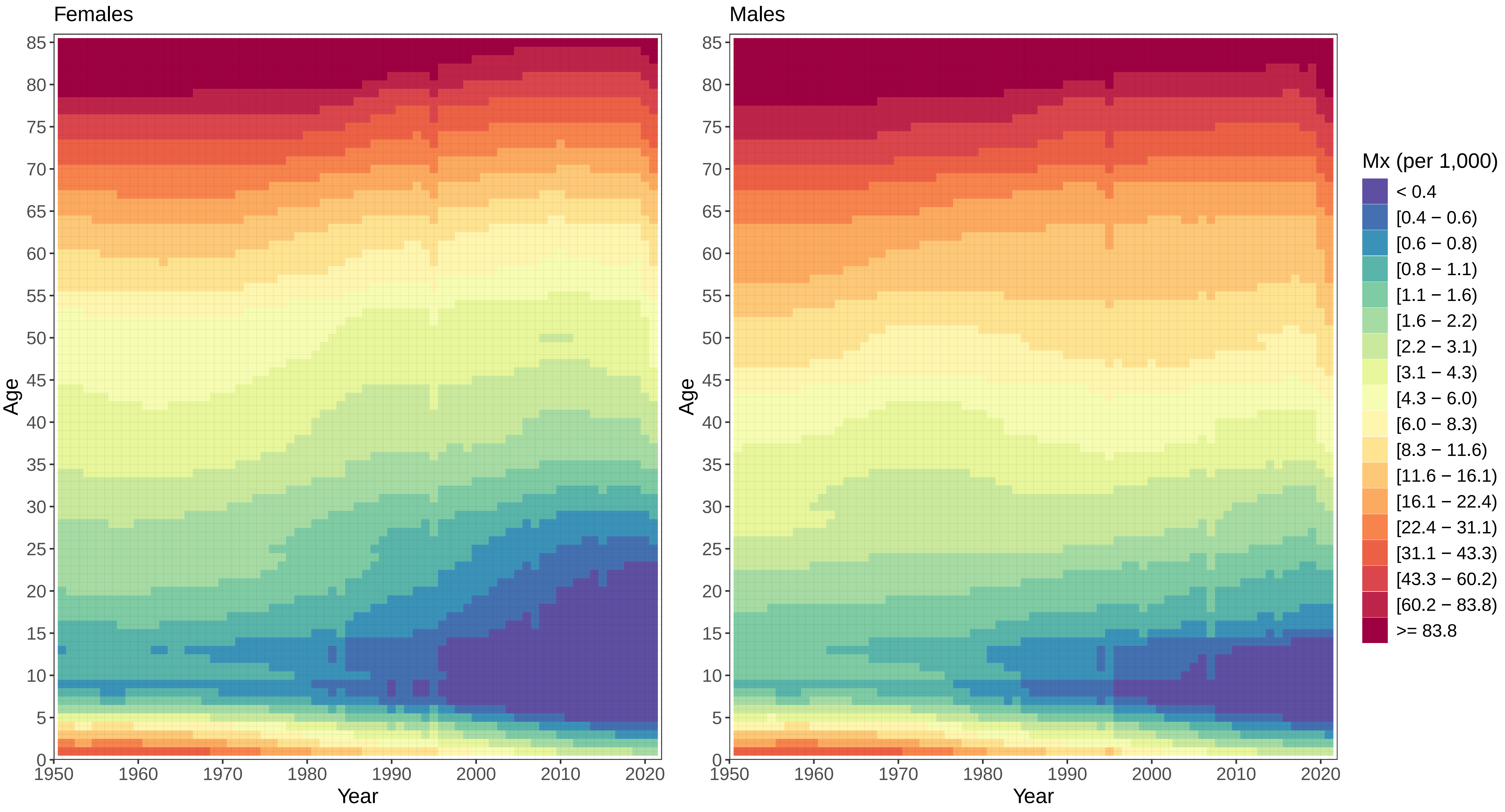

Appendix 2 Figure S4: Cameroon mortality rates, 1950−2023

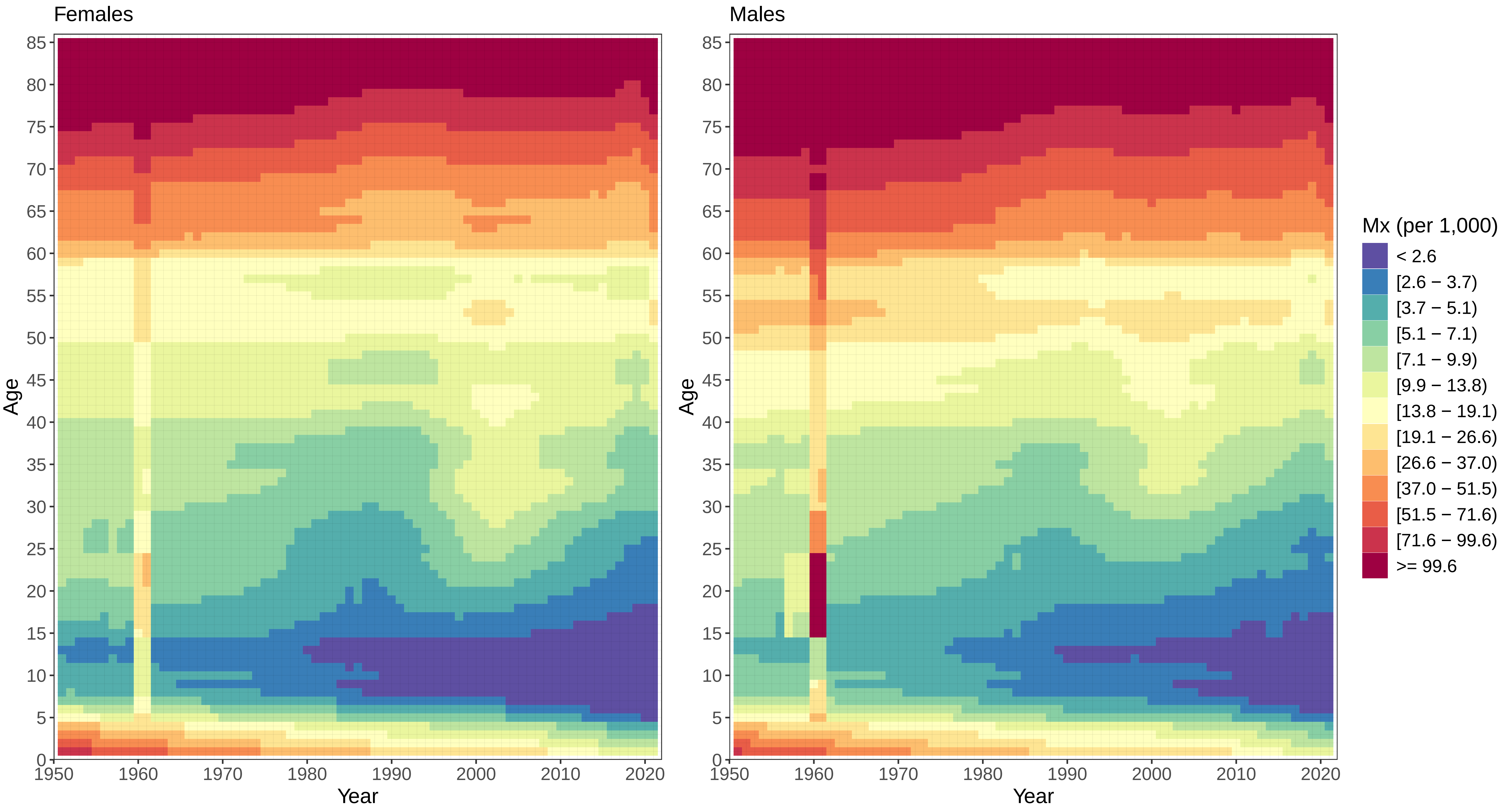

Appendix 2 Figure S4: Chad mortality rates, 1950−2023

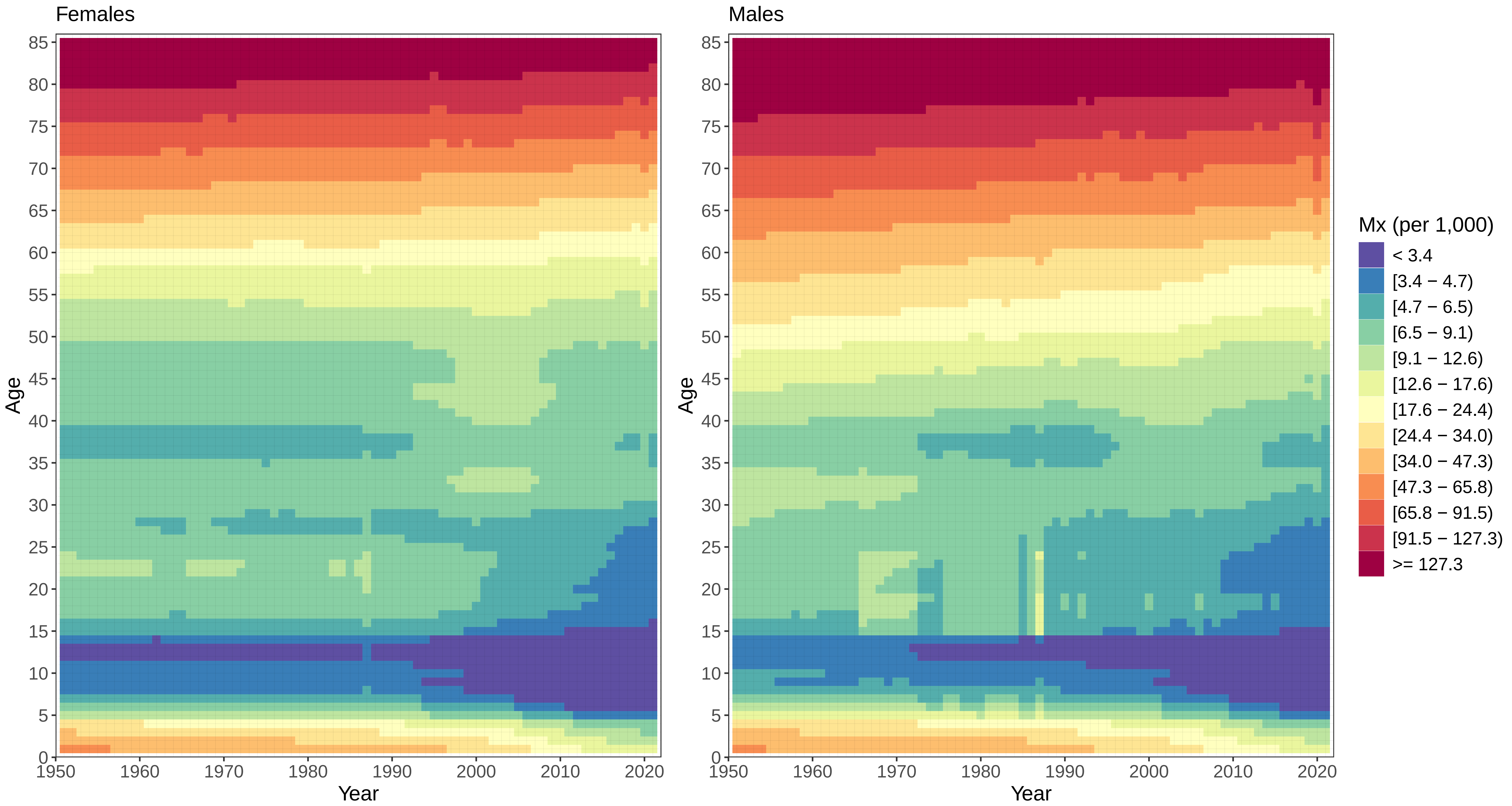

Appendix 2 Figure S4: Côte d'Ivoire mortality rates, 1950−2023

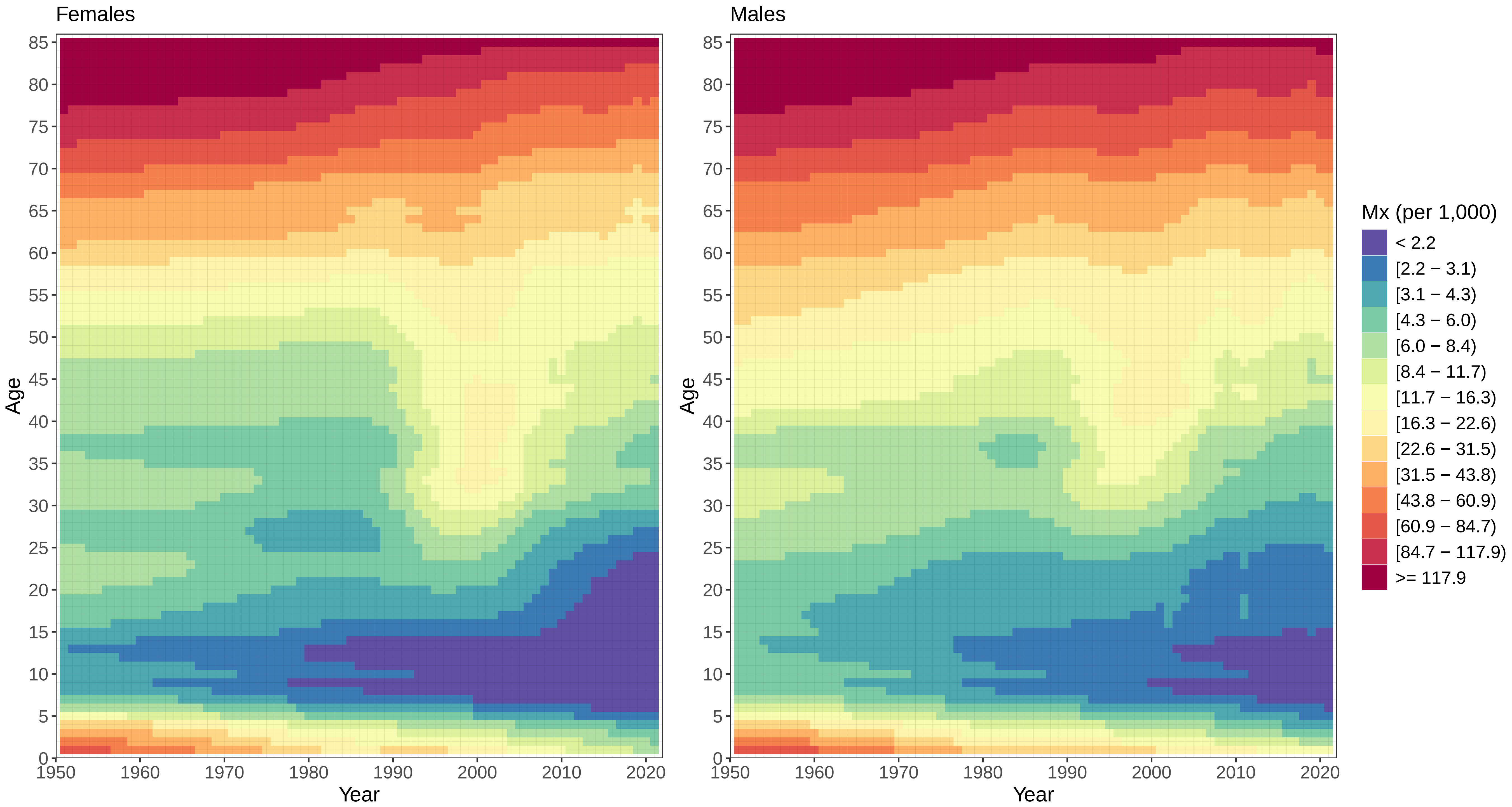

Appendix 2 Figure S4: Ghana mortality rates, 1950−2023

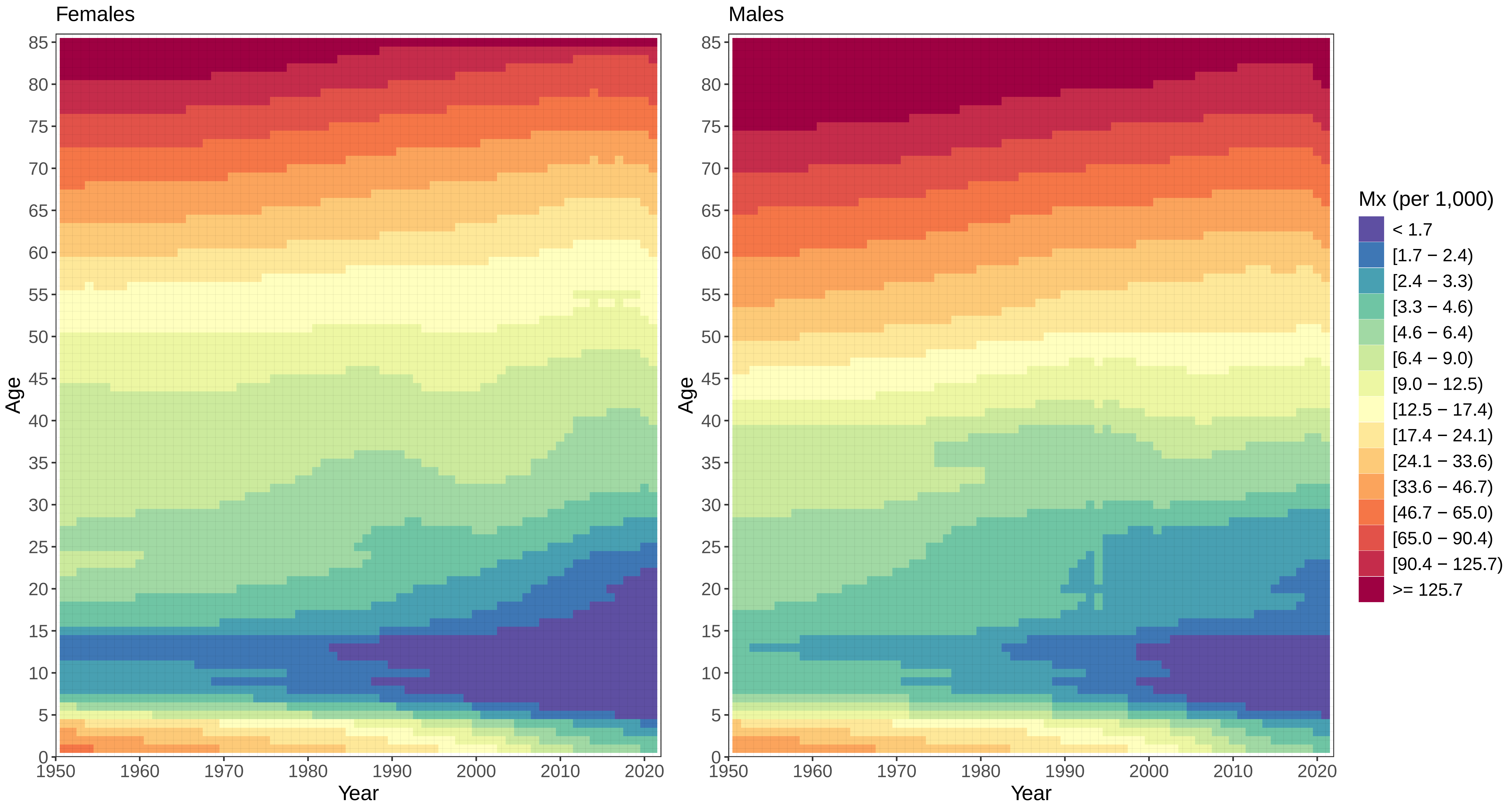

Appendix 2 Figure S4: Guinea mortality rates, 1950−2023

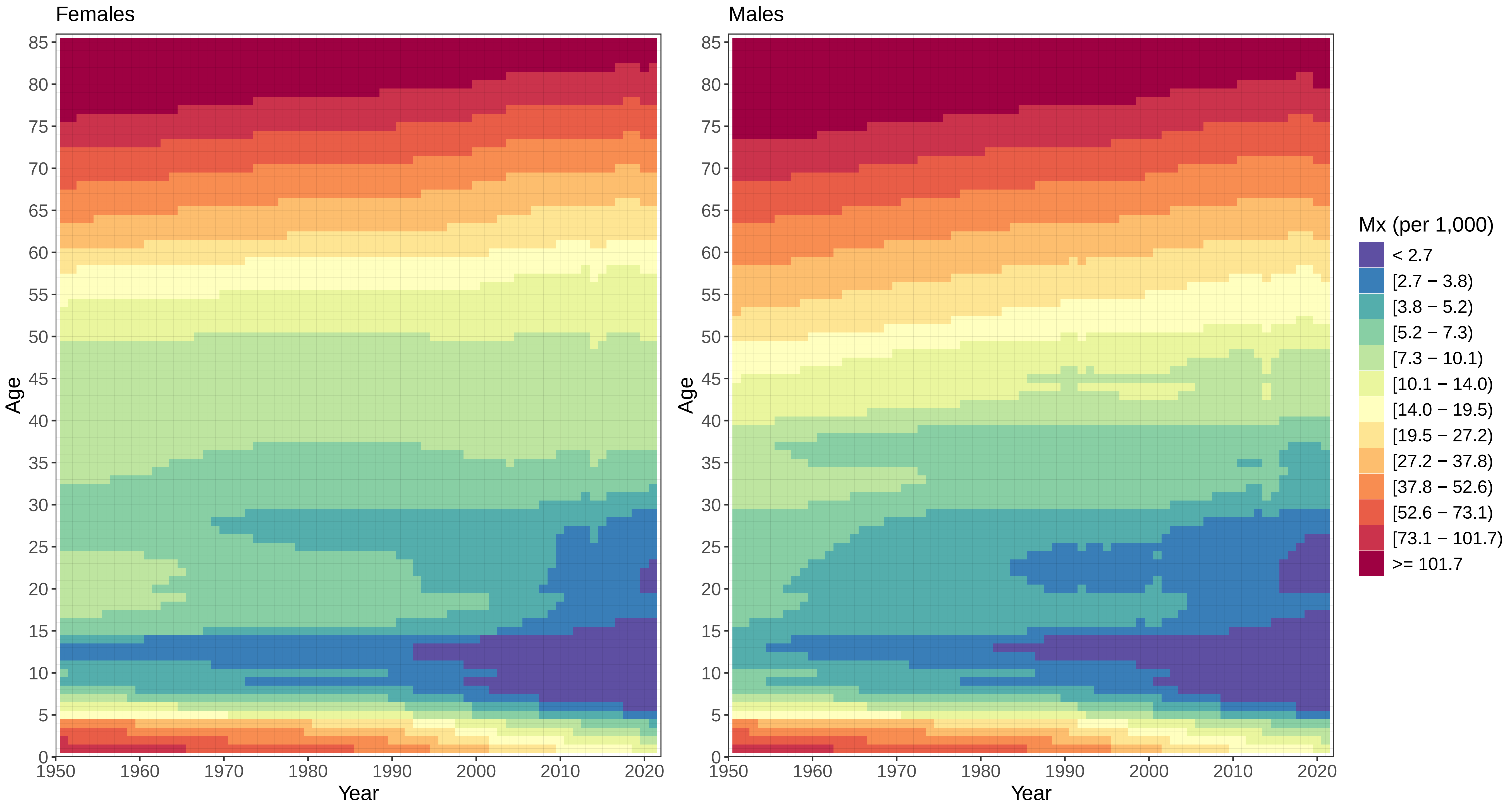

Appendix 2 Figure S4: Guinea−Bissau mortality rates, 1950−2023

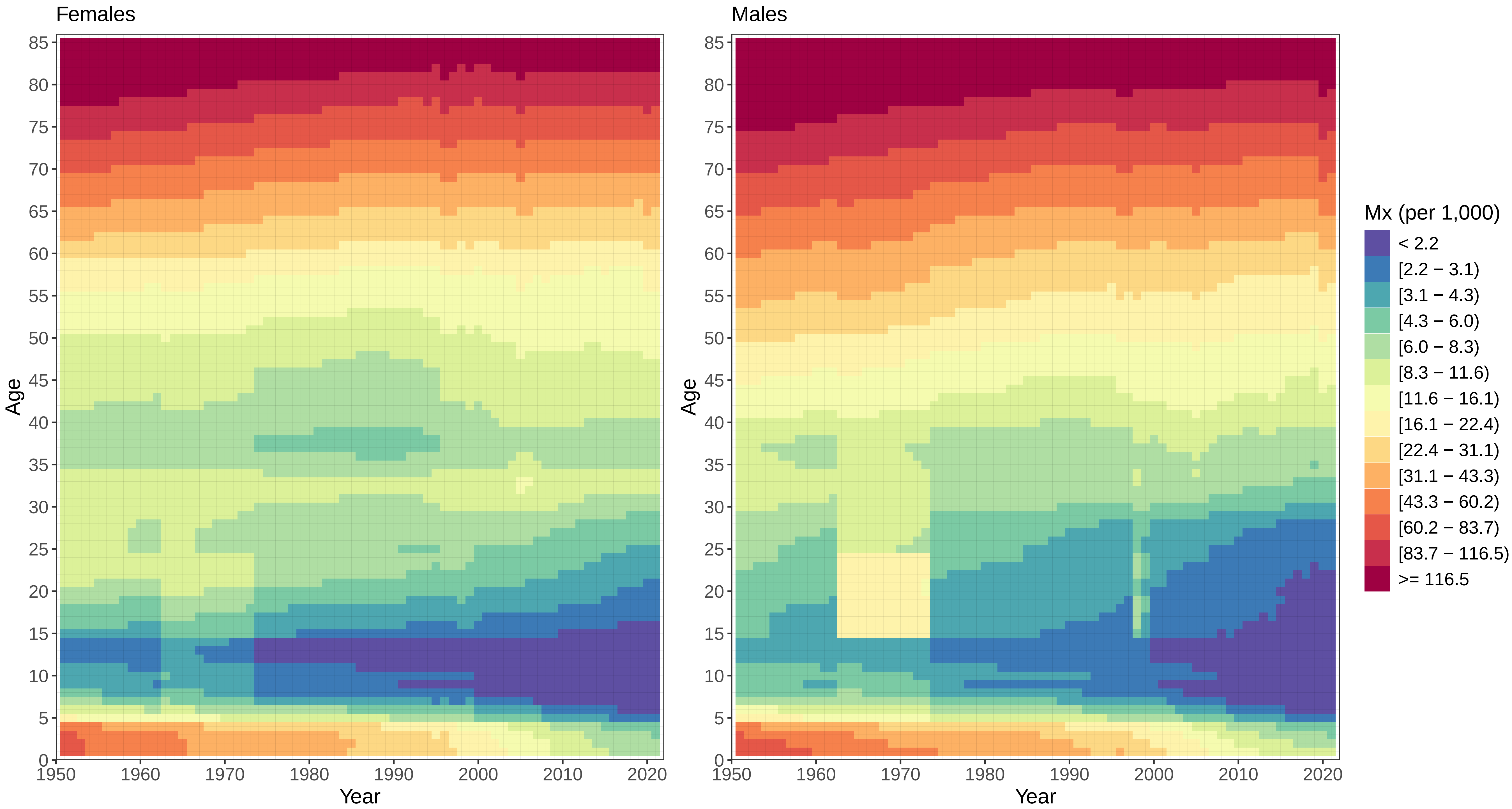

Appendix 2 Figure S4: Liberia mortality rates, 1950−2023

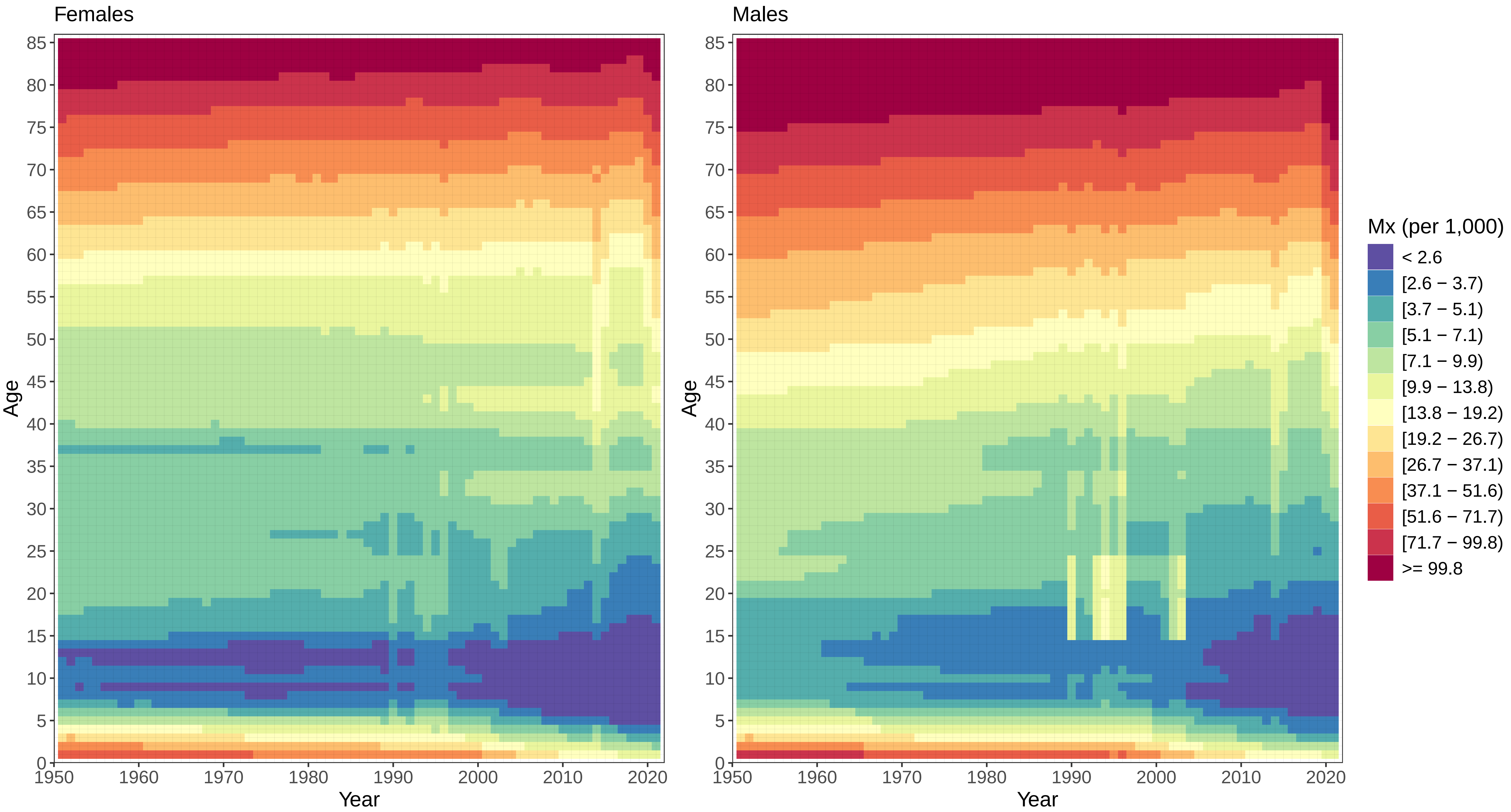

Appendix 2 Figure S4: Mali mortality rates, 1950−2023

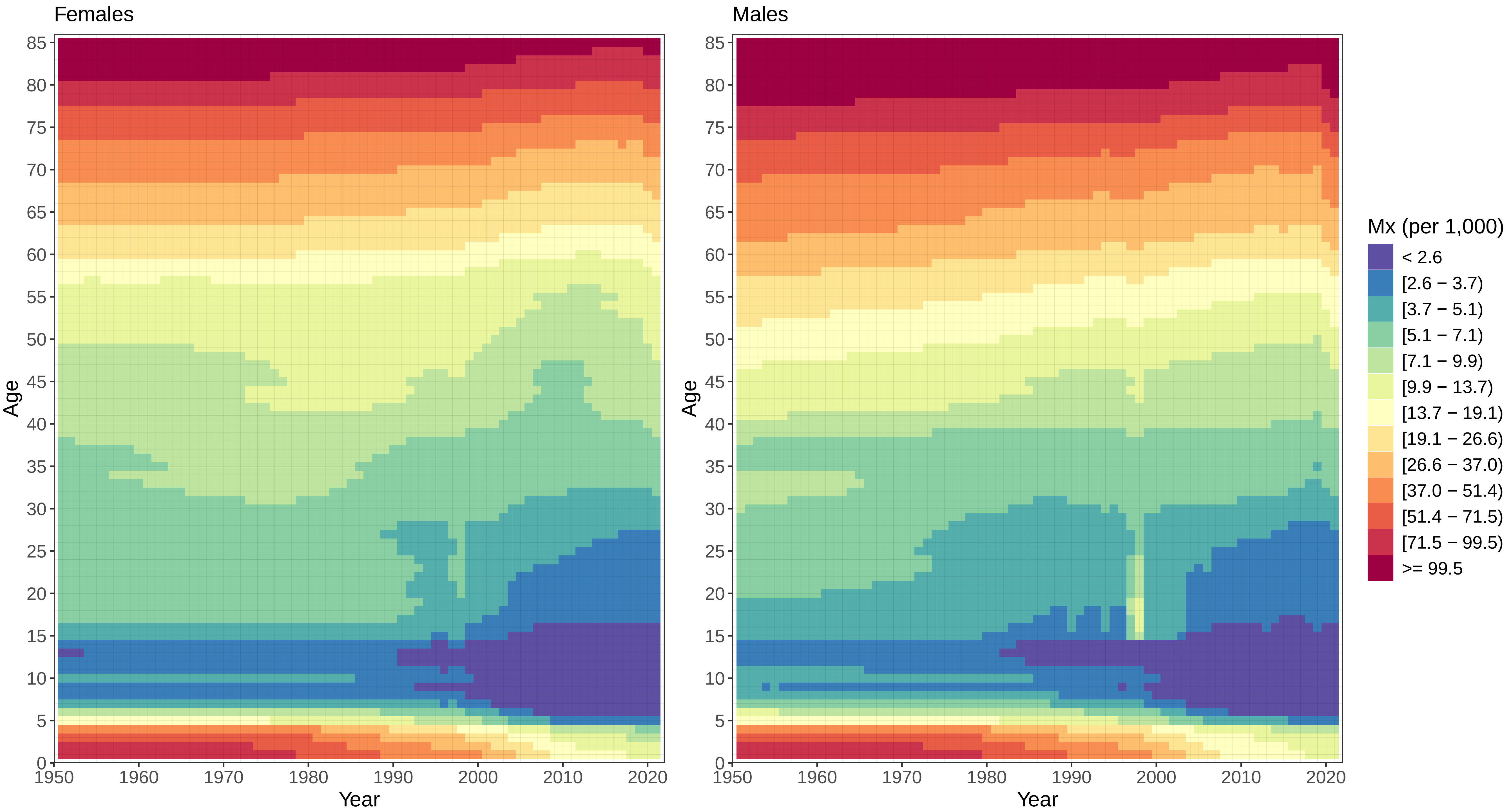

Appendix 2 Figure S4: Mauritania mortality rates, 1950−2023

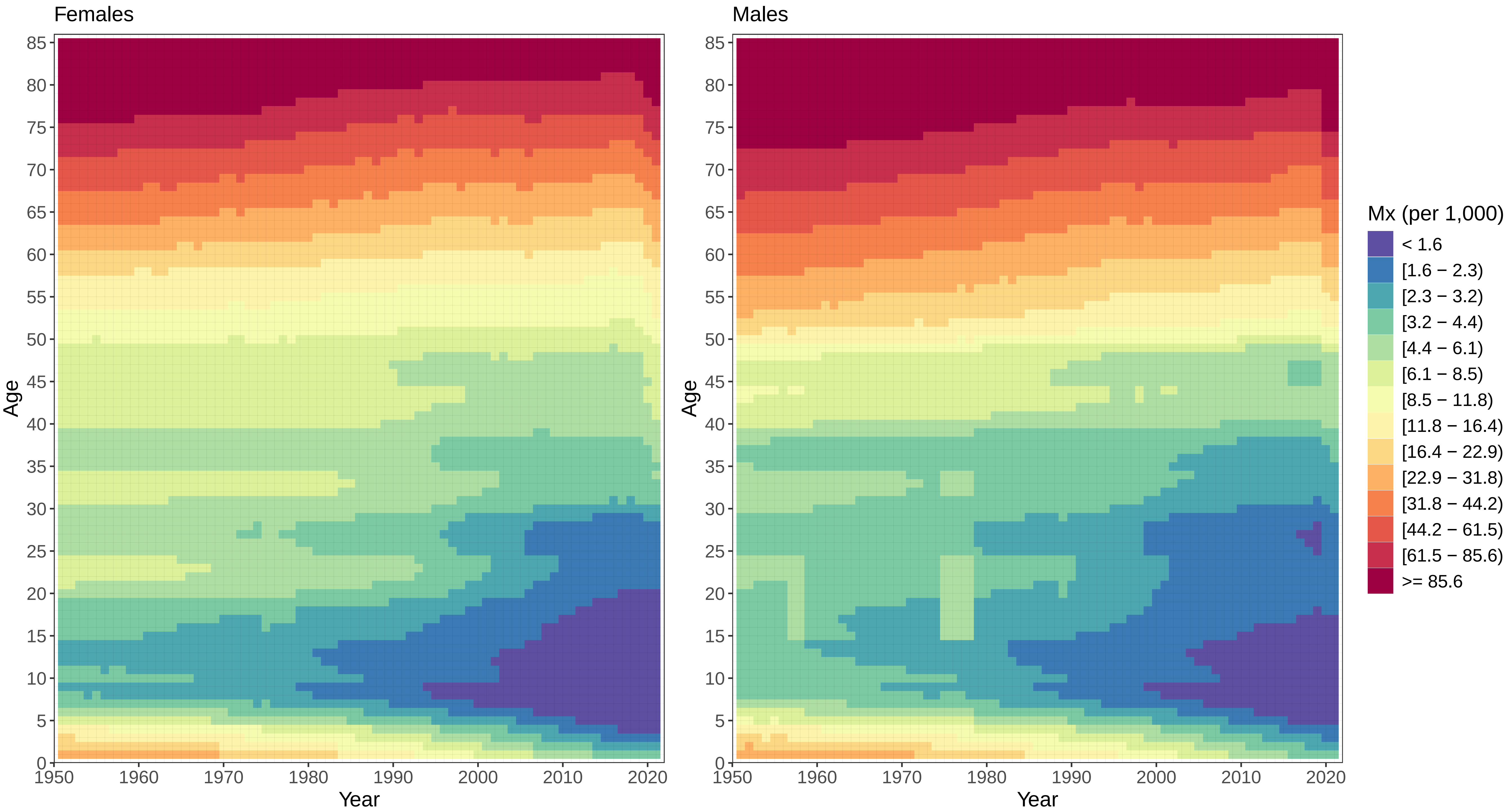

Appendix 2 Figure S4: Niger mortality rates, 1950−2023

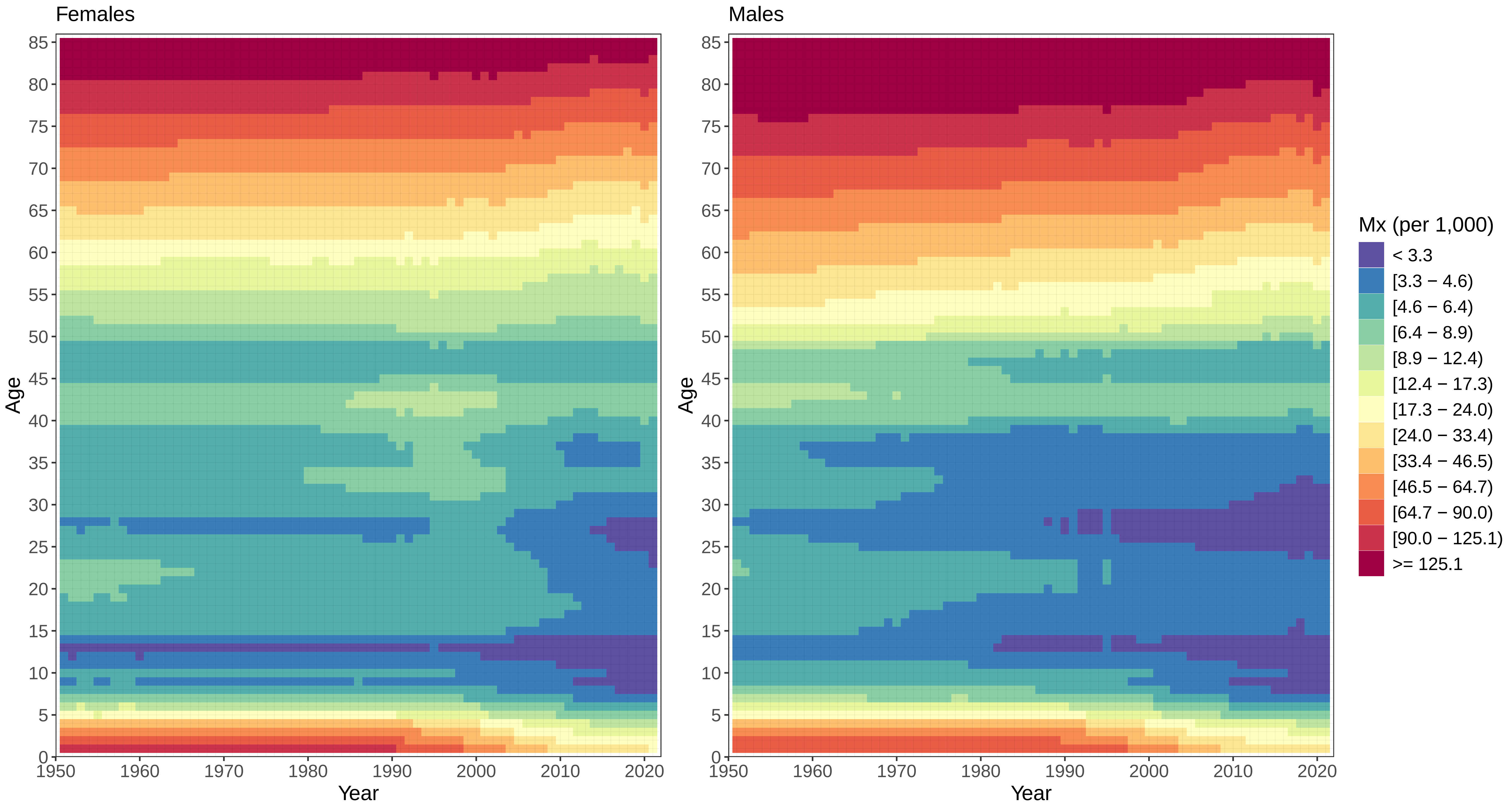

Appendix 2 Figure S4: Nigeria mortality rates, 1950−2023

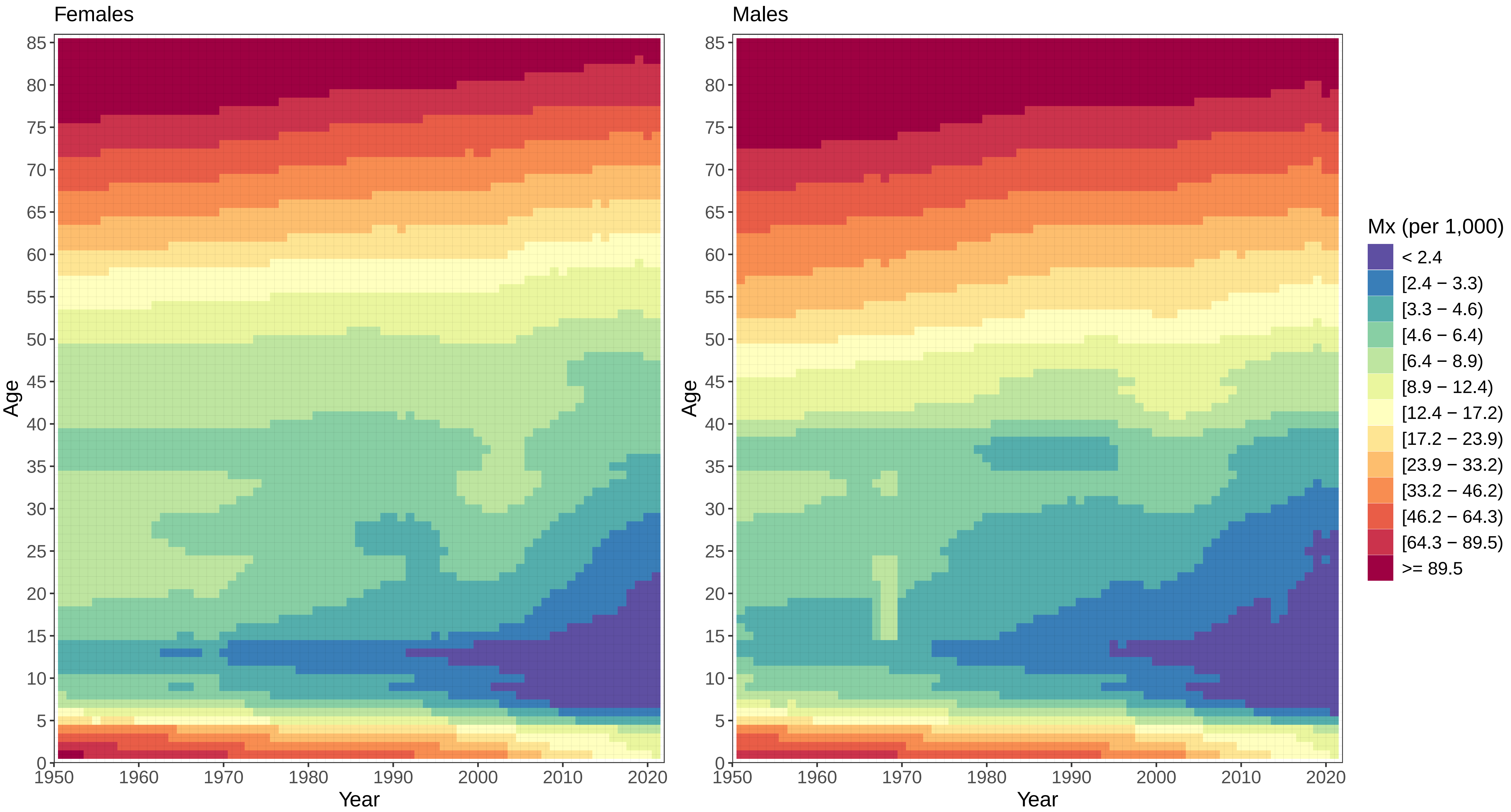

Appendix 2 Figure S4: São Tomé and Príncipe mortality rates, 1950−2023

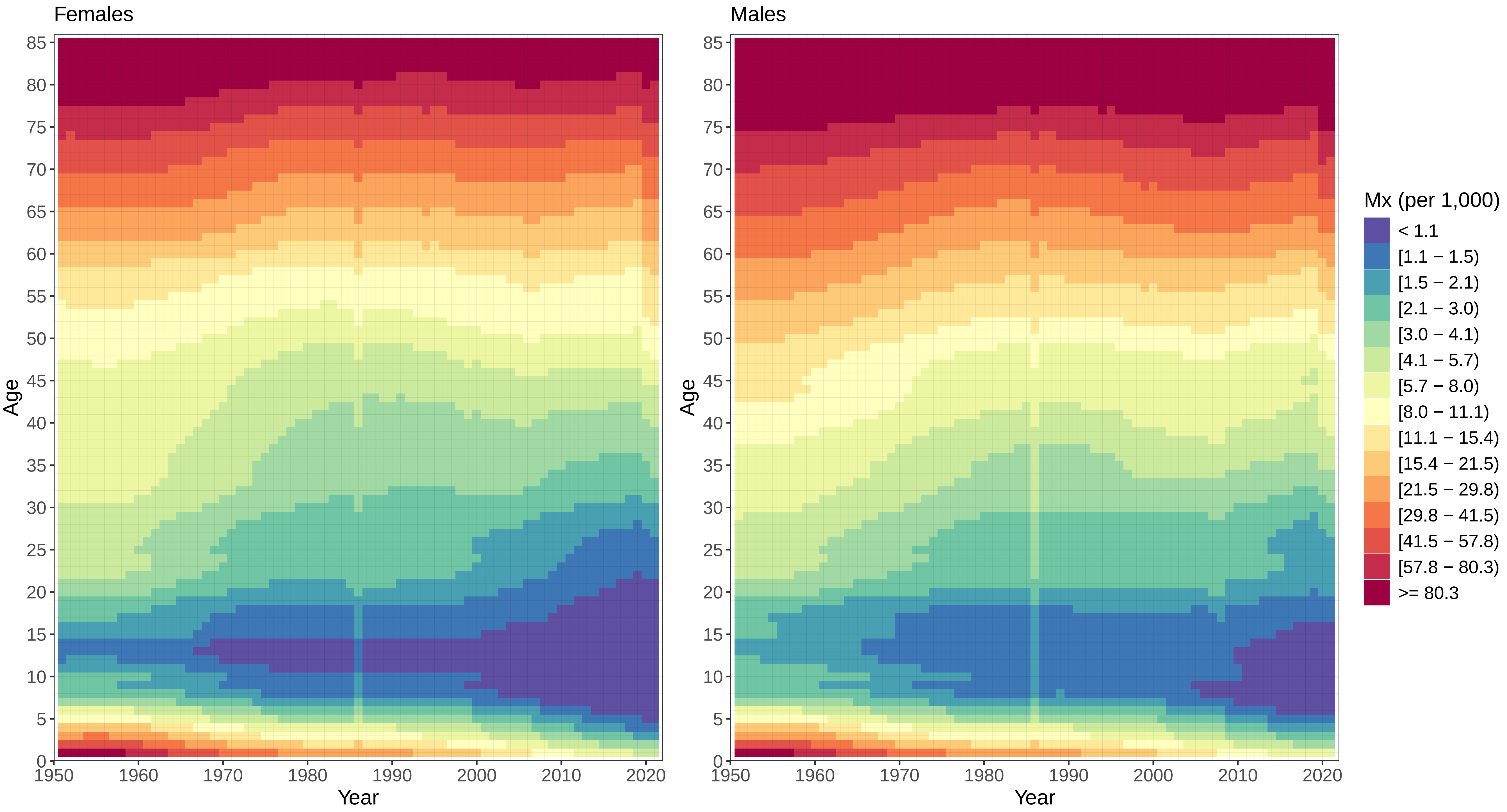

Appendix 2 Figure S4: Senegal mortality rates, 1950−2023

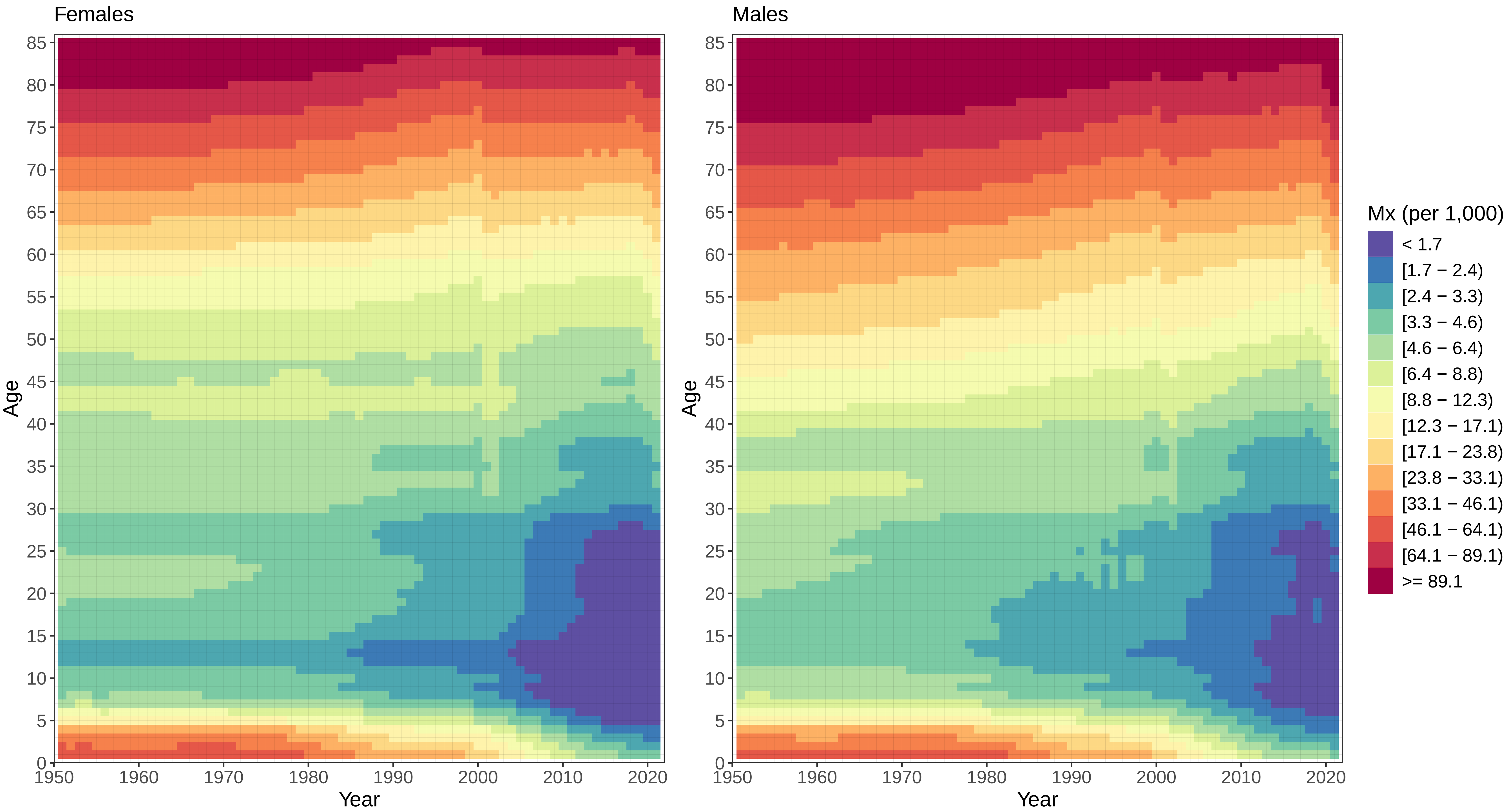

Appendix 2 Figure S4: Sierra Leone mortality rates, 1950−2023

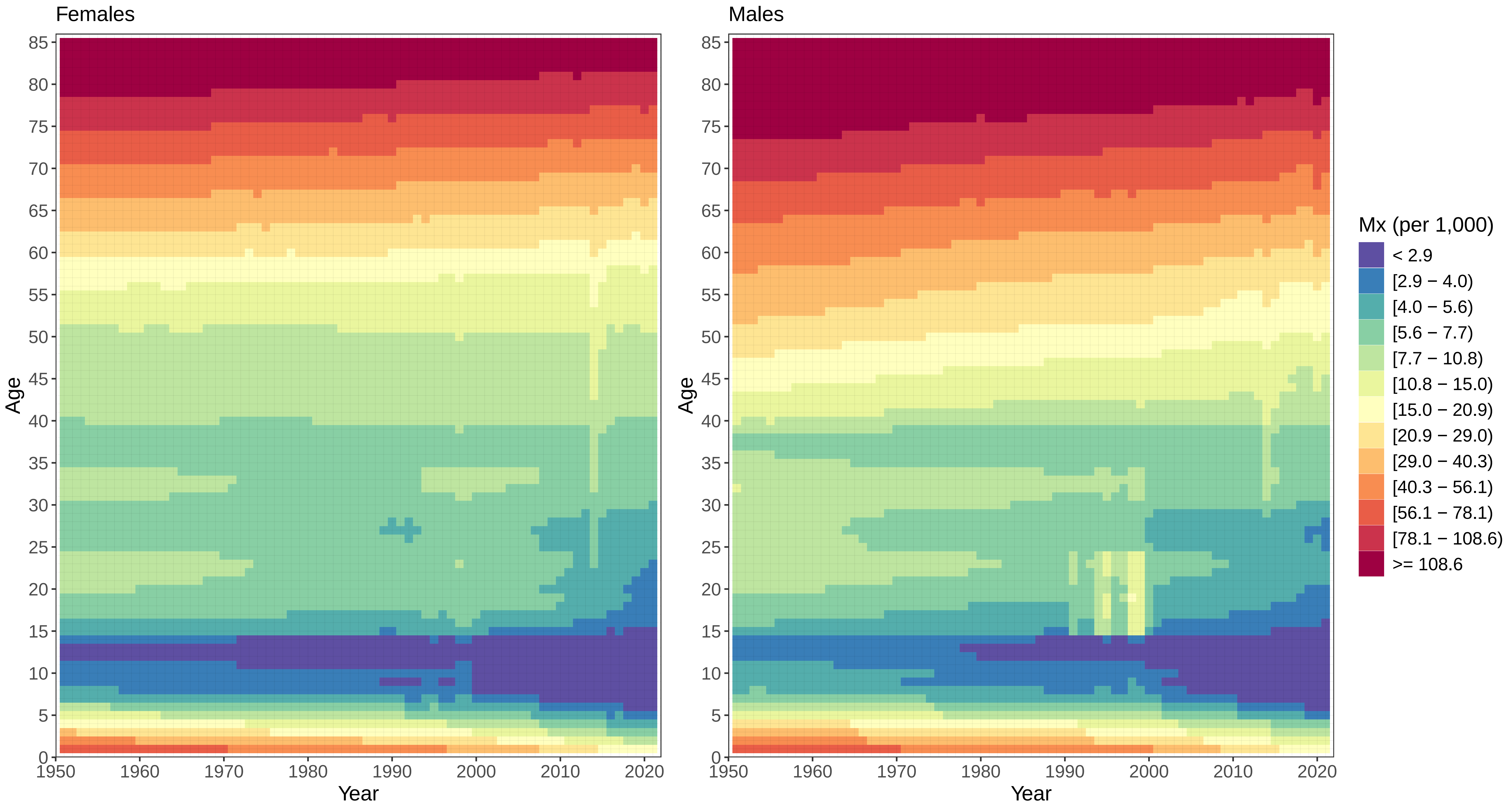

Appendix 2 Figure S4: The Gambia mortality rates, 1950−2023

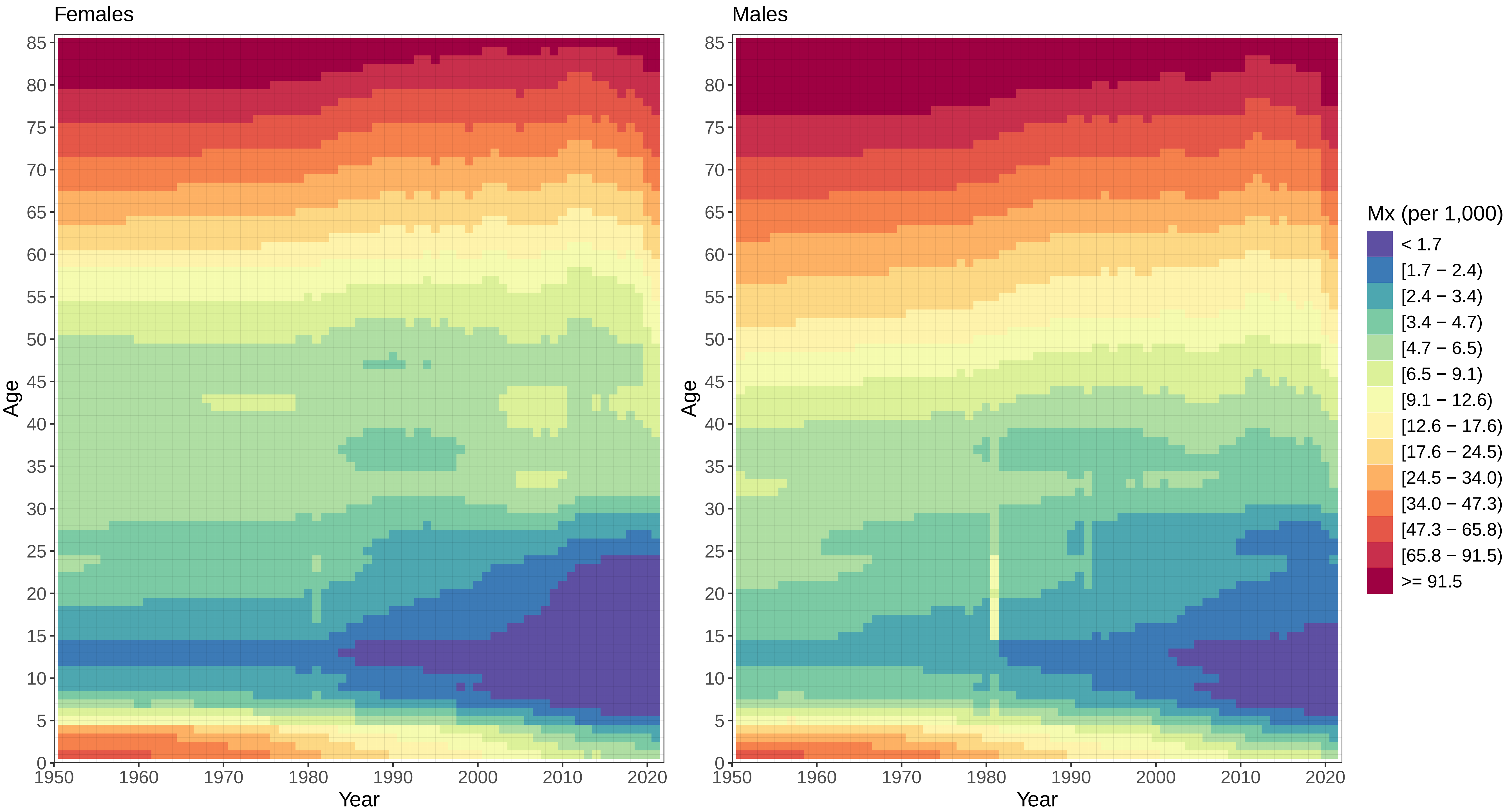

Appendix 2 Figure S4: Togo mortality rates, 1950−2023

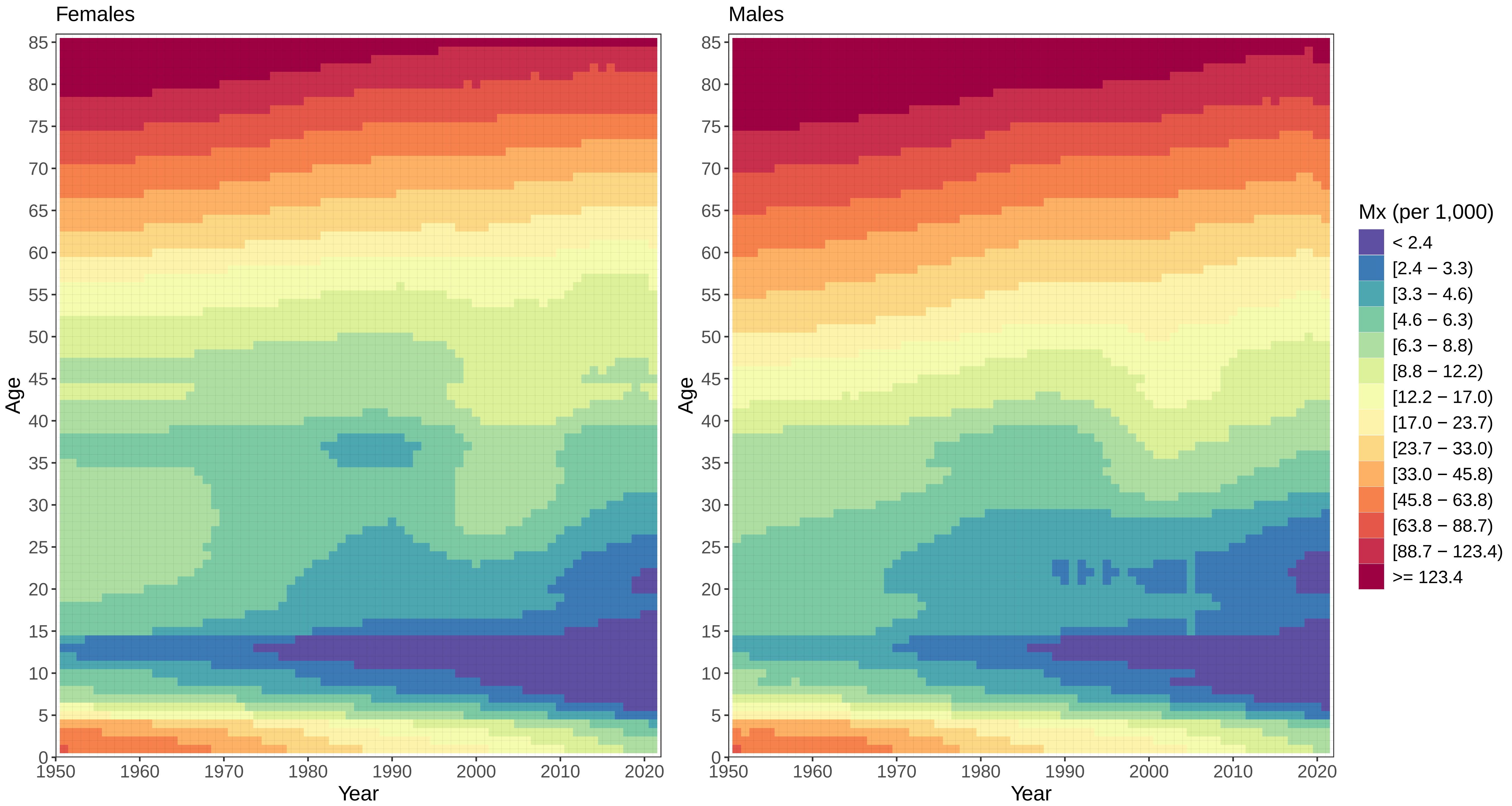

Appendix 2 Figure S5: National Estimated − Expected Life Expectancy based on SDI, by sex, 1950−2023

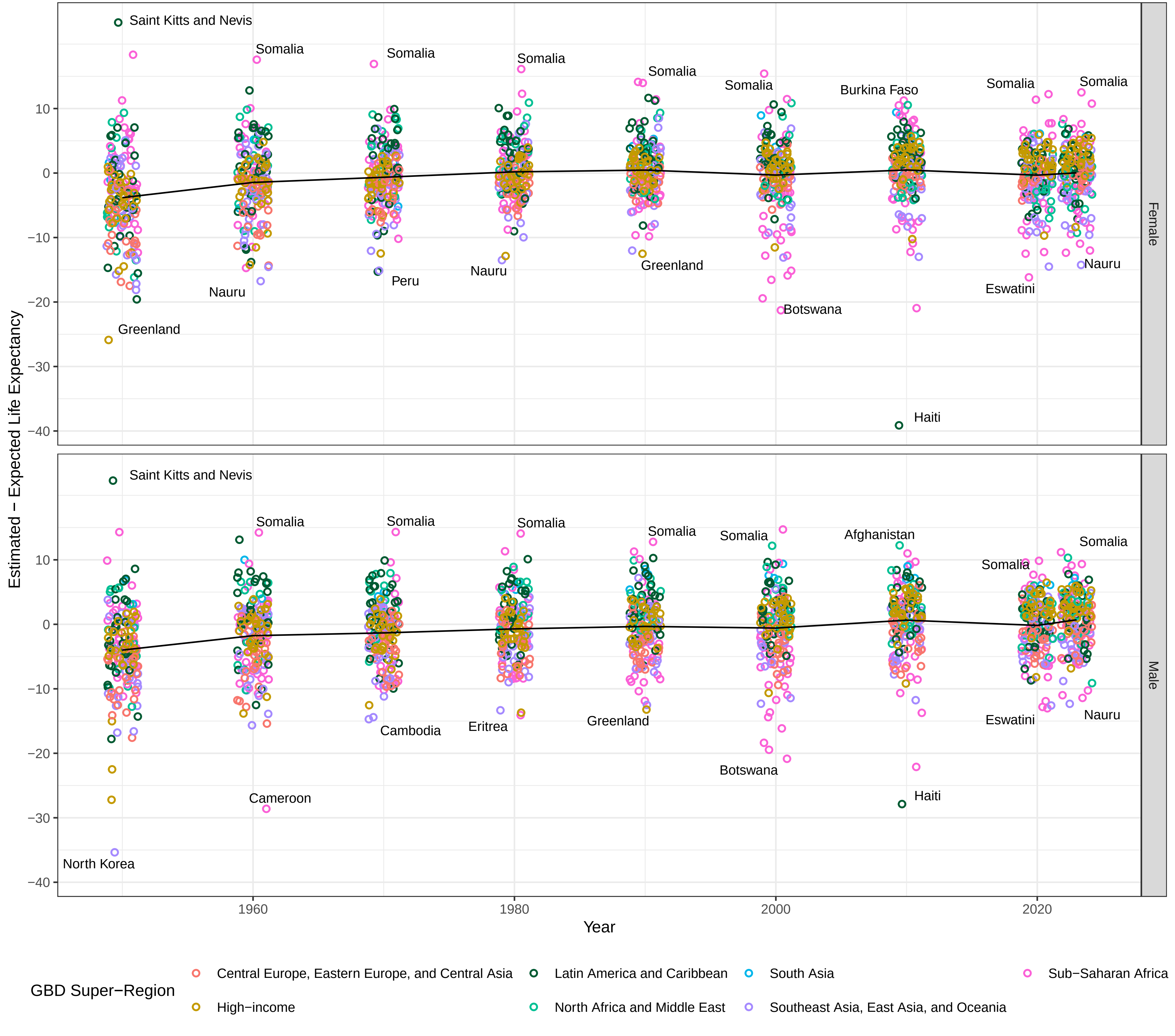

Appendix 2 Figure S6: Comparison of under 5 mortality estimates for 2023 between GBD 2023 and UNICEF 2025, by location

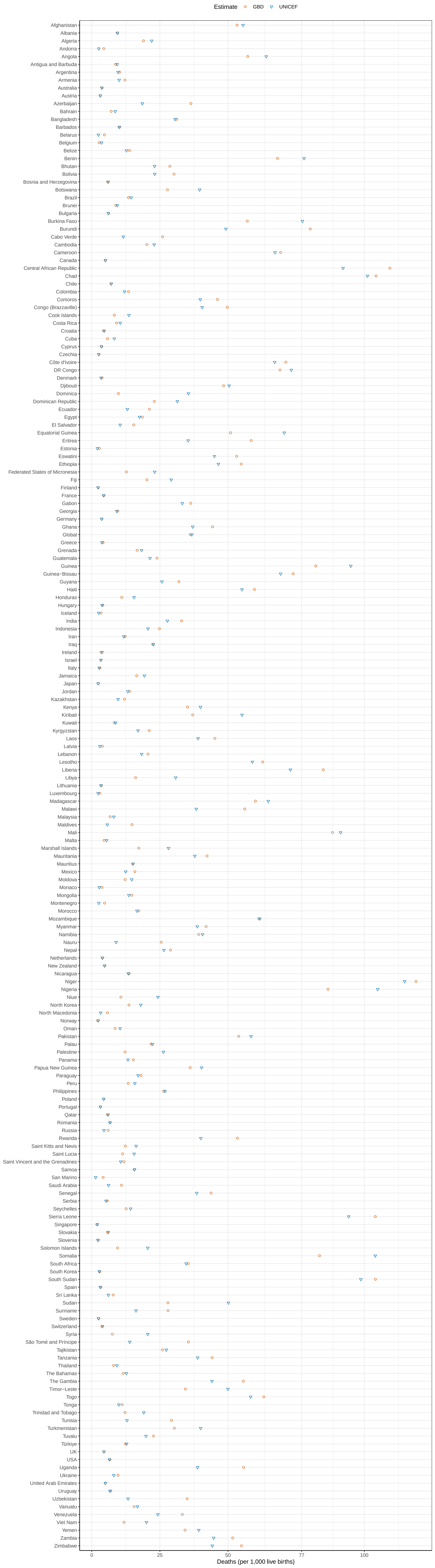

Appendix 2 Figure S7: Comparison of life expectancy at birth estimates for 2023 between GBD 2023 and WPP 2024, by sex and location

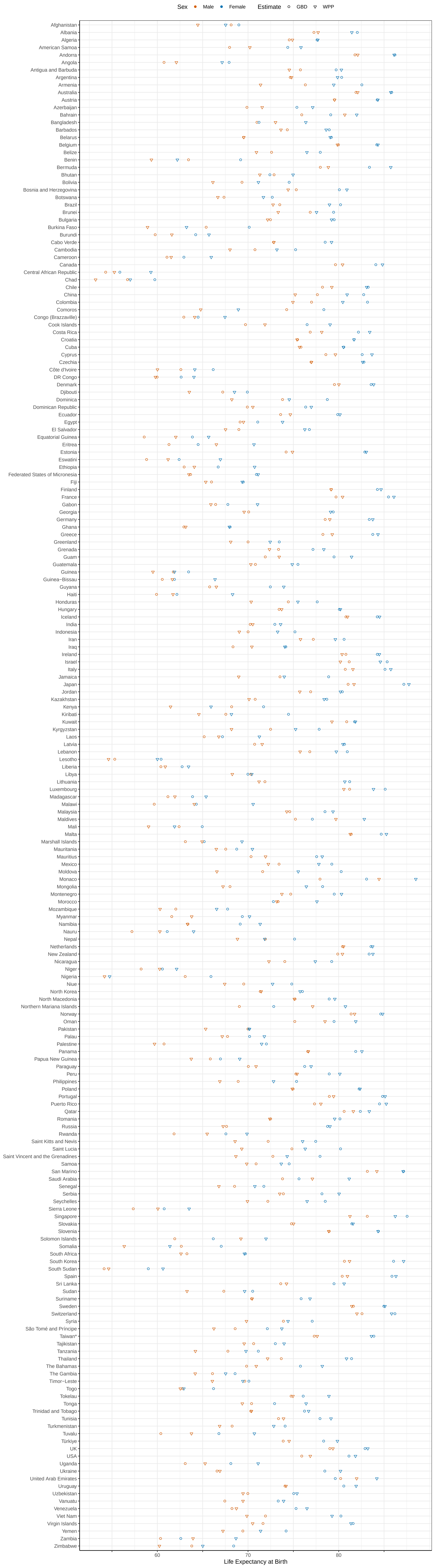

Appendix 2 Figure S8: Comparison of life expectancy at birth estimates for countries in GBD 2023 and WPP 2024 for 1950, both sexes combined

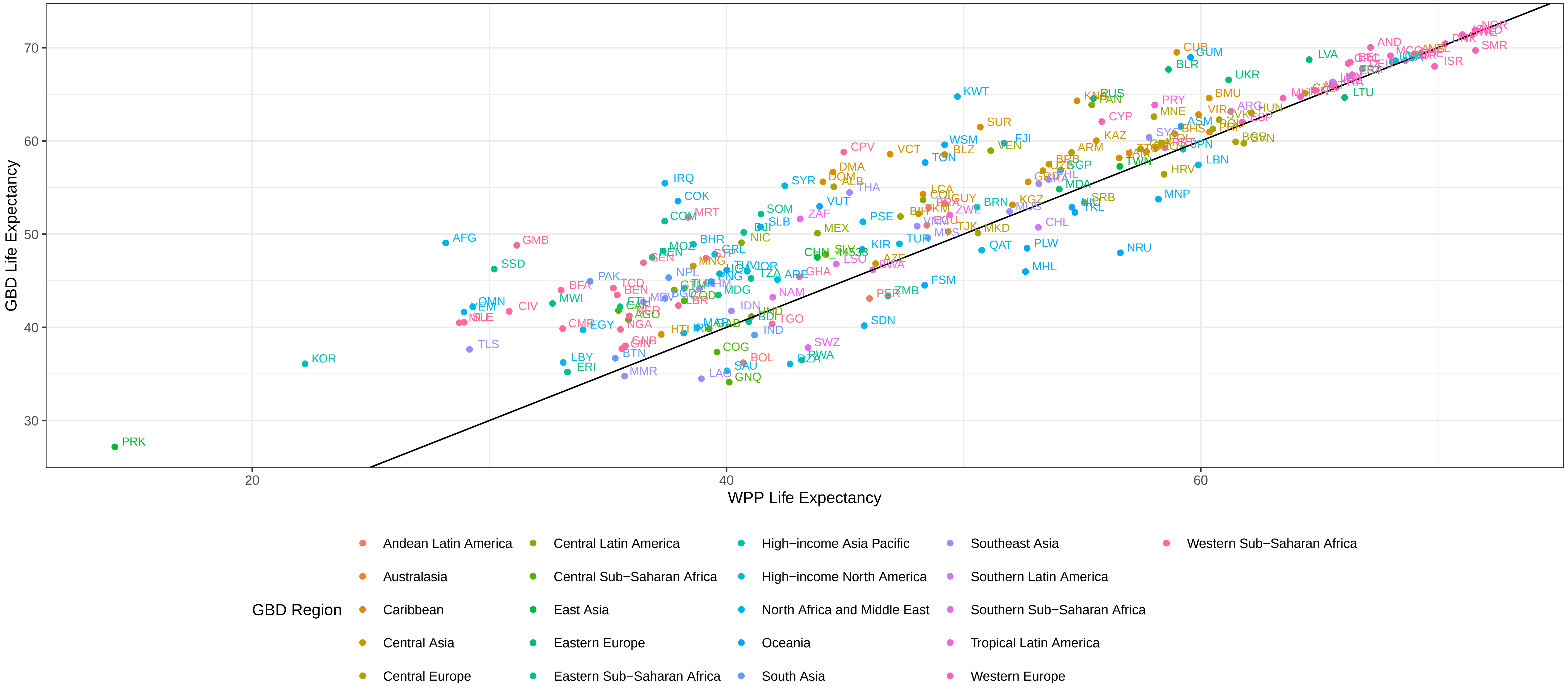

Appendix 2 Figure S8: Comparison of life expectancy at birth estimates for countries in GBD 2023 and WPP 2024 for 1955, both sexes combined

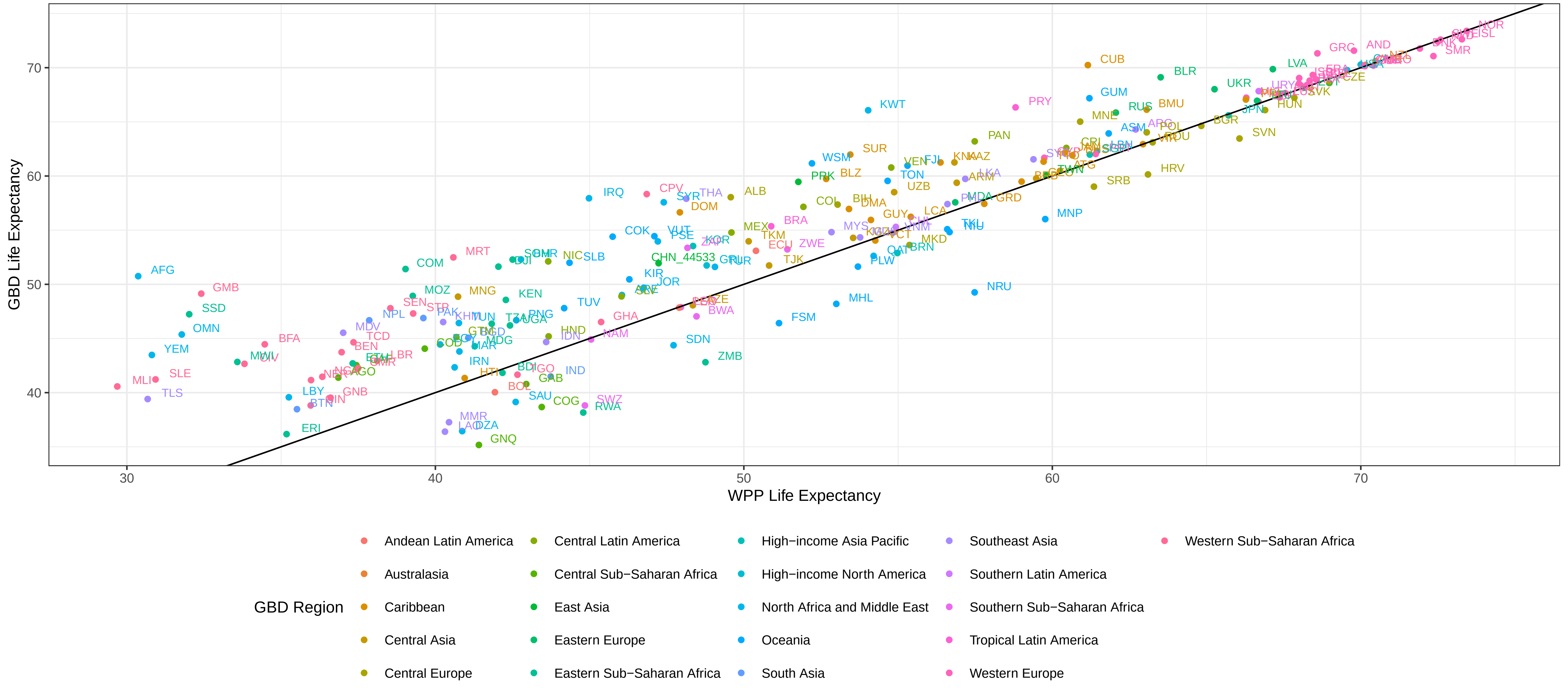

Appendix 2 Figure S8: Comparison of life expectancy at birth estimates for countries in GBD 2023 and WPP 2024 for 1960, both sexes combined

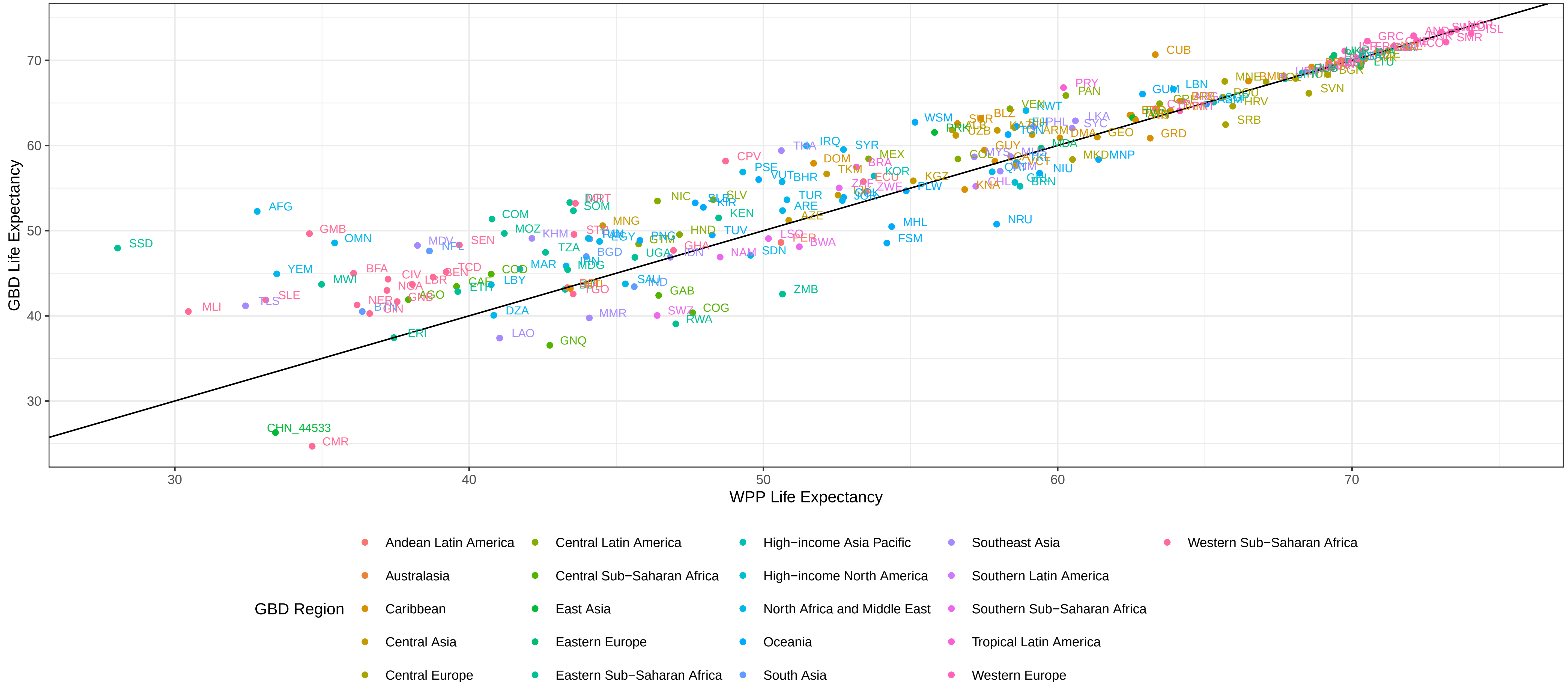

Appendix 2 Figure S8: Comparison of life expectancy at birth estimates for countries in GBD 2023 and WPP 2024 for 1965, both sexes combined

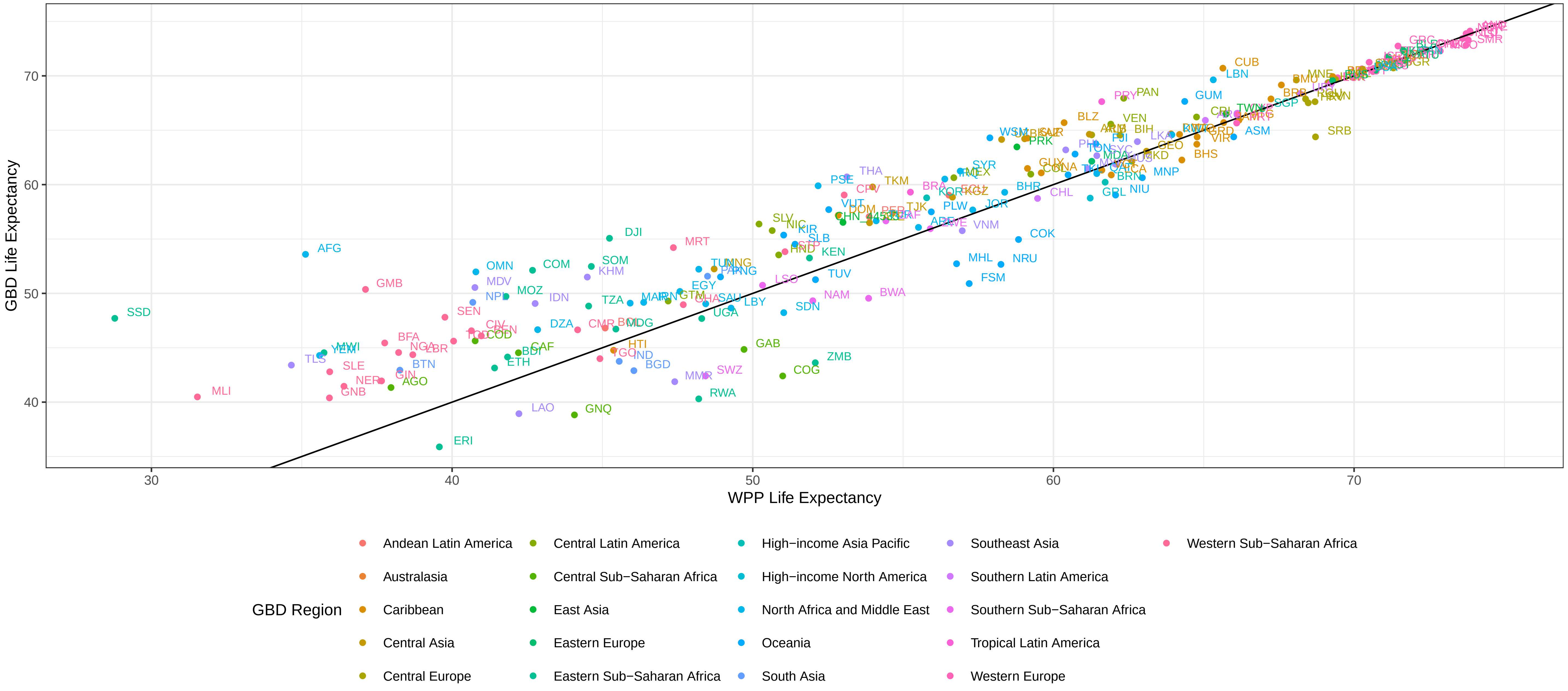

Appendix 2 Figure S8: Comparison of life expectancy at birth estimates for countries in GBD 2023 and WPP 2024 for 1970, both sexes combined

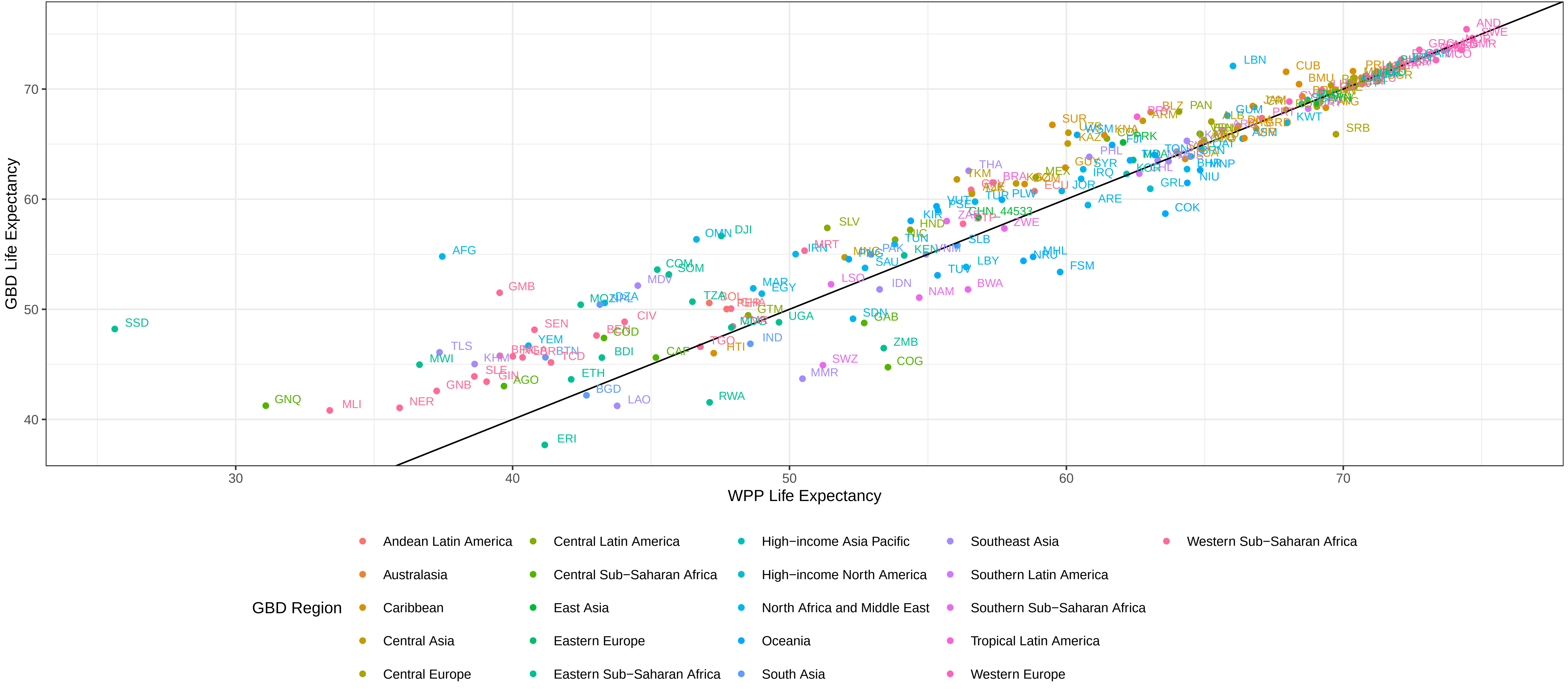

Appendix 2 Figure S8: Comparison of life expectancy at birth estimates for countries in GBD 2023 and WPP 2024 for 1975, both sexes combined

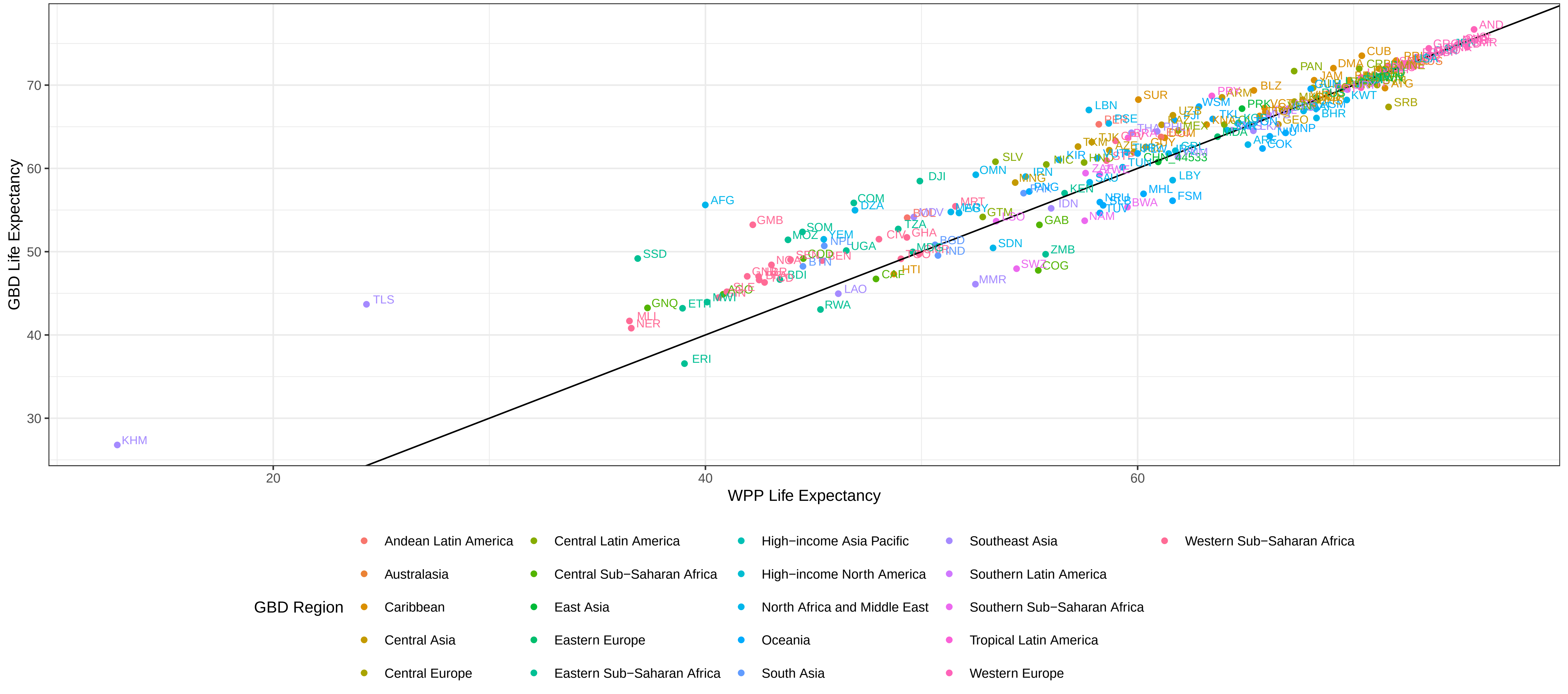

Appendix 2 Figure S8: Comparison of life expectancy at birth estimates for countries in GBD 2023 and WPP 2024 for 1980, both sexes combined

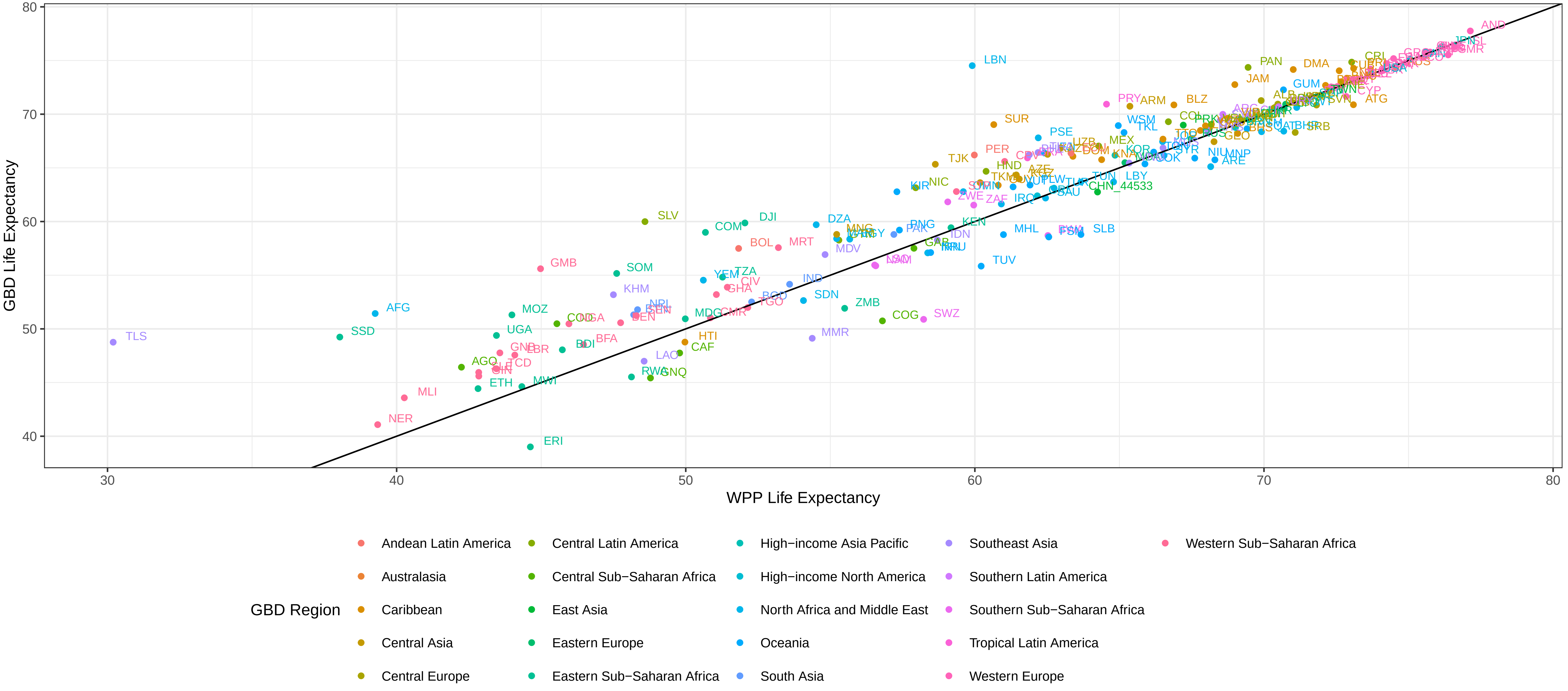

Appendix 2 Figure S8: Comparison of life expectancy at birth estimates for countries in GBD 2023 and WPP 2024 for 1985, both sexes combined

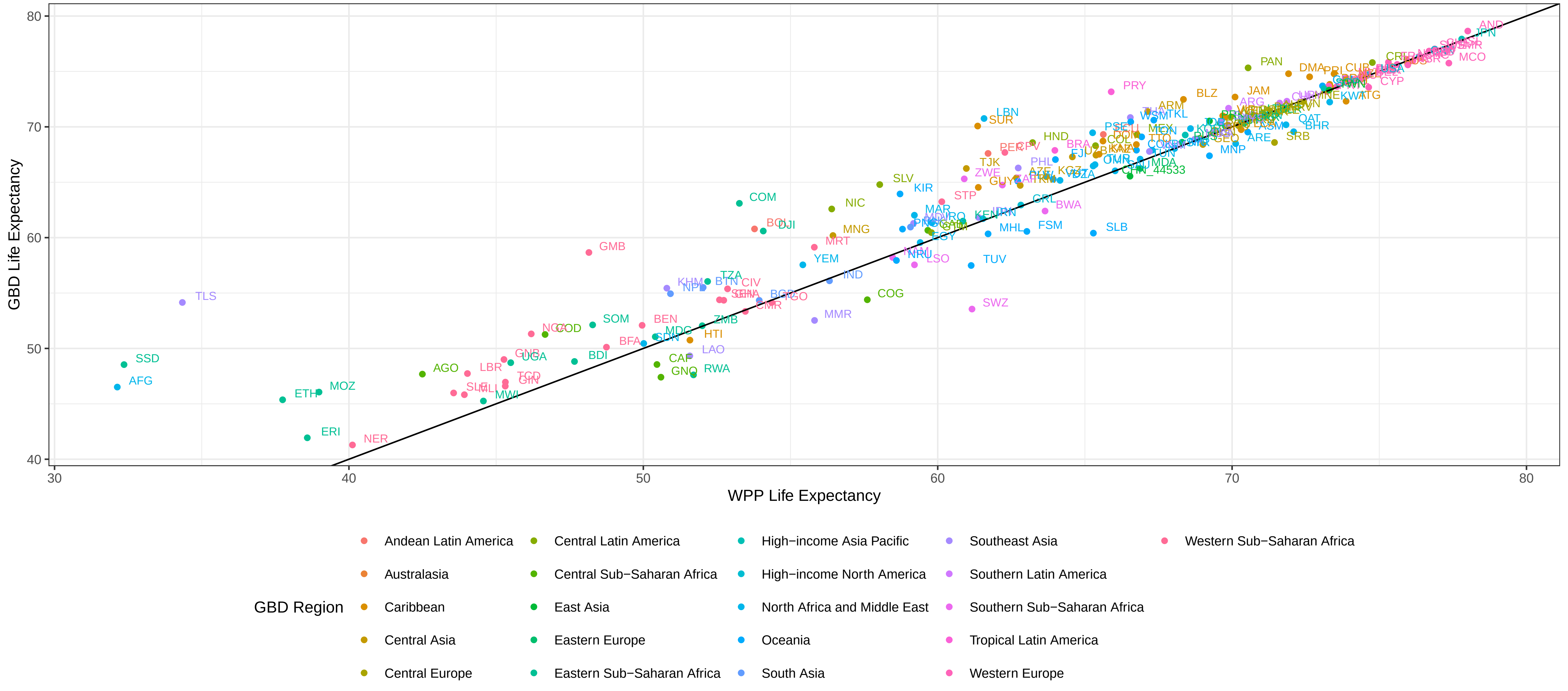

Appendix 2 Figure S8: Comparison of life expectancy at birth estimates for countries in GBD 2023 and WPP 2024 for 1990, both sexes combined

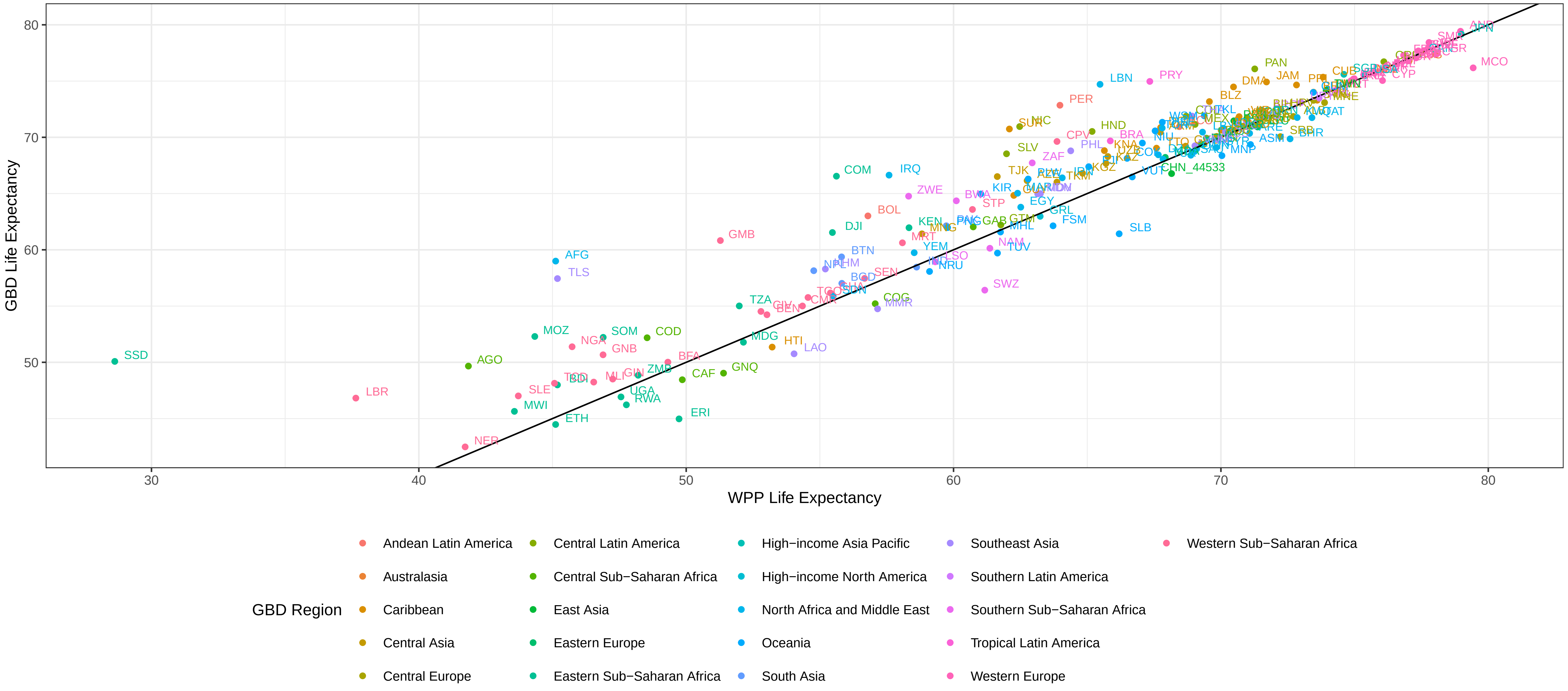

Appendix 2 Figure S8: Comparison of life expectancy at birth estimates for countries in GBD 2023 and WPP 2024 for 1995, both sexes combined

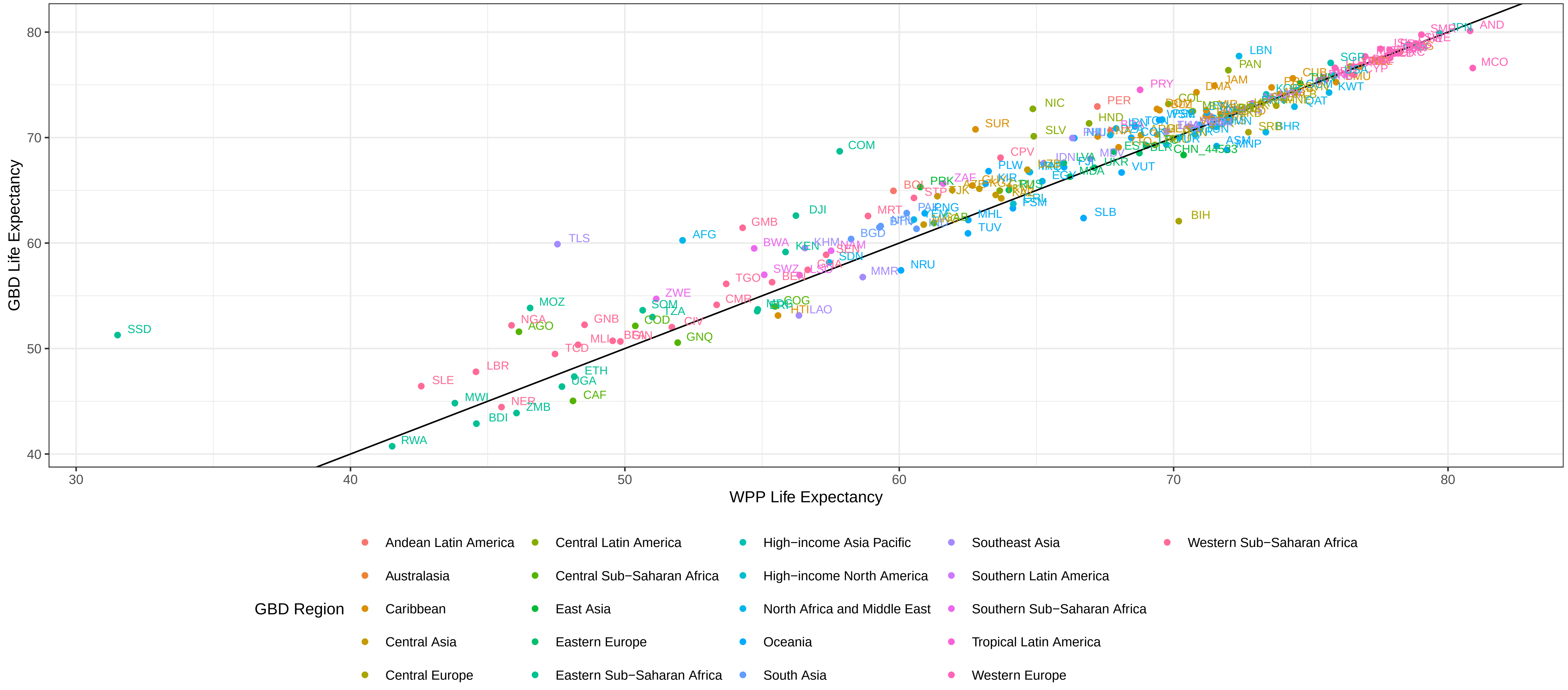

Appendix 2 Figure S8: Comparison of life expectancy at birth estimates for countries in GBD 2023 and WPP 2024 for 2000, both sexes combined

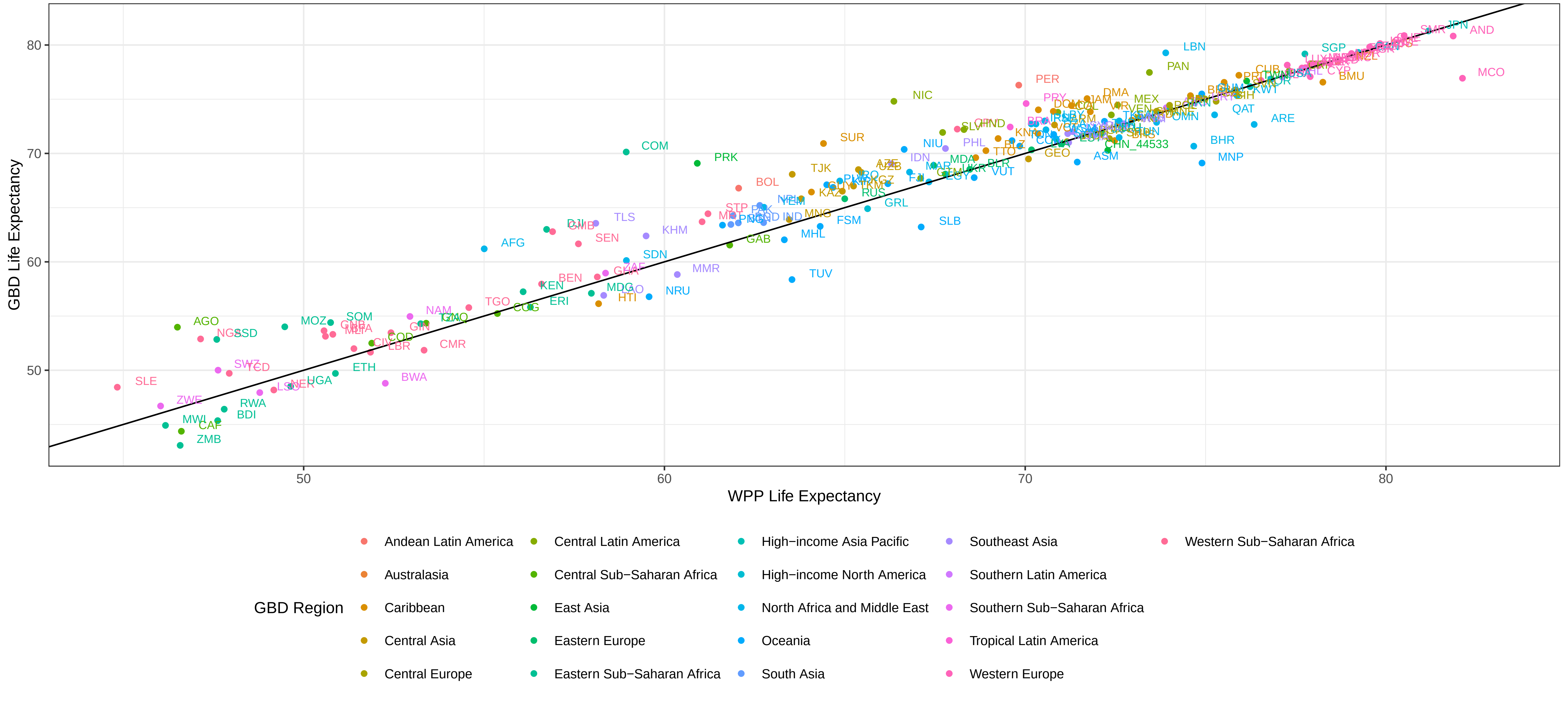

Appendix 2 Figure S8: Comparison of life expectancy at birth estimates for countries in GBD 2023 and WPP 2024 for 2005, both sexes combined

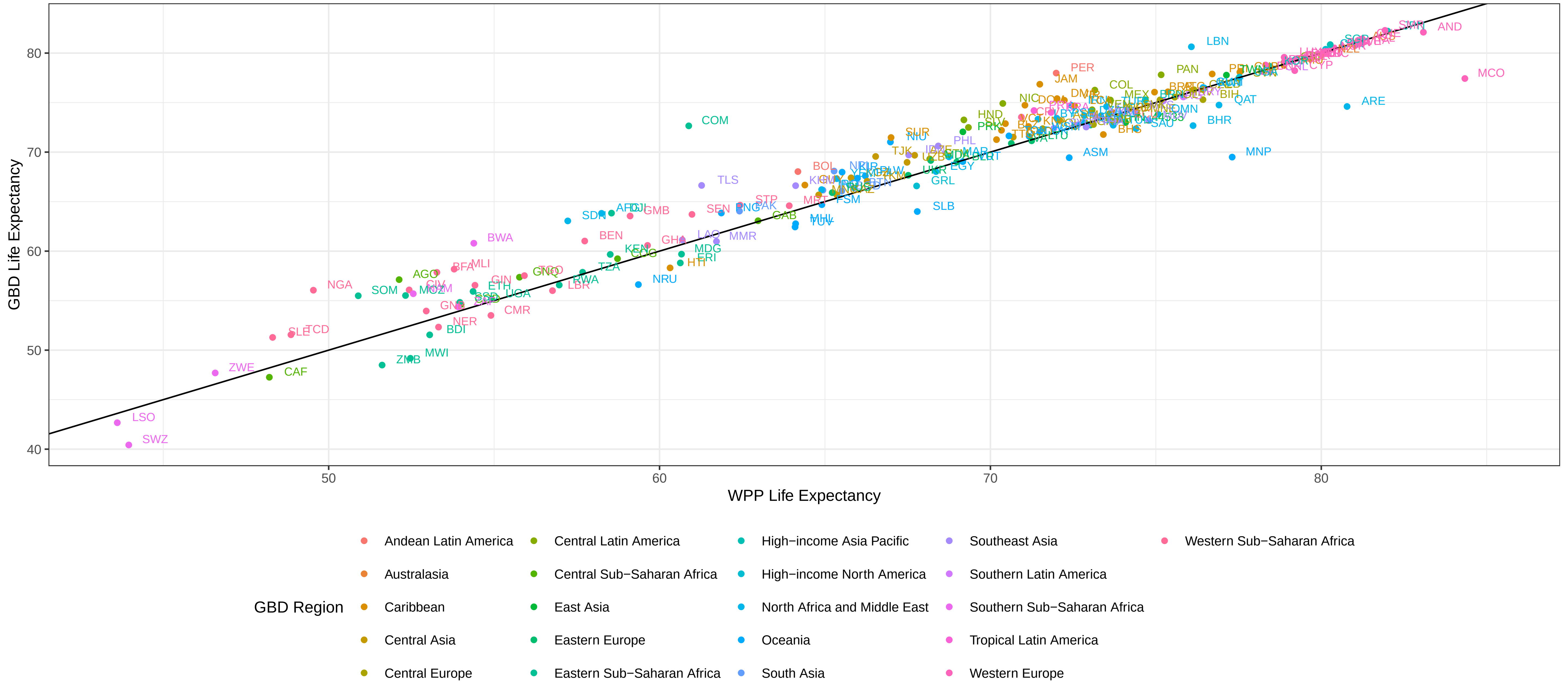

Appendix 2 Figure S8: Comparison of life expectancy at birth estimates for countries in GBD 2023 and WPP 2024 for 2010, both sexes combined

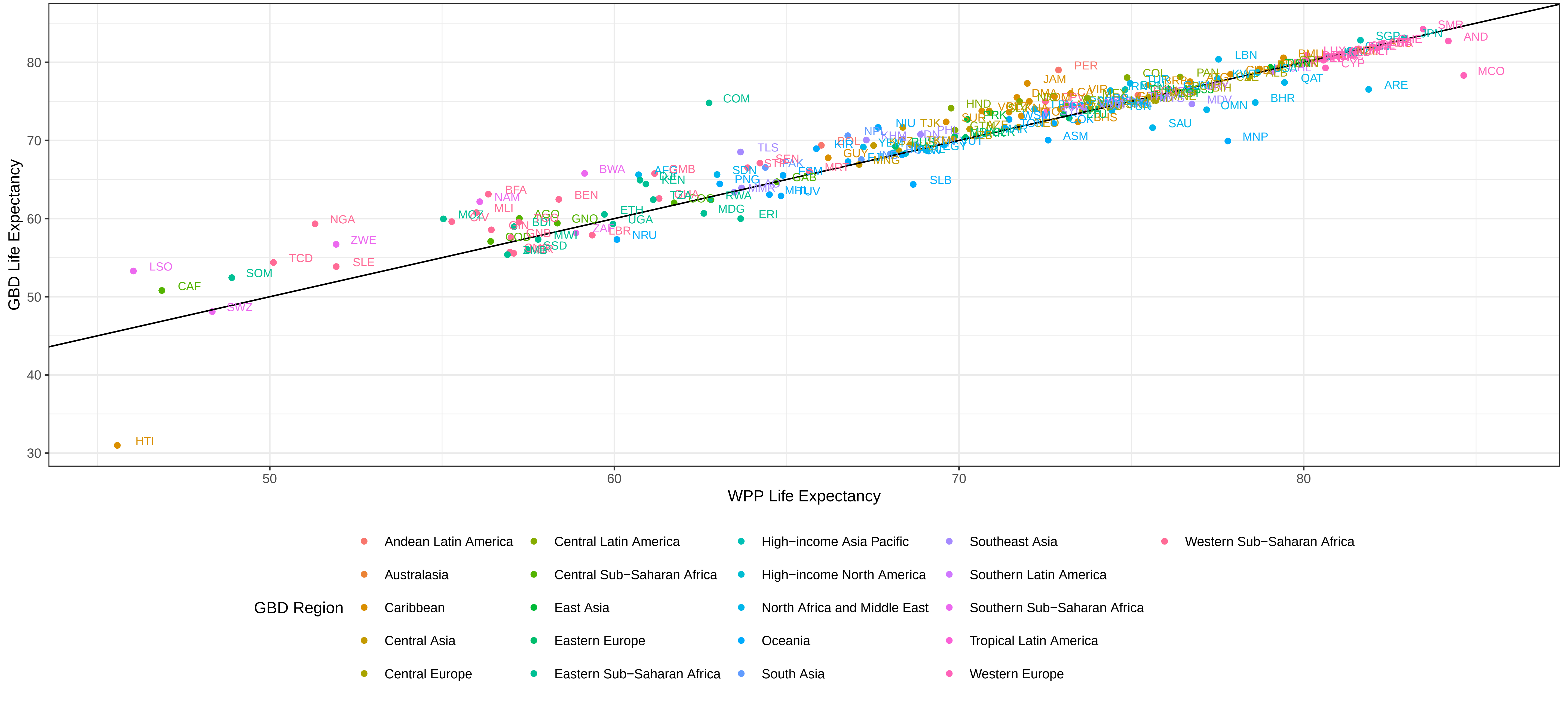

Appendix 2 Figure S8: Comparison of life expectancy at birth estimates for countries in GBD 2023 and WPP 2024 for 2015, both sexes combined

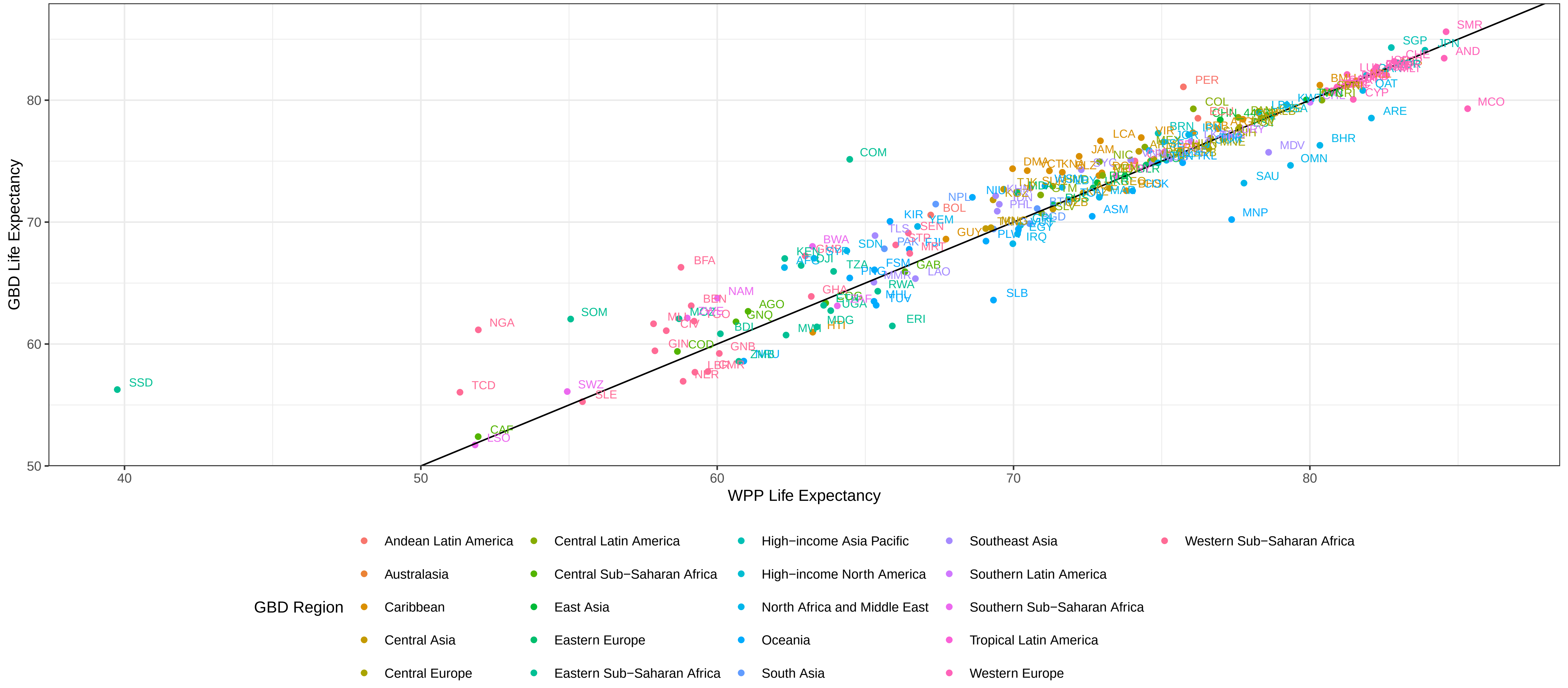

Appendix 2 Figure S8: Comparison of life expectancy at birth estimates for countries in GBD 2023 and WPP 2024 for 2020, both sexes combined

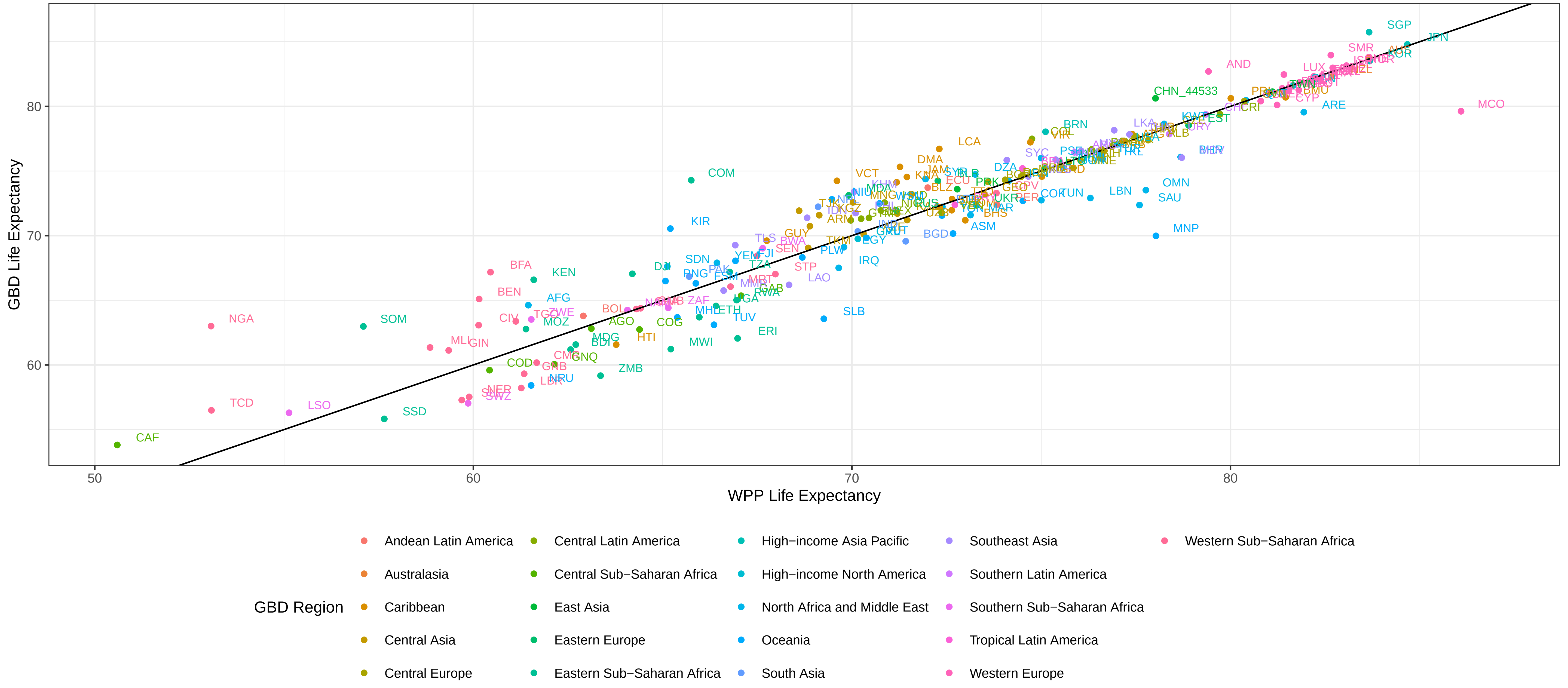

Appendix 2 Figure S8: Comparison of life expectancy at birth estimates for countries in GBD 2023 and WPP 2024 for 2023, both sexes combined

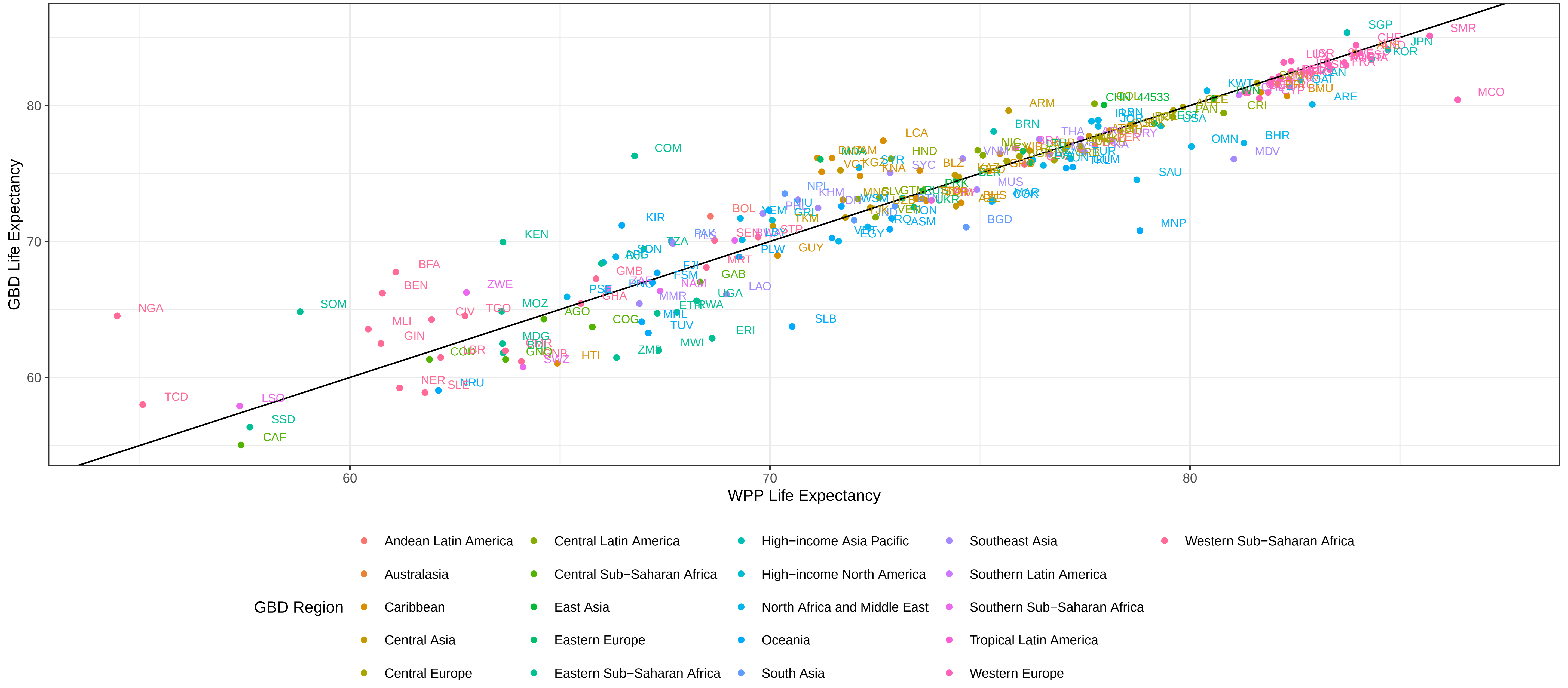

Supplement: Supplementary appendix 2 [file mmc2.pdf]
